# Pi-starvation induced transcriptional changes in barley revealed by a comprehensive RNA-Seq and degradome analyses

**DOI:** 10.1186/s12864-021-07481-w

**Published:** 2021-03-09

**Authors:** Pawel Sega, Katarzyna Kruszka, Dawid Bielewicz, Wojciech Karlowski, Przemyslaw Nuc, Zofia Szweykowska-Kulinska, Andrzej Pacak

**Affiliations:** 1 Department of Gene Expression, Faculty of Biology, Institute of Molecular Biology and Biotechnology, Adam Mickiewicz University, Poznań, Uniwersytetu Poznańskiego 6, 61-614 Poznań, Poland; 2 Center for Advanced Technology, Adam Mickiewicz University, Poznań, Uniwersytetu Poznańskiego 10, 61-614 Poznań, Poland; 3 Department of Computational Biology, Faculty of Biology, Institute of Molecular Biology and Biotechnology, Adam Mickiewicz University, Poznań, Uniwersytetu Poznańskiego 6, 61-614 Poznań, Poland

**Keywords:** Phosphate regulatory network, Barley, Small RNAs, Degradome, RNA-Seq

## Abstract

**Background:**

Small RNAs (sRNAs) are 20–30 nt regulatory elements which are responsible for plant development regulation and participate in many plant stress responses. Insufficient inorganic phosphate (Pi) concentration triggers plant responses to balance the internal Pi level.

**Results:**

In this study, we describe Pi-starvation-responsive small RNAs and transcriptome changes in barley (*Hordeum vulgare* L.) using Next-Generation Sequencing (NGS) RNA-Seq data derived from three different types of NGS libraries: (i) small RNAs, (ii) degraded RNAs, and (iii) functional mRNAs. We find that differentially and significantly expressed miRNAs (DEMs, Bonferroni adjusted *p*-value < 0.05) are represented by 15 molecules in shoot and 13 in root; mainly various miR399 and miR827 isomiRs. The remaining small RNAs (i.e., those without perfect match to reference sequences deposited in miRBase) are considered as differentially expressed other sRNAs (DESs, *p*-value Bonferroni correction < 0.05). In roots, a more abundant and diverse set of other sRNAs (DESs, 1796 unique sequences, 0.13% from the average of the unique small RNA expressed under low-Pi) contributes more to the compensation of low-Pi stress than that in shoots (DESs, 199 unique sequences, 0.01%). More than 80% of differentially expressed other sRNAs are up-regulated in both organs. Additionally, in barley shoots, up-regulation of small RNAs is accompanied by strong induction of two nucleases (S1/P1 endonuclease and 3′-5′ exonuclease). This suggests that most small RNAs may be generated upon nucleolytic cleavage to increase the internal Pi pool. Transcriptomic profiling of Pi-starved barley shoots identifies 98 differentially expressed genes (DEGs). A majority of the DEGs possess characteristic Pi-responsive *cis*-regulatory elements (P1BS and/or PHO element), located mostly in the proximal promoter regions. GO analysis shows that the discovered DEGs primarily alter plant defense, plant stress response, nutrient mobilization, or pathways involved in the gathering and recycling of phosphorus from organic pools.

**Conclusions:**

Our results provide comprehensive data to demonstrate complex responses at the RNA level in barley to maintain Pi homeostasis and indicate that barley adapts to Pi-starvation through elicitation of RNA degradation. Novel P-responsive genes were selected as putative candidates to overcome low-Pi stress in barley plants.

**Supplementary Information:**

The online version contains supplementary material available at 10.1186/s12864-021-07481-w.

## Background

Barley (*Hordeum vulgare* L.) is one of the most commonly cultivated crop plants worldwide. It is a diploid plant with a low chromosome number (*n* = 7) and large genome size (haploid genome size of ~ 5.3 Gbp). In recent years, many resources essential to barley genomic studies have been developed, including a barley genome assembly in Ensembl Plants [1], a large number of expressed sequence tags (ESTs) [2], DNA markers, and useful techniques for stable or transient transformation of barley [3]. The simplicity of cross-breeding and cultivation in a wide range of climatic conditions makes barley a model crop plant in the study of desirable agronomic traits [4]. Studies on the responses of barley to abiotic stresses can help to better its cultivation in variable and adverse conditions. Environmental stressors cause crop damage and reduction of yields, which result in financial losses for agricultural businesses. In plants, abiotic stresses trigger specific stress-induced molecular pathways that often involve different classes of small RNAs (sRNAs) [5–7].

Small RNAs (sRNA) are non-translating into protein class of RNA (20–30 nt) [8]. Best known are siRNA (small interfering RNAs) and miRNA (microRNAs, 18–25 nt) - a class of RNA, which may target chromatin or transcripts to regulate both the genome and transcriptome [9, 10]. Plant small RNAs tend to bind to Argonaute (AGO) family proteins to form either RNA-induced silencing complexes (RISC) for post-transcriptional gene silencing (PTGS) [11] or RNA-induced initiation of transcriptional silencing (RITS) complex for transcriptional gene silencing [12]. Recently, many studies have emerged about various sRNA types, biogenesis, targets, and functions [13–15]. Based on the biogenesis pathway, small RNAs have been classified into miRNAs, siRNAs, phasiRNA and tRFs (tRNA-derived RNA fragments) [16]. Among them, miRNAs and siRNAs are the most extensively studied sRNAs in plants.

Plant *MIR* genes represent independent transcriptional units, which are transcribed by RNA polymerase II (RNA Pol II). Primary transcripts (pri-miRNAs) maturate in a two-step process in the cell nucleus [17]: Firstly, pri-microRNAs are diced out by the DCL1 (DICER-LIKE 1) protein from a stem-loop precursors [18]. The next step of DCL1 protein action leads to the generation of a double-stranded molecule composed of a guide miRNA strand and the passenger miRNA* (star) strand (called the miRNA/miRNA* duplex). Different DCL family members produce miRNA molecules of different lengths; however, the majority of plant miRNAs are 21 nucleotides in length [19]. The miRNA is assembled together with AGO1 (ARGONAUTE 1), in order to create RISC in the cytoplasm which is responsible for mRNA slicing. The cleavage position is precisely determined and occurs in the target mRNA between nucleotides complementary to the 10th and 11th nucleotides of the related miRNA, counting from the miRNA’s 5′-end [20]. Ultimately, target mRNA recognized by the specific miRNA molecule is degraded by 5′-to-3′ exonucleases and the overall pool of valid mRNA transcripts is decreased [21]. Such a mechanism exists in plants to modulate the expression levels of crucial stress-responsive genes [22].

In plants, there are many types of siRNAs, including (i) nat-siRNAs (natural-antisense siRNAs), which are produced from overlapping regions of natural sense–antisense mRNA pairs; (ii) ta-siRNAs (trans-acting siRNAs), processed from non-coding RNA precursors; and (iii) ra-siRNAs (repeat-associated siRNAs), generated from transposable and repetitive elements to mediate further steps of RNAi [9, 23]. tRFs may be produced after cleavage of tRNA ends (to generate 5′-tRF and 3′-tRF) by RNAse T2 [24], as well as DCL (DICER-LIKE) processing in plants [25]. Both miRNAs and siRNAs mediate RNA interference (RNAi) in plants, but there are subtle differences between them. As an endogenous molecule miRNA is diced-out from microRNA precursor folded in stem-loop structure [26], while siRNA is a double-stranded RNA derived from the host genome or directly from viruses or transgenes [27].

The expression of sRNAs changes in response to environmental factors [7, 28] or viral infection [29–31]. Mentioned above classes of sRNAs appear to play important roles in plant growth, development regulation, and adaptation to various stresses. In barley, miRNAs have been shown to (i) mediate tolerance to heat stress [32], (ii) confer drought tolerance [33], (iii) regulate low-potassium tolerance [34], (iv) respond to aluminum stress [35], and (v) maintain inorganic phosphate (Pi) homeostasis [36]. On the other hand, siRNAs mostly function as a defenders of genome integrity in response to foreign nucleic acids [37]. The *TAS3* gene expresses ta-siRNAs, which may negatively regulate auxin signaling by targeting AUXIN RESPONSE FACTOR 3 (ARF3) transcripts [38] and moderate floral architecture in response to drought stress in *Arabidopsis thaliana* L. [39]. The TAS-ARF pathway has been shown to be involved either in the development process of maize (*Zea mays* L.) [40] or regulating lateral root growth in Arabidopsis [41]. In addition, tRNA-derived small RNAs have been shown to accumulate in Arabidopsis roots under Pi-starvation [42], while rhizobial tRFs can regulate nodule formation in soybean (*Glycine max* L.) [13].

Changes in soil nutrient concentrations lead to aberrations in the set of sRNAs, with respect to the prevailing severe environmental conditions [6]. One of the most important macronutrients, which is indispensable for proper plant growth, is phosphorus (P) [43, 44]. P is a component of DNA, RNA, phospholipids, and ATP, and is involved in several biochemical processes such as protein phosphorylation, energy storage and transfer, and regulation of protein synthesis [45]. From soil matrices, P is acquired by the root system in the form of inorganic phosphate ions. Insufficient Pi supply leads to barley growth inhibition [46, 47]. Plant transcriptome response to Pi-starvation involves protein coding genes, sRNAs, and long non-coding RNAs that form regulatory feedback loops. The most widely studied molecules in this context—miRNA399 molecules—are up-regulated in barley shoots and roots under low-Pi conditions [36]. MiRNA399 targets the 5′-UTR of the barley *PHO2* (*PHOSPHATE 2*) transcripts [48], encoding an ubiquitin-conjugating E2 enzyme (UBC24), a negative regulator of Pi uptake and root-to-shoot translocation. PHO2 is involved in ubiquitination of PHOSPHATE TRANSPORTER 1 (PHT1) family [49] and PHOSPHATE TRANSPORTER TRAFFIC FACILITATOR 1 (PHF1) [49]. Transgenic Arabidopsis plants overexpressing miR399 accumulate excessive Pi in shoots and display Pi over-accumulation toxic symptoms. Likewise, such a phenotype has been reported for the *pho2* loss-of-function Arabidopsis mutant [50, 51]. Thus, plants have developed a strategy to regulate the level of miR399 in the cytoplasm. The non-coding RNA molecule, *IPS1* (*INDUCED BY PHOSPHATE STARVATION 1*), has been shown to be highly expressed in plants exposed to Pi-starvation [52–54]. *IPS1* is a non-cleavable miR399 target which inhibits miR399-mediated down-regulation of *PHO2* mRNA by target mimicry [54]. Thus, the RNAi effect of miRNA activity may be counterbalanced by other RNAs, in a stress-dependent manner.

Deep sequencing of sRNAs has uncovered up-regulation of miRNAs like miR156, miR778, miR827, and miR2111, and down-regulation of miR169, miR395, and miR398 in Arabidopsis plants upon Pi deprivation [42, 55]. In rice (*Oryza sativa* L.), Pi-starvation induced the expression level of miR827 molecules, which dysregulate the transcript level of two genes encoding the SPX-MFS (named after proteins SYG1/PHO81/XPR1 and the protein domain Major Facility Superfamily) protein family members SPX-MFS1 and SPX-MFS2 [56, 57]. These two SPX-MFS membrane transporters mediate Pi transport and control Pi homeostasis in shoot [58]. In Arabidopsis, the level of mature miR778 was up-regulated in shoots and roots in low-Pi conditions, while its target gene expression *SUVH6* (*SU(VAR)3–9 HOMOLOG 6*) was accordingly reduced [59]. The *SUVH6* gene encodes a histone H3 lysine 9 (H3K9) methyltransferase, which may enable plants to adapt to environmental conditions by changing their chromatin structure [60]. miR2111 functions as an activator of rhizobial nodulation, which is strictly correlated with the balanced assimilation of nitrogen (N) and P in plants [61, 62]. However, there is still a gap in understanding how Pi-starvation affects the quantity and quality of sRNAs distributed in barley shoots and roots. What kind of sRNAs are preferentially induced? What is the role of sRNAs in responding to Pi-starvation? What are the mRNA targets recognized by those sRNAs in barley?

In this paper, we analyzed changes in the expression levels of RNAs in barley growing under Pi-starvation, as compared to control/Pi sufficient conditions. Our results support the hypothesis that Pi-starvation triggers underlying molecular mechanisms and the expression level of key genes involved in maintaining proper barley growth and development. Combined deep sequencing data (sRNAs, degradome and mRNAs) reveals the widespread importance of low-Pi-dependent miRNAs and genes representing various biological pathways. Using degradome analysis, we identified mRNAs targeted by sRNAs identified in this study. Among these sRNAs, only a small fraction maps perfectly to miRNA sequences deposited in miRBase. Our degradome data show that most sRNAs produced upon Pi-starvation are not involved in gene silencing. In addition, we performed transcriptome analysis of the protein-coding gene expression in barley shoots upon Pi-starvation. Subsequent analyses were performed (GO analysis, chromosomal mapping, and Pi-responsive motifs localization) to characterize specific stress responses in barley plants to accomplish Pi homeostasis.

## Results

### Barley plants display low-Pi symptoms at the morphological and molecular levels

**Fig. 1:**
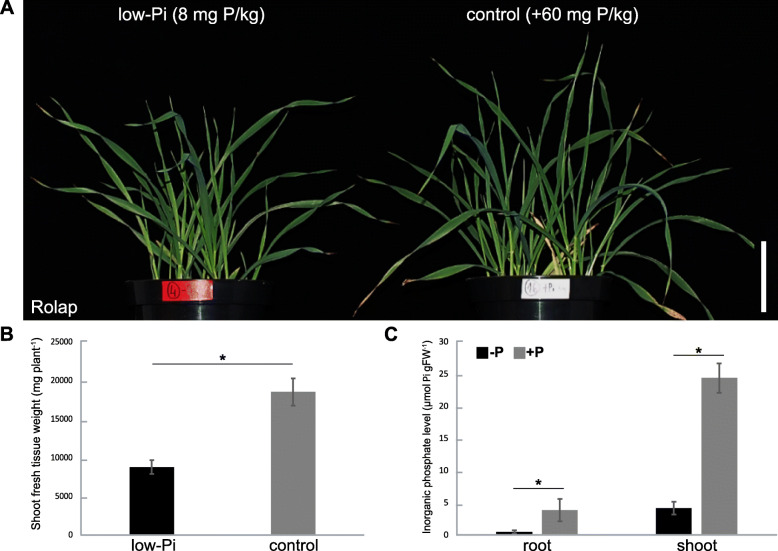
The validation of barley line Rolap plant material under low-Pi stress. **a** Pictures of the plants (*n* = 3) collected on the 23rd day after sowing, grown under low-Pi, 8 mg P/kg soil (left) and control-Pi, addition of 60 mg P/kg soil (right), conditions. **b** Shoot fresh tissue weight (*n* = 3). **c** The Pi concentration measurements performed for barley roots and shoots (*n* = 3). Asterisks indicate a significant difference (* *p*-value < 0.05) calculated using two-tailed Student’s *t-*tests. Scale bar = 10 cm. Error bars = SD

**Fig. 2:**
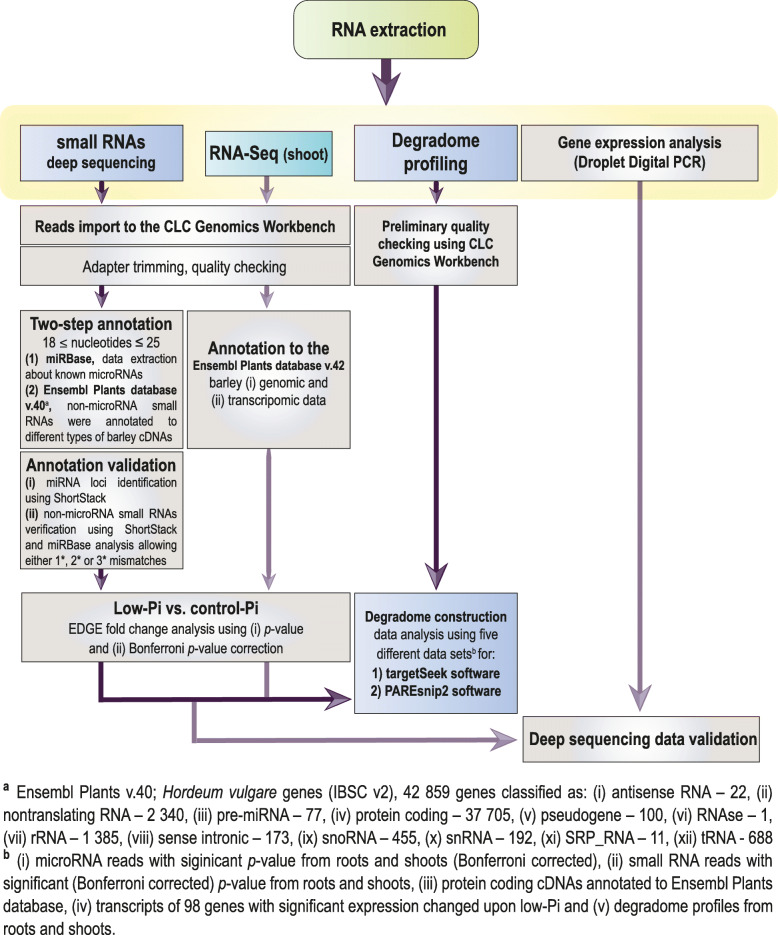
The framework illustrating the data generation protocols used in this study. The low-Pi stress-specific subsets of RNAs were generated following (i) deep sequencing of small RNAs from barley shoots and roots, (ii) transcriptomic RNA-Seq for barley shoots, and (iii) degradome profiling for barley shoots and roots. The obtained data sets were mapped to the references collected from miRBase and Ensembl Plants databases. The log_2_ scale for fold change and Bonferroni corrections were calculated to pick the significantly changed sequences under Pi-deficient and Pi-sufficient conditions

Severe low-Pi responses were induced in the barley plant line Rolap grown in the soil containing 8 mg P/kg. P undernourishment caused over 2-fold reduction of plant shoot biomass (Fig. 1a). Shoot fresh weight of plants at 23rd day post-sowing (dps) was significantly reduced, in comparison with control plants, with average mass 8.8 g for stressed plants and 18.5 g for plants growing under Pi-sufficient conditions (*p* = 0.001) (Fig. 1b). We observed a significantly decreased concentration of Pi ions, with only 0.48 μmol Pi per g of fresh root weight (FW) and 4.2 μmol Pi per g of shoot FW, when compared with the control plants having 3.84 (*p* = 0.0056) and 24.35 μmol Pi/g FW (*p* = 0.0001), respectively (Fig. 1c). To examine the induction of changes at a molecular level by low-Pi stress in barley plants, we measured the absolute gene expression of the low-Pi-responsive marker gene *IPS1*. The barley *IPS1* gene is highly expressed under Pi-deficient conditions in the plant line Rolap. At the tillering stage (23 dps), we detected 4191 copies of *IPS1* RNA for low-Pi treated roots, normalized per 1000 copies of *ADP-RIBOSYLATION FACTOR 1-LIKE* (*ARF1)* reference gene, in comparison to the control plants, with only 58 copies of *IPS1* RNA (*p* = 0.00006) (Additional file 1). Taking validated plant material, we performed tripartite deep-sequencing analysis to: (i) identify Pi-responsive sRNAs, (ii) elucidate changes in the barley transcriptome upon Pi starvation, and (iii) identify mRNA targets for Pi-responsive sRNAs through degradome sequencing (Fig. 2).

### Identification of barley differentially expressed miRNAs (DEMs) under low-Pi

We performed small RNA deep-sequencing to find out which small RNAs are up- or down-regulated by Pi starvation in barley shoots and roots. The average of 30.4 mln reads for roots and 25.2 mln reads for shoots were generated in 50 nt single-read Illumina sequencing (Additional file 2). After adapter and quality trimming, we mapped reads to the miRBase Sequence Database (release 22) to annotate miRNA-derived sequences [63]. A set of parameters were used to define the pool of differentially expressed miRNAs: (i) no mismatches with the reference sequences in the miRBase were allowed; (ii) different types of miRNA sequences were permitted, whether they were annotated as precursor, mature, or isomiR; (iii) miRNA sequences were named accordingly to the name of the assigned reference miRNA; and (iv) significance of fold change (*p*-value < 0.05) was additionally verified using a restricted Bonferroni *p*-value adjustment (Fig. 2).

We found 162 and 138 differentially expressed miRNAs (DEMs) annotated to the miRBase (*p*-value < 0.05) in barley shoots and roots, respectively. Only 25 DEMs were expressed in both examined barley organs (Additional file 3). However, restricted Bonferroni *p*-value correction narrowed down set of miRNAs to 15 in shoots and 13 in roots (Table 1). Those 28 annotated miRNAs were comprehensively analyzed using ShortStack tool to obtain useful annotations for 5 miRNAs. Among them, 3 out of 5 represent DEMs identified in both tested organs: miR399b (root ID: 75, shoot ID: 2019), miR399a (root ID: 105, shoot ID: 2063), miR827 (root ID: 114, shoot ID: 2073). The ShortStack analysis supports two more miRNAs identified in barley shoot: miRNA399b (ID: 2060) and miR827 (ID: 2096) (Table 1, Additional file 4).

**Table 1:**
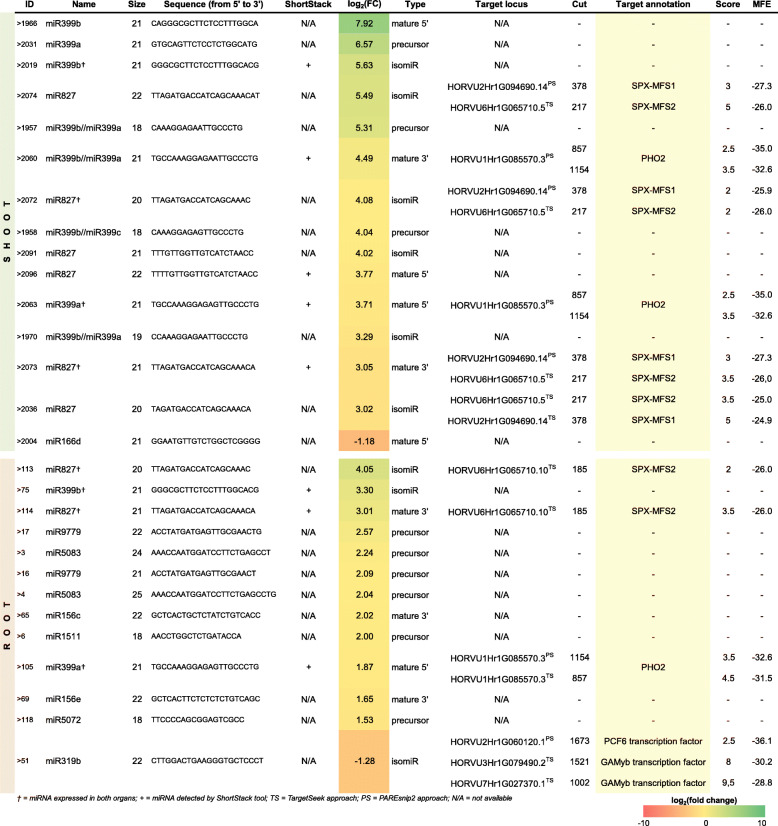
List of differentially expressed miRNAs (DEMs, Bonferroni adjusted *p*-value < 0.05) identified in this study. The ID number specifies the miRNA sequence according to data sets obtained in sRNA-Seq (Additional file 3). The given fold change is shown as a log_2_ value in the column log_2_(FC). Predicted target genes are presented in the table based on dual degradome profiling (Additional files 15, 17, 19 and 23). Type categorizes miRNAs based on the sequences deposited in miRBase without mismatches, isomiRs include miRNAs with nucleotide shift (super or sub) at their 5′, 3′, or at both ends [64]

† = miRNA expressed in both organs; + = miRNA detected by ShortStack tool; *TS* = TargetSeek approach, *PS* = PAREsnip2 approach, *N/A* = not available

**Fig. 3:**
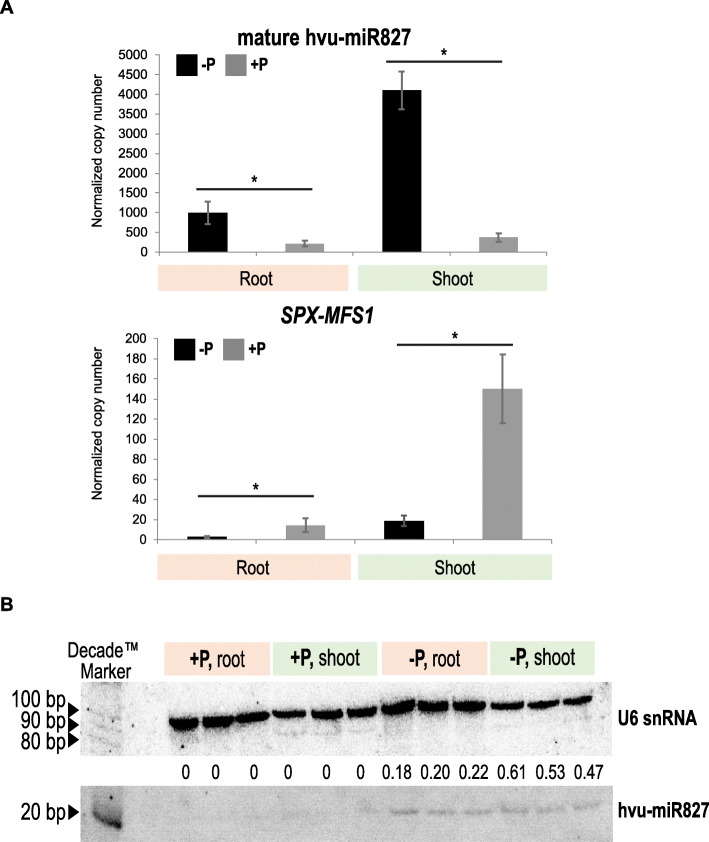
The induced expression level of miR827 (root ID: 114, shoot ID: 2073) correlates with downregulation of its target SPX-MFS1 in barley. **a** The absolute gene expression quantification of identified mature hvu-miR827 and its predicted target gene *SPX-MFS1* using ddPCR. The bars represent copy numbers normalized to 1000 copies of the *ARF1* reference gene; * *p*-value < 0.05, calculated using two-tailed Student’s *t*-tests for three biological and two technical replicates. Error bars = SD. **b** Detection of hvu-miR827 expression pattern in barley samples used in this study for NGS analysis. Specific probes for hvu-miR827 mature sequence and U6 reference gene were used for Northern hybridization performed on a single membrane. The number represents hvu-miR827 band intensity compared to U6 snRNA. The blots were cropped and original, full-length blots are presented in Additional files 32 and 33

sRNA-Seq (small RNA Sequencing) data were experimentally validated by complex analysis of mature miR827 derived from 3′ arm (root ID: 114, shoot ID: 2073) in all samples taken for deep sequencing. The absolute expression level of miR827 is significantly up-regulated in both shoots and roots under a low-Pi regime (Fig. 3a). The log_2_ fold change of miR827 molecules defined by deep-sequencing in shoot was found on the same level in root, log_2_(fc) = 3.05 and 3.01, respectively (Fig. 3a). The ddPCR results were consistent with NGS data showing up-regulation of mature miR827 molecule in both tested organs. These data were confirmed by northern blot hybridization (Fig. 3b).

### Barley plants express an organ-specific set of microRNAs in response to low-Pi conditions

In both organs, majority of the DEMs were significantly up-regulated. Interestingly, out of 15 miRNA, only miRNA166d (ID: 2004) was down-regulated in shoot under low-Pi (log_2_(fold change) = − 1.18). In our previous work, we showed that miRNA166 is expressed in barley during different developmental stages reaching the highest level in 2-week-old plants [65]. miRNA166 plays an important role in plant development, including root and leaf patterning, by targeting mRNA encoding HOMEODOMAIN LEUCINE-ZIPPER CLASS III (HD-ZIP III) transcription factors [66]. Similarly, only miRNA319b (ID: 51) out of 13 DEMs was down-regulated in low-Pi treated roots (log_2_(fold change) = − 1.28). In a previous study, we presented data that Arabidopsis miR319 is a multi-stress responsiveness miRNA [22]. For example, *MIR319b* gene expression was down-regulated in response to drought, heat, and salinity, but up-regulated in response to copper and sulfur deficiency stresses [22].

A specific set of miRNAs was expressed in barley shoot or root under low-Pi (Table 1). In shoot, only two miRNA families, miRNA399 and miRNA827, were induced, while in root we observed a more diverse response. Apart from miRNA399/miRNA827 induction, we found the following additional miRNA to be up-regulated in root: two miRNA5083 (ID: 3, and ID: 4), miRNA1511 (ID: 6), two miRNA9779 (ID: 16, and ID: 17), two miRNA156 (ID: 65, and ID: 69), and miRNA5072 (ID: 118). Among these eight miRNAs, only miR156 has been reported before as Pi-responsive in Arabidopsis [42, 55]. The miR156 isomiRs were also found dysregulated in shoot, but none of them pass the Bonferroni test (Additional file 3). Our results suggest that there is a more complex response to low-Pi stress regarding miRNA expression in roots than in shoots, where the miRNA action is directed to control the transcript level of either *PHO2*, *SPX-MFS1*, or *SPX-MFS2* by just two miRNA families.

### Different classes of small RNAs in barley accumulate in an organ-specific manner under low-Pi regime

**Fig. 4:**
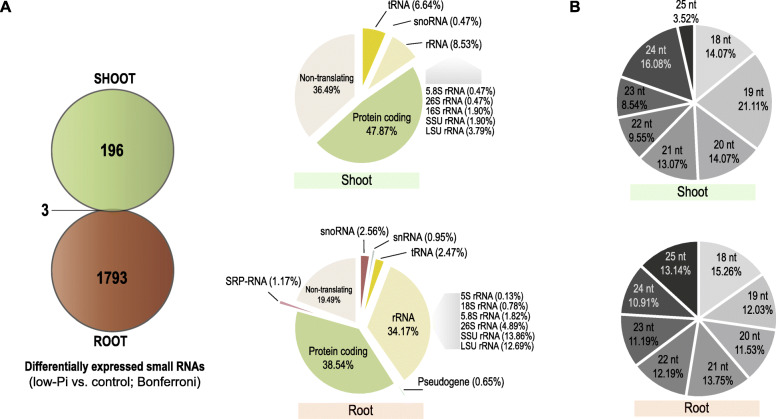
Differentially expressed other small RNAs (DESs) in barley plants under the low-Pi regime. **a** Venn’s diagram illustrating the quantity of identified DESs with Bonferroni corrected *p*-value (left panel). The annotation distribution of DESs in barley shoots and roots based on the calculations present in Additional file 8 (right panel). **b** The length distribution of DESs in roots and shoots

The small RNAs which did not map to miRBase were mapped to particular classes of barley cDNAs derived from the Ensembl Plants database (release 40). Each small RNA was annotated to (i) each class of cDNA in separate analysis, and (ii) to all cDNA classes in a single analysis (Fig. 2). These two-fold annotation provide in-depth analysis and delivers more reliable data about the localization of particular small RNA in barley genome. All sequences mapped to barley cDNAs are listed in Additional file 3. We found that small RNAs, other than miRNAs, differentially expressed sRNAs (DESs) in barley under Pi starvation were represented by 199 unique sequences identified in shoot (0.01% of the average of unique small RNA found in shoots of barley growing under Pi starvation (Additional file 5) and by 1796 (0.13%, respectively) unique sequences identified in roots (Fig. 4a, Additional file 6).

We analyzed whether different lengths (taking sequences from 18 to 25 nt in lenght) and classes of small RNAs contributed to either root or shoot response to low-Pi conditions. In roots, the length distribution of DESs remained balanced, from 10.91% for the representation of 24 nt sequences to 15.26% for the 18 nt sequences, which were the most abundant (including 274 DESs) (Fig. 4b). In shoots, the representations of DES lengths fluctuated more than in roots. The 19 nt sequences were the most visible (21.11%), while three representations did not score more than 10%: the 22 nt (9.55%), 23 nt (8.54%), and 25 nt (3.52%) sequences (Fig. 4b, Additional file 7).

In roots, 1070 unique small RNAs were mapped to cDNA sequences annotated in the Ensembl Plants databases (non-translating, protein-coding, pseudogenes, rRNA, snoRNA, snRNA, sRP-RNA, tRNA), while 726 unique sequences remained without match (Additional file 6). The DESs obtained from low-Pi roots were mostly annotated to protein-coding mRNAs (38.54%), rRNAs (34.17%), and non-translating RNAs (19.49%). Below 5% of overall DESs, we found a number of remaining cDNA classes, such as snoRNAs (2.49%), tRNAs (2.47%), SRP-RNAs (1.17%), snRNAs (0.95%), and pseudogenes (0.65%). While in shoot, we found 199 DESs under the low-Pi regime. Altogether, 116 out of 199 differentially expressed small RNAs (DESs) were annotated to the barley Ensembl Plants database, where 83 sequences remained without match (Additional file 5). In the case of shoot samples, 85% of annotated DESs represented only protein-coding mRNAs (47.87%) and non-translating RNAs (36.49%) (Fig. 4a; Additional file 8). We did not find any DESs annotated to the snRNAs, SRP-RNAs, or tRNAs from barley shoot upon low-Pi. In addition, total numbers of 166 DESs (83%) in shoots and 1560 DESs (87%) in roots were significantly up-regulated after exposure to low-Pi stress (Additional files 5 and 6).

Among the unannotated sRNAs in roots, the highest fold change was observed for a 19 nt DES ID: 388 (log_2_(fold change) = 8.02, induction) and a 22 nt DES ID: 1133 (− 5.87, repression). The BLAST (Basic Local Alignment Search Tool) analysis of first (19 nt) molecule showed a perfect match to either the intergenic region of barley chromosome no. 5, soil bacteria (mesorhizobium), or *Linum usitatissimum* L., while the second molecule (22 nt) mapped to RNA encodes 16S rRNA. Furthermore, in roots, the most abundant small RNA was a 25 nt DES ID: 331 (15,847.7 and 65,590.5 mean of normalized counts in barley root in control and low-Pi conditions, log_2_(fc) = 2.82). This small RNA matched several barley loci encoding SSU (small subunit) rRNAs (Additional file 6).

In our results from low-Pi treated shoot samples, the highest fold change was represented by a 24 nt DES ID: 2112 (log_2_(fc) = 8.72, induction). This 24 nt molecule is a part of transcript encoding a putative pentatricopeptide repeat (PPR) protein. The PPR protein family facilitates the processing, splicing, editing, stability, and translation of RNAs in plants [67]. The most abundant small RNA was a 19 nt DES ID: 2216 (9471.5 and 49,914.1 normalized mean counts in barley shoot in control and low-Pi, respectively, log_2_(fc) = 2,45). This sRNA was mapped to the barley genomic loci (EPlHVUG00000039813), which encodes arginyl-tRNA (trnR-ACG) and a cDNA encoding uncharacterized protein (HORVU2Hr1G084630) which is likely involved in carbon fixation. Interestingly, the pool of DESs was selective, considering organ-specific expression change, providing only three unique sequences that were significantly changed in both barley organs under low-Pi regime (Fig. 4a, left panel). These molecules were: (i) 20 nt DES ID: 2143 (log_2_(fc) = 2.01 in root and 1.16 in shoot, respectively) annotated to the 26S rRNAs, (ii) 24 nt DES ID: 2161 (3.69 in root and 2.07 in shoot) annotated to the RNA encoding the barley MYB21 transcription factor, and (iii) 21 nt DES ID: 2265 (4.64 in root and 6.27 in shoot) mapped to the intergenic region of barley chromosome no. 3 (Additional file 5).

The proper annotation of DESs was confirmed by ShortStack analysis. Among DES representatives only one small RNA (shoot ID: 2265, root ID: 1813, unannotated) has features of potential miRNA molecule and it is upregulated in both tested organs (Additional file 9). All DES molecules were once again annotated to miRbase allowing either 1, 2, or 3 mismatches. The new potential miRNA has one mismatch and belongs to miR399 family. Less restricted annotation revealed two more miR399 molecules (ids = 2141, 2222) and three miR827 (ids = 2279, 2280, 2281) expressed in shoot. In root we found three miR9779 (ids = 396, 645, 1629), two miR1511 (ids = 140, 141), two miR9653a (ids = 403, 404), miR319b (ID: 1266) and miR9675 (ID: 556) (Additional files 5 and 6). Nonetheless, all of them were classified as unannotated.

The results obtained in this study show again that barley roots exhibit a more diverse pool of Pi-responsive small RNAs which may trigger developmental adaptation of the root to Pi-starvation. Additionally, 613 rRNA-derived sRNAs are up-regulated, whereas 176 rRNA-derived sRNAs are down-regulated in barley roots (Additional file 6). We believe that such sRNA may be further processed, serving as a Pi source to compensate Pi deficiency.

### Identification of barley genes responsive to Pi-starvation

Since we observed, that most of the other sRNAs in shoot were derived from either protein-coding mRNAs or non-translating RNAs, we checked whether this observation is correlated with gene expression changes of polyadenylated RNAs in barley shoot under Pi-starvation. Among 98 of identified DEGs, the transcripts of 56 annotated loci were significantly up-regulated, while those derived from 42 loci were down-regulated in Pi-starved barley shoots (Table 2). Repressed loci were found to be preferentially located at barley chromosome no. 2, while induced loci were found mostly at barley chromosomes no. 3, no. 5 and no. 6 (Additional file 10).

**Table 2:**
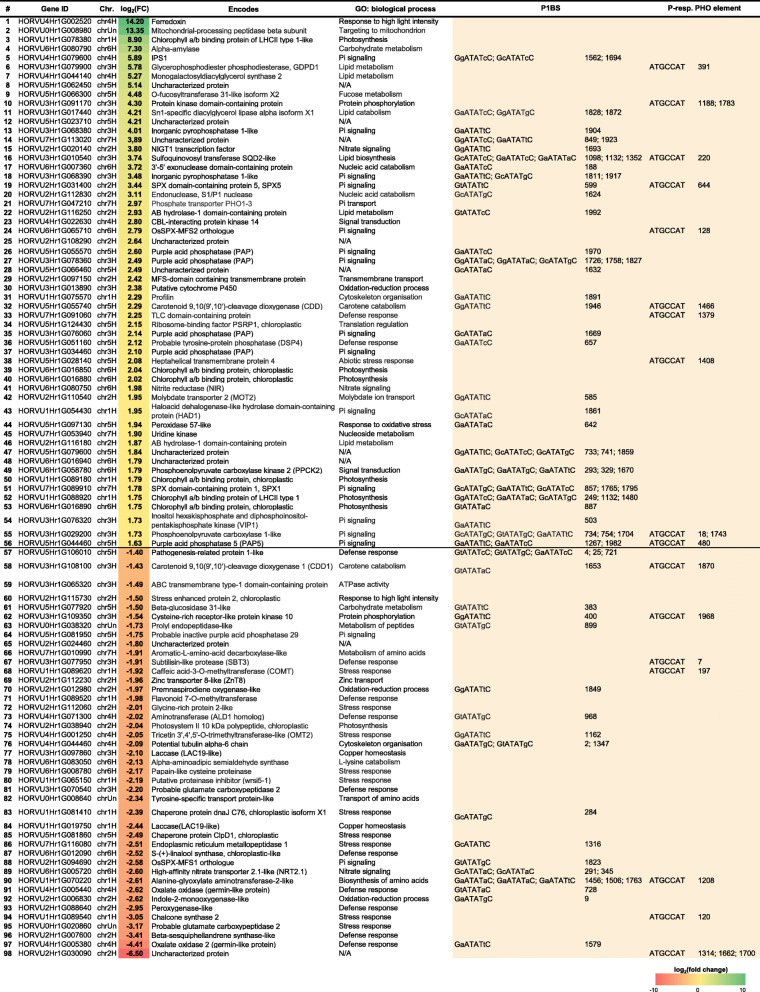
List of 98 DEGs from barley shoots (low-Pi vs. control/sufficient Pi) identified in this study

**Fig. 5:**
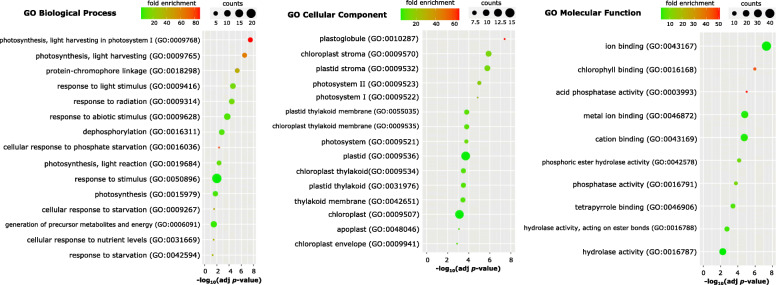
The significantly enriched GO terms in the categories of biological process, molecular function, and cellular localization ranked based on Bonferroni adjusted *p*-values < 0.05. The fold enrichment underlines the overrepresented GO terms, which contribute more to the total number of observed GO terms in the background frequency

The highest enrichment of shoot DEGs was found in the GO terms, either (i) belonging to the cellular components of the chloroplasts; (ii) showing catalytic activity, either ion or chlorophyll binding properties; and (iii) involved in the various biological and metabolic processes related to photosynthesis, stress response and plant defense (Fig. 5, Additional file 11). A major set of up-regulated DEGs represent genes involved in the Pi signaling. Among them, we found genes encoding: *IPS1* (log_2_(fc) = 5.89) [54], inorganic pyrophosphatase (PPase, 4.01) [68], SPX-domain containing protein 5 (SPX5, 3.44) [69], phosphate transporter PHOSPHATE 1–3 (PHO1–3, 2.97) [70], SPX-MFS2 (2.79) [56], haloacid dehalogenase-like hydrolase (HAD1, 1.95), [71] and five different purple acid phosphatases (PAPs) (Table 2) [72]. Interestingly, four genes were induced to a higher extent than the low-Pi stress marker, *IPS1* gene. These genes encode ferredoxin (FD1, log_2_(fc) = 14.20), mitochondrial-processing peptidase (13.35), chlorophyll a/b binding protein (8.90), and alpha-amylase (7.30), and are engaged in photosynthesis, redox reactions, reactive oxygen species (ROS) homeostasis, and co-ordinated mobilization of nutrients. Chloroplasts and mitochondria are the organelles with the highest Pi requirements. Strong *FD1* gene up-regulation most likely reflects the accumulation of reduced ferredoxin in chloroplasts. Low-Pi lowers the capacity to process incoming light and enhances starch accumulation in chloroplasts, thereby leading to photoinhibition [73, 74]. Within the category of genes that were significantly down-regulated, most of them were related to stress and defense responses (Table 2); for instance, uncharacterized protein (HORVU2Hr1G030090, − 6.50), oxalate oxidase (− 4.41) [75], beta-sesquiphellandrene synthase (− 3.41), glutamate carboxypeptidase (− 3.17), chalcone synthase (− 3.05) [76], or caleosin-like protein (− 2.95). Only two repressed genes are known to be directly involved in Pi signaling and metabolism, *SPX-MFS1* (− 2.58), targeted by miR827 [57] and probable inactive purple acid phosphatase (− 1.75). Additionally, two genes encoding laccases (*LAC19-like*, Table 2), cell wall-localized multi-copper oxidases, were significantly down-regulated (− 2.10 and − 2.44) in our mRNA RNA-Seq data. Laccases are involved in copper homeostasis and lignin biosynthesis, and have been shown to be targeted by miR397 in maize [77] and Arabidopsis [78]. Furthermore, key genes encoding proteins involved in the nitrate and phosphate cross-talk were affected by low-Pi conditions in barley shoots, such as *NIGT1* (NITRATE-INDUCIBLE, GARP-TYPE TRANSCRIPTIONAL REPRESSOR 1) transcription factor (3.80) [79, 80] and nitrite reductase (1.98), as well as high-affinity nitrate transporter *NRT2.1* (NITRATE TRANSPORTER 2.1) (− 2.60) [81].

**Fig. 6:**
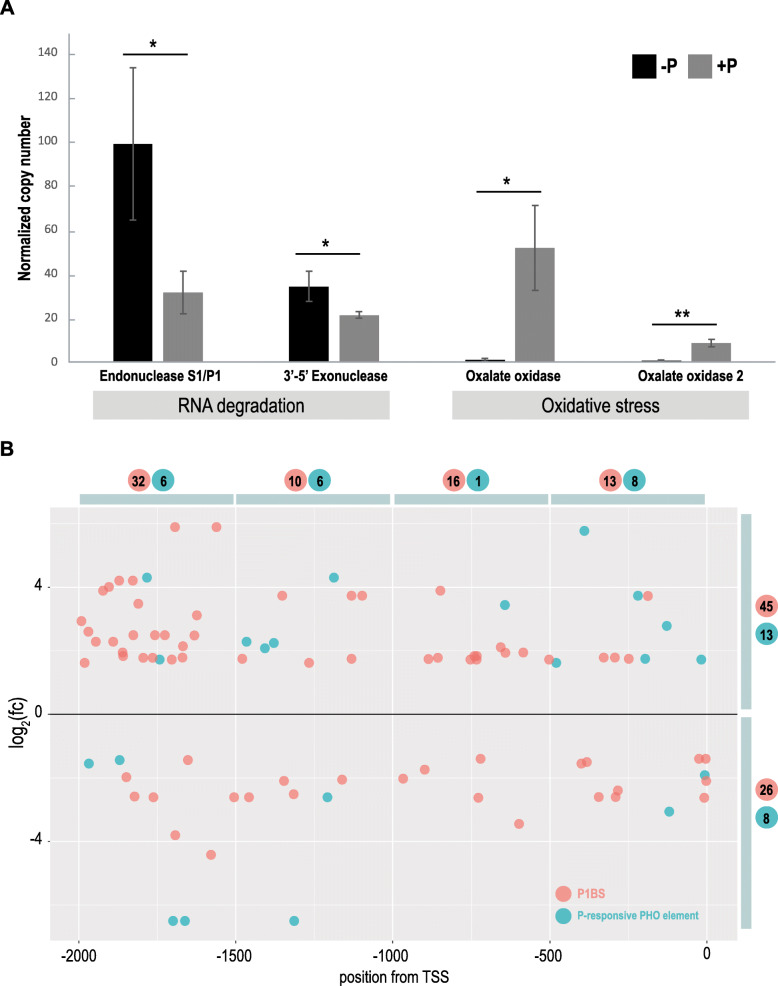
Molecular characterization of identified DEGs from barley shoot. **a** Quantification by ddPCR of the absolute expression levels of the DEGs belonging to two selected pathways in barley shoots. Two up-regulated DEGs, which encode endonuclease S1/P1 and 3′-5′ exonuclease, are involved in nucleic acid metabolism and further RNA degradation. Two down-regulated DEGs, which encode two different oxalate oxidases, are involved in the reduction of oxidative stress. The bars represent copy numbers normalized to 1000 copies of the *ARF1* reference gene; *p-value < 0.05, **p-value < 0.001, calculated using two-tailed Student’s *t*-tests for three biological and two technical replicates. **b** Localization of all P1BS and P-responsive PHO *cis*-regulatory elements within the 2000 bp upstream from the DEG TSSs. On the graph, grouped motifs are specifically located in every 500 bp, induced (log2(fc) > 0), or repressed (log2(fc) < 0). The motif quantity in each group is shown in either red (P1BS) or blue (PHO elements) dots

Absolute quantification of a few selected transcripts was performed to validate RNA-Seq data obtained in this study. Two genes which were highly induced (encoding endonuclease S1/P1 and 3′-5′-exonuclease) and two which were severely repressed (encoding oxalate oxidases) under the low-Pi regime were taken for ddPCR (droplet digital PCR) analysis (Fig. 6a). We confirmed statistically significant changes (*p < 0.05*) in normalized copy number (per 1000 copies of the *ARF1* reference gene) of all genes taken for analysis.

### Pi-responsive motifs found in the promoters of DEGs

In general, genes that are affected by Pi status possess characteristic *cis*-regulatory elements within either promoter or 5′-UTR regions [82]. Previously, we have shown the importance of the P1BS motif (PHR1 binding sequence, consensus GnATATnC, [83]) and P-responsive PHO elements (consensus ATGCCAT, [84]) in the expression efficiency of the barley *PHO2* gene [48]. Both motifs may bind PHR-like (PHOSPHATE STARVATION RESPONSE) transcription factors (TFs) and act as activators or repressors of downstream gene expression in a Pi-dependent manner [85]. Likewise, we hypothesized that regulatory regions of the identified DEGs had Pi-responsive motifs, which may be bound by PHR TFs, causing gene expression dysregulation. To confirm this hypothesis, we analyzed DNA sequences from the 2000 bp region upstream of the predicted transcription start sites from all 98 DEGs (Additional file 12). In the next step, promoter data were directly screened for P1BS and P-responsive PHO element consensus sequences by multiple promoter analysis using the PlantPAN3.0 tool. We confirmed the presence of Pi-dependent motif in 55 out of 98 DEGs promoters. An in silico approach detected 46 DEGs having at least one P1BS motif (Additional file 13) and 17 DEGs with at least one P-responsive PHO element (Fig. 6b, Additional file 14). The most over-represented motifs were found in the promoters of genes encoding sulfoquinovosyl transferase SQD2-like (log_2_(fc) = 3.74) [86], phosphoenolpyruvate carboxylase 1-like (log_2_(fc) = 1.73) [87], and pyridoxal phosphate-dependent transferase (log_2_(fc) = − 2.61) [88]. Each of the genes harbor three P1BSs and one P-responsive PHO element, as well (Table 2).

### Degradome profiling describes post-transcriptional regulatory network of identified DEMs

After identification of (i) differentially expressed miRNAs (DEMs), (ii) other sRNAs (DESs), and (iii) mRNAs (DEGs), we used this comprehensive data together with cDNAs annotated in the Ensembl Plants database to identify the sRNAs directly involved in RNA degradation. The DESs were also examined, because we assumed that there may have been putative miRNAs that were not mapped to the miRbase, due to restricted query settings allowing no mismatch or that there are other small RNAs which could be involved in mRNA degradation. It was shown that human Ago proteins are associated with short RNA originated from non-miRNA sequences (mRNA, snRNA, snoRNA, tRNA, vRNA) [89]. Molecules which exhibited a single mismatch (or more) may still function as miRNA in barley. Degradome libraries were carried out for root, as well as for shoot, and sequenced using an Illumina System. The received data were analyzed using two independent in silico approaches: PAREsnip2 (PS) and TargetSeek (TS) (Fig. 2). At times, the different algorithms used elicited different miRNA targets; however, the general degradome pattern was equivalent for both approaches (Table 1, Additional files 15, 16, 17, 18, 19, 20, 21, 22, 23, 24, 25 and 26).

In order to determine the potential cleavage activity of miRNAs identified in shoot and root we performed degradome analysis. Firstly, we searched for the potential target mRNAs for differentially expressed miRNAs with significant fold change (Bonferroni adjusted *p*-value), taking 15 DEMs from shoot and 13 DEMs from root, respectively (Table 1). A total of 168 scores were obtained for shoot DEMs (113 using the TargetSeek approach and 55 using PAREsnip2) (Additional files 15, 19 and 20), while in root there were 26 records (24 and 2, respectively) (Additional files 17, 23 and 24).

**Fig. 7:**
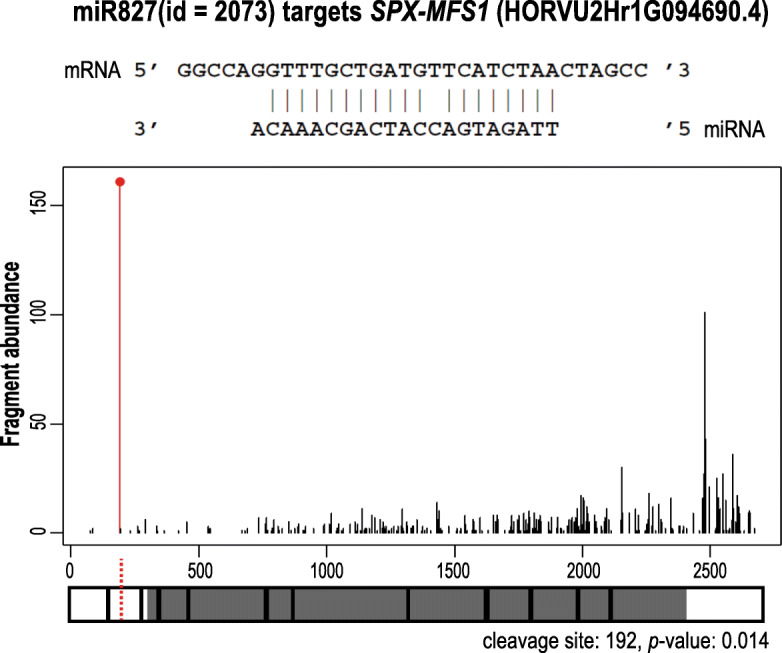
Degradome profile of *SPX-MFS1* mRNA directed for cleavage by miR827 in barley shoot using the PAREsnip2 approach. The red vertical line shows the cleavage position; the cleavage position 192 is within exon no. 2 in the 5′-UTR of the *SPX-MFS1* transcript (*p*-value = 0.014). The black vertical lines show the positions within the *SPX-MFS1* cDNA to which degradome fragments (reads) were mapped. The number of reads (fragment abundance) is depicted by the height of the red and black lines. Below the graph, the structure of the *SPX-MFS1* transcript is presented. The white boxes denote UTRs, the gray boxes denote coding sequence, and the red dotted line denote the cleavage site within the 5′-UTR

None of the DEM annotated as part of the pre-miRNA was found in the degradome platform. While 10 out of 19 DEMs annotated as mature/isomiR molecule scored for target prediction. In shoot, a majority of records corresponded to different miR399 and/or miR827 isomiRs and their known targets PHO2 or SPX-MFS1/SPX-MFS2, respectively. One of the best scoring miRNA:mRNA match was found for mature miRNA827 (21 nt, ID: 2073), which guides cleavage within the 5′-UTR of *SPX-MFS1* mRNA (isoform no. 4) in position 192 (*p = 0.014*) (Fig. 7). In roots, the most downregulated miR319b (22 nt, ID: 51) has predicted three different target loci in barley. The miR319b guides for cleavage PCF6 TF and two GaMyb-like TFs (Table 1). The plant GAMyb TFs, have been shown to activate gibberellin-responsive gene expression of α-amylase in barley [90, 91].

### Putative regulatory small RNAs identified in degradome data

**Fig. 8:**
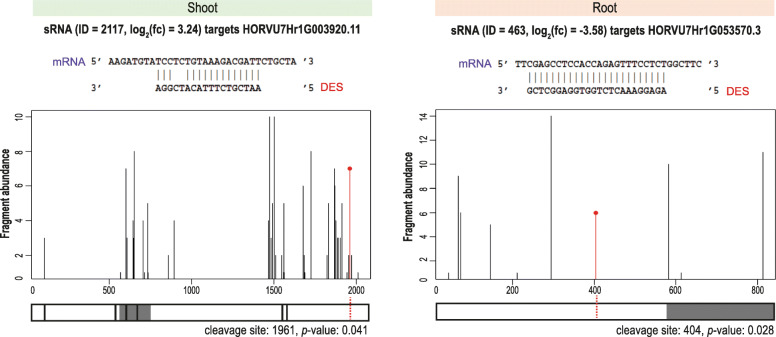
Degradome profiles of two DES representatives that are significantly changed in shoot or root and exhibit high possibility to cleave mRNA targets. The HORVU7Hr1G053570 locus encodes an uncharacterized protein with unknown PTHR47188 domain; the HORVU7Hr1G003920 locus encodes glutaminyl-peptide cyclotransferase. The white boxes denote UTRs, the gray boxes denote coding sequence, and the red dotted line denote cleavage site

Degradome profiling was performed to test whether any of the sequences from the 1796 DESs found in roots or 199 DESs found in shoots contribute to the complexity of gene regulation during low-Pi stress. A total of 759 records (245 using the TargetSeek approach and 514 using PAREsnip2) were found in the degradome profiles matching root DESs (Additional files 18, 25 and 26) and 160 records (87 and 73, respectively) matching shoot DESs (Additional files 16, 21 and 22). Taking only either the most up-regulated or the most down-regulated sRNAs for degradome screening, we found six promising target genes in shoot and five in root (Table 3). For example, in roots, the highly up-regulated 20 nt DES ID: 348 (log_2_(fc) = 6.46) binds to the 3′-UTR region of the MYB44 TF’s mRNA and guides/promotes cleavage in the 1037 position (PAREsnip2: score = 4; MFE = − 33.3) (Table 3). RNA-Seq data for potato (*Solanum tuberosum* L.) proved that expression of the *MYB44* gene is highly downregulated under low-Pi in roots [92], which may be the result of miRNA-guided PTGS. Studies in potato have indicated that MYB44 TF may form a regulatory complex together with WRKY6 TF, which negatively regulates Pi transport by suppressing *PHO1* expression [92]. Other degradome records in this study, among the most differentially expressed sRNAs, were found to target mRNAs of the V-ATPase assembly factor (VMA21-like) and three barley genomic loci encoding uncharacterized proteins (HORVU7Hr1G053570, HORVU1Hr1G027340, and HORVU0Hr1G023910) (Table 3). For example, the potential cleavage activity was predicted for 24 nt DES ID: 463 (log_2_(fc) = − 3.58), which may target the mRNA encoding uncharacterized protein with unknown PTHR47188 domain (Fig. 8).

**Table 3:**
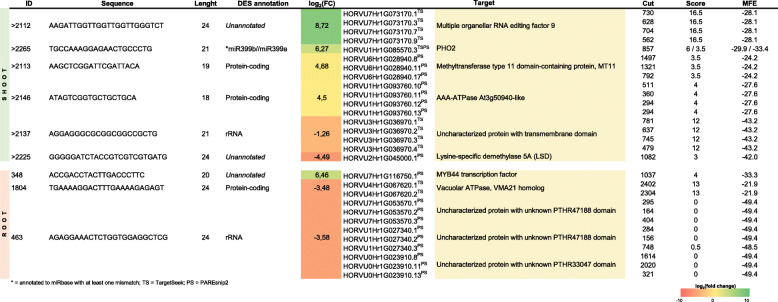
List of genes predicted in degradome analysis to be guided for cleavage by the most up- and down-regulated DES identified in this study

* = annotated to miRbase with at least one mismatch; *TS* = TargetSeek, *PS* = PAREsnip2

Analogous degradome screening was done for shoot data. Among all identified DESs, we found that the most upregulated sequence, 24 nt DES ID: 2112 (log_2_(fc) = 8.72), targets the 3′-UTR of mRNA encoding multiple organellar RNA editing factor 9 (MORF9, HORVU7Hr1G073170, TargetSeek: score = 16.5, MFE = − 28.1) (Table 3). MORF9 proteins are required for RNA editing in plastid mRNAs, which may contribute to stress adaptation in plants [93, 94]. In both approaches, we found that the 21 nt DES ID: 2265 (log_2_(fc) = 6.27) targeted the same isoform of *PHO2* mRNA (HORVU1Hr1G085570.3, TargetSeek: score = 6, MFE = − 29.9, PAREsnip2: score = 3.5, MFE = − 33.4). When we browsed the miRBase using this 21 nt sRNA as a query, we found high similarity to the osa-miR399a, exhibiting only one mismatch. Thus, we suspect that such sRNA may function as another miR399 isomiR in barley. Most dysregulated DESs were also found to target mRNAs encoding methyltransferase type 11 domain-containing protein (MT11), AAA-ATPase (At3g50940-like), lysine-specific demethylase 5A (LSD), or an uncharacterized protein with a predicted transmembrane domain (HORVU3Hr1G036970). The best scoring degradome records were found for the 21 nt DES ID: 2279 (log_2_(fc) = 3.11), which targets mRNAs encoding SPX-MFS1 (PAREsnip2: score = 2.5, MFE = − 27.5) and SPX-MFS2 (TargetSeek: score = 3.5, MFE = − 26) (Additional file 27). Further analysis revealed that such DES annotates to osa-miR827 with one mismatch. This result suggests that such sRNA may exist as another miR827 isomiR in barley. Moreover, this is consistent with the screening made for differentially expressed miRNAs, where miR827 targeted both SPX-MFS proteins, depending on the approach we used. In addition, the 18 nt DES ID: 2117 (log_2_(fc) = 3.24) was found to target mRNA encoding glutaminyl-peptide cyclotransferase (HORVU7Hr1G003920, PAREsnip2: score = 2.0, MFE = − 19.6) (Fig. 8), which may be involved in plant defense reactions [95].

Some other interesting Pi-related targets which are recognized by DESs were found in our root degradome data, but the prediction scores were weaker than those in the examples described above. For instance: nitrate reductase (HORVU6Hr1G003300), high-affinity nitrate transporter-activating protein (HORVU5Hr1G115500), MYB-like TF (HORVU7Hr1G027370), and stress-induced TF NAC1 (HORVU5Hr1G111590) were found. Interestingly, among the 98 DEGs identified in this study, only two of them (SPX-MFS1 and SPX-MFS2) were found as putative targets of miRNA guided activity. In addition, none of the DEGs gene IDs were found to match with any of the identified IDs classified for differentially expressed small RNAs.

## Discussion

**Fig. 9:**
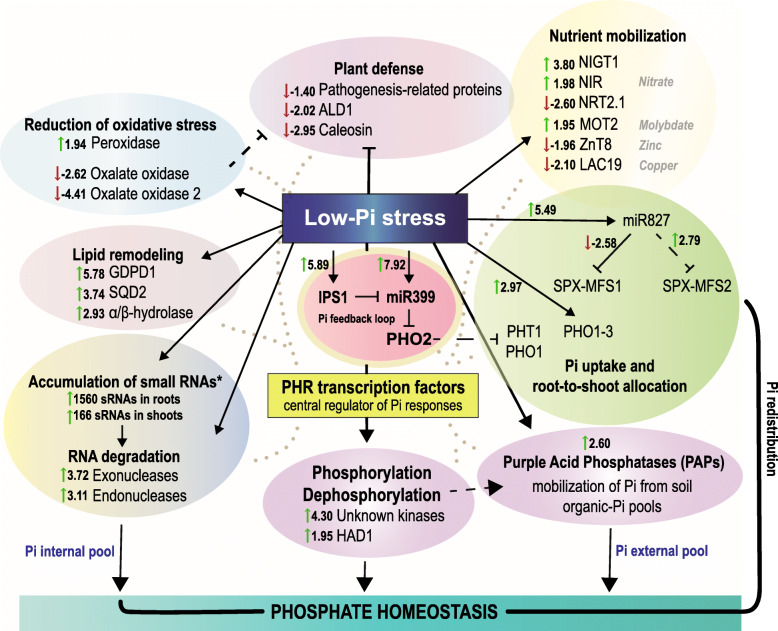
Barley pathways triggered by Pi-starvation to maintain plant homeostasis. Graphical overview illustrates primary strategies in Pi-starved barley plants based on our shoot transcriptomic analysis, small RNA-Seq, and degradome profiling. The low-Pi induced feedback loop is located in the middle part, which is involved in the positive regulation of phosphate transporters (i.e., PHT1, PHO1) prompting Pi uptake. The MYB–coiled-coil (MYB-CC) protein family includes PHR transcription factors (yellow frame), which act as a major regulator to either induce or repress Pi-responsive genes in plants. The asterisk represents the data from shoot and root sRNA-Seq. Dotted lines display wide area of molecular networks, connecting most of the plant low-Pi stress responses. All components depicted on the graph are listed in Table 2. Values correspond to log_2_(fold change) with Bonferroni adjusted *p*-values

In this study, we used a tripartite approach (sRNA-Seq, mRNA-Seq, and degradome-seq) to describe the set of small RNAs differentially expressed in barley roots and shoots under low-Pi stress. We detailed the sophisticated responses of barley shoots and roots involved in the maintaining of Pi homeostasis (Fig. 9). Integrated deep-sequencing data were used to describe organ-specific adaptations to low-Pi through either activation or repression of different classes of 18–25 nt small RNAs. Additionally, the mRNA-Seq analysis of low-Pi treated barley shoot was performed to analyze the correlation between shoot-derived small RNAs, annotated to either protein-coding mRNAs (47.87%) or non-translating RNAs (36.49%), and gene expression changes of polyadenylated RNAs. We identified a total of 28 differentially expressed miRNAs (Bonferroni adjusted *p*-value) annotated to miRBase (release 22) without mismatches and a total of 1995 differentially expressed other small RNAs (Bonferroni adjusted *p*-value).

In plants, a limited number of miRNA have been shown to be specifically and strongly induced by Pi limitation, including miRNA399 [96], miRNA778 [59], miRNA827 [55], and miRNA2111 [55, 97]. In this work, the majority of DEMs represent various miR399 and miR827 isomiRs in both tested organs. Our results are consistent with sRNA sequencing data published for Arabidopsis [42, 55] and *Nicotiana benthamiana* L. [98]. In both plant species, authors have shown that the number of various miR399 isomiRs was the most abundant in shoots and roots under low-Pi. Eight of the 15 DEMs (after Bonferroni *p*-value adjustment) we found in barley shoots belonged to the miR399 family. However, in root, miR399 was represented only by two DEMs; the miR399a and miR399b (Table 1). Previously, our absolute copy number analysis of mature miR399 demonstrated that its normalized expression level is 4-fold downregulated in barley roots, as compared to in shoots, under a low-Pi regime [99]. The long-distance movement of signal molecules is known to be crucial for Pi recycling and allocation from root to shoot. The root system is responsible for Pi acquisition conducted by phosphate transporters belonging to PHT1 family, which saturate cell membranes during Pi deficiency [100]. The level of PHT1 proteins is negatively controlled by the PHO2, which is suppressed by miR399 (see model in Fig. 9) [101]. A high level of miR399 molecules was detected in Arabidopsis wild type rootstocks grafted with miR399-overexpressing scions [42, 102]. Thus, miR399 is involved in a plant’s systemic response to low-Pi conditions and acts as a long-distance signal, moving from shoot to root to control Pi homeostasis [102]. In Arabidopsis, miR827 has been shown in multiple studies to target the 5′-UTR of the *NITROGEN LIMITATION ADAPTATION* (*NLA*) gene [103, 104]. In rice, the *OsNLA* mRNA has a ‘degenerate’ osa-miR827 potential cleavage site, that is why miR827 does not cleave the *OsNLA* transcript in vivo [56, 57, 105]. Likewise, we did not find *NLA* mRNAs to be targeted by any of the identified hvu-miR827 isomiRs in our barley degradome records. The *NLA* gene encodes an E3 ubiquitin-protein ligase with RING and SPX domains, which interacts with the PHO2 to prevent the excessive accumulation of Pi [106]. In roots, a more diverse set of miRNAs contributed to the compensation of low-Pi stress, compared to that in shoots. We found six up-regulated miRNA molecules (DEMs) in roots mapped to pre-miRNAs, such as: two miR9779, two miR5083, miR1511 and miR5072. In addition, none of them was found in our degradome analysis. The differentially expressed other small RNAs in roots (DESs, 1796 molecules) were represented by 90% of the total set of other sRNAs (DESs from both organs), annotated to all classes of cDNAs taken for analysis. Among the identified set of DESs, we found non-miRNA small RNAs with high probabilities to target various mRNAs involved in plant adaptations to abiotic stresses, plant defense, and/or transcription (Table 3, Additional file 27). Further analysis will be performed to experimentally validate the in silico predicted PTGS role of Pi-responsive small RNAs found in this study, as well.

In this paper, we detailed the shoot differentially expressed genes (DEGs) harboring Pi-responsive *cis*-regulatory elements, involving various molecular pathways and biological processes. These DEGs were mostly engaged in Pi mobilization and utilization upon Pi-starvation in barley shoots. Other sRNAs selected from shoots were much less abundant and represent sequences belonging mostly to non-translating and/or protein-coding mRNAs. None of the sRNAs mapped to the differentially expressed mRNAs found in the transcriptomic analysis, suggesting that they may inhibit gene expression through translational repression or may serve as a Pi source for developing plant organs. Plants are adapted to recycle nutrients from senescing organs. For example, class II RNases are involved in the degradation of housekeeping rRNAs before cell death occurs [107]. During senescence extracellular class I RNases were shown to degrade RNA during Pi-starvation in Arabidopsis as well [108]. In 2018, Ren et al. published RNA-Seq data describing the barley transcriptome under low-Pi stress [109]. The authors compared the transcriptomes of two barley genotypes with contrasting low-Pi stress tolerance. In roots, they observed 28 DEGs classified into the following functional groups: Pi transport, transcription, lipid metabolism, metabolism, and phosphorylation/dephosphorylation [109]. Likewise, our mRNA-Seq data from barley shoot discovered the DEGs involved in all mentioned functional groups. In our shoot transcriptome analysis, we found the same four DEGs: (i) *GLYCEROPHOSPHODIESTER PHOSPHODIESTERASE 1* (*GDPD1)* gene (HORVU3Hr1G079900, log_2_(fc) = 5.78), (ii) *MONOGALACTOSYLDIACYLGLYCEROL SYNTHASE 2* gene (MGD2, HORVU4Hr1G044140, 5.27), (iii) *SPX5* (HORVU2Hr1G031400, 3.44), and (iv) *SPX1* (HORVU7Hr1G089910, 1.78). Furthermore, Ren et al. found three genes encoding purple acid phosphatases (PAPs) [109]; however, they appeared from different barley genome loci than the five PAPs we found in our study. It was shown that vacuolar and secreted PAPs are involved in Pi scavenging and remobilization during Pi-starvation and leaf senescence. Other related RNA-Seq data published for either wheat (*Triticum aestivum* L.) [110], rice [111], soybean [112], *Plantago major* L. [113], and maize [114] demonstrated similar molecular patterns to those in our study (Table 2). Based on our work we propose a model of barley adaptations to Pi-starvation (Fig. 9). Interestingly, the presence of crucial Pi-responsive *cis*-regulatory elements within the promoter regions of more than 50% of identified DEGs may indicate their essential and direct role in conditioning low-Pi tolerance (Fig. 6b). The most widely studied PHR TFs, such as PHR1 in Arabidopsis [83] and PHR2 in rice [115], bind to P1BS elements present in the promoter of a broad range of Pi-related genes. Moreover, the PHR protein family exhibits high functional redundancy and its protein members may co-operatively form a regulatory network to maintain Pi homeostasis in plants [85]. In our previous paper, we showed that, within the 5′-UTR of the *PHO2* gene, there is another Pi-responsive motif called the PHO element in close proximity to the P1BS [48]. The PHO element can be bound by PHR-like transcription factors in barley plants, as well [48], and has been found in the promoters of many DEGs in independent Arabidopsis [116, 117] or soybean [112] studies.

The elevated abundance of sRNAs has been associated with the up-regulation of two types of nucleases (endonuclease S1/P1 and 3′-5′ exonuclease), which may catalyze the degradation of RNA into shorter fragments [118, 119] and play a relevant role in nutrient mobilization under Pi-starvation. It seems likely that sRNA production upon Pi-starvation is an effect of RNA degradation by different types of nucleases. Thus, degraded RNA may serve as a source of Pi necessary in emerging plant organs. We found also two genes encoding oxalate oxidases, the expression of which was significantly downregulated during Pi-starvation. This class of genes is responsible for the inactivation of oxalic acid, which mediates fungal–plant pathogenesis in barley [120].

## Conclusion

To conclude, our studies provide comprehensive data sets, which may serve as a rich platform for the characterization of barley responses to Pi-starvation at an RNA level. Furthermore, our data may be used as a reference tool for parallel studies in other crop plants.

## Materials and methods

### Plant material

Three biological replicates of barley root and shoot samples were analyzed. One replicate consisted of three plants growing in a single pot containing 1.5 kg of soil mixed with sand in a 7:2 ratio. Material was collected from the barley line Rolap (obtained from the Institute of Plant Genetics of the Polish Academy of Sciences, Poznań, Poland [121]) growing under low-Pi (8 mg P/kg soil) and Pi-sufficient conditions (after addition of 60 mg P/kg soil), as described before [48]. On the 23rd day after sowing plant shoots (Zadoks decimal code 22–23 [122]), they were cut off and fresh tissue weight was measured. Immediately afterwards, shoots and roots were collected and frozen in liquid nitrogen to be kept at − 80 °C until use.

### Pi concentration measurements

Measurements of inorganic phosphate level were performed according to the protocol we have described before [46]. The samples were measured in two technical and three biological replicates using an Infinite F200 Pro (TECAN, Switzerland).

### RNA isolation

Four procedures of RNA isolation were used, depending on the following experiments: (i) small RNA expression level analysis (ddPCR using TaqMan™ MicroRNA assays, NGS of small RNAs, Northern hybridization); (ii) shoot transcriptome analysis; (iii) degradome - PARE (Parallel Analysis of RNA Ends) [123] analysis for mRNA cleaved by miRNA; or (iv) validation of RNA-Seq data using ddPCR.

(i) Small RNA expression level analysis

RNA isolation was performed using a modified method allowing enrichment of small RNAs, according to the detailed protocol we published before [65].

(ii) RNA for RNA-Seq

RNA was extracted from a 100 mg of shoot sample using RNA extraction buffer [99] and a Direct-Zol RNA MiniPrep Kit (Zymo Research). According to the Lexogen’s SENSE mRNA-Seq Library prep kit v2 user guide DNase treatment step was omitted to avoid RNA hydrolysis.

(iii) RNA for degradome analysis

Procedure of RNA isolation from barley root and shoot (growing in low-Pi conditions) used for degradome profiling was performed using a method described by German et al. using RNA extraction buffer [123], along with some modifications that we have described previously [65, 124].

(iv) mRNA-Seq data validation

To validate the transcript level of significantly changed genes, we used precise dd-PCR analysis. To isolate RNA for these analyses, we used a Direct-Zol RNA MiniPrep Kit (Zymo Research) with some modifications that we have described in detail previously [99]. The RNA material was treated using DNase I enzyme from the above kit (Zymo Research).

### Preparation of NGS libraries

We prepared three different NGS libraries: (i) small RNA, (ii) transcriptome - mRNA, and (iii) degradome.

(i) Small RNA libraries

Small RNA libraries were prepared using a TruSeq Small RNA Library Prep Kit (Illumina). In brief, small RNAs of 15–30 nt in length were separated on denaturing 8 M urea 15% polyacrylamide (PA) gel and purified and ligated to 3′ and 5′ RNA adapters. Next, the RNA fragments were reverse transcribed to run PCR: PCR products were indexed by utilization of specific RNA PCR Index Primers and PCR profile, according to the Illumina protocol (RPI, Illumina). PCR products were separated on 7% PA gel containing 1% glycerol. After 10′ staining by SYBR™ Gold Nucleic Acid Gel Stain (Invitrogen, Thermo Fisher Scientific)/0.5xTBE buffer, DNA fragments of 140–160 bp in length were cut and eluted using 400 μl elution buffer (50 mM Mg-acetate, 0.5 M ammonium acetate, 1 mM EDTA, 0.1% SDS) after O/N incubation, 28 °C, 400 rpm. Then, chloroform/phenol pH = 8.0 purification libraries were precipitated using 1.5 μl GlycoBlue™ coprecipitant (15 mg/mL) (Ambion, Thermo Fisher Scientific) and three volumes of 100% ethanol. Purified libraries were quantified using a Qubit® dsDNA HS Assay kit (Invitrogen, Thermo Fisher Scientific) and Qubit 3.0 Fluorometer (Invitrogen, Thermo Fisher Scientific). The quality of the libraries was analyzed using a High Sensitivity D1000 ScreenTape Assay (Agilent Technologies) and a 2200 TapeStation (Agilent Technologies). A total of 12 libraries were pooled together in equal molar ratio and sequenced by Fasteris SA (Switzerland).

(ii) Degradome library construction

Degradome library construction was performed according PARE technique described by German et al. [123]. Ligation was performed using a Rapid DNA Ligation Kit (Roche), according to the manufacturer instructions. The ligation mixture was composed of MmeI-digested PCR product and 3′ DNA Adapter, kept for 6 h at RT and at 4 °C overnight, then purified using phenol/chloroform extraction. PCR reaction was performed in a 50 μl volume containing MmeI fragment-3’Adapter template, appropriate index-containing primer (0.5 μM final concentration), MmeI Universal Fwd primer (0.5 μM final concentration), 350 μM dNTPs, Q5 reaction buffer, and Q5® Hot Start High-Fidelity DNA Polymerase (New England Biolabs), using the following steps: 94 °C for 2 min; 94 °C for 30 s, 60 °C for 30 s, 72 °C for 30 s—14 cycles; and 72 °C for 7 min. PCR products were separated on 8% PA gel containing 1% glycerol. Appropriate in length bands were cut and eluted O/N. Quantitative analysis of the purified libraries was performed using a Qubit® dsDNA HS Assay kit (Invitrogen, Thermo Fisher Scientific) and a Qubit 3.0 Fluorometer (Invitrogen, Thermo Fisher Scientific). Finally, the quality of the libraries was analyzed using a High Sensitivity D1000 ScreenTape Assay (Agilent Technologies) and a 2200 TapeStation (Agilent Technologies). Each library possessed an individual specific index. The four libraries were pooled together in equal molar ratio and sequenced by Fasteris SA (Switzerland). In present study two degradome libraries were analyzed.

(iii) mRNA libraries

Shoot transcriptome libraries were carried out using a SENSE mRNA-Seq library prep kit v2 (Lexogen), according to the manufacturer’s protocol and as previously described [48]. A 420 pg of Spike-In RNA Variants SIRV-set3 (Lexogen) was added to 1500 ng of total RNA. ERCC mix was used for Spike-in analysis.

### Library sequencing

Sequencing of small RNAs was performed (i) internally, using a MiSeq paired-end kit to check the library quality using a MiSeq® Reagent Kit v3 (Illumina) at the Laboratory of High Throughput Technologies, Adam Mickiewicz University, Poznań, Poland. Received data showed proper library quality and low-Pi induced changes in small RNA levels. (ii) The main deep sequencing (12 small RNA libraries, degradome, mRNA) was performed externally by Fasteris SA (Switzerland).

### Data analysis

Differences in small RNAs, RNAs levels, and preliminary degradome data analysis were performed using a CLC Genomics Workbench (Qiagen Aarhus A/S).

### Small RNA data analysis

The trimming procedure was used with default settings for quality trimming (quality score limit 0.02), adapter trimming, and for removal of small RNAs longer than 25 nt and shorter than 18 nt. Reads were extracted, counted, and normalized per 1,000,000 reads. Then, we set up the Experiment analysis for samples derived from roots and shoots separately (two-group comparison, unpaired). Empirical analysis of DGE (EDGE) was used to find significant fold changes in small RNA expression levels between samples derived from different treated barley. Moreover, we performed EDGE Bonferroni and EDGE FDR *p*-value correction calculation. First, all small RNAs were annotated to miRBase (release 22) without mismatches and with strand-specific alignment. Then, unannotated small RNAs (i.e., those not identified in miRbase) were sorted according to the lowest *p*-value. The annotation reports for shoot small RNAs are present in Additional file 28 and for root small RNAs in Additional file 29. All identified differentially expressed sRNAs were annotated with internal ID numbers: IDs from 1 to 138 represent miRNAs identified in roots, and successfully mapped to miRBase (*p*-value < 0.05); IDs 139–1934 represent other small RNAs identified in roots (Bonferroni *p*-value correction < 0.05); IDs 1935–2096 represent miRNAs identified in shoots, and successfully mapped to miRBase (*p*-value < 0.05), and IDs represent 2097–2295 other small RNAs identified in shoots (Bonferroni *p*-value correction < 0.05).

### Annotation validation

ShortStack version 3.8.5 [125] was used for the identification of potential microRNA molecules. The software was run with: mismatches - 0, fold size - 400 parameters. The input files for the analysis were: fastq files containing small RNA sequences after adapter removal and fasta file with *Hordeum vulgare* genome (IBSC_v2) from Ensembl Plants database. The forna tool was used to visualize secondary structure of RNA [126].

### Degradome data analysis

Degradome construction was performed using two different approaches, which allowed for a more in-depth analysis (Fig. 2). In the first method, the raw sequencing reads were processed by Cutadpt program (https://cutadapt.readthedocs.io/en/stable/) to trim low-quality and adapter sequences. Only sequences of length 15 nt and above were selected for further analyses. The processed sequencing reads were aligned to the reference sequences using bowtie. The count of 5′-end marked cleavage sites was scored by Perl script and normalized to the depth of sequencing and total signal for each of the reference transcripts. The putative miRNA:target pairs were predicted by a custom program (targetSeek) which included the following steps: (i) calculation of perfect match MFE (minimum free energy); (ii) RNAplex-based (Vienna package) screening for sRNA:transcript pairs; (iii) filtering number of bulges and length of sequence overhangs by MFE (percent of the perfect MFE match); and (iv) calculation of prediction score using a penalty schema for loops, bulges, and G:U wobble pairing. In the second approach, we used the PAREsnip2 software [127] to generate t-plots in conjunction with five databases (Fig. 2). Potential miRNA targets are classified into one of five categories, where category 0 indicates the best miRNA-target match. The lower the alignment score, the better the alignment between the sRNA and the target site [127]. During PAREsnip2 analysis, we set the Fahlgren and Carrington targeting rules to permit a mismatch or G:U wobble at position 10 [128].

### Identification of DEGs

Experiments were performed in three biological replicates of plants grown under low-Pi and control conditions. Paired-end sequencing reactions of the 150 nt reads were performed using an Illumina System. Total read numbers from six samples were mapped to the barley reference genome from Ensembl Plants Genes 42 (*Hordeum vulgare* IBSC v2). The library’s quality and sequencing accuracy were verified carefully (i) by adding Spike-in RNA Variant Control Mixes (Lexogen) (Additional file 30) and (ii) by quality trimming. RNA-Seq analysis was performed using following normalization method - TPM expression values. TPM (Transcripts Per Million) is computed with the following equation TPM $=\frac{RPKM\times {10}^6}{\sum RPKM}$. RPKM (Reads Per Kilobase of exon model per Million mapped reads) is computed using following equation RPKM $=\frac{total\ exon\ reads}{mapped\ reads\ (millions)\times exon\ length\ (kb)}$. RNA-Seq reads were mapped to the gene track = *Hordeum vulgare*. IBSC_v2.42(Gene), mRNA track = *Hordeum vulgare*. IBSC_v2.42 (RNA) using the CLC Genomics Workbench (QIAGEN) software, as previously described [48]. Differentially expressed genes (DEGs) were selected using Differential Expression in Two Groups tool (present in CLC Genomics Workbench software). This tool uses multi-factorial statistics based on a negative binomial Generalized Linear Model (GLM). Among potential differentially expressed transcripts only those which went through restricted Bonferroni *p*-value adjustment (< 0.05) were considered as differentially expressed genes (DEGs).

### GO analysis

Gene ontology (GO) analyses were performed using the gProfiler tool (version e102_eg49_p15_7a9b4d6) [129]. The over-representation binomial tests classified DEGs within GO domains (cellular component, biological process, and molecular function) with Bonferroni adjusted *p*-value < 0.05. Fold enrichment was calculated as described before [130]: ((number of genes annotated to specific term/term size)/(total number of inputted genes/total number of genes used for selection)). The calculations are present in Additional file 11. The plots were generated using already published protocol [131].

### *cis*-regulatory motif localization within DEG promoters

To analyze the enrichment of Pi-related *cis*-regulatory motifs, we extracted 2000 bps upstream of transcription start site from each identified DEG. Such data were directly screened to look for any either P1BS- or P-responsive PHO element consensus sequences using multiple promoter analysis with the PlantPAN3.0 tool [132].

### ddPCR

To determine the absolute copy number of genes encoding *IPS1*, SPX-MFS1, endonuclease S1/P1, 3′-5′ exonuclease, oxalate oxidase, and oxalate oxidase 2, we performed ddPCR using either EvaGreen Supermix (Bio-Rad) or TaqMan Assay (Bio-Rad) for mature miR827, according to the protocols previously described [48, 99]. TaqMan Small RNA Assay ID 008386_mat (Thermo Fisher Scientific) was used to detect and quantify mature 3′ miR827 molecule (sequence ID: 2073). To normalize the copy number of miR827, we ran ddPCR for the *ARF1* reference gene using the TaqMan Assay ID: AIMSIL4 (Thermo Fisher Scientific). Absolute gene expression was shown as normalized copy number per 1000 copies of the barley *ARF1* reference gene. All specific primers and probes (mature miR827, U6 snRNA) used in this paper are listed in Additional file 31.

### Northern blot of mature miR827

To determine the mature miR827 expression level, we performed northern blot hybridization using a specific probe for analysis. All steps of these experiments were done according to a detailed protocol as described previously [65]. 10 μg of each RNA sample was run alongside a radioactively labelled Decade Marker (Invitrogen, Thermo Scientific) on a 15% polyacrylamide gel with 8 M urea. The miR827and U6 probe sequences are available in Additional file 31. The Decade Marker (Ambion) was loaded to control the length of the tested RNAs. Original blots are presented in Additional files 32 and 33. To calculate band intensity, we used the ImageQuant TL 8.1 software (GE Healthcare Life Sciences).

## Supplementary Information

**Additional file 1 **Normalized copy numbers of barley *IPS1* gene transcript in low-Pi treated root material. DdPCR was performed to examine the absolute gene expression of the barley *IPS1* gene. Obtained copy numbers were normalized per 1000 copies of the *ARF1* reference gene transcript. Asterisks indicate a significant differences (*p-value < 0.05) calculated using two-tailed Student’s *t*-tests.

**Additional file 2.** Characteristic of reads obtained from small RNA deep sequencing.

**Additional file 3 **MicroRNAs and small RNAs (other) for which expression is significantly changed during Pi-starvation in barley roots and shoots. ID numbers 1–138: miRNAs identified in roots (*p*-value < 0.05); ID numbers 139–1934: other small RNAs identified in roots (Bonferroni *p*-value correction < 0.05); ID numbers 1935–2096: miRNAs identified in shoots (*p*-value < 0.05); ID numbers 2097–2295: other small RNAs identified in shoots (Bonferroni *p*-value correction < 0.05). Samples R4–R6 = low-Pi root; R16–R18 = control; S4–S6 = low-Pi shoot; S16–S18 = control shoot. NaN means “Not a Number”, describing molecules that were exclusively expressed in low-Pi or control samples. Yellow color marks DEMs with Bonferroni adjusted *p*-value < 0.05. Data created using CLC Genomics Workbench.

**Additional file 4.** Identification of differentially expressed miRNAs (DEMs) in barley plants under low-Pi regime. The graph illustrates step-by-step annotation of unique small RNAs obtained in this study. The table summarizes the ShortStack output data.

**Additional file 5 **List of differentially expressed other small RNAs (ID 2097–2295) in barley shoots (low-Pi vs. control) identified in this study (Bonferroni corrected *p*-value < 0.05). Based on the available Ensembl Plants database, we classified each sequence into best-matching functional classes of cDNAs. DESs were also mapped to miRbase allowing 1, 2 or 3 mismatches.

**Additional file 6 **List of differentially expressed other small RNAs (ID 139–1934) in barley roots (low-Pi vs. control) identified in this study (Bonferroni corrected *p*-value < 0.05). Based on the available Ensembl Plants database, we classified each sequence into best-matching functional classes of cDNAs. DESs were also mapped to miRbase allowing 1, 2 or 3 mismatches.

**Additional file 7.** Length distribution of DESs identified in barley roots and shoots.

**Additional file 8.** Annotation distribution of DESs identified in barley roots and shoots.

**Additional file 9.** The output of DES ShortStack analysis (upper panel) and RNA secondary structure visualization of potential new miRNA generated by forna tool (lower panel). Red color marks miRNA; yellow color marks miRNA star.

**Additional file 10.** Chromosomal mapping of 98 DEGs identified in this study. Lower panel illustrates the percentage between quantitative distribution of either up-regulated or down-regulated genes under low-Pi conditions and total number of protein-coding genes in each barley chromosome. Scale bar for chromosomes = 160 Mbp.

**Additional file 11.** The extracted data from GO analysis for 98 DEGs used as a query.

**Additional file 12 **Upstream sequences (2 kb) extracted from all 98 DEGs used for *cis*-regulatory motif prediction analysis.

**Additional file 13.** List of identified P1BS motifs within the DEG promoters.

**Additional file 14.** List of identified P-responsive PHO motifs within the DEG promoters.

**Additional file 15.** Degradome profile (TargetSeek approach) demonstrates potential mRNA targets for differentially expressed miRNAs (DEMs) identified in barley shoots (low-Pi vs. control). The lower the alignment score, the more reliable the prediction.

**Additional file 16.** Degradome profile (TargetSeek approach) demonstrates potential mRNA targets for differentially expressed other sRNAs (DESs) identified in barley shoots (low-Pi vs. control). The lower the alignment score, the more reliable the prediction.

**Additional file 17.** Degradome profile (TargetSeek approach) demonstrates potential mRNA targets for differentially expressed miRNAs (DEMs) identified in barley roots (low-Pi vs. control). The lower the alignment score, the more reliable the prediction.

**Additional file 18.** Degradome profile (TargetSeek approach) demonstrates potential mRNA targets for differentially expressed other sRNAs (DESs) identified in barley roots (low-Pi vs. control). The lower the alignment score, the more reliable the prediction.

**Additional file 19.** Degradome profile (PAREsnip2 approach) demonstrates potential mRNA targets for differentially expressed miRNAs (DEMs) identified in barley shoots (low-Pi vs. control). The lower the alignment score, the more reliable the prediction.

**Additional file 20.** The t-plots generated by PAREsnip2 software showing the potential mRNA targets for differentially expressed miRNAs (DEMs) identified in barley shoots (low-Pi vs. control).

**Additional file 21.** Degradome profile (PAREsnip2 approach) demonstrates potential mRNA targets for differentially expressed other sRNAs (DESs) identified in barley shoots (low-Pi vs. control). The lower the alignment score, the more reliable the prediction.

**Additional file 22.** The t-plots generated by PAREsnip2 software showing the potential mRNA targets for differentially expressed other sRNAs (DESs) identified in barley shoots (low-Pi vs. control).

**Additional file 23.** Degradome profile (PAREsnip2 approach) demonstrates potential mRNA targets for differentially expressed miRNAs (DEMs) identified in barley roots (low-Pi vs. control). The lower the alignment score, the more reliable the prediction.

**Additional file 24.** The t-plots generated by PAREsnip2 software showing the potential mRNA targets for differentially expressed miRNAs (DEMs) identified in barley roots (low-Pi vs. control).

**Additional file 25.** Degradome profile (PAREsnip2 approach) demonstrates potential mRNA targets for differentially expressed other sRNAs (DESs) identified in barley roots (low-Pi vs. control). The lower the alignment score, the more reliable the prediction.

**Additional file 26.** The t-plots generated by PAREsnip2 software showing the potential mRNA targets for differentially expressed other sRNAs (DESs) identified in barley roots (low-Pi vs. control).

**Additional file 27.** List of genes predicted in degradome analysis to be guided for cleavage by putative regulatory sRNAs (identified as DES) with best scoring matches.

**Additional file 28.** miRbase annotation report from CLC Workbench (QIAGEN) analysis of shoot small RNAs.

**Additional file 29.** miRbase annotation report from CLC Workbench (QIAGEN) analysis of root small RNAs.

**Additional file 30.** Spike-in quality control of RNA-Seq samples from barley shoots (low-Pi vs. control). Correlation between known and measured spike-in concentrations.

**Additional file 31.** List of primers and probes used in this study.

**Additional file 32.** Original, full-length blot of mature hvu-miR827 analysis. Lane 1: Decade™ Marker System (Invitrogen, Thermo Fisher Scientific); Lane 2: empty space (no sample loaded); Lane 3–5: RNA samples from root (Pi sufficient); Lane 6–8: shoot (Pi sufficient); Lane 9–11: root (low-Pi); Lane: 12–14: shoot (low-Pi).

**Additional file 33.** Original, full-length blot of U6 snRNA analysis. Lane 1: Decade™ Marker System (Invitrogen, Thermo Fisher Scientific); Lane 2: empty space (no sample loaded); Lane 3–5: RNA samples from root (Pi sufficient); Lane 6–8: shoot (Pi sufficient); Lane 9–11: root (low-Pi); Lane: 12–14: shoot (low-Pi).

## Data Availability

The datasets generated and/or analyzed during the current study have been submitted to GEO database (SuperSeries GSE145427 contain following SubSeries: degradome RNA-Seq - GSE145423, sRNA-Seq - GSE145425, shoot transcriptome mRNA-Seq - GSE145426). All accession numbers mentioned in this study or listed in the tables and additional files can be found in the open-access Ensembl Plants database (http://plants.ensembl.org) for the barley genome.

## Abbreviations

sRNA: Small RNA
siRNA: Small interfering RNA
Pi: Inorganic phosphate
NGS: Next-Generation Sequencing
RNA-Seq: RNA Sequencing
DEM: Differentially expressed miRNA
DES: Differentially expressed small RNA
DEG: Differentially expressed gene
GO: Gene ontology
miRBase: The microRNA database
BLAST: Basic Local Alignment Search Tool
ddPCR: Droplet digital Polymerase Chain Reaction
P1BS: PHR1 binding sequence
TF: Transcription factor
PARE: Parallel Analysis of RNA Ends
EDGE: Empirical analysis of Differential Gene Expression
FDR: False discovery rate
MFE: Minimum free energy
TPM: Transcripts per million
RPKM: Reads per kilo base of exon model per million mapped reads
