# Supplementary material for: Pi-starvation induced transcriptional changes in barley revealed by a comprehensive RNA-Seq and degradome analyses
Source: BMC Genomics. 2021 Mar 9;22:165. doi: 10.1186/s12864-021-07481-w (PMC7941915; doi:10.1186/s12864-021-07481-w)
Supplement: Supplementary file 1 — Additional file 1 Normalized copy numbers of barley IPS1 gene transcript in low-Pi treated root material. DdPCR was performed to examine the absolute gene expression of the barley IPS1 gene. Obtained copy numbers were normalized per 1000 copies of the ARF1 reference gene transcript. Asterisks indicate a significant differences (*p-value < 0.05) calculated using two-tailed Student’s t-tests. [file 12864_2021_7481_MOESM1_ESM.pdf]

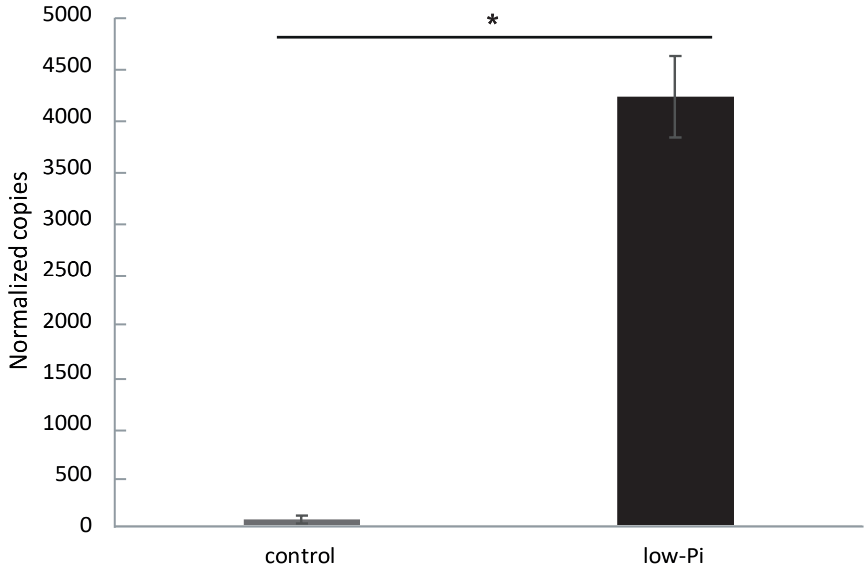

Additional file 1. Normalized copy numbers of barley *IPS1* gene in low-Pi treated root material.

DdPCR was performed to examine the absolute gene expression of the barley *IPS1* gene. Obtained copy numbers were normalized per 1000 copies of the *ARF1* reference gene. Asterisks indicate a significant differences (*$p < 0.05$) calculated using two-tailed Student's *t*-tests. Error bars = SD.
