# Supplementary material for: Pi-starvation induced transcriptional changes in barley revealed by a comprehensive RNA-Seq and degradome analyses
Source: BMC Genomics. 2021 Mar 9;22:165. doi: 10.1186/s12864-021-07481-w (PMC7941915; doi:10.1186/s12864-021-07481-w)
Supplement: Supplementary file 2 — Additional file 2. Characteristic of reads obtained from small RNA deep sequencing. [file 12864_2021_7481_MOESM2_ESM.pdf]

Additional file 2. Characteristic of reads obtained from small RNA deep sequencing

Sample[a]	Imported reads	18-25 reads trimmed[b]	Count[c]	Unique small RNAs
IDG1-1 R4	34735164	15551099	14999744	1300624
IDG1-2 R5	35333413	18255470	17614581	1399307
IDG1-3 R6	33730981	15633917	15074481	1386357
IDG1-4 S4	25840628	15397986	14644436	2878110
IDG1-5 S5	22518257	12862872	12168277	2246671
IDG1-6 S6	33960212	11083898	10565625	2053332
IDG1-7 R16	29891715	11801770	11434616	1479725
IDG1-8 R17	26274498	8450311	8091106	1145909
IDG1-9 R18	22516020	7744130	7470998	1069374
IDG1-10 S16	24148668	13971695	13414482	2672149
IDG1-11 S17	21928170	12710312	12249247	2403672
IDG1-12 S18	22971234	11013610	10640396	1882556

[a]Named after R4-R6 – root low-Pi; S4-S6 – shoot low-Pi; R16-R18 – root control-Pi; S16-S18 – shoot control-Pi

[b]Number of reads counted after adapter removal and length selection (18 nt – 25 nt). Reads is the oligonucleotide that has been sequences

[c]Counts are the number of reads that overlap at a particular genomic position
