# Supplementary material for: Pi-starvation induced transcriptional changes in barley revealed by a comprehensive RNA-Seq and degradome analyses
Source: BMC Genomics. 2021 Mar 9;22:165. doi: 10.1186/s12864-021-07481-w (PMC7941915; doi:10.1186/s12864-021-07481-w)
Supplement: Supplementary file 4 — Additional file 4. Identification of differentially expressed miRNAs (DEMs) in barley plants under low-Pi regime. The graph illustrates step-by-step annotation of unique small RNAs obtained in this study. The table summarizes the ShortStack output data. [file 12864_2021_7481_MOESM4_ESM.pdf]

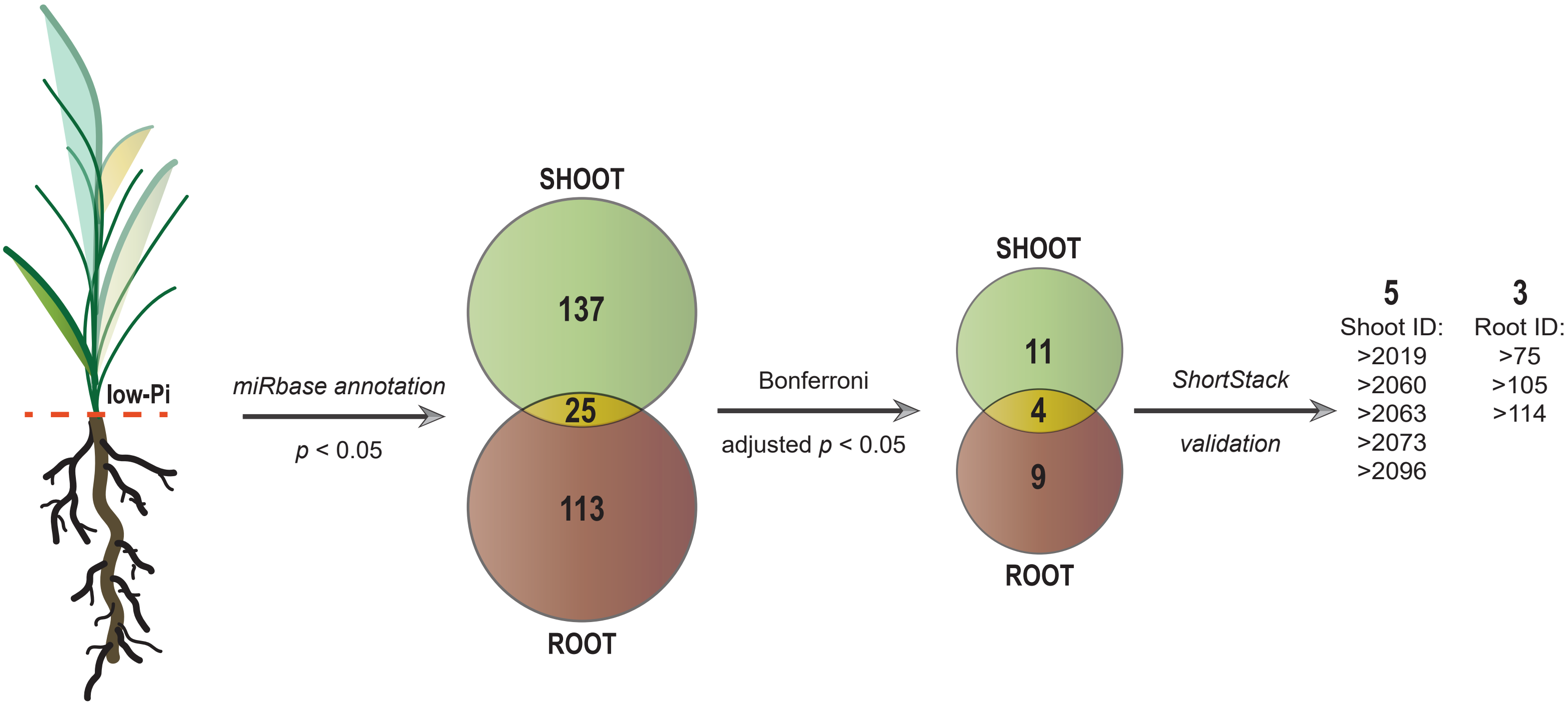

	ID	Name	Sequence (5'-3')	Prediction	Locus	Length	Reads	Strand	Major RNA reads	Complexity	Dicer Call
ROOT	>75	miR399b†	GGGCGCTTCTCCTTTGGCACG	+	chr4H:491992339-491992480	142	535	+	389	0.043	21
	>105	miR399a†	TGCCAAAGGAGAGTTGCCCTG	+	chr2H:666055710-666055856	147	2030	+	1813	0.016	21
	>114	miR827†	TTAGATGACCATCAGCAAACA	+	chr2H:620316212-620316424	213	1090	+	693	0.039	21
SHOOT	>2019	miR399b†	GGGCGCTTCTCCTTTGGCACG	+	chr4H:491992339-491992480	142	535	+	389	0.043	21
	>2060	miR399b//miR399a	TGCCAAAGGAGAATTGCCCTG	+	chr7H:540840753-540840845	93	1626	-	1143	0.009	21
	>2063	miR399a†	TGCCAAAGGAGAGTTGCCCTG	+	chr2H:666055710-666055856	147	2030	+	1813	0.016	21
	>2073	miR827†	TTAGATGACCATCAGCAAACA	+	chr2H:620316212-620316424	213	1090	+	693	0.039	21
	>2096	miR827	TTTTGTTGGTTGTCATCTAACC	+	chr2H:620316273-620316498	226	5203	+	2678	0.007	22

† = miRNA expressed in both organs

Additional file 4. Identification of differentially expressed miRNAs (DEMs) in barley plants under low-Pi regime. The graph illustrates step-by-step annotation of unique small RNAs obtained in this study. The table summarizes the ShortStack output data.
