# Supplementary material for: Pi-starvation induced transcriptional changes in barley revealed by a comprehensive RNA-Seq and degradome analyses
Source: BMC Genomics. 2021 Mar 9;22:165. doi: 10.1186/s12864-021-07481-w (PMC7941915; doi:10.1186/s12864-021-07481-w)
Supplement: Supplementary file 7 — Additional file 7. Length distribution of DESs identified in barley roots and shoots. [file 12864_2021_7481_MOESM7_ESM.pdf]

Additional file 7. Length distribution of DESs identified in barley roots and shoots

Length [nt]	Root	Root [%]	Shoot	Shoot [%]
18	274	15.26	28	14.07
19	216	12.03	42	21.11
20	207	11.53	28	14.07
21	247	13.75	26	13.07
22	219	12.19	19	9.55
23	201	11.19	17	8.54
24	196	10.91	32	16.08
25	236	13.14	7	3.52
	**Σ1796**	**=100%**	**Σ199**	**=100%**
