# Supplementary material for: Pi-starvation induced transcriptional changes in barley revealed by a comprehensive RNA-Seq and degradome analyses
Source: BMC Genomics. 2021 Mar 9;22:165. doi: 10.1186/s12864-021-07481-w (PMC7941915; doi:10.1186/s12864-021-07481-w)
Supplement: Supplementary file 8 — Additional file 8. Annotation distribution of DESs identified in barley roots and shoots. [file 12864_2021_7481_MOESM8_ESM.pdf]

Additional file 8. Annotation distribution of DESs identified in barley roots and shoots

cDNA class	Root	Root %	Shoot	Shoot %
Protein coding	890	38.54	101	47.87
total rRNA	789	34.17	18	8.53
5S rRNA	3	0.13	-	-
5.8S rRNA	42	1.82	1	0.47
16S rRNA	-	-	4	1.90
18S rRNA	18	0.78		0.00
26S rRNA	113	4.89	1	0.47
SSU rRNA	320	13.86	4	1.90
LSU rRNA	293	12.69	8	3.79
Non-translating CDS	450	19.49	77	36.49
snoRNA	59	2.56	1	0.47
tRNA	57	2.47	14	6.64
SRP_RNA	27	1.17	0	0.00
snRNA	22	0.95	0	0.00
pseudogene	15	0.65	0	0.00
	**Σ2309***	**=100%**	**Σ211***	**=100%**

*Overall number of DESs is higher (we identified 1796 DESs in roots and 199 DESs in shoots), cause some sequences were matching to more than one barley genomic locus and may represent several cDNA's classes

SSU = small subunit ribosomal, LSU = large subunit ribosomal
