# Supplementary material for: Pi-starvation induced transcriptional changes in barley revealed by a comprehensive RNA-Seq and degradome analyses
Source: BMC Genomics. 2021 Mar 9;22:165. doi: 10.1186/s12864-021-07481-w (PMC7941915; doi:10.1186/s12864-021-07481-w)
Supplement: Supplementary file 10 — Additional file 10. Chromosomal mapping of 98 DEGs identified in this study. Lower panel illustrates the percentage between quantitative distribution of either up-regulated or down-regulated genes under low-Pi conditions and total number of protein-coding genes in each barley chromosome. Scale bar for chromosomes = 160 Mbp. [file 12864_2021_7481_MOESM10_ESM.pdf]

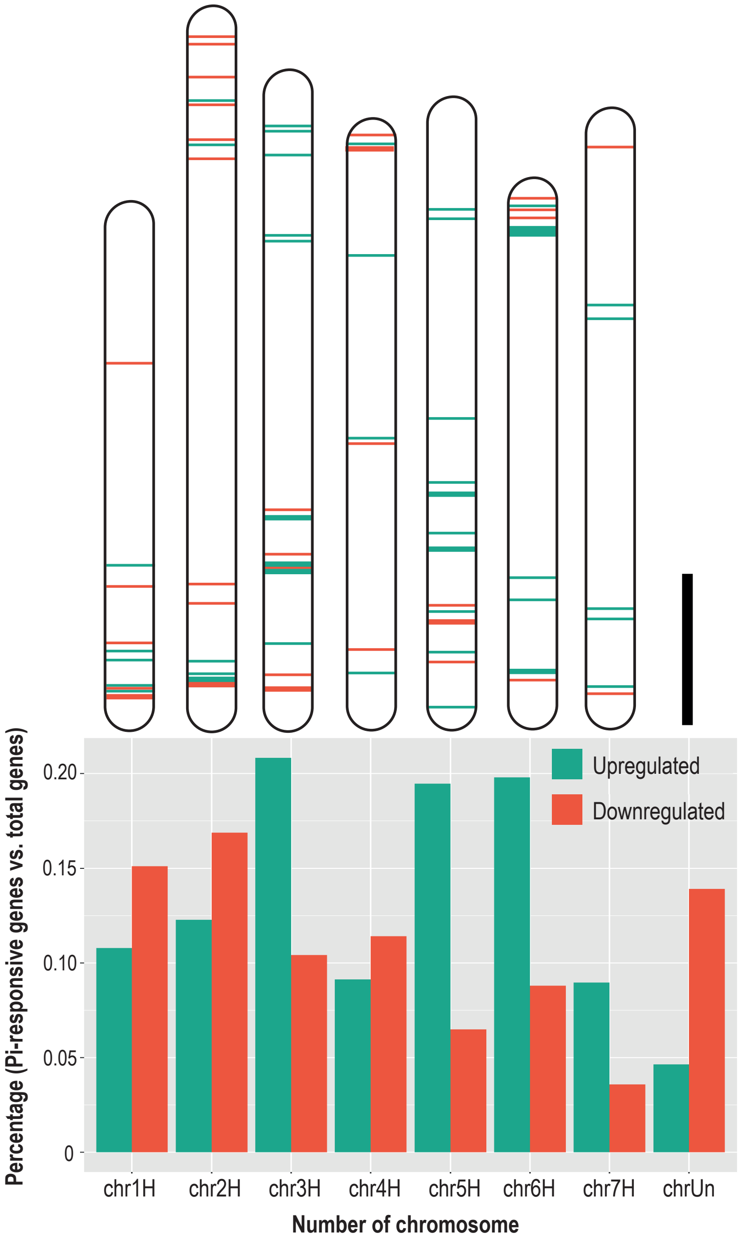

Additional file 10. Chromosomal mapping of 98 DEGs identified in this study. Lower panel illustrates the percentage between quantitative distribution of either up-regulated or down--regulated genes under low-Pi conditions and total number of protein-coding genes in each barley chromosome. Scale bar for chromosomes = 160 Mbp.
