# Supplementary material for: Pi-starvation induced transcriptional changes in barley revealed by a comprehensive RNA-Seq and degradome analyses
Source: BMC Genomics. 2021 Mar 9;22:165. doi: 10.1186/s12864-021-07481-w (PMC7941915; doi:10.1186/s12864-021-07481-w)

Additional file 13. List of identified P1BS motifs within the DEGs' promoters

#	Gene ID	FC	log_2(FC)	Function	Motif consensus	Position	Strand	Hit sequence
**1**	HORVU4Hr1G079600	59,41	5,89	IPS1	GNATATNC	1562	+	GGATATCC
	HORVU4Hr1G079600		5,89		GNATATNC	1694	+	GCATATCC
**2**	HORVU3Hr1G017440	18,54	4,21	Sn1-specific diacylglycerol lipase alpha isoform X1	GNATATNC	1828	+	GAATATCC
	HORVU3Hr1G017440		4,21		GNATATNC	1872	+	GGATATGC
**3**	HORVU3Hr1G068380	16,16	4,01	Inorganic pyrophosphatase 1-like	GNATATNC	1904	+	GAATATTC
**4**	HORVU7Hr1G113020	14,84	3,89	Hydrophobic protein RCI2A-like	GNATATNC	849	+	GGATATCC
	HORVU7Hr1G113020		3,89		GNATATNC	1923	+	GAATATTC
**5**	HORVU3Hr1G010540	13,35	3,74	Sulfoquinovosyl transferase SQD2-like	GNATATNC	1098	+	GCATATCC
	HORVU3Hr1G010540		3,74		GNATATNC	1132	+	GAATATCC
	HORVU3Hr1G010540		3,74		GNATATNC	1352	+	GAATATAC
**6**	HORVU6Hr1G007360	13,22	3,72	Uncharacterized protein	GNATATNC	188	+	GAATATCC
**7**	HORVU3Hr1G068390	11,17	3,48	Inorganic pyrophosphatase 1-like	GNATATNC	1811	+	GAATATTC
	HORVU3Hr1G068390		3,48		GNATATNC	1917	+	GCATATGC
**8**	HORVU2Hr1G112830	8,66	3,11	Endonuclease, S1/P1 nuclease	GNATATNC	1624	+	GCATATGC
**9**	HORVU2Hr1G116250	7,64	2,93	Alpha/beta hydrolase superfamily	GNATATNC	1992	+	GTATATCC
**10**	HORVU5Hr1G055570	6,08	2,60	Purple acid phosphatase	GNATATNC	1970	+	GAATATCC
**11**	HORVU3Hr1G078360	5,62	2,49	Purple acid phosphatase	GNATATNC	1726	+	GGATATAC
	HORVU3Hr1G078360		2,49		GNATATNC	1758	+	GGATATAC
	HORVU3Hr1G078360		2,49		GNATATNC	1827	+	GCATATGC
**12**	HORVU5Hr1G066460	5,61	2,49	Uncharacterized protein	GNATATNC	1632	+	GCATATAC
**13**	HORVU1Hr1G075570	4,89	2,29	Profilin	GNATATNC	1891	+	GAATATTC
**14**	HORVU5Hr1G055740	4,88	2,29	Carotenoid 9,10(9',10')-cleavage dioxygenase	GNATATNC	1946	+	GGATATTC
**15**	HORVU3Hr1G076060	4,42	2,14	Purple acid phosphatase	GNATATNC	1669	+	GCATATAC
**16**	HORVU5Hr1G051160	4,34	2,12	Probable Tyrosine-protein phosphatase, DSP4	GNATATNC	657	+	GAATATCC
**17**	HORVU2Hr1G110540	3,85	1,95	Molybdate transporter 2	GNATATNC	585	+	GGATATTC
**18**	HORVU1Hr1G054430	3,85	1,95	Haloacid dehalogenase-like hydrolase domain-containing	GNATATNC	1861	+	GCATATAC
**19**	HORVU5Hr1G097130	3,84	1,94	Peroxidase 57-like	GNATATNC	642	+	GAATATAC
**20**	HORVU5Hr1G079600	3,58	1,84	Uncharacterized protein	GNATATNC	733	+	GGATATTC
	HORVU5Hr1G079600		1,84		GNATATNC	741	+	GCATATCC
	HORVU5Hr1G079600		1,84		GNATATNC	1859	+	GCATATGC
**21**	HORVU6Hr1G058780	3,45	1,79	Phosphoenolpyruvate carboxylase kinase 2	GNATATNC	293	+	GAATATGC
	HORVU6Hr1G058780		1,79		GNATATNC	329	+	GAATATGC
	HORVU6Hr1G058780		1,79		GNATATNC	1670	+	GAATATTC
**22**	HORVU7Hr1G089910	3,44	1,78	SPX domain-containing protein 1-like	GNATATNC	857	+	GCATATGC
	HORVU7Hr1G089910		1,78		GNATATNC	1765	+	GAATATTC
	HORVU7Hr1G089910		1,78		GNATATNC	1795	+	GCATATCC
**23**	HORVU1Hr1G088920	3,36	1,75	Chlorophyll a-b binding protein of LHCII type 1-like	GNATATNC	249	+	GGATATCC
	HORVU1Hr1G088920		1,75		GNATATNC	1132	+	GAATATAC
	HORVU1Hr1G089620		1,75		GNATATNC	1480	+	GCATATGC
**24**	HORVU6Hr1G016890	3,35	1,75	Chlorophyll a-b binding protein, chloroplastic	GNATATNC	887	+	GTATATAC
**25**	HORVU3Hr1G076320	3,31	1,73	Inositol hexakisphosphate and diphosphoinositol-	GNATATNC	503	+	GAATATTC
**26**	HORVU3Hr1G029200	3,31	1,73	Phosphoenolpyruvate carboxylase 1-like	GNATATNC	734	+	GCATATGC
	HORVU3Hr1G029200		1,73		GNATATNC	754	+	GTATATGC
	HORVU3Hr1G029200		1,73		GNATATNC	1704	+	GAATATTC
**27**	HORVU5Hr1G044460	3,09	1,63	Purple acid phosphatase 5	GNATATNC	1267	+	GAATATTC
	HORVU5Hr1G044460		1,63		GNATATNC	1982	+	GAATATCC
**28**	HORVU5Hr1G106010	-2,63	-1,40	Pathogenesis-related protein 1-like	GNATATNC	4	+	GTATATCC
	HORVU5Hr1G106010		-1,40		GNATATNC	25	+	GTATATGC
	HORVU5Hr1G106010		-1,40		GNATATNC	721	+	GAATATCC
**29**	HORVU3Hr1G108100	-2,70	-1,43	Carotenoid 9,10(9',10')-cleavage dioxygenase 1	GNATATNC	1653	+	GTATATAC
**30**	HORVU5Hr1G077920	-2,82	-1,50	Beta-glucosidase 31-like	GNATATNC	383	+	GTATATTC
**31**	HORVU3Hr1G109350	-2,92	-1,54	Cysteine-rich receptor-like protein kinase 10	GNATATNC	400	+	GGATATTC
**32**	HORVU0Hr1G038320	-3,32	-1,73	Prolyl endopeptidase-like	GNATATNC	899	+	GTATATGC
**33**	HORVU2Hr1G012980	-3,92	-1,97	Premnaspirodiene oxygenase-like	GNATATNC	1849	+	GGATATGC
**34**	HORVU4Hr1G071300	-4,05	-2,02	Aminotransferase ALD1 homolog	GNATATNC	968	+	GTATATGC
**35**	HORVU4Hr1G001250	-4,14	-2,05	Caffeic acid-O-methyltransferase (COMT)	GNATATNC	1162	+	GGATATTC
**36**	HORVU4Hr1G044460	-4,24	-2,09	Potential Tubulin alpha-6 chain	GNATATNC	2	+	GAATATGC
	HORVU4Hr1G044460		-2,09		GNATATNC	1347	+	GTATATGC
**37**	HORVU1Hr1G081410	-5,24	-2,39	Chaperone protein dnaJ C76, chloroplastic isoform X1	GNATATNC	284	+	GCATATGC
**38**	HORVU7Hr1G116080	-5,70	-2,51	Endoplasmic reticulum metallopeptidase 1	GNATATNC	1316	+	GCATATTC
**39**	HORVU2Hr1G094690	-5,97	-2,58	OsSPX -MFS1 ortholog (targeted by miR827)	GNATATNC	1823	+	GTATATGC
**40**	HORVU6Hr1G005720	-6,05	-2,60	High-affinity nitrate transporter 2.1-like	GNATATNC	291	+	GCATATAC
	HORVU6Hr1G005720		-2,60		GNATATNC	345	+	GCATATAC
**41**	HORVU1Hr1G070220	-6,09	-2,61	Pyridoxal phosphate-dependent transferase	GNATATNC	1458	+	GAATATAC
	HORVU1Hr1G070220		-2,61		GNATATNC	1506	+	GAATATAC
	HORVU1Hr1G070220		-2,61		GNATATNC	1763	+	GAATATTC
**42**	HORVU4Hr1G005440	-6,13	-2,62	Oxalate oxidase	GNATATNC	728	+	GTATATAC
**43**	HORVU2Hr1G006830	-6,15	-2,62	Cytochrome P450	GNATATNC	9	+	GAATATGC
**44**	HORVU2Hr1G031400	10,87	-3,44	SPX domain-containing protein 6-like	GNATATNC	599	+	GTATATTC
**45**	HORVU2Hr1G020140	13,90	-3,80	Transcription factor NIGT1	GNATATNC	1693	+	GGATATTC
**46**	HORVU4Hr1G005380	-21,29	-4,41	Oxalate oxidase 2	GNATATNC	1579	+	GAATATTC

log_2(fold change)

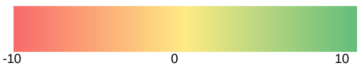

Supplement: Supplementary file 13 — Additional file 13. List of identified P1BS motifs within the DEG promoters. [file 12864_2021_7481_MOESM13_ESM.pdf]
