# Supplementary material for: Pi-starvation induced transcriptional changes in barley revealed by a comprehensive RNA-Seq and degradome analyses
Source: BMC Genomics. 2021 Mar 9;22:165. doi: 10.1186/s12864-021-07481-w (PMC7941915; doi:10.1186/s12864-021-07481-w)

Additional file 14. List of identified P-responsive PHO motifs within the DEGs' promoters

#	Gene ID	FC	log_2(FC)	Function	Motif consensus	Position	Strand	Hit sequence
**1**	HORVU3Hr1G079900	54,85	5,78	Glycerophosphodiester phosphodiesterase GDPD1, chloroplastic-like	ATGCCAT	391	+	ATGCCAT
**2**	HORVU3Hr1G091170	19,72	4,30	Inactive protein kinase	ATGCCAT	1188	+	ATGCCAT
	HORVU3Hr1G091170		4,30		ATGCCAT	1783	+	ATGCCAT
**3**	HORVU3Hr1G010540	13,35	3,74	Sulfoquinovosyl transferase SQD2-like	ATGCCAT	220	+	ATGCCAT
**4**	HORVU2Hr1G031400	10,87	3,44	SPX domain-containing protein 6-like	ATGCCAT	644	+	ATGCCAT
**5**	HORVU6Hr1G065710	6,89	2,79	OsSPX-MFS2 ortholog	ATGCCAT	128	+	ATGCCAT
**6**	HORVU5Hr1G055740	4,88	2,29	Carotenoid 9,10(9',10')-cleavage dioxygenase	ATGCCAT	1466	+	ATGCCAT
**7**	HORVU7Hr1G091060	4,76	2,25	TLC domain-containing protein	ATGCCAT	1379	+	ATGCCAT
**8**	HORVU5Hr1G028140	4,24	2,08	Heptahelical transmembrane protein 4	ATGCCAT	1408	+	ATGCCAT
**9**	HORVU1Hr1G089620	3,36	1,75	Chlorophyll a-b binding protein of LHCII type 1-like	ATGCCAT	197	+	ATGCCAT
**10**	HORVU3Hr1G029200	3,31	1,73	Phosphoenolpyruvate carboxylase 1-like	ATGCCAT	18	+	ATGCCAT
	HORVU3Hr1G029200		1,73		ATGCCAT	1743	+	ATGCCAT
**11**	HORVU5Hr1G044460	3,09	1,63	Purple acid phosphatase 5	ATGCCAT	480	+	ATGCCAT
**12**	HORVU3Hr1G108100	-2,70	-1,43	Carotenoid 9,10(9',10')-cleavage dioxygenase 1	ATGCCAT	1870	+	ATGCCAT
**13**	HORVU3Hr1G109350	-2,92	-1,54	Cysteine-rich receptor-like protein kinase 10	ATGCCAT	1968	+	ATGCCAT
**14**	HORVU3Hr1G077950	-3,76	-1,91	Subtilisin-like protease SBT3.8	ATGCCAT	7	+	ATGCCAT
**15**	HORVU1Hr1G070220	-6,09	-2,61	Pyridoxal phosphate-dependent transferase	ATGCCAT	1208	+	ATGCCAT
**16**	HORVU1Hr1G089540	-8,29	-3,05	Chalcone synthase 2	ATGCCAT	120	+	ATGCCAT
**17**	HORVU2Hr1G030090	-90,52	-6,50	Uncharacterized protein	ATGCCAT	1314	+	ATGCCAT
	HORVU2Hr1G030090		-6,50		ATGCCAT	1662	+	ATGCCAT
	HORVU2Hr1G030090		-6,50		ATGCCAT	1700	+	ATGCCAT

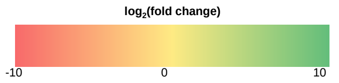

Supplement: Supplementary file 14 — Additional file 14. List of identified P-responsive PHO motifs within the DEG promoters. [file 12864_2021_7481_MOESM14_ESM.pdf]
