# Supplementary material for: Pi-starvation induced transcriptional changes in barley revealed by a comprehensive RNA-Seq and degradome analyses
Source: BMC Genomics. 2021 Mar 9;22:165. doi: 10.1186/s12864-021-07481-w (PMC7941915; doi:10.1186/s12864-021-07481-w)
Supplement: Supplementary file 20 — Additional file 20. The t-plots generated by PAREsnip2 software showing the potential mRNA targets for differentially expressed miRNAs (DEMs) identified in barley shoots (low-Pi vs. control). [file 12864_2021_7481_MOESM20_ESM.pdf]

**Additional file 20.** The t-plots generated by PAREsnip2 software showing the potential mRNA targets for differentially expressed miRNAs (DEMs) identified in barley shoots (low-Pi vs. control).

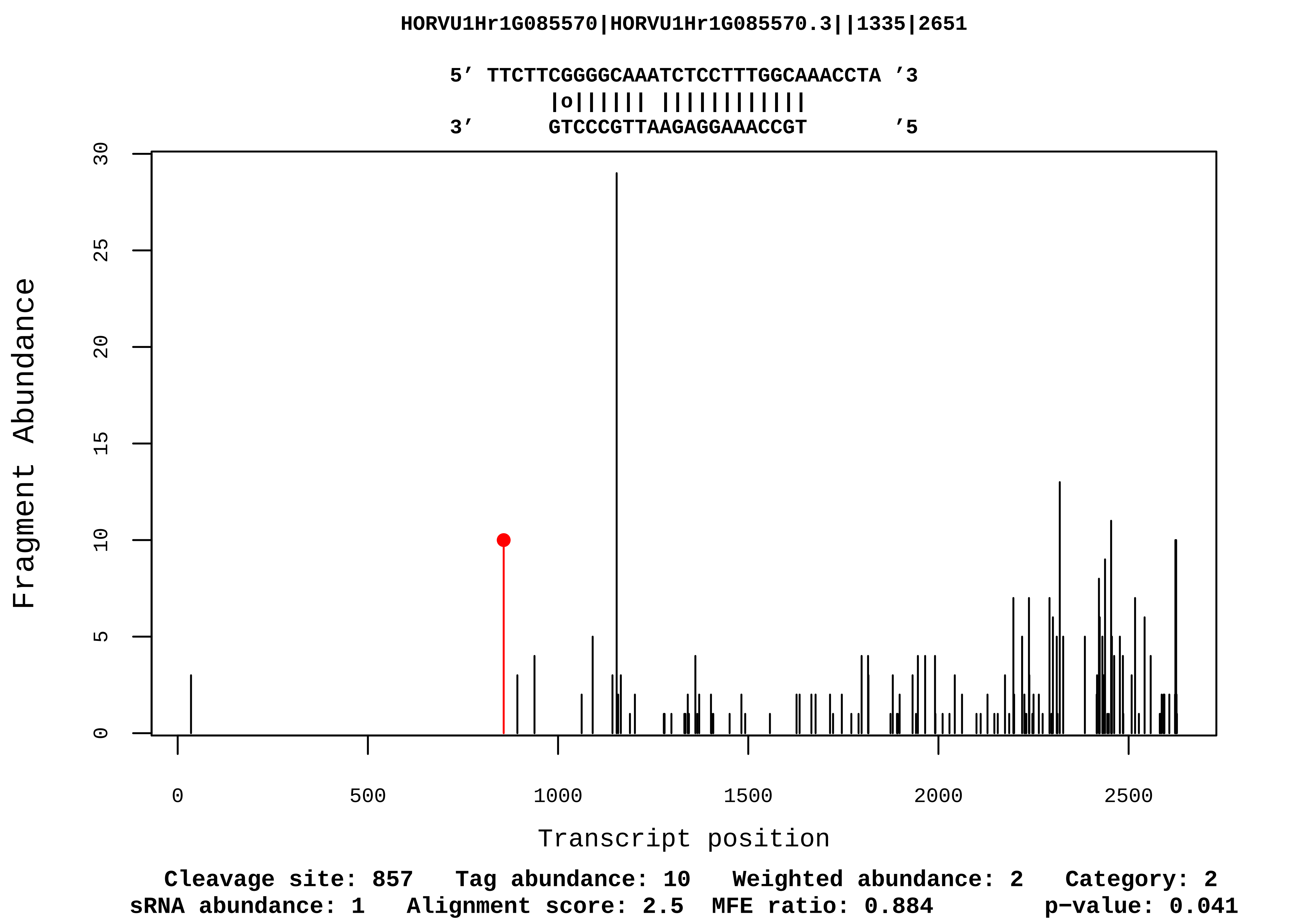
HORVU1Hr1G085570|HORVU1Hr1G085570.3||1335|2651
5′ TTCTTCGGGGCAAATCTCCTTTGGCAAACCTA ′3
|o|||||| ||||||||||||
3′ GTCCCGTTAAGAGGAAACCGT ′5
Fragment Abundance
0
5
10
15
20
25
30
0
500
1000
1500
2000
2500
Transcript position
Cleavage site: 857 Tag abundance: 10 Weighted abundance: 2 Category: 2
sRNA abundance: 1 Alignment score: 2.5 MFE ratio: 0.884 p−value: 0.041

HORVU1Hr1G085570|HORVU1Hr1G085570.3||1335|2651

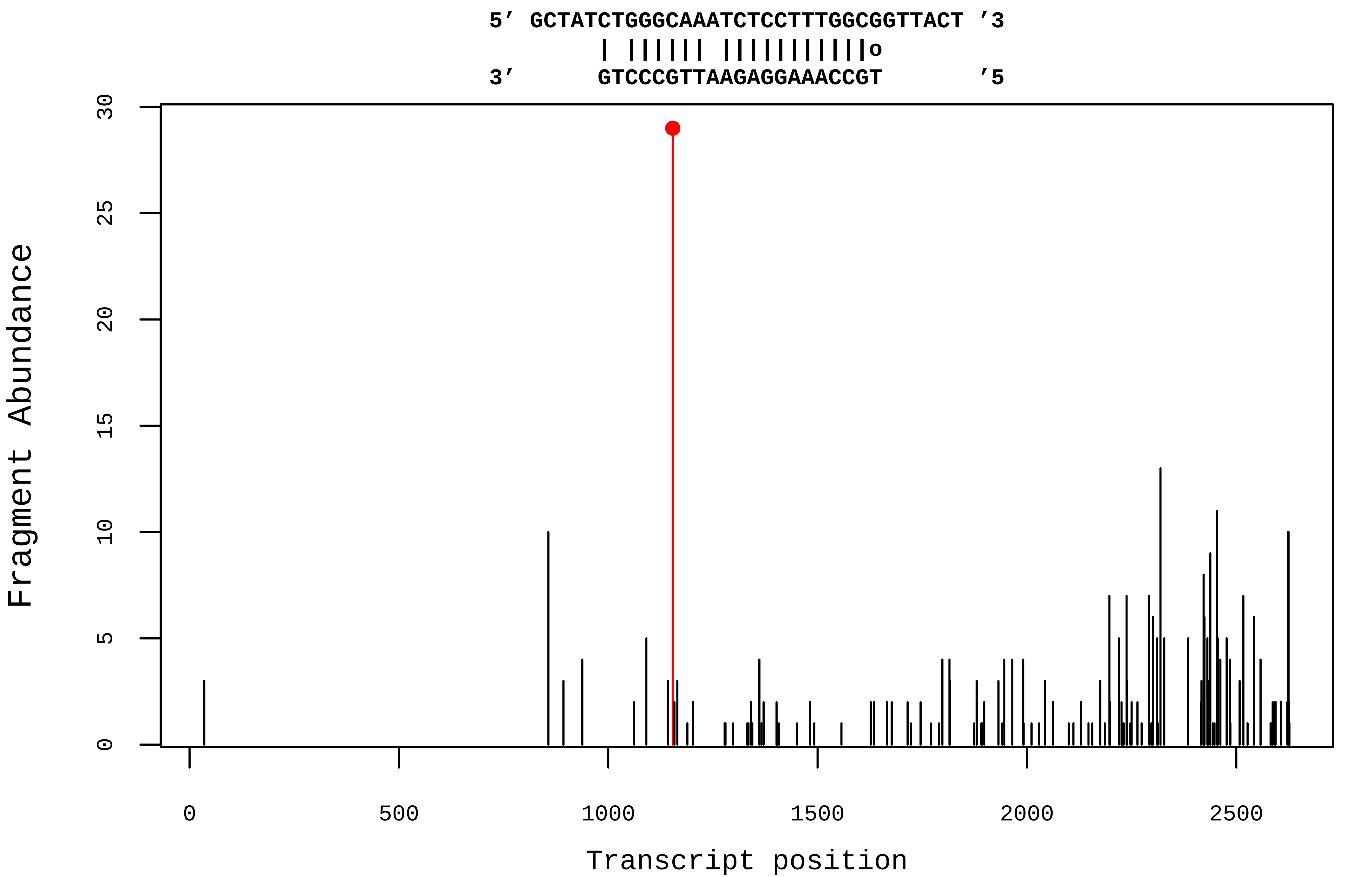


**Cleavage site: 1154 Tag abundance: 29 Weighted abundance: 5.8 Category: 0**
**sRNA abundance: 1 Alignment score: 3.5 MFE ratio: 0.823 p-value: 0.006**

**HORVU1Hr1G085570|HORVU1Hr1G085570.3||1335|2651**

```
5’ TTCTTCGGGGCAAATCTCCTTTGGCAAACCTA ’3
           |o|||||| ||||||||||||
3’         GTCCCGTTGAGAGGAAACCGT       ’5
```

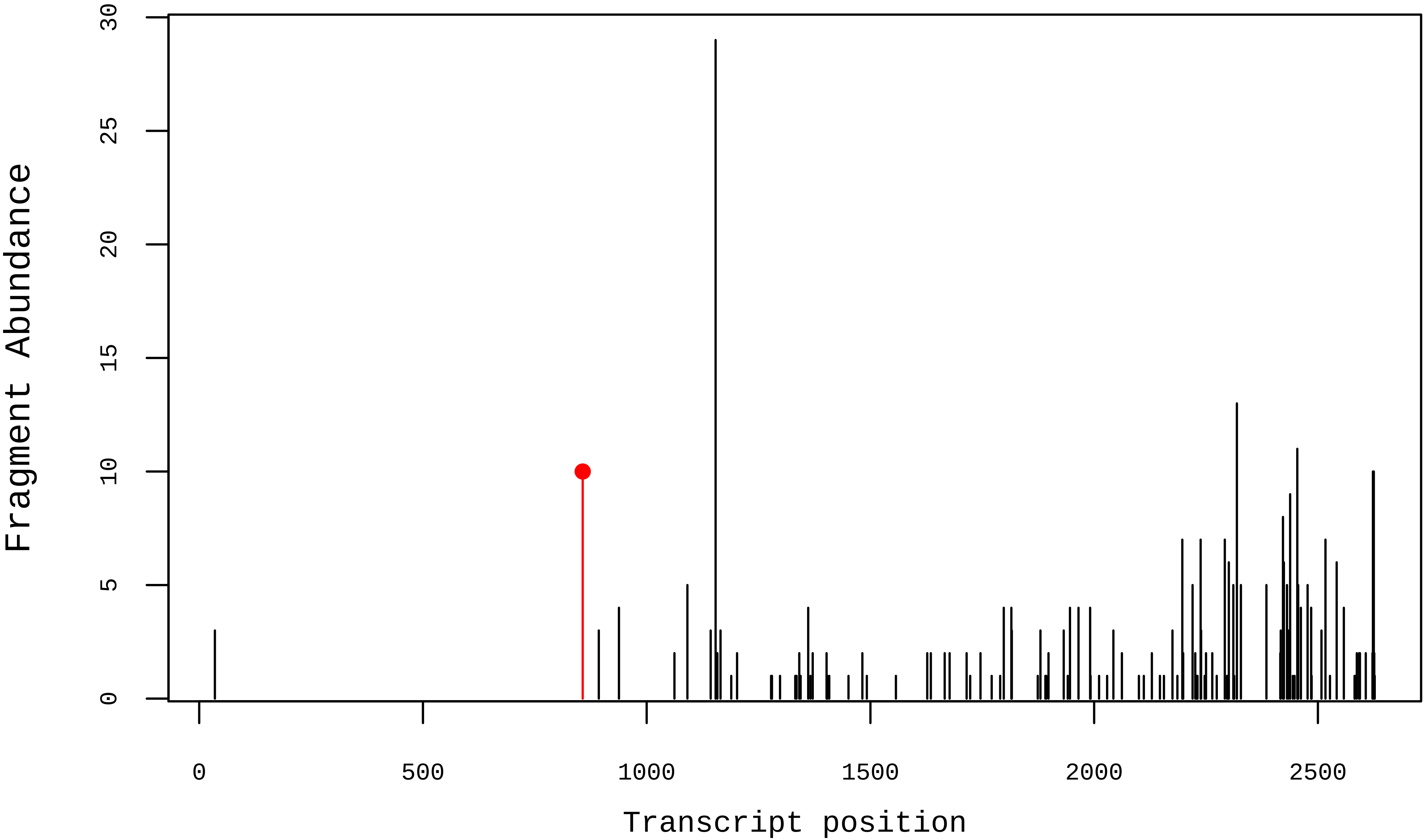


**Cleavage site: 857 Tag abundance: 10 Weighted abundance: 2 Category: 2**
**sRNA abundance: 1 Alignment score: 2.5 MFE ratio: 0.835 p-value: 0.041**

**HORVU1Hr1G085570|HORVU1Hr1G085570.3||1335|2651**

```
5' GCTATCTGGGCAAATCTCCTTTGGCGGTTACT '3
         | |||||| |||||||||||o
3'       GTCCCGTTGAGAGGAAACCGT       '5
```

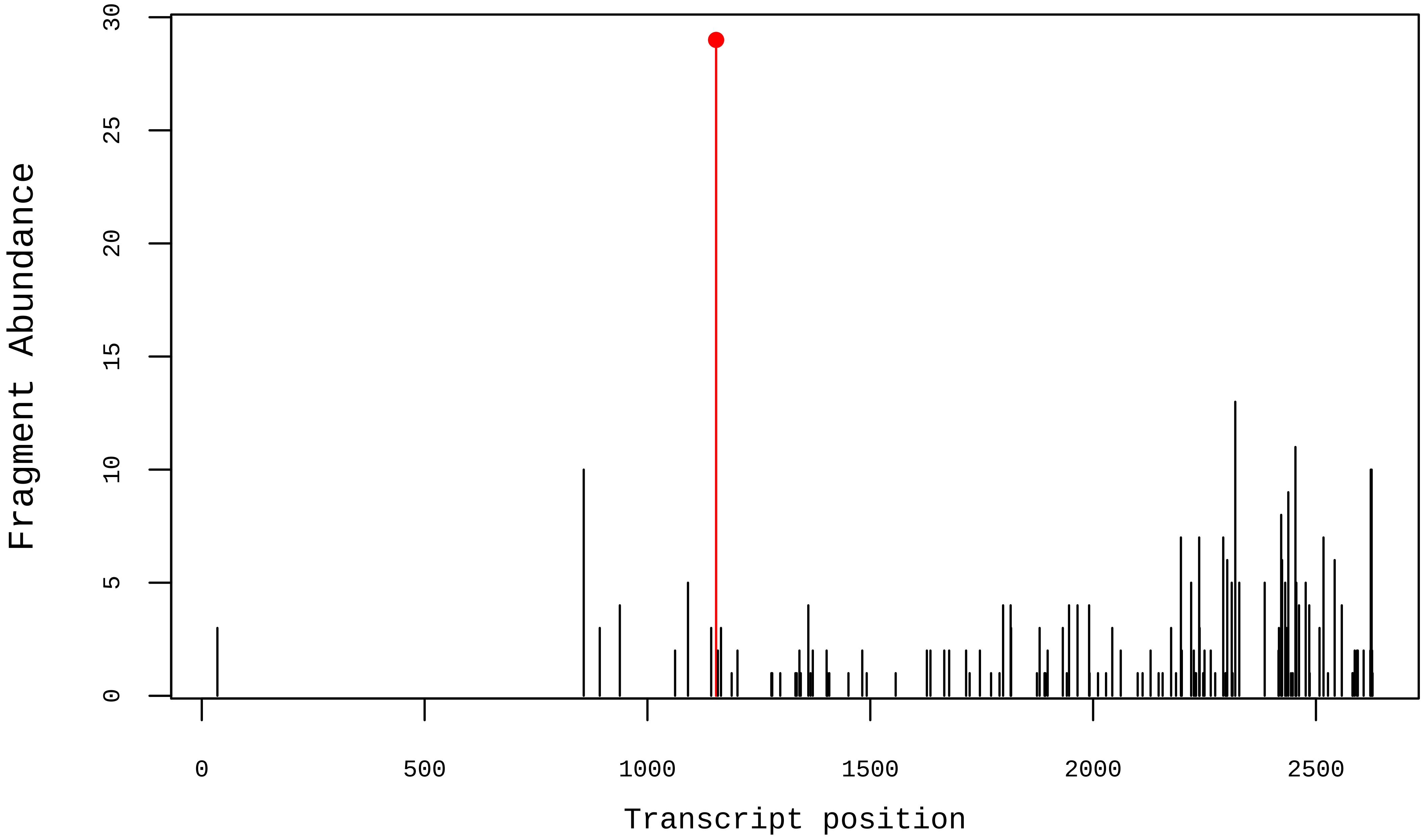


**Cleavage site: 1154   Tag abundance: 29   Weighted abundance: 5.8   Category: 0**
**sRNA abundance: 1   Alignment score: 3.5   MFE ratio: 0.778   p-value: 0.004**

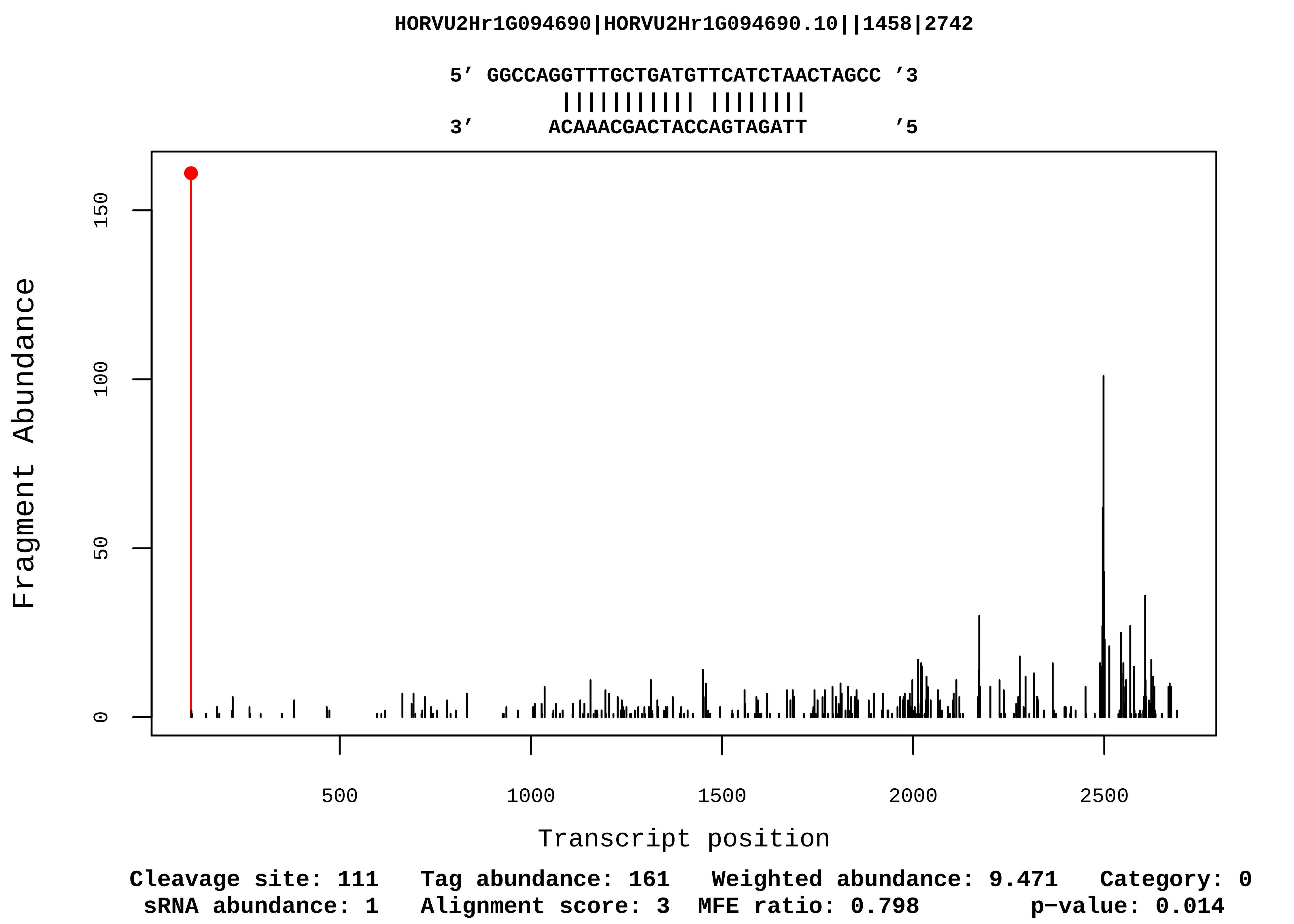
HORVU2Hr1G094690|HORVU2Hr1G094690.10||1458|2742
5′ GGCCAGGTTTGCTGATGTTCATCTAACTAGCC ′3
||||||||||| ||||||||
3′ ACAAACGACTACCAGTAGATT ′5
Fragment Abundance
0
50
100
150
500
1000
1500
2000
2500
Transcript position
Cleavage site: 111 Tag abundance: 161 Weighted abundance: 9.471 Category: 0
sRNA abundance: 1 Alignment score: 3 MFE ratio: 0.798 p-value: 0.014

HORVU2Hr1G094690|HORVU2Hr1G094690.11||1458|2742

```
5' GGCCAGGTTTGCTGATGTTCATCTAACTAGCC '3
         ||||||||||| ||||||||
3'      ACAAACGACTACCAGTAGATT       '5
```

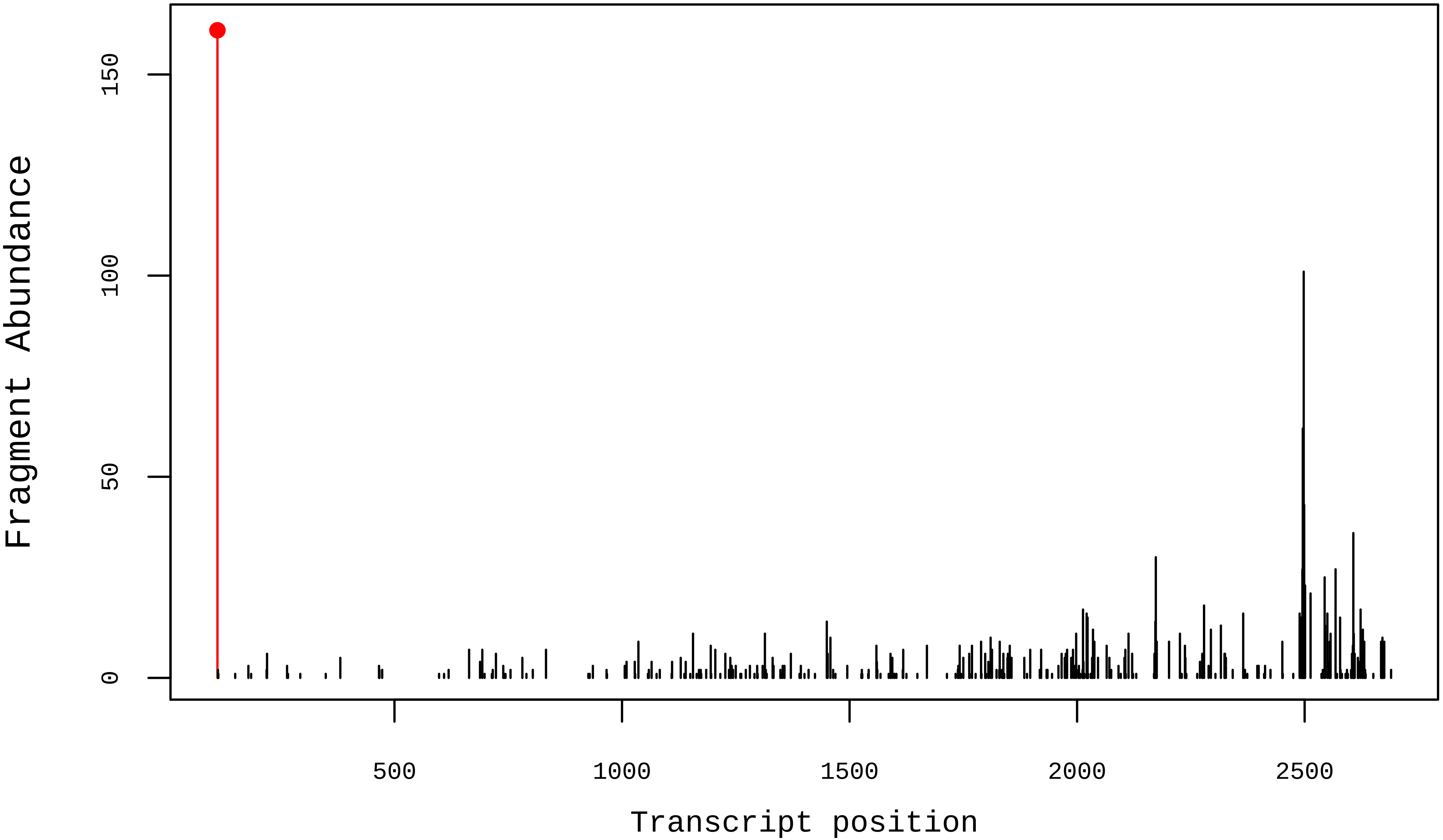


Cleavage site: 111   Tag abundance: 161   Weighted abundance: 9.471   Category: 0
sRNA abundance: 1   Alignment score: 3   MFE ratio: 0.798   p-value: 0.014

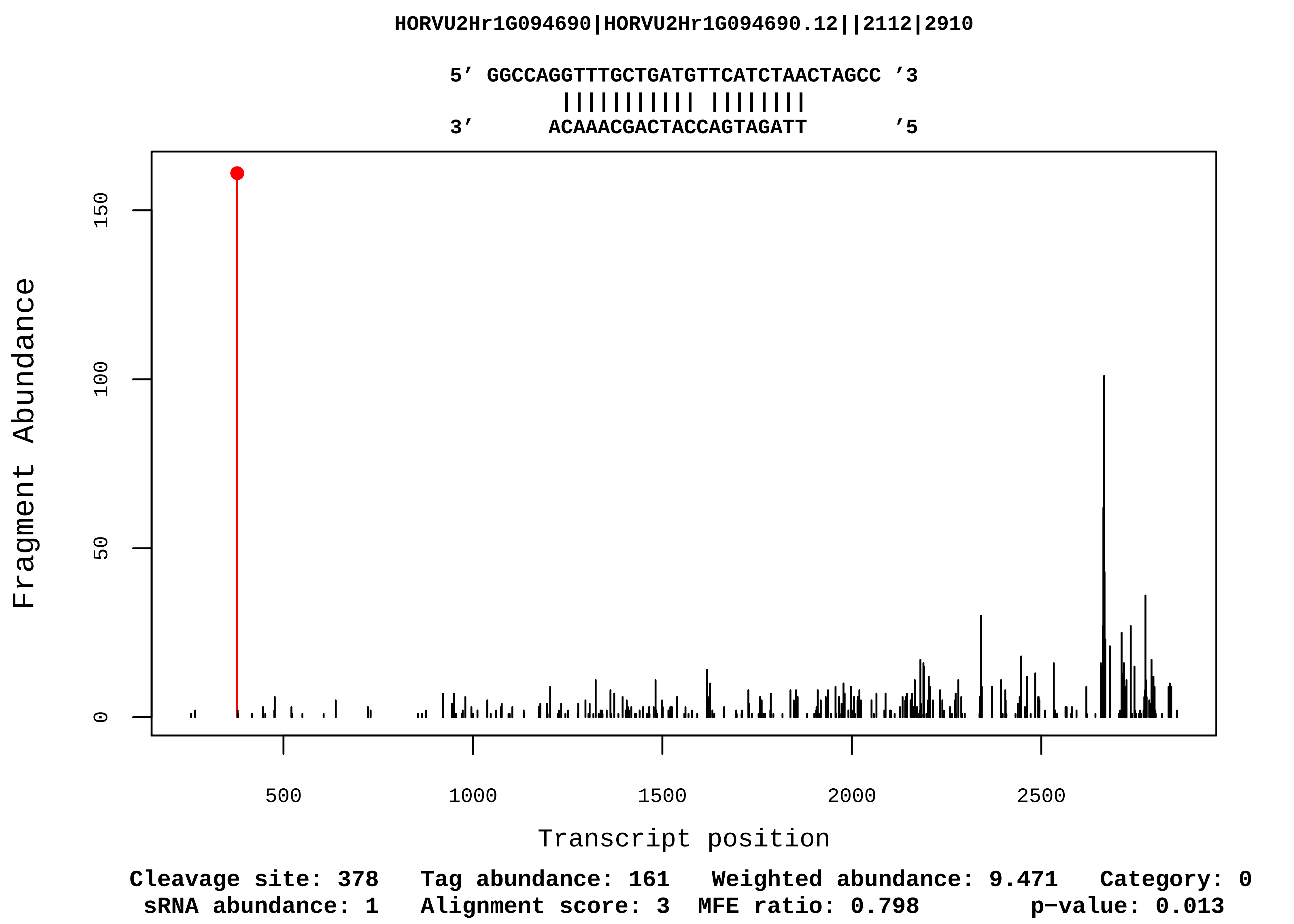
HORVU2Hr1G094690|HORVU2Hr1G094690.12||2112|2910
5' GGCCAGGTTTGCTGATGTTCATCTAACTAGCC '3
||||||||||| ||||||||
3' ACAAACGACTACCAGTAGATT '5
Fragment Abundance
0
50
100
150
500
1000
1500
2000
2500
Transcript position
Cleavage site: 378 Tag abundance: 161 Weighted abundance: 9.471 Category: 0
sRNA abundance: 1 Alignment score: 3 MFE ratio: 0.798 p-value: 0.013

HORVU2Hr1G094690|HORVU2Hr1G094690.13||2112|2916

```
5' GGCCAGGTTTGCTGATGTTCATCTAACTAGCC '3
         ||||||||||| ||||||||
3'       ACAAACGACTACCAGTAGATT        '5
```

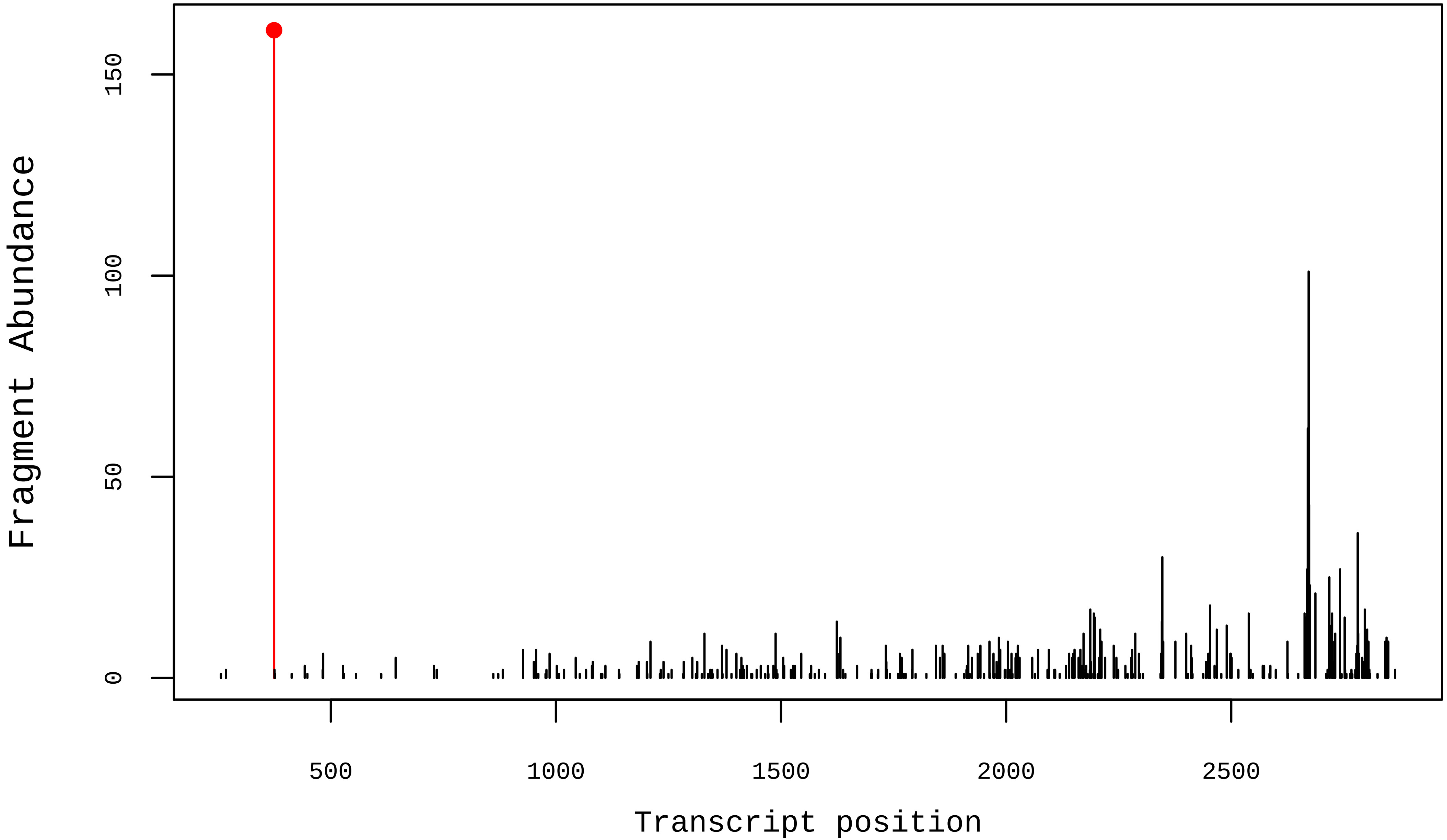


**Cleavage site: 374 Tag abundance: 161 Weighted abundance: 9.471 Category: 0**
**sRNA abundance: 1 Alignment score: 3 MFE ratio: 0.798 p-value: 0.013**

HORVU2Hr1G094690|HORVU2Hr1G094690.14||2112|2920

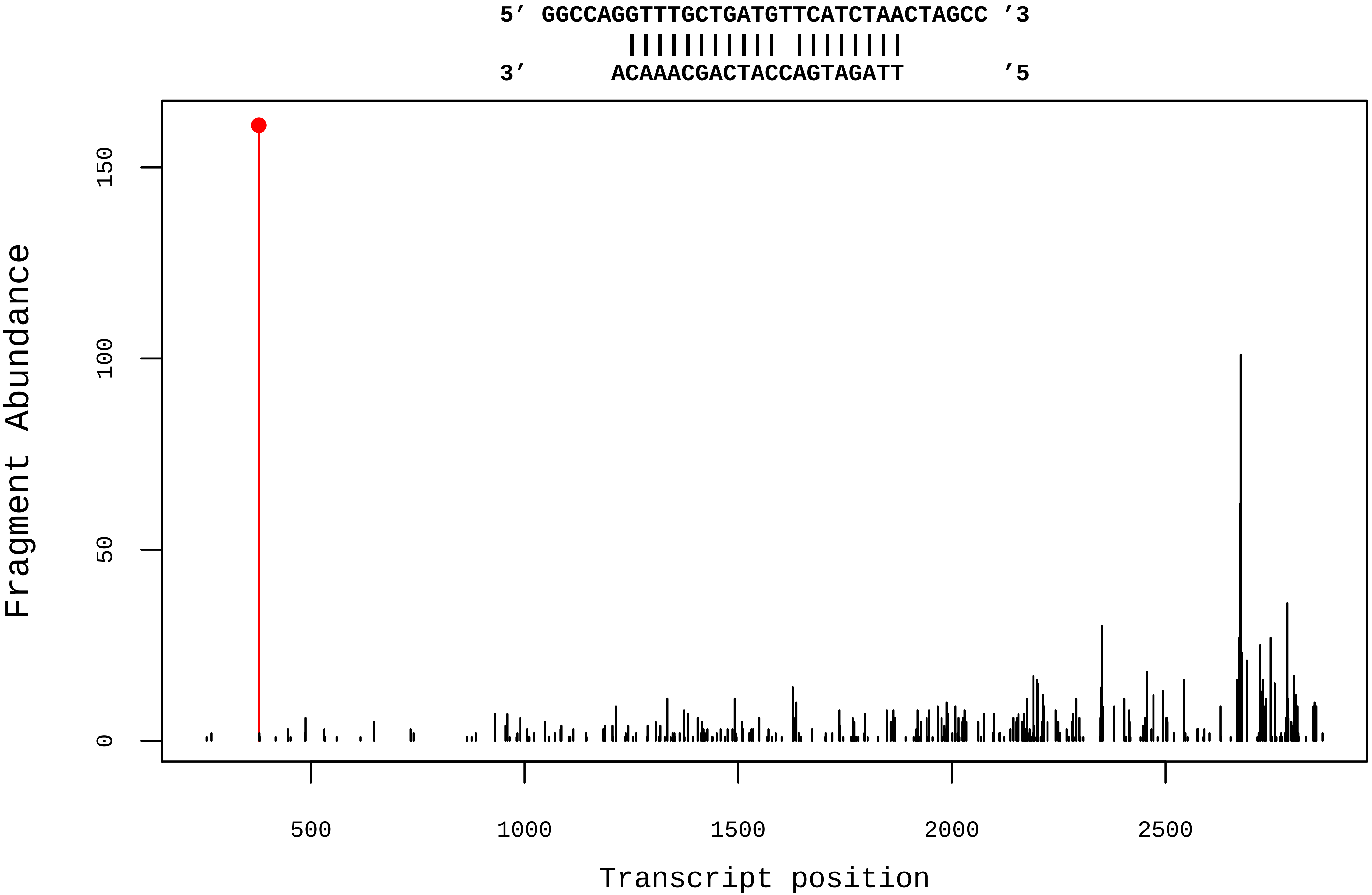


**Cleavage site: 378 Tag abundance: 161 Weighted abundance: 9.471 Category: 0**
**sRNA abundance: 1 Alignment score: 3 MFE ratio: 0.798 p-value: 0.013**

HORVU2Hr1G094690|HORVU2Hr1G094690.15||2112|2920

```
5' GGCCAGGTTTGCTGATGTTCATCTAACTAGCC '3
        |||||||||||| |||||||||
3'      ACAAACGACTACCAGTAGATT       '5
```

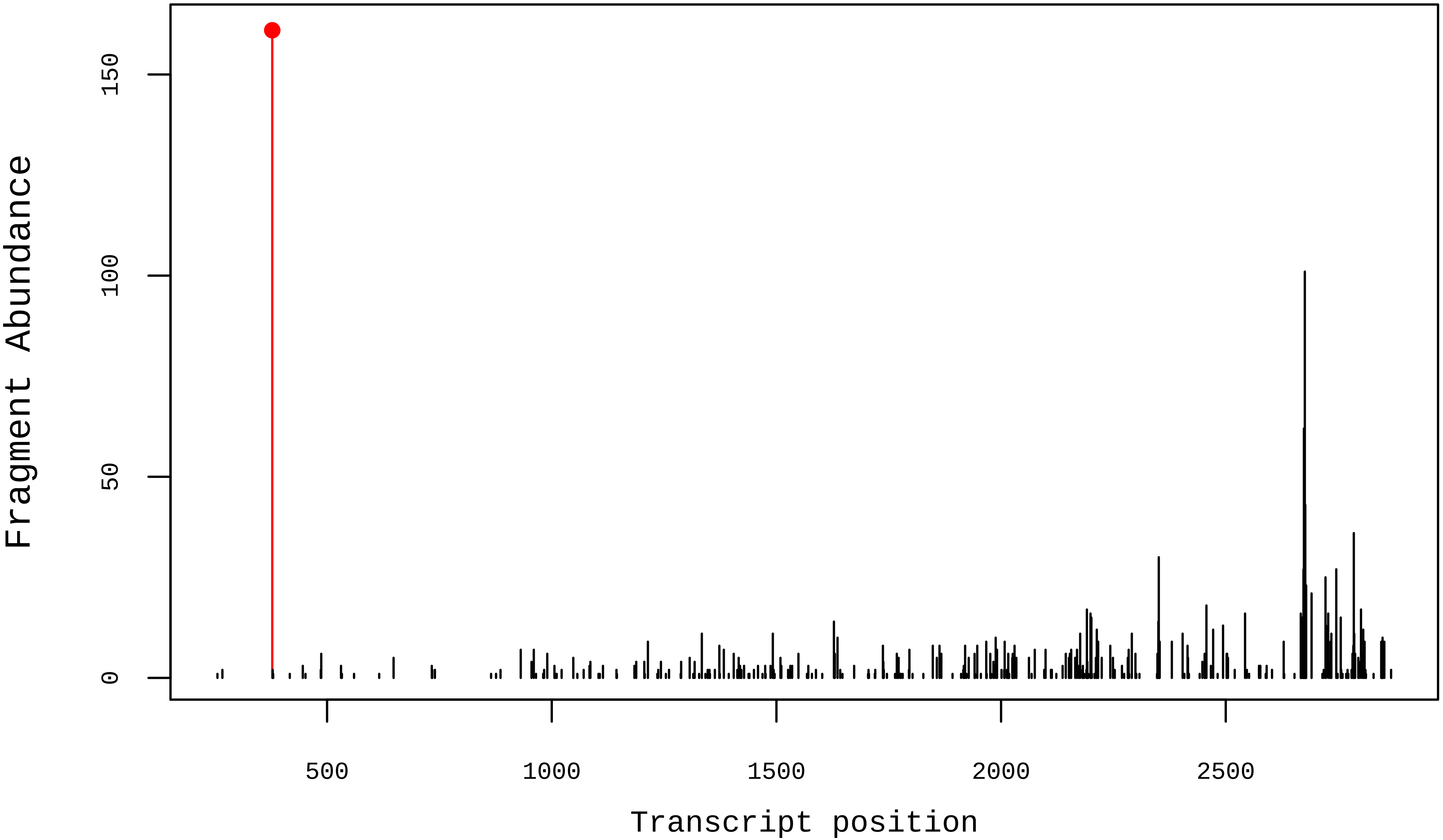


**Cleavage site: 378   Tag abundance: 161   Weighted abundance: 9.471   Category: 0**
**sRNA abundance: 1   Alignment score: 3   MFE ratio: 0.798   p-value: 0.013**

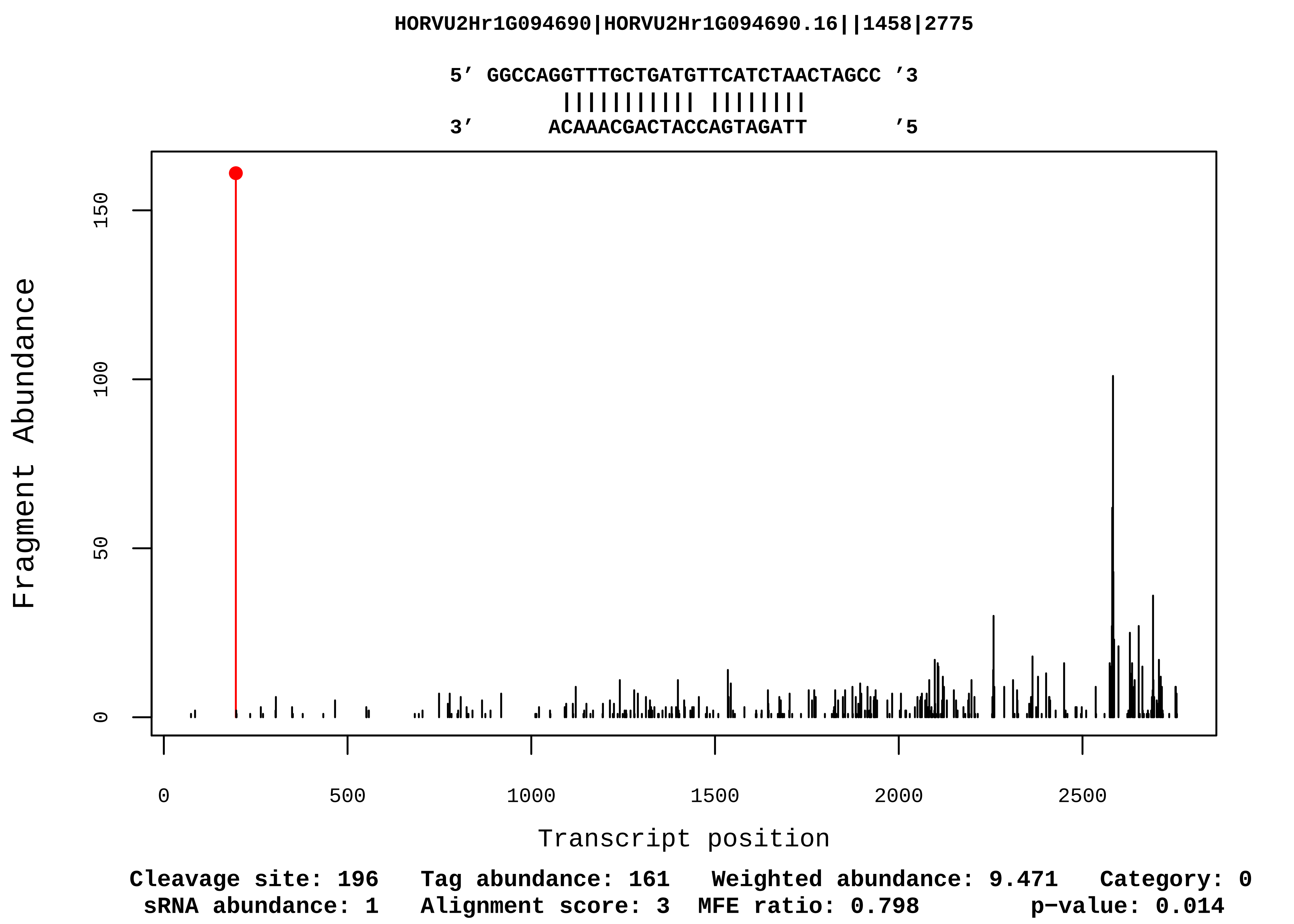
HORVU2Hr1G094690|HORVU2Hr1G094690.16||1458|2775
5′ GGCCAGGTTTGCTGATGTTCATCTAACTAGCC ′3
3′ ACAAACGACTACCAGTAGATT ′5
Fragment Abundance
0
50
100
150
0
500
1000
1500
2000
2500
Transcript position
Cleavage site: 196 Tag abundance: 161 Weighted abundance: 9.471 Category: 0
sRNA abundance: 1 Alignment score: 3 MFE ratio: 0.798 p-value: 0.014

HORVU2Hr1G094690|HORVU2Hr1G094690.17||1458|2771

```
5' GGCCAGGTTTGCTGATGTTCATCTAACTAGCC '3
          ||||||||||| ||||||||
3'      ACAAACGACTACCAGTAGATT       '5
```

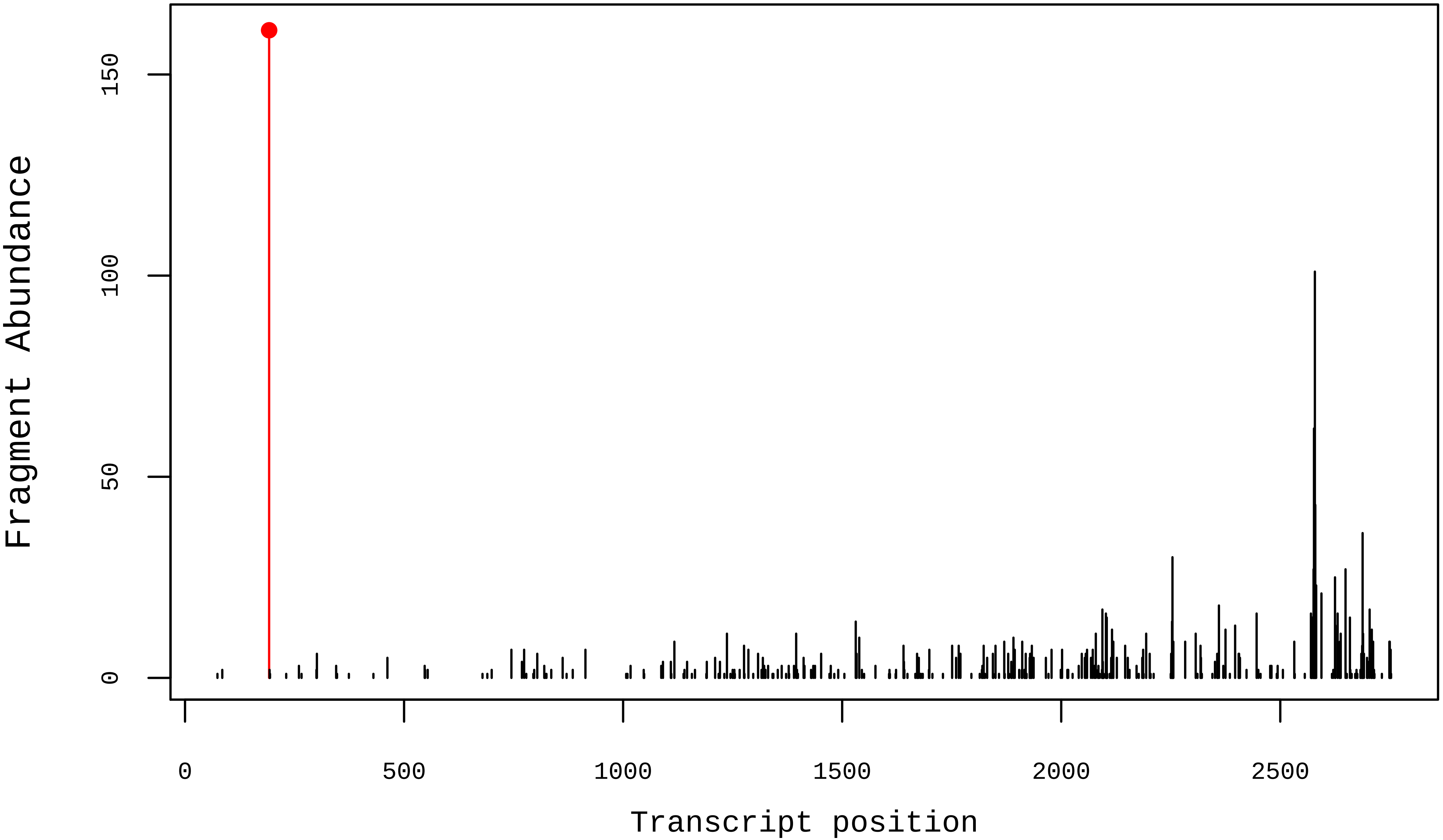


**Cleavage site: 192   Tag abundance: 161   Weighted abundance: 9.471   Category: 0**
**sRNA abundance: 1   Alignment score: 3   MFE ratio: 0.798   p-value: 0.014**

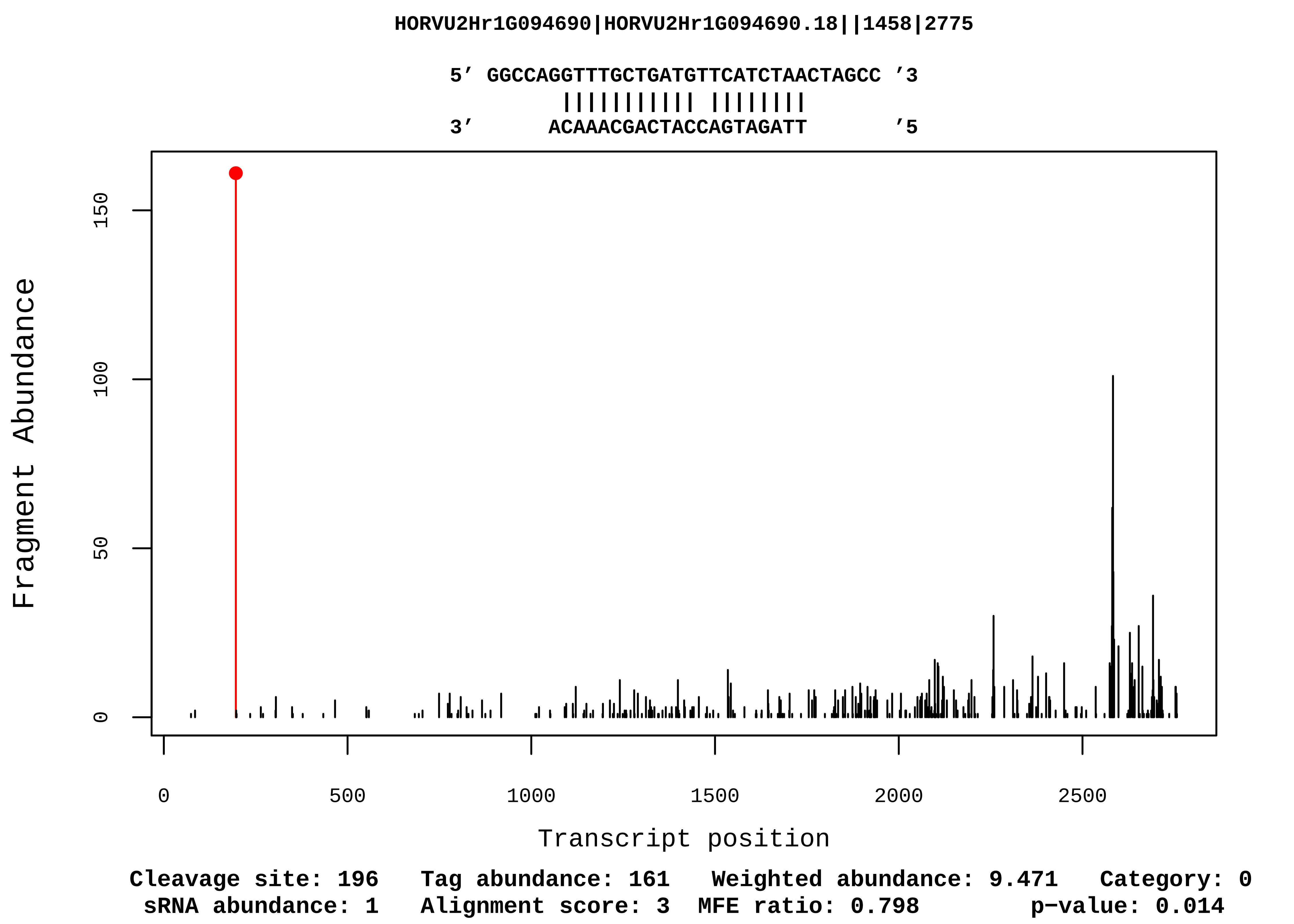
HORVU2Hr1G094690|HORVU2Hr1G094690.18||1458|2775
5' GGCCAGGTTTGCTGATGTTCATCTAACTAGCC '3
||||||||||| ||||||||
3' ACAAACGACTACCAGTAGATT '5
Fragment Abundance
0
50
100
150
0
500
1000
1500
2000
2500
Transcript position
Cleavage site: 196 Tag abundance: 161 Weighted abundance: 9.471 Category: 0
sRNA abundance: 1 Alignment score: 3 MFE ratio: 0.798 p-value: 0.014

HORVU2Hr1G094690|HORVU2Hr1G094690.19||1458|2630

```
5' GGCCAGGTTTGCTGATGTTCATCTAACTAGCC '3
            |||||||||||| |||||||||
3'      ACAAACGACTACCAGTAGATT         '5
```

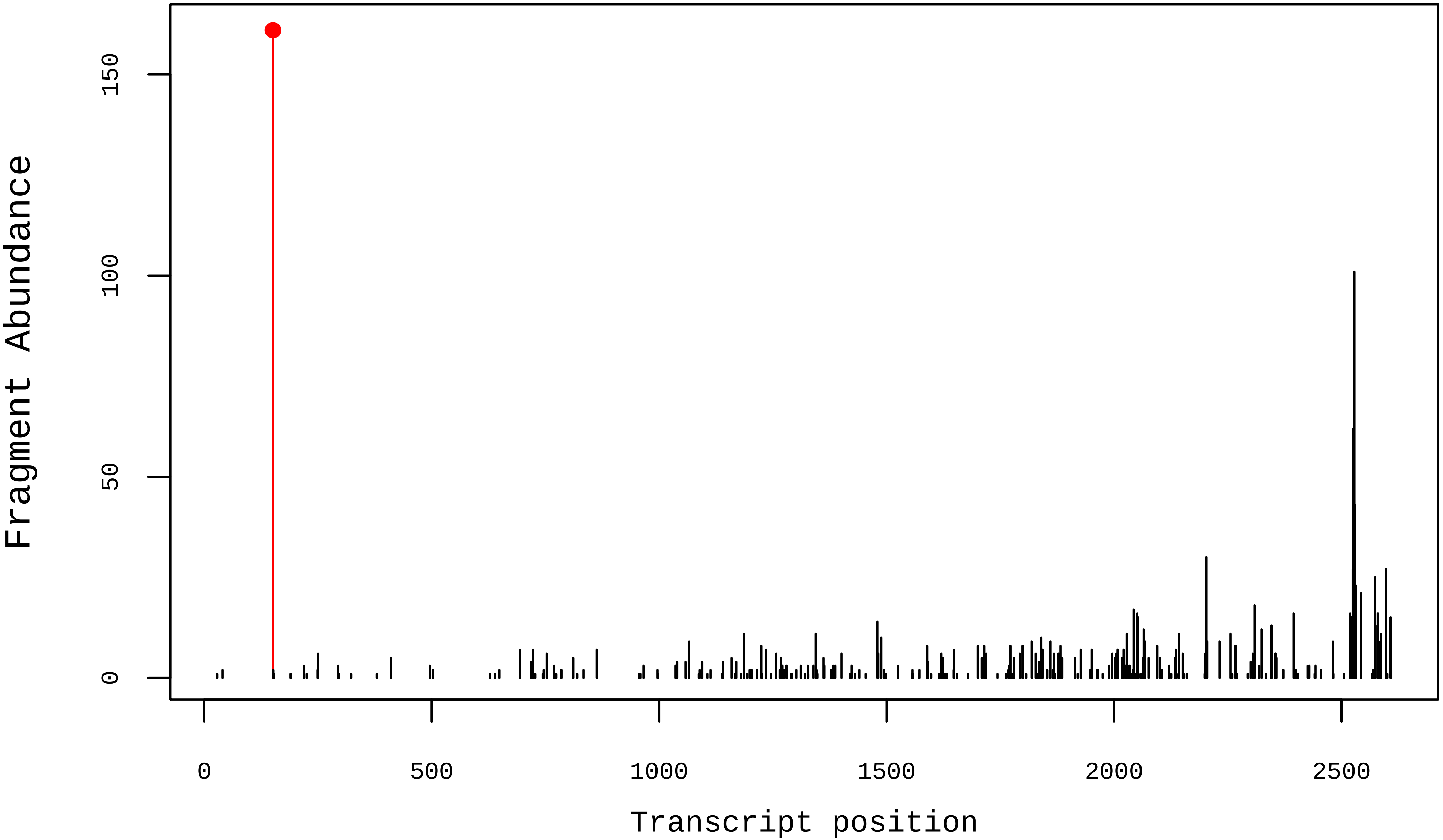


**Cleavage site: 151   Tag abundance: 161   Weighted abundance: 9.471   Category: 0**

**sRNA abundance: 1   Alignment score: 3   MFE ratio: 0.798   p-value: 0.014**

HORVU2Hr1G094690|HORVU2Hr1G094690.1||2112|2767

```
5' GGCCAGGTTTGCTGATGTTCATCTAACTAGCC '3
        |||||||||||| |||||||||
3'      ACAAACGACTACCAGTAGATT       '5
```

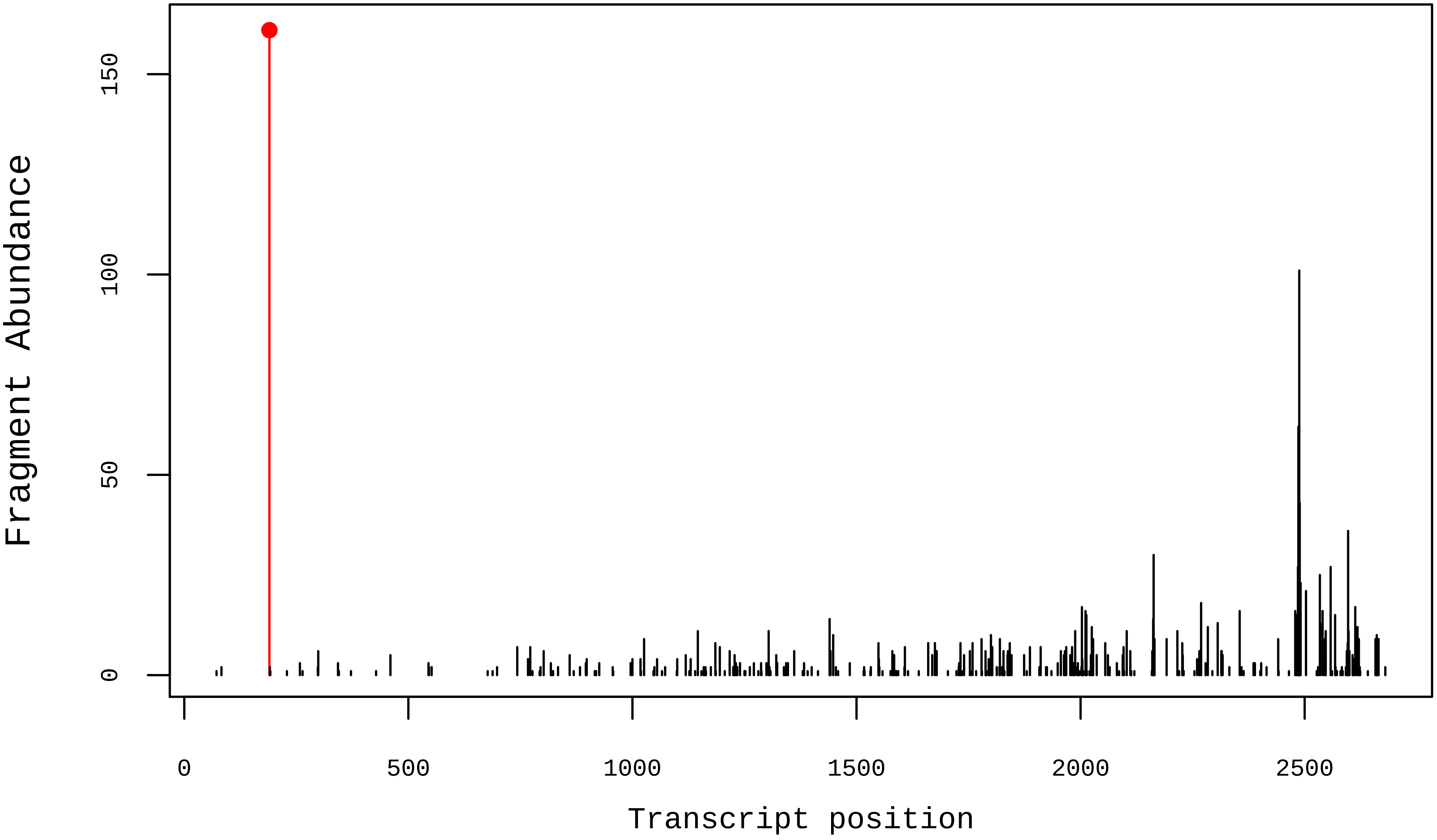


**Cleavage site: 190   Tag abundance: 161   Weighted abundance: 9.471   Category: 0**
**sRNA abundance: 1   Alignment score: 3   MFE ratio: 0.798   p-value: 0.014**

HORVU2Hr1G094690|HORVU2Hr1G094690.2||2112|2761

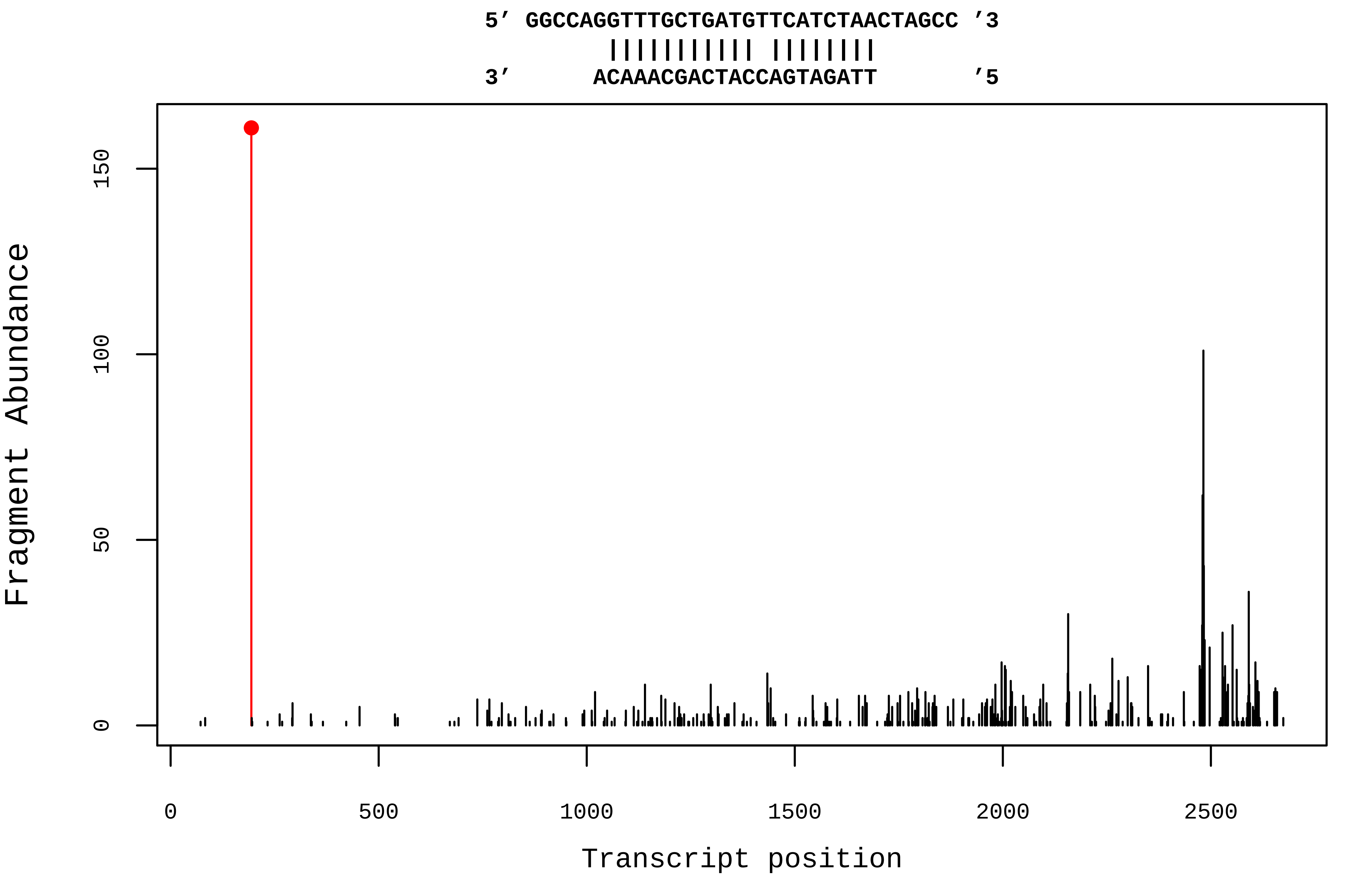


Cleavage site: 194 Tag abundance: 161 Weighted abundance: 9.471 Category: 0
sRNA abundance: 1 Alignment score: 3 MFE ratio: 0.798 p-value: 0.014

HORVU2Hr1G094690|HORVU2Hr1G094690.3||2112|2734

```
5' GGCCAGGTTTGCTGATGTTCATCTAACTAGCC '3
          ||||||||||| ||||||||
3'      ACAAACGACTACCAGTAGATT       '5
```

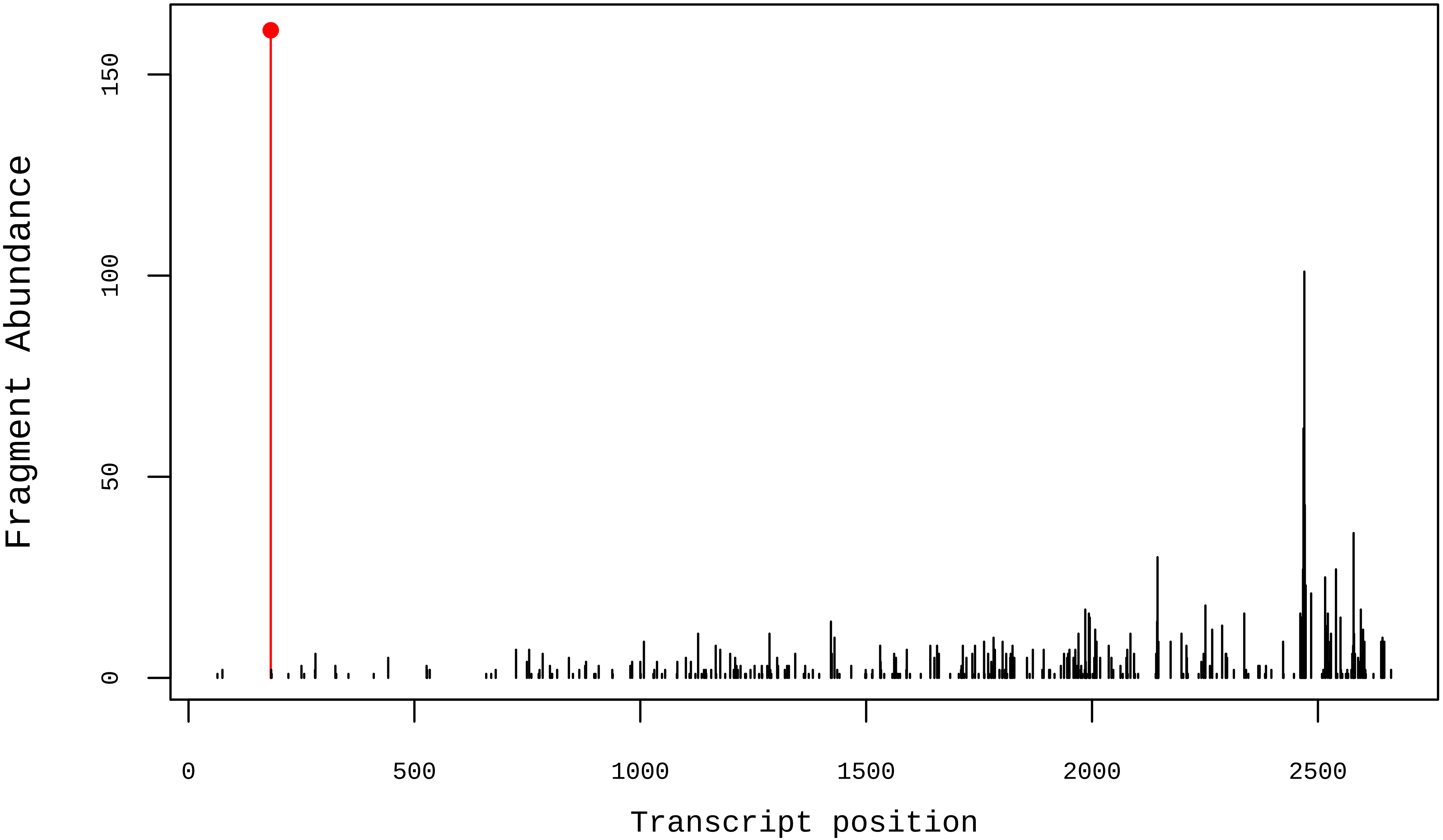


Cleavage site: 182   Tag abundance: 161   Weighted abundance: 9.471   Category: 0
sRNA abundance: 1   Alignment score: 3   MFE ratio: 0.798   p-value: 0.014

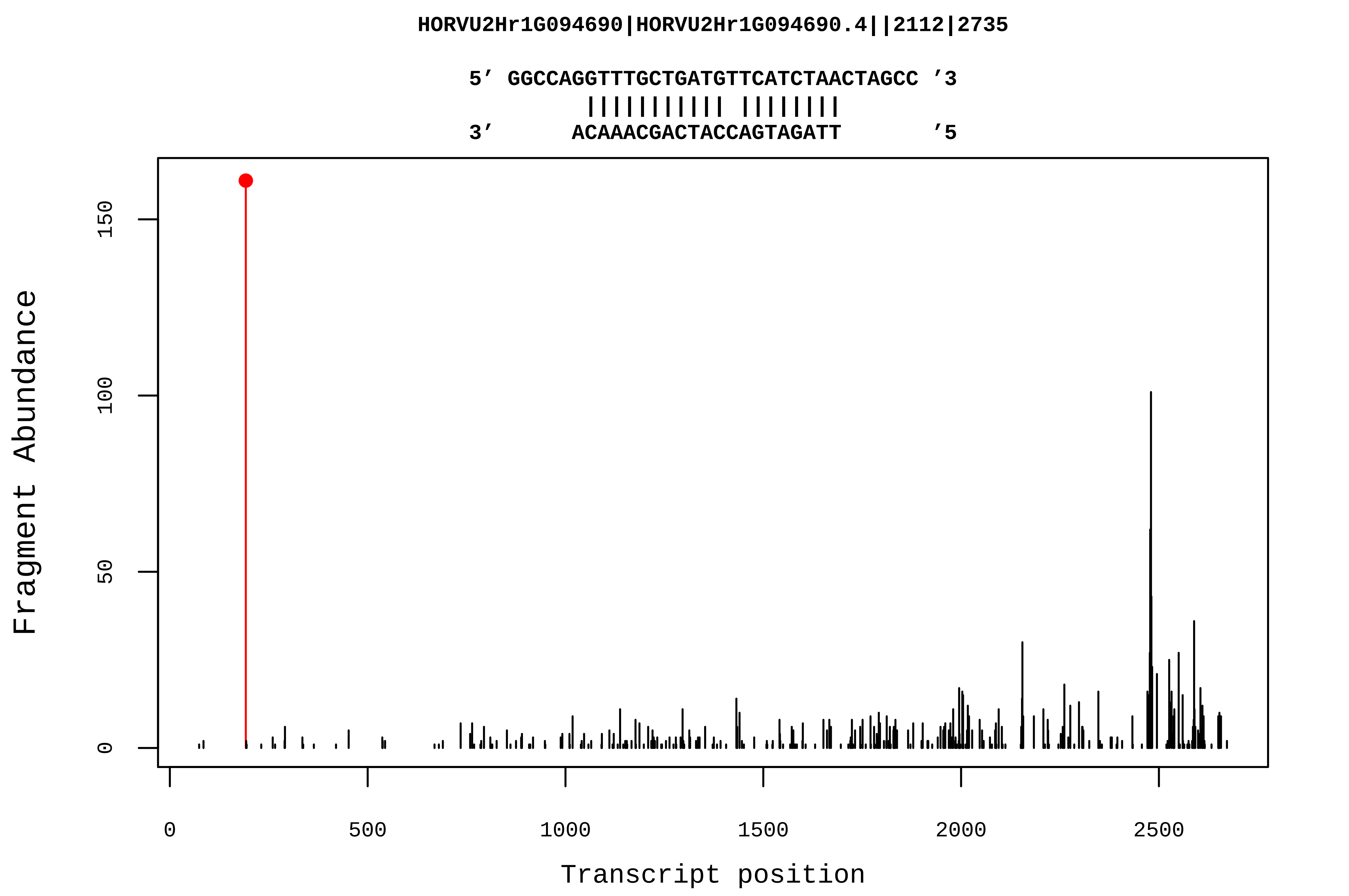


**Cleavage site: 192 Tag abundance: 161 Weighted abundance: 9.471 Category: 0**
**sRNA abundance: 1 Alignment score: 3 MFE ratio: 0.798 p-value: 0.014**

HORVU2Hr1G094690|HORVU2Hr1G094690.7||720|2747

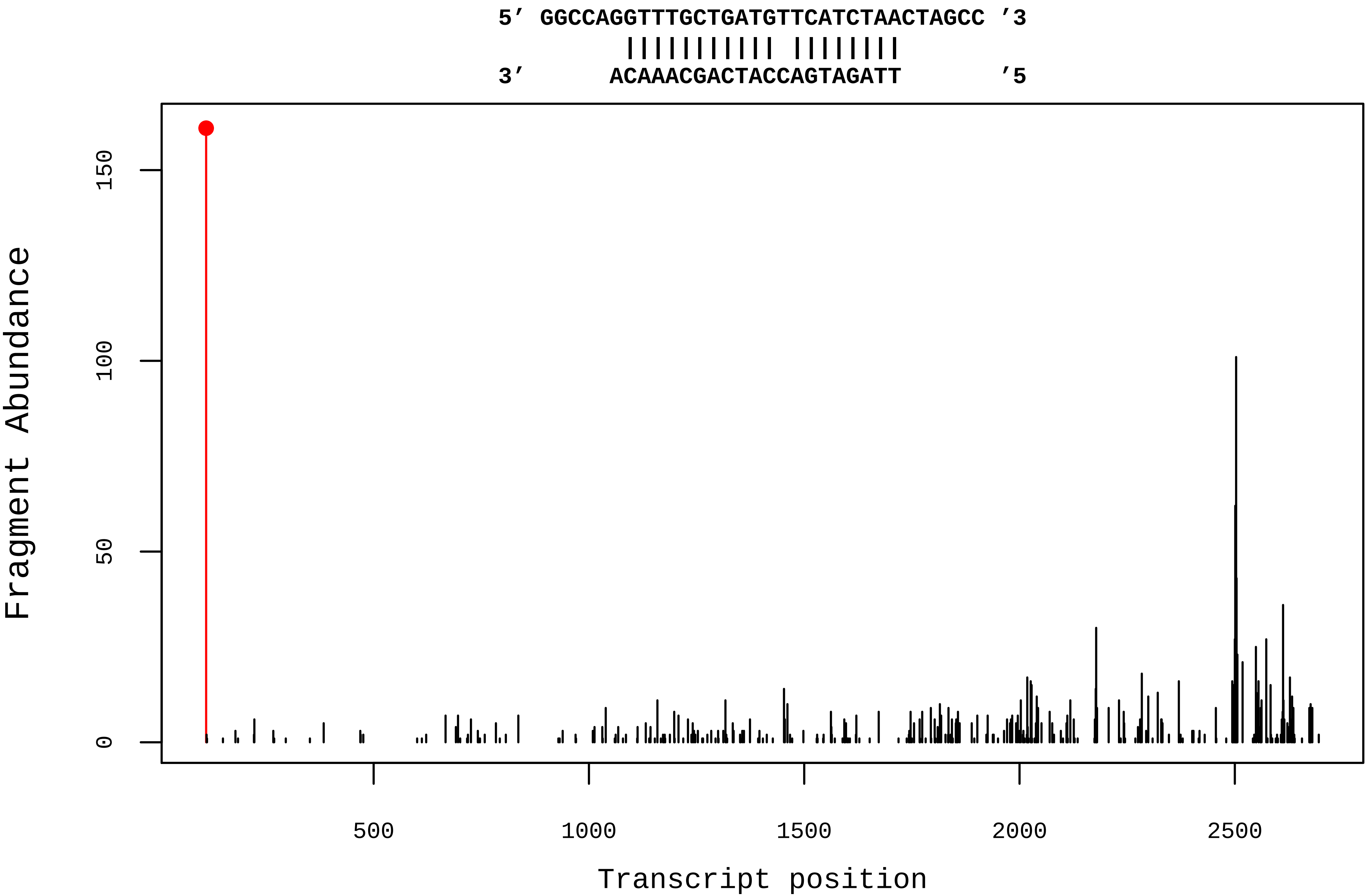


**Cleavage site: 111 Tag abundance: 161 Weighted abundance: 9.471 Category: 0**
**sRNA abundance: 1 Alignment score: 3 MFE ratio: 0.798 p-value: 0.014**

HORVU2Hr1G094690|HORVU2Hr1G094690.8||1458|2744

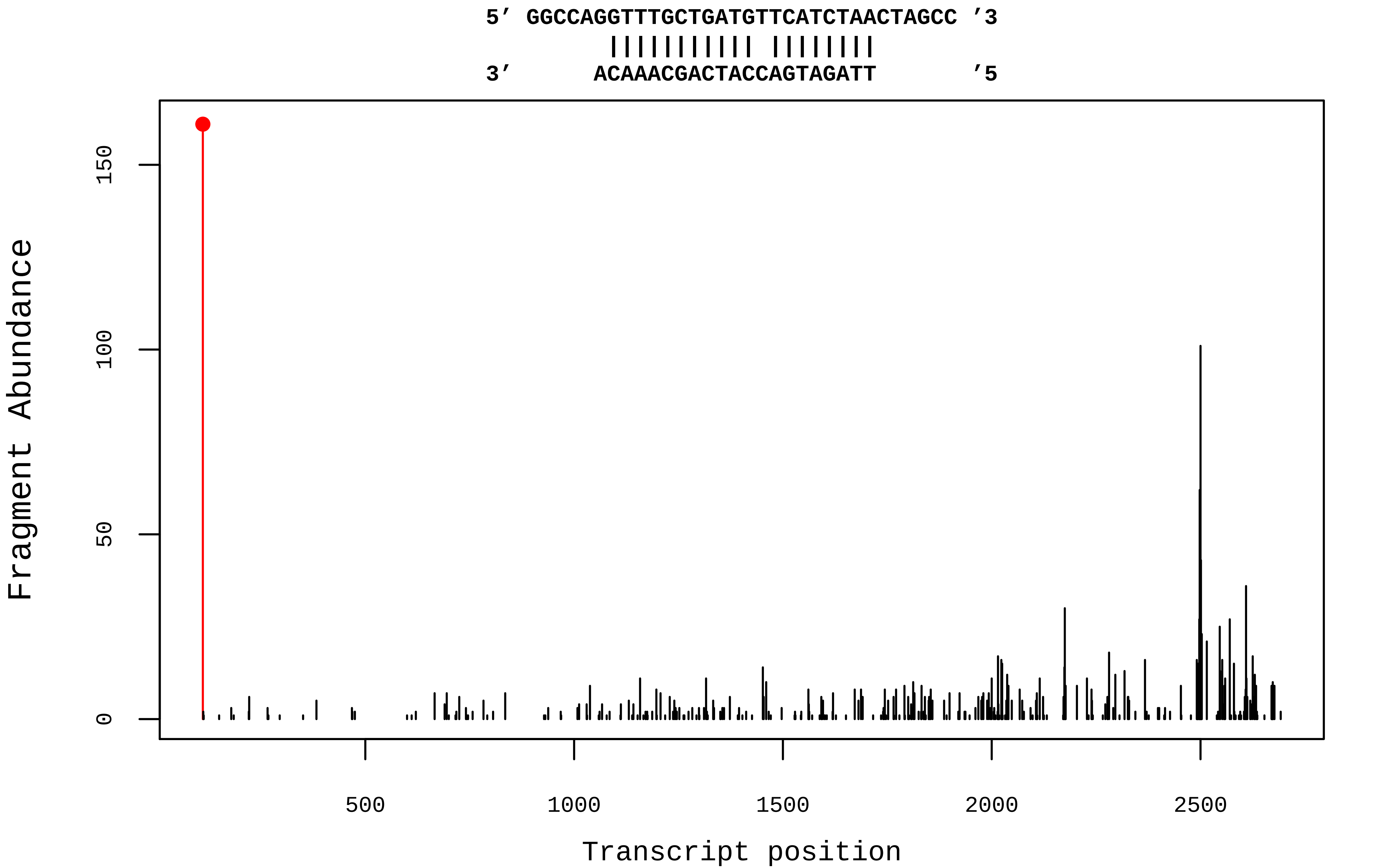


Cleavage site: 111 Tag abundance: 161 Weighted abundance: 9.471 Category: 0
sRNA abundance: 1 Alignment score: 3 MFE ratio: 0.798 p-value: 0.014

HORVU2Hr1G094690|HORVU2Hr1G094690.9||1227|2744

```
5' GGCCAGGTTTGCTGATGTTCATCTAACTAGCC '3
             |||||||||||| |||||||||
3'       ACAAACGACTACCAGTAGATT        '5
```

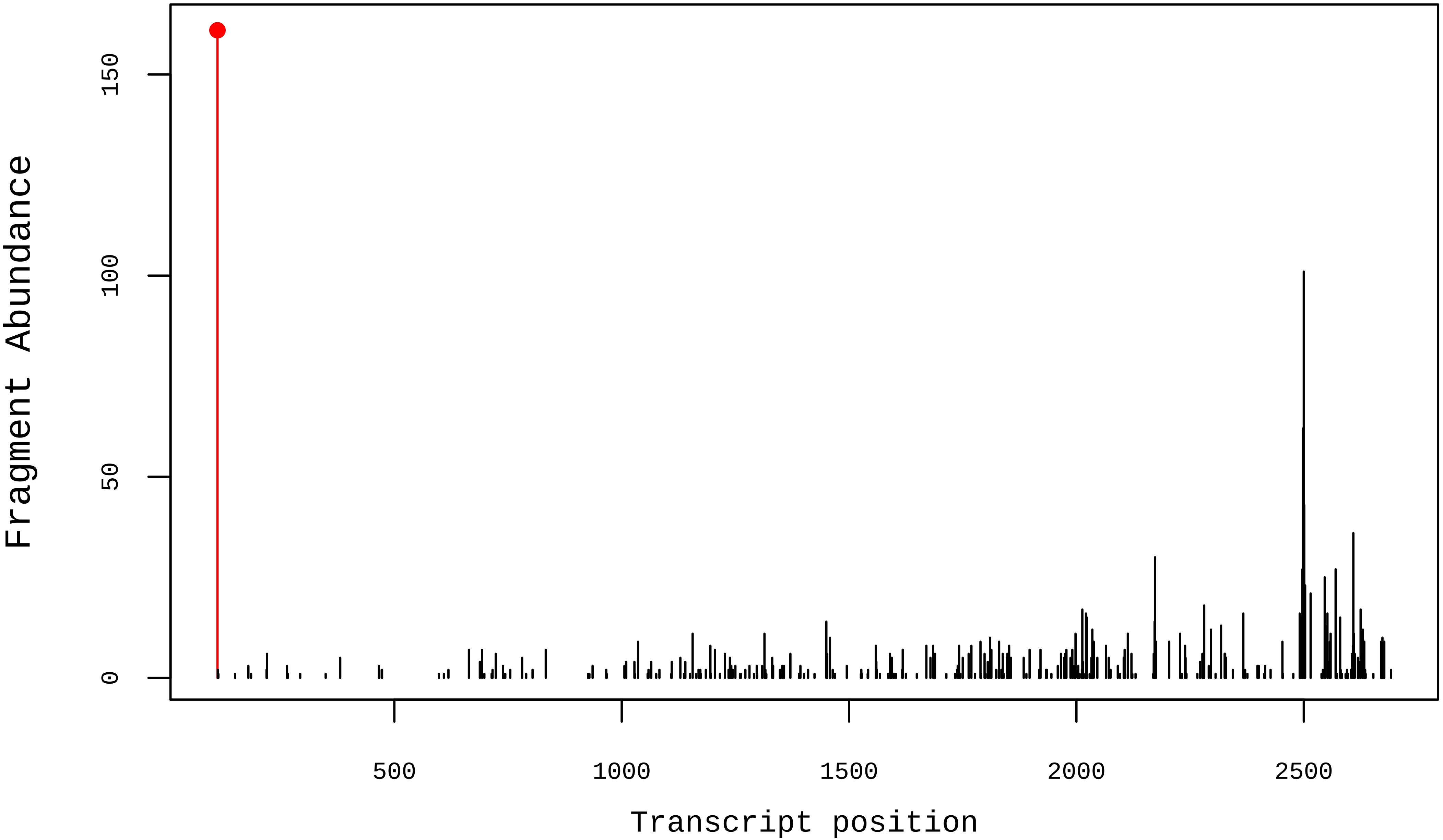


Cleavage site: 111   Tag abundance: 161   Weighted abundance: 9.471   Category: 0
sRNA abundance: 1   Alignment score: 3   MFE ratio: 0.798   p-value: 0.014

**HORVU2Hr1G094690|HORVU2Hr1G094690.10||1458|2742**

```
5' GGCCAGGTTTGCTGATGTTCATCTAACTAGCC '3
         | ||||||||||| ||||||||
3'       TACAAACGACTACCAGTAGATT       '5
```

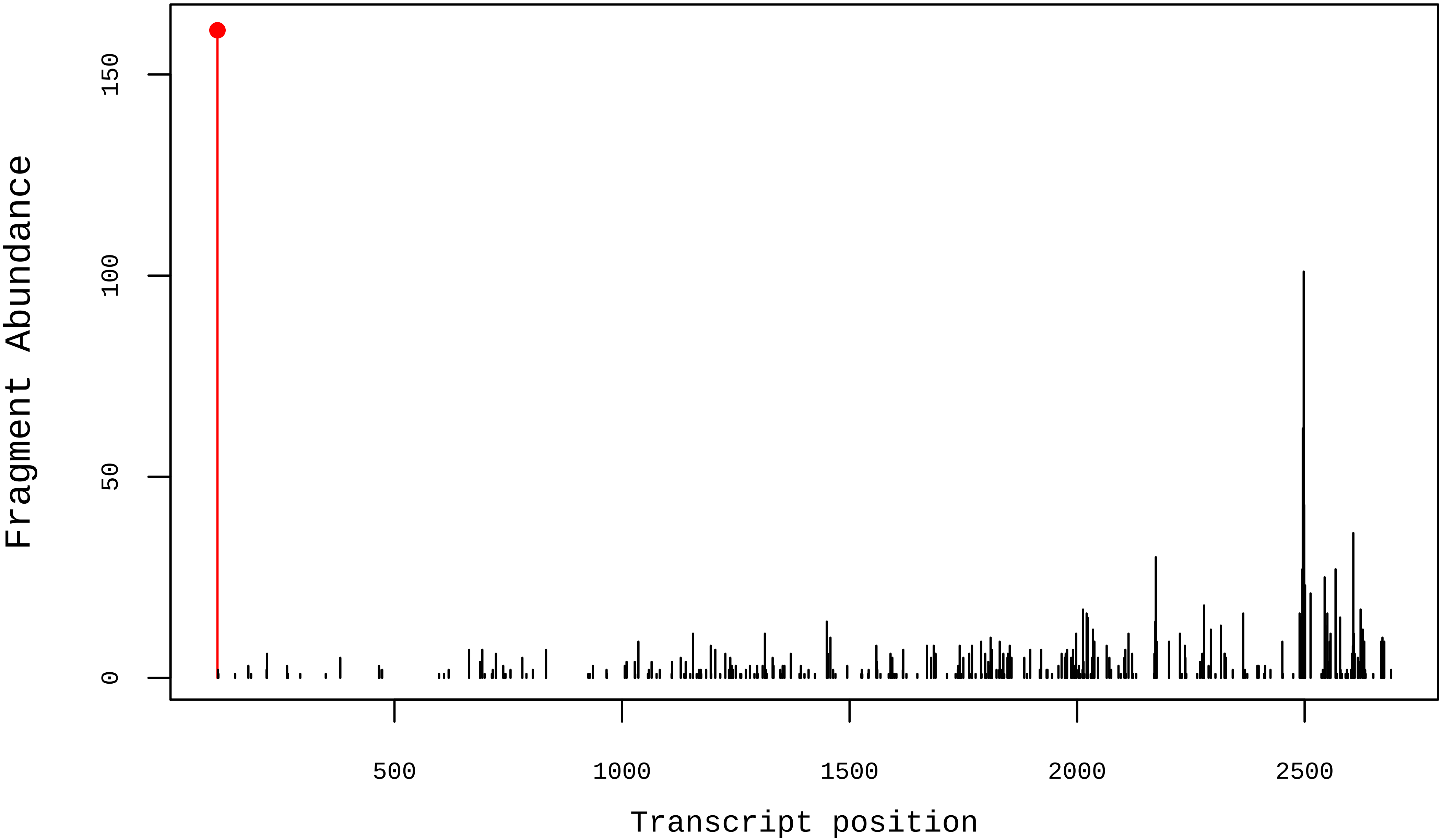


**Cleavage site: 111   Tag abundance: 161   Weighted abundance: 9.471   Category: 0**
**sRNA abundance: 1   Alignment score: 3   MFE ratio: 0.773   p-value: 0.014**

HORVU2Hr1G094690|HORVU2Hr1G094690.11||1458|2742

```
5' GGCCAGGTTTGCTGATGTTCATCTAACTAGCC '3
         | ||||||||||| ||||||||
3'       TACAAACGACTACCAGTAGATT       '5
```

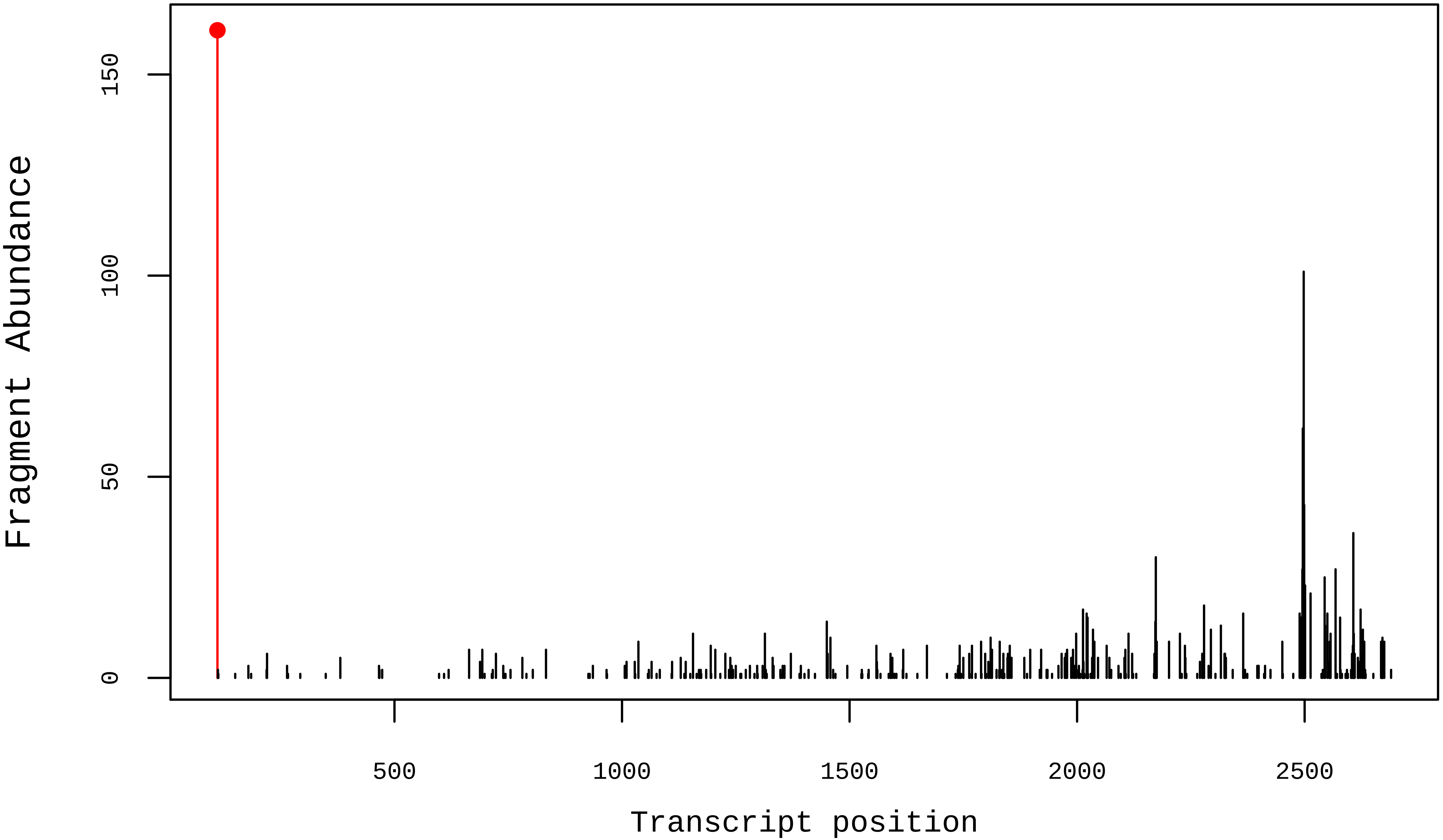


Cleavage site: 111 Tag abundance: 161 Weighted abundance: 9.471 Category: 0
sRNA abundance: 1 Alignment score: 3 MFE ratio: 0.773 p-value: 0.014

HORVU2Hr1G094690|HORVU2Hr1G094690.12||2112|2910

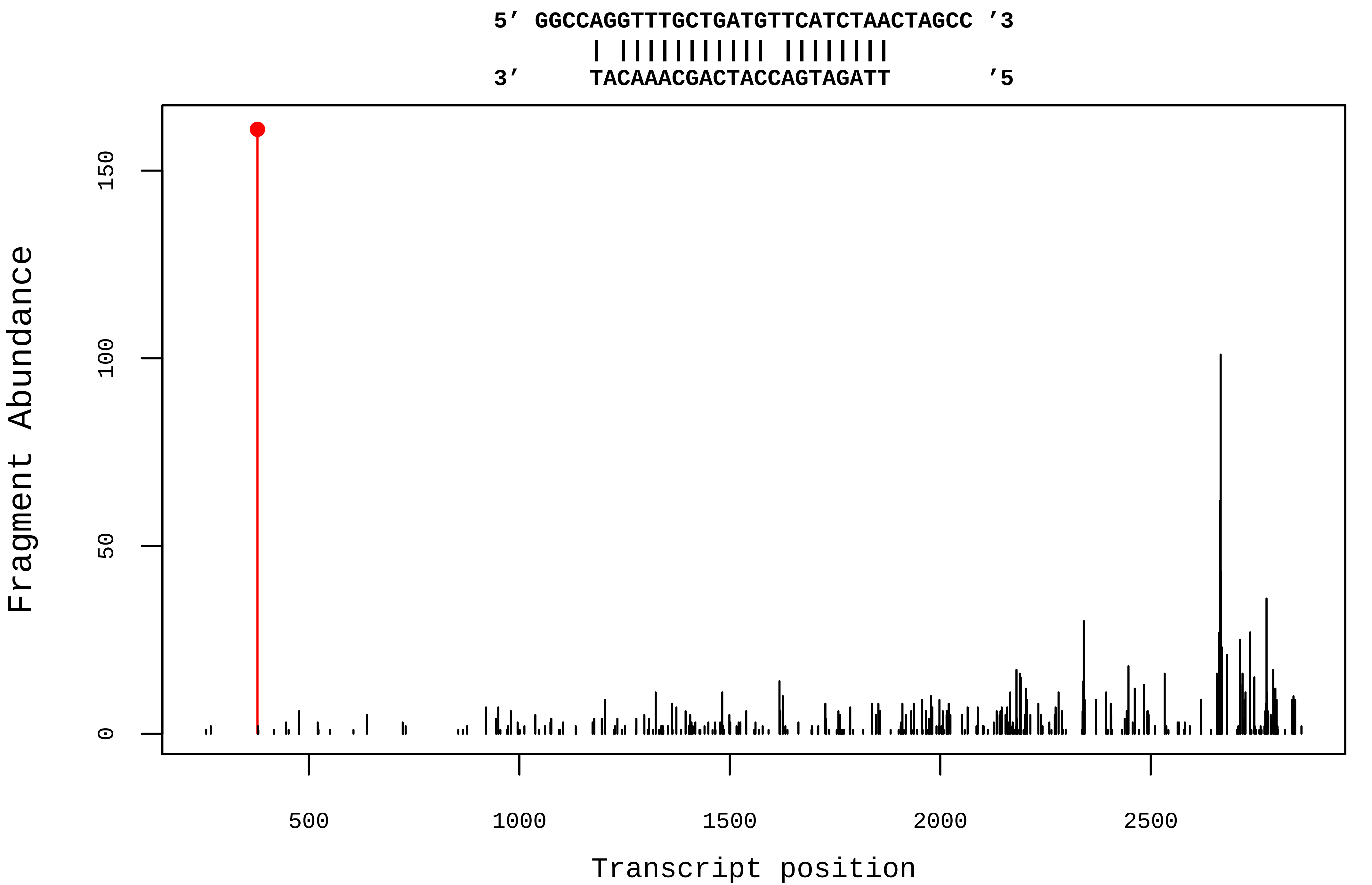


Cleavage site: 378 Tag abundance: 161 Weighted abundance: 9.471 Category: 0
sRNA abundance: 1 Alignment score: 3 MFE ratio: 0.773 p-value: 0.013

HORVU2Hr1G094690|HORVU2Hr1G094690.13||2112|2916

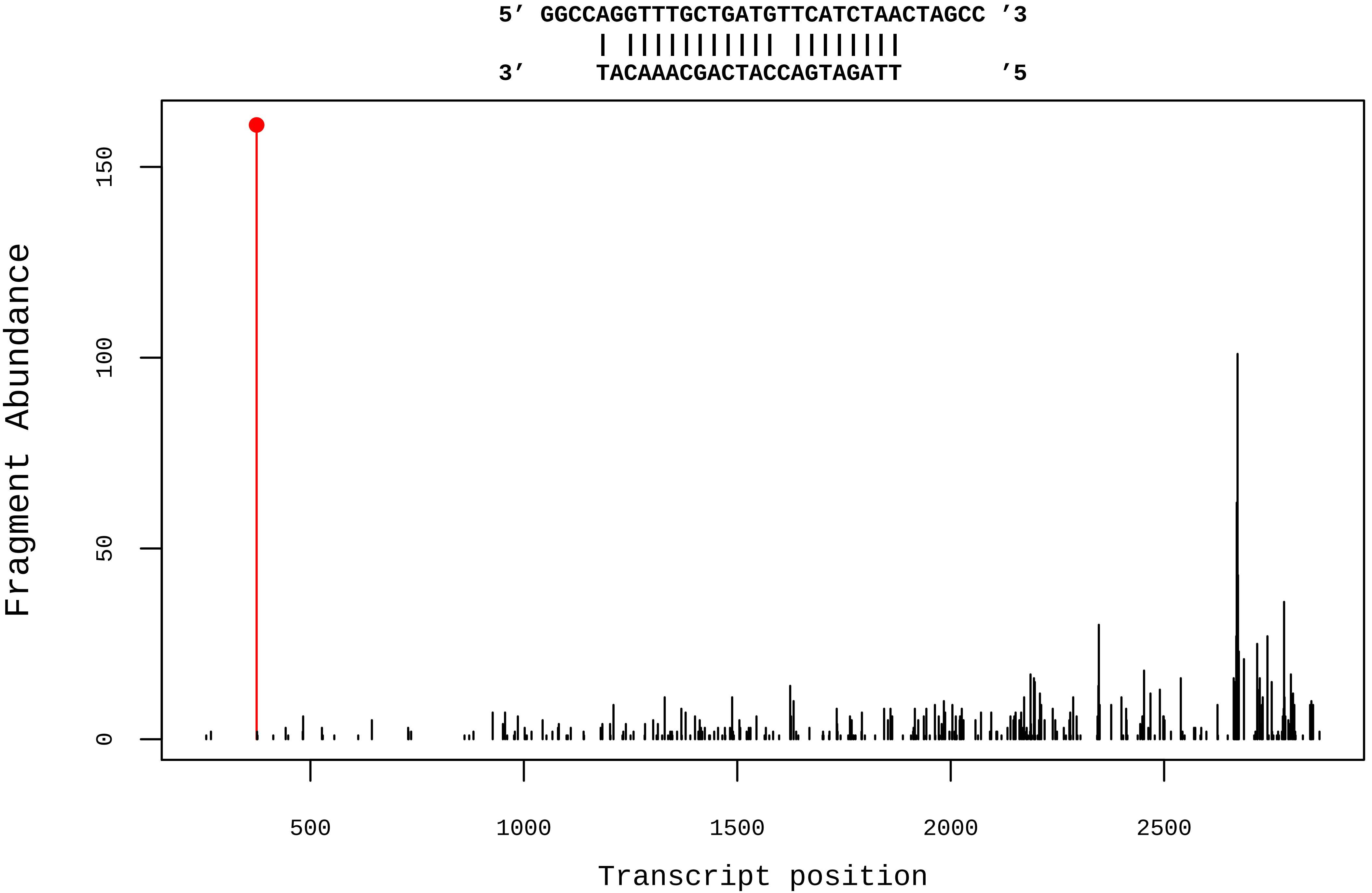


**Cleavage site: 374 Tag abundance: 161 Weighted abundance: 9.471 Category: 0**
**sRNA abundance: 1 Alignment score: 3 MFE ratio: 0.773 p-value: 0.013**

HORVU2Hr1G094690|HORVU2Hr1G094690.14||2112|2920

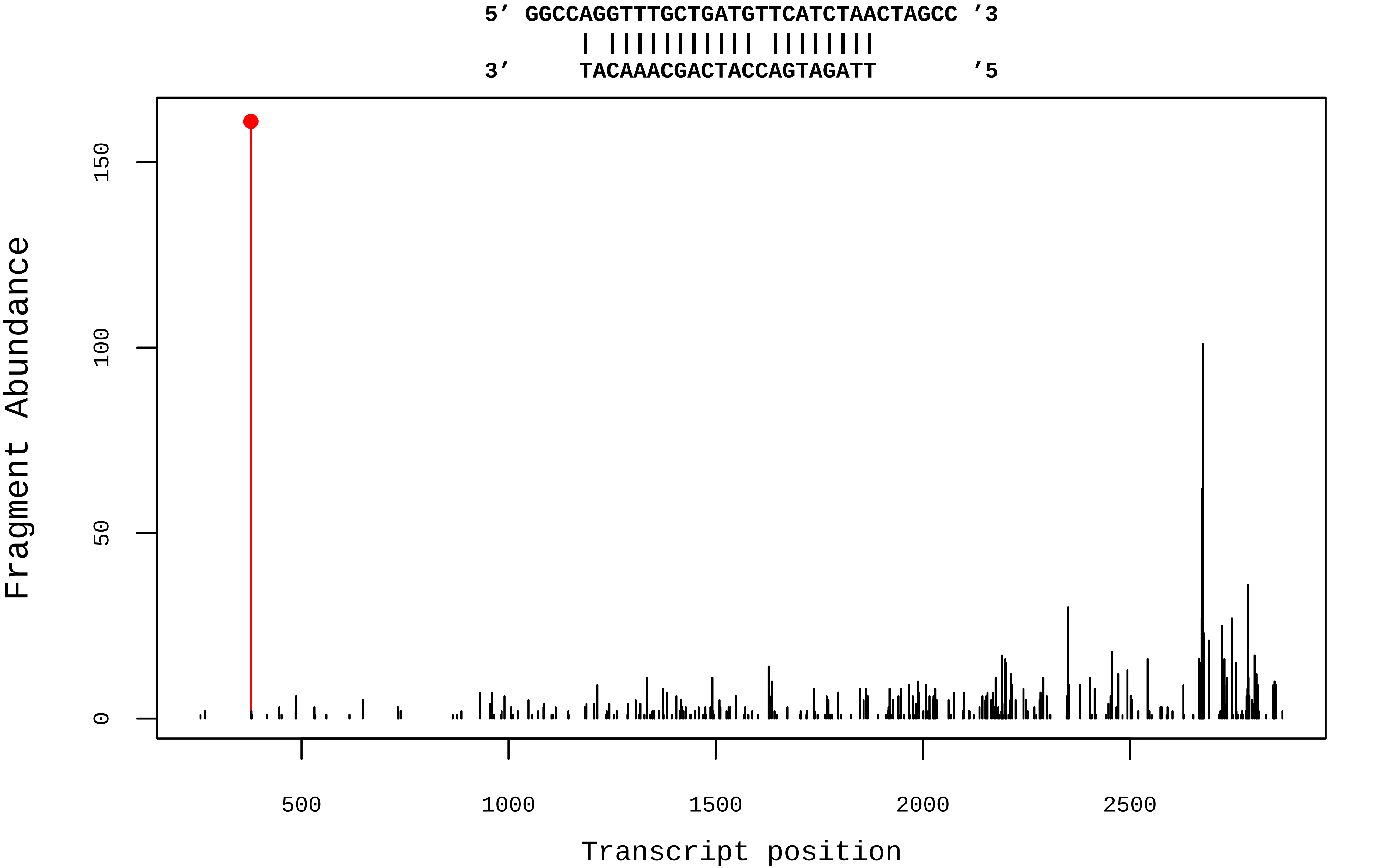


Cleavage site: 378 Tag abundance: 161 Weighted abundance: 9.471 Category: 0
sRNA abundance: 1 Alignment score: 3 MFE ratio: 0.773 p-value: 0.013

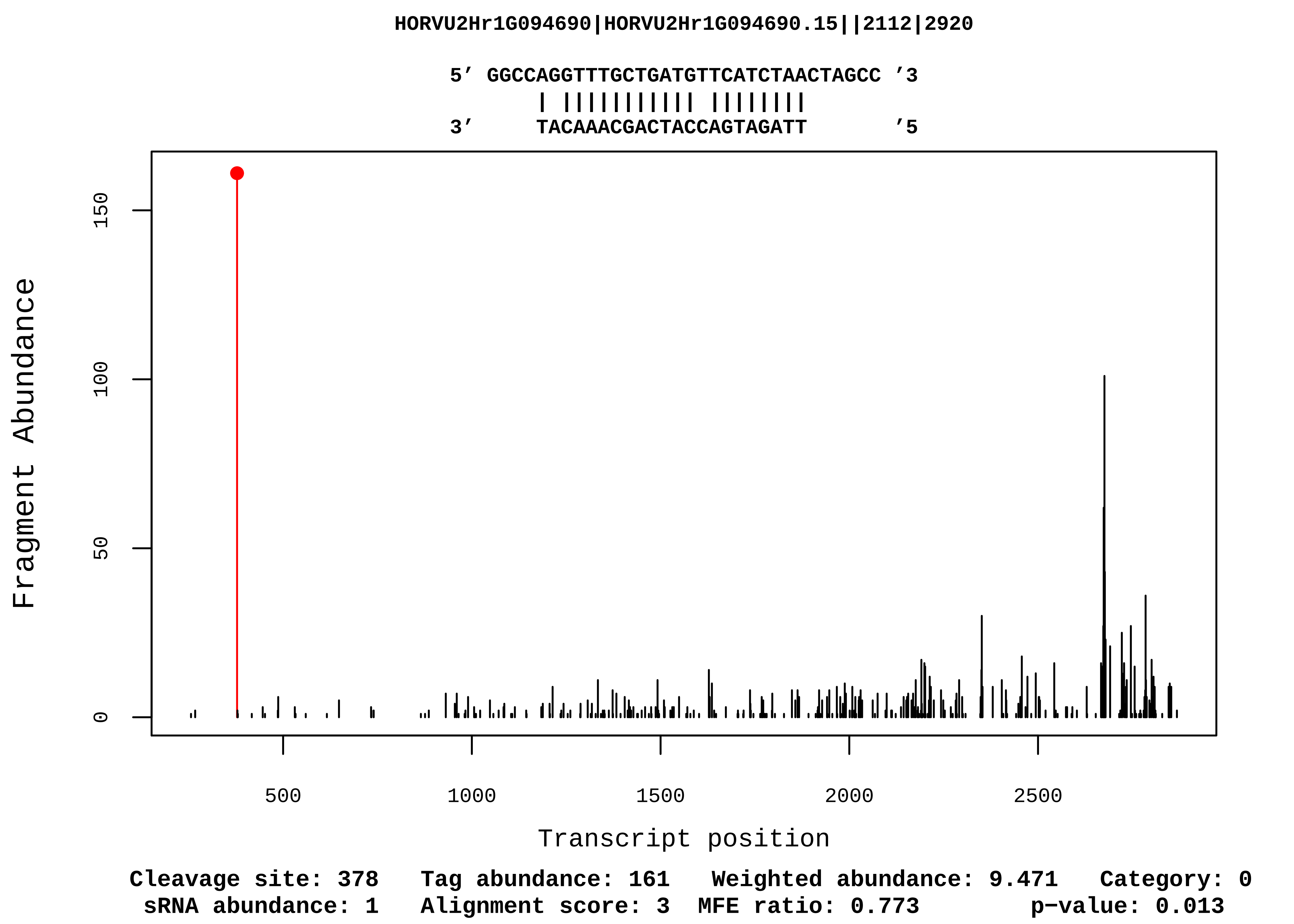
HORVU2Hr1G094690|HORVU2Hr1G094690.15||2112|2920
5' GGCCAGGTTTGCTGATGTTCATCTAACTAGCC '3
3' TACAAACGACTACCAGTAGATT '5
Fragment Abundance
0
50
100
150
500
1000
1500
2000
2500
Transcript position
Cleavage site: 378 Tag abundance: 161 Weighted abundance: 9.471 Category: 0
sRNA abundance: 1 Alignment score: 3 MFE ratio: 0.773 p-value: 0.013

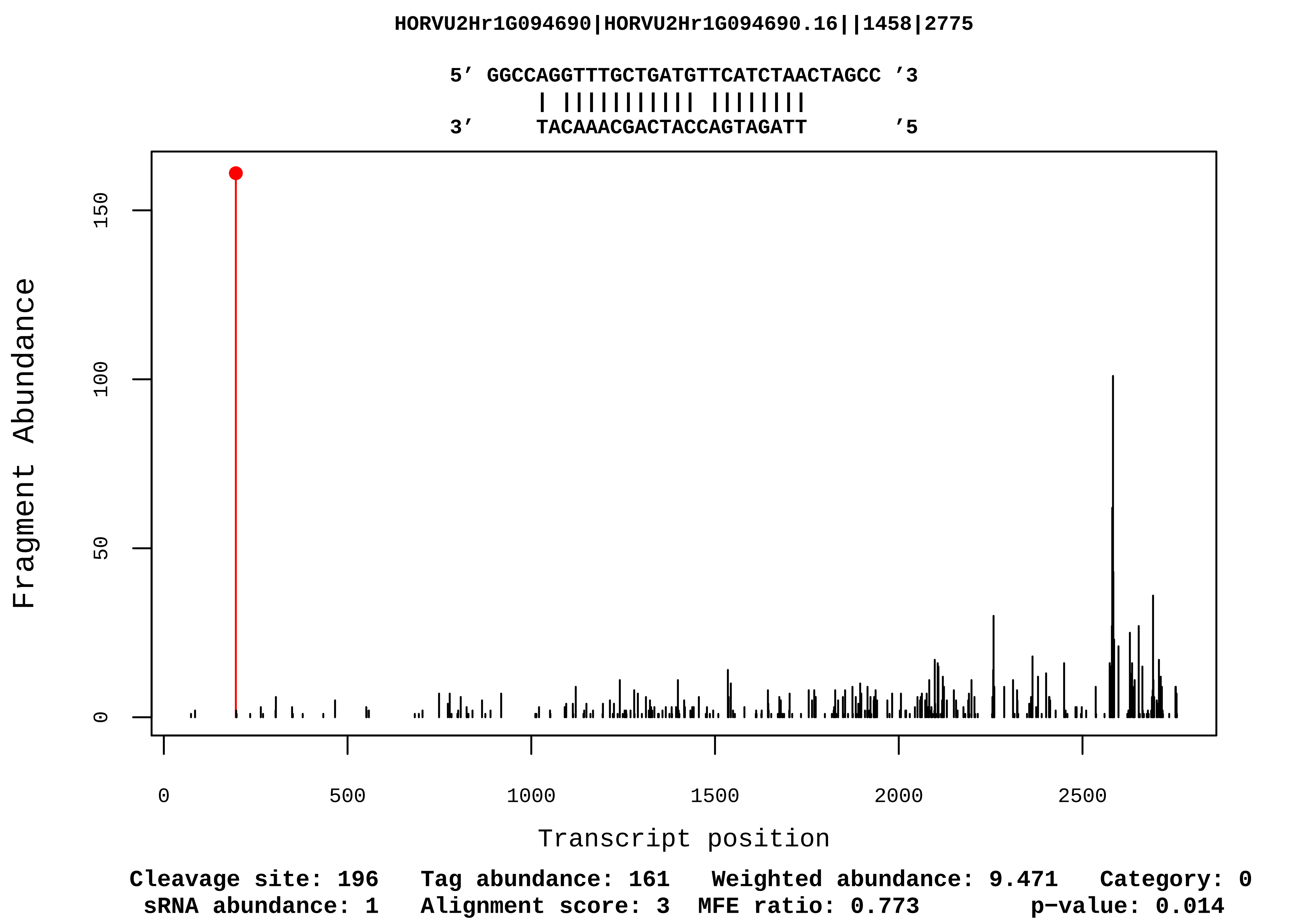
HORVU2Hr1G094690|HORVU2Hr1G094690.16||1458|2775
5' GGCCAGGTTTGCTGATGTTCATCTAACTAGCC '3
3' TACAAACGACTACCAGTAGATT '5
Fragment Abundance
Transcript position
0
50
100
150
0
500
1000
1500
2000
2500
Cleavage site: 196 Tag abundance: 161 Weighted abundance: 9.471 Category: 0
sRNA abundance: 1 Alignment score: 3 MFE ratio: 0.773 p-value: 0.014

HORVU2Hr1G094690|HORVU2Hr1G094690.17||1458|2771

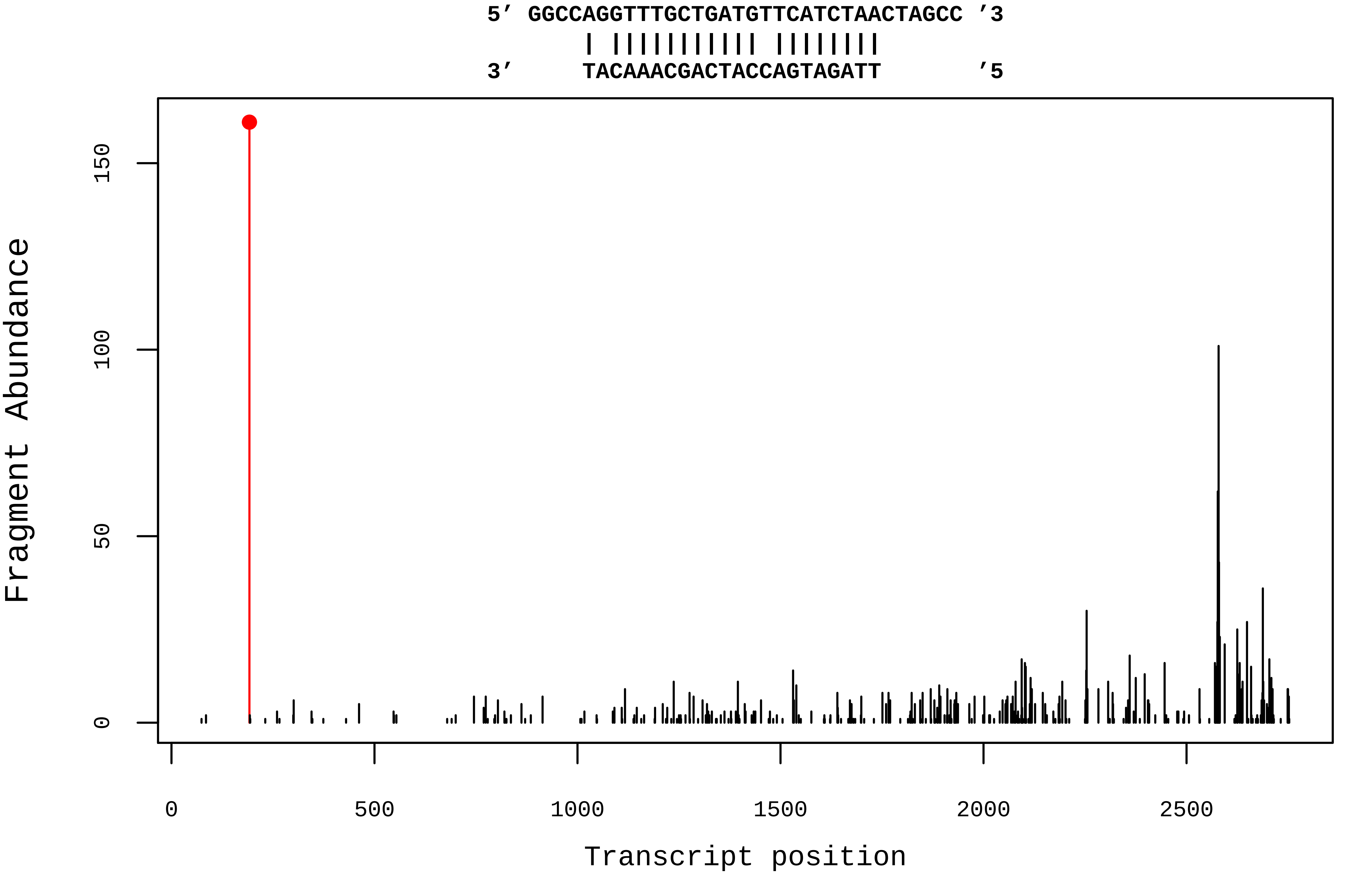


Cleavage site: 192 Tag abundance: 161 Weighted abundance: 9.471 Category: 0
sRNA abundance: 1 Alignment score: 3 MFE ratio: 0.773 p-value: 0.014

**HORVU2Hr1G094690|HORVU2Hr1G094690.18||1458|2775**

```
5′ GGCCAGGTTTGCTGATGTTCATCTAACTAGCC ′3
       | ||||||||||| ||||||||
3′     TACAAACGACTACCAGTAGATT       ′5
```

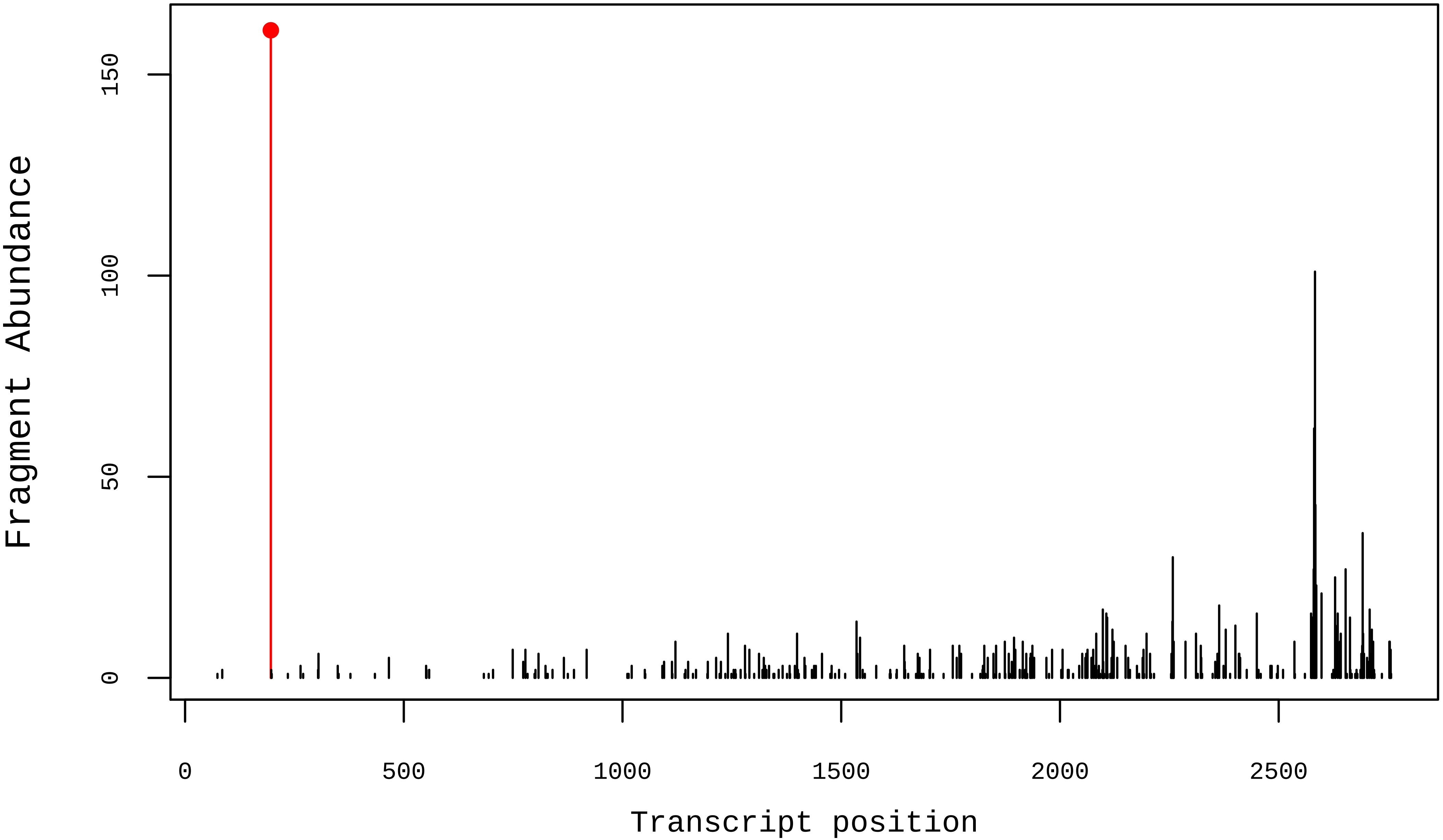


**Cleavage site: 196  Tag abundance: 161  Weighted abundance: 9.471  Category: 0**
**sRNA abundance: 1  Alignment score: 3  MFE ratio: 0.773  p-value: 0.014**

**HORVU2Hr1G094690|HORVU2Hr1G094690.19||1458|2630**

```
5' GGCCAGGTTTGCTGATGTTCATCTAACTAGCC '3
       | ||||||||||| ||||||||
3'     TACAAACGACTACCAGTAGATT        '5
```

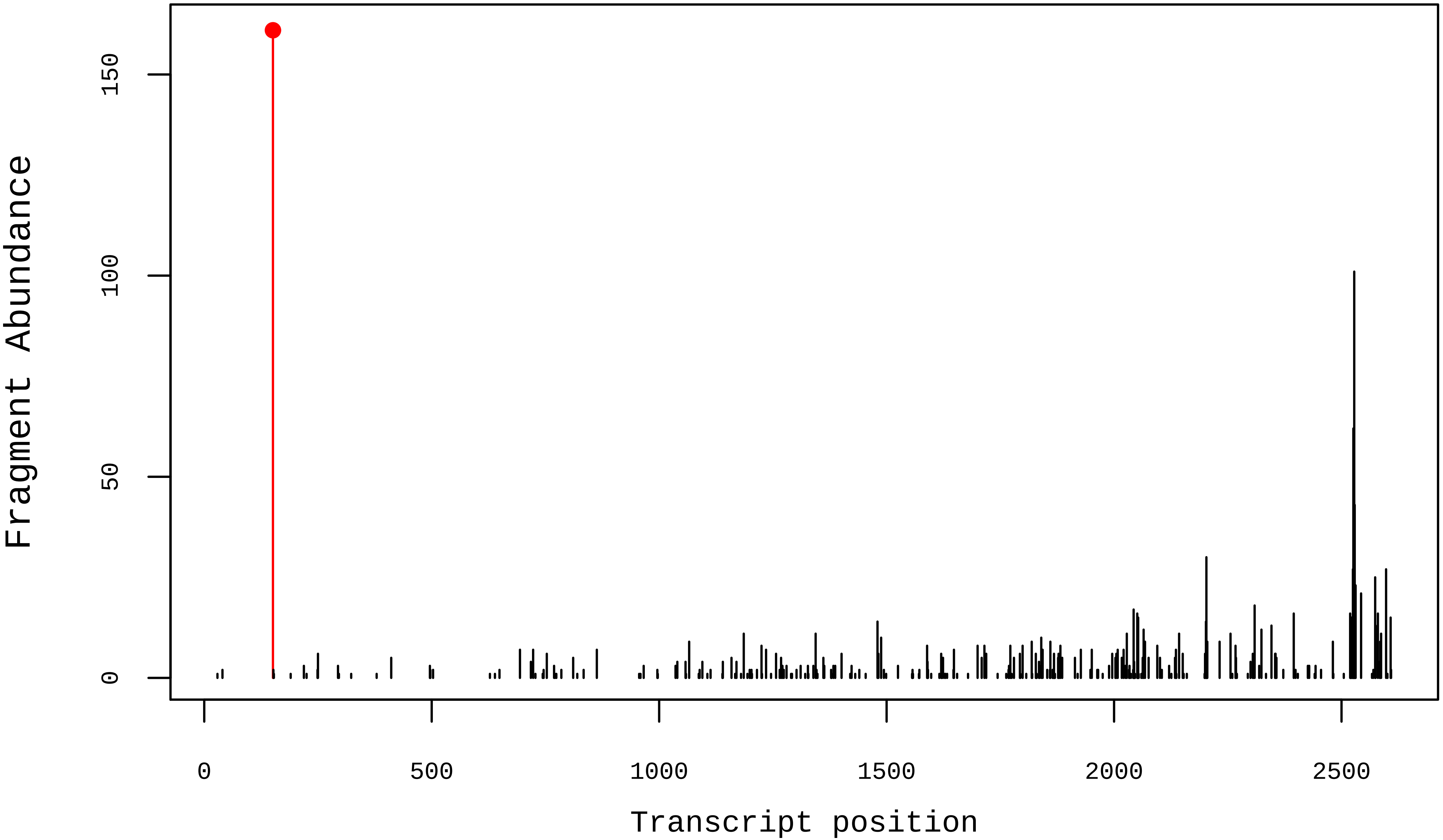


**Cleavage site: 151   Tag abundance: 161   Weighted abundance: 9.471   Category: 0**
**sRNA abundance: 1   Alignment score: 3   MFE ratio: 0.773   p-value: 0.014**

**HORVU2Hr1G094690|HORVU2Hr1G094690.1||2112|2767**

```
5' GGCCAGGTTTGCTGATGTTCATCTAACTAGCC '3
         | |||||||||||| ||||||||
3'       TACAAACGACTACCAGTAGATT       '5
```

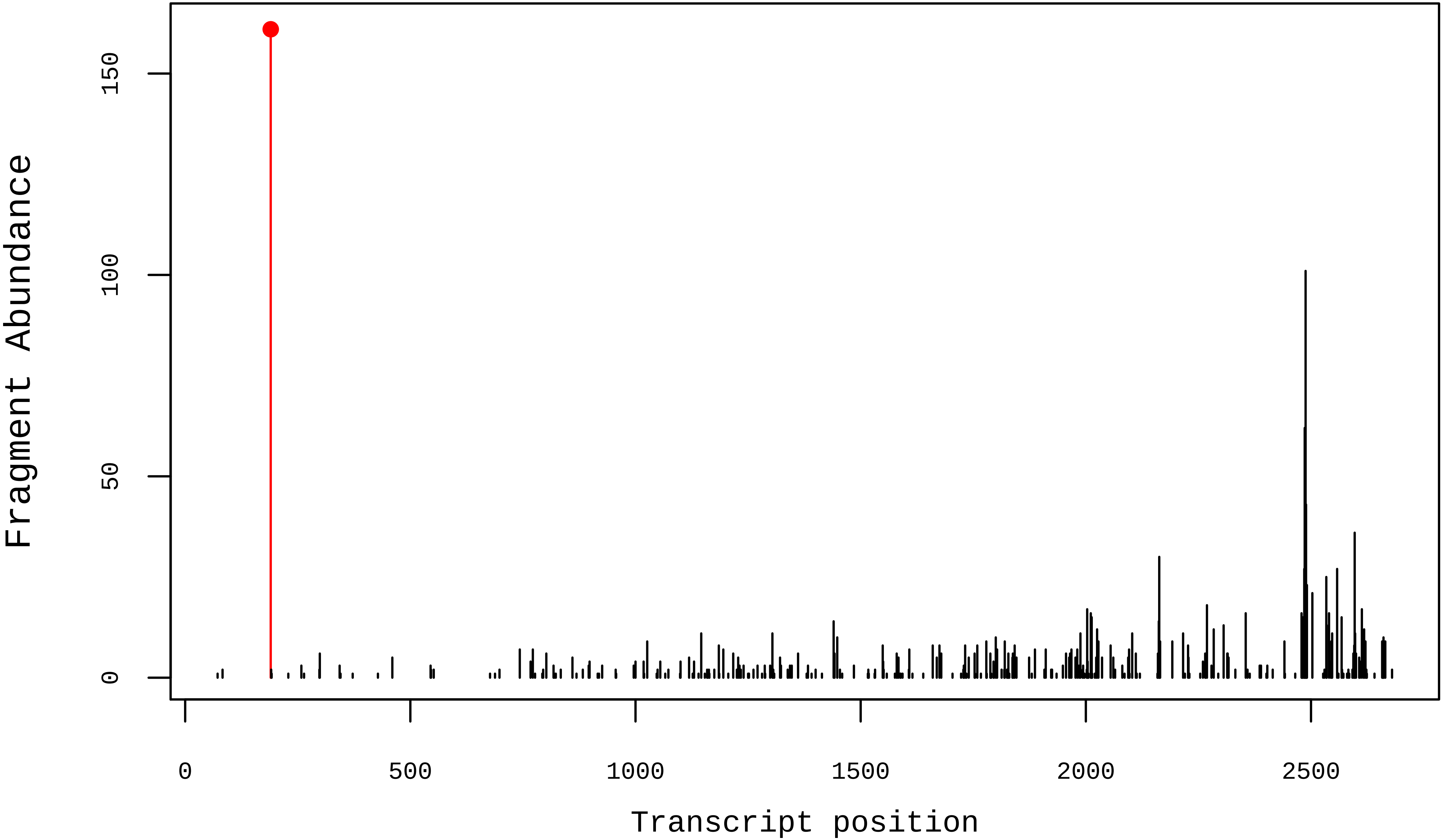


**Cleavage site: 190   Tag abundance: 161   Weighted abundance: 9.471   Category: 0**
**sRNA abundance: 1   Alignment score: 3   MFE ratio: 0.773   p-value: 0.014**

HORVU2Hr1G094690|HORVU2Hr1G094690.2||2112|2761

```
5' GGCCAGGTTTGCTGATGTTCATCTAACTAGCC '3
        | |||||||||||| ||||||||
3'      TACAAACGACTACCAGTAGATT       '5
```

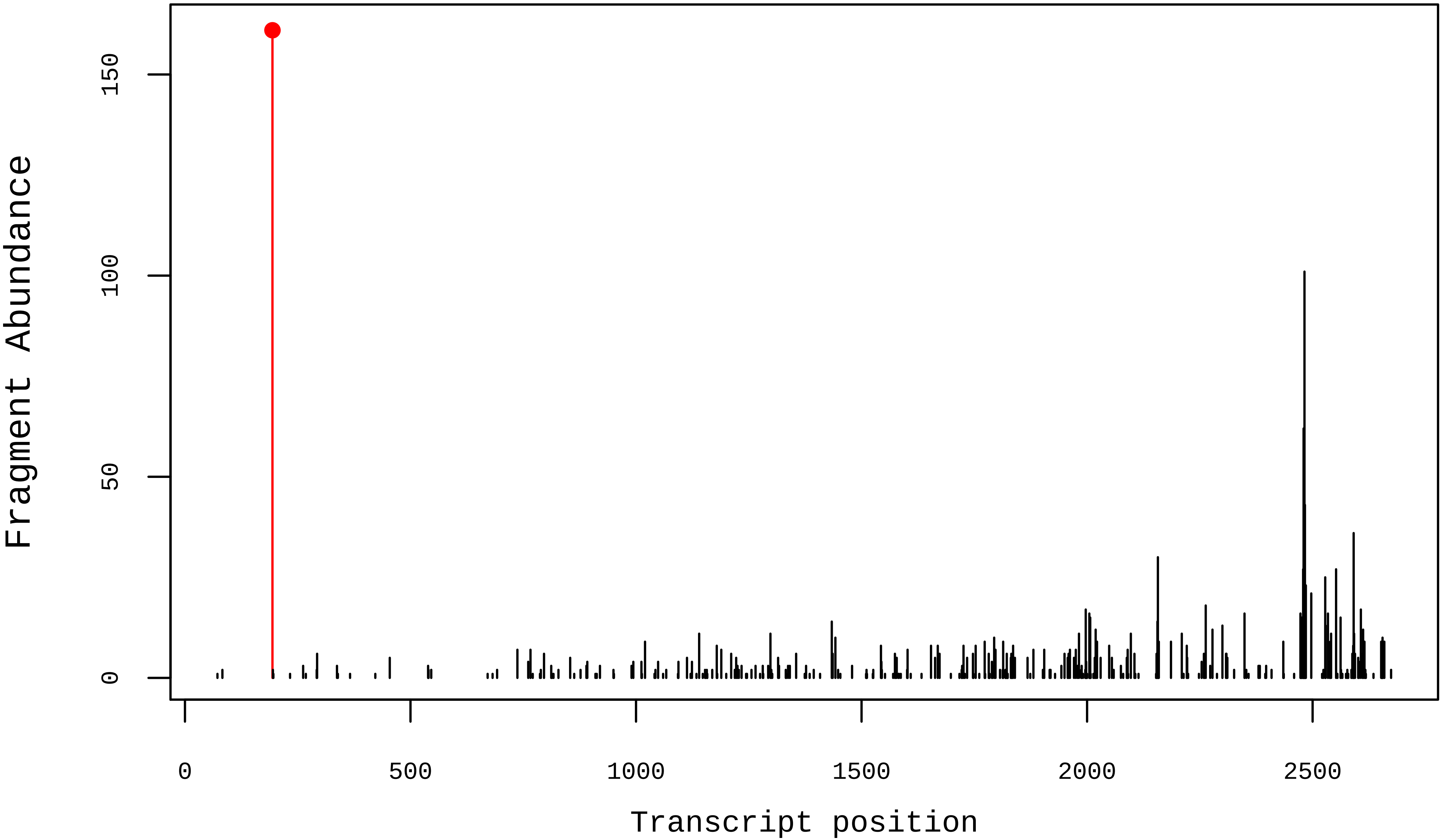


Cleavage site: 194 Tag abundance: 161 Weighted abundance: 9.471 Category: 0
sRNA abundance: 1 Alignment score: 3 MFE ratio: 0.773 p-value: 0.014

**HORVU2Hr1G094690|HORVU2Hr1G094690.3||2112|2734**

```
5' GGCCAGGTTTGCTGATGTTCATCTAACTAGCC '3
          | ||||||||||| ||||||||
3'        TACAAACGACTACCAGTAGATT       '5
```

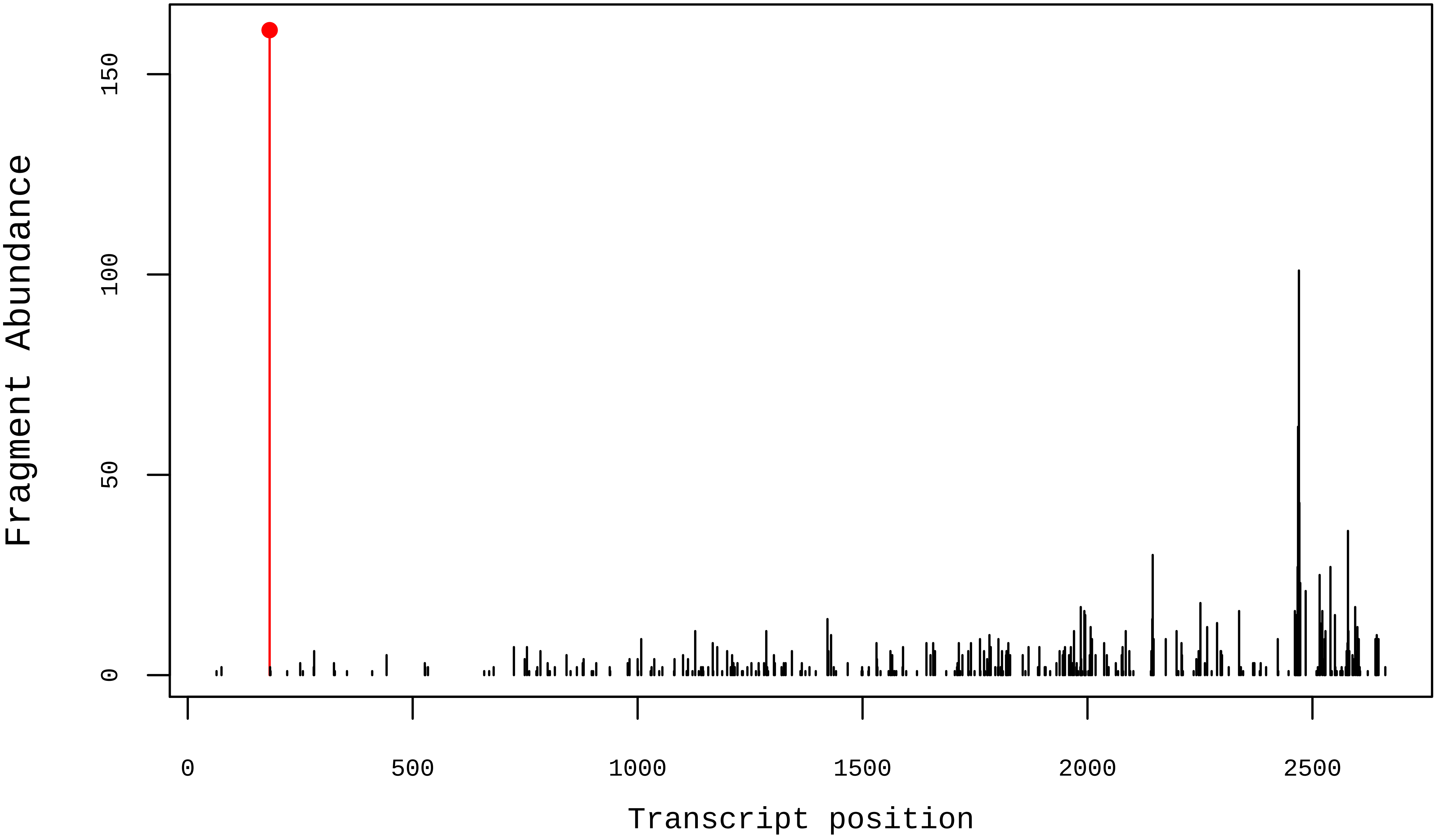


**Cleavage site: 182   Tag abundance: 161   Weighted abundance: 9.471   Category: 0**
**sRNA abundance: 1   Alignment score: 3   MFE ratio: 0.773   p-value: 0.014**

HORVU2Hr1G094690|HORVU2Hr1G094690.4||2112|2735

```
5' GGCCAGGTTTGCTGATGTTCATCTAACTAGCC '3
          | |||||||||||| |||||||||
3'        TACAAACGACTACCAGTAGATT       '5
```

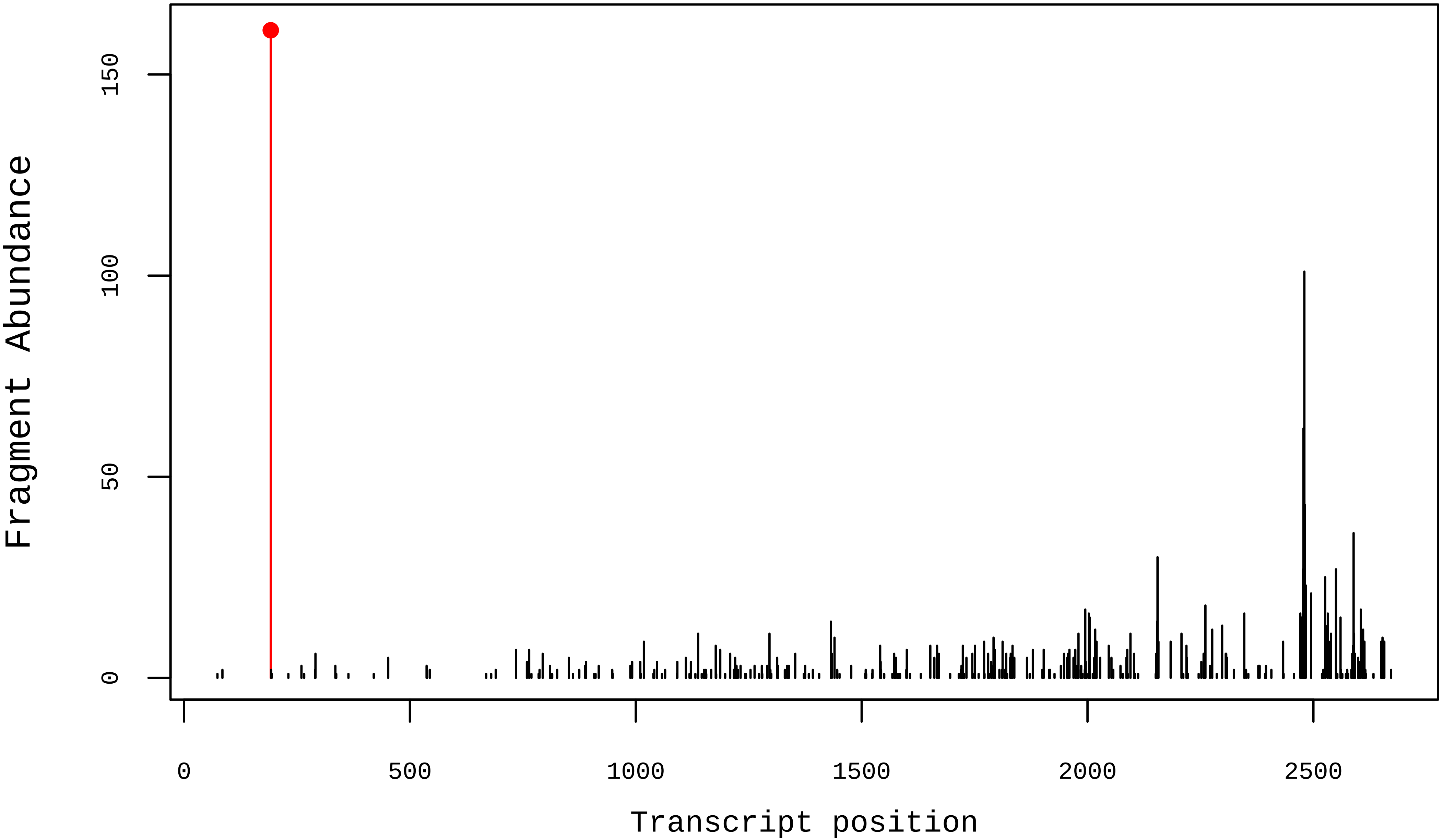


Cleavage site: 192  Tag abundance: 161  Weighted abundance: 9.471  Category: 0
sRNA abundance: 1  Alignment score: 3  MFE ratio: 0.773  p-value: 0.014

**HORVU2Hr1G094690|HORVU2Hr1G094690.7||720|2747**

```
5' GGCCAGGTTTGCTGATGTTCATCTAACTAGCC '3
         | ||||||||||| ||||||||
3'       TACAAACGACTACCAGTAGATT       '5
```

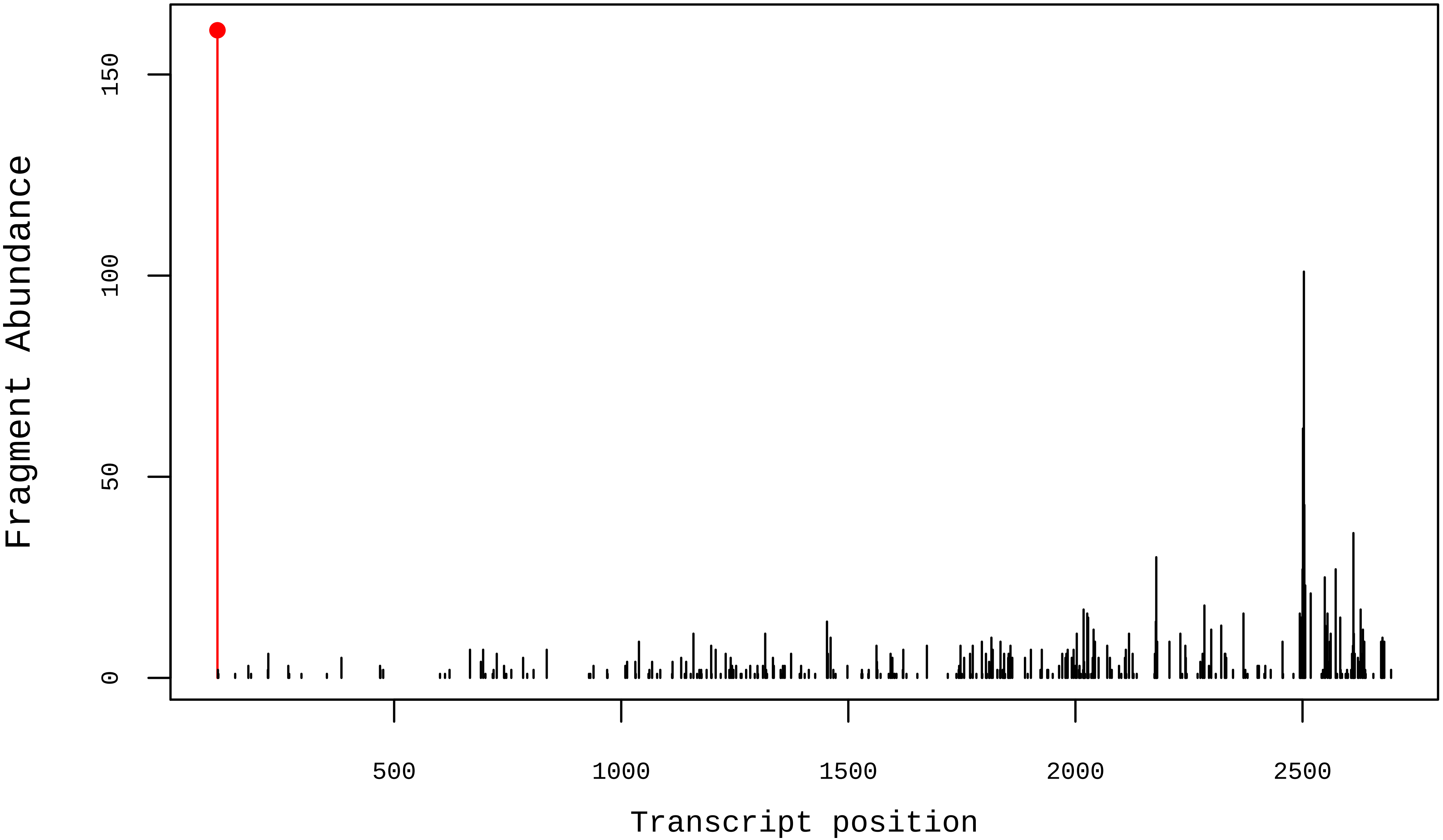


**Cleavage site: 111   Tag abundance: 161   Weighted abundance: 9.471   Category: 0**
**sRNA abundance: 1   Alignment score: 3   MFE ratio: 0.773   p-value: 0.014**

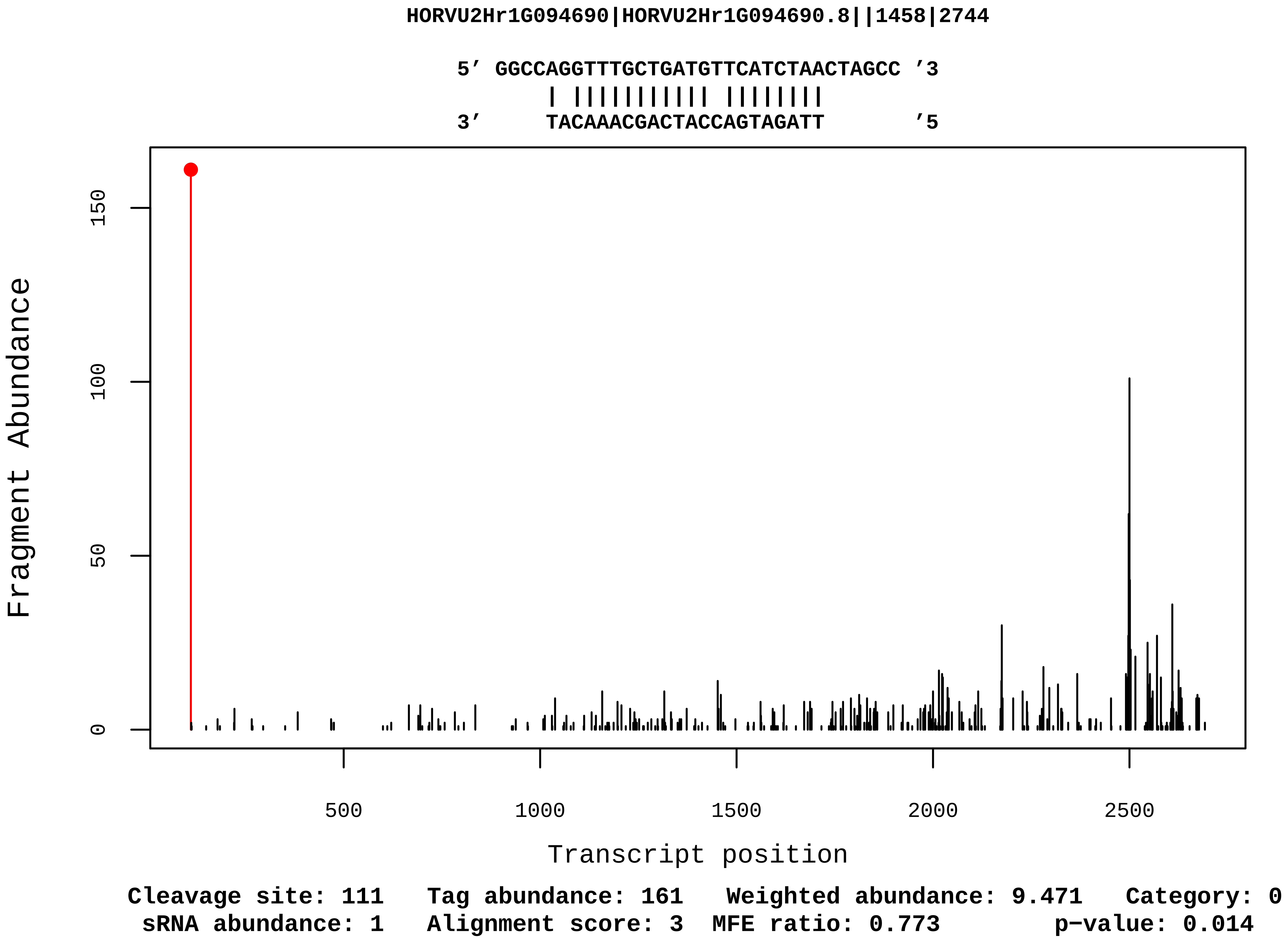
HORVU2Hr1G094690|HORVU2Hr1G094690.8||1458|2744
5′ GGCCAGGTTTGCTGATGTTCATCTAACTAGCC ′3
3′ TACAAACGACTACCAGTAGATT ′5
Fragment Abundance
0
50
100
150
500
1000
1500
2000
2500
Transcript position
Cleavage site: 111 Tag abundance: 161 Weighted abundance: 9.471 Category: 0
sRNA abundance: 1 Alignment score: 3 MFE ratio: 0.773 p-value: 0.014

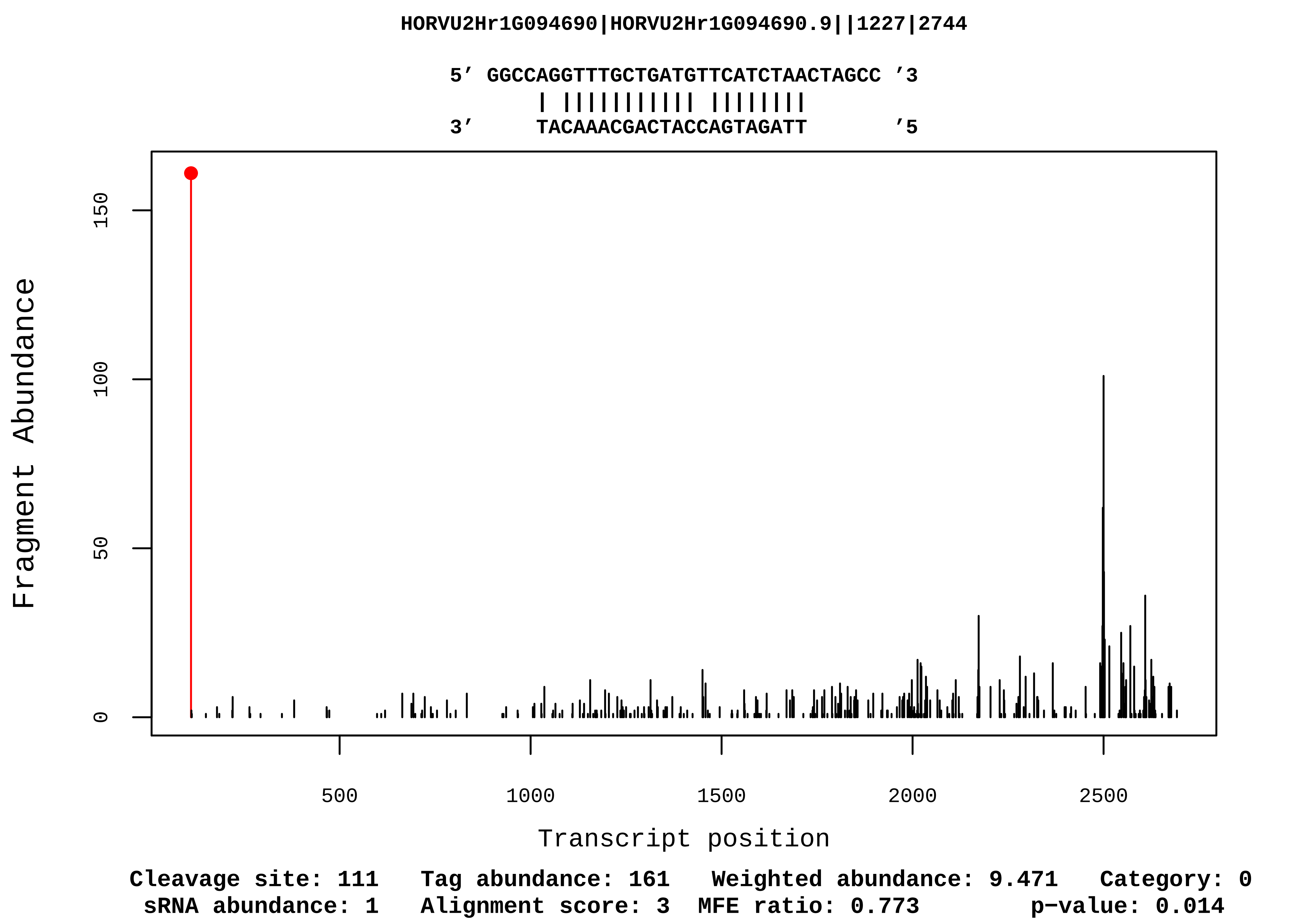
HORVU2Hr1G094690|HORVU2Hr1G094690.9||1227|2744
5' GGCCAGGTTTGCTGATGTTCATCTAACTAGCC '3
| |||||||||||| ||||||||
3' TACAAACGACTACCAGTAGATT '5
Fragment Abundance
0
50
100
150
500
1000
1500
2000
2500
Transcript position
Cleavage site: 111 Tag abundance: 161 Weighted abundance: 9.471 Category: 0
sRNA abundance: 1 Alignment score: 3 MFE ratio: 0.773 p-value: 0.014

HORVU2Hr1G094690|HORVU2Hr1G094690.10||1458|2742

```
5' GGCCAGGTTTGCTGATGTTCATCTAACTAGCC '3
         |||||||||||| |||||||||
3'       CAAACGACTACCAGTAGATT       '5
```

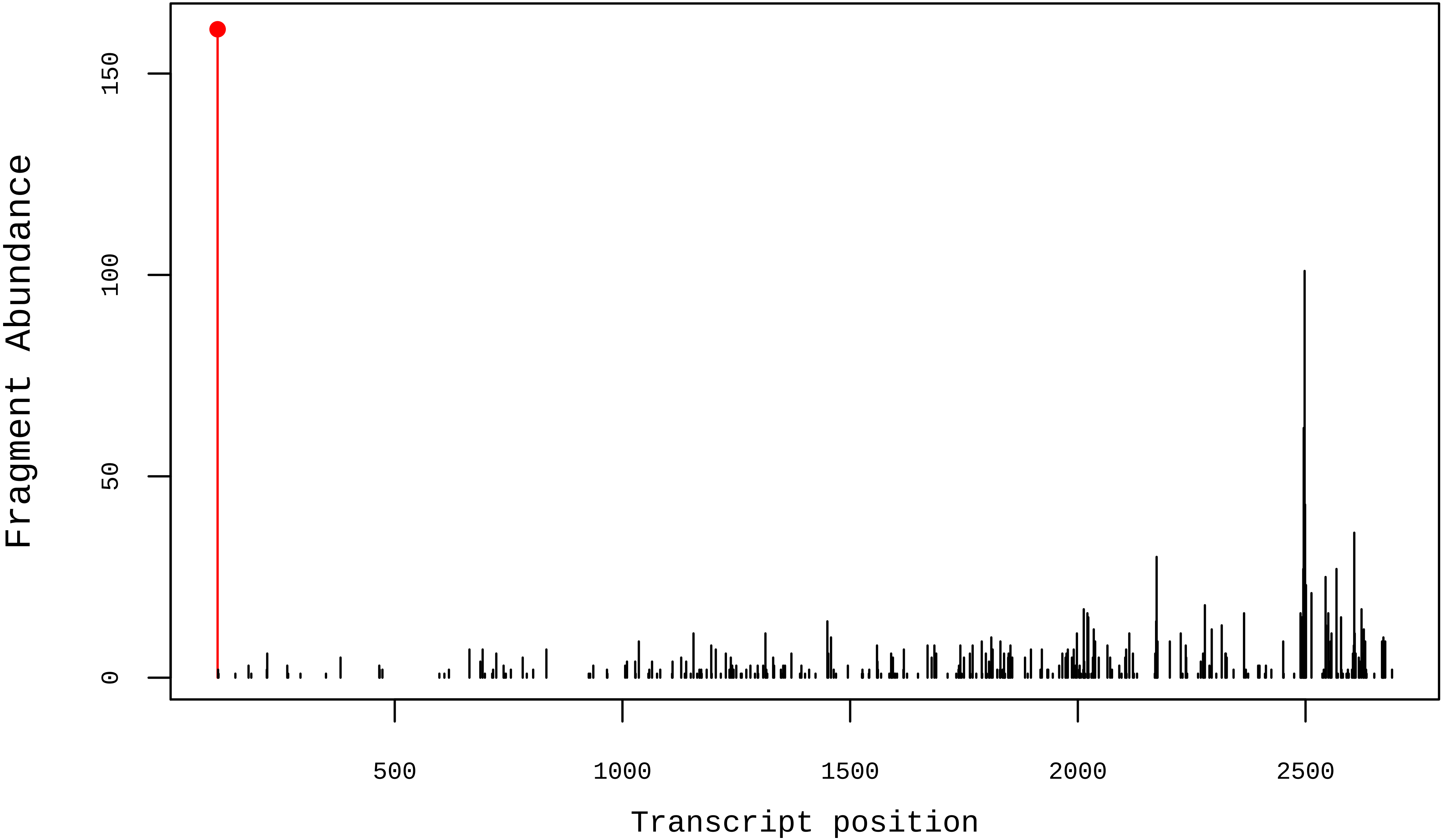


Cleavage site: 111   Tag abundance: 161   Weighted abundance: 9.471   Category: 0
sRNA abundance: 1   Alignment score: 2   MFE ratio: 0.794   p-value: 0.014

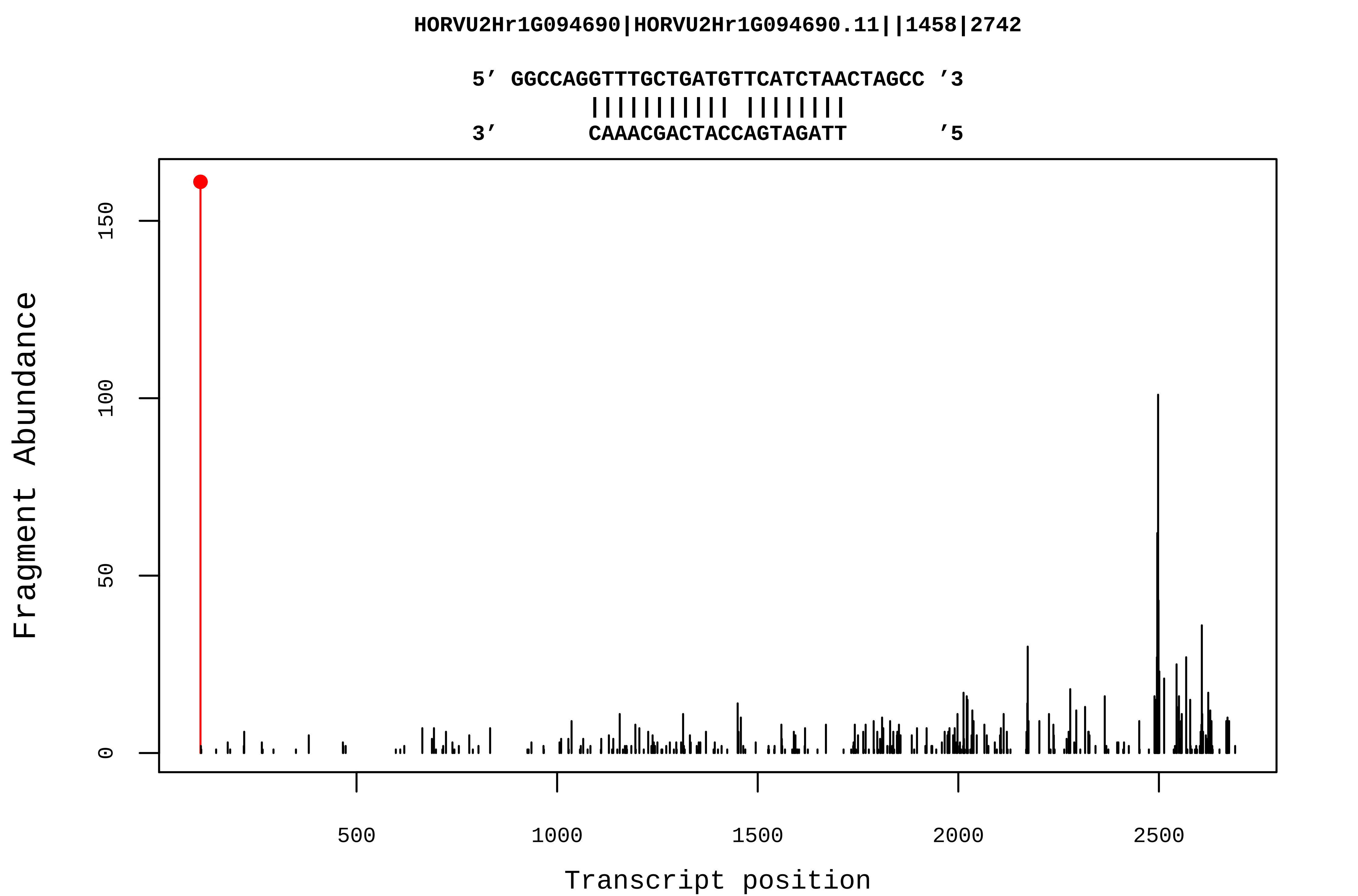


Cleavage site: 111 Tag abundance: 161 Weighted abundance: 9.471 Category: 0
sRNA abundance: 1 Alignment score: 2 MFE ratio: 0.794 p-value: 0.014

HORVU2Hr1G094690|HORVU2Hr1G094690.12||2112|2910

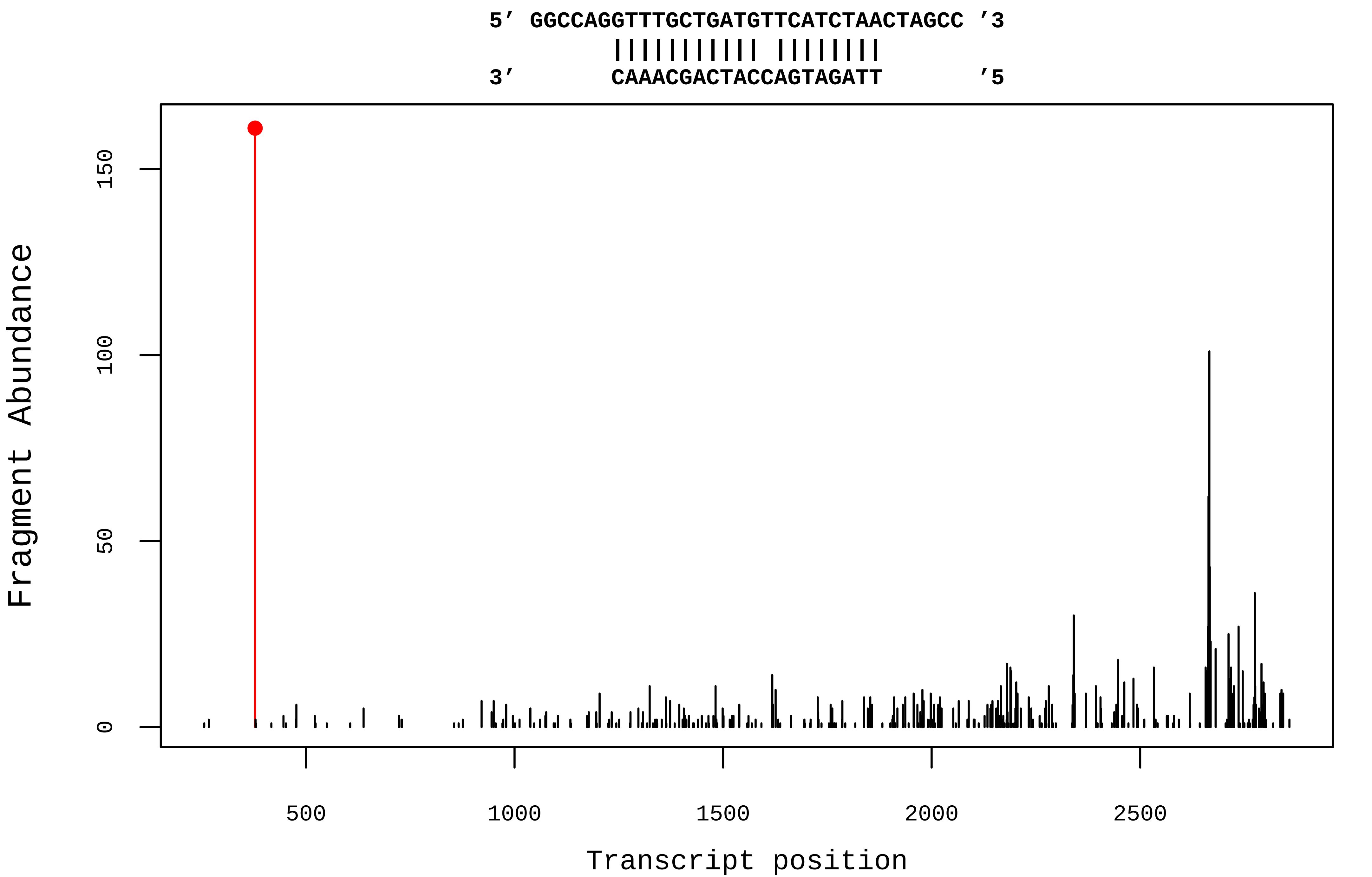


Cleavage site: 378 Tag abundance: 161 Weighted abundance: 9.471 Category: 0
sRNA abundance: 1 Alignment score: 2 MFE ratio: 0.794 p-value: 0.013

HORVU2Hr1G094690|HORVU2Hr1G094690.13||2112|2916

```
5' GGCCAGGTTTGCTGATGTTCATCTAACTAGCC '3
         |||||||||||| |||||||||
3'       CAAACGACTACCAGTAGATT       '5
```

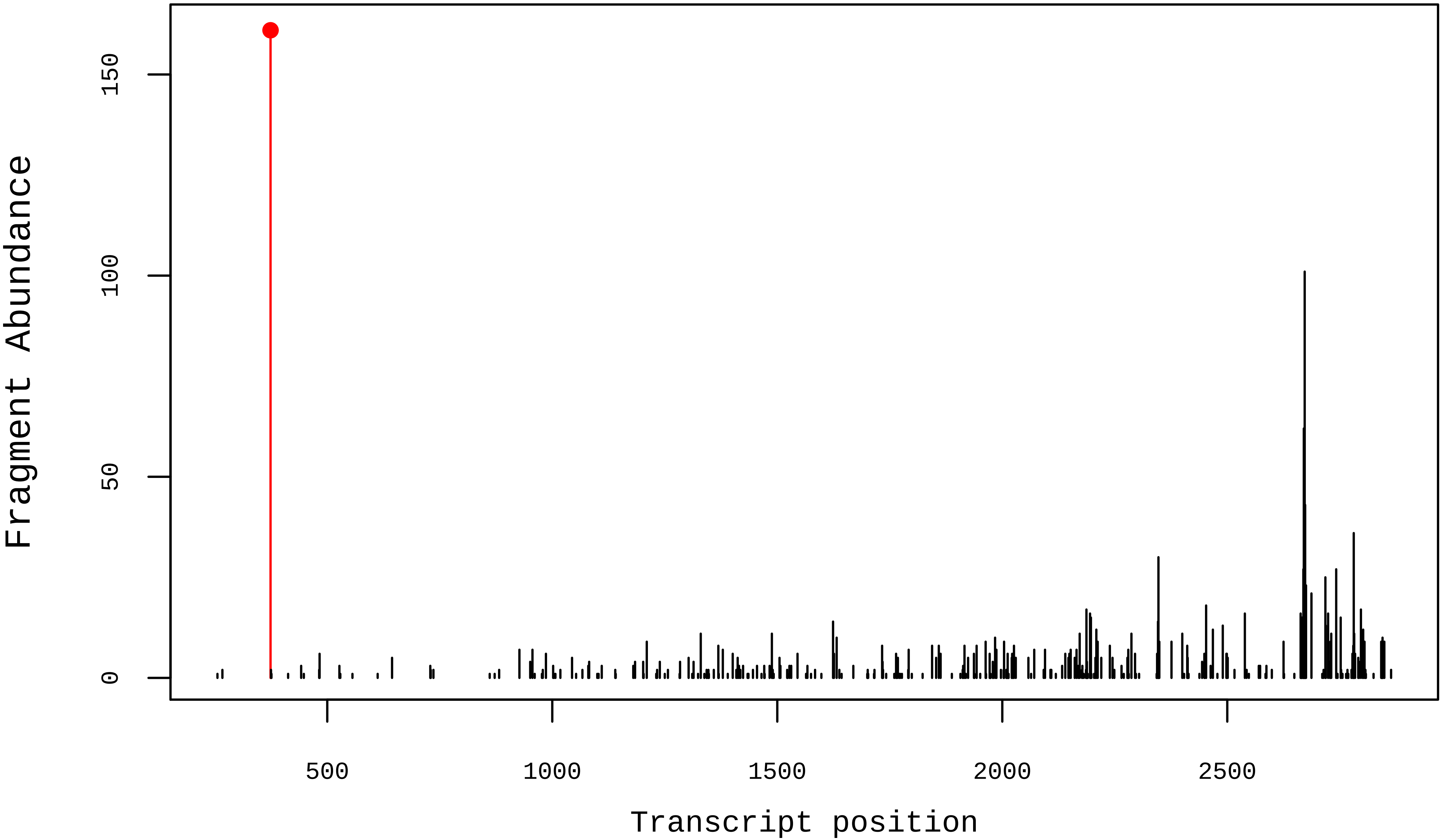


**Cleavage site: 374   Tag abundance: 161   Weighted abundance: 9.471   Category: 0**
**sRNA abundance: 1   Alignment score: 2   MFE ratio: 0.794   p-value: 0.013**

HORVU2Hr1G094690|HORVU2Hr1G094690.14||2112|2920

```
5' GGCCAGGTTTGCTGATGTTCATCTAACTAGCC '3
         ||||||||||| ||||||||
3'       CAAACGACTACCAGTAGATT       '5
```

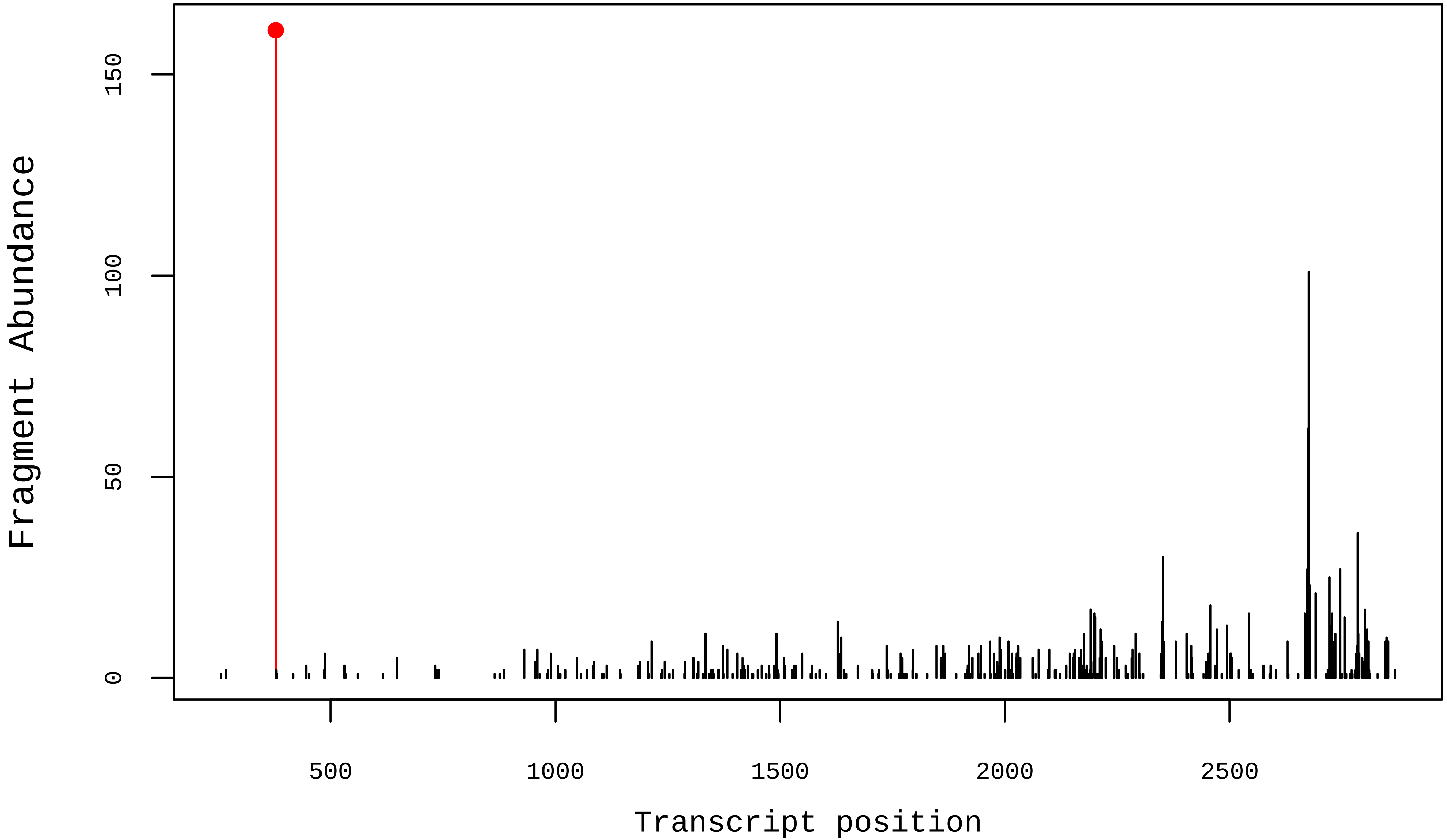


**Cleavage site: 378   Tag abundance: 161   Weighted abundance: 9.471   Category: 0**
**sRNA abundance: 1   Alignment score: 2   MFE ratio: 0.794   p-value: 0.013**

HORVU2Hr1G094690|HORVU2Hr1G094690.15||2112|2920

```
5’ GGCCAGGTTTGCTGATGTTCATCTAACTAGCC ’3
         |||||||||||| |||||||||
3’       CAAACGACTACCAGTAGATT        ’5
```

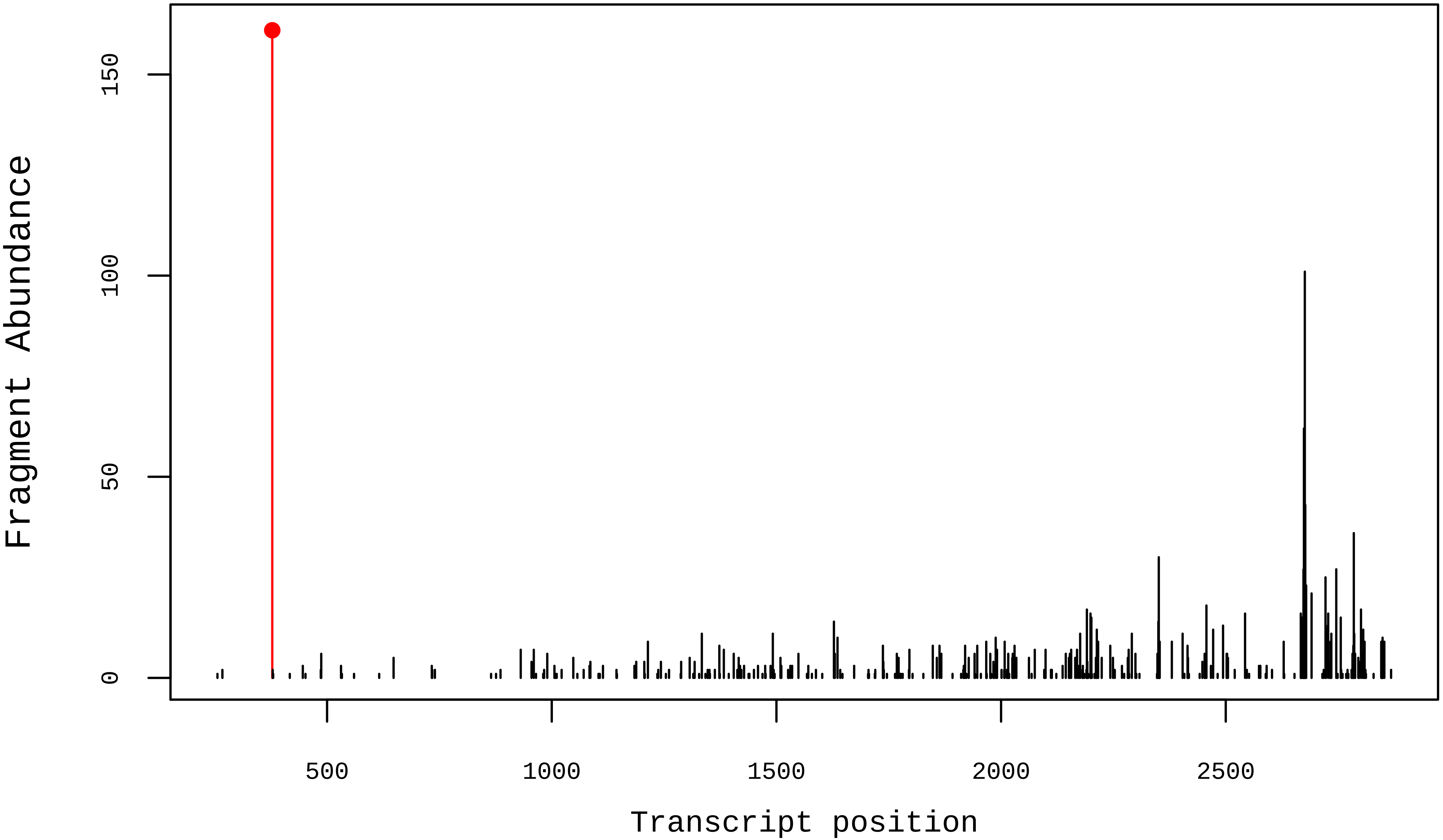


Cleavage site: 378   Tag abundance: 161   Weighted abundance: 9.471   Category: 0
sRNA abundance: 1   Alignment score: 2   MFE ratio: 0.794   p-value: 0.013

HORVU2Hr1G094690|HORVU2Hr1G094690.16||1458|2775

```
5' GGCCAGGTTTGCTGATGTTCATCTAACTAGCC '3
         ||||||||||| ||||||||
3'       CAAACGACTACCAGTAGATT       '5
```

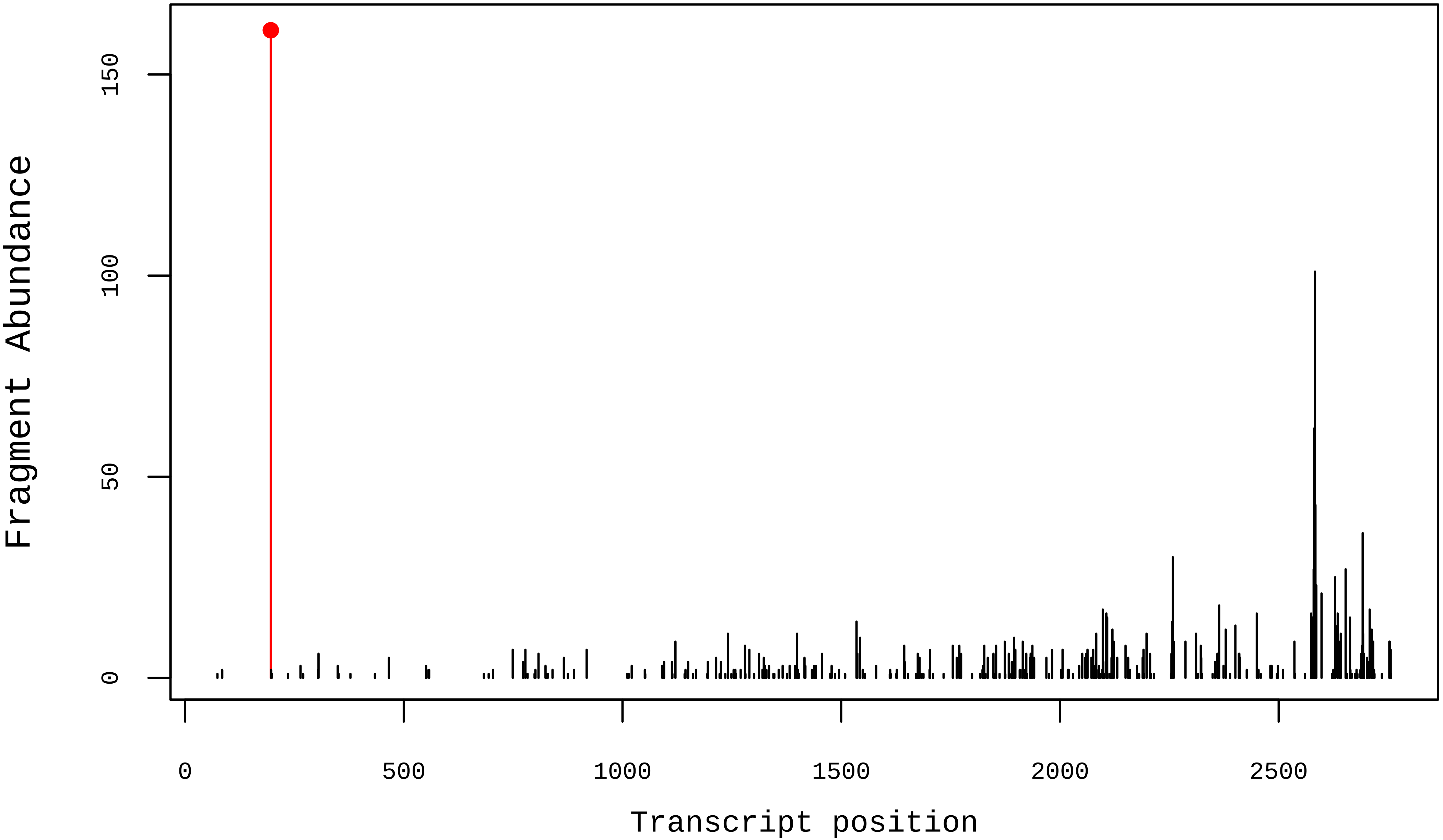


**Cleavage site: 196   Tag abundance: 161   Weighted abundance: 9.471   Category: 0**
**sRNA abundance: 1   Alignment score: 2   MFE ratio: 0.794   p-value: 0.014**

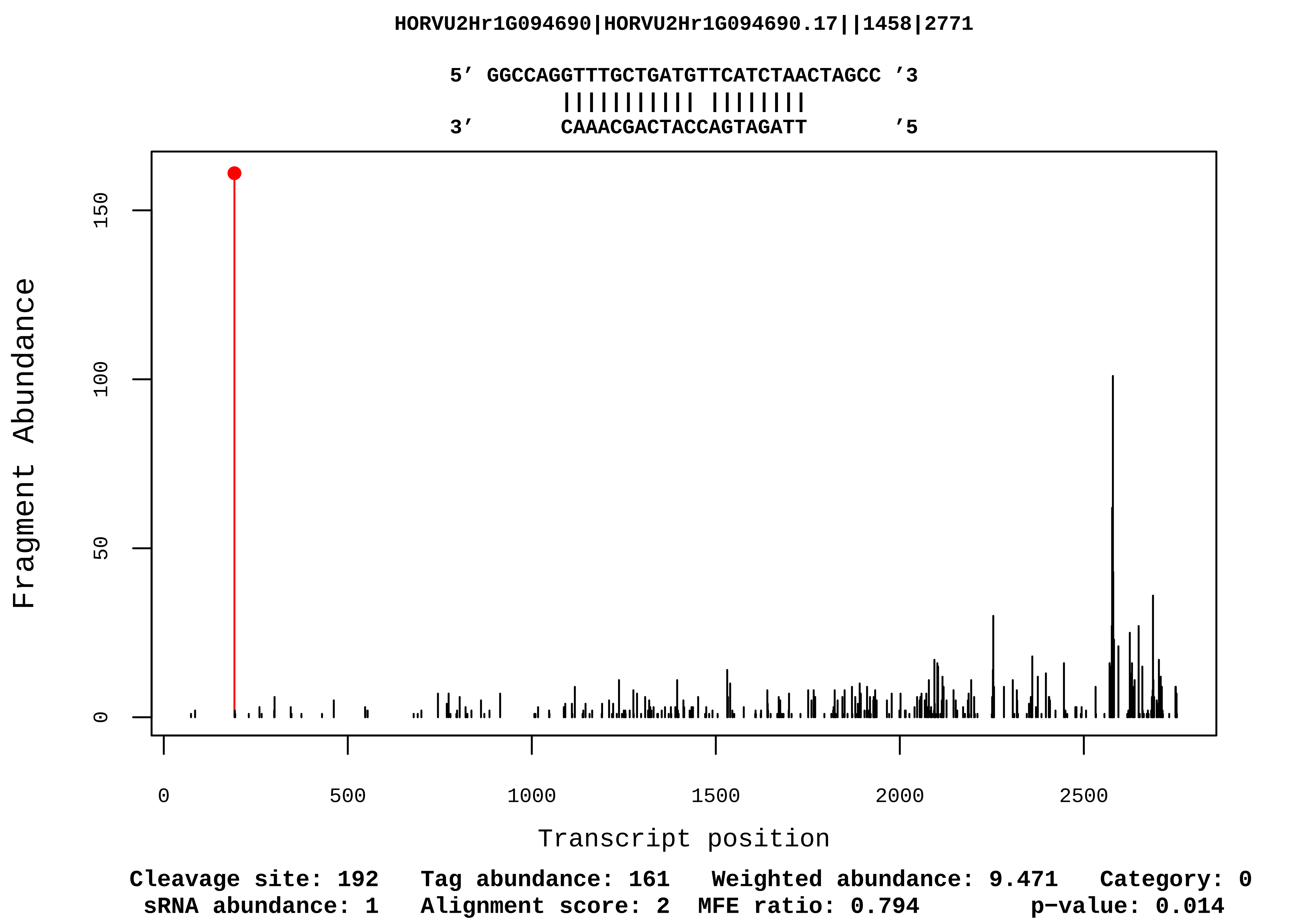
HORVU2Hr1G094690|HORVU2Hr1G094690.17||1458|2771
5′ GGCCAGGTTTGCTGATGTTCATCTAACTAGCC ′3
||||||||||| ||||||||
3′ CAAACGACTACCAGTAGATT ′5
Fragment Abundance
0
50
100
150
0
500
1000
1500
2000
2500
Transcript position
Cleavage site: 192 Tag abundance: 161 Weighted abundance: 9.471 Category: 0
sRNA abundance: 1 Alignment score: 2 MFE ratio: 0.794 p-value: 0.014

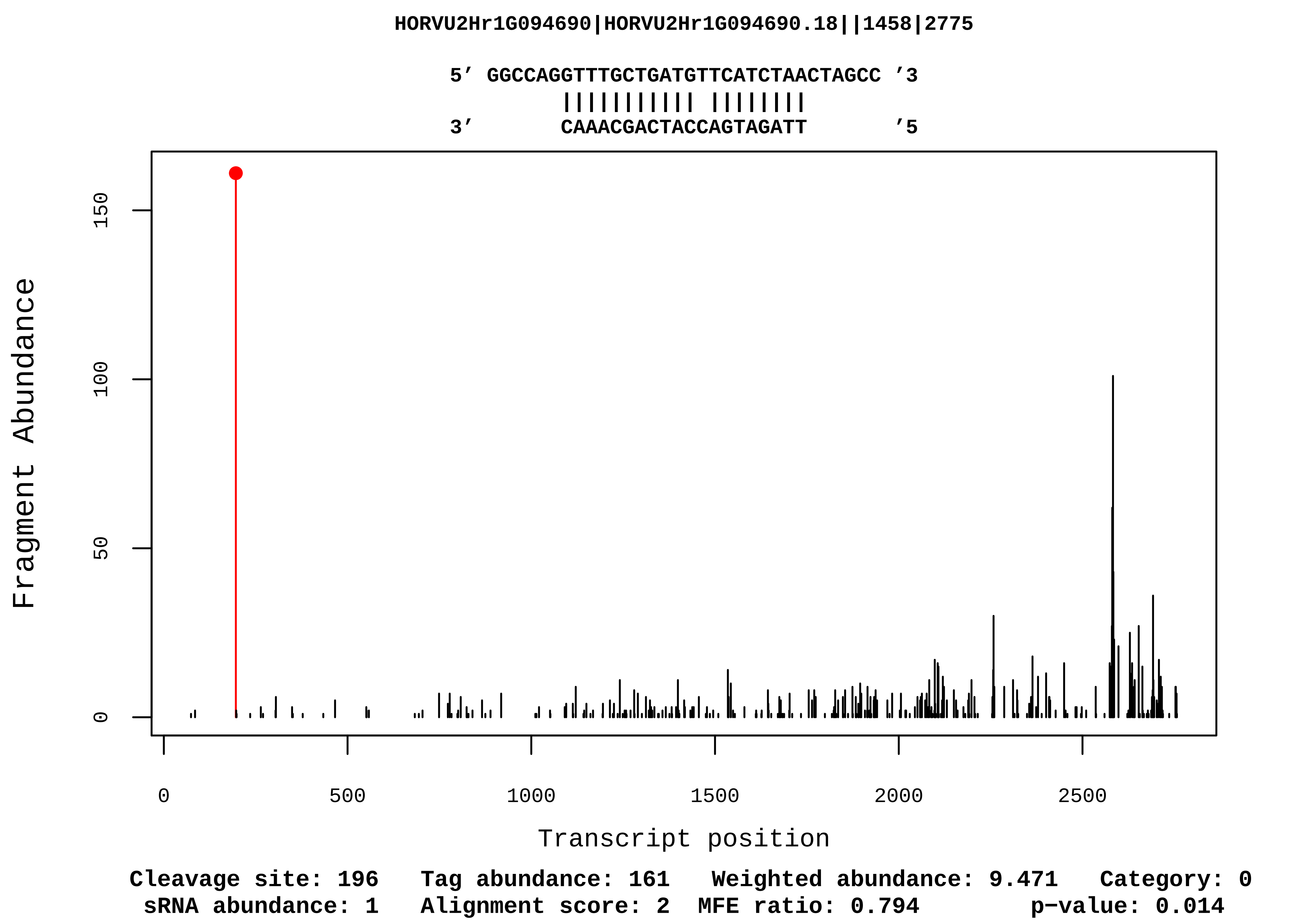
HORVU2Hr1G094690|HORVU2Hr1G094690.18||1458|2775
5′ GGCCAGGTTTGCTGATGTTCATCTAACTAGCC ′3
||||||||||| ||||||||
3′ CAAACGACTACCAGTAGATT ′5
Fragment Abundance
0
50
100
150
0
500
1000
1500
2000
2500
Transcript position
Cleavage site: 196 Tag abundance: 161 Weighted abundance: 9.471 Category: 0
sRNA abundance: 1 Alignment score: 2 MFE ratio: 0.794 p−value: 0.014

HORVU2Hr1G094690|HORVU2Hr1G094690.19||1458|2630

```
5' GGCCAGGTTTGCTGATGTTCATCTAACTAGCC '3
         |||||||||||| ||||||||
3'       CAAACGACTACCAGTAGATT       '5
```

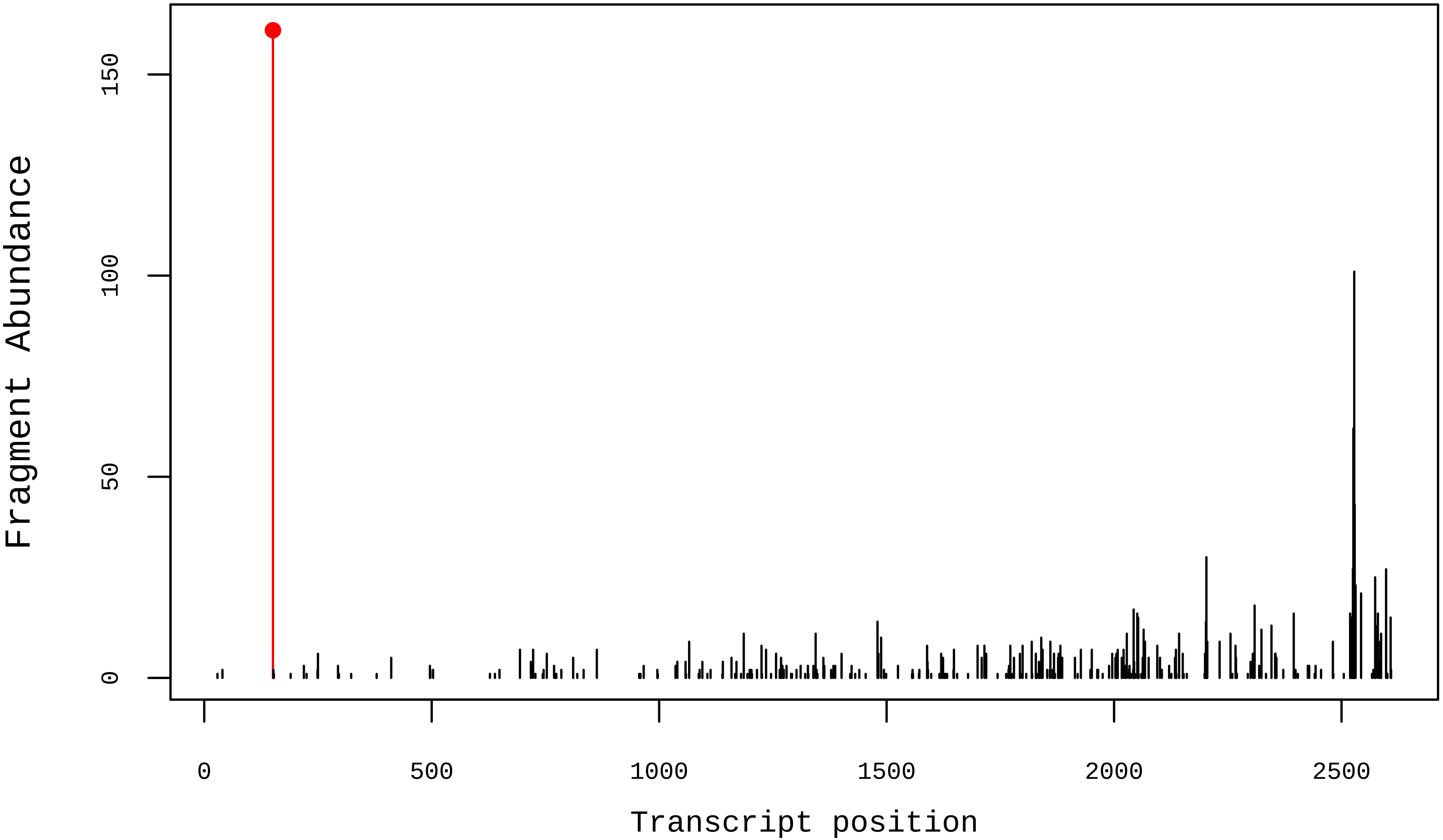


**Cleavage site: 151   Tag abundance: 161   Weighted abundance: 9.471   Category: 0**
**sRNA abundance: 1   Alignment score: 2   MFE ratio: 0.794   p-value: 0.014**

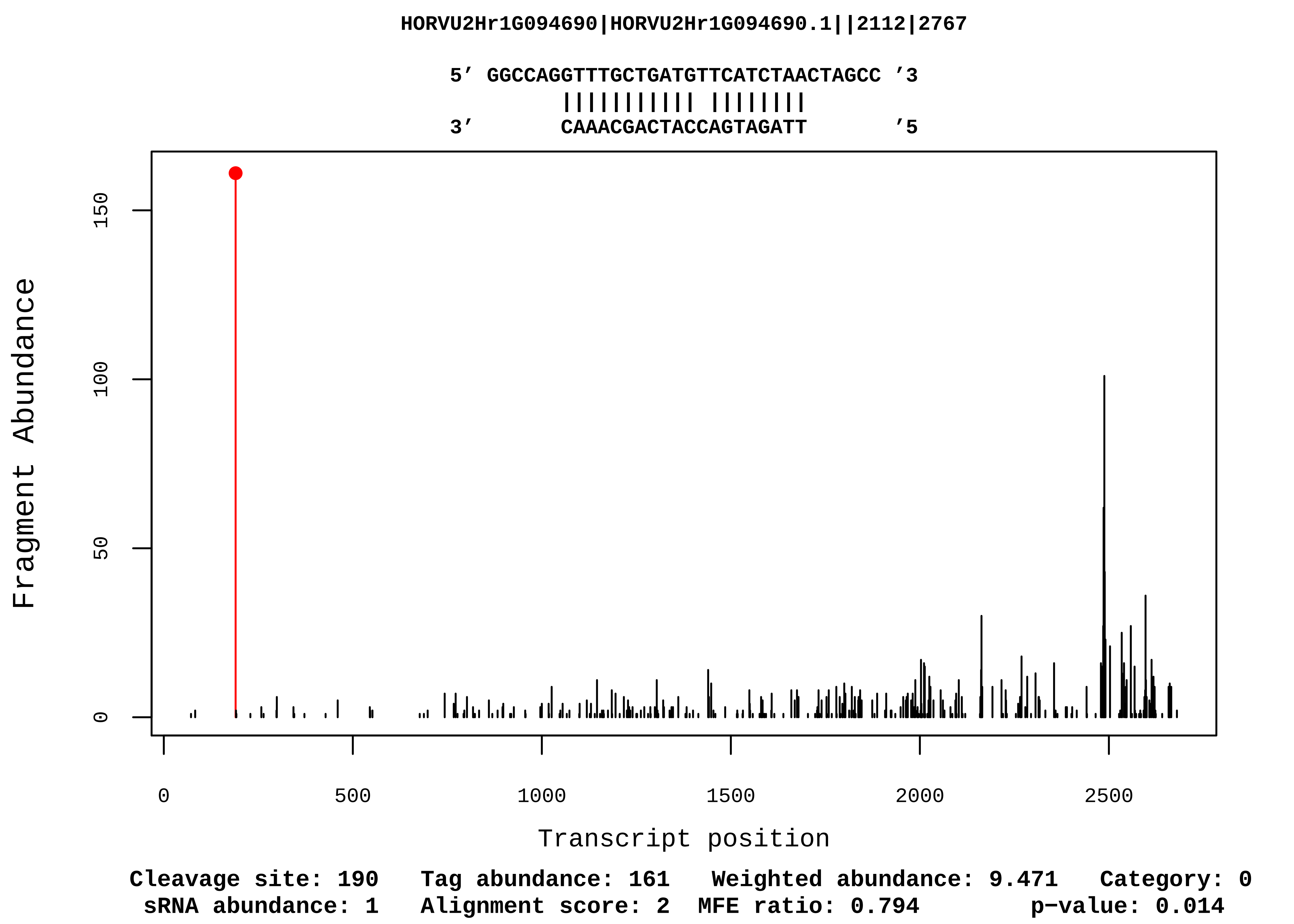
HORVU2Hr1G094690|HORVU2Hr1G094690.1||2112|2767
5' GGCCAGGTTTGCTGATGTTCATCTAACTAGCC '3
|||||||||||| ||||||||
3' CAAACGACTACCAGTAGATT '5
Fragment Abundance
0
50
100
150
0
500
1000
1500
2000
2500
Transcript position
Cleavage site: 190 Tag abundance: 161 Weighted abundance: 9.471 Category: 0
sRNA abundance: 1 Alignment score: 2 MFE ratio: 0.794 p-value: 0.014

**HORVU2Hr1G094690|HORVU2Hr1G094690.2||2112|2761**

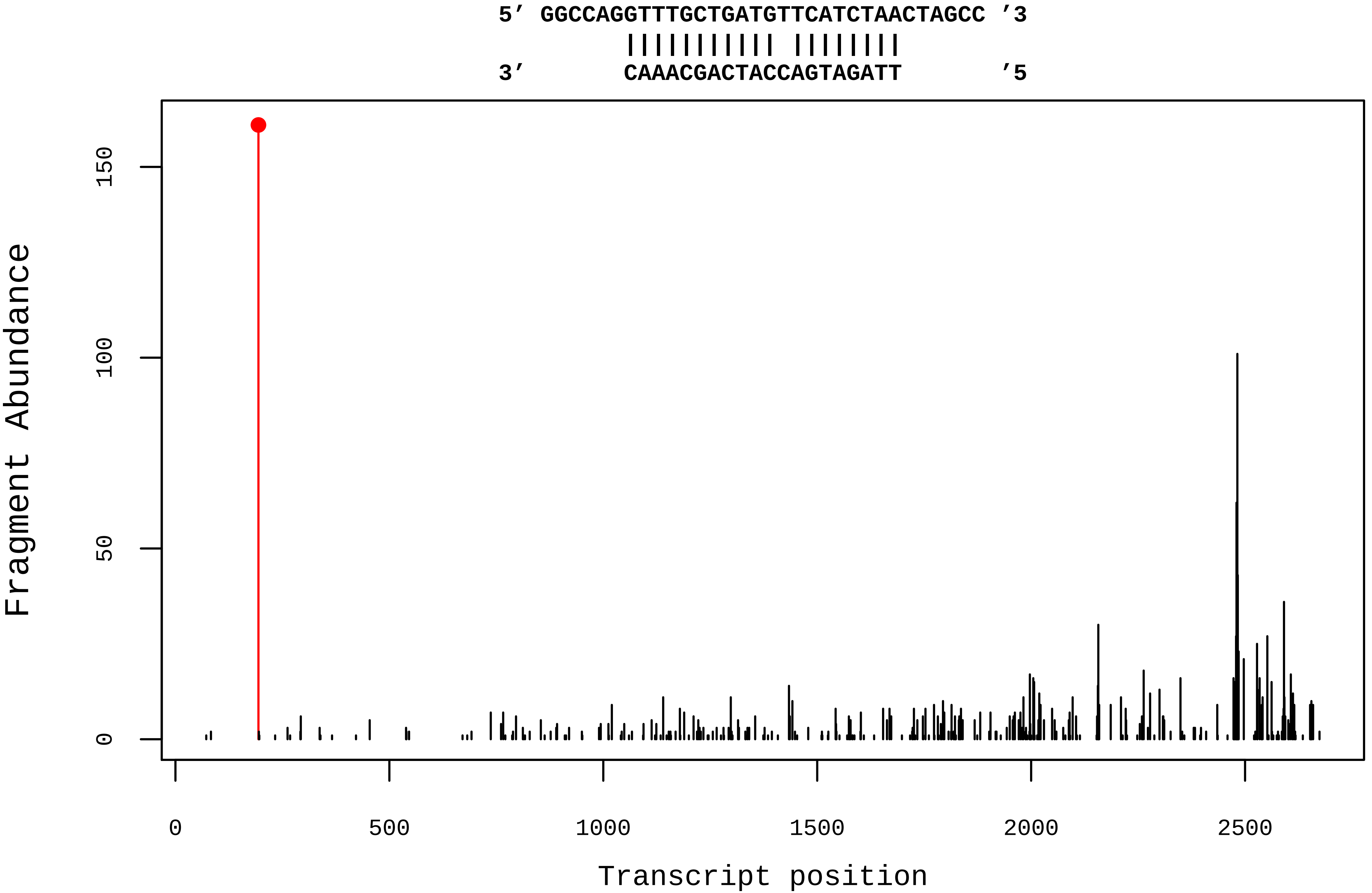


**Cleavage site: 194 Tag abundance: 161 Weighted abundance: 9.471 Category: 0**
**sRNA abundance: 1 Alignment score: 2 MFE ratio: 0.794 p-value: 0.014**

HORVU2Hr1G094690|HORVU2Hr1G094690.3||2112|2734

```
5' GGCCAGGTTTGCTGATGTTCATCTAACTAGCC '3
          |||||||||||| ||||||||
3'        CAAACGACTACCAGTAGATT        '5
```

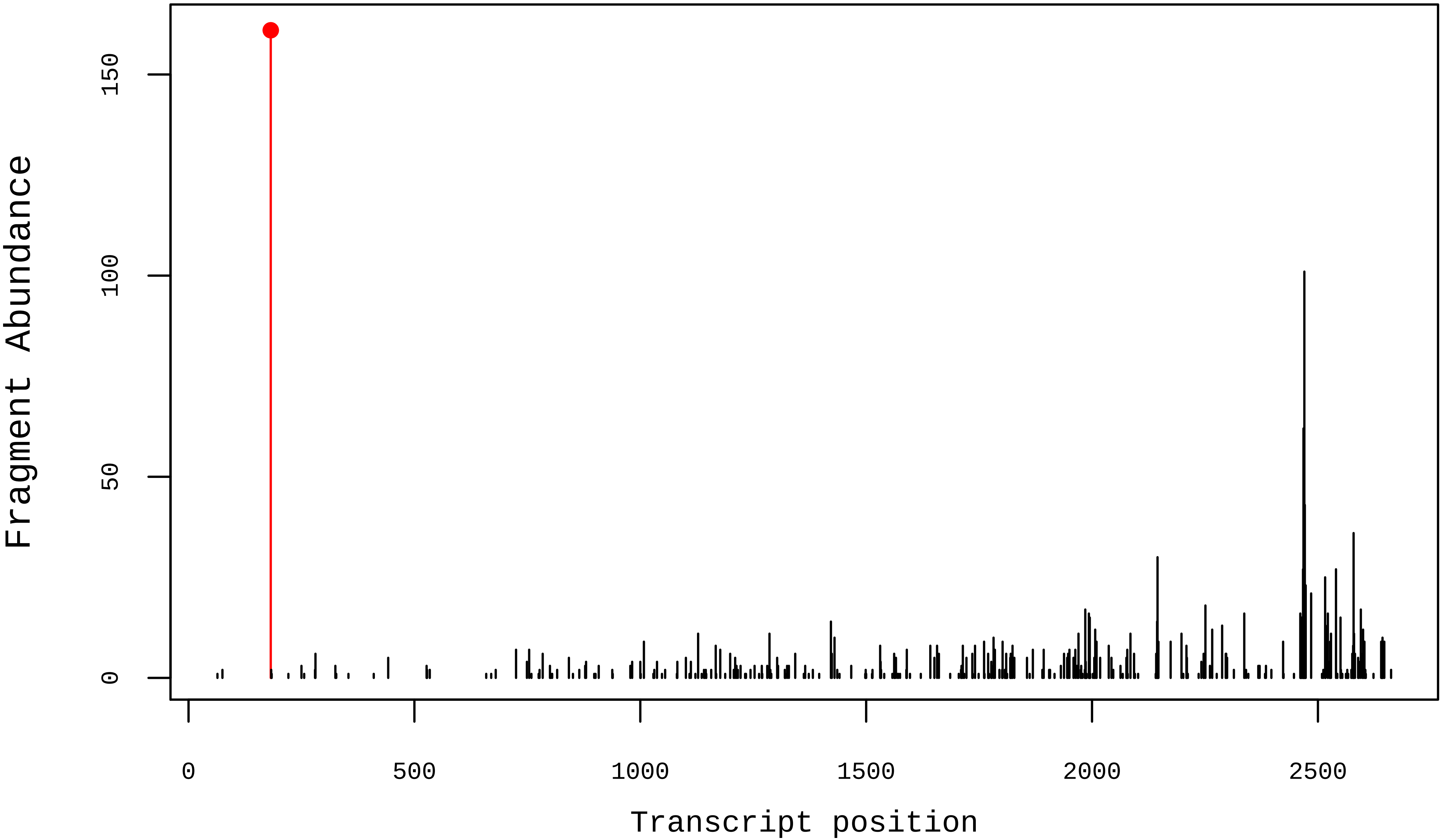


**Cleavage site: 182 Tag abundance: 161 Weighted abundance: 9.471 Category: 0**

**sRNA abundance: 1 Alignment score: 2 MFE ratio: 0.794 p-value: 0.014**

HORVU2Hr1G094690|HORVU2Hr1G094690.4||2112|2735

```
5' GGCCAGGTTTGCTGATGTTCATCTAACTAGCC '3
         ||||||||||| ||||||||
3'       CAAACGACTACCAGTAGATT       '5
```

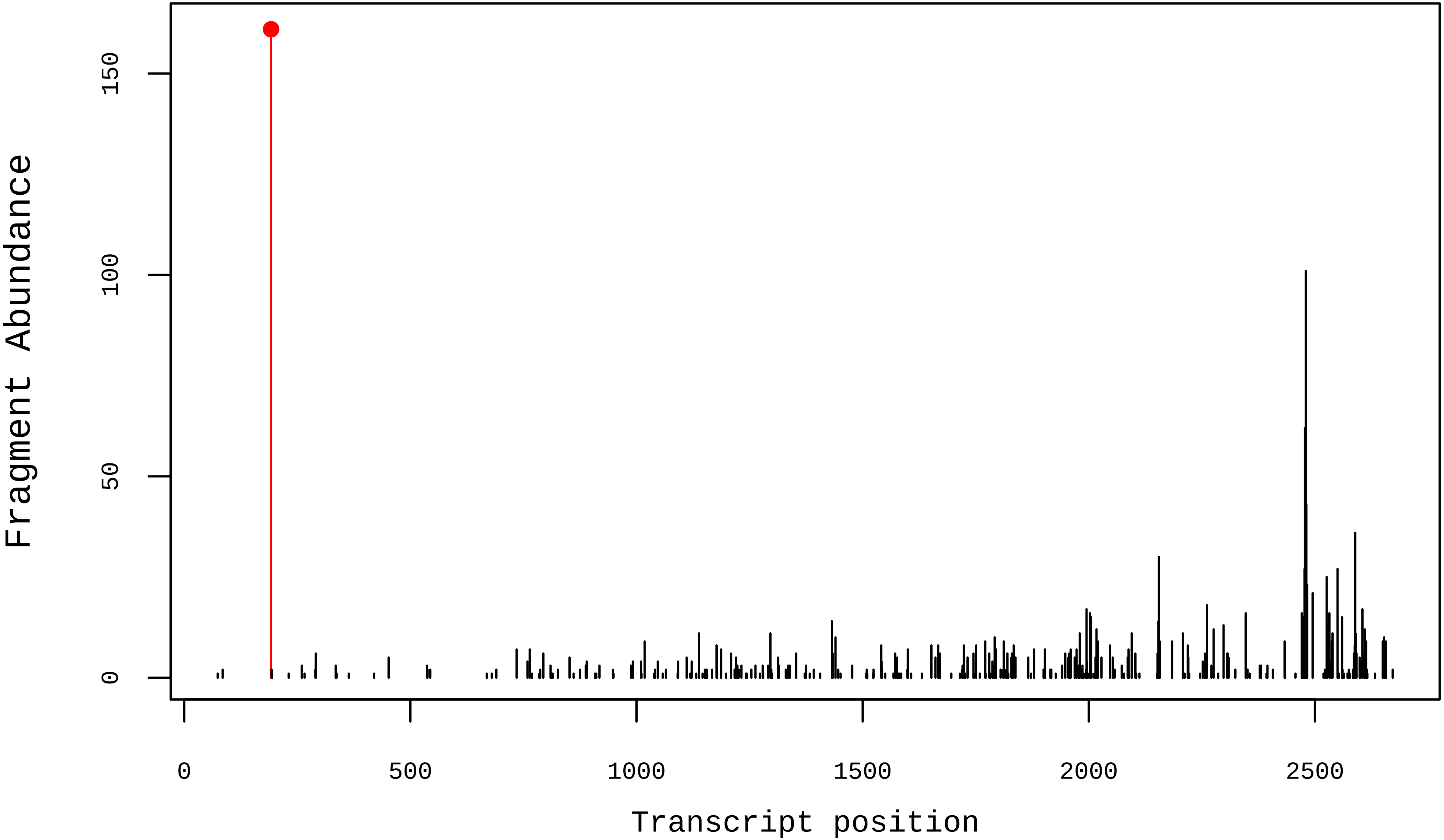


Cleavage site: 192 Tag abundance: 161 Weighted abundance: 9.471 Category: 0
sRNA abundance: 1 Alignment score: 2 MFE ratio: 0.794 p-value: 0.014

**HORVU2Hr1G094690|HORVU2Hr1G094690.7||720|2747**

```
5’ GGCCAGGTTTGCTGATGTTCATCTAACTAGCC ’3
         |||||||||||| ||||||||
3’       CAAACGACTACCAGTAGATT       ’5
```

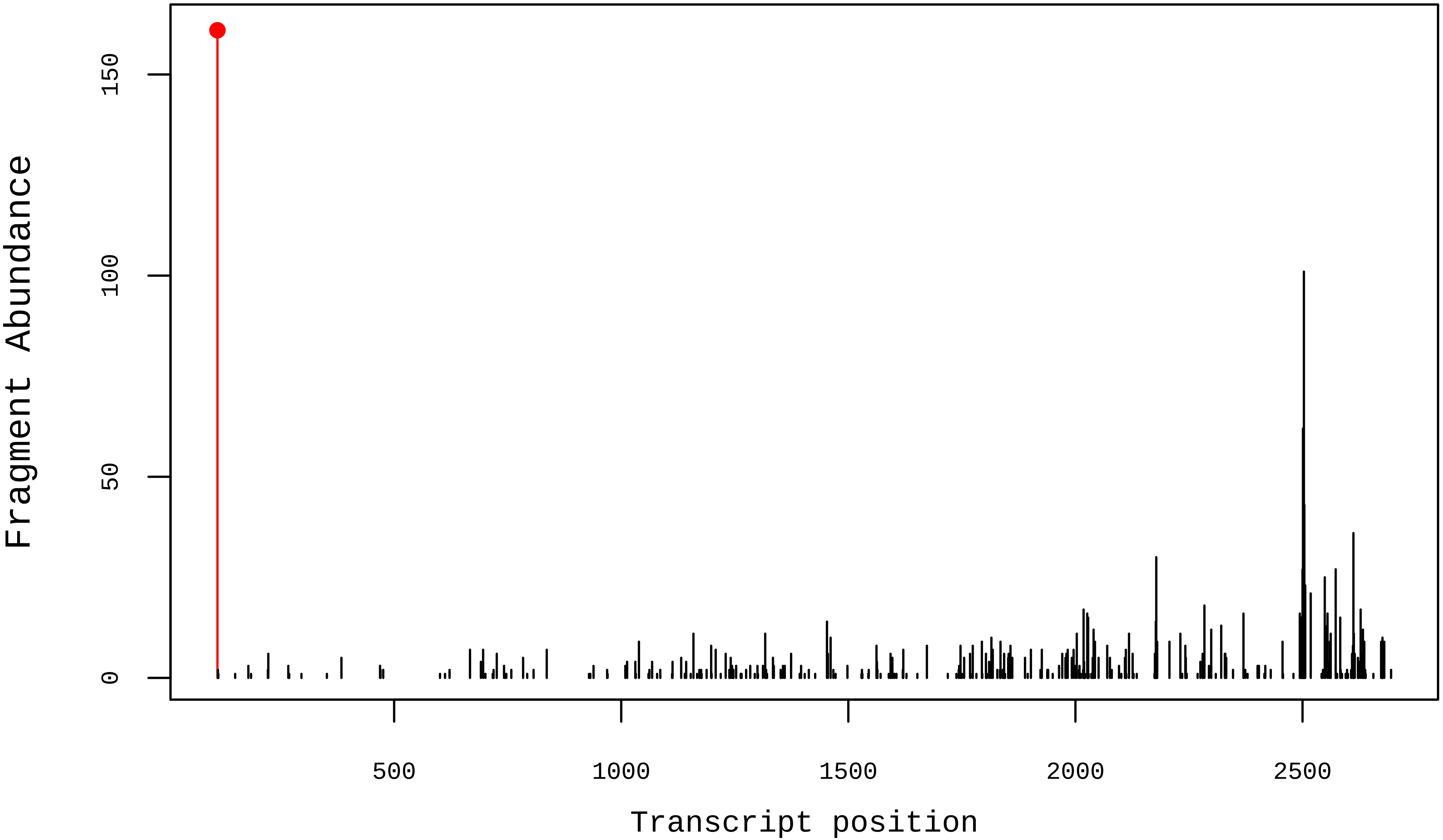


**Cleavage site: 111   Tag abundance: 161   Weighted abundance: 9.471   Category: 0**

**sRNA abundance: 1   Alignment score: 2   MFE ratio: 0.794   p-value: 0.014**

**HORVU2Hr1G094690|HORVU2Hr1G094690.8||1458|2744**

```
5' GGCCAGGTTTGCTGATGTTCATCTAACTAGCC '3
         ||||||||||| ||||||||
3'       CAAACGACTACCAGTAGATT       '5
```

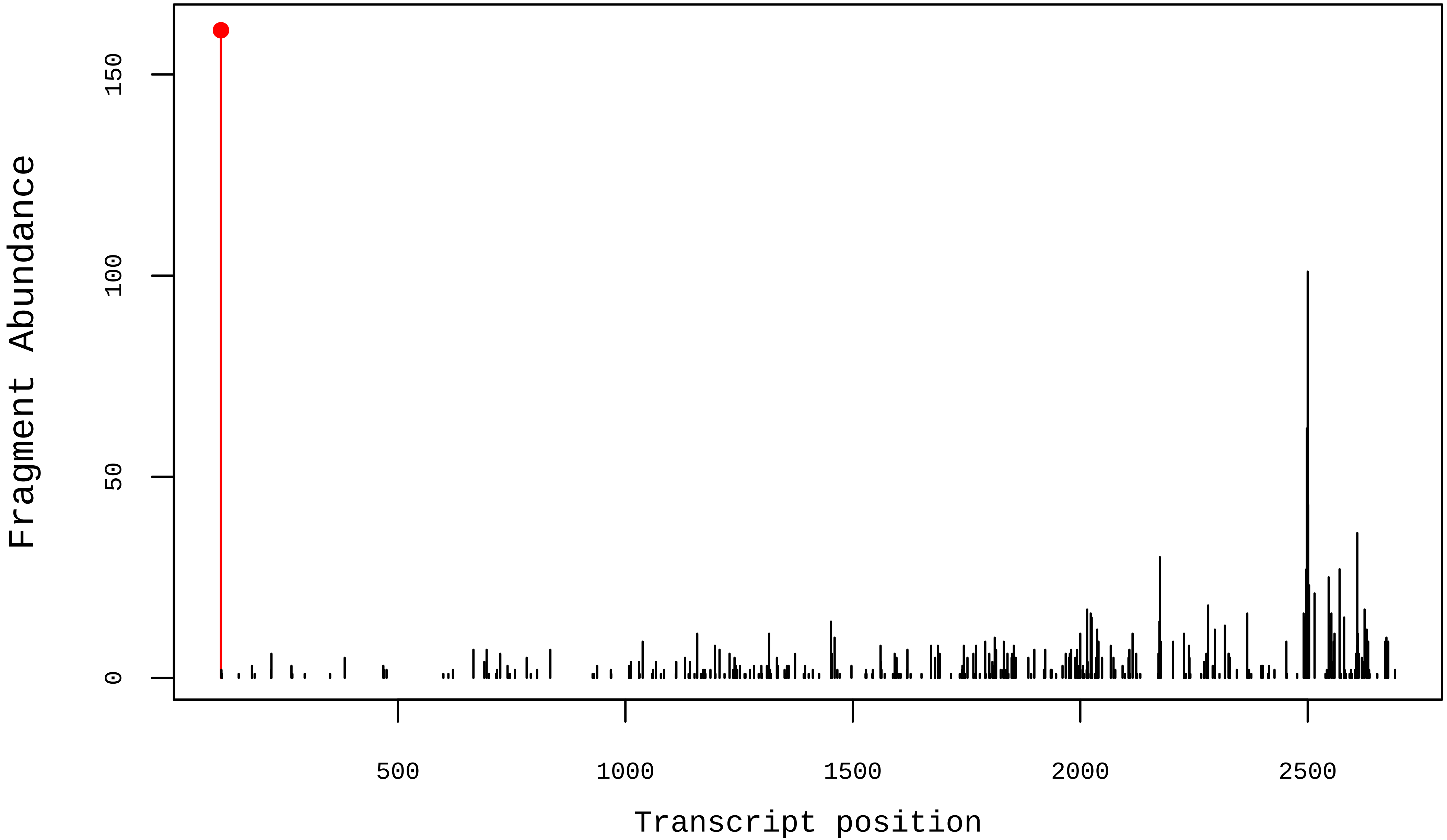


**Cleavage site: 111 Tag abundance: 161 Weighted abundance: 9.471 Category: 0**
**sRNA abundance: 1 Alignment score: 2 MFE ratio: 0.794 p-value: 0.014**

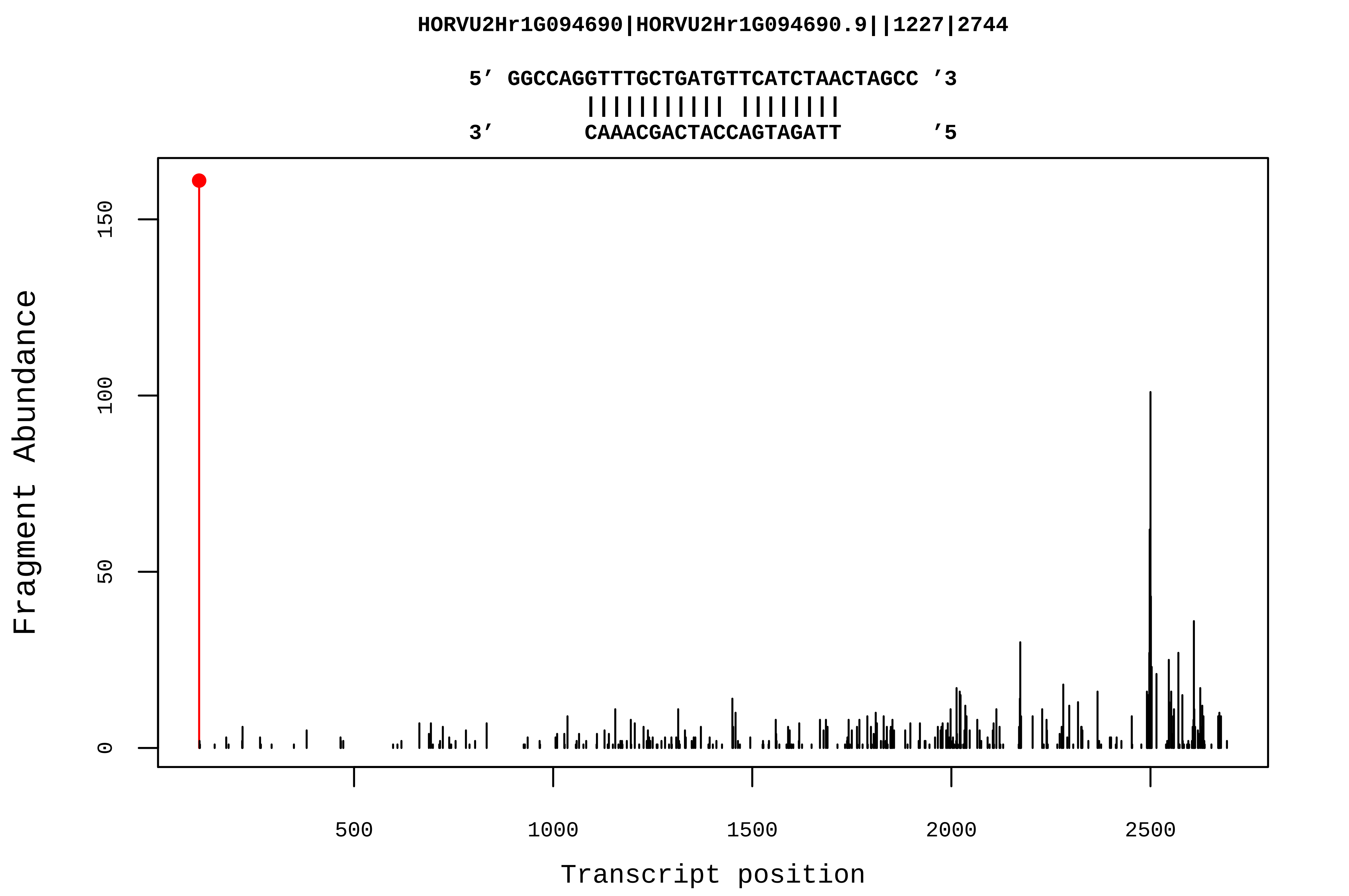


Cleavage site: 111 Tag abundance: 161 Weighted abundance: 9.471 Category: 0
sRNA abundance: 1 Alignment score: 2 MFE ratio: 0.794 p-value: 0.014
