# Supplementary material for: Pi-starvation induced transcriptional changes in barley revealed by a comprehensive RNA-Seq and degradome analyses
Source: BMC Genomics. 2021 Mar 9;22:165. doi: 10.1186/s12864-021-07481-w (PMC7941915; doi:10.1186/s12864-021-07481-w)
Supplement: Supplementary file 22 — Additional file 22. The t-plots generated by PAREsnip2 software showing the potential mRNA targets for differentially expressed other sRNAs (DESs) identified in barley shoots (low-Pi vs. control). [file 12864_2021_7481_MOESM22_ESM.pdf]

Additional file 22. The t-plots generated by PAREsnip2 software showing the potential mRNA targets for differentially expressed other sRNAs (DESs) identified in barley shoots (low-Pi vs. control)

HORVU6Hr1G028940|HORVU6Hr1G028940.11||1062|2874

```
5' ATCTATTTGTAGTCGGATCCTAGCTTATACGG '3
           ||||o|||o|||| |||||
3'         ACATTAGCTTAGGCTCGAA        '5
```

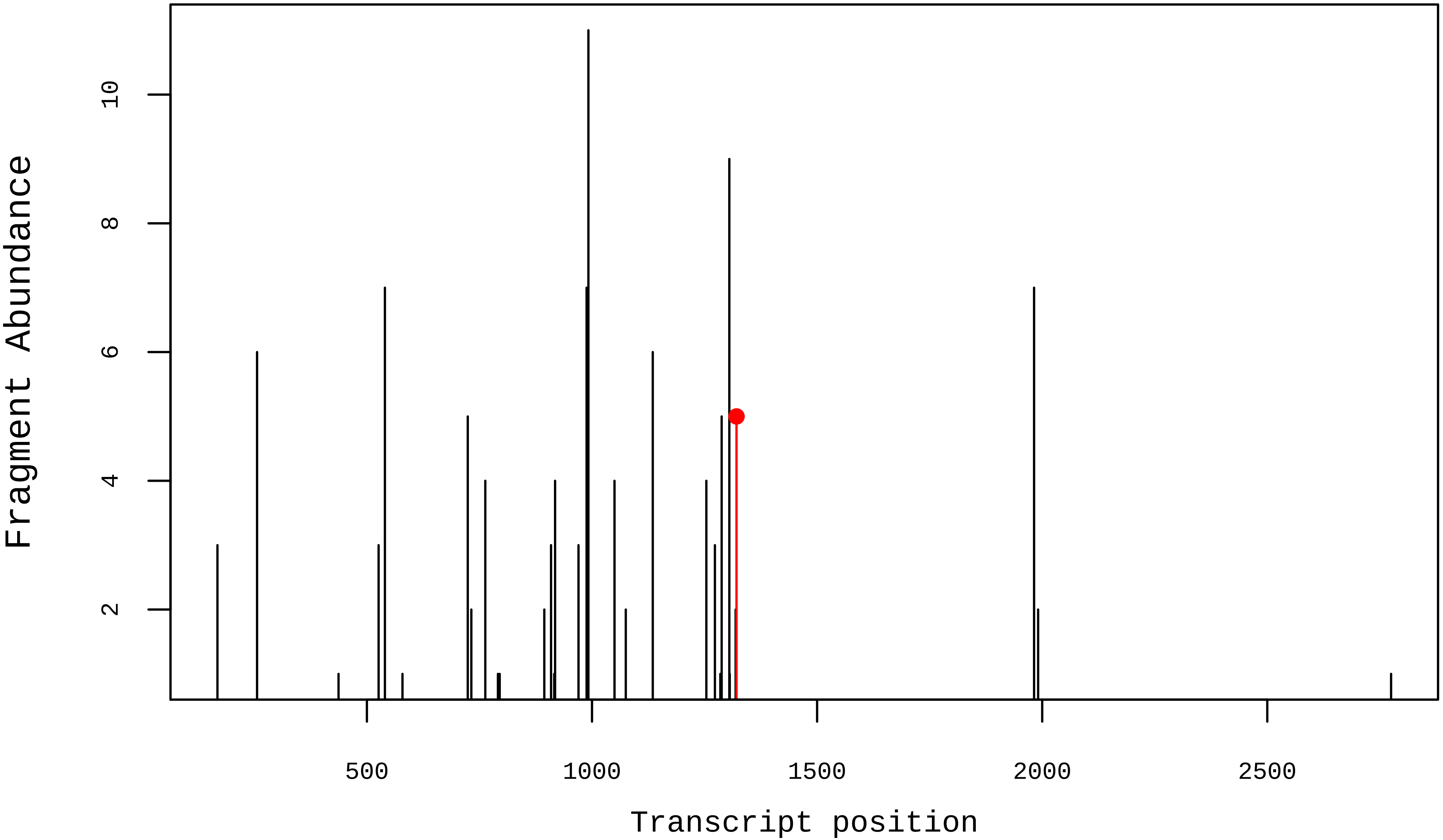

Cleavage site: 1321   Tag abundance: 5   Weighted abundance: 1.667   Category: 2
sRNA abundance: 1   Alignment score: 3.5   MFE ratio: 0.788   p-value: 0.01

HORVU6Hr1G028940|HORVU6Hr1G028940.17||408|2345

```
5' ATCTATTTGTAGTCGGATCCTAGCTTATACGG '3
          ||||o|||o|||| |||||
3'        ACATTAGCTTAGGCTCGAA          '5
```

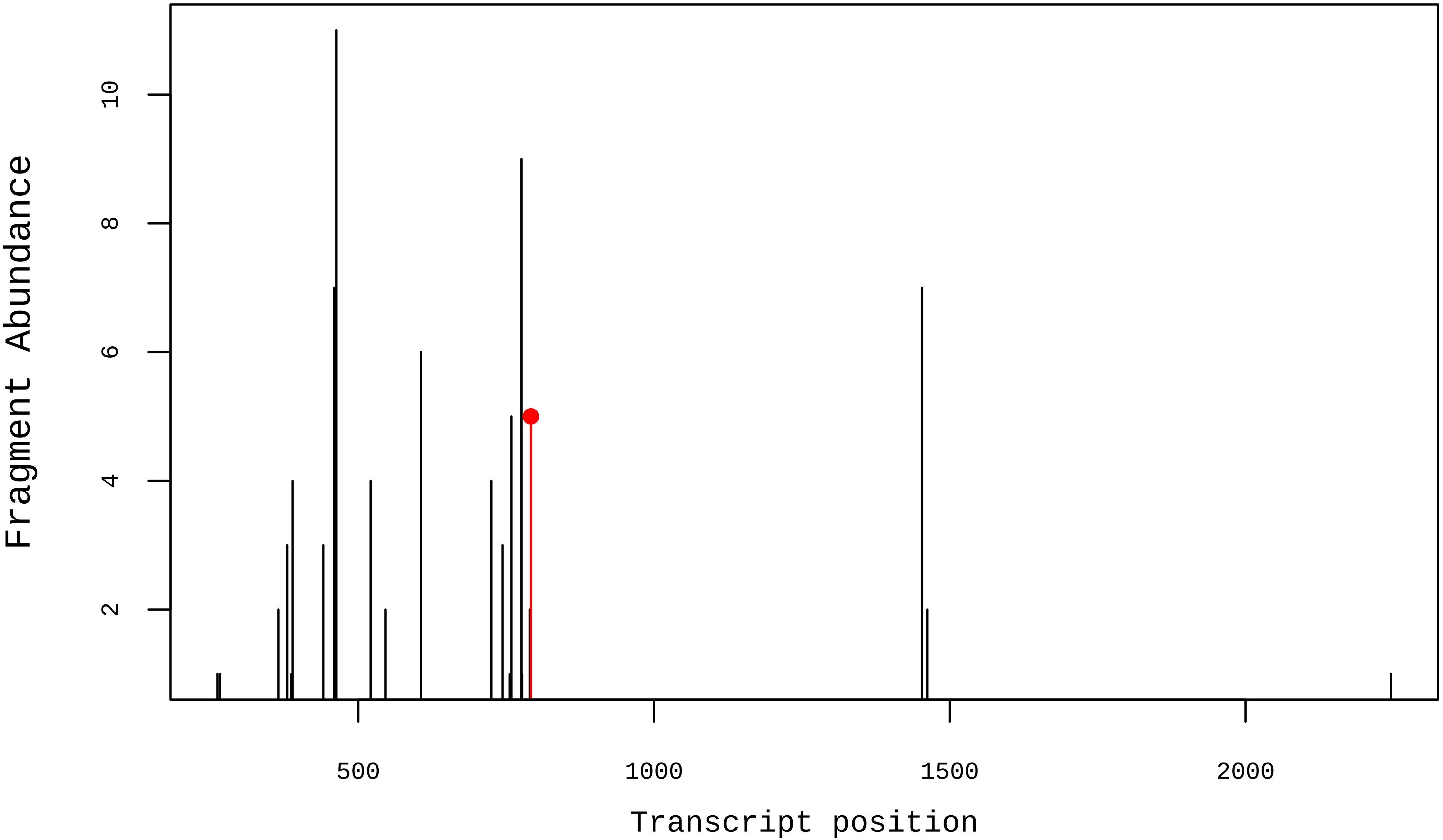

Cleavage site: 792   Tag abundance: 5   Weighted abundance: 1.667   Category: 2
sRNA abundance: 1   Alignment score: 3.5   MFE ratio: 0.788   p-value: 0.009

HORVU6Hr1G028940|HORVU6Hr1G028940.8||585|1720

```
5' ATCTATTTGTAGTCGGATCCTAGCTTATACGG '3
          ||||o|||o|||| |||||
3'        ACATTAGCTTAGGCTCGAA        '5
```

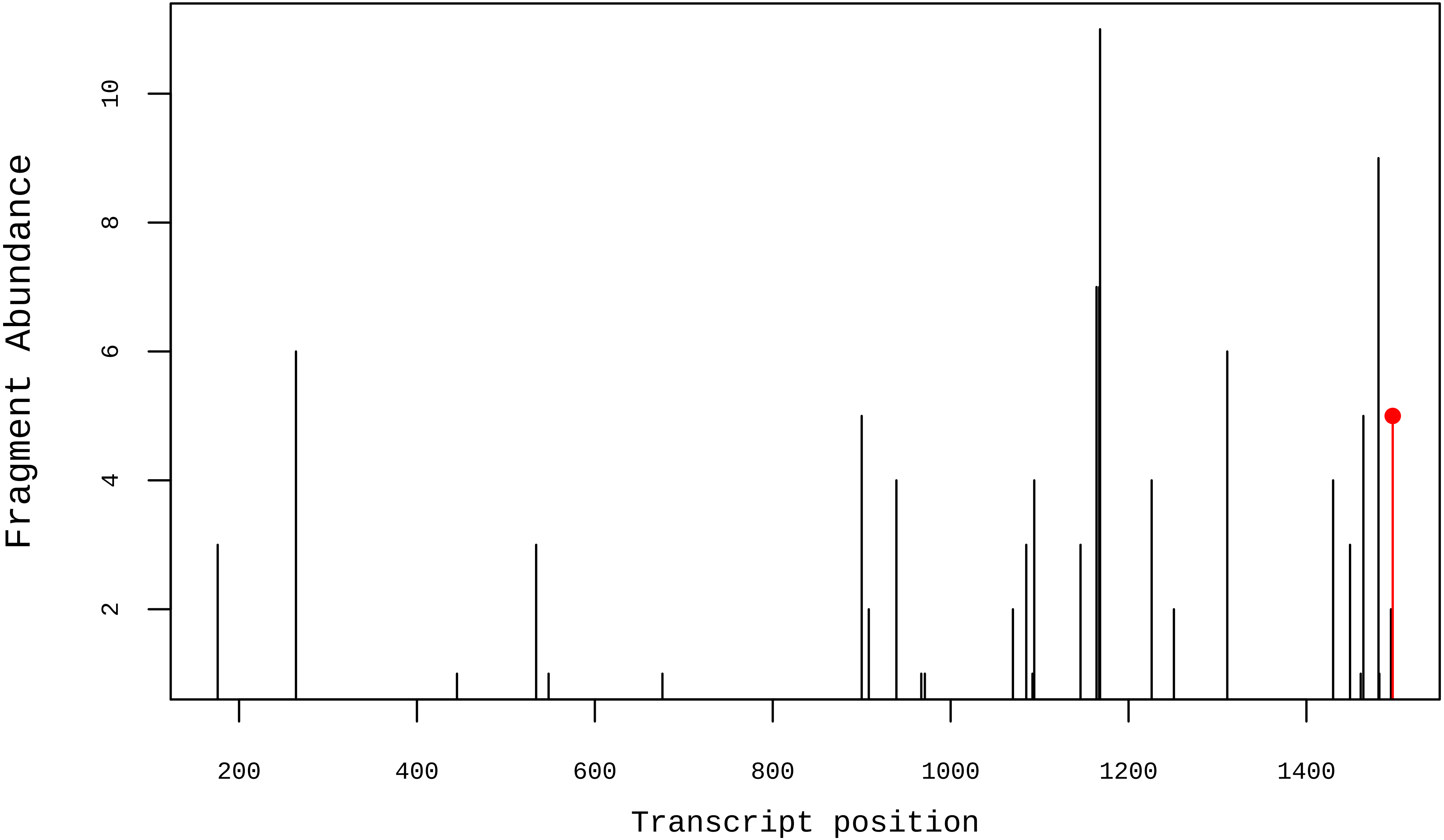

Cleavage site: 1497   Tag abundance: 5   Weighted abundance: 1.667   Category: 2
sRNA abundance: 1   Alignment score: 3.5   MFE ratio: 0.788   p-value: 0.014

HORVU1Hr1G085570|HORVU1Hr1G085570.3||1335|2651

```
5' TTCTTCGGGGCAAATCTCCTTTGGCAAACCTA '3
         |o|||||  ||||||||||||
3'       GTCCCGTCAAGAGGAAACCGT       '5
```

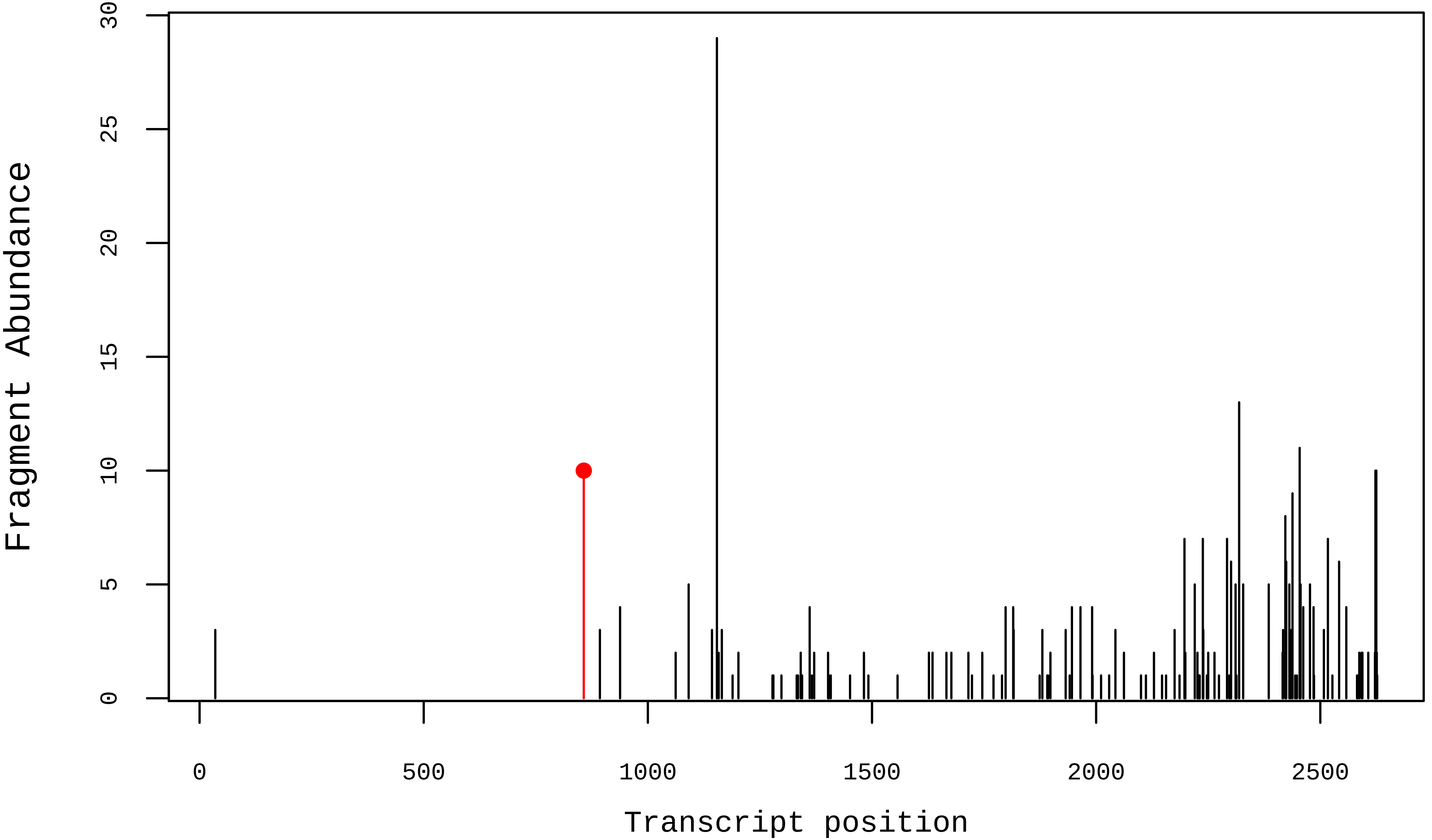

Cleavage site: 857   Tag abundance: 10   Weighted abundance: 2   Category: 2
sRNA abundance: 1   Alignment score: 3.5   MFE ratio: 0.797   p-value: 0.041

HORVU2Hr1G094690|HORVU2Hr1G094690.10||1458|2742

```
5' GGCCAGGTTTGCTGATGTTCATCTAACTAGCC '3
           o|||||||||||| |||||||||
3'         TCAAACGACTACCAGTAGATT        '5
```

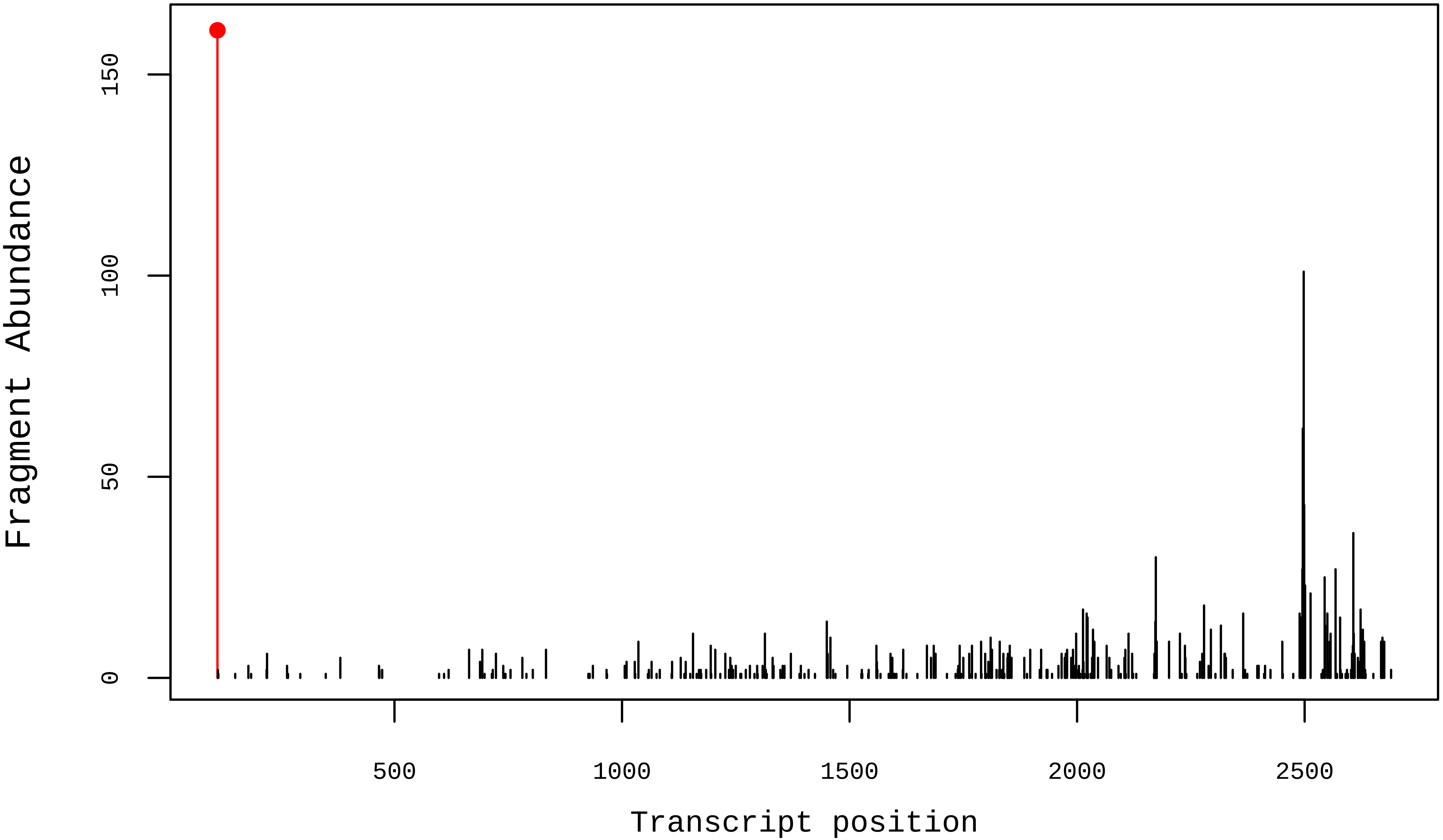

Cleavage site: 111   Tag abundance: 161   Weighted abundance: 9.471   Category: 0
sRNA abundance: 1   Alignment score: 2.5   MFE ratio: 0.804   p-value: 0.017

HORVU2Hr1G094690|HORVU2Hr1G094690.11||1458|2742

```
5' GGCCAGGTTTGCTGATGTTCATCTAACTAGCC '3
        o||||||||||| ||||||||
3'      TCAAACGACTACCAGTAGATT        '5
```

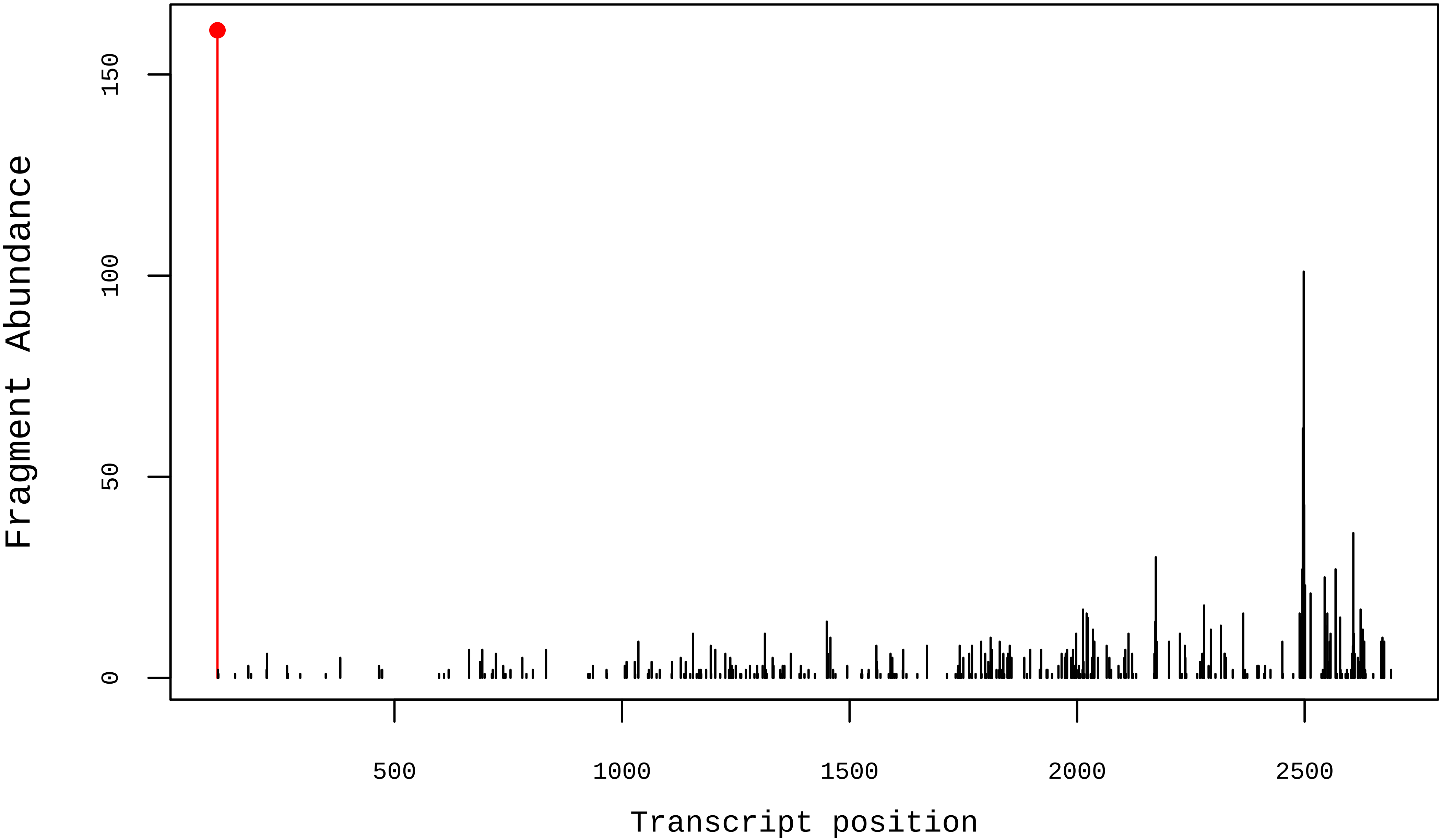

Cleavage site: 111   Tag abundance: 161   Weighted abundance: 9.471   Category: 0
sRNA abundance: 1   Alignment score: 2.5   MFE ratio: 0.804   p-value: 0.017

HORVU2Hr1G094690|HORVU2Hr1G094690.12||2112|2910

```
5' GGCCAGGTTTGCTGATGTTCATCTAACTAGCC '3
        o||||||||||| ||||||||
3'      TCAAACGACTACCAGTAGATT       '5
```

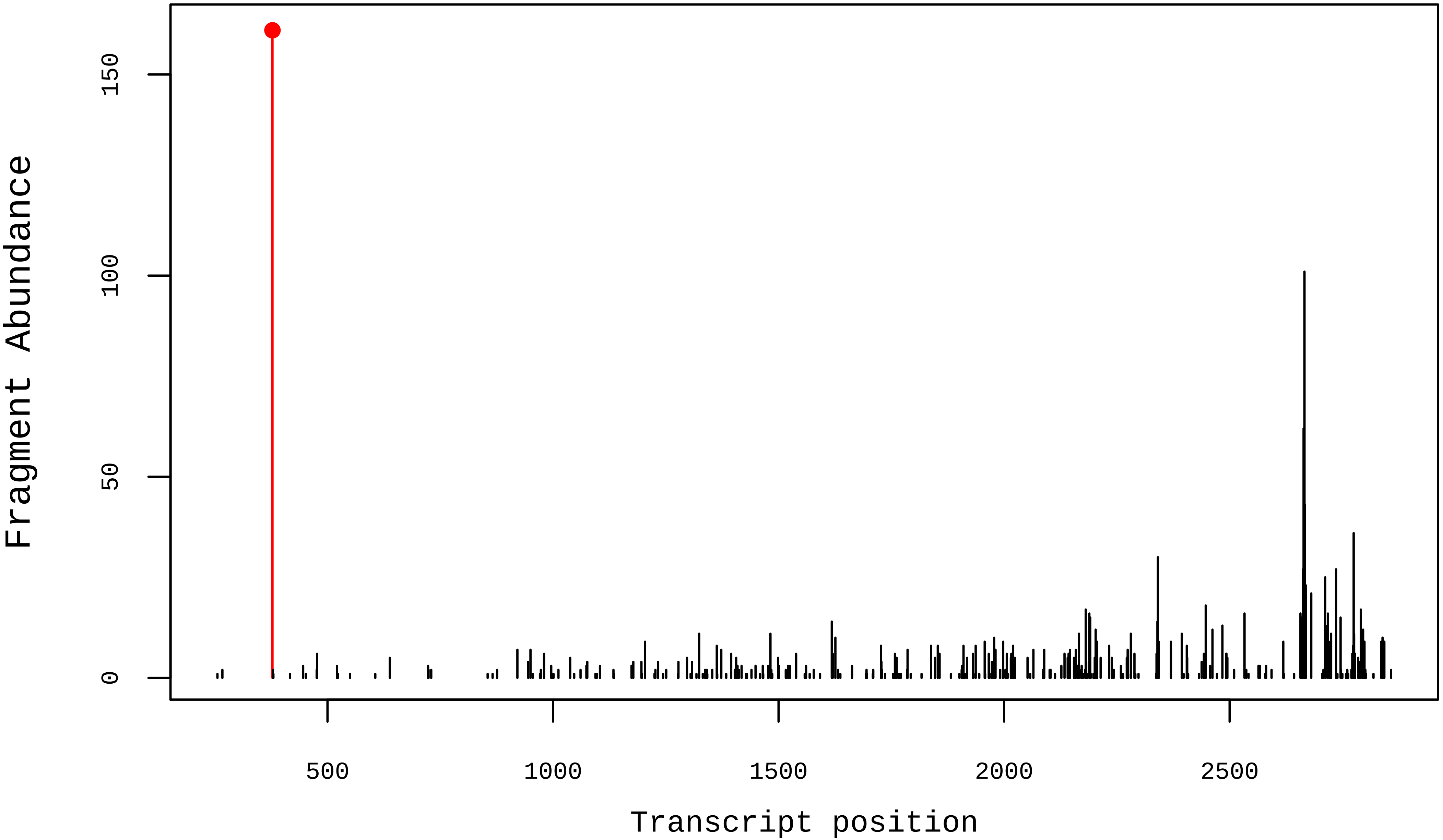

Cleavage site: 378   Tag abundance: 161   Weighted abundance: 9.471   Category: 0
sRNA abundance: 1   Alignment score: 2.5   MFE ratio: 0.804   p-value: 0.016

HORVU2Hr1G094690|HORVU2Hr1G094690.13||2112|2916

```
5' GGCCAGGTTTGCTGATGTTCATCTAACTAGCC '3
         o|||||||||||| |||||||||
3'       TCAAACGACTACCAGTAGATT        '5
```

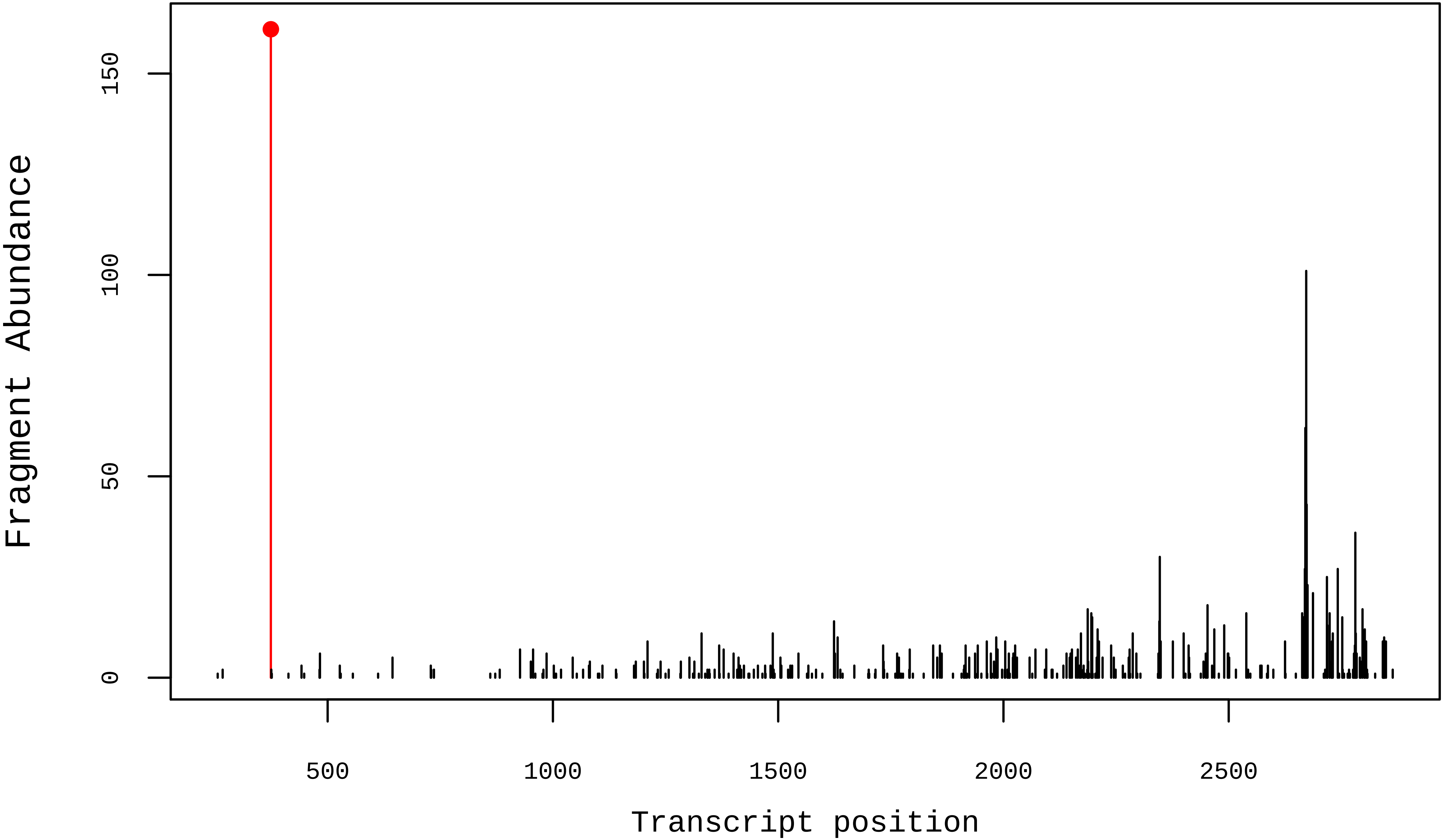

Cleavage site: 374   Tag abundance: 161   Weighted abundance: 9.471   Category: 0
sRNA abundance: 1   Alignment score: 2.5   MFE ratio: 0.804   p-value: 0.016

HORVU2Hr1G094690|HORVU2Hr1G094690.14||2112|2920

```
5' GGCCAGGTTTGCTGATGTTCATCTAACTAGCC '3
         o||||||||||| ||||||||
3'       TCAAACGACTACCAGTAGATT       '5
```

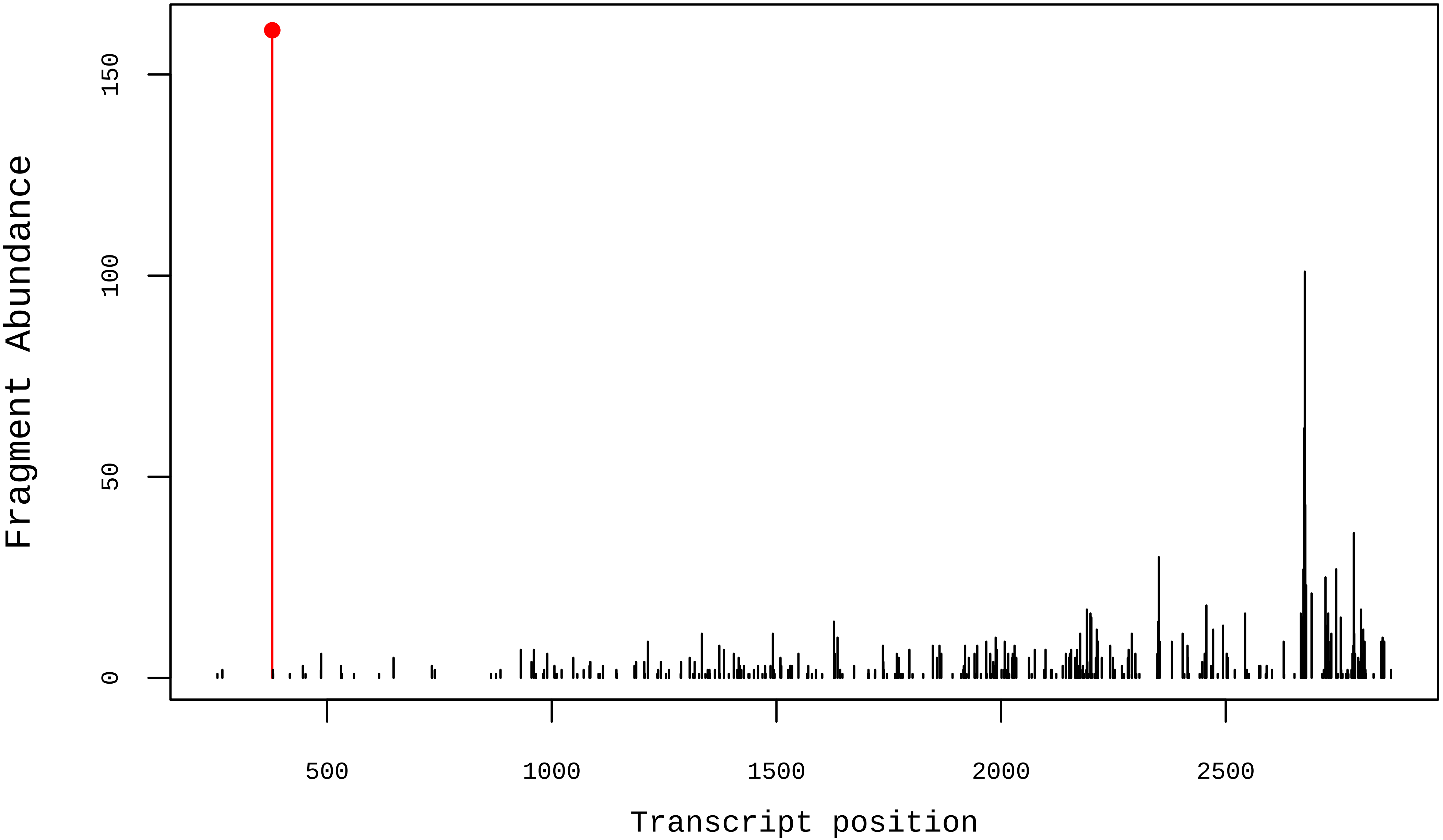

Cleavage site: 378   Tag abundance: 161   Weighted abundance: 9.471   Category: 0
sRNA abundance: 1   Alignment score: 2.5   MFE ratio: 0.804   p-value: 0.016

HORVU2Hr1G094690|HORVU2Hr1G094690.15||2112|2920

```
5' GGCCAGGTTTGCTGATGTTCATCTAACTAGCC '3
           o|||||||||||| |||||||||
3'         TCAAACGACTACCAGTAGATT       '5
```

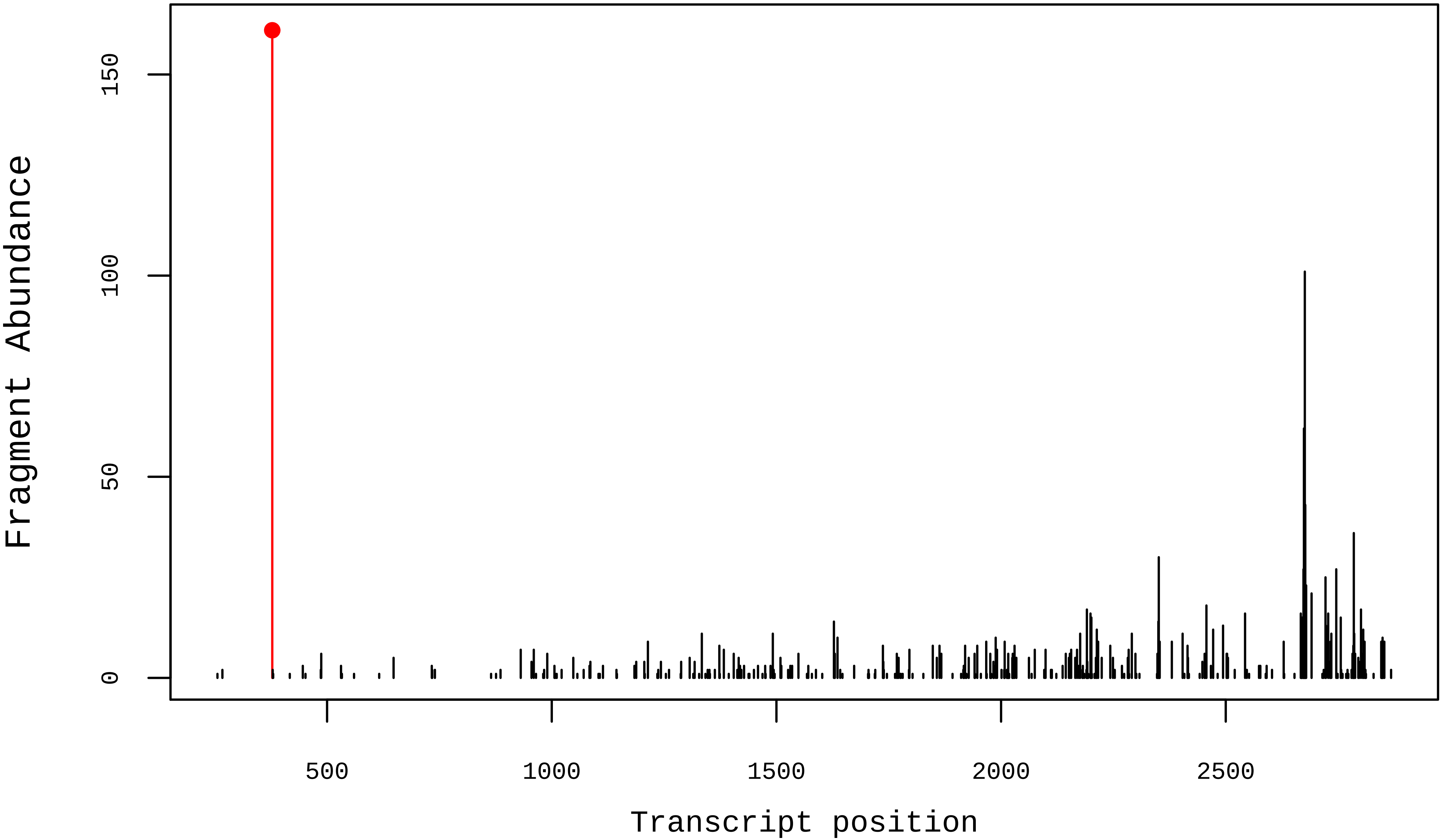

Cleavage site: 378   Tag abundance: 161   Weighted abundance: 9.471   Category: 0
sRNA abundance: 1   Alignment score: 2.5   MFE ratio: 0.804   p-value: 0.016

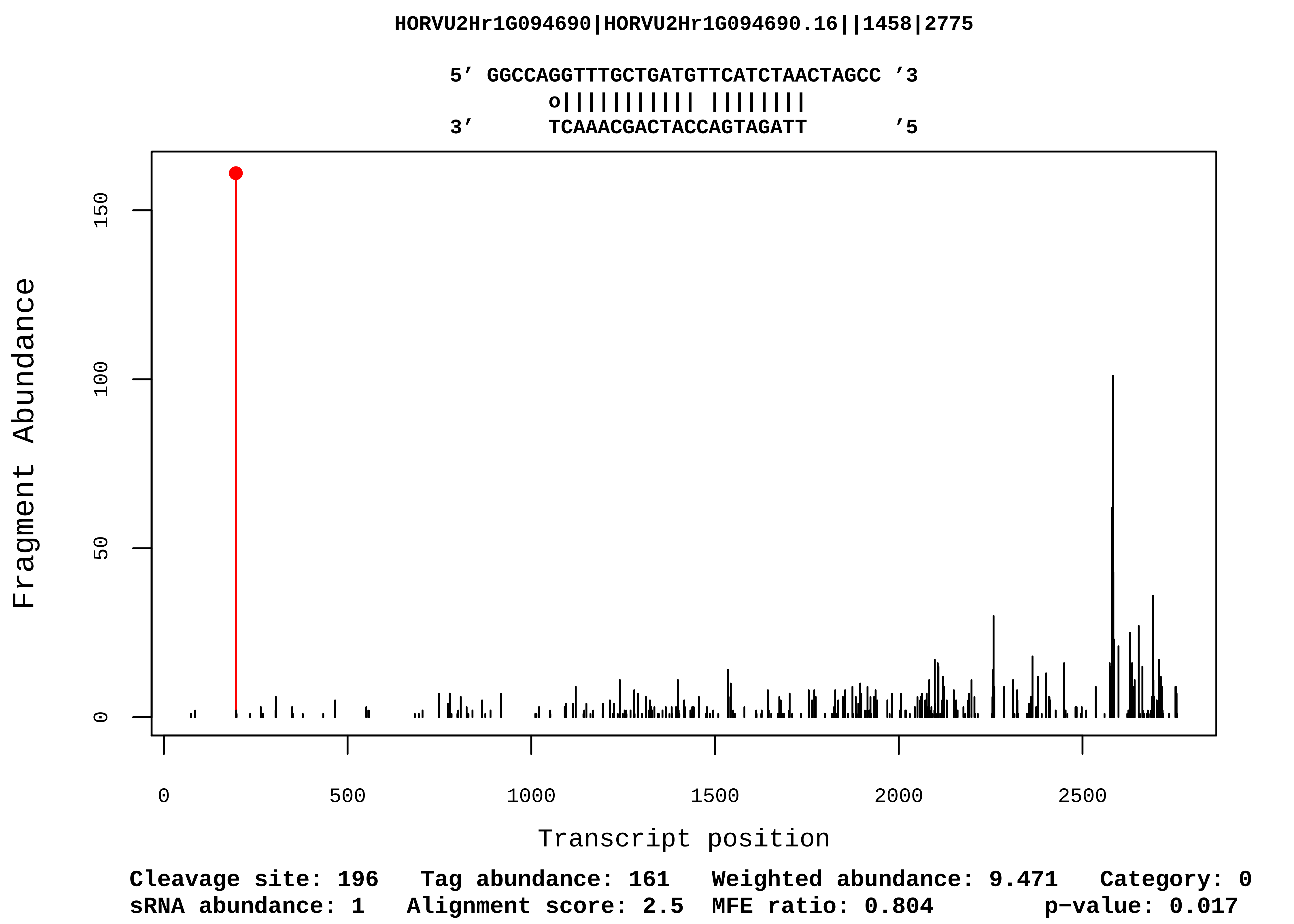
HORVU2Hr1G094690|HORVU2Hr1G094690.16||1458|2775
5′ GGCCAGGTTTGCTGATGTTCATCTAACTAGCC ′3
o||||||||||| ||||||||
3′ TCAAACGACTACCAGTAGATT ′5
Fragment Abundance
0
50
100
150
0
500
1000
1500
2000
2500
Transcript position
Cleavage site: 196
Tag abundance: 161
Weighted abundance: 9.471
Category: 0
sRNA abundance: 1
Alignment score: 2.5
MFE ratio: 0.804
p-value: 0.017

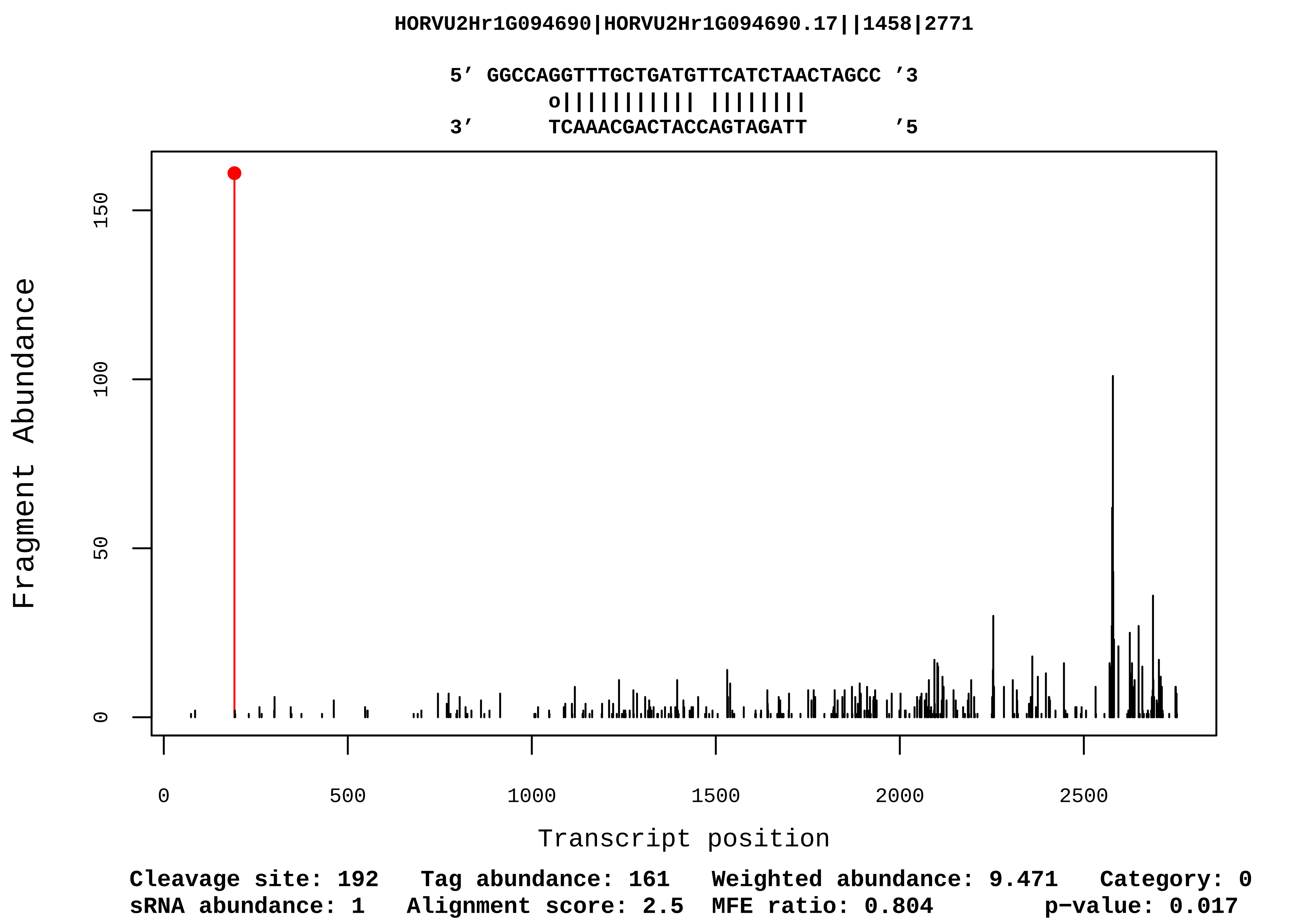
HORVU2Hr1G094690|HORVU2Hr1G094690.17||1458|2771
5' GGCCAGGTTTGCTGATGTTCATCTAACTAGCC '3
o||||||||||| ||||||||
3' TCAAACGACTACCAGTAGATT '5
Fragment Abundance
0
50
100
150
0
500
1000
1500
2000
2500
Transcript position
Cleavage site: 192 Tag abundance: 161 Weighted abundance: 9.471 Category: 0
sRNA abundance: 1 Alignment score: 2.5 MFE ratio: 0.804 p-value: 0.017

HORVU2Hr1G094690|HORVU2Hr1G094690.18||1458|2775

```
5' GGCCAGGTTTGCTGATGTTCATCTAACTAGCC '3
         o|||||||||||| |||||||||
3'       TCAAACGACTACCAGTAGATT       '5
```

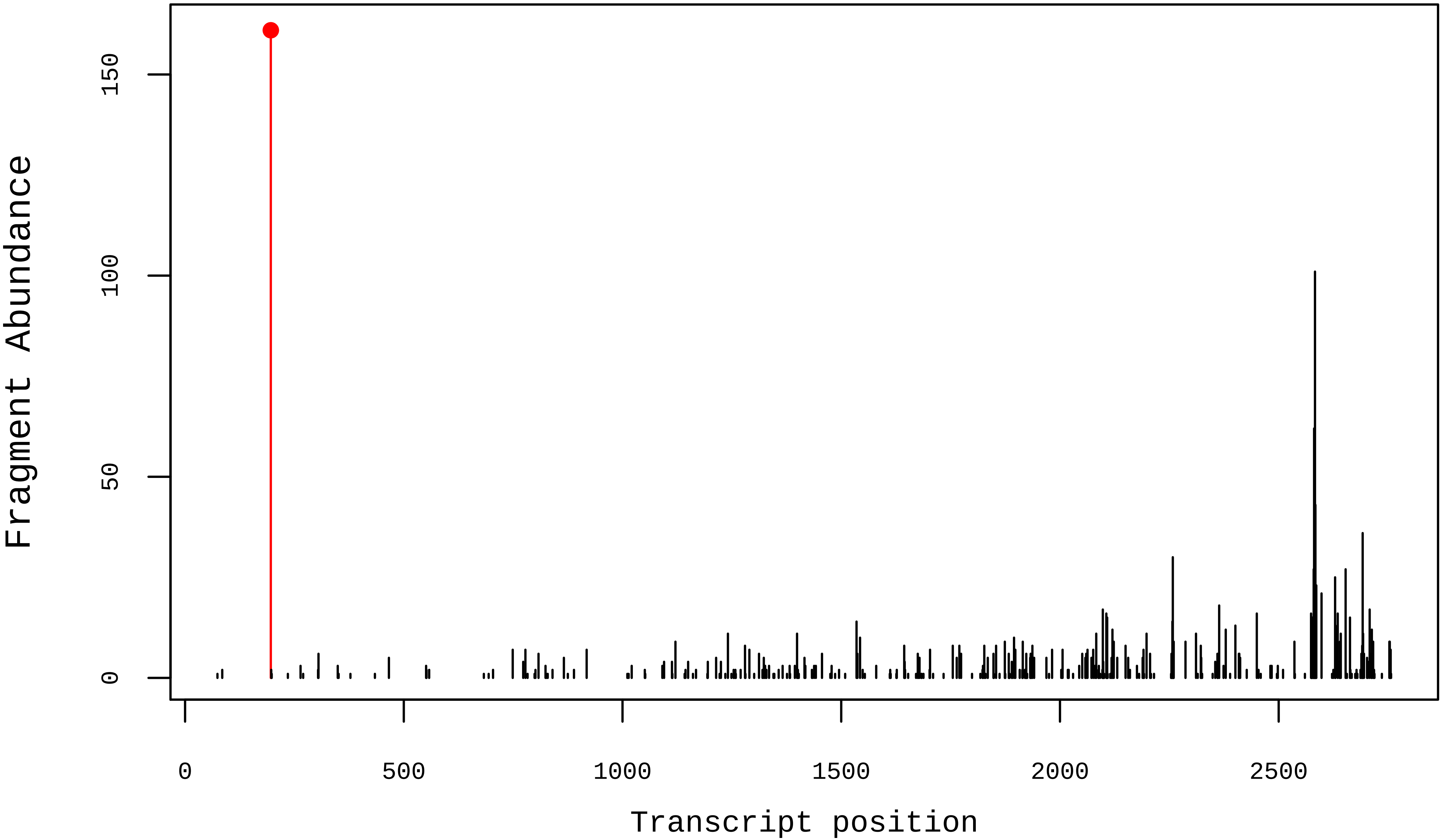

Cleavage site: 196 Tag abundance: 161 Weighted abundance: 9.471 Category: 0
sRNA abundance: 1 Alignment score: 2.5 MFE ratio: 0.804 p-value: 0.017

HORVU2Hr1G094690|HORVU2Hr1G094690.19||1458|2630

```
5' GGCCAGGTTTGCTGATGTTCATCTAACTAGCC '3
          o||||||||||| ||||||||
3'        TCAAACGACTACCAGTAGATT        '5
```

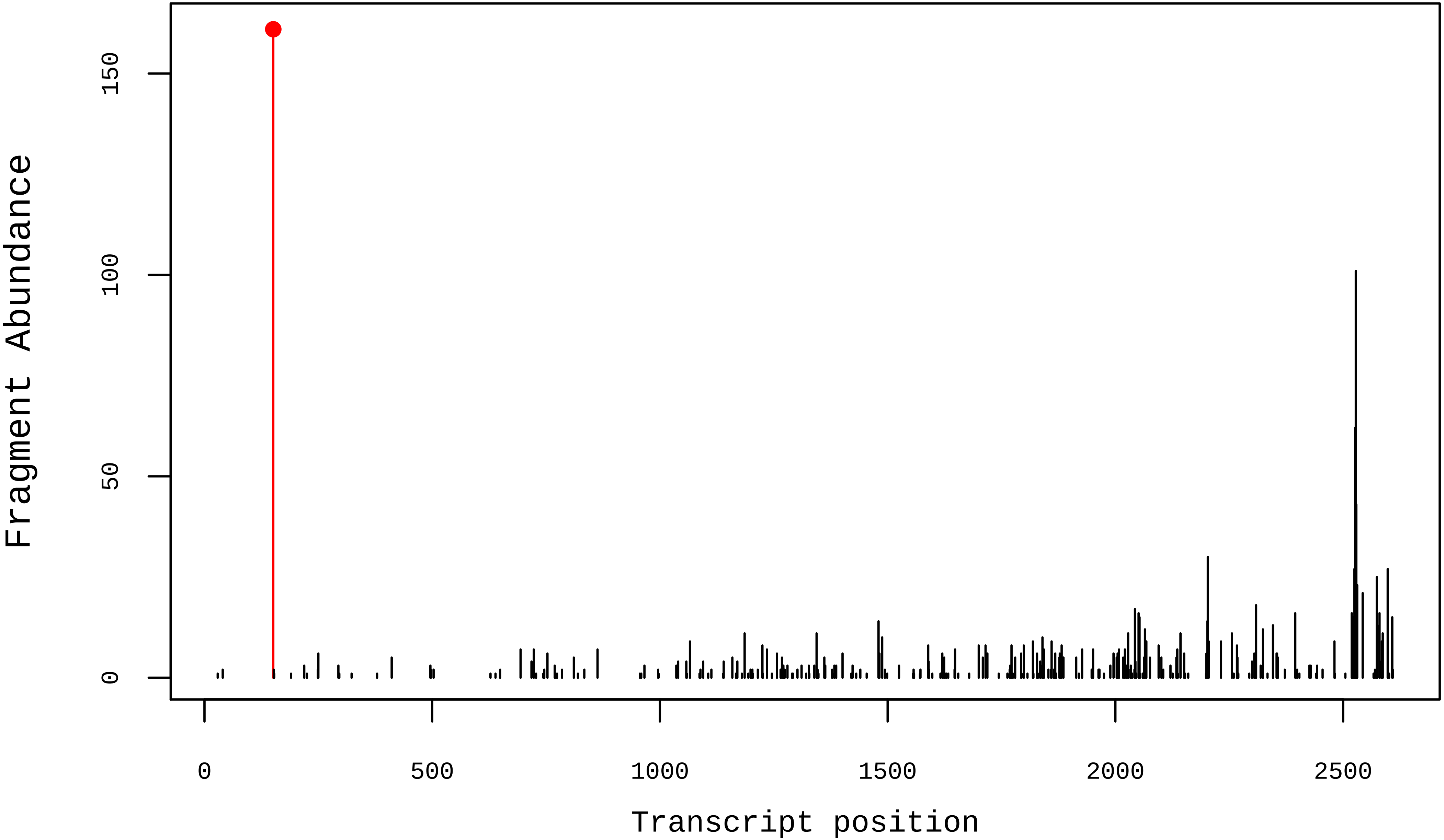

Cleavage site: 151   Tag abundance: 161   Weighted abundance: 9.471   Category: 0
sRNA abundance: 1   Alignment score: 2.5   MFE ratio: 0.804   p-value: 0.017

HORVU2Hr1G094690|HORVU2Hr1G094690.1||2112|2767

```
5' GGCCAGGTTTGCTGATGTTCATCTAACTAGCC '3
          o||||||||||| ||||||||
3'        TCAAACGACTACCAGTAGATT        '5
```

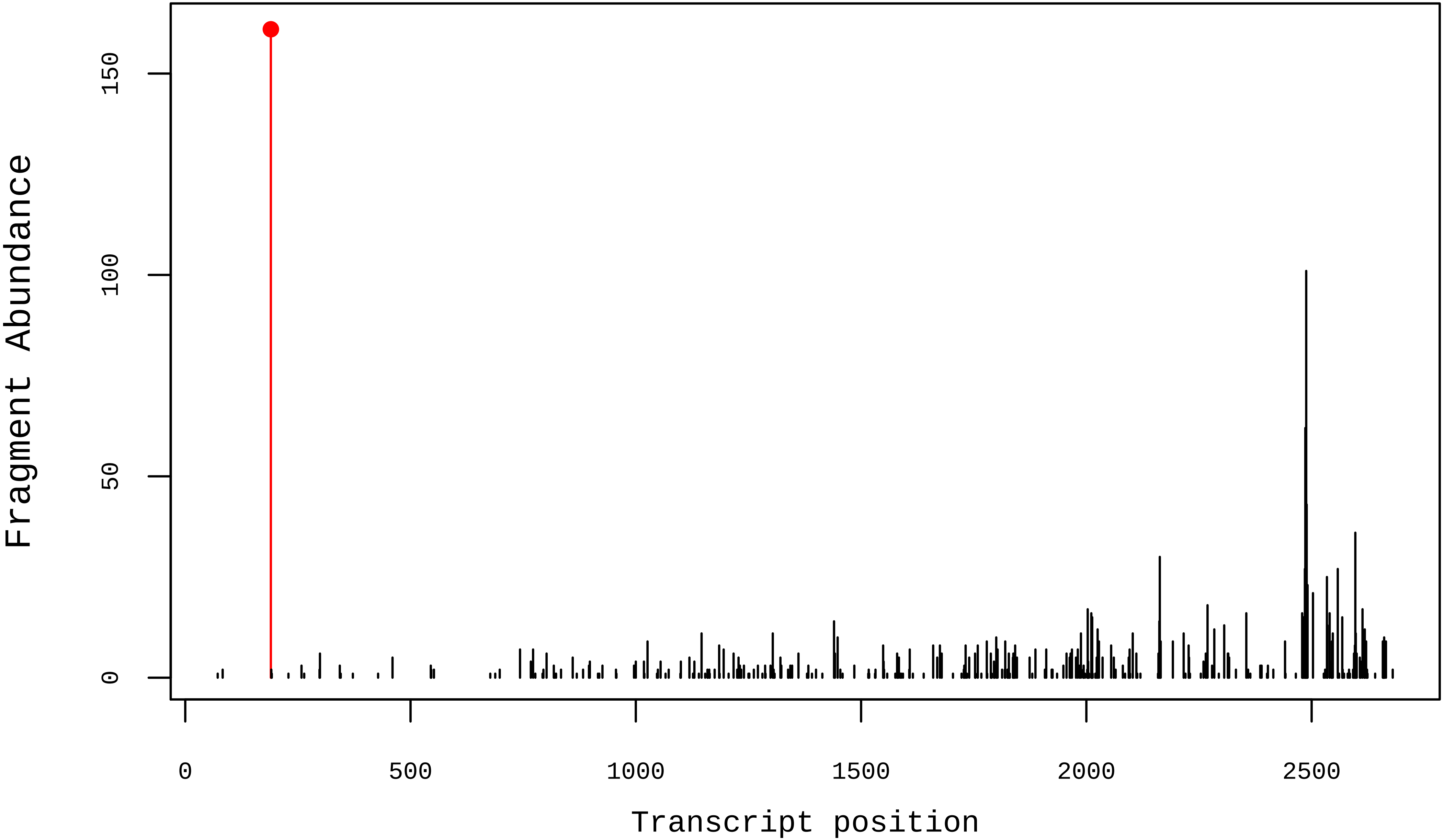

Cleavage site: 190   Tag abundance: 161   Weighted abundance: 9.471   Category: 0
sRNA abundance: 1   Alignment score: 2.5   MFE ratio: 0.804   p-value: 0.017

HORVU2Hr1G094690|HORVU2Hr1G094690.2||2112|2761

```
5' GGCCAGGTTTGCTGATGTTCATCTAACTAGCC '3
         o||||||||||| ||||||||
3'       TCAAACGACTACCAGTAGATT       '5
```

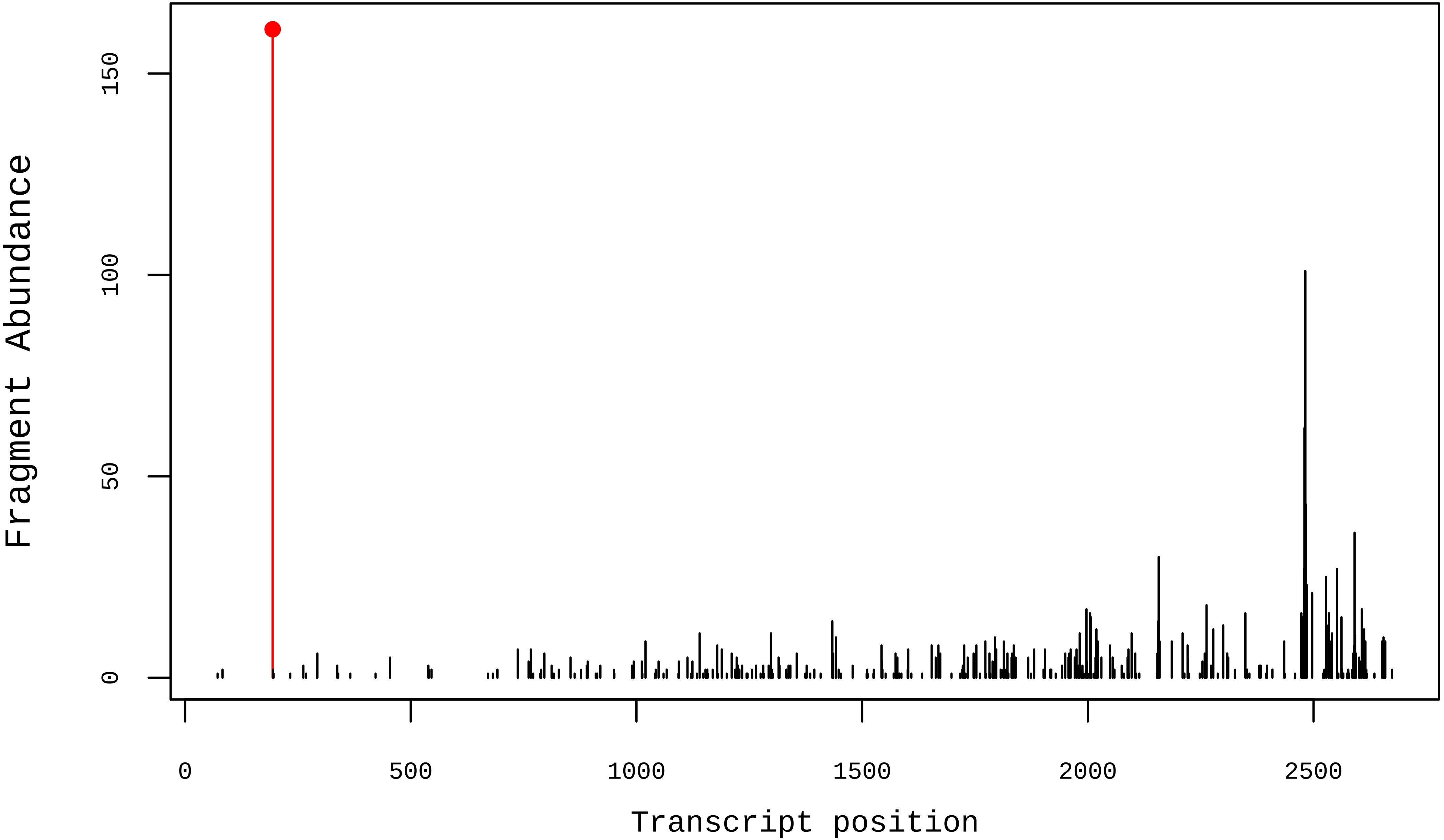

Cleavage site: 194 Tag abundance: 161 Weighted abundance: 9.471 Category: 0
sRNA abundance: 1 Alignment score: 2.5 MFE ratio: 0.804 p-value: 0.017

HORVU2Hr1G094690|HORVU2Hr1G094690.3||2112|2734

```
5' GGCCAGGTTTGCTGATGTTCATCTAACTAGCC '3
         o||||||||||| ||||||||
3'       TCAAACGACTACCAGTAGATT       '5
```

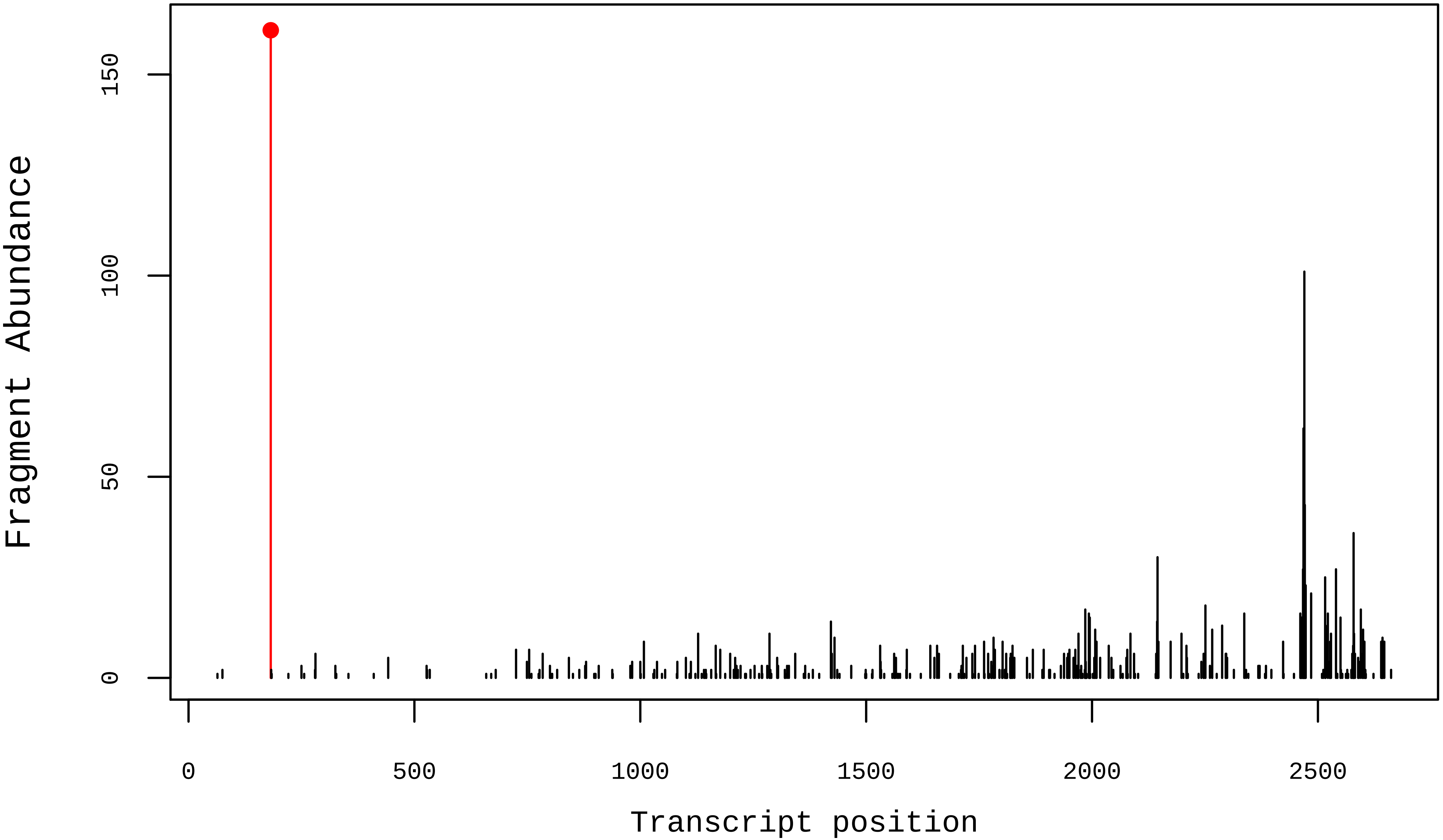

Cleavage site: 182   Tag abundance: 161   Weighted abundance: 9.471   Category: 0
sRNA abundance: 1   Alignment score: 2.5   MFE ratio: 0.804   p-value: 0.017

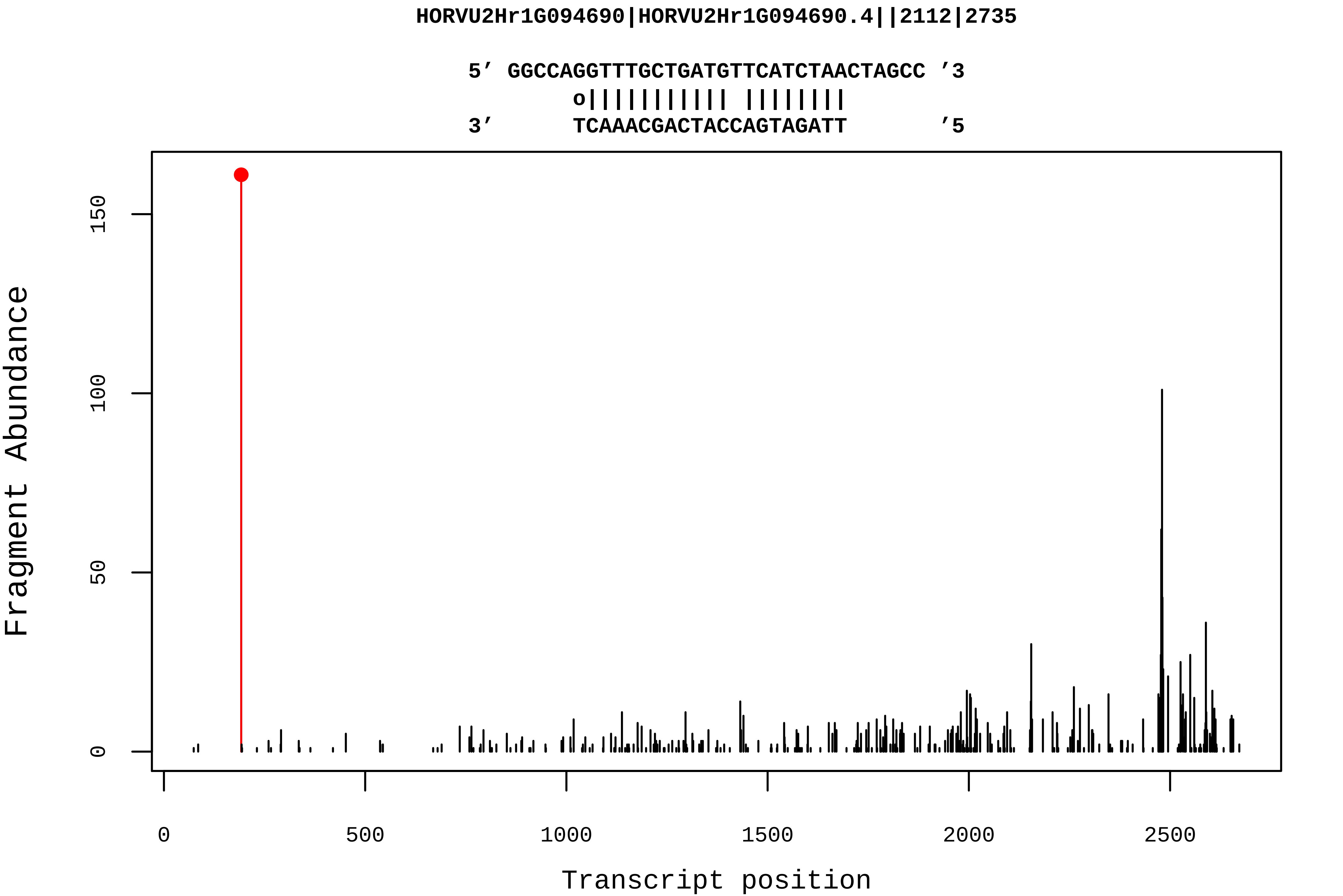

Cleavage site: 192 Tag abundance: 161 Weighted abundance: 9.471 Category: 0
sRNA abundance: 1 Alignment score: 2.5 MFE ratio: 0.804 p-value: 0.017

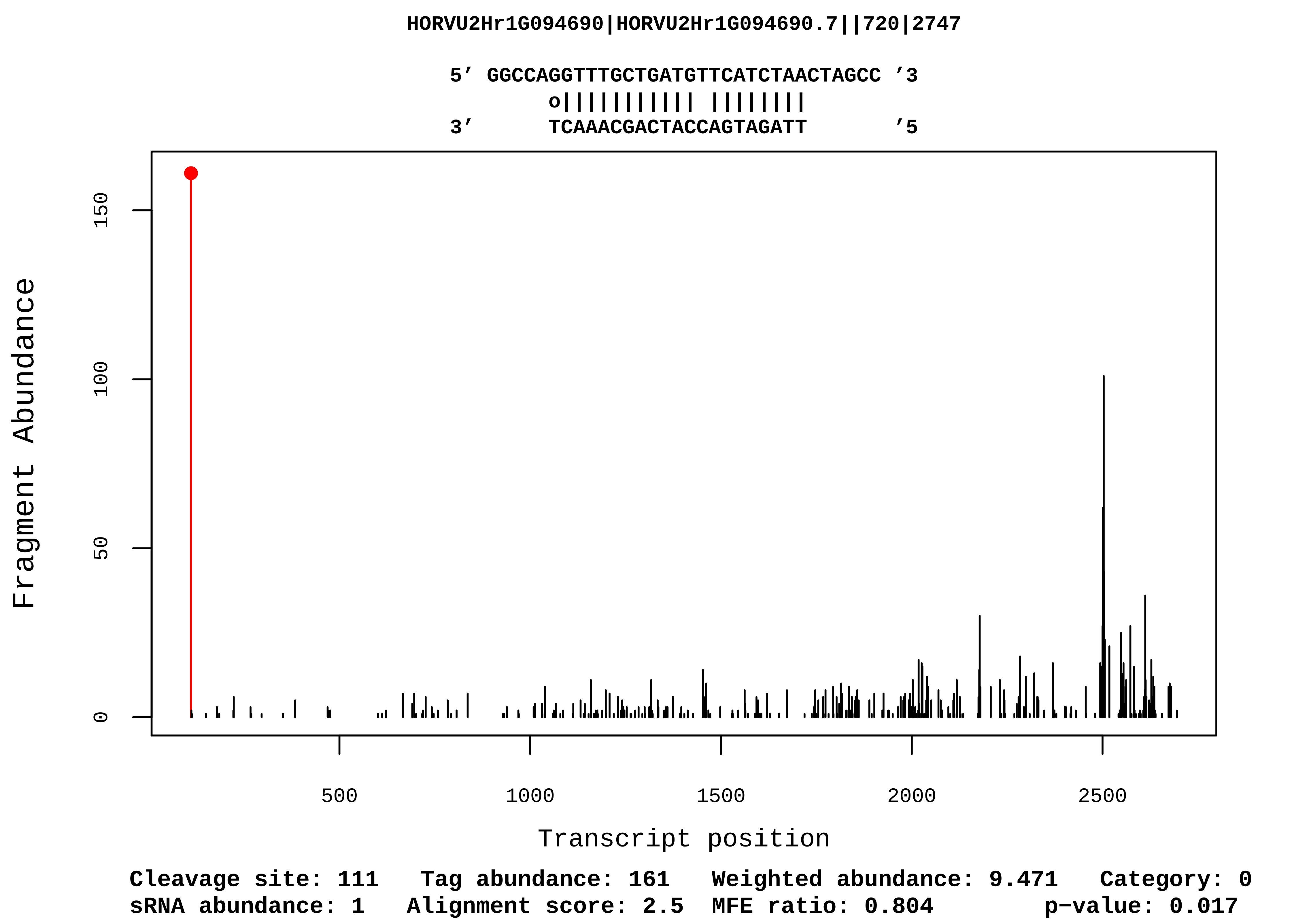
HORVU2Hr1G094690|HORVU2Hr1G094690.7||720|2747
5' GGCCAGGTTTGCTGATGTTCATCTAACTAGCC '3
o||||||||||| ||||||||
3' TCAAACGACTACCAGTAGATT '5
Fragment Abundance
0
50
100
150
500
1000
1500
2000
2500
Transcript position
Cleavage site: 111 Tag abundance: 161 Weighted abundance: 9.471 Category: 0
sRNA abundance: 1 Alignment score: 2.5 MFE ratio: 0.804 p-value: 0.017

HORVU2Hr1G094690|HORVU2Hr1G094690.8||1458|2744

```
5’ GGCCAGGTTTGCTGATGTTCATCTAACTAGCC ’3
         o||||||||||| ||||||||
3’       TCAAACGACTACCAGTAGATT       ’5
```

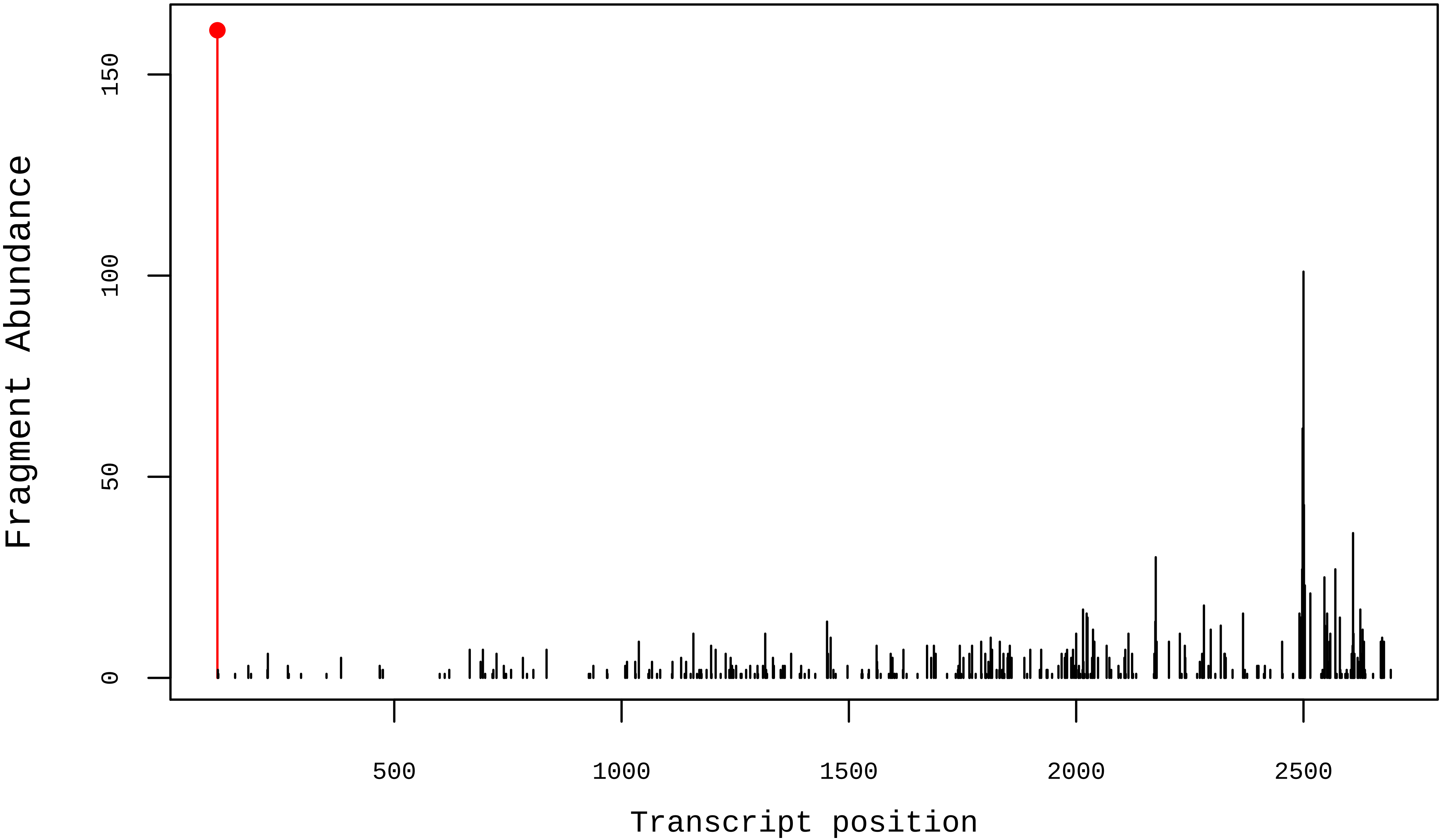

Cleavage site: 111   Tag abundance: 161   Weighted abundance: 9.471   Category: 0
sRNA abundance: 1   Alignment score: 2.5   MFE ratio: 0.804   p-value: 0.017

HORVU2Hr1G094690|HORVU2Hr1G094690.9||1227|2744

```
5’ GGCCAGGTTTGCTGATGTTCATCTAACTAGCC ’3
         o|||||||||| ||||||||
3’       TCAAACGACTACCAGTAGATT       ’5
```

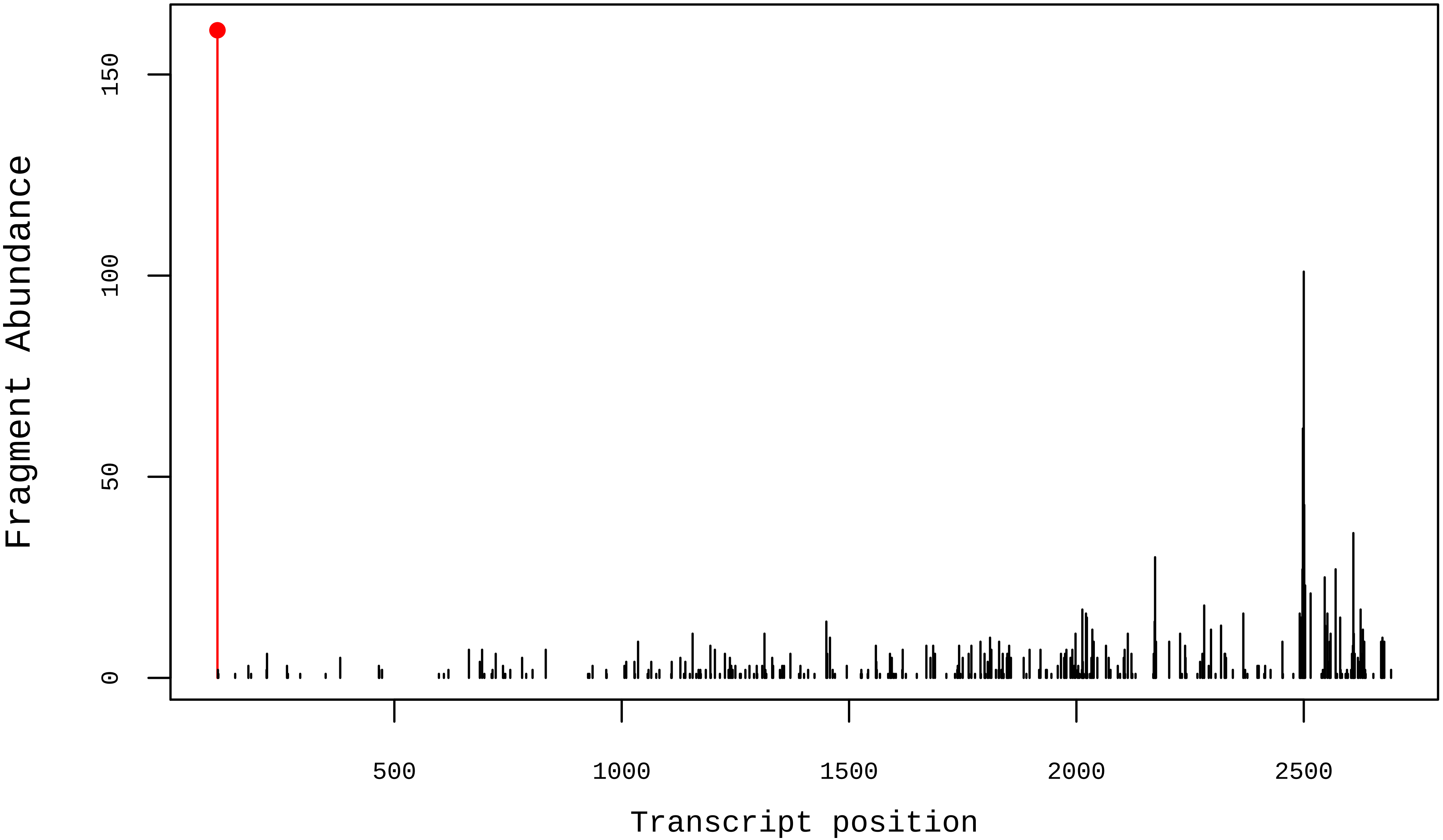

Cleavage site: 111   Tag abundance: 161   Weighted abundance: 9.471   Category: 0
sRNA abundance: 1   Alignment score: 2.5   MFE ratio: 0.804   p-value: 0.017

HORVU1Hr1G093760|HORVU1Hr1G093760.10||1521|1988

```
5’ GCCTGCGCGT-CAGCAGCACCGACGAATCTGAG ’3
              | ||||||||||||| |
3’            ACGTCGTCGTGGCTGATA       ’5
```

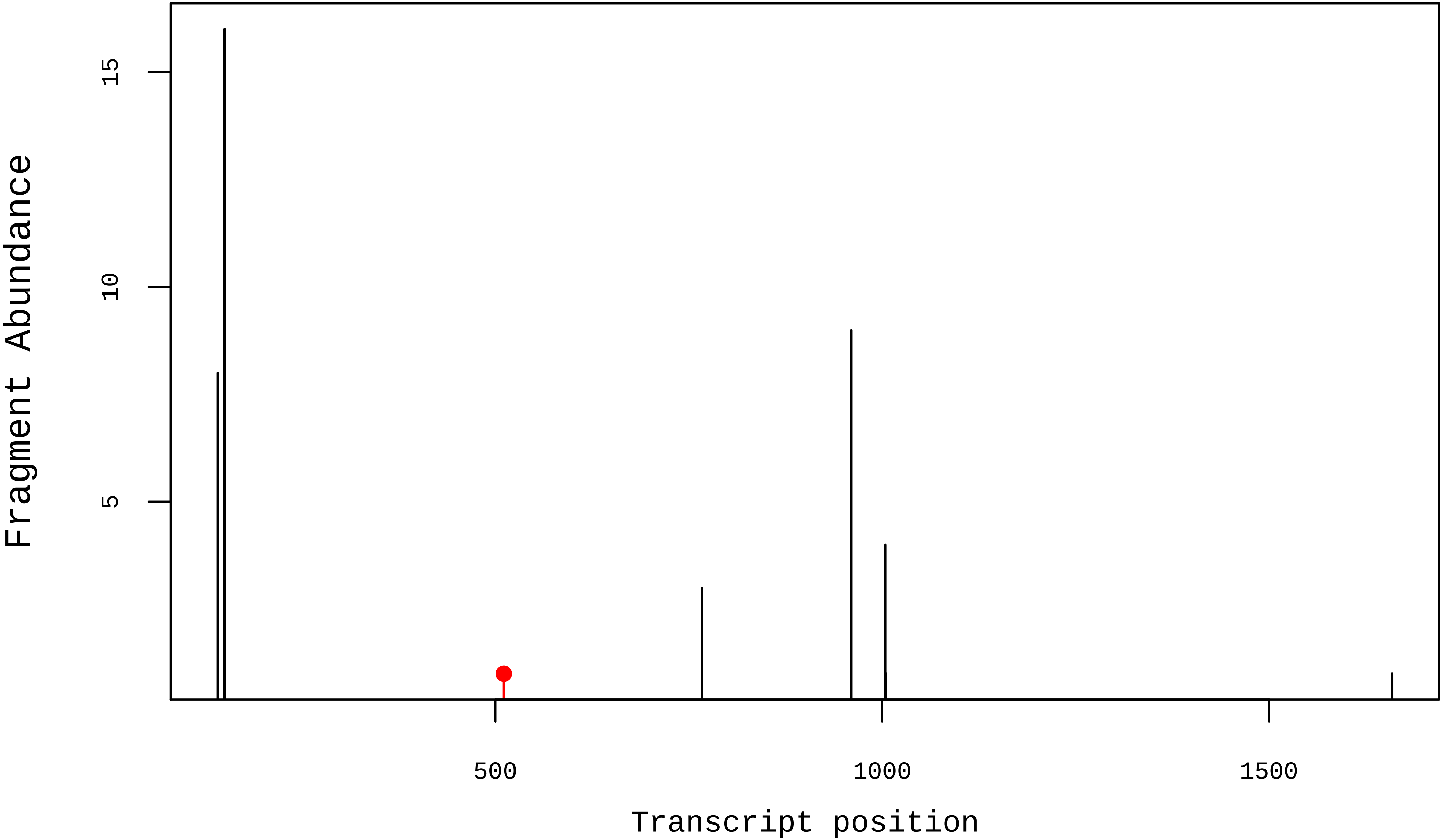

Cleavage site: 511   Tag abundance: 1   Weighted abundance: 0.25   Category: 4
sRNA abundance: 1   Alignment score: 4   MFE ratio: 0.795   p-value: 0.018

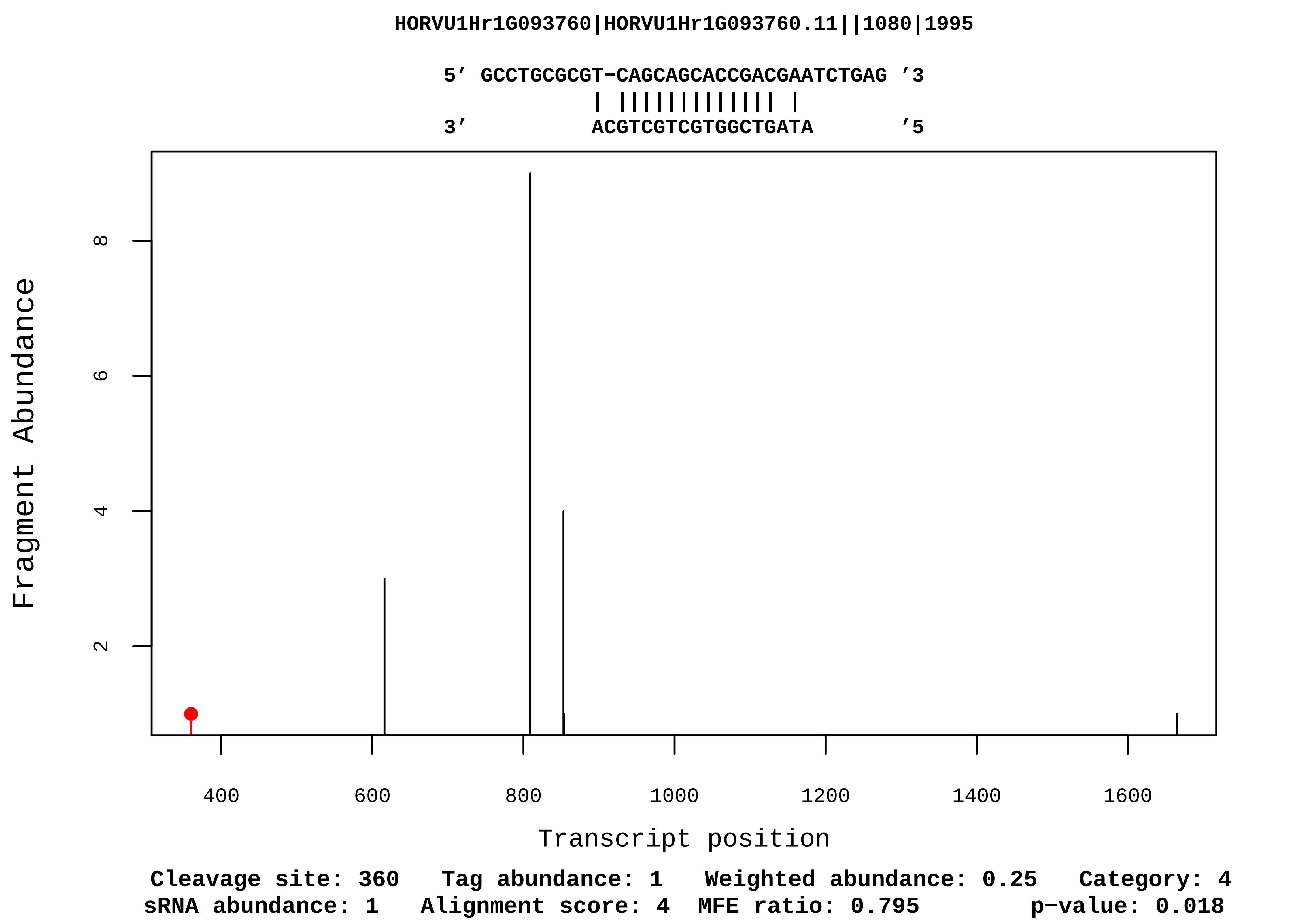
HORVU1Hr1G093760|HORVU1Hr1G093760.11||1080|1995
5′ GCCTGCGCGT−CAGCAGCACCGACGAATCTGAG ′3
| ||||||||||||| |
3′ ACGTCGTCGTGGCTGATA ′5
Fragment Abundance
2
4
6
8
400
600
800
1000
1200
1400
1600
Transcript position
Cleavage site: 360 Tag abundance: 1 Weighted abundance: 0.25 Category: 4
sRNA abundance: 1 Alignment score: 4 MFE ratio: 0.795 p−value: 0.018

HORVU1Hr1G093760|HORVU1Hr1G093760.12||1383|1383

```
5' GCCTGCGCGT-CAGCAGCACCGACGAATCTGAG '3
              | ||||||||||||| |
3'            ACGTCGTCGTGGCTGATA       '5
```

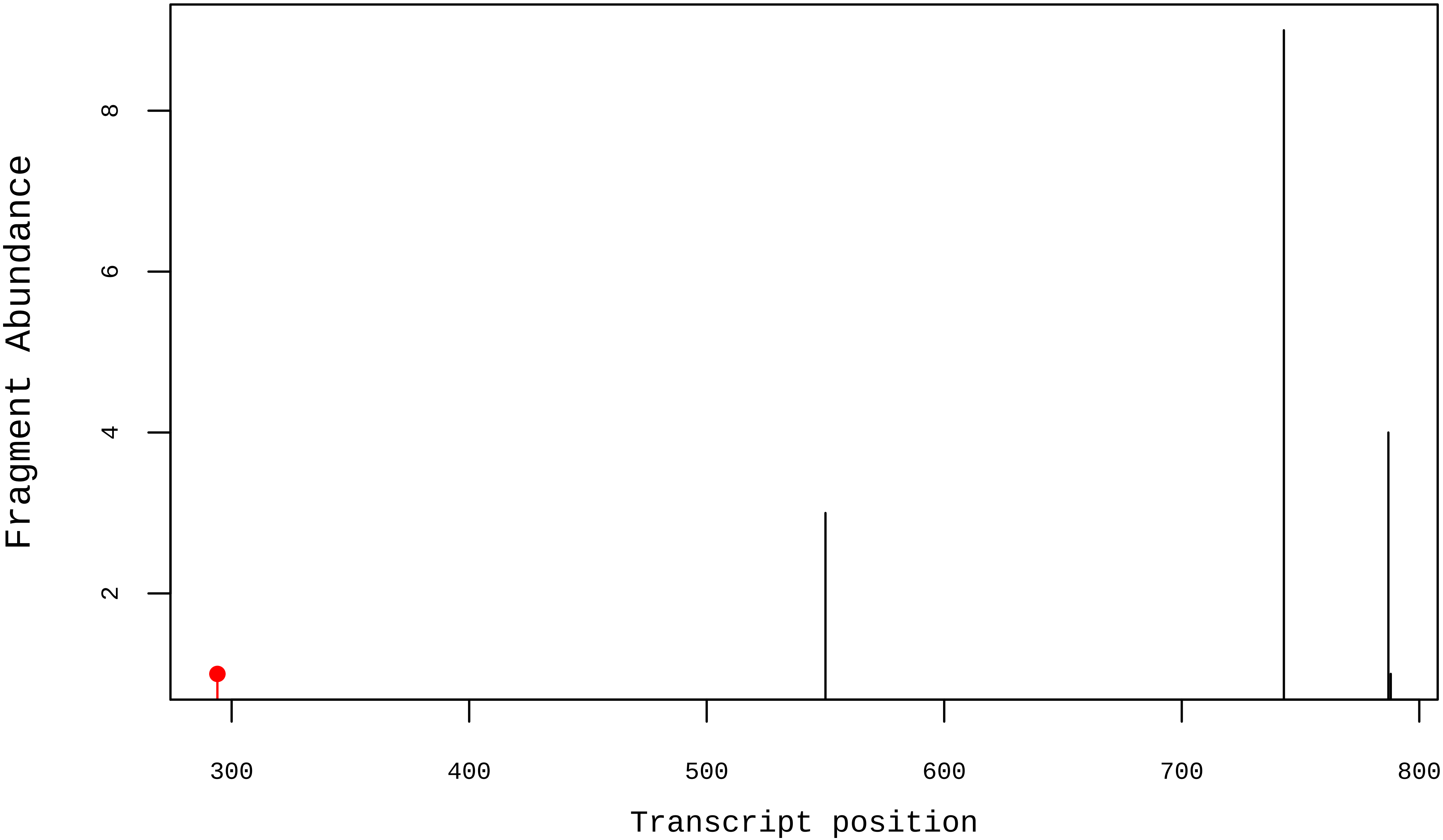

Cleavage site: 294   Tag abundance: 1   Weighted abundance: 0.25   Category: 4
sRNA abundance: 1   Alignment score: 4   MFE ratio: 0.795   p-value: 0.017

HORVU1Hr1G093760|HORVU1Hr1G093760.13||1455|1470

```
5’ GCCTGCGCGT-CAGCAGCACCGACGAATCTGAG ’3
            | ||||||||||||| |
3’          ACGTCGTCGTGGCTGATA        ’5
```

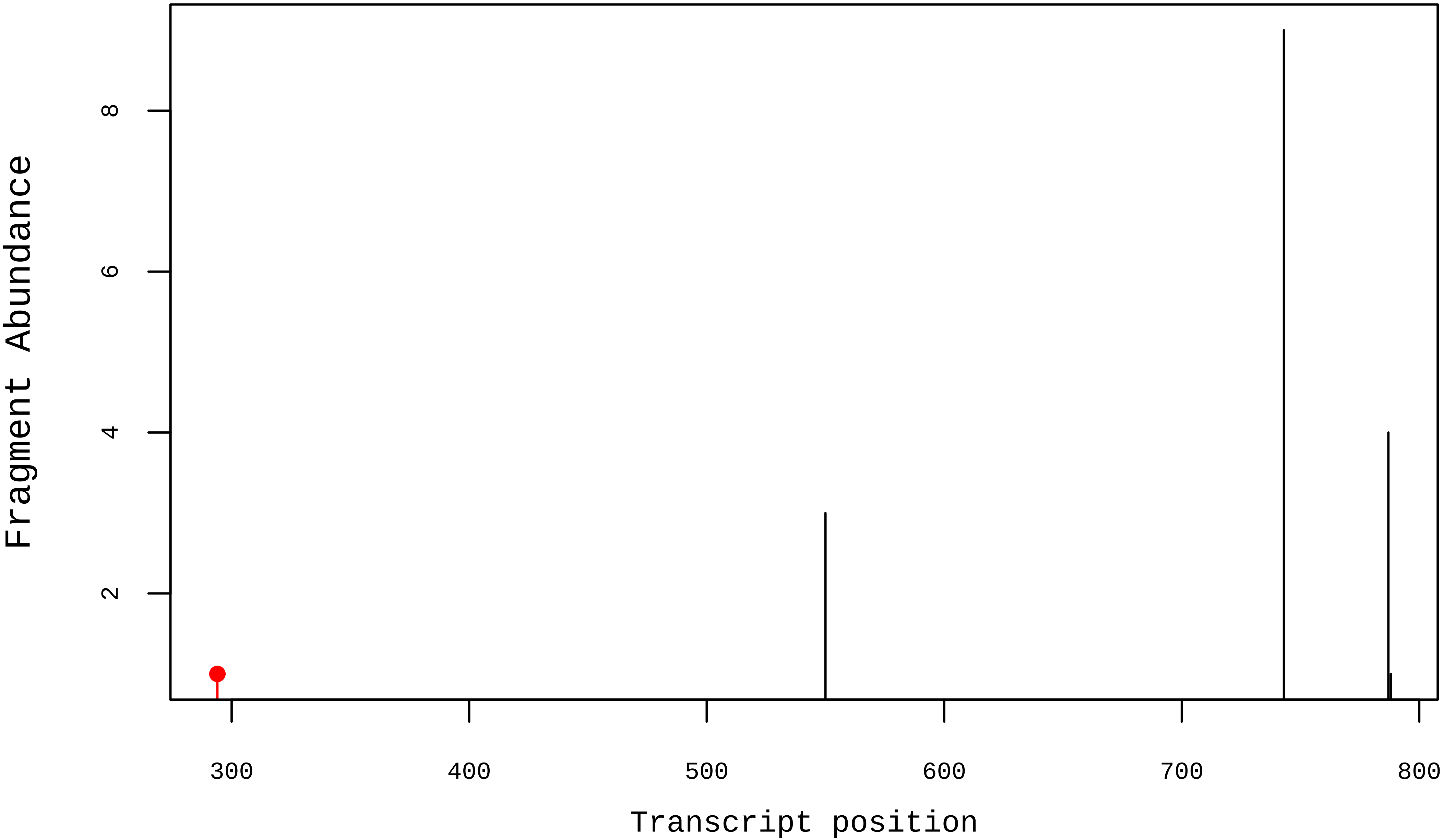

Cleavage site: 294   Tag abundance: 1   Weighted abundance: 0.25   Category: 4
sRNA abundance: 1   Alignment score: 4   MFE ratio: 0.795   p-value: 0.016

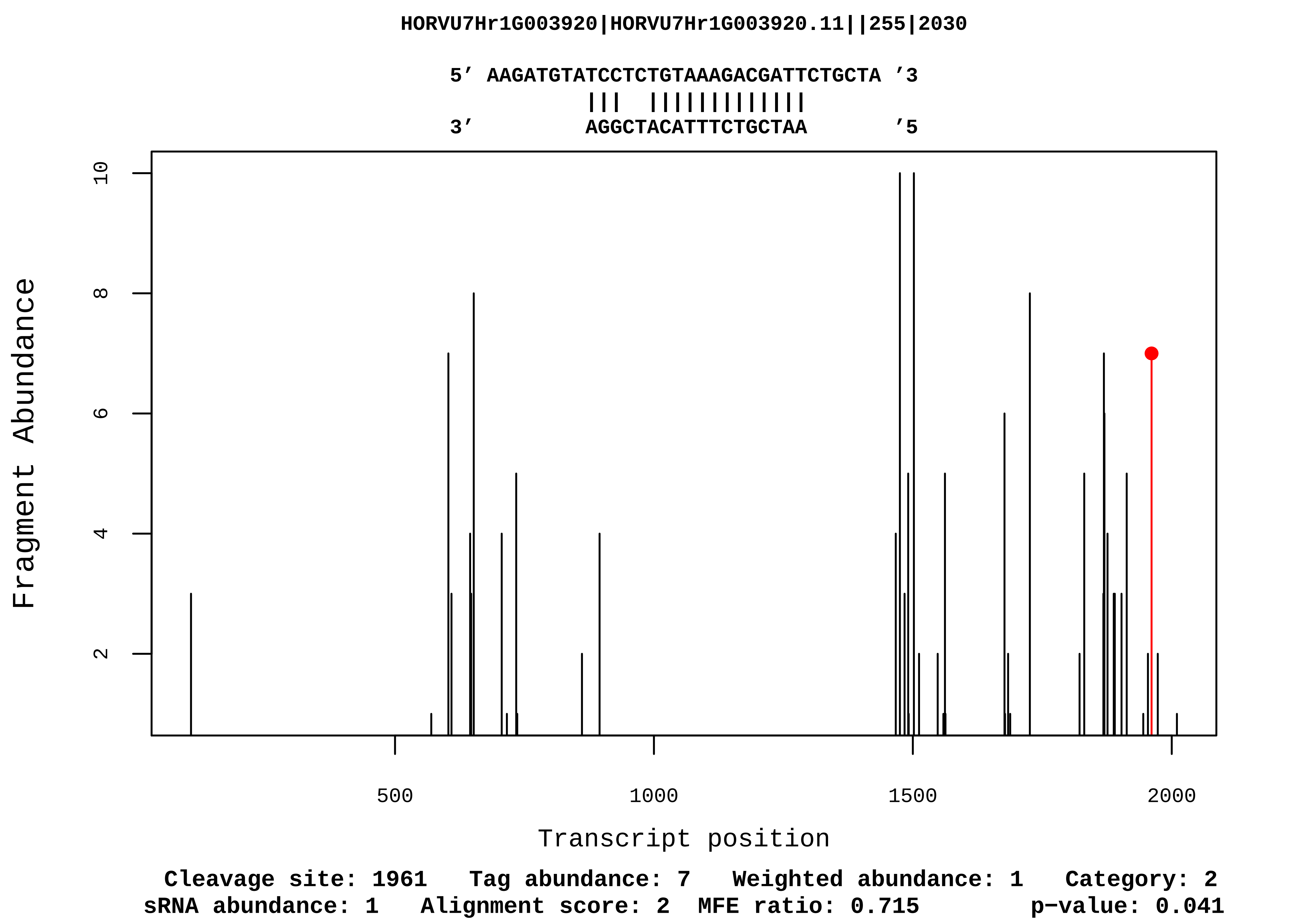
HORVU7Hr1G003920|HORVU7Hr1G003920.11||255|2030
5′ AAGATGTATCCTCTGTAAAGACGATTCTGCTA ′3
||| |||||||||||||
3′ AGGCTACATTTCTGCTAA ′5
Fragment Abundance
2
4
6
8
10
500
1000
1500
2000
Transcript position
Cleavage site: 1961 Tag abundance: 7 Weighted abundance: 1 Category: 2
sRNA abundance: 1 Alignment score: 2 MFE ratio: 0.715 p-value: 0.041

HORVU1Hr1G093760|HORVU1Hr1G093760.10||1521|1988

```
5' GCCTGCGCGT-CAGCAGCACCGACGAATCTGAG '3
           || ||||||||||||| |
3'         CACGTCGTCGTGGCTGATA       '5
```

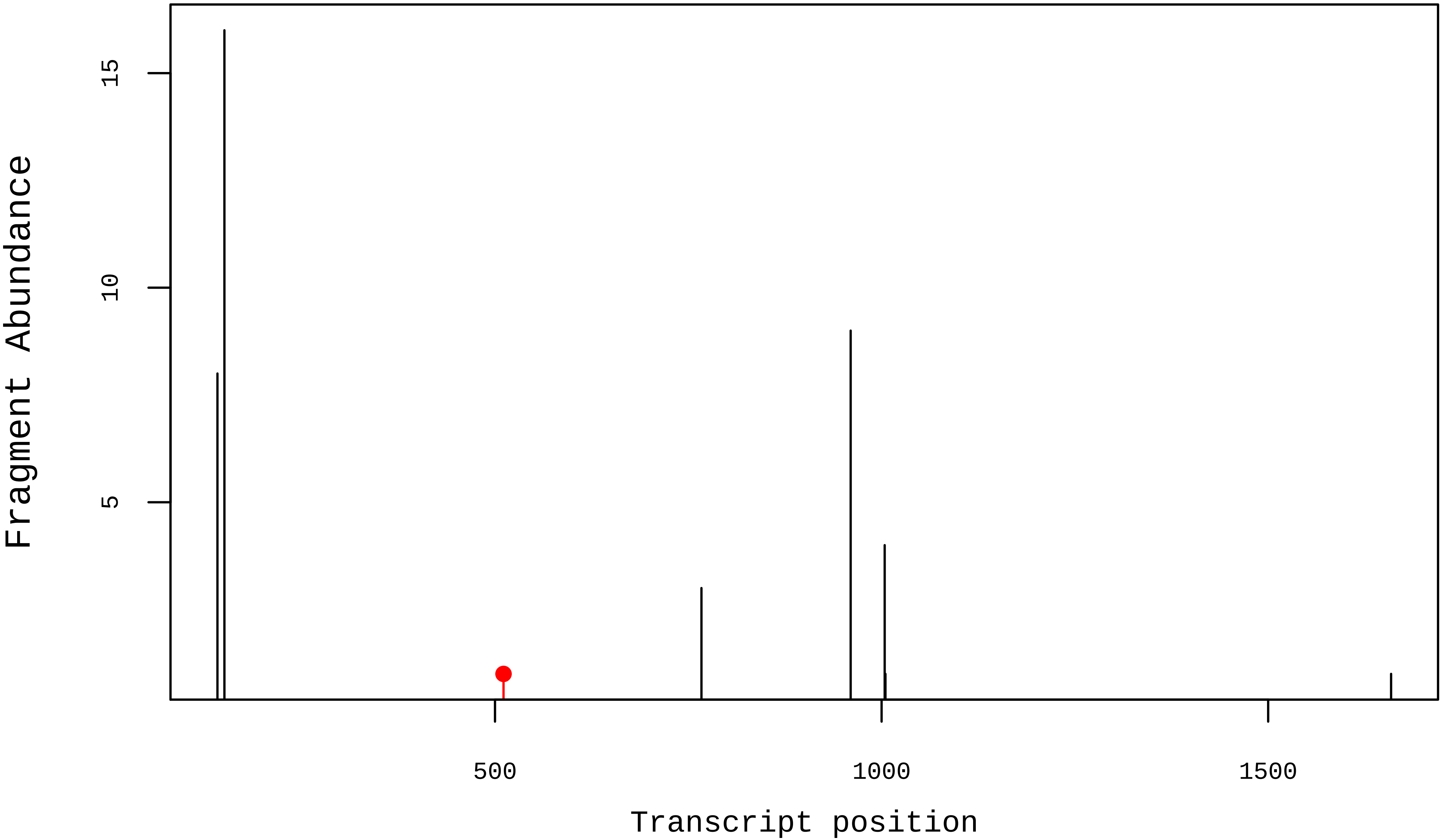

Cleavage site: 511   Tag abundance: 1   Weighted abundance: 0.25   Category: 4
sRNA abundance: 1   Alignment score: 4   MFE ratio: 0.751   p-value: 0.006

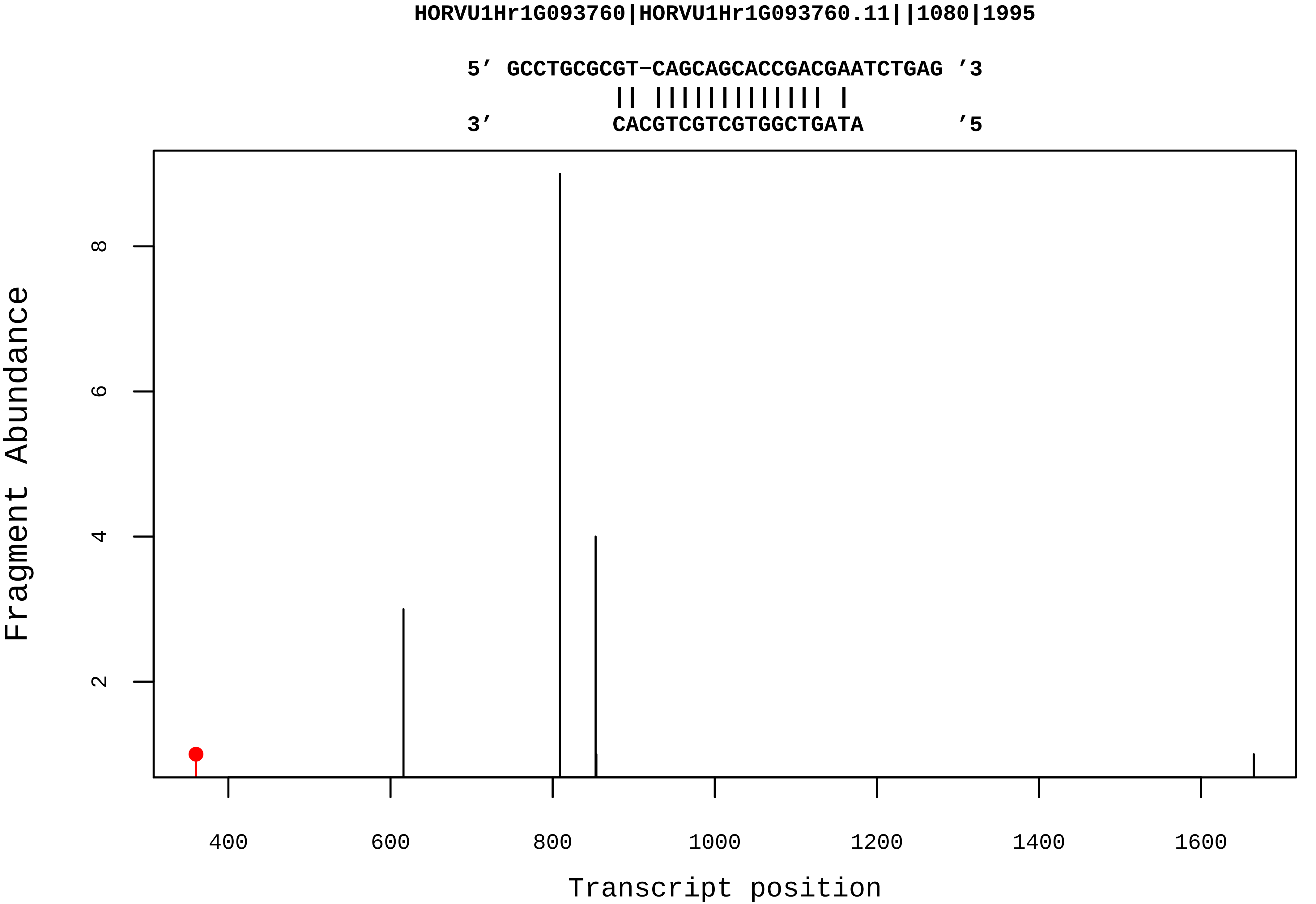

Cleavage site: 360 Tag abundance: 1 Weighted abundance: 0.25 Category: 4
sRNA abundance: 1 Alignment score: 4 MFE ratio: 0.751 p-value: 0.006

HORVU1Hr1G093760|HORVU1Hr1G093760.12||1383|1383

```
5' GCCTGCGCGT-CAGCAGCACCGACGAATCTGAG '3
             || |||||||||||||| |
3'           CACGTCGTCGTGGCTGATA       '5
```

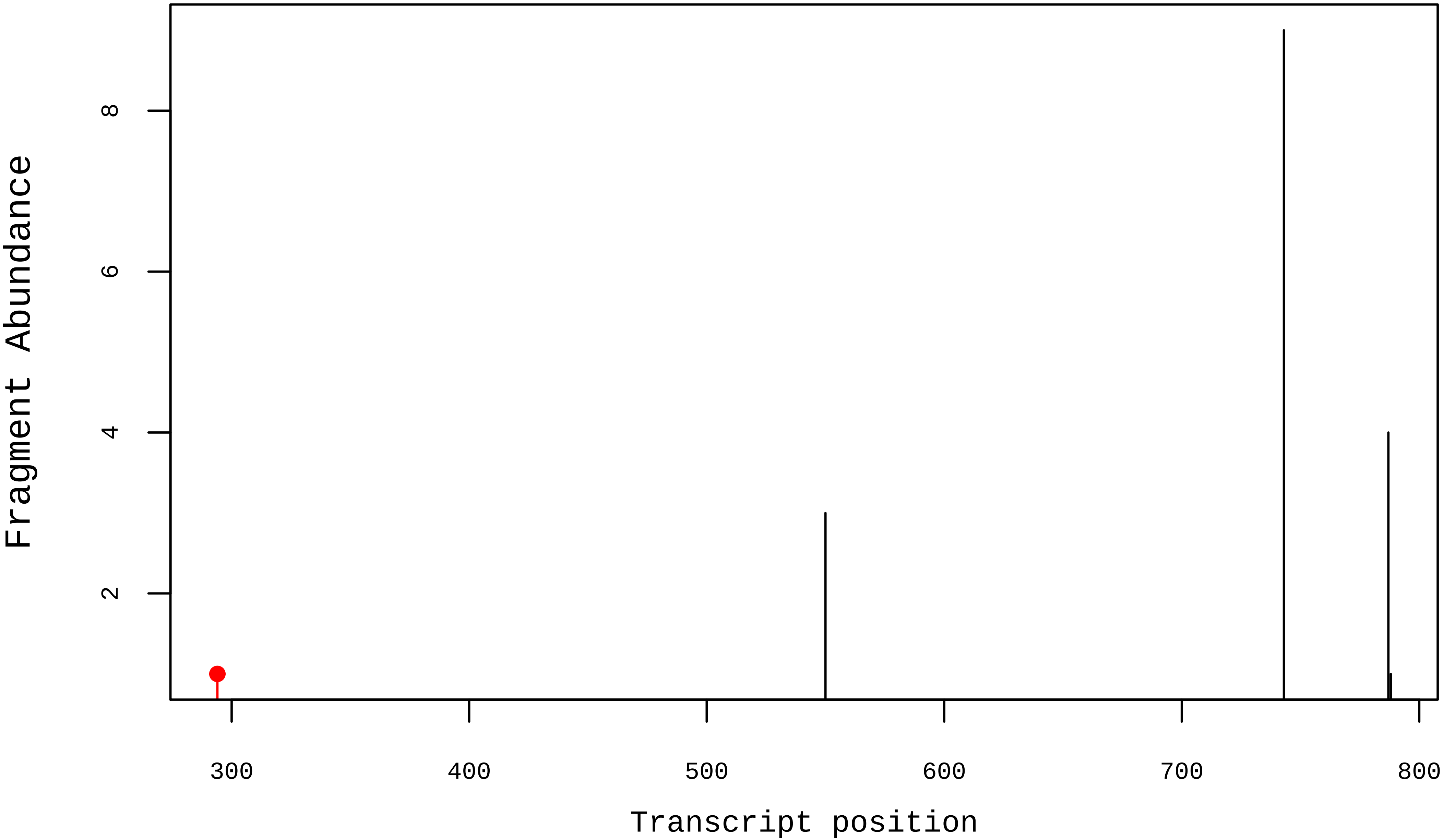

Cleavage site: 294   Tag abundance: 1   Weighted abundance: 0.25   Category: 4
sRNA abundance: 1   Alignment score: 4   MFE ratio: 0.751   p-value: 0.006

HORVU1Hr1G093760|HORVU1Hr1G093760.13||1455|1470

```
5' GCCTGCGCGT-CAGCAGCACCGACGAATCTGAG '3
            || ||||||||||||| |
3'          CACGTCGTCGTGGCTGATA       '5
```

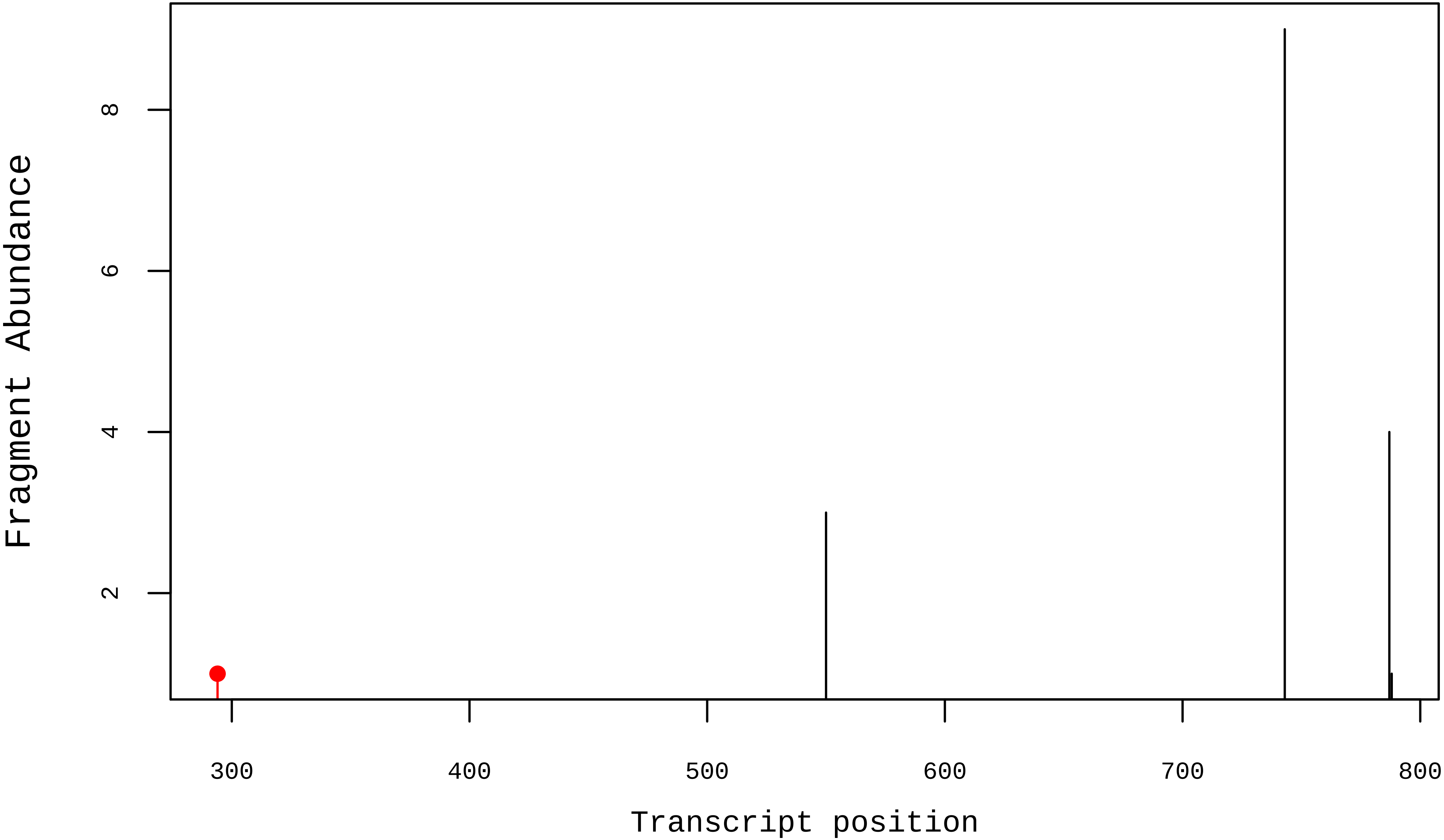

Cleavage site: 294 Tag abundance: 1 Weighted abundance: 0.25 Category: 4
sRNA abundance: 1 Alignment score: 4 MFE ratio: 0.751 p-value: 0.006

HORVU3Hr1G094250|HORVU3Hr1G094250.1||879|1653

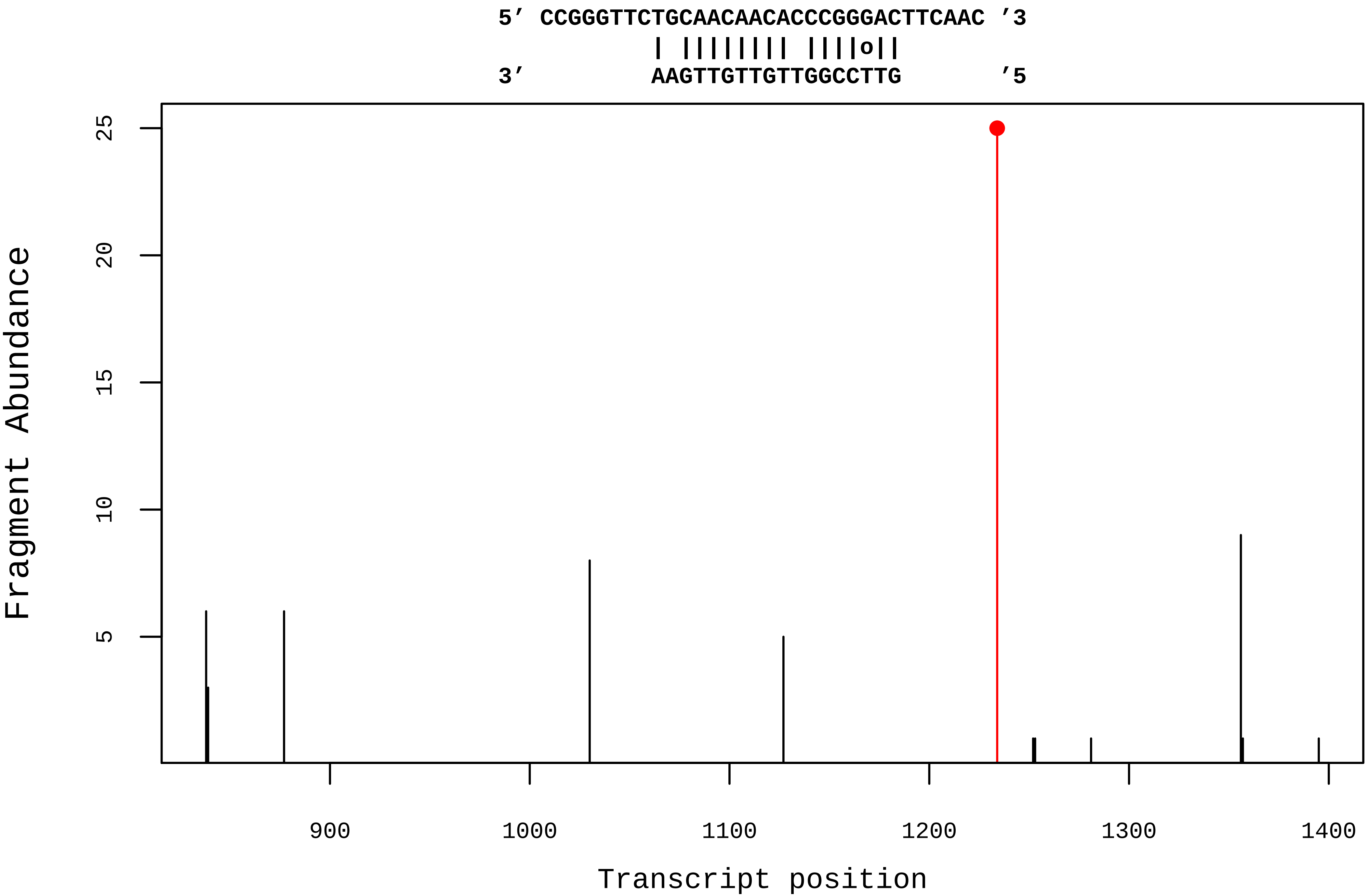

Cleavage site: 1234 Tag abundance: 25 Weighted abundance: 3.125 Category: 0
sRNA abundance: 1 Alignment score: 4 MFE ratio: 0.779 p-value: 0.015

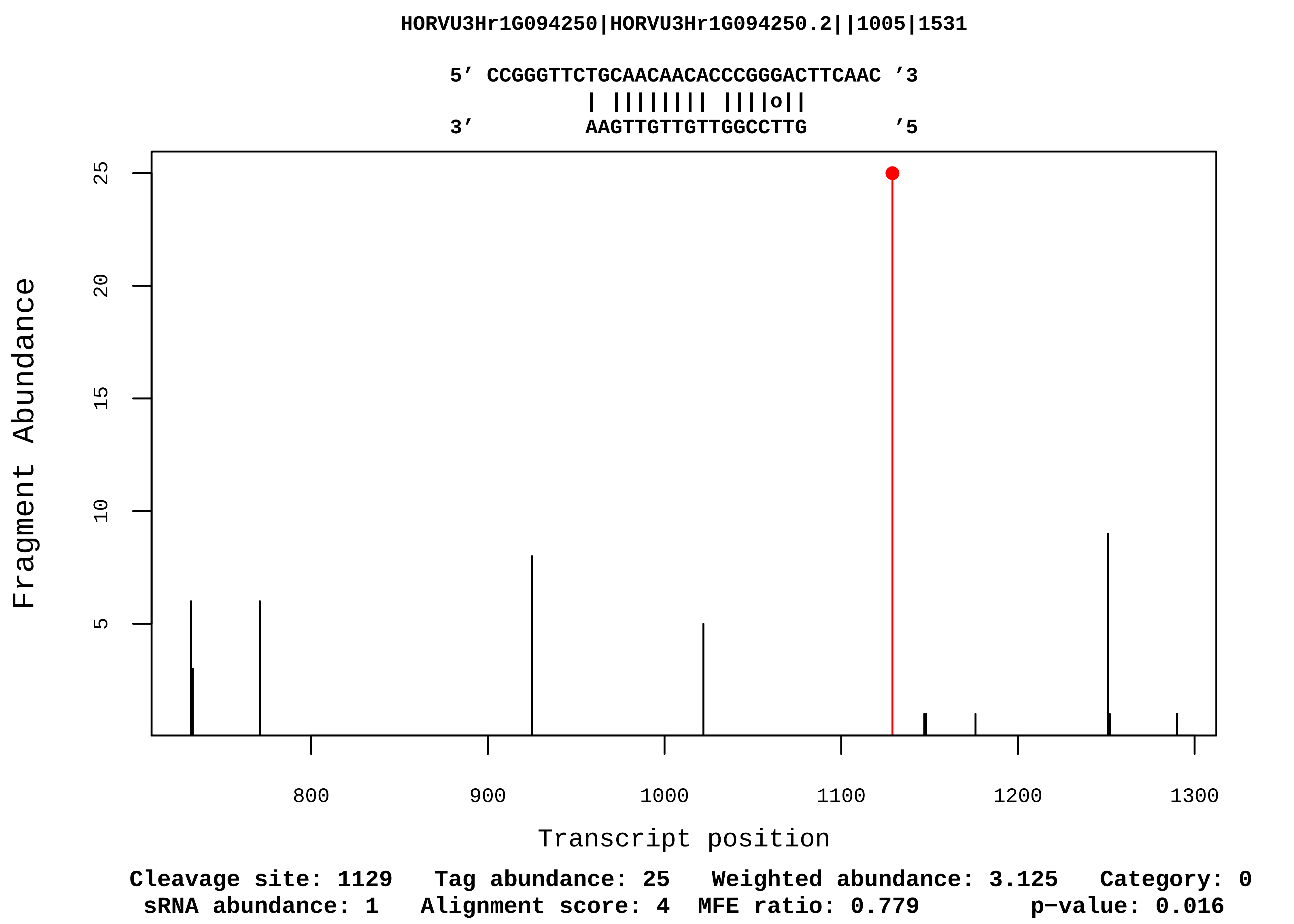
HORVU3Hr1G094250|HORVU3Hr1G094250.2||1005|1531
5' CCGGGTTCTGCAACAACACCCGGGACTTCAAC '3
| |||||||| ||||o||
3' AAGTTGTTGTTGGCCTTG '5
Fragment Abundance
25
20
15
10
5
800
900
1000
1100
1200
1300
Transcript position
Cleavage site: 1129 Tag abundance: 25 Weighted abundance: 3.125 Category: 0
sRNA abundance: 1 Alignment score: 4 MFE ratio: 0.779 p-value: 0.016

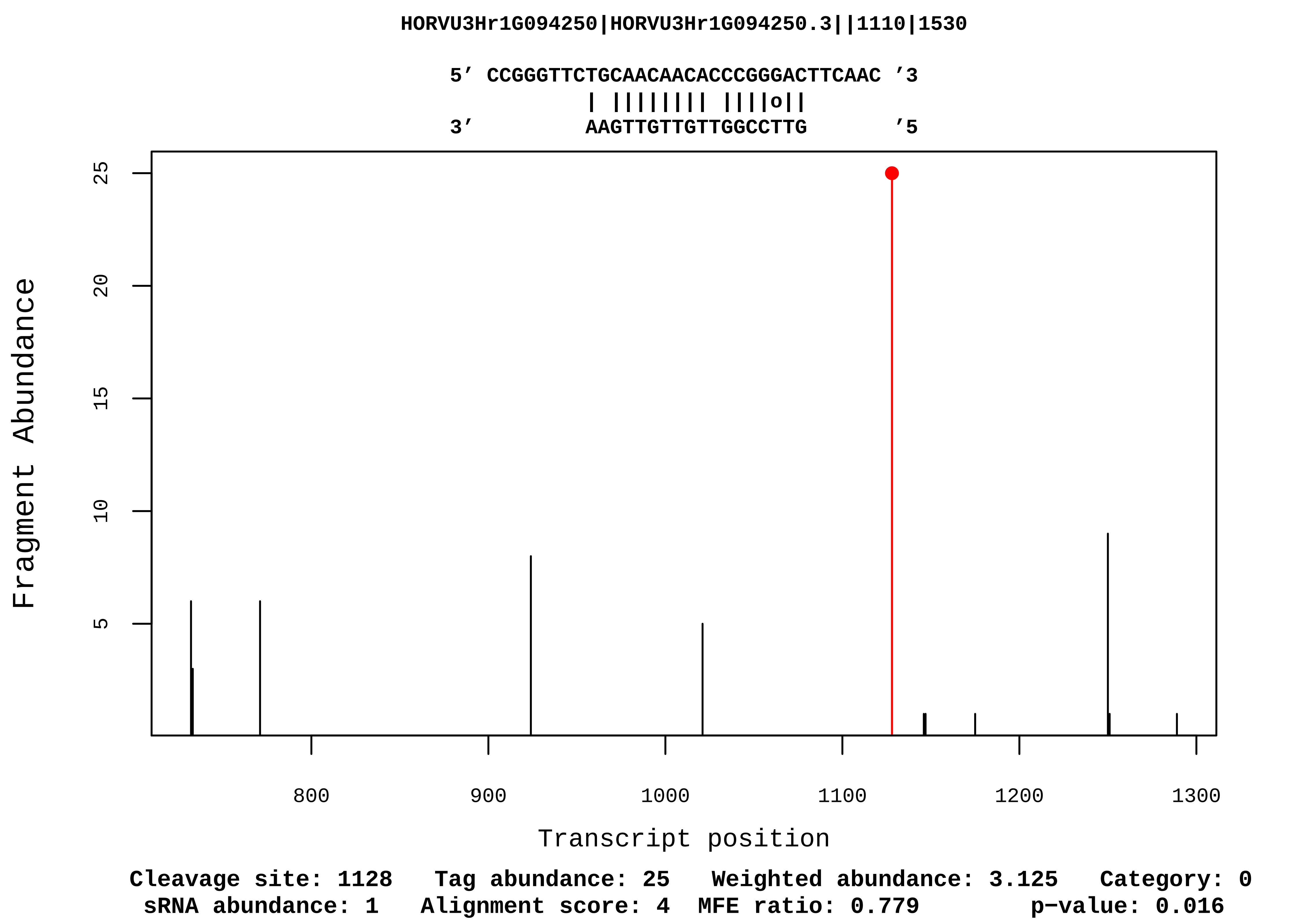
HORVU3Hr1G094250|HORVU3Hr1G094250.3||1110|1530
5′ CCGGGTTCTGCAACAACACCCGGGACTTCAAC ′3
| ||||||||| ||||o||
3′ AAGTTGTTGTTGGCCTTG ′5
Fragment Abundance
25
20
15
10
5
800
900
1000
1100
1200
1300
Transcript position
Cleavage site: 1128 Tag abundance: 25 Weighted abundance: 3.125 Category: 0
sRNA abundance: 1 Alignment score: 4 MFE ratio: 0.779 p-value: 0.016

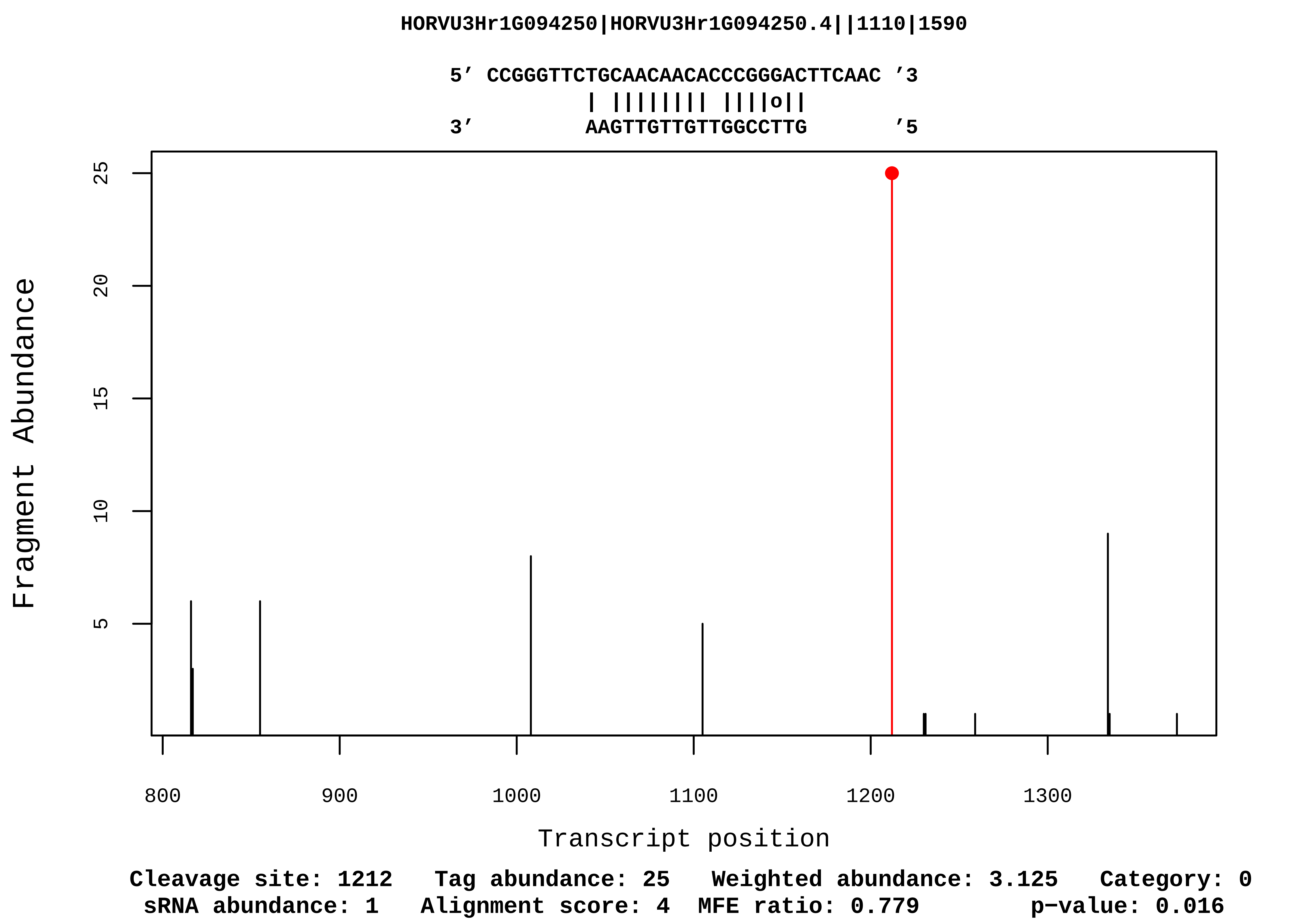

HORVU3Hr1G094250|HORVU3Hr1G094250.4||1110|1590
5′ CCGGGTTCTGCAACAACACCCGGGACTTCAAC ′3
| ||||||||| ||||o||
3′ AAGTTGTTGTTGGCCTTG ′5
Fragment Abundance
25
20
15
10
5
800
900
1000
1100
1200
1300
Transcript position
Cleavage site: 1212 Tag abundance: 25 Weighted abundance: 3.125 Category: 0
sRNA abundance: 1 Alignment score: 4 MFE ratio: 0.779 p-value: 0.016

HORVU3Hr1G094250|HORVU3Hr1G094250.5||1005|1591

```
5' CCGGGTTCTGCAACAACACCCGGGACTTCAAC '3
            | |||||||| ||||o||
3'          AAGTTGTTGTTGGCCTTG          '5
```

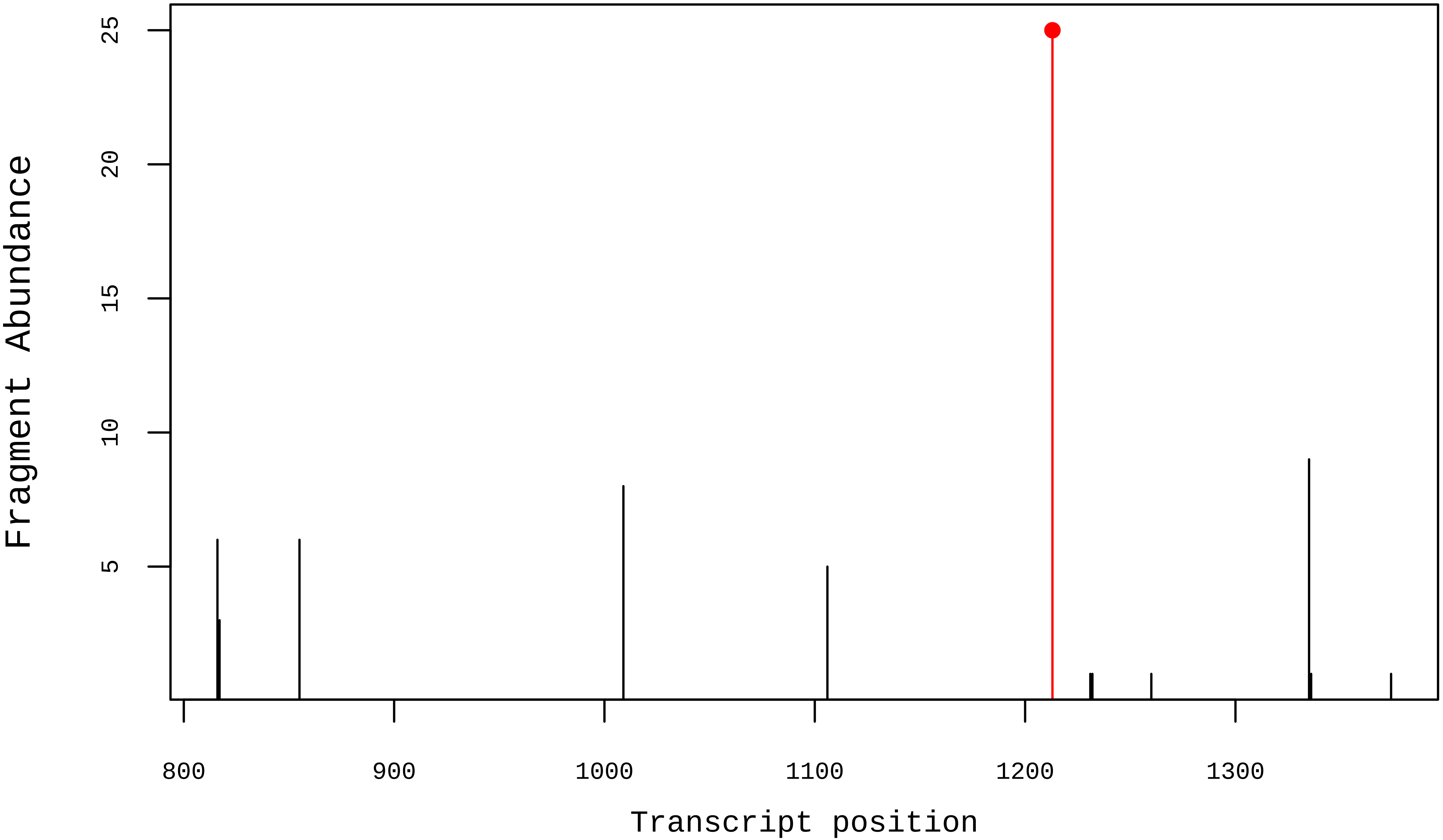

Cleavage site: 1213   Tag abundance: 25   Weighted abundance: 3.125   Category: 0
sRNA abundance: 1   Alignment score: 4   MFE ratio: 0.779   p-value: 0.016

HORVU3Hr1G094250|HORVU3Hr1G094250.6||1113|1593

```
5’ CCGGGTTCTGCAACAACACCCGGGACTTCAAC ’3
           | ||||||||| ||||o||
3’         AAGTTGTTGTTGGCCTTG         ’5
```

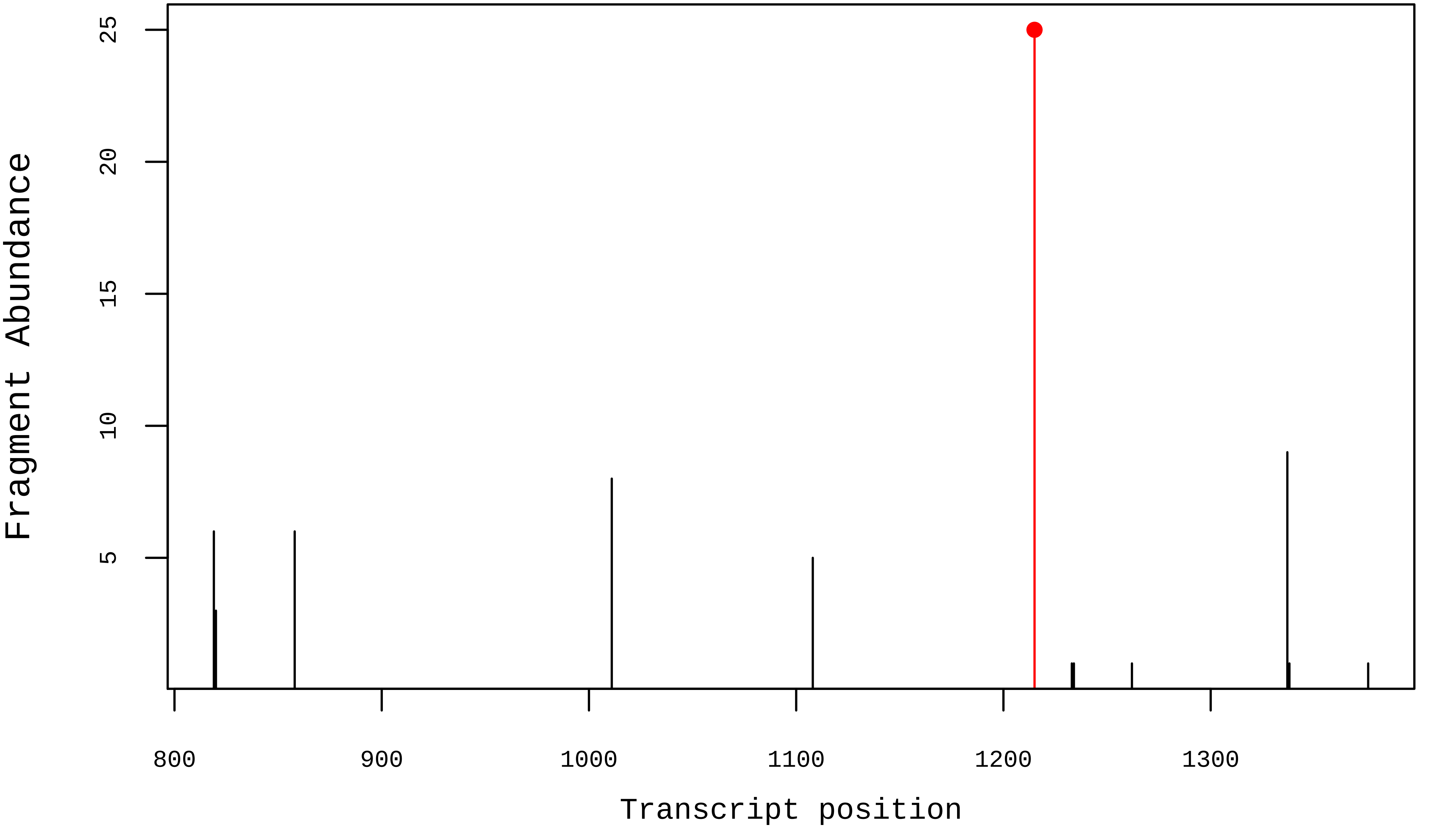

Cleavage site: 1215 Tag abundance: 25 Weighted abundance: 3.125 Category: 0
sRNA abundance: 1 Alignment score: 4 MFE ratio: 0.779 p-value: 0.016

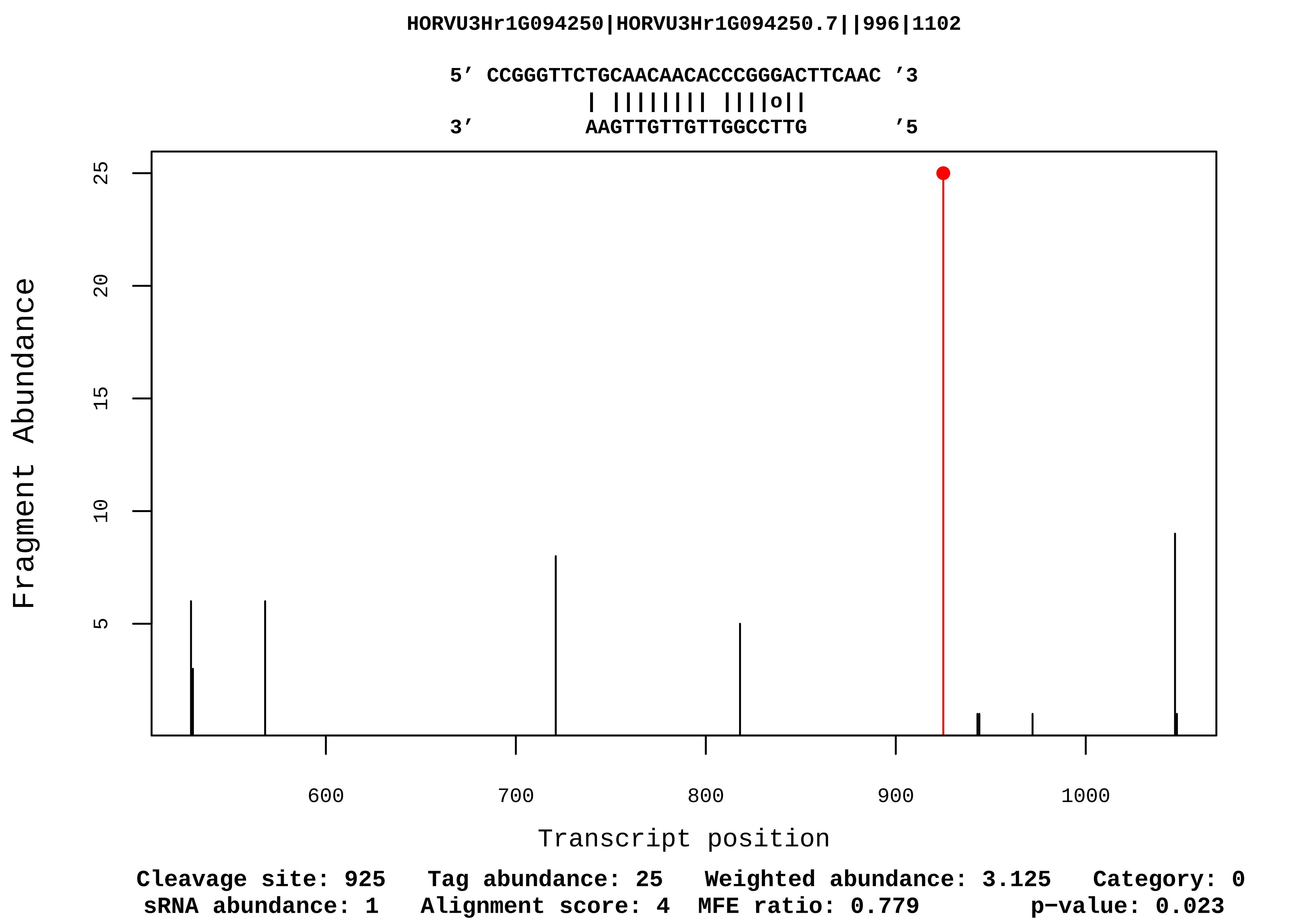
HORVU3Hr1G094250|HORVU3Hr1G094250.7||996|1102
5' CCGGGTTCTGCAACAACACCCGGGACTTCAAC '3
| ||||||||| ||||o||
3' AAGTTGTTGTTGGCCTTG '5
Fragment Abundance
25
20
15
10
5
600
700
800
900
1000
Transcript position
Cleavage site: 925 Tag abundance: 25 Weighted abundance: 3.125 Category: 0
sRNA abundance: 1 Alignment score: 4 MFE ratio: 0.779 p-value: 0.023

HORVU3Hr1G094250|HORVU3Hr1G094250.8||1116|1116

```
5' CCGGGTTCTGCAACAACACCCGGGACTTCAAC '3
              | ||||||||| ||||o||
3'            AAGTTGTTGTTGGCCTTG       '5
```

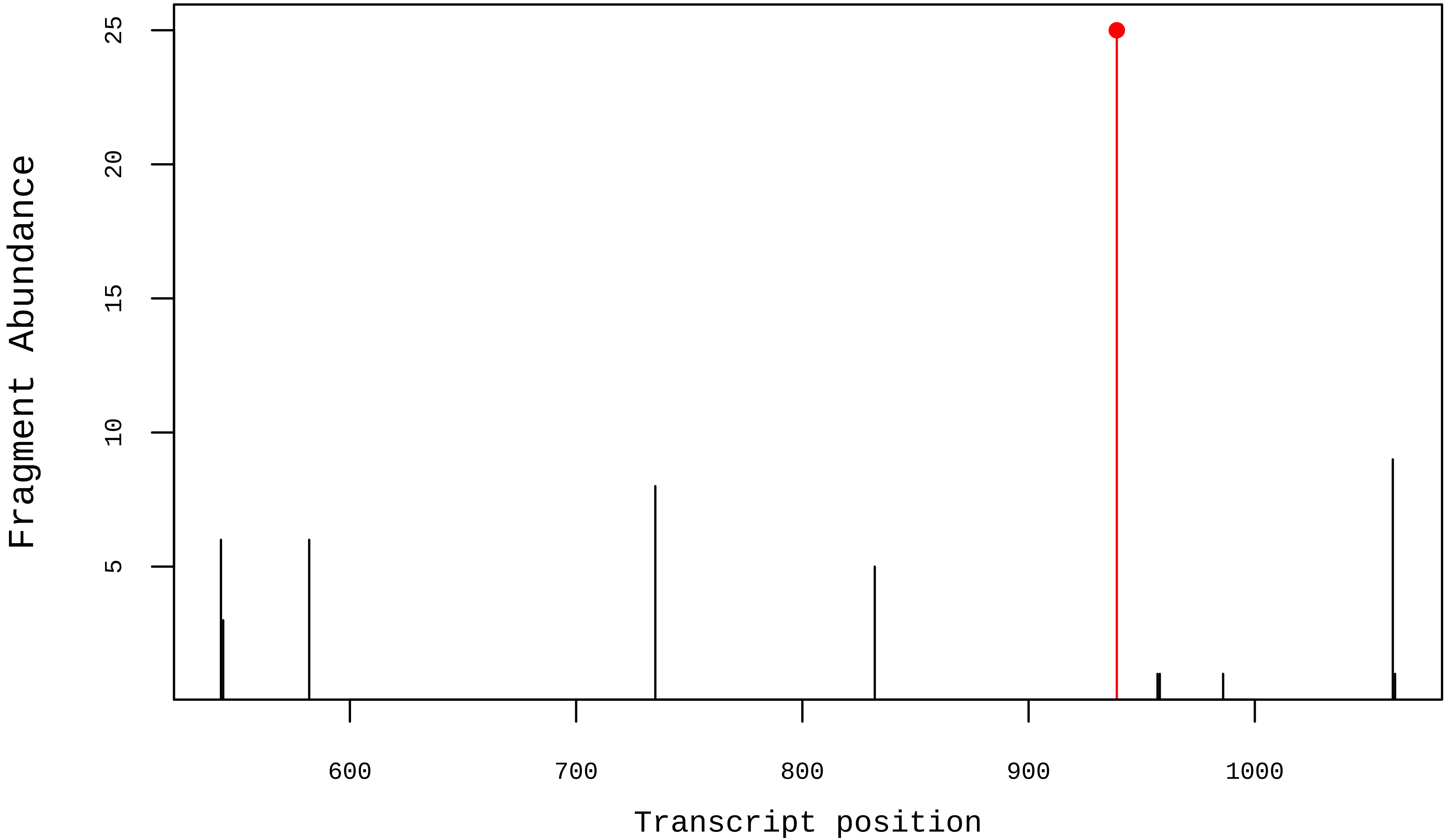

Cleavage site: 939   Tag abundance: 25   Weighted abundance: 3.125   Category: 0
sRNA abundance: 1   Alignment score: 4   MFE ratio: 0.779   p-value: 0.023

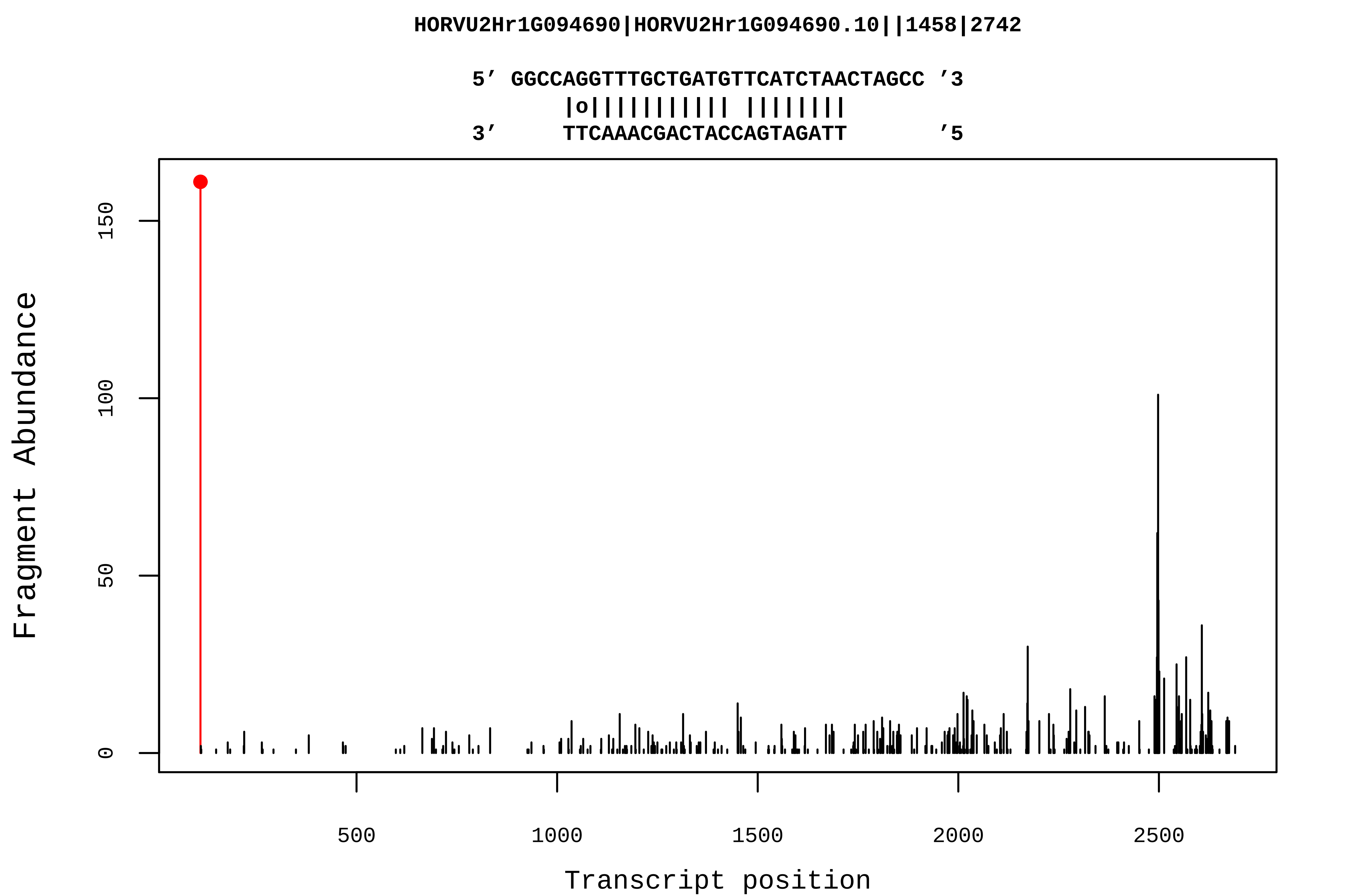

Cleavage site: 111 Tag abundance: 161 Weighted abundance: 9.471 Category: 0
sRNA abundance: 1 Alignment score: 2.5 MFE ratio: 0.803 p-value: 0.009

HORVU2Hr1G094690|HORVU2Hr1G094690.11||1458|2742

```
5' GGCCAGGTTTGCTGATGTTCATCTAACTAGCC '3
       |o|||||||||| ||||||||
3'     TTCAAACGACTACCAGTAGATT        '5
```

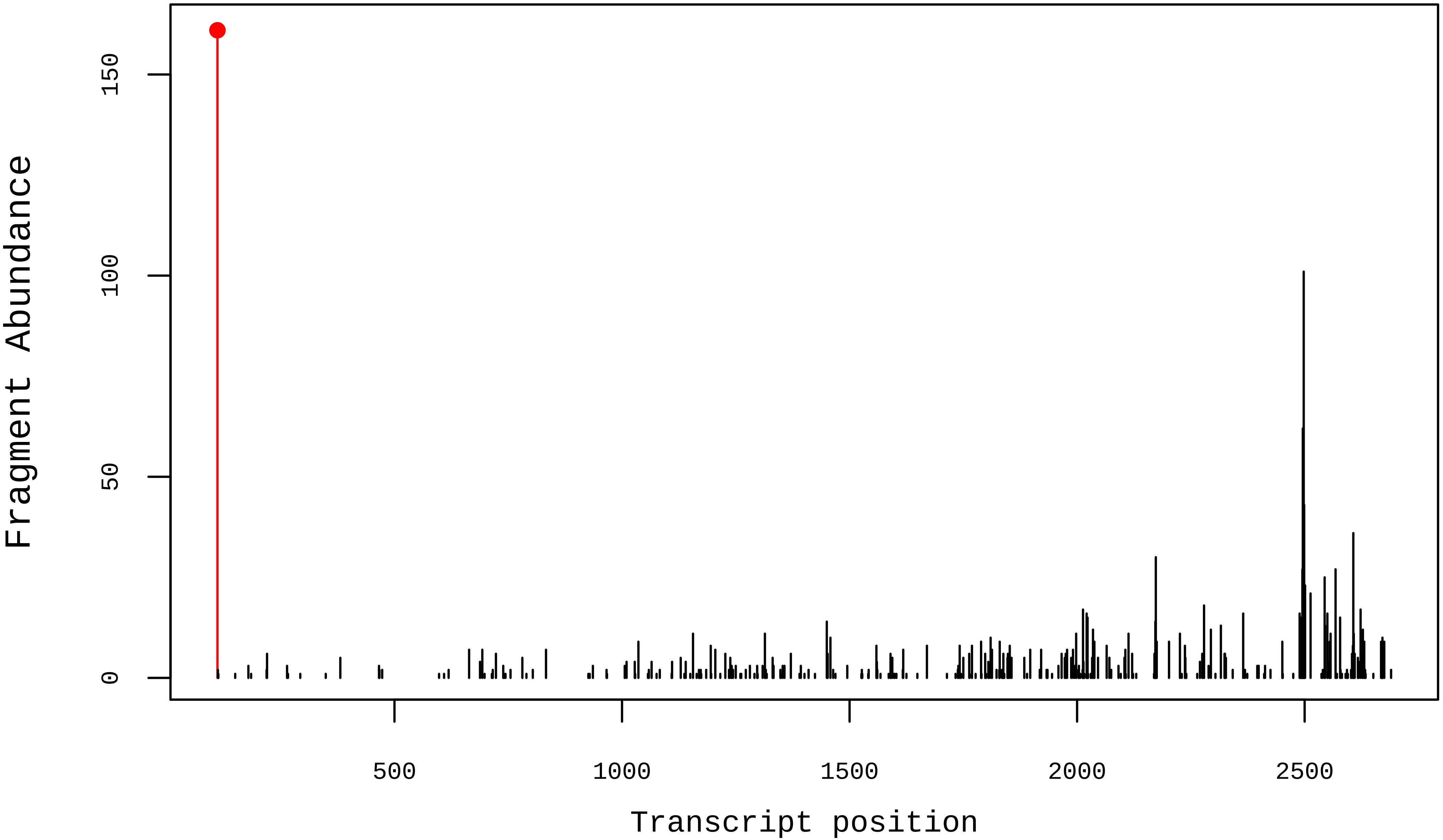

Cleavage site: 111   Tag abundance: 161   Weighted abundance: 9.471   Category: 0
sRNA abundance: 1   Alignment score: 2.5   MFE ratio: 0.803   p-value: 0.009

HORVU2Hr1G094690|HORVU2Hr1G094690.12||2112|2910

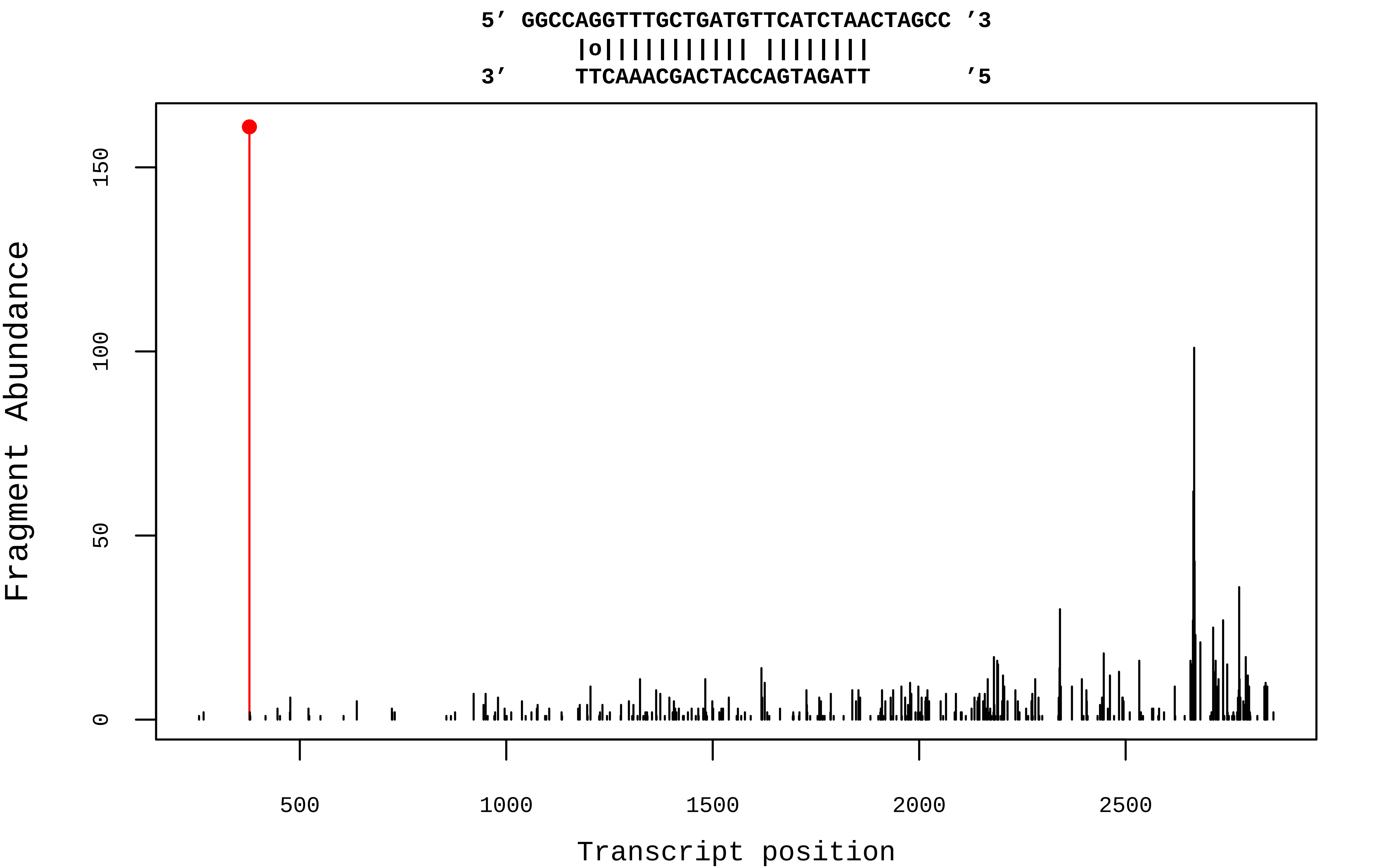

Cleavage site: 378 Tag abundance: 161 Weighted abundance: 9.471 Category: 0
sRNA abundance: 1 Alignment score: 2.5 MFE ratio: 0.803 p-value: 0.009

HORVU2Hr1G094690|HORVU2Hr1G094690.13||2112|2916

```
5' GGCCAGGTTTGCTGATGTTCATCTAACTAGCC '3
         |o|||||||||||| ||||||||
3'       TTCAAACGACTACCAGTAGATT        '5
```

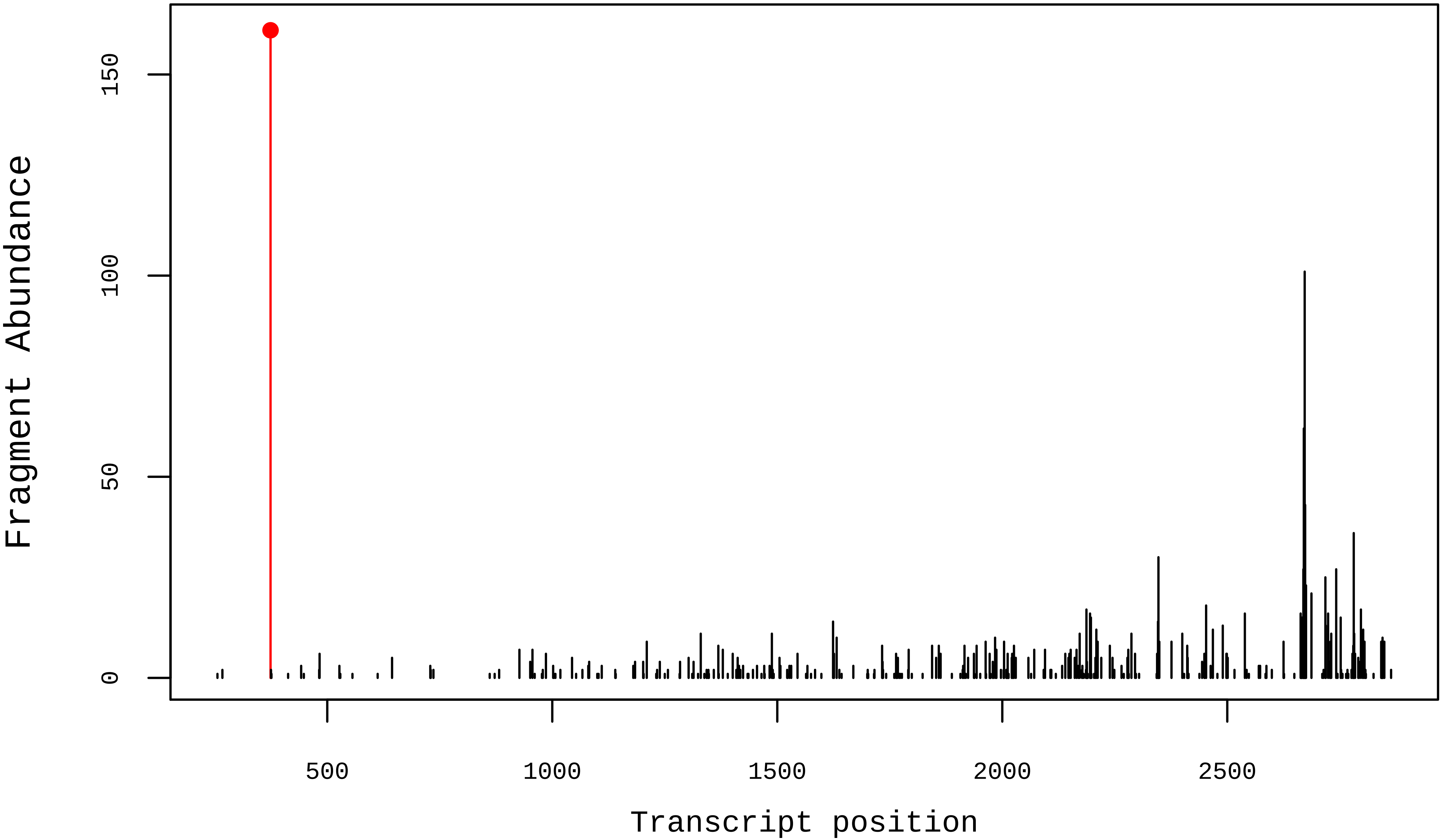

Cleavage site: 374   Tag abundance: 161   Weighted abundance: 9.471   Category: 0
sRNA abundance: 1   Alignment score: 2.5   MFE ratio: 0.803   p-value: 0.009

HORVU2Hr1G094690|HORVU2Hr1G094690.14||2112|2920

```
5' GGCCAGGTTTGCTGATGTTCATCTAACTAGCC '3
         |o||||||||||| ||||||||
3'       TTCAAACGACTACCAGTAGATT       '5
```

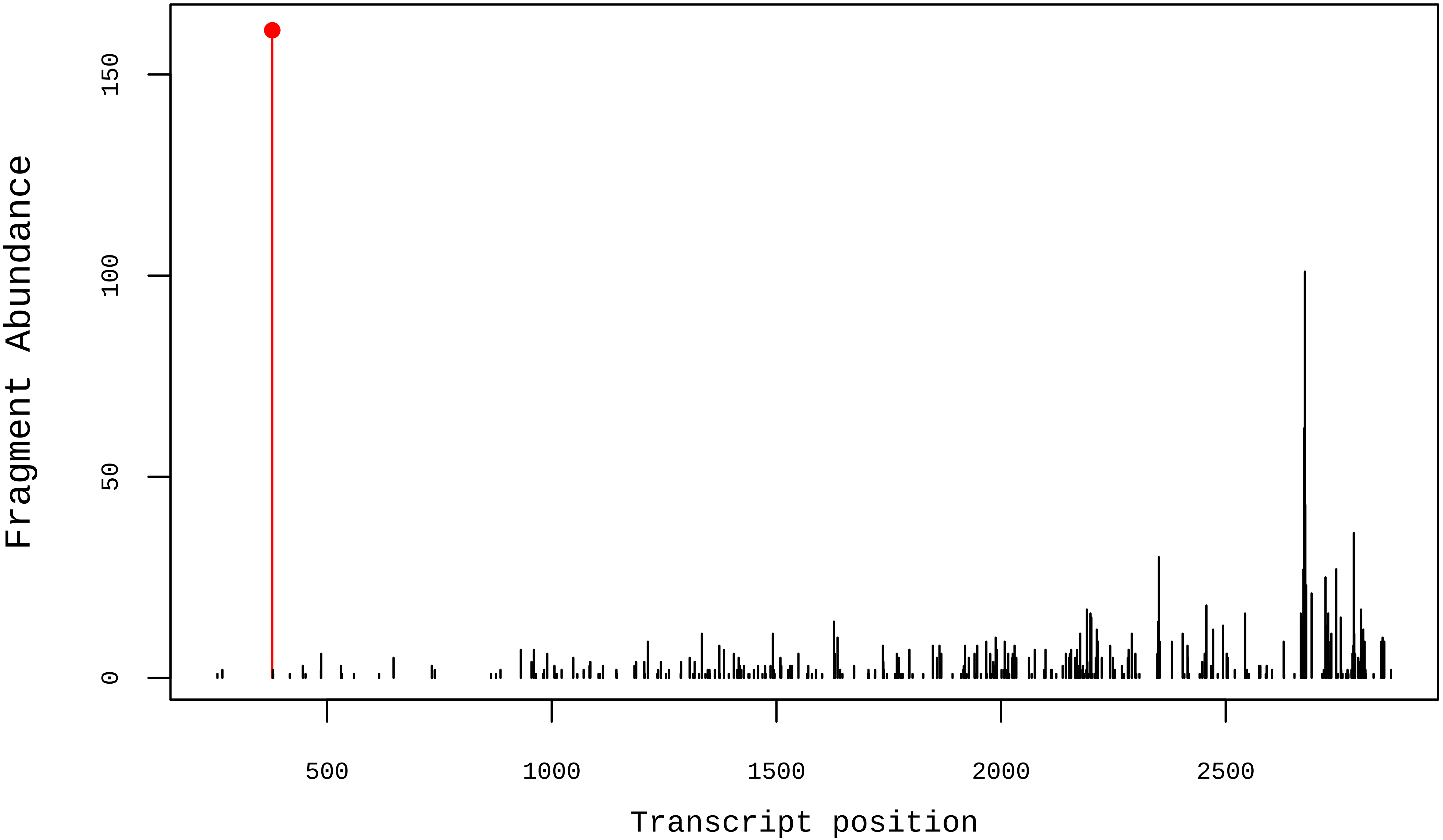

Cleavage site: 378 Tag abundance: 161 Weighted abundance: 9.471 Category: 0
sRNA abundance: 1 Alignment score: 2.5 MFE ratio: 0.803 p-value: 0.009

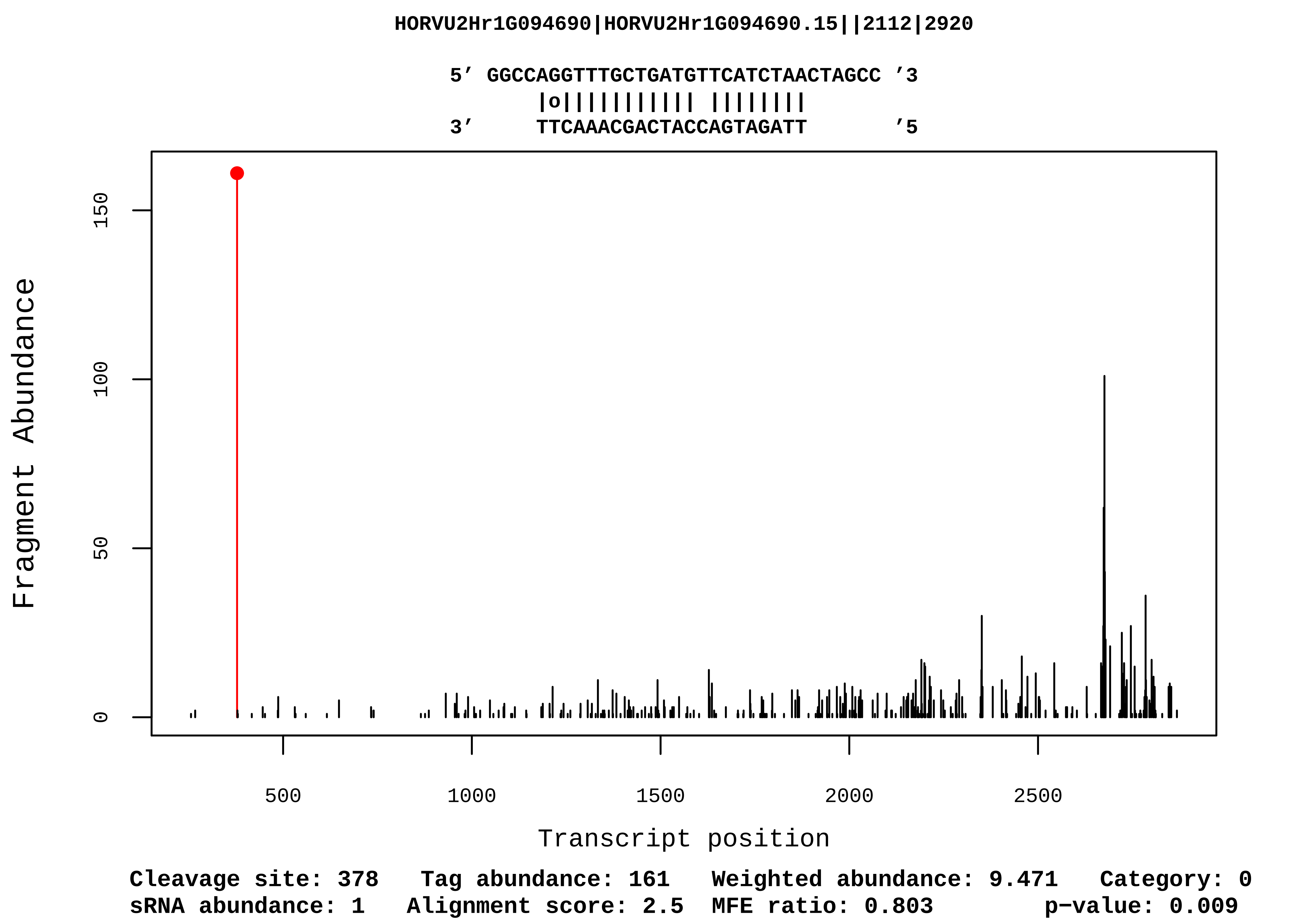
HORVU2Hr1G094690|HORVU2Hr1G094690.15||2112|2920
5' GGCCAGGTTTGCTGATGTTCATCTAACTAGCC '3
|o||||||||||| ||||||||
3' TTCAAACGACTACCAGTAGATT '5
Fragment Abundance
0
50
100
150
500
1000
1500
2000
2500
Transcript position
Cleavage site: 378
Tag abundance: 161
Weighted abundance: 9.471
Category: 0
sRNA abundance: 1
Alignment score: 2.5
MFE ratio: 0.803
p-value: 0.009

HORVU2Hr1G094690|HORVU2Hr1G094690.16||1458|2775

```
5' GGCCAGGTTTGCTGATGTTCATCTAACTAGCC '3
       |o||||||||||| ||||||||
3'     TTCAAACGACTACCAGTAGATT       '5
```

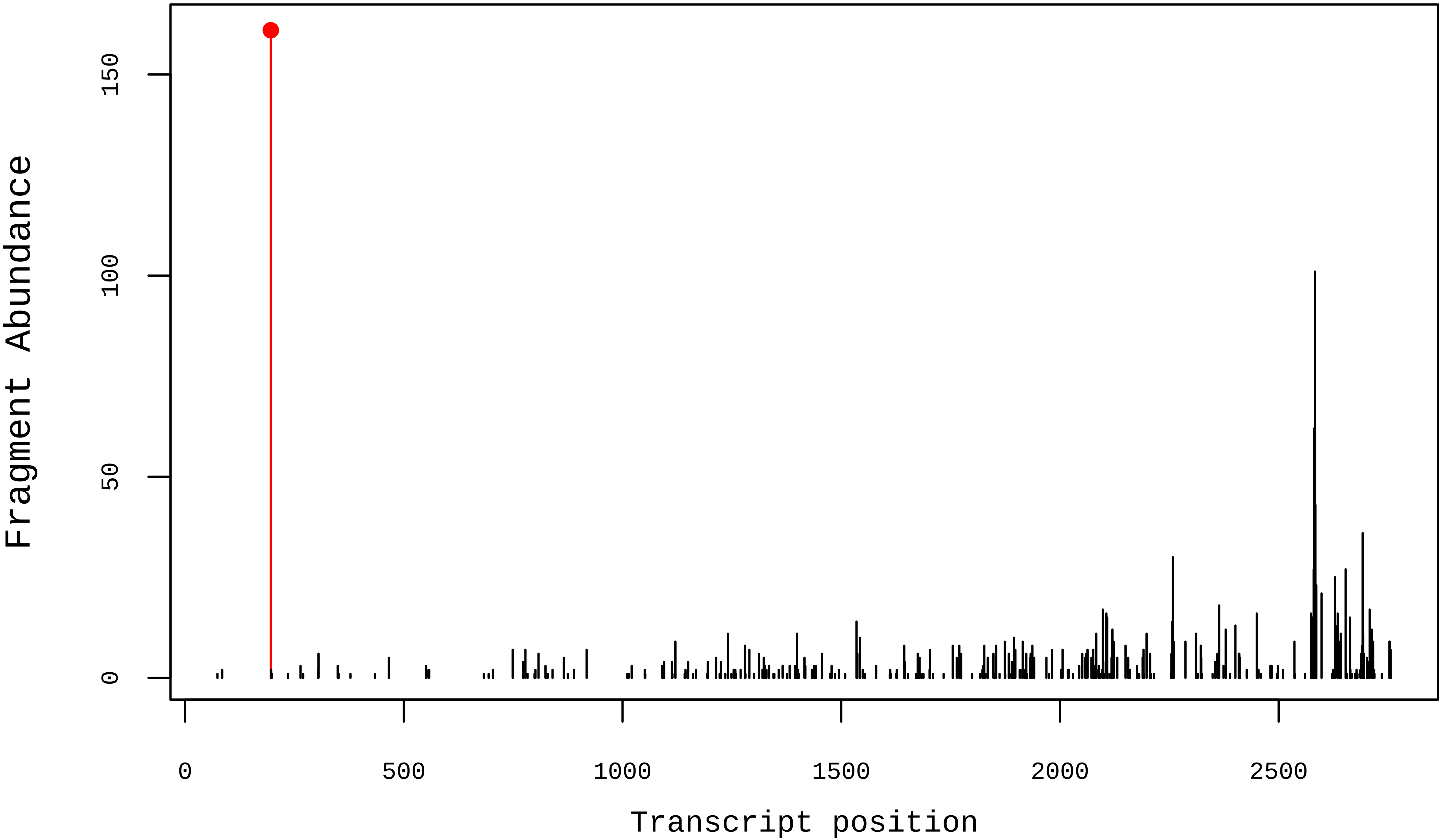

Cleavage site: 196   Tag abundance: 161   Weighted abundance: 9.471   Category: 0
sRNA abundance: 1   Alignment score: 2.5   MFE ratio: 0.803   p-value: 0.009

HORVU2Hr1G094690|HORVU2Hr1G094690.17||1458|2771

```
5' GGCCAGGTTTGCTGATGTTCATCTAACTAGCC '3
         |o||||||||||| ||||||||
3'       TTCAAACGACTACCAGTAGATT       '5
```

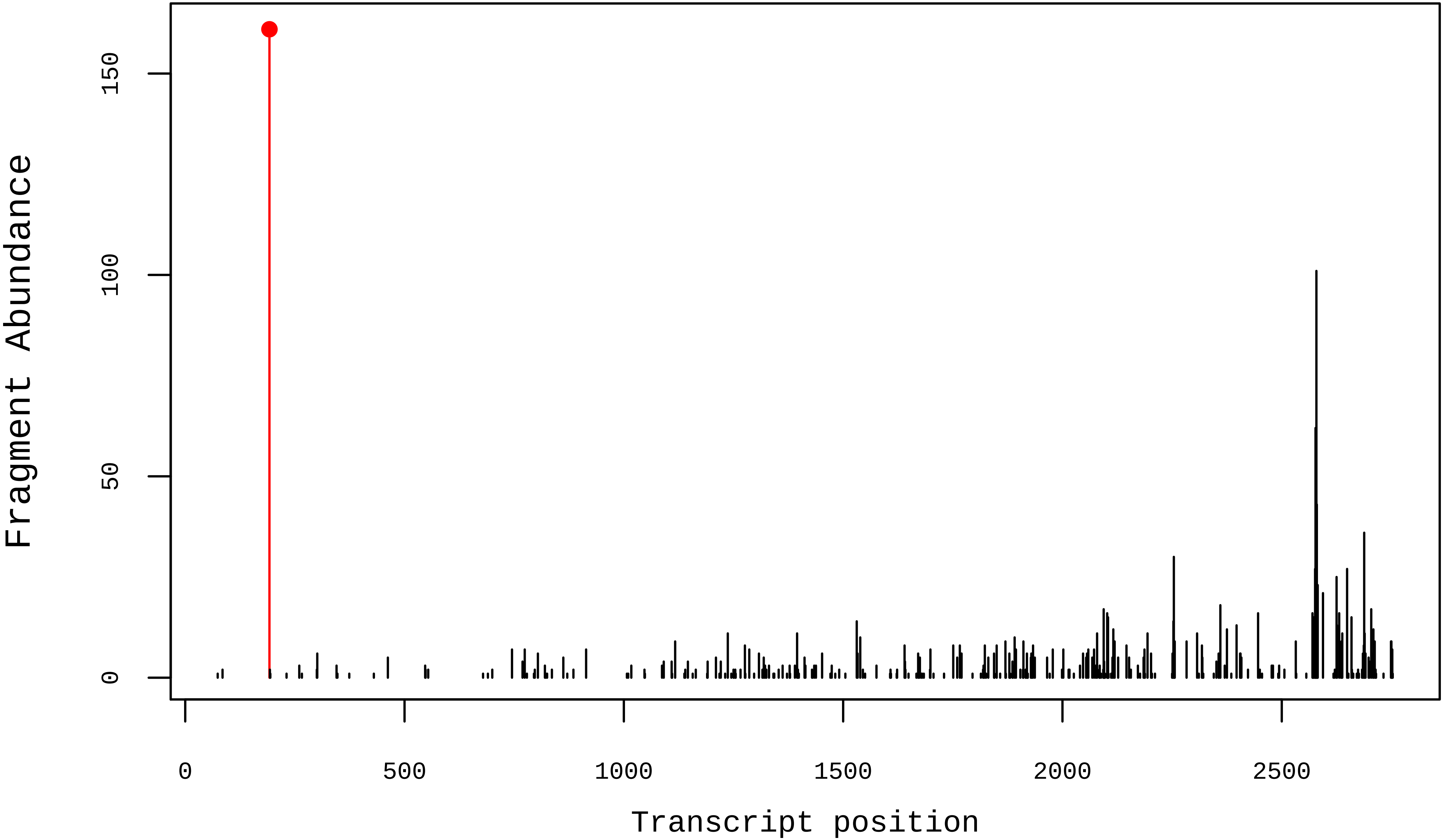

Cleavage site: 192   Tag abundance: 161   Weighted abundance: 9.471   Category: 0
sRNA abundance: 1   Alignment score: 2.5   MFE ratio: 0.803   p-value: 0.009

HORVU2Hr1G094690|HORVU2Hr1G094690.18||1458|2775

```
5' GGCCAGGTTTGCTGATGTTCATCTAACTAGCC '3
      |o|||||||||| ||||||||
3'    TTCAAACGACTACCAGTAGATT       '5
```

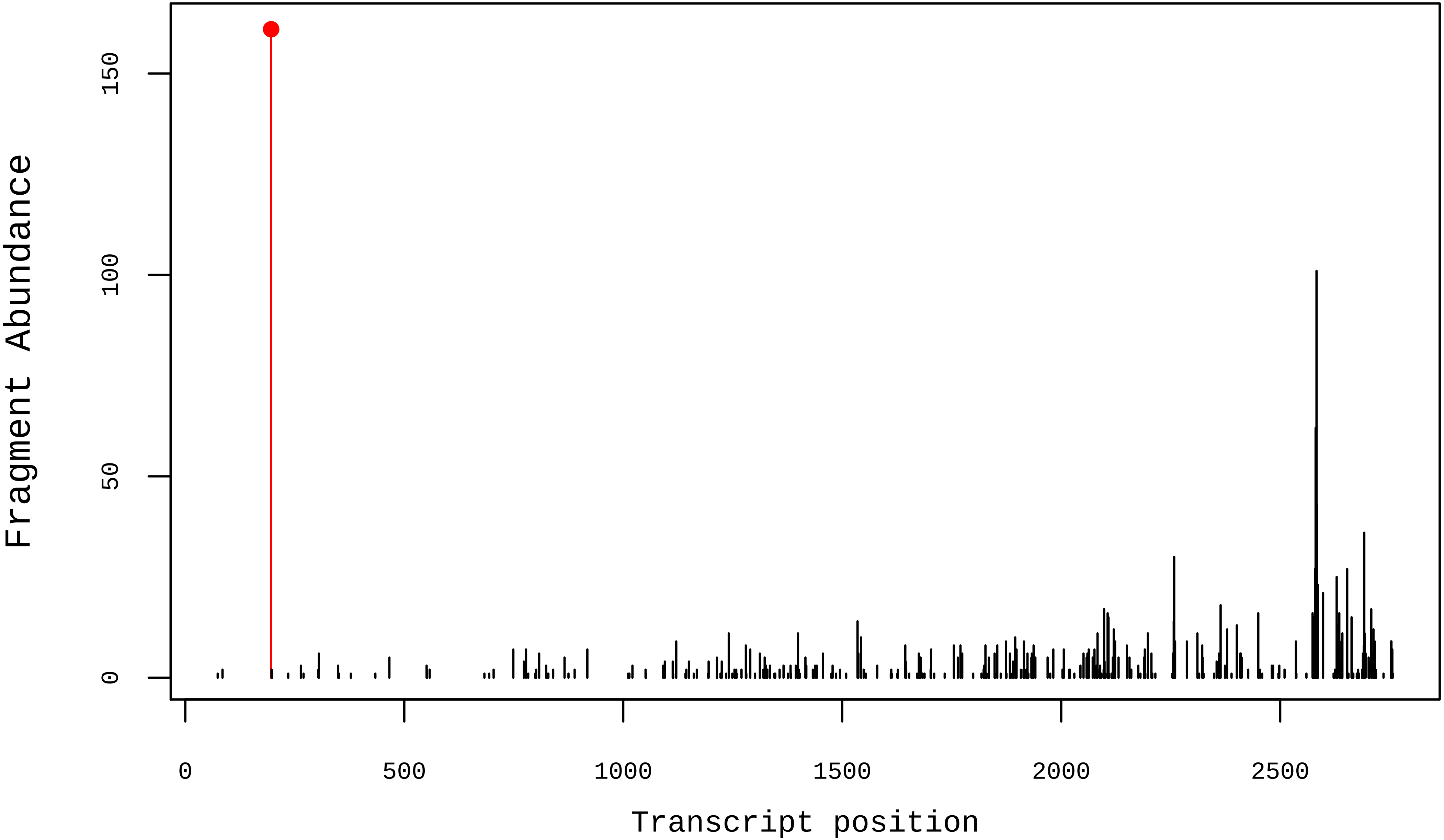

Cleavage site: 196   Tag abundance: 161   Weighted abundance: 9.471   Category: 0
sRNA abundance: 1   Alignment score: 2.5   MFE ratio: 0.803   p-value: 0.009

HORVU2Hr1G094690|HORVU2Hr1G094690.19||1458|2630

5′ GGCCAGGTTTGCTGATGTTCATCTAACTAGCC ′3
|o||||||||||| ||||||||
3′ TTCAAACGACTACCAGTAGATT ′5

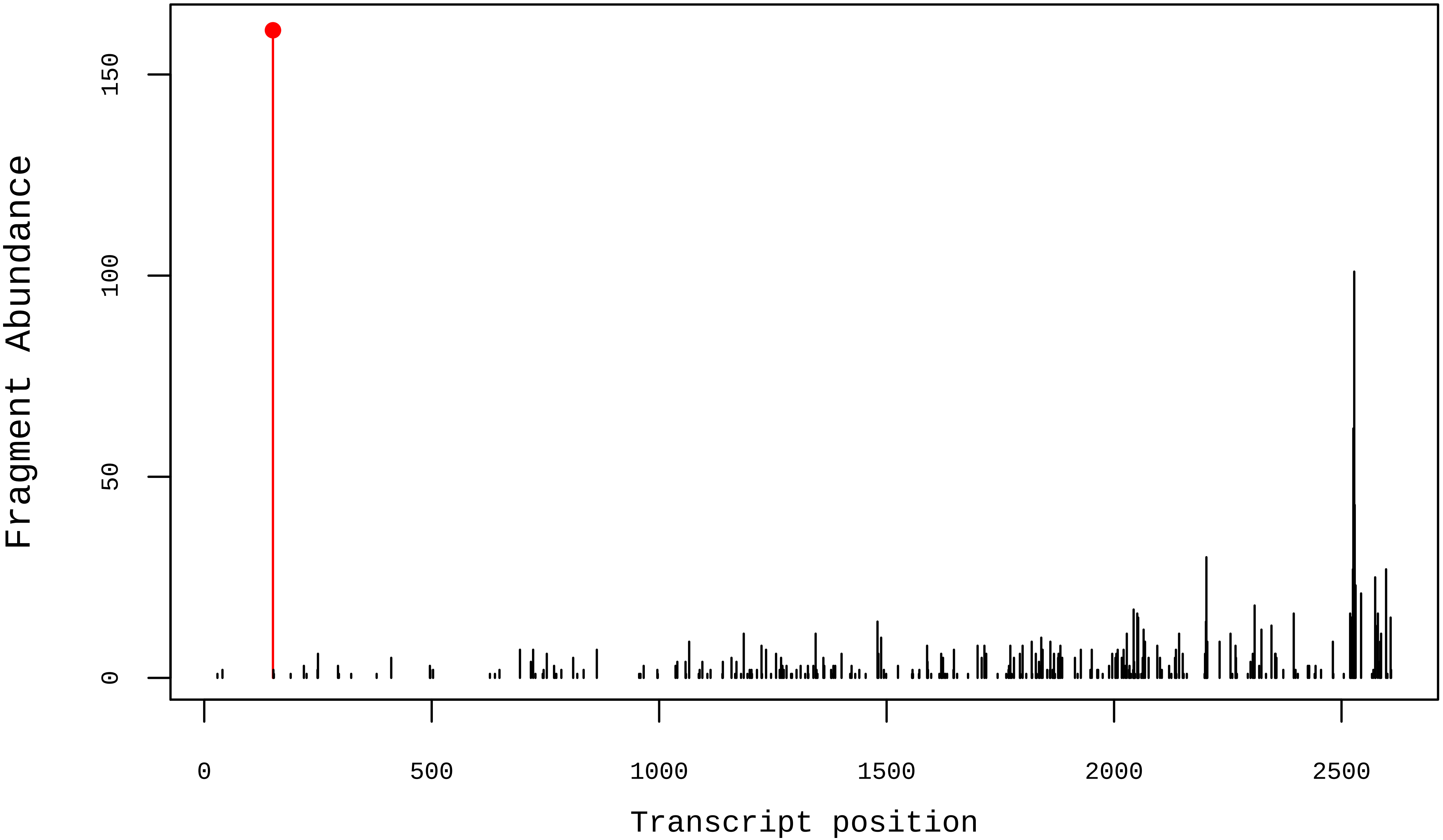

Cleavage site: 151 Tag abundance: 161 Weighted abundance: 9.471 Category: 0
sRNA abundance: 1 Alignment score: 2.5 MFE ratio: 0.803 p-value: 0.01

HORVU2Hr1G094690|HORVU2Hr1G094690.1||2112|2767

```
5' GGCCAGGTTTGCTGATGTTCATCTAACTAGCC '3
        |o||||||||||| ||||||||
3'      TTCAAACGACTACCAGTAGATT        '5
```

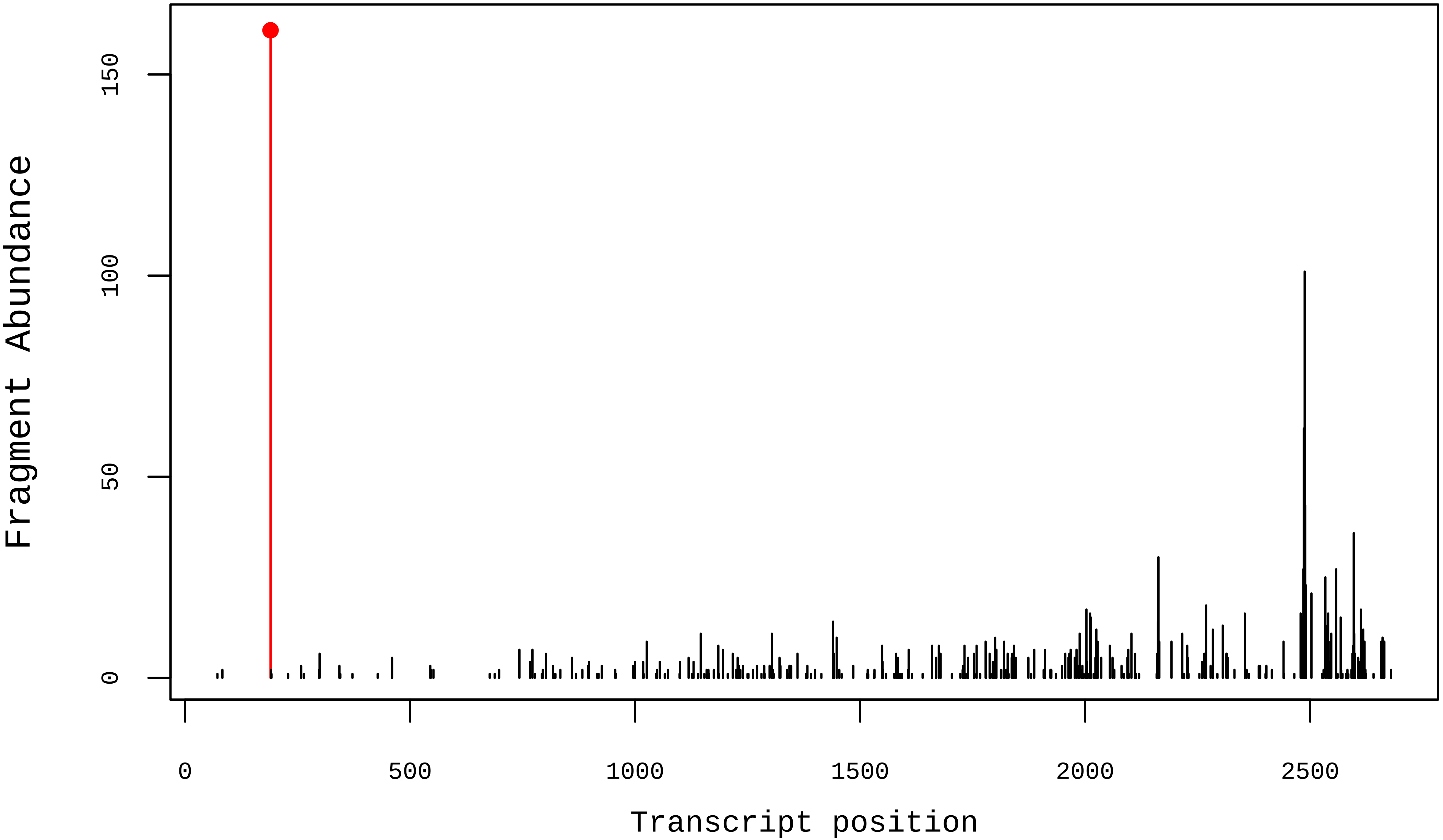

Cleavage site: 190   Tag abundance: 161   Weighted abundance: 9.471   Category: 0
sRNA abundance: 1   Alignment score: 2.5   MFE ratio: 0.803   p-value: 0.009

HORVU2Hr1G094690|HORVU2Hr1G094690.2||2112|2761

```
5' GGCCAGGTTTGCTGATGTTCATCTAACTAGCC '3
         |o|||||||||| ||||||||
3'       TTCAAACGACTACCAGTAGATT       '5
```

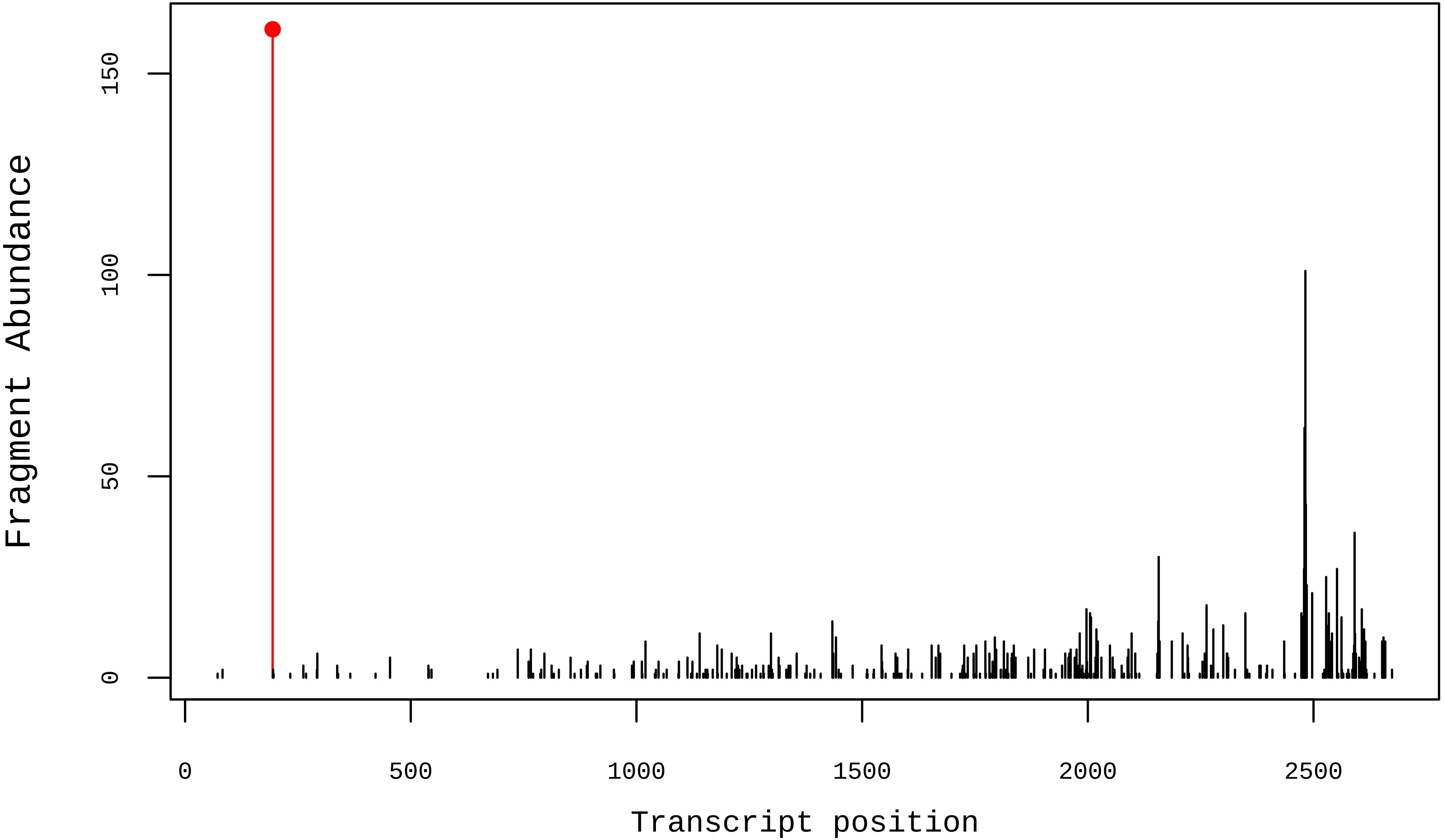

Cleavage site: 194   Tag abundance: 161   Weighted abundance: 9.471   Category: 0
sRNA abundance: 1   Alignment score: 2.5   MFE ratio: 0.803   p-value: 0.009

HORVU2Hr1G094690|HORVU2Hr1G094690.3||2112|2734

```
5' GGCCAGGTTTGCTGATGTTCATCTAACTAGCC '3
         |o||||||||||| ||||||||
3'       TTCAAACGACTACCAGTAGATT        '5
```

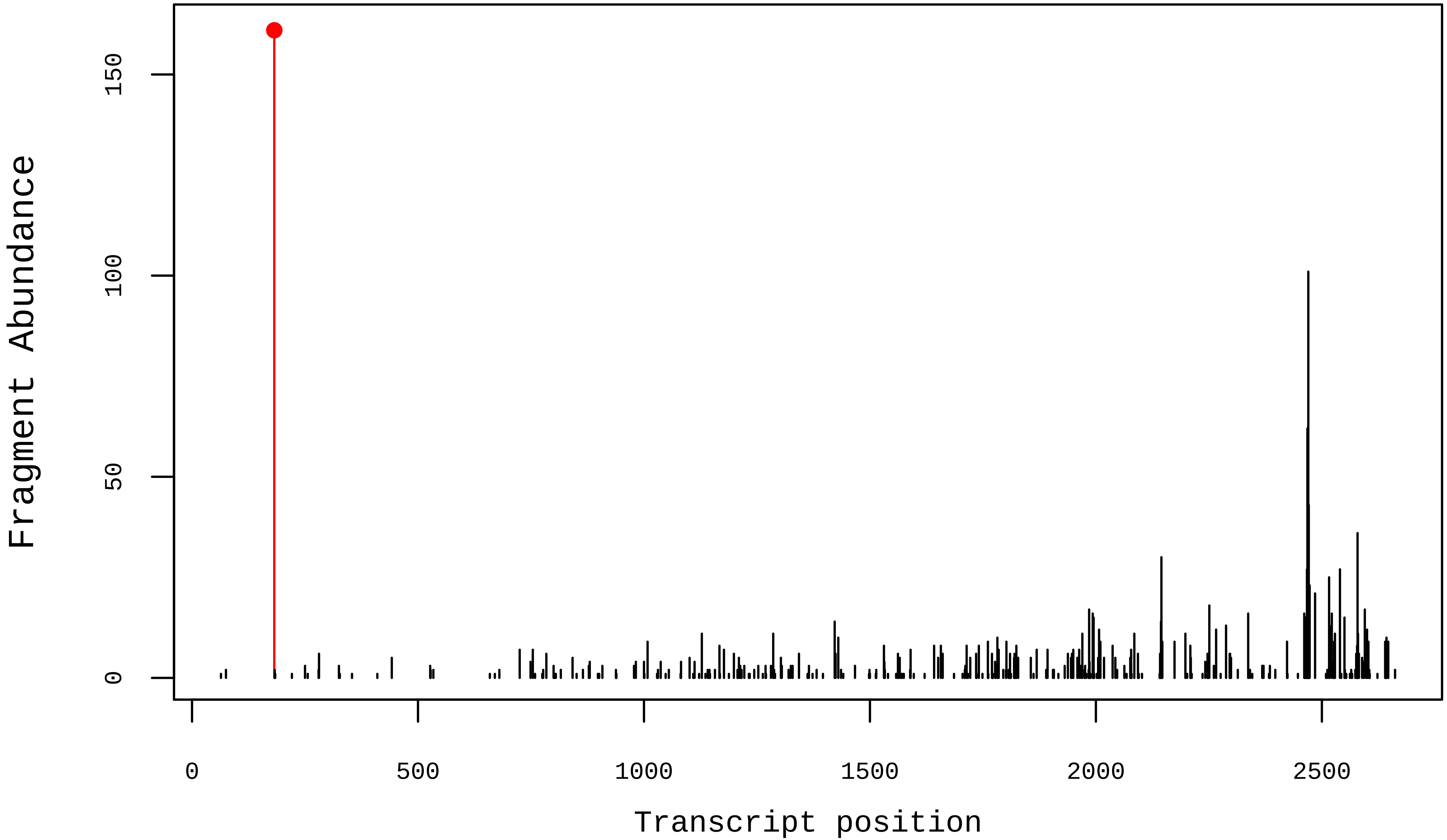

Cleavage site: 182 Tag abundance: 161 Weighted abundance: 9.471 Category: 0
sRNA abundance: 1 Alignment score: 2.5 MFE ratio: 0.803 p-value: 0.009

HORVU2Hr1G094690|HORVU2Hr1G094690.4||2112|2735

```
5' GGCCAGGTTTGCTGATGTTCATCTAACTAGCC '3
       |o||||||||||| ||||||||
3'     TTCAAACGACTACCAGTAGATT       '5
```

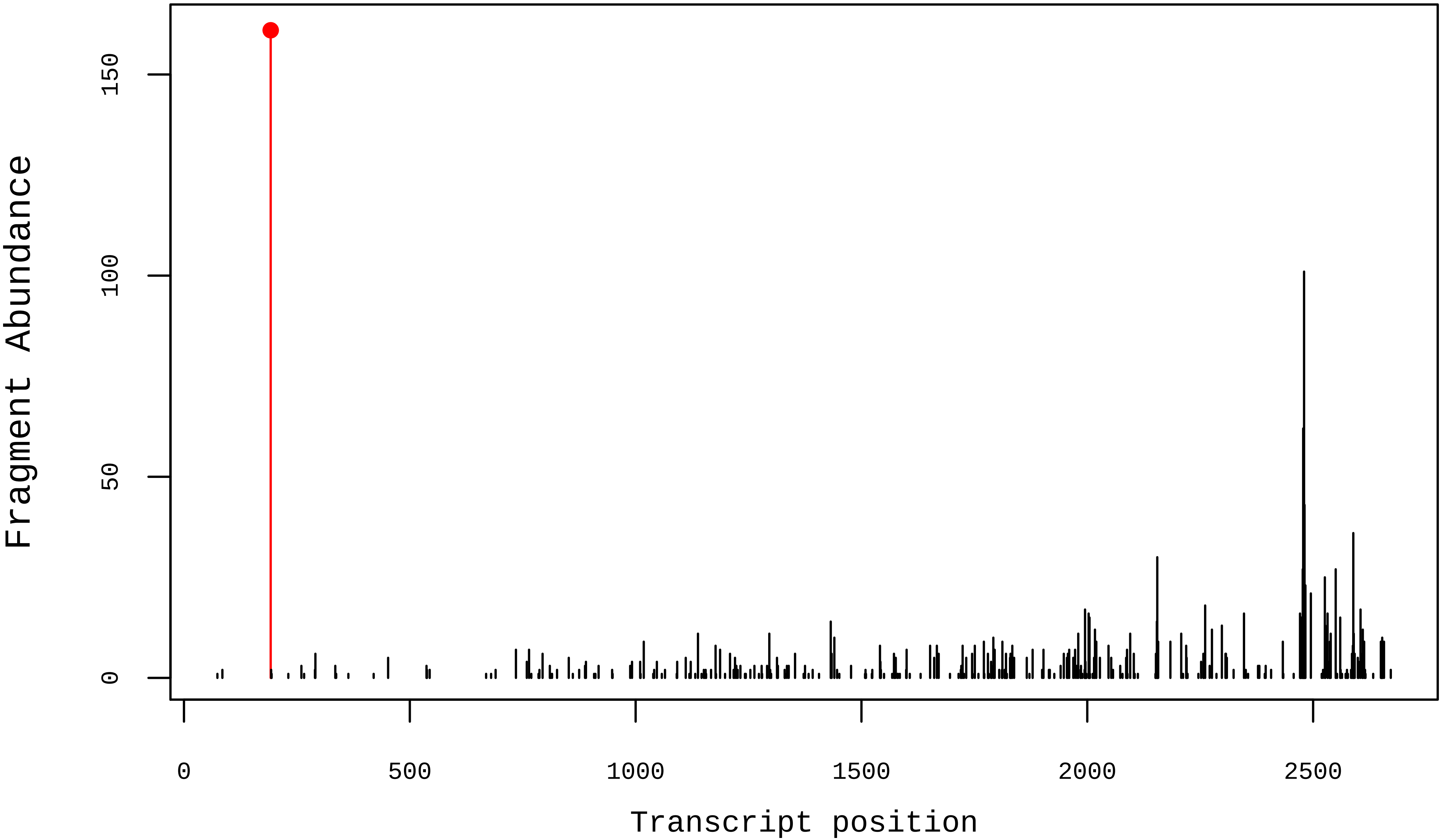

Cleavage site: 192   Tag abundance: 161   Weighted abundance: 9.471   Category: 0
sRNA abundance: 1   Alignment score: 2.5   MFE ratio: 0.803   p-value: 0.009

HORVU2Hr1G094690|HORVU2Hr1G094690.7||720|2747

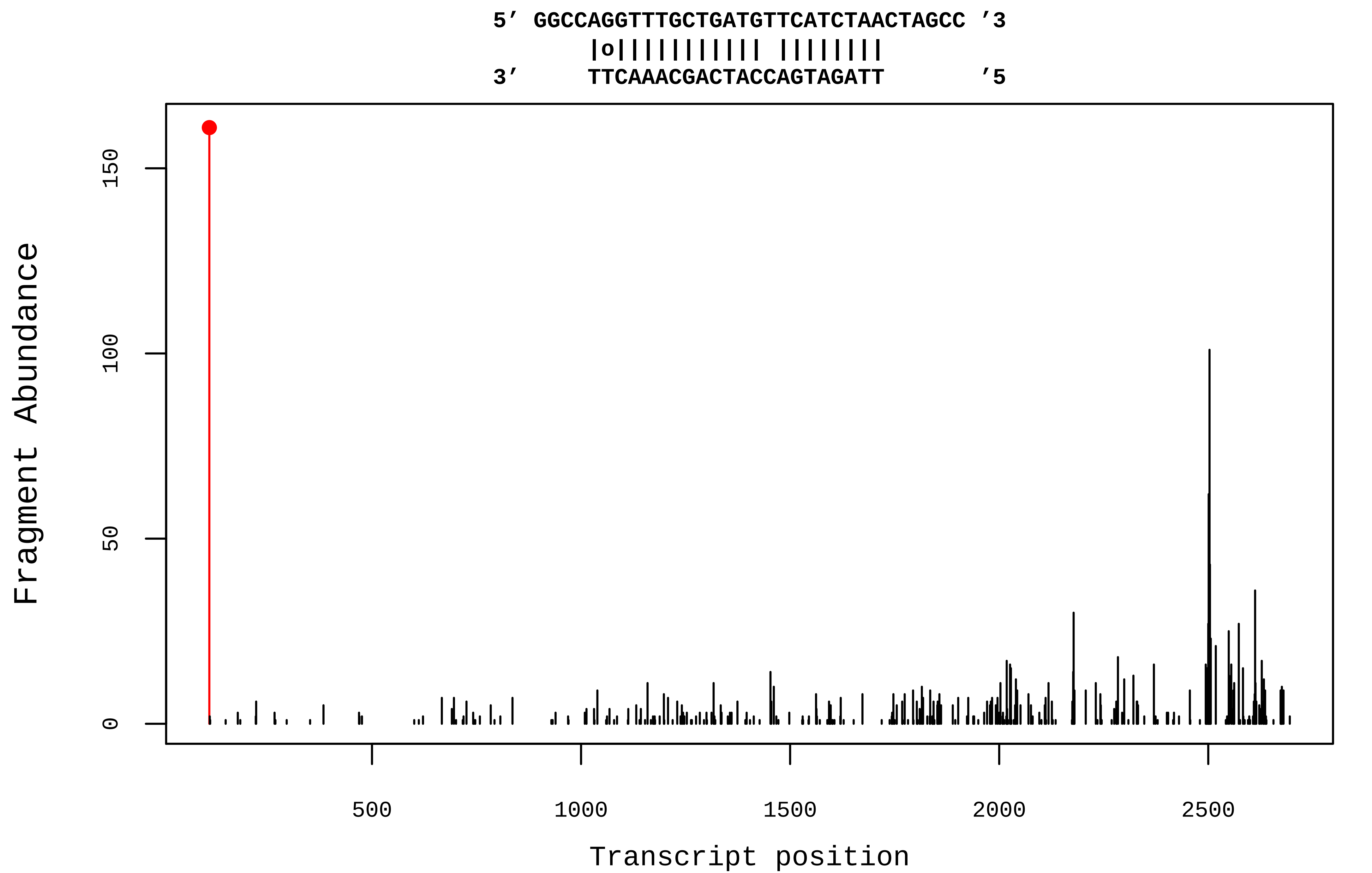

Cleavage site: 111 Tag abundance: 161 Weighted abundance: 9.471 Category: 0
sRNA abundance: 1 Alignment score: 2.5 MFE ratio: 0.803 p-value: 0.009

HORVU2Hr1G094690|HORVU2Hr1G094690.8||1458|2744

```
5' GGCCAGGTTTGCTGATGTTCATCTAACTAGCC '3
         |o||||||||||| ||||||||
3'       TTCAAACGACTACCAGTAGATT       '5
```

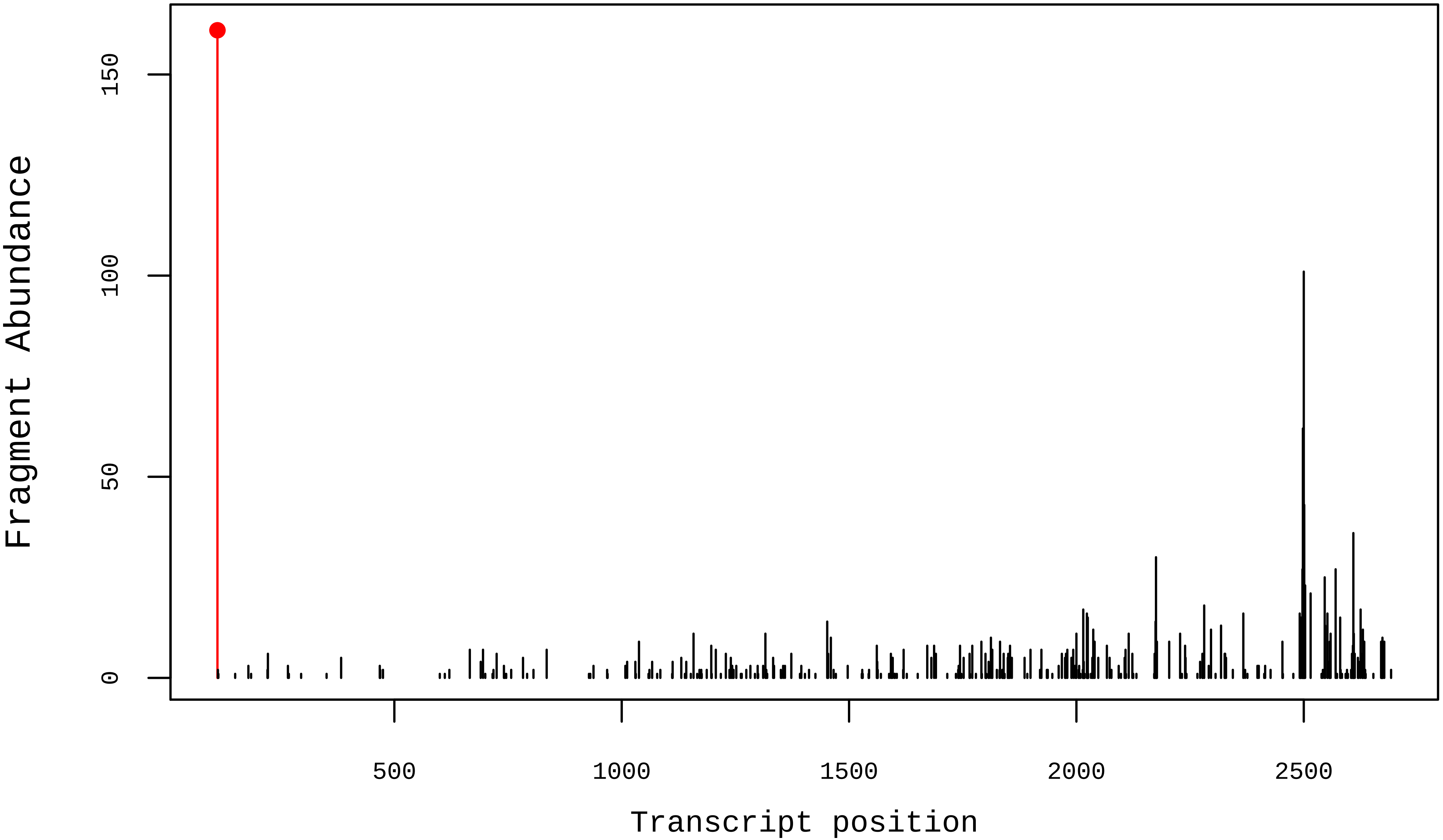

Cleavage site: 111   Tag abundance: 161   Weighted abundance: 9.471   Category: 0
sRNA abundance: 1   Alignment score: 2.5   MFE ratio: 0.803   p-value: 0.009

HORVU2Hr1G094690|HORVU2Hr1G094690.9||1227|2744

```
5' GGCCAGGTTTGCTGATGTTCATCTAACTAGCC '3
         |o|||||||||| ||||||||
3'       TTCAAACGACTACCAGTAGATT       '5
```

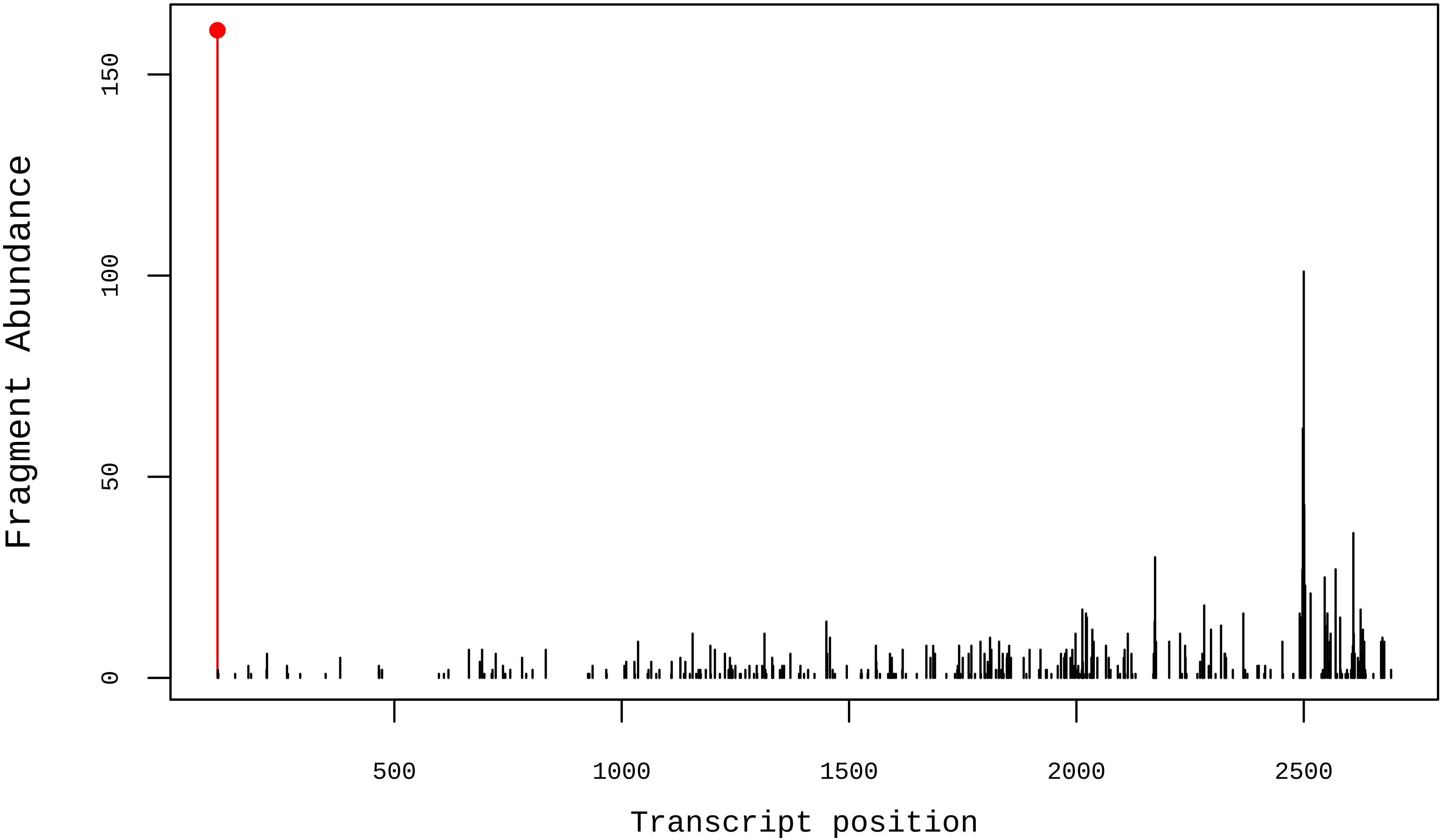

Cleavage site: 111   Tag abundance: 161   Weighted abundance: 9.471   Category: 0
sRNA abundance: 1   Alignment score: 2.5   MFE ratio: 0.803   p-value: 0.009

HORVU2Hr1G094690|HORVU2Hr1G094690.10||1458|2742

```
5' GGCCAG-GTTTGCTGATGTTCATCTAACTAGCC '3
       |o ||||||||||| ||||||||
3'     TTTCAAACGACTACCAGTAGATT       '5
```

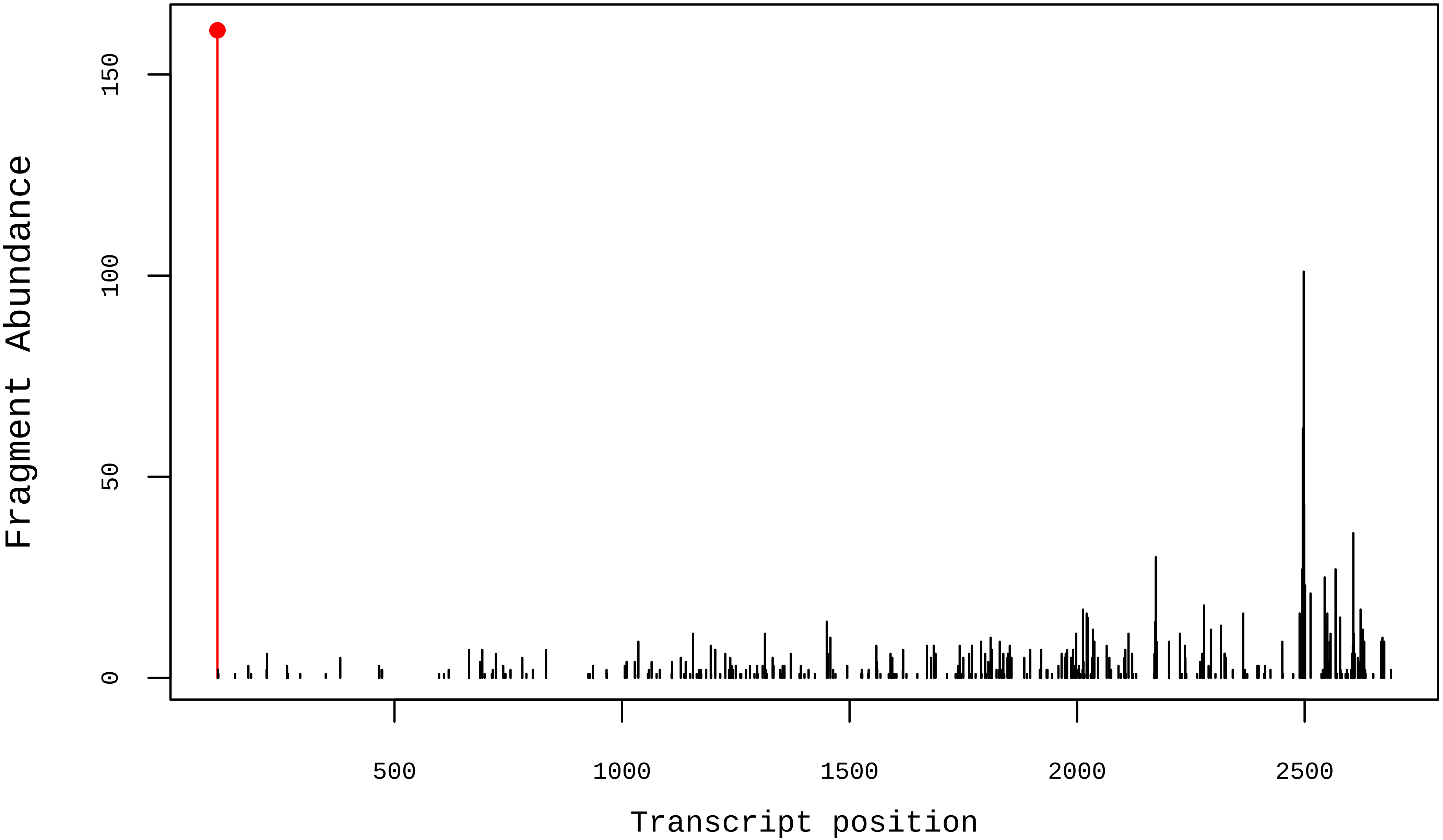

Cleavage site: 111   Tag abundance: 161   Weighted abundance: 9.471   Category: 0
sRNA abundance: 1   Alignment score: 3.5   MFE ratio: 0.747   p-value: 0.009

HORVU2Hr1G094690|HORVU2Hr1G094690.11||1458|2742

```
5' GGCCAG-GTTTGCTGATGTTCATCTAACTAGCC '3
          |o |||||||||||| |||||||||
3'        TTTCAAACGACTACCAGTAGATT        '5
```

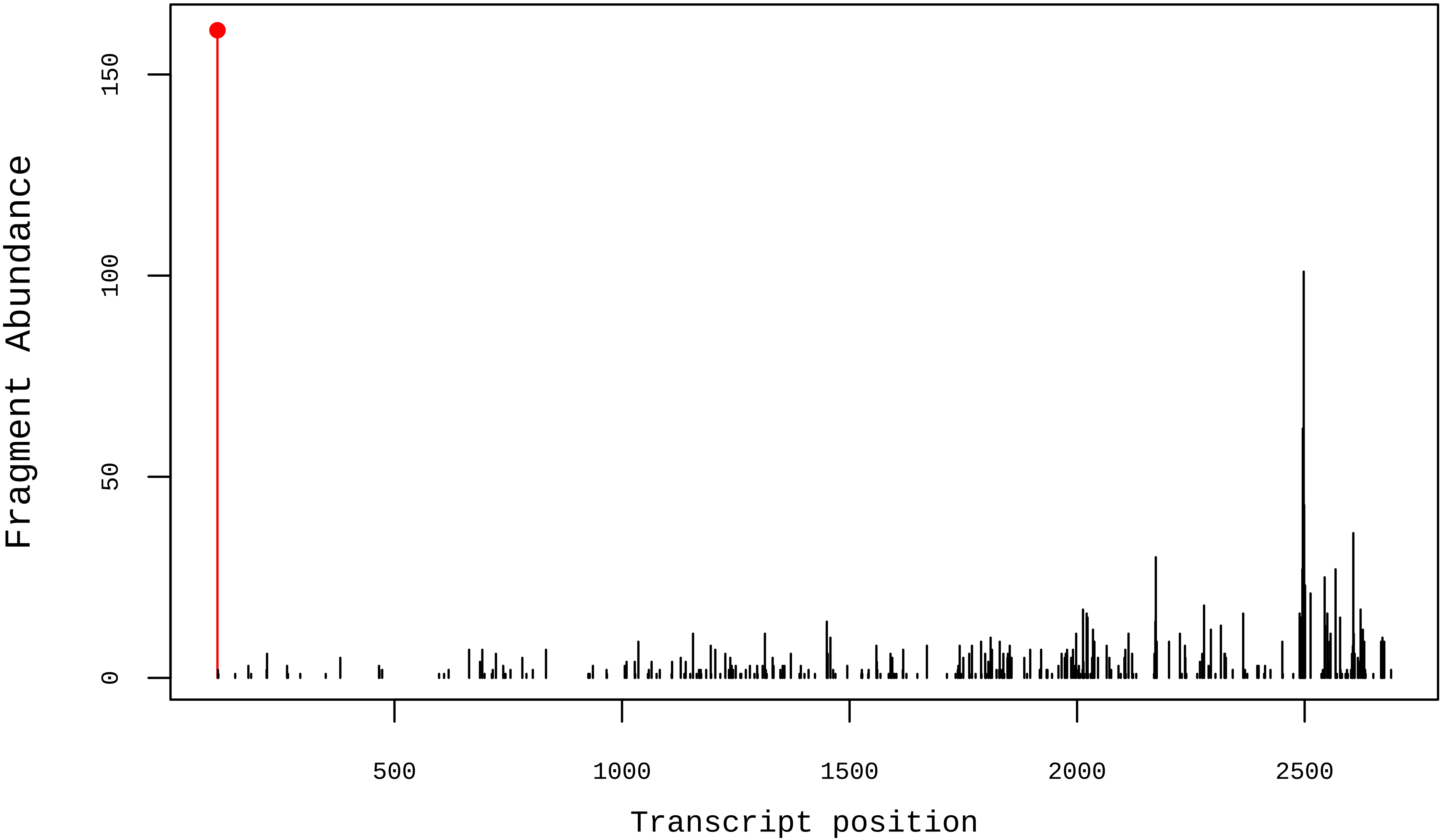

Cleavage site: 111   Tag abundance: 161   Weighted abundance: 9.471   Category: 0
sRNA abundance: 1   Alignment score: 3.5   MFE ratio: 0.747   p-value: 0.009

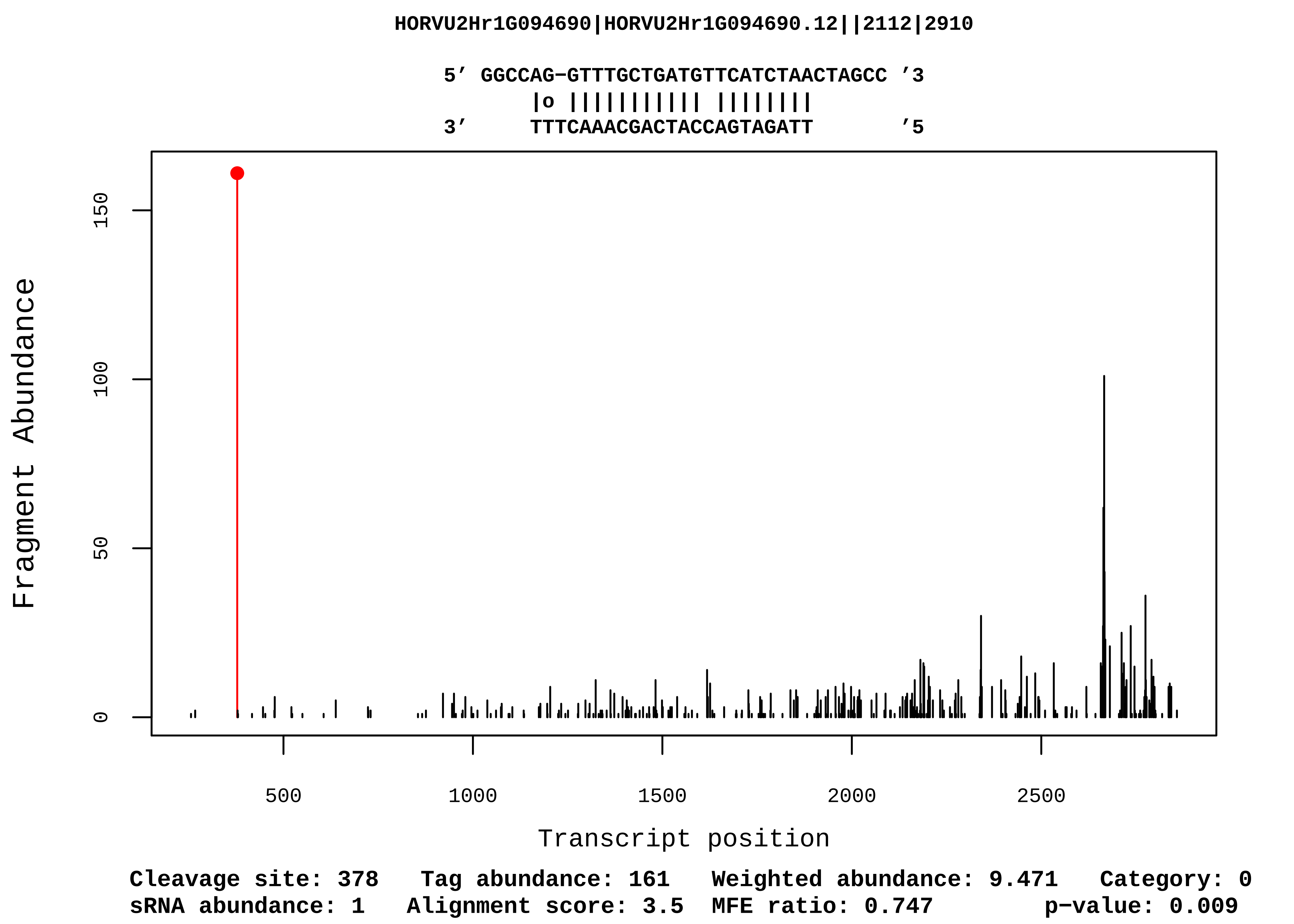
HORVU2Hr1G094690|HORVU2Hr1G094690.12||2112|2910
5' GGCCAG-GTTTGCTGATGTTCATCTAACTAGCC '3
|o ||||||||||| ||||||||
3' TTTCAAACGACTACCAGTAGATT '5
Fragment Abundance
0
50
100
150
500
1000
1500
2000
2500
Transcript position
Cleavage site: 378 Tag abundance: 161 Weighted abundance: 9.471 Category: 0
sRNA abundance: 1 Alignment score: 3.5 MFE ratio: 0.747 p-value: 0.009

HORVU2Hr1G094690|HORVU2Hr1G094690.13||2112|2916

```
5' GGCCAG-GTTTGCTGATGTTCATCTAACTAGCC '3
        |o ||||||||||| ||||||||
3'      TTTCAAACGACTACCAGTAGATT        '5
```

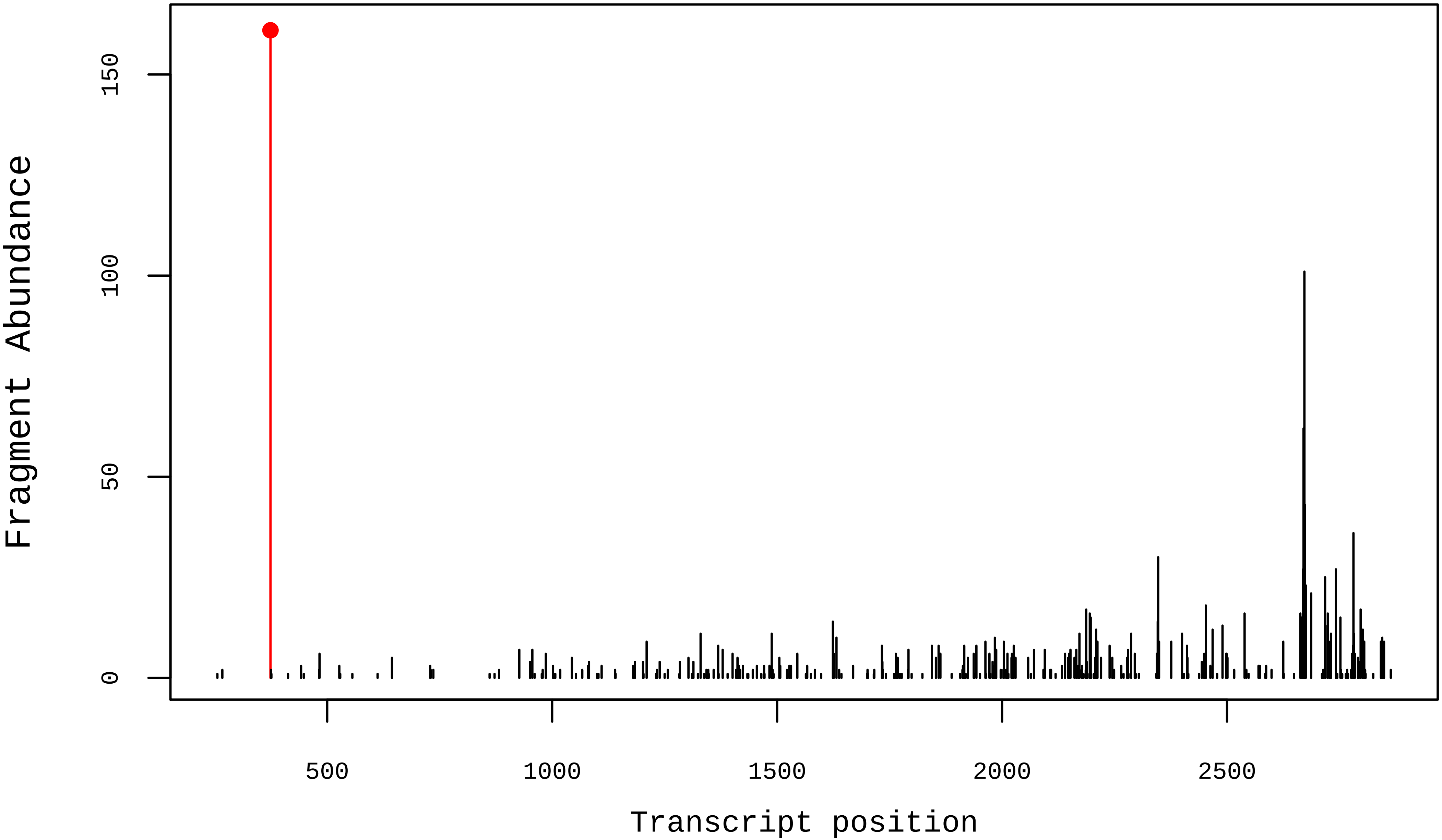

Cleavage site: 374   Tag abundance: 161   Weighted abundance: 9.471   Category: 0
sRNA abundance: 1   Alignment score: 3.5   MFE ratio: 0.747   p-value: 0.009

HORVU2Hr1G094690|HORVU2Hr1G094690.14||2112|2920

```
5' GGCCAG-GTTTGCTGATGTTCATCTAACTAGCC '3
       |o ||||||||||| ||||||||
3'     TTTCAAACGACTACCAGTAGATT       '5
```

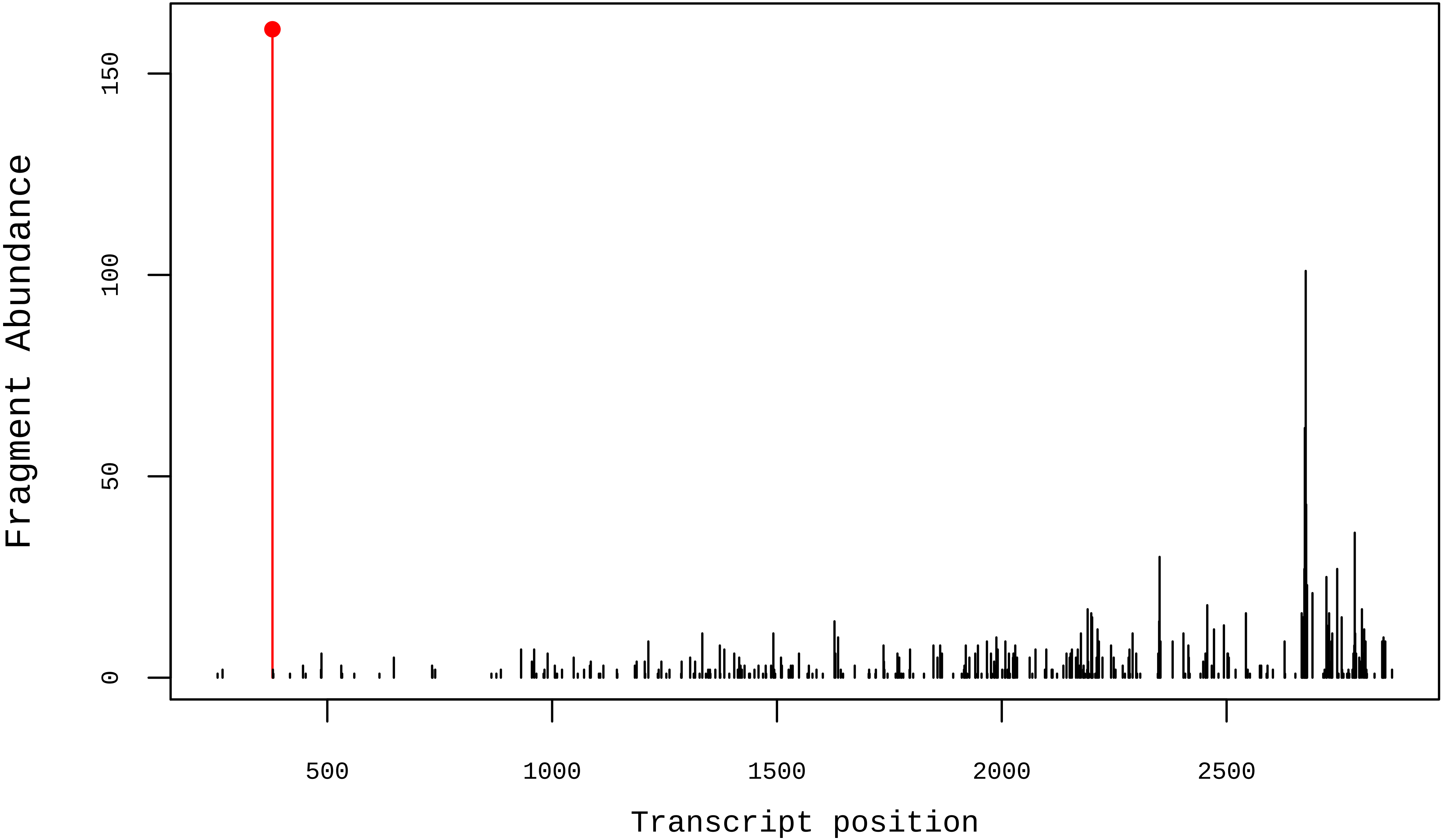

Cleavage site: 378 Tag abundance: 161 Weighted abundance: 9.471 Category: 0
sRNA abundance: 1 Alignment score: 3.5 MFE ratio: 0.747 p-value: 0.009

HORVU2Hr1G094690|HORVU2Hr1G094690.15||2112|2920

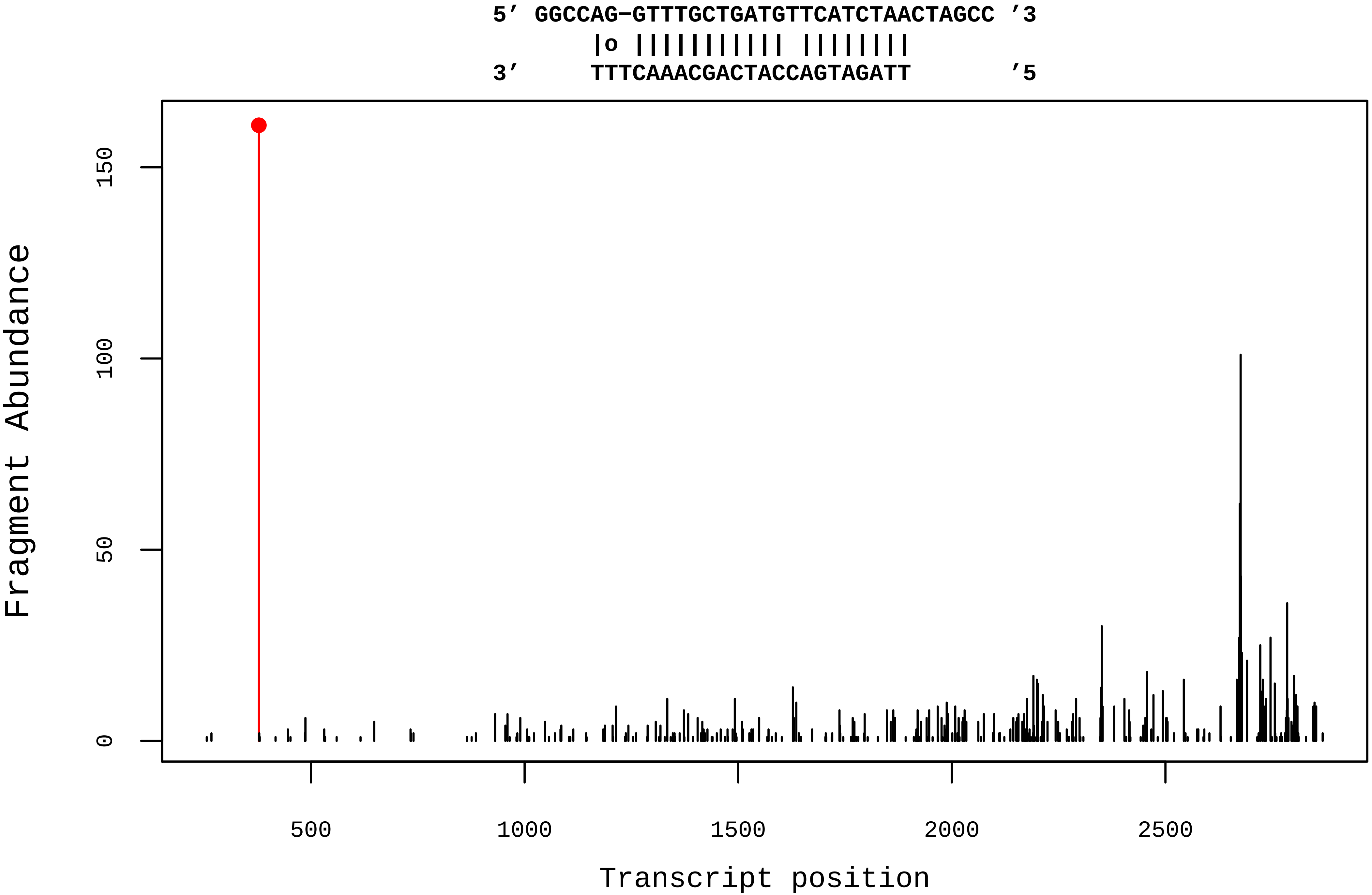

Cleavage site: 378 Tag abundance: 161 Weighted abundance: 9.471 Category: 0
sRNA abundance: 1 Alignment score: 3.5 MFE ratio: 0.747 p−value: 0.009

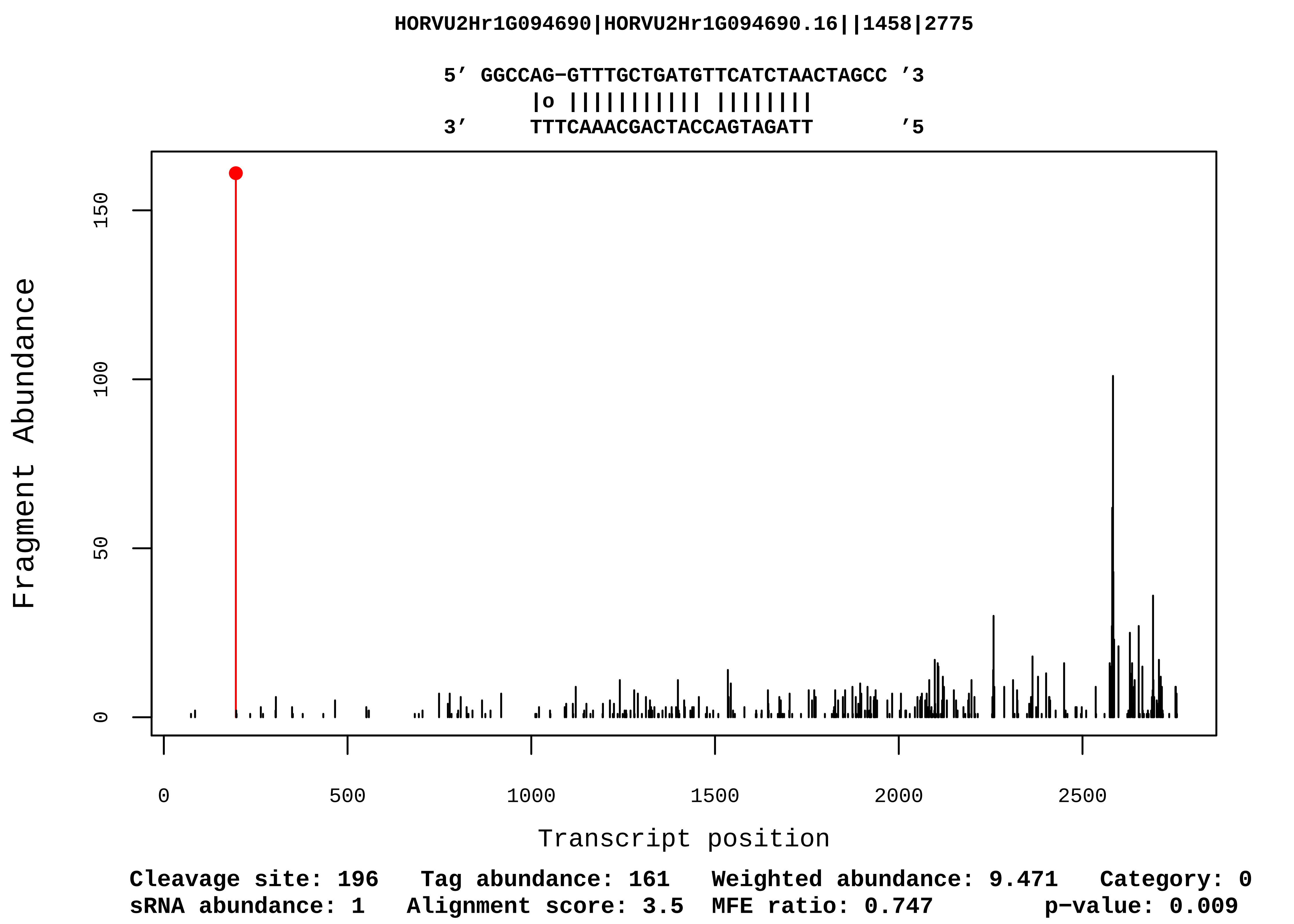
HORVU2Hr1G094690|HORVU2Hr1G094690.16||1458|2775
5′ GGCCAG−GTTTGCTGATGTTCATCTAACTAGCC ′3
|o ||||||||||| ||||||||
3′ TTTCAAACGACTACCAGTAGATT ′5
Fragment Abundance
0
50
100
150
0
500
1000
1500
2000
2500
Transcript position
Cleavage site: 196 Tag abundance: 161 Weighted abundance: 9.471 Category: 0
sRNA abundance: 1 Alignment score: 3.5 MFE ratio: 0.747 p−value: 0.009

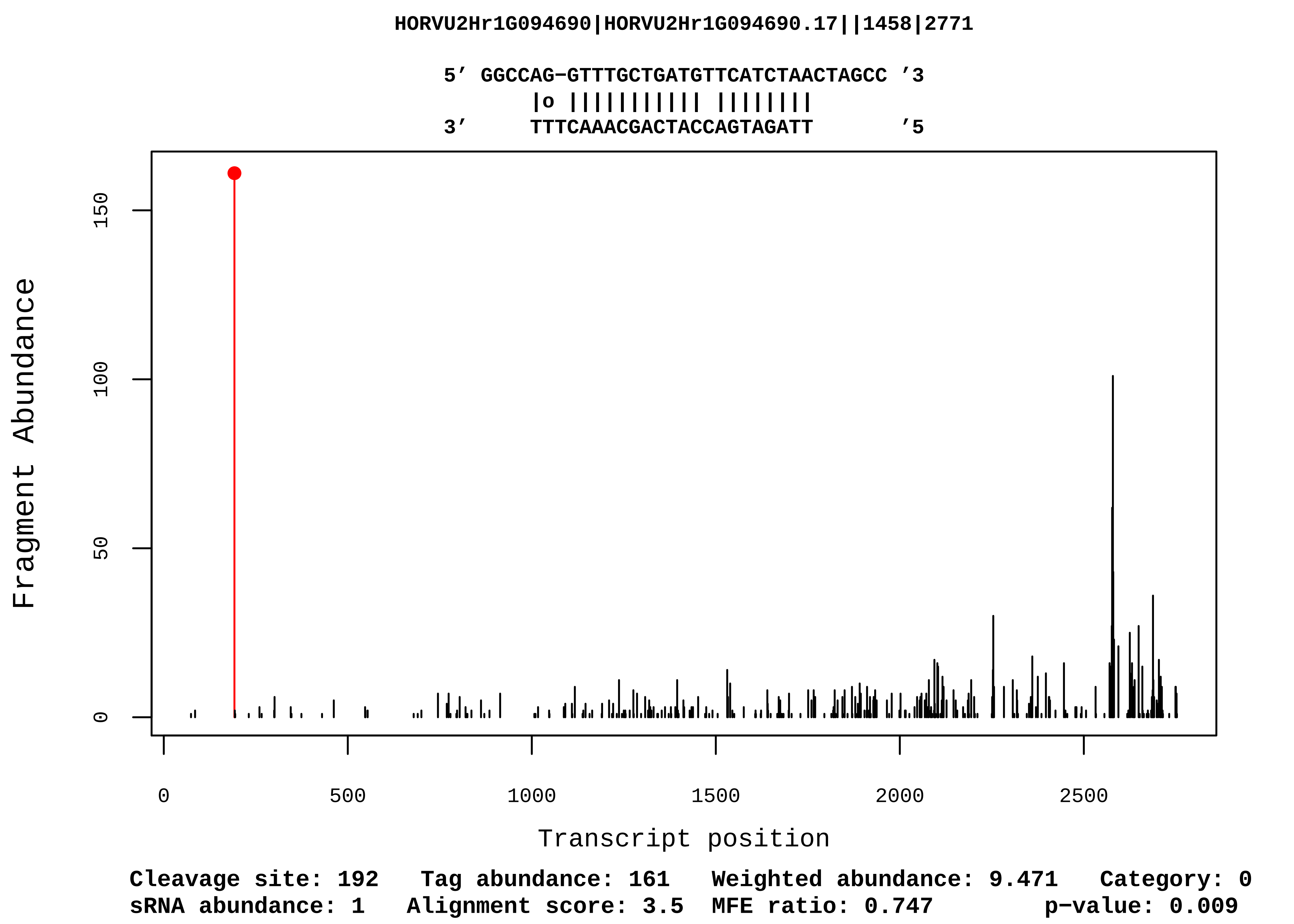
HORVU2Hr1G094690|HORVU2Hr1G094690.17||1458|2771
5′ GGCCAG−GTTTGCTGATGTTCATCTAACTAGCC ′3
|o ||||||||||| ||||||||
3′ TTTCAAACGACTACCAGTAGATT ′5
Fragment Abundance
0
50
100
150
0
500
1000
1500
2000
2500
Transcript position
Cleavage site: 192 Tag abundance: 161 Weighted abundance: 9.471 Category: 0
sRNA abundance: 1 Alignment score: 3.5 MFE ratio: 0.747 p−value: 0.009

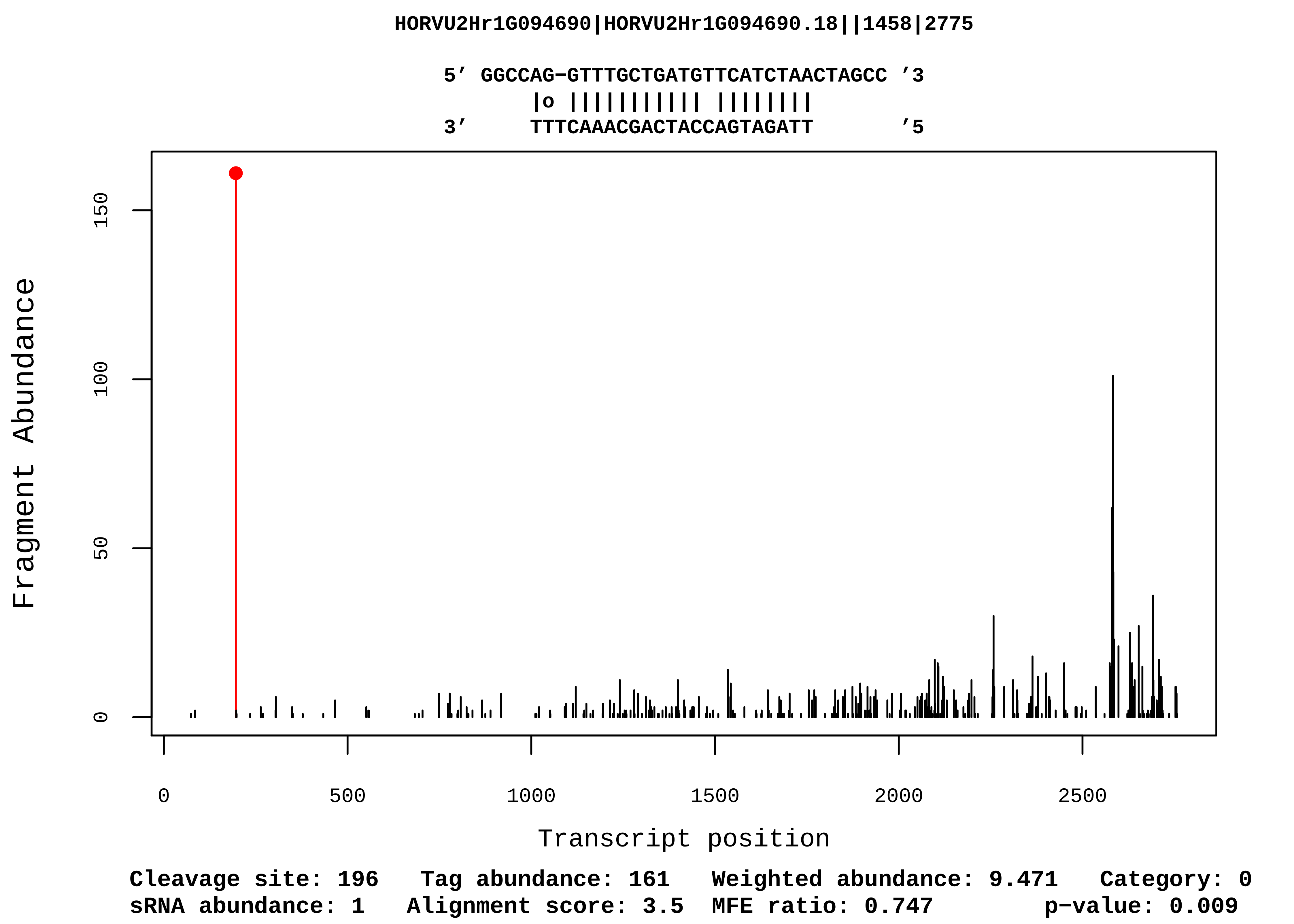
HORVU2Hr1G094690|HORVU2Hr1G094690.18||1458|2775
5' GGCCAG-GTTTGCTGATGTTCATCTAACTAGCC '3
|o ||||||||||| ||||||||
3' TTTCAAACGACTACCAGTAGATT '5
Fragment Abundance
0
50
100
150
0
500
1000
1500
2000
2500
Transcript position
Cleavage site: 196 Tag abundance: 161 Weighted abundance: 9.471 Category: 0
sRNA abundance: 1 Alignment score: 3.5 MFE ratio: 0.747 p-value: 0.009

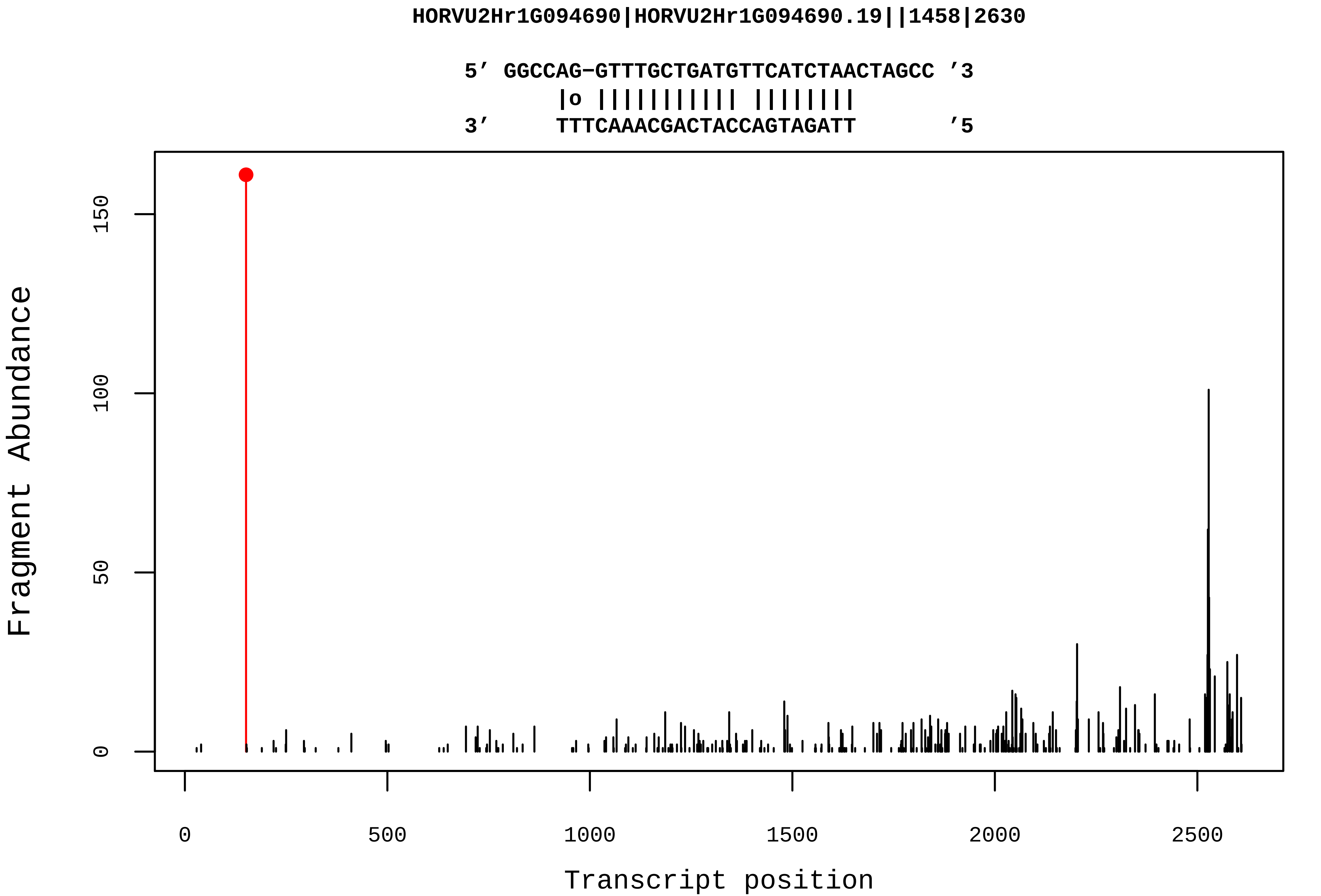

Cleavage site: 151 Tag abundance: 161 Weighted abundance: 9.471 Category: 0
sRNA abundance: 1 Alignment score: 3.5 MFE ratio: 0.747 p-value: 0.01

HORVU2Hr1G094690|HORVU2Hr1G094690.1||2112|2767

```
5′ GGCCAG−GTTTGCTGATGTTCATCTAACTAGCC ′3
       |o ||||||||||| ||||||||
3′     TTTCAAACGACTACCAGTAGATT        ′5
```

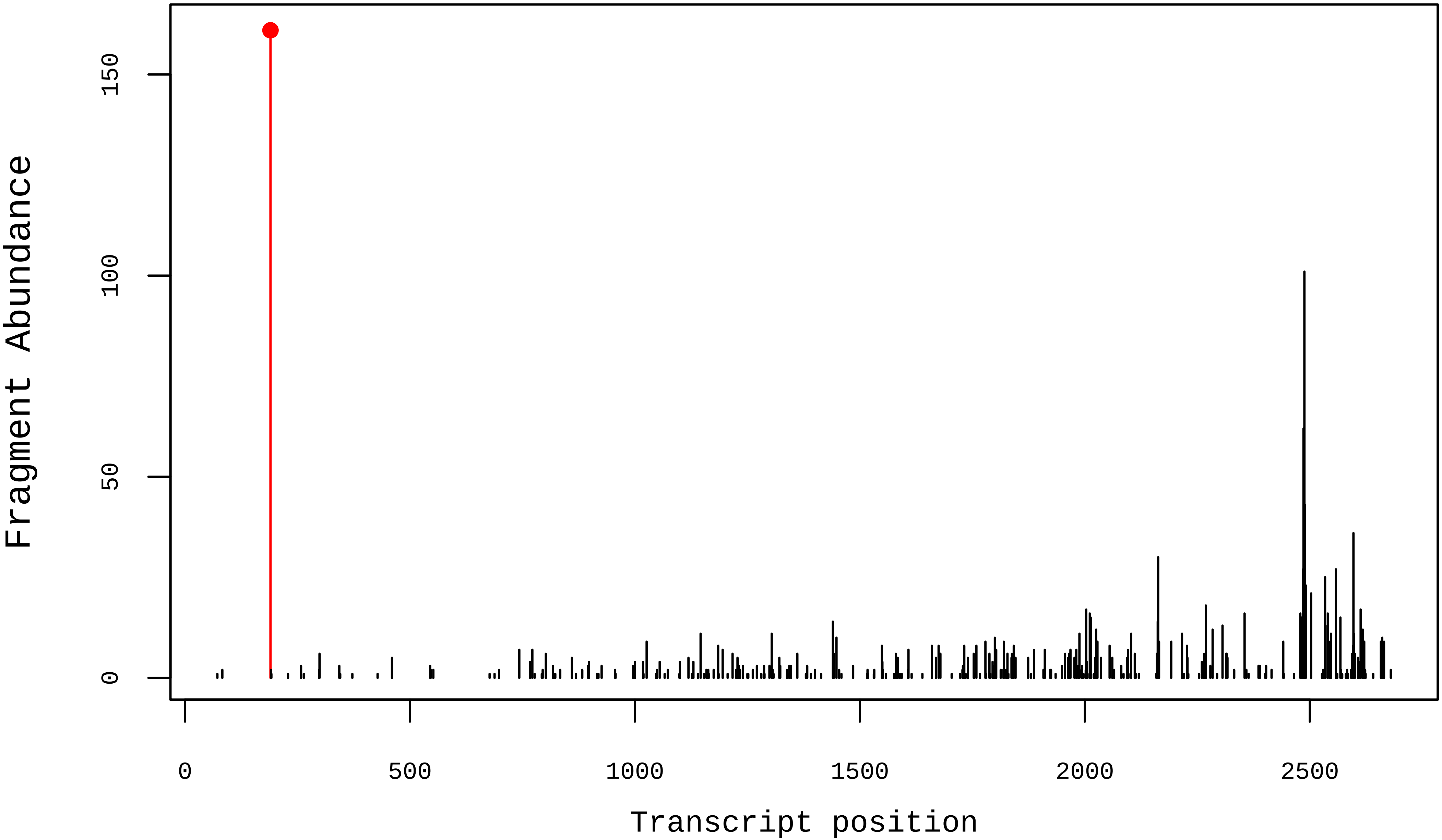

Cleavage site: 190 Tag abundance: 161 Weighted abundance: 9.471 Category: 0
sRNA abundance: 1 Alignment score: 3.5 MFE ratio: 0.747 p-value: 0.009

HORVU2Hr1G094690|HORVU2Hr1G094690.2||2112|2761

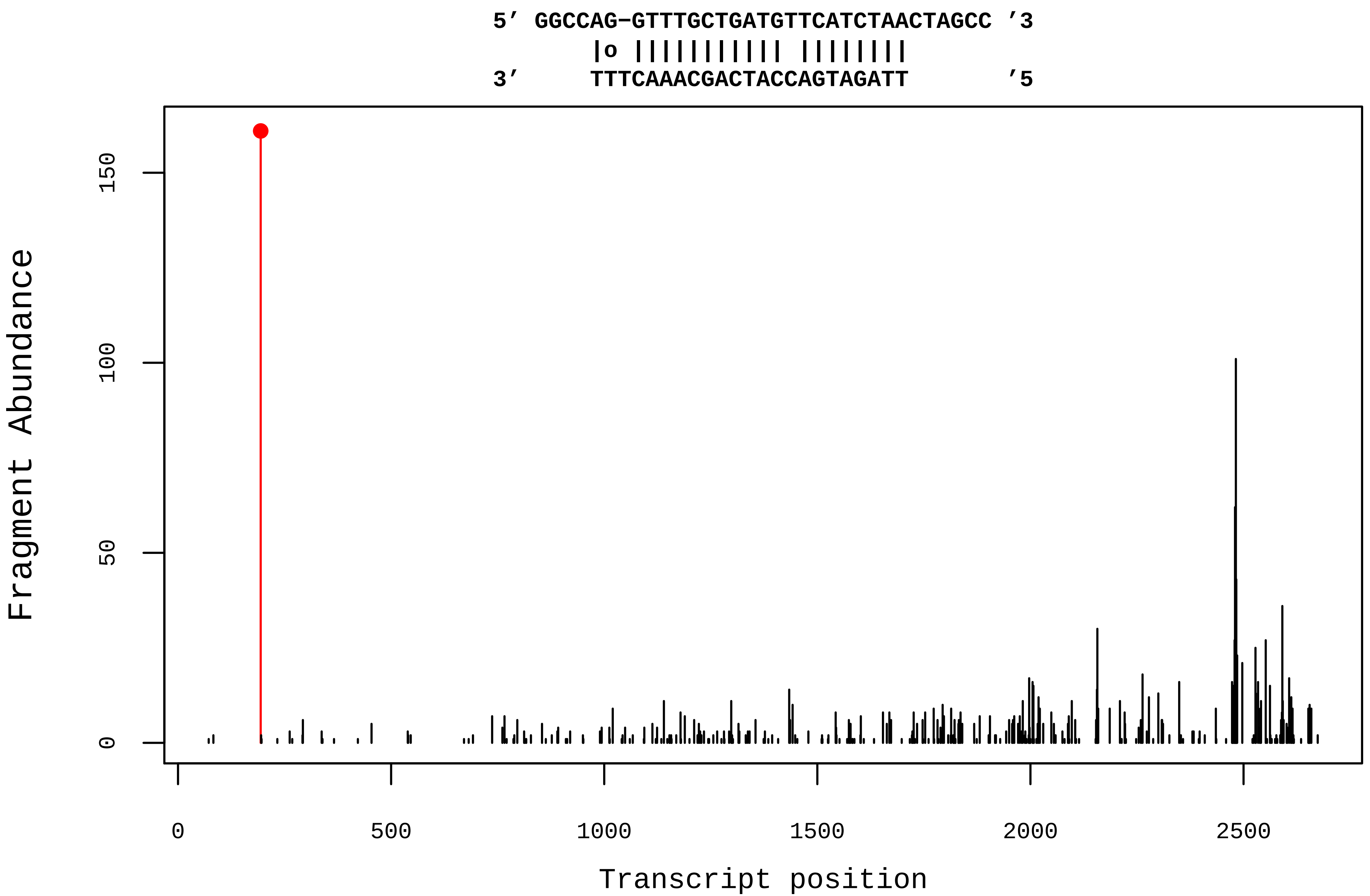

Cleavage site: 194 Tag abundance: 161 Weighted abundance: 9.471 Category: 0
sRNA abundance: 1 Alignment score: 3.5 MFE ratio: 0.747 p-value: 0.009

HORVU2Hr1G094690|HORVU2Hr1G094690.3||2112|2734

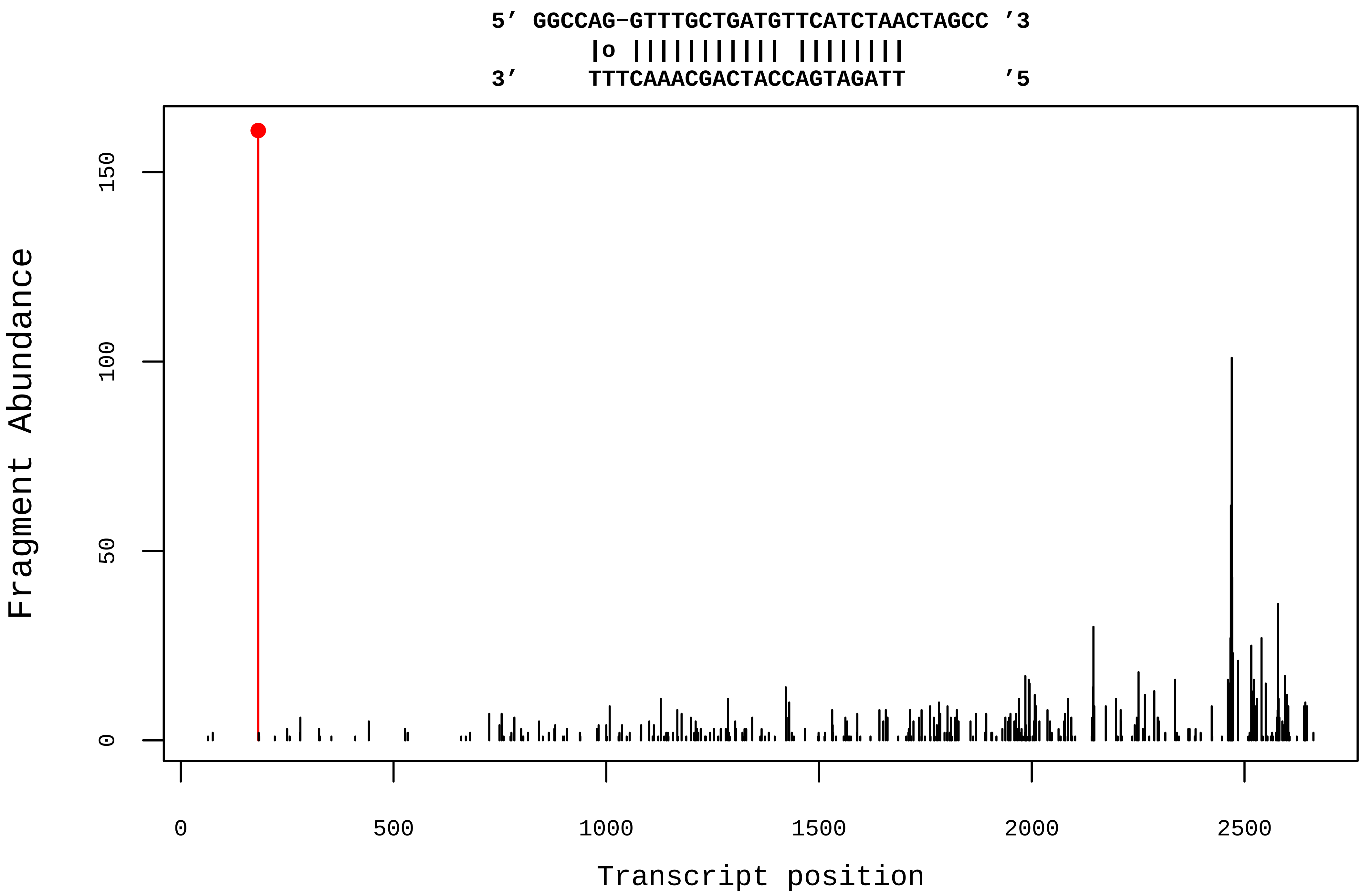

Cleavage site: 182 Tag abundance: 161 Weighted abundance: 9.471 Category: 0
sRNA abundance: 1 Alignment score: 3.5 MFE ratio: 0.747 p−value: 0.009

HORVU2Hr1G094690|HORVU2Hr1G094690.4||2112|2735

```
5′  GGCCAG-GTTTGCTGATGTTCATCTAACTAGCC ′3
          |o ||||||||||| ||||||||
3′        TTTCAAACGACTACCAGTAGATT        ′5
```

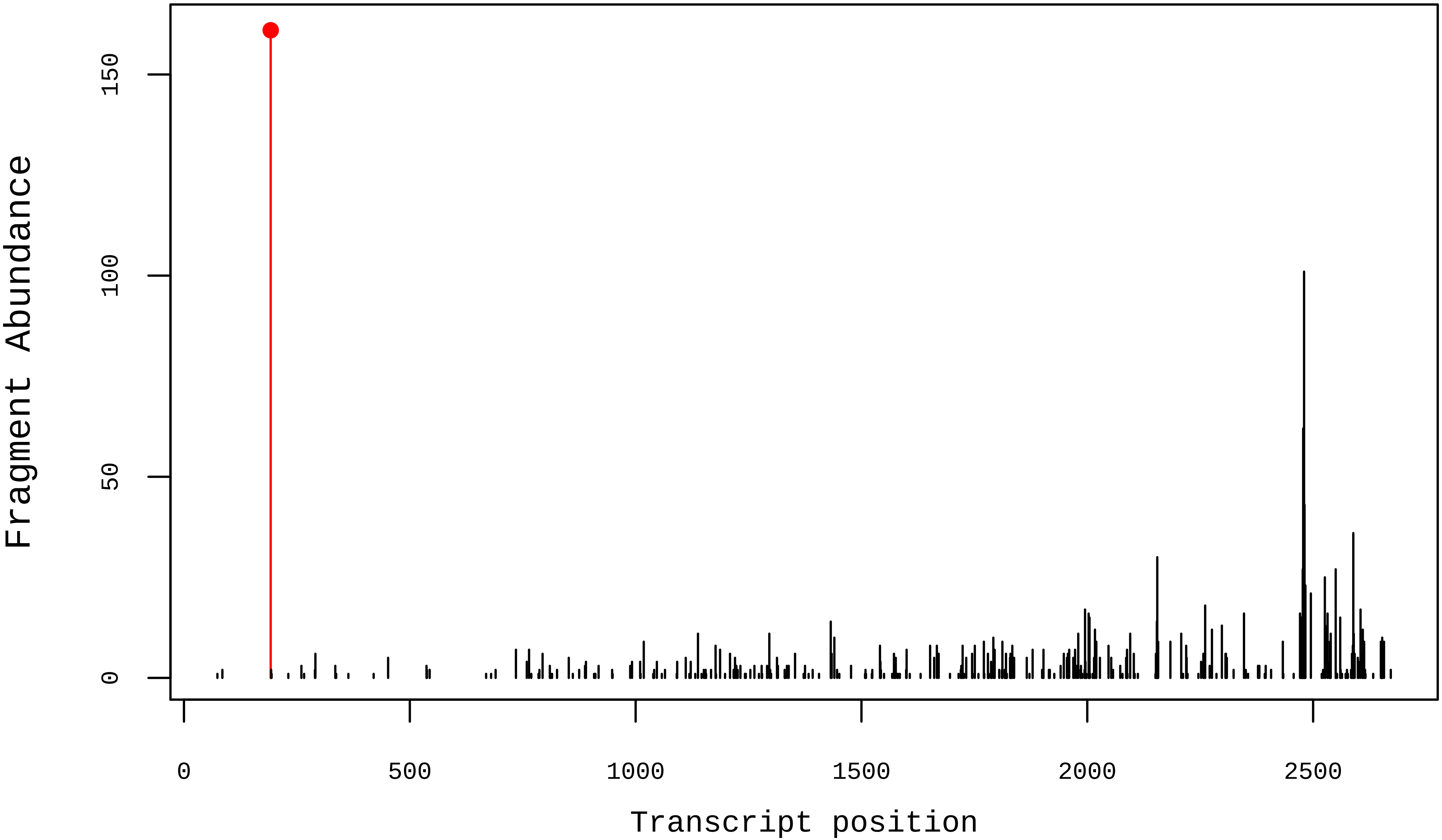

Cleavage site: 192   Tag abundance: 161   Weighted abundance: 9.471   Category: 0
sRNA abundance: 1   Alignment score: 3.5   MFE ratio: 0.747   p-value: 0.009

HORVU2Hr1G094690|HORVU2Hr1G094690.7||720|2747

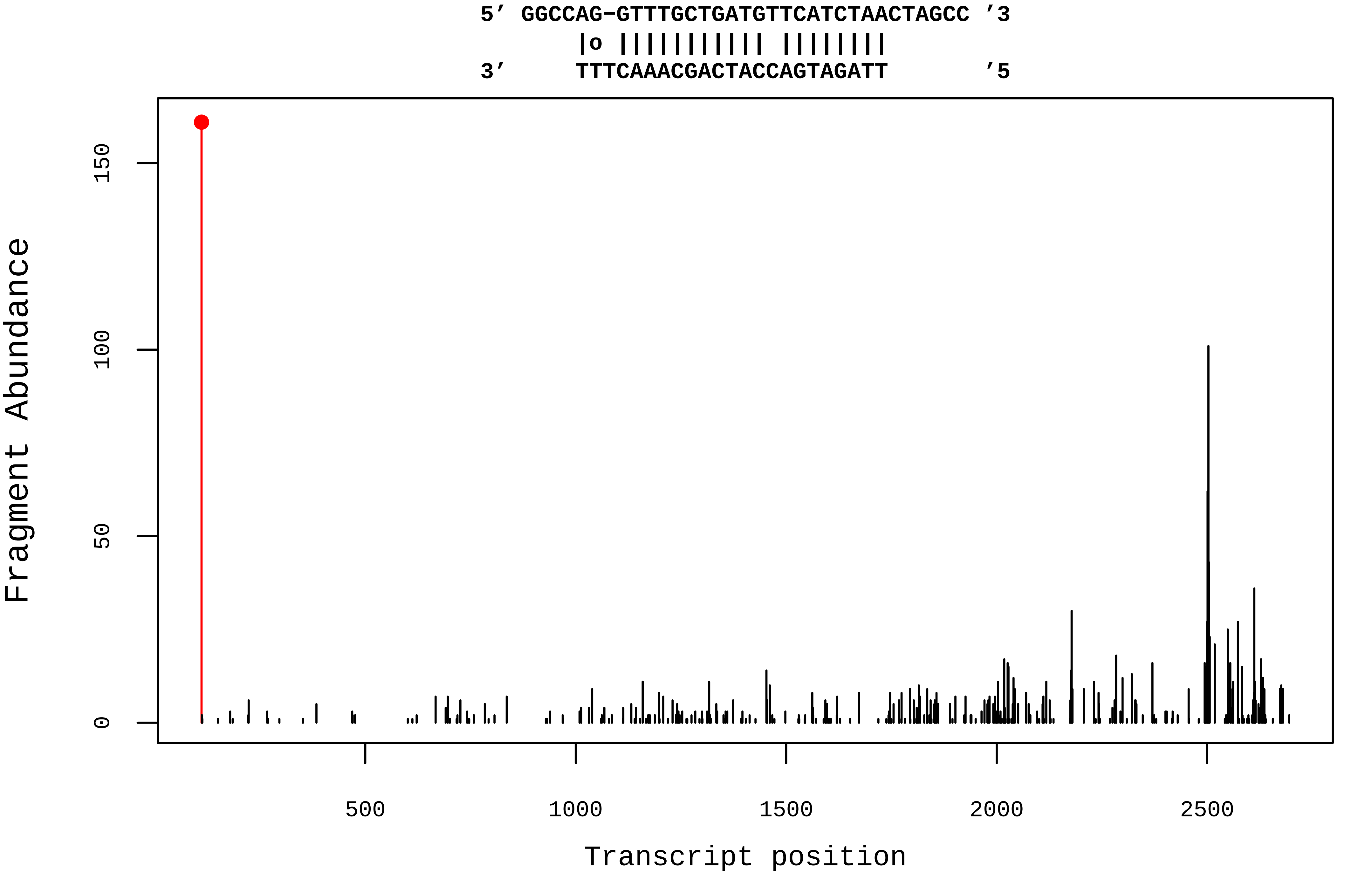

Cleavage site: 111 Tag abundance: 161 Weighted abundance: 9.471 Category: 0
sRNA abundance: 1 Alignment score: 3.5 MFE ratio: 0.747 p-value: 0.009

HORVU2Hr1G094690|HORVU2Hr1G094690.8||1458|2744

```
5' GGCCAG-GTTTGCTGATGTTCATCTAACTAGCC '3
       |o ||||||||||| ||||||||
3'     TTTCAAACGACTACCAGTAGATT       '5
```

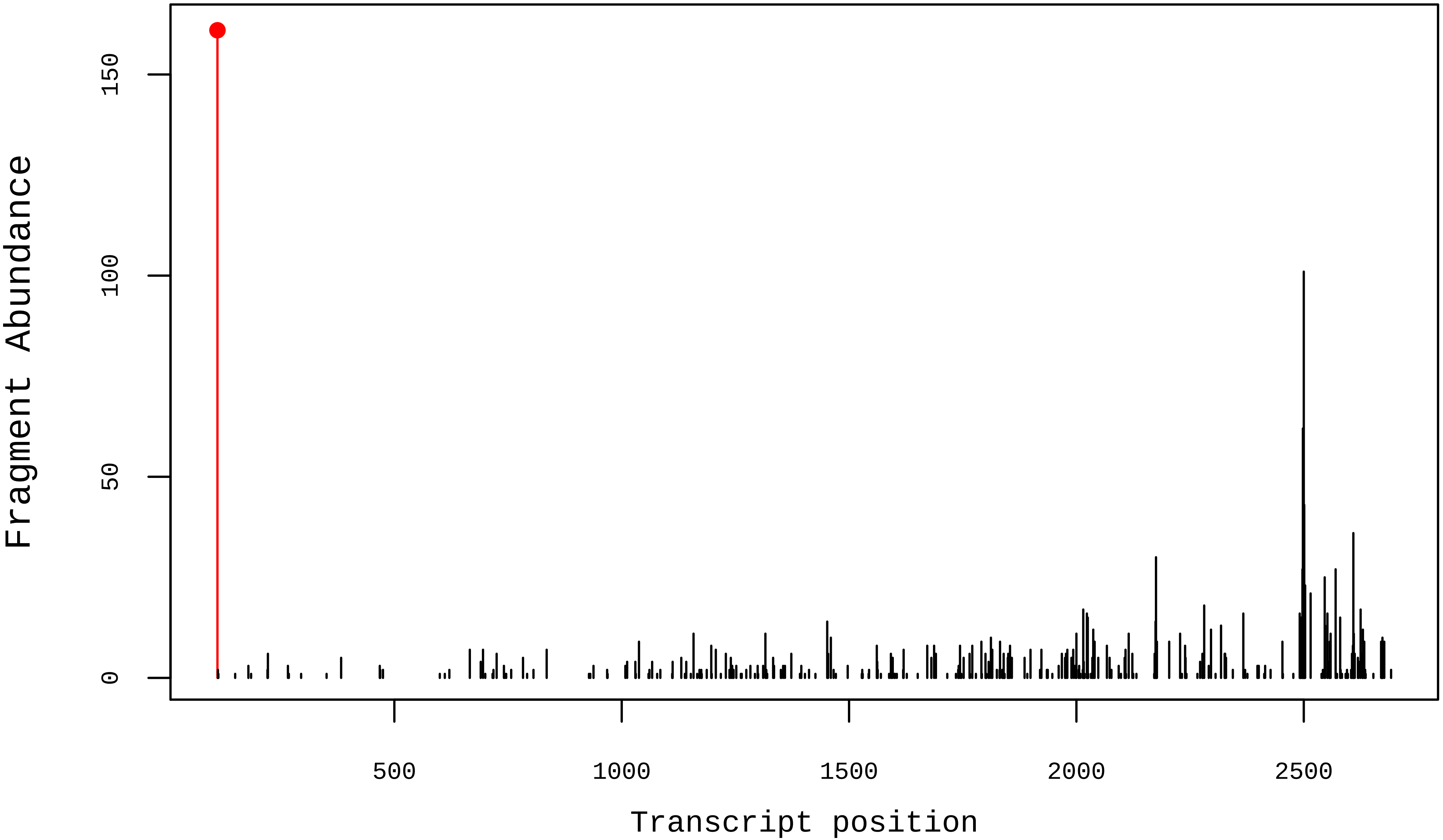

Cleavage site: 111 Tag abundance: 161 Weighted abundance: 9.471 Category: 0
sRNA abundance: 1 Alignment score: 3.5 MFE ratio: 0.747 p-value: 0.009

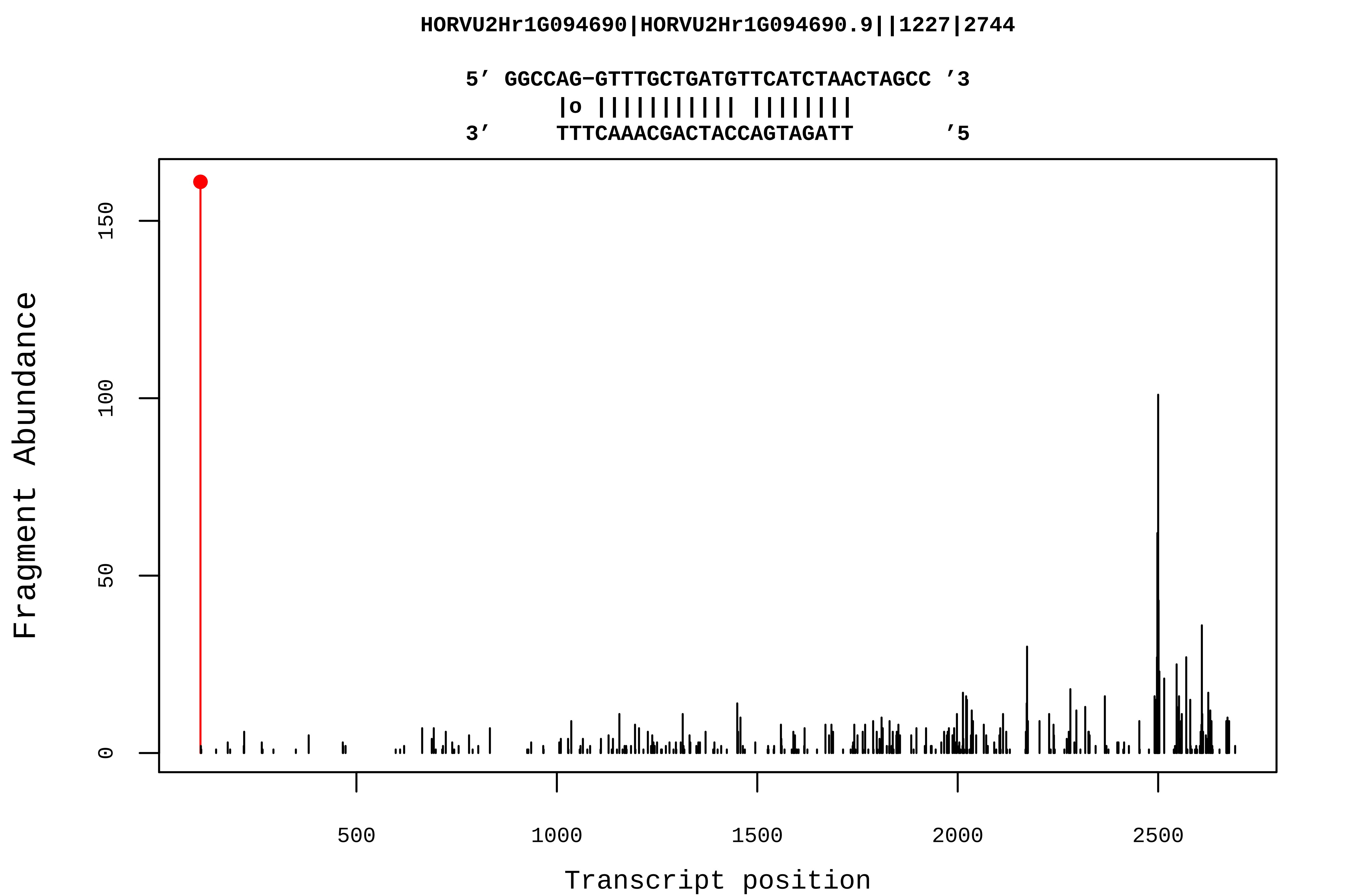

Cleavage site: 111 Tag abundance: 161 Weighted abundance: 9.471 Category: 0
sRNA abundance: 1 Alignment score: 3.5 MFE ratio: 0.747 p-value: 0.009

HORVU2Hr1G045000|HORVU2Hr1G045000.1||468|1186

```
5' ACCATCACGATGACGGCAGATCCCCTTTAATA '3
      ||||||||o||||| |||||||||o
3'    GTAGTGCTGCTGCCATCTAGGGGG       '5
```

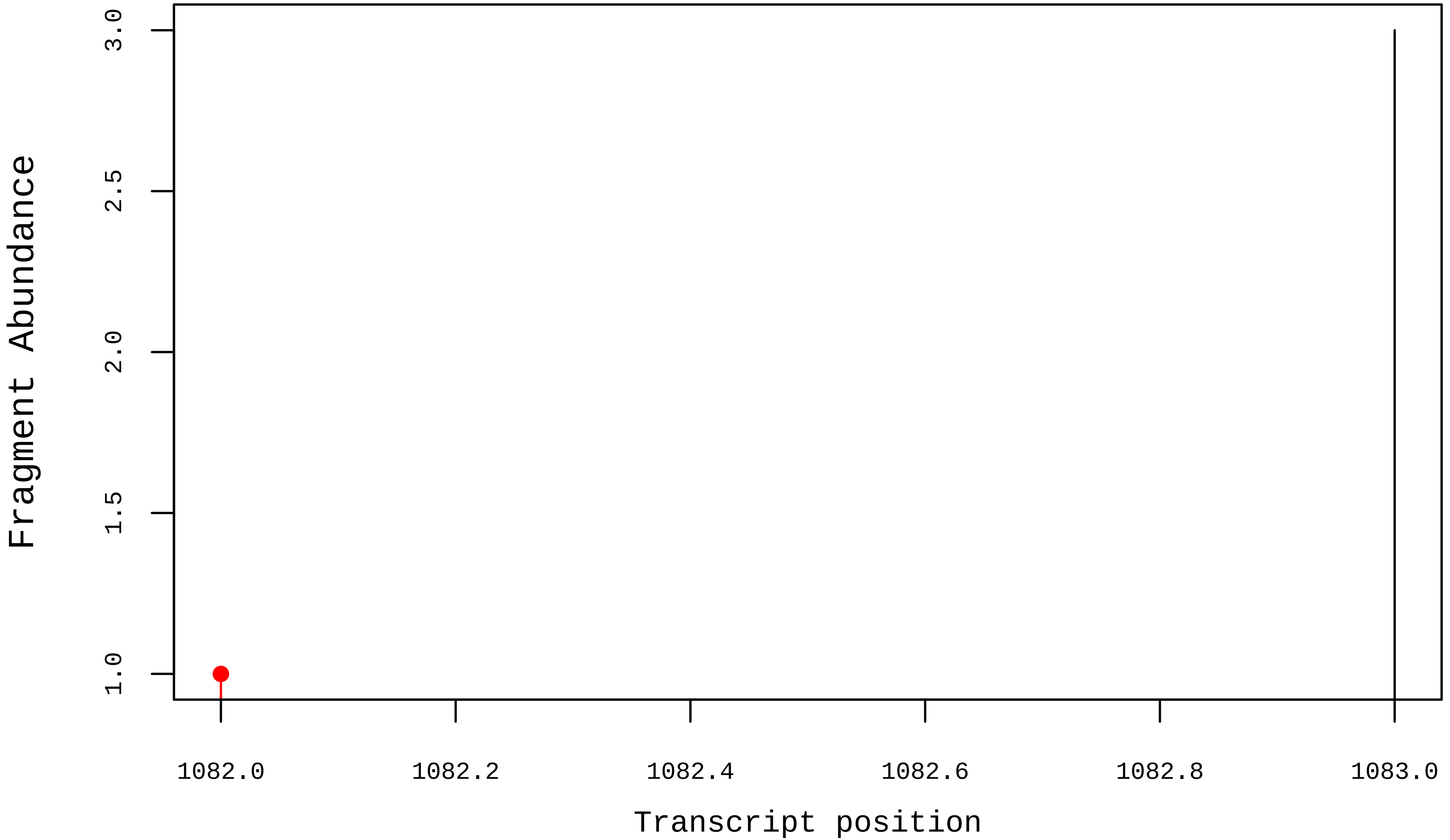

Cleavage site: 1082   Tag abundance: 1   Weighted abundance: 0.037   Category: 4
sRNA abundance: 1   Alignment score: 3   MFE ratio: 0.837   p-value: 0.001
