# Supplementary material for: Pi-starvation induced transcriptional changes in barley revealed by a comprehensive RNA-Seq and degradome analyses
Source: BMC Genomics. 2021 Mar 9;22:165. doi: 10.1186/s12864-021-07481-w (PMC7941915; doi:10.1186/s12864-021-07481-w)
Supplement: Supplementary file 24 — Additional file 24. The t-plots generated by PAREsnip2 software showing the potential mRNA targets for differentially expressed miRNAs (DEMs) identified in barley roots (low-Pi vs. control). [file 12864_2021_7481_MOESM24_ESM.pdf]

Additional file 24. The t-plots generated by PAREsnip2 software showing the potential mRNA targets for differentially expressed miRNAs (DEMs) identified in barley roots (low--Pi vs. control).

HORVU1Hr1G085570|HORVU1Hr1G085570.3||1335|2651

```
5' GCTATCTGGGCAAATCTCCTTTGGCGGTTACT '3
          | |||||| |||||||||||o
3'        GTCCCGTTGAGAGGAAACCGT        '5
```

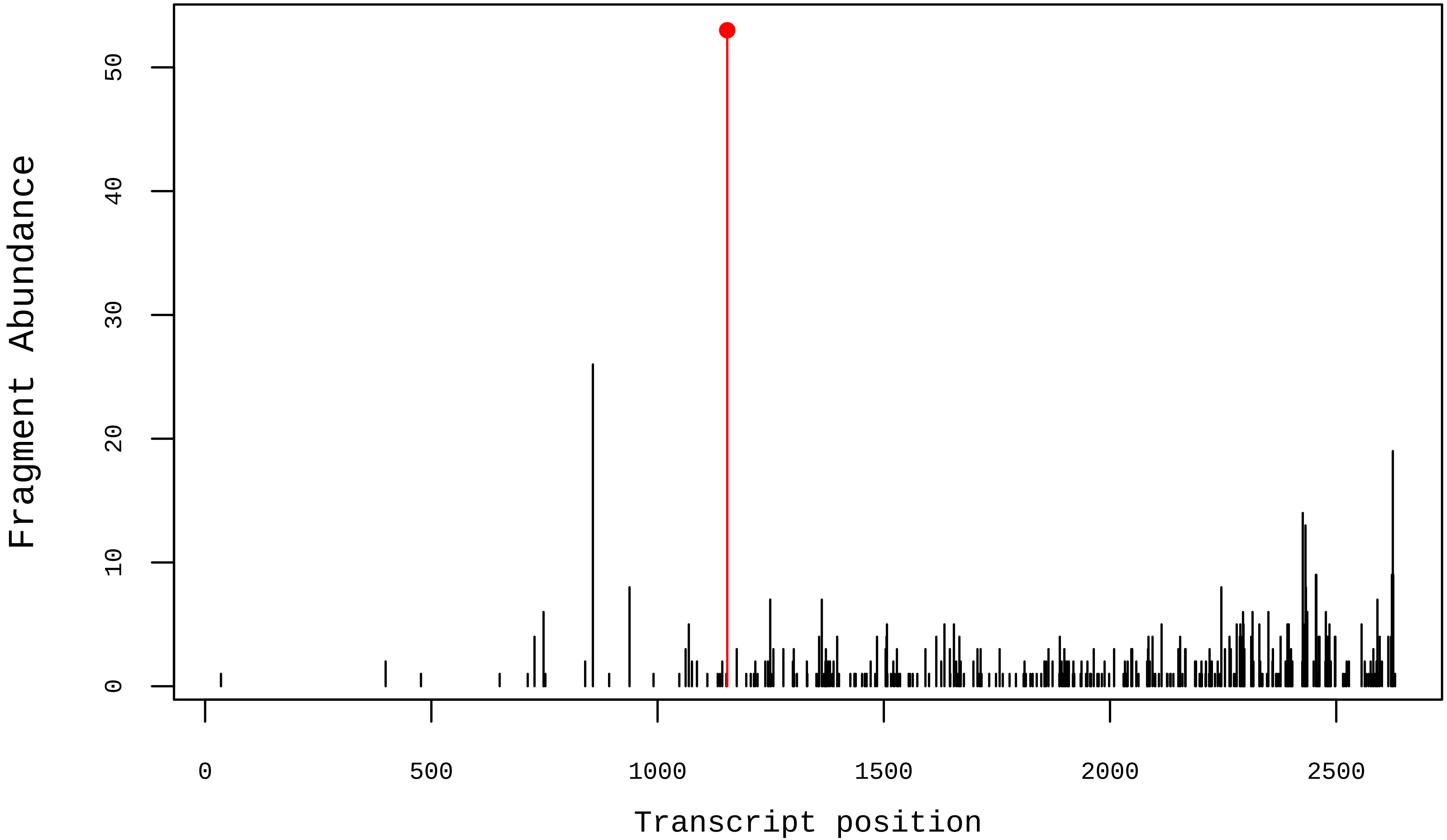

Cleavage site: 1154 Tag abundance: 53 Weighted abundance: 10.6 Category: 0
sRNA abundance: 1 Alignment score: 3.5 MFE ratio: 0.778 p-value: 0.006

HORVU2Hr1G060120|HORVU2Hr1G060120.1||1239|2454

```
5' ACACCAGGGGG-ACCCTTCAGTCCAATTCGCAG '3
           ||||o| ||||||||||||||
3'         TCCCTCGTGGGAAGTCAGGTTC       '5
```

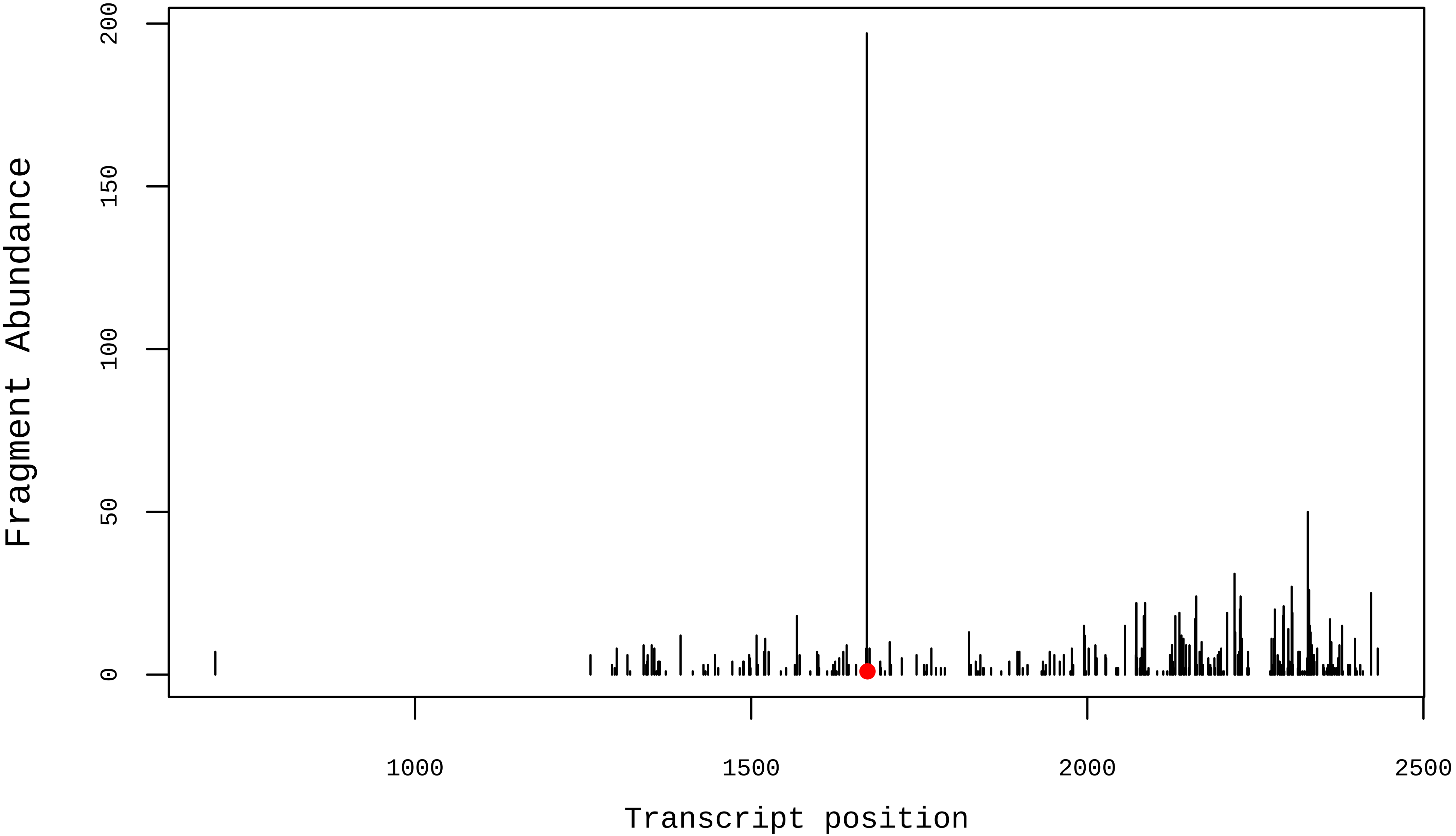

Cleavage site: 1673   Tag abundance: 1   Weighted abundance: 1   Category: 4
sRNA abundance: 1   Alignment score: 2.5   MFE ratio: 0.793   p-value: 0.019
