# Supplementary material for: Pi-starvation induced transcriptional changes in barley revealed by a comprehensive RNA-Seq and degradome analyses
Source: BMC Genomics. 2021 Mar 9;22:165. doi: 10.1186/s12864-021-07481-w (PMC7941915; doi:10.1186/s12864-021-07481-w)
Supplement: Supplementary file 26 — Additional file 26. The t-plots generated by PAREsnip2 software showing the potential mRNA targets for differentially expressed other sRNAs (DESs) identified in barley roots (low-Pi vs. control). [file 12864_2021_7481_MOESM26_ESM.pdf]

Additional file 26. The t-plots generated by PAREsnip2 software showing the potential mRNA targets for differentially expressed other sRNAs (DESs) identified in barley roots (low-Pi vs. control).

HORVU7Hr1G024570|HORVU7Hr1G024570.2||432|4386

```
5' GCCCGAGCCCACC-CGCTACGGCGACTGGGAGC '3
                ||| |||||||||o||
3'              GTGTCGCGATGCCGTTGC      '5
```

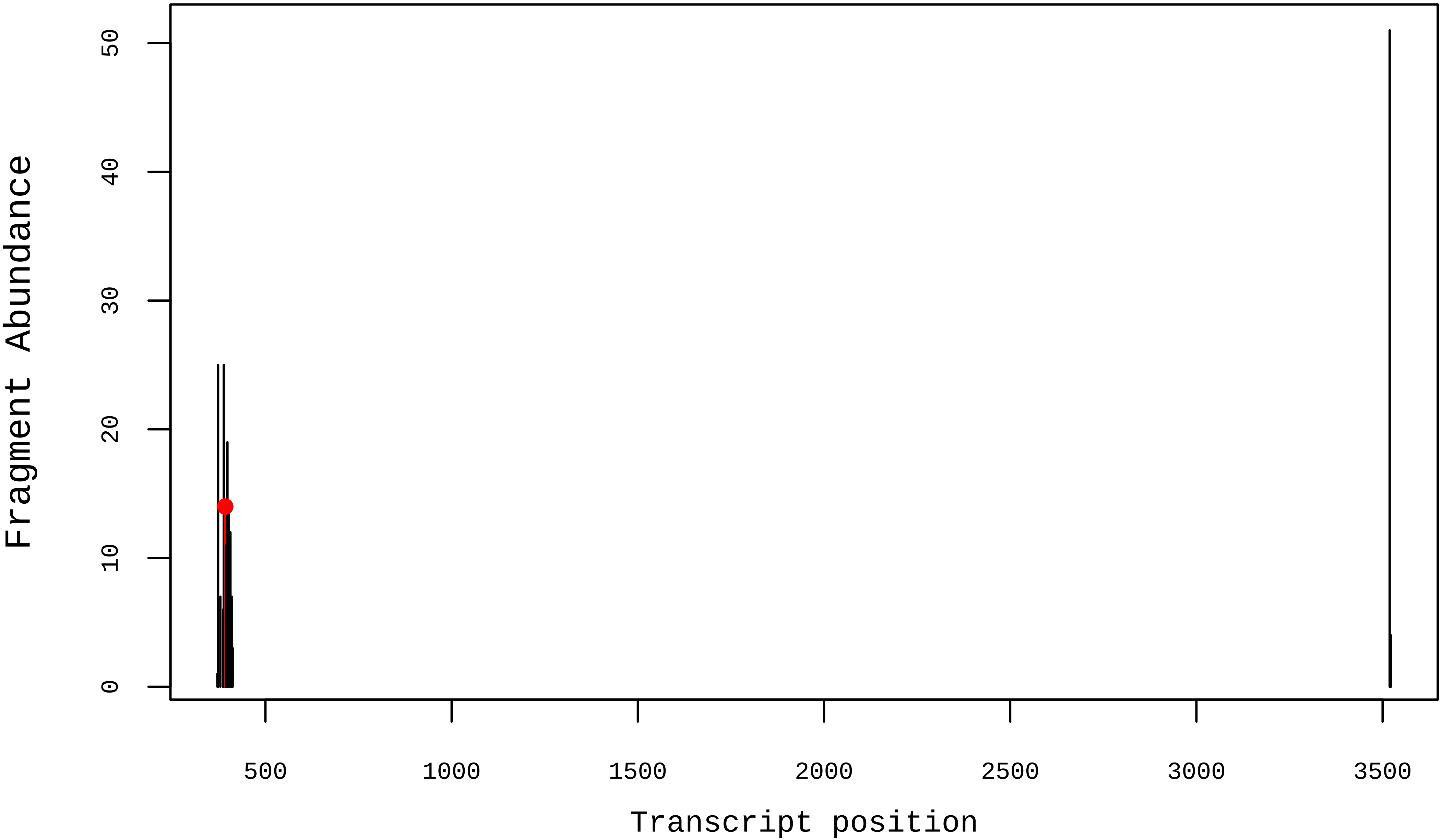

Cleavage site: 392   Tag abundance: 14   Weighted abundance: 4.667   Category: 2
sRNA abundance: 1   Alignment score: 4   MFE ratio: 0.719   p-value: 0.015

HORVU2Hr1G042740|HORVU2Hr1G042740.1||348|1174

```
5' CTCCTGCTGCTTCTGCTTTCGATG-CTTCGCGT '3
          |||||| ||||||o|||||
3'        CGACGACGACGAAGGCTACAC         '5
```

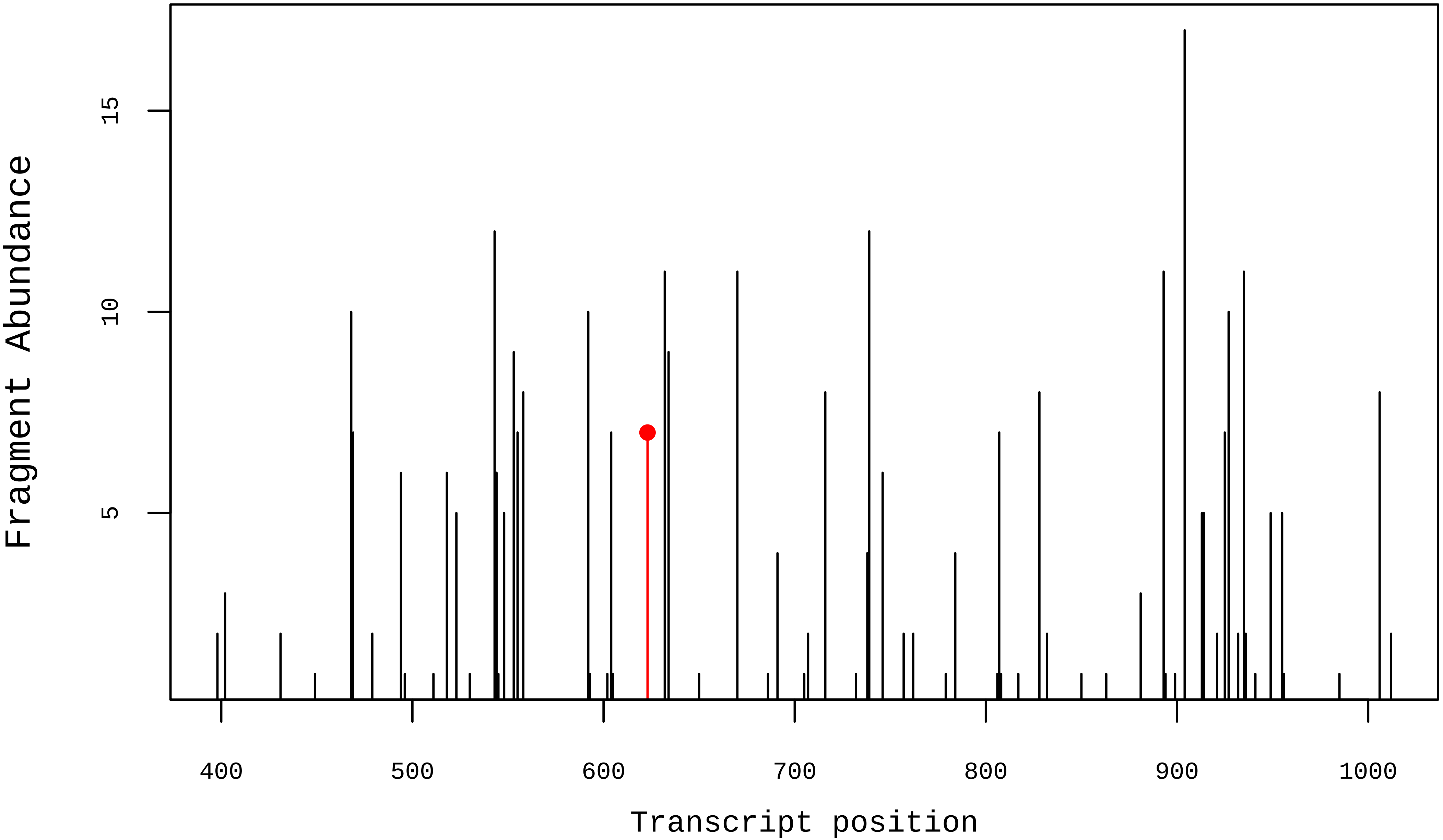

Cleavage site: 623 Tag abundance: 7 Weighted abundance: 7 Category: 2
sRNA abundance: 1 Alignment score: 4 MFE ratio: 0.703 p-value: 0.036

HORVU0Hr1G003660|HORVU0Hr1G003660.1||297|1955

```
5’ GCCGGCCGAAGGGTCGAGTAGGTCGGTGCTCG ’3
     ||||||||||||| ||||||||||
3’   GCCGGCTTCCCAGATCATCCAGCC       ’5
```

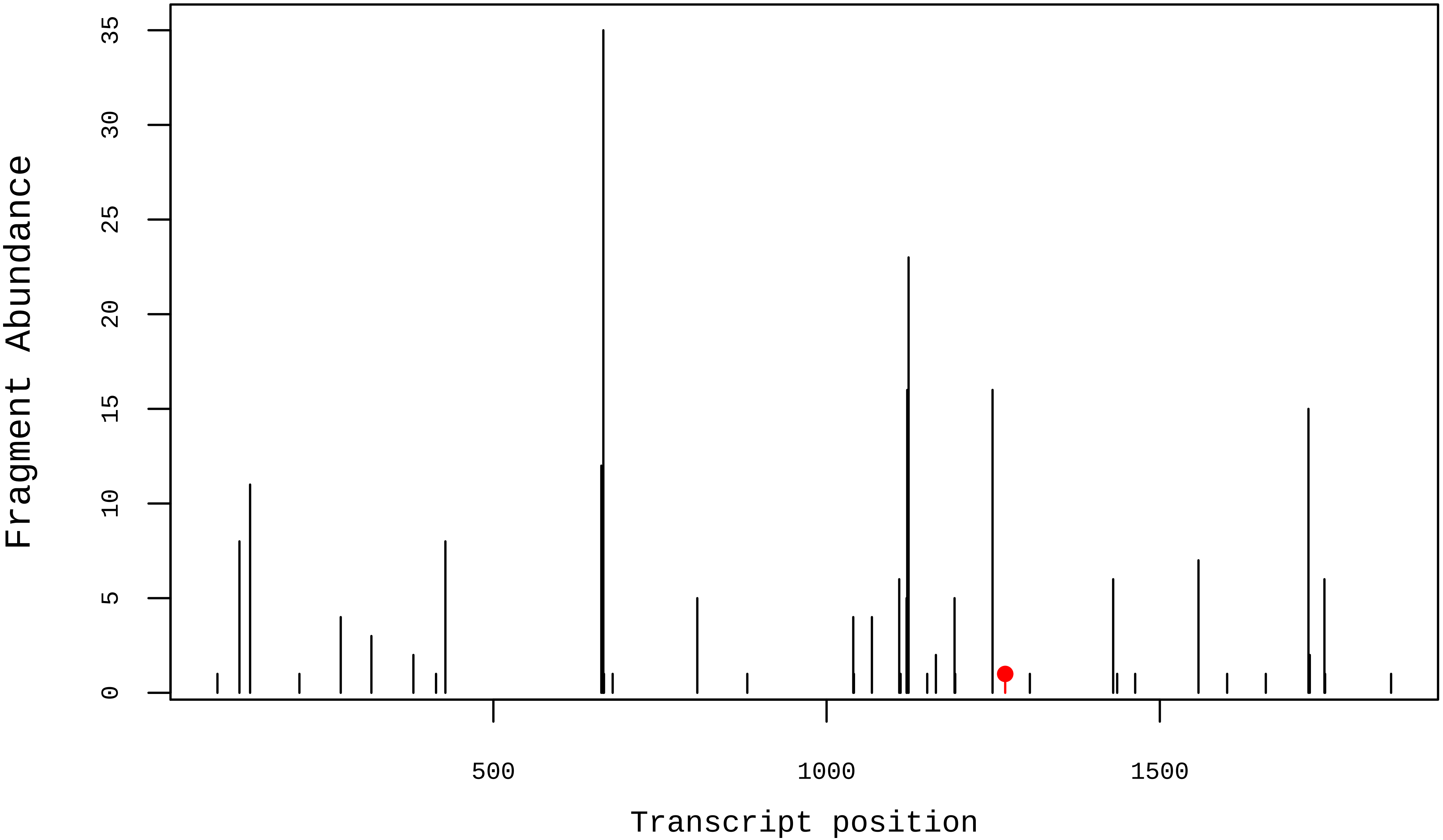

Cleavage site: 1268   Tag abundance: 1   Weighted abundance: 0.143   Category: 4
sRNA abundance: 1   Alignment score: 2   MFE ratio: 0.912   p-value: 0.046

HORVU0Hr1G023930|HORVU0Hr1G023930.3||396|2180

```
5' GTCGGCGGAAGGGTCGAGTAGGTCGGTGCTCG '3
       |||| |||||||| ||||||||||
3'     GCCGGCTTCCCAGATCATCCAGCC       '5
```

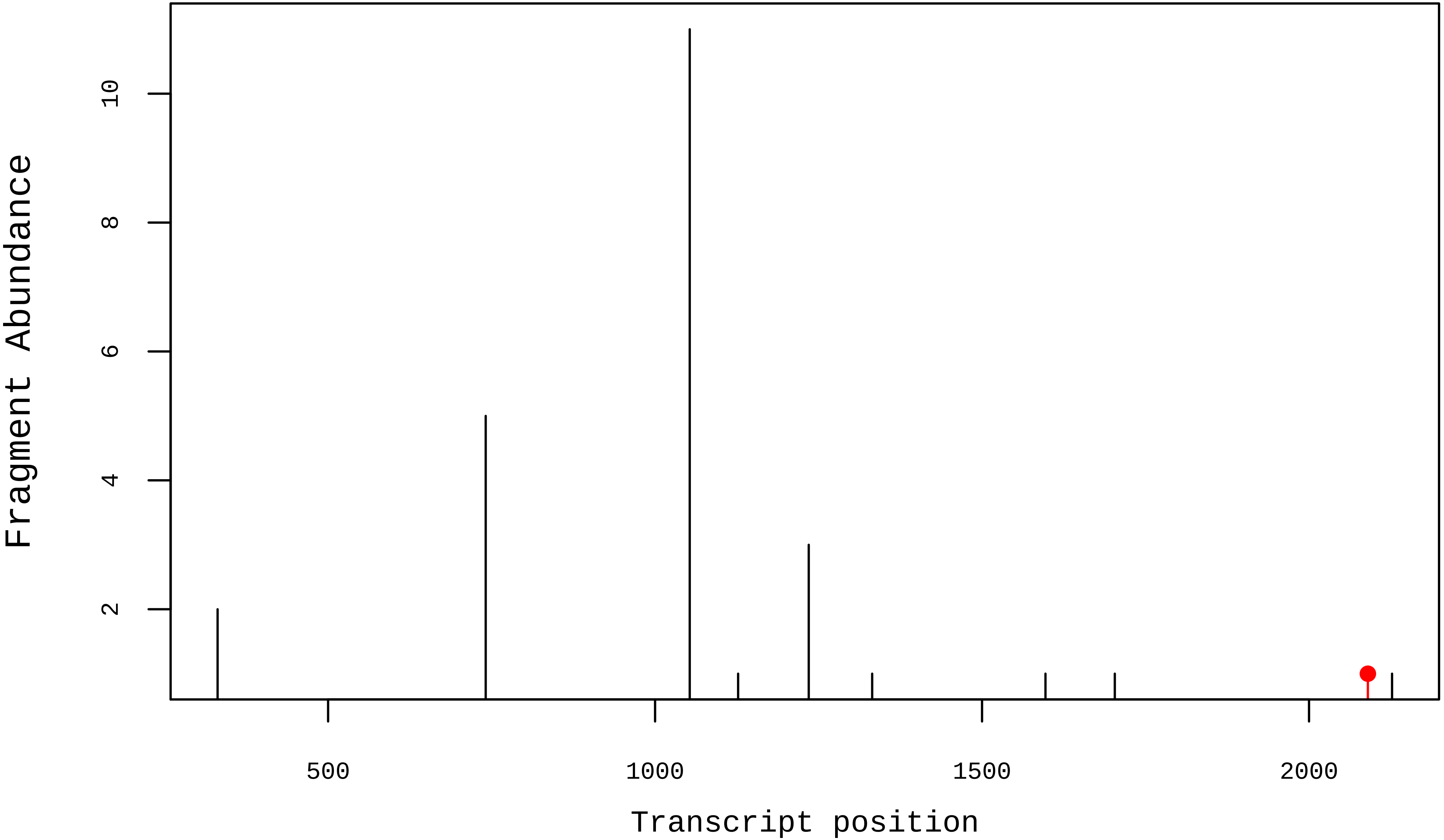

Cleavage site: 2090   Tag abundance: 1   Weighted abundance: 0.143   Category: 4
sRNA abundance: 1   Alignment score: 3   MFE ratio: 0.829   p-value: 0.019

HORVU2Hr1G072960|HORVU2Hr1G072960.1||600|3353

```
5' GCCGGCCGAAGGGTCGAGTAGGTCGGTGCTCG '3
      ||||||||||||| ||||||||||
3'    GCCGGCTTCCCAGATCATCCAGCC        '5
```

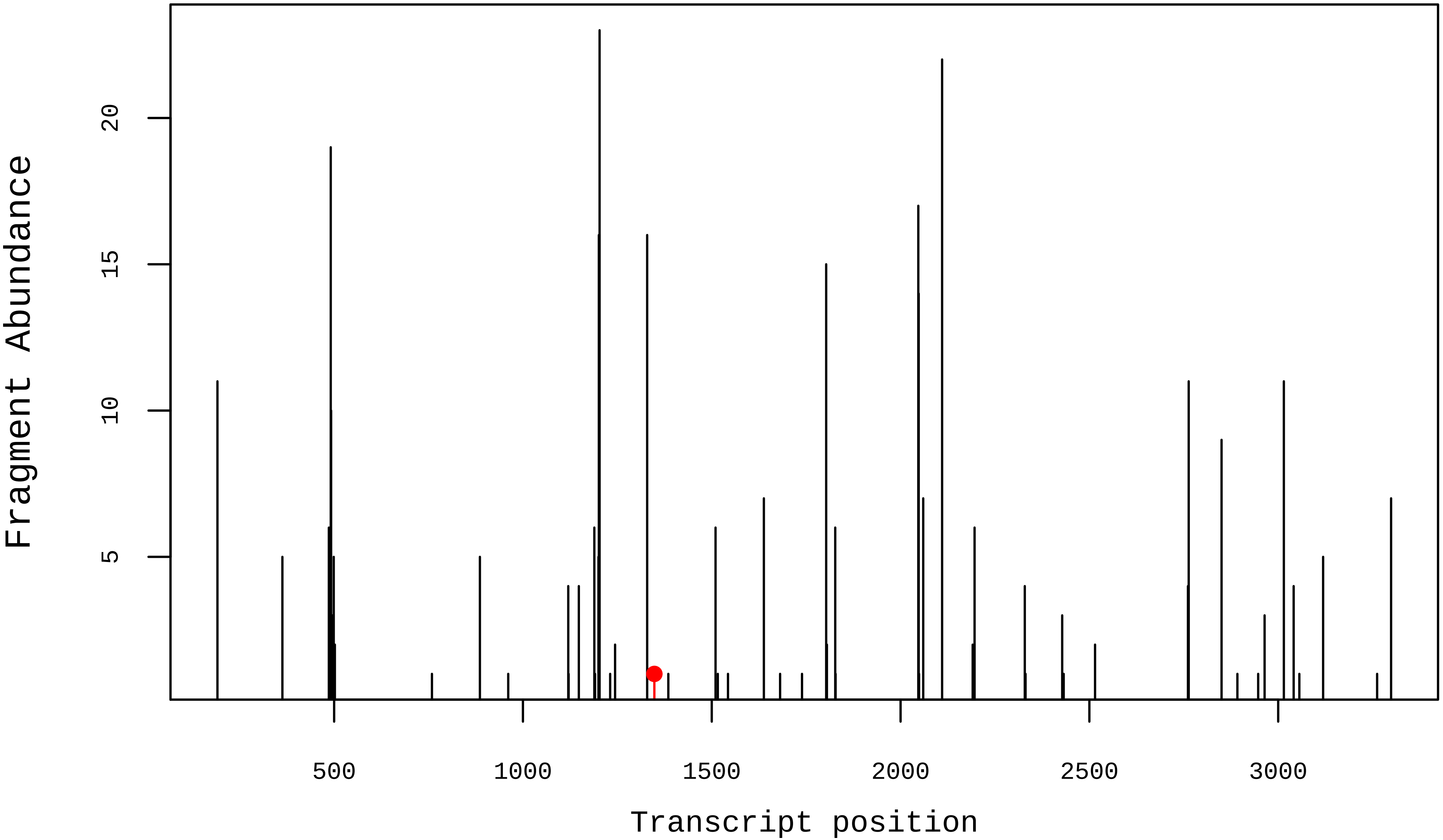

Cleavage site: 1348   Tag abundance: 1   Weighted abundance: 0.143   Category: 4
sRNA abundance: 1   Alignment score: 2   MFE ratio: 0.912   p-value: 0.03

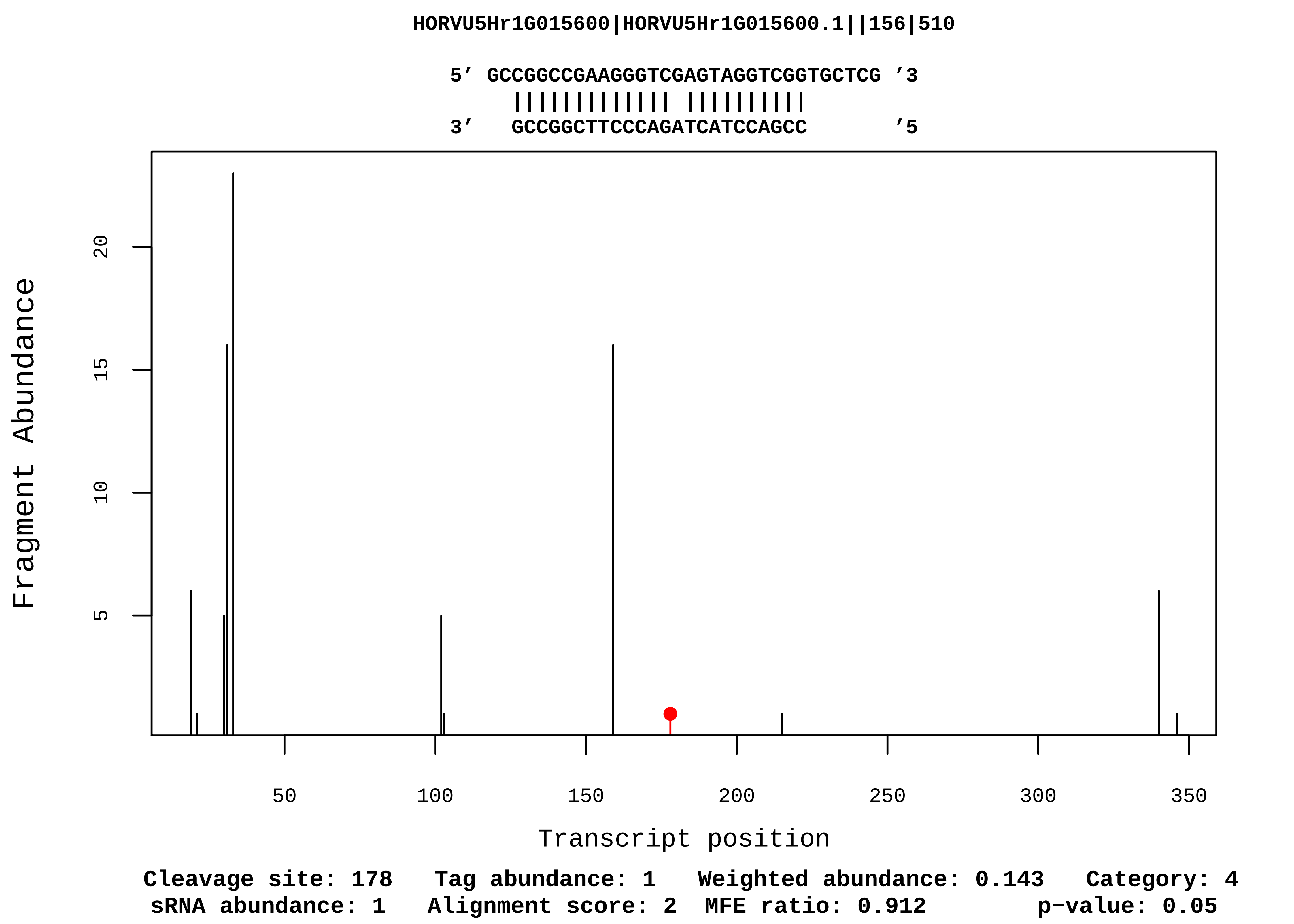
HORVU5Hr1G015600|HORVU5Hr1G015600.1||156|510
5' GCCGGCCGAAGGGTCGAGTAGGTCGGTGCTCG '3
3' GCCGGCTTCCCAGATCATCCAGCC '5
Fragment Abundance
5
10
15
20
50
100
150
200
250
300
350
Transcript position
Cleavage site: 178 Tag abundance: 1 Weighted abundance: 0.143 Category: 4
sRNA abundance: 1 Alignment score: 2 MFE ratio: 0.912 p-value: 0.05

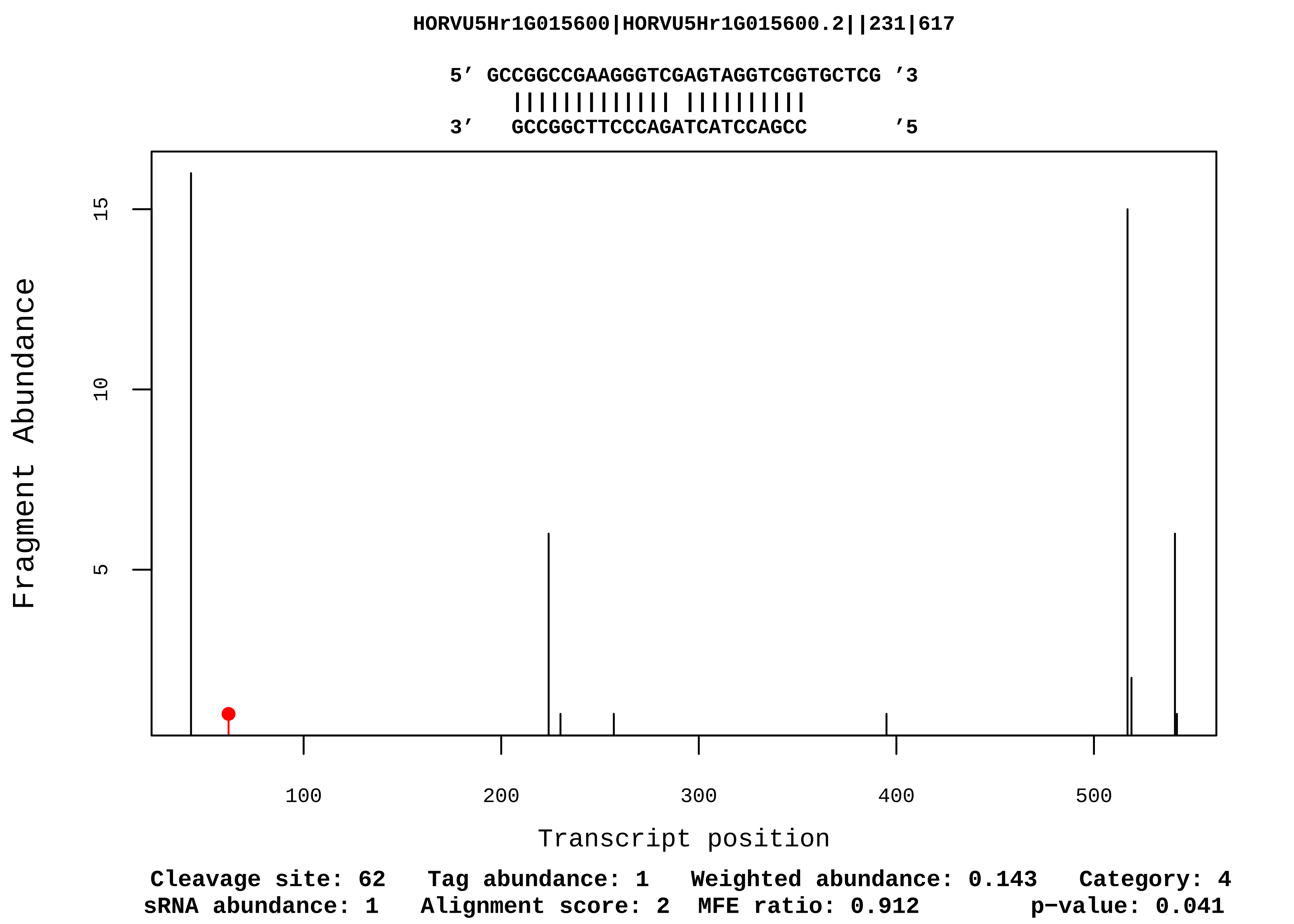

HORVU5Hr1G015600|HORVU5Hr1G015600.2||231|617
5′ GCCGGCCGAAGGGTCGAGTAGGTCGGTGCTCG ′3
||||||||||||| ||||||||||
3′ GCCGGCTTCCCAGATCATCCAGCC ′5
Fragment Abundance
5
10
15
100
200
300
400
500
Transcript position
Cleavage site: 62 Tag abundance: 1 Weighted abundance: 0.143 Category: 4
sRNA abundance: 1 Alignment score: 2 MFE ratio: 0.912 p-value: 0.041

HORVU5Hr1G015600|HORVU5Hr1G015600.3||276|1709

```
5' GCCGGCCGAAGGGTCGAGTAGGTCGGTGCTCG '3
      ||||||||||||| ||||||||||
3'    GCCGGCTTCCCAGATCATCCAGCC       '5
```

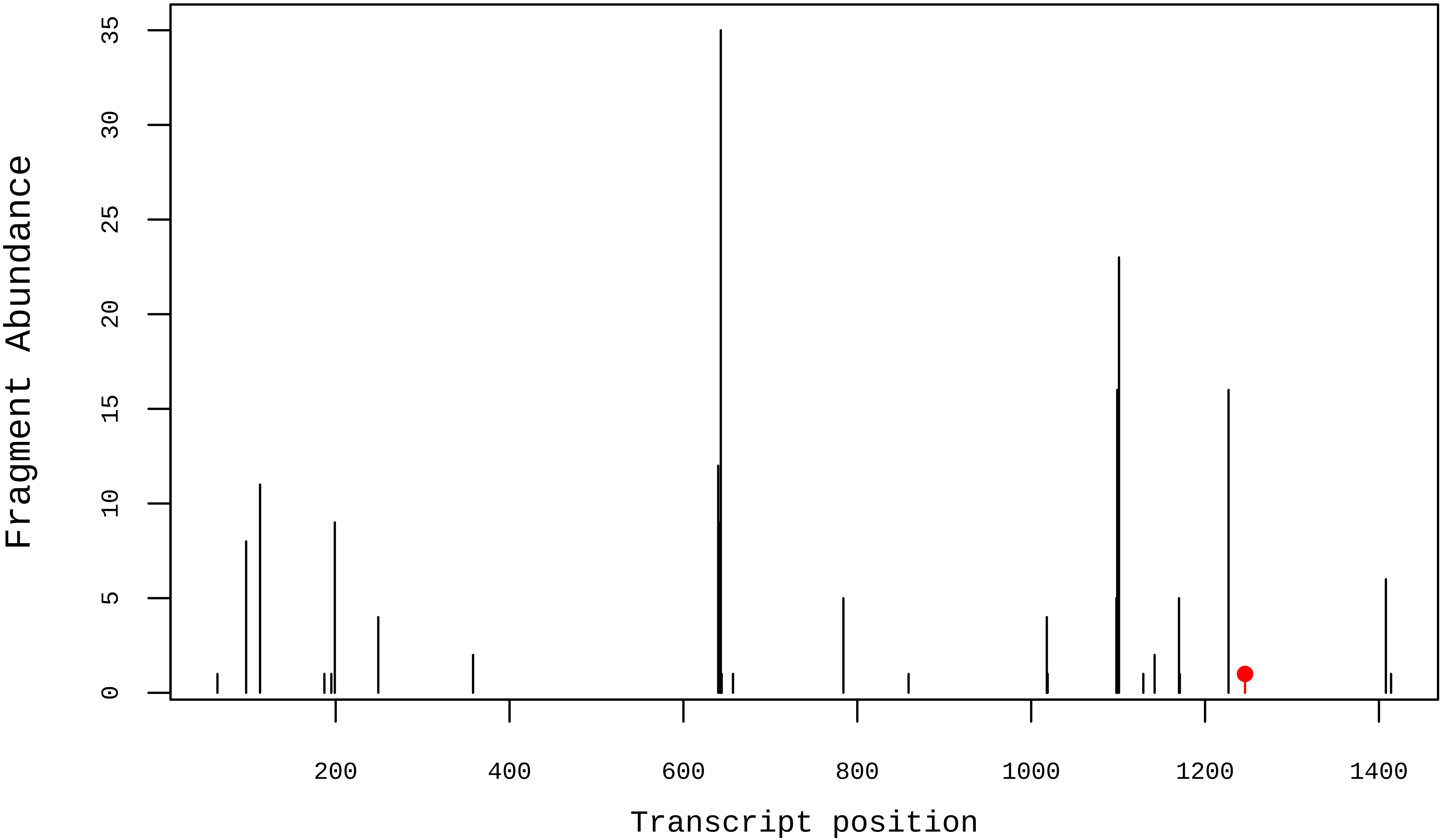

Cleavage site: 1246 Tag abundance: 1 Weighted abundance: 0.143 Category: 4
sRNA abundance: 1 Alignment score: 2 MFE ratio: 0.912 p-value: 0.032

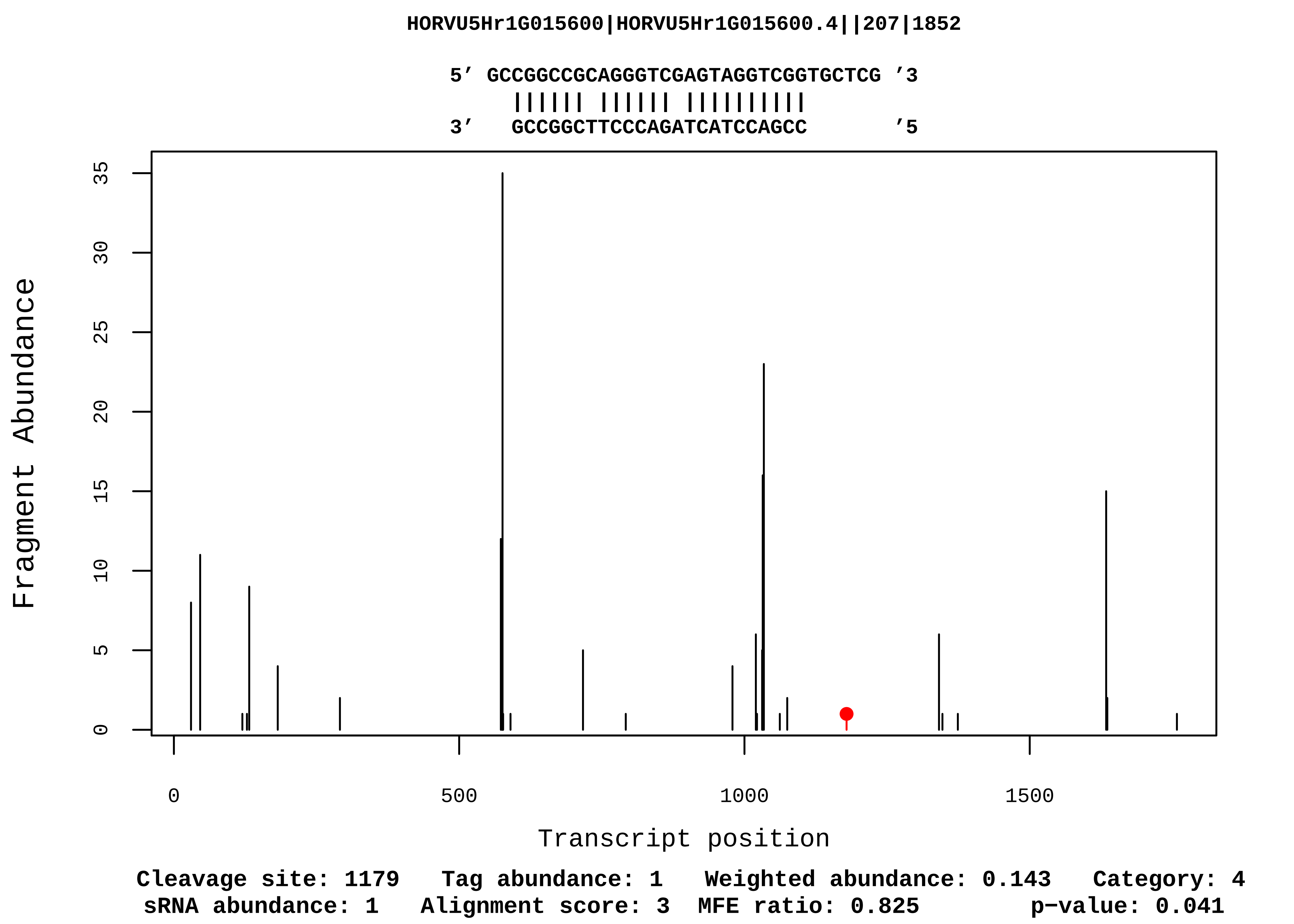
HORVU5Hr1G015600|HORVU5Hr1G015600.4||207|1852
5' GCCGGCCGCAGGGTCGAGTAGGTCGGTGCTCG '3
|||||| |||||| ||||||||||
3' GCCGGCTTCCCAGATCATCCAGCC '5
Fragment Abundance
0
5
10
15
20
25
30
35
0
500
1000
1500
Transcript position
Cleavage site: 1179 Tag abundance: 1 Weighted abundance: 0.143 Category: 4
sRNA abundance: 1 Alignment score: 3 MFE ratio: 0.825 p-value: 0.041

HORVU2Hr1G083660|HORVU2Hr1G083660.1||513|513

```
5' ATGGTGACGGG-CTTGGAGCCGAGGGAGCAGCG '3
          |||| |||||||||o|||o
3'        GCCCGGAACCTCGGTTCCTC       '5
```

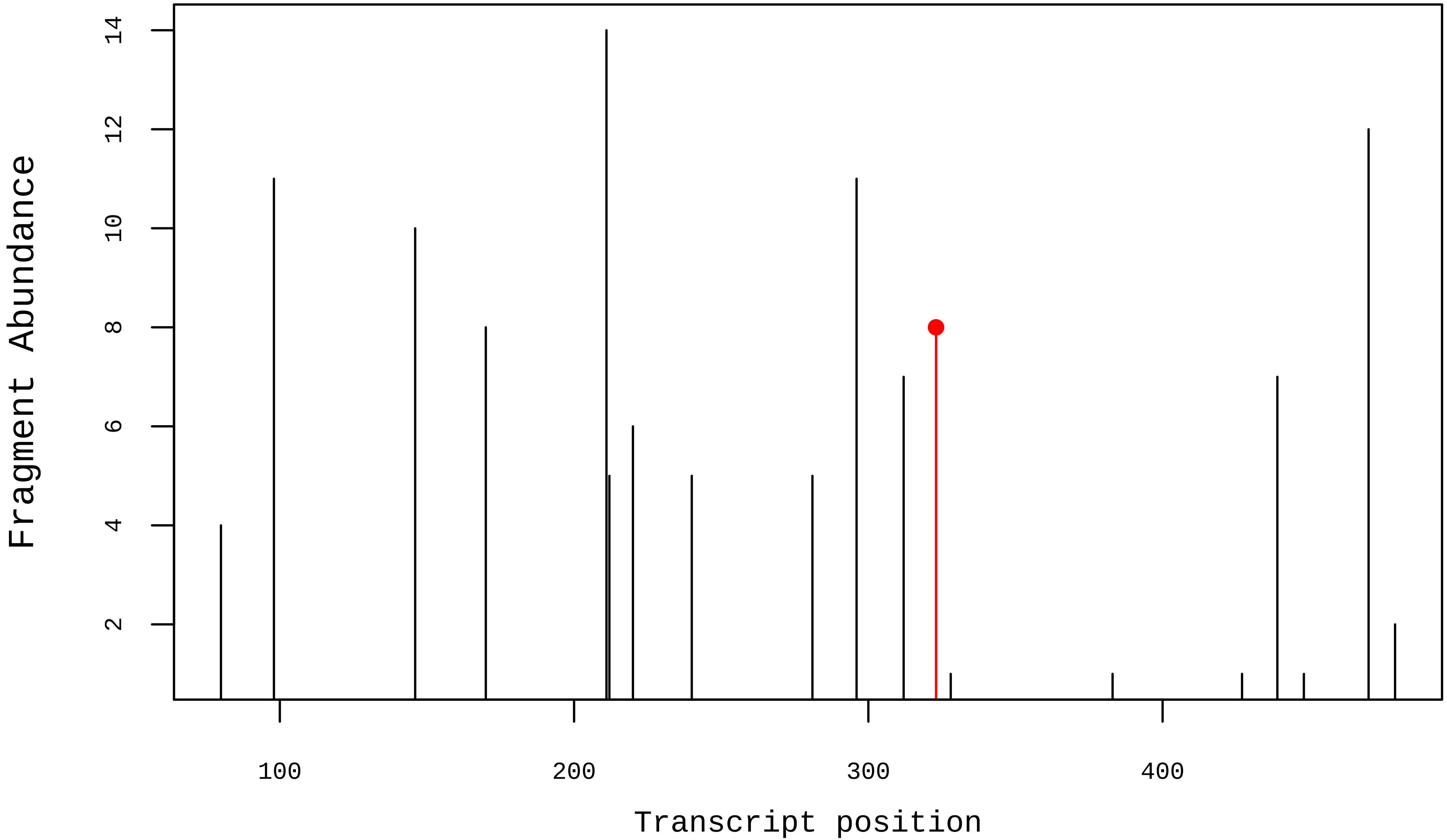

Cleavage site: 323 Tag abundance: 8 Weighted abundance: 4 Category: 2
sRNA abundance: 1 Alignment score: 4 MFE ratio: 0.773 p-value: 0.036

HORVU2Hr1G083680|HORVU2Hr1G083680.1||372|577

```
5' ATGGTGACGGG-CTTGGAGCCGAGGGAGCAGCG '3
               |||| |||||||||o|||o
3'             GCCCGGAACCTCGGTTCCTC       '5
```

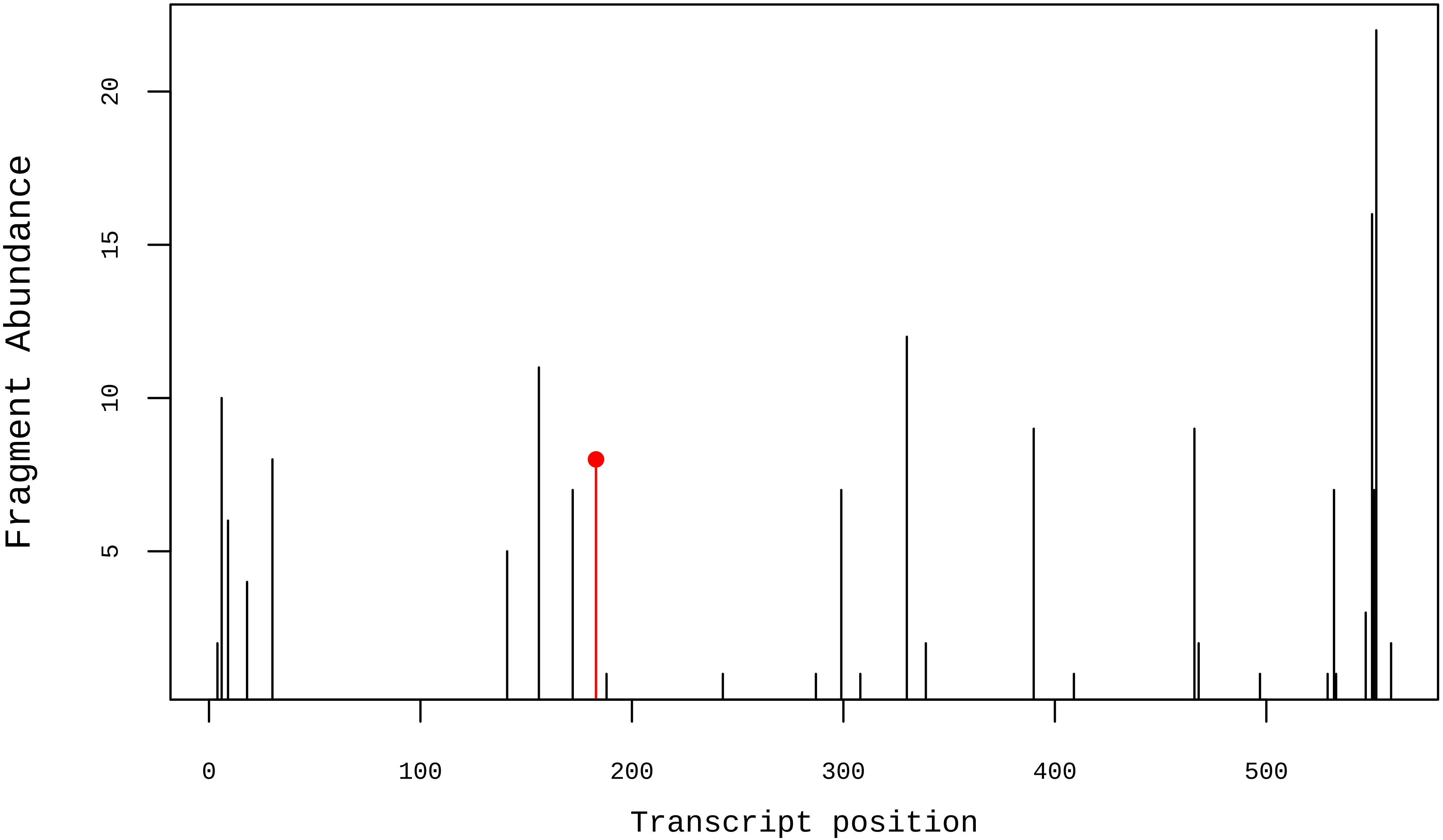

Cleavage site: 183   Tag abundance: 8   Weighted abundance: 4   Category: 2
sRNA abundance: 1   Alignment score: 4   MFE ratio: 0.773   p-value: 0.037

HORVU3Hr1G054090|HORVU3Hr1G054090.1||1272|2478

```
5' GACGAGCTTG-CCAAGTACGCTTAGCTGGATAC '3
          o| |||||o|||||||o||
3'        GCGGGTTCGTGCGAATTGA        '5
```

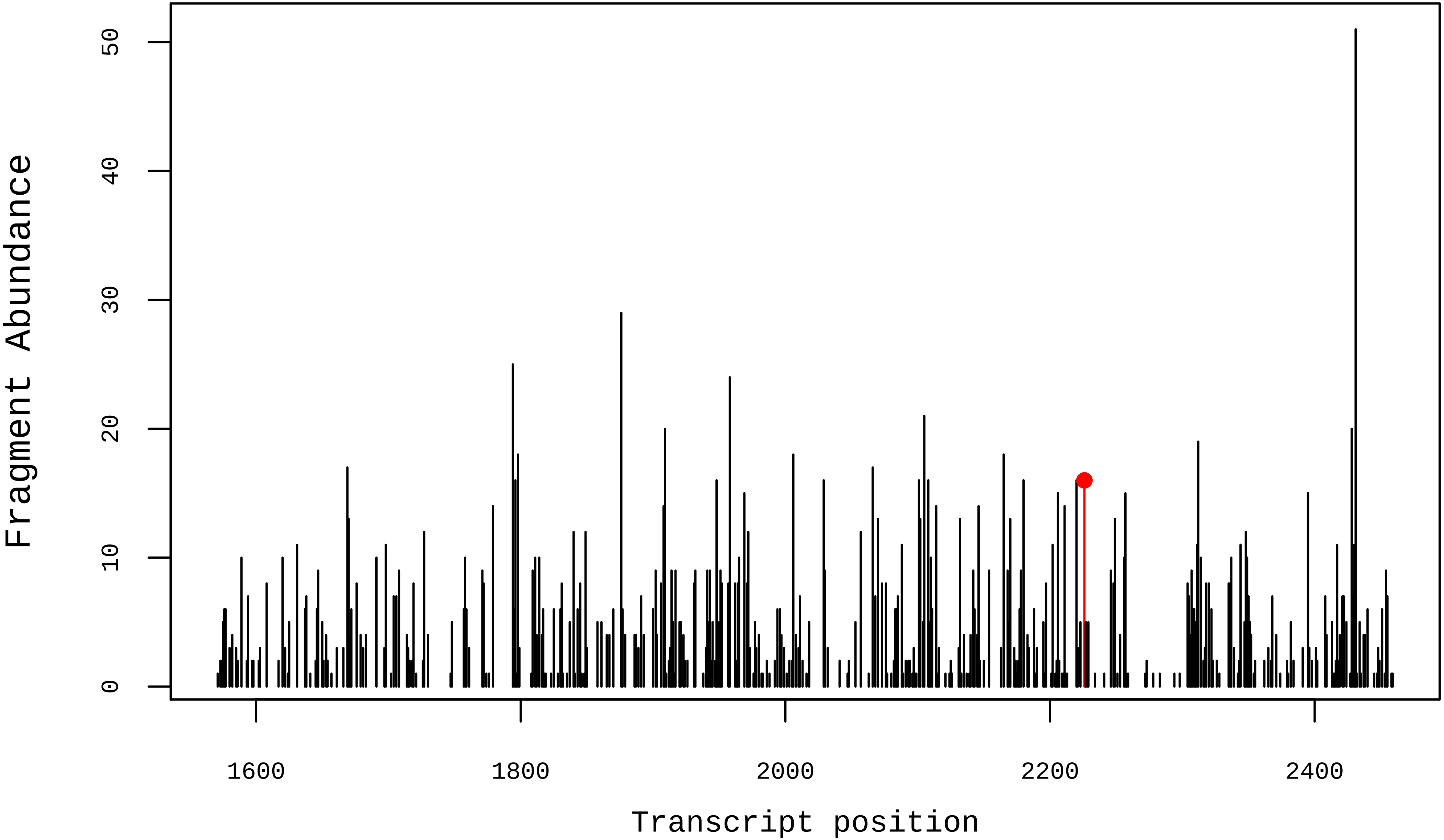

Cleavage site: 2226   Tag abundance: 16   Weighted abundance: 16   Category: 2
sRNA abundance: 1   Alignment score: 3.5   MFE ratio: 0.73   p-value: 0.048

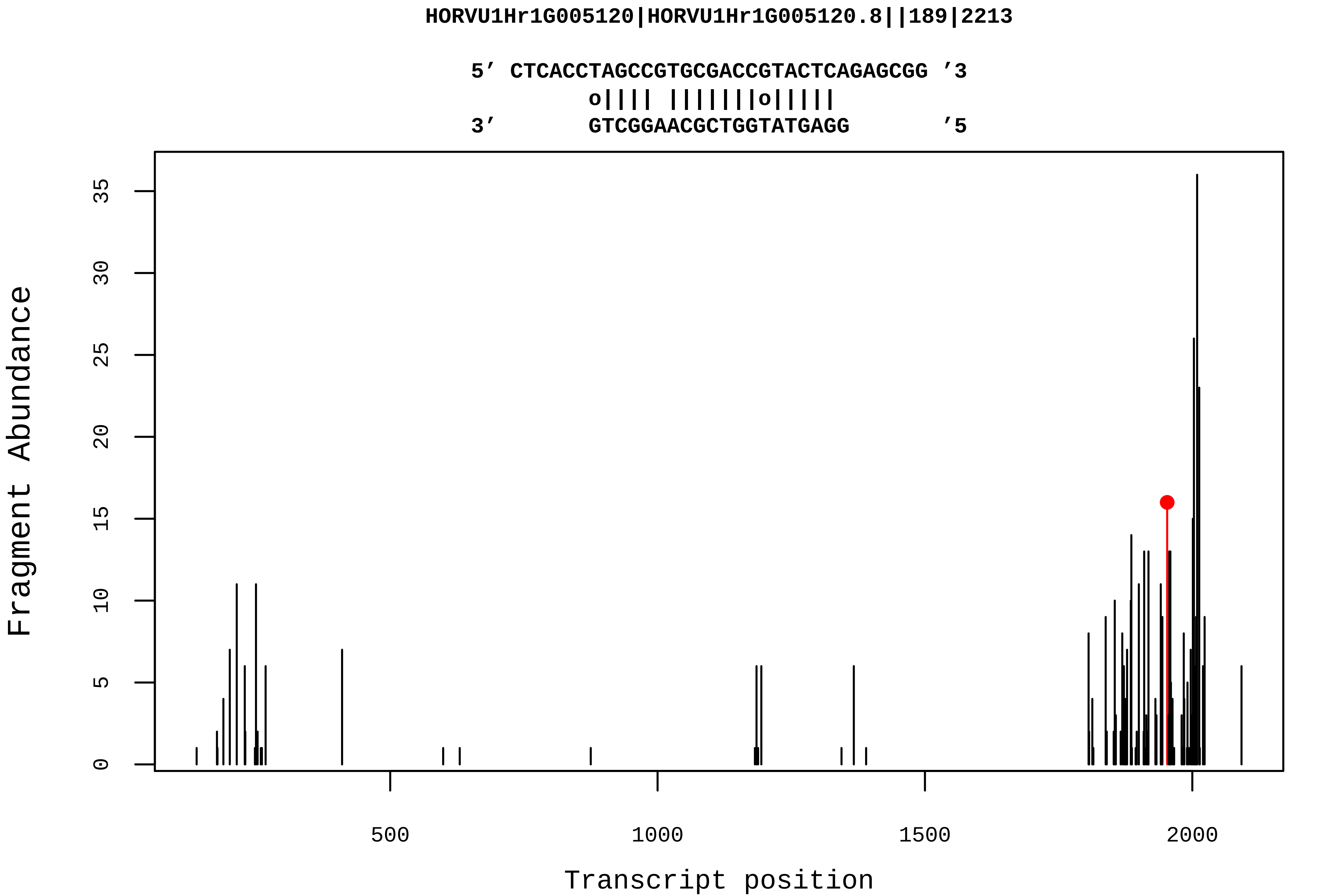

Cleavage site: 1953 Tag abundance: 16 Weighted abundance: 4 Category: 2
sRNA abundance: 1 Alignment score: 3.5 MFE ratio: 0.798 p-value: 0.04

HORVU0Hr1G023930|HORVU0Hr1G023930.3||396|2180

```
5' GTCGGCGGAAGGGTCGAGTAGGTCGGTGCTCG '3
       ||||||||||||||||||||||||
3'     GCCGCCTTCCCAGCTCATCCAGCC        '5
```

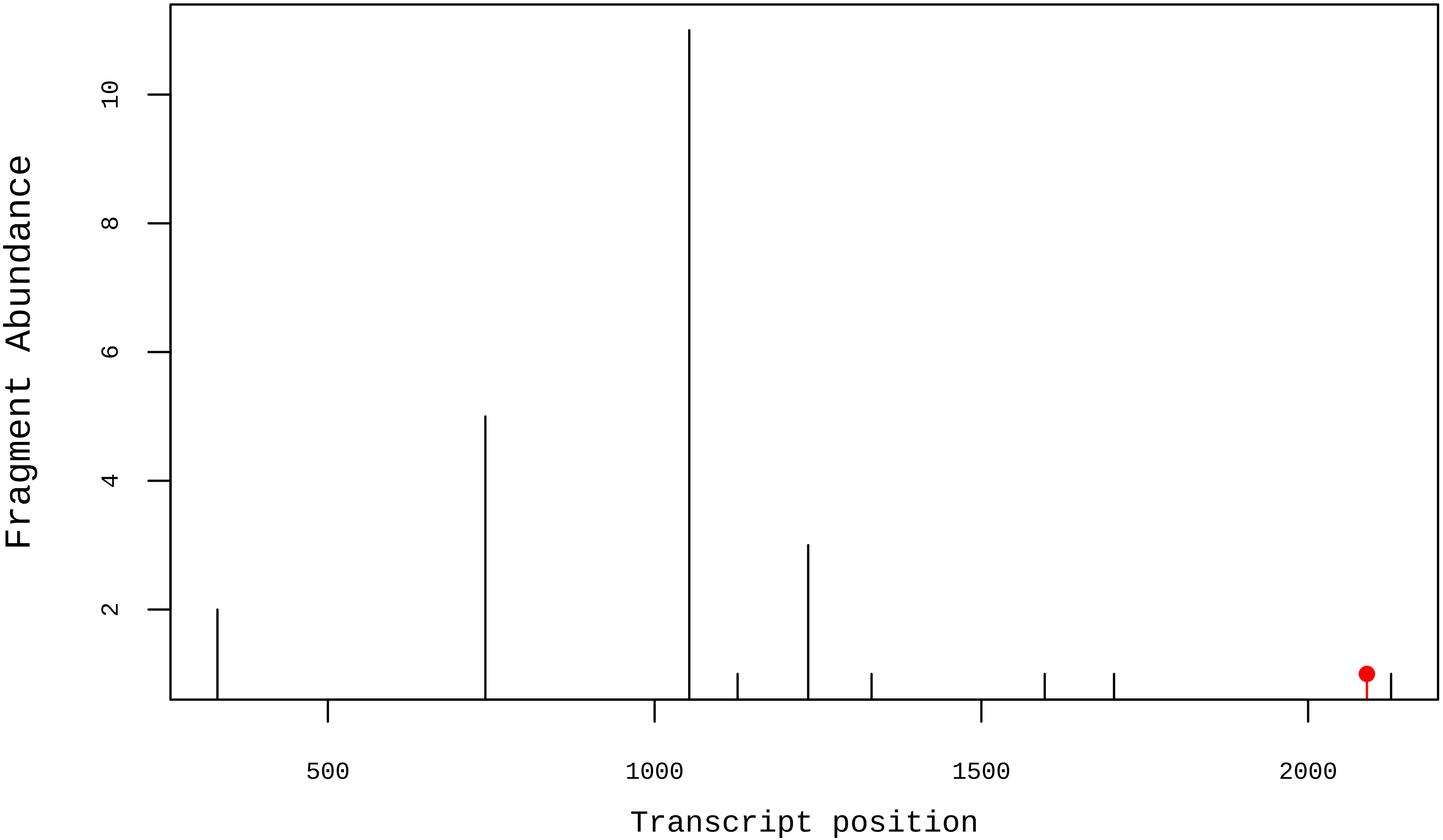

Cleavage site: 2090   Tag abundance: 1   Weighted abundance: 0.143   Category: 4
sRNA abundance: 1   Alignment score: 0   MFE ratio: 1   p-value: 0.003

HORVU2Hr1G072960|HORVU2Hr1G072960.1||600|3353

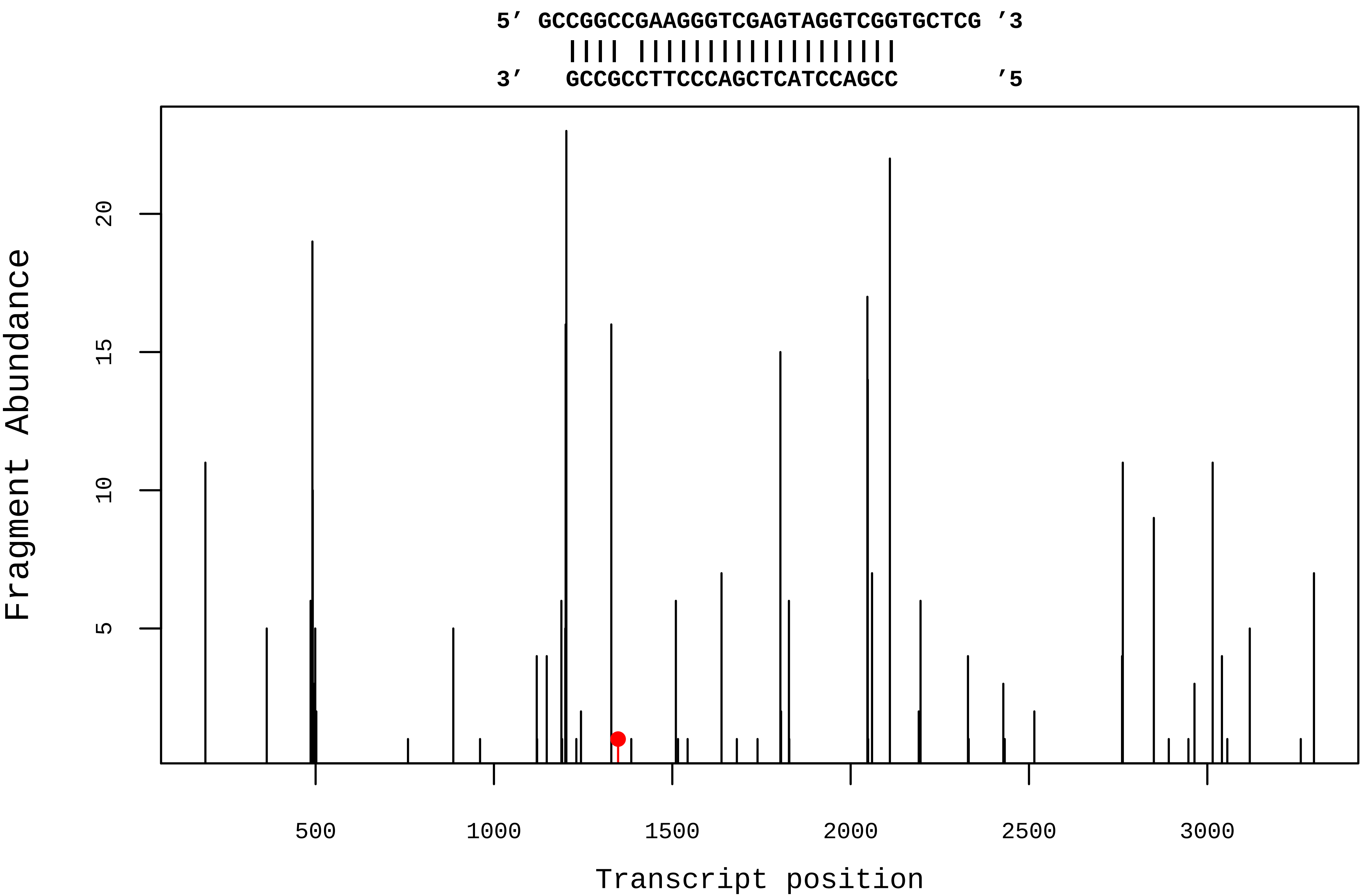

Cleavage site: 1348 Tag abundance: 1 Weighted abundance: 0.143 Category: 4
sRNA abundance: 1 Alignment score: 1 MFE ratio: 0.884 p-value: 0.035

HORVU5Hr1G015600|HORVU5Hr1G015600.2||231|617

```
5' GCCGGCCGAAGGGTCGAGTAGGTCGGTGCTCG '3
      |||| ||||||||||||||||||||
3'    GCCGCCTTCCCAGCTCATCCAGCC       '5
```

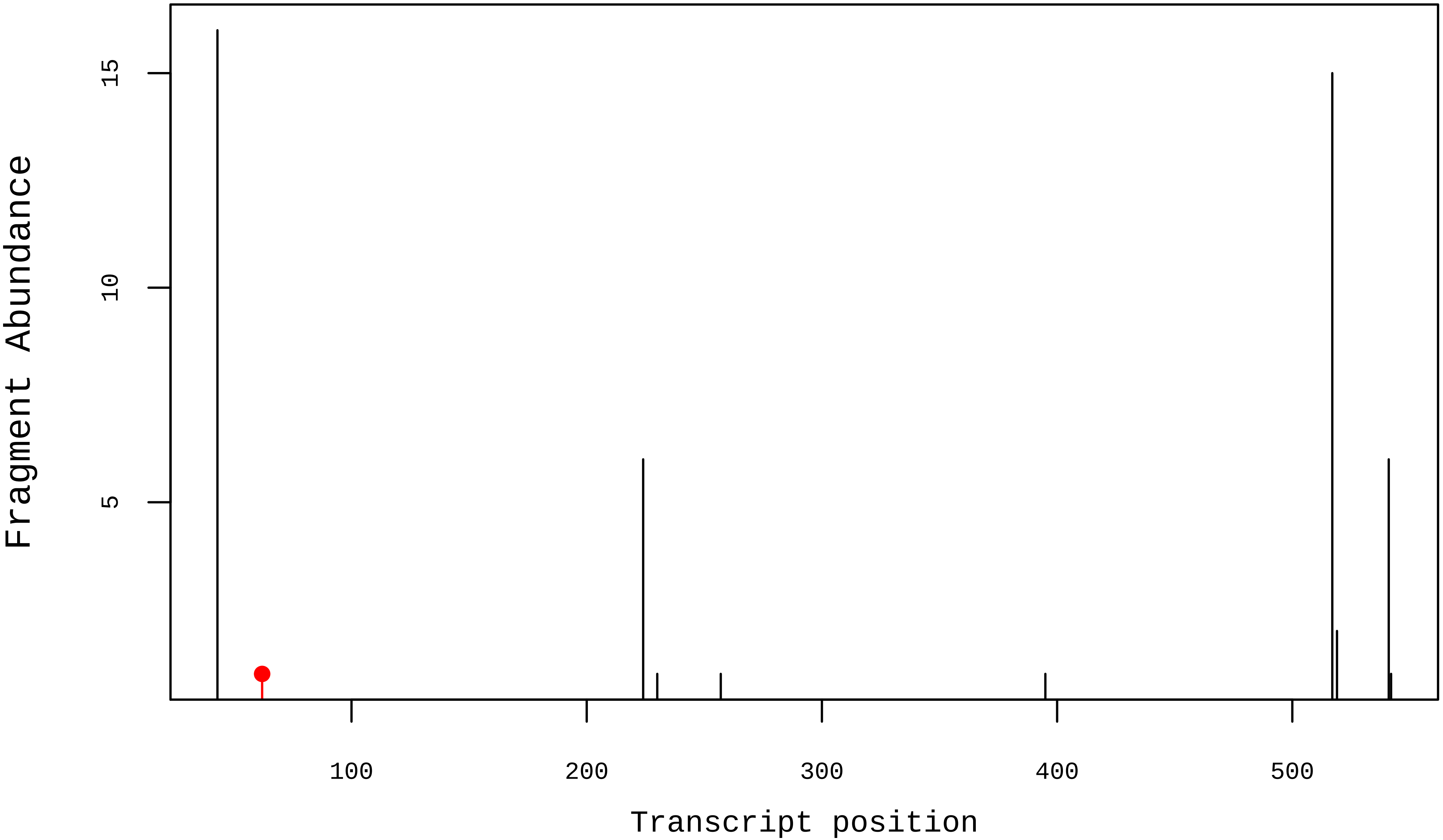

Cleavage site: 62   Tag abundance: 1   Weighted abundance: 0.143   Category: 4
sRNA abundance: 1   Alignment score: 1   MFE ratio: 0.884   p-value: 0.049

HORVU5Hr1G015600|HORVU5Hr1G015600.3||276|1709

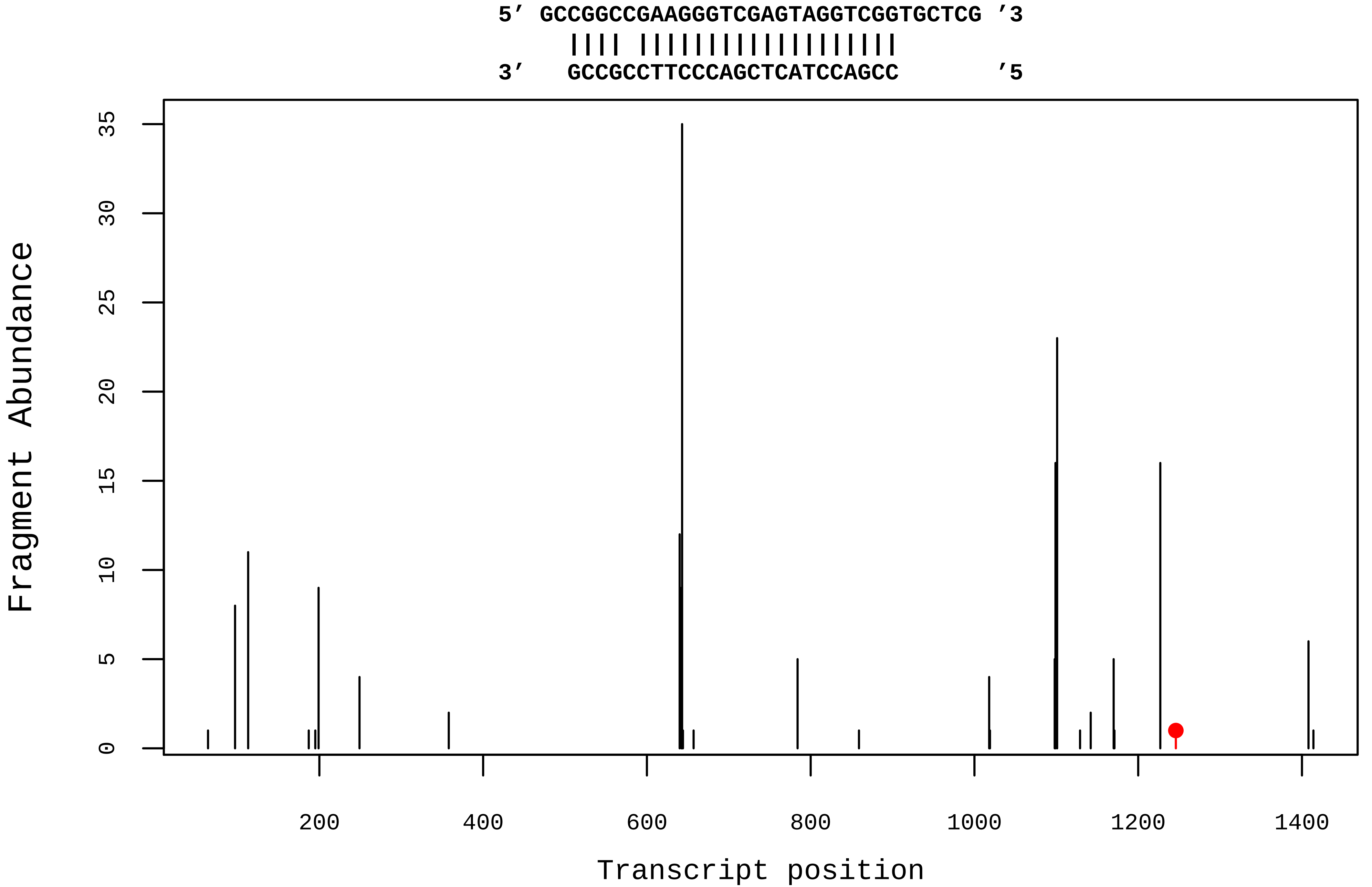

Cleavage site: 1246 Tag abundance: 1 Weighted abundance: 0.143 Category: 4
sRNA abundance: 1 Alignment score: 1 MFE ratio: 0.884 p-value: 0.038

HORVU5Hr1G015600|HORVU5Hr1G015600.4||207|1852

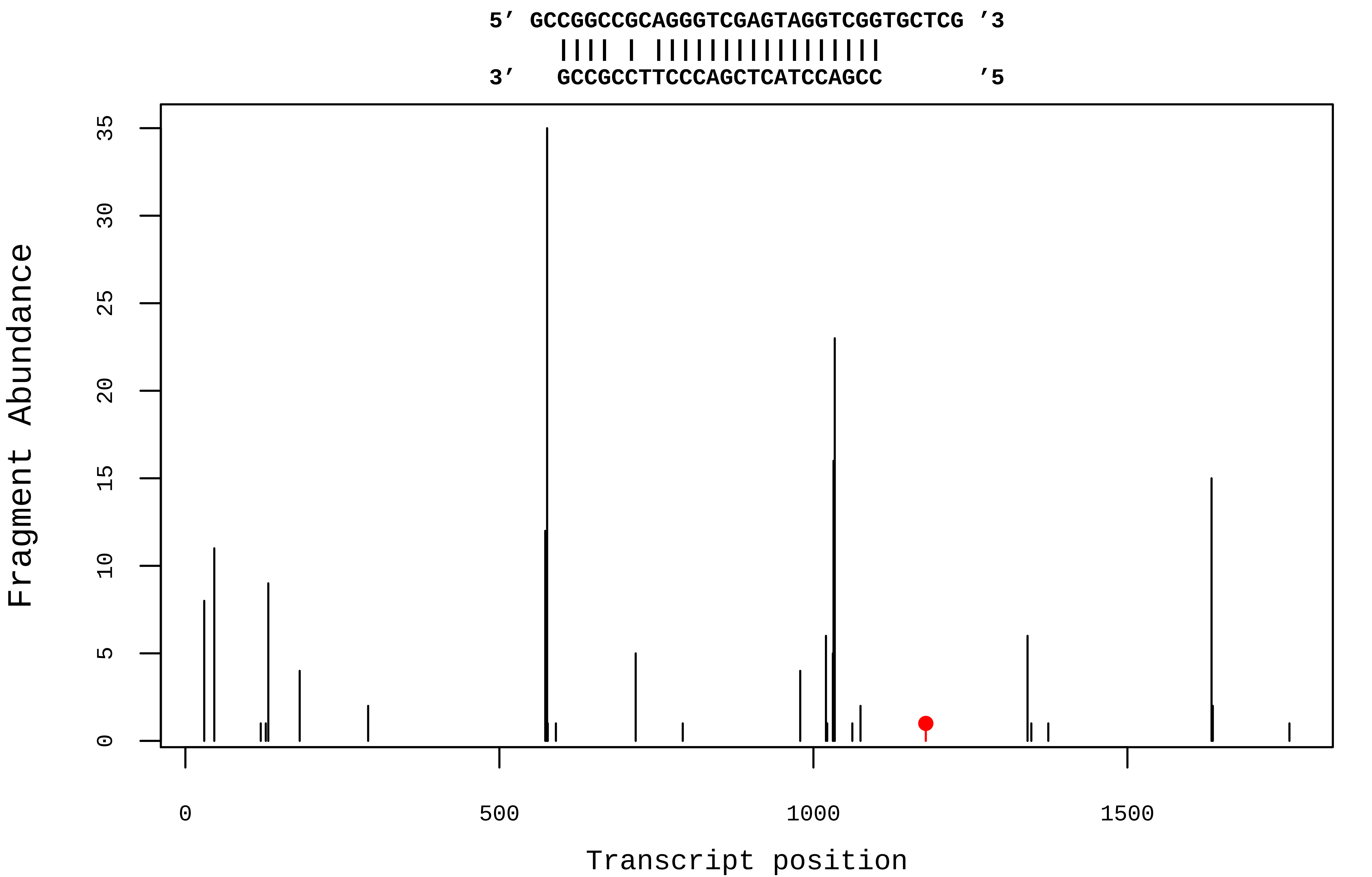

Cleavage site: 1179   Tag abundance: 1   Weighted abundance: 0.143   Category: 4
sRNA abundance: 1   Alignment score: 2   MFE ratio: 0.8   p-value: 0.041

HORVU0Hr1G003660|HORVU0Hr1G003660.1||297|1955

```
5' GCCGGCCGAAGGGTCGAGTAGGTCGGTGCTCG '3
      ||||||||||||||||o|||||||
3'    GCCGGCTTCCCAGCTCGTCCAGCC        '5
```

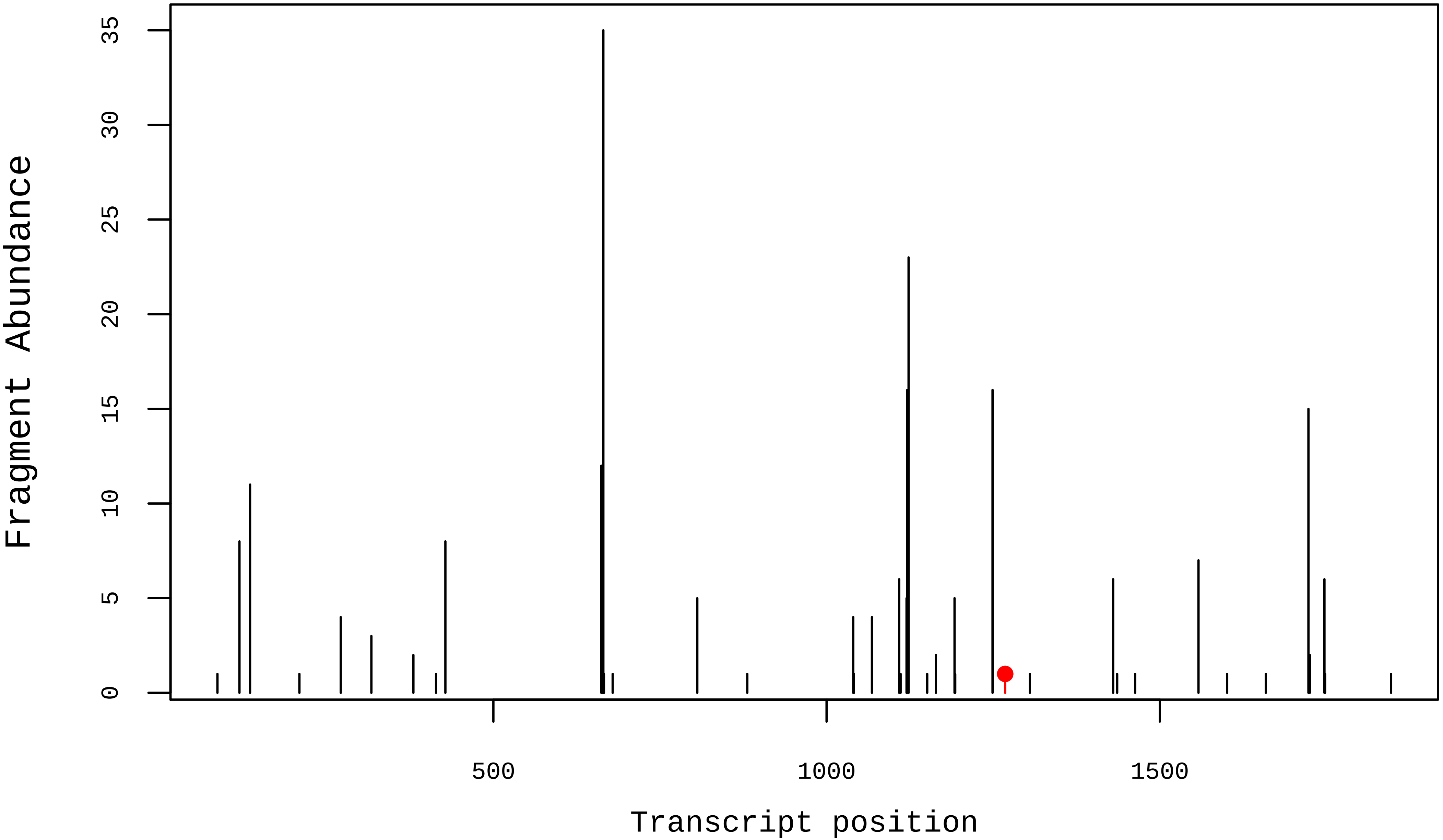

Cleavage site: 1268 Tag abundance: 1 Weighted abundance: 0.143 Category: 4
sRNA abundance: 1 Alignment score: 1 MFE ratio: 0.964 p-value: 0.046

HORVU0Hr1G023930|HORVU0Hr1G023930.3||396|2180

```
5' GTCGGCGGAAGGGTCGAGTAGGTCGGTGCTCG '3
      |||| |||||||||||o|||||||
3'   GCCGGCTTCCCAGCTCGTCCAGCC       '5
```

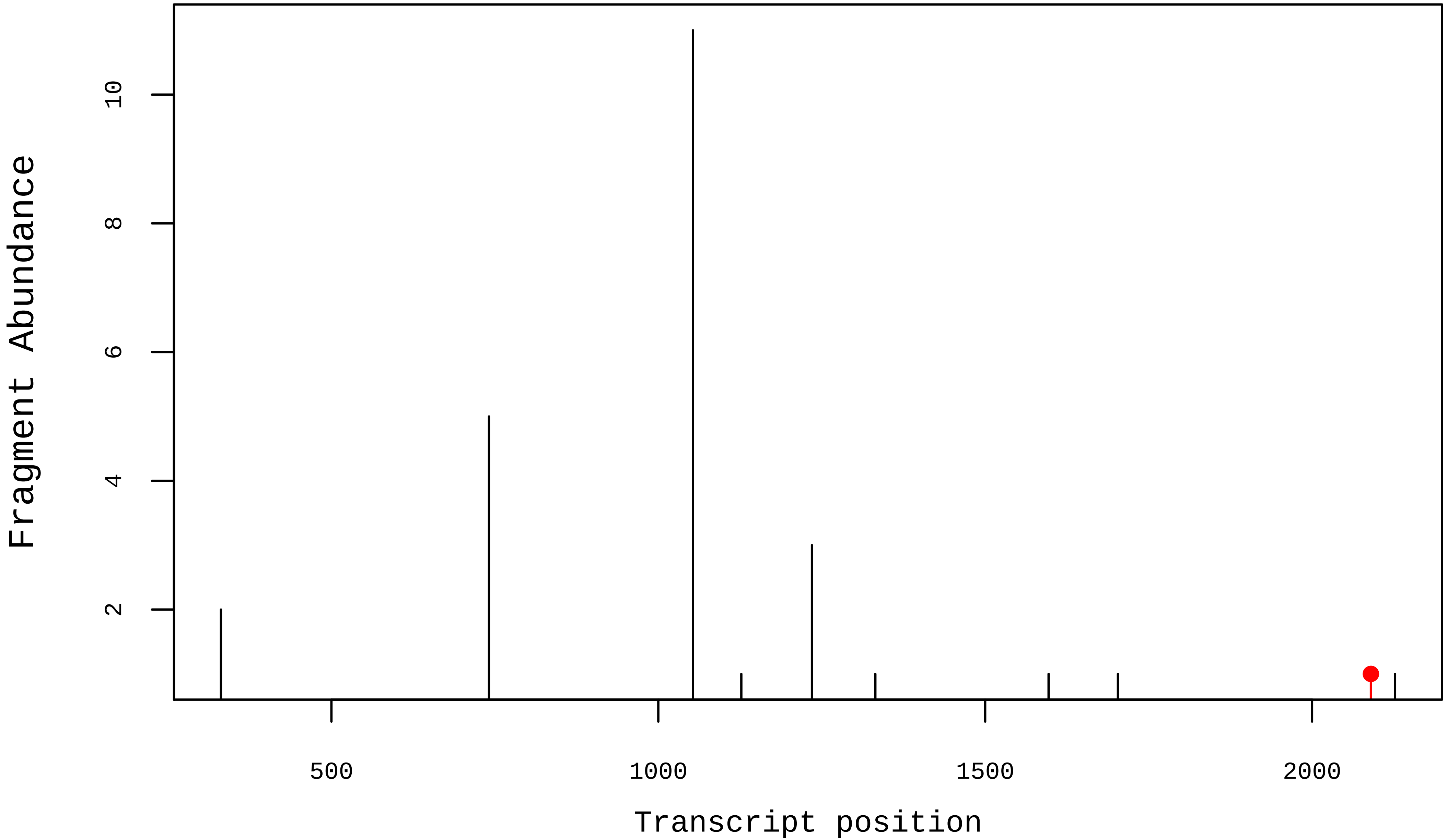

Cleavage site: 2090 Tag abundance: 1 Weighted abundance: 0.143 Category: 4
sRNA abundance: 1 Alignment score: 2 MFE ratio: 0.886 p-value: 0.019

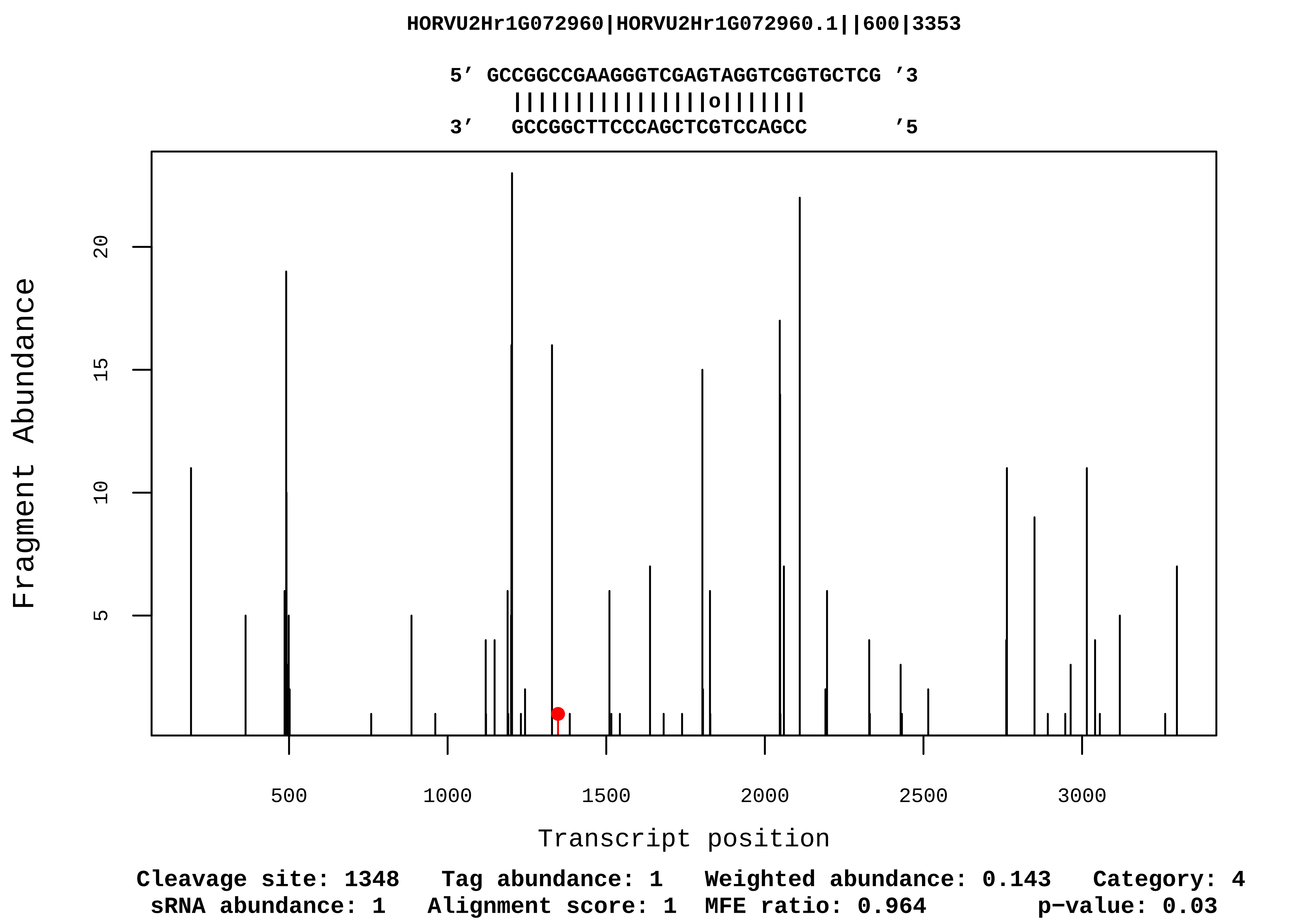

HORVU2Hr1G072960|HORVU2Hr1G072960.1||600|3353
5' GCCGGCCGAAGGGTCGAGTAGGTCGGTGCTCG '3
||||||||||||||||o|||||||
3' GCCGGCTTCCCAGCTCGTCCAGCC '5
Fragment Abundance
5
10
15
20
500
1000
1500
2000
2500
3000
Transcript position
Cleavage site: 1348 Tag abundance: 1 Weighted abundance: 0.143 Category: 4
sRNA abundance: 1 Alignment score: 1 MFE ratio: 0.964 p-value: 0.03

HORVU5Hr1G015600|HORVU5Hr1G015600.1||156|510

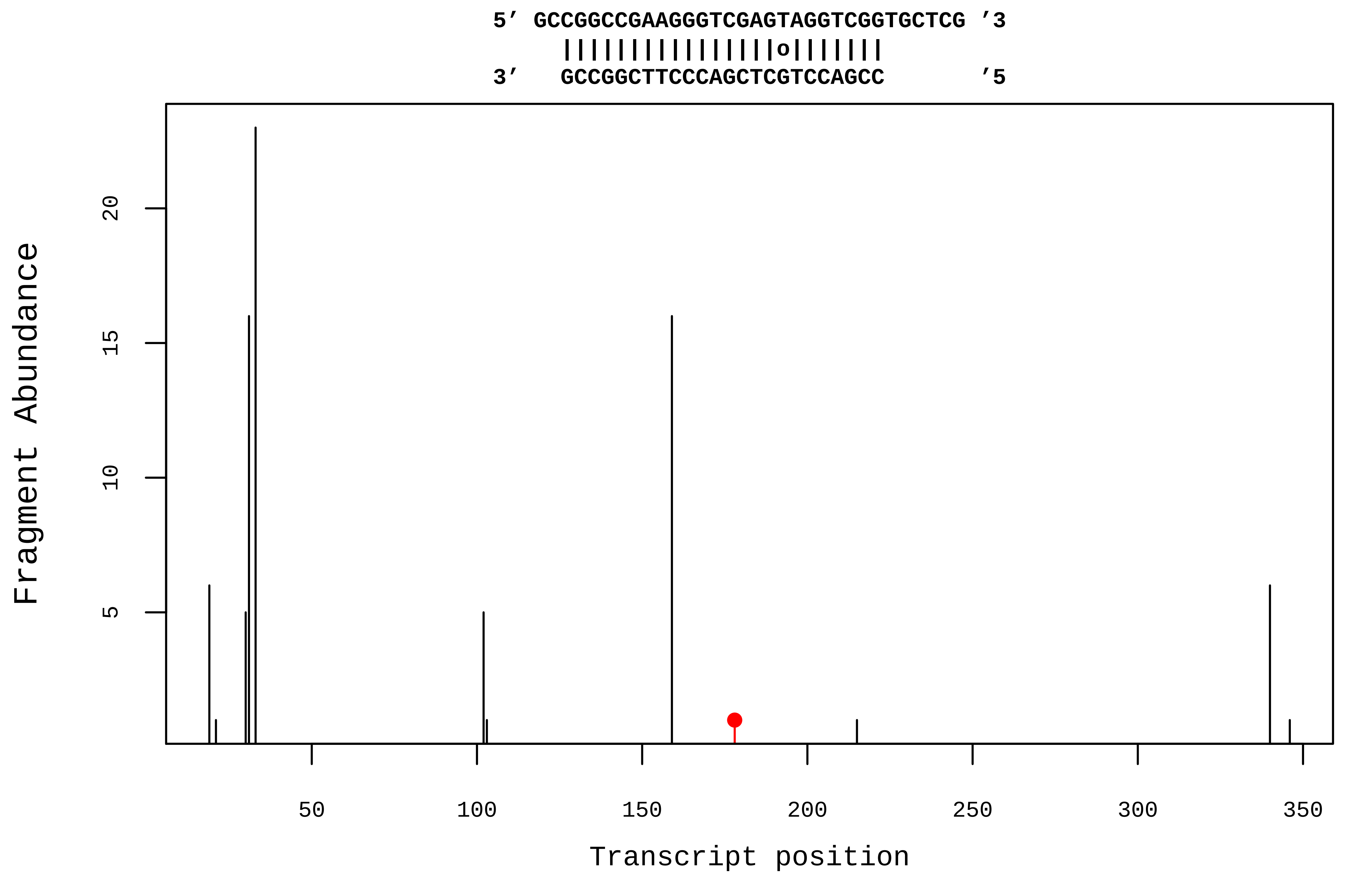

Cleavage site: 178 Tag abundance: 1 Weighted abundance: 0.143 Category: 4
sRNA abundance: 1 Alignment score: 1 MFE ratio: 0.964 p-value: 0.05

HORVU5Hr1G015600|HORVU5Hr1G015600.2||231|617

```
5' GCCGGCCGAAGGGTCGAGTAGGTCGGTGCTCG '3
      ||||||||||||||||o||||||||
3'   GCCGGCTTCCCAGCTCGTCCAGCC       '5
```

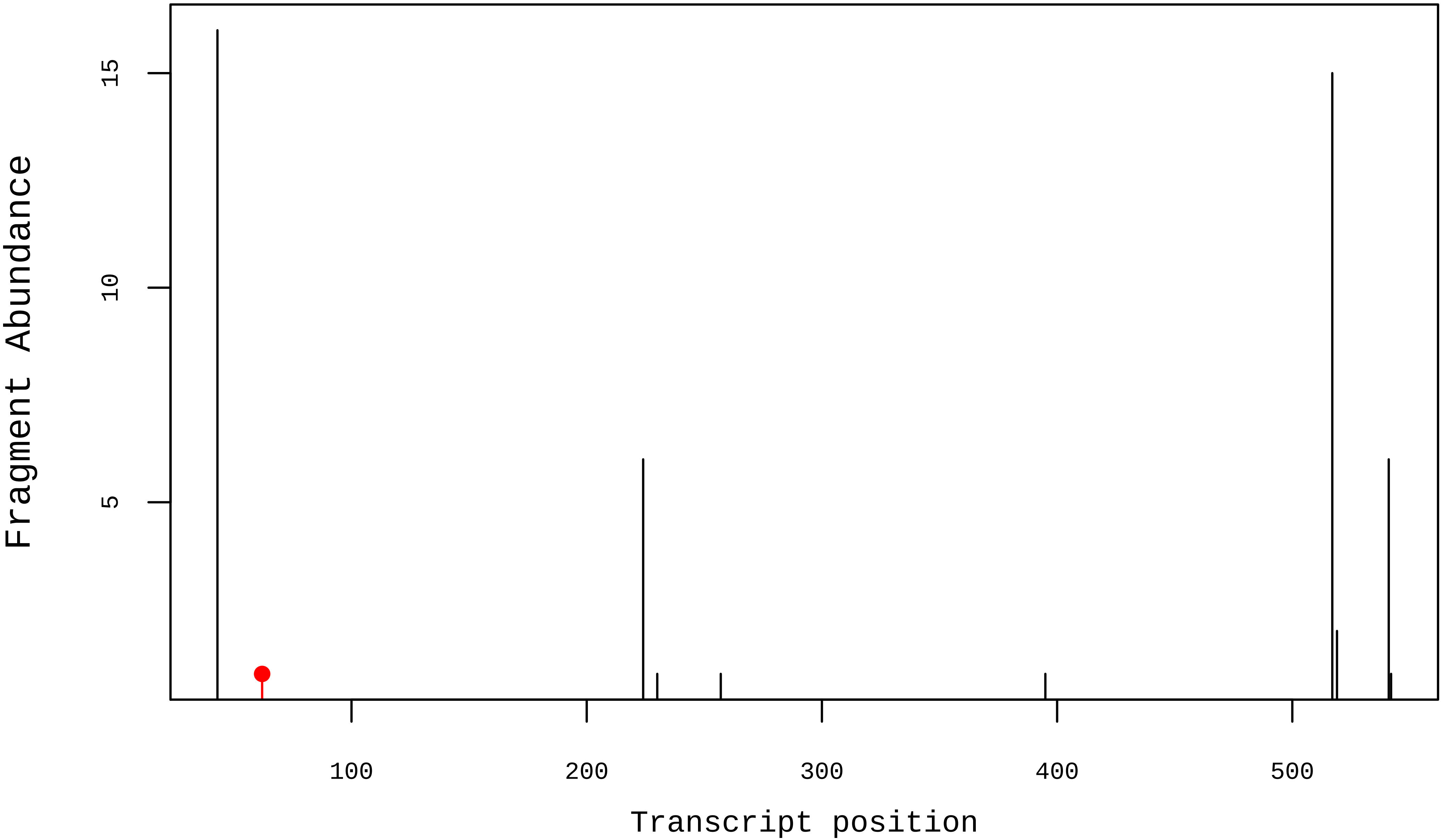

Cleavage site: 62   Tag abundance: 1   Weighted abundance: 0.143   Category: 4
sRNA abundance: 1   Alignment score: 1   MFE ratio: 0.964   p-value: 0.041

HORVU5Hr1G015600|HORVU5Hr1G015600.3||276|1709

```
5' GCCGGCCGAAGGGTCGAGTAGGTCGGTGCTCG '3
     ||||||||||||||||o||||||||
3'   GCCGGCTTCCCAGCTCGTCCAGCC       '5
```

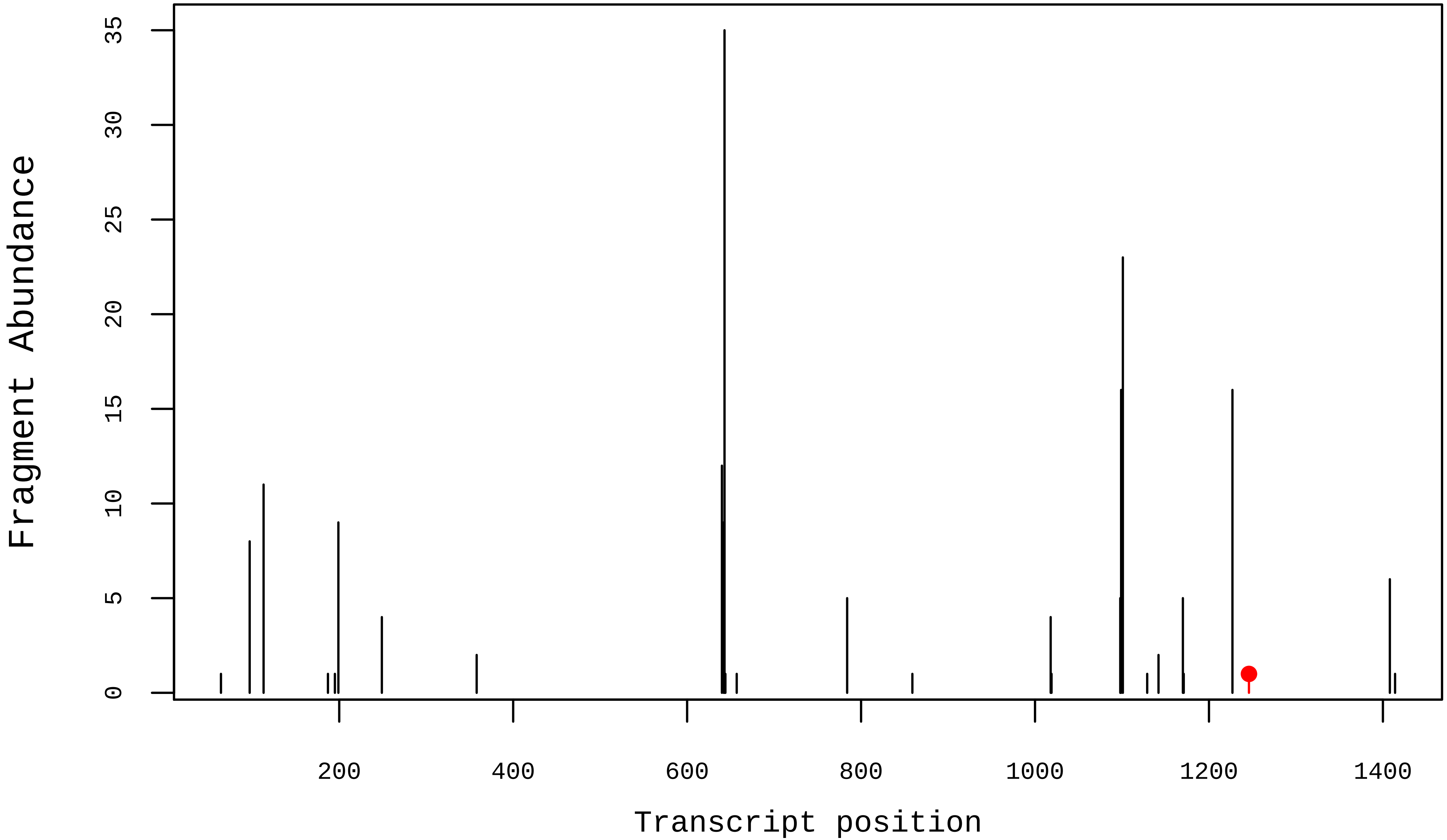

Cleavage site: 1246 Tag abundance: 1 Weighted abundance: 0.143 Category: 4
sRNA abundance: 1 Alignment score: 1 MFE ratio: 0.964 p-value: 0.032

HORVU5Hr1G015600|HORVU5Hr1G015600.4||207|1852

```
5' GCCGGCCGCAGGGTCGAGTAGGTCGGTGCTCG '3
      |||||| |||||||||o|||||||
3'    GCCGGCTTCCCAGCTCGTCCAGCC       '5
```

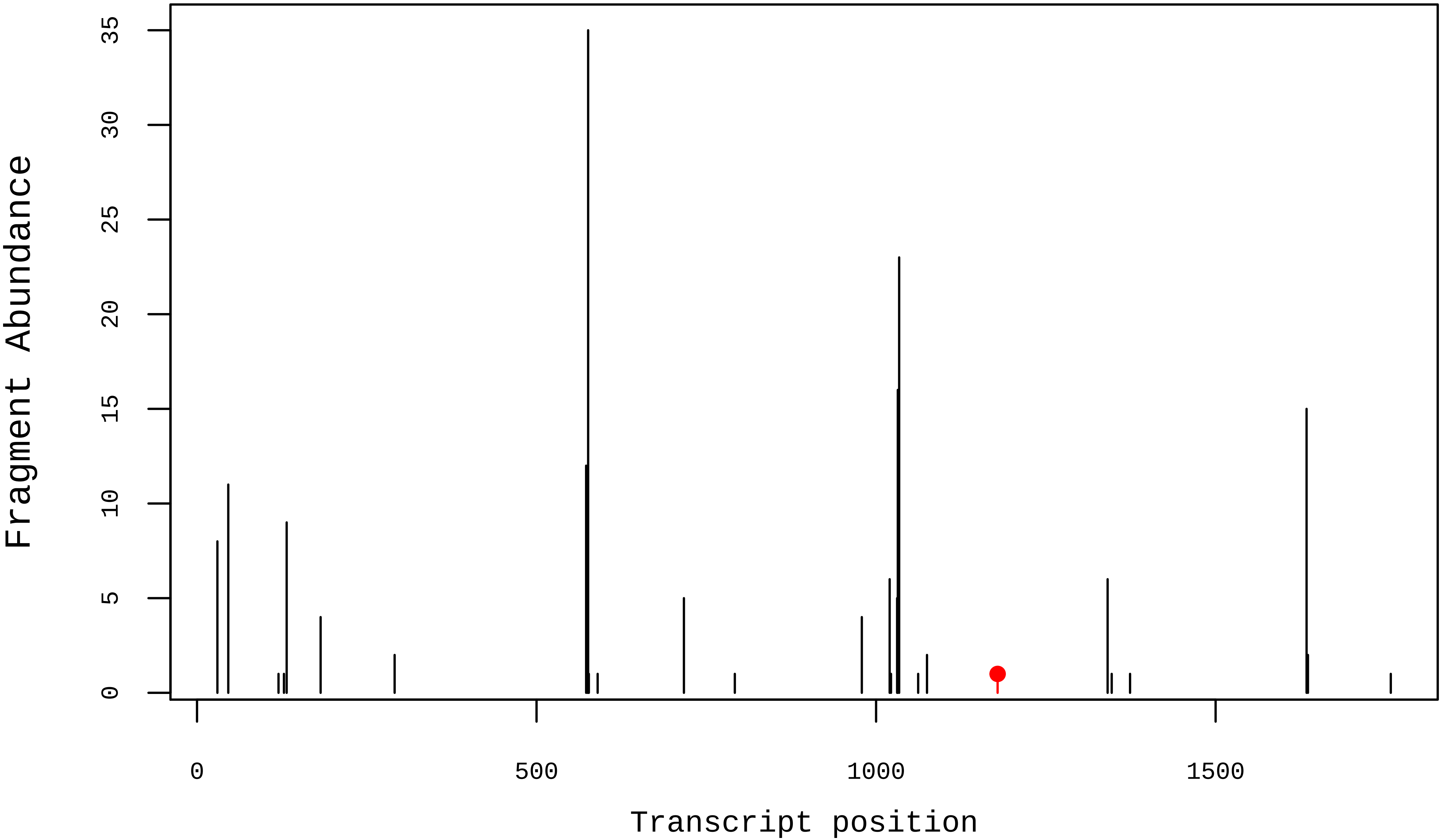

Cleavage site: 1179   Tag abundance: 1   Weighted abundance: 0.143   Category: 4
sRNA abundance: 1   Alignment score: 2   MFE ratio: 0.883   p-value: 0.041

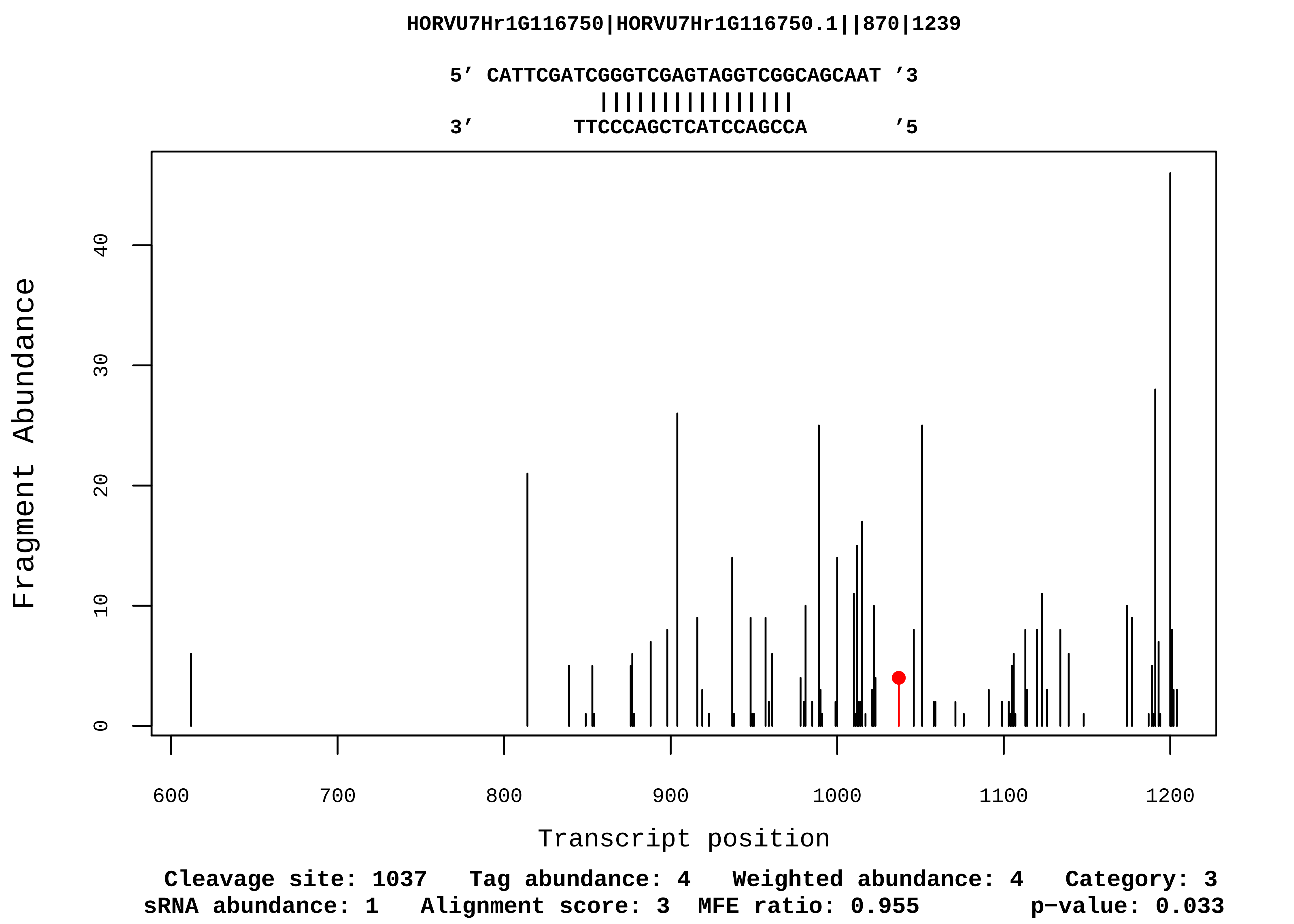

HORVU7Hr1G116750|HORVU7Hr1G116750.1||870|1239
5' CATTCGATCGGGTCGAGTAGGTCGGCAGCAAT '3
3' TTCCCAGCTCATCCAGCCA '5
Fragment Abundance
0
10
20
30
40
600
700
800
900
1000
1100
1200
Transcript position
Cleavage site: 1037 Tag abundance: 4 Weighted abundance: 4 Category: 3
sRNA abundance: 1 Alignment score: 3 MFE ratio: 0.955 p-value: 0.033

HORVU2Hr1G083660|HORVU2Hr1G083660.1||513|513

```
5' ATGGTGACGGG-CTTGGAGCCGAGGGAGCAGCG '3
           |||| |||||||||o|o|||
3'         GCCCGGAACCTCGGTTTCCT       '5
```

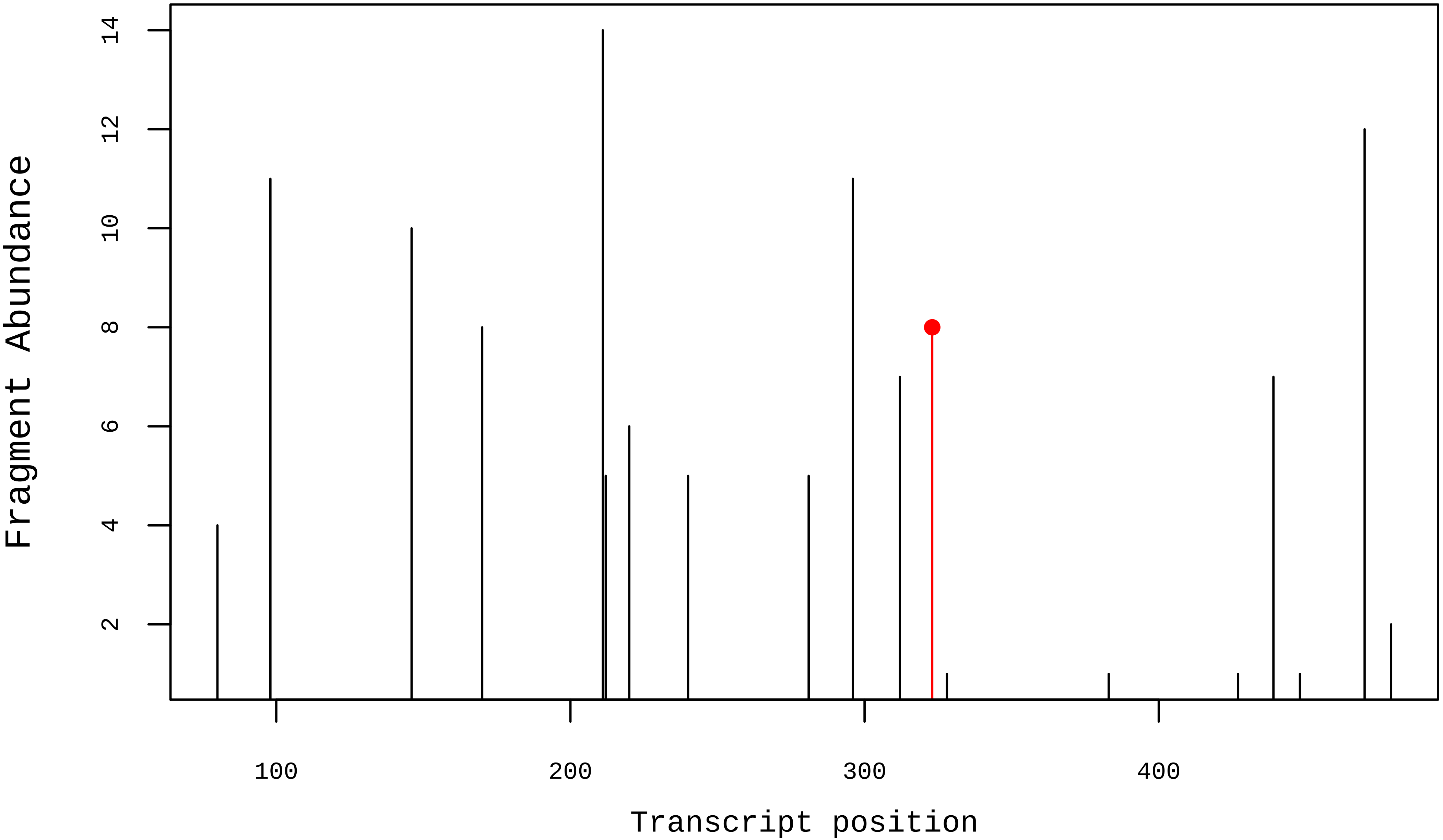

Cleavage site: 323   Tag abundance: 8   Weighted abundance: 4   Category: 2
sRNA abundance: 1   Alignment score: 3   MFE ratio: 0.816   p-value: 0.024

HORVU2Hr1G083680|HORVU2Hr1G083680.1||372|577

```
5' ATGGTGACGGG-CTTGGAGCCGAGGGAGCAGCG '3
          |||| |||||||||o|o|||
3'        GCCCGGAACCTCGGTTTCCT       '5
```

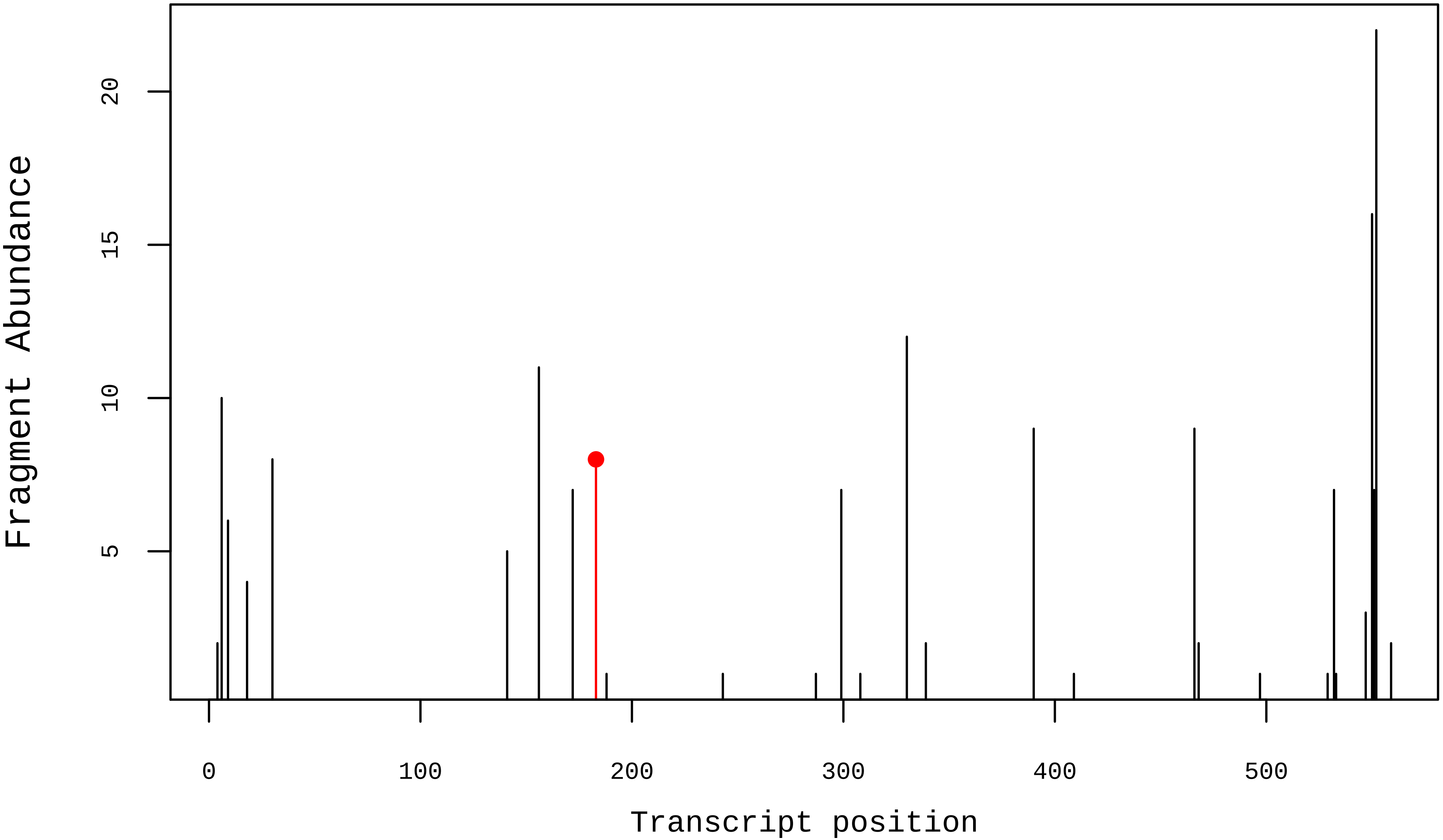

Cleavage site: 183   Tag abundance: 8   Weighted abundance: 4   Category: 2
sRNA abundance: 1   Alignment score: 3   MFE ratio: 0.816   p-value: 0.025

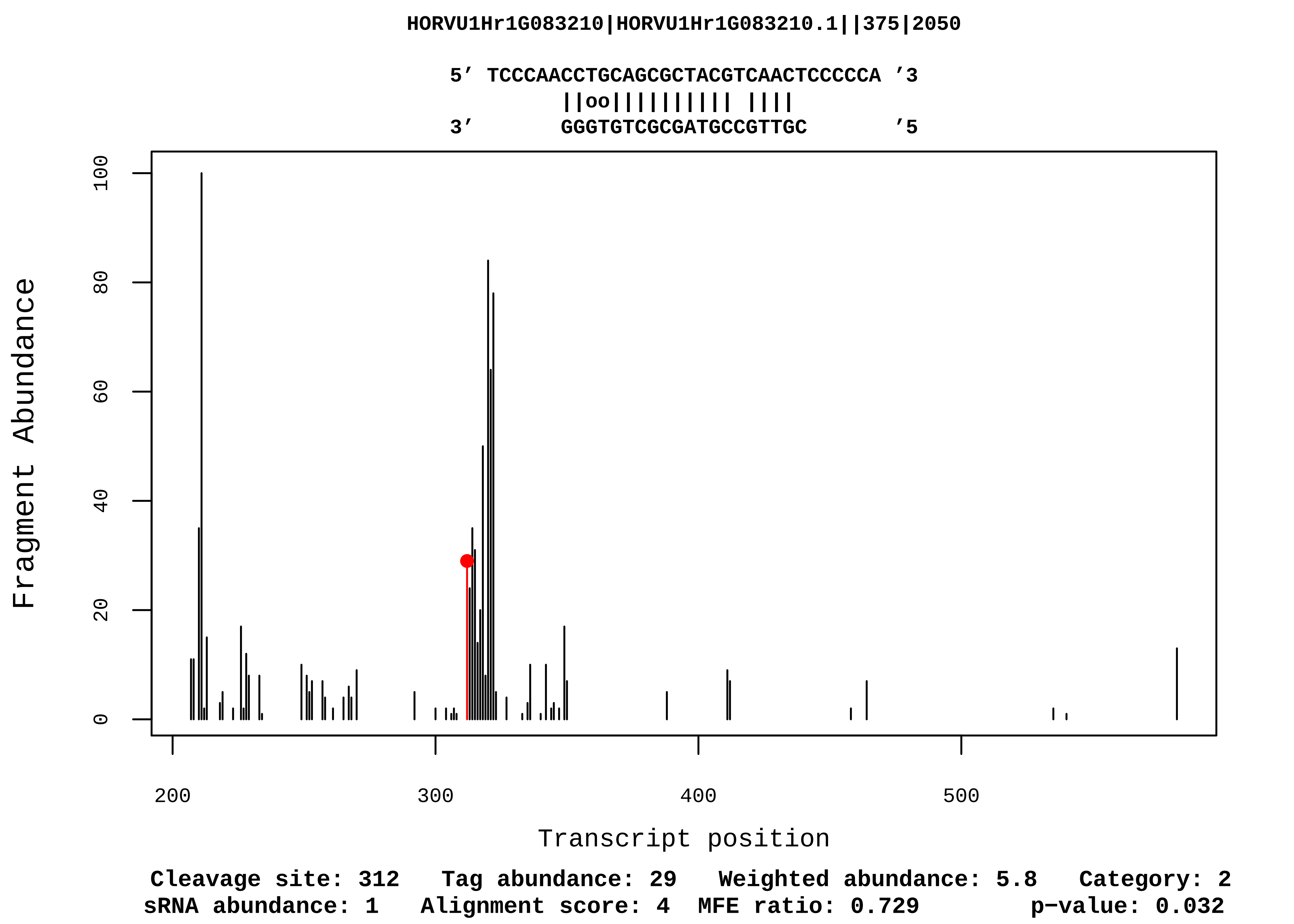

HORVU1Hr1G083210|HORVU1Hr1G083210.1||375|2050
5′ TCCCAACCTGCAGCGCTACGTCAACTCCCCCA ′3
||oo|||||||||| ||||
3′ GGGTGTCGCGATGCCGTTGC ′5
Fragment Abundance
0
20
40
60
80
100
200
300
400
500
Transcript position
Cleavage site: 312 Tag abundance: 29 Weighted abundance: 5.8 Category: 2
sRNA abundance: 1 Alignment score: 4 MFE ratio: 0.729 p-value: 0.032

HORVU7Hr1G024570|HORVU7Hr1G024570.2||432|4386

```
5' GCCCGAGCCCACC-CGCTACGGCGACTGGGAGC '3
             |||||  |||||||||o||
3'           GGGTGTCGCGATGCCGTTGC       '5
```

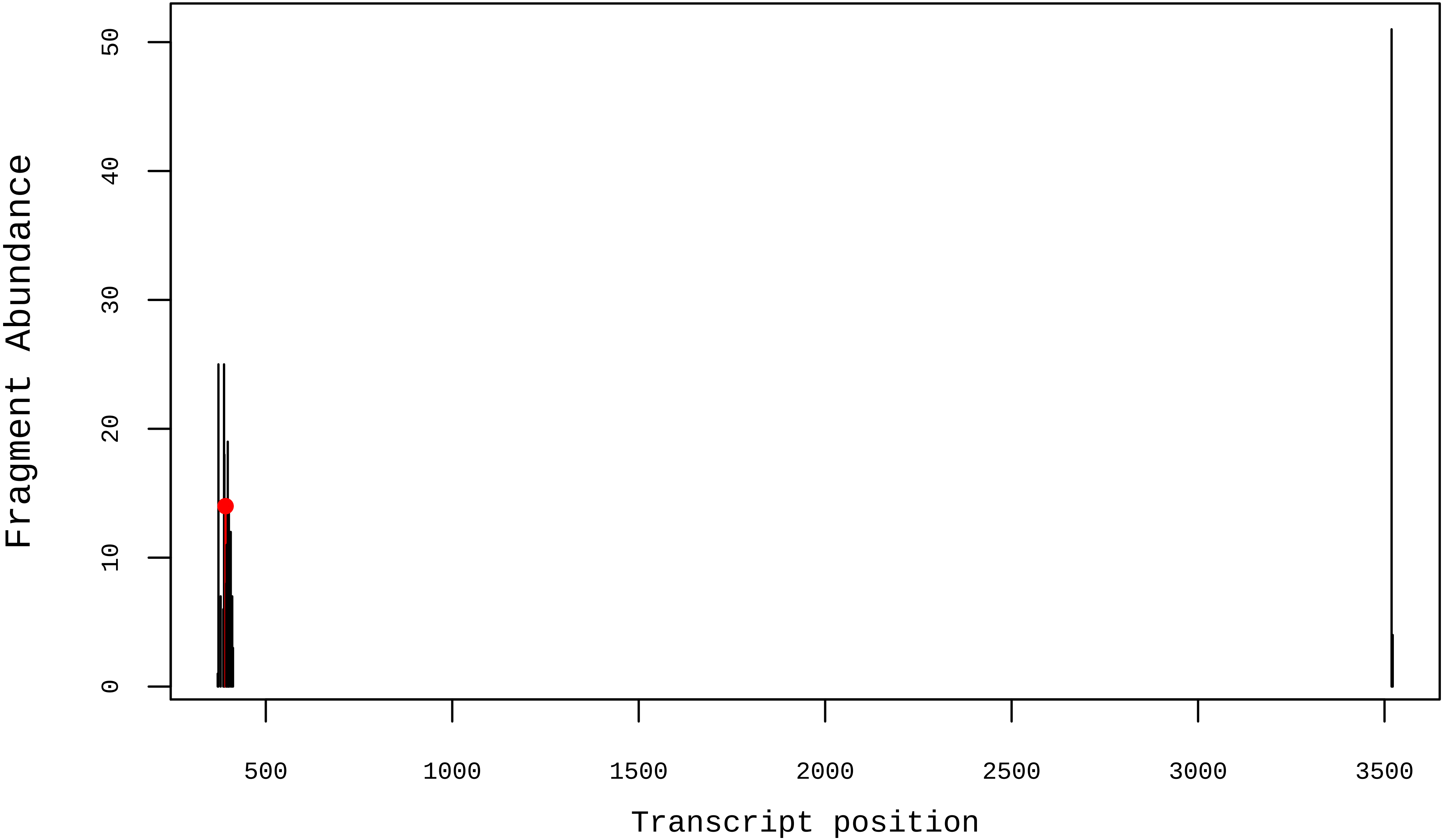

Cleavage site: 392   Tag abundance: 14   Weighted abundance: 4.667   Category: 2
sRNA abundance: 1   Alignment score: 4   MFE ratio: 0.762   p-value: 0.007

HORVU5Hr1G063430|HORVU5Hr1G063430.8||2820|2820

```
5' CAAGAGG-GTGGGTGCAGCCGACGAGCGCCGCG '3
         || ||| |o|||||||||||
3'       CCACACACGCGTCGGCTGCTCC       '5
```

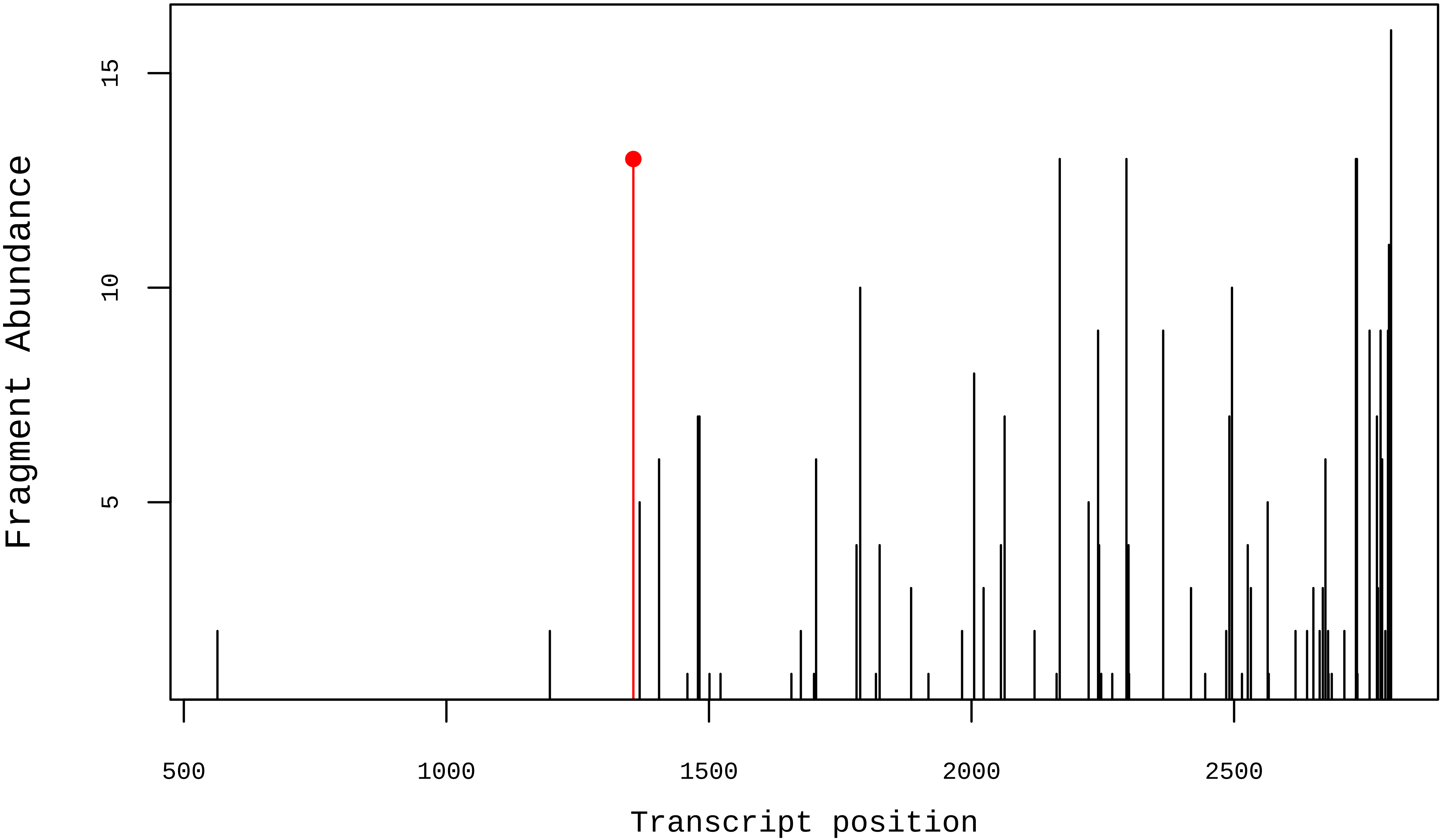

Cleavage site: 1356 Tag abundance: 13 Weighted abundance: 2.167 Category: 2
sRNA abundance: 1 Alignment score: 3.5 MFE ratio: 0.706 p-value: 0.048

HORVU5Hr1G063430|HORVU5Hr1G063430.9||1848|1850

```
5′ CAAGAGG-GTGGGTGCAGCCGACGAGCGCCGCG ′3
           || ||| |o||||||||||||
3′         CCACACACGCGTCGGCTGCTCC         ′5
```

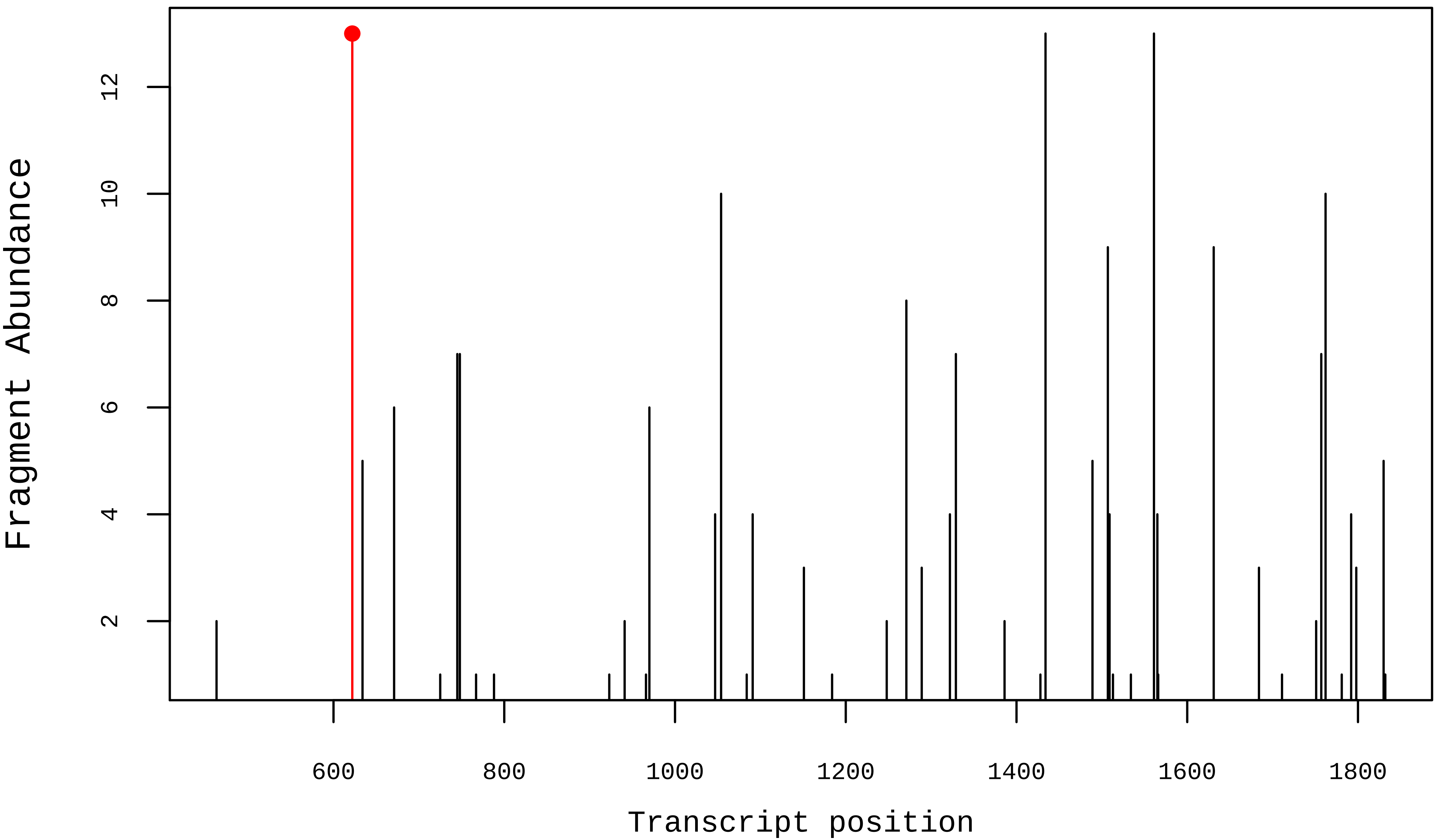

Cleavage site: 622 Tag abundance: 13 Weighted abundance: 2.167 Category: 1
sRNA abundance: 1 Alignment score: 3.5 MFE ratio: 0.706 p-value: 0.01

HORVU3Hr1G068830|HORVU3Hr1G068830.1||513|790

```
5' TGACGCTCCCCGAC-GGGCTGCAGGCGGTCTAC '3
                || || |o|||o||||||
3'              GGGTGACTCGATGTCCGC       '5
```

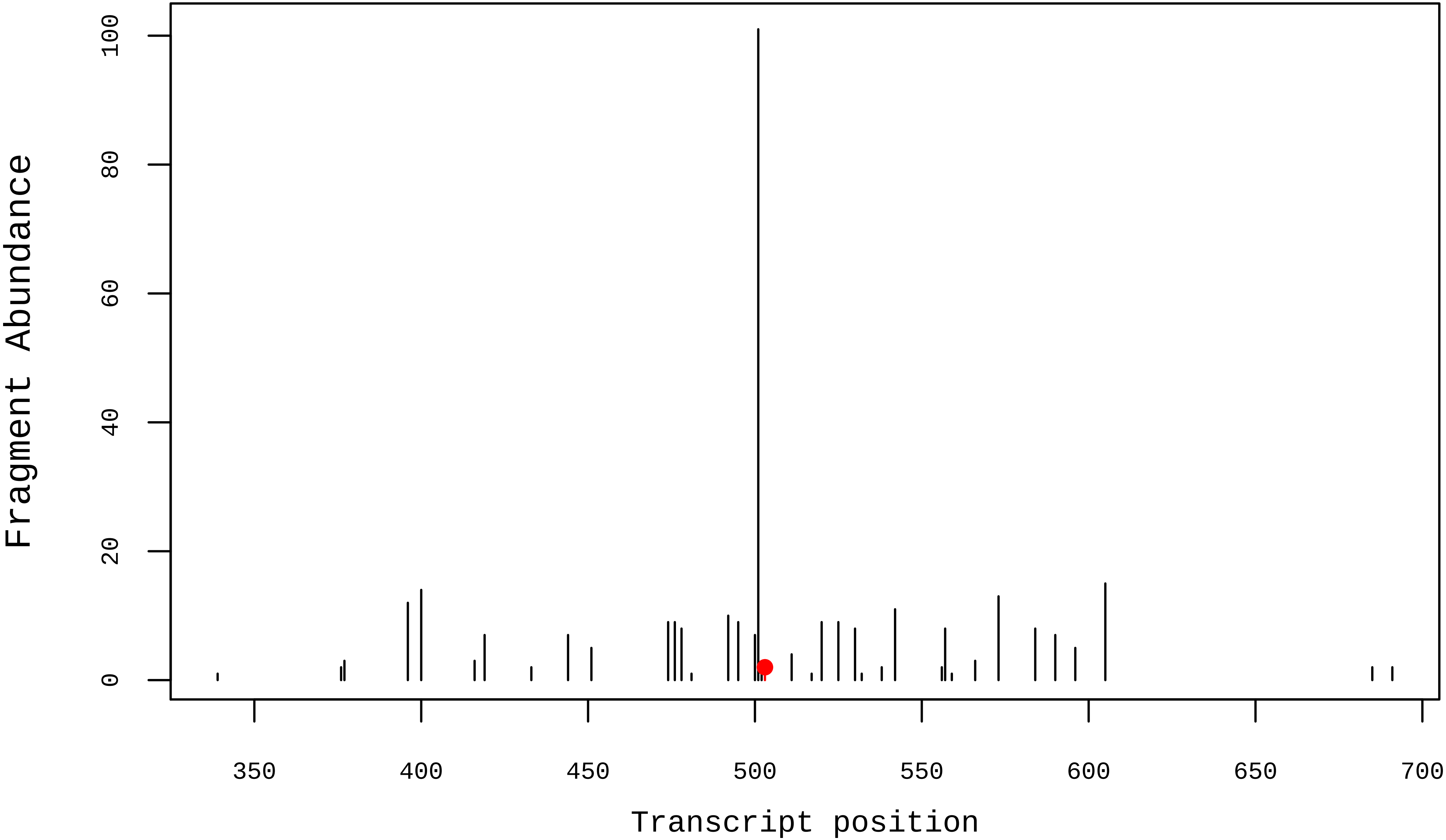

Cleavage site: 503   Tag abundance: 2   Weighted abundance: 2   Category: 3
sRNA abundance: 1   Alignment score: 4   MFE ratio: 0.735   p-value: 0.034

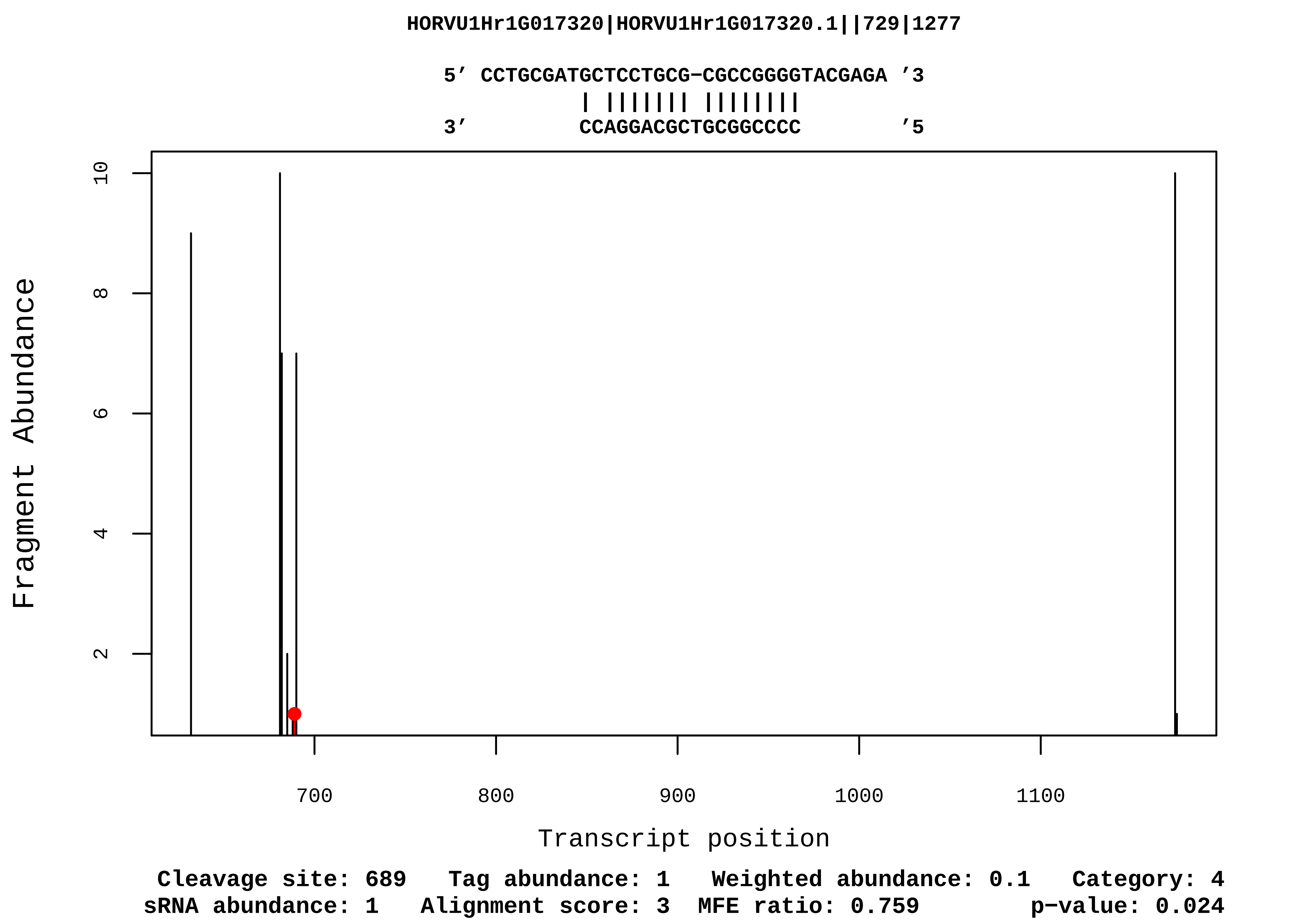

HORVU1Hr1G017320|HORVU1Hr1G017320.1||729|1277
5' CCTGCGATGCTCCTGCG-CGCCGGGGTACGAGA '3
| |||||||| |||||||||
3' CCAGGACGCTGCGGCCCC '5
Fragment Abundance
2
4
6
8
10
700
800
900
1000
1100
Transcript position
Cleavage site: 689 Tag abundance: 1 Weighted abundance: 0.1 Category: 4
sRNA abundance: 1 Alignment score: 3 MFE ratio: 0.759 p-value: 0.024

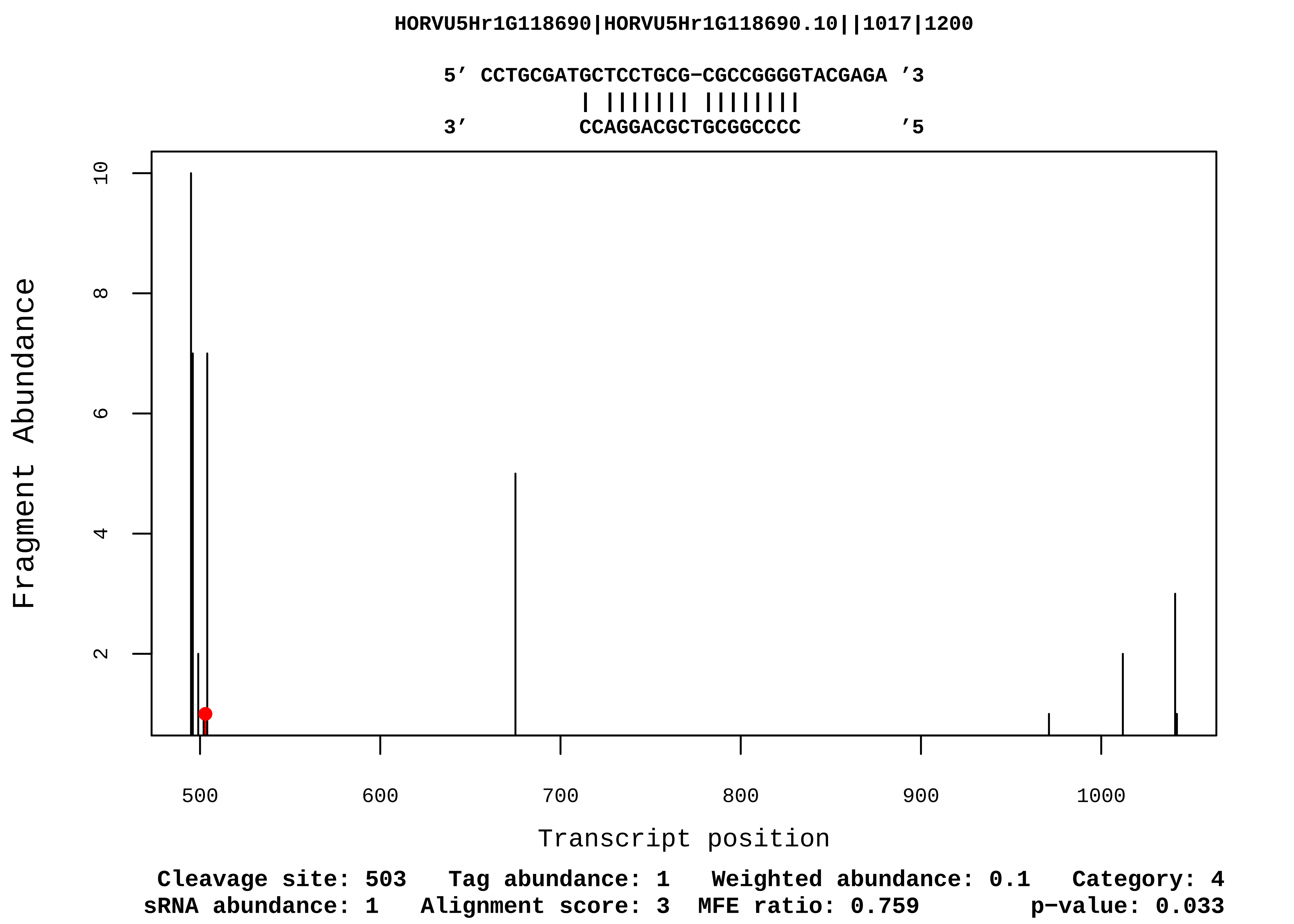
HORVU5Hr1G118690|HORVU5Hr1G118690.10||1017|1200
5′ CCTGCGATGCTCCTGCG−CGCCGGGGTACGAGA ′3
3′ CCAGGACGCTGCGGCCCC ′5
Fragment Abundance
Transcript position
10
8
6
4
2
500
600
700
800
900
1000
Cleavage site: 503 Tag abundance: 1 Weighted abundance: 0.1 Category: 4
sRNA abundance: 1 Alignment score: 3 MFE ratio: 0.759 p-value: 0.033

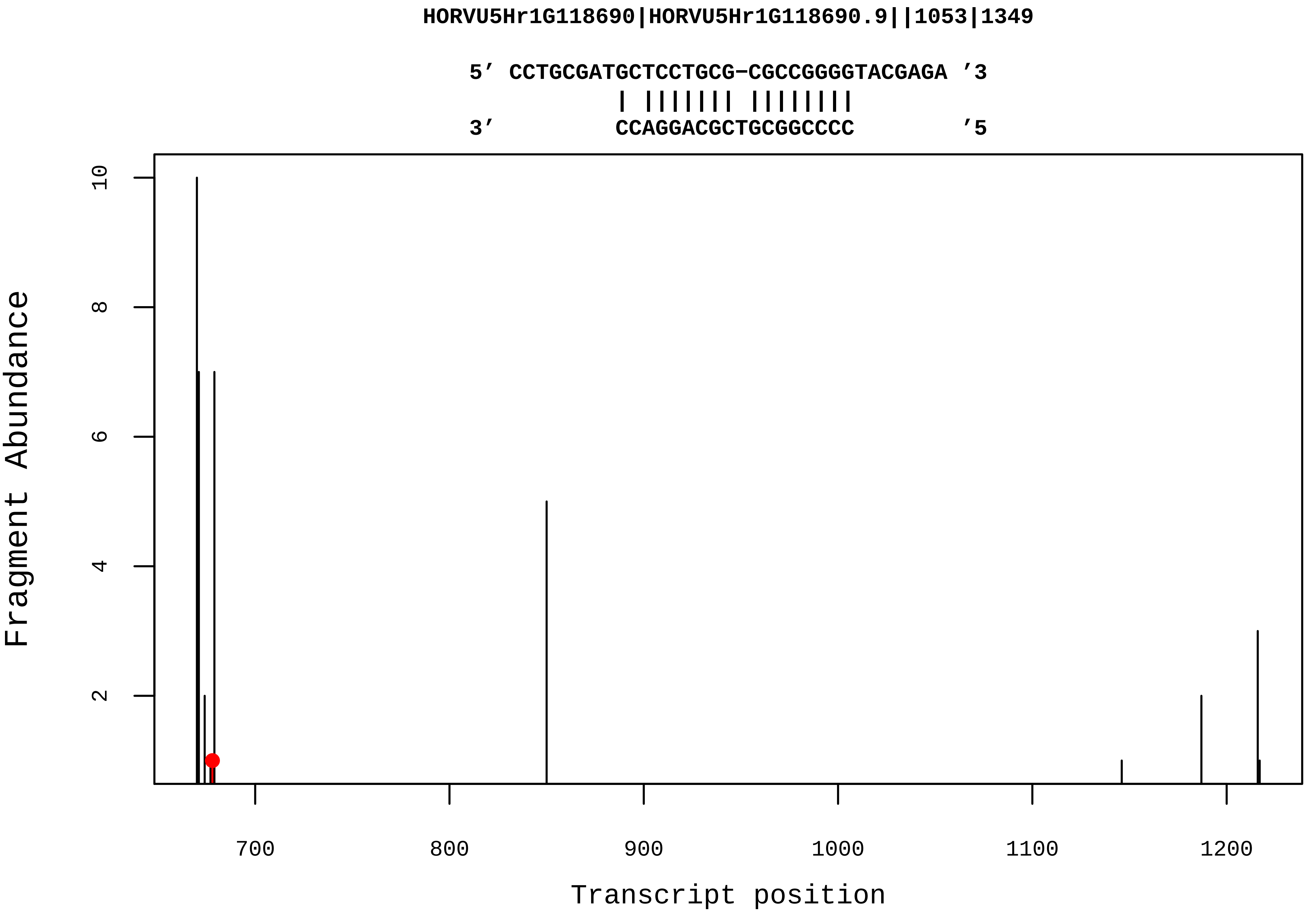

Cleavage site: 678 Tag abundance: 1 Weighted abundance: 0.1 Category: 4
sRNA abundance: 1 Alignment score: 3 MFE ratio: 0.759 p-value: 0.03

HORVU0Hr1G003660|HORVU0Hr1G003660.1||297|1955

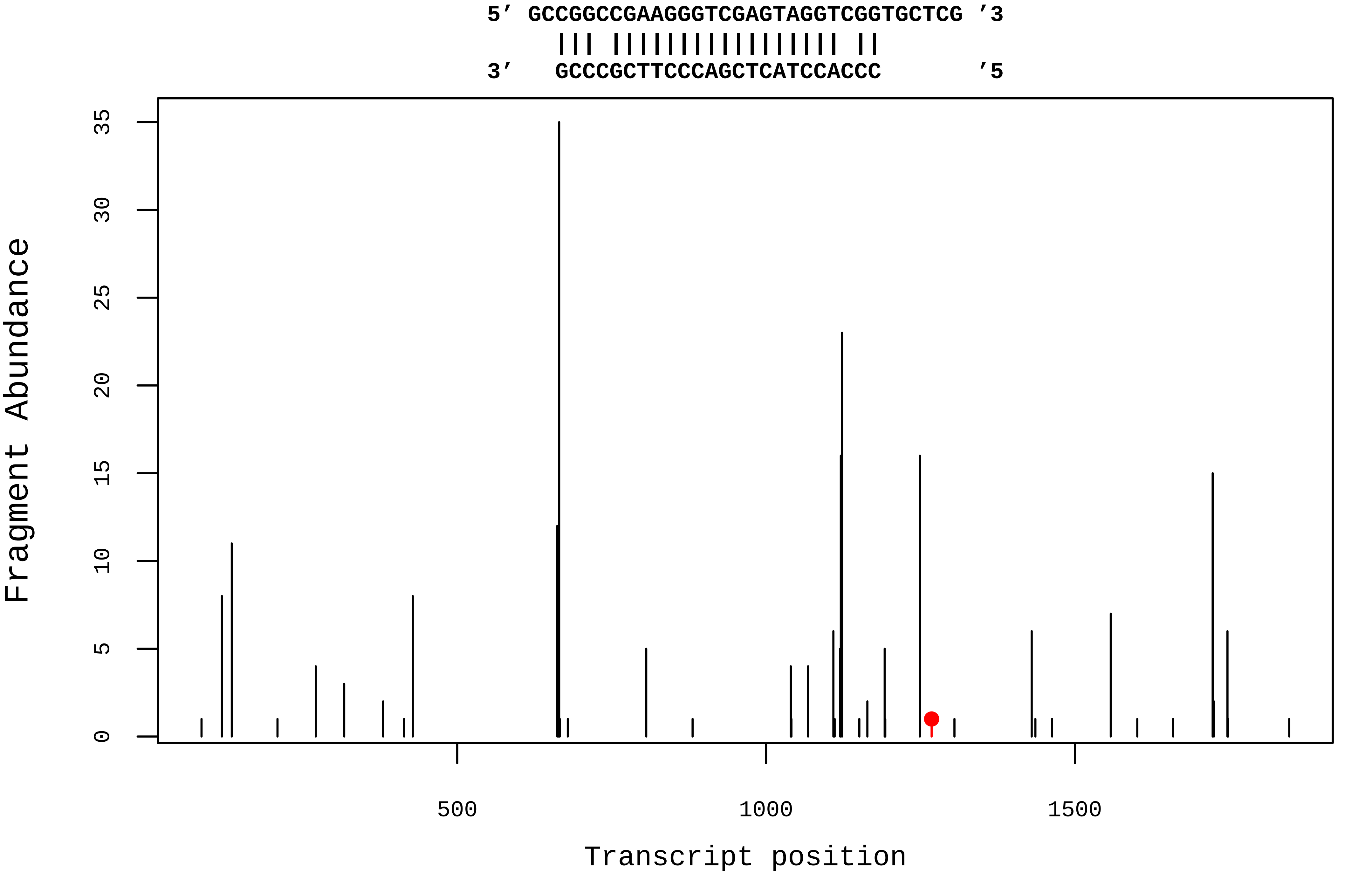

Cleavage site: 1268 Tag abundance: 1 Weighted abundance: 0.143 Category: 4
sRNA abundance: 1 Alignment score: 3 MFE ratio: 0.744 p-value: 0.046

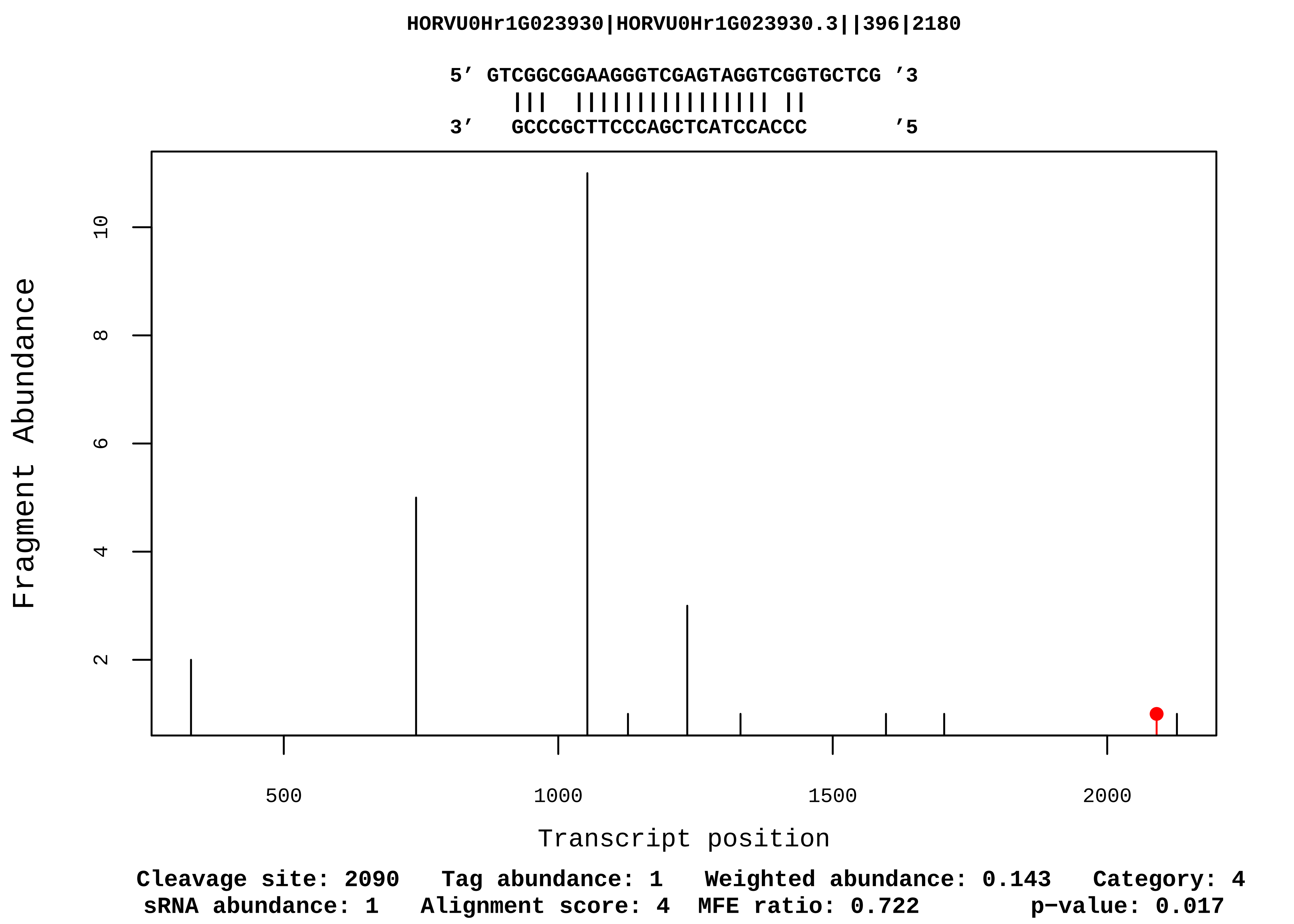

HORVU0Hr1G023930|HORVU0Hr1G023930.3||396|2180
5' GTCGGCGGAAGGGTCGAGTAGGTCGGTGCTCG '3
||| ||||||||||||||||| ||
3' GCCCGCTTCCCAGCTCATCCACCC '5
Fragment Abundance
2
4
6
8
10
500
1000
1500
2000
Transcript position
Cleavage site: 2090 Tag abundance: 1 Weighted abundance: 0.143 Category: 4
sRNA abundance: 1 Alignment score: 4 MFE ratio: 0.722 p-value: 0.017

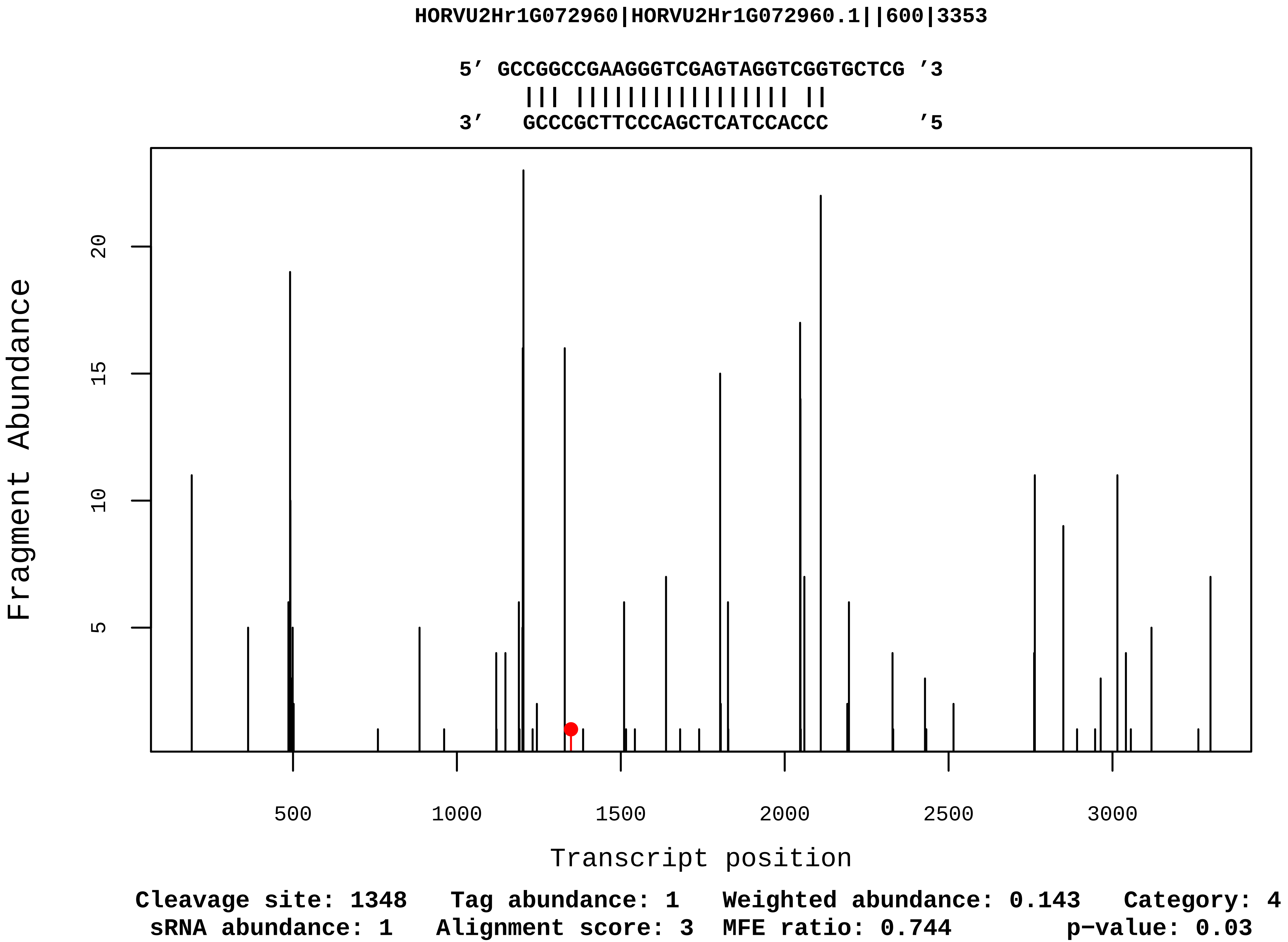
HORVU2Hr1G072960|HORVU2Hr1G072960.1||600|3353
5′ GCCGGCCGAAGGGTCGAGTAGGTCGGTGCTCG ′3
3′ GCCCGCTTCCCAGCTCATCCACCC ′5
Fragment Abundance
5
10
15
20
500
1000
1500
2000
2500
3000
Transcript position
Cleavage site: 1348 Tag abundance: 1 Weighted abundance: 0.143 Category: 4
sRNA abundance: 1 Alignment score: 3 MFE ratio: 0.744 p−value: 0.03

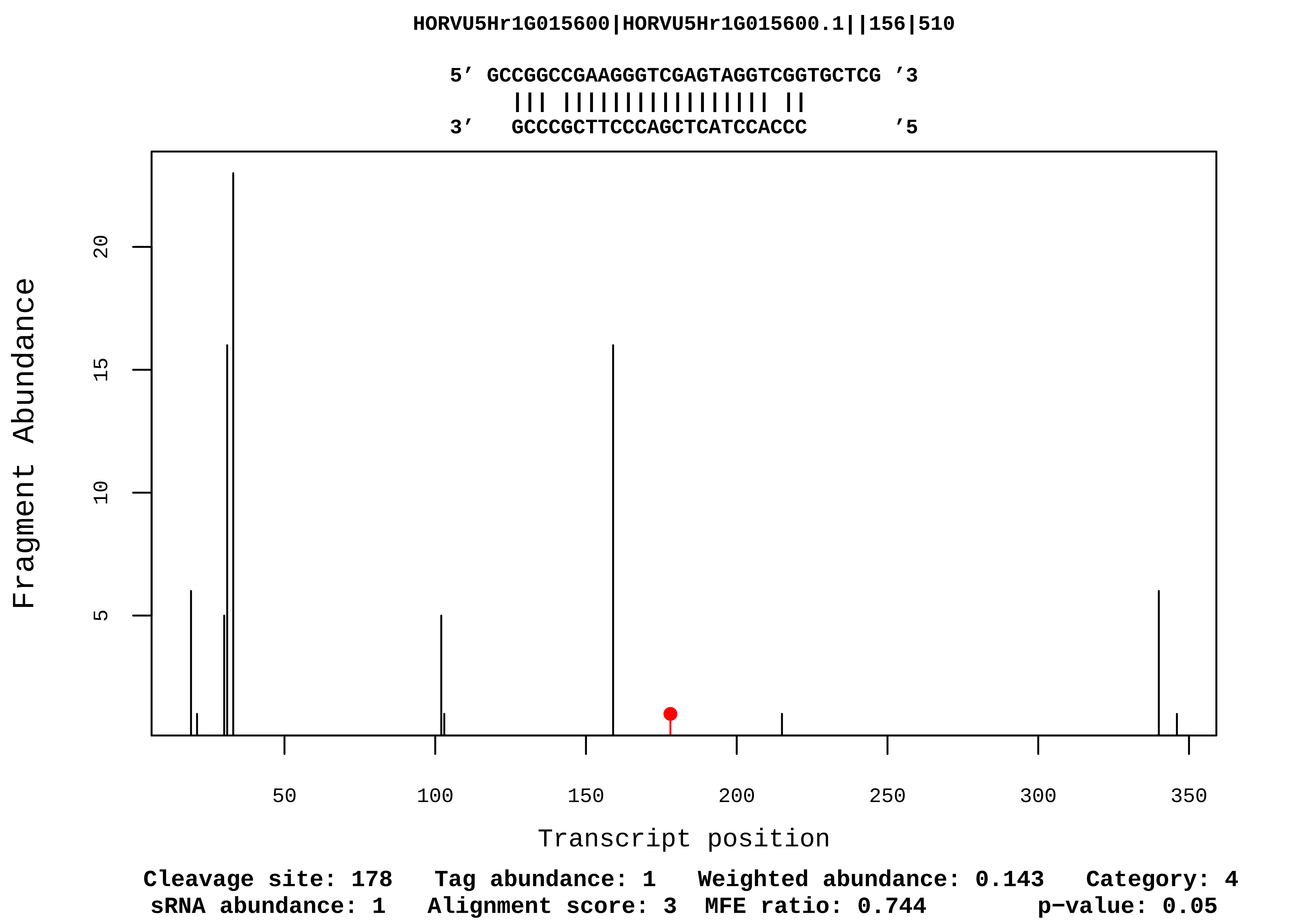

HORVU5Hr1G015600|HORVU5Hr1G015600.1||156|510
5' GCCGGCCGAAGGGTCGAGTAGGTCGGTGCTCG '3
||| ||||||||||||||||| ||
3'   GCCCGCTTCCCAGCTCATCCACCC   '5
Fragment Abundance
5
10
15
20
50
100
150
200
250
300
350
Transcript position
Cleavage site: 178   Tag abundance: 1   Weighted abundance: 0.143   Category: 4
sRNA abundance: 1   Alignment score: 3   MFE ratio: 0.744   p-value: 0.05

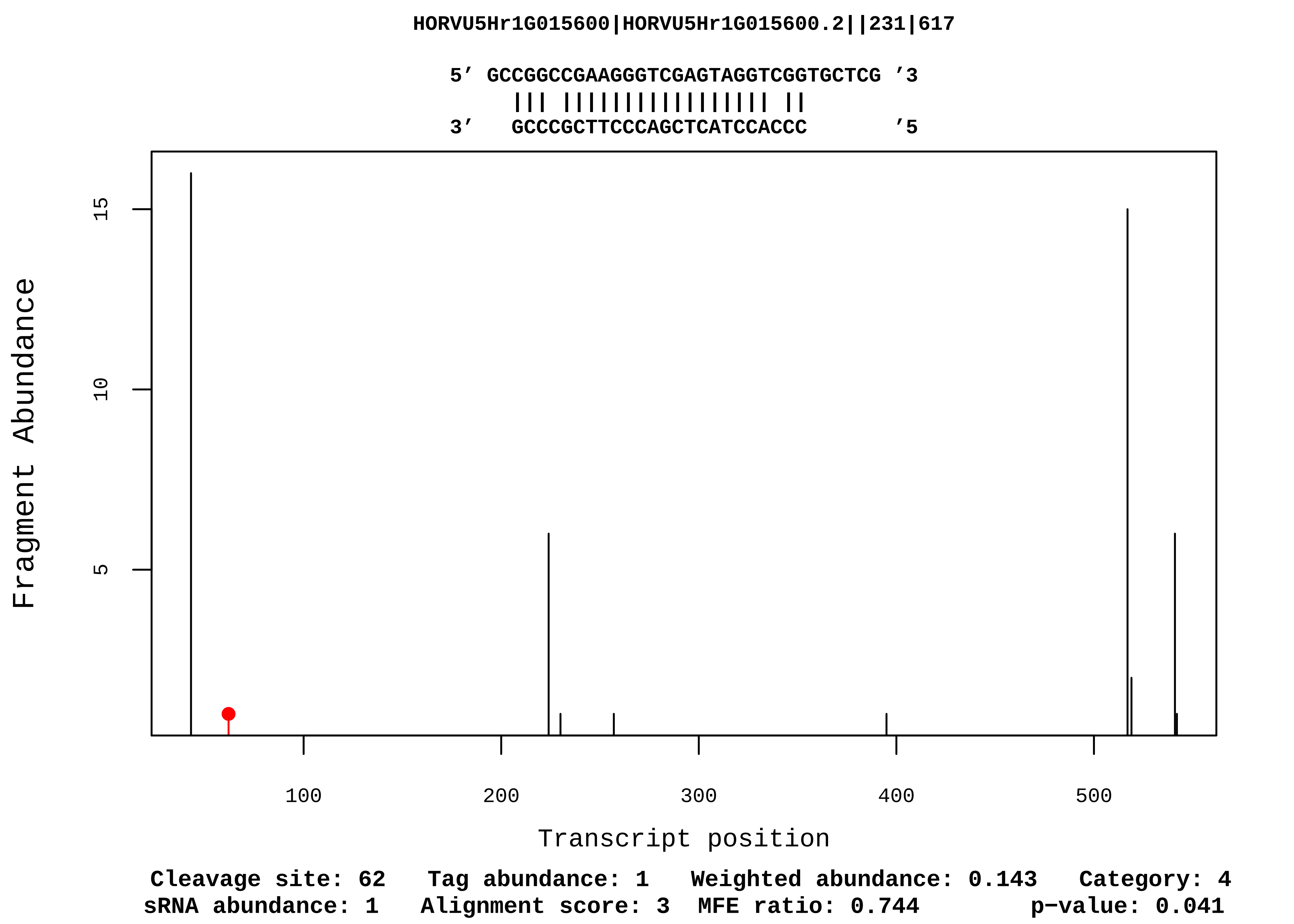
HORVU5Hr1G015600|HORVU5Hr1G015600.2||231|617
5' GCCGGCCGAAGGGTCGAGTAGGTCGGTGCTCG '3
3' GCCCGCTTCCCAGCTCATCCACCC '5
Fragment Abundance
15
10
5
100
200
300
400
500
Transcript position
Cleavage site: 62 Tag abundance: 1 Weighted abundance: 0.143 Category: 4
sRNA abundance: 1 Alignment score: 3 MFE ratio: 0.744 p-value: 0.041

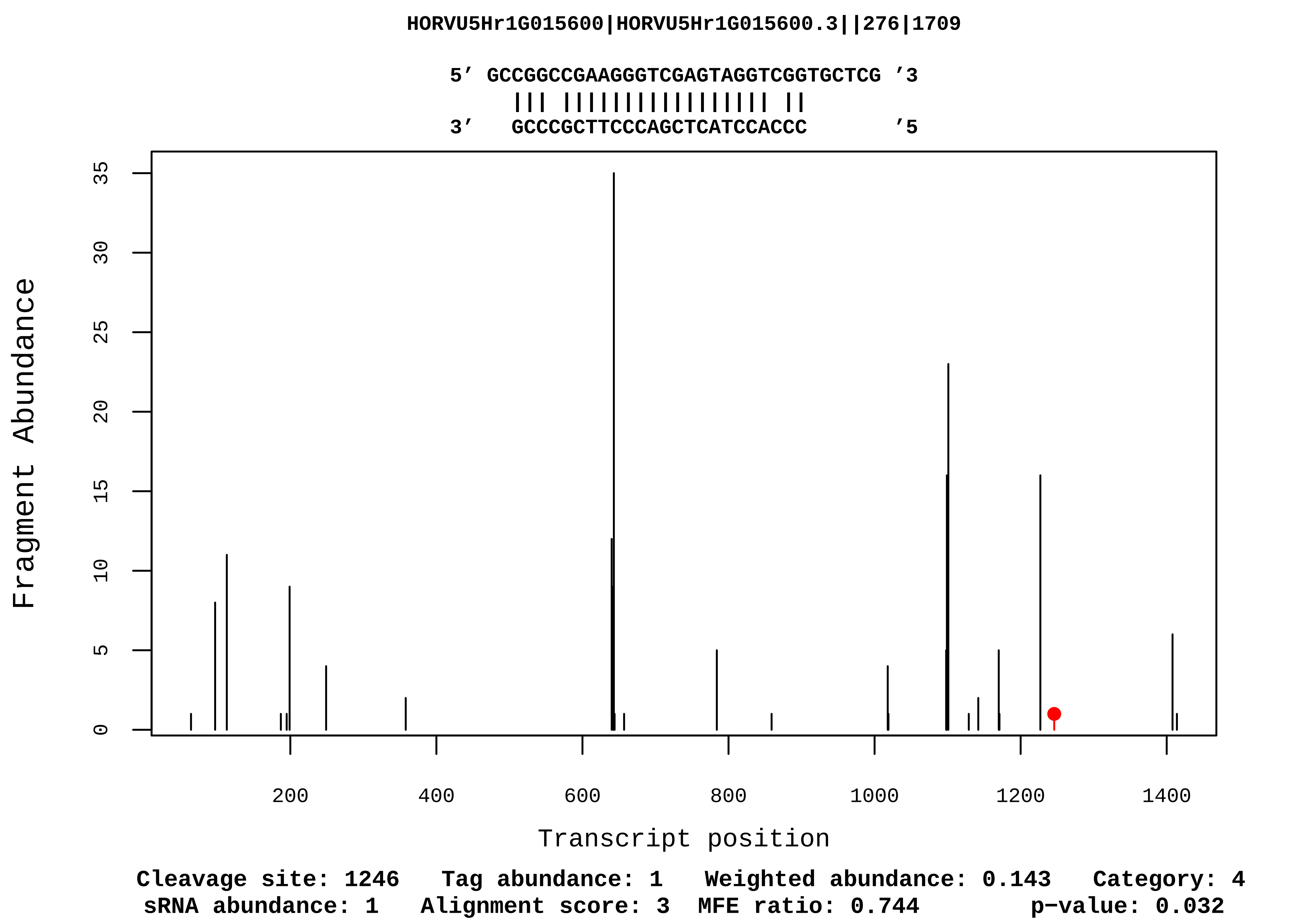

HORVU5Hr1G015600|HORVU5Hr1G015600.3||276|1709
5′ GCCGGCCGAAGGGTCGAGTAGGTCGGTGCTCG ′3
||| ||||||||||||||||| ||
3′ GCCCGCTTCCCAGCTCATCCACCC ′5
Fragment Abundance
0
5
10
15
20
25
30
35
200
400
600
800
1000
1200
1400
Transcript position
Cleavage site: 1246 Tag abundance: 1 Weighted abundance: 0.143 Category: 4
sRNA abundance: 1 Alignment score: 3 MFE ratio: 0.744 p-value: 0.032

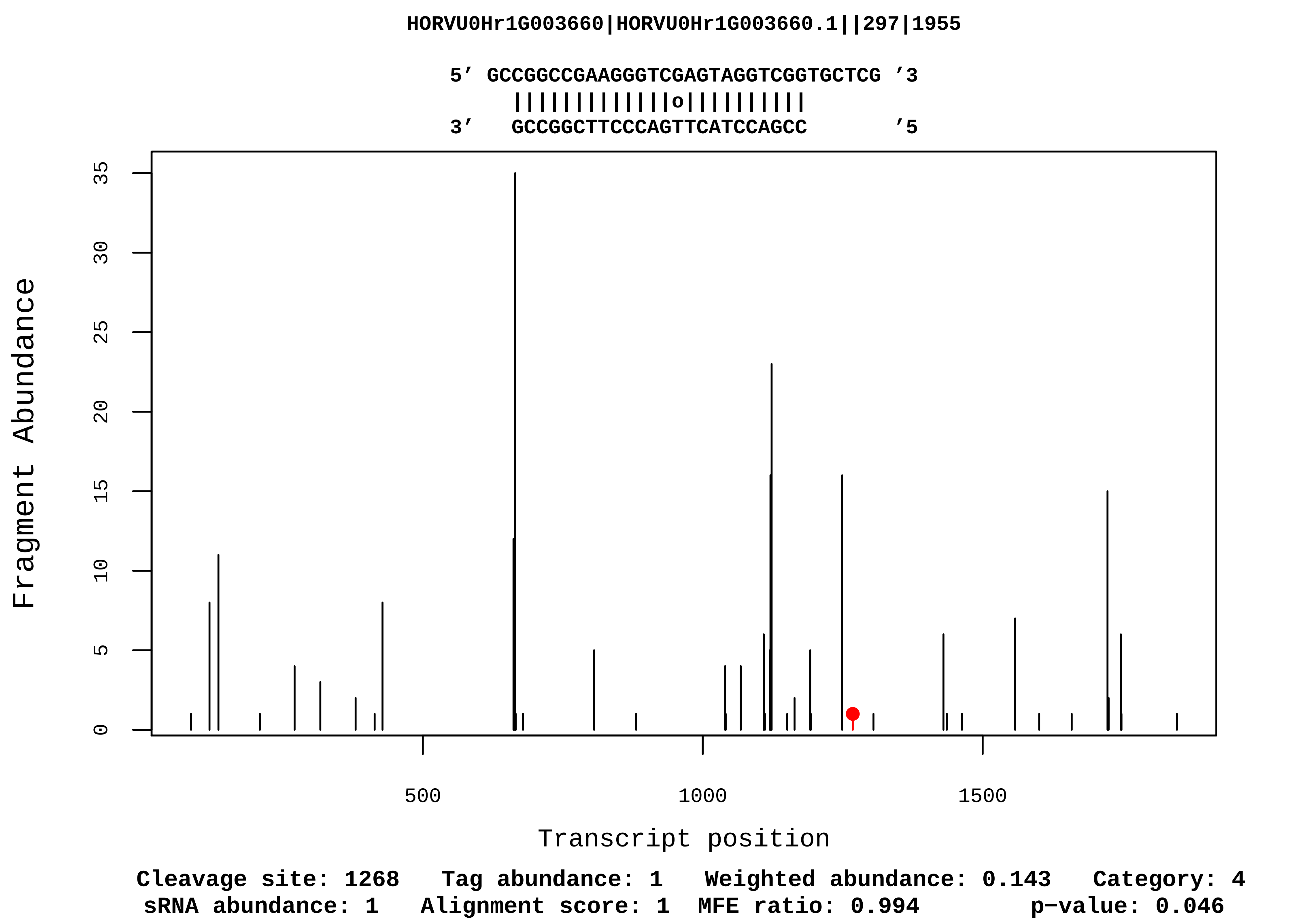
HORVU0Hr1G003660|HORVU0Hr1G003660.1||297|1955
5' GCCGGCCGAAGGGTCGAGTAGGTCGGTGCTCG '3
|||||||||||||o||||||||||
3' GCCGGCTTCCCAGTTCATCCAGCC '5
Fragment Abundance
0
5
10
15
20
25
30
35
500
1000
1500
Transcript position
Cleavage site: 1268 Tag abundance: 1 Weighted abundance: 0.143 Category: 4
sRNA abundance: 1 Alignment score: 1 MFE ratio: 0.994 p-value: 0.046

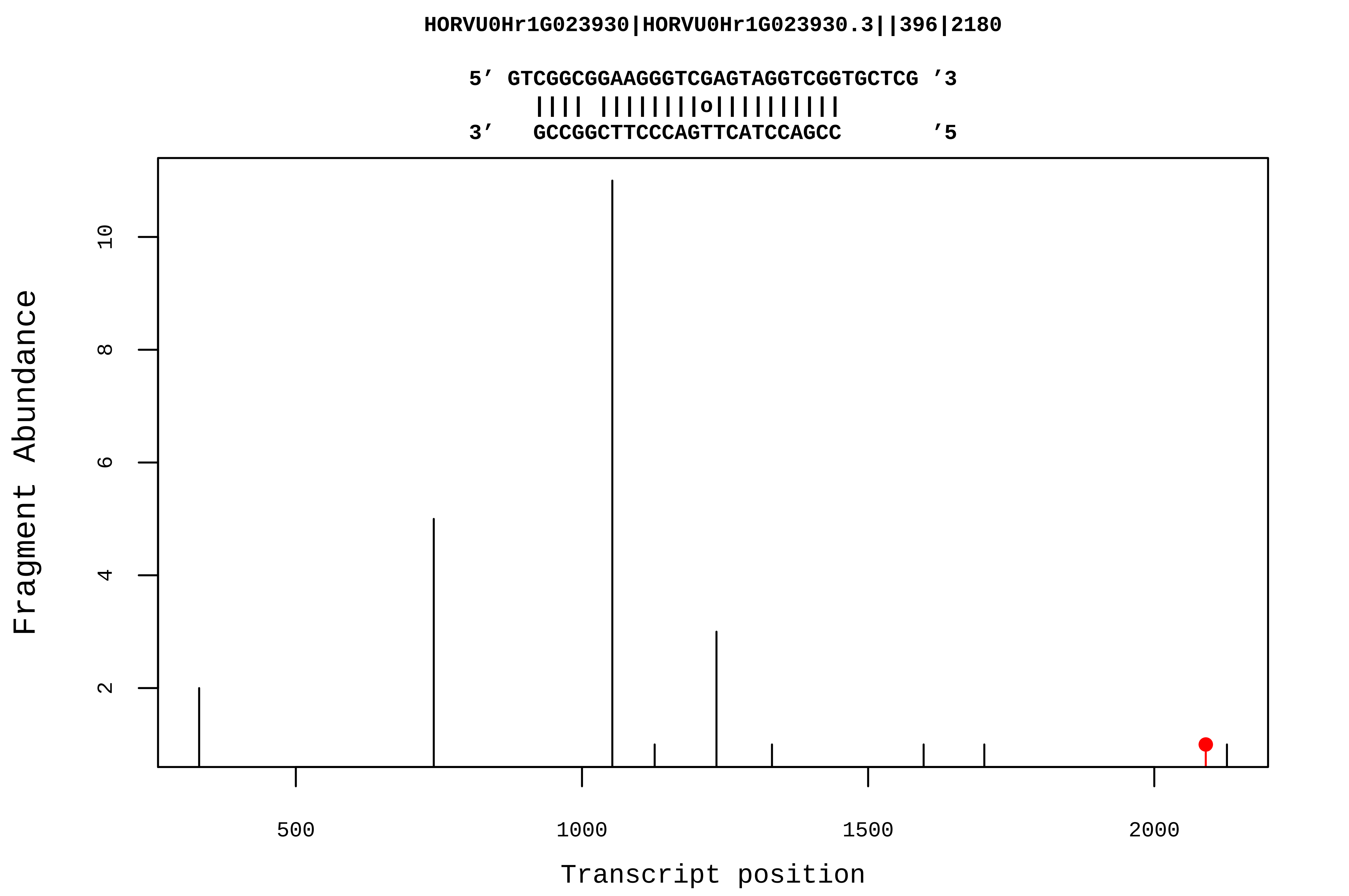

Cleavage site: 2090 Tag abundance: 1 Weighted abundance: 0.143 Category: 4
sRNA abundance: 1 Alignment score: 2 MFE ratio: 0.911 p-value: 0.019

HORVU2Hr1G072960|HORVU2Hr1G072960.1||600|3353

```
5' GCCGGCCGAAGGGTCGAGTAGGTCGGTGCTCG '3
      |||||||||||||o||||||||||
3'    GCCGGCTTCCCAGTTCATCCAGCC       '5
```

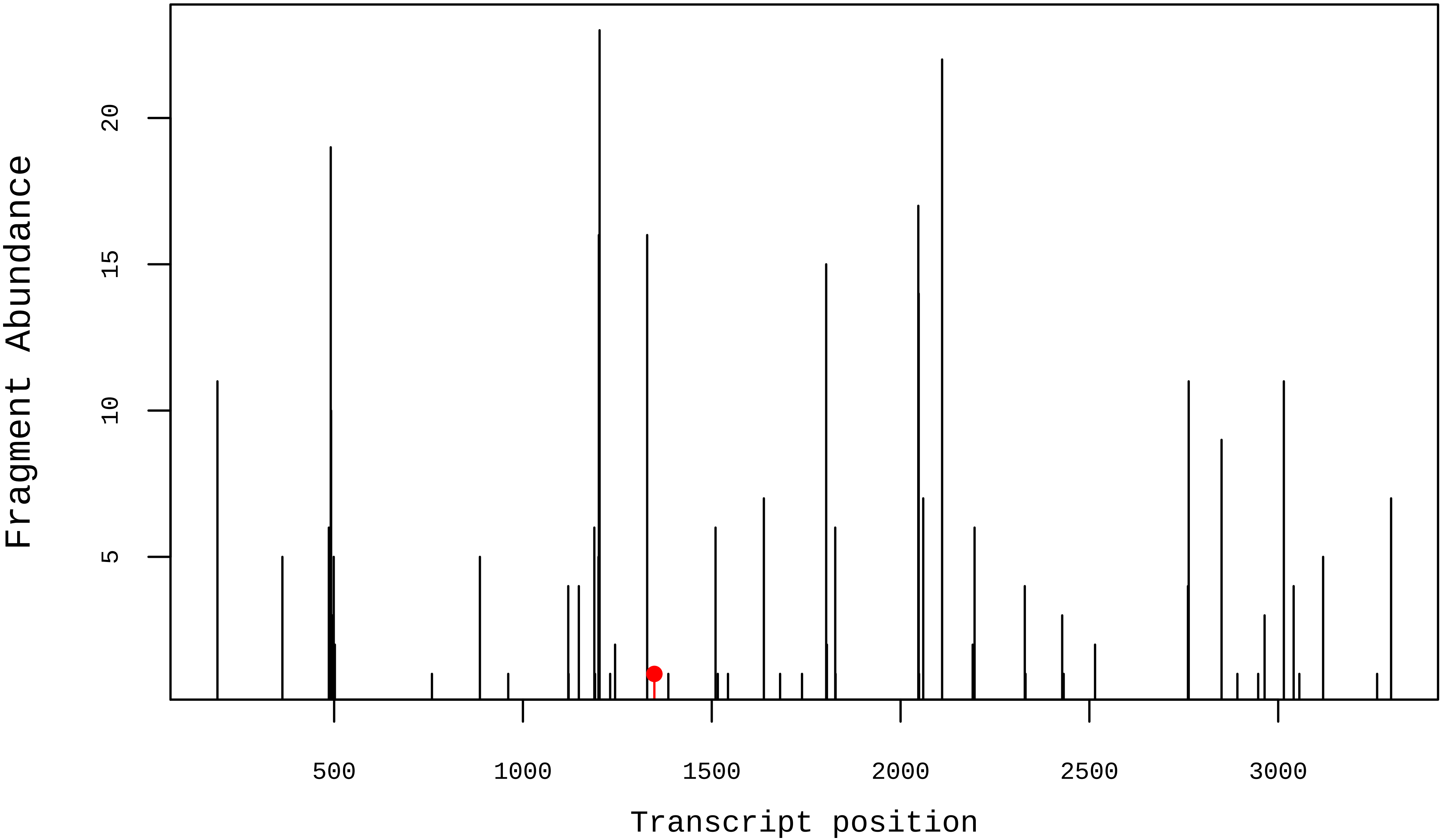

Cleavage site: 1348   Tag abundance: 1   Weighted abundance: 0.143   Category: 4
sRNA abundance: 1   Alignment score: 1   MFE ratio: 0.994   p-value: 0.03

HORVU5Hr1G015600|HORVU5Hr1G015600.1||156|510

```
5' GCCGGCCGAAGGGTCGAGTAGGTCGGTGCTCG '3
      |||||||||||||o||||||||||
3'    GCCGGCTTCCCAGTTCATCCAGCC       '5
```

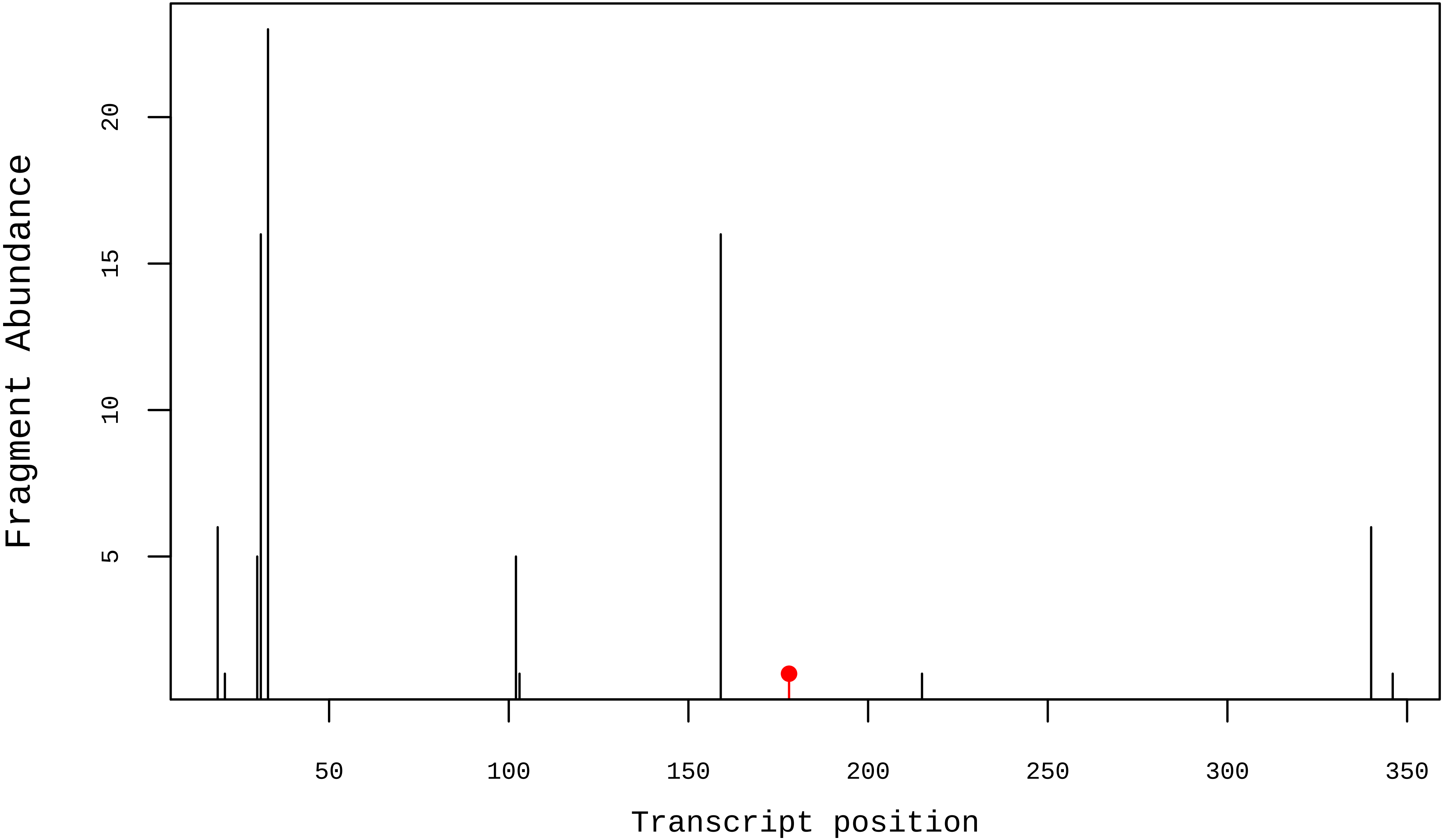

Cleavage site: 178   Tag abundance: 1   Weighted abundance: 0.143   Category: 4
sRNA abundance: 1   Alignment score: 1   MFE ratio: 0.994   p-value: 0.05

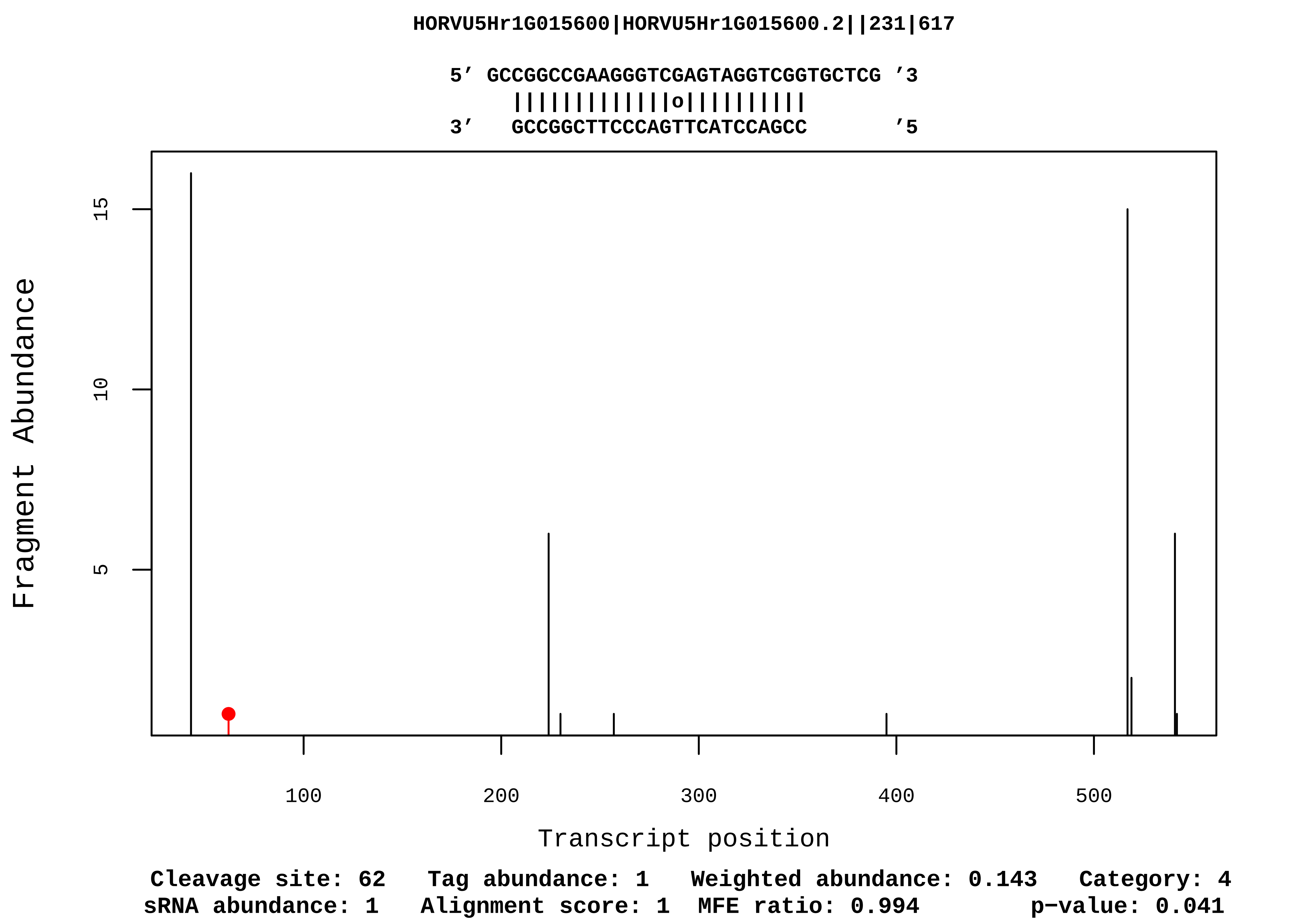
HORVU5Hr1G015600|HORVU5Hr1G015600.2||231|617
5′ GCCGGCCGAAGGGTCGAGTAGGTCGGTGCTCG ′3
|||||||||||||o||||||||||
3′ GCCGGCTTCCCAGTTCATCCAGCC ′5
Fragment Abundance
5
10
15
100
200
300
400
500
Transcript position
Cleavage site: 62 Tag abundance: 1 Weighted abundance: 0.143 Category: 4
sRNA abundance: 1 Alignment score: 1 MFE ratio: 0.994 p-value: 0.041

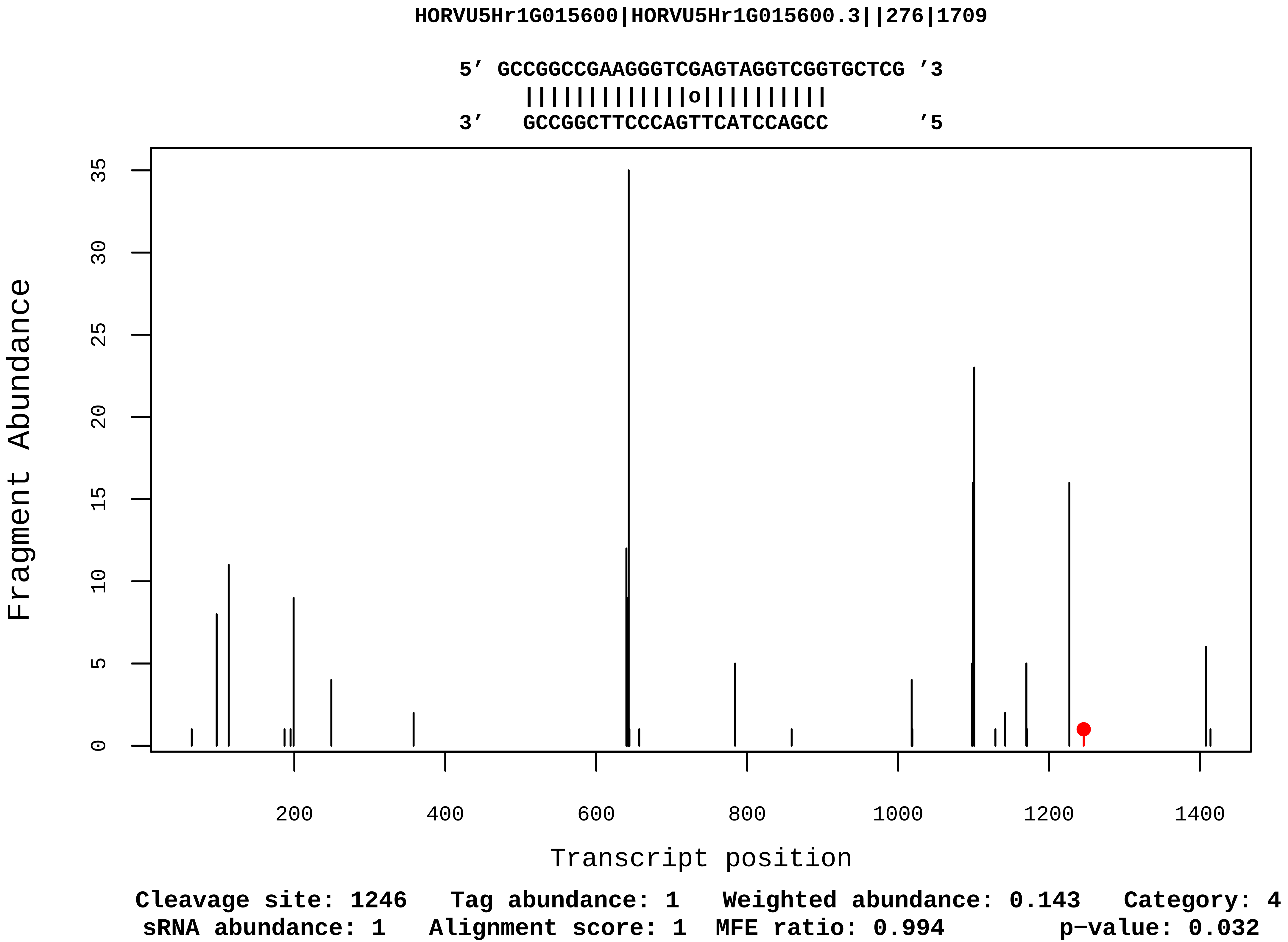
HORVU5Hr1G015600|HORVU5Hr1G015600.3||276|1709
5' GCCGGCCGAAGGGTCGAGTAGGTCGGTGCTCG '3
|||||||||||||o||||||||||
3' GCCGGCTTCCCAGTTCATCCAGCC '5
Fragment Abundance
0
5
10
15
20
25
30
35
200
400
600
800
1000
1200
1400
Transcript position
Cleavage site: 1246 Tag abundance: 1 Weighted abundance: 0.143 Category: 4
sRNA abundance: 1 Alignment score: 1 MFE ratio: 0.994 p-value: 0.032

HORVU5Hr1G015600|HORVU5Hr1G015600.4||207|1852

```
5’ GCCGGCCGCAGGGTCGAGTAGGTCGGTGCTCG ’3
      |||||| ||||||o||||||||||
3’    GCCGGCTTCCCAGTTCATCCAGCC        ’5
```

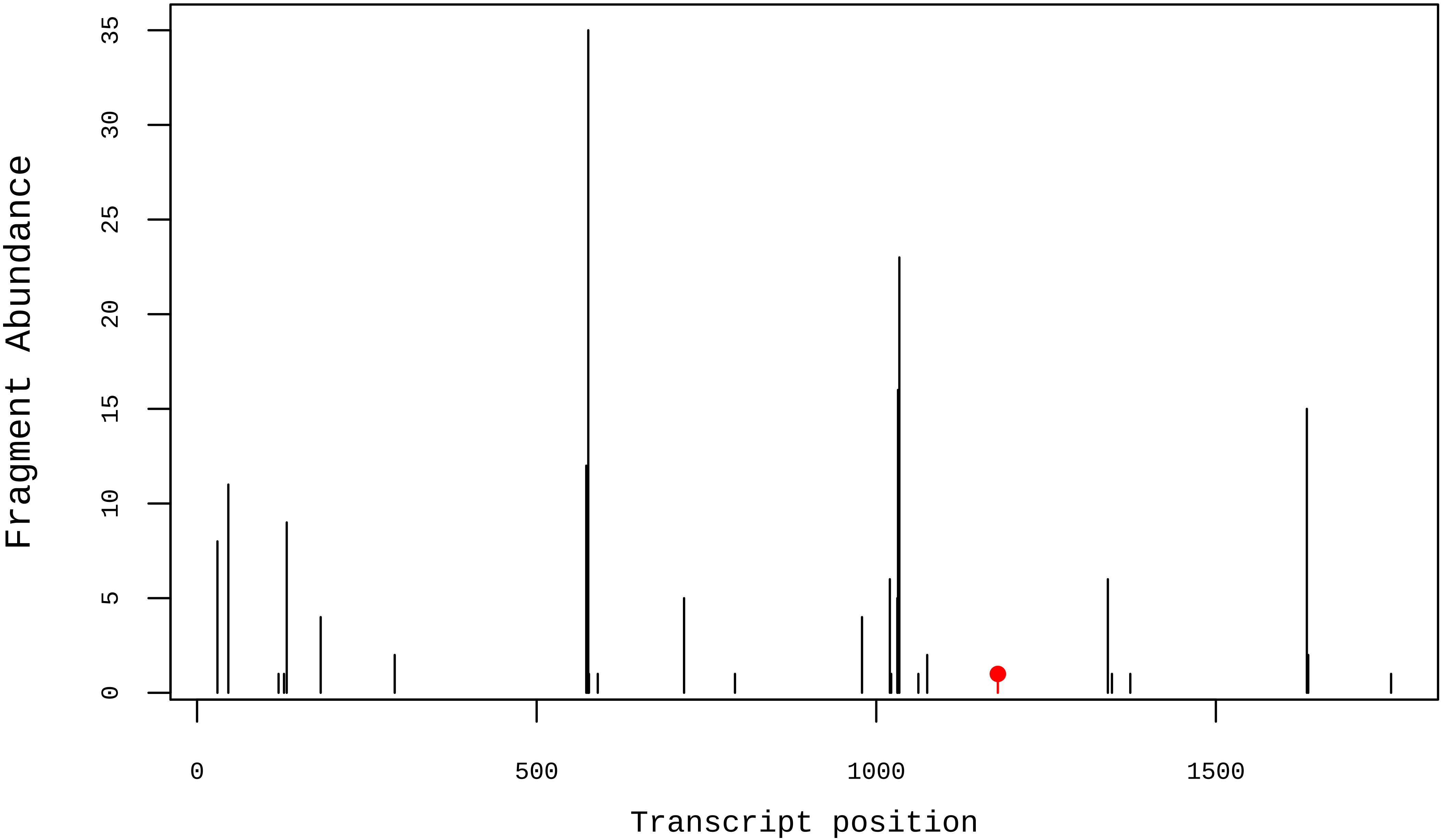

Cleavage site: 1179   Tag abundance: 1   Weighted abundance: 0.143   Category: 4
sRNA abundance: 1   Alignment score: 2   MFE ratio: 0.907   p-value: 0.041

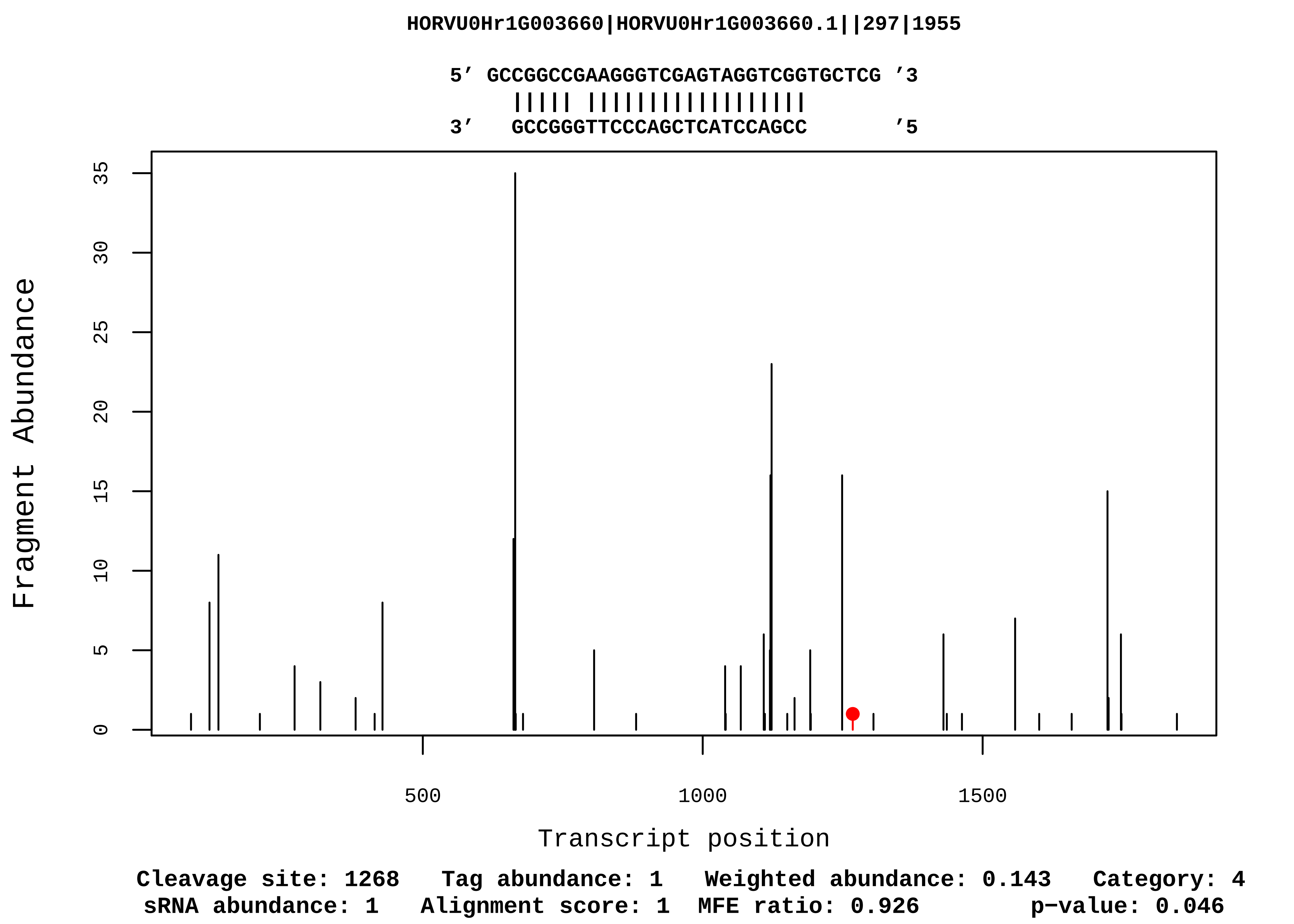
HORVU0Hr1G003660|HORVU0Hr1G003660.1||297|1955
5' GCCGGCCGAAGGGTCGAGTAGGTCGGTGCTCG '3
||||| ||||||||||||||||||
3' GCCGGGTTCCCAGCTCATCCAGCC '5
Fragment Abundance
0
5
10
15
20
25
30
35
500
1000
1500
Transcript position
Cleavage site: 1268 Tag abundance: 1 Weighted abundance: 0.143 Category: 4
sRNA abundance: 1 Alignment score: 1 MFE ratio: 0.926 p-value: 0.046

HORVU0Hr1G023930|HORVU0Hr1G023930.3||396|2180

5' GTCGGCGGAAGGGTCGAGTAGGTCGGTGCTCG '3
|||| ||||||||||||||||||
3' GCCGGGTTCCCAGCTCATCCAGCC '5

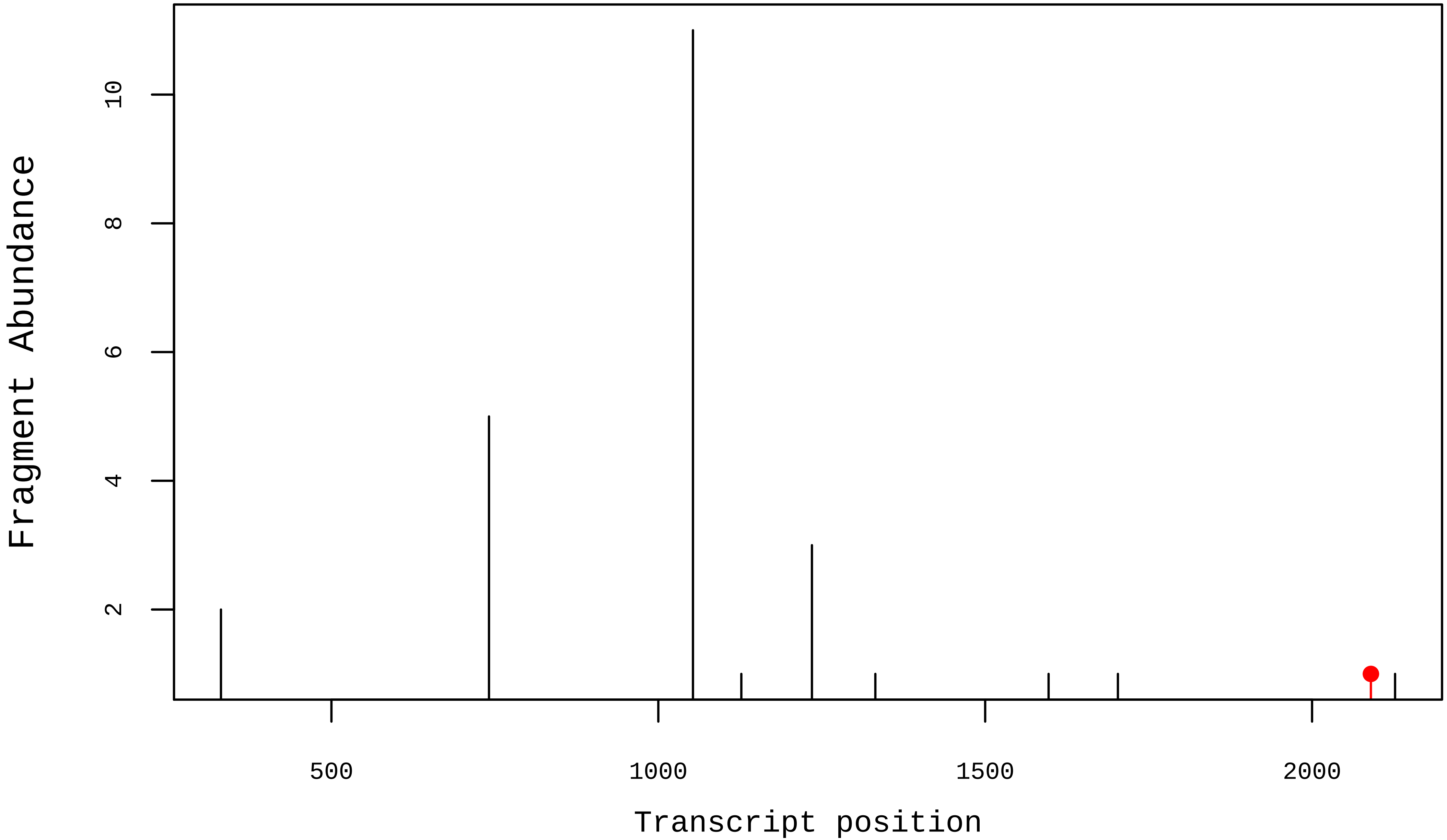

Cleavage site: 2090 Tag abundance: 1 Weighted abundance: 0.143 Category: 4
sRNA abundance: 1 Alignment score: 2 MFE ratio: 0.811 p-value: 0.019

HORVU2Hr1G072960|HORVU2Hr1G072960.1||600|3353

```
5' GCCGGCCGAAGGGTCGAGTAGGTCGGTGCTCG '3
      ||||| ||||||||||||||||||
3'    GCCGGGTTCCCAGCTCATCCAGCC        '5
```

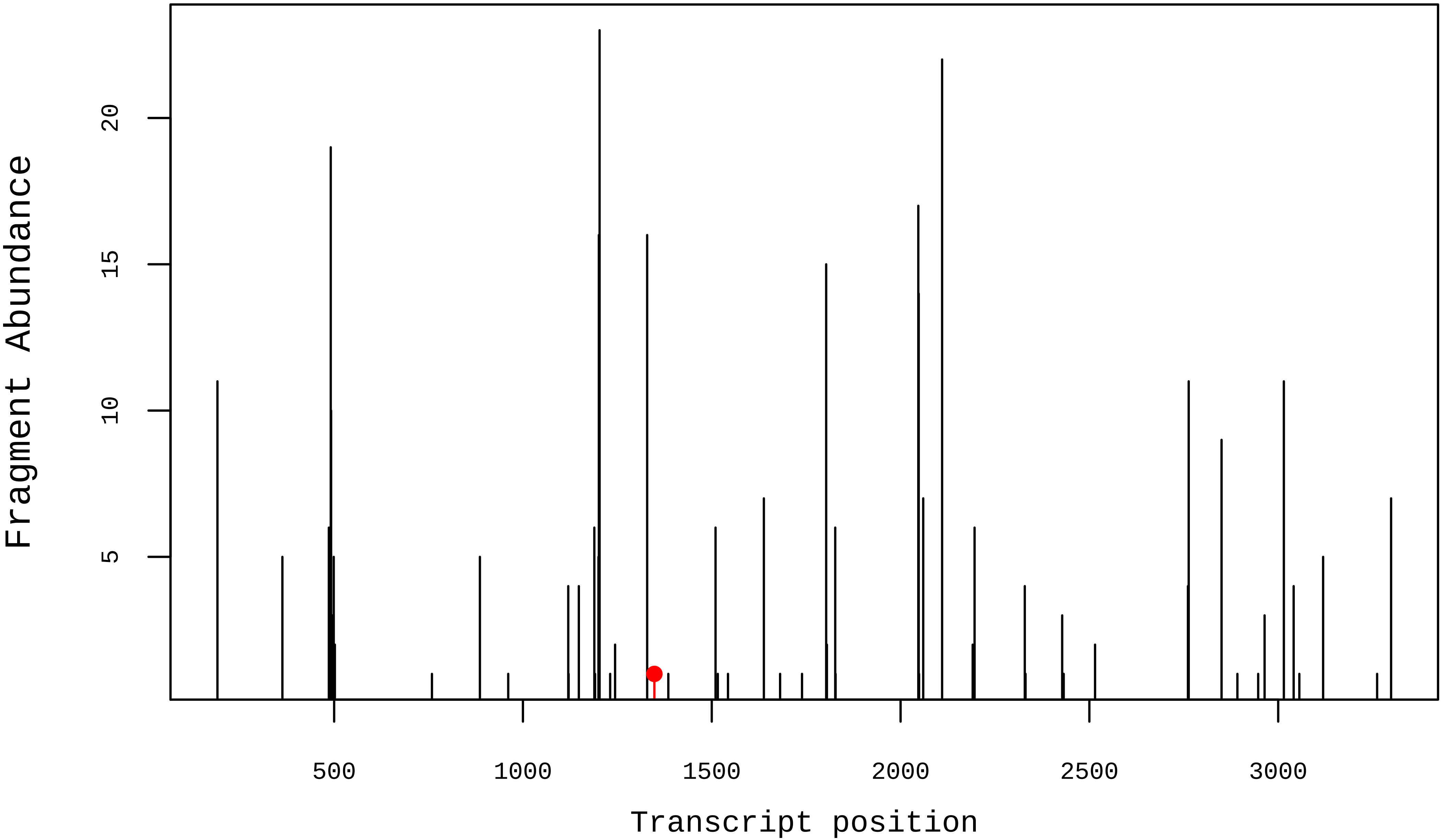

Cleavage site: 1348   Tag abundance: 1   Weighted abundance: 0.143   Category: 4
sRNA abundance: 1   Alignment score: 1   MFE ratio: 0.926   p-value: 0.03

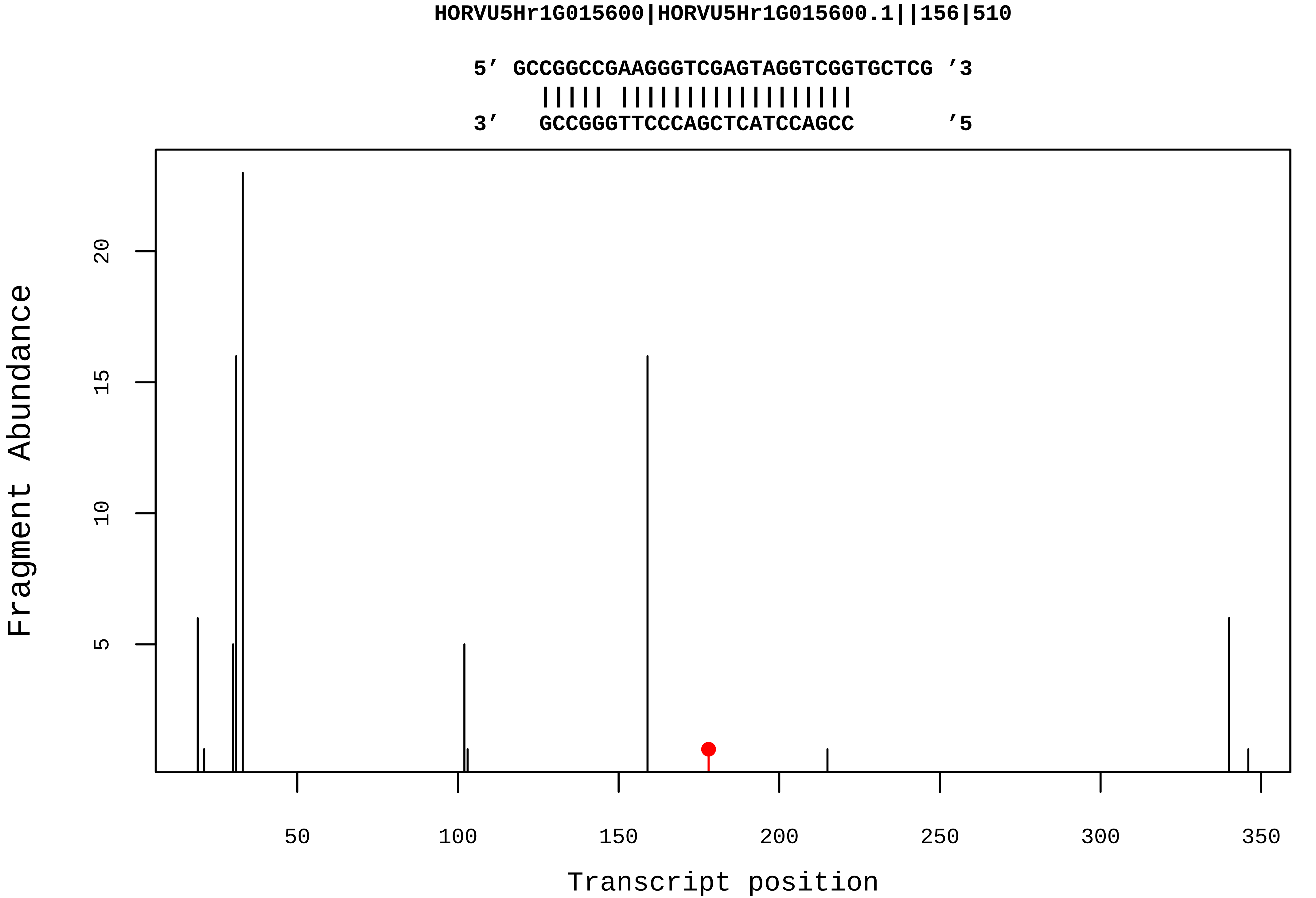

Cleavage site: 178 Tag abundance: 1 Weighted abundance: 0.143 Category: 4
sRNA abundance: 1 Alignment score: 1 MFE ratio: 0.926 p-value: 0.05

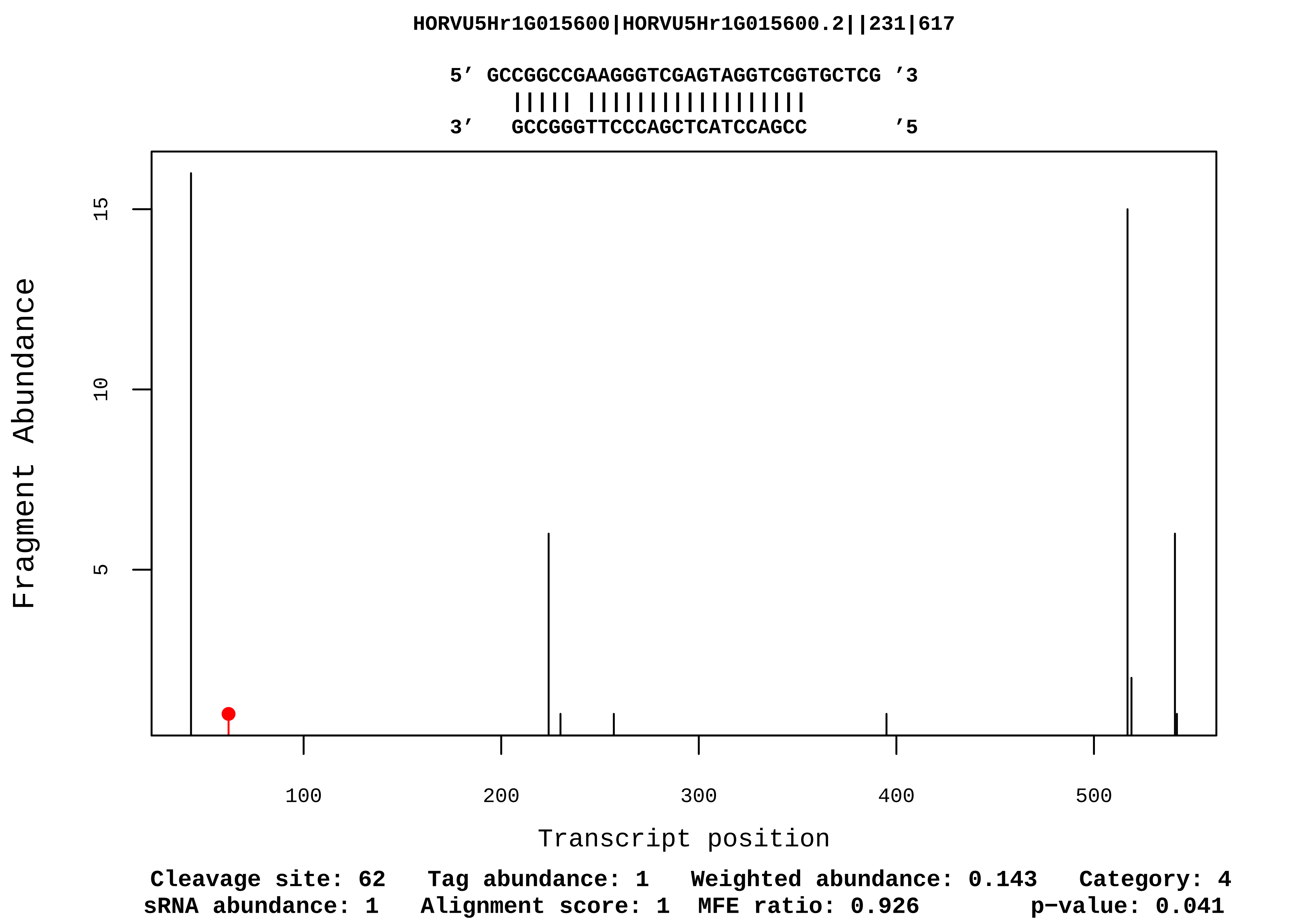
HORVU5Hr1G015600|HORVU5Hr1G015600.2||231|617
5' GCCGGCCGAAGGGTCGAGTAGGTCGGTGCTCG '3
3' GCCGGGTTCCCAGCTCATCCAGCC '5
Fragment Abundance
5
10
15
100
200
300
400
500
Transcript position
Cleavage site: 62 Tag abundance: 1 Weighted abundance: 0.143 Category: 4
sRNA abundance: 1 Alignment score: 1 MFE ratio: 0.926 p-value: 0.041

HORVU5Hr1G015600|HORVU5Hr1G015600.3||276|1709

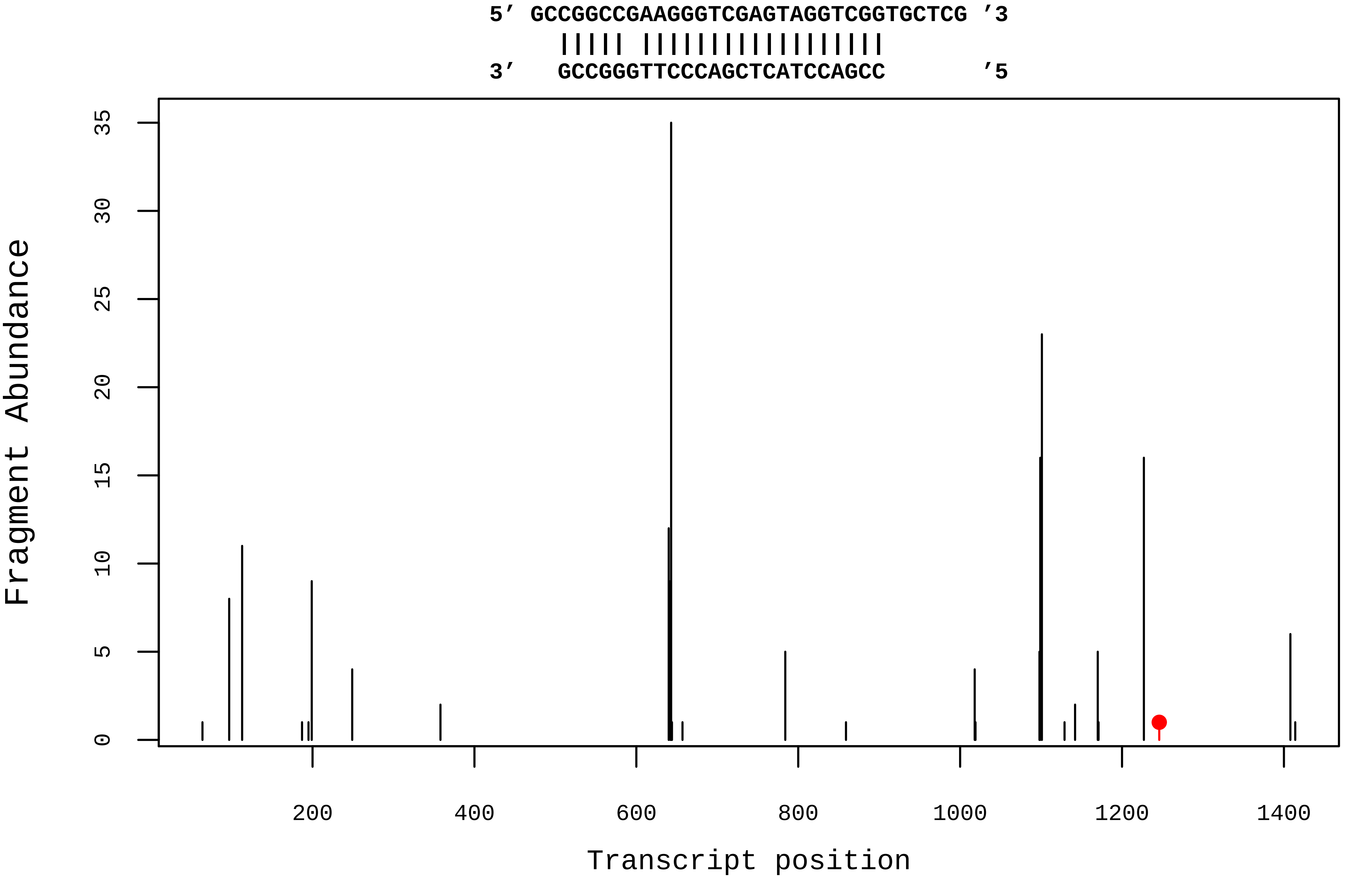

Cleavage site: 1246   Tag abundance: 1   Weighted abundance: 0.143   Category: 4
sRNA abundance: 1   Alignment score: 1   MFE ratio: 0.926   p-value: 0.032

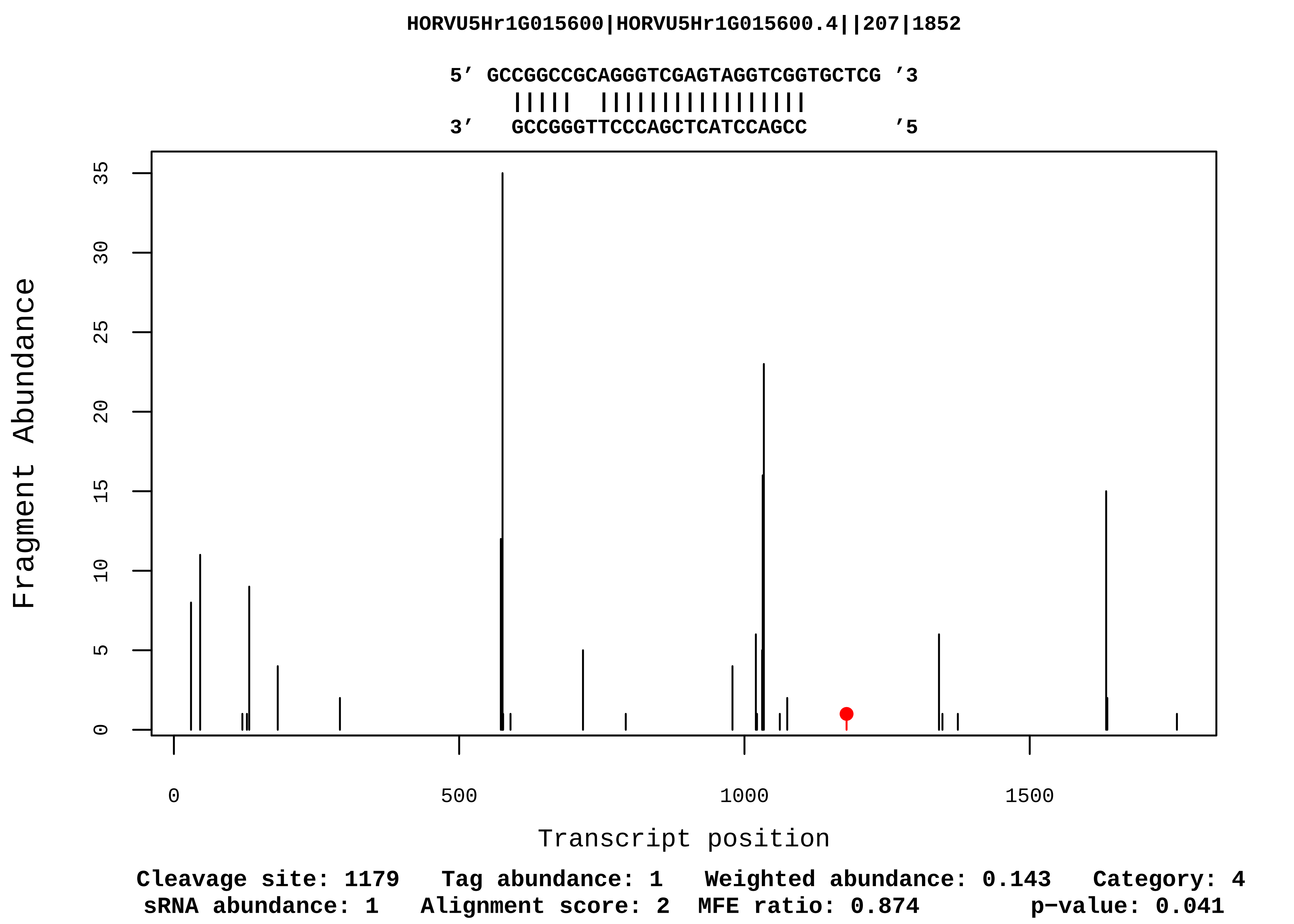

HORVU5Hr1G015600|HORVU5Hr1G015600.4||207|1852
5' GCCGGCCGCAGGGTCGAGTAGGTCGGTGCTCG '3
||||| |||||||||||||||||
3' GCCGGGTTCCCAGCTCATCCAGCC '5
Fragment Abundance
0
5
10
15
20
25
30
35
0
500
1000
1500
Transcript position
Cleavage site: 1179 Tag abundance: 1 Weighted abundance: 0.143 Category: 4
sRNA abundance: 1 Alignment score: 2 MFE ratio: 0.874 p-value: 0.041

HORVU7Hr1G096650|HORVU7Hr1G096650.4||960|1103

```
5' AGGTTCACGAAGATTTCTCAGGAGGCATCGTG '3
         ||||| ||o|||o|||||||
3'       GTGCTCCTGAAGGGTCCTCCA        '5
```

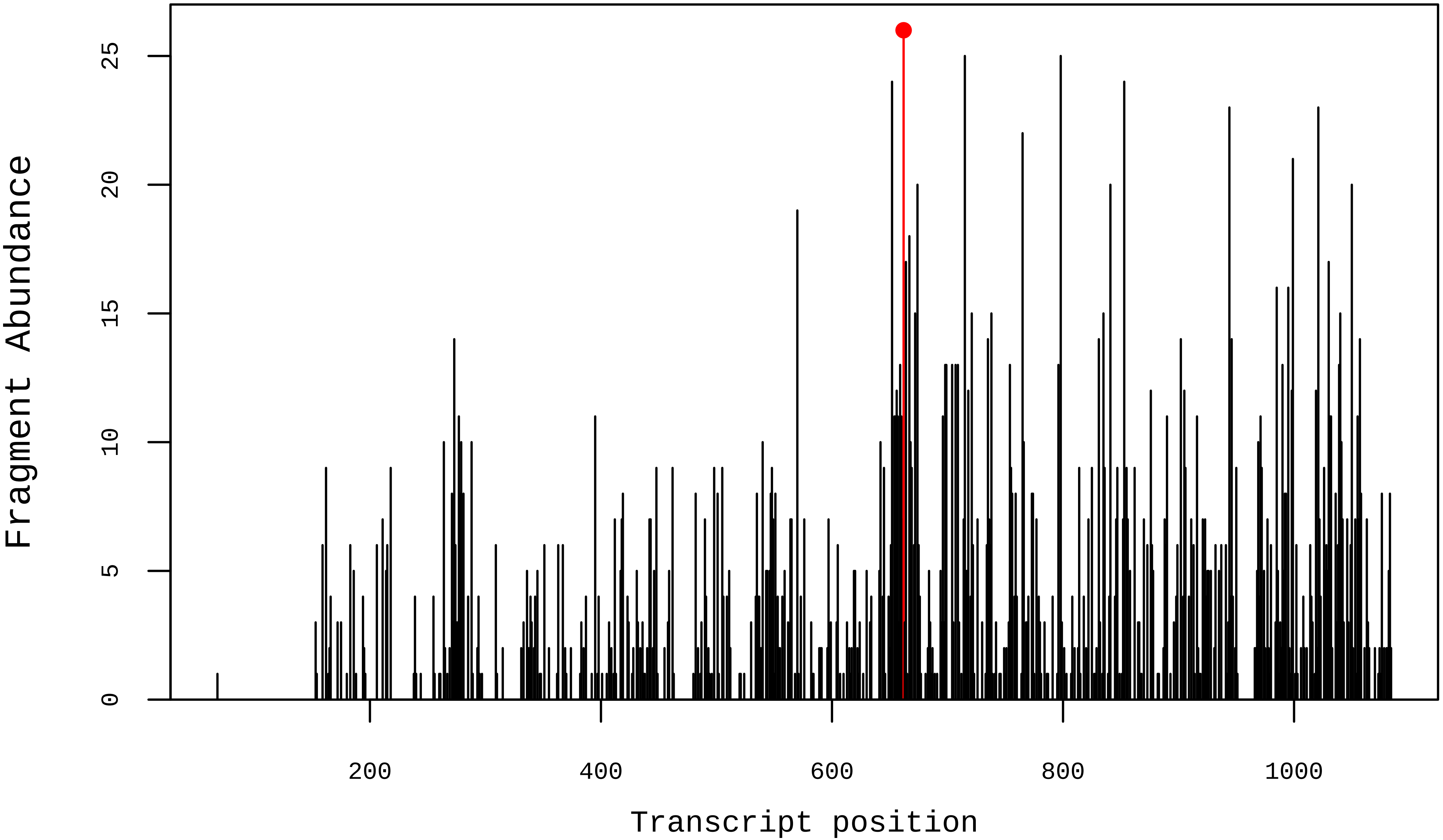

Cleavage site: 662   Tag abundance: 26   Weighted abundance: 2.364   Category: 0
sRNA abundance: 1   Alignment score: 4   MFE ratio: 0.716   p-value: 0.011

HORVU7Hr1G096650|HORVU7Hr1G096650.5||1107|1107

```
5' AGGTTCACGAAGATTTCTCAGGAGGCATCGTG '3
         ||||| ||o|||o|||||||
3'       GTGCTCCTGAAGGGTCCTCCA        '5
```

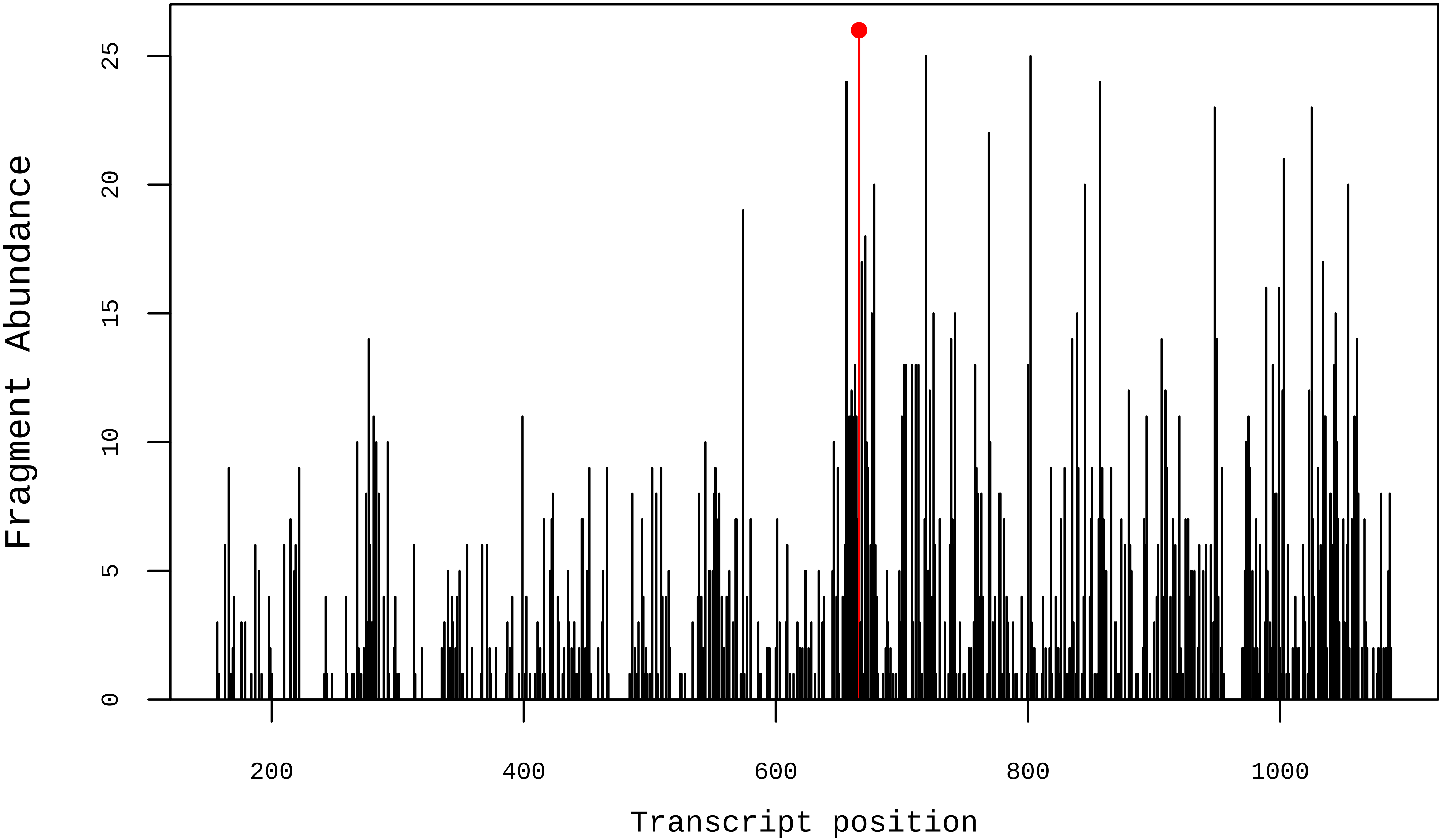

Cleavage site: 666   Tag abundance: 26   Weighted abundance: 2.364   Category: 0
sRNA abundance: 1   Alignment score: 4   MFE ratio: 0.716   p-value: 0.011

HORVU7Hr1G096650|HORVU7Hr1G096650.6||942|1092

```
5' AGGTTCACGAAGATTTCTCAGGAGGCATCGTG '3
        ||||| ||o|||o|||||||
3'      GTGCTCCTGAAGGGTCCTCCA        '5
```

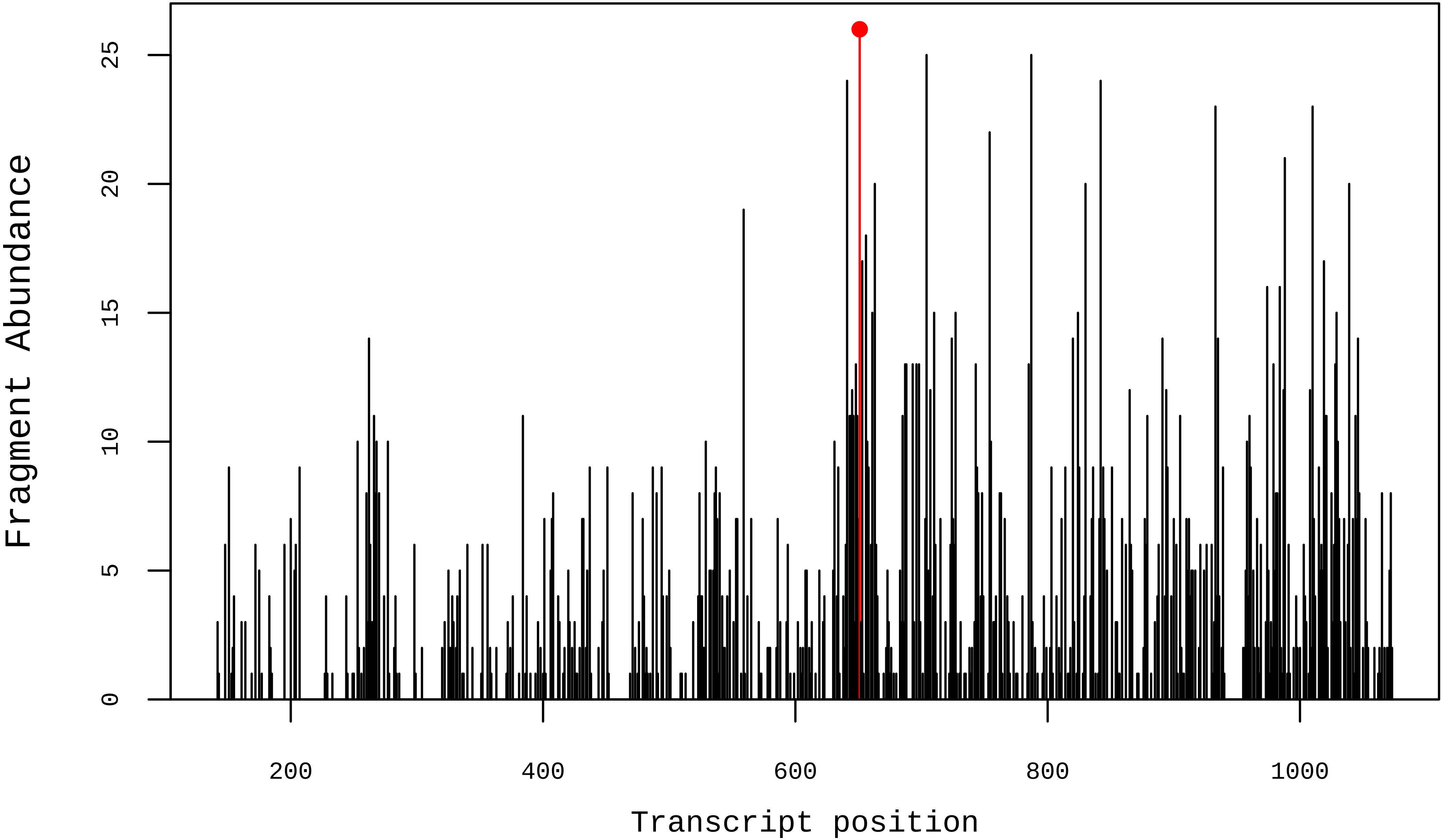

Cleavage site: 651   Tag abundance: 26   Weighted abundance: 2.364   Category: 0
sRNA abundance: 1   Alignment score: 4   MFE ratio: 0.716   p-value: 0.011

HORVU7Hr1G096650|HORVU7Hr1G096650.7||942|1103

```
5' AGGTTCACGAAGATTTCTCAGGAGGCATCGTG '3
          ||||| ||o|||o|||||||
3'        GTGCTCCTGAAGGGTCCTCCA        '5
```

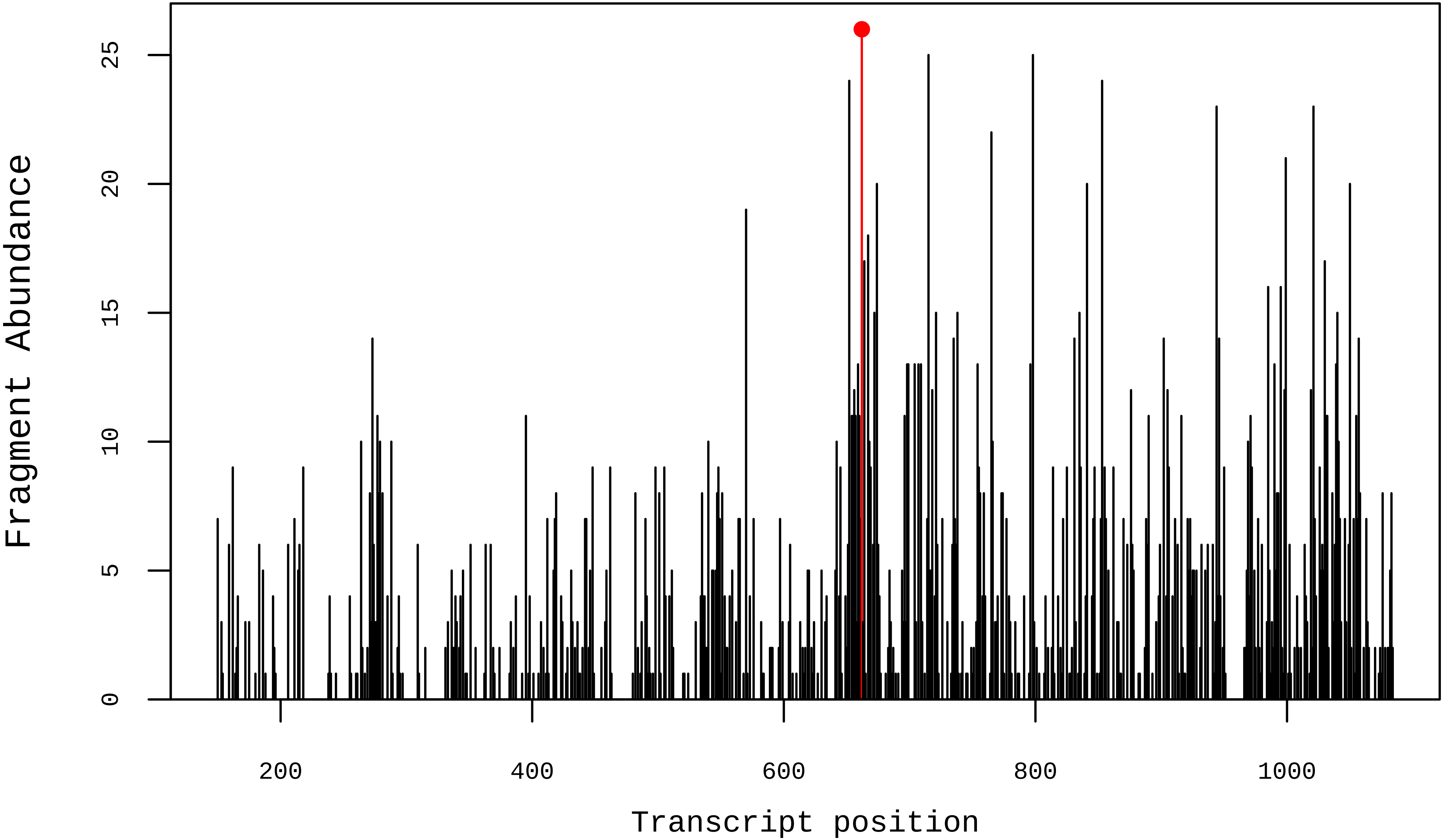

Cleavage site: 662   Tag abundance: 26   Weighted abundance: 2.364   Category: 0
sRNA abundance: 1   Alignment score: 4   MFE ratio: 0.716   p-value: 0.011

HORVU7Hr1G096670|HORVU7Hr1G096670.3||960|1101

```
5' AGGTTCACGAAGATTTCTCAGGAGGCATCGTG '3
          ||||| ||o|||o|||||||
3'        GTGCTCCTGAAGGGTCCTCCA       '5
```

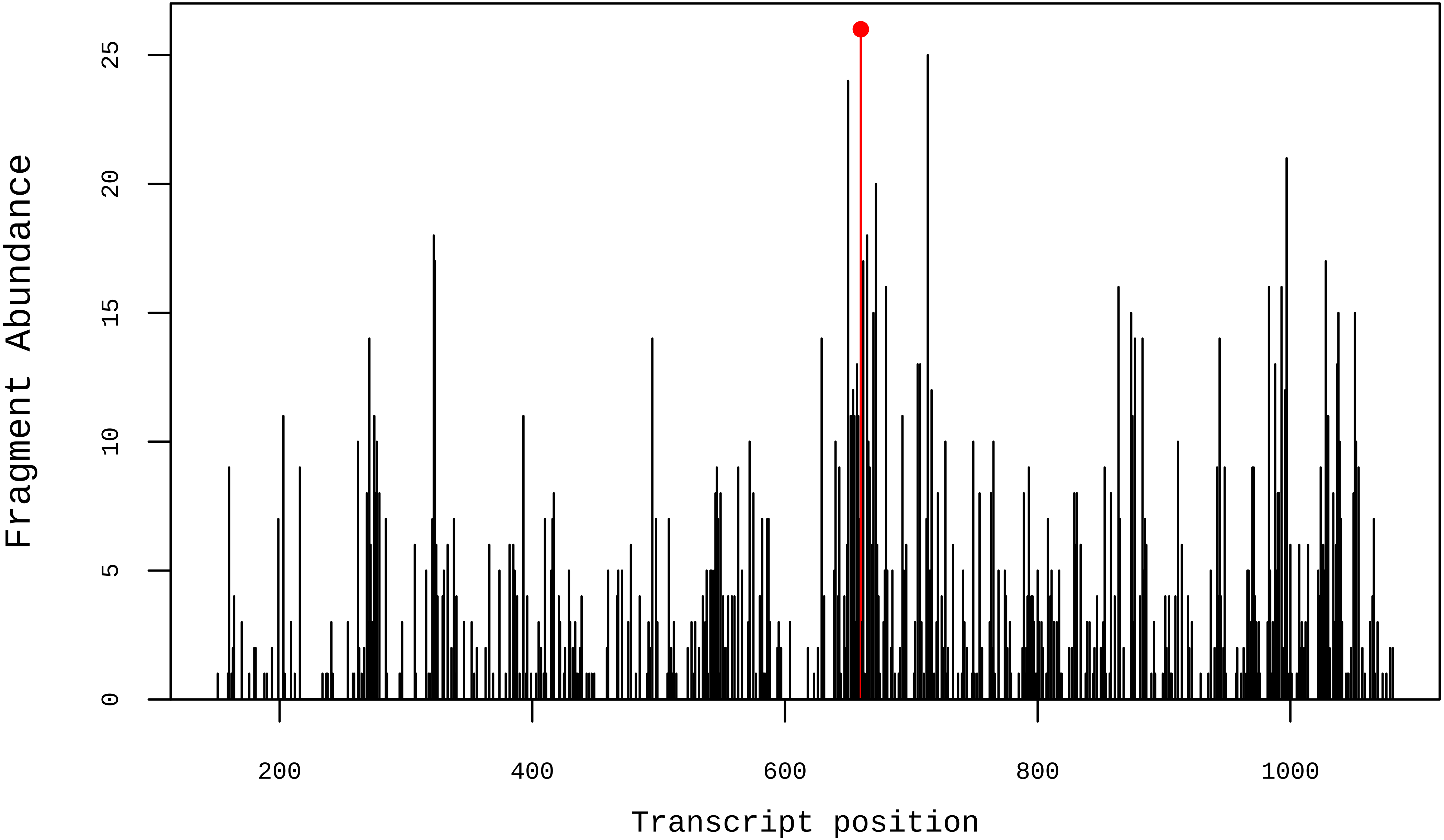

Cleavage site: 660   Tag abundance: 26   Weighted abundance: 2.364   Category: 0
sRNA abundance: 1   Alignment score: 4   MFE ratio: 0.716   p-value: 0.011

HORVU0Hr1G003660|HORVU0Hr1G003660.1||297|1955

```
5' GCCGGCCGAAGGGTCGAGTAGGTCGGTGCTCG '3
        ||||||||||||o||||||||||
3'      CCGGCTTCCCAGTTCATCCAGCC         '5
```

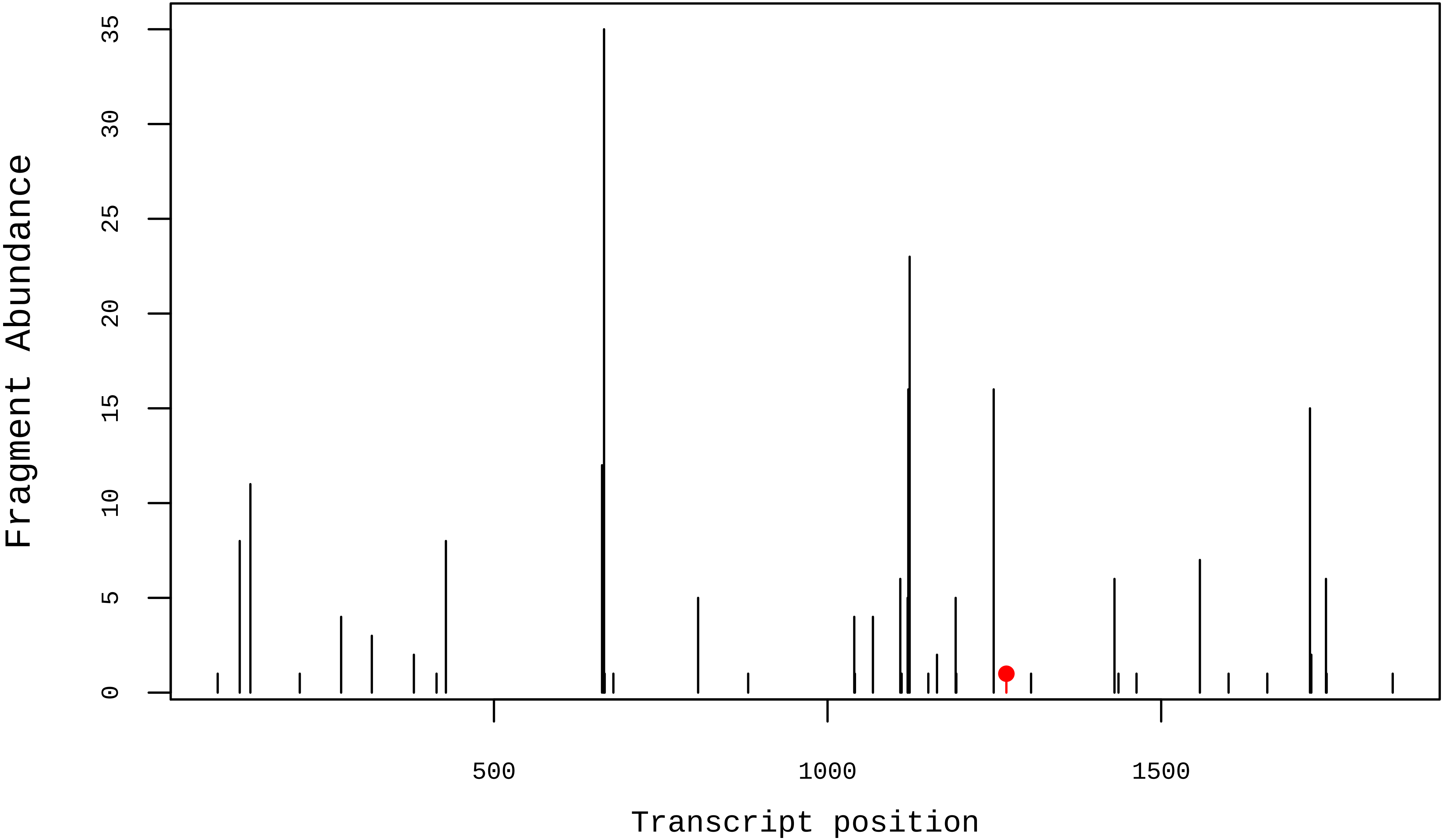

Cleavage site: 1268   Tag abundance: 1   Weighted abundance: 0.143   Category: 4
sRNA abundance: 1   Alignment score: 1   MFE ratio: 0.994   p-value: 0.046

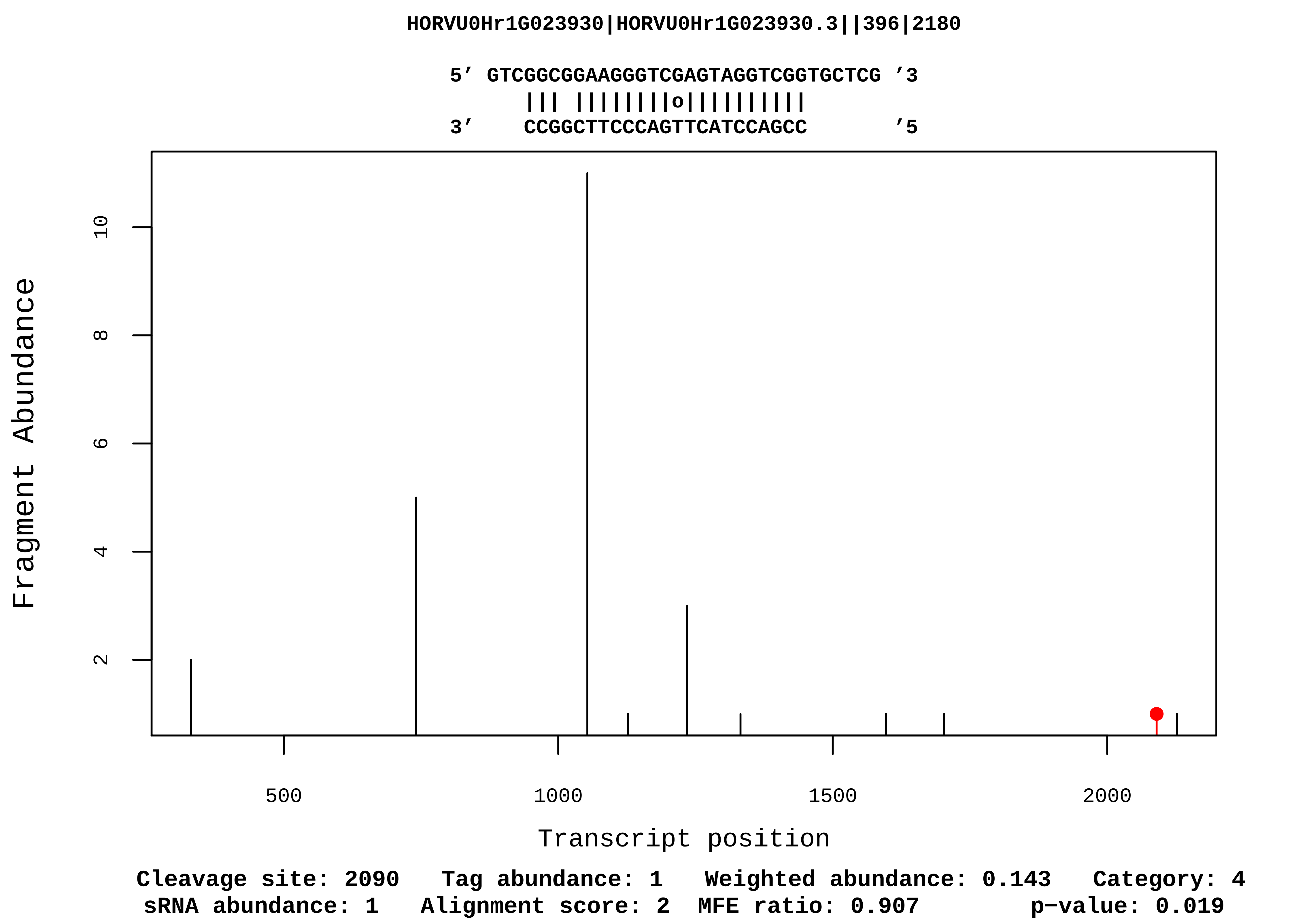
HORVU0Hr1G023930|HORVU0Hr1G023930.3||396|2180
5' GTCGGCGGAAGGGTCGAGTAGGTCGGTGCTCG '3
||| ||||||||o||||||||||
3' CCGGCTTCCCAGTTCATCCAGCC '5
Fragment Abundance
2
4
6
8
10
500
1000
1500
2000
Transcript position
Cleavage site: 2090 Tag abundance: 1 Weighted abundance: 0.143 Category: 4
sRNA abundance: 1 Alignment score: 2 MFE ratio: 0.907 p-value: 0.019

HORVU2Hr1G072960|HORVU2Hr1G072960.1||600|3353

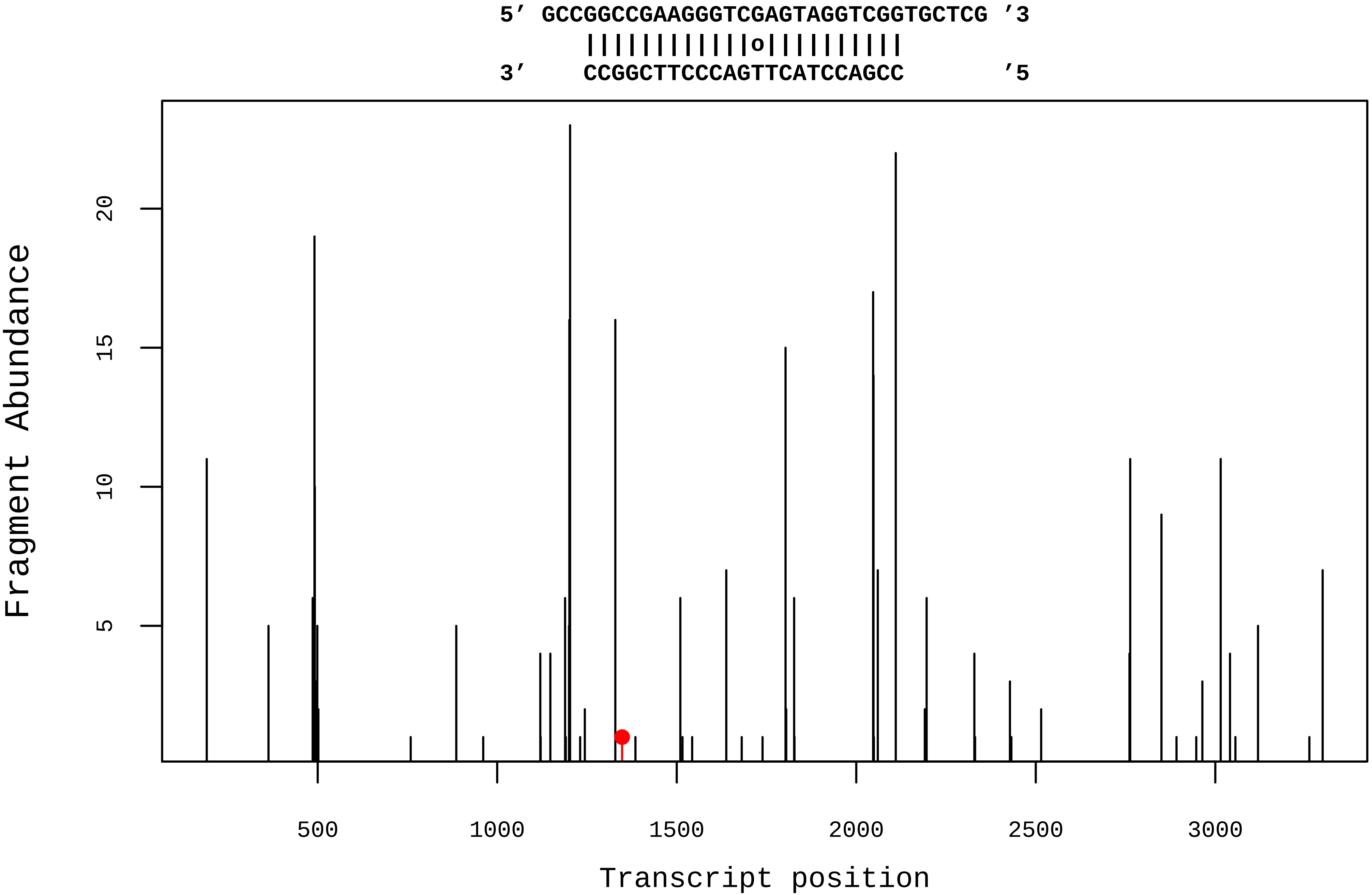

Cleavage site: 1348 Tag abundance: 1 Weighted abundance: 0.143 Category: 4
sRNA abundance: 1 Alignment score: 1 MFE ratio: 0.994 p-value: 0.03

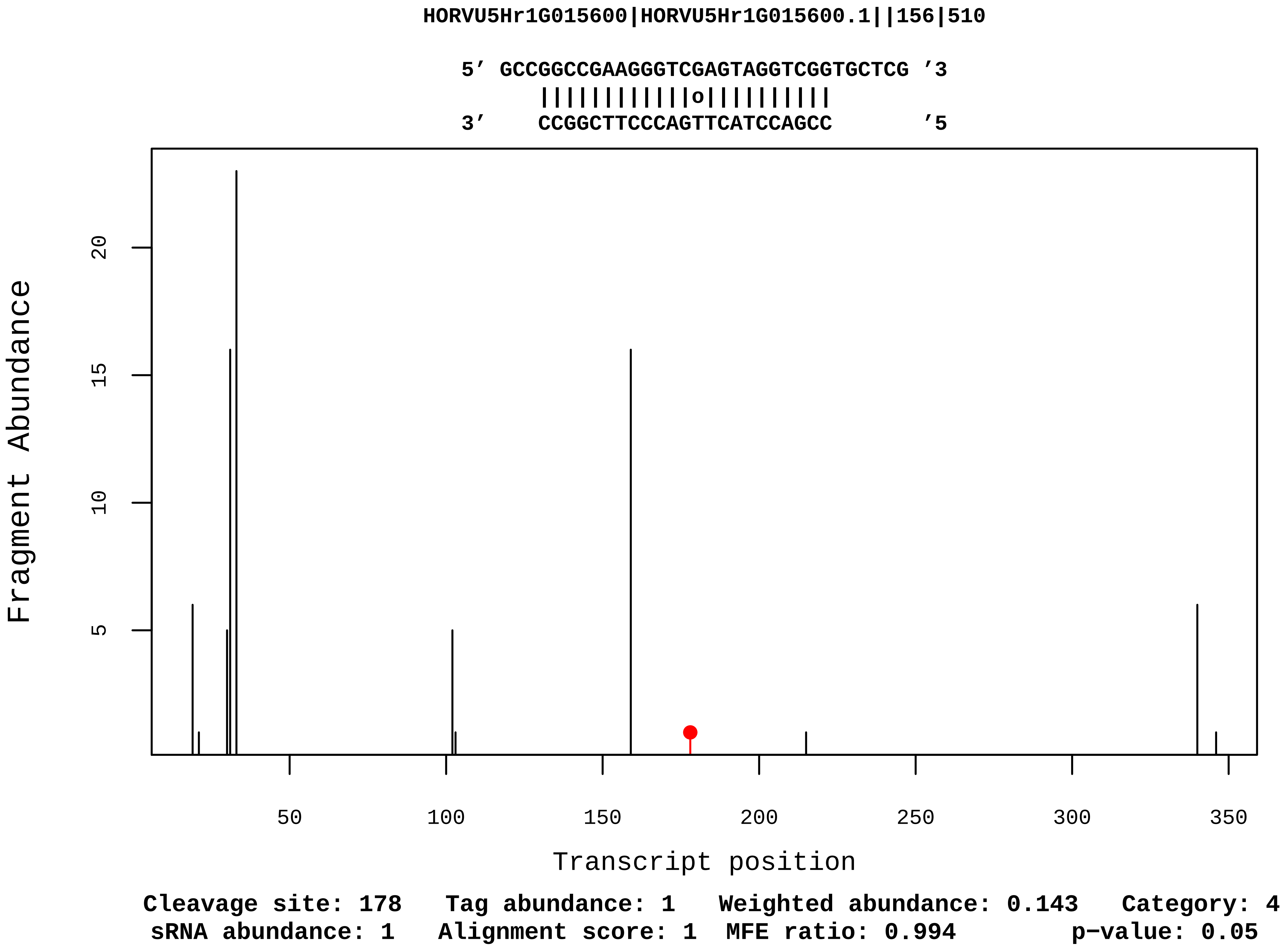

HORVU5Hr1G015600|HORVU5Hr1G015600.1||156|510
5' GCCGGCCGAAGGGTCGAGTAGGTCGGTGCTCG '3
||||||||||||o||||||||||
3' CCGGCTTCCCAGTTCATCCAGCC '5
Fragment Abundance
5
10
15
20
50
100
150
200
250
300
350
Transcript position
Cleavage site: 178 Tag abundance: 1 Weighted abundance: 0.143 Category: 4
sRNA abundance: 1 Alignment score: 1 MFE ratio: 0.994 p-value: 0.05

HORVU5Hr1G015600|HORVU5Hr1G015600.2||231|617

```
5' GCCGGCCGAAGGGTCGAGTAGGTCGGTGCTCG '3
      ||||||||||||o||||||||||
3'    CCGGCTTCCCAGTTCATCCAGCC       '5
```

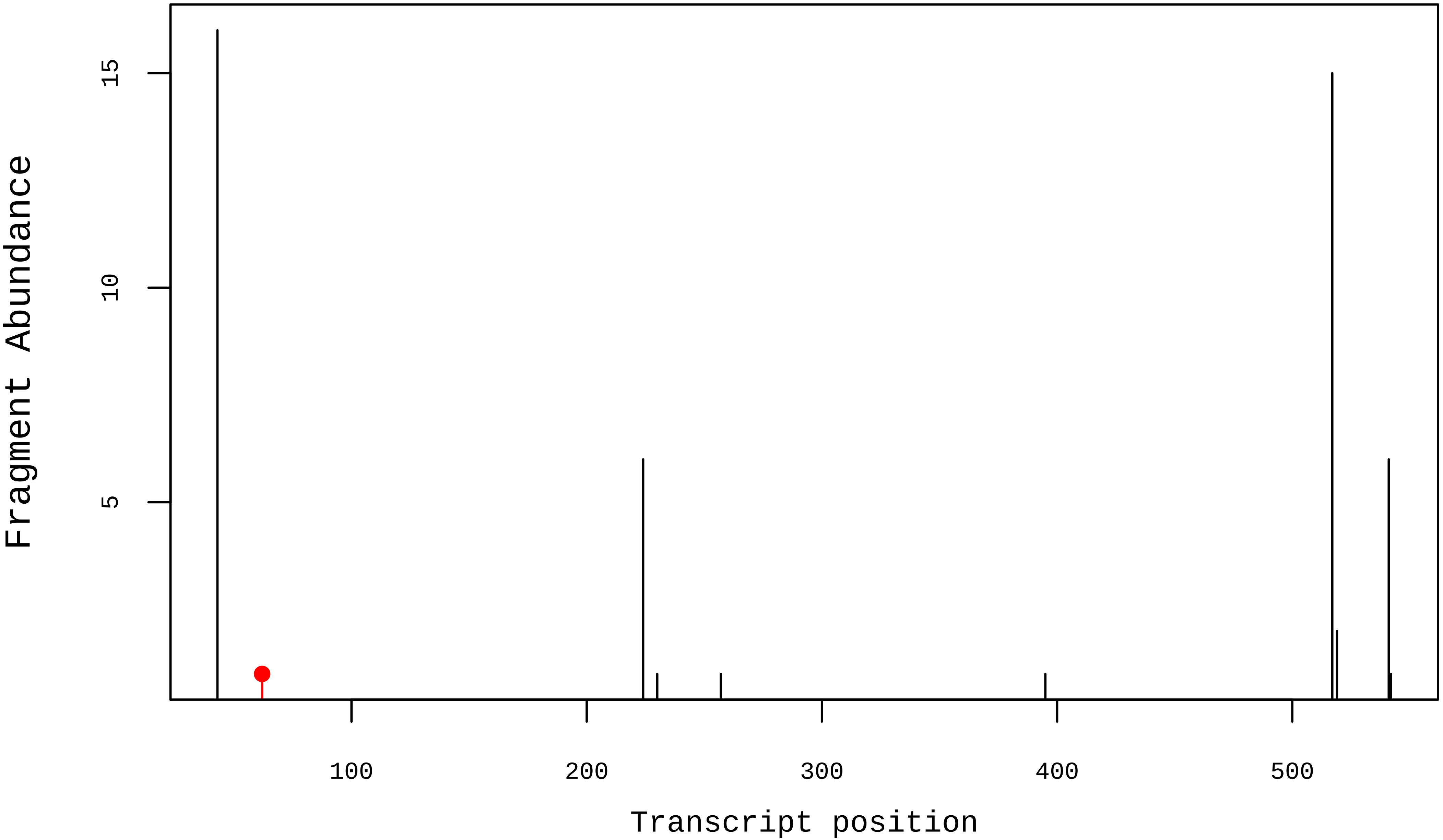

Cleavage site: 62   Tag abundance: 1   Weighted abundance: 0.143   Category: 4
sRNA abundance: 1   Alignment score: 1   MFE ratio: 0.994   p-value: 0.041

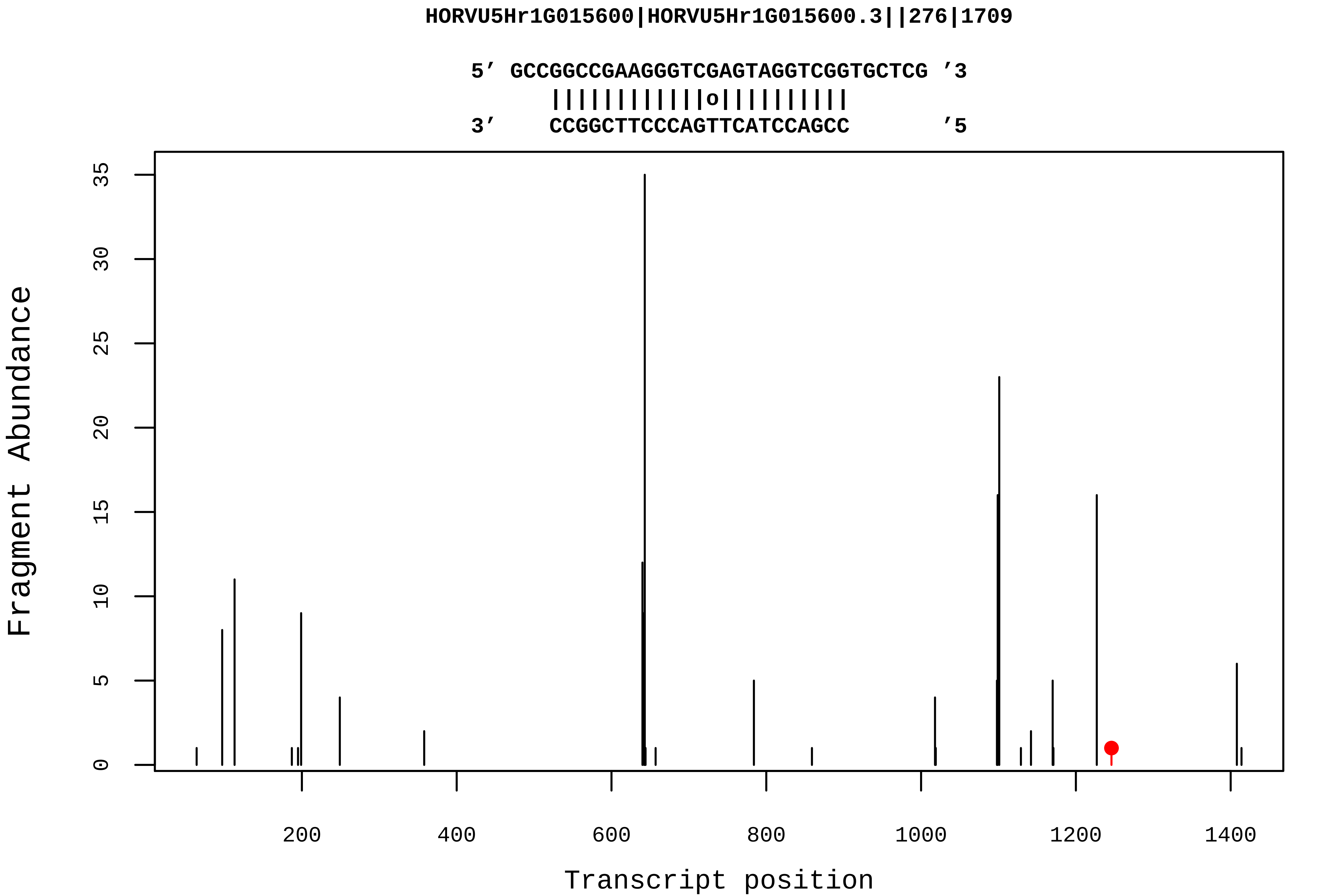

Cleavage site: 1246 Tag abundance: 1 Weighted abundance: 0.143 Category: 4
sRNA abundance: 1 Alignment score: 1 MFE ratio: 0.994 p-value: 0.032

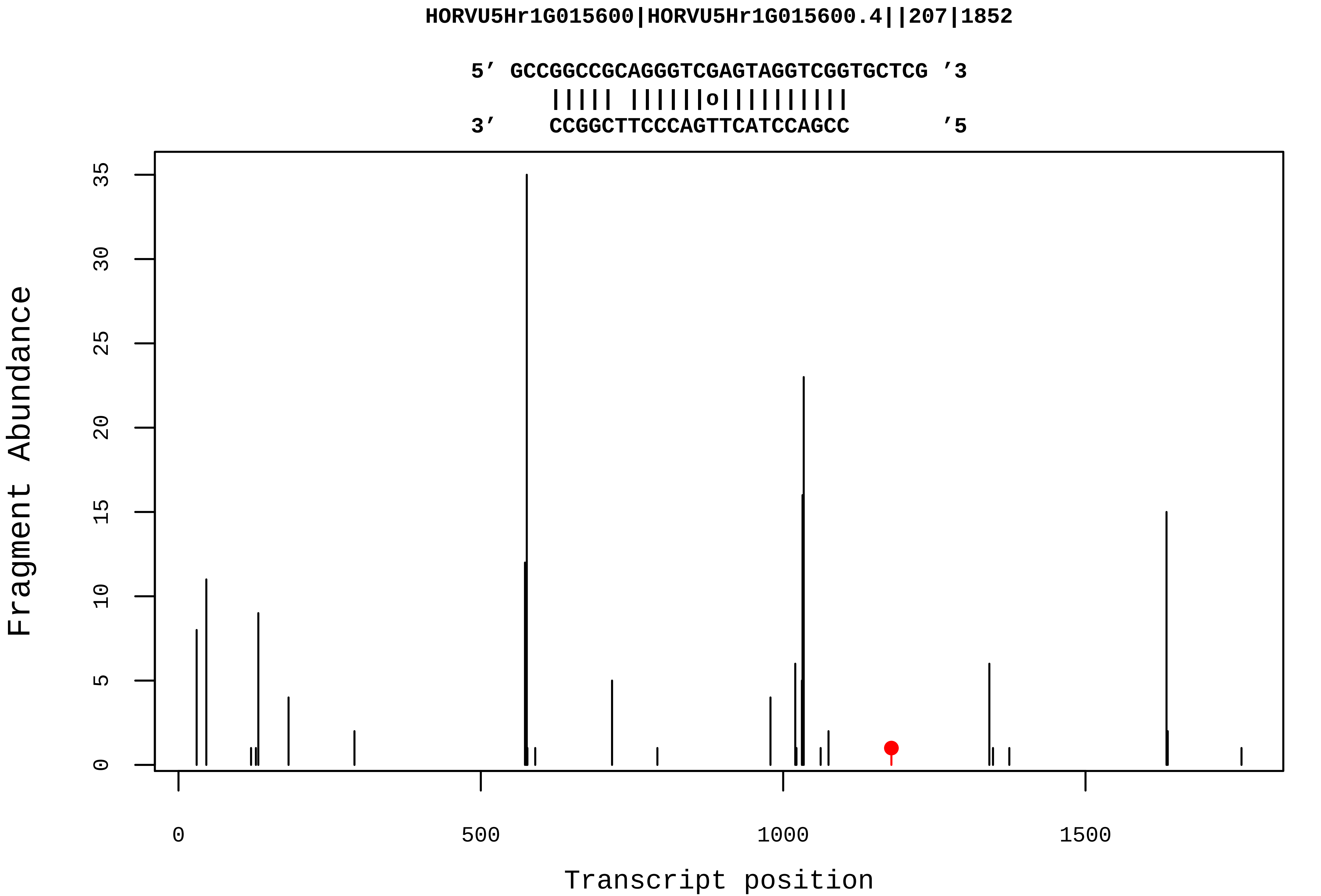

Cleavage site: 1179 Tag abundance: 1 Weighted abundance: 0.143 Category: 4
sRNA abundance: 1 Alignment score: 2 MFE ratio: 0.902 p-value: 0.041

HORVU0Hr1G003660|HORVU0Hr1G003660.1||297|1955

```
5' CATTACTCCGATCCCGAAGGCCAACACAATAG '3
         ||||||||||||||||||||
3'       GAGGCTAGGGCTTCCGGTTGT       '5
```

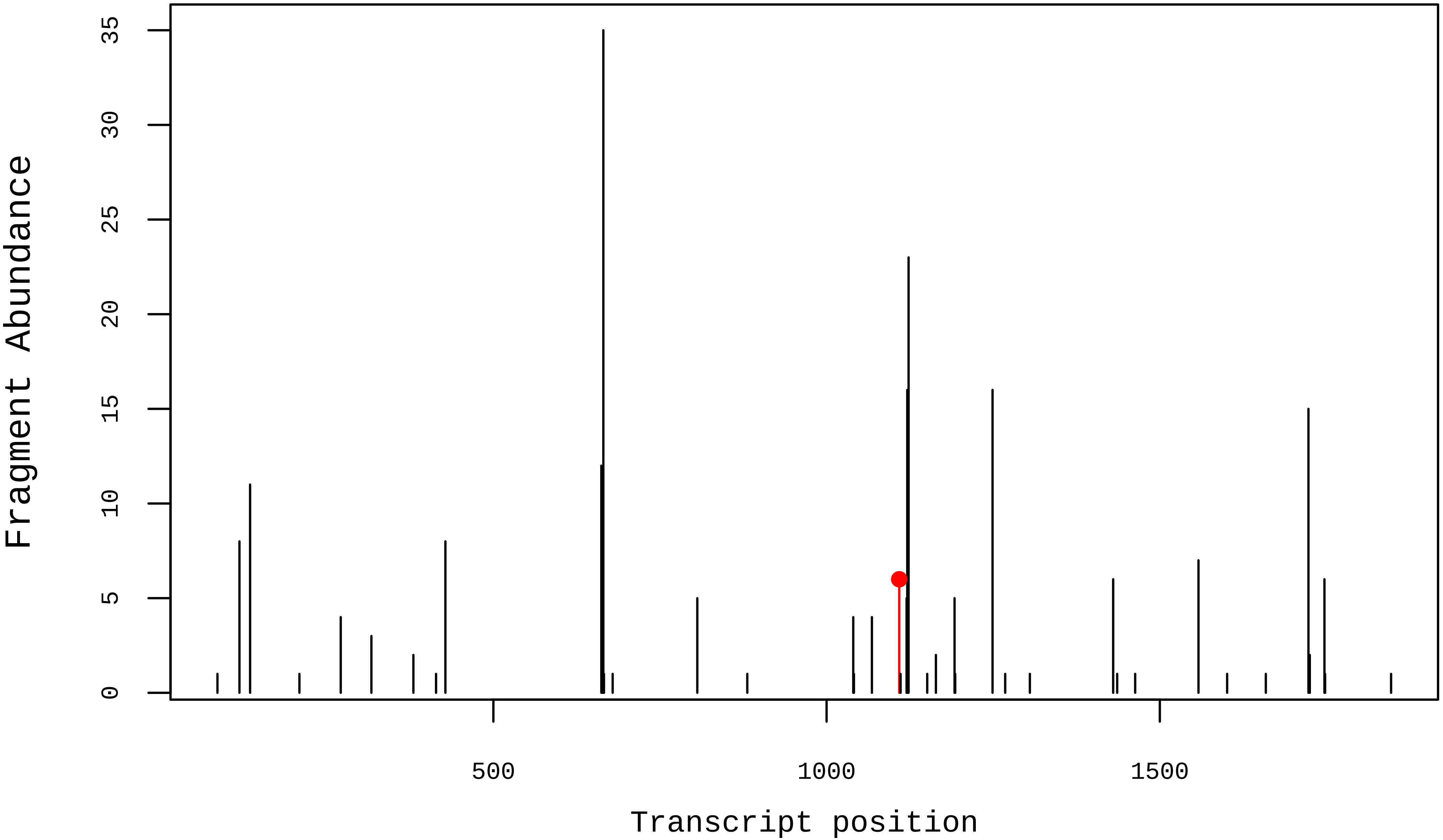

Cleavage site: 1109   Tag abundance: 6   Weighted abundance: 1.5   Category: 3

sRNA abundance: 1   Alignment score: 0   MFE ratio: 1   p−value: 0.033

HORVU2Hr1G072960|HORVU2Hr1G072960.1||600|3353

```
5’ CATTACTCCGATCCCGAAGGCCAACACAATAG ’3
           ||||||||||||||||||||
3’         GAGGCTAGGGCTTCCGGTTGT       ’5
```

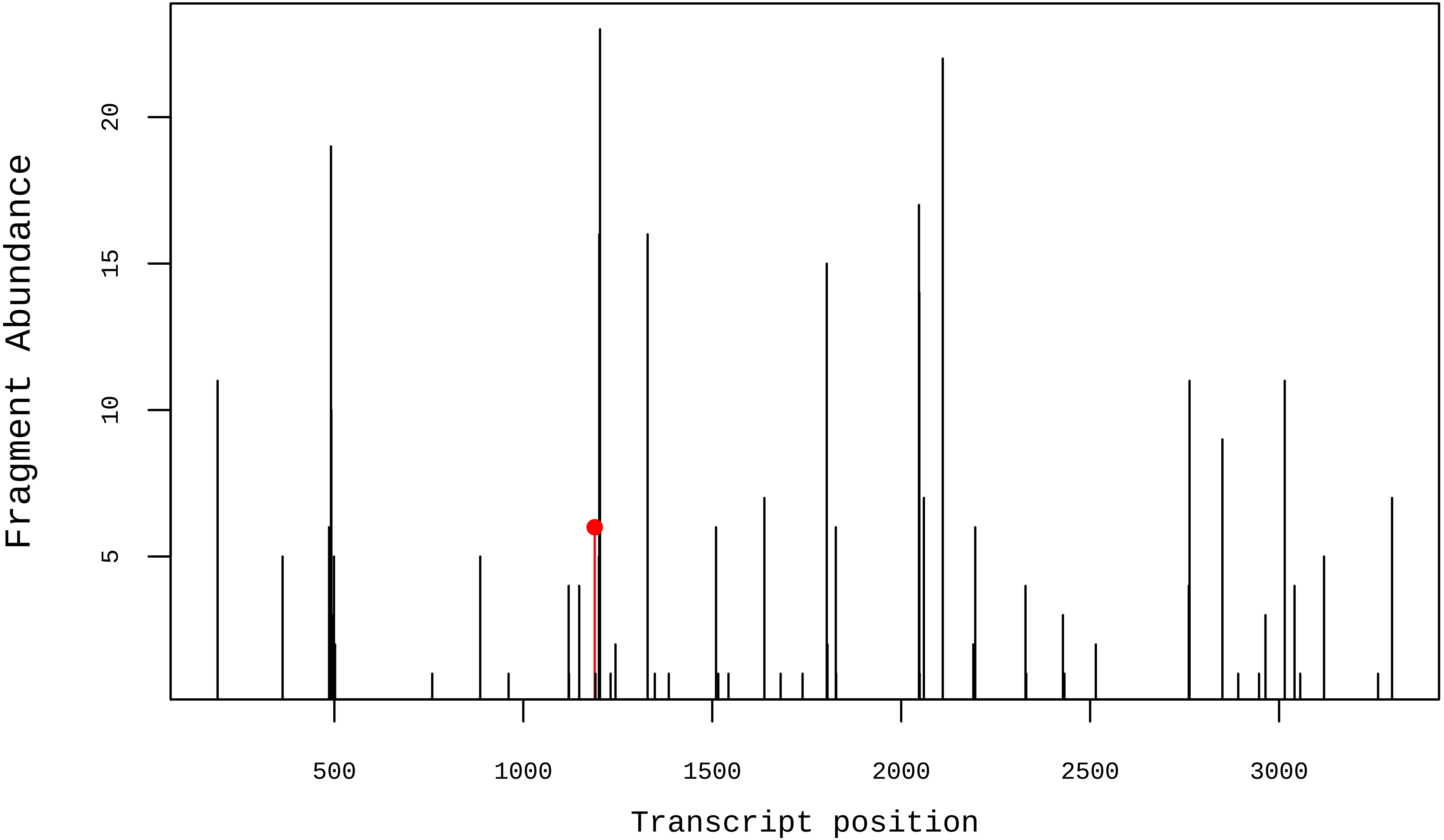

Cleavage site: 1189   Tag abundance: 6   Weighted abundance: 1.5   Category: 3
sRNA abundance: 1   Alignment score: 0   MFE ratio: 1   p-value: 0.031

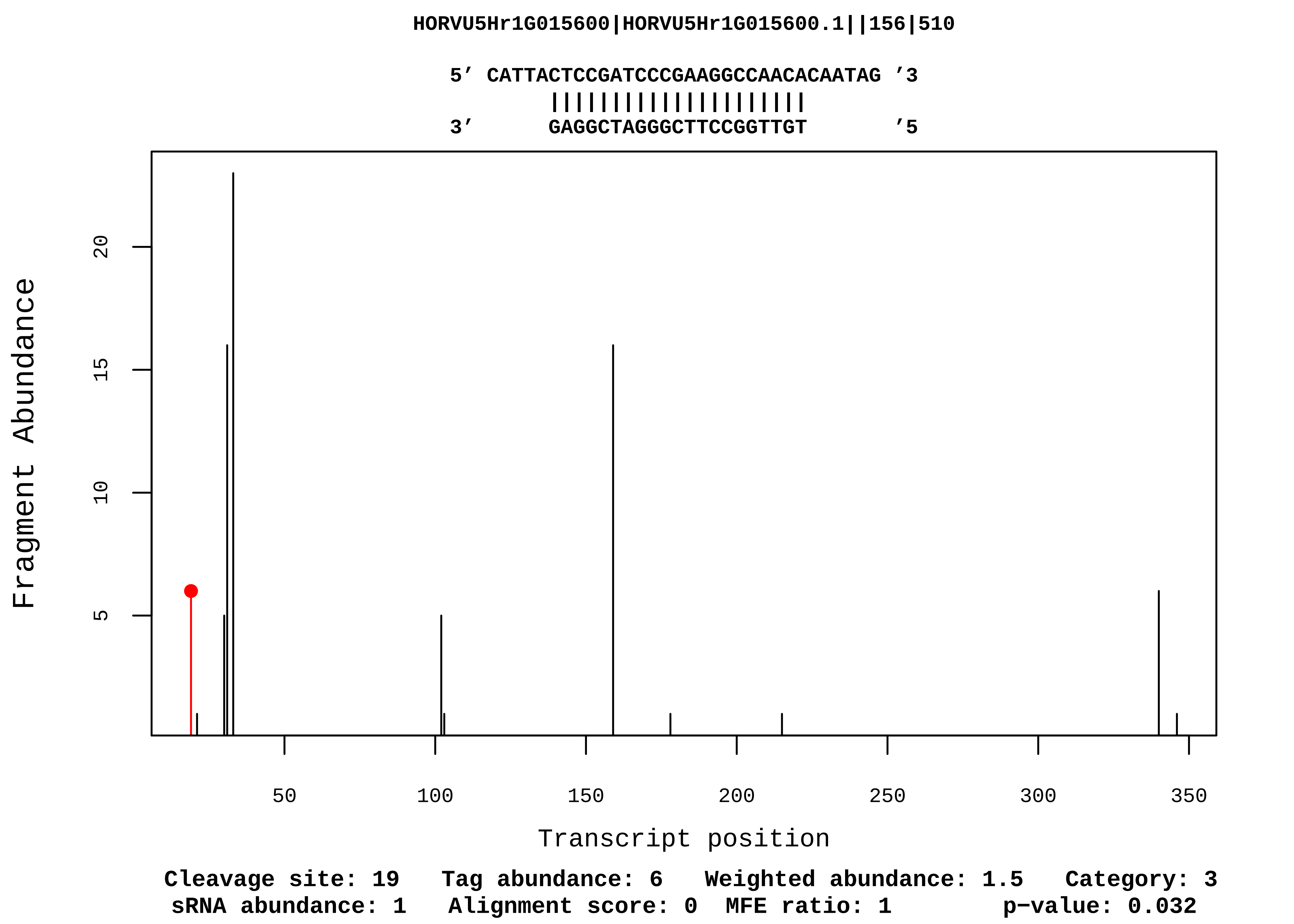

HORVU5Hr1G015600|HORVU5Hr1G015600.1||156|510
5' CATTACTCCGATCCCGAAGGCCAACACAATAG '3
||||||||||||||||||||||
3' GAGGCTAGGGCTTCCGGTTGT '5
Fragment Abundance
5
10
15
20
50
100
150
200
250
300
350
Transcript position
Cleavage site: 19 Tag abundance: 6 Weighted abundance: 1.5 Category: 3
sRNA abundance: 1 Alignment score: 0 MFE ratio: 1 p-value: 0.032

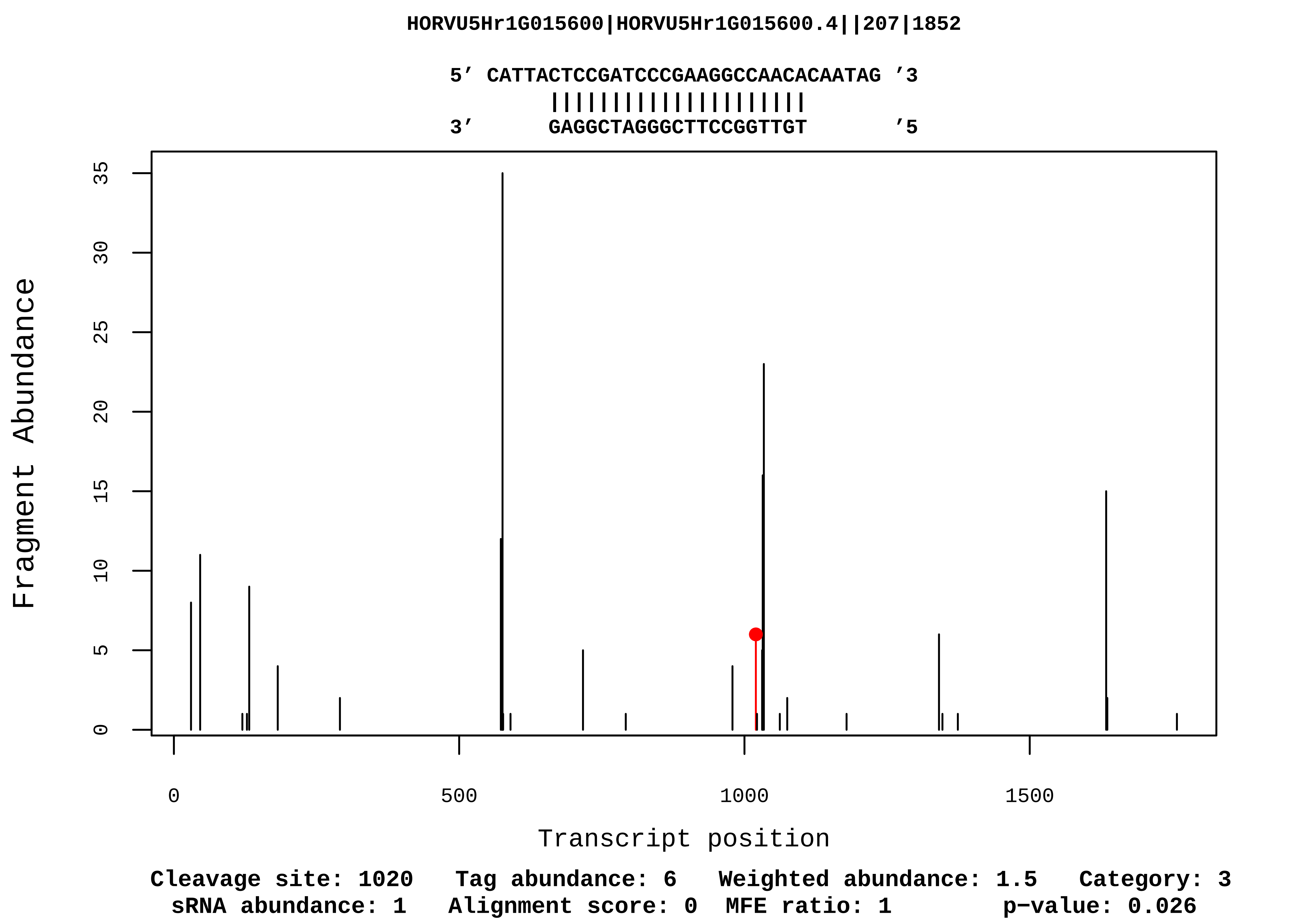
HORVU5Hr1G015600|HORVU5Hr1G015600.4||207|1852
5' CATTACTCCGATCCCGAAGGCCAACACAATAG '3
||||||||||||||||||||
3' GAGGCTAGGGCTTCCGGTTGT '5
Fragment Abundance
0
5
10
15
20
25
30
35
0
500
1000
1500
Transcript position
Cleavage site: 1020 Tag abundance: 6 Weighted abundance: 1.5 Category: 3
sRNA abundance: 1 Alignment score: 0 MFE ratio: 1 p−value: 0.026

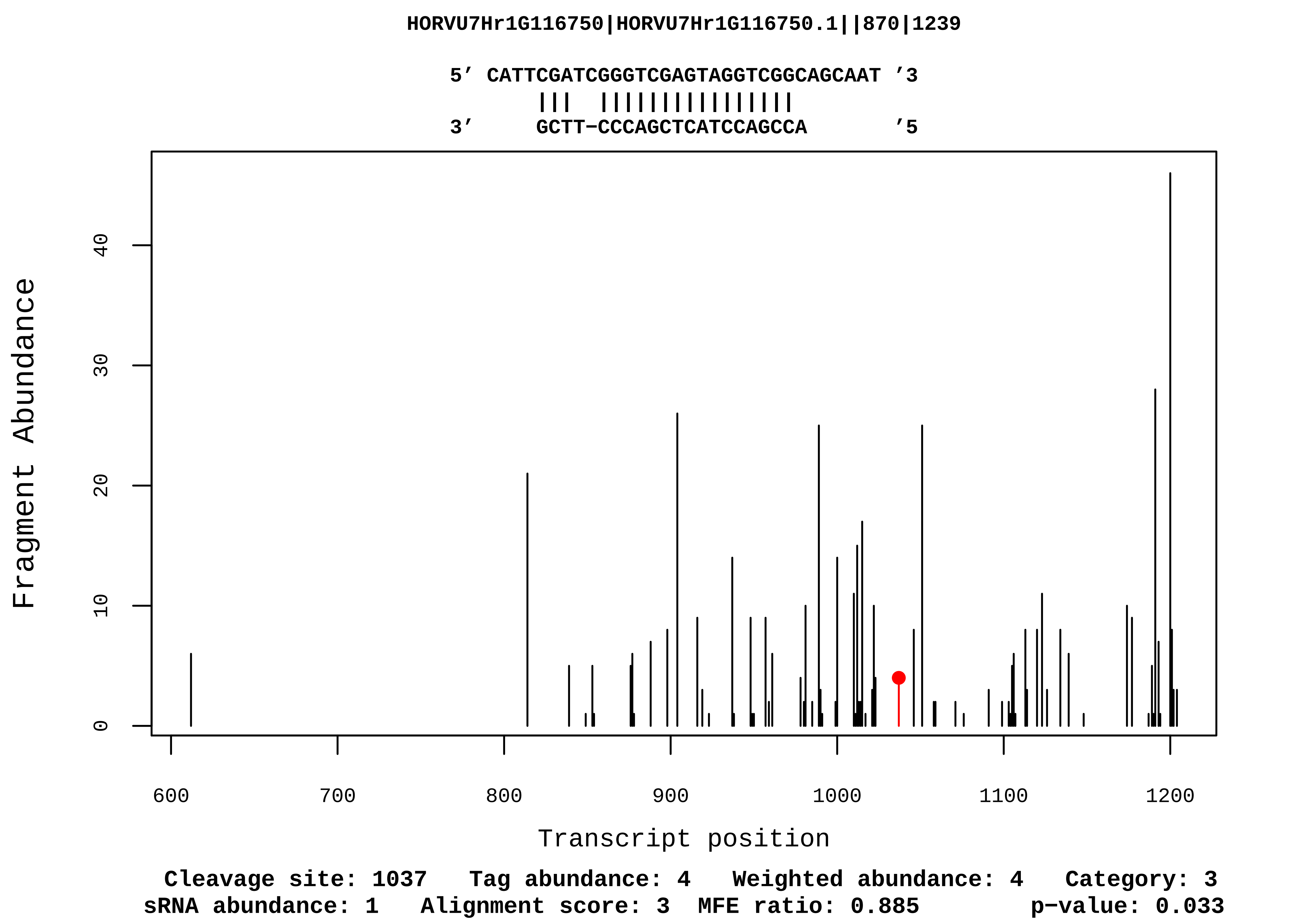
HORVU7Hr1G116750|HORVU7Hr1G116750.1||870|1239
5′ CATTCGATCGGGTCGAGTAGGTCGGCAGCAAT ′3
||| ||||||||||||||||
3′ GCTT−CCCAGCTCATCCAGCCA ′5
Fragment Abundance
0
10
20
30
40
600
700
800
900
1000
1100
1200
Transcript position
Cleavage site: 1037 Tag abundance: 4 Weighted abundance: 4 Category: 3
sRNA abundance: 1 Alignment score: 3 MFE ratio: 0.885 p-value: 0.033

HORVU0Hr1G003660|HORVU0Hr1G003660.1||297|1955

```
5' GCCGGCCGAAGGGTCGAGTAGGTCGGTGCTCG '3
         |||||o|||||||||||||
3'       GCTTCTCAGCTCATCCAGCC       '5
```

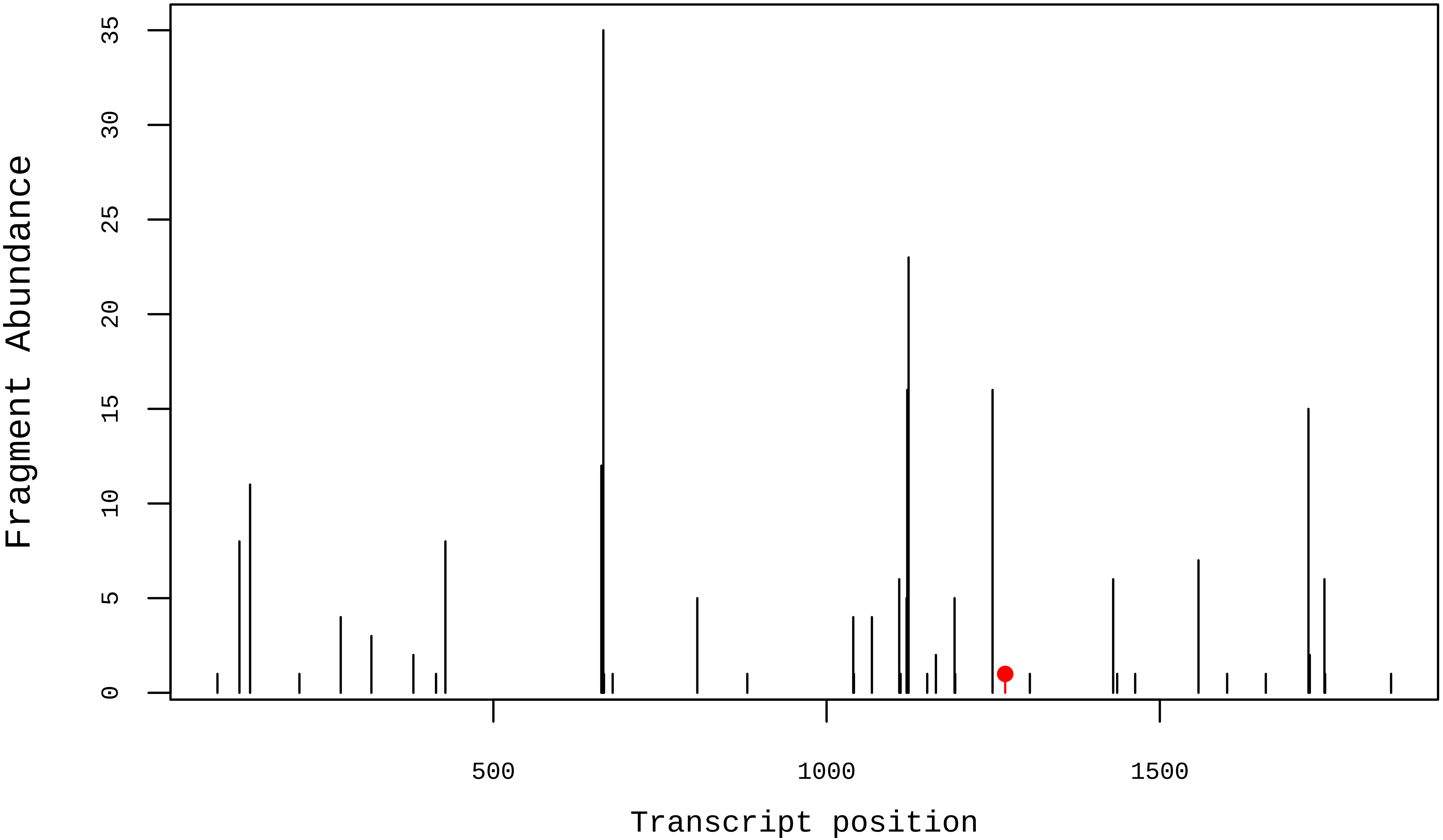

Cleavage site: 1268 Tag abundance: 1 Weighted abundance: 0.143 Category: 4
sRNA abundance: 1 Alignment score: 0.5 MFE ratio: 0.977 p-value: 0.046

HORVU0Hr1G023930|HORVU0Hr1G023930.3||396|2180

```
5' GTCGGCGGAAGGGTCGAGTAGGTCGGTGCTCG '3
          ||||o||||||||||||||
3'        GCTTCTCAGCTCATCCAGCC        '5
```

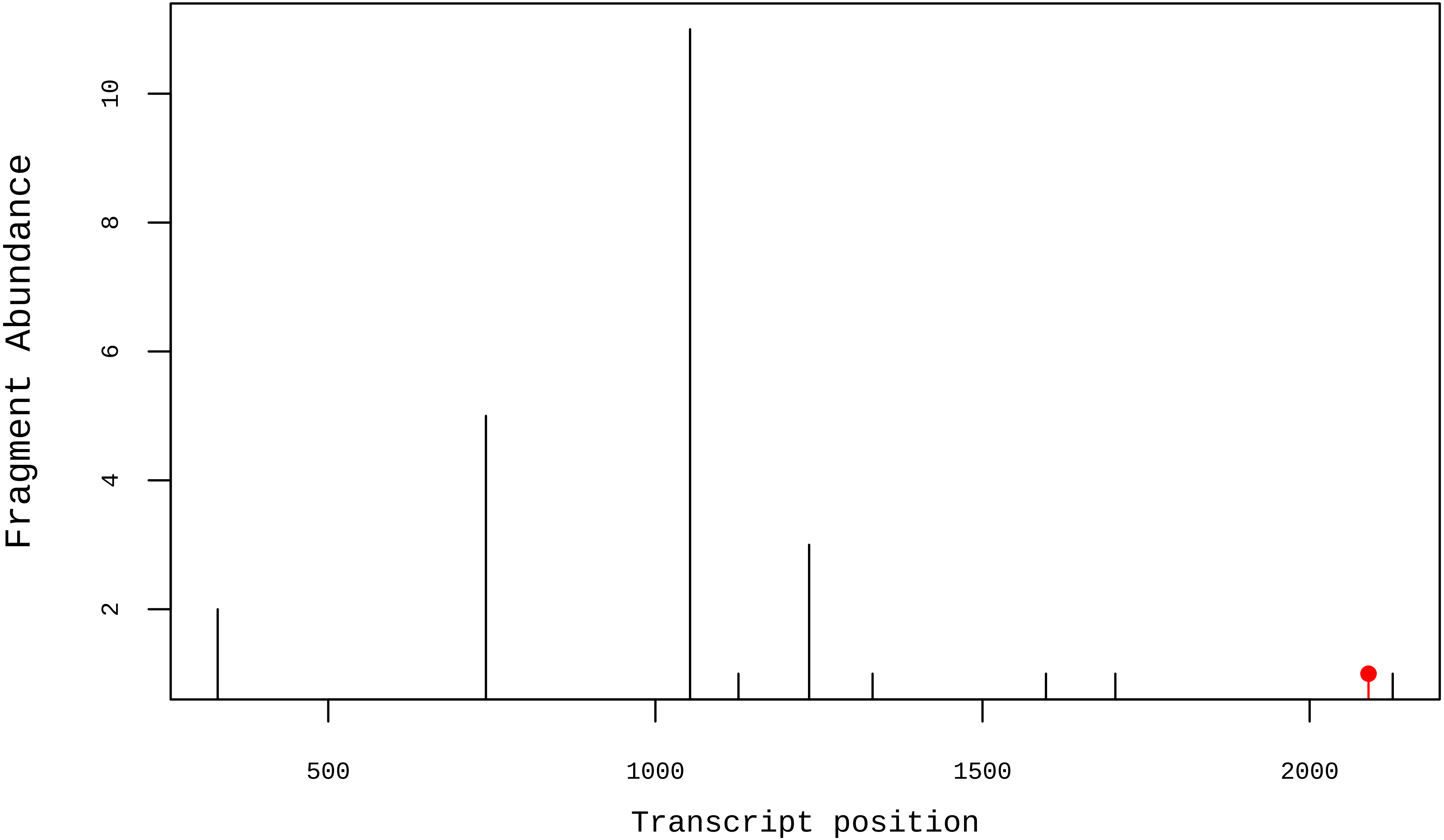

Cleavage site: 2090 Tag abundance: 1 Weighted abundance: 0.143 Category: 4
sRNA abundance: 1 Alignment score: 1.5 MFE ratio: 0.956 p-value: 0.019

HORVU2Hr1G072960|HORVU2Hr1G072960.1||600|3353

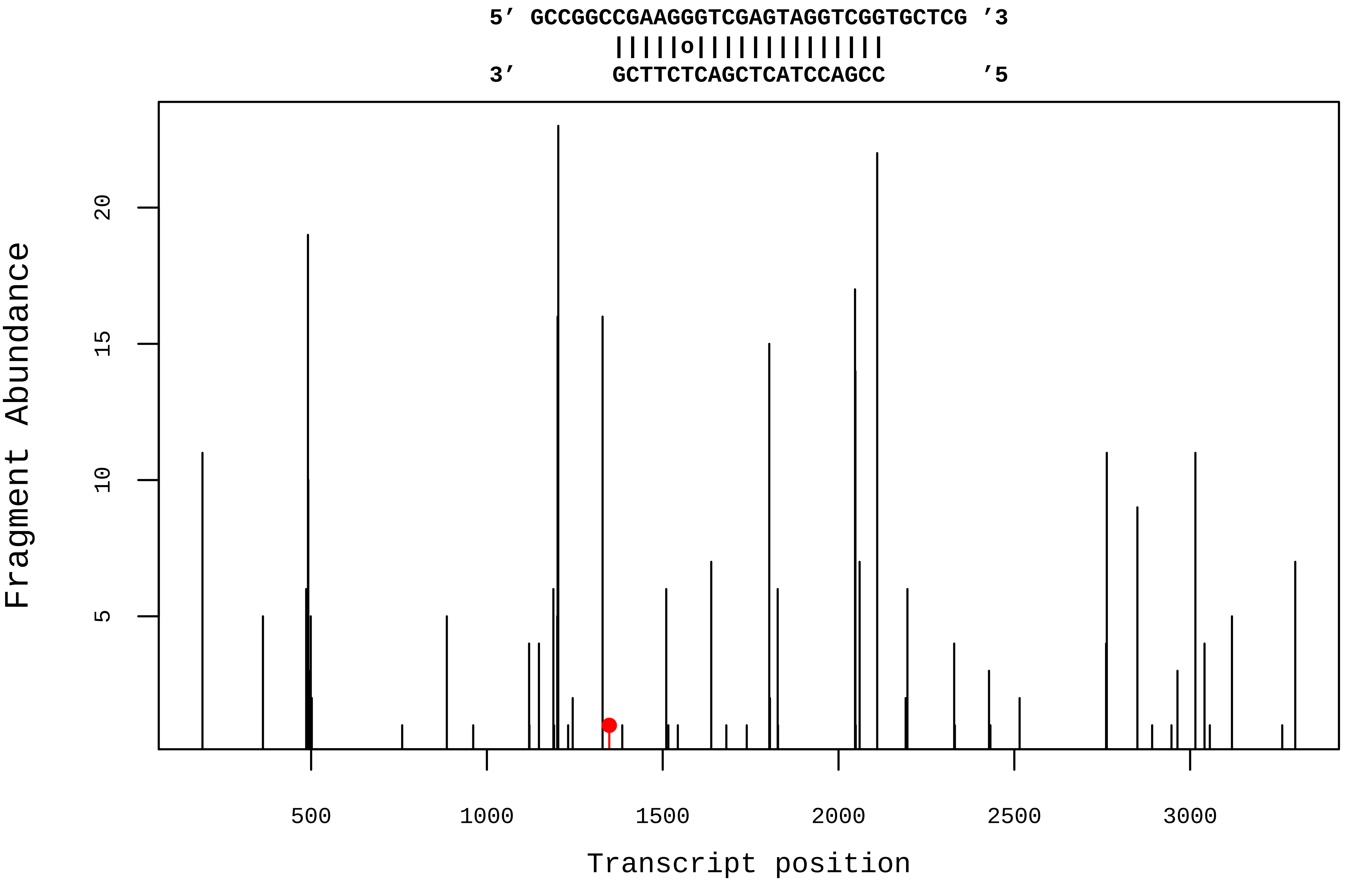

Cleavage site: 1348 Tag abundance: 1 Weighted abundance: 0.143 Category: 4
sRNA abundance: 1 Alignment score: 0.5 MFE ratio: 0.977 p-value: 0.03

HORVU5Hr1G015600|HORVU5Hr1G015600.1||156|510

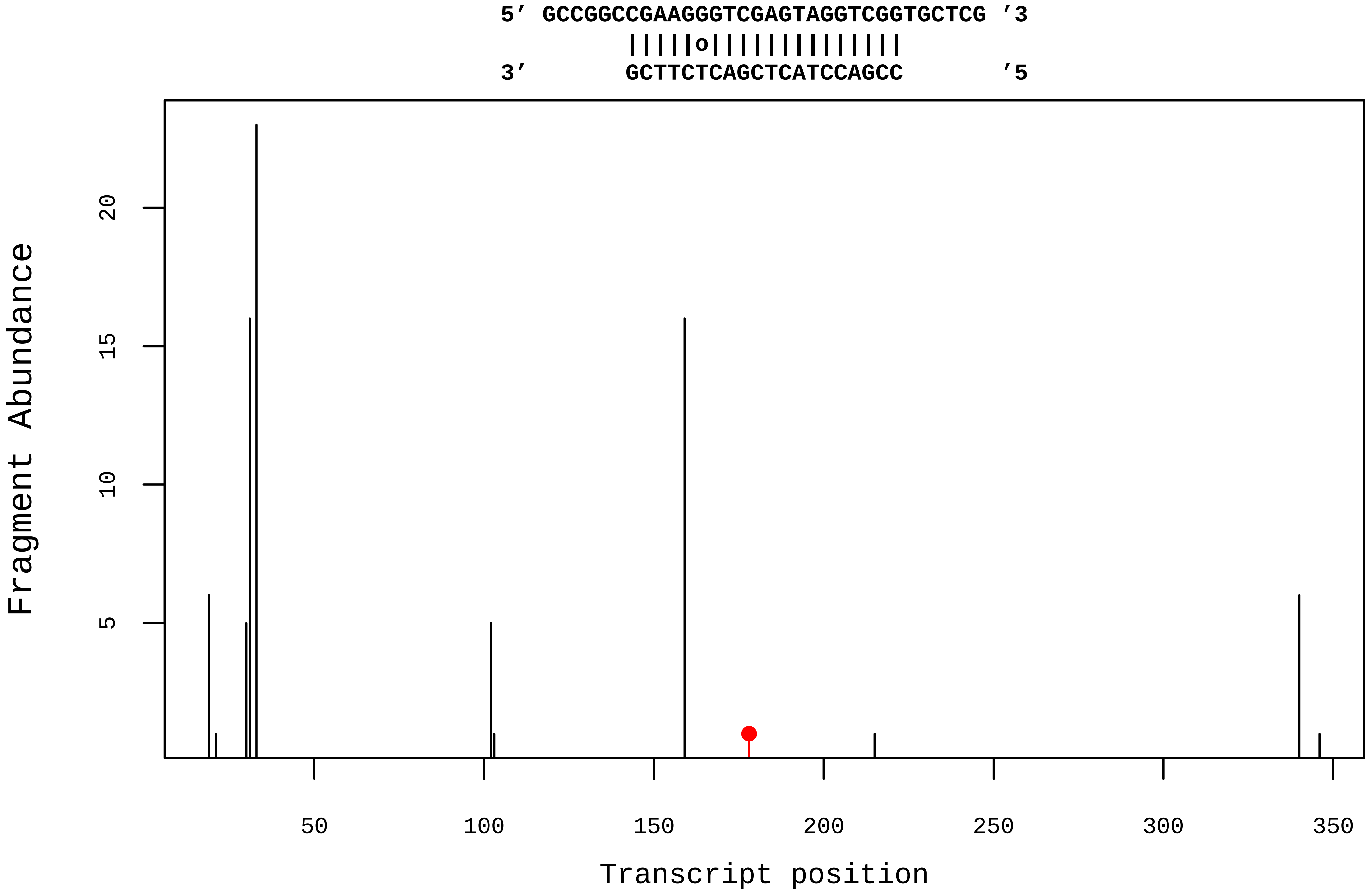

Cleavage site: 178 Tag abundance: 1 Weighted abundance: 0.143 Category: 4
sRNA abundance: 1 Alignment score: 0.5 MFE ratio: 0.977 p-value: 0.05

HORVU5Hr1G015600|HORVU5Hr1G015600.2||231|617

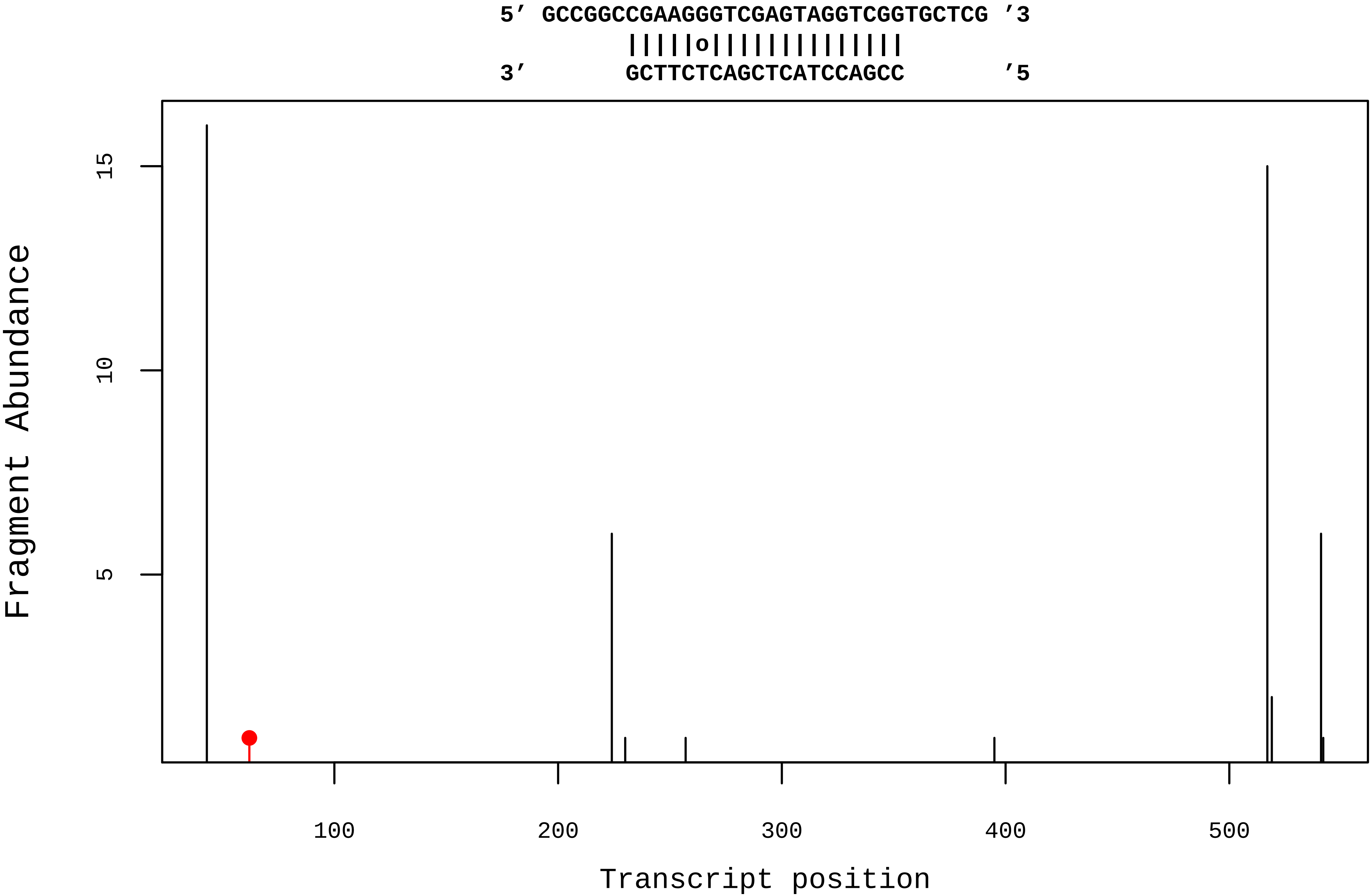

Cleavage site: 62 Tag abundance: 1 Weighted abundance: 0.143 Category: 4
sRNA abundance: 1 Alignment score: 0.5 MFE ratio: 0.977 p−value: 0.041

HORVU5Hr1G015600|HORVU5Hr1G015600.3||276|1709

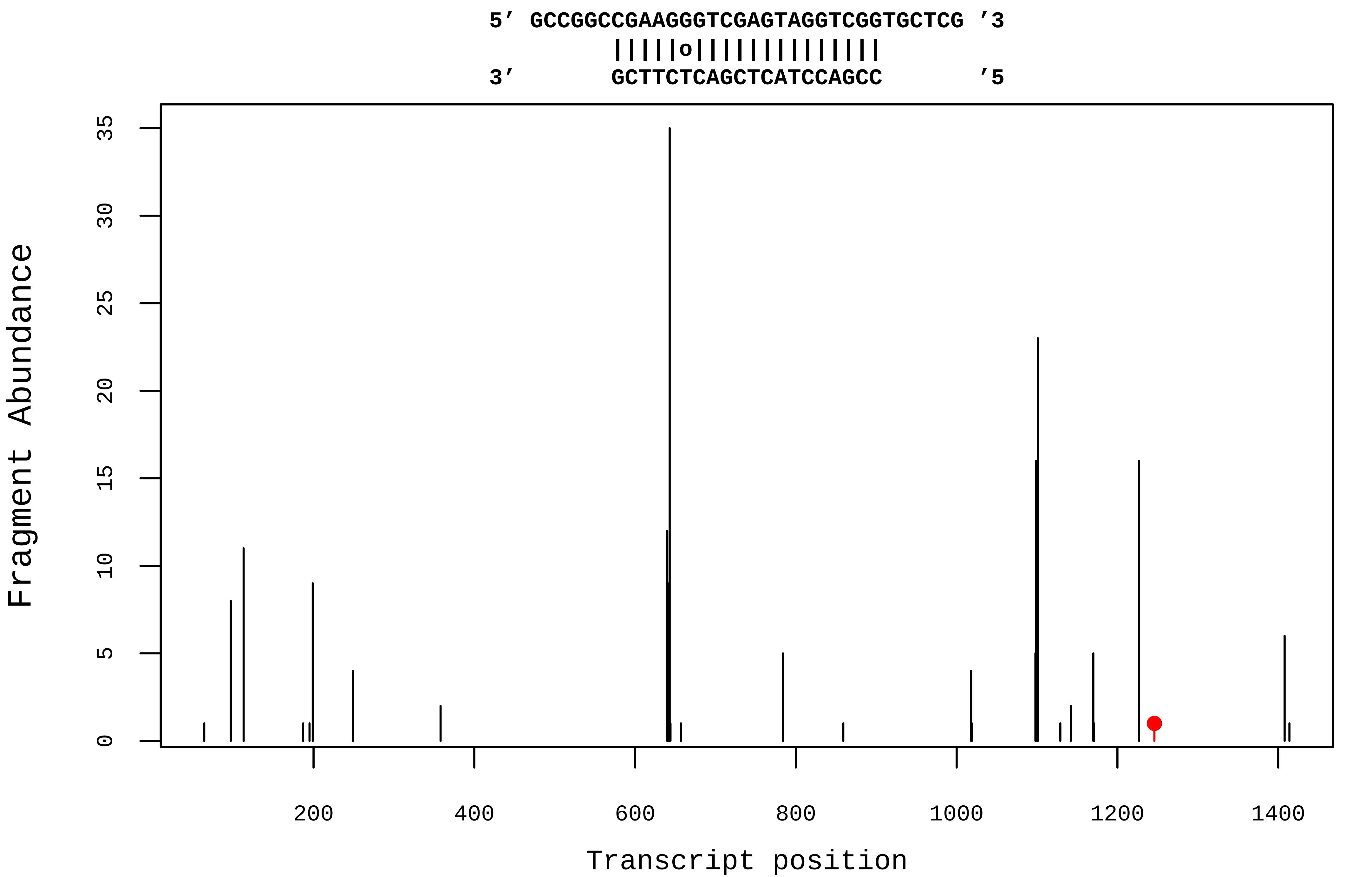

Cleavage site: 1246 Tag abundance: 1 Weighted abundance: 0.143 Category: 4
sRNA abundance: 1 Alignment score: 0.5 MFE ratio: 0.977 p-value: 0.032

HORVU5Hr1G015600|HORVU5Hr1G015600.4||207|1852

5′ GCCGGCCGCAGGGTCGAGTAGGTCGGTGCTCG ′3

3′ GCTTCTCAGCTCATCCAGCC ′5

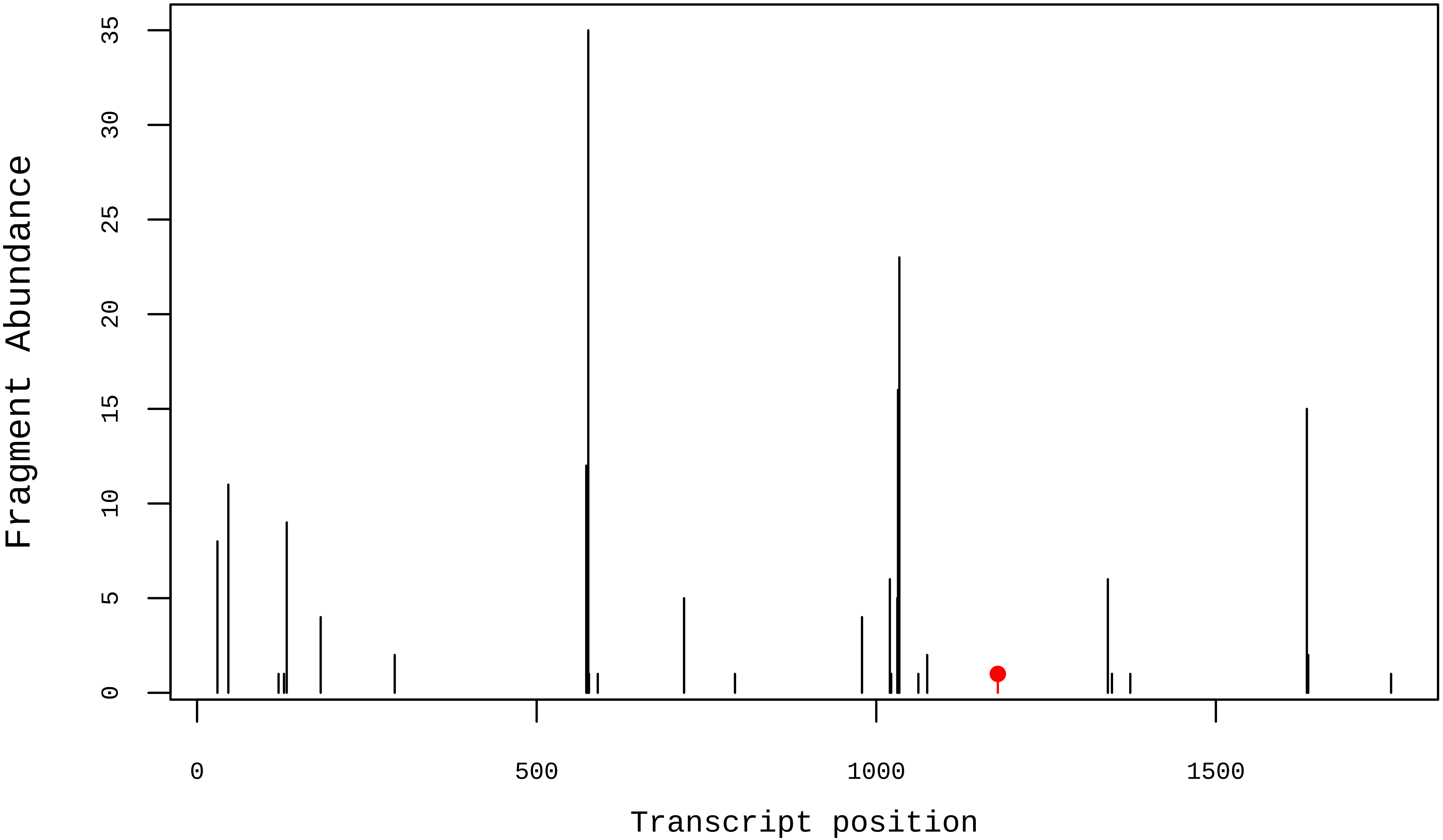

Cleavage site: 1179 Tag abundance: 1 Weighted abundance: 0.143 Category: 4
sRNA abundance: 1 Alignment score: 1.5 MFE ratio: 0.861 p-value: 0.041

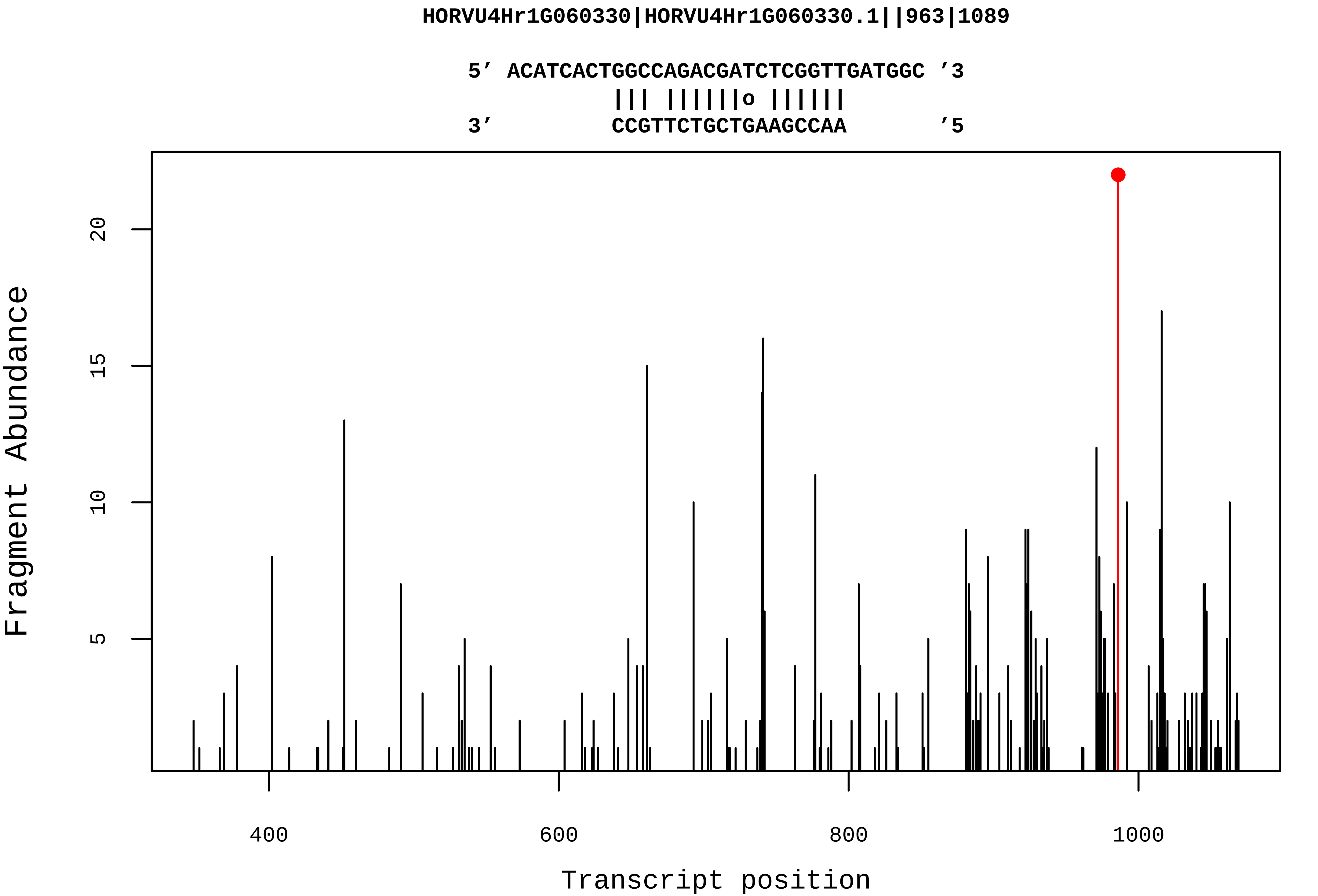

Cleavage site: 986 Tag abundance: 22 Weighted abundance: 3.667 Category: 0
sRNA abundance: 1 Alignment score: 4 MFE ratio: 0.7 p-value: 0.008

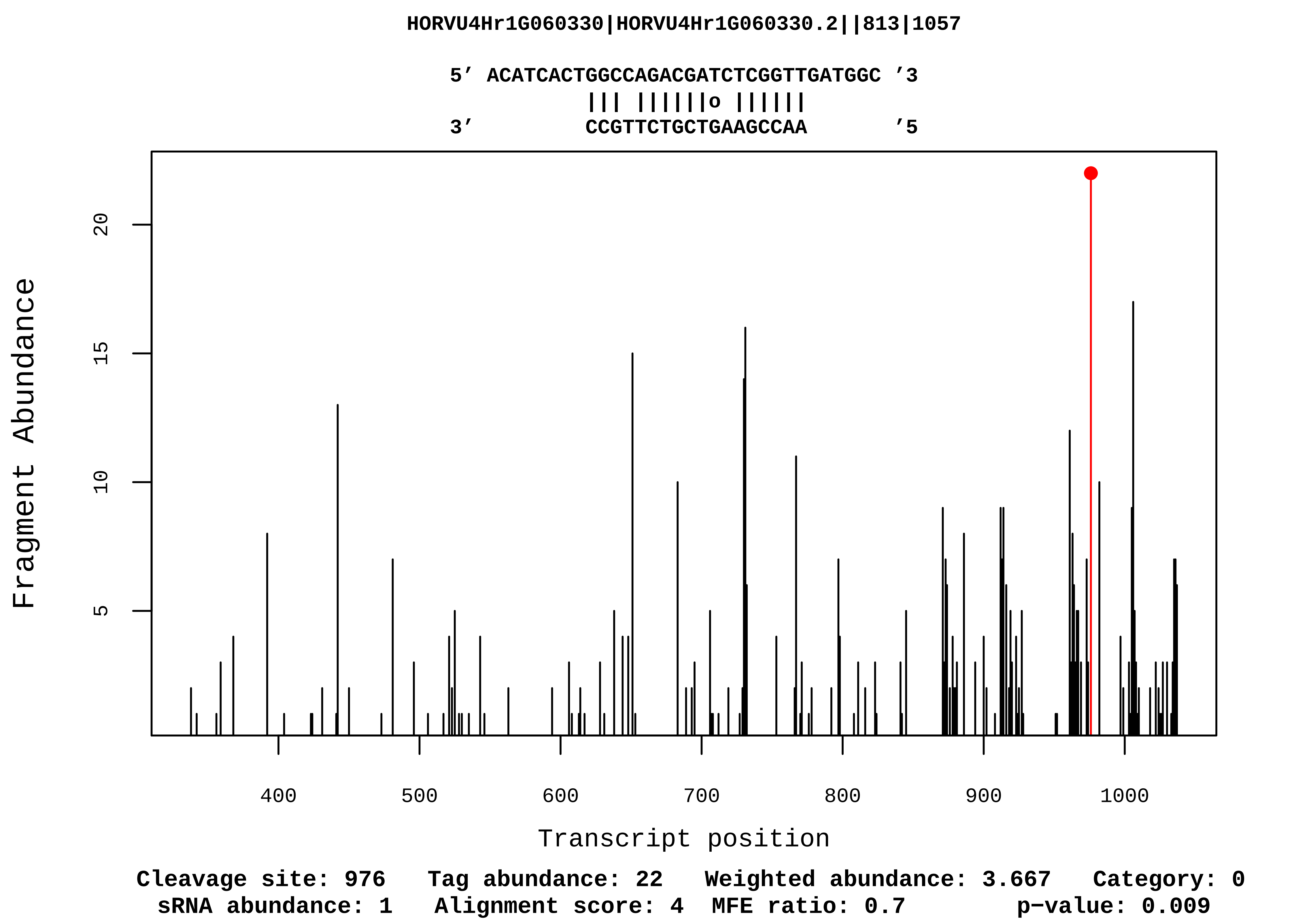
HORVU4Hr1G060330|HORVU4Hr1G060330.2||813|1057
5' ACATCACTGGCCAGACGATCTCGGTTGATGGC '3
||| ||||||o ||||||
3' CCGTTCTGCTGAAGCCAA '5
Fragment Abundance
5
10
15
20
400
500
600
700
800
900
1000
Transcript position
Cleavage site: 976 Tag abundance: 22 Weighted abundance: 3.667 Category: 0
sRNA abundance: 1 Alignment score: 4 MFE ratio: 0.7 p-value: 0.009

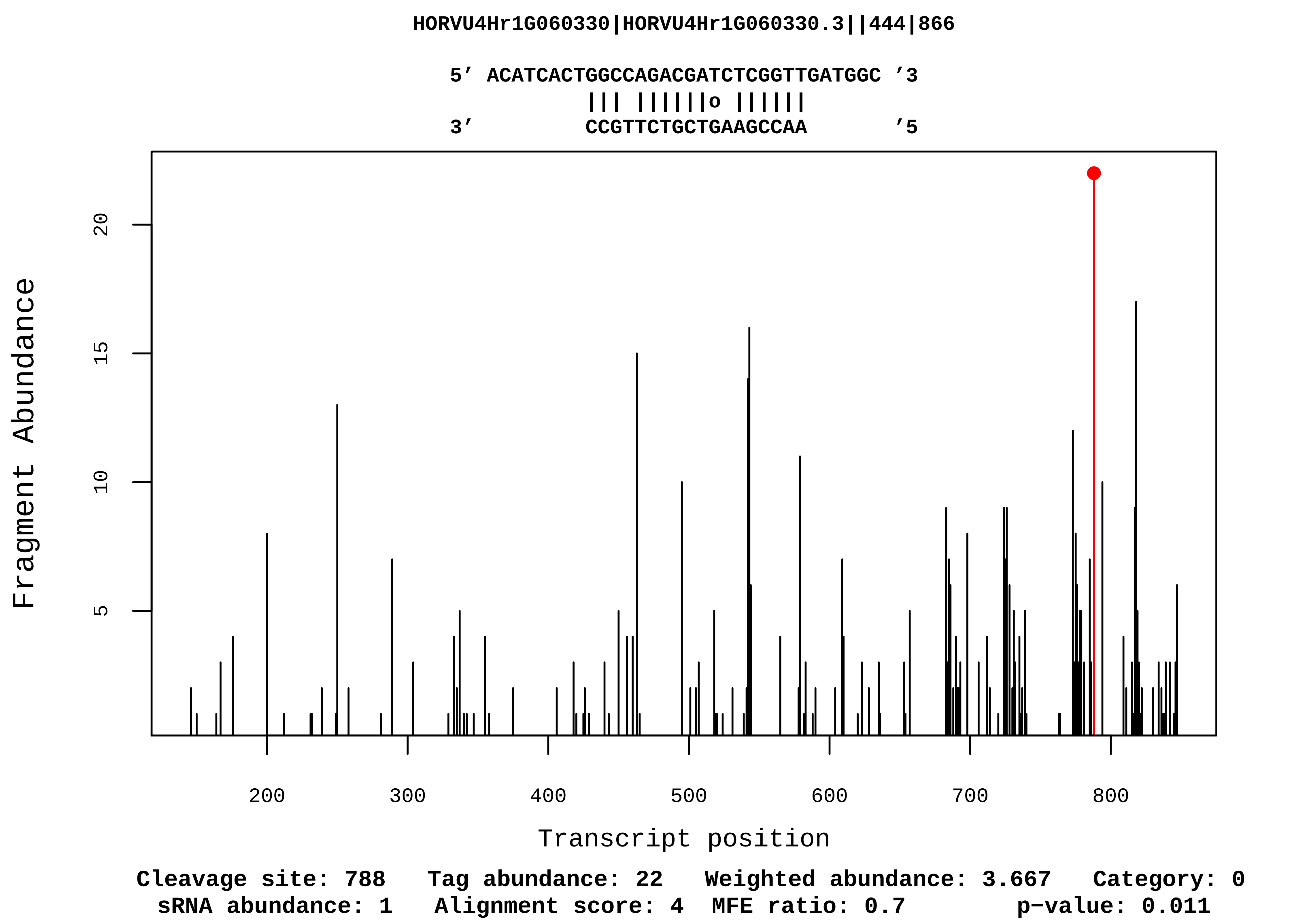

HORVU4Hr1G060330|HORVU4Hr1G060330.3||444|866
5' ACATCACTGGCCAGACGATCTCGGTTGATGGC '3
||| ||||||o ||||||
3' CCGTTCTGCTGAAGCCAA '5
Fragment Abundance
5
10
15
20
200
300
400
500
600
700
800
Transcript position
Cleavage site: 788 Tag abundance: 22 Weighted abundance: 3.667 Category: 0
sRNA abundance: 1 Alignment score: 4 MFE ratio: 0.7 p-value: 0.011

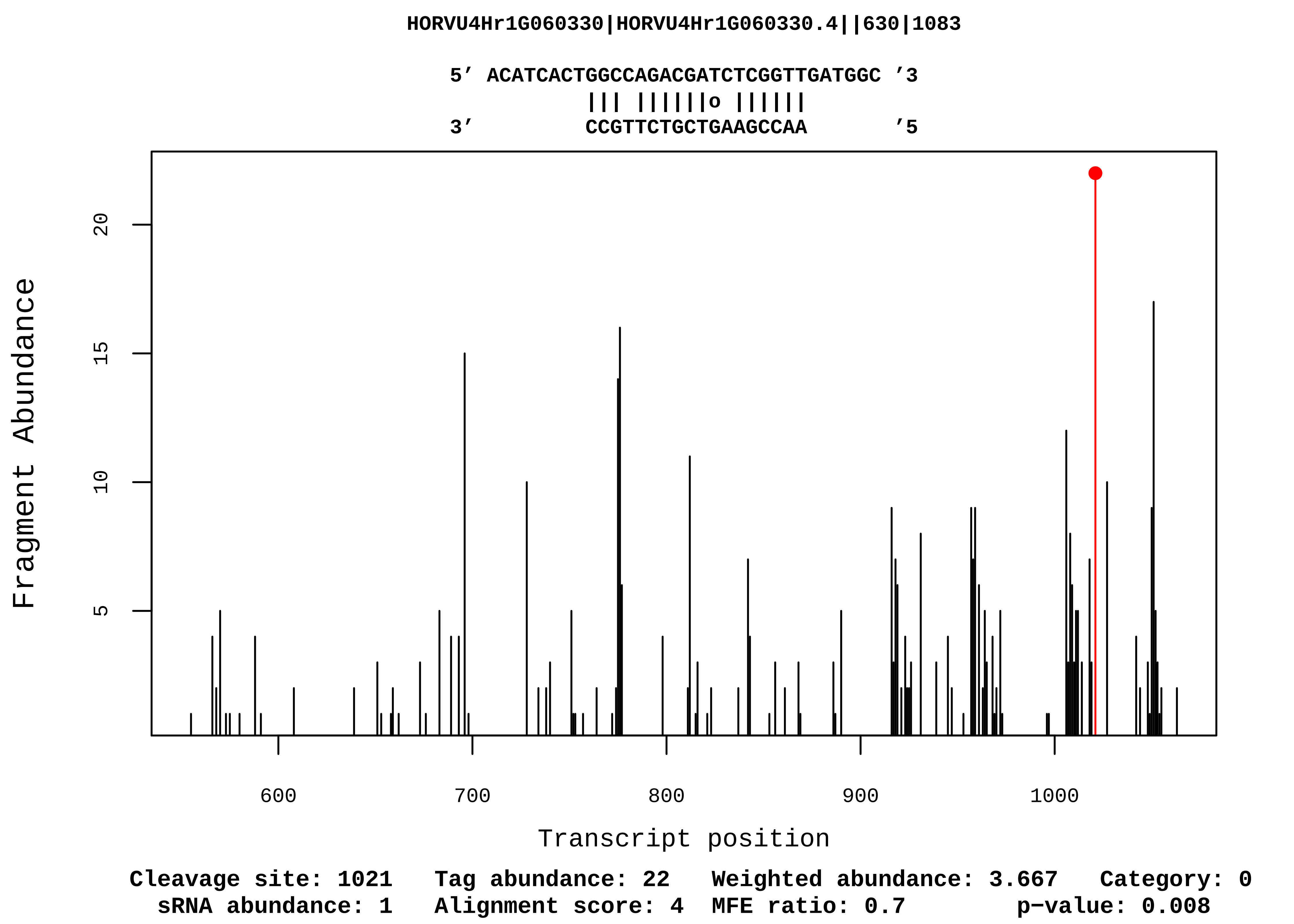
HORVU4Hr1G060330|HORVU4Hr1G060330.4||630|1083
5' ACATCACTGGCCAGACGATCTCGGTTGATGGC '3
||| ||||||o ||||||
3' CCGTTCTGCTGAAGCCAA '5
Fragment Abundance
5
10
15
20
600
700
800
900
1000
Transcript position
Cleavage site: 1021
Tag abundance: 22
Weighted abundance: 3.667
Category: 0
sRNA abundance: 1
Alignment score: 4
MFE ratio: 0.7
p-value: 0.008

HORVU4Hr1G060330|HORVU4Hr1G060330.5||444|897

```
5' ACATCACTGGCCAGACGATCTCGGTTGATGGC '3
              ||| ||||||o ||||||
3'            CCGTTCTGCTGAAGCCAA         '5
```

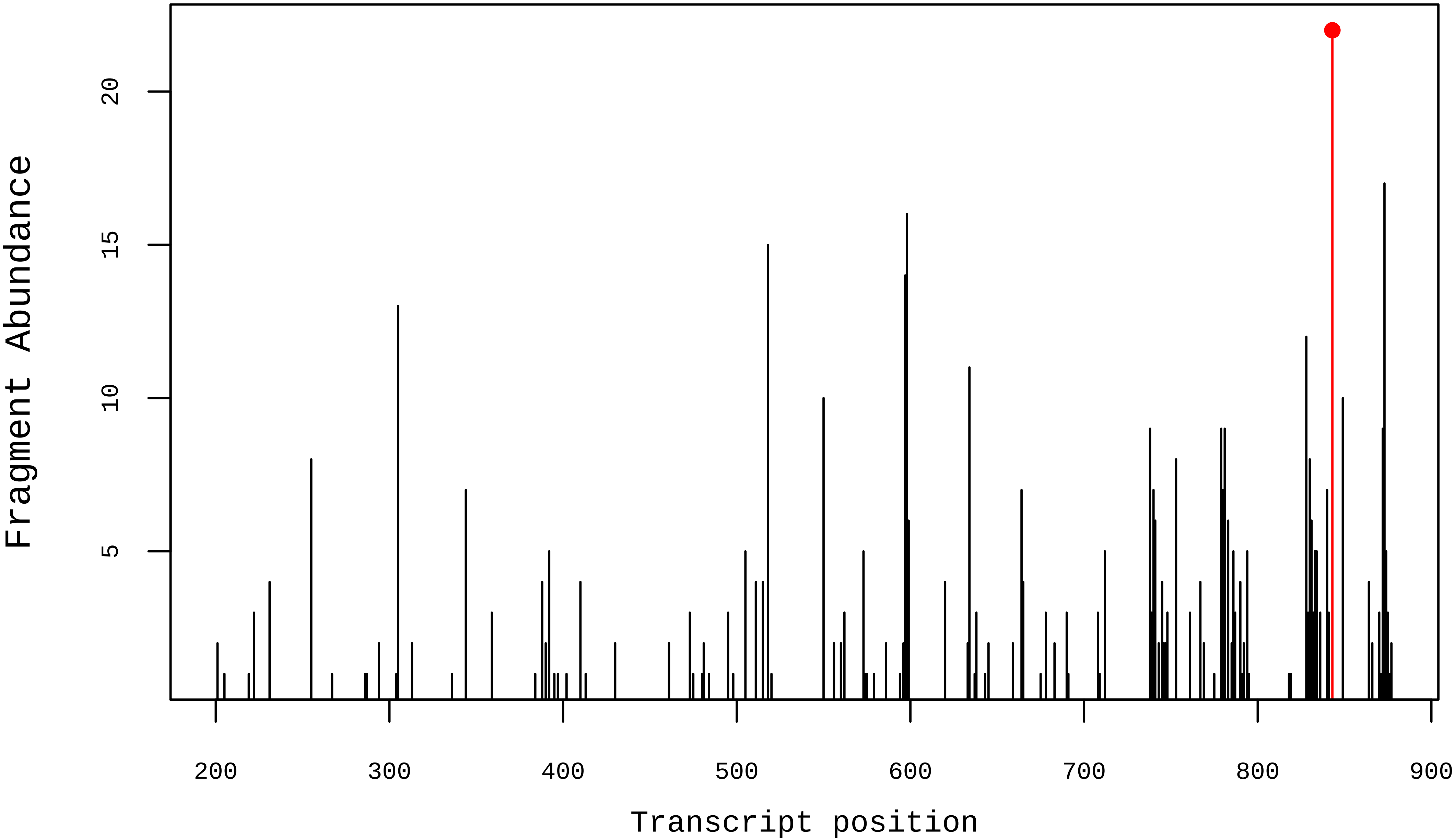

Cleavage site: 843   Tag abundance: 22   Weighted abundance: 3.667   Category: 0
sRNA abundance: 1   Alignment score: 4   MFE ratio: 0.7   p-value: 0.01

HORVU4Hr1G060330|HORVU4Hr1G060330.6||117|300

```
5' ACATCACTGGCCAGACGATCTCGGTTGATGGC '3
          ||| ||||||o ||||||
3'        CCGTTCTGCTGAAGCCAA        '5
```

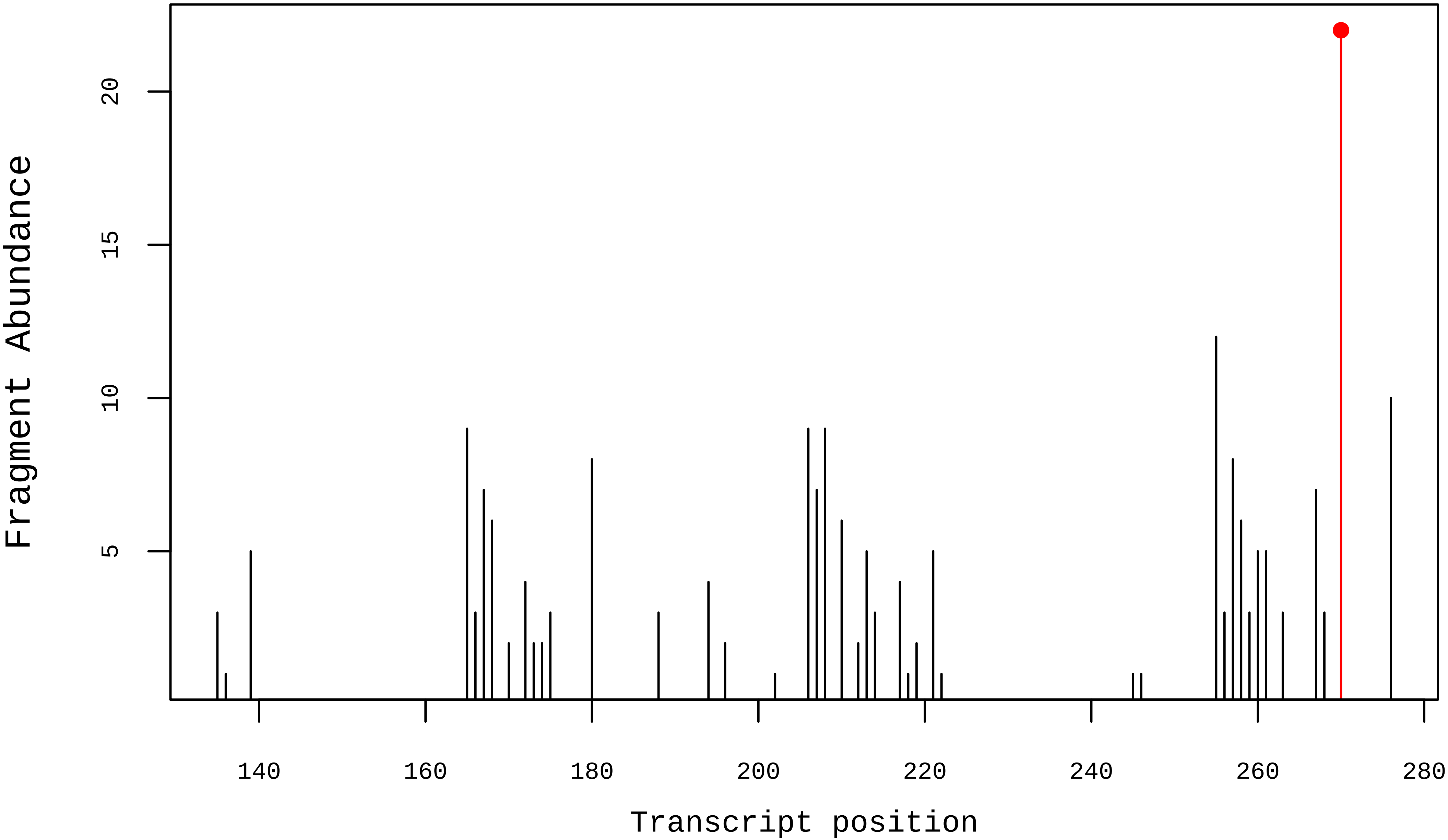

Cleavage site: 270   Tag abundance: 22   Weighted abundance: 3.667   Category: 0
sRNA abundance: 1   Alignment score: 4   MFE ratio: 0.7   p-value: 0.032

HORVU0Hr1G023930|HORVU0Hr1G023930.1||297|1937

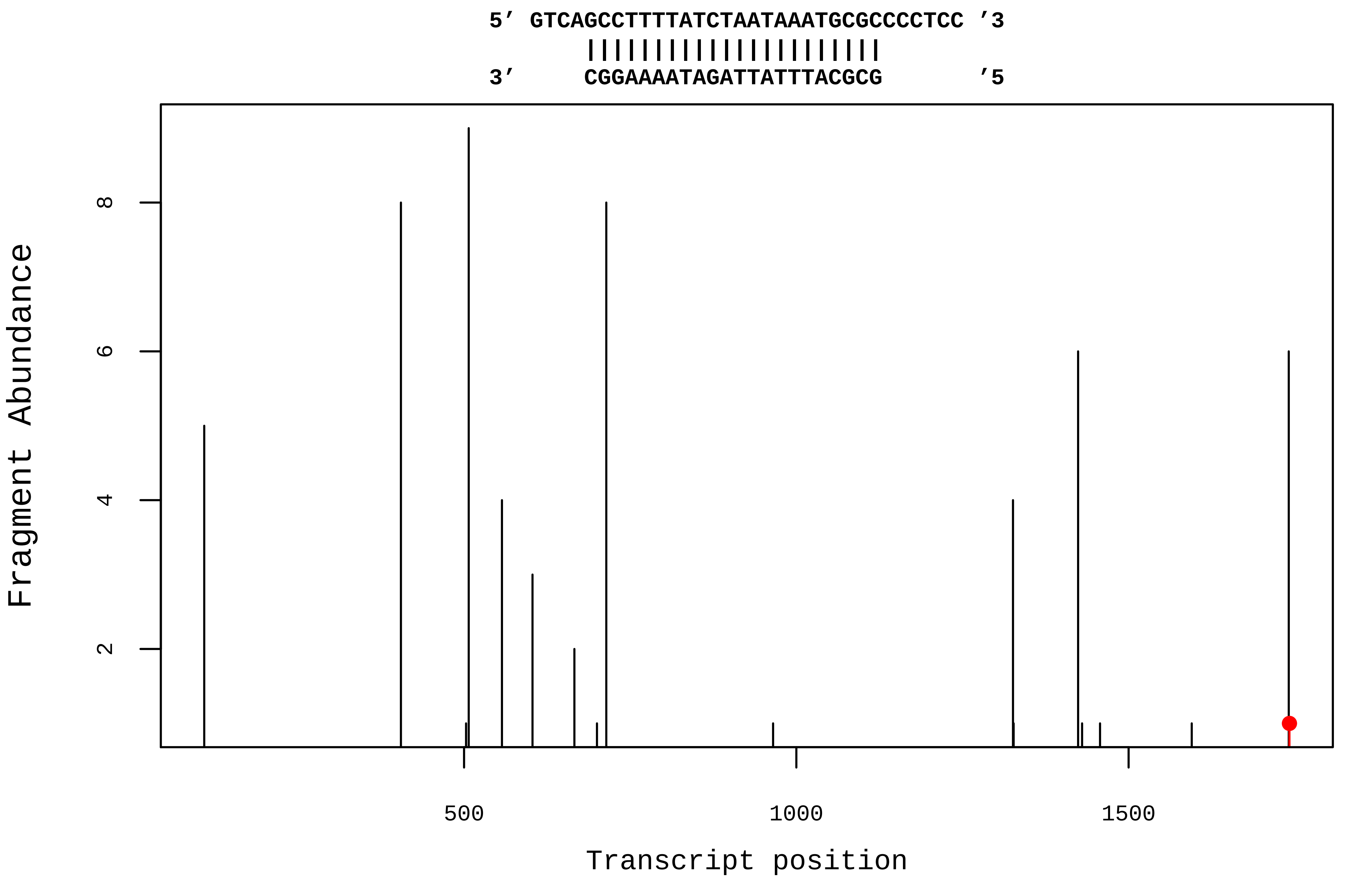

Cleavage site: 1742 Tag abundance: 1 Weighted abundance: 0.125 Category: 4
sRNA abundance: 1 Alignment score: 0 MFE ratio: 1 p-value: 0.033

HORVU2Hr1G072960|HORVU2Hr1G072960.1||600|3353

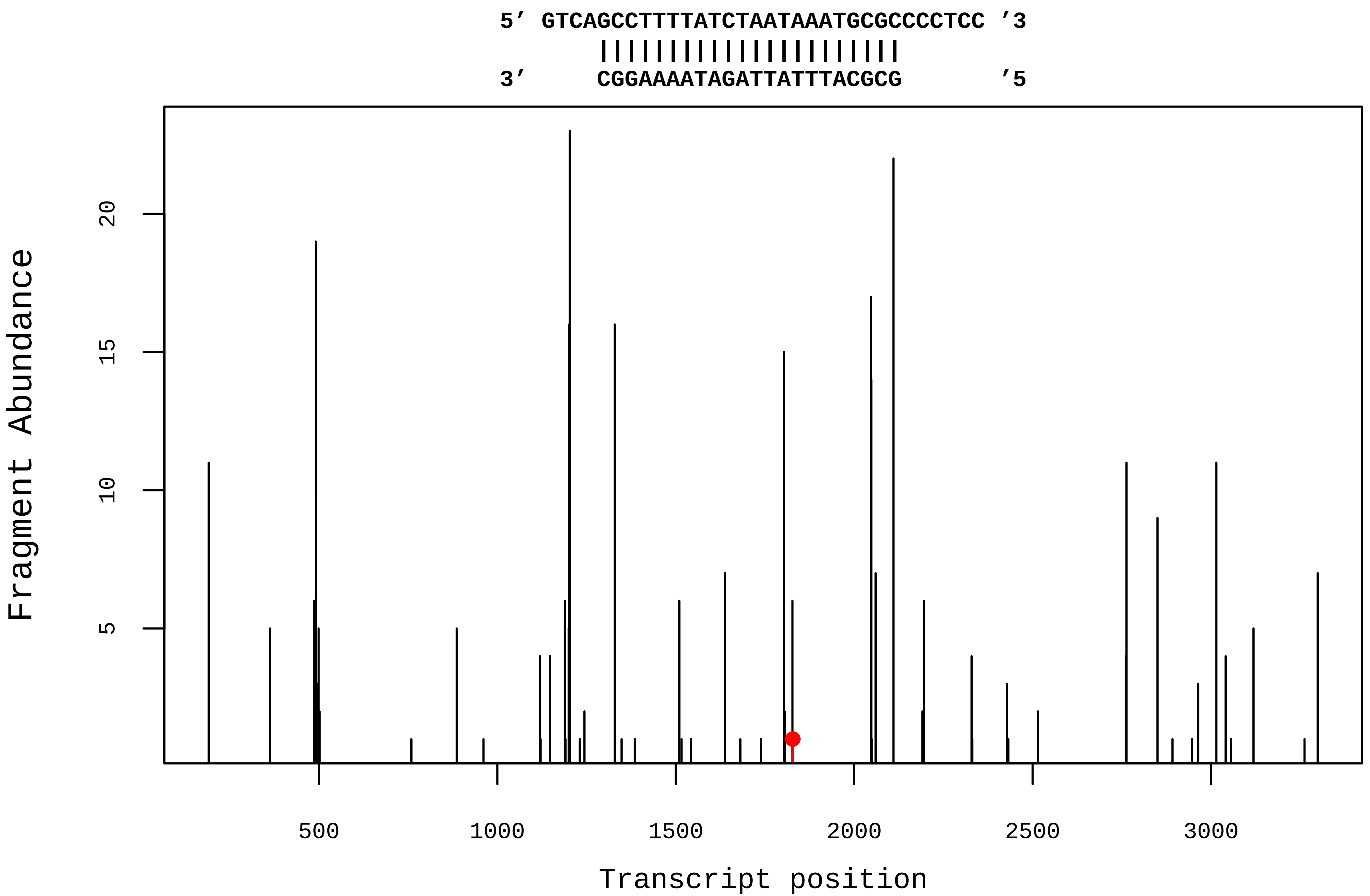

Cleavage site: 1828 Tag abundance: 1 Weighted abundance: 0.125 Category: 4
sRNA abundance: 1 Alignment score: 0 MFE ratio: 1 p-value: 0.047

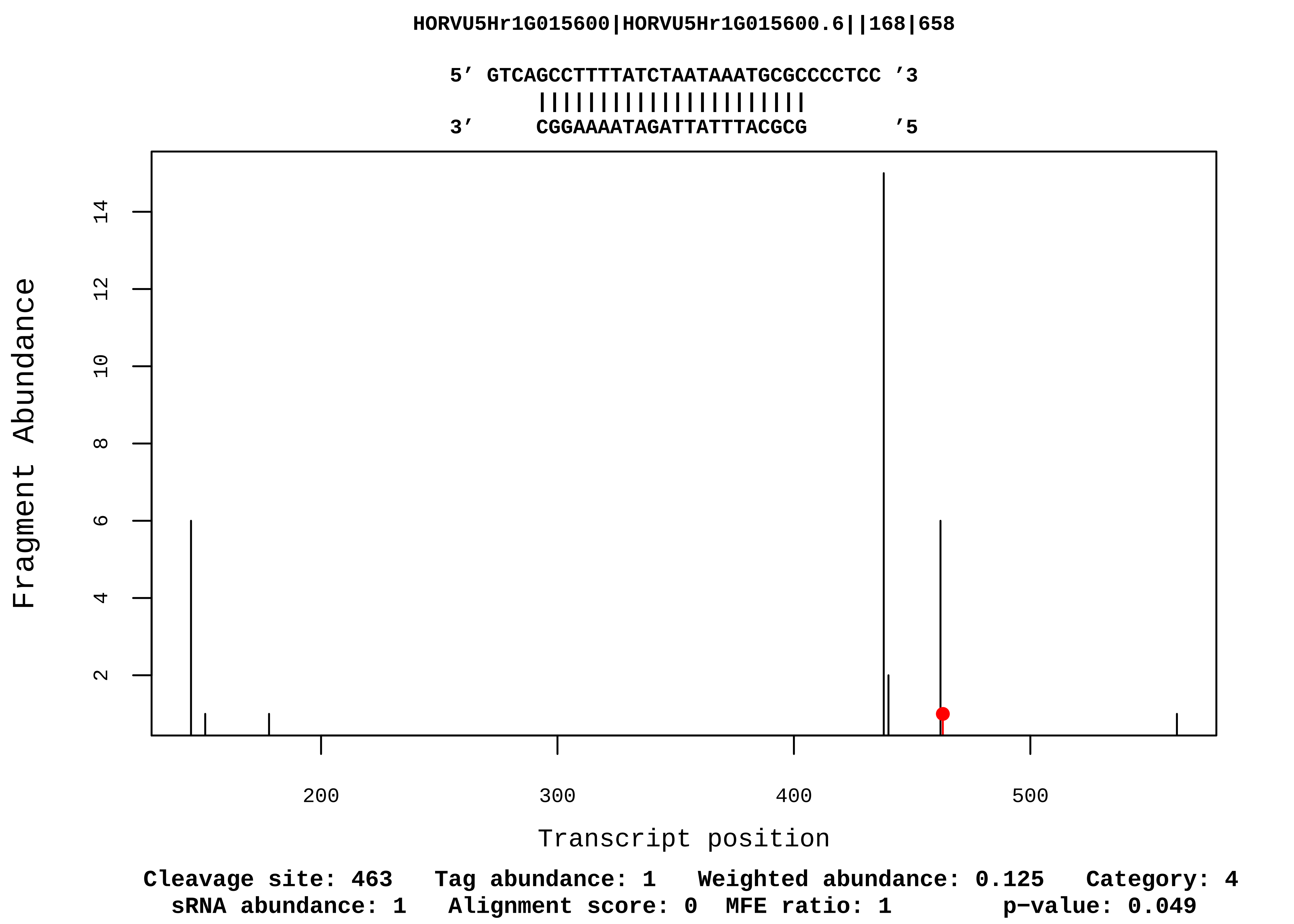
HORVU5Hr1G015600|HORVU5Hr1G015600.6||168|658
5′ GTCAGCCTTTTATCTAATAAATGCGCCCCTCC ′3
3′ CGGAAAATAGATTATTTACGCG ′5
Fragment Abundance
2
4
6
8
10
12
14
200
300
400
500
Transcript position
Cleavage site: 463 Tag abundance: 1 Weighted abundance: 0.125 Category: 4
sRNA abundance: 1 Alignment score: 0 MFE ratio: 1 p-value: 0.049

HORVU3Hr1G086030|HORVU3Hr1G086030.1||1308|3149

```
5' AGCGCGGCCGCGT-CGGGCTCGCGCCCGTCCGC '3
         ||  ||o |||||||||||||
3'       CGAGGCGGGCCCGAGCGCGGG       '5
```

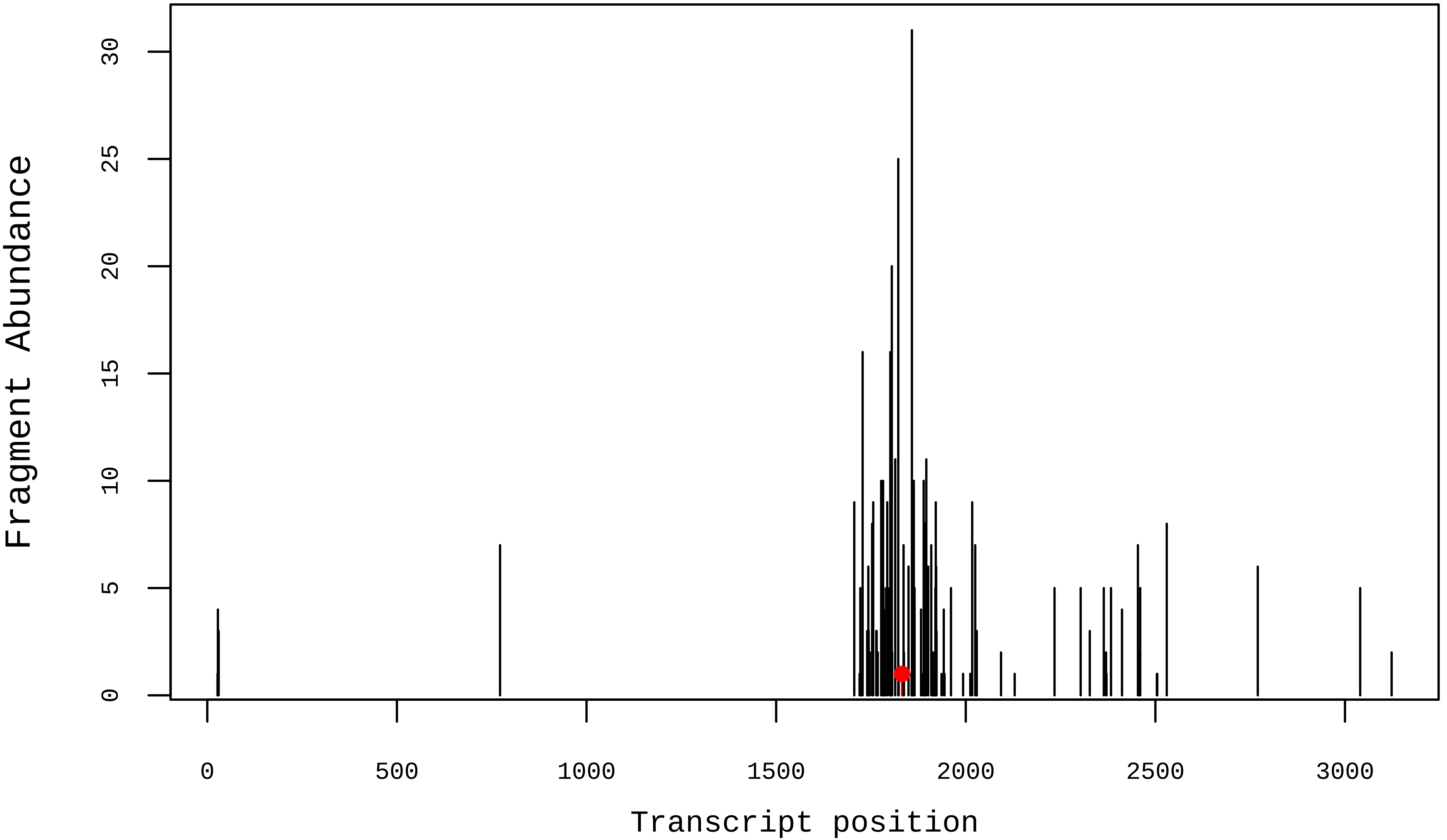

Cleavage site: 1832   Tag abundance: 1   Weighted abundance: 1   Category: 4
sRNA abundance: 1   Alignment score: 3.5   MFE ratio: 0.716   p-value: 0.008

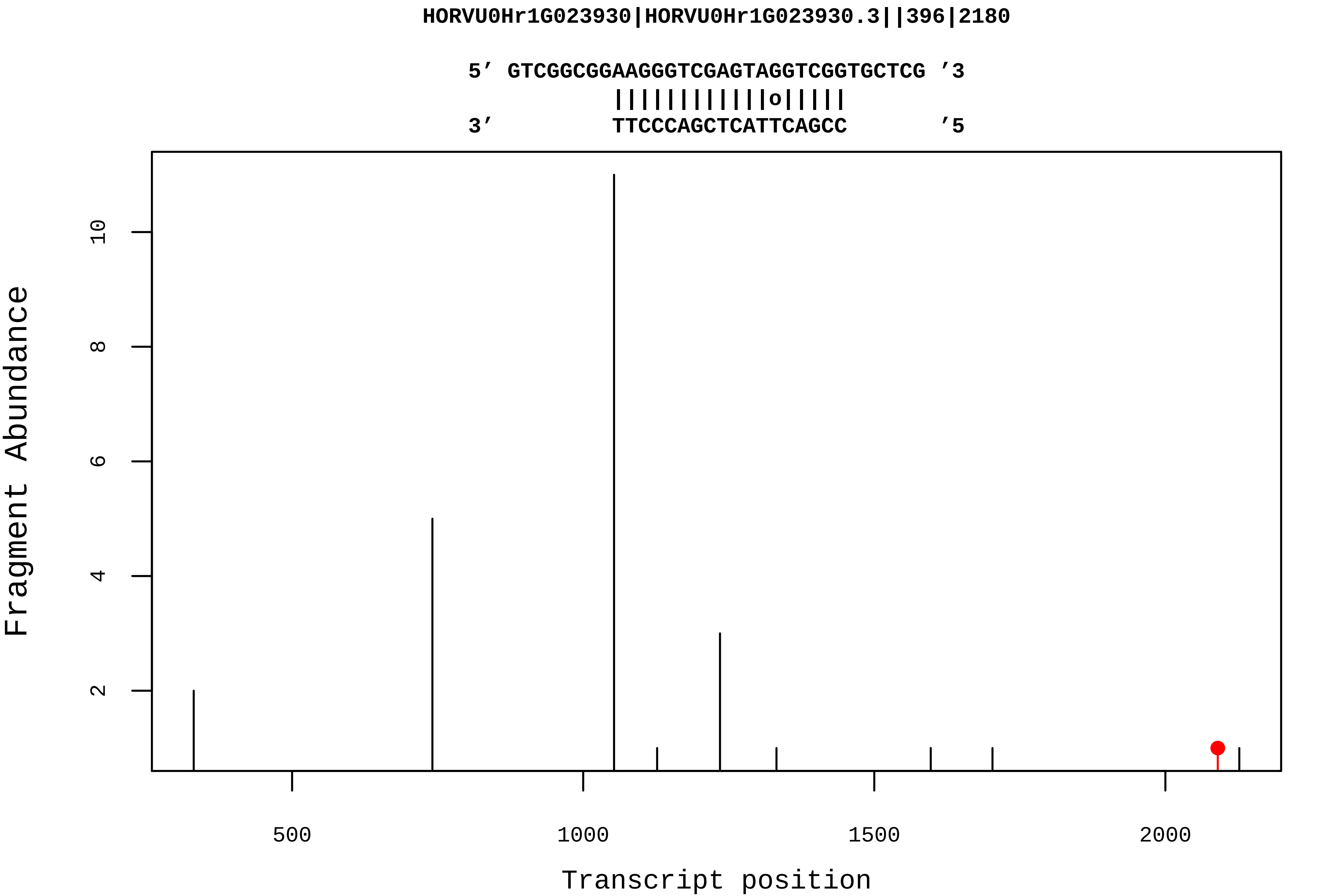

Cleavage site: 2090 Tag abundance: 1 Weighted abundance: 0.143 Category: 4
sRNA abundance: 1 Alignment score: 1 MFE ratio: 0.991 p-value: 0.017

HORVU2Hr1G072960|HORVU2Hr1G072960.1||600|3353

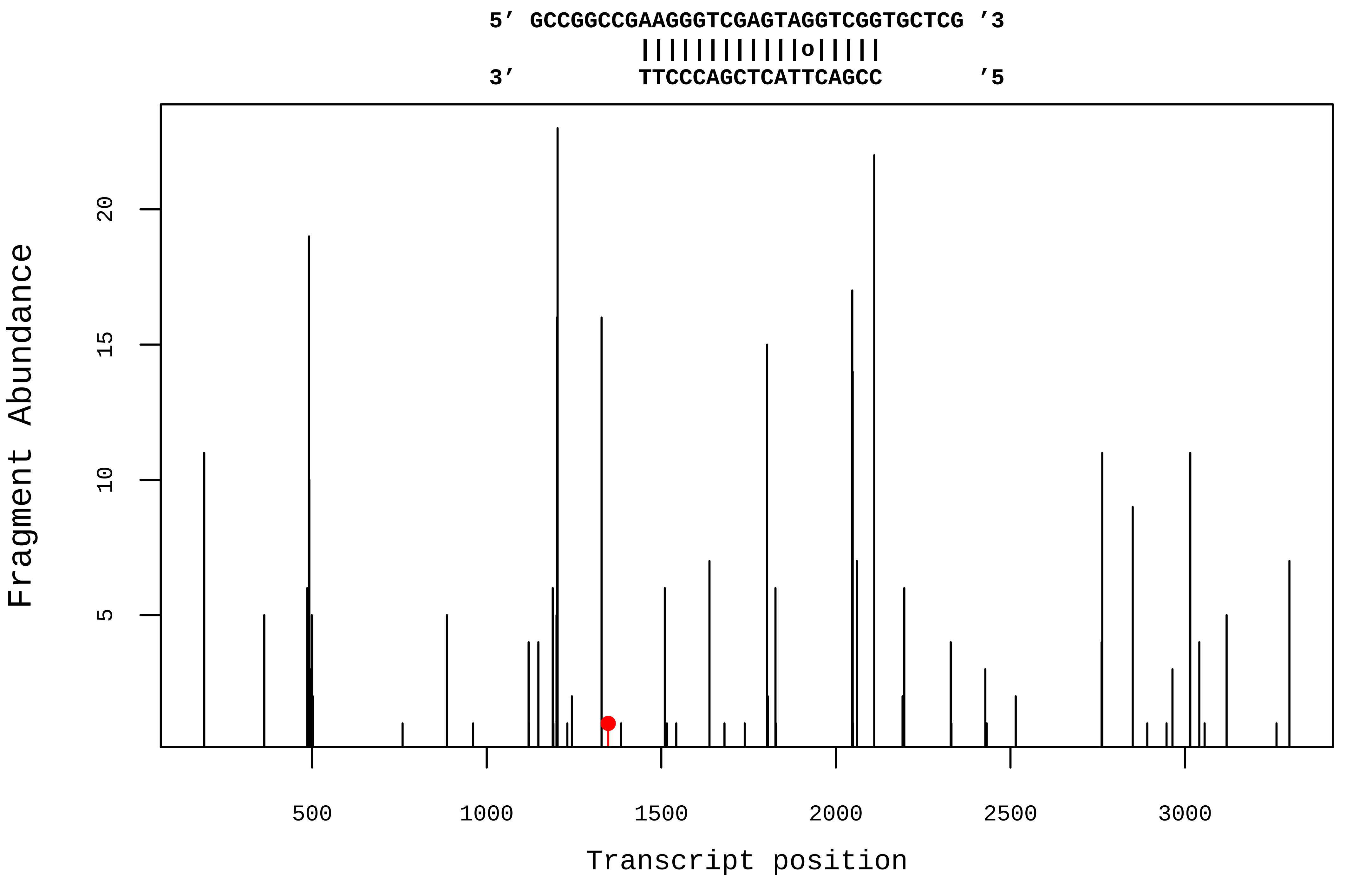

Cleavage site: 1348 Tag abundance: 1 Weighted abundance: 0.143 Category: 4
sRNA abundance: 1 Alignment score: 1 MFE ratio: 0.991 p-value: 0.035

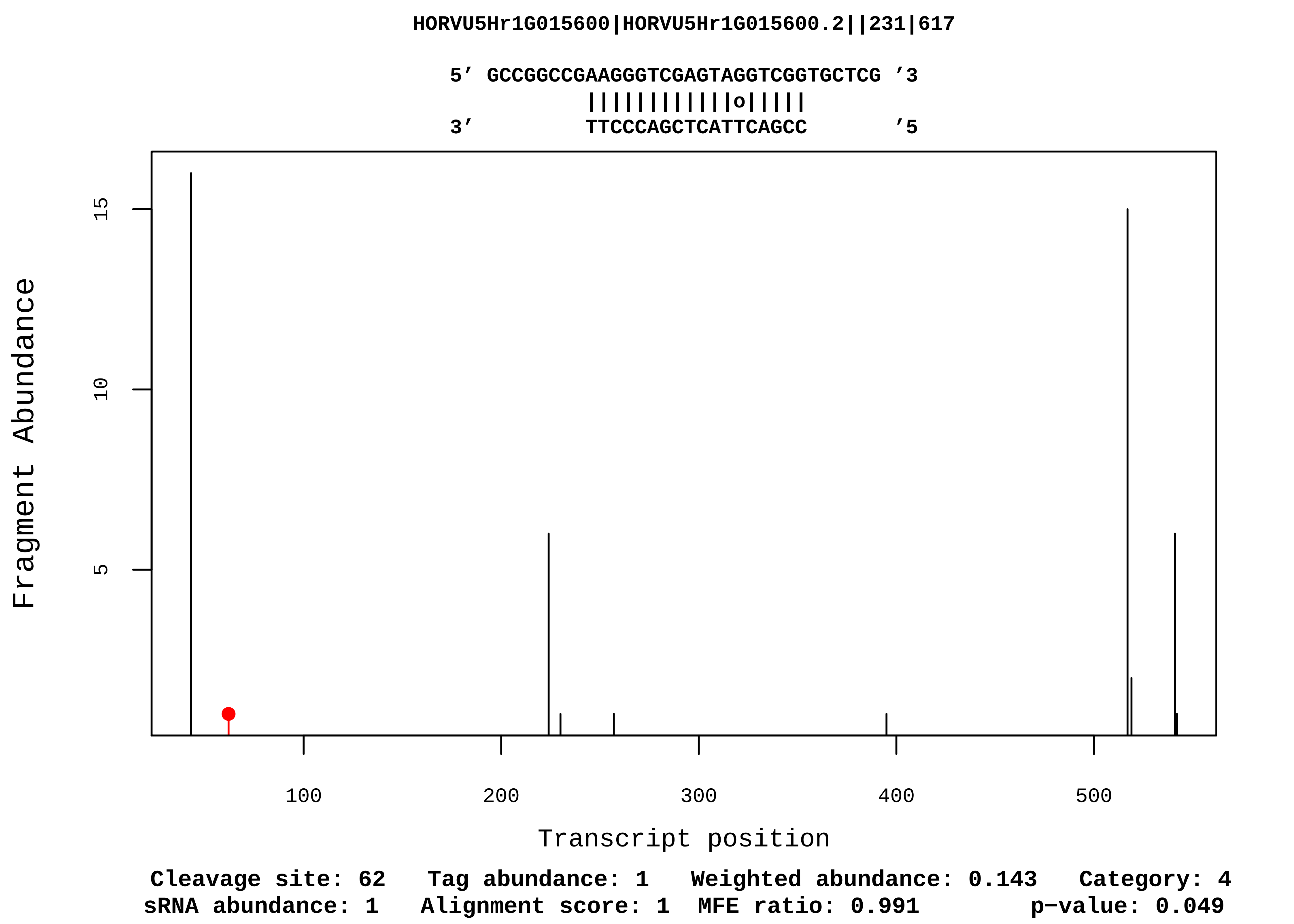
HORVU5Hr1G015600|HORVU5Hr1G015600.2||231|617
5' GCCGGCCGAAGGGTCGAGTAGGTCGGTGCTCG '3
||||||||||||o|||||
3' TTCCCAGCTCATTCAGCC '5
Fragment Abundance
5
10
15
100
200
300
400
500
Transcript position
Cleavage site: 62 Tag abundance: 1 Weighted abundance: 0.143 Category: 4
sRNA abundance: 1 Alignment score: 1 MFE ratio: 0.991 p-value: 0.049

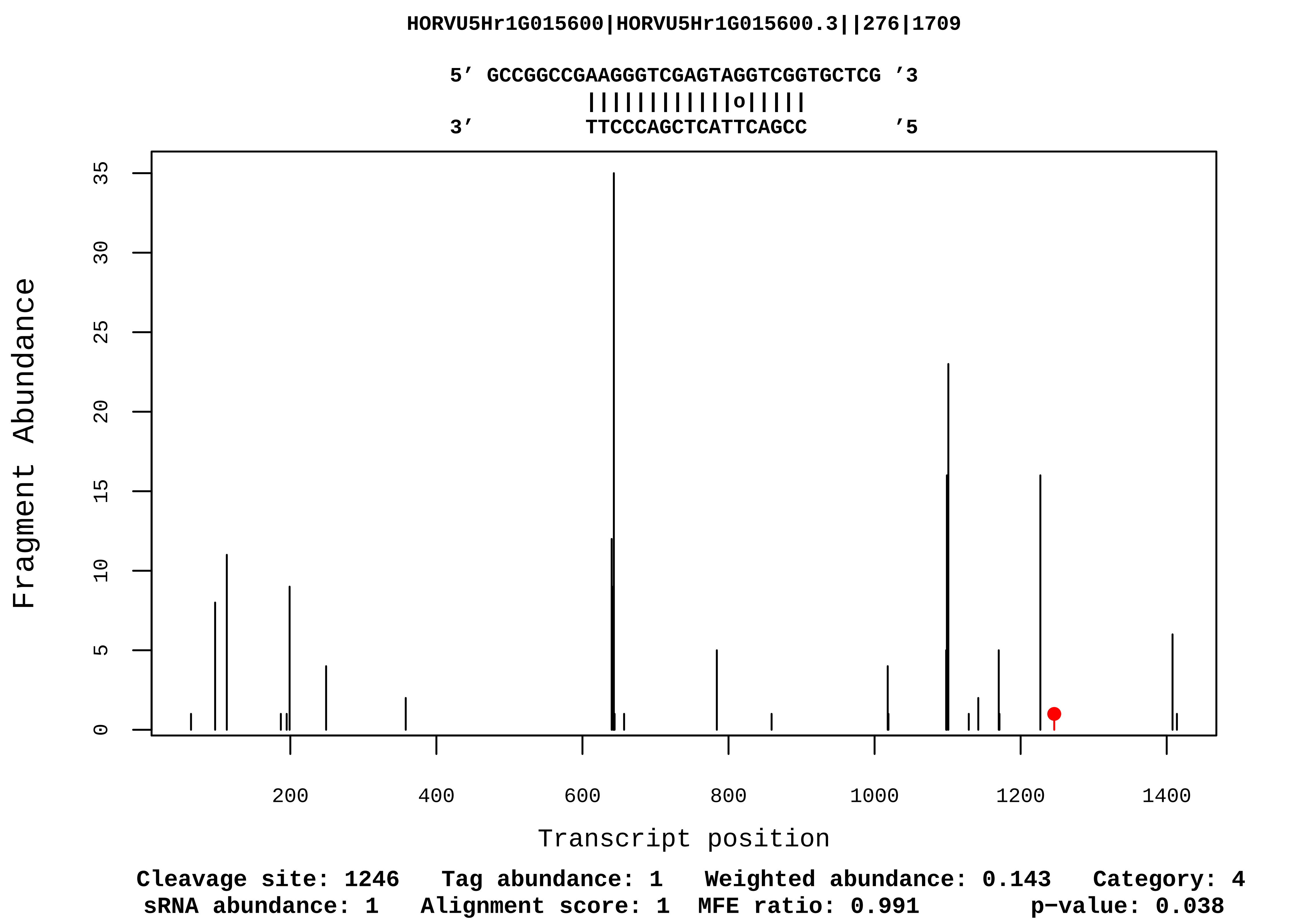

HORVU5Hr1G015600|HORVU5Hr1G015600.3||276|1709
5' GCCGGCCGAAGGGTCGAGTAGGTCGGTGCTCG '3
||||||||||||o|||||
3' TTCCCAGCTCATTCAGCC '5
Fragment Abundance
0
5
10
15
20
25
30
35
200
400
600
800
1000
1200
1400
Transcript position
Cleavage site: 1246 Tag abundance: 1 Weighted abundance: 0.143 Category: 4
sRNA abundance: 1 Alignment score: 1 MFE ratio: 0.991 p-value: 0.038

HORVU5Hr1G015600|HORVU5Hr1G015600.4||207|1852

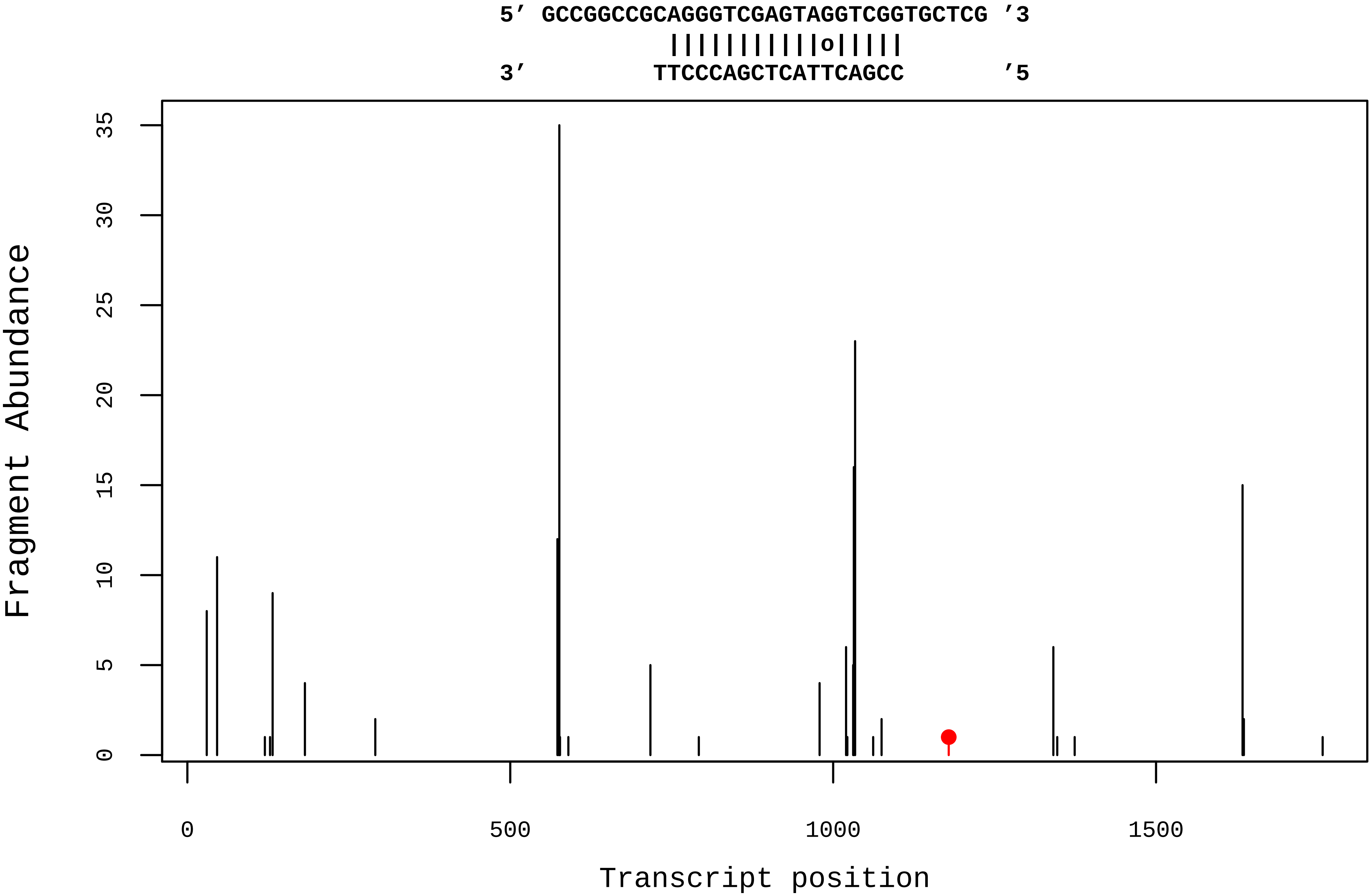

Cleavage site: 1179 Tag abundance: 1 Weighted abundance: 0.143 Category: 4
sRNA abundance: 1 Alignment score: 2 MFE ratio: 0.982 p-value: 0.041

HORVU0Hr1G003660|HORVU0Hr1G003660.1||297|1955

```
5' GCCGGCCGAAGGGTCGAGTAGGTCGGTGCTCG '3
        ||||||||||||||||||||||
3'   ACCGGCTTCCCAGCTCATCCAGCC        '5
```

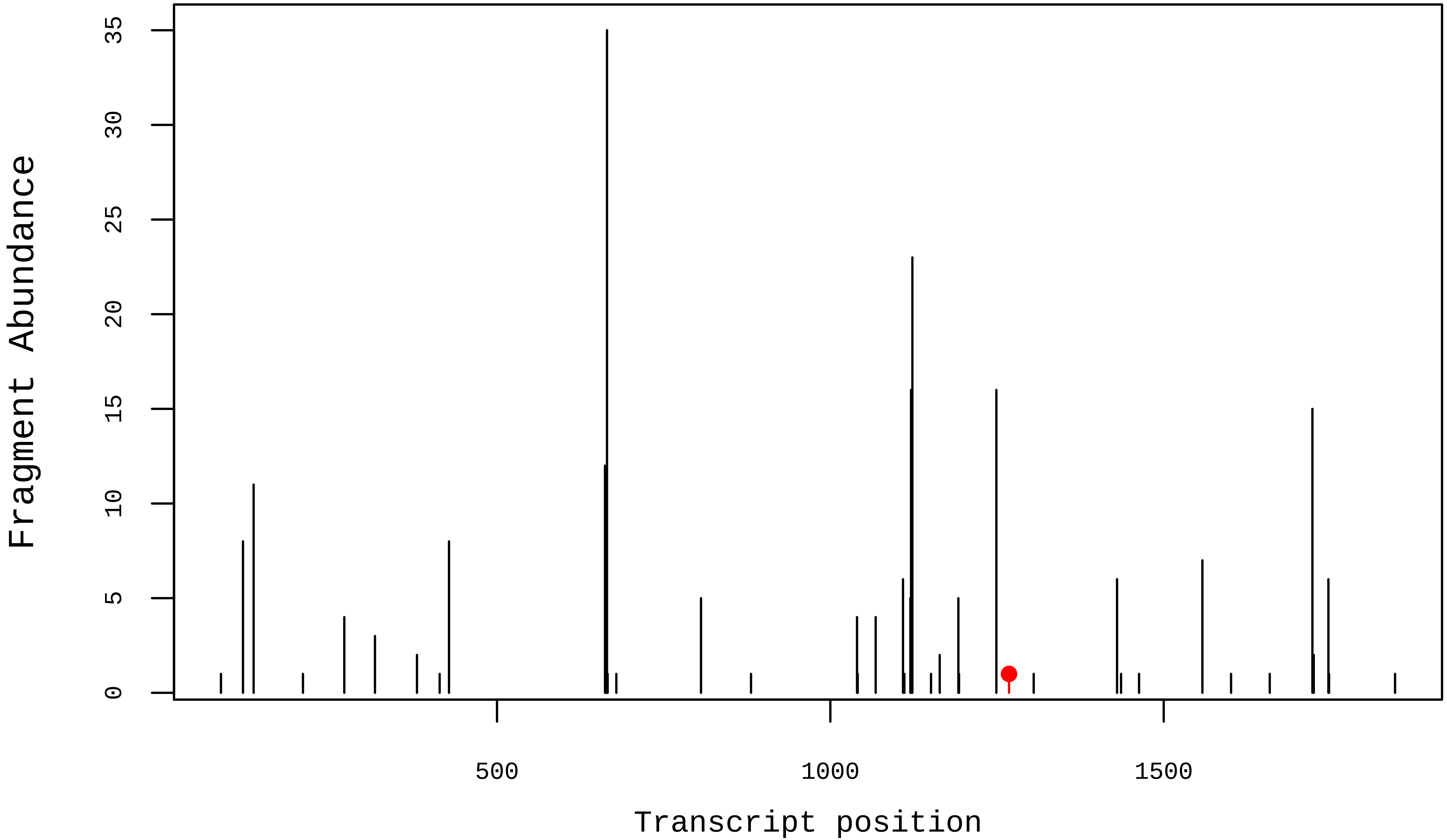

Cleavage site: 1268 Tag abundance: 1 Weighted abundance: 0.143 Category: 4
sRNA abundance: 1 Alignment score: 1 MFE ratio: 0.998 p-value: 0.046

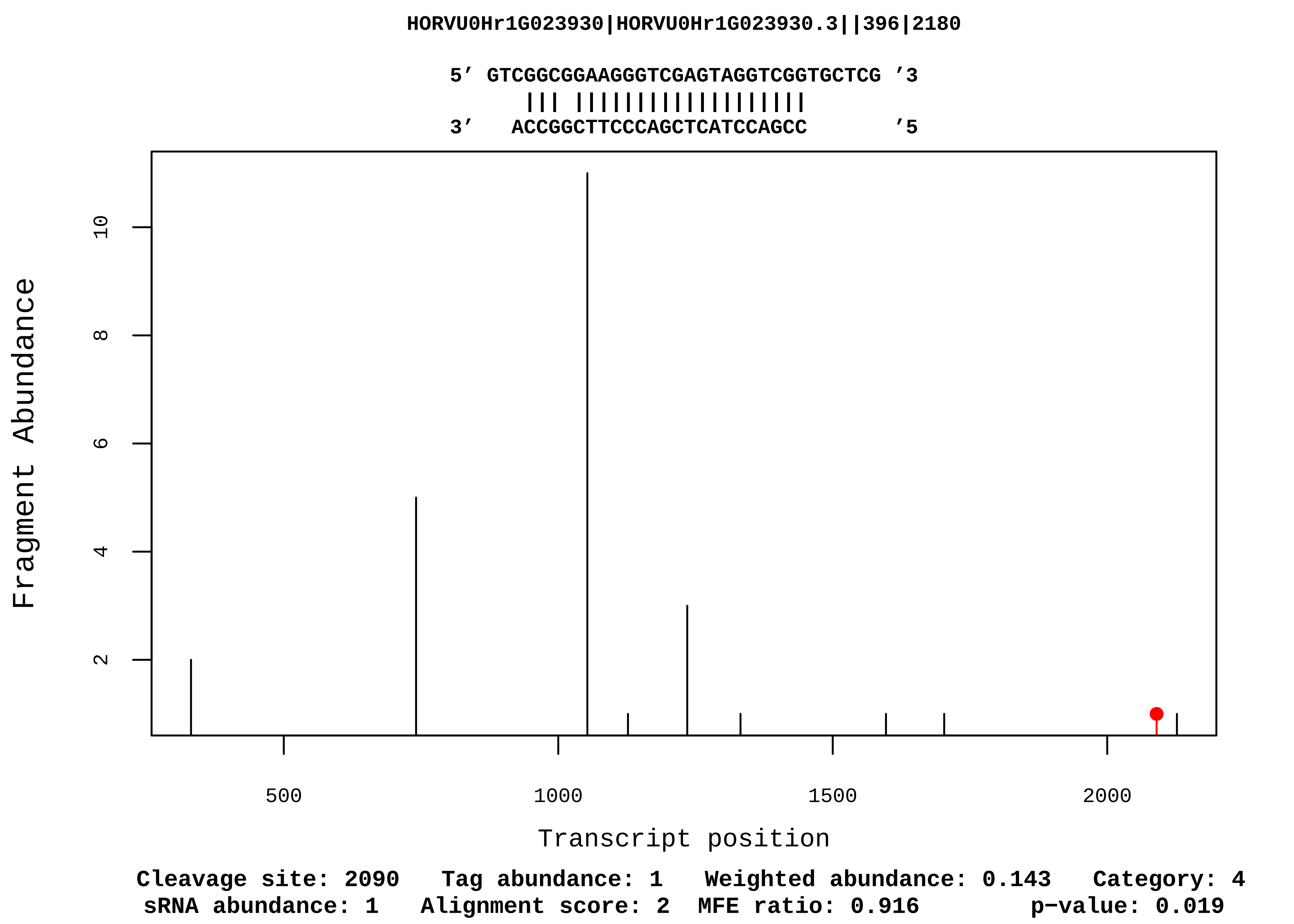

HORVU0Hr1G023930|HORVU0Hr1G023930.3||396|2180
5' GTCGGCGGAAGGGTCGAGTAGGTCGGTGCTCG '3
||| ||||||||||||||||||||
3' ACCGGCTTCCCAGCTCATCCAGCC '5
Fragment Abundance
2
4
6
8
10
500
1000
1500
2000
Transcript position
Cleavage site: 2090 Tag abundance: 1 Weighted abundance: 0.143 Category: 4
sRNA abundance: 1 Alignment score: 2 MFE ratio: 0.916 p-value: 0.019

HORVU2Hr1G072960|HORVU2Hr1G072960.1||600|3353

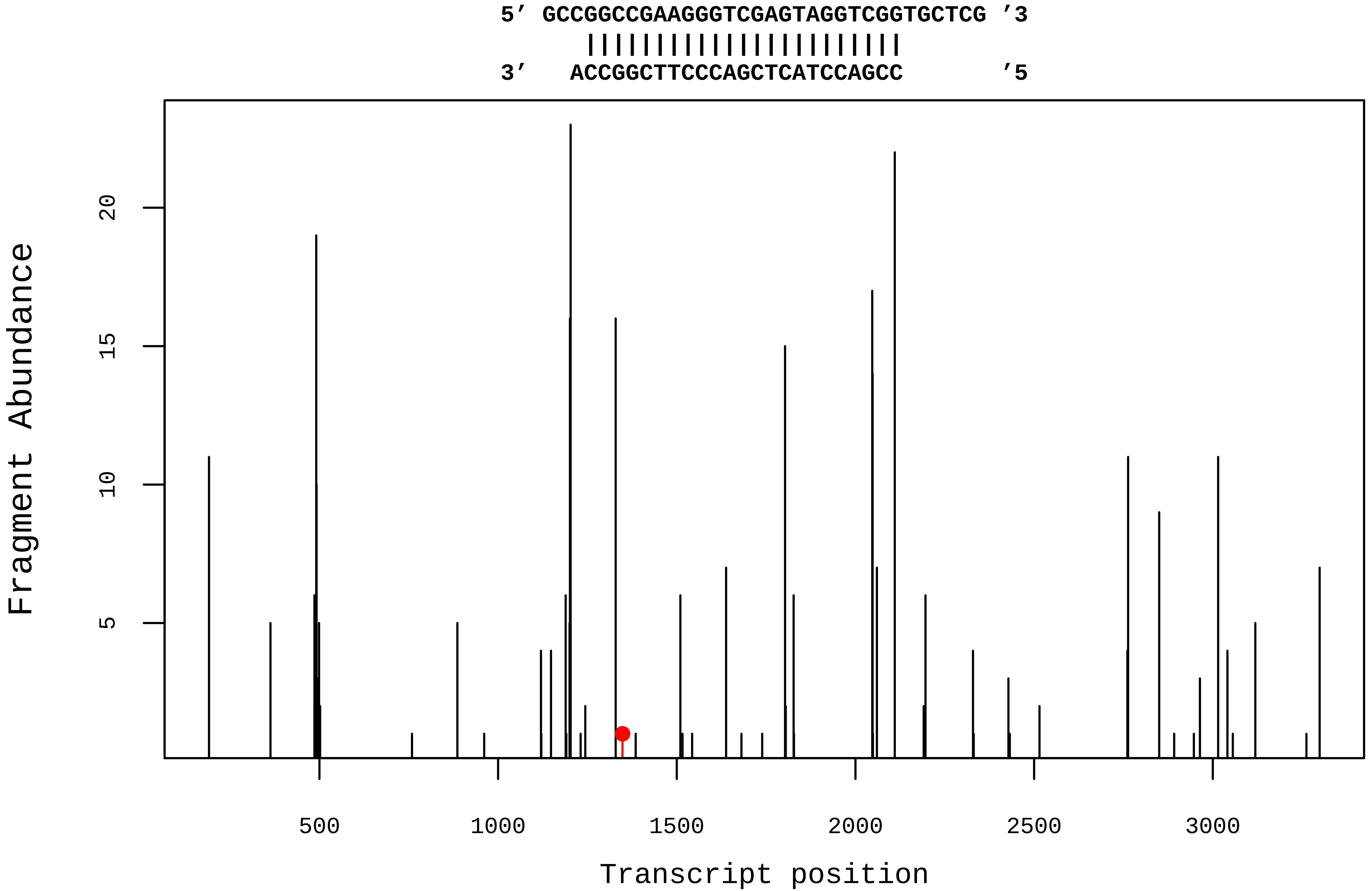

Cleavage site: 1348 Tag abundance: 1 Weighted abundance: 0.143 Category: 4
sRNA abundance: 1 Alignment score: 1 MFE ratio: 0.998 p-value: 0.03

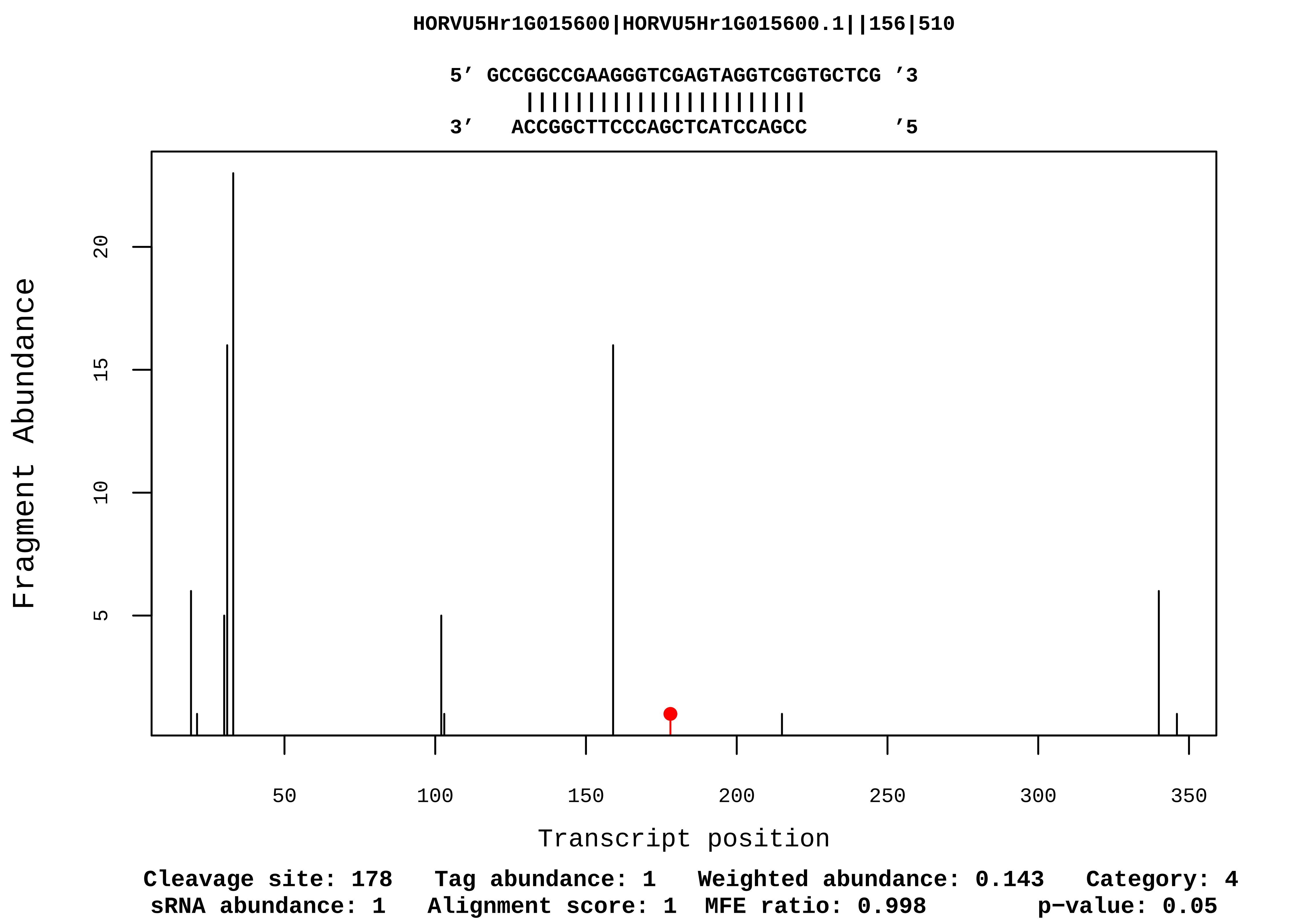

HORVU5Hr1G015600|HORVU5Hr1G015600.1||156|510
5' GCCGGCCGAAGGGTCGAGTAGGTCGGTGCTCG '3
||||||||||||||||||||||||
3' ACCGGCTTCCCAGCTCATCCAGCC '5
Fragment Abundance
5
10
15
20
50
100
150
200
250
300
350
Transcript position
Cleavage site: 178 Tag abundance: 1 Weighted abundance: 0.143 Category: 4
sRNA abundance: 1 Alignment score: 1 MFE ratio: 0.998 p-value: 0.05

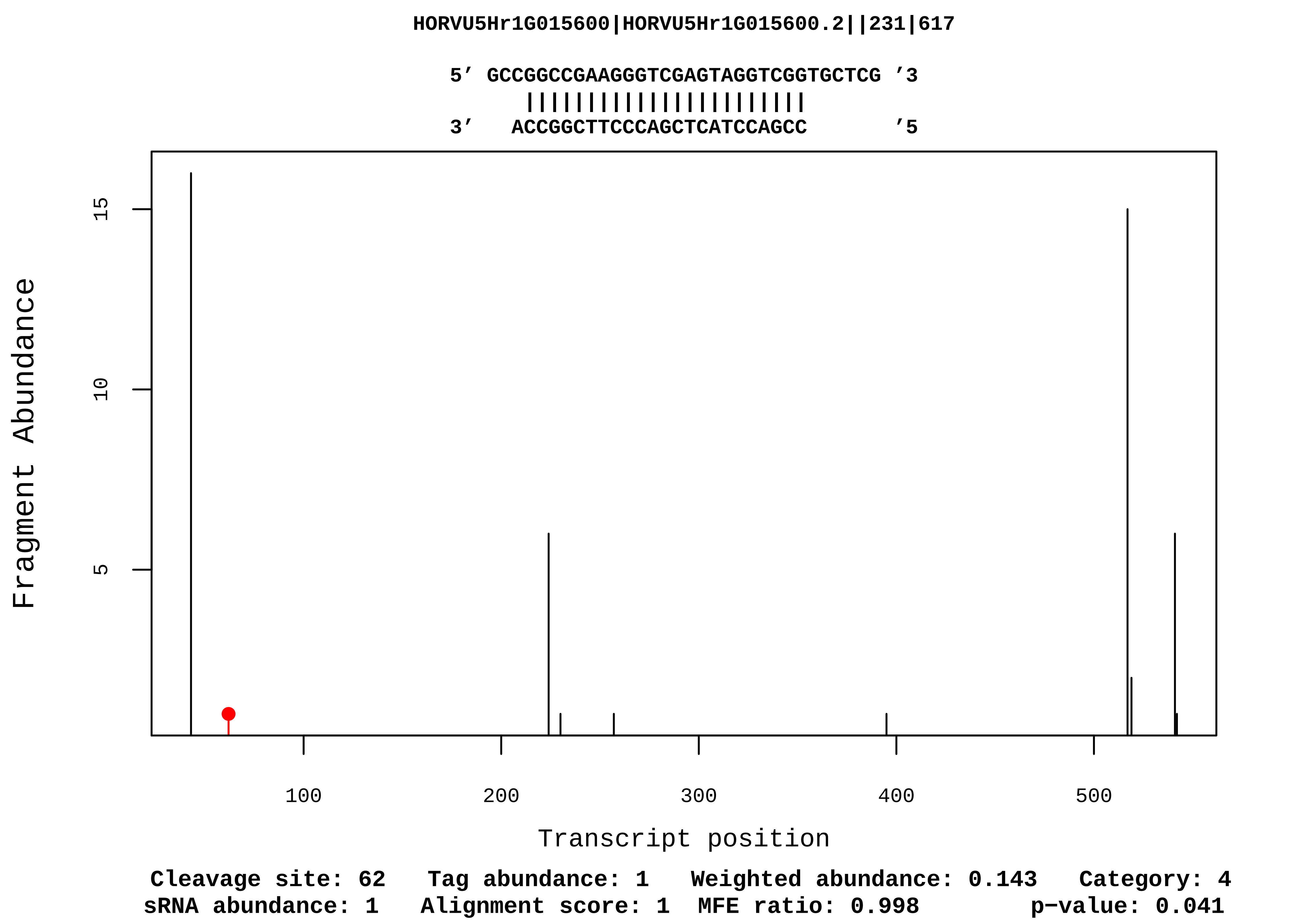
HORVU5Hr1G015600|HORVU5Hr1G015600.2||231|617
5' GCCGGCCGAAGGGTCGAGTAGGTCGGTGCTCG '3
||||||||||||||||||||||||
3' ACCGGCTTCCCAGCTCATCCAGCC '5
Fragment Abundance
5
10
15
100
200
300
400
500
Transcript position
Cleavage site: 62 Tag abundance: 1 Weighted abundance: 0.143 Category: 4
sRNA abundance: 1 Alignment score: 1 MFE ratio: 0.998 p-value: 0.041

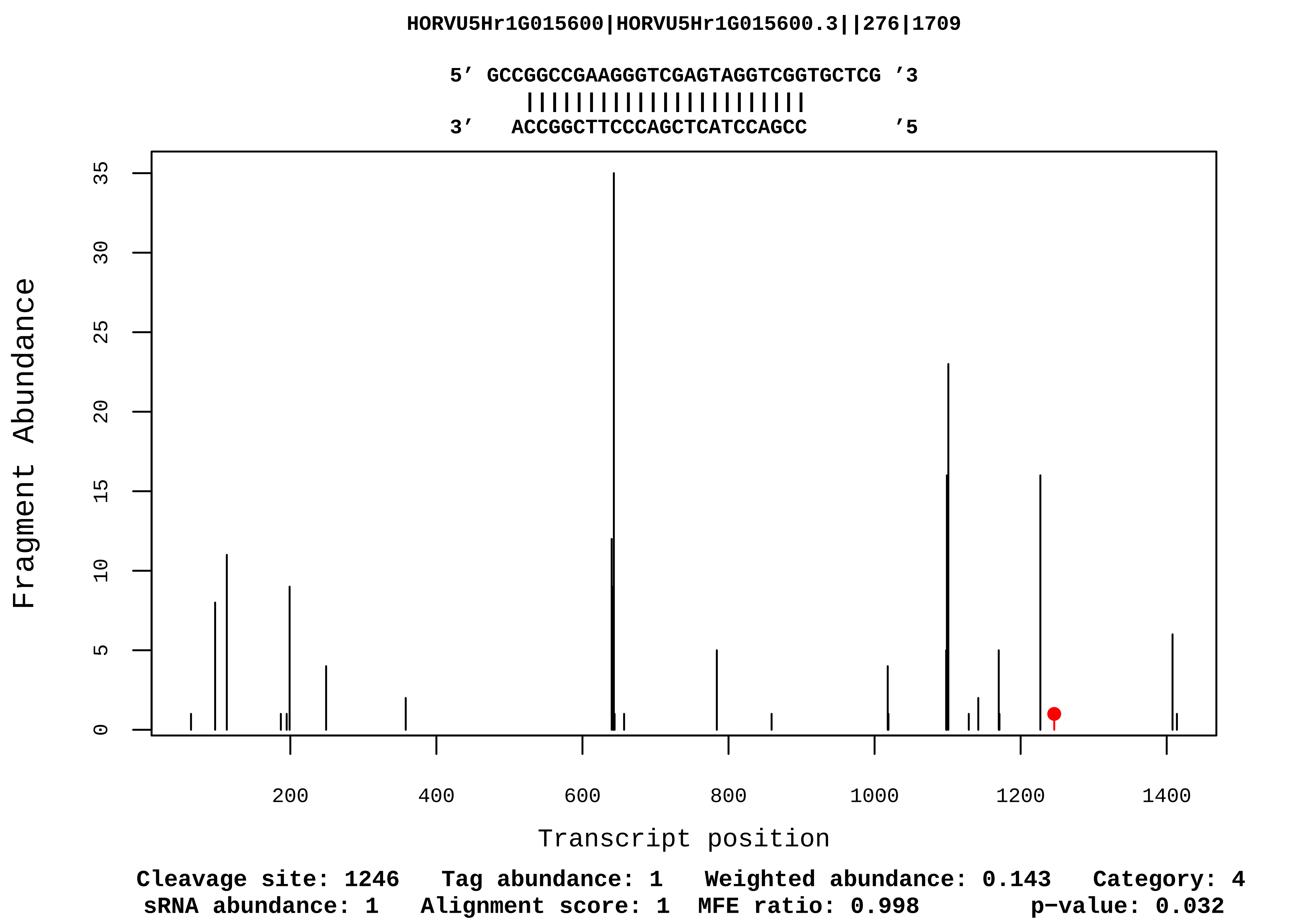
HORVU5Hr1G015600|HORVU5Hr1G015600.3||276|1709
5′ GCCGGCCGAAGGGTCGAGTAGGTCGGTGCTCG ′3
||||||||||||||||||||||
3′ ACCGGCTTCCCAGCTCATCCAGCC ′5
Fragment Abundance
0
5
10
15
20
25
30
35
200
400
600
800
1000
1200
1400
Transcript position
Cleavage site: 1246 Tag abundance: 1 Weighted abundance: 0.143 Category: 4
sRNA abundance: 1 Alignment score: 1 MFE ratio: 0.998 p-value: 0.032

HORVU5Hr1G015600|HORVU5Hr1G015600.4||207|1852

```
5' GCCGGCCGCAGGGTCGAGTAGGTCGGTGCTCG '3
         ||||| ||||||||||||||||
3'   ACCGGCTTCCCAGCTCATCCAGCC       '5
```

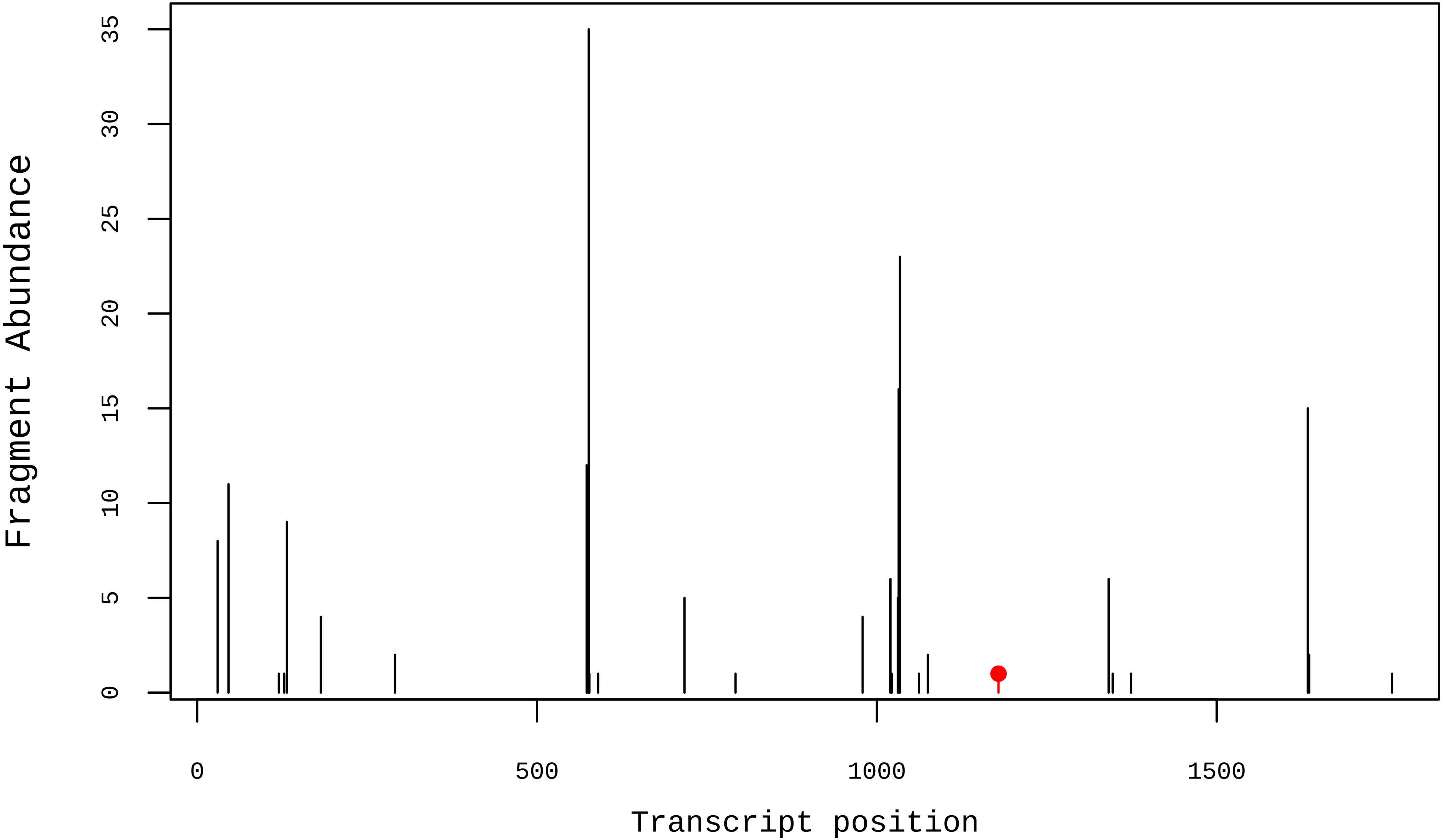

Cleavage site: 1179   Tag abundance: 1   Weighted abundance: 0.143   Category: 4
sRNA abundance: 1   Alignment score: 2   MFE ratio: 0.913   p-value: 0.041

HORVU2Hr1G126440|HORVU2Hr1G126440.3||2823|2994

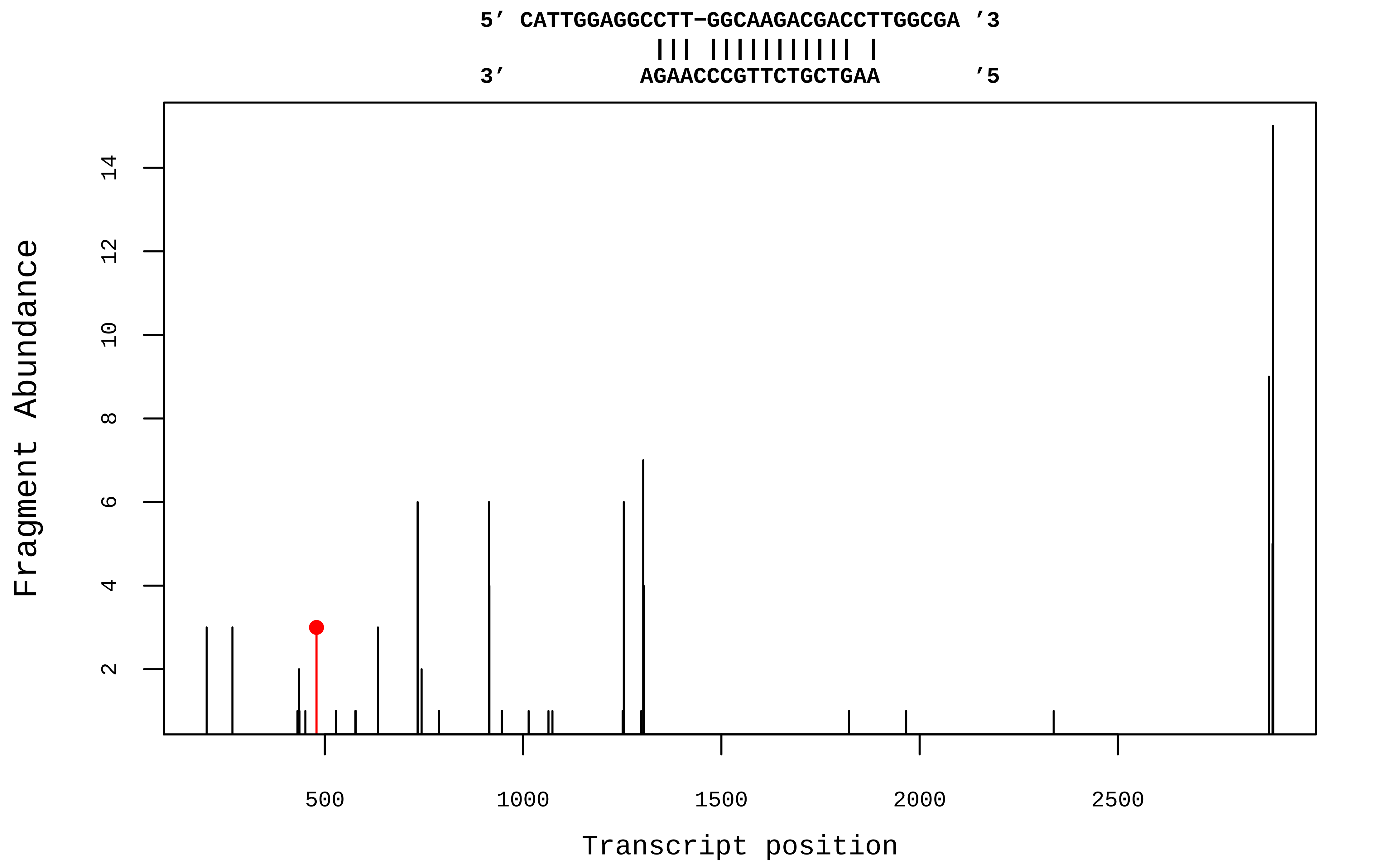

Cleavage site: 479 Tag abundance: 3 Weighted abundance: 0.375 Category: 3
sRNA abundance: 1 Alignment score: 4 MFE ratio: 0.735 p-value: 0.033

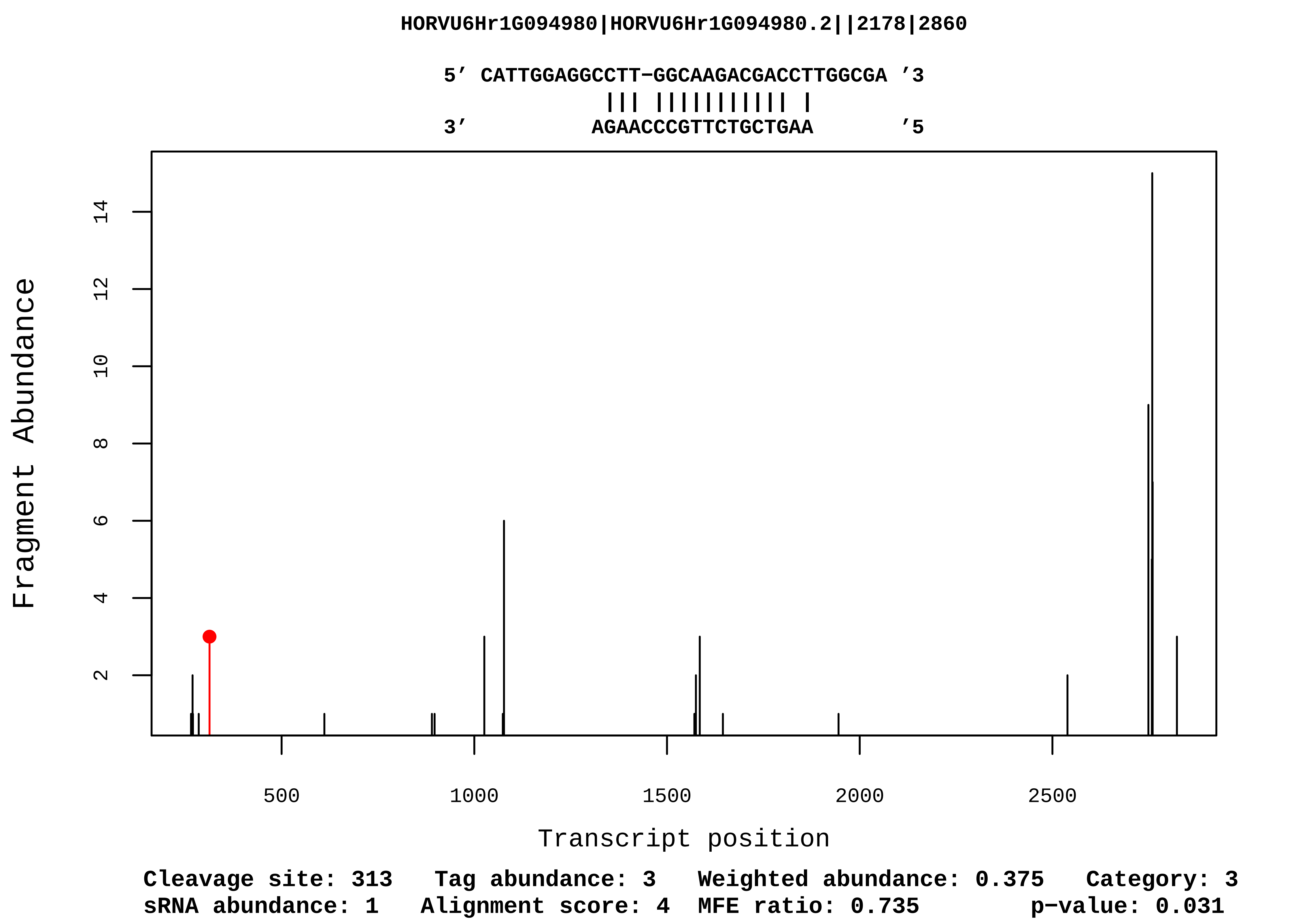
HORVU6Hr1G094980|HORVU6Hr1G094980.2||2178|2860
5' CATTGGAGGCCTT-GGCAAGACGACCTTGGCGA '3
||| ||||||||||| |
3' AGAACCCGTTCTGCTGAA '5
Fragment Abundance
2
4
6
8
10
12
14
500
1000
1500
2000
2500
Transcript position
Cleavage site: 313 Tag abundance: 3 Weighted abundance: 0.375 Category: 3
sRNA abundance: 1 Alignment score: 4 MFE ratio: 0.735 p-value: 0.031

HORVU0Hr1G003660|HORVU0Hr1G003660.1||297|1955

```
5' GCCGGCCGAAGGGTCGAGTAGGTCGGTGCTCG '3
      ||||||||||||||||||||||||
3'    GCCGGCTTCCCAGCTCATCCAGCG       '5
```

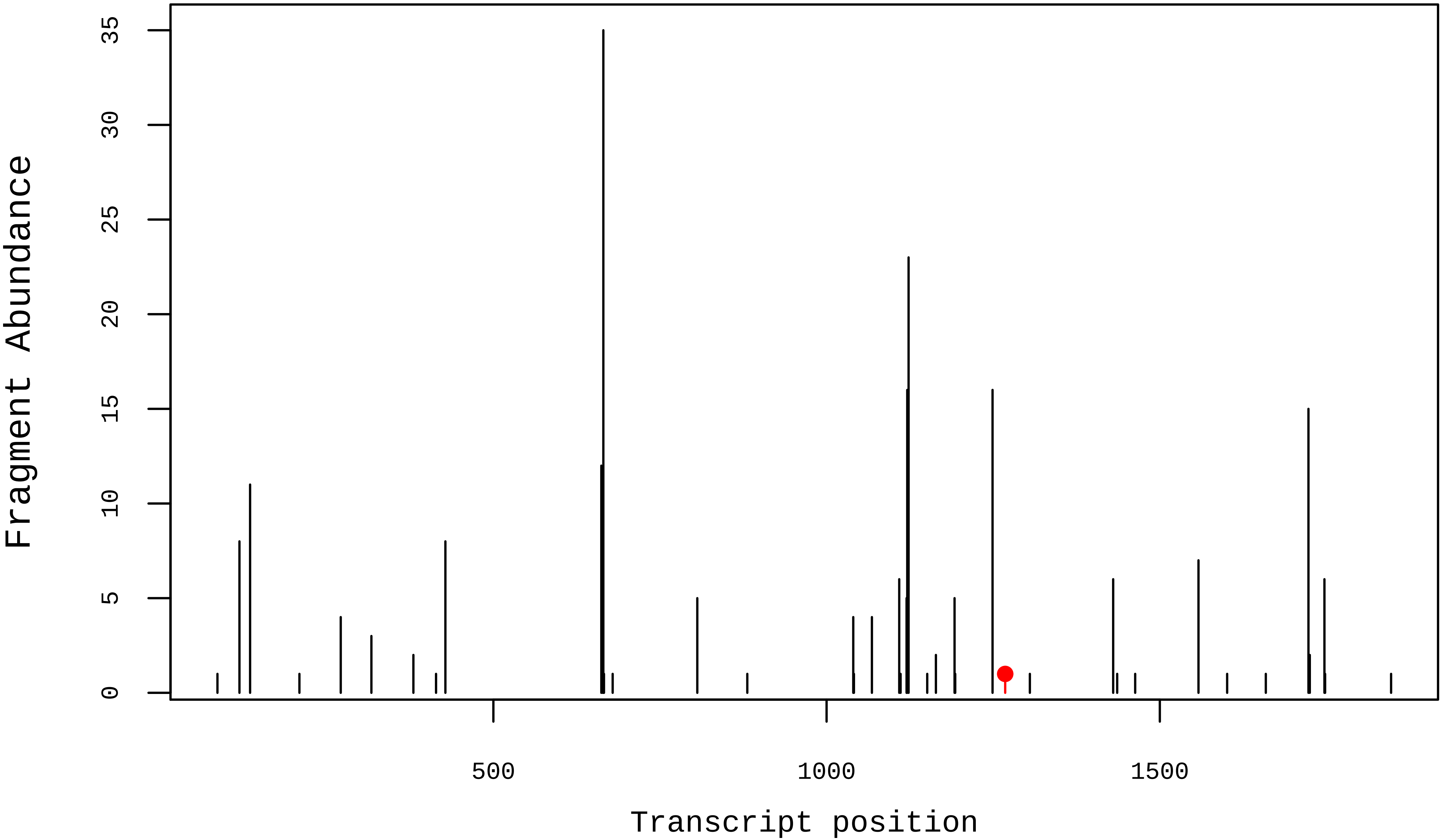

Cleavage site: 1268   Tag abundance: 1   Weighted abundance: 0.143   Category: 4
sRNA abundance: 1   Alignment score: 1   MFE ratio: 0.963   p-value: 0.046

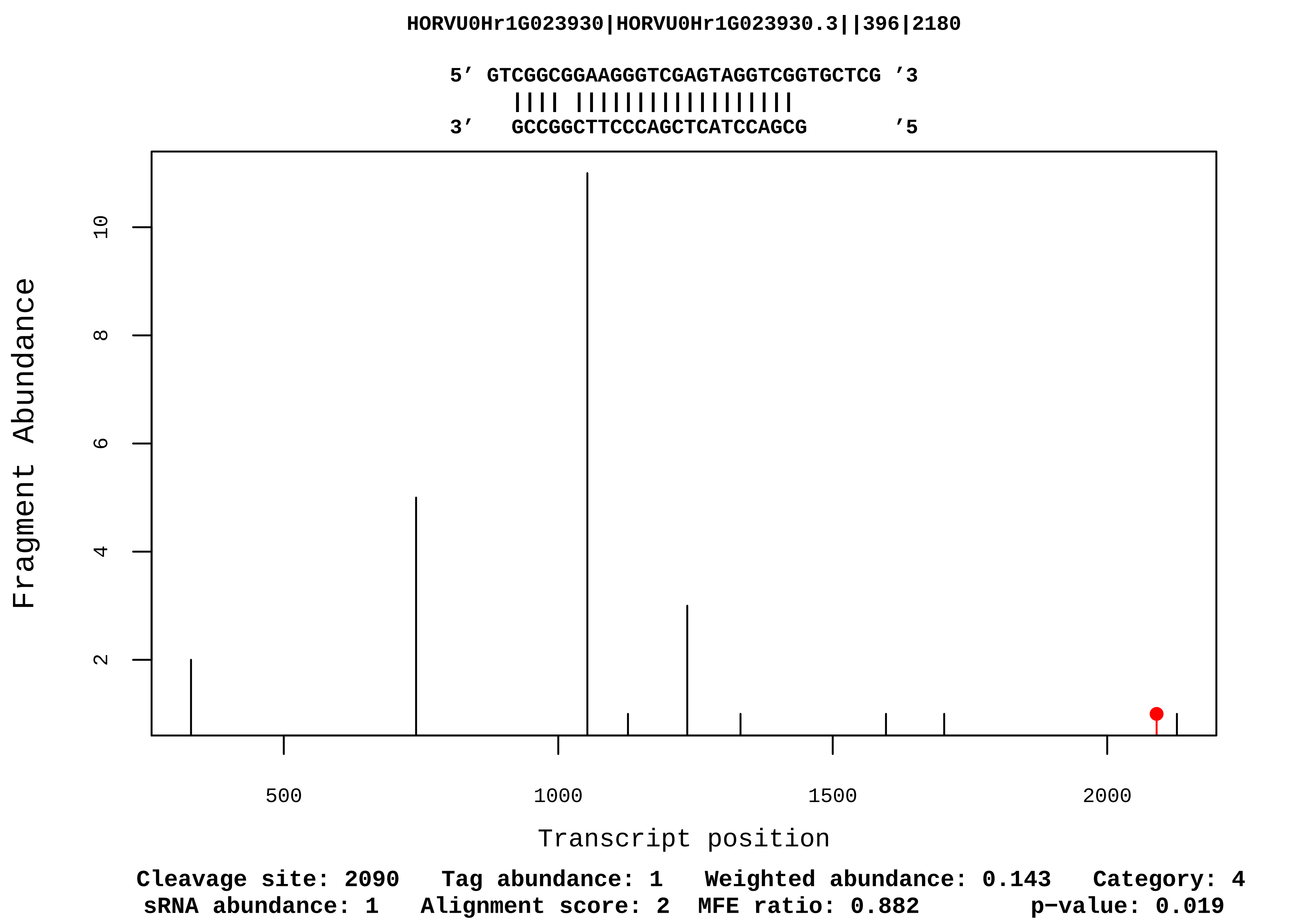

HORVU0Hr1G023930|HORVU0Hr1G023930.3||396|2180
5' GTCGGCGGAAGGGTCGAGTAGGTCGGTGCTCG '3
3' GCCGGCTTCCCAGCTCATCCAGCG '5
Fragment Abundance
2
4
6
8
10
500
1000
1500
2000
Transcript position
Cleavage site: 2090 Tag abundance: 1 Weighted abundance: 0.143 Category: 4
sRNA abundance: 1 Alignment score: 2 MFE ratio: 0.882 p-value: 0.019

HORVU2Hr1G072960|HORVU2Hr1G072960.1||600|3353

```
5' GCCGGCCGAAGGGTCGAGTAGGTCGGTGCTCG '3
      ||||||||||||||||||||||
3'    GCCGGCTTCCCAGCTCATCCAGCG       '5
```

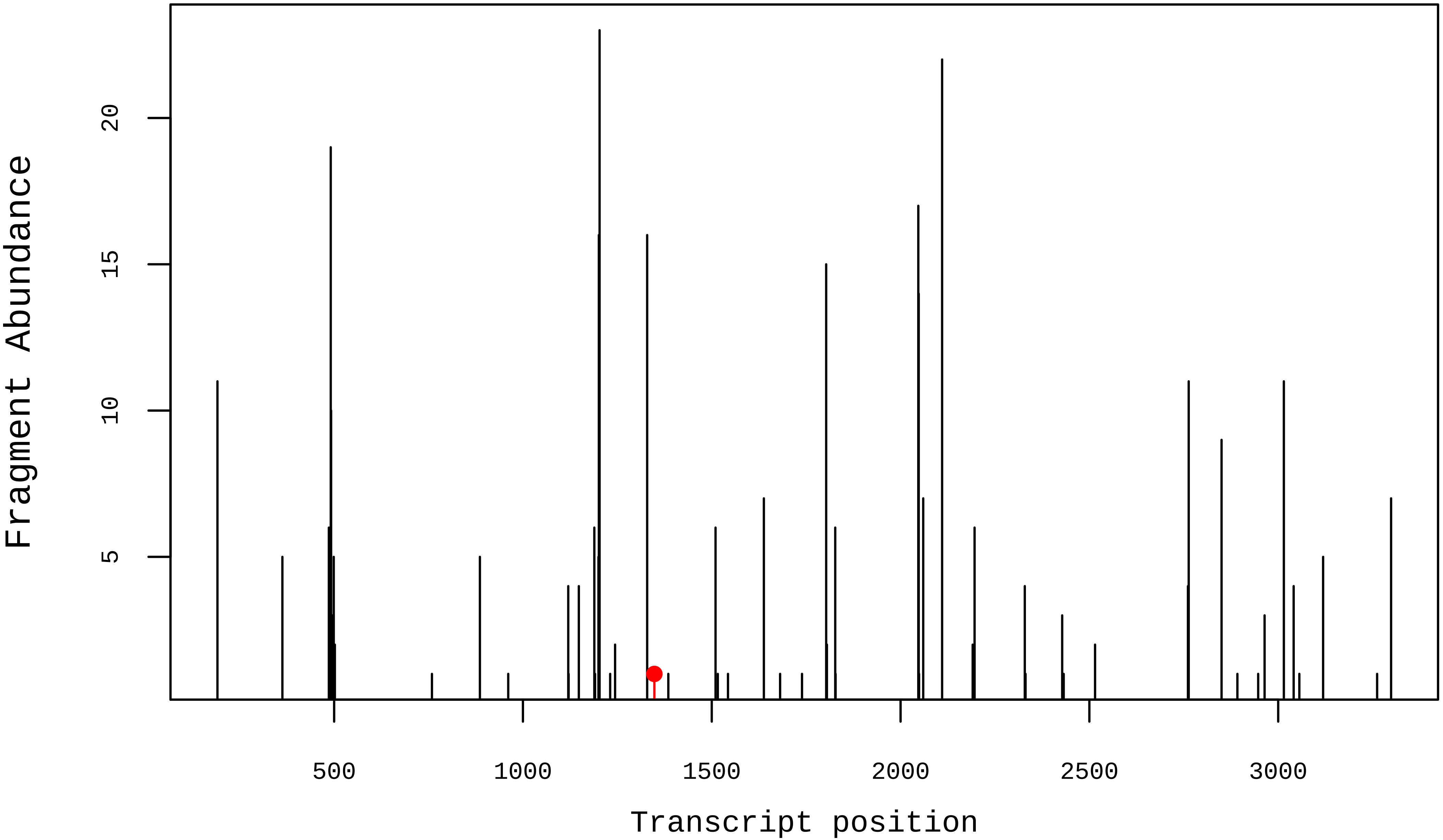

Cleavage site: 1348 Tag abundance: 1 Weighted abundance: 0.143 Category: 4
sRNA abundance: 1 Alignment score: 1 MFE ratio: 0.963 p-value: 0.03

HORVU5Hr1G015600|HORVU5Hr1G015600.1||156|510

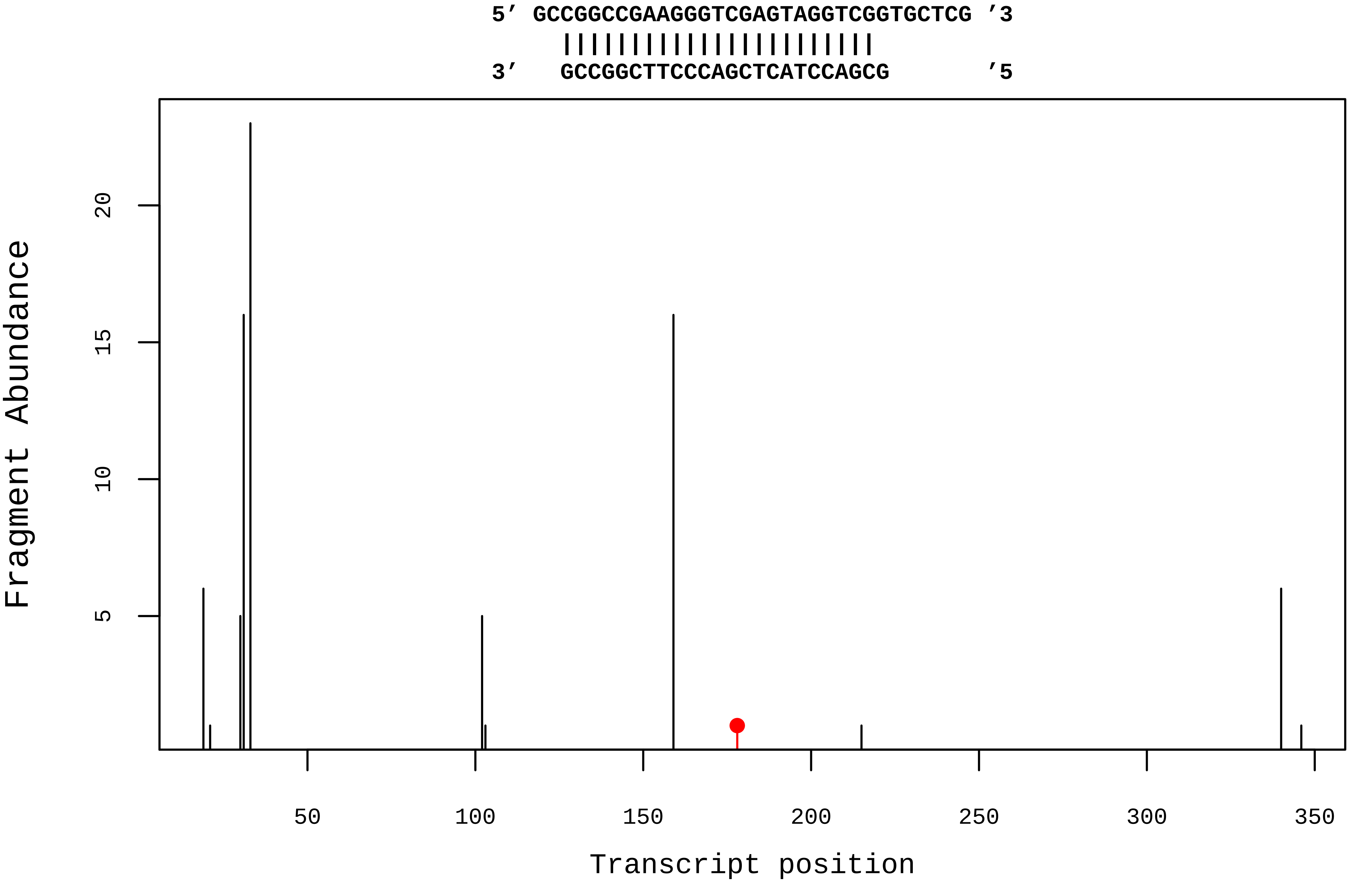

Cleavage site: 178   Tag abundance: 1   Weighted abundance: 0.143   Category: 4
sRNA abundance: 1   Alignment score: 1   MFE ratio: 0.963   p-value: 0.05

HORVU5Hr1G015600|HORVU5Hr1G015600.2||231|617

```
5' GCCGGCCGAAGGGTCGAGTAGGTCGGTGCTCG '3
      ||||||||||||||||||||||
3'    GCCGGCTTCCCAGCTCATCCAGCG        '5
```

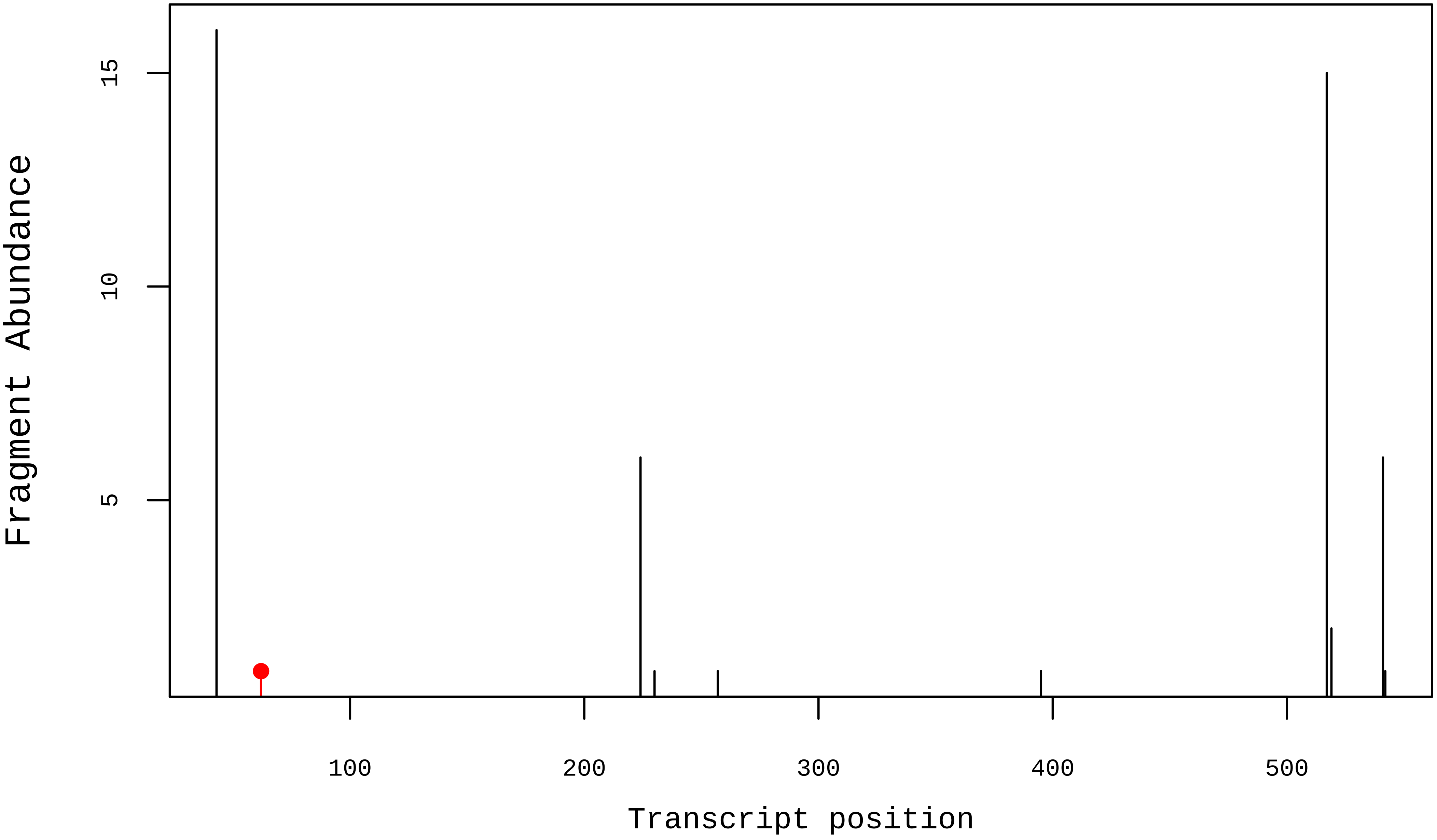

Cleavage site: 62   Tag abundance: 1   Weighted abundance: 0.143   Category: 4
sRNA abundance: 1   Alignment score: 1   MFE ratio: 0.963   p-value: 0.041

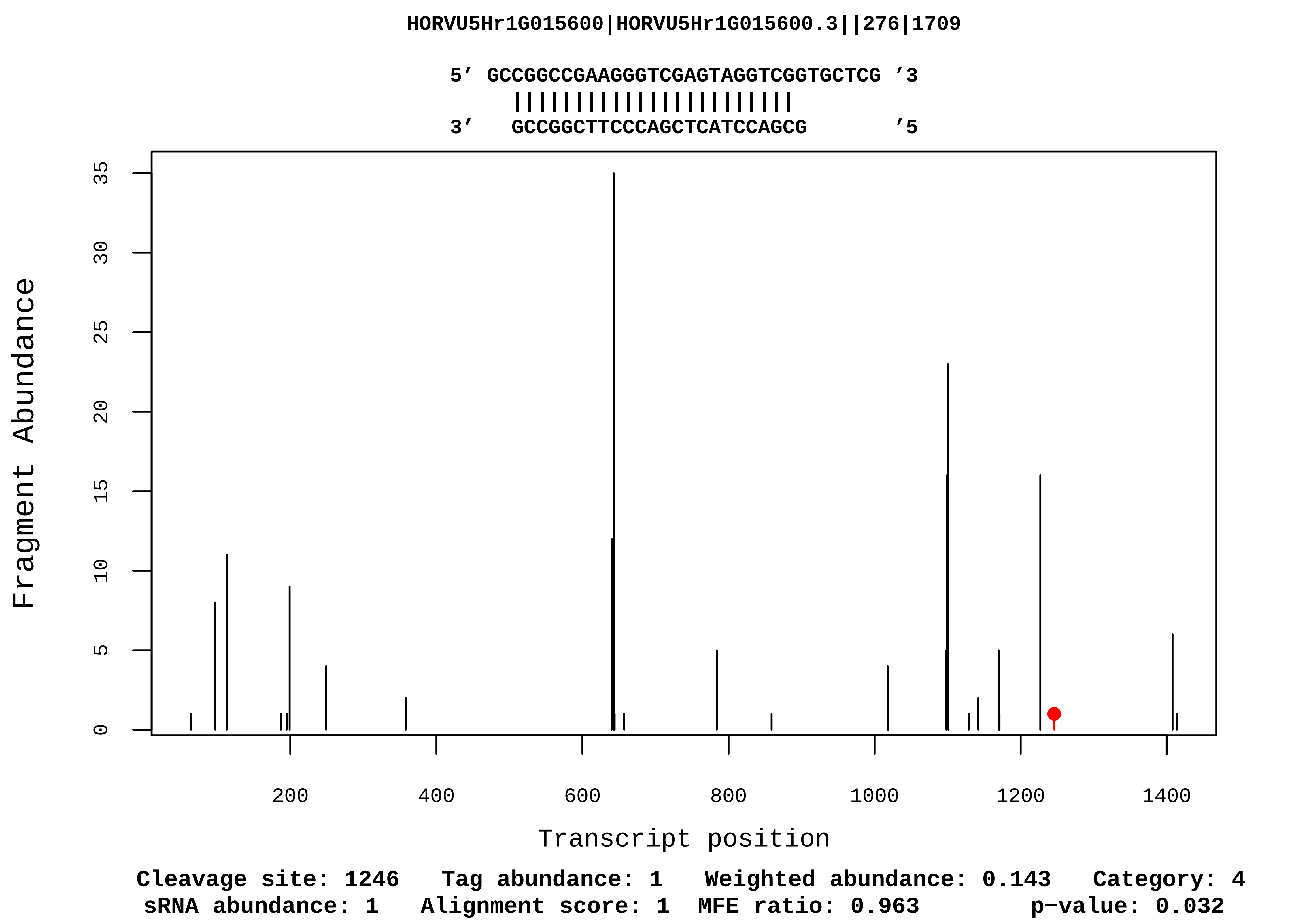

HORVU5Hr1G015600|HORVU5Hr1G015600.3||276|1709
5' GCCGGCCGAAGGGTCGAGTAGGTCGGTGCTCG '3
||||||||||||||||||||||
3' GCCGGCTTCCCAGCTCATCCAGCG '5
Fragment Abundance
0
5
10
15
20
25
30
35
200
400
600
800
1000
1200
1400
Transcript position
Cleavage site: 1246 Tag abundance: 1 Weighted abundance: 0.143 Category: 4
sRNA abundance: 1 Alignment score: 1 MFE ratio: 0.963 p-value: 0.032

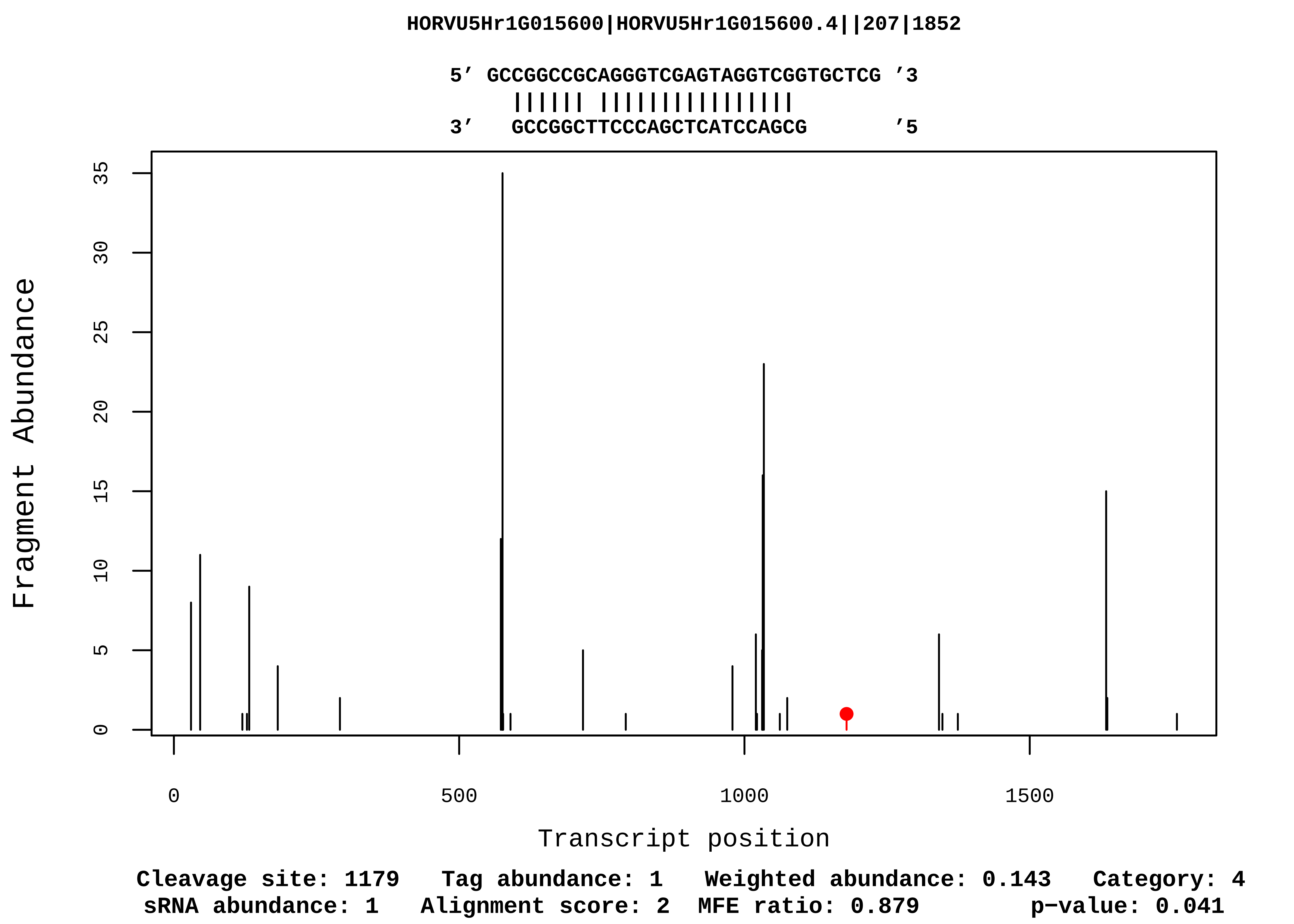
HORVU5Hr1G015600|HORVU5Hr1G015600.4||207|1852
5' GCCGGCCGCAGGGTCGAGTAGGTCGGTGCTCG '3
3' GCCGGCTTCCCAGCTCATCCAGCG '5
Fragment Abundance
0
5
10
15
20
25
30
35
0
500
1000
1500
Transcript position
Cleavage site: 1179 Tag abundance: 1 Weighted abundance: 0.143 Category: 4
sRNA abundance: 1 Alignment score: 2 MFE ratio: 0.879 p-value: 0.041

HORVU7Hr1G024570|HORVU7Hr1G024570.1||432|1574

```
5' GCCCGAGCCCACC-CGCTACGGCGACTGGGAGC '3
              ||||||  |||||||||o||
3'            CGGGTGTCGCGATGCCGTTGC       '5
```

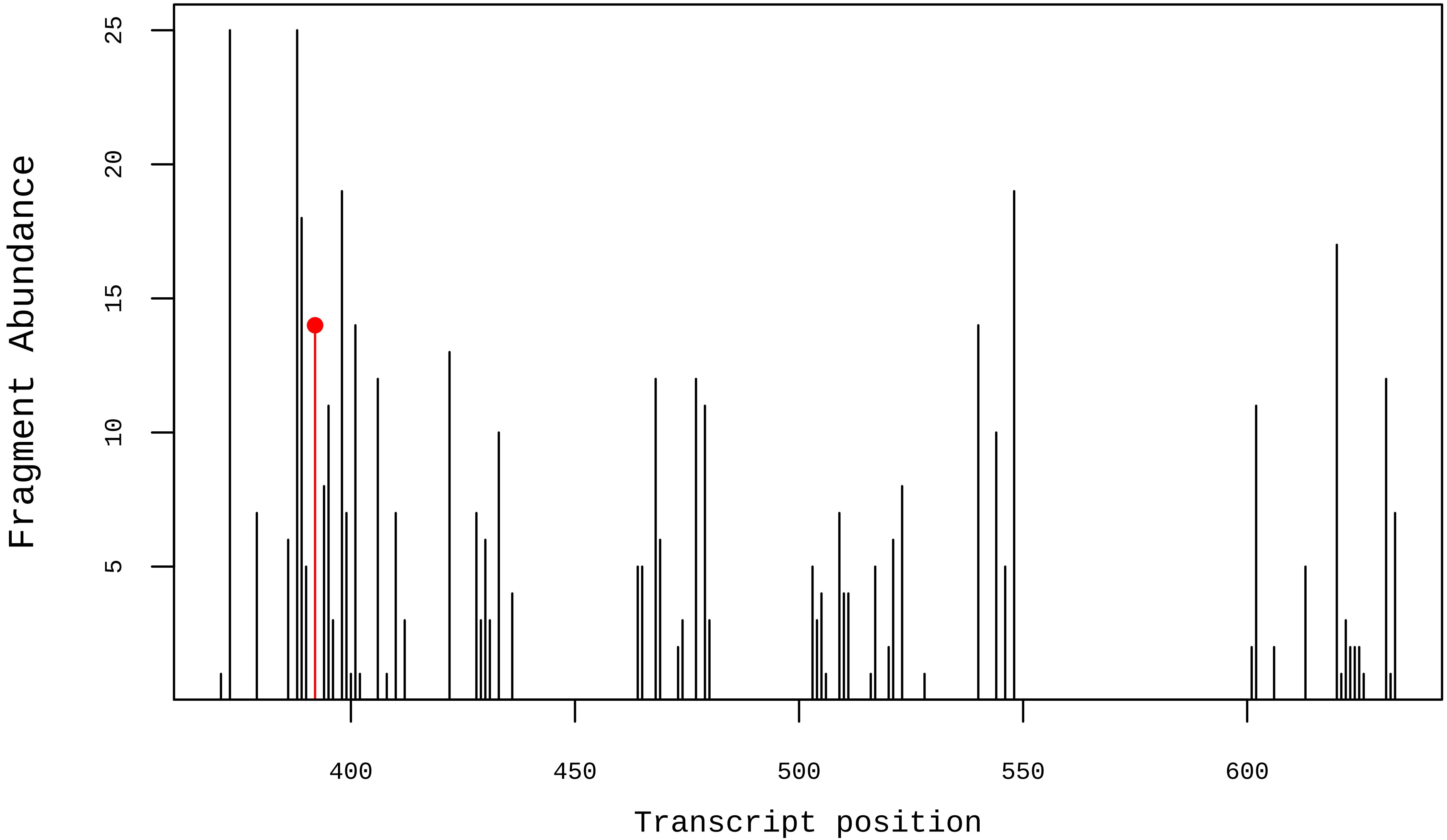

Cleavage site: 392   Tag abundance: 14   Weighted abundance: 4.667   Category: 2
sRNA abundance: 1   Alignment score: 4   MFE ratio: 0.78   p-value: 0.036

HORVU7Hr1G024570|HORVU7Hr1G024570.2||432|4386

```
5' GCCCGAGCCCACC-CGCTACGGCGACTGGGAGC '3
         ||||||  |||||||||o||
3'       CGGGTGTCGCGATGCCGTTGC       '5
```

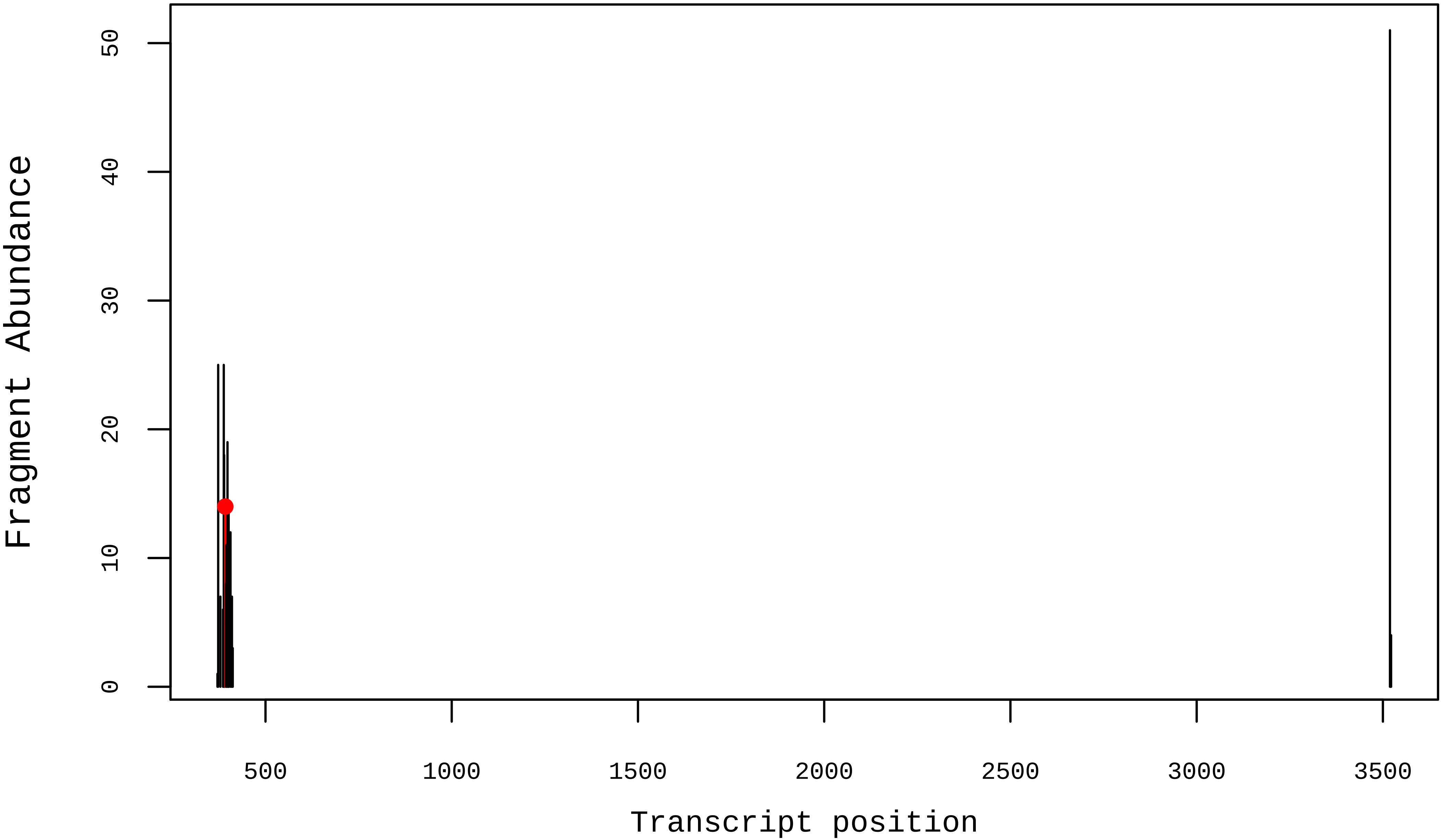

Cleavage site: 392   Tag abundance: 14   Weighted abundance: 4.667   Category: 2
sRNA abundance: 1   Alignment score: 4   MFE ratio: 0.78   p-value: 0.004

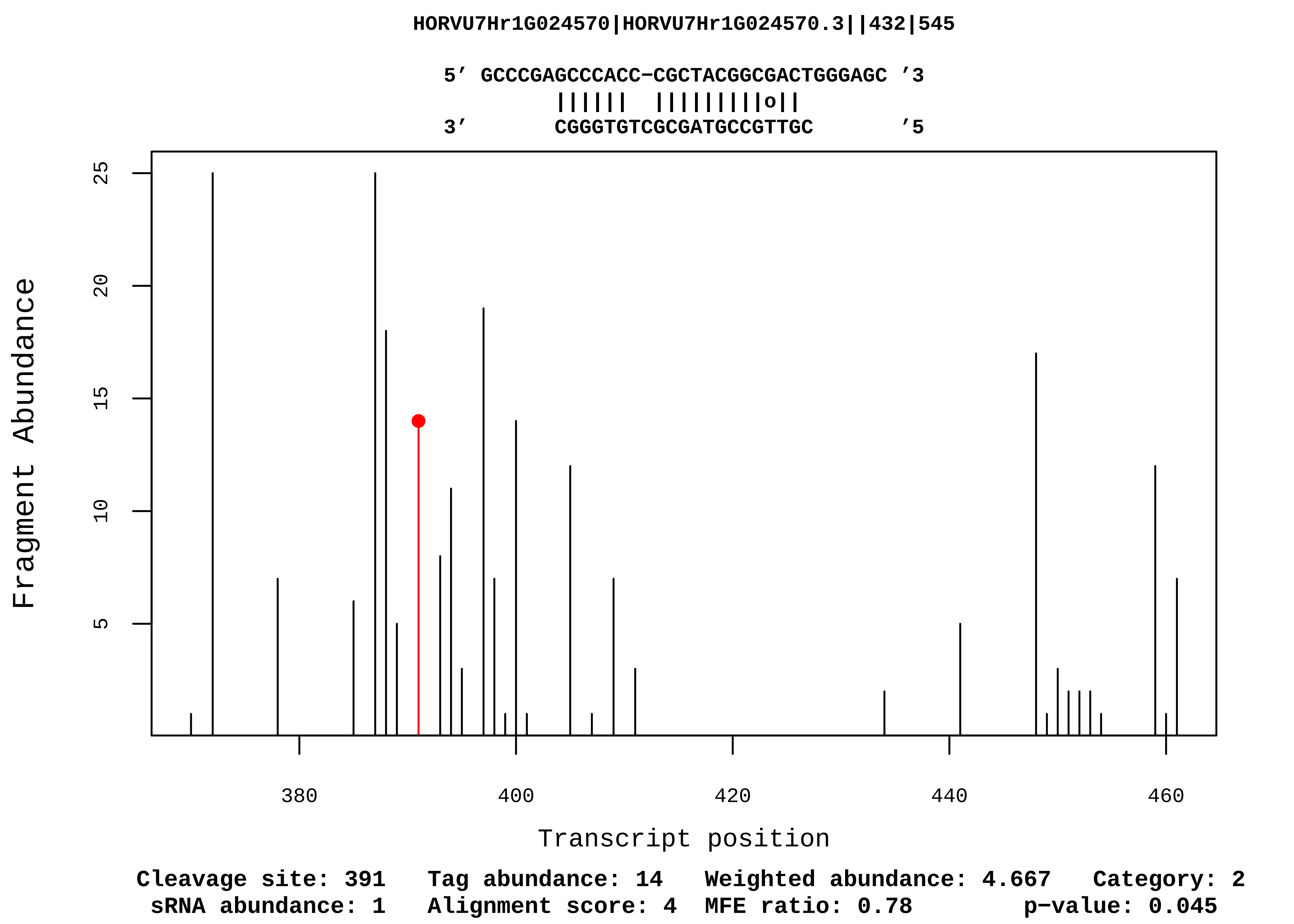
HORVU7Hr1G024570|HORVU7Hr1G024570.3||432|545
5' GCCCGAGCCCACC-CGCTACGGCGACTGGGAGC '3
3' CGGGTGTCGCGATGCCGTTGC '5
Fragment Abundance
Transcript position
Cleavage site: 391
Tag abundance: 14
Weighted abundance: 4.667
Category: 2
sRNA abundance: 1
Alignment score: 4
MFE ratio: 0.78
p-value: 0.045

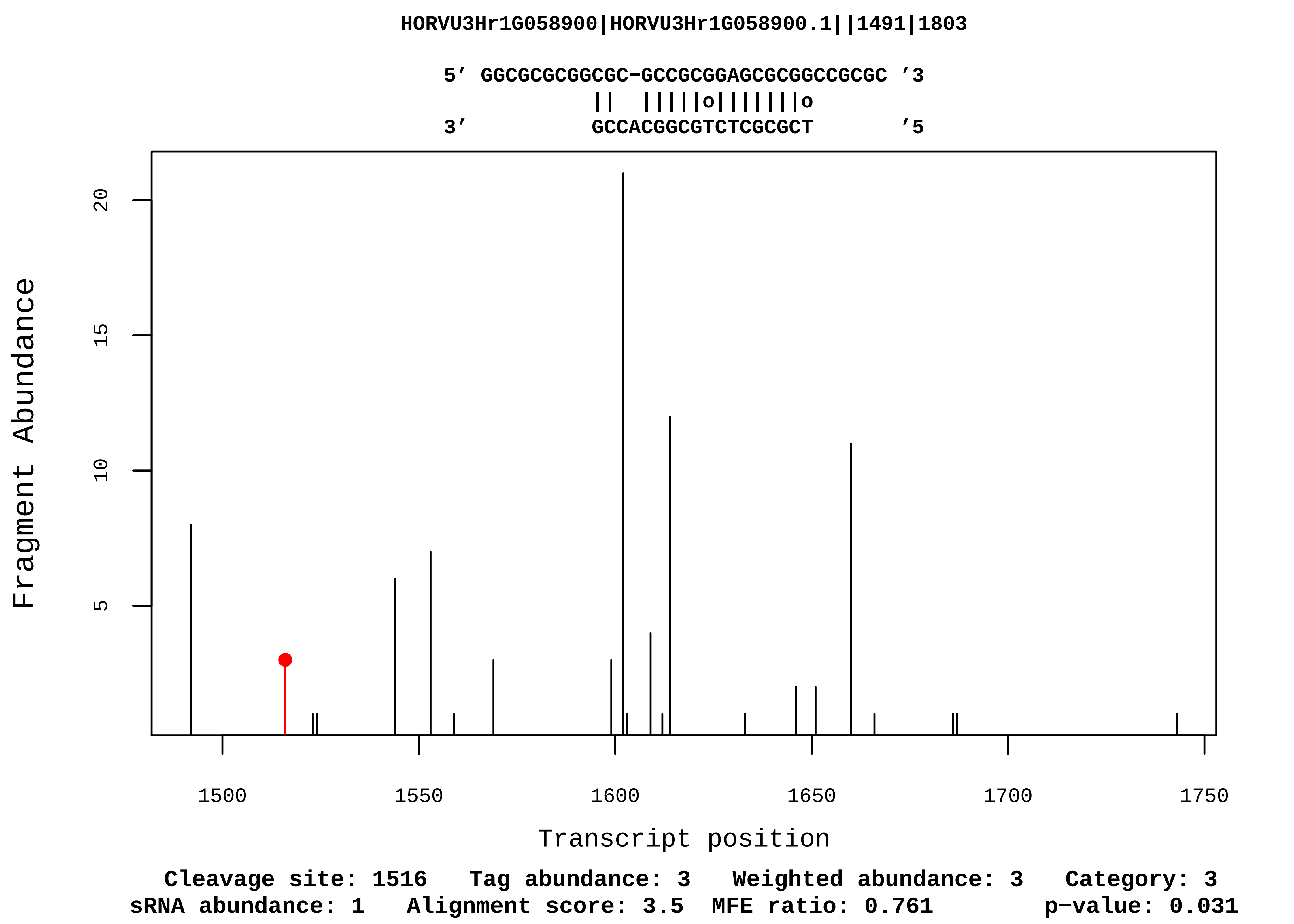
HORVU3Hr1G058900|HORVU3Hr1G058900.1||1491|1803
5' GGCGCGCGGCGC-GCCGCGGAGCGCGGCCGCGC '3
|| |||||o|||||||o
3' GCCACGGCGTCTCGCGCT '5
Fragment Abundance
5
10
15
20
1500
1550
1600
1650
1700
1750
Transcript position
Cleavage site: 1516 Tag abundance: 3 Weighted abundance: 3 Category: 3
sRNA abundance: 1 Alignment score: 3.5 MFE ratio: 0.761 p-value: 0.031

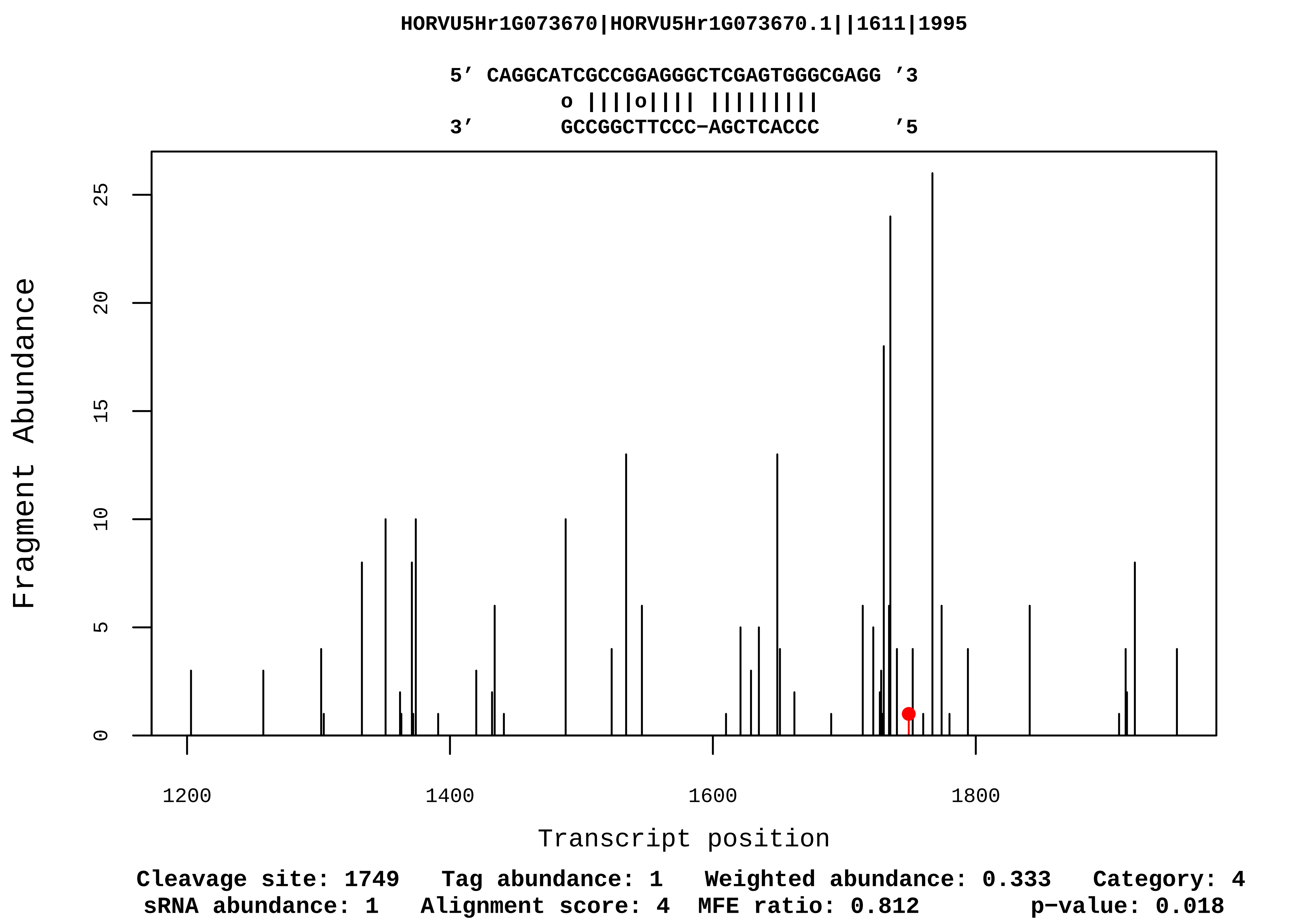

HORVU5Hr1G073670|HORVU5Hr1G073670.1||1611|1995
5' CAGGCATCGCCGGAGGGCTCGAGTGGGCGAGG '3
o ||||o|||| |||||||||
3' GCCGGCTTCCC-AGCTCACCC '5
Fragment Abundance
0
5
10
15
20
25
1200
1400
1600
1800
Transcript position
Cleavage site: 1749 Tag abundance: 1 Weighted abundance: 0.333 Category: 4
sRNA abundance: 1 Alignment score: 4 MFE ratio: 0.812 p-value: 0.018

HORVU5Hr1G073670|HORVU5Hr1G073670.2||1236|1976

```
5' CAGGCATCGCCGGAGGGCTCGAGTGGGCGAGG '3
         o ||||o|||| |||||||||
3'       GCCGGCTTCCC-AGCTCACCC       '5
```

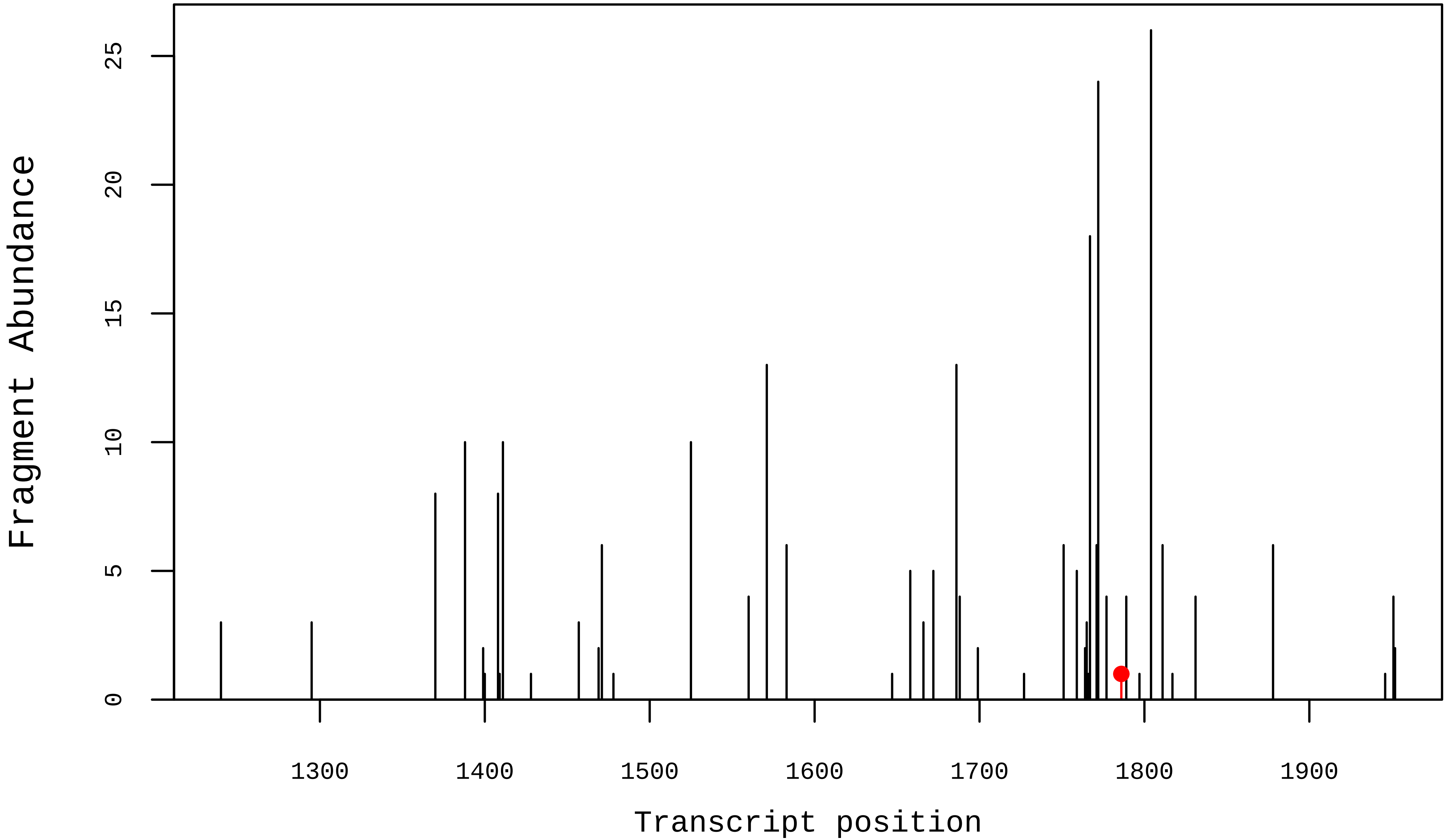

Cleavage site: 1786 Tag abundance: 1 Weighted abundance: 0.333 Category: 4
sRNA abundance: 1 Alignment score: 4 MFE ratio: 0.812 p-value: 0.017

HORVU5Hr1G073670|HORVU5Hr1G073670.3||1236|2220

```
5’ CAGGCATCGCCGGAGGGCTCGAGTGGGCGAGG ’3
          o ||||o|||| |||||||||
3’        GCCGGCTTCCC-AGCTCACCC      ’5
```

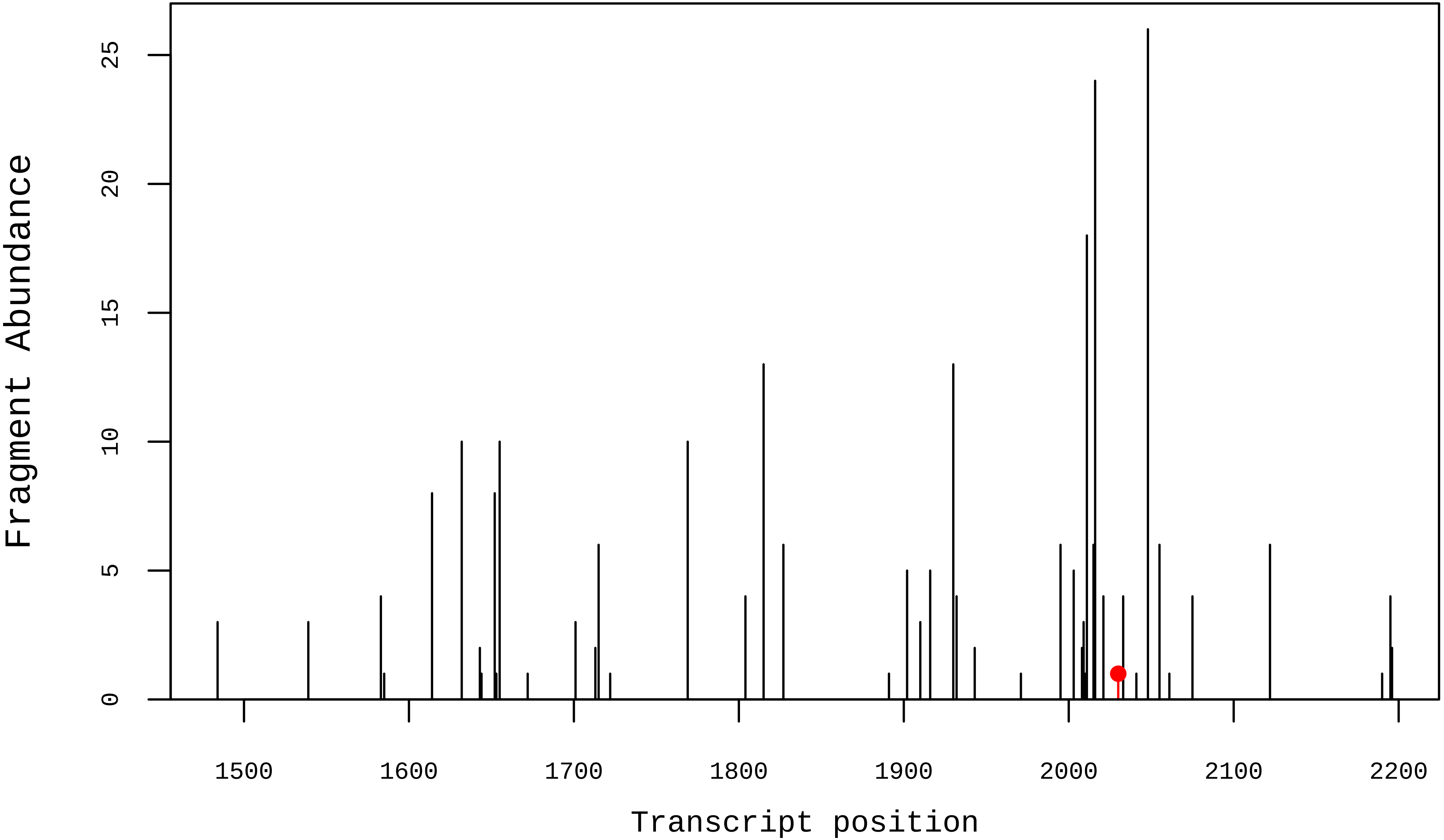

Cleavage site: 2030   Tag abundance: 1   Weighted abundance: 0.333   Category: 4
sRNA abundance: 1   Alignment score: 4   MFE ratio: 0.812   p-value: 0.016

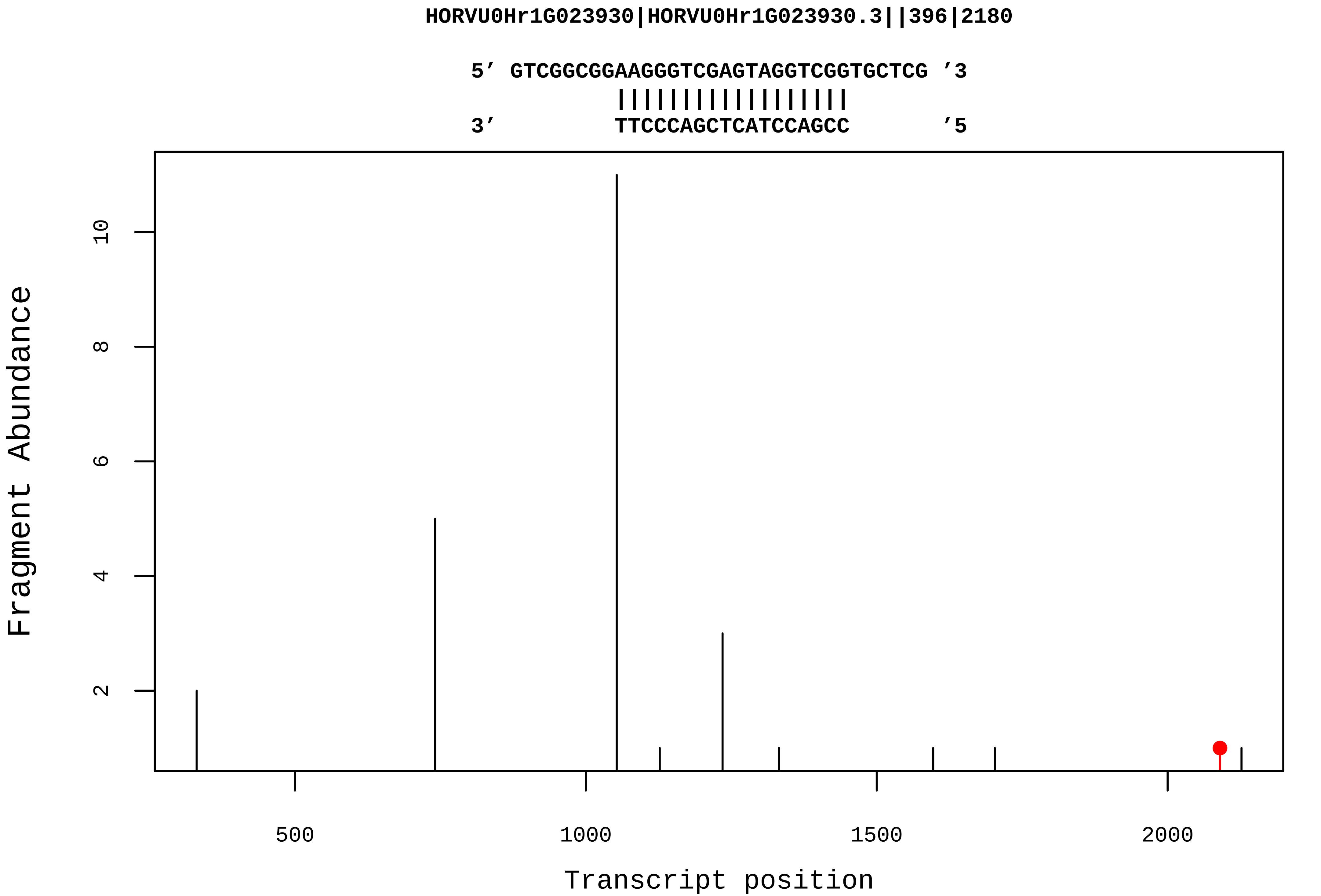

Cleavage site: 2090 Tag abundance: 1 Weighted abundance: 0.143 Category: 4
sRNA abundance: 1 Alignment score: 0 MFE ratio: 1 p-value: 0.017

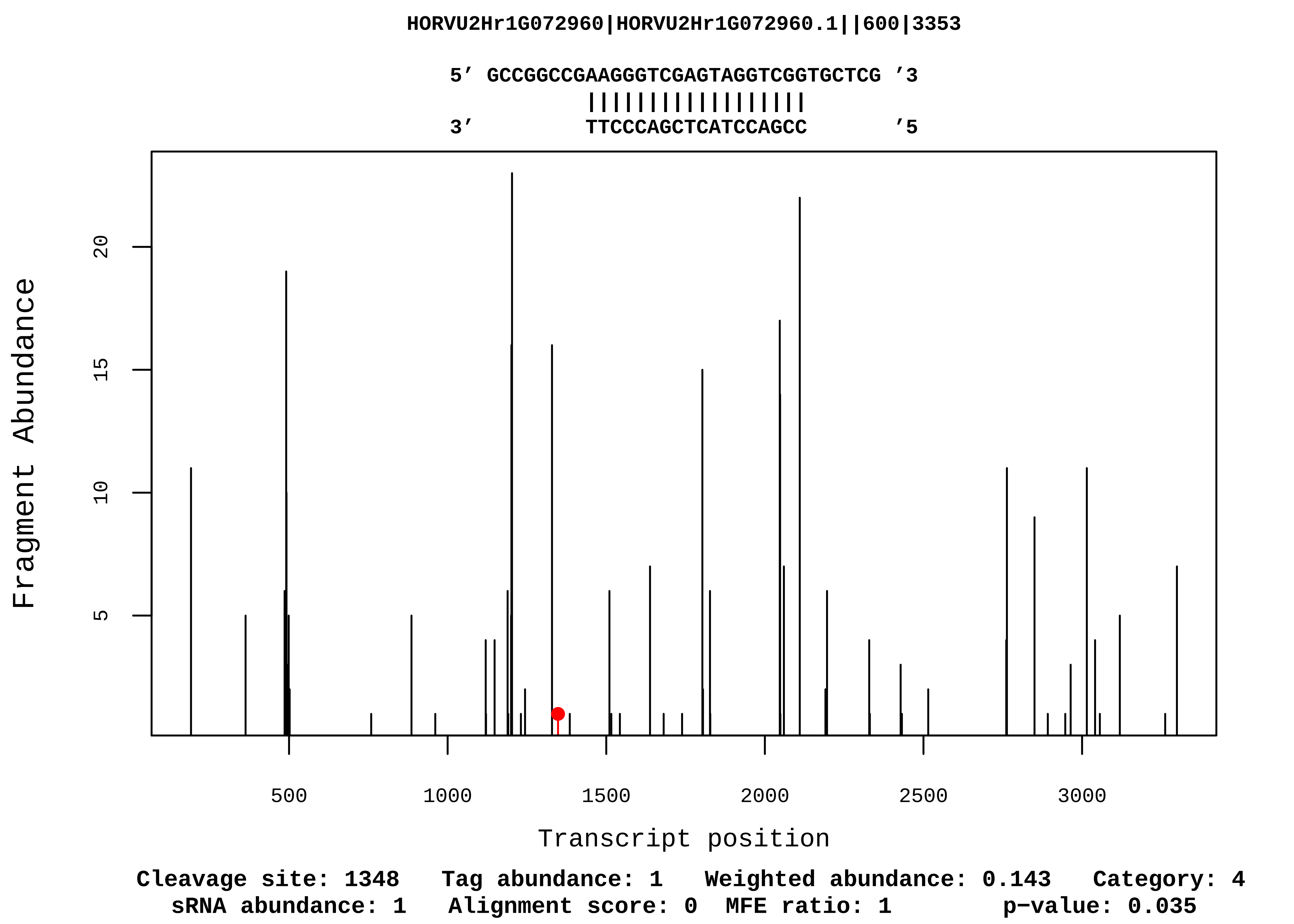

HORVU2Hr1G072960|HORVU2Hr1G072960.1||600|3353
5' GCCGGCCGAAGGGTCGAGTAGGTCGGTGCTCG '3
||||||||||||||||||
3' TTCCCAGCTCATCCAGCC '5
Fragment Abundance
Transcript position
5
10
15
20
500
1000
1500
2000
2500
3000
Cleavage site: 1348 Tag abundance: 1 Weighted abundance: 0.143 Category: 4
sRNA abundance: 1 Alignment score: 0 MFE ratio: 1 p-value: 0.035

HORVU4Hr1G059950|HORVU4Hr1G059950.1||309|1973

```
5' GAGATGGAGGGTGTCGAGTAGG-CGGCTAACCG '3
           oo| |||||||||| |||
3'         TTCCCAGCTCATCCAGCC         '5
```

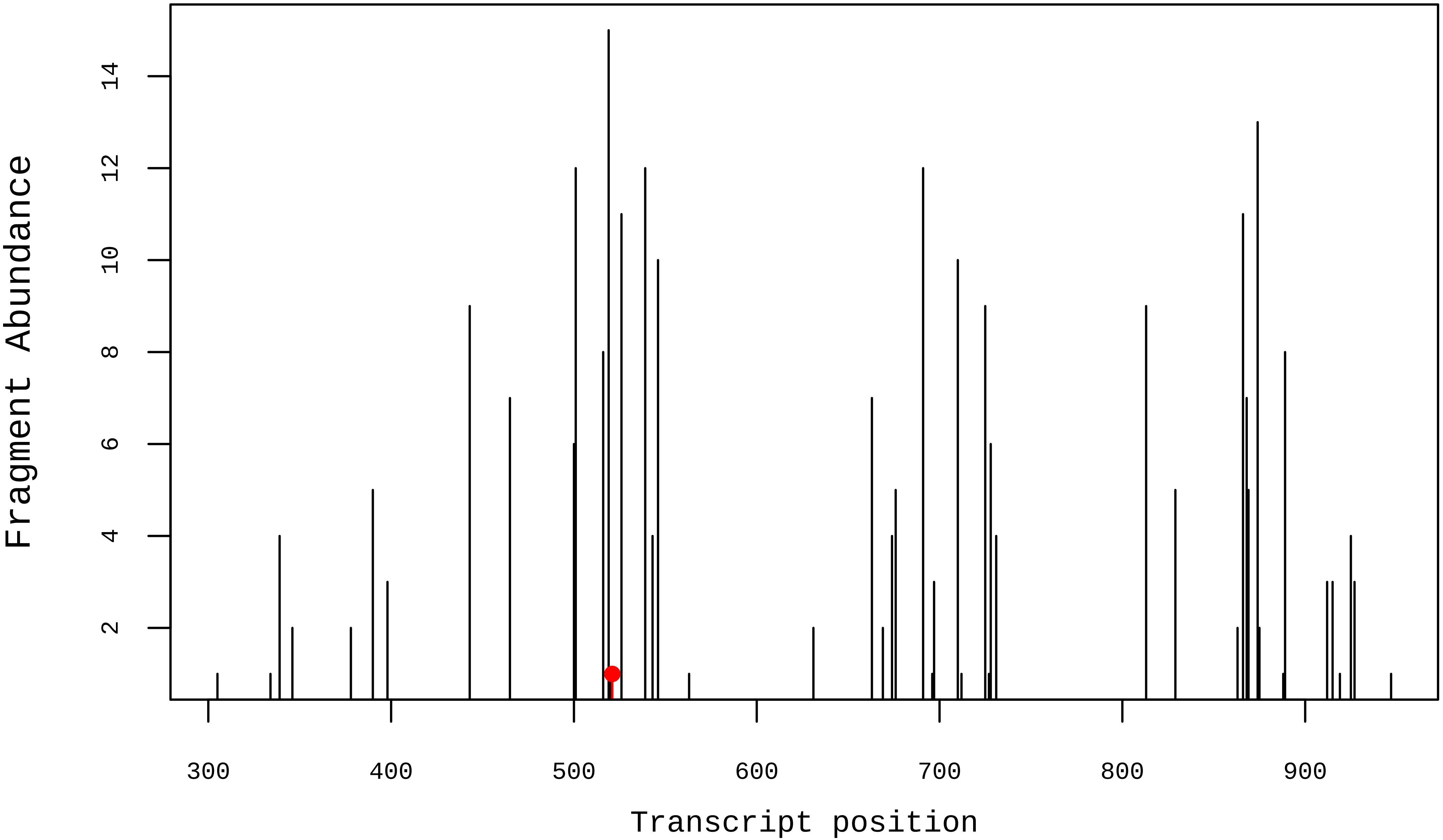

Cleavage site: 521 Tag abundance: 1 Weighted abundance: 1 Category: 4
sRNA abundance: 1 Alignment score: 4 MFE ratio: 0.72 p-value: 0.044

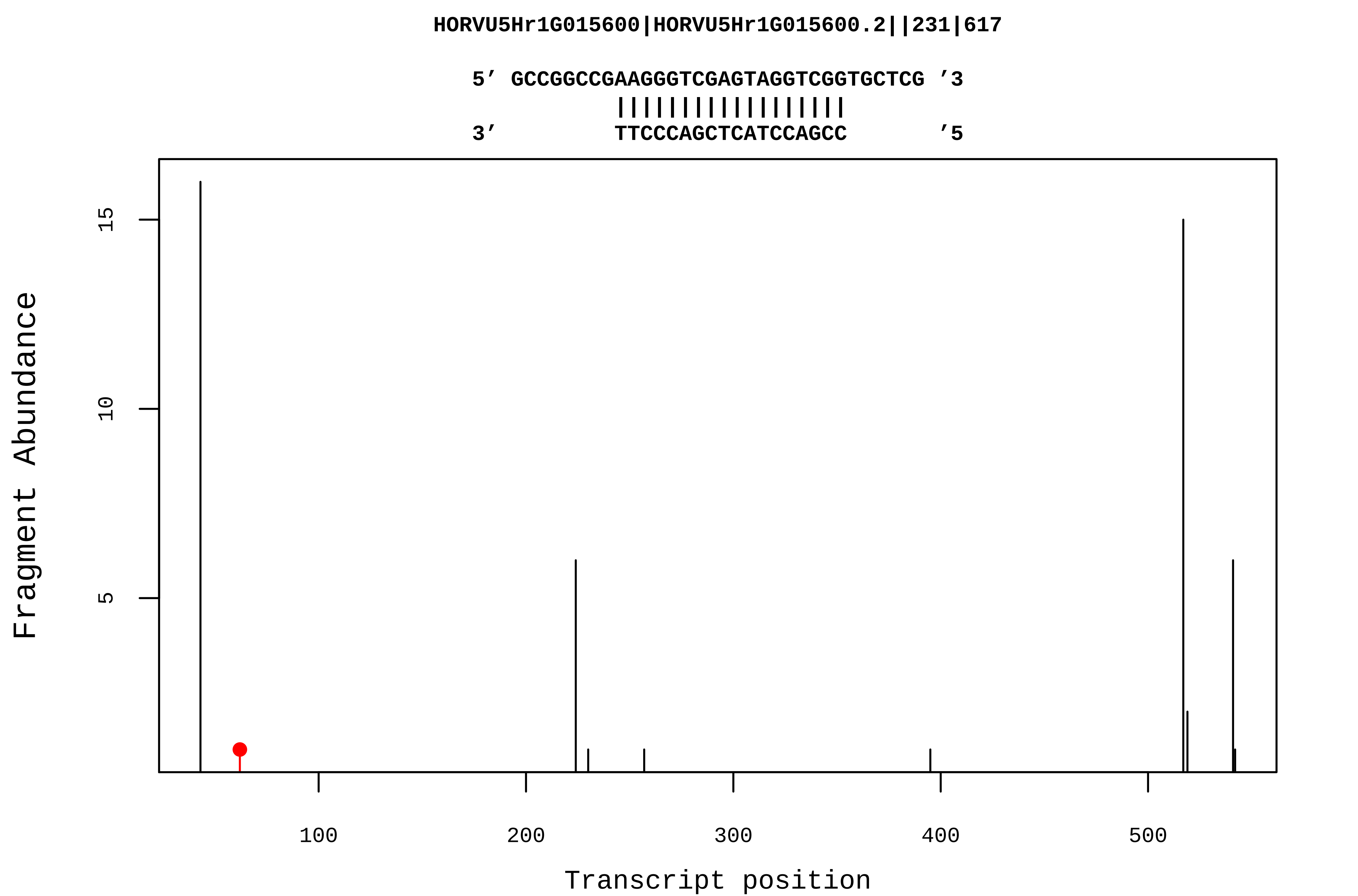

Cleavage site: 62 Tag abundance: 1 Weighted abundance: 0.143 Category: 4
sRNA abundance: 1 Alignment score: 0 MFE ratio: 1 p-value: 0.049

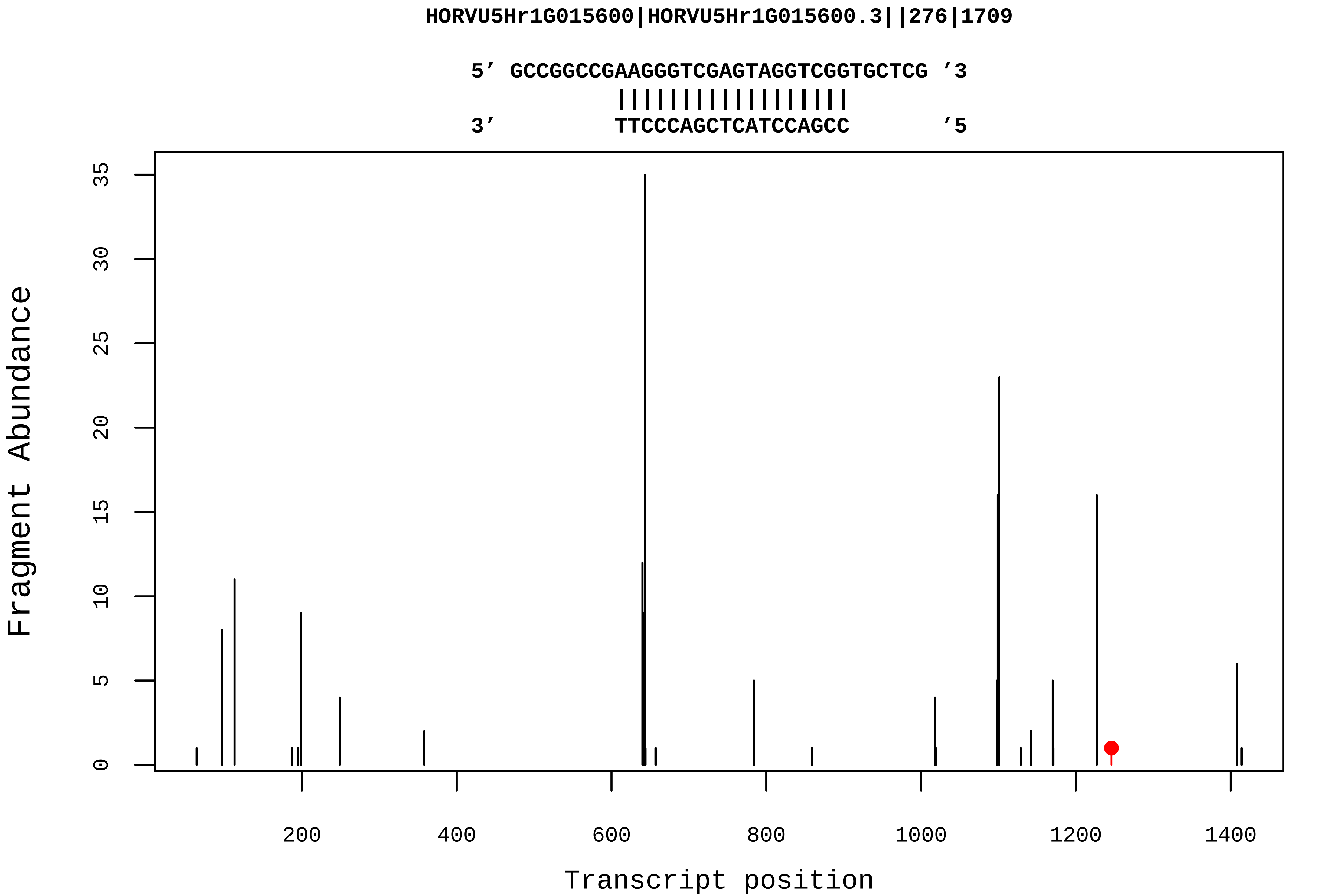

Cleavage site: 1246 Tag abundance: 1 Weighted abundance: 0.143 Category: 4
sRNA abundance: 1 Alignment score: 0 MFE ratio: 1 p−value: 0.038

HORVU5Hr1G015600|HORVU5Hr1G015600.4||207|1852

```
5' GCCGGCCGCAGGGTCGAGTAGGTCGGTGCTCG '3
             ||||||||||||||||
3'          TTCCCAGCTCATCCAGCC        '5
```

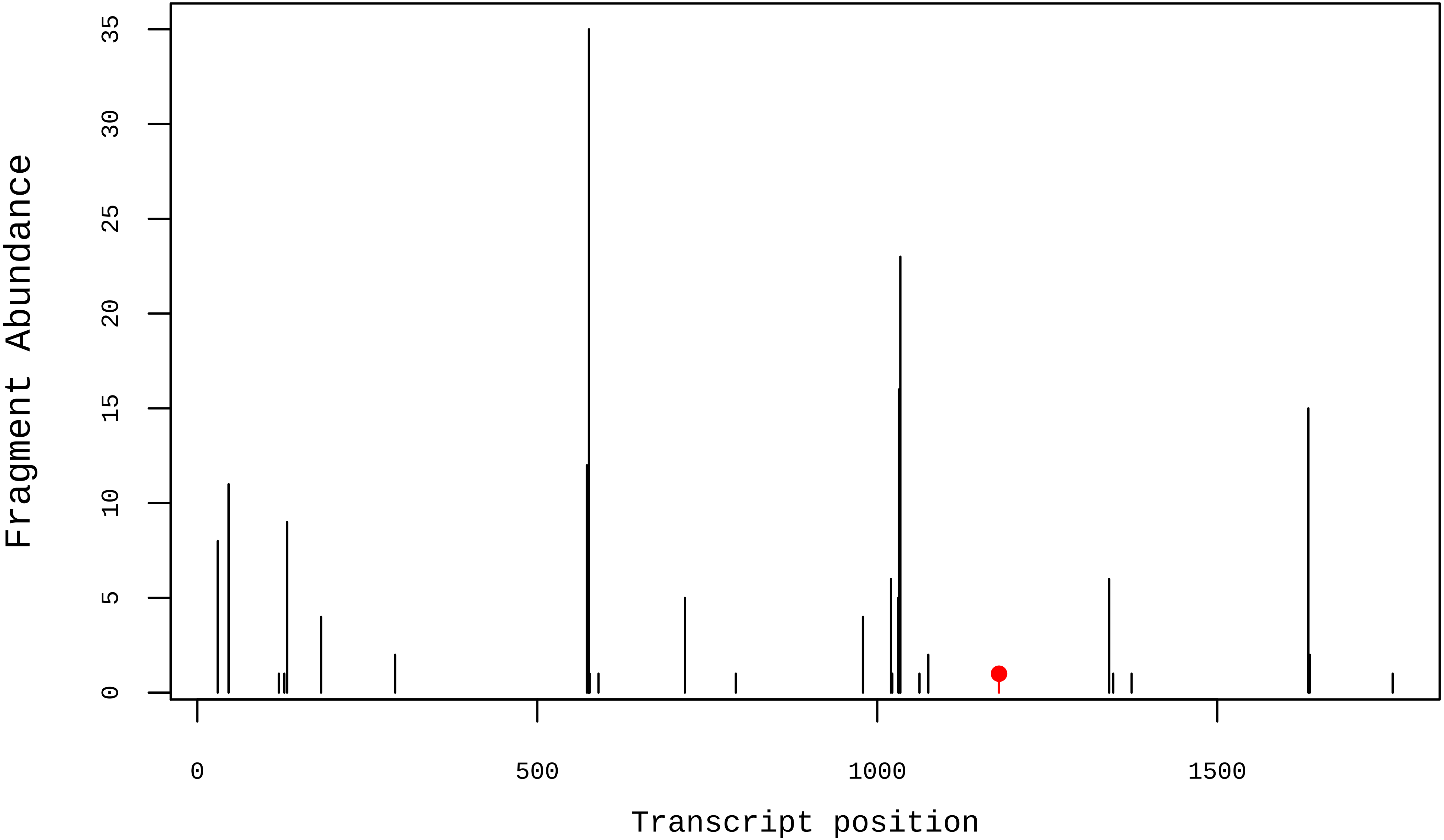

Cleavage site: 1179   Tag abundance: 1   Weighted abundance: 0.143   Category: 4
sRNA abundance: 1   Alignment score: 1   MFE ratio: 0.992   p-value: 0.041

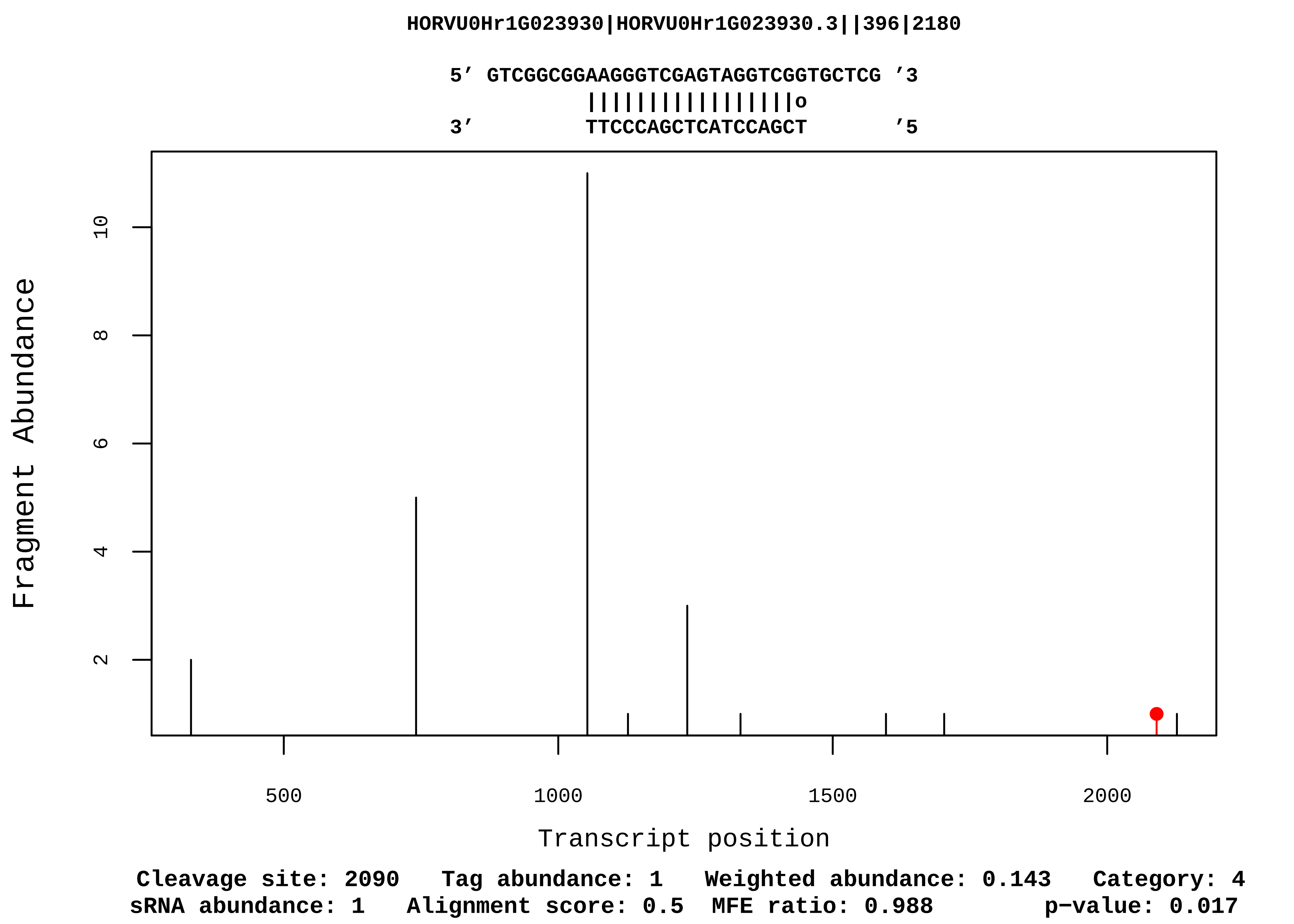

HORVU0Hr1G023930|HORVU0Hr1G023930.3||396|2180
5′ GTCGGCGGAAGGGTCGAGTAGGTCGGTGCTCG ′3
|||||||||||||||||o
3′ TTCCCAGCTCATCCAGCT ′5
Fragment Abundance
2
4
6
8
10
500
1000
1500
2000
Transcript position
Cleavage site: 2090 Tag abundance: 1 Weighted abundance: 0.143 Category: 4
sRNA abundance: 1 Alignment score: 0.5 MFE ratio: 0.988 p-value: 0.017

HORVU2Hr1G072960|HORVU2Hr1G072960.1||600|3353

```
5' GCCGGCCGAAGGGTCGAGTAGGTCGGTGCTCG '3
          ||||||||||||||||o
3'        TTCCCAGCTCATCCAGCT         '5
```

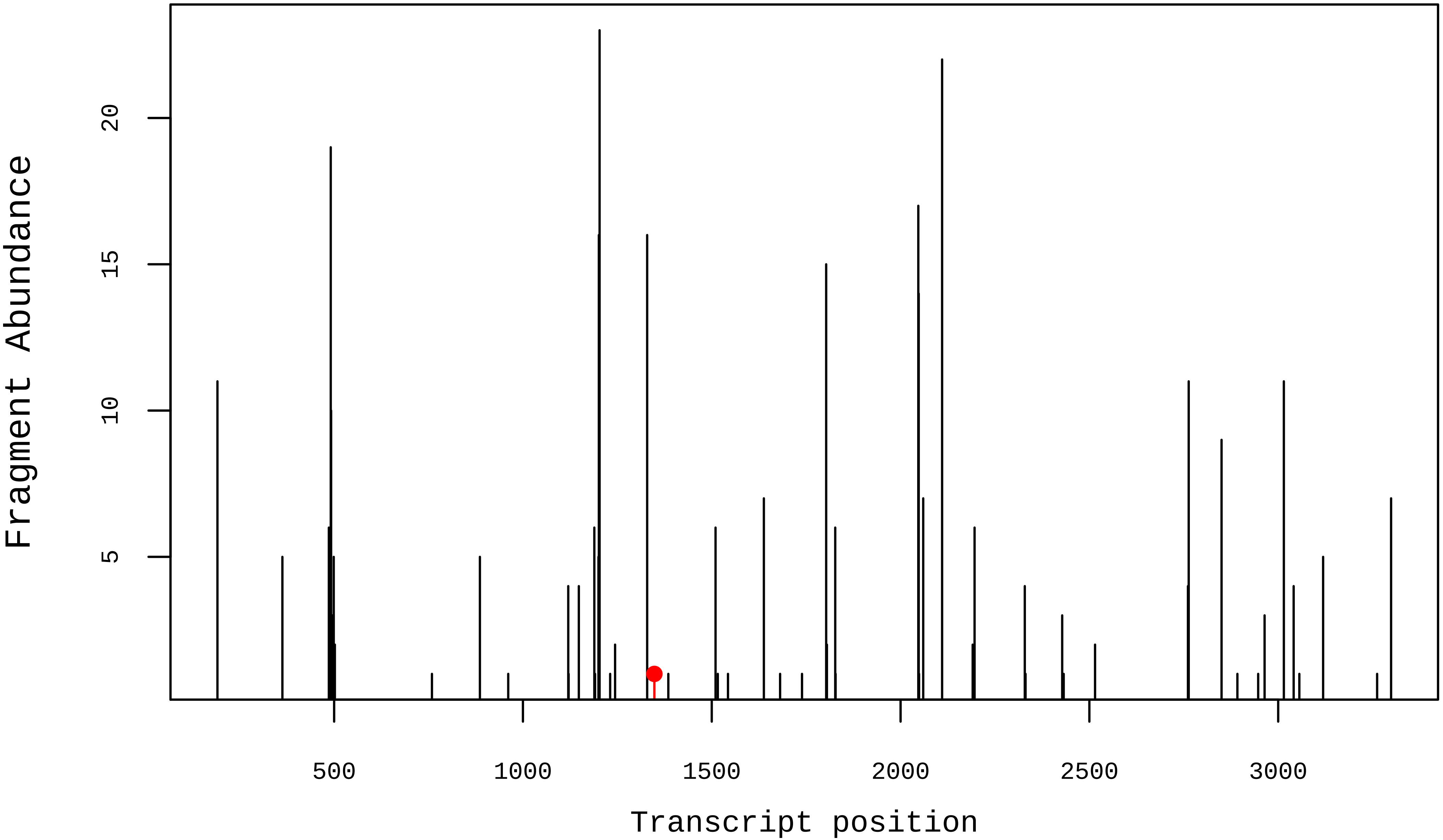

Cleavage site: 1348 Tag abundance: 1 Weighted abundance: 0.143 Category: 4
sRNA abundance: 1 Alignment score: 0.5 MFE ratio: 0.988 p-value: 0.035

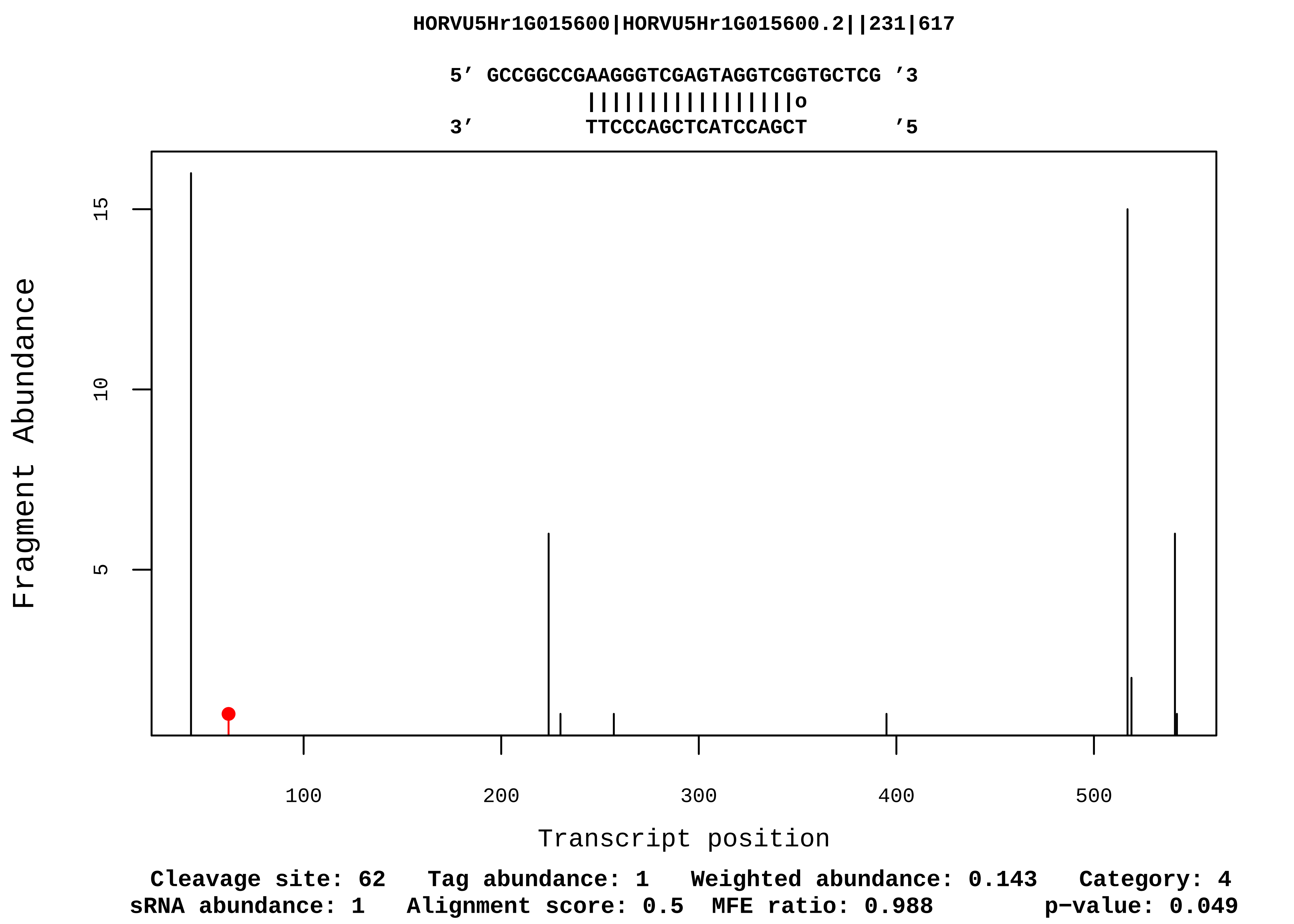
HORVU5Hr1G015600|HORVU5Hr1G015600.2||231|617
5' GCCGGCCGAAGGGTCGAGTAGGTCGGTGCTCG '3
||||||||||||||||o
3' TTCCCAGCTCATCCAGCT '5
Fragment Abundance
5
10
15
100
200
300
400
500
Transcript position
Cleavage site: 62 Tag abundance: 1 Weighted abundance: 0.143 Category: 4
sRNA abundance: 1 Alignment score: 0.5 MFE ratio: 0.988 p-value: 0.049

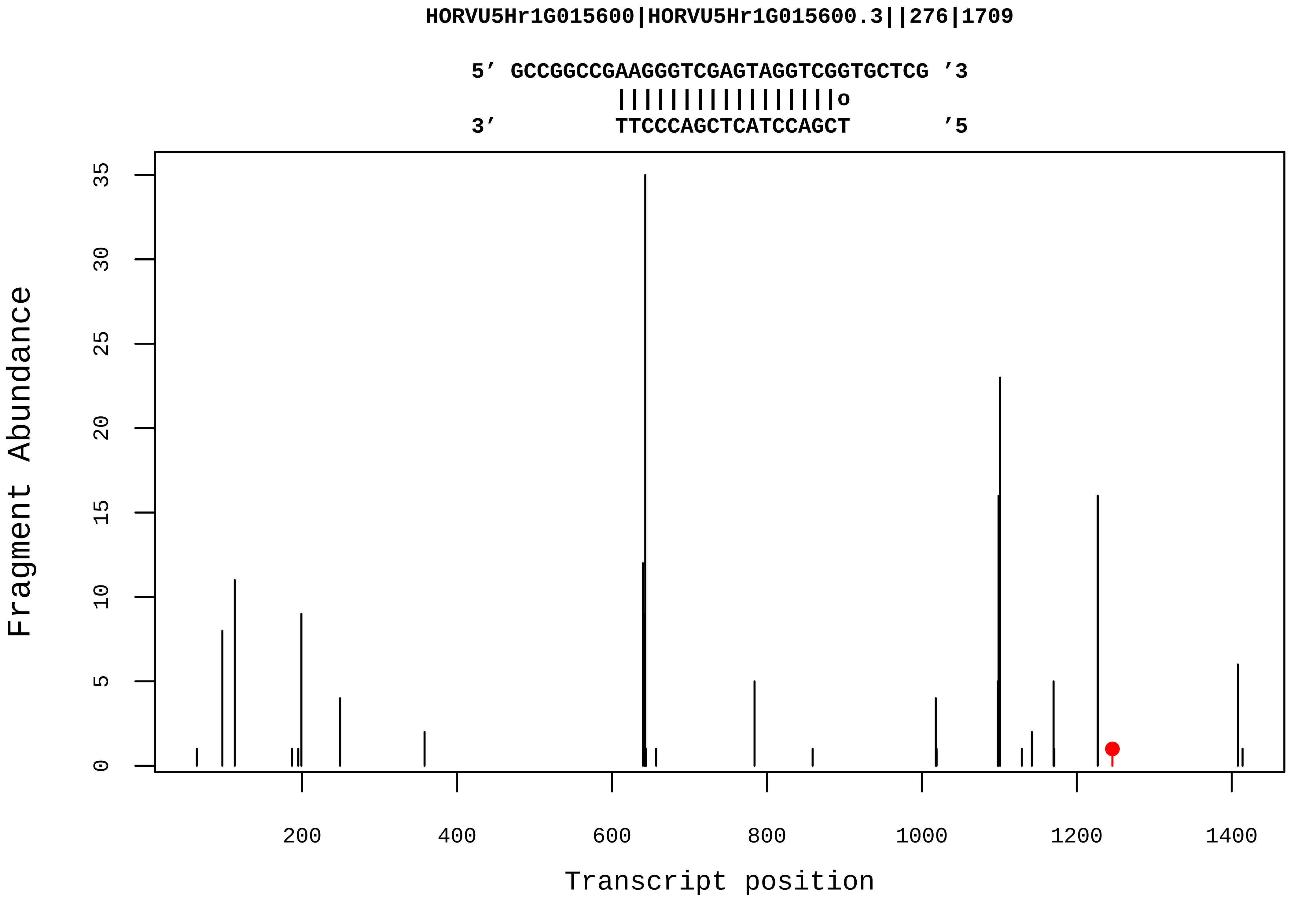

Cleavage site: 1246 Tag abundance: 1 Weighted abundance: 0.143 Category: 4
sRNA abundance: 1 Alignment score: 0.5 MFE ratio: 0.988 p-value: 0.038

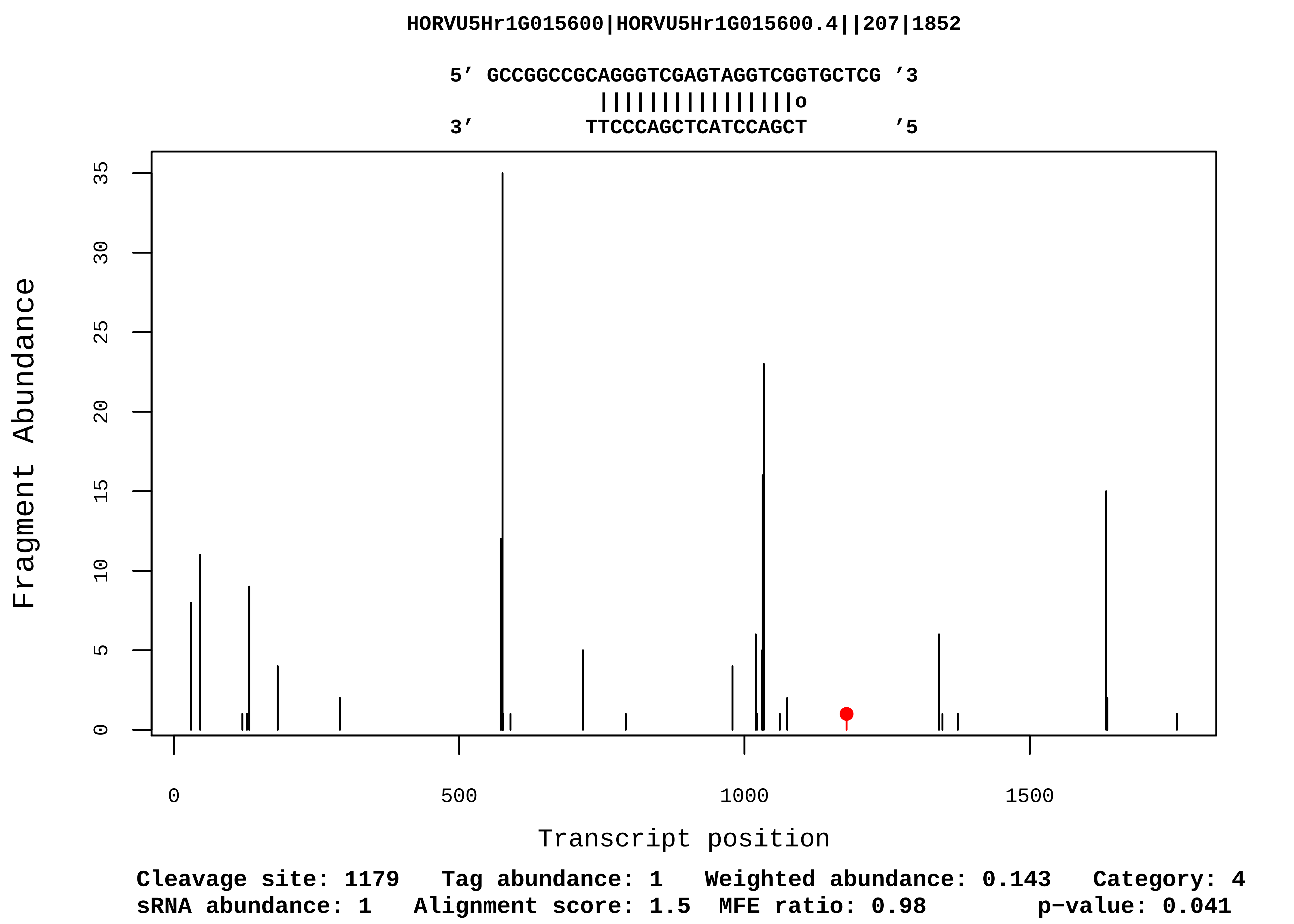
HORVU5Hr1G015600|HORVU5Hr1G015600.4||207|1852
5' GCCGGCCGCAGGGTCGAGTAGGTCGGTGCTCG '3
||||||||||||||||o
3' TTCCCAGCTCATCCAGCT '5
Fragment Abundance
0
5
10
15
20
25
30
35
0
500
1000
1500
Transcript position
Cleavage site: 1179 Tag abundance: 1 Weighted abundance: 0.143 Category: 4
sRNA abundance: 1 Alignment score: 1.5 MFE ratio: 0.98 p-value: 0.041

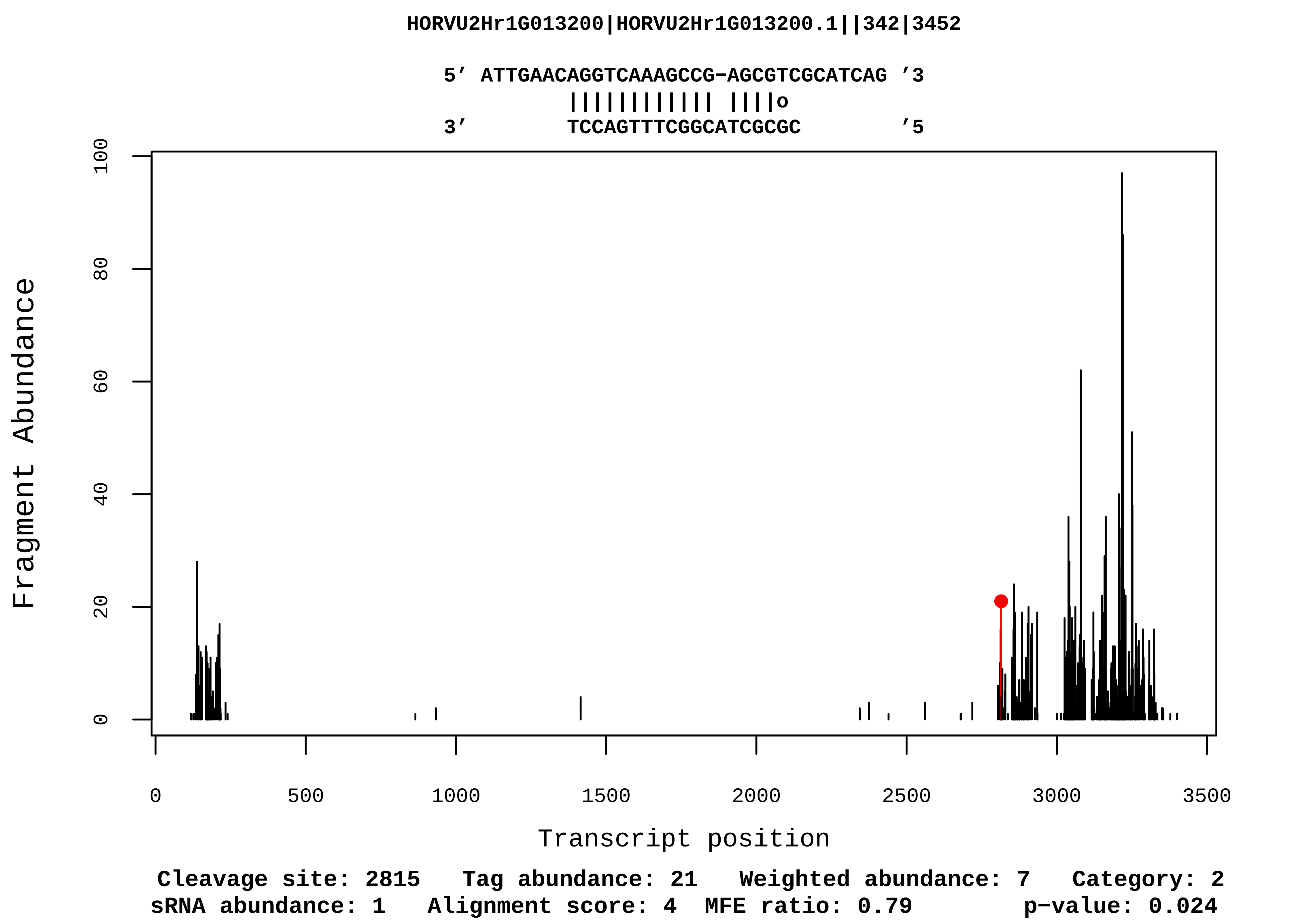
HORVU2Hr1G013200|HORVU2Hr1G013200.1||342|3452
5′ ATTGAACAGGTCAAAGCCG−AGCGTCGCATCAG ′3
||||||||||||| ||||o
3′ TCCAGTTTCGGCATCGCGC ′5
Fragment Abundance
0
20
40
60
80
100
0
500
1000
1500
2000
2500
3000
3500
Transcript position
Cleavage site: 2815 Tag abundance: 21 Weighted abundance: 7 Category: 2
sRNA abundance: 1 Alignment score: 4 MFE ratio: 0.79 p−value: 0.024

HORVU0Hr1G003660|HORVU0Hr1G003660.1||297|1955

```
5' GCCGGCCGAAGGGTCGAGTAGGTCGGTGCTCG '3
      |||||||||||| ||||||||||||
3'    GCCGGCTTCCCCGCTCATCCAGCC       '5
```

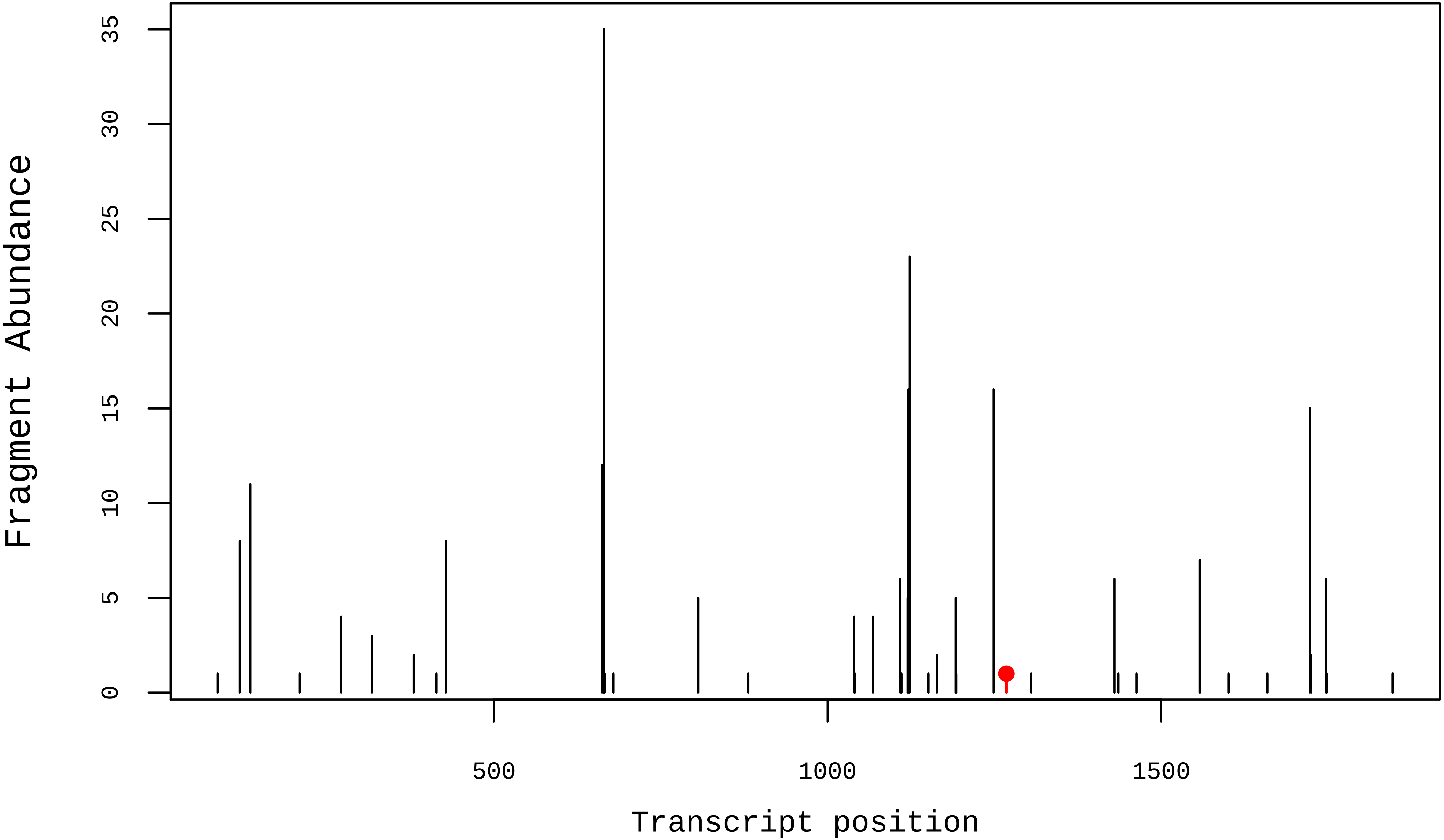

Cleavage site: 1268 Tag abundance: 1 Weighted abundance: 0.143 Category: 4
sRNA abundance: 1 Alignment score: 2 MFE ratio: 0.87 p-value: 0.046

HORVU0Hr1G023930|HORVU0Hr1G023930.3||396|2180

```
5' GTCGGCGGAAGGGTCGAGTAGGTCGGTGCTCG '3
      |||| |||||| ||||||||||||
3'    GCCGGCTTCCCCGCTCATCCAGCC       '5
```

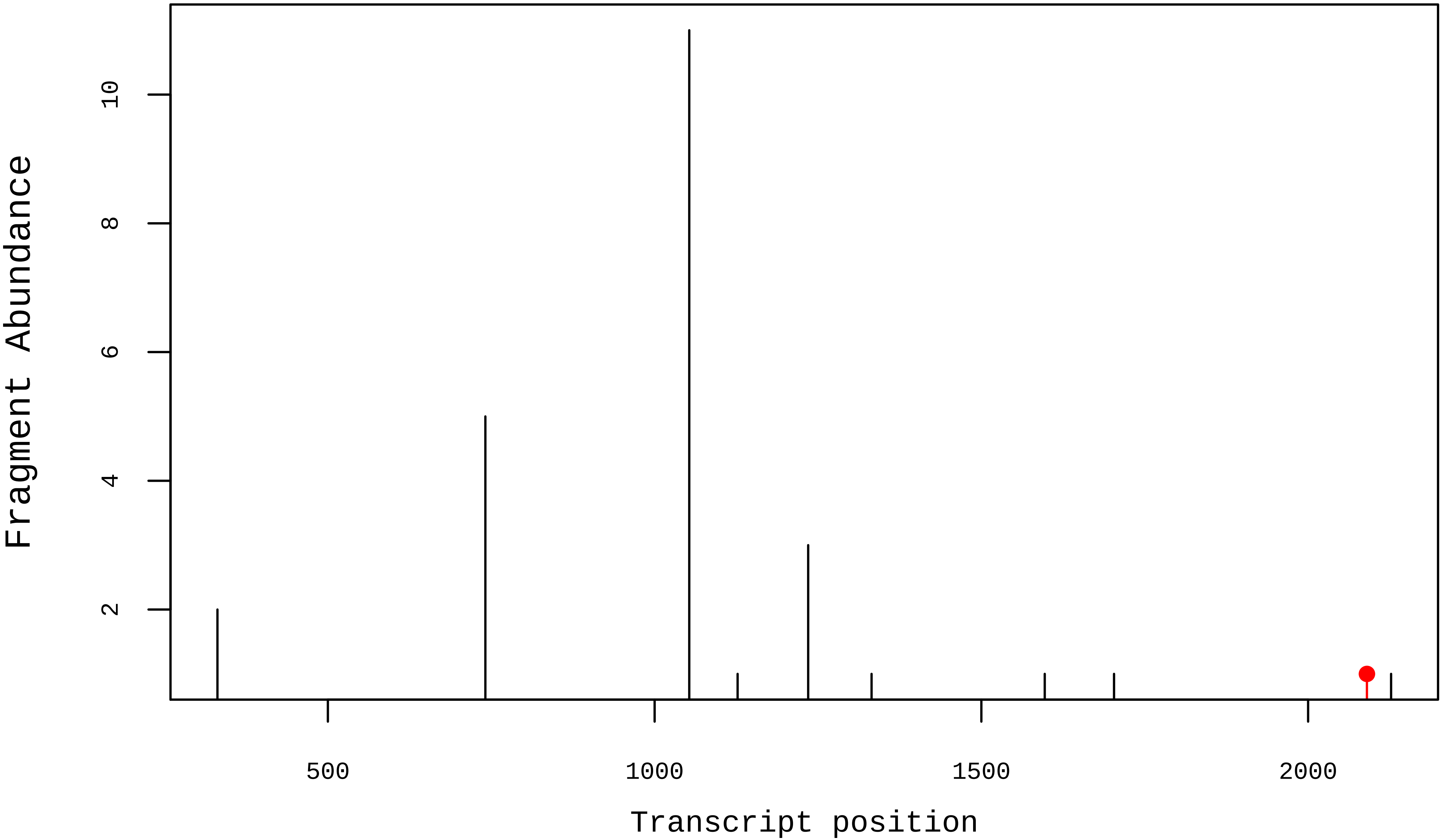

Cleavage site: 2090 Tag abundance: 1 Weighted abundance: 0.143 Category: 4
sRNA abundance: 1 Alignment score: 3 MFE ratio: 0.793 p-value: 0.019

HORVU2Hr1G072960|HORVU2Hr1G072960.1||600|3353

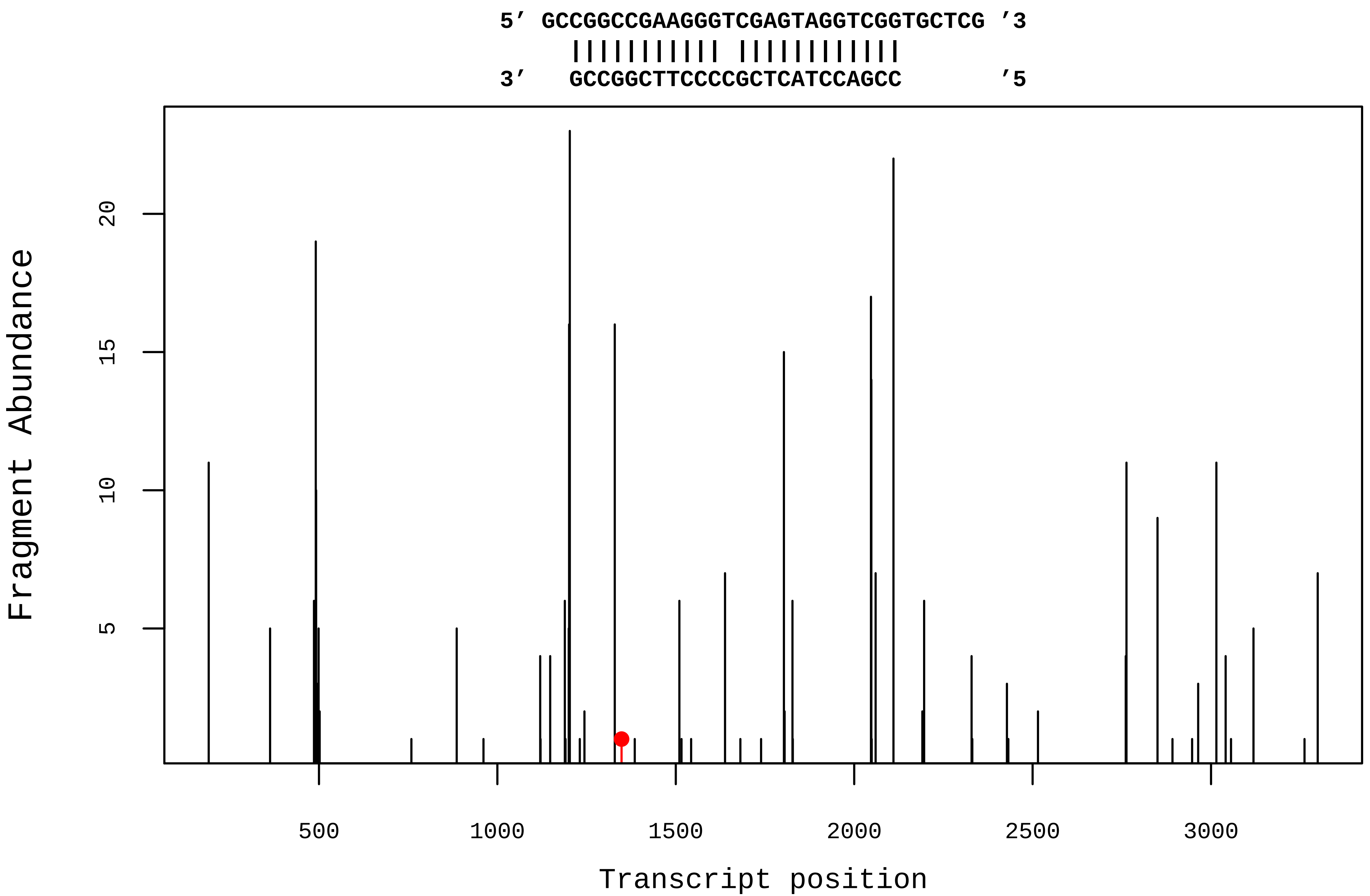

Cleavage site: 1348 Tag abundance: 1 Weighted abundance: 0.143 Category: 4
sRNA abundance: 1 Alignment score: 2 MFE ratio: 0.87 p-value: 0.03

HORVU5Hr1G015600|HORVU5Hr1G015600.1||156|510

```
5' GCCGGCCGAAGGGTCGAGTAGGTCGGTGCTCG '3
      ||||||||||| ||||||||||||
3'    GCCGGCTTCCCCGCTCATCCAGCC       '5
```

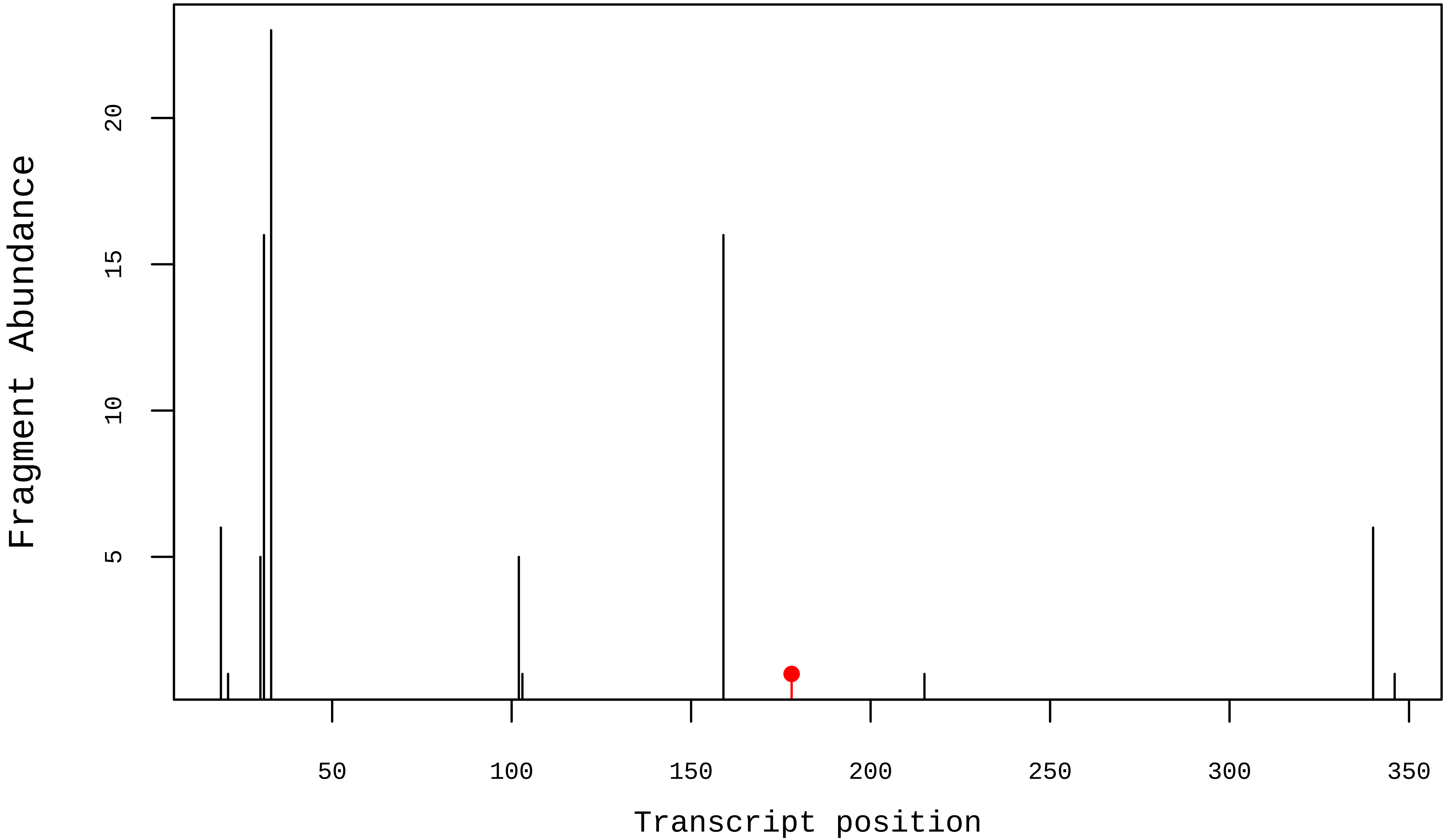

Cleavage site: 178   Tag abundance: 1   Weighted abundance: 0.143   Category: 4
sRNA abundance: 1   Alignment score: 2   MFE ratio: 0.87   p-value: 0.05

HORVU5Hr1G015600|HORVU5Hr1G015600.2||231|617

```
5' GCCGGCCGAAGGGTCGAGTAGGTCGGTGCTCG '3
     ||||||||||| ||||||||||||
3'   GCCGGCTTCCCCGCTCATCCAGCC       '5
```

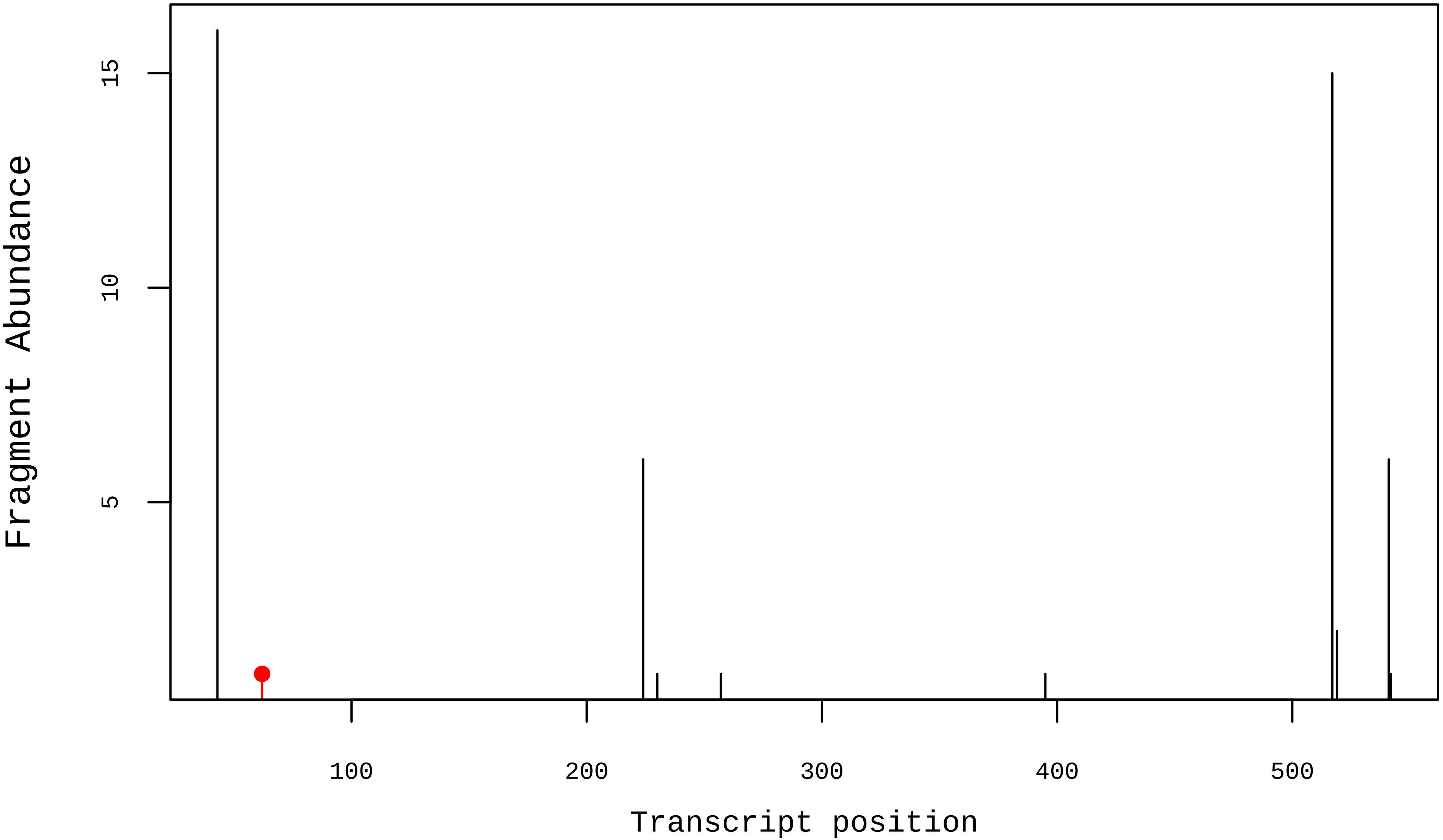

Cleavage site: 62   Tag abundance: 1   Weighted abundance: 0.143   Category: 4
sRNA abundance: 1   Alignment score: 2   MFE ratio: 0.87   p-value: 0.041

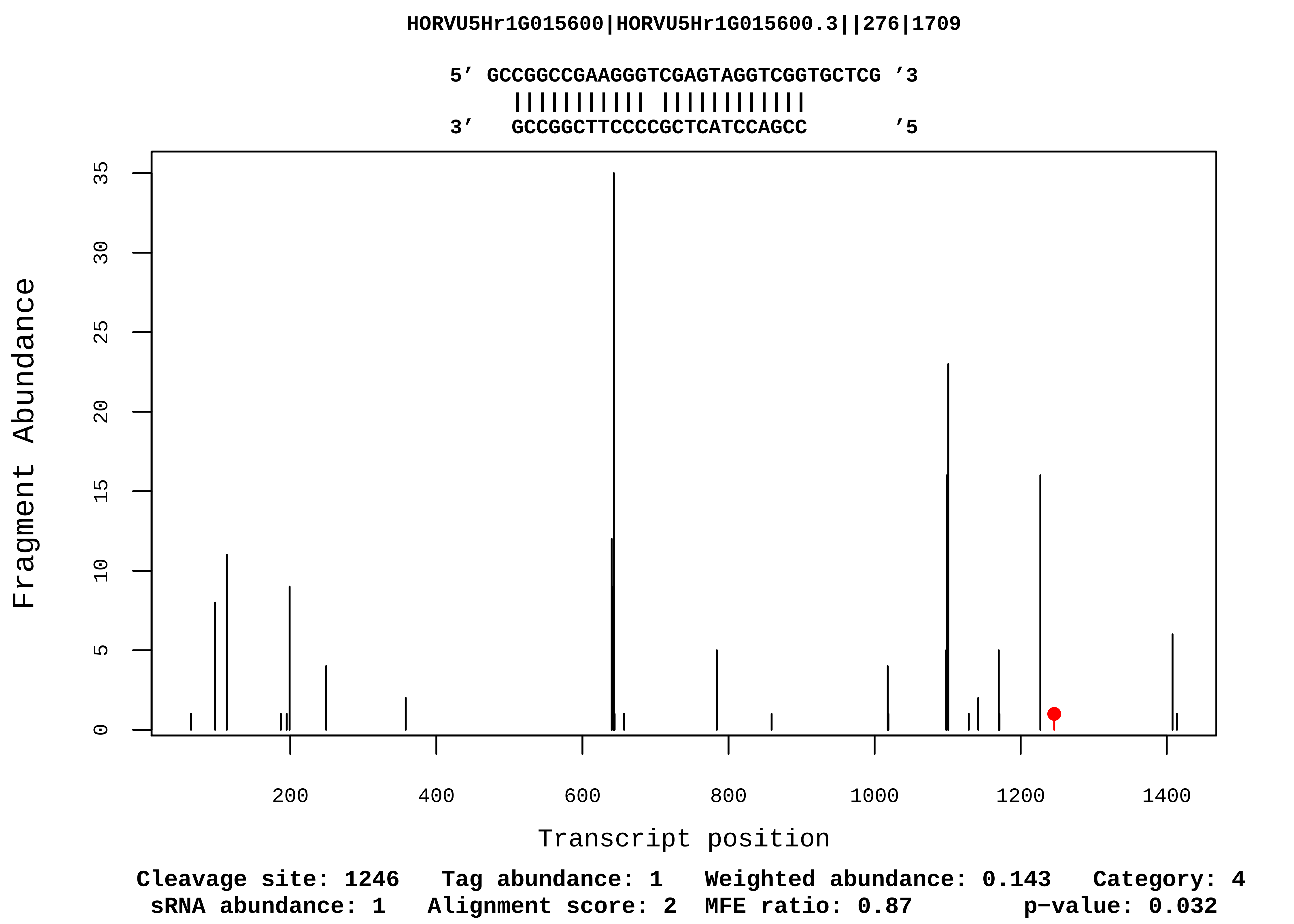
HORVU5Hr1G015600|HORVU5Hr1G015600.3||276|1709
5' GCCGGCCGAAGGGTCGAGTAGGTCGGTGCTCG '3
3' GCCGGCTTCCCCGCTCATCCAGCC '5
Fragment Abundance
0
5
10
15
20
25
30
35
200
400
600
800
1000
1200
1400
Transcript position
Cleavage site: 1246 Tag abundance: 1 Weighted abundance: 0.143 Category: 4
sRNA abundance: 1 Alignment score: 2 MFE ratio: 0.87 p-value: 0.032

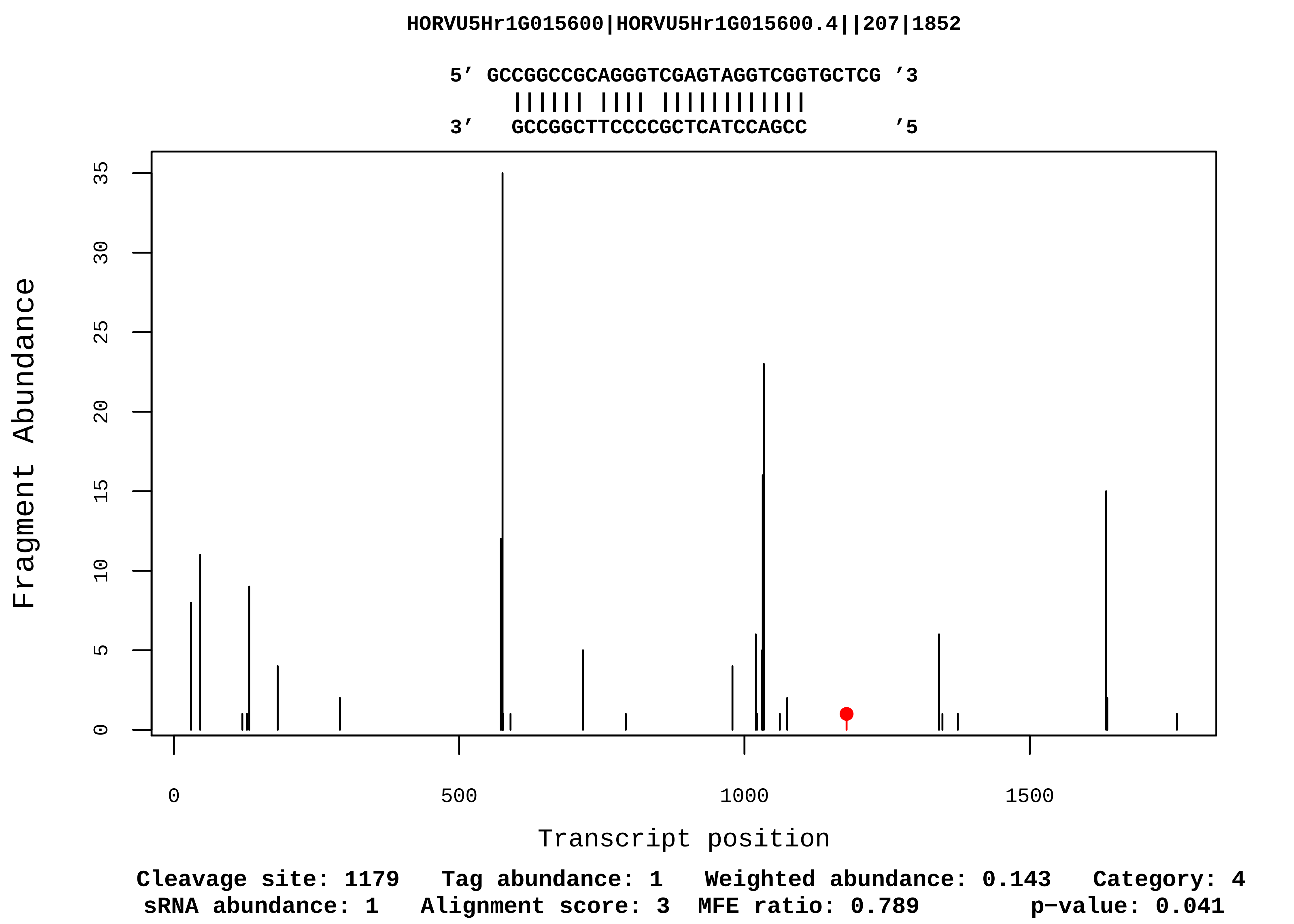
HORVU5Hr1G015600|HORVU5Hr1G015600.4||207|1852
5' GCCGGCCGCAGGGTCGAGTAGGTCGGTGCTCG '3
|||||| |||| ||||||||||||
3' GCCGGCTTCCCCGCTCATCCAGCC '5
Fragment Abundance
0
5
10
15
20
25
30
35
0
500
1000
1500
Transcript position
Cleavage site: 1179 Tag abundance: 1 Weighted abundance: 0.143 Category: 4
sRNA abundance: 1 Alignment score: 3 MFE ratio: 0.789 p-value: 0.041

HORVU0Hr1G023930|HORVU0Hr1G023930.3||396|2180

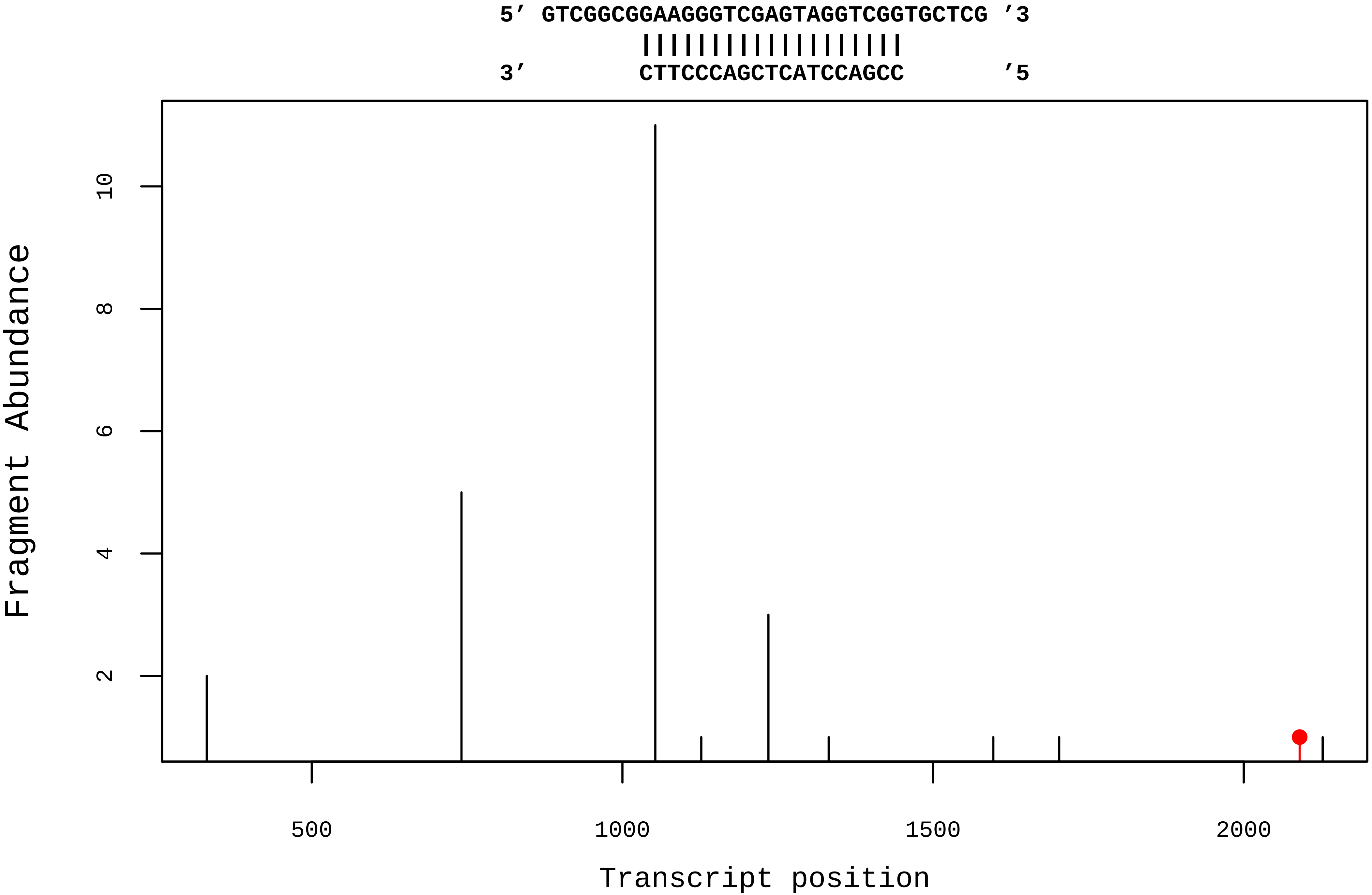

Cleavage site: 2090 Tag abundance: 1 Weighted abundance: 0.143 Category: 4
sRNA abundance: 1 Alignment score: 0 MFE ratio: 1 p-value: 0.017

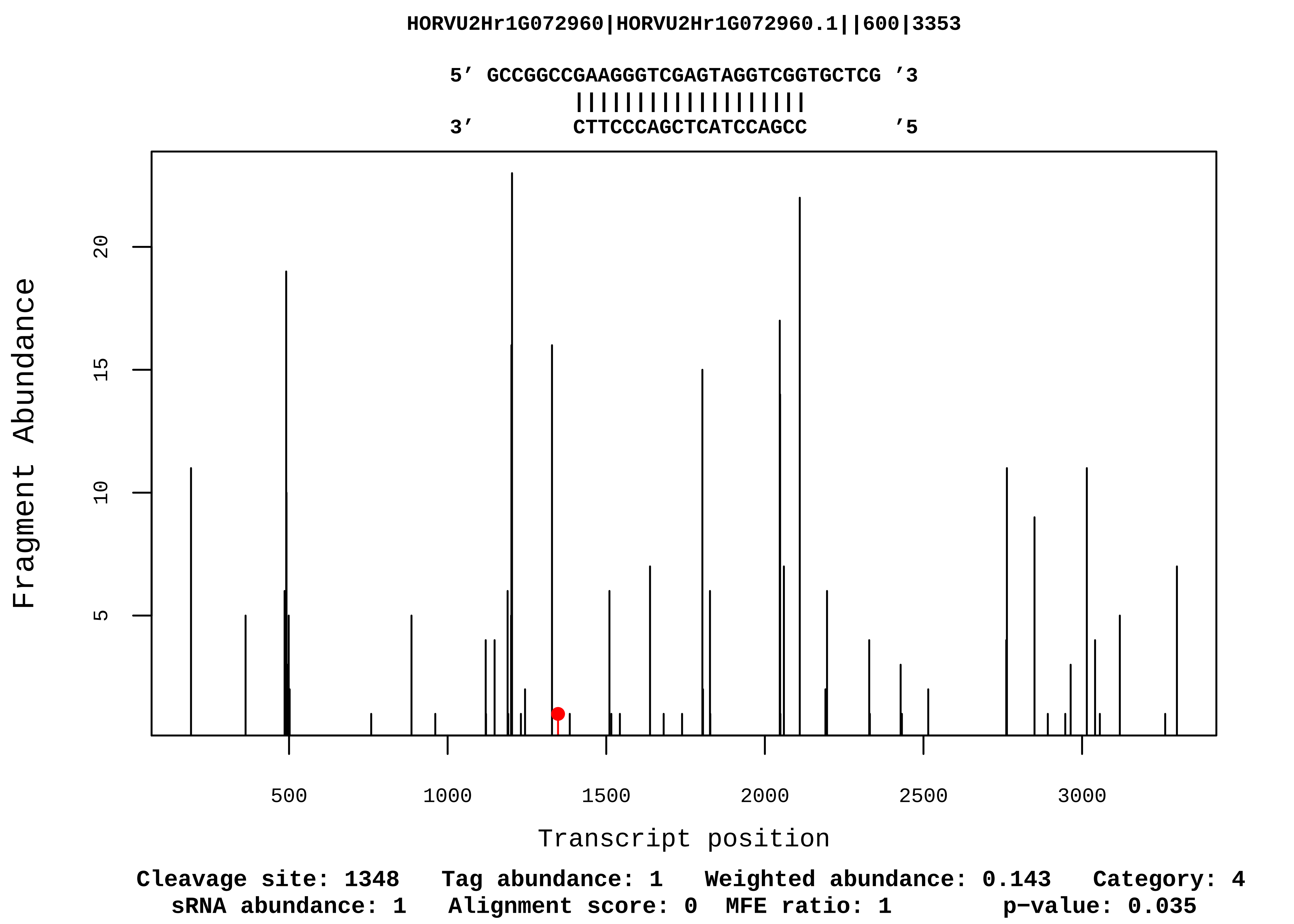
HORVU2Hr1G072960|HORVU2Hr1G072960.1||600|3353
5’ GCCGGCCGAAGGGTCGAGTAGGTCGGTGCTCG ’3
||||||||||||||||||
3’ CTTCCCAGCTCATCCAGCC ’5
Fragment Abundance
5
10
15
20
500
1000
1500
2000
2500
3000
Transcript position
Cleavage site: 1348 Tag abundance: 1 Weighted abundance: 0.143 Category: 4
sRNA abundance: 1 Alignment score: 0 MFE ratio: 1 p-value: 0.035

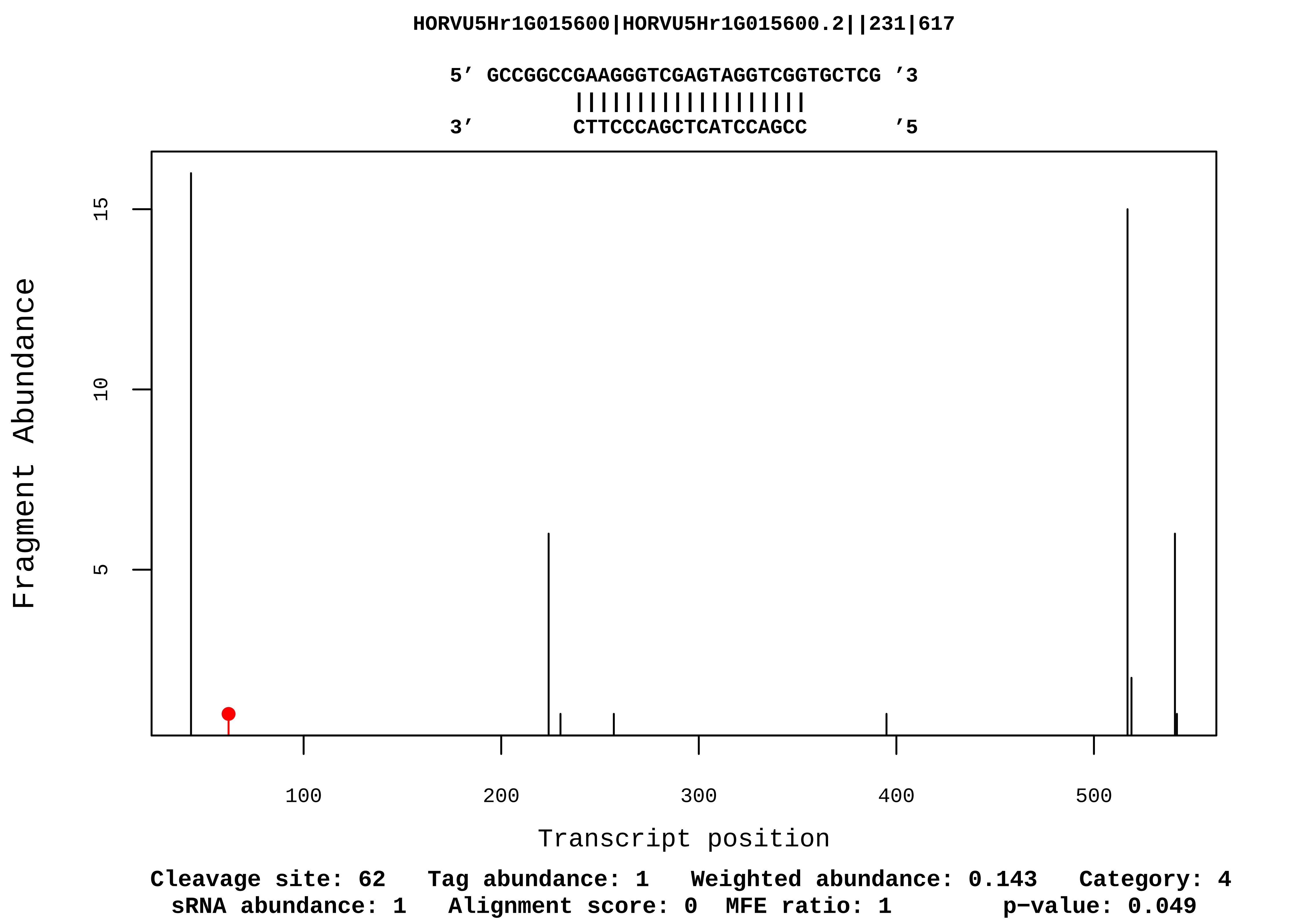
HORVU5Hr1G015600|HORVU5Hr1G015600.2||231|617
5' GCCGGCCGAAGGGTCGAGTAGGTCGGTGCTCG '3
||||||||||||||||||
3' CTTCCCAGCTCATCCAGCC '5
Fragment Abundance
5
10
15
100
200
300
400
500
Transcript position
Cleavage site: 62 Tag abundance: 1 Weighted abundance: 0.143 Category: 4
sRNA abundance: 1 Alignment score: 0 MFE ratio: 1 p-value: 0.049

HORVU5Hr1G015600|HORVU5Hr1G015600.3||276|1709

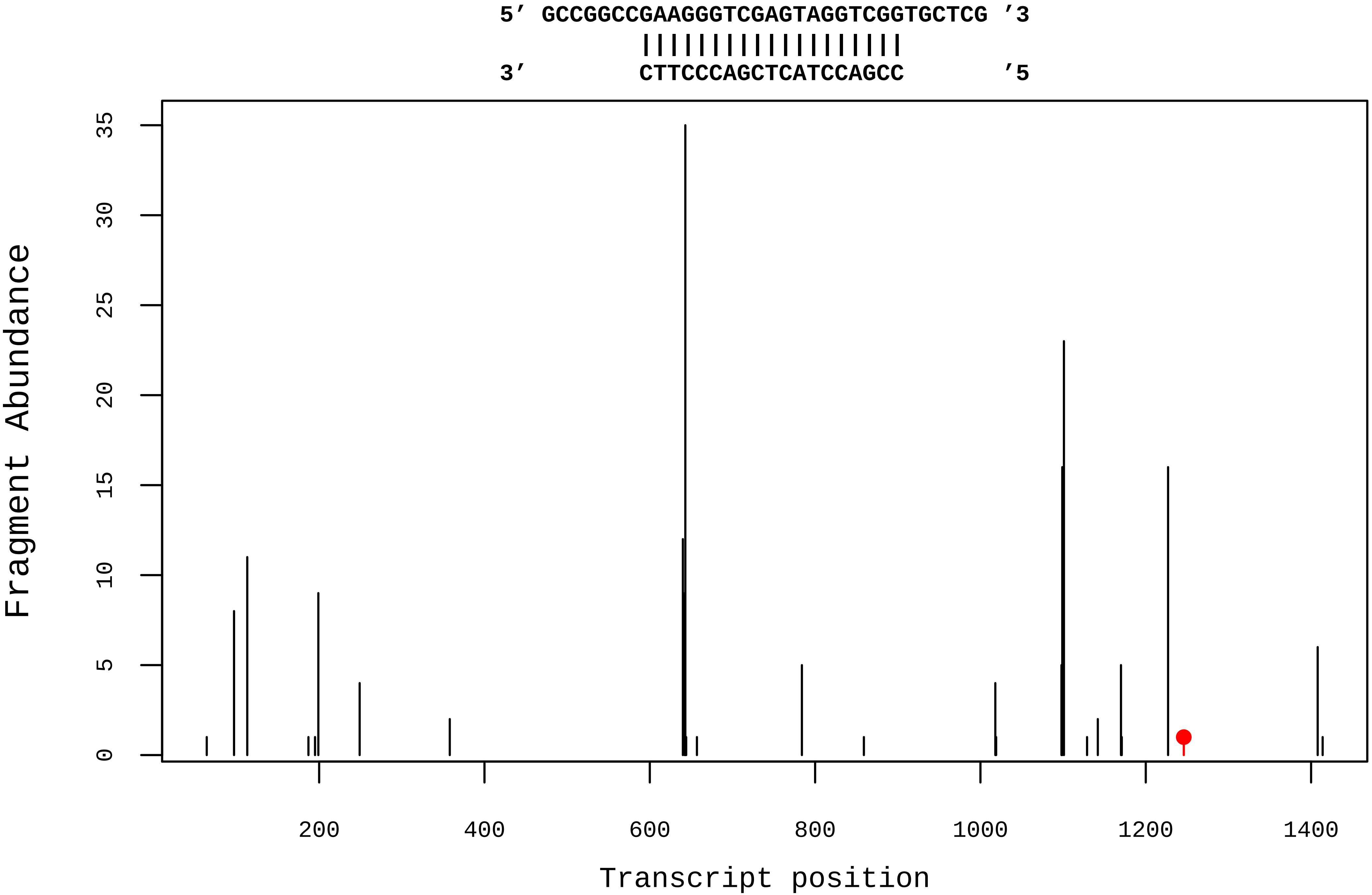

Cleavage site: 1246 Tag abundance: 1 Weighted abundance: 0.143 Category: 4
sRNA abundance: 1 Alignment score: 0 MFE ratio: 1 p-value: 0.038

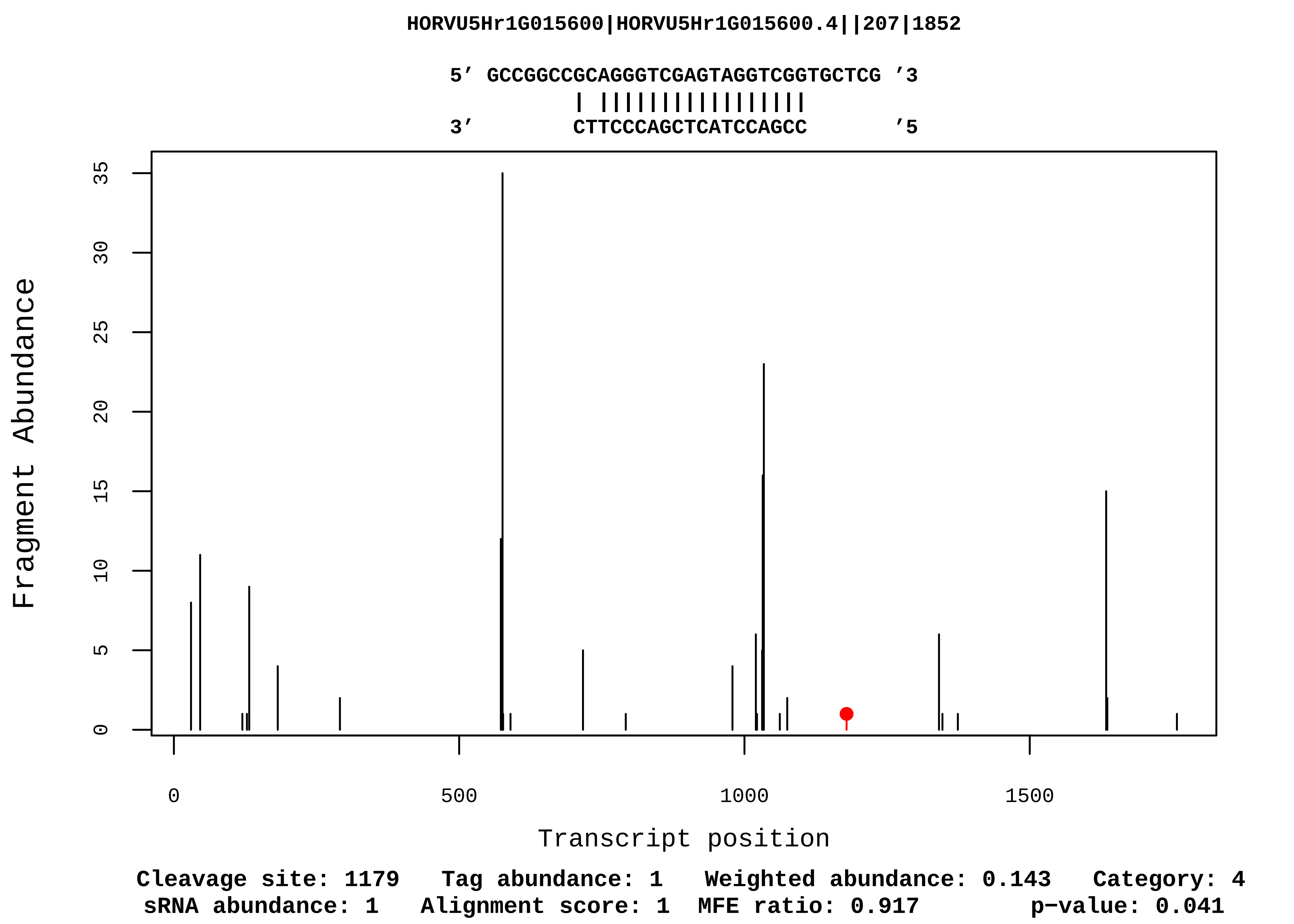

HORVU5Hr1G015600|HORVU5Hr1G015600.4||207|1852
5' GCCGGCCGCAGGGTCGAGTAGGTCGGTGCTCG '3
3' CTTCCCAGCTCATCCAGCC '5
Fragment Abundance
0
5
10
15
20
25
30
35
0
500
1000
1500
Transcript position
Cleavage site: 1179 Tag abundance: 1 Weighted abundance: 0.143 Category: 4
sRNA abundance: 1 Alignment score: 1 MFE ratio: 0.917 p-value: 0.041

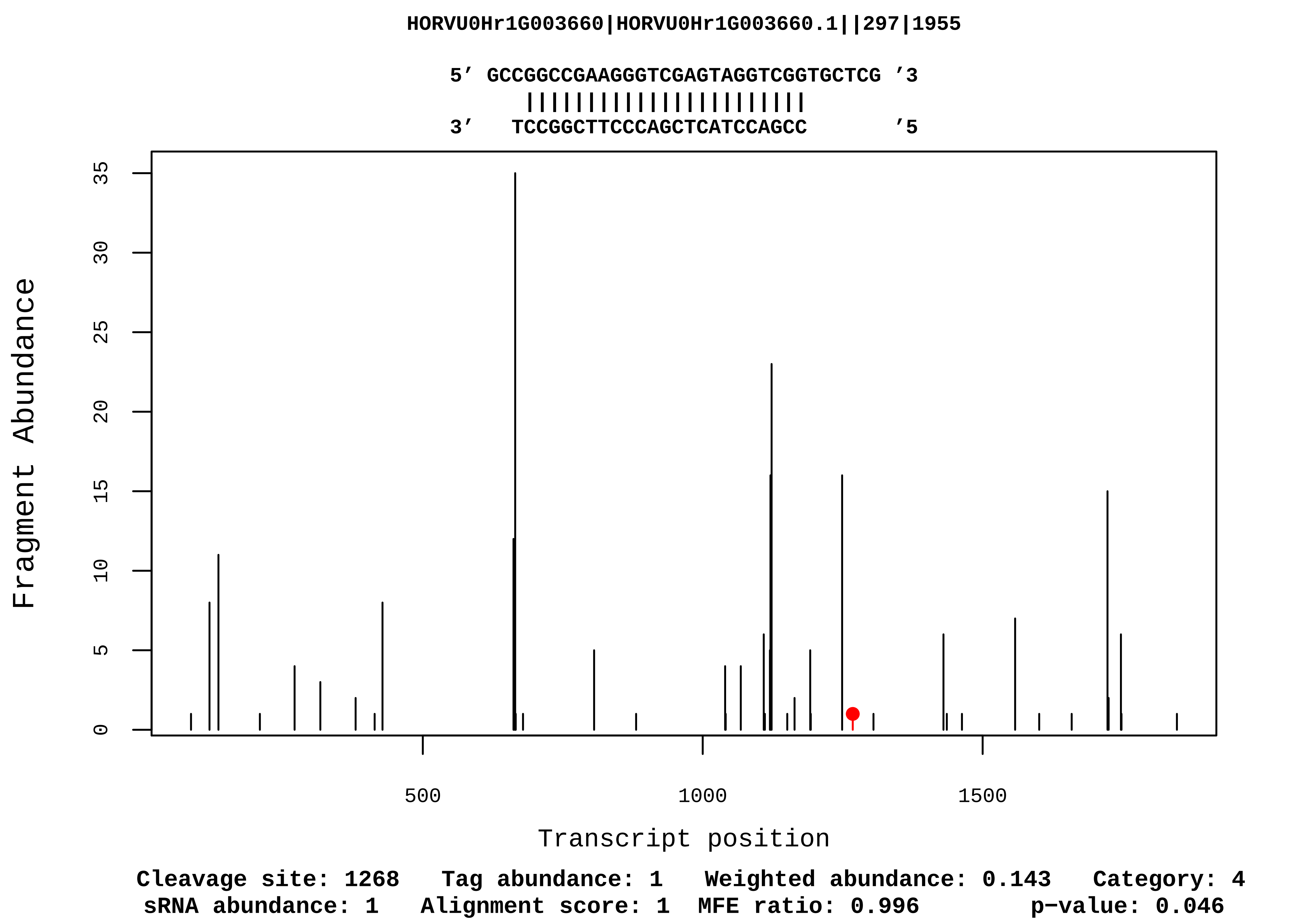

HORVU0Hr1G003660|HORVU0Hr1G003660.1||297|1955
5' GCCGGCCGAAGGGTCGAGTAGGTCGGTGCTCG '3
||||||||||||||||||||||
3' TCCGGCTTCCCAGCTCATCCAGCC '5
Fragment Abundance
0
5
10
15
20
25
30
35
500
1000
1500
Transcript position
Cleavage site: 1268 Tag abundance: 1 Weighted abundance: 0.143 Category: 4
sRNA abundance: 1 Alignment score: 1 MFE ratio: 0.996 p-value: 0.046

HORVU0Hr1G023930|HORVU0Hr1G023930.3||396|2180

```
5' GTCGGCGGAAGGGTCGAGTAGGTCGGTGCTCG '3
        ||| ||||||||||||||||||||
3'   TCCGGCTTCCCAGCTCATCCAGCC       '5
```

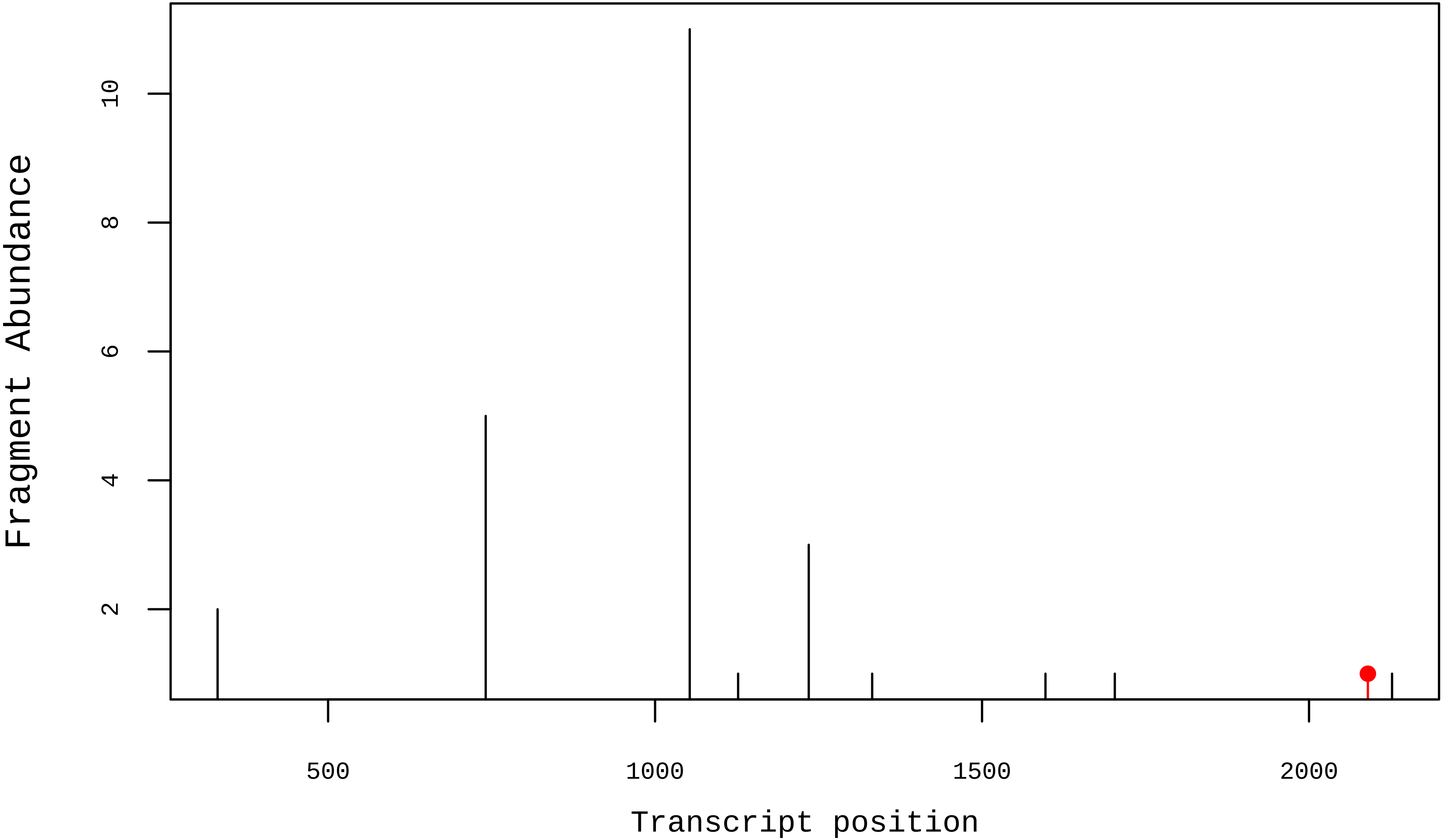

Cleavage site: 2090   Tag abundance: 1   Weighted abundance: 0.143   Category: 4
sRNA abundance: 1   Alignment score: 2   MFE ratio: 0.914   p-value: 0.019

HORVU2Hr1G072960|HORVU2Hr1G072960.1||600|3353

```
5' GCCGGCCGAAGGGTCGAGTAGGTCGGTGCTCG '3
       ||||||||||||||||||||||
3'   TCCGGCTTCCCAGCTCATCCAGCC       '5
```

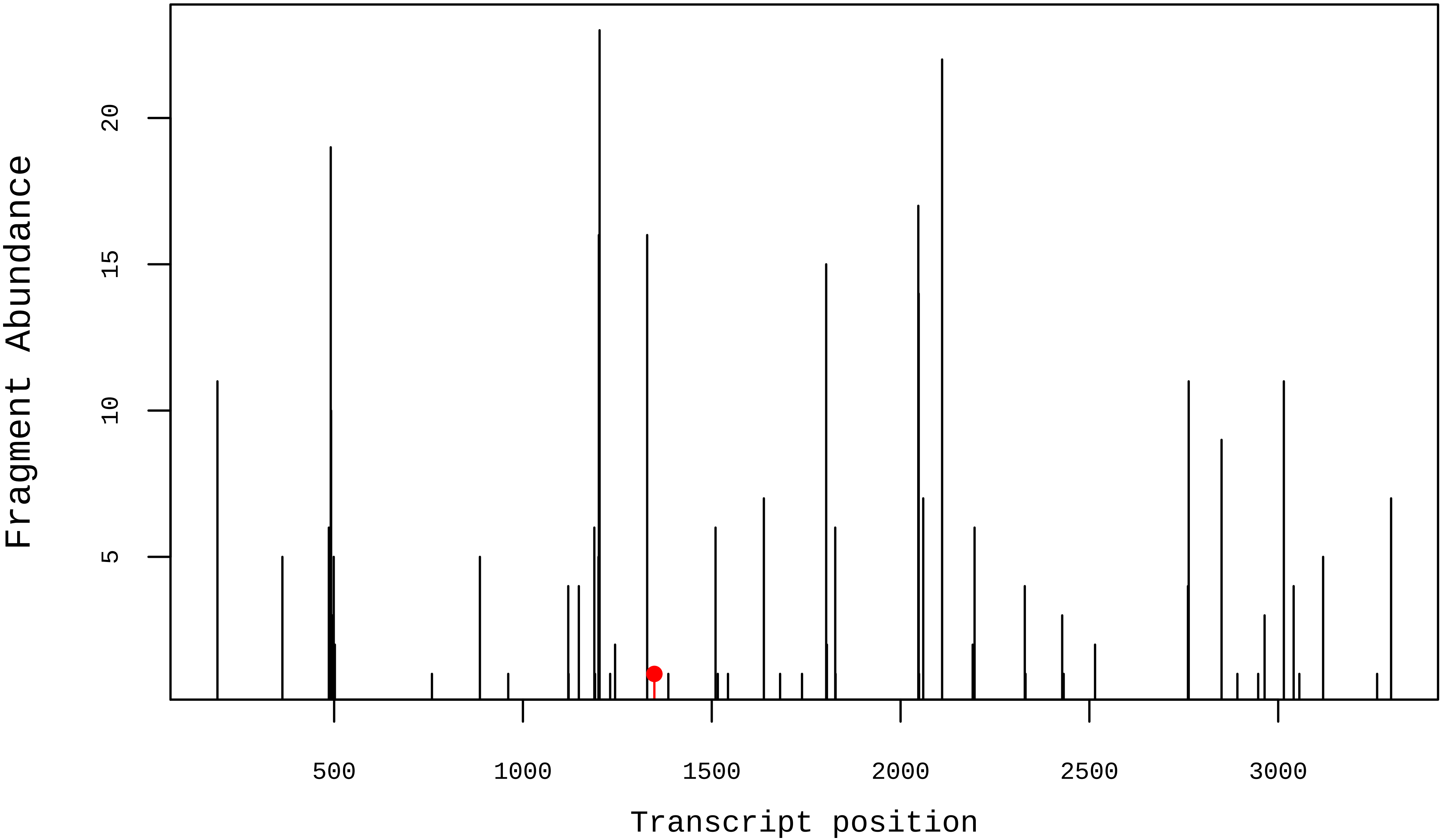

Cleavage site: 1348 Tag abundance: 1 Weighted abundance: 0.143 Category: 4
sRNA abundance: 1 Alignment score: 1 MFE ratio: 0.996 p-value: 0.03

HORVU5Hr1G015600|HORVU5Hr1G015600.1||156|510

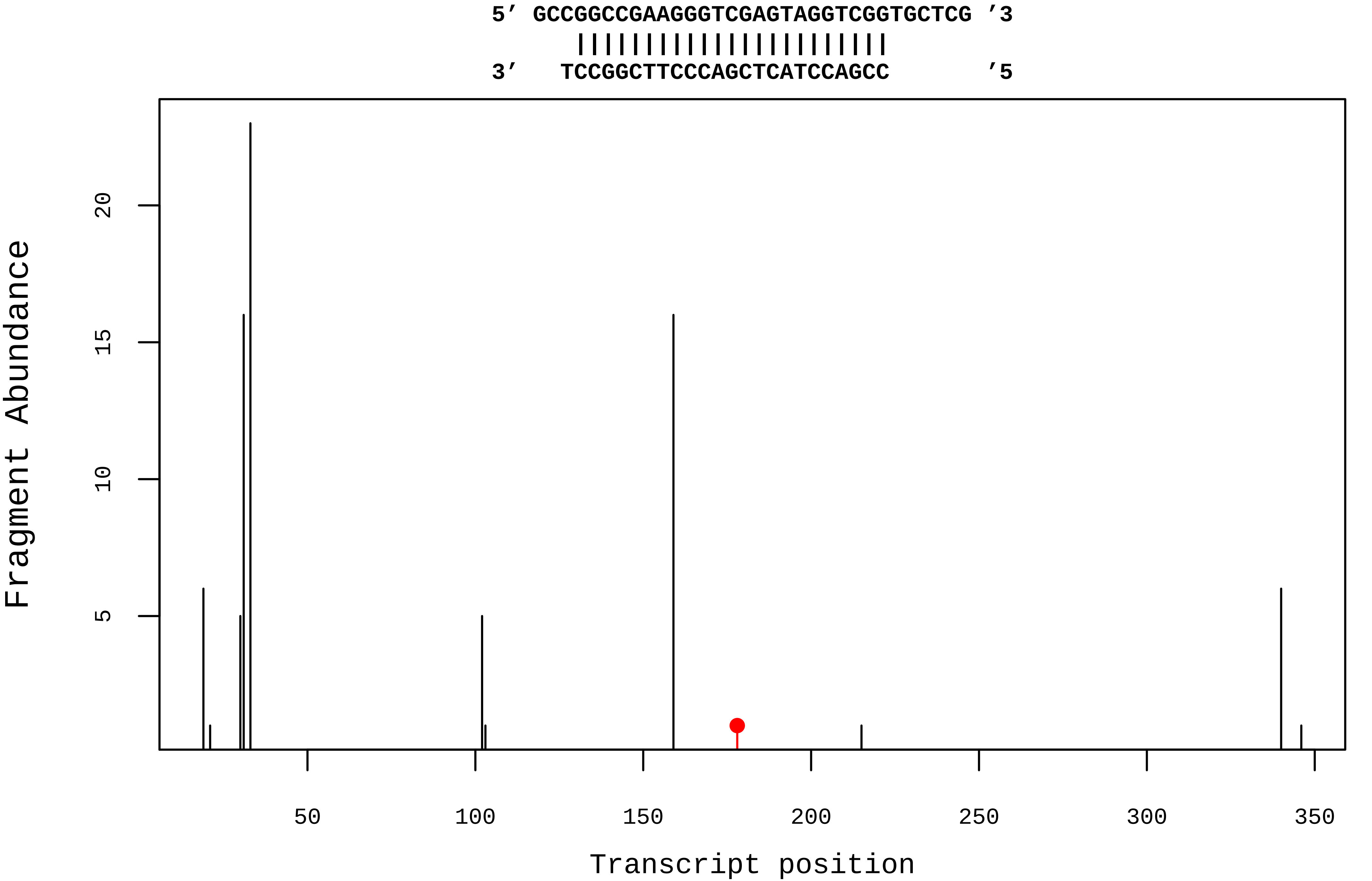

Cleavage site: 178 Tag abundance: 1 Weighted abundance: 0.143 Category: 4
sRNA abundance: 1 Alignment score: 1 MFE ratio: 0.996 p-value: 0.05

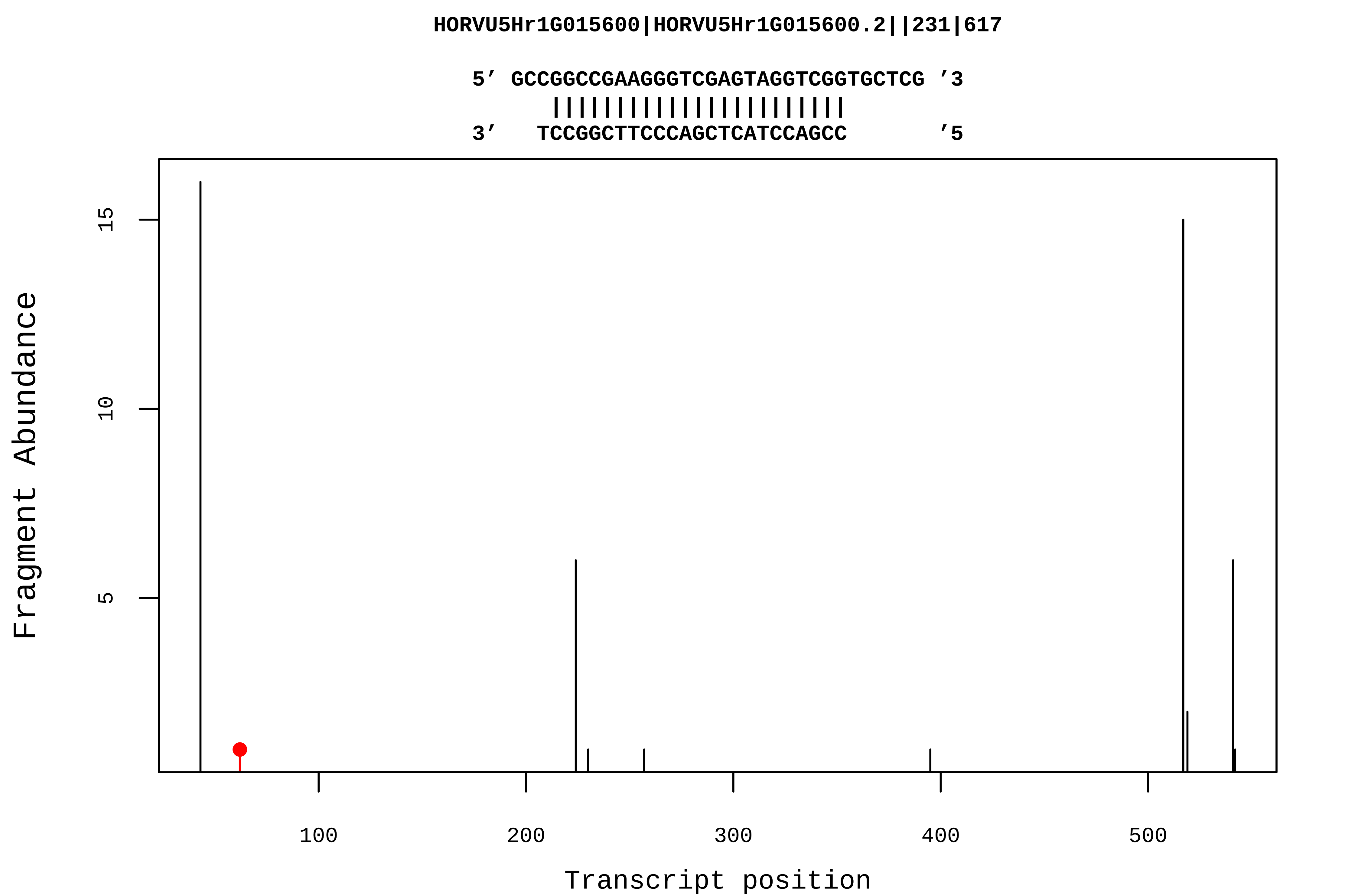

Cleavage site: 62 Tag abundance: 1 Weighted abundance: 0.143 Category: 4
sRNA abundance: 1 Alignment score: 1 MFE ratio: 0.996 p-value: 0.041

HORVU5Hr1G015600|HORVU5Hr1G015600.3||276|1709

```
5' GCCGGCCGAAGGGTCGAGTAGGTCGGTGCTCG '3
       |||||||||||||||||||||||
3'   TCCGGCTTCCCAGCTCATCCAGCC       '5
```

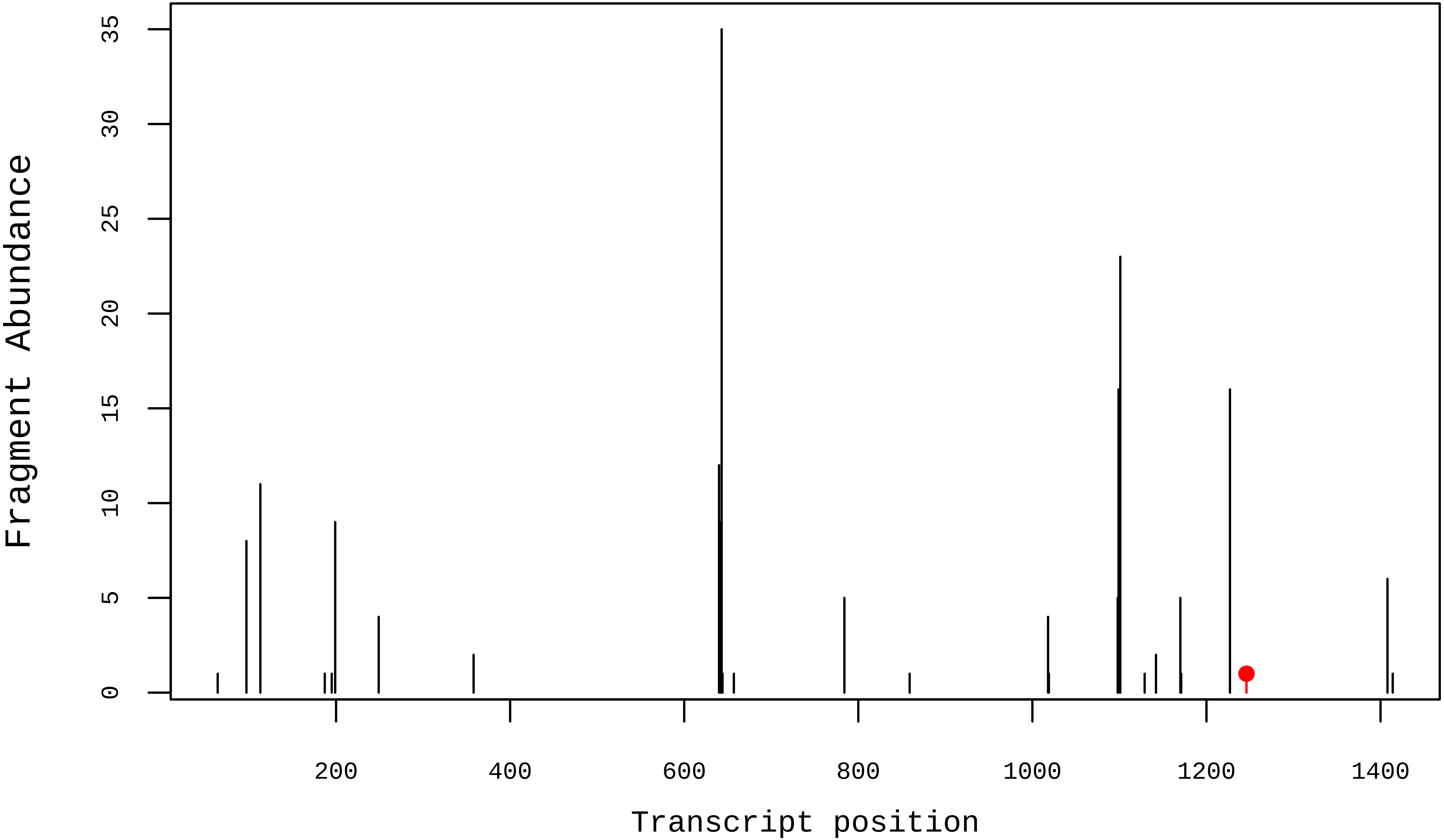

Cleavage site: 1246 Tag abundance: 1 Weighted abundance: 0.143 Category: 4
sRNA abundance: 1 Alignment score: 1 MFE ratio: 0.996 p-value: 0.032

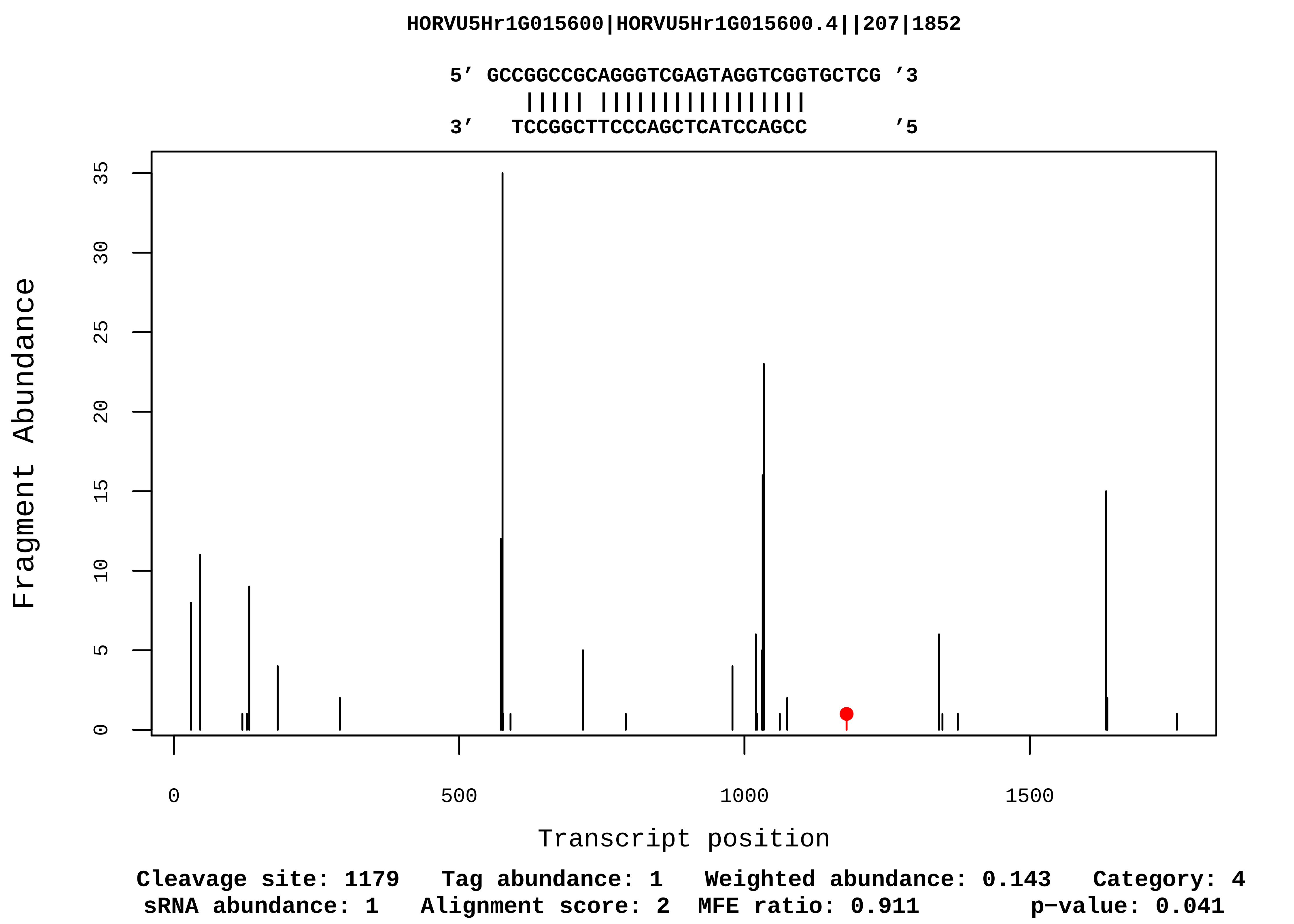

HORVU5Hr1G015600|HORVU5Hr1G015600.4||207|1852
5' GCCGGCCGCAGGGTCGAGTAGGTCGGTGCTCG '3
||||| |||||||||||||||||
3' TCCGGCTTCCCAGCTCATCCAGCC '5
Fragment Abundance
0
5
10
15
20
25
30
35
0
500
1000
1500
Transcript position
Cleavage site: 1179 Tag abundance: 1 Weighted abundance: 0.143 Category: 4
sRNA abundance: 1 Alignment score: 2 MFE ratio: 0.911 p-value: 0.041

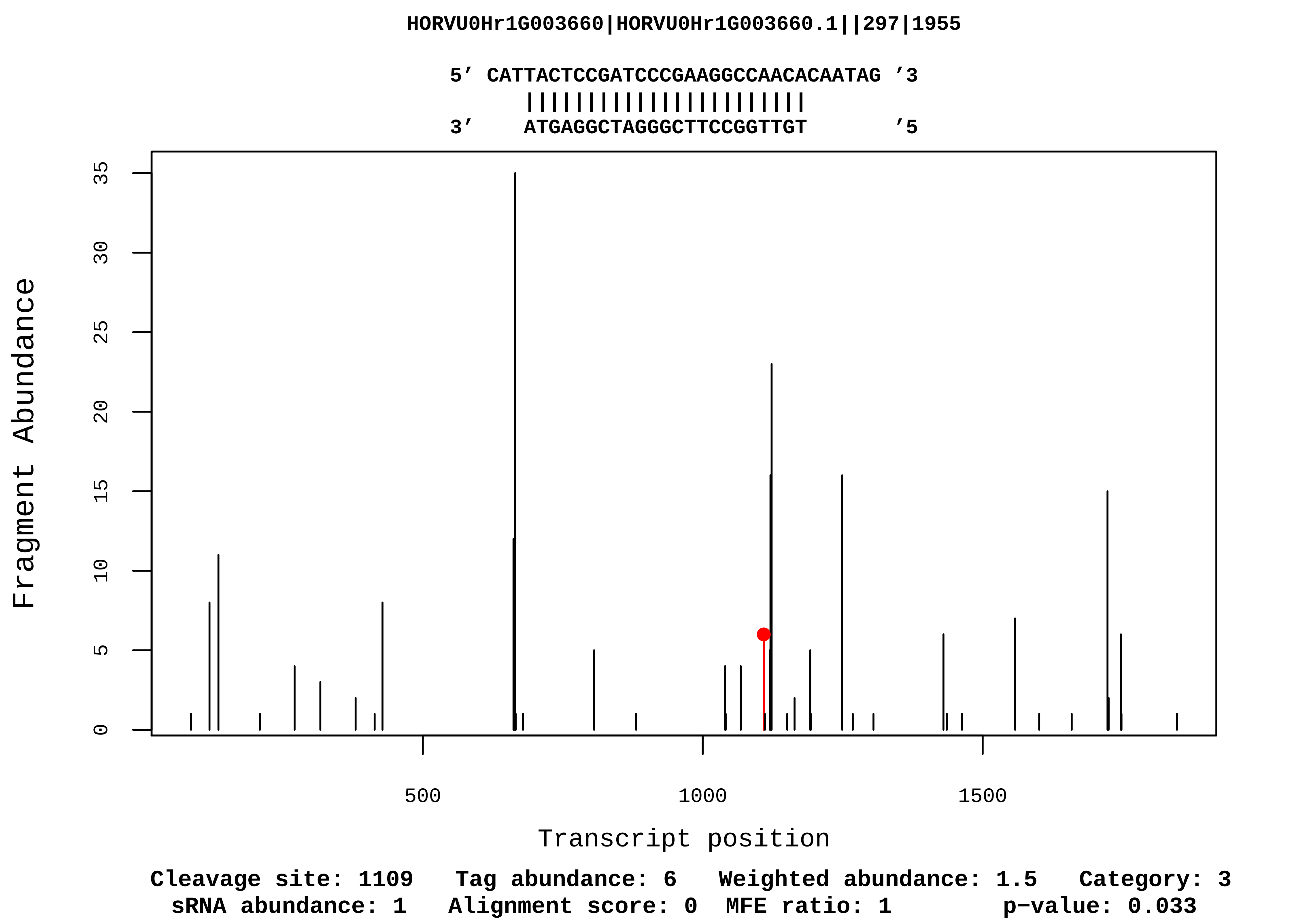
HORVU0Hr1G003660|HORVU0Hr1G003660.1||297|1955
5′ CATTACTCCGATCCCGAAGGCCAACACAATAG ′3
||||||||||||||||||||||
3′ ATGAGGCTAGGGCTTCCGGTTGT ′5
Fragment Abundance
0
5
10
15
20
25
30
35
500
1000
1500
Transcript position
Cleavage site: 1109 Tag abundance: 6 Weighted abundance: 1.5 Category: 3
sRNA abundance: 1 Alignment score: 0 MFE ratio: 1 p−value: 0.033

HORVU2Hr1G072960|HORVU2Hr1G072960.1||600|3353

```
5' CATTACTCCGATCCCGAAGGCCAACACAATAG '3
        ||||||||||||||||||||||
3'      ATGAGGCTAGGGCTTCCGGTTGT        '5
```

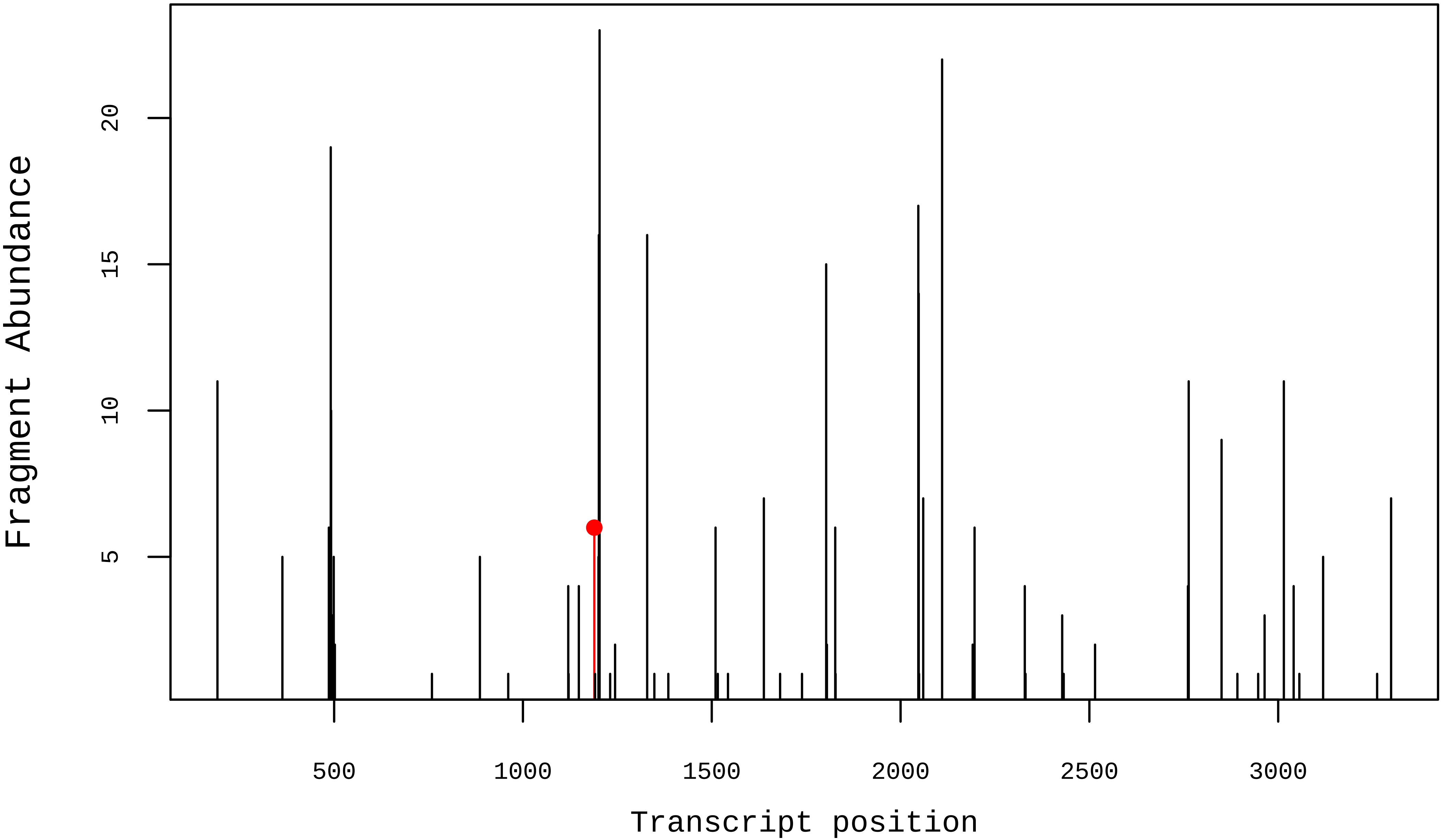

Cleavage site: 1189   Tag abundance: 6   Weighted abundance: 1.5   Category: 3
sRNA abundance: 1   Alignment score: 0   MFE ratio: 1   p-value: 0.031

HORVU5Hr1G015600|HORVU5Hr1G015600.1||156|510

```
5' CATTACTCCGATCCCGAAGGCCAACACAATAG '3
        |||||||||||||||||||||||
3'      ATGAGGCTAGGGCTTCCGGTTGT       '5
```

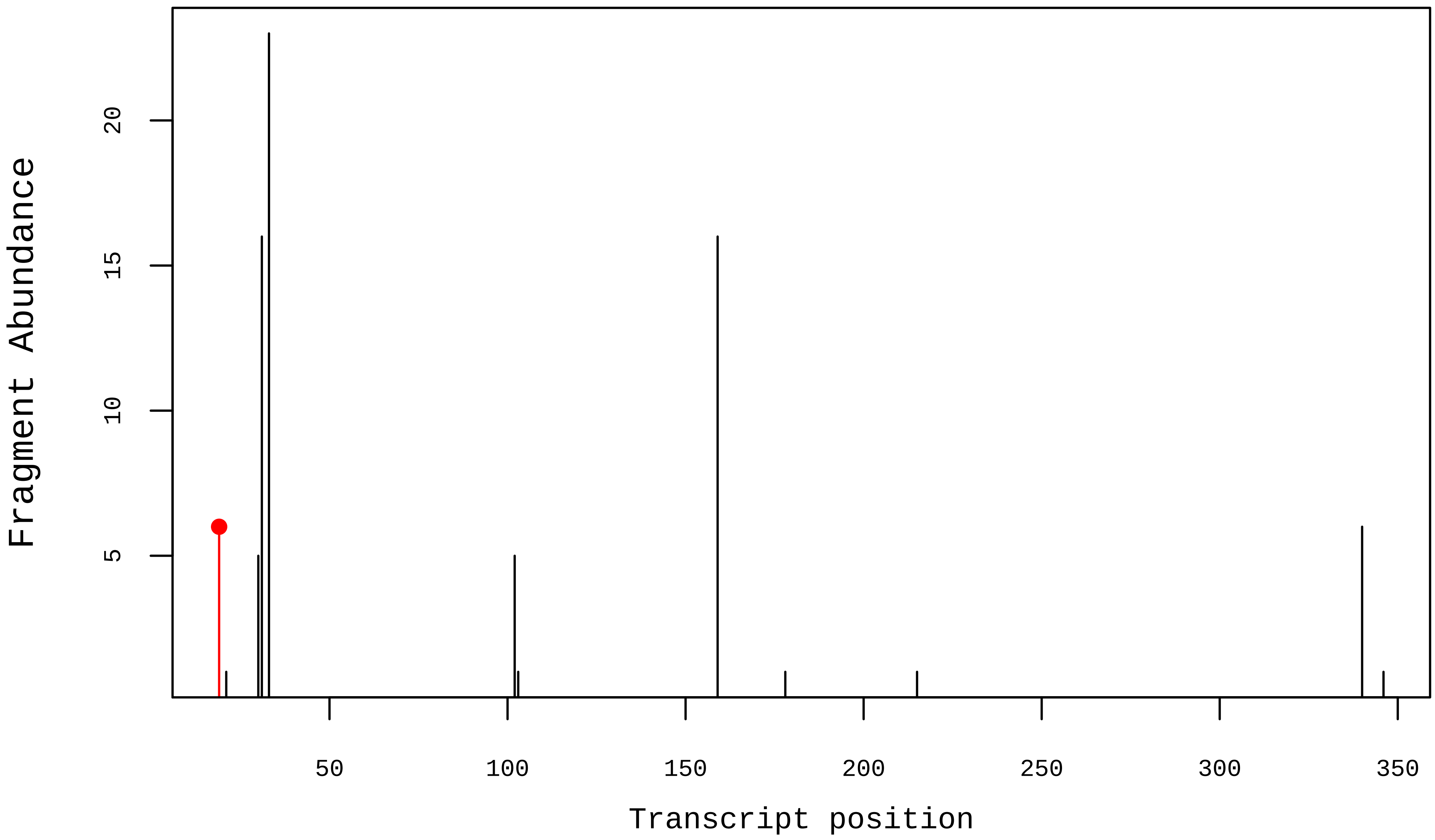

Cleavage site: 19   Tag abundance: 6   Weighted abundance: 1.5   Category: 3
sRNA abundance: 1   Alignment score: 0   MFE ratio: 1   p-value: 0.032

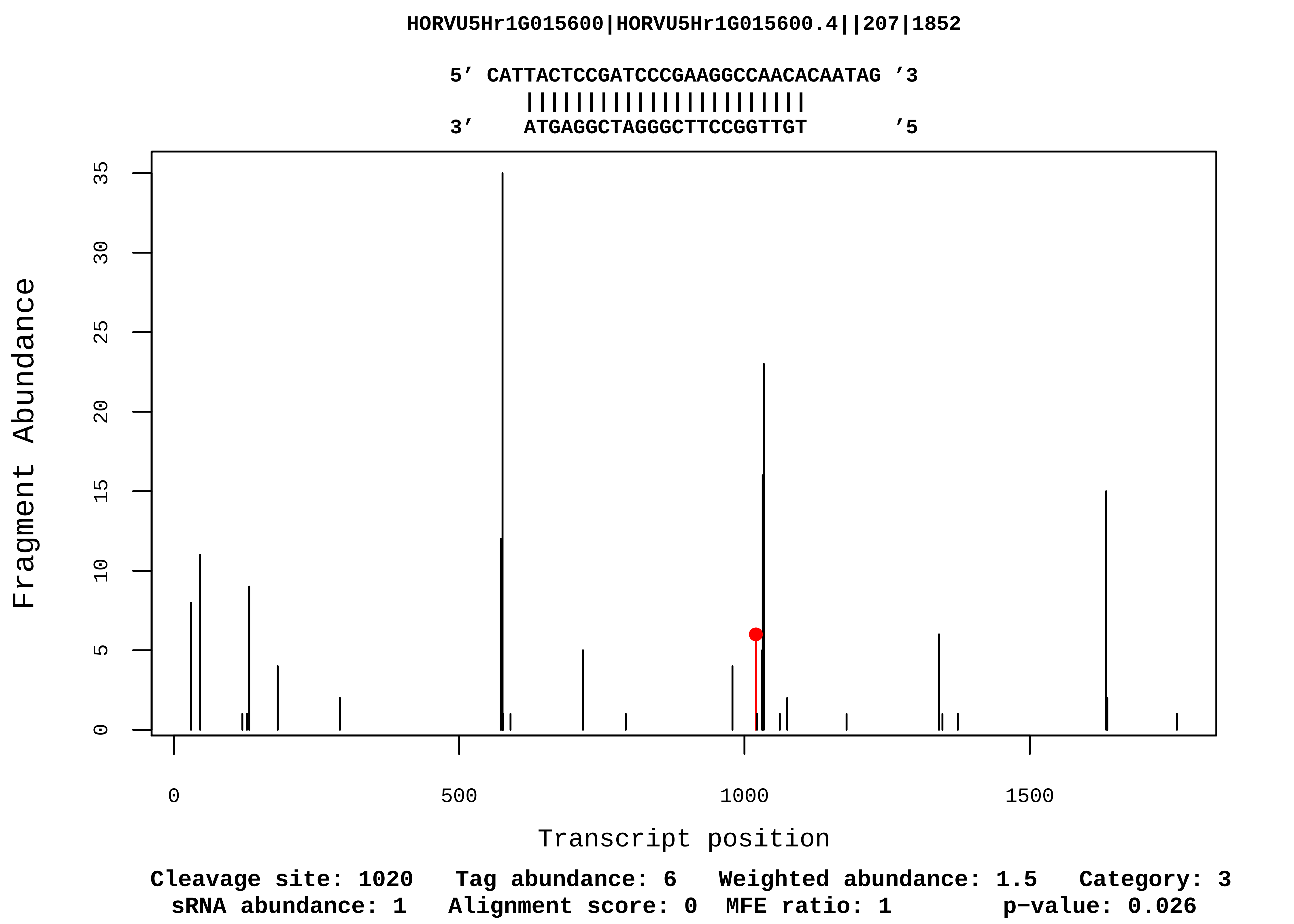
HORVU5Hr1G015600|HORVU5Hr1G015600.4||207|1852
5′ CATTACTCCGATCCCGAAGGCCAACACAATAG ′3
||||||||||||||||||||||
3′ ATGAGGCTAGGGCTTCCGGTTGT ′5
Fragment Abundance
0
5
10
15
20
25
30
35
0
500
1000
1500
Transcript position
Cleavage site: 1020 Tag abundance: 6 Weighted abundance: 1.5 Category: 3
sRNA abundance: 1 Alignment score: 0 MFE ratio: 1 p−value: 0.026

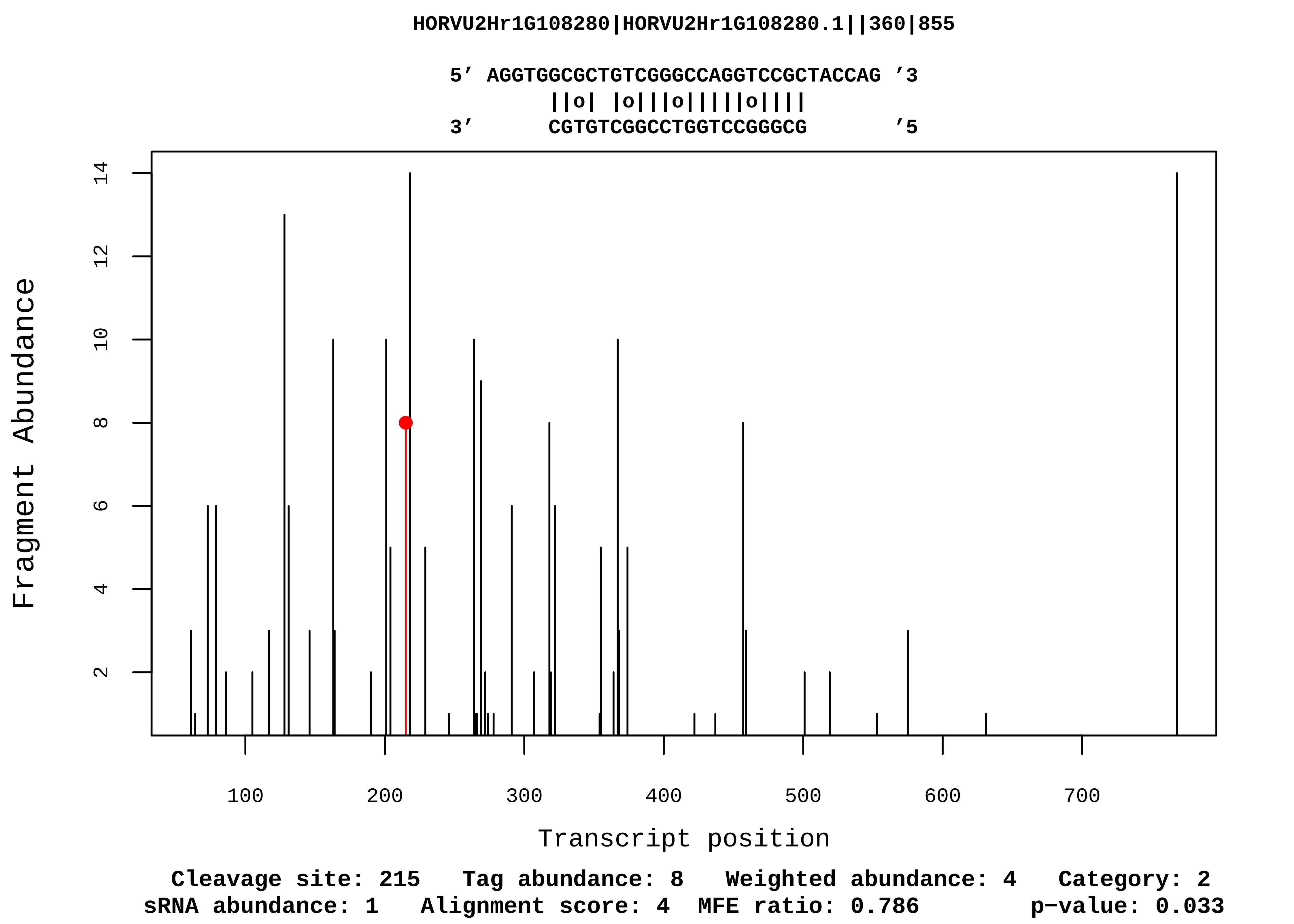
HORVU2Hr1G108280|HORVU2Hr1G108280.1||360|855
5' AGGTGGCGCTGTCGGGCCAGGTCCGCTACCAG '3
||o| |o|||o|||||o||||
3' CGTGTCGGCCTGGTCCGGGCG '5
Fragment Abundance
2
4
6
8
10
12
14
100
200
300
400
500
600
700
Transcript position
Cleavage site: 215 Tag abundance: 8 Weighted abundance: 4 Category: 2
sRNA abundance: 1 Alignment score: 4 MFE ratio: 0.786 p-value: 0.033

HORVU2Hr1G108350|HORVU2Hr1G108350.1||666|1835

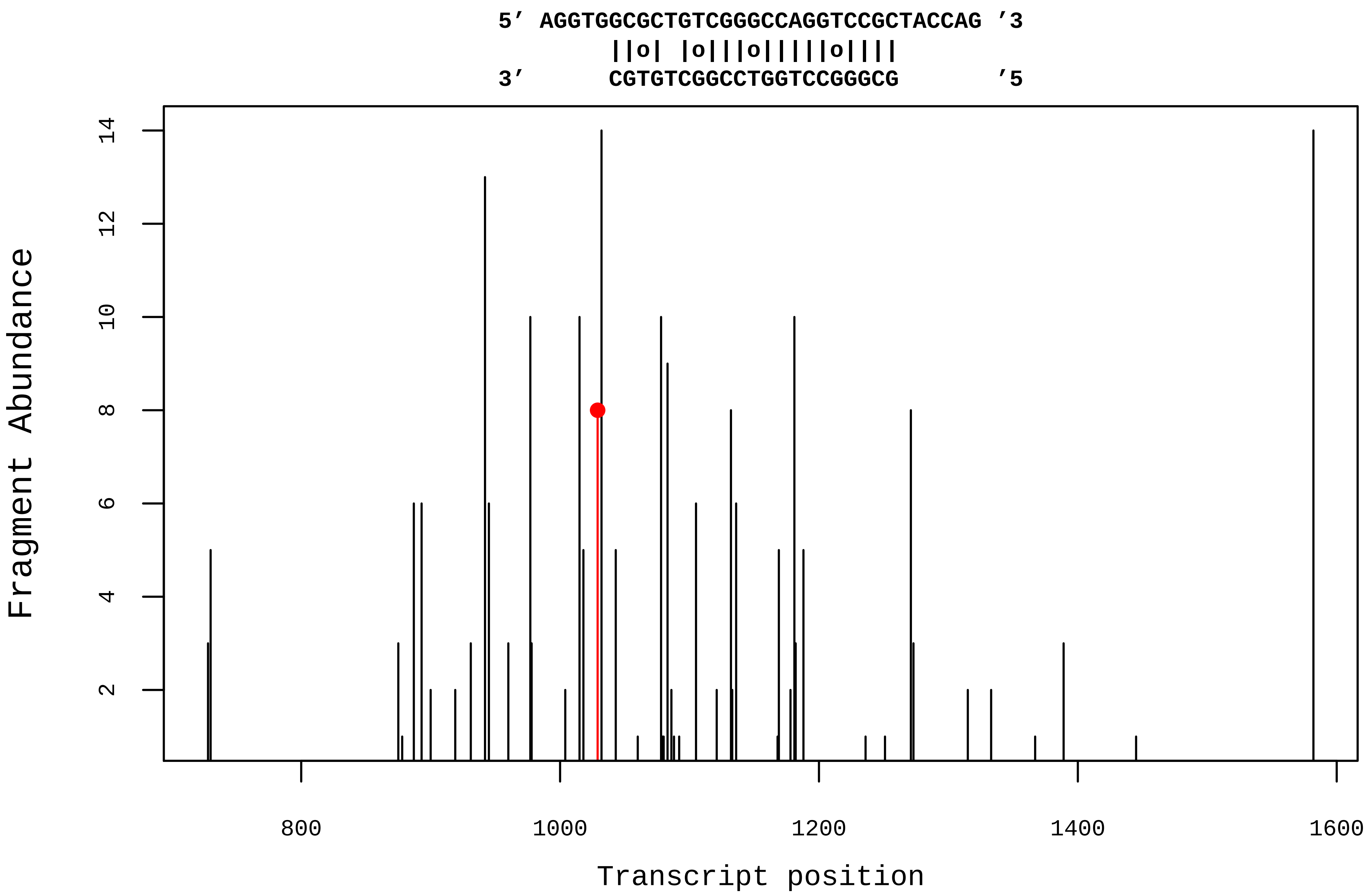

Cleavage site: 1029 Tag abundance: 8 Weighted abundance: 4 Category: 2
sRNA abundance: 1 Alignment score: 4 MFE ratio: 0.786 p-value: 0.015

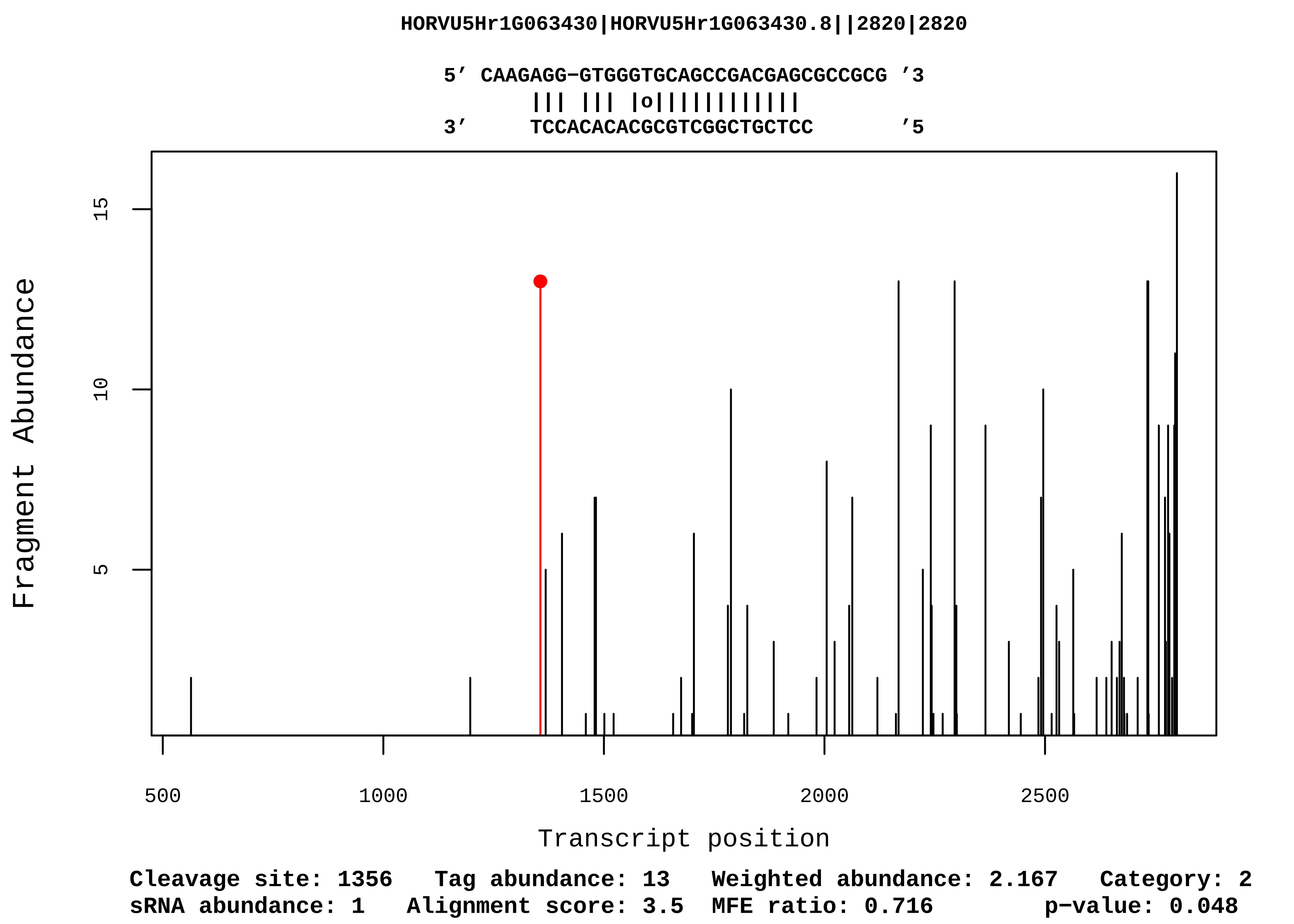
HORVU5Hr1G063430|HORVU5Hr1G063430.8||2820|2820
5' CAAGAGG-GTGGGTGCAGCCGACGAGCGCCGCG '3
||| ||| |o||||||||||||
3' TCCACACACGCGTCGGCTGCTCC '5
Fragment Abundance
15
10
5
500
1000
1500
2000
2500
Transcript position
Cleavage site: 1356 Tag abundance: 13 Weighted abundance: 2.167 Category: 2
sRNA abundance: 1 Alignment score: 3.5 MFE ratio: 0.716 p-value: 0.048

HORVU5Hr1G063430|HORVU5Hr1G063430.9||1848|1850

```
5' CAAGAGG-GTGGGTGCAGCCGACGAGCGCCGCG '3
         ||| ||| |o|||||||||||
3'       TCCACACACGCGTCGGCTGCTCC       '5
```

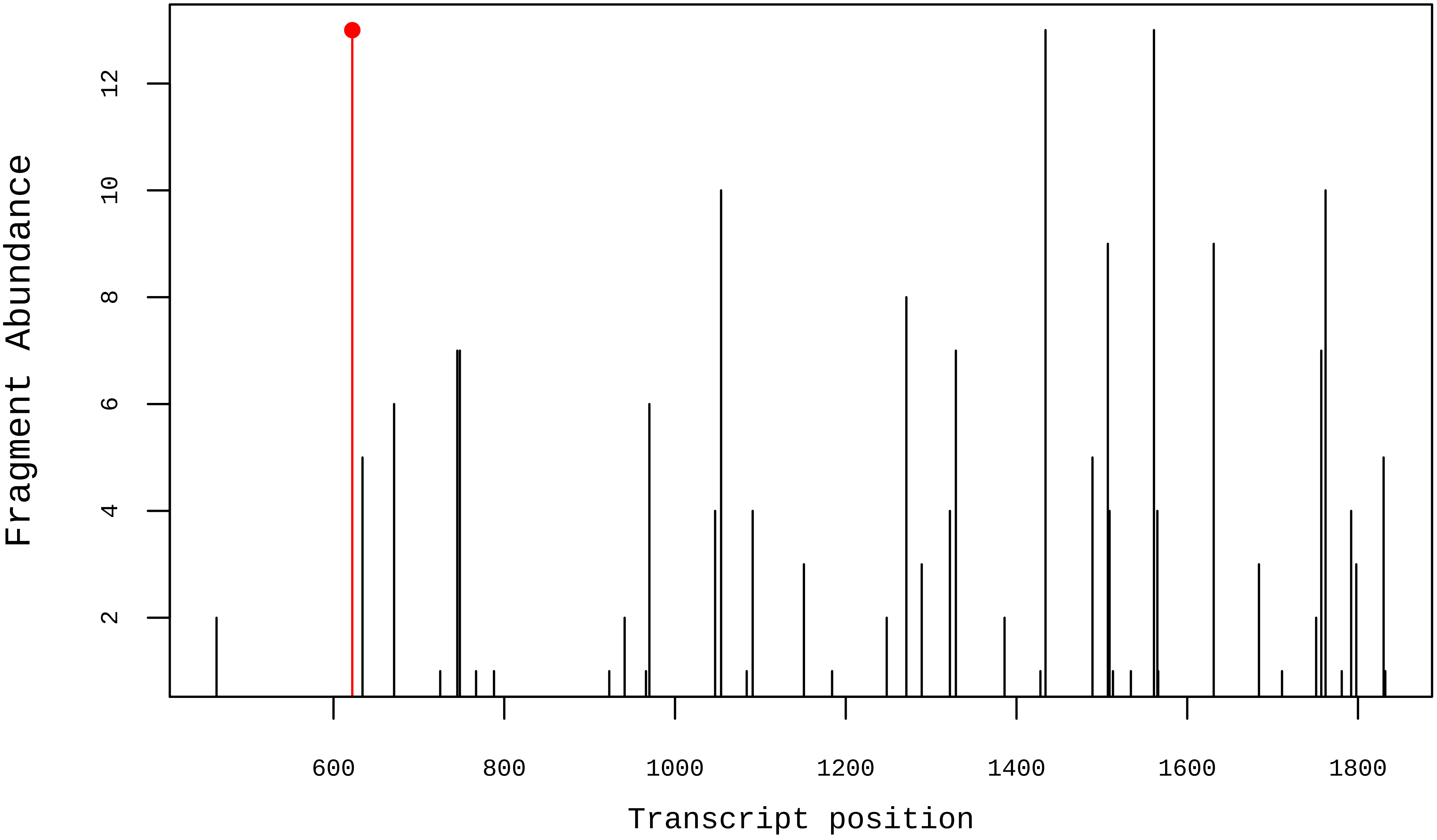

Cleavage site: 622 Tag abundance: 13 Weighted abundance: 2.167 Category: 1
sRNA abundance: 1 Alignment score: 3.5 MFE ratio: 0.716 p-value: 0.01

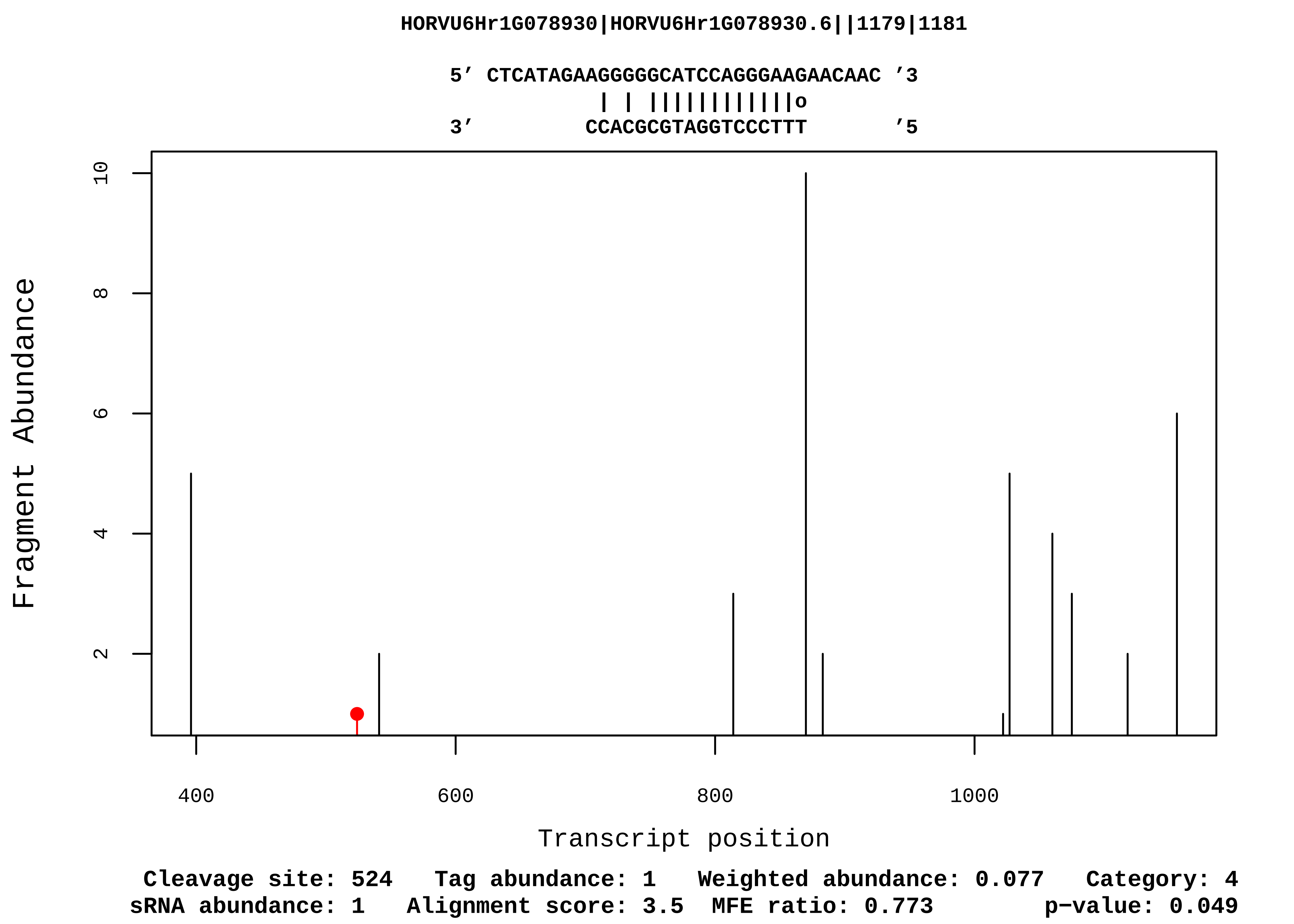
HORVU6Hr1G078930|HORVU6Hr1G078930.6||1179|1181
5′ CTCATAGAAGGGGGCATCCAGGGAAGAACAAC ′3
| | |||||||||||o
3′ CCACGCGTAGGTCCCTTT ′5
Fragment Abundance
2
4
6
8
10
400
600
800
1000
Transcript position
Cleavage site: 524 Tag abundance: 1 Weighted abundance: 0.077 Category: 4
sRNA abundance: 1 Alignment score: 3.5 MFE ratio: 0.773 p-value: 0.049

HORVU0Hr1G023930|HORVU0Hr1G023930.3||396|2180

```
5' GTCGGCGGAAGGGTCGAGTAGGTCGGTGCTCG '3
      |||| | ||||||||||||||||||
3'    GCCGGCATCCCAGCTCATCCAGCC       '5
```

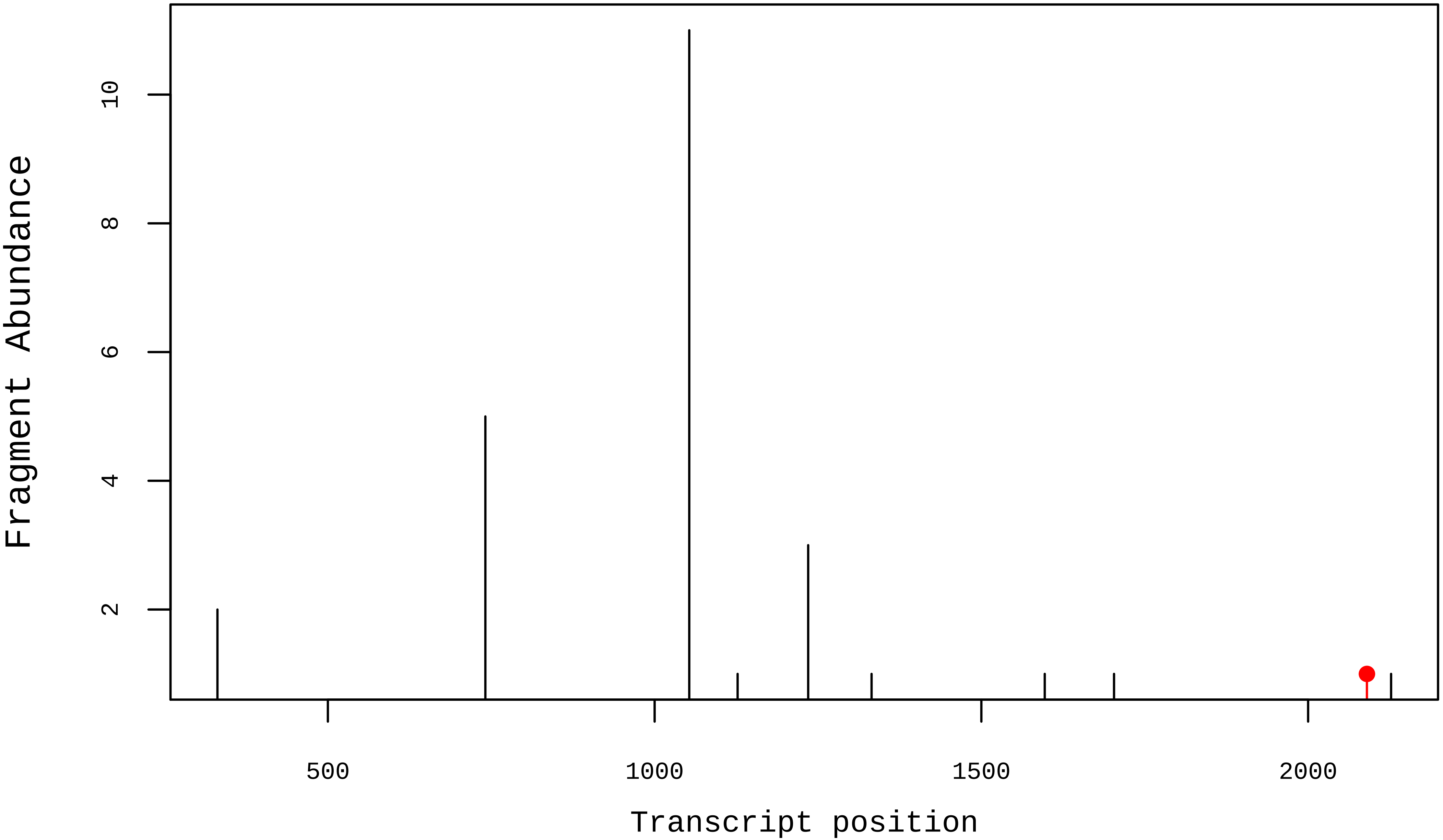

Cleavage site: 2090   Tag abundance: 1   Weighted abundance: 0.143   Category: 4
sRNA abundance: 1   Alignment score: 2   MFE ratio: 0.832   p-value: 0.019

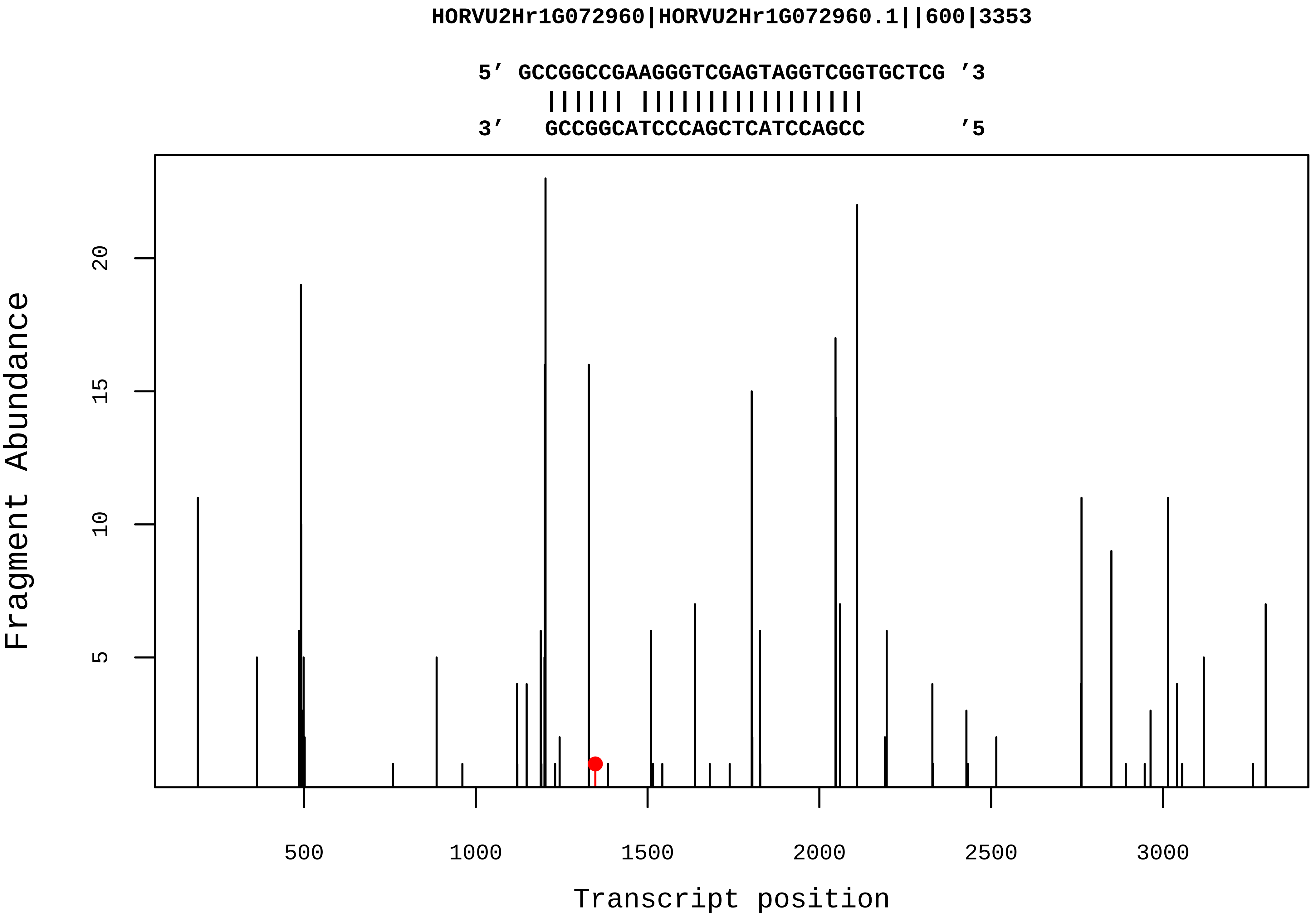

Cleavage site: 1348 Tag abundance: 1 Weighted abundance: 0.143 Category: 4
sRNA abundance: 1 Alignment score: 1 MFE ratio: 0.912 p-value: 0.035

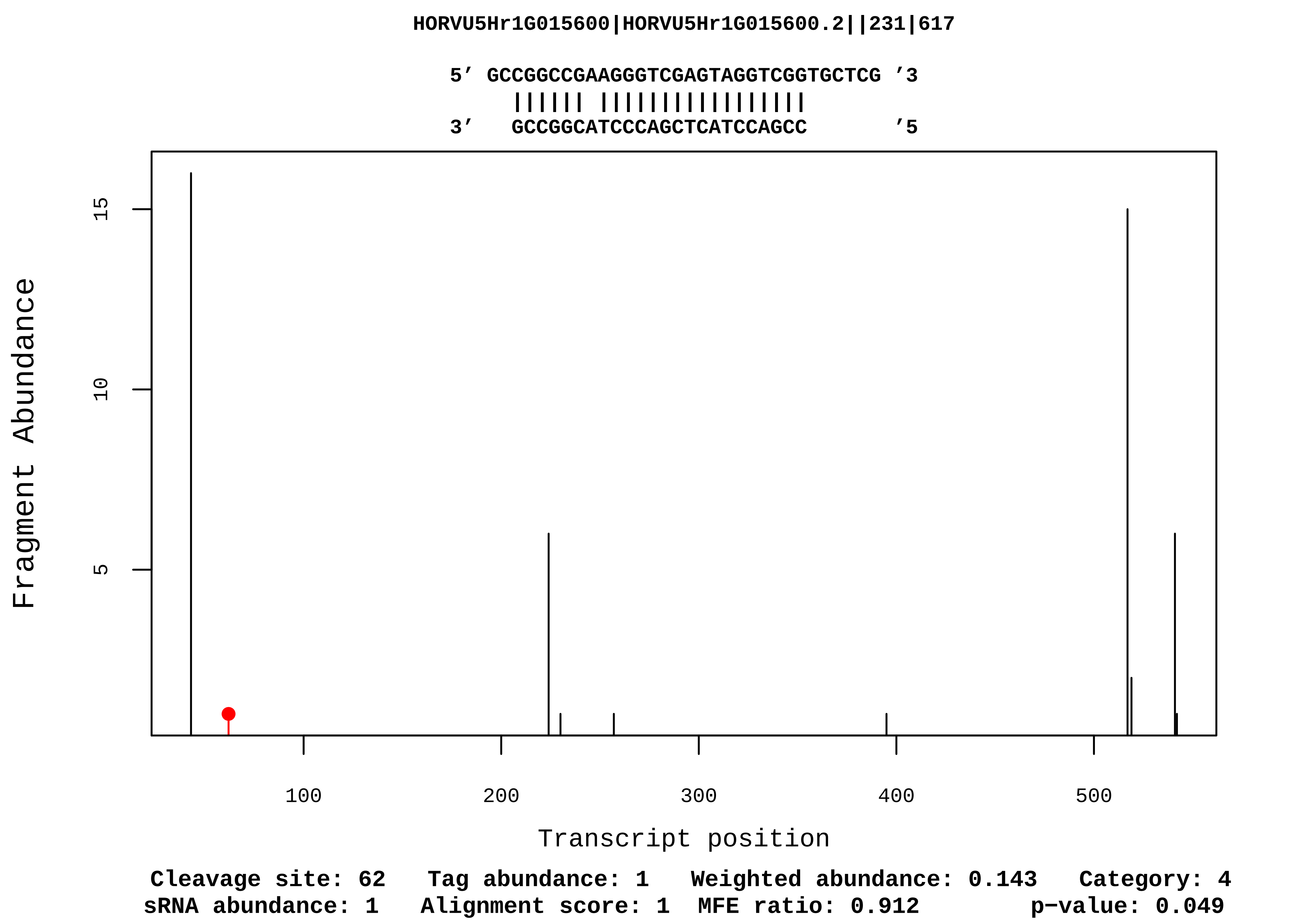

HORVU5Hr1G015600|HORVU5Hr1G015600.2||231|617
5' GCCGGCCGAAGGGTCGAGTAGGTCGGTGCTCG '3
|||||| |||||||||||||||||
3' GCCGGCATCCCAGCTCATCCAGCC '5
Fragment Abundance
5
10
15
100
200
300
400
500
Transcript position
Cleavage site: 62 Tag abundance: 1 Weighted abundance: 0.143 Category: 4
sRNA abundance: 1 Alignment score: 1 MFE ratio: 0.912 p-value: 0.049

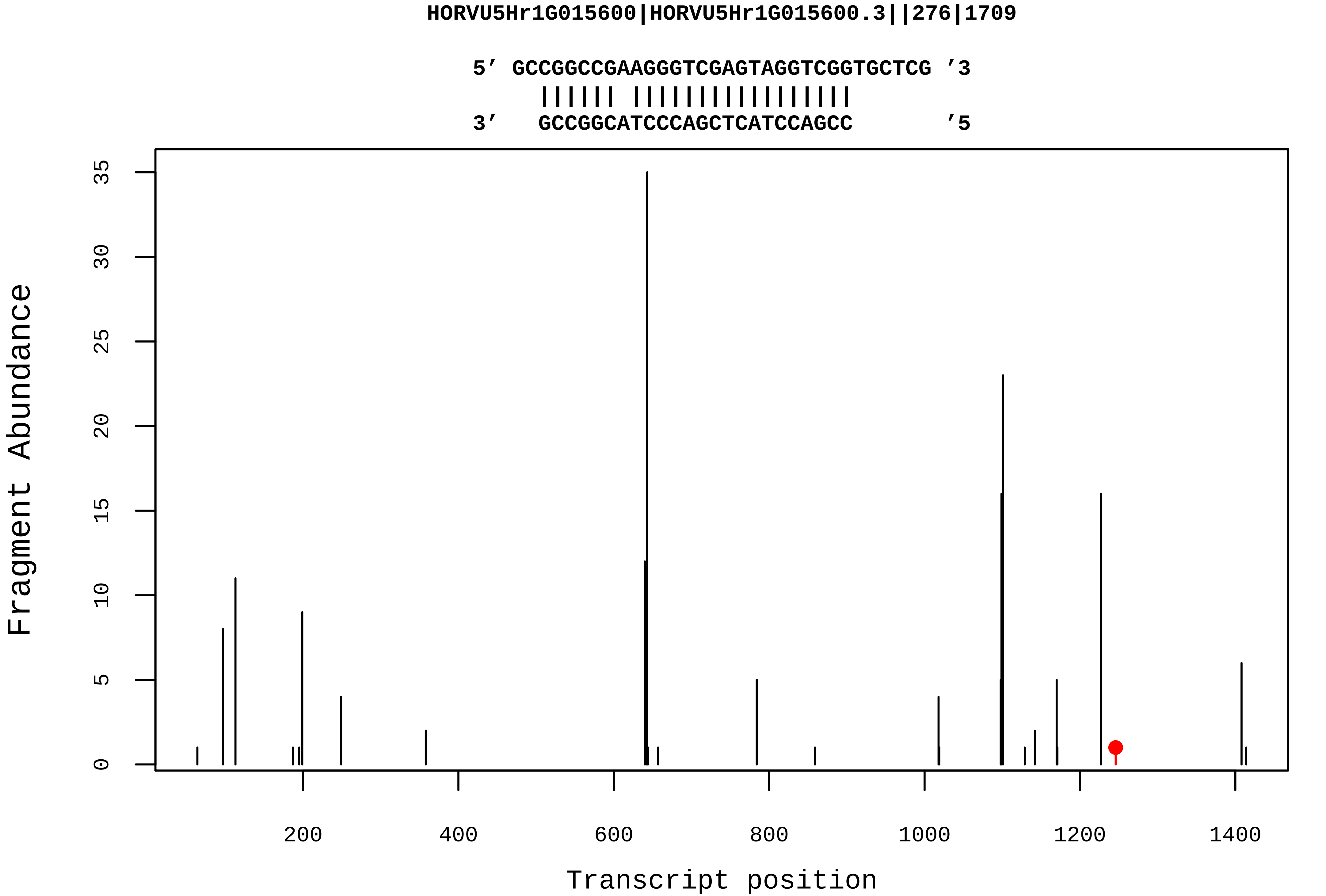

Cleavage site: 1246 Tag abundance: 1 Weighted abundance: 0.143 Category: 4
sRNA abundance: 1 Alignment score: 1 MFE ratio: 0.912 p-value: 0.038

HORVU5Hr1G015600|HORVU5Hr1G015600.4||207|1852

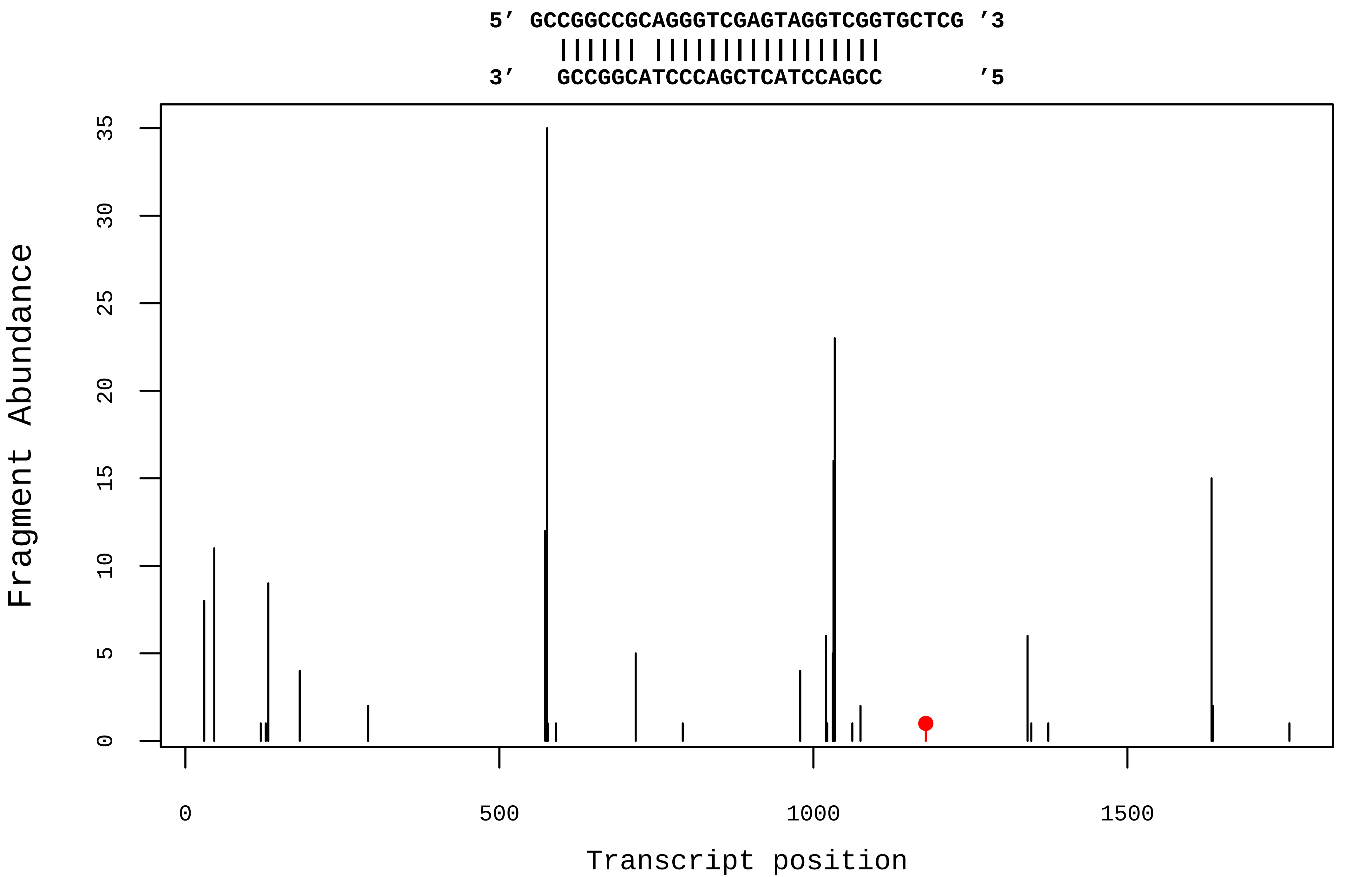

Cleavage site: 1179   Tag abundance: 1   Weighted abundance: 0.143   Category: 4
sRNA abundance: 1   Alignment score: 1   MFE ratio: 0.912   p-value: 0.035

HORVU0Hr1G023930|HORVU0Hr1G023930.3||396|2180

```
5' GTCGGCGGAAG-GGTCGAGTAGGTCGGTGCTCG '3
         |||  | |||||||||||||||
3'       GCCGGCTCCAGCTCATCCAGCC       '5
```

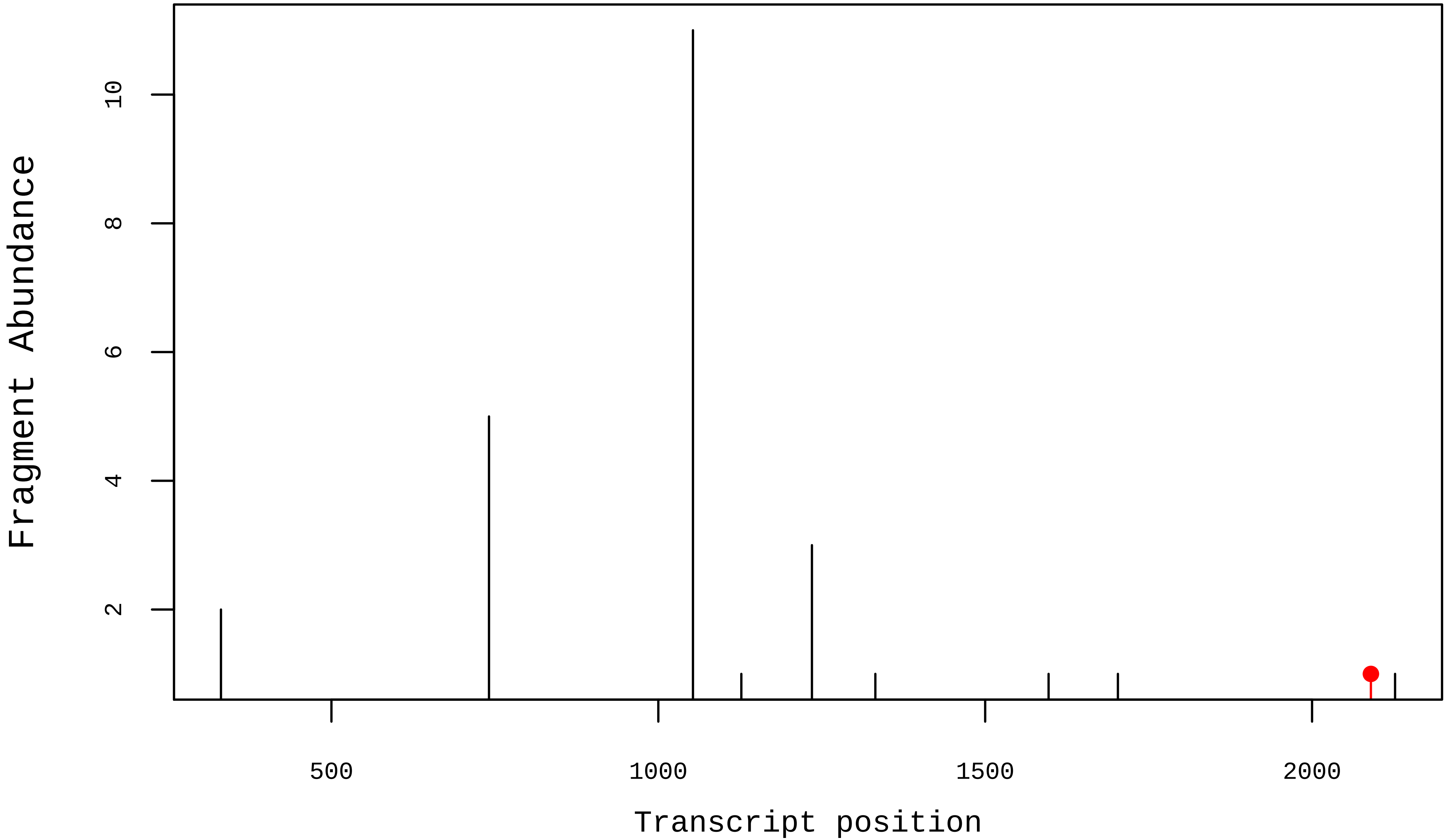

Cleavage site: 2090 Tag abundance: 1 Weighted abundance: 0.143 Category: 4
sRNA abundance: 1 Alignment score: 3 MFE ratio: 0.724 p-value: 0.003

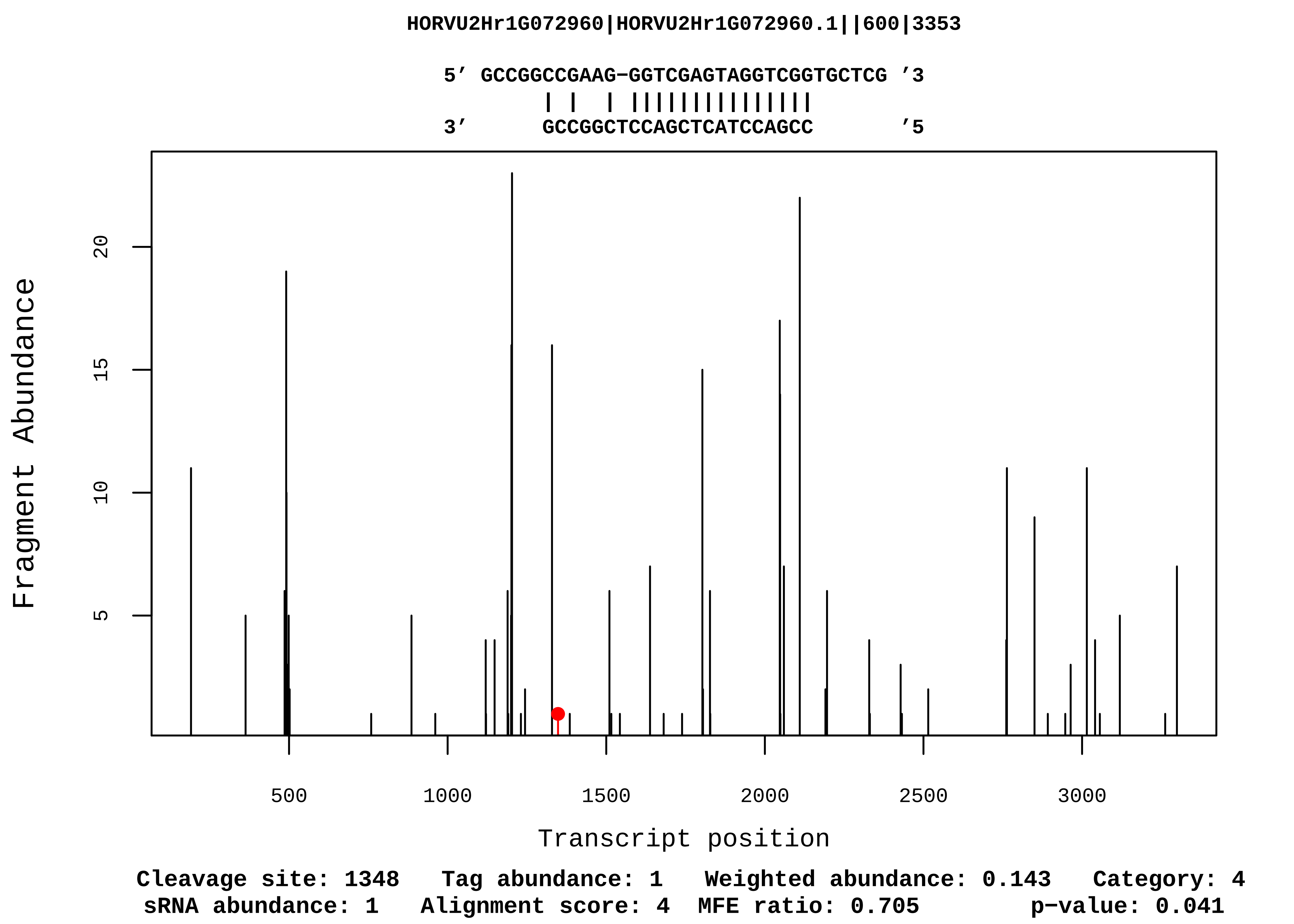
HORVU2Hr1G072960|HORVU2Hr1G072960.1||600|3353
5′ GCCGGCCGAAG−GGTCGAGTAGGTCGGTGCTCG ′3
3′ GCCGGCTCCAGCTCATCCAGCC ′5
Fragment Abundance
5
10
15
20
500
1000
1500
2000
2500
3000
Transcript position
Cleavage site: 1348 Tag abundance: 1 Weighted abundance: 0.143 Category: 4
sRNA abundance: 1 Alignment score: 4 MFE ratio: 0.705 p-value: 0.041

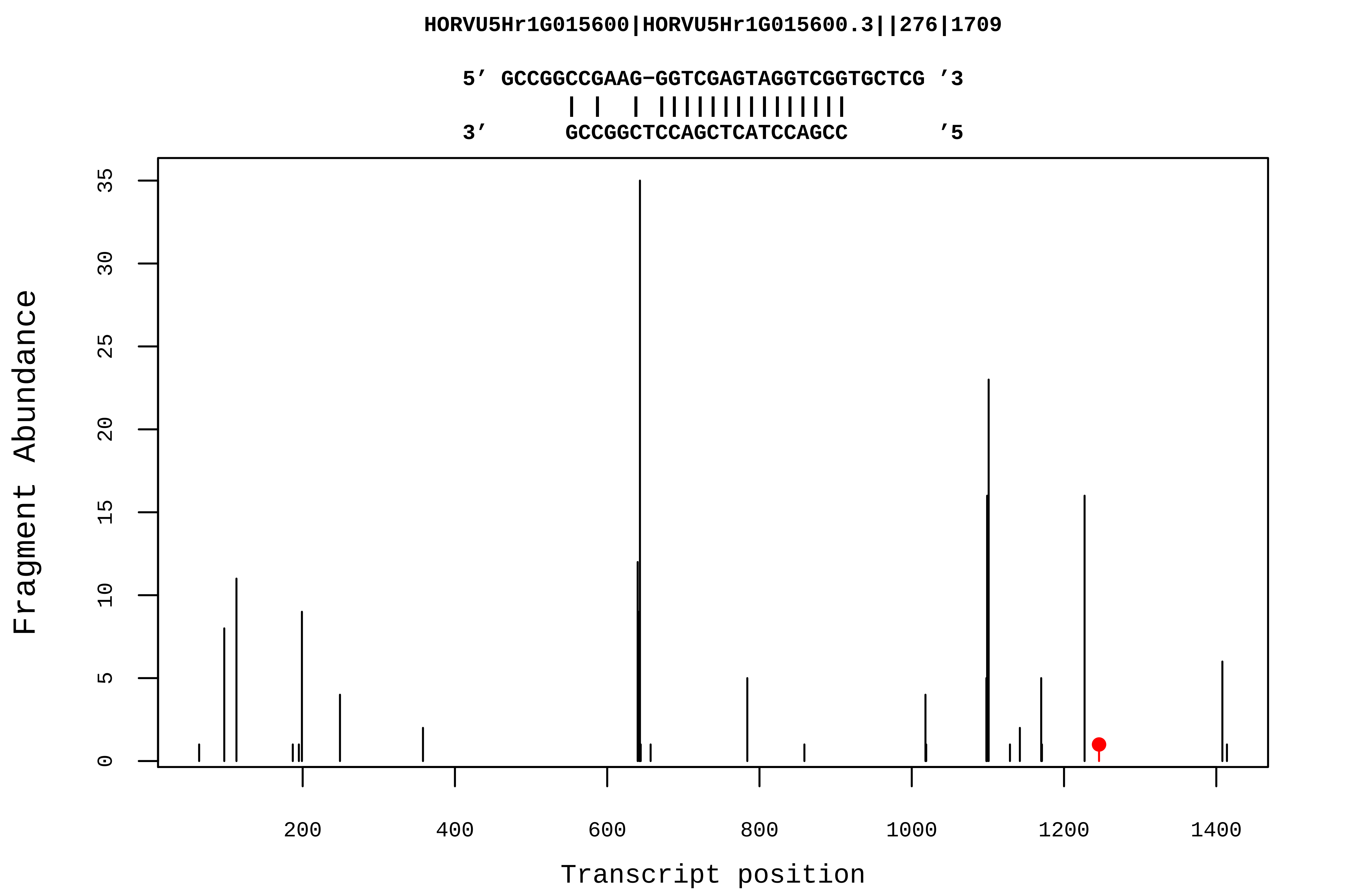

Cleavage site: 1246 Tag abundance: 1 Weighted abundance: 0.143 Category: 4
sRNA abundance: 1 Alignment score: 4 MFE ratio: 0.705 p-value: 0.045

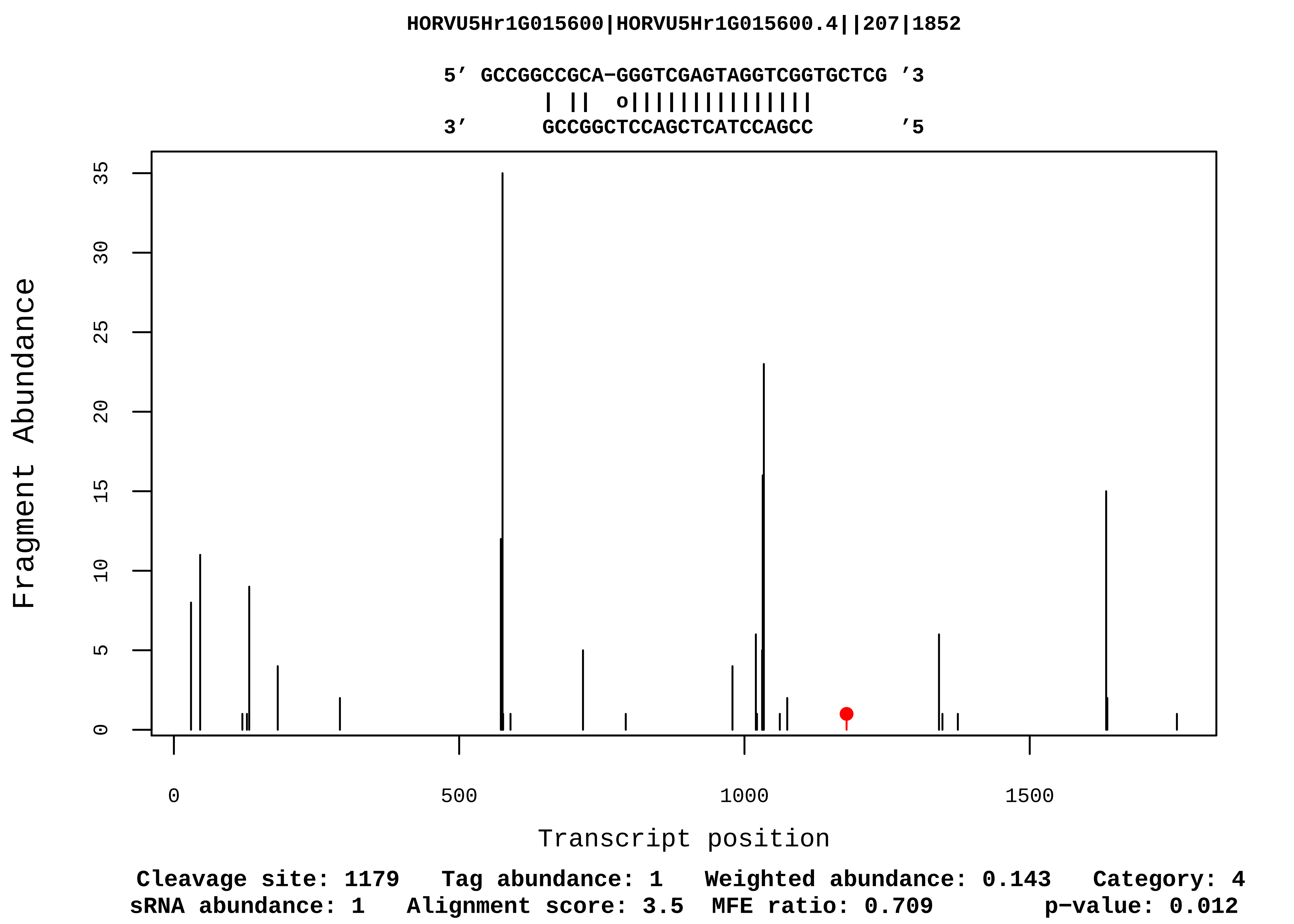
HORVU5Hr1G015600|HORVU5Hr1G015600.4||207|1852
5' GCCGGCCGCA-GGGTCGAGTAGGTCGGTGCTCG '3
| || o||||||||||||||
3' GCCGGCTCCAGCTCATCCAGCC '5
Fragment Abundance
0
5
10
15
20
25
30
35
0
500
1000
1500
Transcript position
Cleavage site: 1179 Tag abundance: 1 Weighted abundance: 0.143 Category: 4
sRNA abundance: 1 Alignment score: 3.5 MFE ratio: 0.709 p-value: 0.012

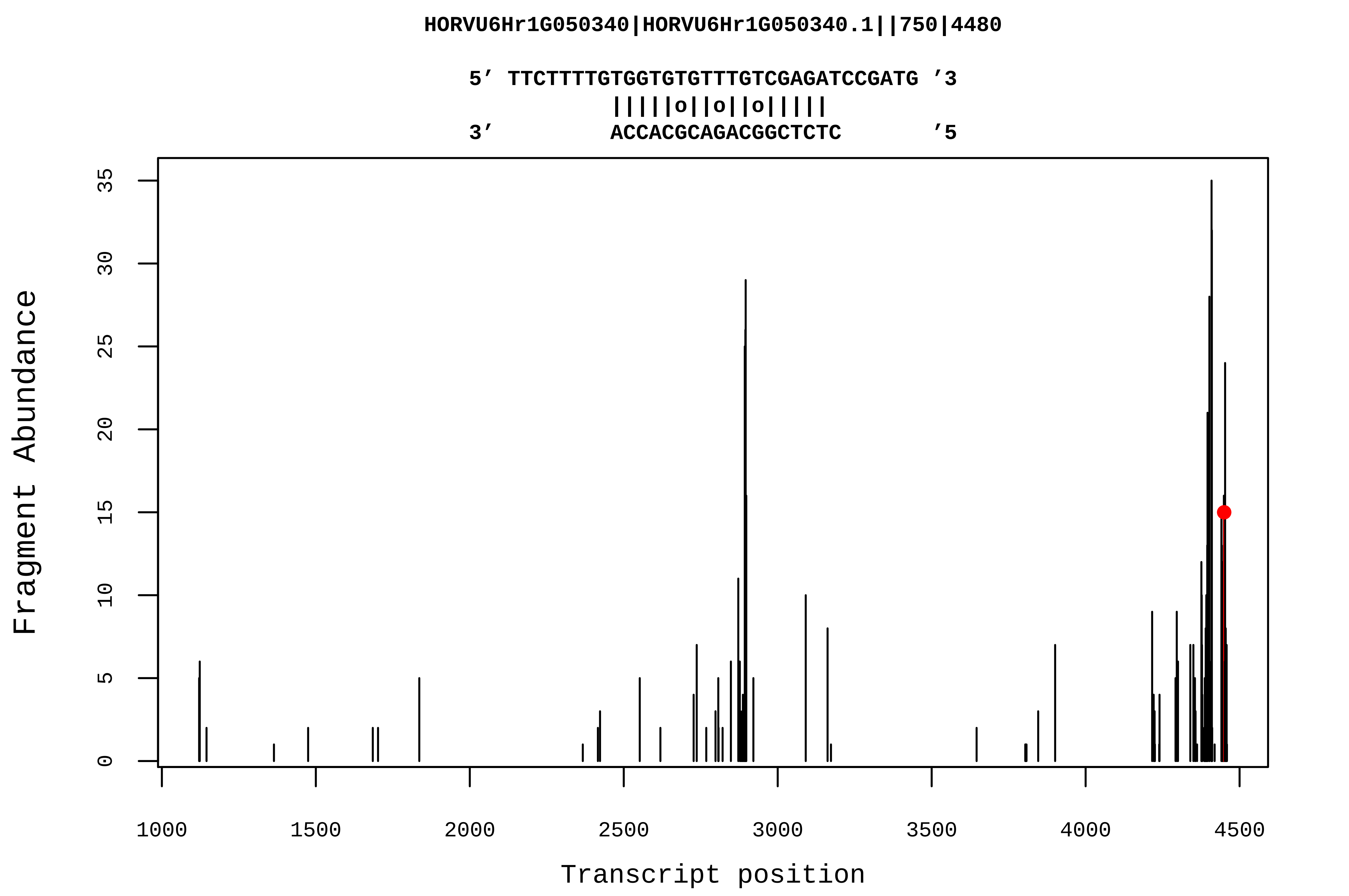

Cleavage site: 4450   Tag abundance: 15   Weighted abundance: 0.807   Category: 2
sRNA abundance: 1   Alignment score: 4   MFE ratio: 0.762   p-value: 0.005

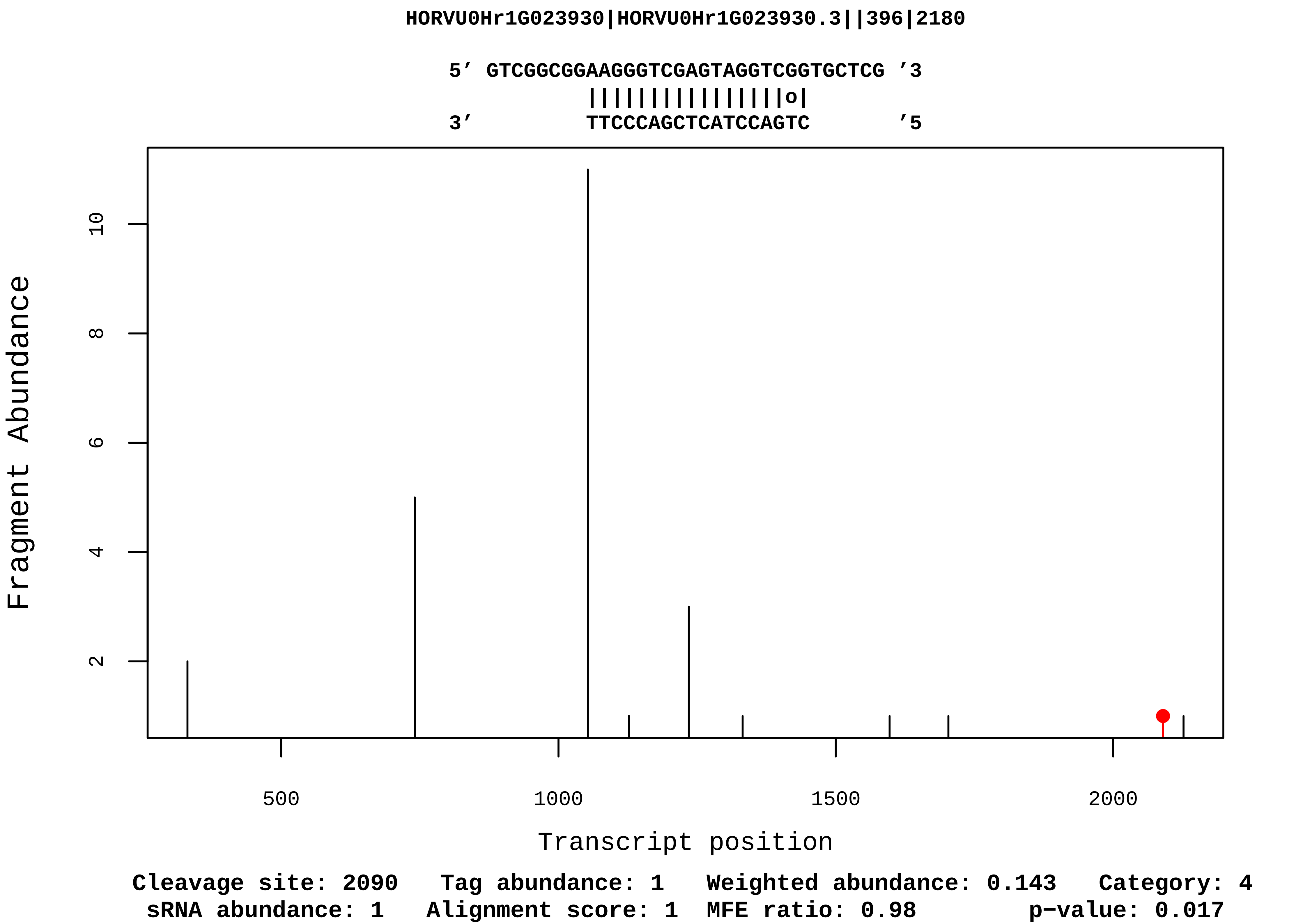
HORVU0Hr1G023930|HORVU0Hr1G023930.3||396|2180
5' GTCGGCGGAAGGGTCGAGTAGGTCGGTGCTCG '3
||||||||||||||||o|
3' TTCCCAGCTCATCCAGTC '5
Fragment Abundance
2
4
6
8
10
500
1000
1500
2000
Transcript position
Cleavage site: 2090 Tag abundance: 1 Weighted abundance: 0.143 Category: 4
sRNA abundance: 1 Alignment score: 1 MFE ratio: 0.98 p-value: 0.017

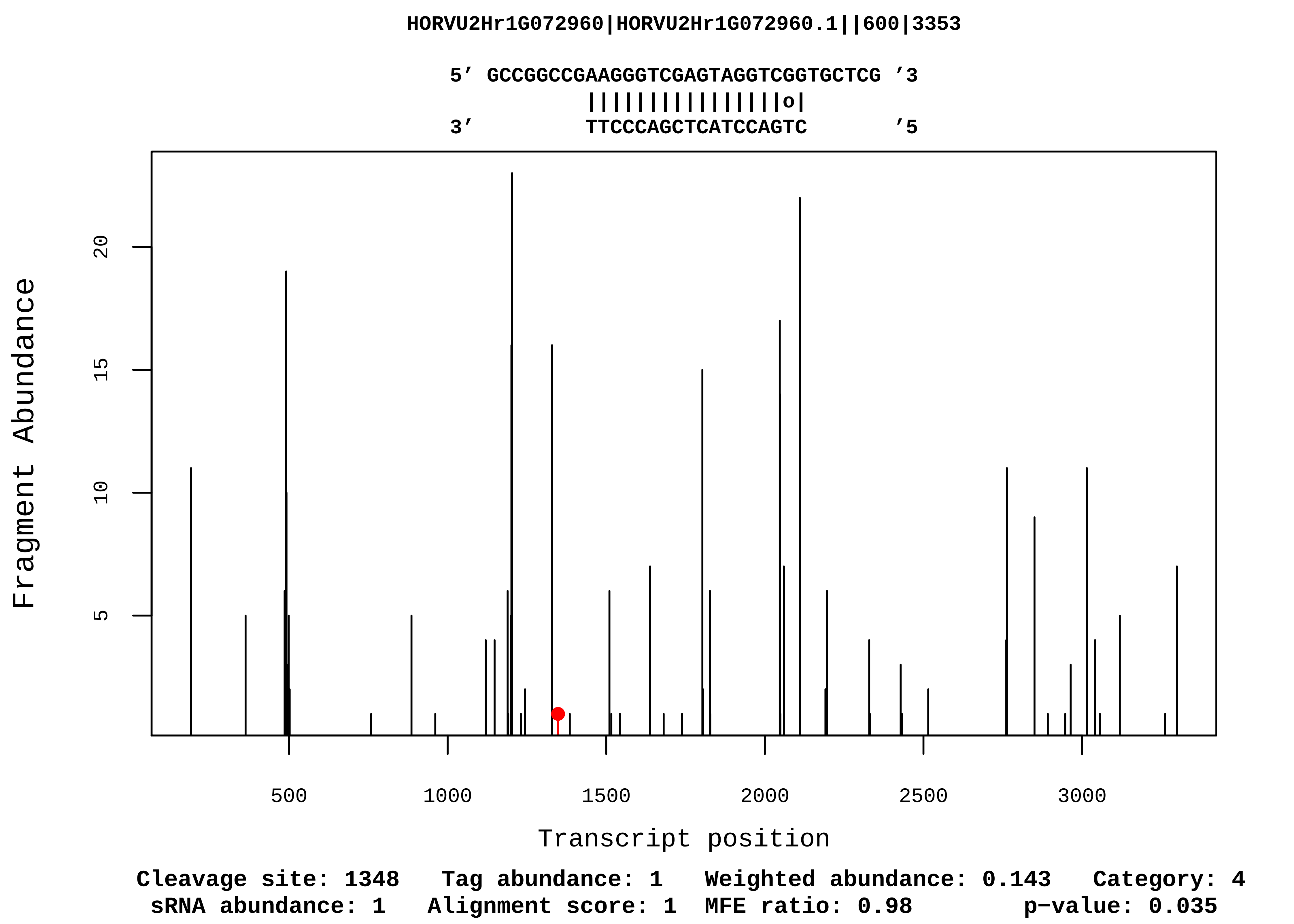
HORVU2Hr1G072960|HORVU2Hr1G072960.1||600|3353
5' GCCGGCCGAAGGGTCGAGTAGGTCGGTGCTCG '3
||||||||||||||||o|
3' TTCCCAGCTCATCCAGTC '5
Fragment Abundance
5
10
15
20
500
1000
1500
2000
2500
3000
Transcript position
Cleavage site: 1348 Tag abundance: 1 Weighted abundance: 0.143 Category: 4
sRNA abundance: 1 Alignment score: 1 MFE ratio: 0.98 p-value: 0.035

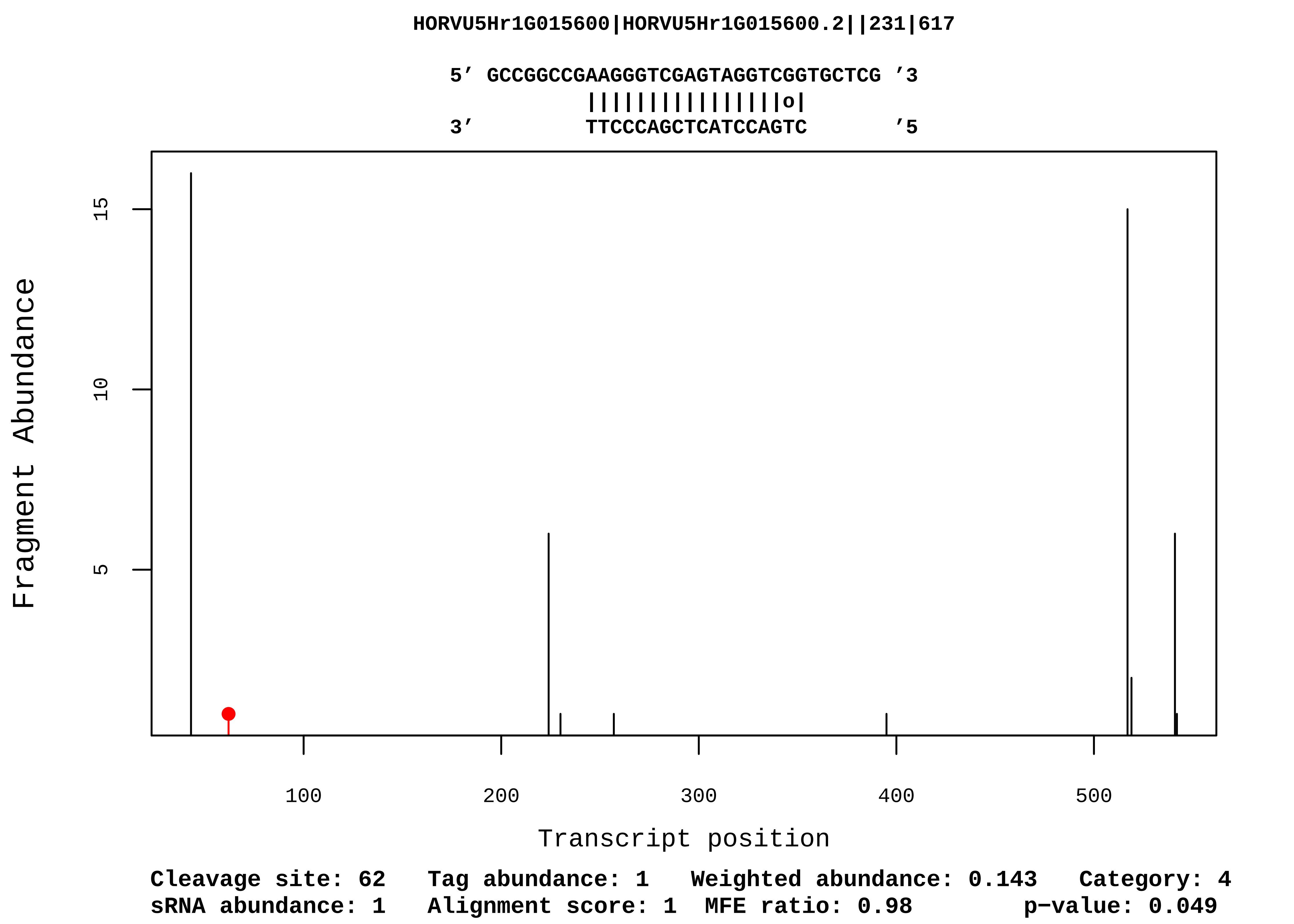
HORVU5Hr1G015600|HORVU5Hr1G015600.2||231|617
5' GCCGGCCGAAGGGTCGAGTAGGTCGGTGCTCG '3
||||||||||||||||o|
3' TTCCCAGCTCATCCAGTC '5
Fragment Abundance
15
10
5
100
200
300
400
500
Transcript position
Cleavage site: 62 Tag abundance: 1 Weighted abundance: 0.143 Category: 4
sRNA abundance: 1 Alignment score: 1 MFE ratio: 0.98 p-value: 0.049

HORVU5Hr1G015600|HORVU5Hr1G015600.3||276|1709

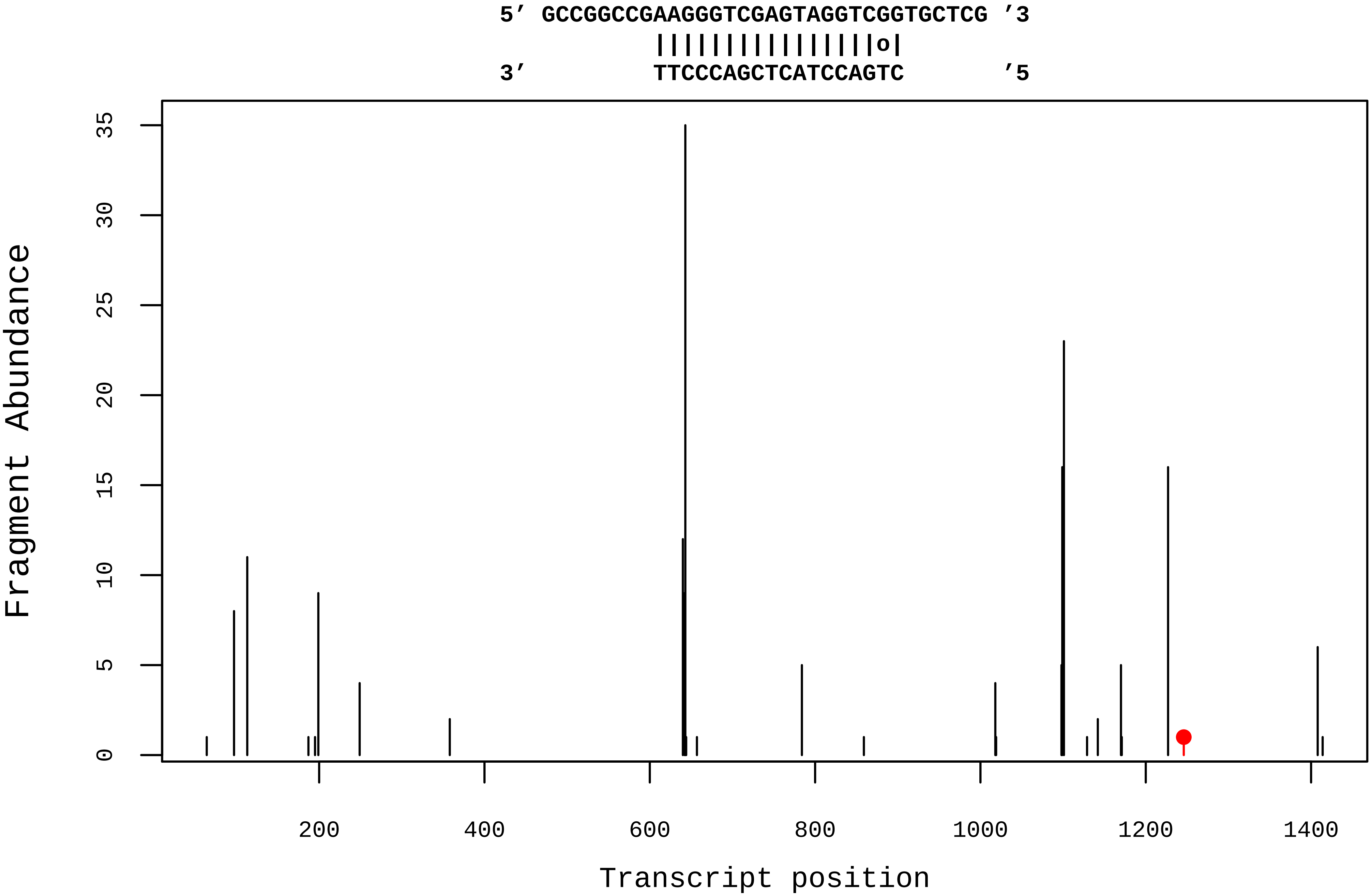

Cleavage site: 1246 Tag abundance: 1 Weighted abundance: 0.143 Category: 4
sRNA abundance: 1 Alignment score: 1 MFE ratio: 0.98 p-value: 0.038

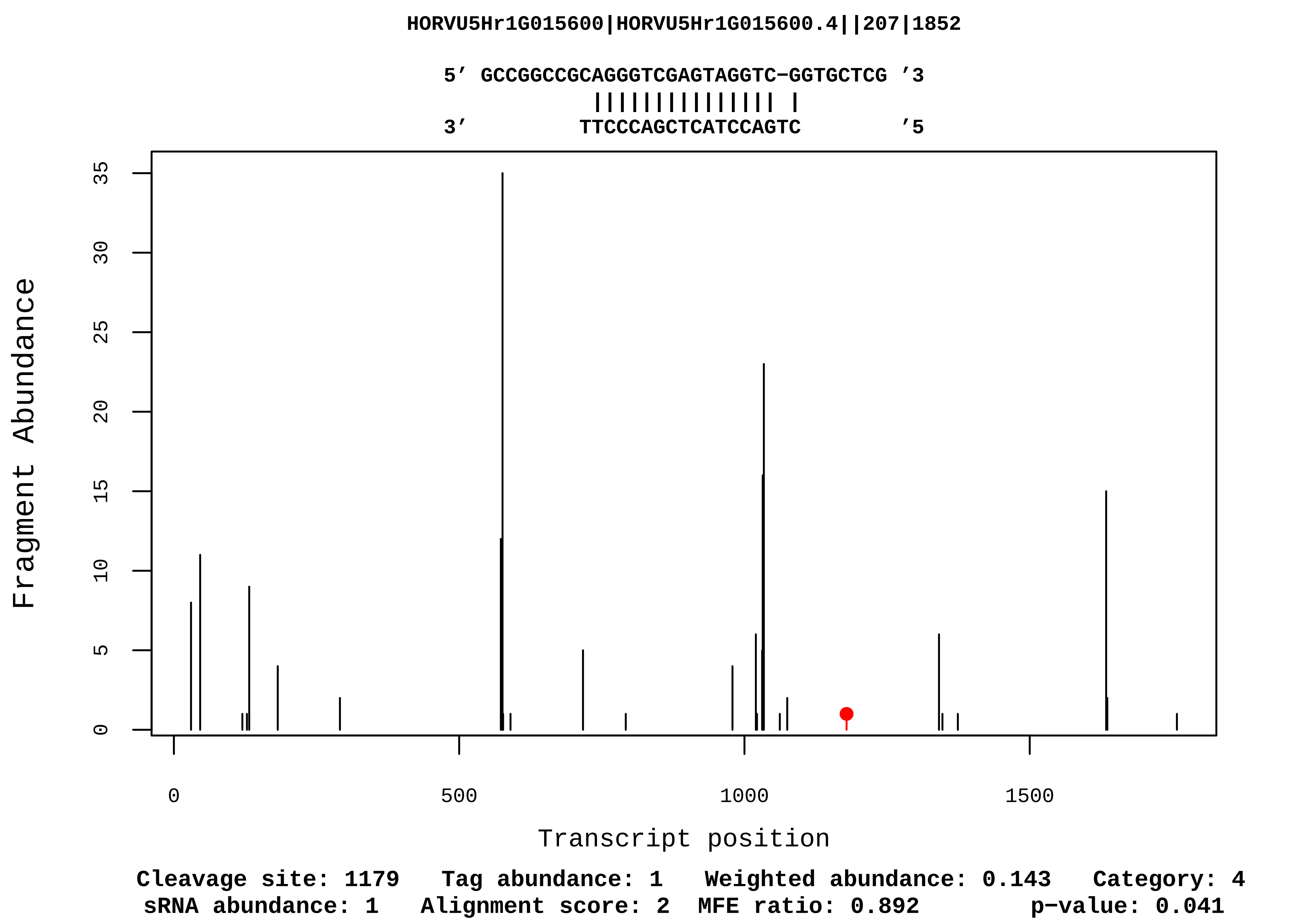

HORVU5Hr1G015600|HORVU5Hr1G015600.4||207|1852
5′ GCCGGCCGCAGGGTCGAGTAGGTC−GGTGCTCG ′3
|||||||||||||||| |
3′ TTCCCAGCTCATCCAGTC ′5
Fragment Abundance
0
5
10
15
20
25
30
35
0
500
1000
1500
Transcript position
Cleavage site: 1179 Tag abundance: 1 Weighted abundance: 0.143 Category: 4
sRNA abundance: 1 Alignment score: 2 MFE ratio: 0.892 p-value: 0.041

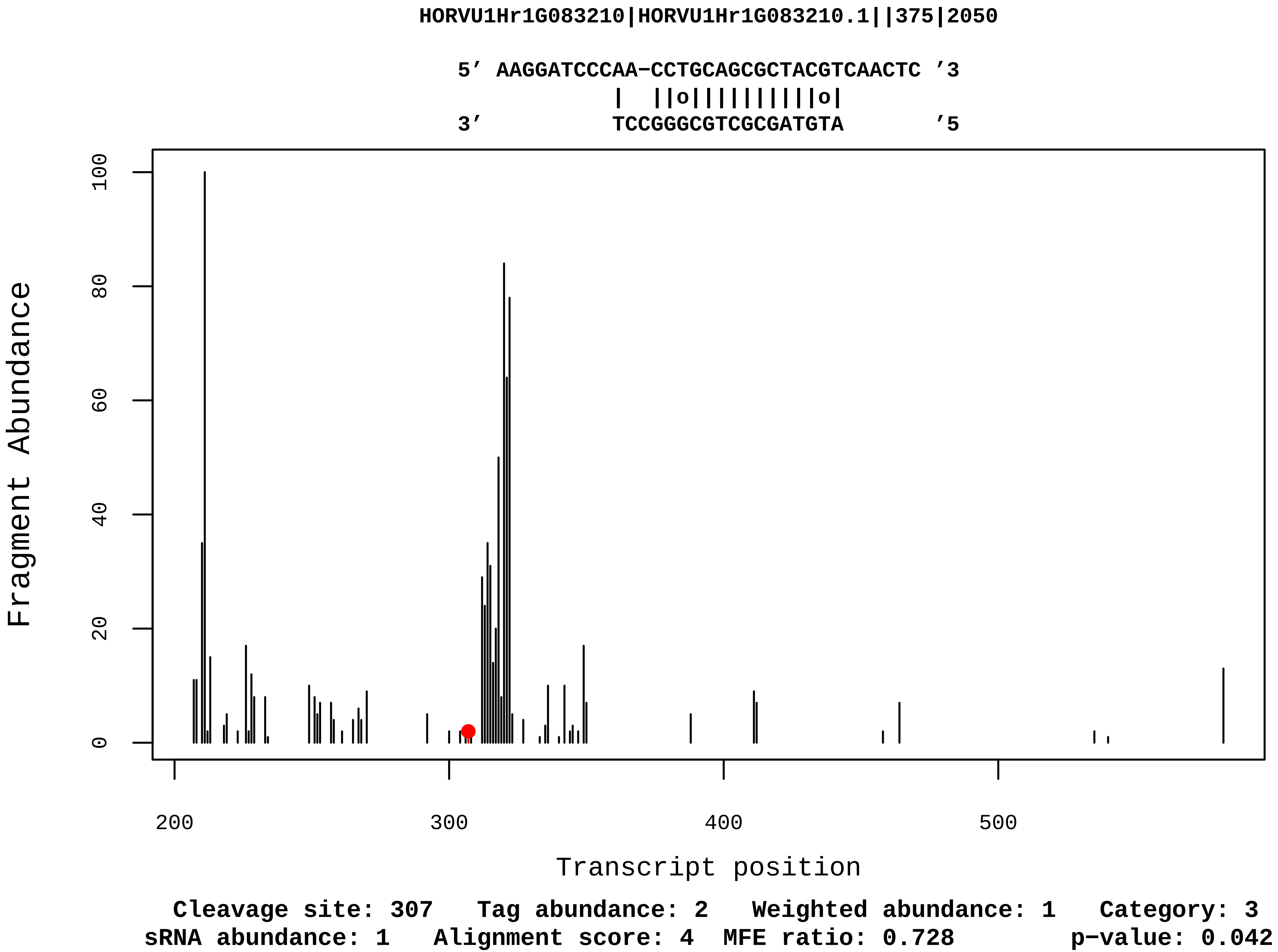

HORVU1Hr1G083210|HORVU1Hr1G083210.1||375|2050
5′ AAGGATCCCAA−CCTGCAGCGCTACGTCAACTC ′3
| ||o||||||||||o|
3′ TCCGGGCGTCGCGATGTA ′5
Fragment Abundance
0
20
40
60
80
100
200
300
400
500
Transcript position
Cleavage site: 307 Tag abundance: 2 Weighted abundance: 1 Category: 3
sRNA abundance: 1 Alignment score: 4 MFE ratio: 0.728 p-value: 0.042

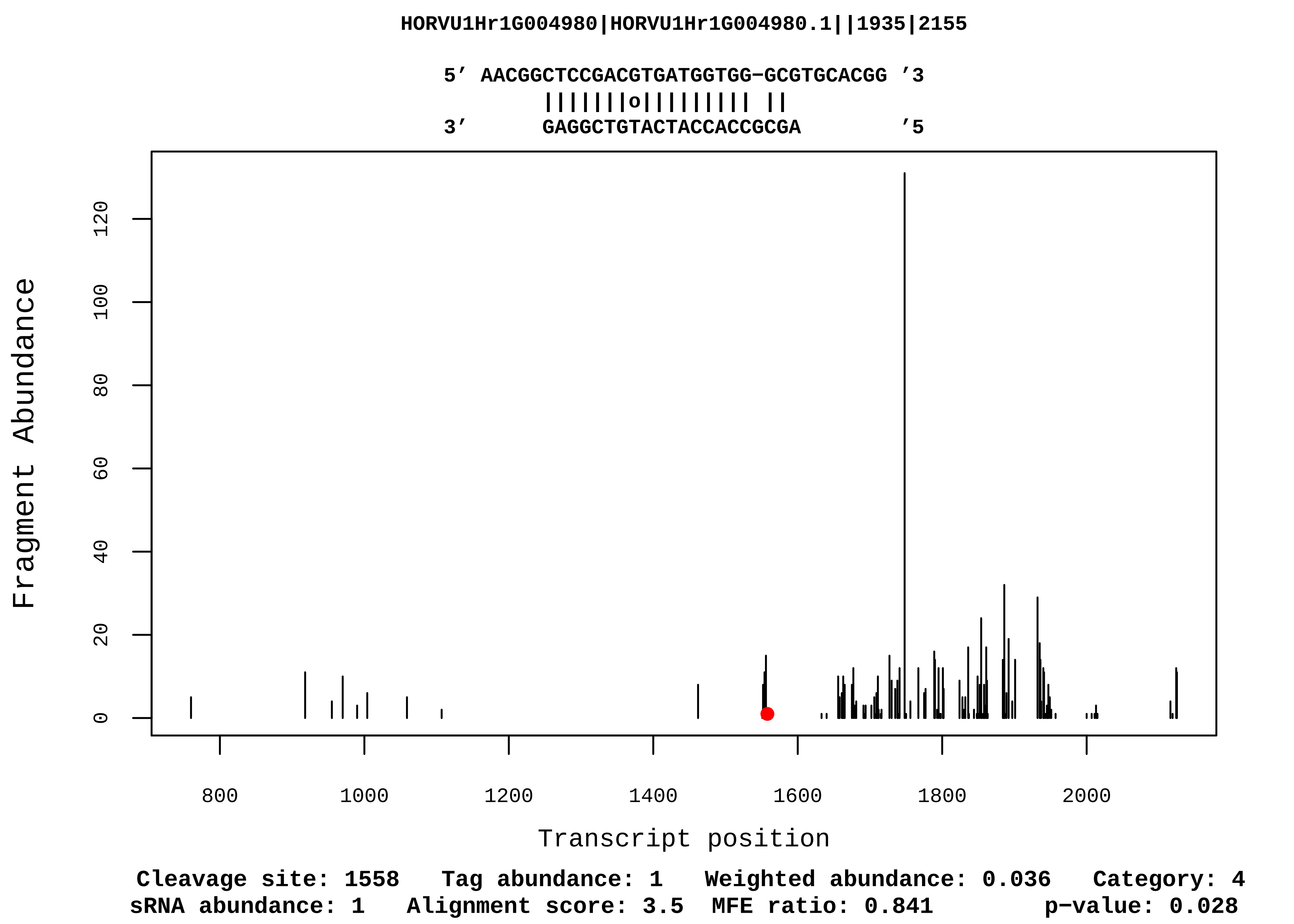

HORVU1Hr1G004980|HORVU1Hr1G004980.1||1935|2155
5′ AACGGCTCCGACGTGATGGTGG−GCGTGCACGG ′3
||||||||o||||||||| ||
3′ GAGGCTGTACTACCACCGCGA ′5
Fragment Abundance
0
20
40
60
80
100
120
800
1000
1200
1400
1600
1800
2000
Transcript position
Cleavage site: 1558 Tag abundance: 1 Weighted abundance: 0.036 Category: 4
sRNA abundance: 1 Alignment score: 3.5 MFE ratio: 0.841 p−value: 0.028

HORVU1Hr1G004980|HORVU1Hr1G004980.2||1551|1551

```
5′ AACGGCTCCGACGTGATGGTGG-GCGTGCACGG ′3
           |||||||o||||||||| ||
3′         GAGGCTGTACTACCACCGCGA          ′5
```

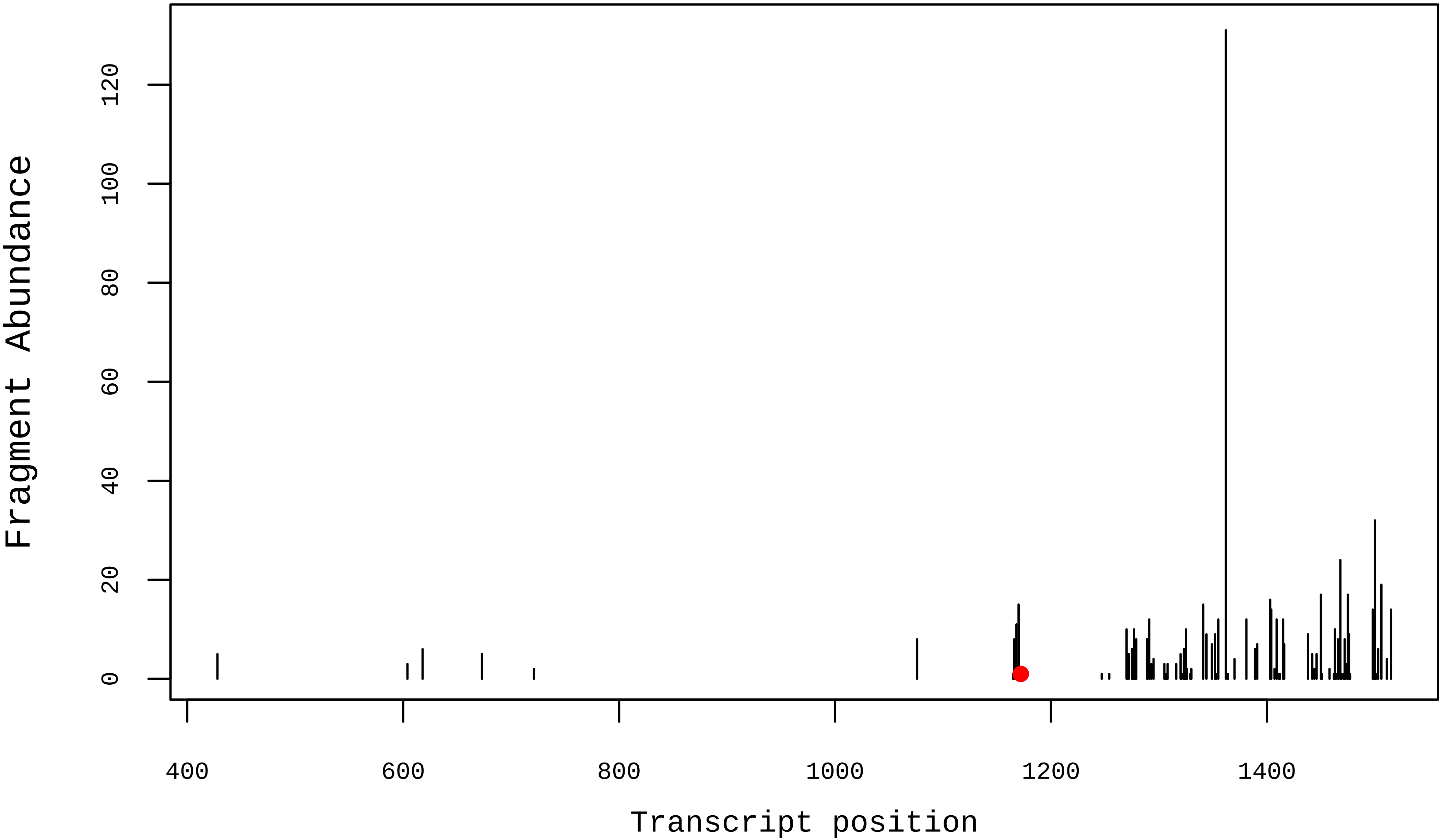

Cleavage site: 1172   Tag abundance: 1   Weighted abundance: 0.036   Category: 4
sRNA abundance: 1   Alignment score: 3.5   MFE ratio: 0.841   p-value: 0.029

HORVU3Hr1G001430|HORVU3Hr1G001430.1||1884|2633

```
5' CAGAGTGCAACTGGA-CCTAAGACTGGTCTCAG '3
            | |o||| |||||||||||
3'          TCGGCCTAGGATTCTGACC        '5
```

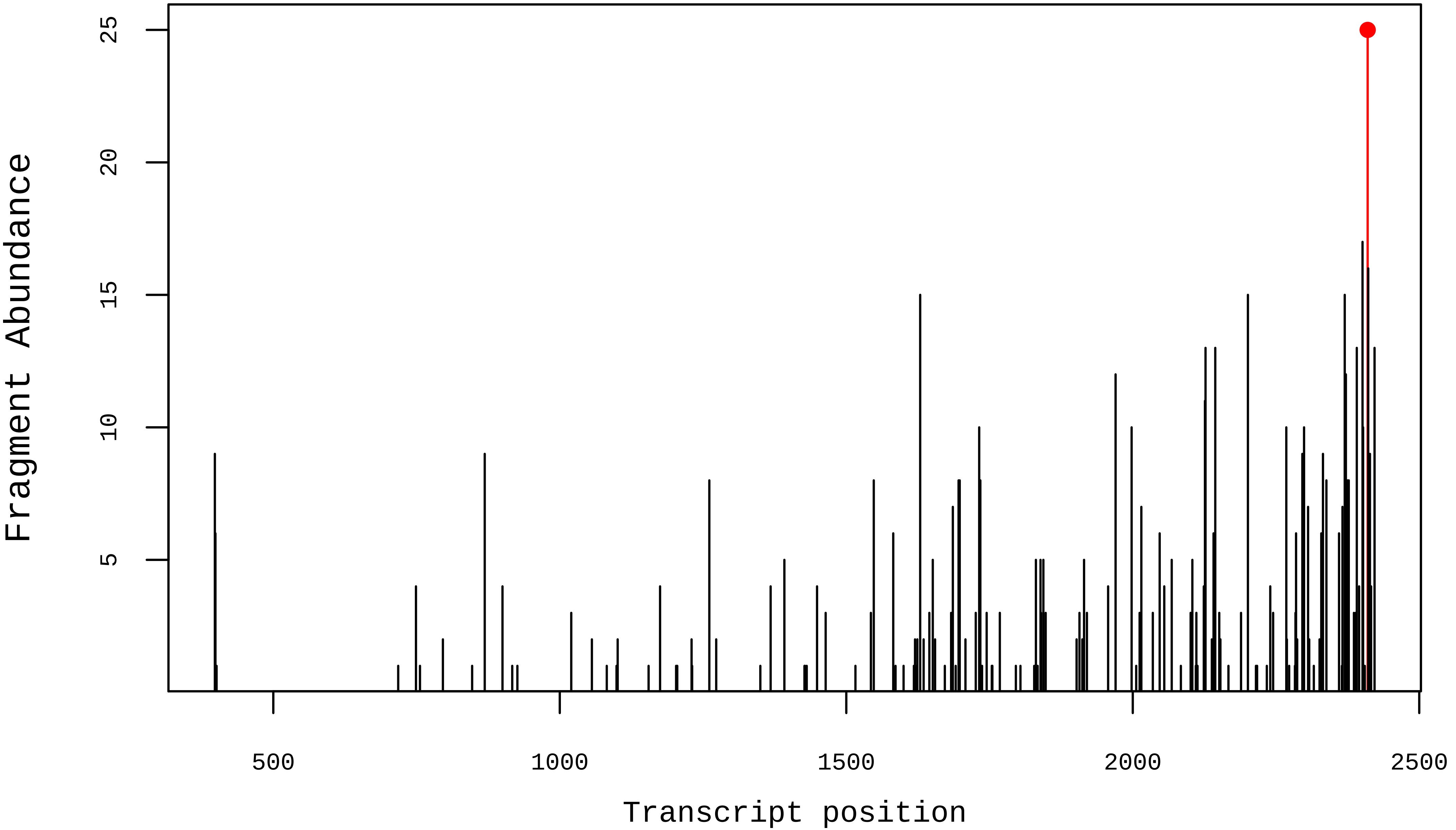

Cleavage site: 2410 Tag abundance: 25 Weighted abundance: 4.167 Category: 0
sRNA abundance: 1 Alignment score: 3.5 MFE ratio: 0.713 p-value: 0.002

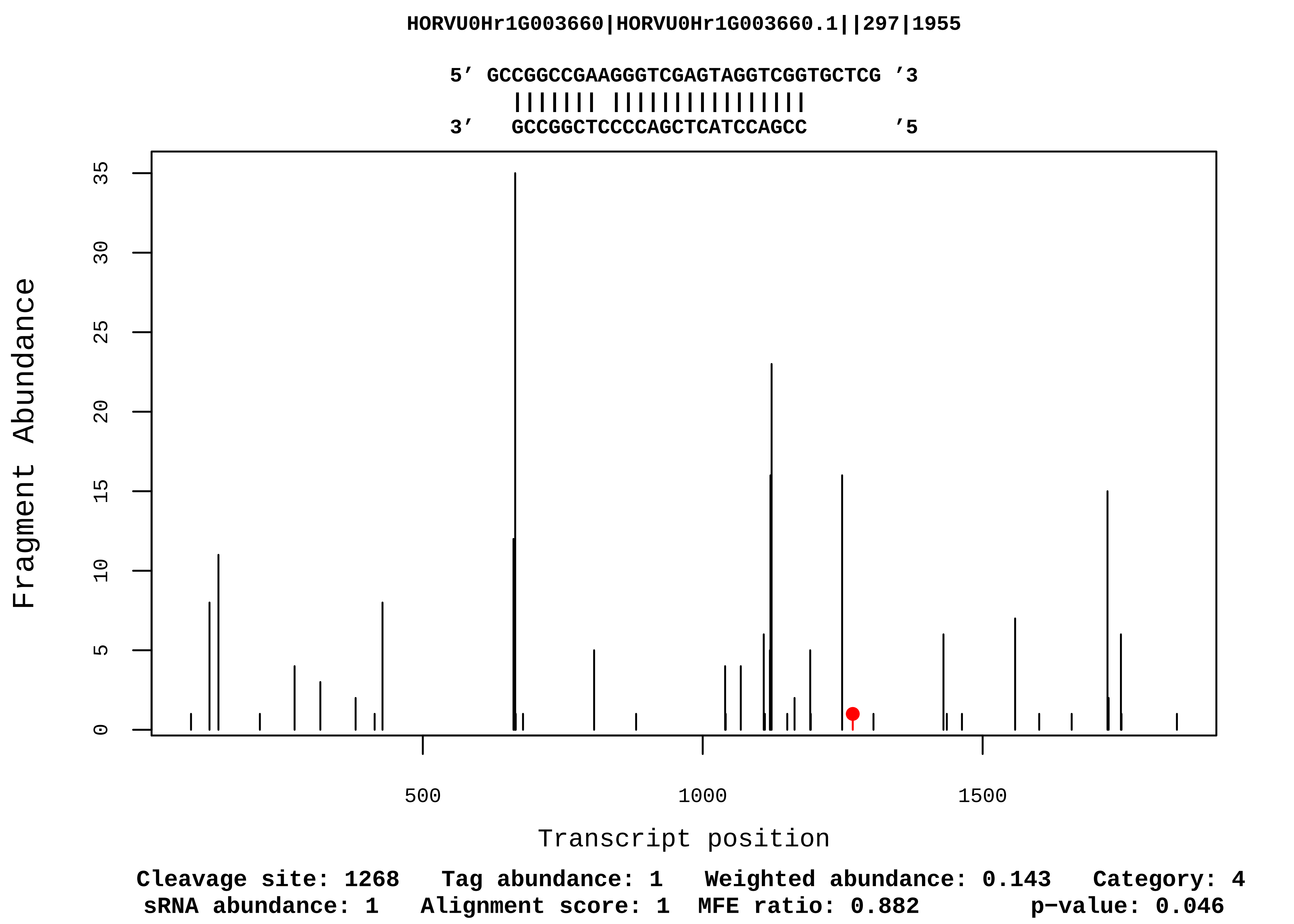

HORVU0Hr1G003660|HORVU0Hr1G003660.1||297|1955
5' GCCGGCCGAAGGGTCGAGTAGGTCGGTGCTCG '3
3' GCCGGCTCCCCAGCTCATCCAGCC '5
Fragment Abundance
0
5
10
15
20
25
30
35
500
1000
1500
Transcript position
Cleavage site: 1268 Tag abundance: 1 Weighted abundance: 0.143 Category: 4
sRNA abundance: 1 Alignment score: 1 MFE ratio: 0.882 p-value: 0.046

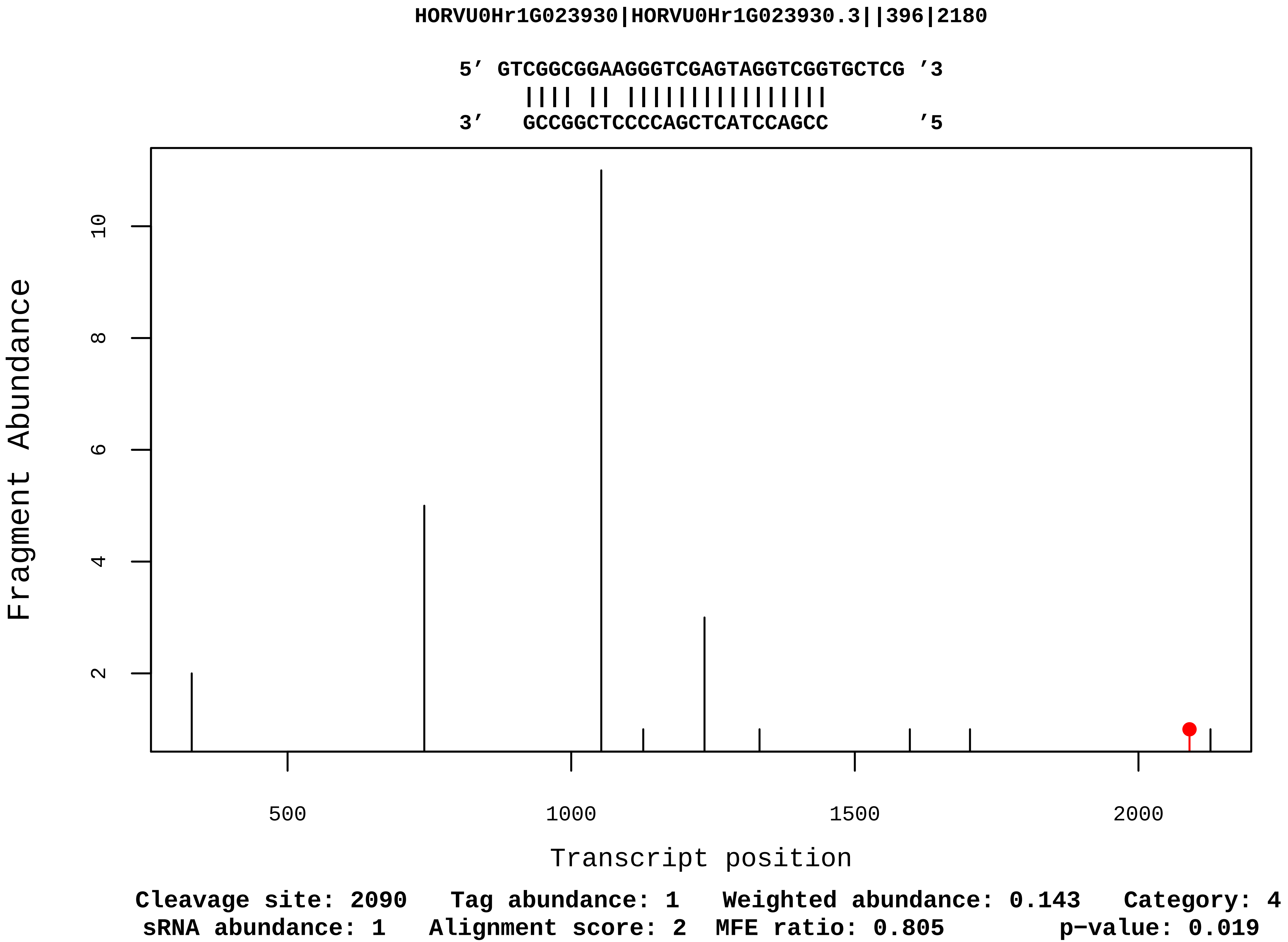
HORVU0Hr1G023930|HORVU0Hr1G023930.3||396|2180
5' GTCGGCGGAAGGGTCGAGTAGGTCGGTGCTCG '3
3' GCCGGCTCCCCAGCTCATCCAGCC '5
Fragment Abundance
2
4
6
8
10
500
1000
1500
2000
Transcript position
Cleavage site: 2090 Tag abundance: 1 Weighted abundance: 0.143 Category: 4
sRNA abundance: 1 Alignment score: 2 MFE ratio: 0.805 p-value: 0.019

HORVU2Hr1G072960|HORVU2Hr1G072960.1||600|3353

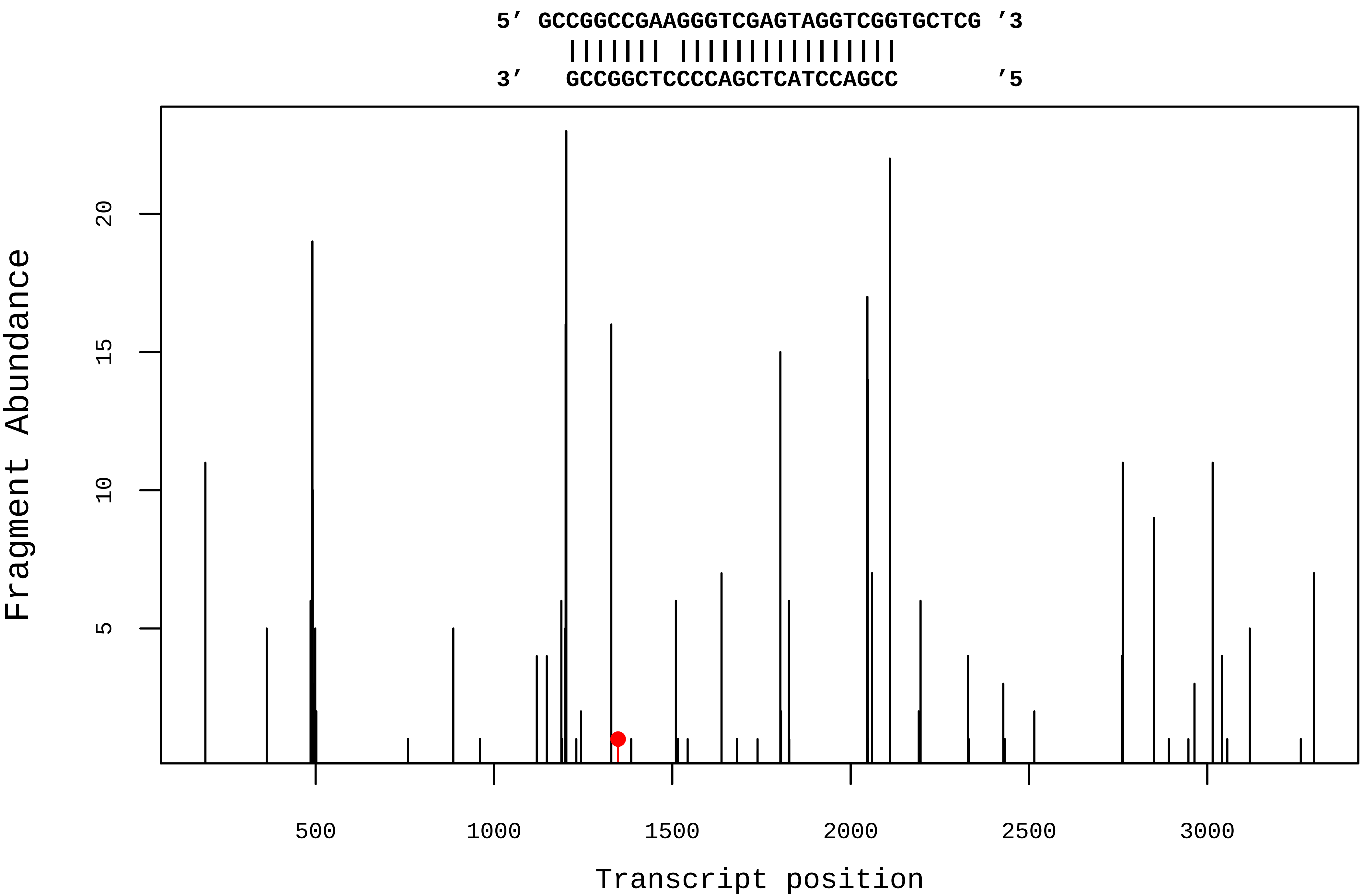

Cleavage site: 1348 Tag abundance: 1 Weighted abundance: 0.143 Category: 4
sRNA abundance: 1 Alignment score: 1 MFE ratio: 0.882 p-value: 0.03

HORVU5Hr1G015600|HORVU5Hr1G015600.1||156|510

```
5' GCCGGCCGAAGGGTCGAGTAGGTCGGTGCTCG '3
       ||||||| ||||||||||||||||
3'    GCCGGCTCCCCAGCTCATCCAGCC       '5
```

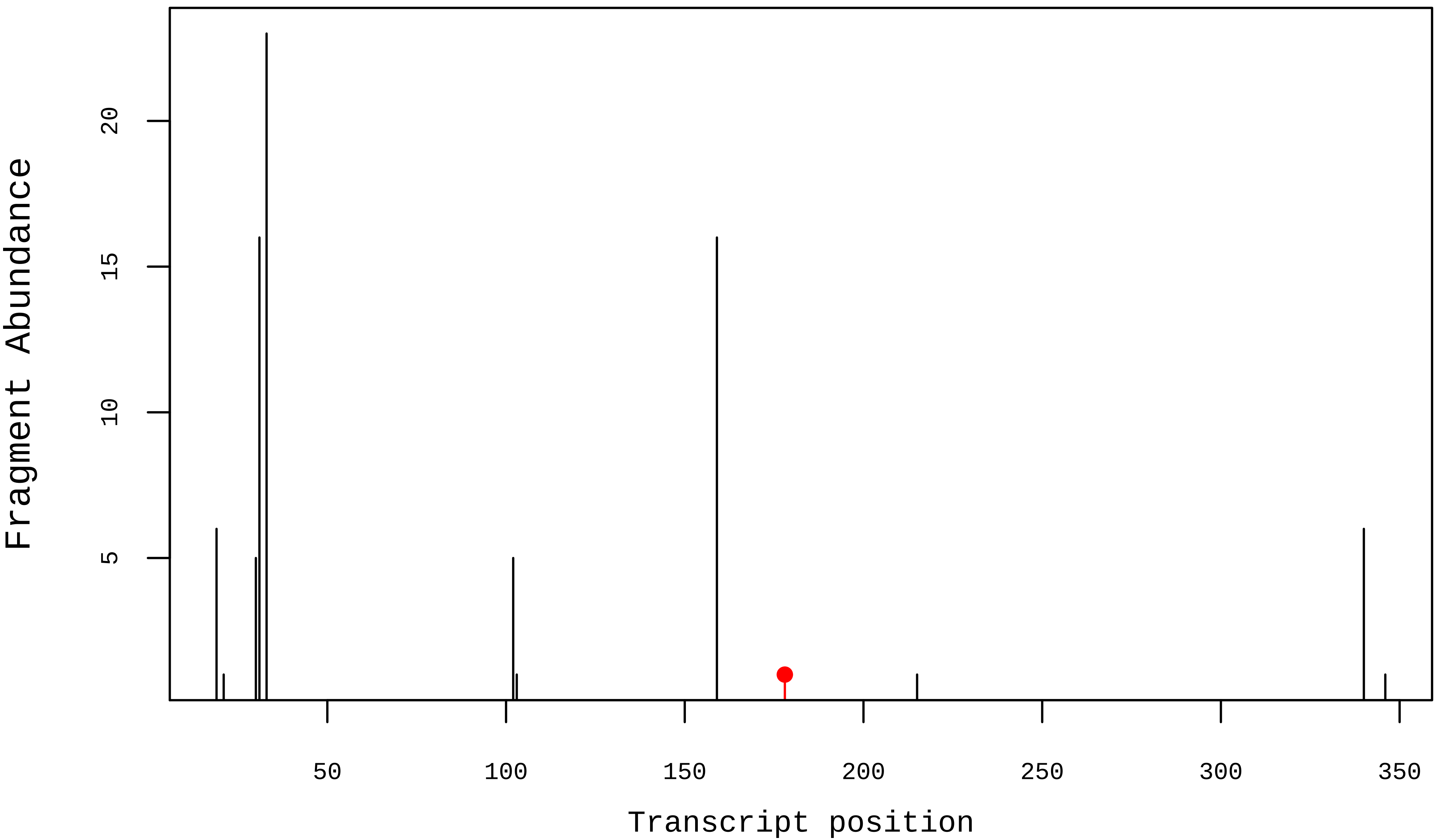

Cleavage site: 178   Tag abundance: 1   Weighted abundance: 0.143   Category: 4
sRNA abundance: 1   Alignment score: 1   MFE ratio: 0.882   p-value: 0.05

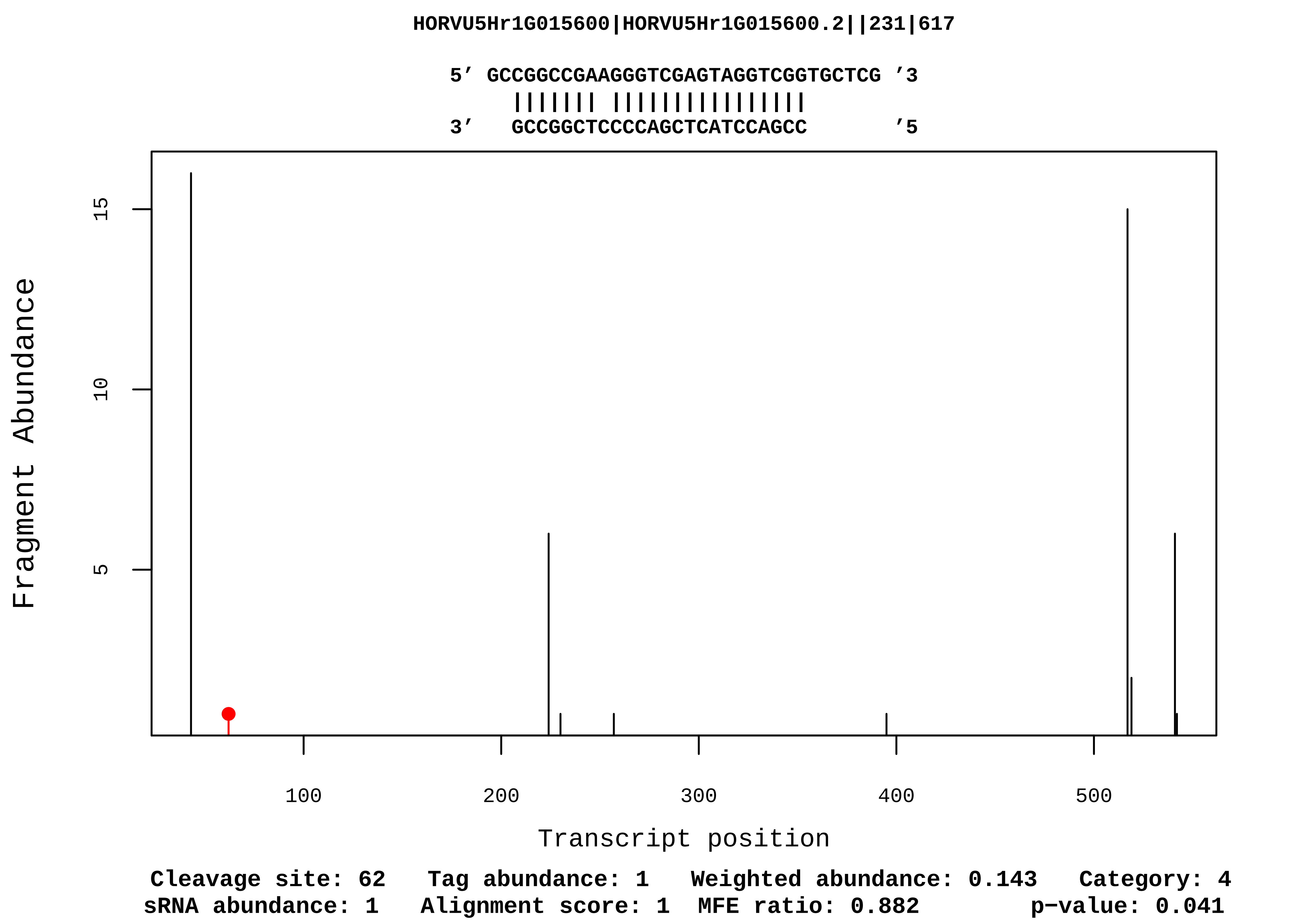
HORVU5Hr1G015600|HORVU5Hr1G015600.2||231|617
5' GCCGGCCGAAGGGTCGAGTAGGTCGGTGCTCG '3
||||||| ||||||||||||||||
3' GCCGGCTCCCCAGCTCATCCAGCC '5
Fragment Abundance
5
10
15
100
200
300
400
500
Transcript position
Cleavage site: 62 Tag abundance: 1 Weighted abundance: 0.143 Category: 4
sRNA abundance: 1 Alignment score: 1 MFE ratio: 0.882 p-value: 0.041

HORVU5Hr1G015600|HORVU5Hr1G015600.3||276|1709

```
5' GCCGGCCGAAGGGTCGAGTAGGTCGGTGCTCG '3
      ||||||| ||||||||||||||||
3'    GCCGGCTCCCCAGCTCATCCAGCC       '5
```

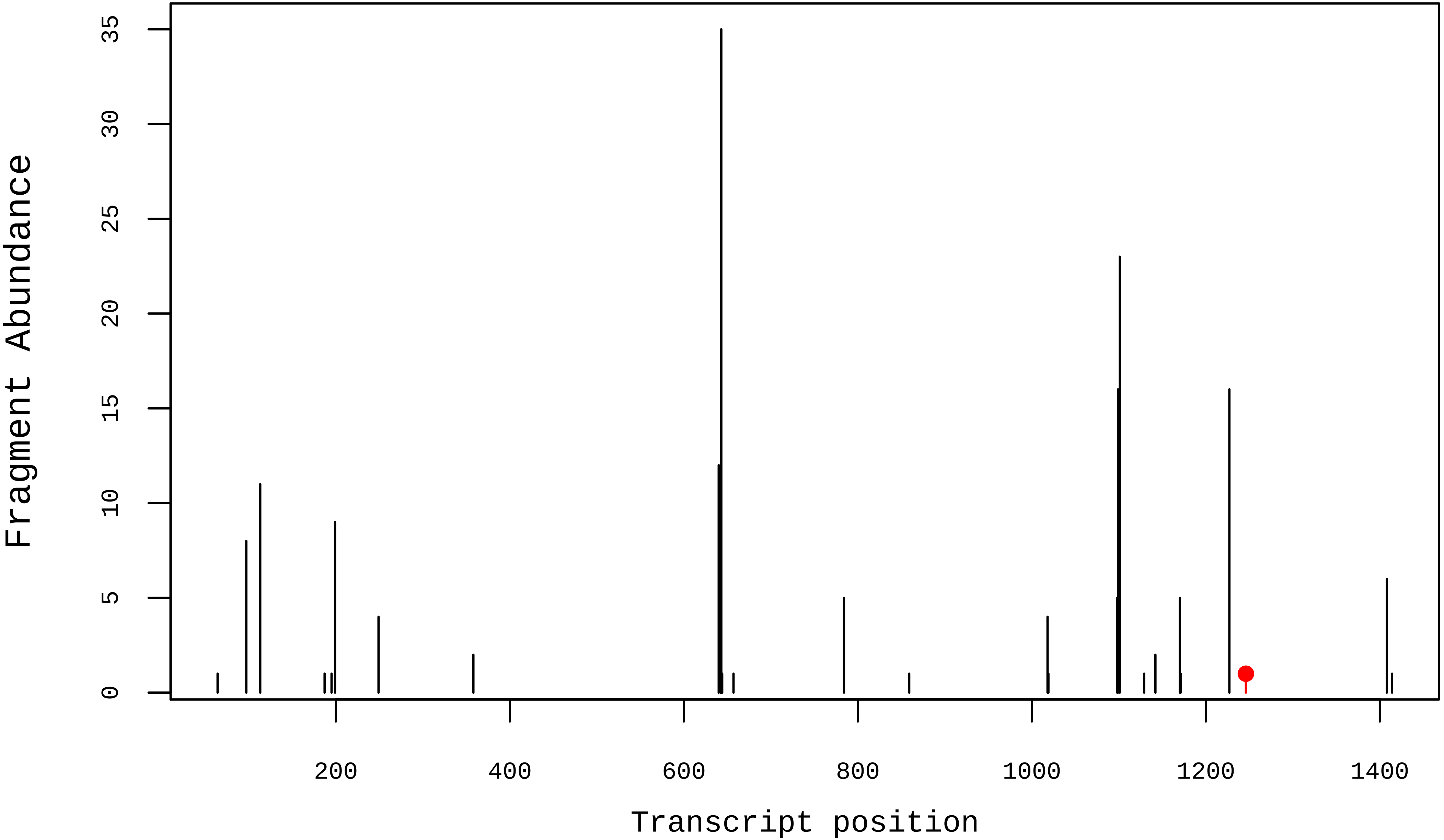

Cleavage site: 1246   Tag abundance: 1   Weighted abundance: 0.143   Category: 4
sRNA abundance: 1   Alignment score: 1   MFE ratio: 0.882   p-value: 0.032

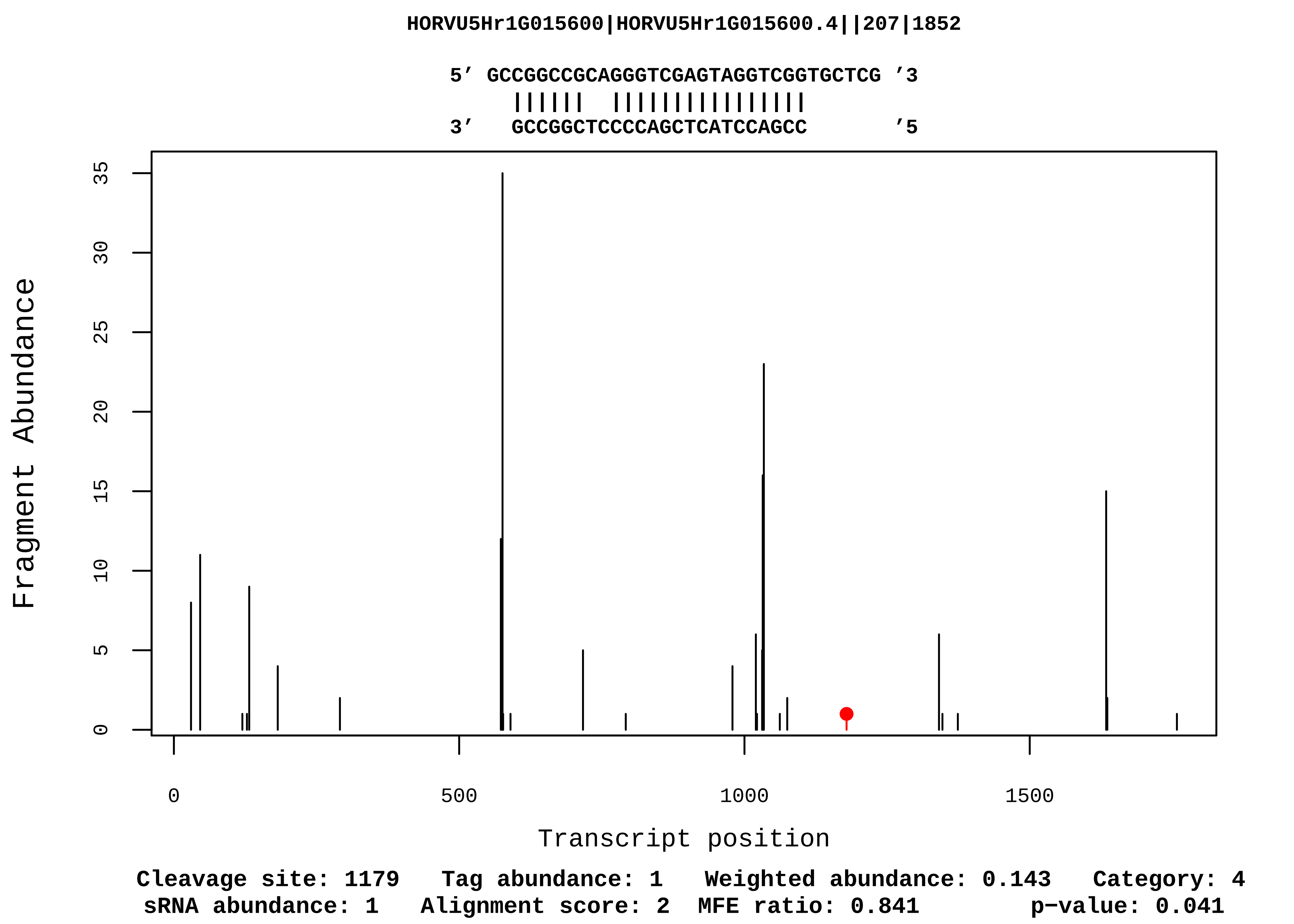

HORVU5Hr1G015600|HORVU5Hr1G015600.4||207|1852
5' GCCGGCCGCAGGGTCGAGTAGGTCGGTGCTCG '3
|||||| ||||||||||||||||
3' GCCGGCTCCCCAGCTCATCCAGCC '5
Fragment Abundance
0
5
10
15
20
25
30
35
0
500
1000
1500
Transcript position
Cleavage site: 1179 Tag abundance: 1 Weighted abundance: 0.143 Category: 4
sRNA abundance: 1 Alignment score: 2 MFE ratio: 0.841 p-value: 0.041

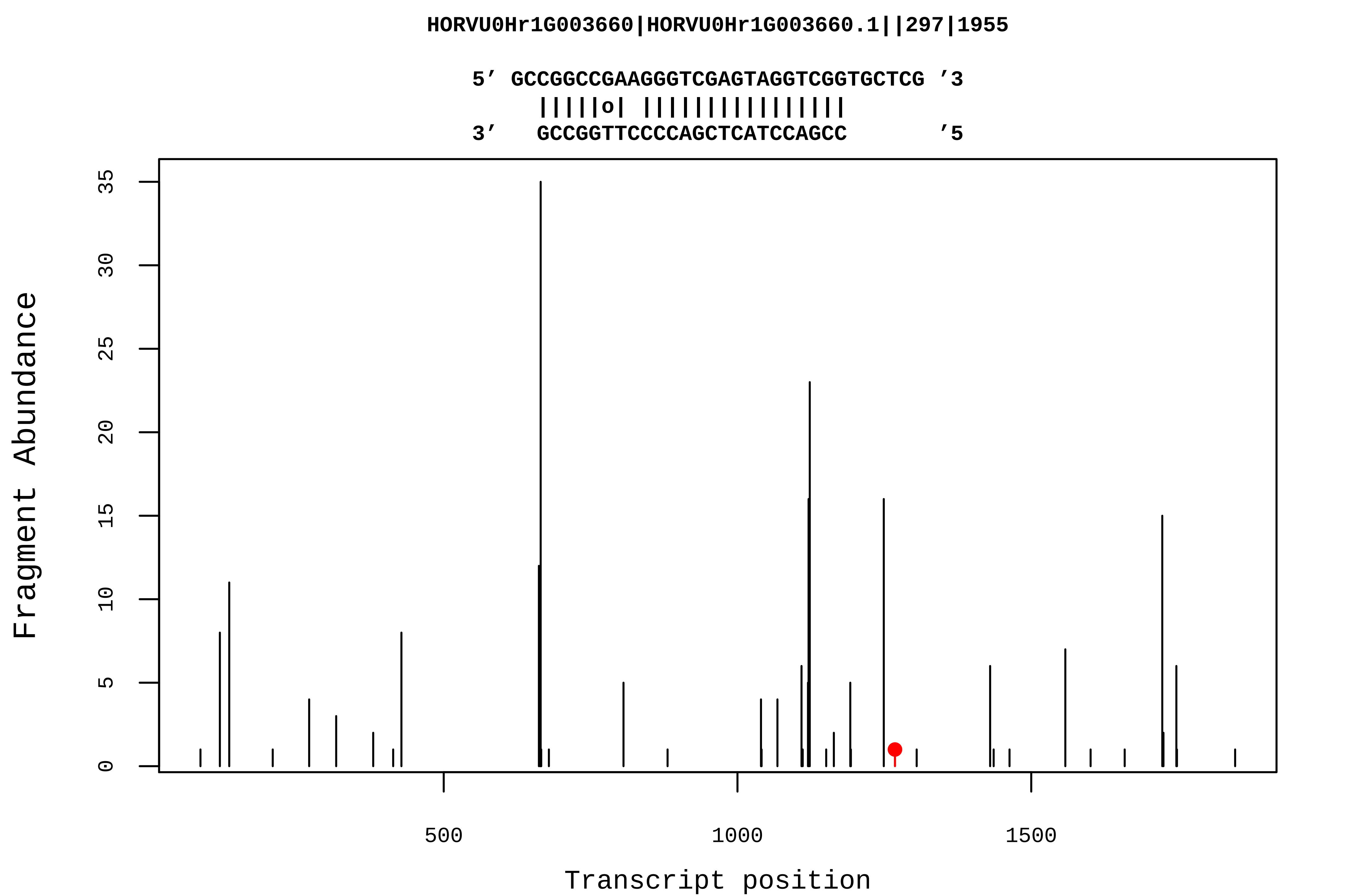

Cleavage site: 1268 Tag abundance: 1 Weighted abundance: 0.143 Category: 4
sRNA abundance: 1 Alignment score: 1.5 MFE ratio: 0.872 p-value: 0.046

HORVU0Hr1G023930|HORVU0Hr1G023930.3||396|2180

```
5' GTCGGCGGAAGGGTCGAGTAGGTCGGTGCTCG '3
      |||| o| ||||||||||||||||
3'    GCCGGTTCCCCAGCTCATCCAGCC       '5
```

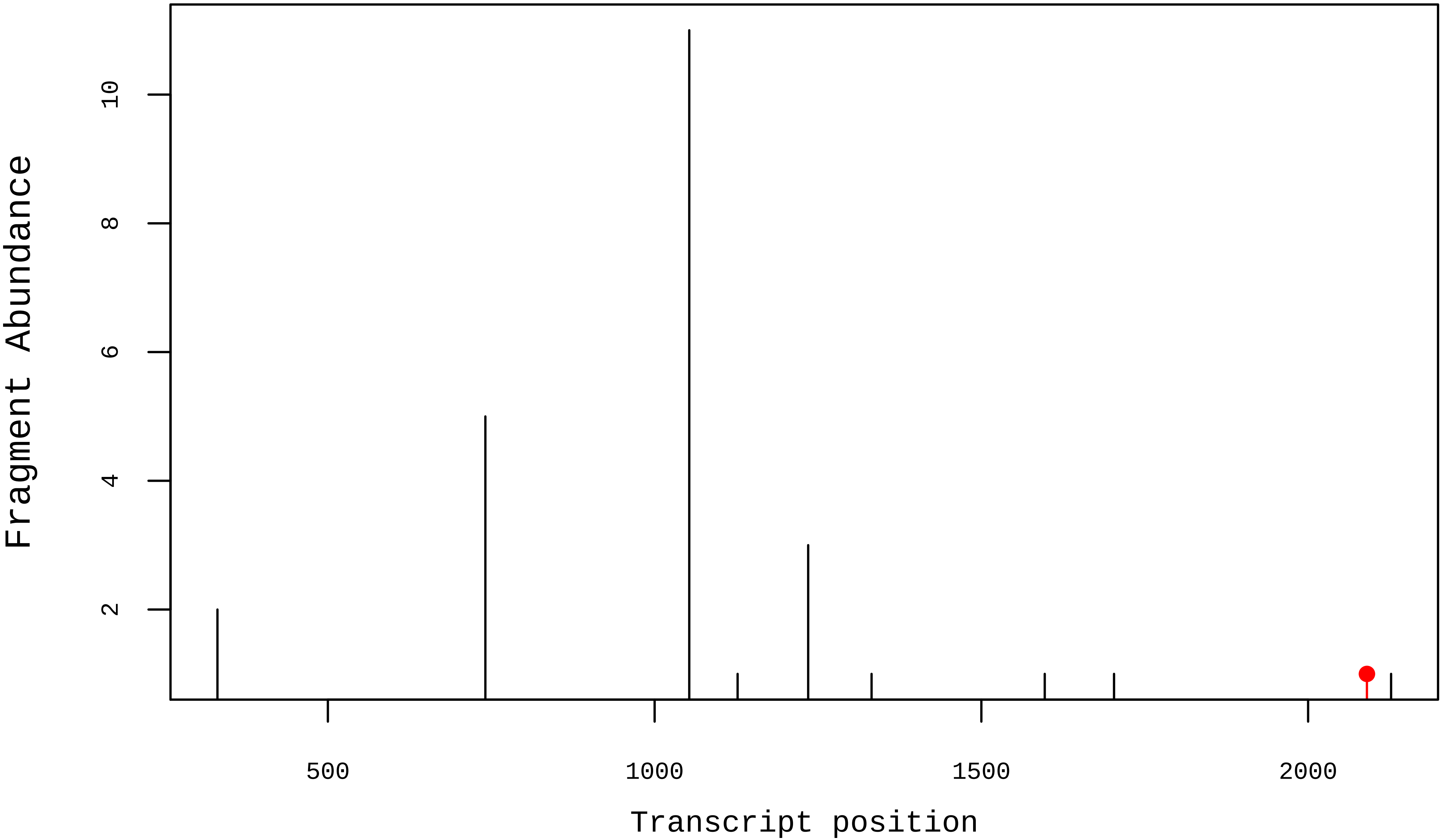

Cleavage site: 2090   Tag abundance: 1   Weighted abundance: 0.143   Category: 4
sRNA abundance: 1   Alignment score: 2.5   MFE ratio: 0.811   p-value: 0.019

HORVU2Hr1G072960|HORVU2Hr1G072960.1||600|3353

```
5' GCCGGCCGAAGGGTCGAGTAGGTCGGTGCTCG '3
      |||||o| ||||||||||||||||
3'    GCCGGTTCCCCAGCTCATCCAGCC       '5
```

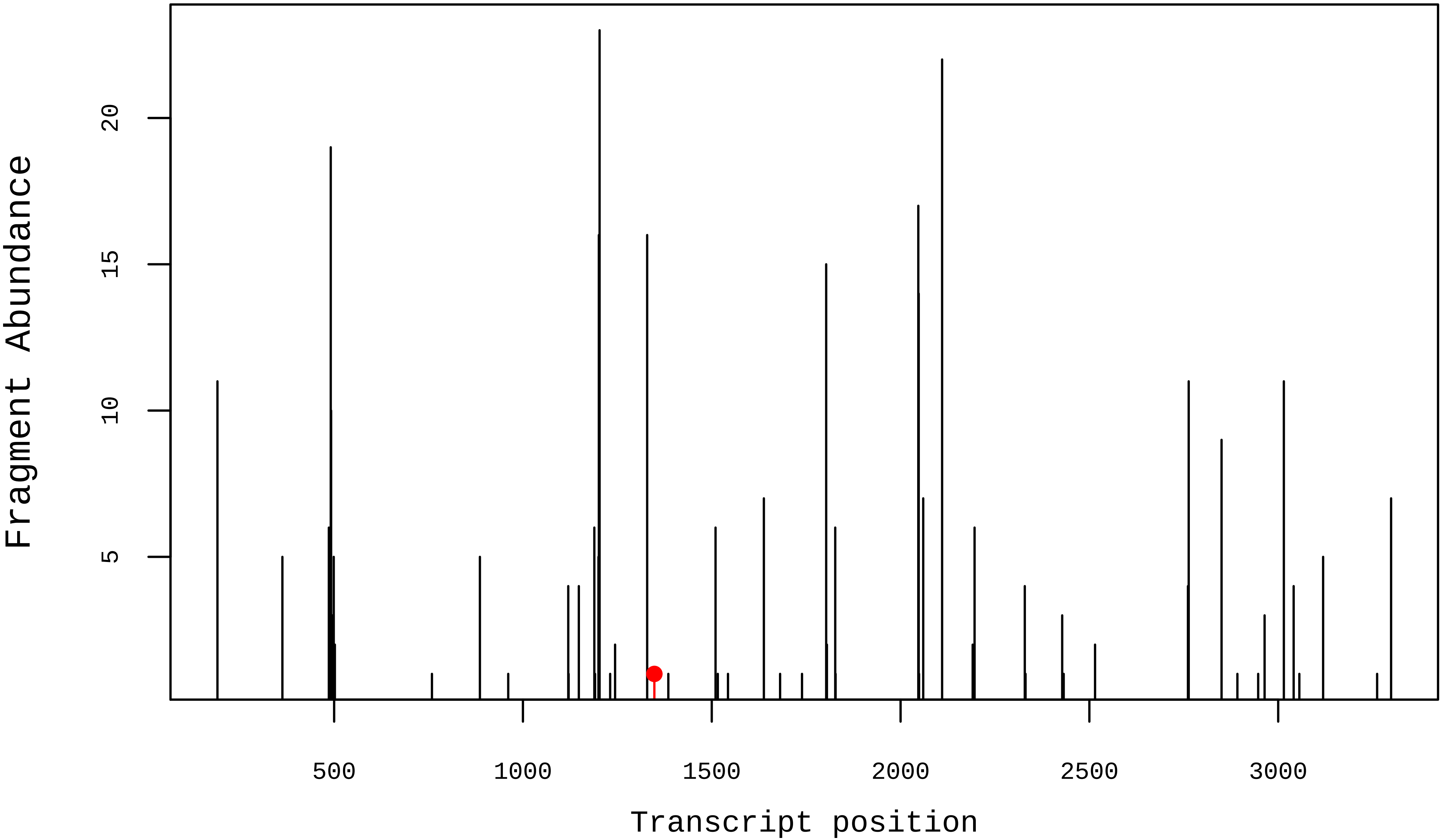

Cleavage site: 1348 Tag abundance: 1 Weighted abundance: 0.143 Category: 4
sRNA abundance: 1 Alignment score: 1.5 MFE ratio: 0.872 p-value: 0.03

HORVU5Hr1G015600|HORVU5Hr1G015600.1||156|510

```
5' GCCGGCCGAAGGGTCGAGTAGGTCGGTGCTCG '3
      |||||o| ||||||||||||||||
3'    GCCGGTTCCCCAGCTCATCCAGCC        '5
```

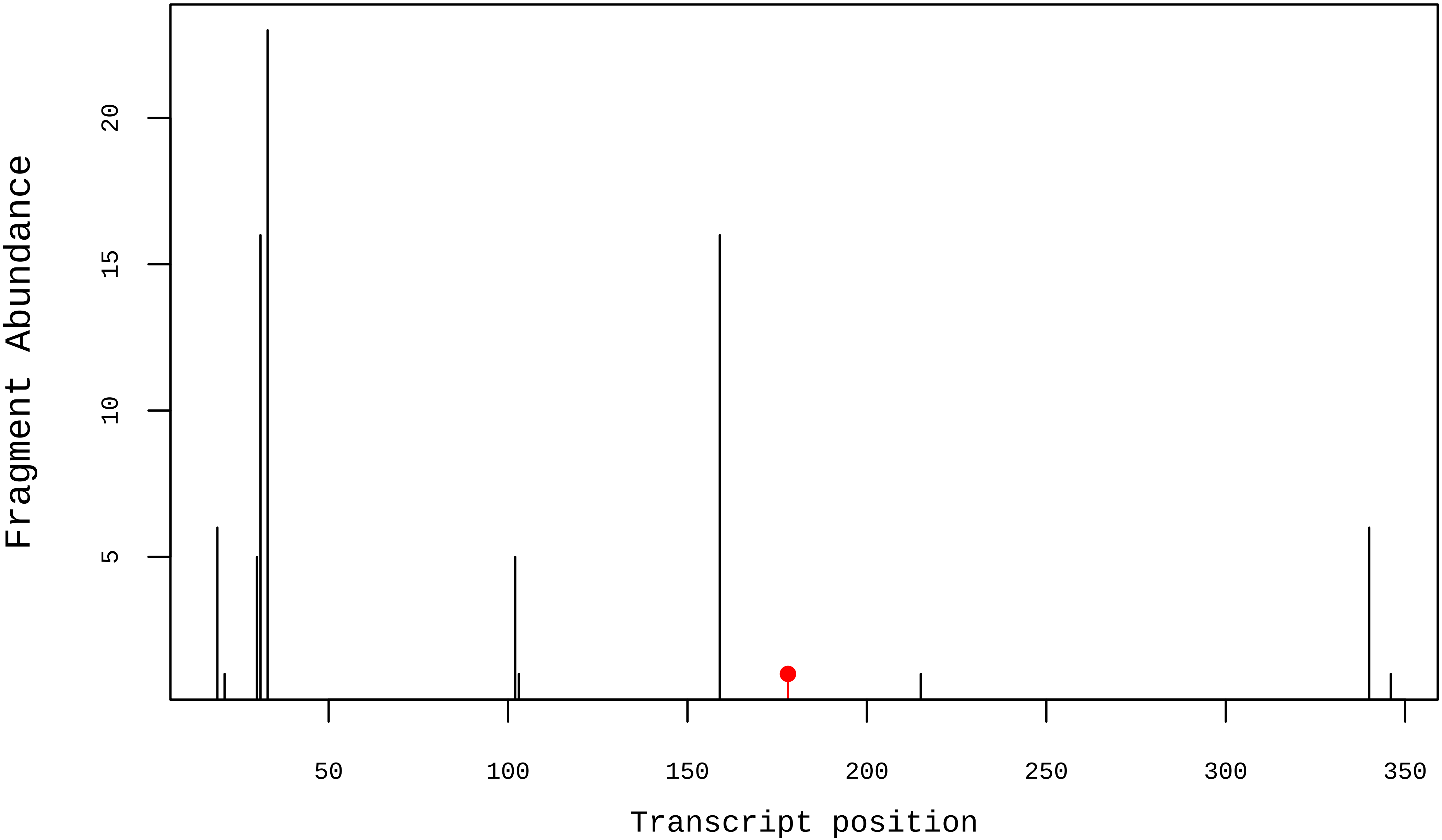

Cleavage site: 178   Tag abundance: 1   Weighted abundance: 0.143   Category: 4
sRNA abundance: 1   Alignment score: 1.5   MFE ratio: 0.872   p-value: 0.05

HORVU5Hr1G015600|HORVU5Hr1G015600.2||231|617

```
5’ GCCGGCCGAAGGGTCGAGTAGGTCGGTGCTCG ’3
      |||||o| ||||||||||||||||
3’    GCCGGTTCCCCAGCTCATCCAGCC        ’5
```

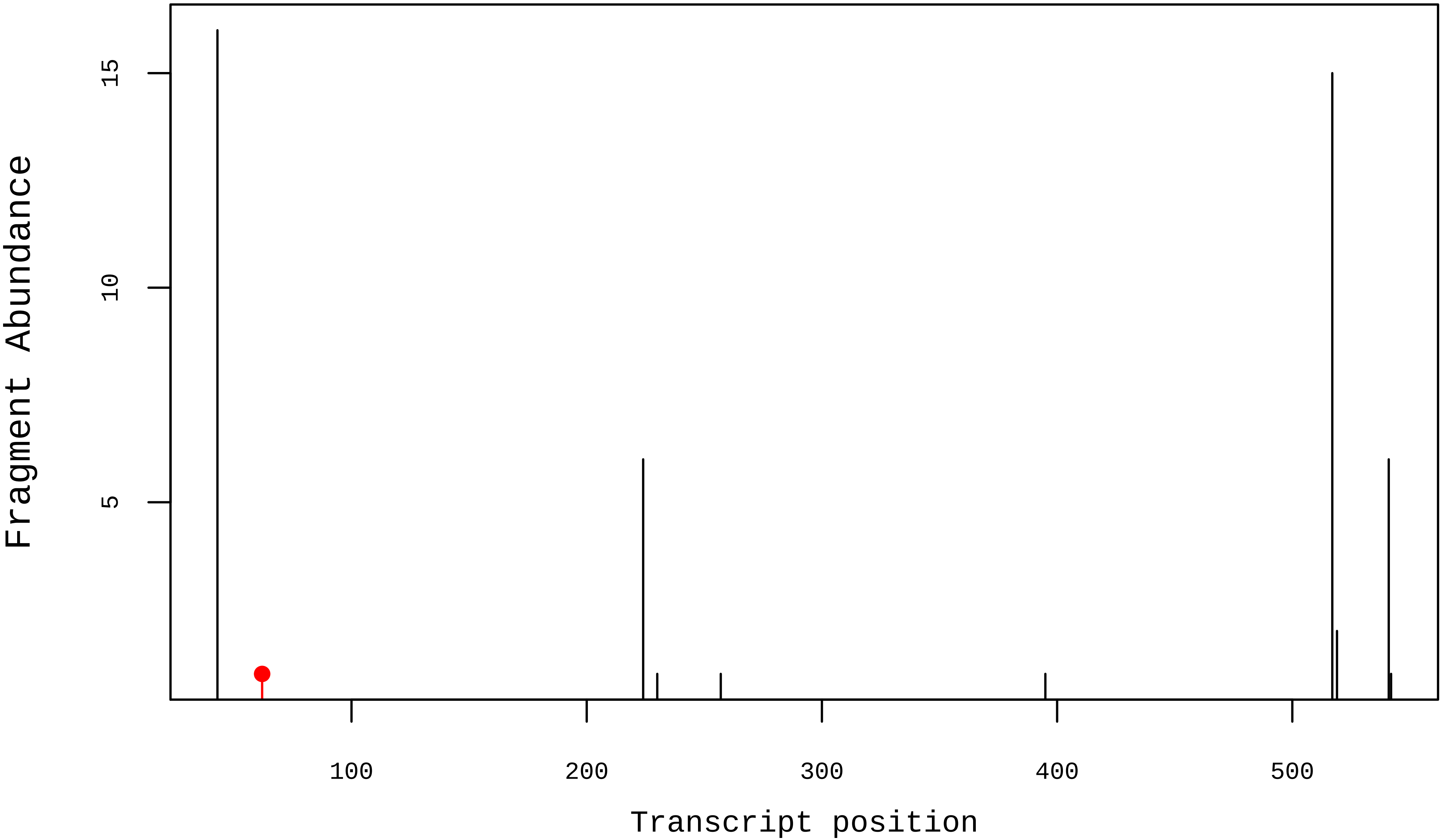

Cleavage site: 62   Tag abundance: 1   Weighted abundance: 0.143   Category: 4
sRNA abundance: 1   Alignment score: 1.5   MFE ratio: 0.872   p−value: 0.041

HORVU5Hr1G015600|HORVU5Hr1G015600.3||276|1709

```
5' GCCGGCCGAAGGGTCGAGTAGGTCGGTGCTCG '3
      ||||||o| ||||||||||||||||
3'    GCCGGTTCCCCAGCTCATCCAGCC       '5
```

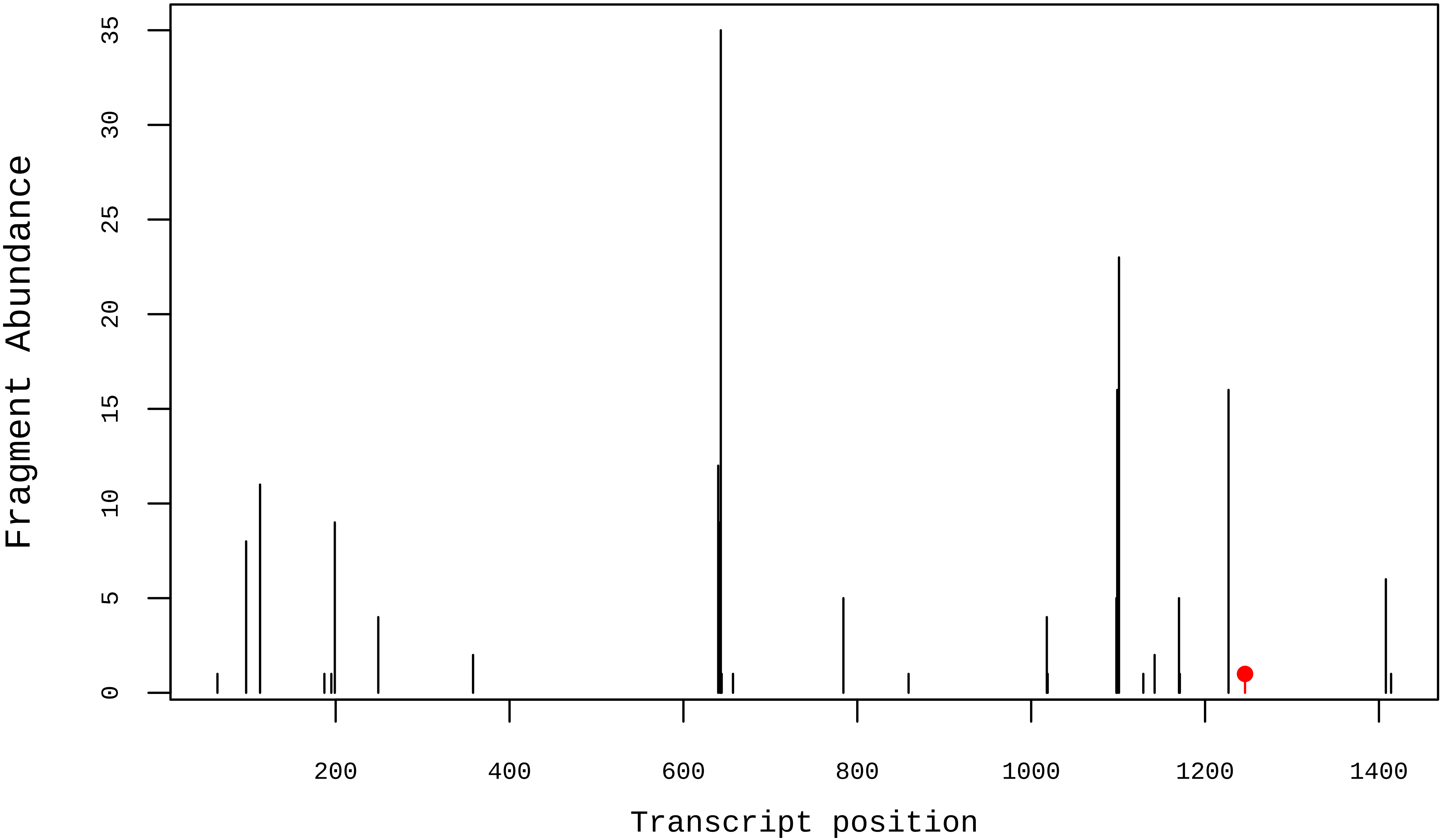

Cleavage site: 1246   Tag abundance: 1   Weighted abundance: 0.143   Category: 4
sRNA abundance: 1   Alignment score: 1.5   MFE ratio: 0.872   p-value: 0.032

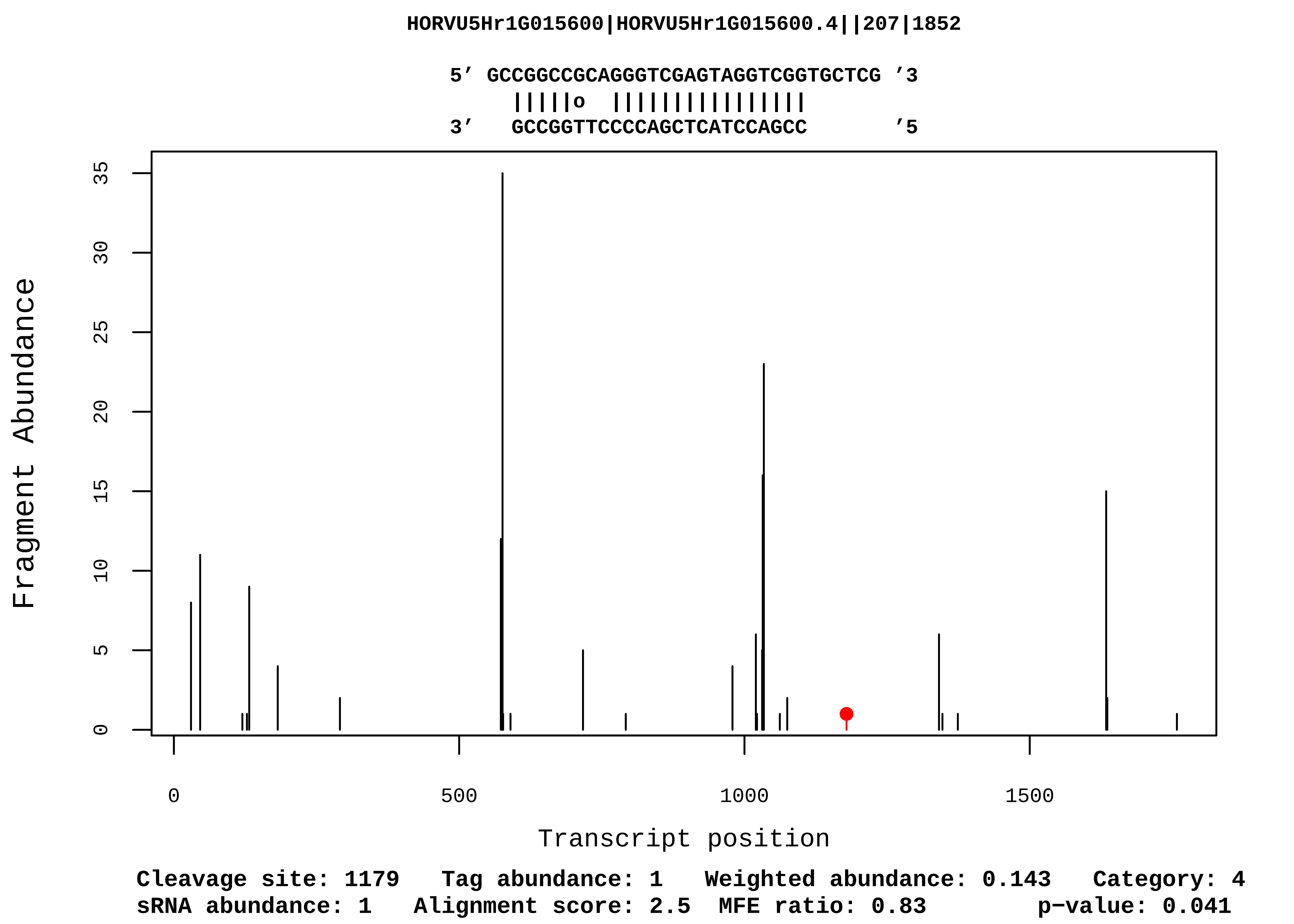

HORVU5Hr1G015600|HORVU5Hr1G015600.4||207|1852
5' GCCGGCCGCAGGGTCGAGTAGGTCGGTGCTCG '3
|||||o ||||||||||||||||
3' GCCGGTTCCCCAGCTCATCCAGCC '5
Fragment Abundance
0
5
10
15
20
25
30
35
0
500
1000
1500
Transcript position
Cleavage site: 1179 Tag abundance: 1 Weighted abundance: 0.143 Category: 4
sRNA abundance: 1 Alignment score: 2.5 MFE ratio: 0.83 p-value: 0.041

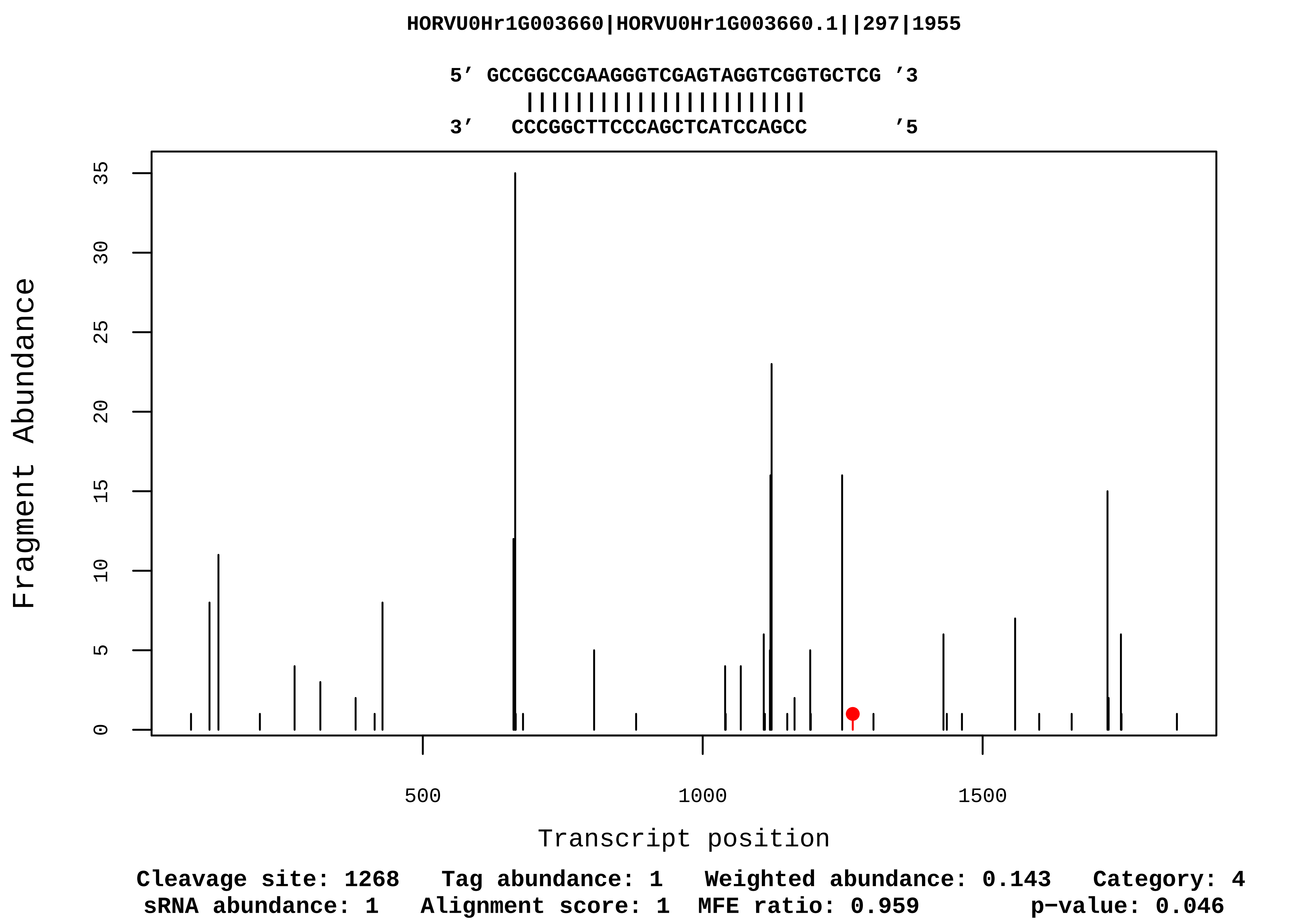

HORVU0Hr1G003660|HORVU0Hr1G003660.1||297|1955
5' GCCGGCCGAAGGGTCGAGTAGGTCGGTGCTCG '3
||||||||||||||||||||||
3' CCCGGCTTCCCAGCTCATCCAGCC '5
Fragment Abundance
0
5
10
15
20
25
30
35
500
1000
1500
Transcript position
Cleavage site: 1268 Tag abundance: 1 Weighted abundance: 0.143 Category: 4
sRNA abundance: 1 Alignment score: 1 MFE ratio: 0.959 p-value: 0.046

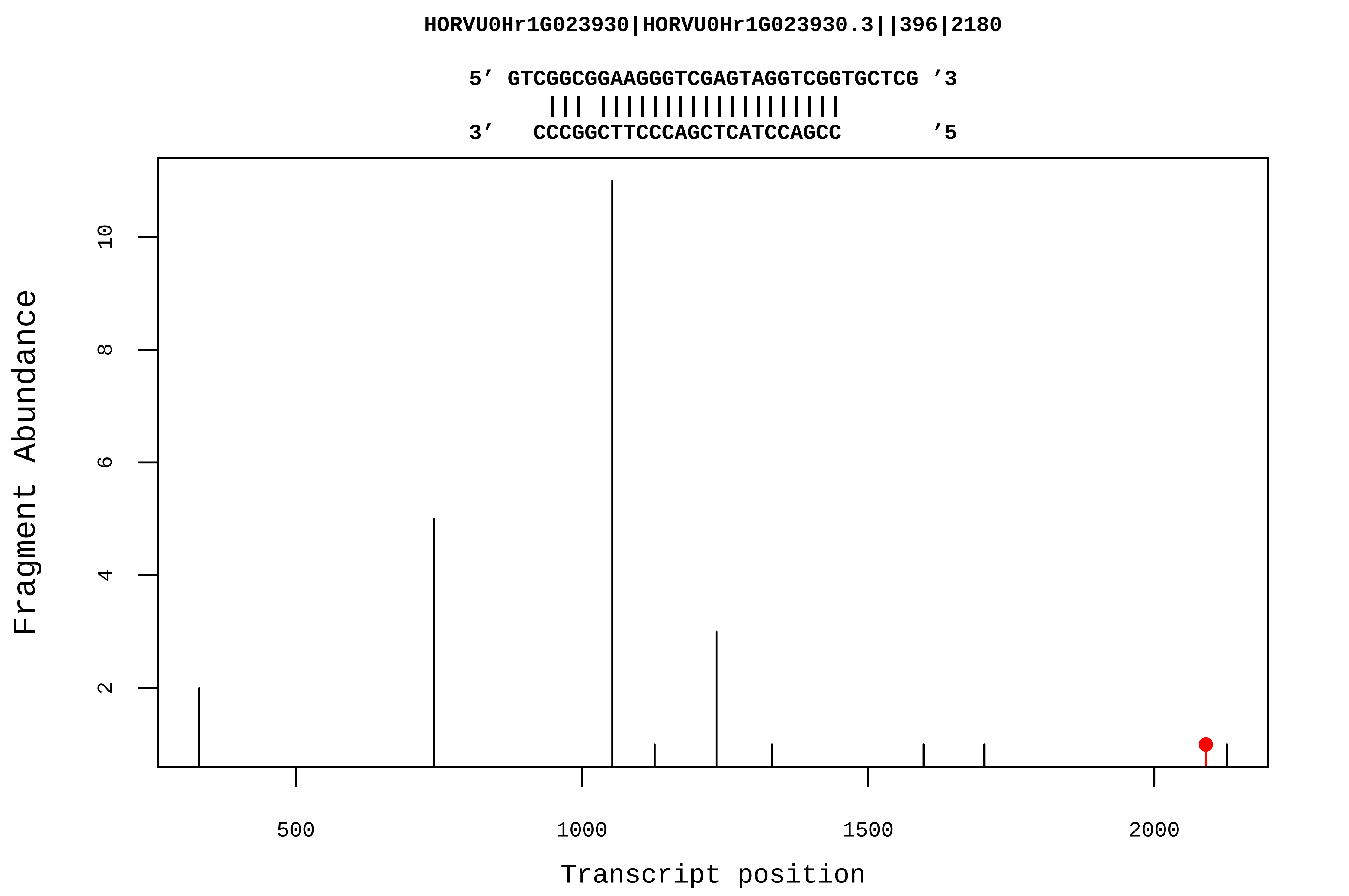

Cleavage site: 2090 Tag abundance: 1 Weighted abundance: 0.143 Category: 4
sRNA abundance: 1 Alignment score: 2 MFE ratio: 0.88 p-value: 0.019

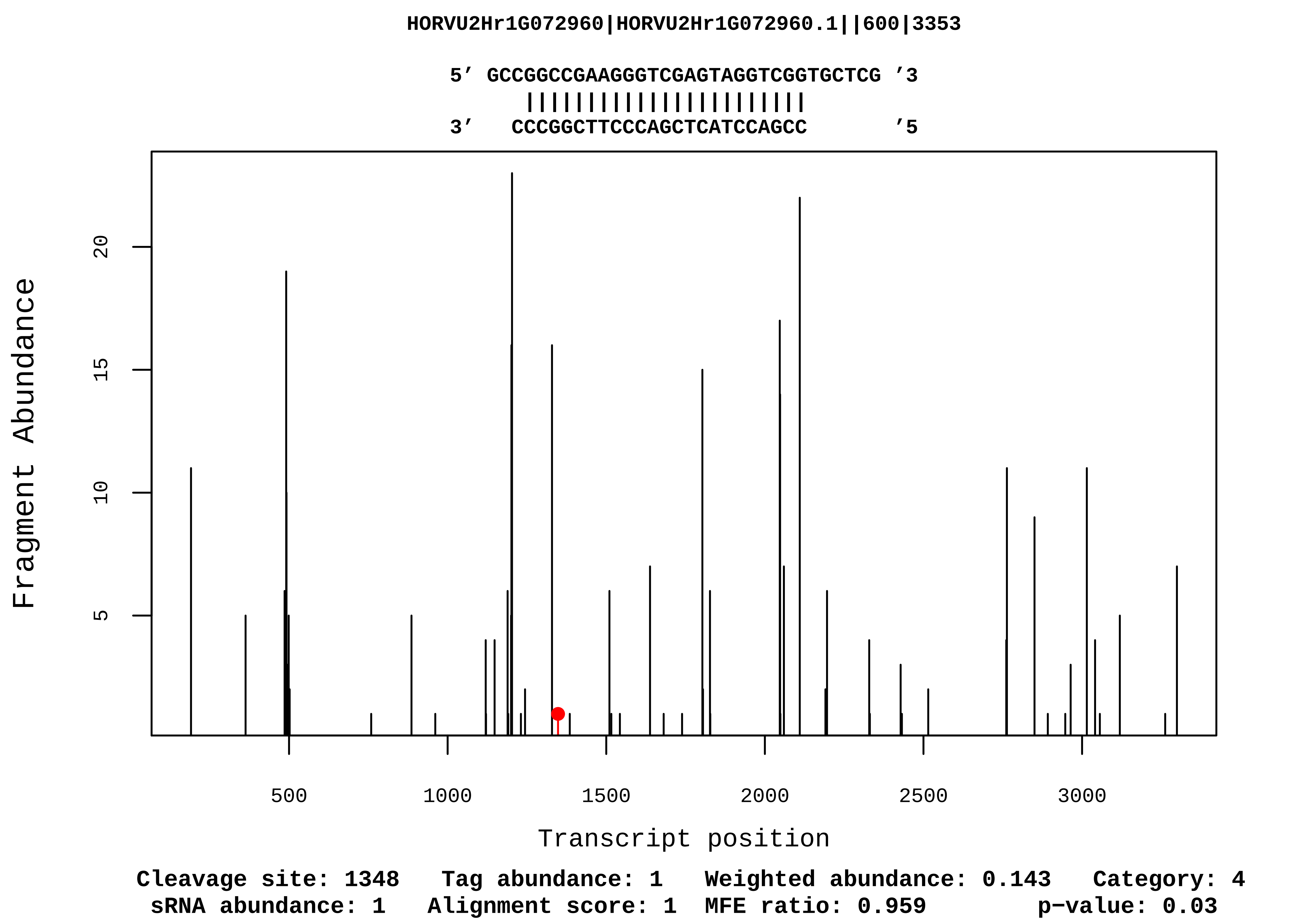

HORVU2Hr1G072960|HORVU2Hr1G072960.1||600|3353
5′ GCCGGCCGAAGGGTCGAGTAGGTCGGTGCTCG ′3
||||||||||||||||||||||||
3′ CCCGGCTTCCCAGCTCATCCAGCC ′5
Fragment Abundance
5
10
15
20
500
1000
1500
2000
2500
3000
Transcript position
Cleavage site: 1348 Tag abundance: 1 Weighted abundance: 0.143 Category: 4
sRNA abundance: 1 Alignment score: 1 MFE ratio: 0.959 p-value: 0.03

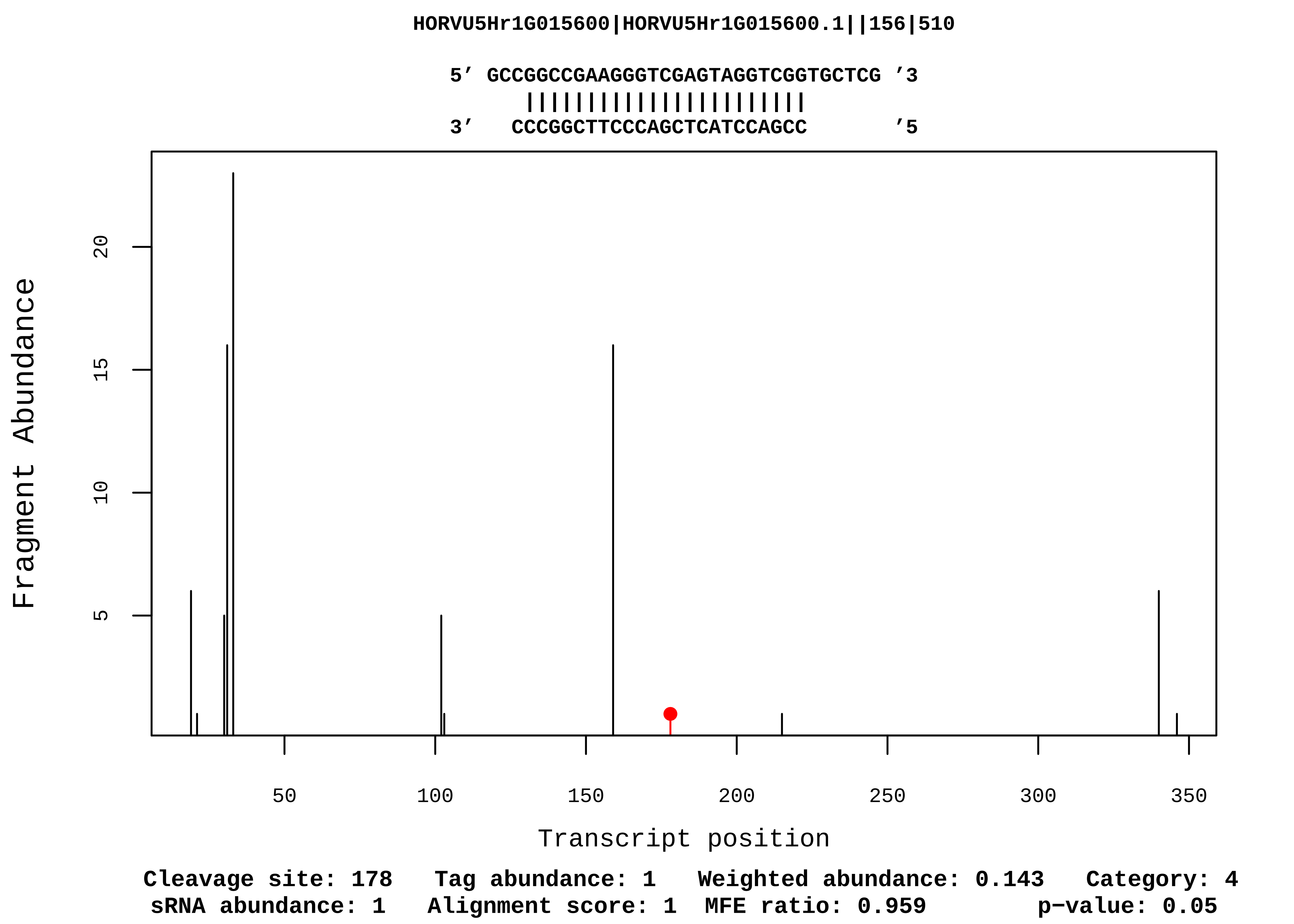
HORVU5Hr1G015600|HORVU5Hr1G015600.1||156|510
5′ GCCGGCCGAAGGGTCGAGTAGGTCGGTGCTCG ′3
||||||||||||||||||||||
3′ CCCGGCTTCCCAGCTCATCCAGCC ′5
Fragment Abundance
5
10
15
20
50
100
150
200
250
300
350
Transcript position
Cleavage site: 178 Tag abundance: 1 Weighted abundance: 0.143 Category: 4
sRNA abundance: 1 Alignment score: 1 MFE ratio: 0.959 p-value: 0.05

HORVU5Hr1G015600|HORVU5Hr1G015600.2||231|617

```
5' GCCGGCCGAAGGGTCGAGTAGGTCGGTGCTCG '3
      ||||||||||||||||||||||
3'   CCCGGCTTCCCAGCTCATCCAGCC       '5
```

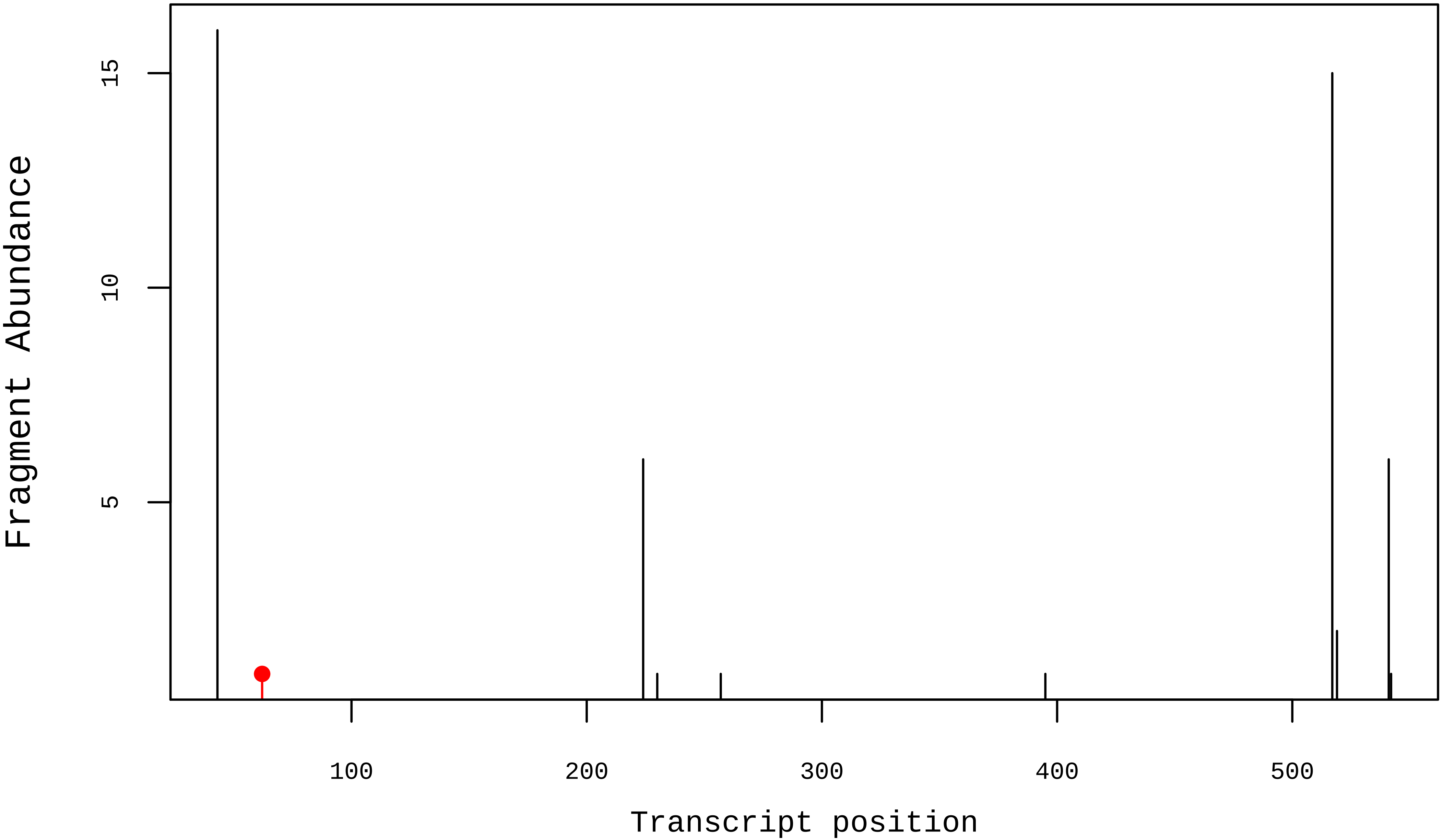

Cleavage site: 62 Tag abundance: 1 Weighted abundance: 0.143 Category: 4
sRNA abundance: 1 Alignment score: 1 MFE ratio: 0.959 p-value: 0.041

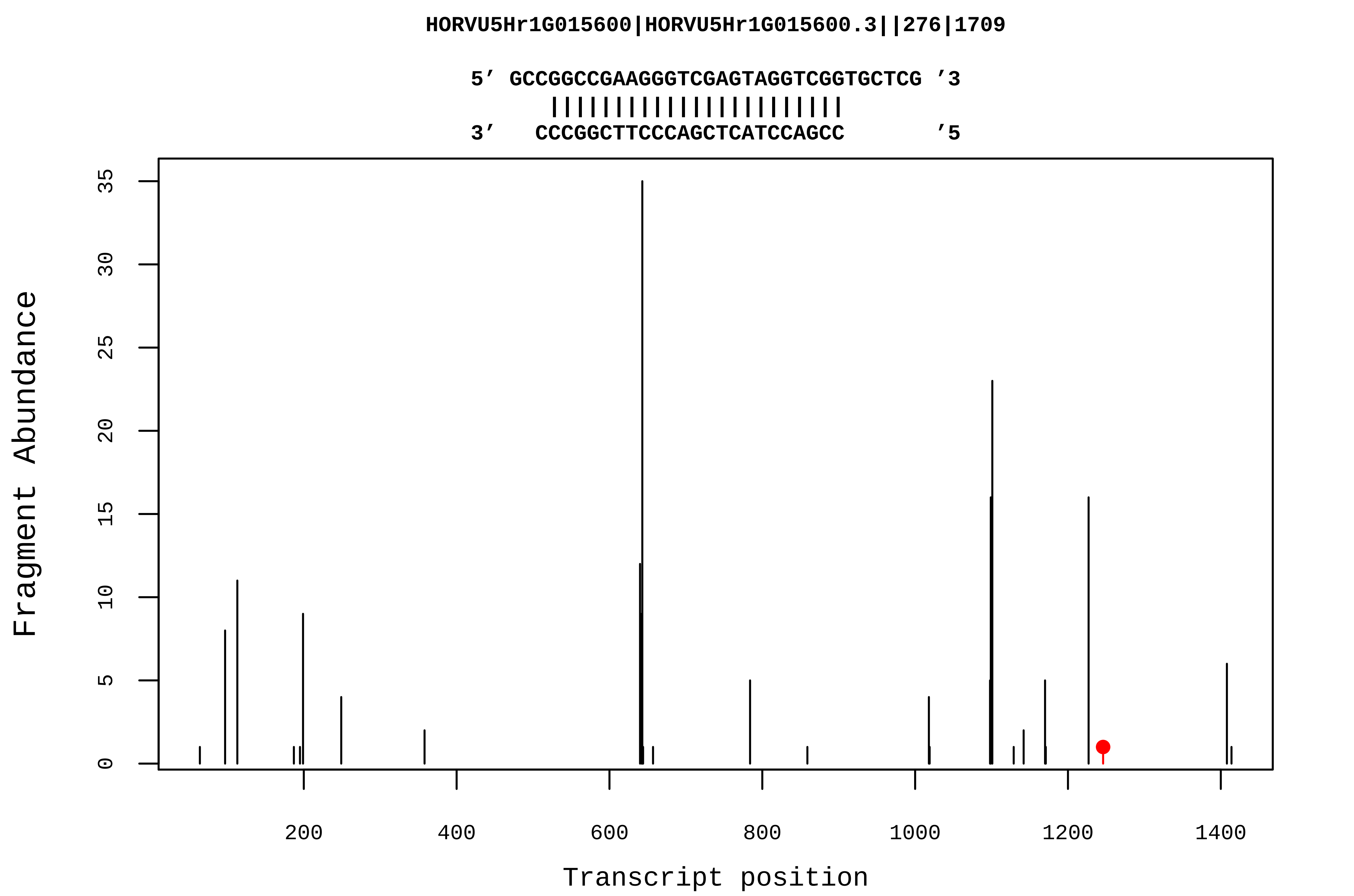

Cleavage site: 1246   Tag abundance: 1   Weighted abundance: 0.143   Category: 4
sRNA abundance: 1   Alignment score: 1   MFE ratio: 0.959   p-value: 0.032

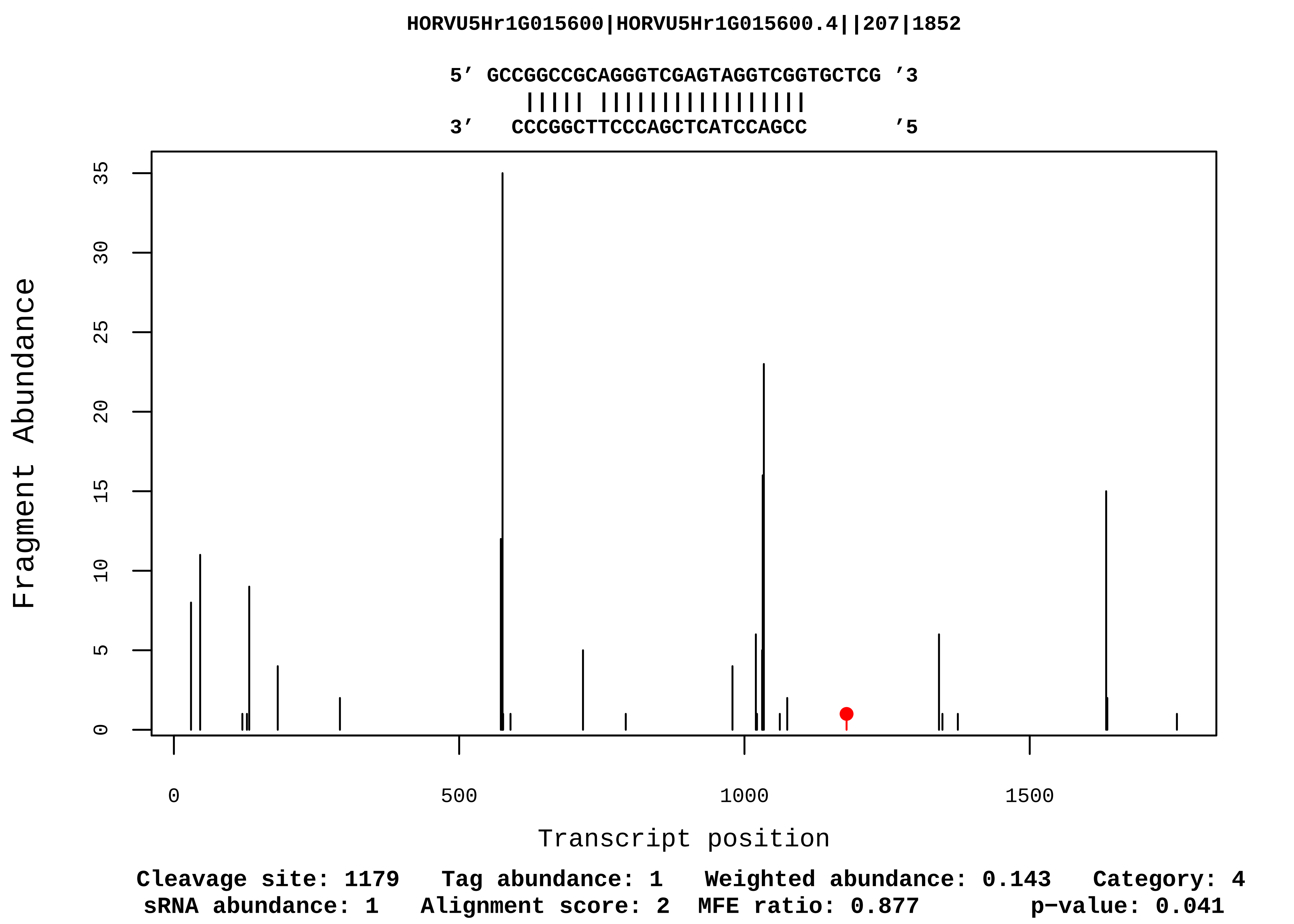

HORVU5Hr1G015600|HORVU5Hr1G015600.4||207|1852
5' GCCGGCCGCAGGGTCGAGTAGGTCGGTGCTCG '3
||||| |||||||||||||||||
3' CCCGGCTTCCCAGCTCATCCAGCC '5
Fragment Abundance
0
5
10
15
20
25
30
35
0
500
1000
1500
Transcript position
Cleavage site: 1179 Tag abundance: 1 Weighted abundance: 0.143 Category: 4
sRNA abundance: 1 Alignment score: 2 MFE ratio: 0.877 p-value: 0.041

HORVU5Hr1G041250|HORVU5Hr1G041250.1||1638|2005

```
5' ATTACCAGTCC-AACGAGAGTGATCGTCACAGC '3
        ||o|  |||||||||||||o|
3'      TCGGTTTTGCTCTCACTAGTA        '5
```

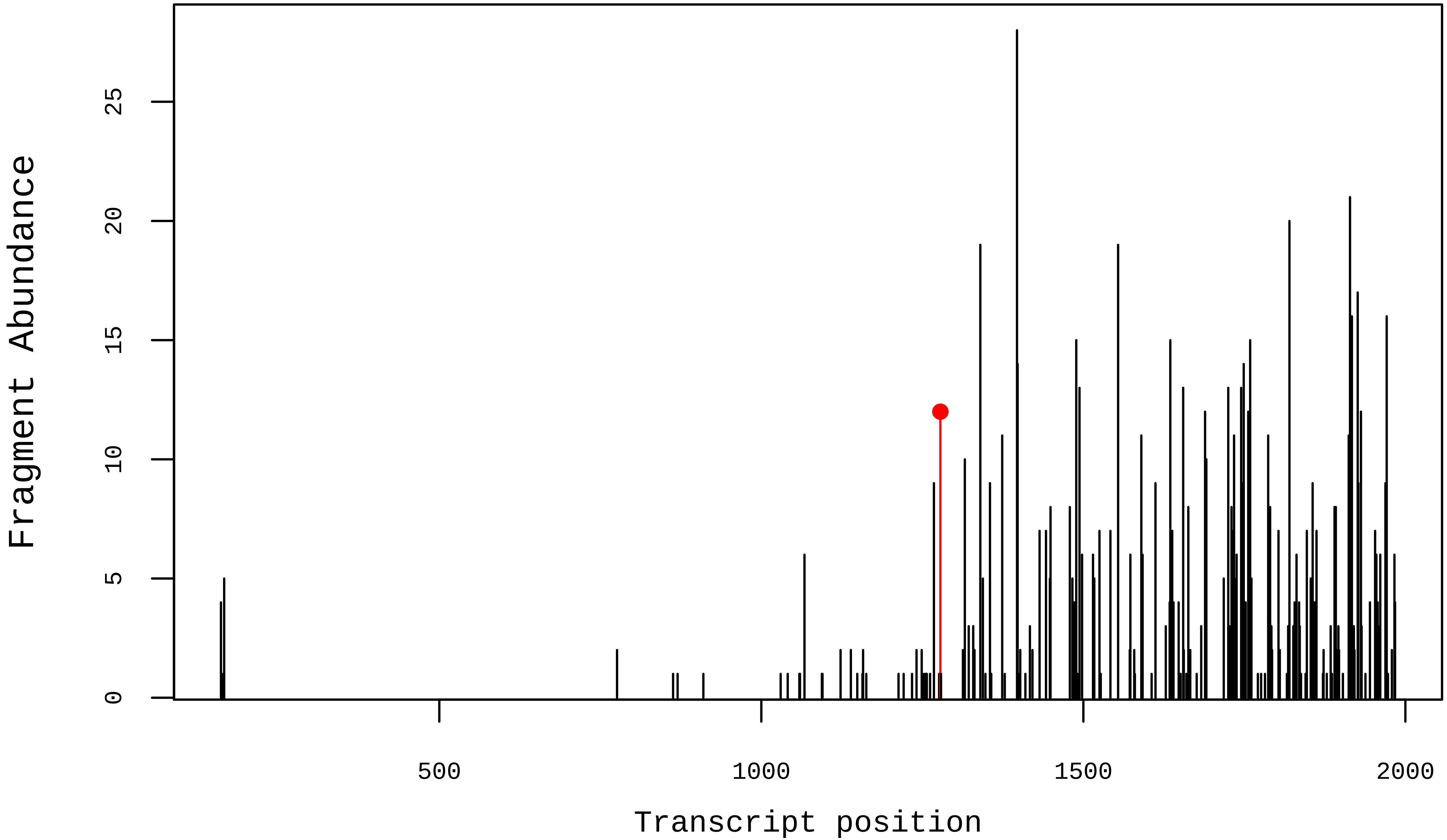

Cleavage site: 1278   Tag abundance: 12   Weighted abundance: 12   Category: 2
sRNA abundance: 1   Alignment score: 3.5   MFE ratio: 0.744   p-value: 0.026

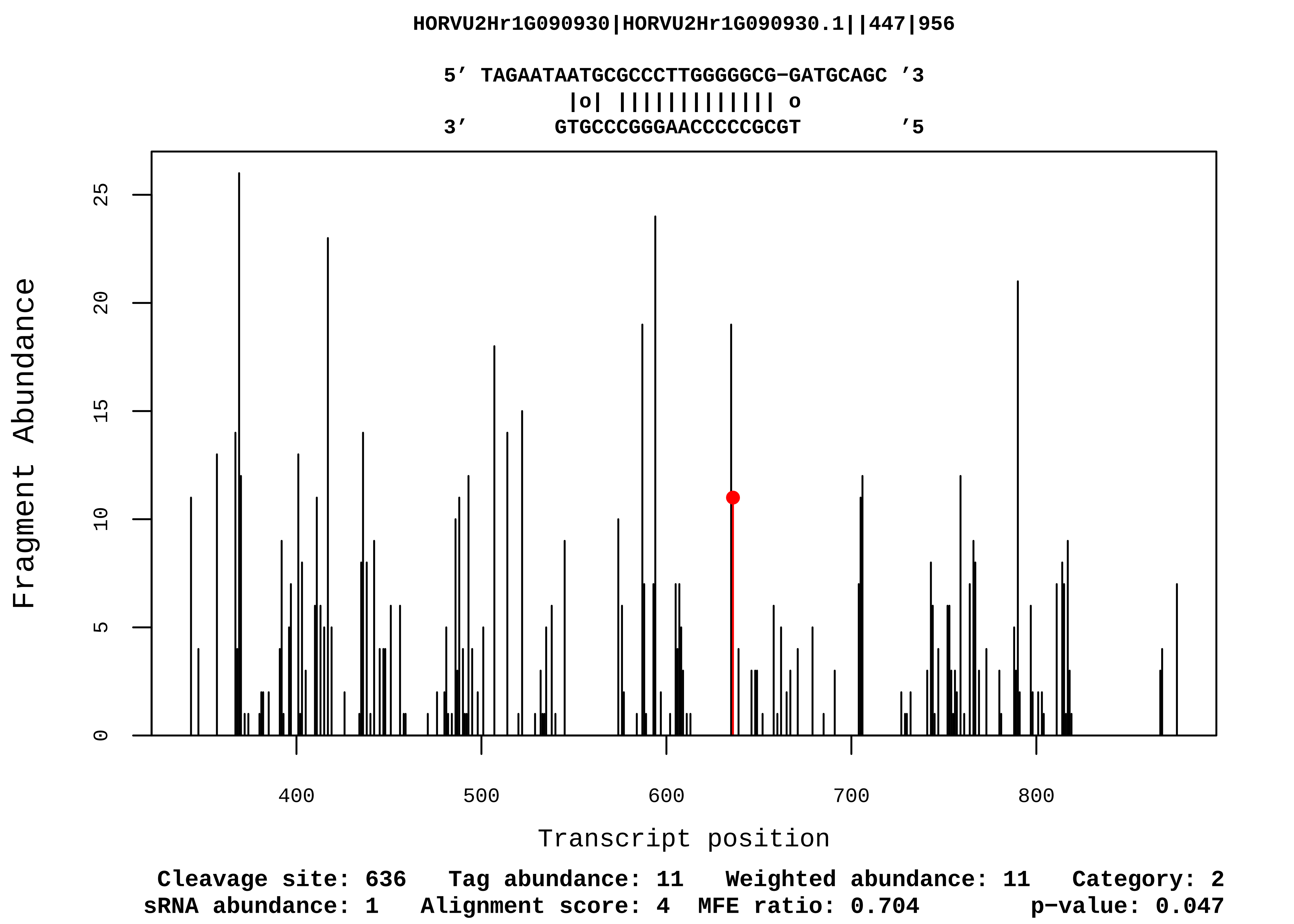
HORVU2Hr1G090930|HORVU2Hr1G090930.1||447|956
5' TAGAATAATGCGCCCTTGGGGGCG-GATGCAGC '3
|o| ||||||||||||| o
3' GTGCCCGGGAACCCCCGCGT '5
Fragment Abundance
0
5
10
15
20
25
400
500
600
700
800
Transcript position
Cleavage site: 636 Tag abundance: 11 Weighted abundance: 11 Category: 2
sRNA abundance: 1 Alignment score: 4 MFE ratio: 0.704 p-value: 0.047

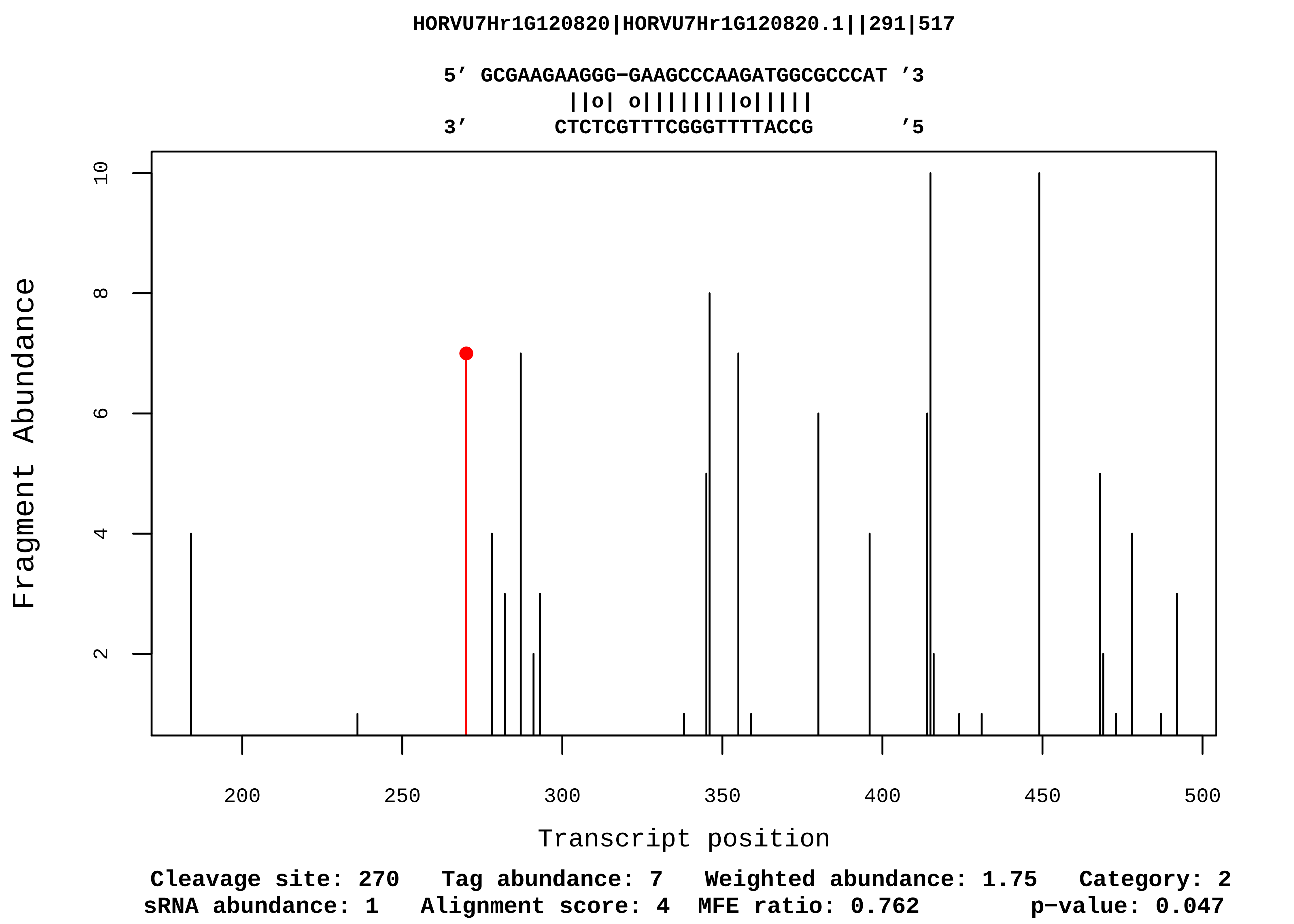

HORVU7Hr1G120820|HORVU7Hr1G120820.1||291|517
5' GCGAAGAAGGG-GAAGCCCAAGATGGCGCCCAT '3
||o| o||||||||o|||||
3' CTCTCGTTTCGGGTTTTACCG '5
Fragment Abundance
Transcript position
2
4
6
8
10
200
250
300
350
400
450
500
Cleavage site: 270 Tag abundance: 7 Weighted abundance: 1.75 Category: 2
sRNA abundance: 1 Alignment score: 4 MFE ratio: 0.762 p-value: 0.047

HORVU7Hr1G120820|HORVU7Hr1G120820.2||345|683

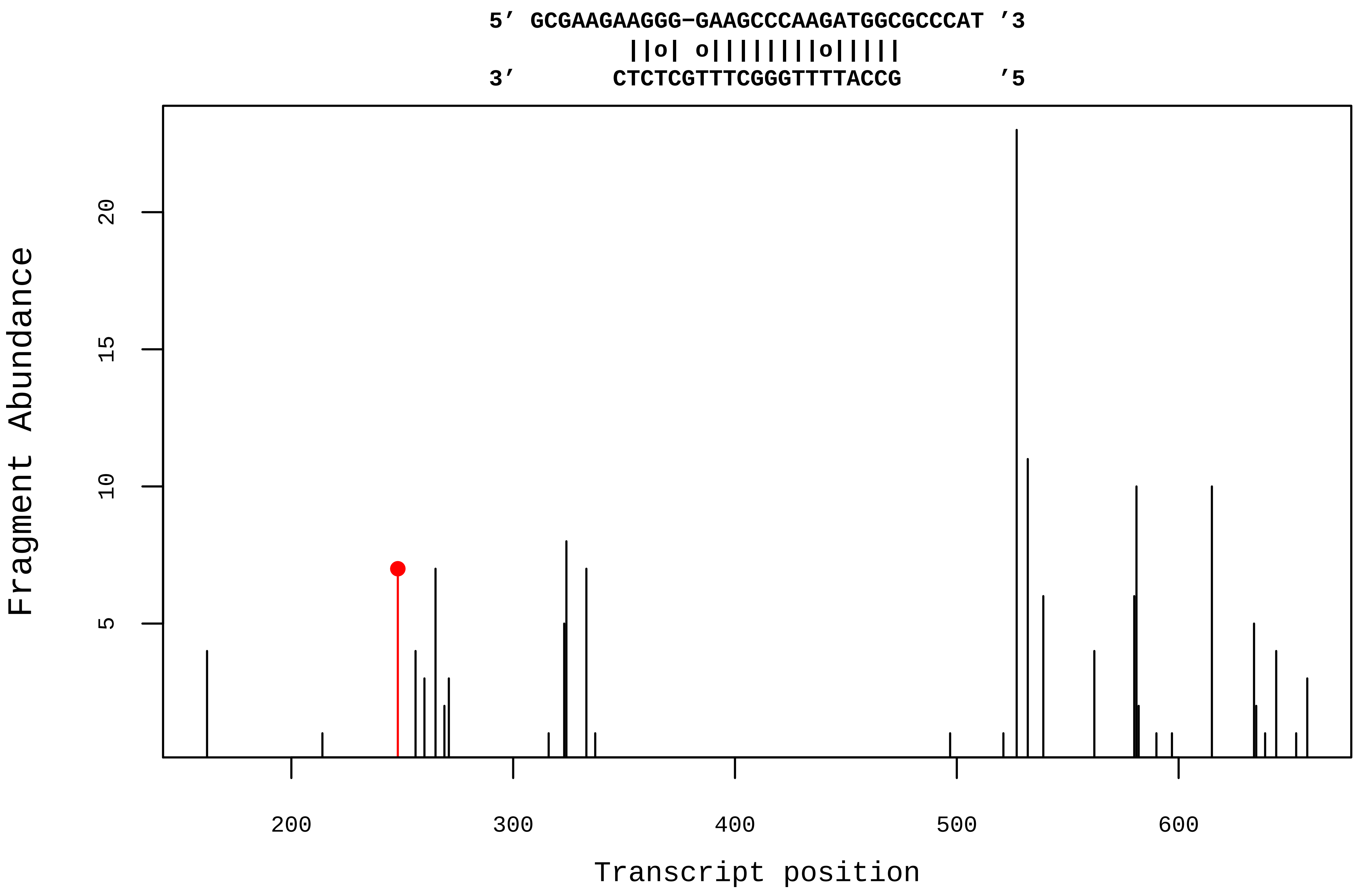

Cleavage site: 248 Tag abundance: 7 Weighted abundance: 1.75 Category: 2
sRNA abundance: 1 Alignment score: 4 MFE ratio: 0.762 p-value: 0.042

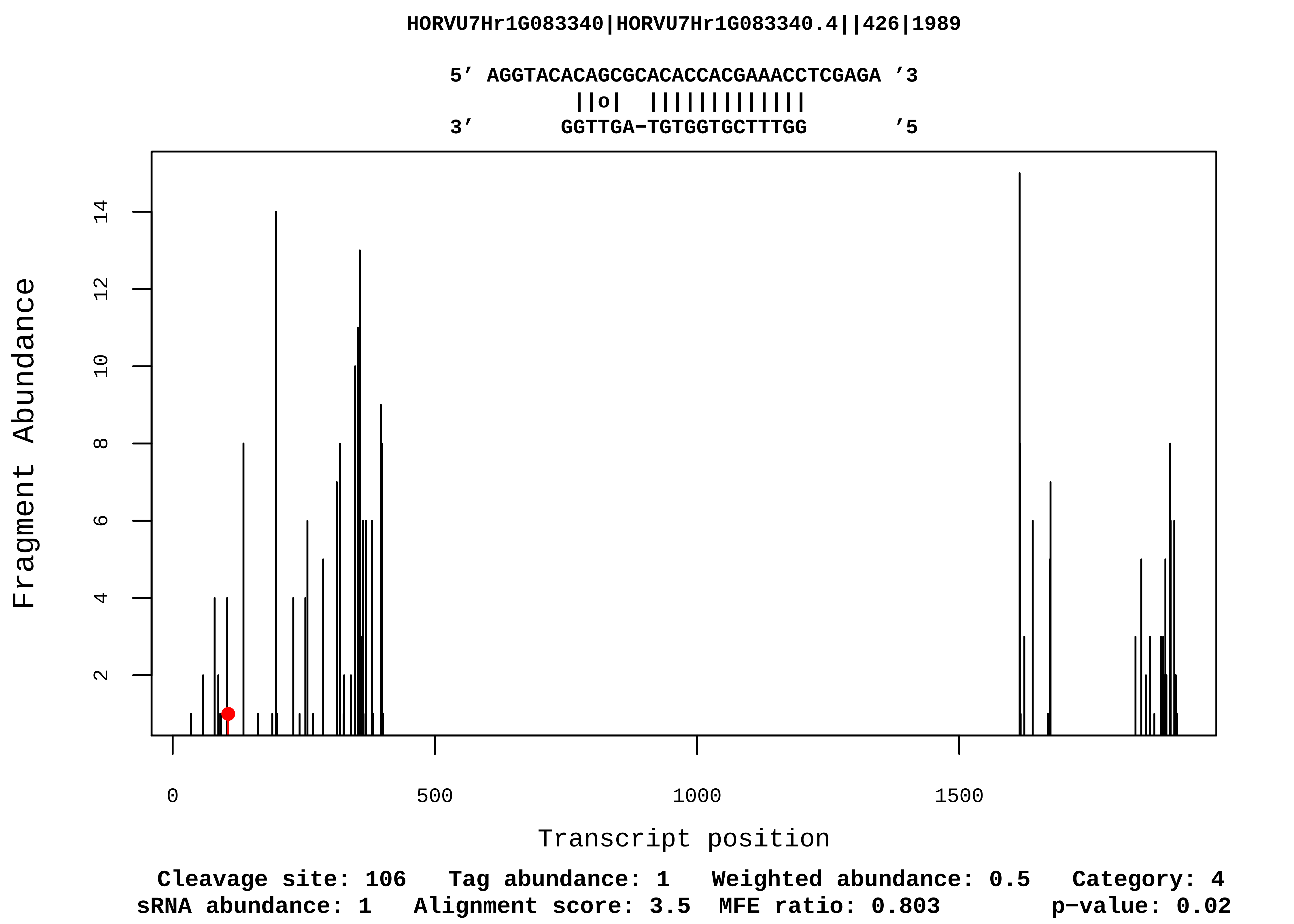
HORVU7Hr1G083340|HORVU7Hr1G083340.4||426|1989
5′ AGGTACACAGCGCACACCACGAAACCTCGAGA ′3
||o| |||||||||||||
3′ GGTTGA−TGTGGTGCTTTGG ′5
Fragment Abundance
2
4
6
8
10
12
14
0
500
1000
1500
Transcript position
Cleavage site: 106   Tag abundance: 1   Weighted abundance: 0.5   Category: 4
sRNA abundance: 1   Alignment score: 3.5   MFE ratio: 0.803   p−value: 0.02

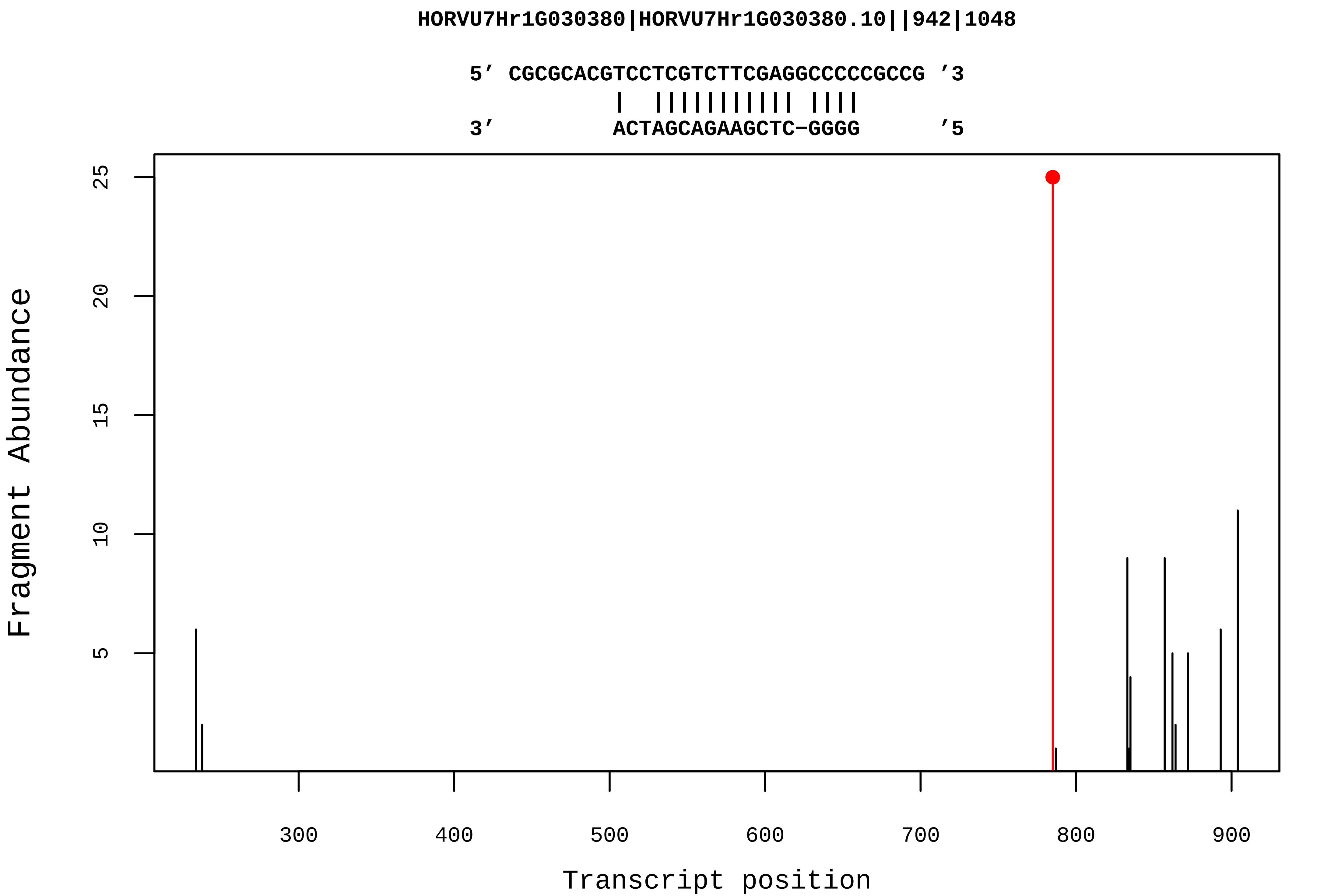

Cleavage site: 785 Tag abundance: 25 Weighted abundance: 1.562 Category: 0
sRNA abundance: 1 Alignment score: 4 MFE ratio: 0.758 p-value: 0.032

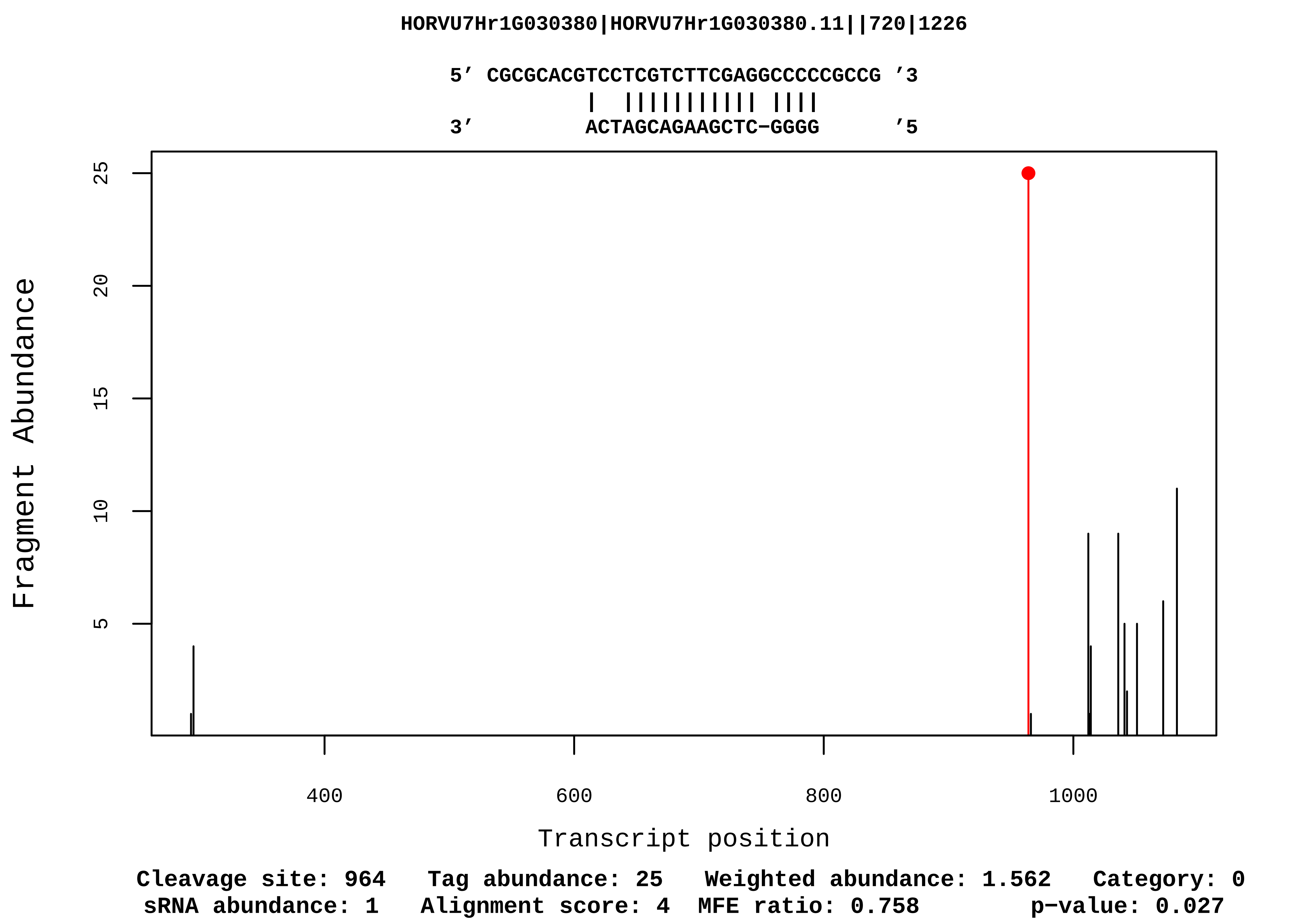

HORVU7Hr1G030380|HORVU7Hr1G030380.11||720|1226
5′ CGCGCACGTCCTCGTCTTCGAGGCCCCCGCCG ′3
| |||||||||||| ||||
3′ ACTAGCAGAAGCTC−GGGG ′5
Fragment Abundance
5
10
15
20
25
400
600
800
1000
Transcript position
Cleavage site: 964 Tag abundance: 25 Weighted abundance: 1.562 Category: 0
sRNA abundance: 1 Alignment score: 4 MFE ratio: 0.758 p-value: 0.027

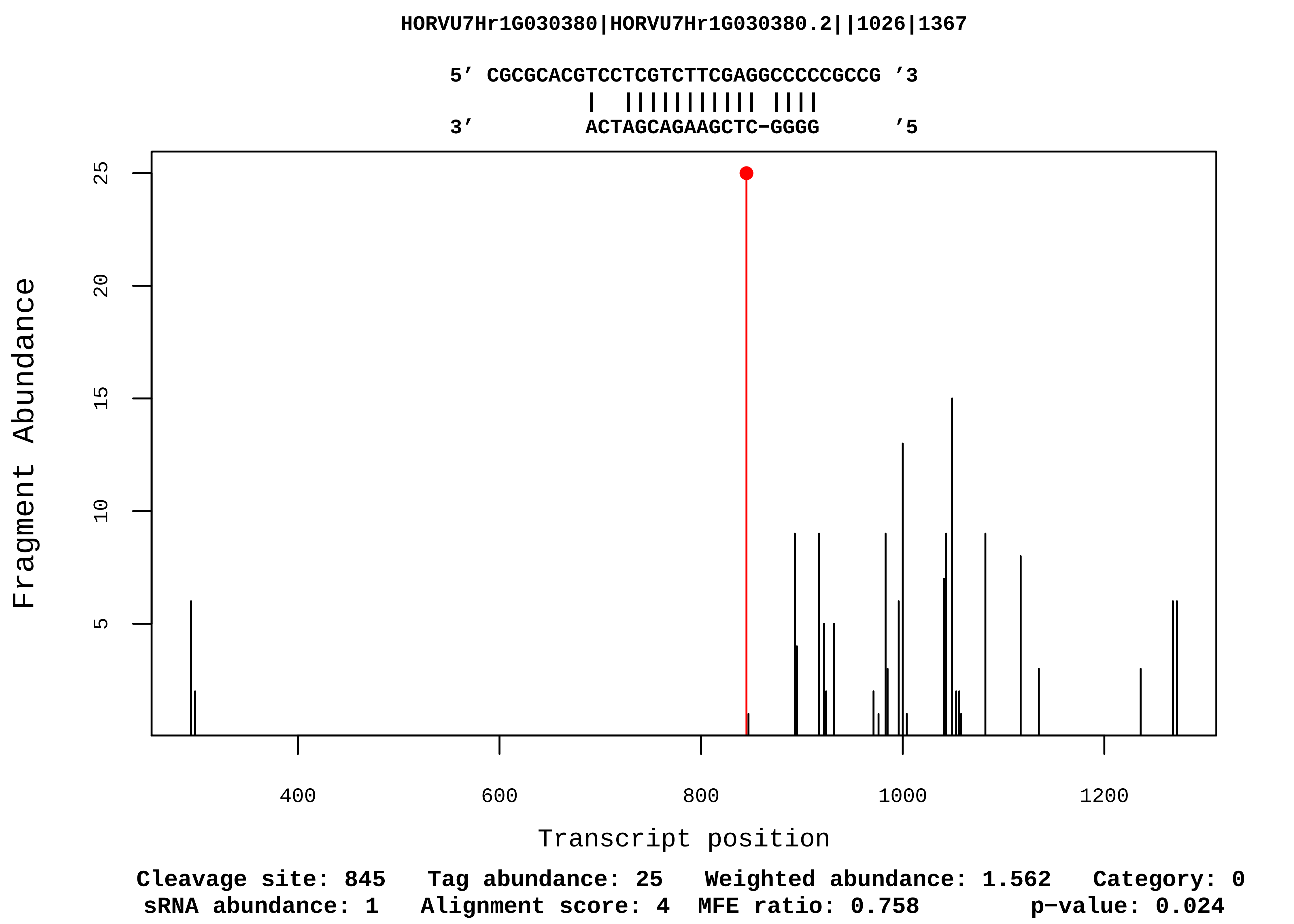

HORVU7Hr1G030380|HORVU7Hr1G030380.2||1026|1367
5’ CGCGCACGTCCTCGTCTTCGAGGCCCCCGCCG ’3
3’ ACTAGCAGAAGCTC-GGGG ’5
Fragment Abundance
5
10
15
20
25
400
600
800
1000
1200
Transcript position
Cleavage site: 845 Tag abundance: 25 Weighted abundance: 1.562 Category: 0
sRNA abundance: 1 Alignment score: 4 MFE ratio: 0.758 p-value: 0.024

HORVU7Hr1G030380|HORVU7Hr1G030380.3||1026|1364

```
5' CGCGCACGTCCTCGTCTTCGAGGCCCCCGCCG '3
            |  |||||||||| ||||
3'          ACTAGCAGAAGCTC-GGGG      '5
```

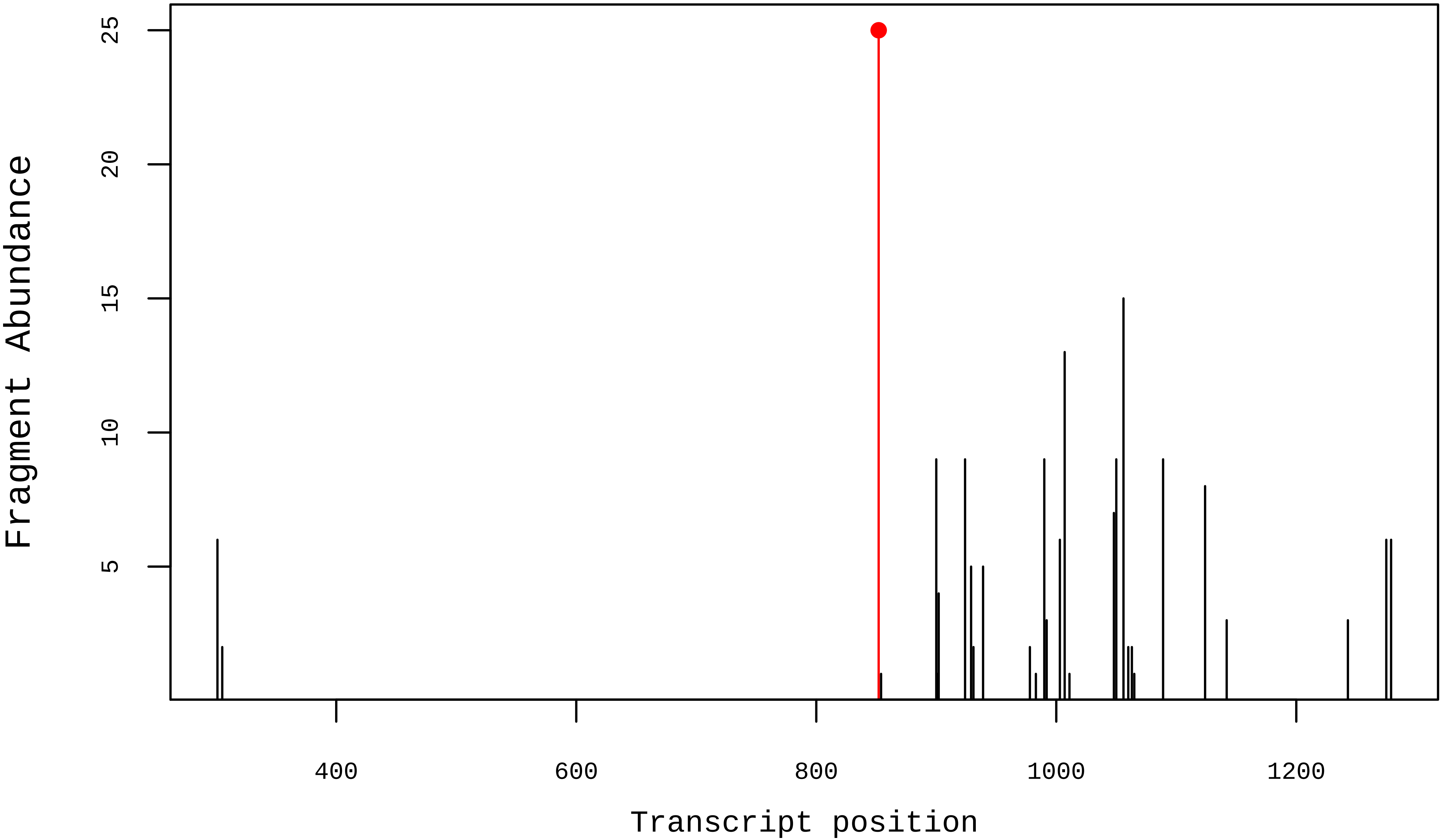

Cleavage site: 852   Tag abundance: 25   Weighted abundance: 1.562   Category: 0
sRNA abundance: 1   Alignment score: 4   MFE ratio: 0.758   p-value: 0.024

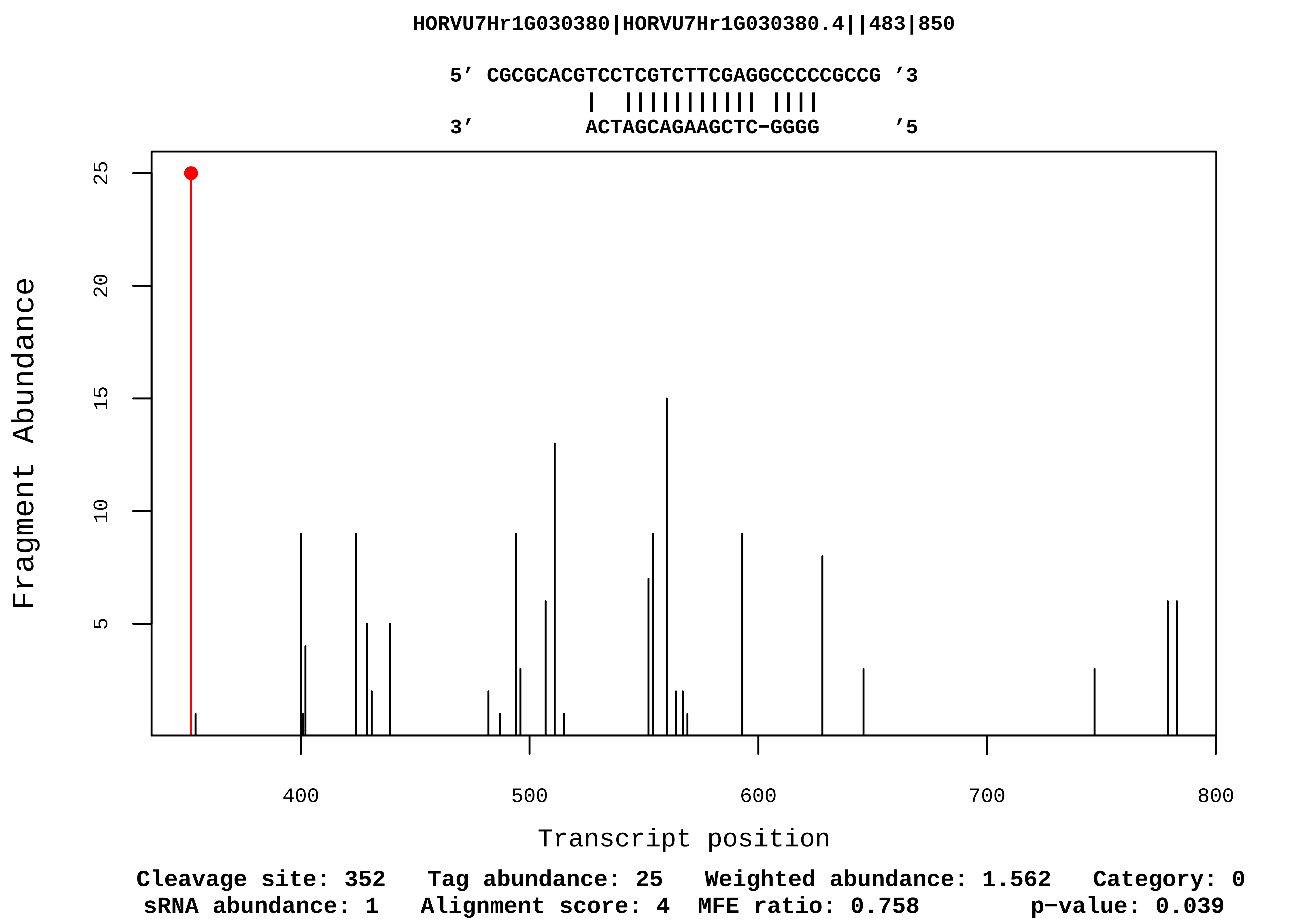
HORVU7Hr1G030380|HORVU7Hr1G030380.4||483|850
5' CGCGCACGTCCTCGTCTTCGAGGCCCCCGCCG '3
3' ACTAGCAGAAGCTC-GGGG '5
Fragment Abundance
Transcript position
25
20
15
10
5
400
500
600
700
800
Cleavage site: 352 Tag abundance: 25 Weighted abundance: 1.562 Category: 0
sRNA abundance: 1 Alignment score: 4 MFE ratio: 0.758 p-value: 0.039

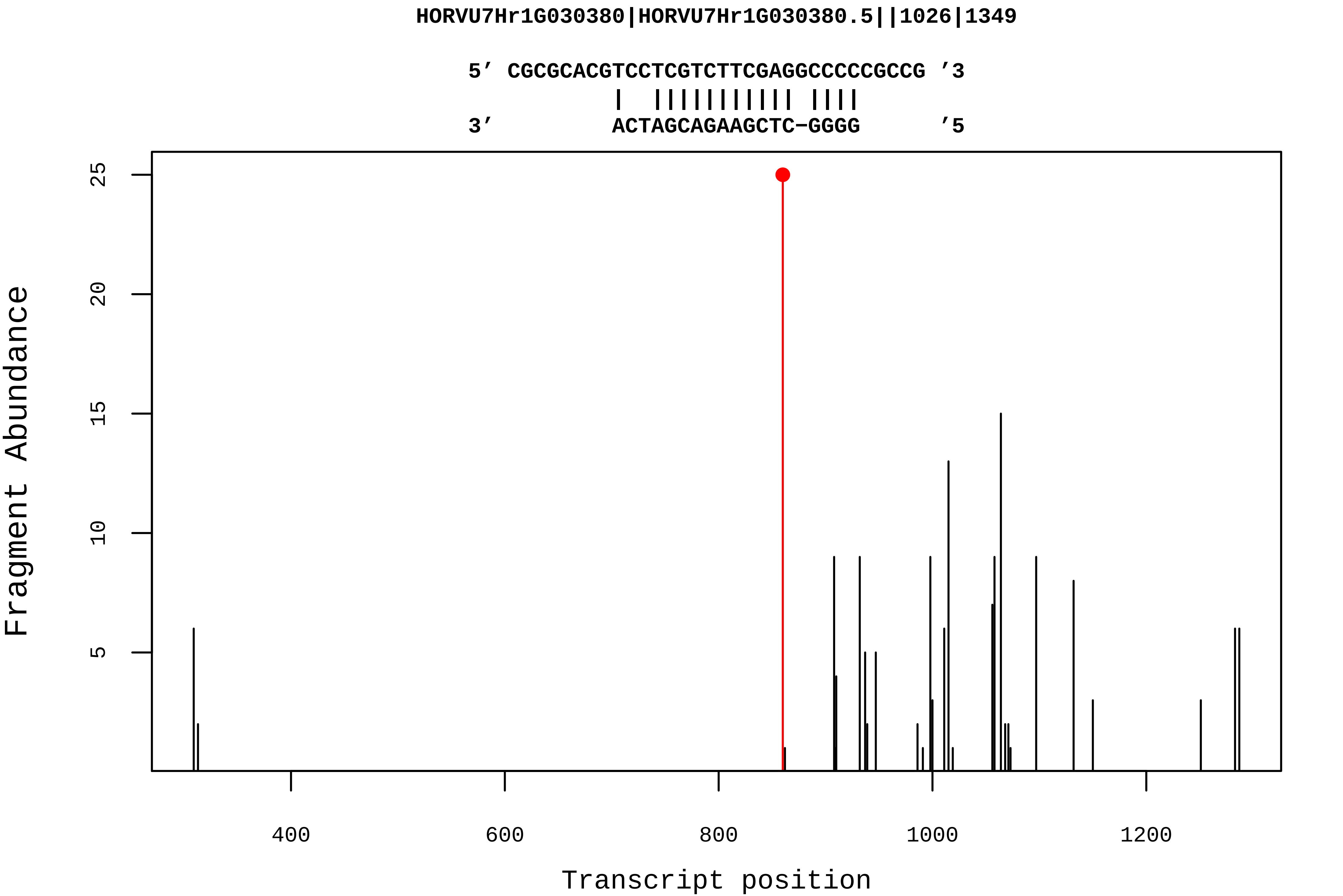

Cleavage site: 860 Tag abundance: 25 Weighted abundance: 1.562 Category: 0
sRNA abundance: 1 Alignment score: 4 MFE ratio: 0.758 p-value: 0.025

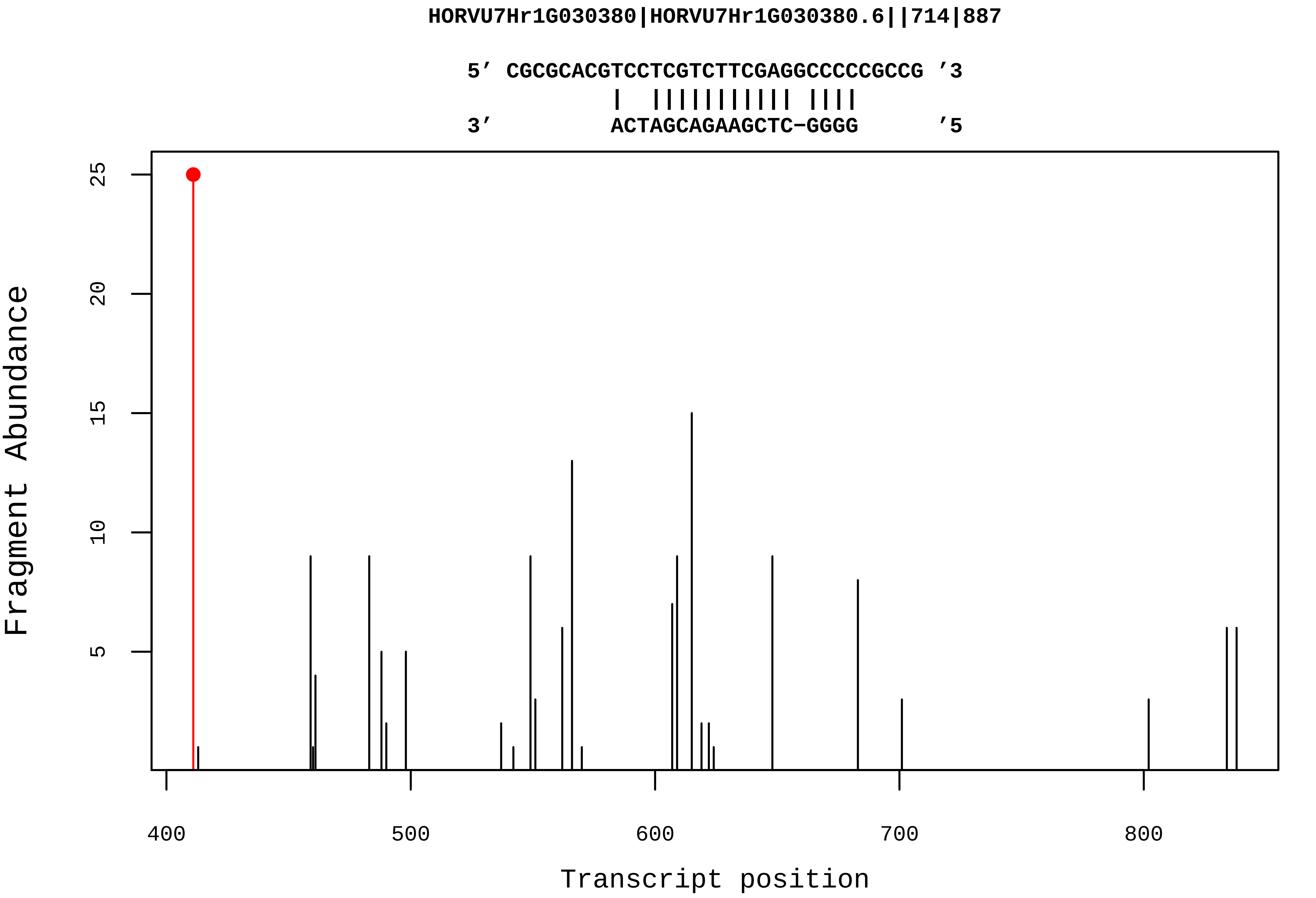

Cleavage site: 411 Tag abundance: 25 Weighted abundance: 1.562 Category: 0
sRNA abundance: 1 Alignment score: 4 MFE ratio: 0.758 p-value: 0.037

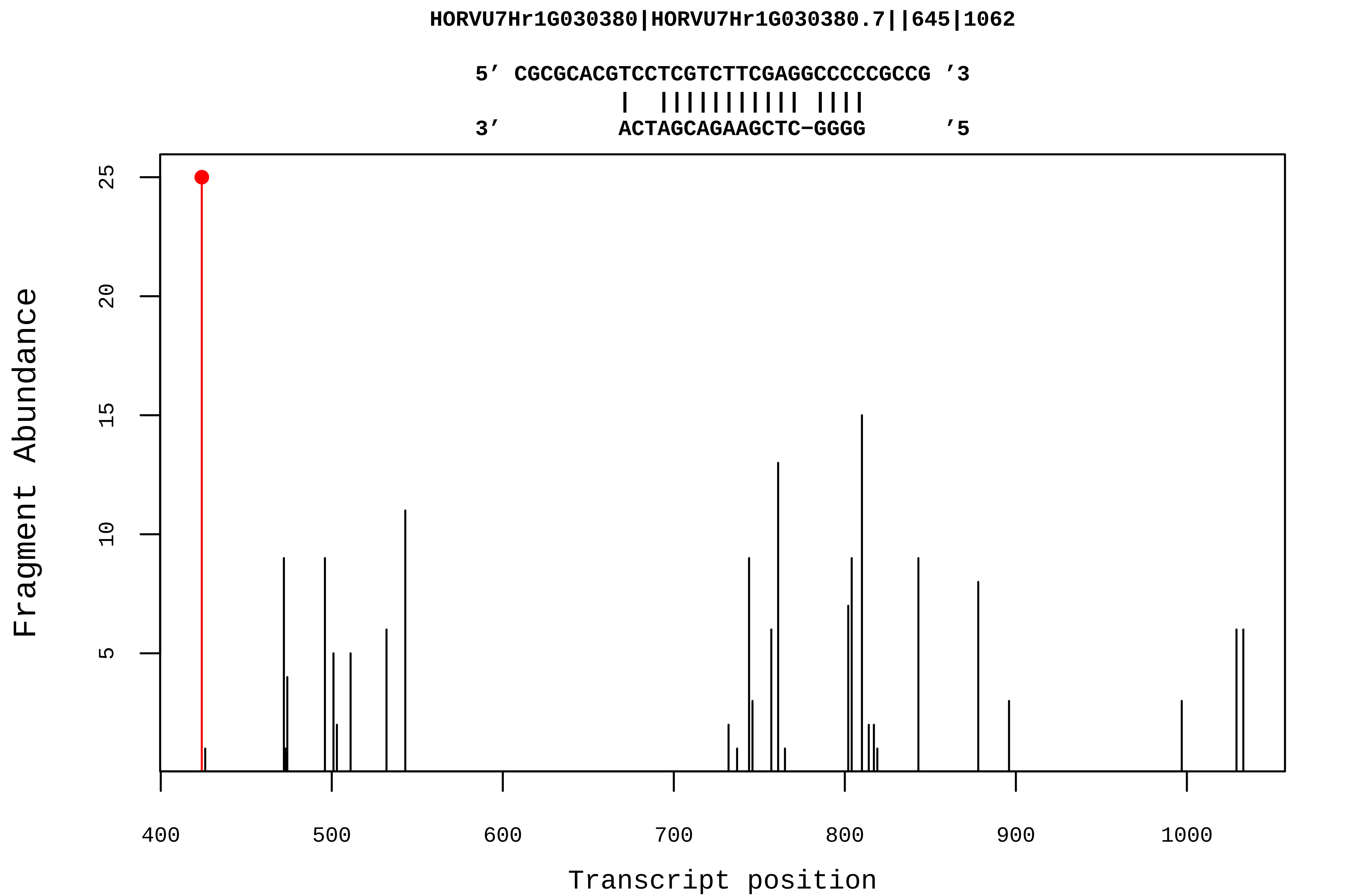

Cleavage site: 424 Tag abundance: 25 Weighted abundance: 1.562 Category: 0
sRNA abundance: 1 Alignment score: 4 MFE ratio: 0.758 p-value: 0.031

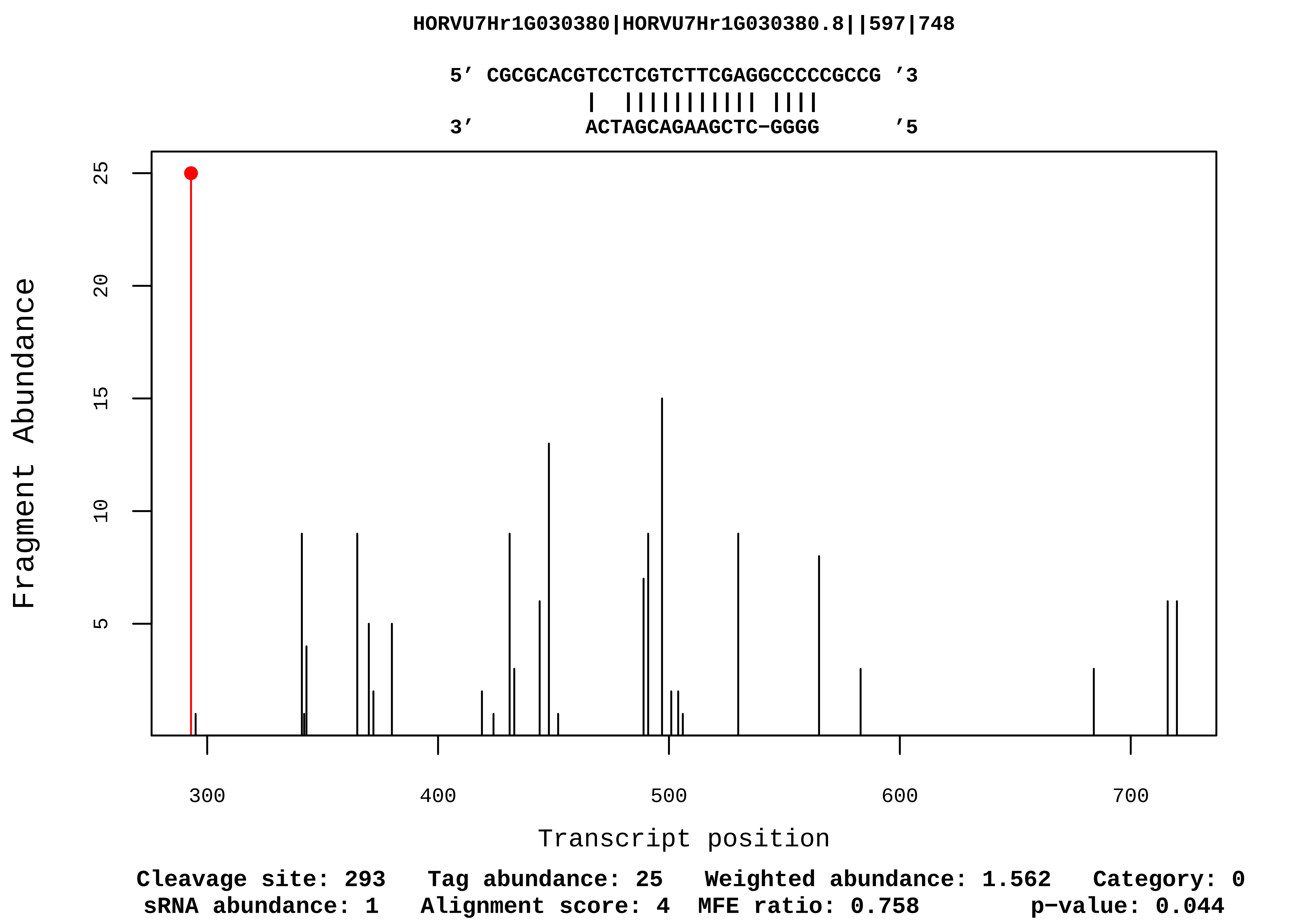
HORVU7Hr1G030380|HORVU7Hr1G030380.8||597|748
5' CGCGCACGTCCTCGTCTTCGAGGCCCCCGCCG '3
| |||||||||||| ||||
3' ACTAGCAGAAGCTC-GGGG '5
Fragment Abundance
5
10
15
20
25
300
400
500
600
700
Transcript position
Cleavage site: 293 Tag abundance: 25 Weighted abundance: 1.562 Category: 0
sRNA abundance: 1 Alignment score: 4 MFE ratio: 0.758 p-value: 0.044

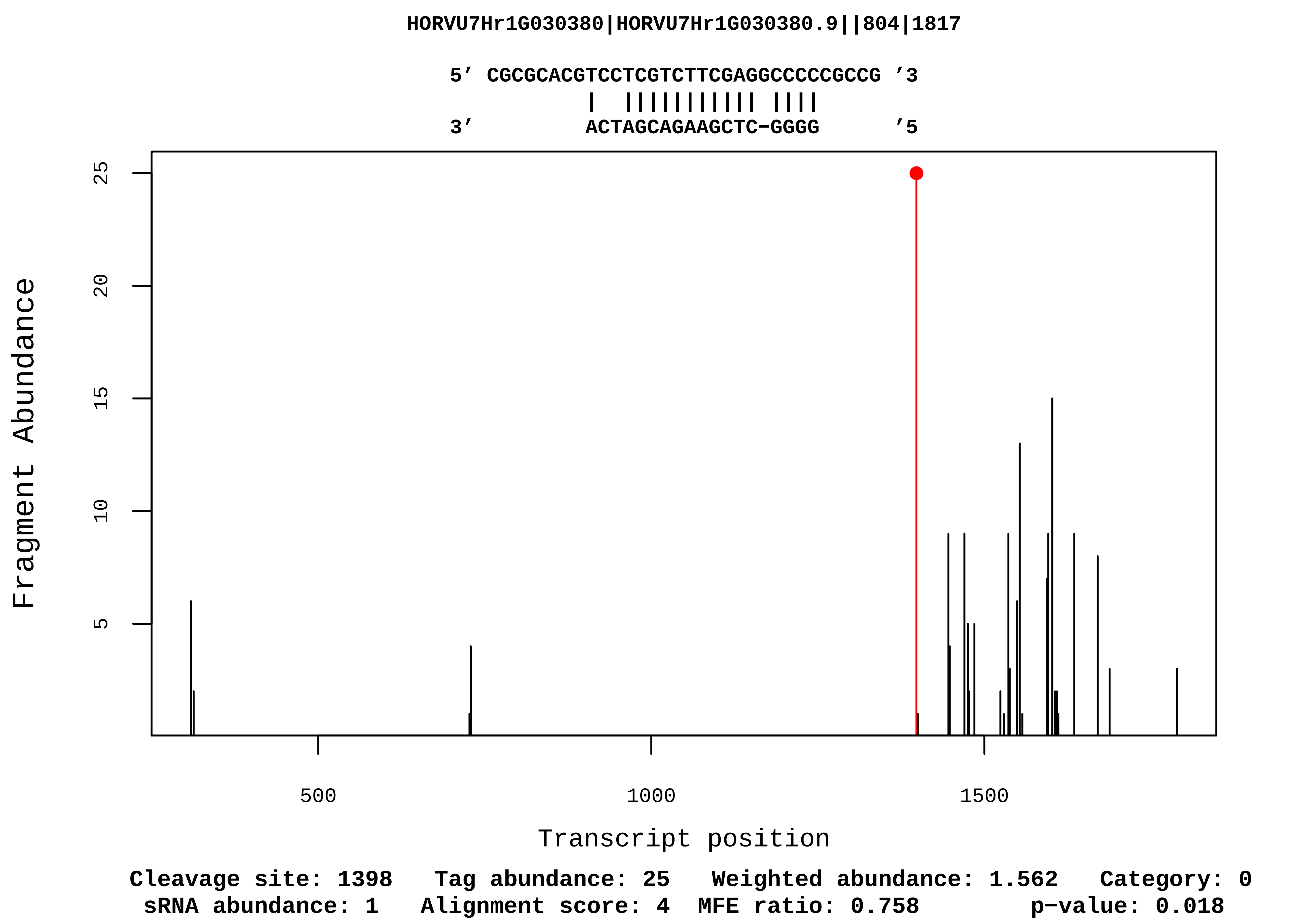

HORVU7Hr1G030380|HORVU7Hr1G030380.9||804|1817
5' CGCGCACGTCCTCGTCTTCGAGGCCCCCGCCG '3
3' ACTAGCAGAAGCTC-GGGG '5
Fragment Abundance
25
20
15
10
5
500
1000
1500
Transcript position
Cleavage site: 1398 Tag abundance: 25 Weighted abundance: 1.562 Category: 0
sRNA abundance: 1 Alignment score: 4 MFE ratio: 0.758 p-value: 0.018

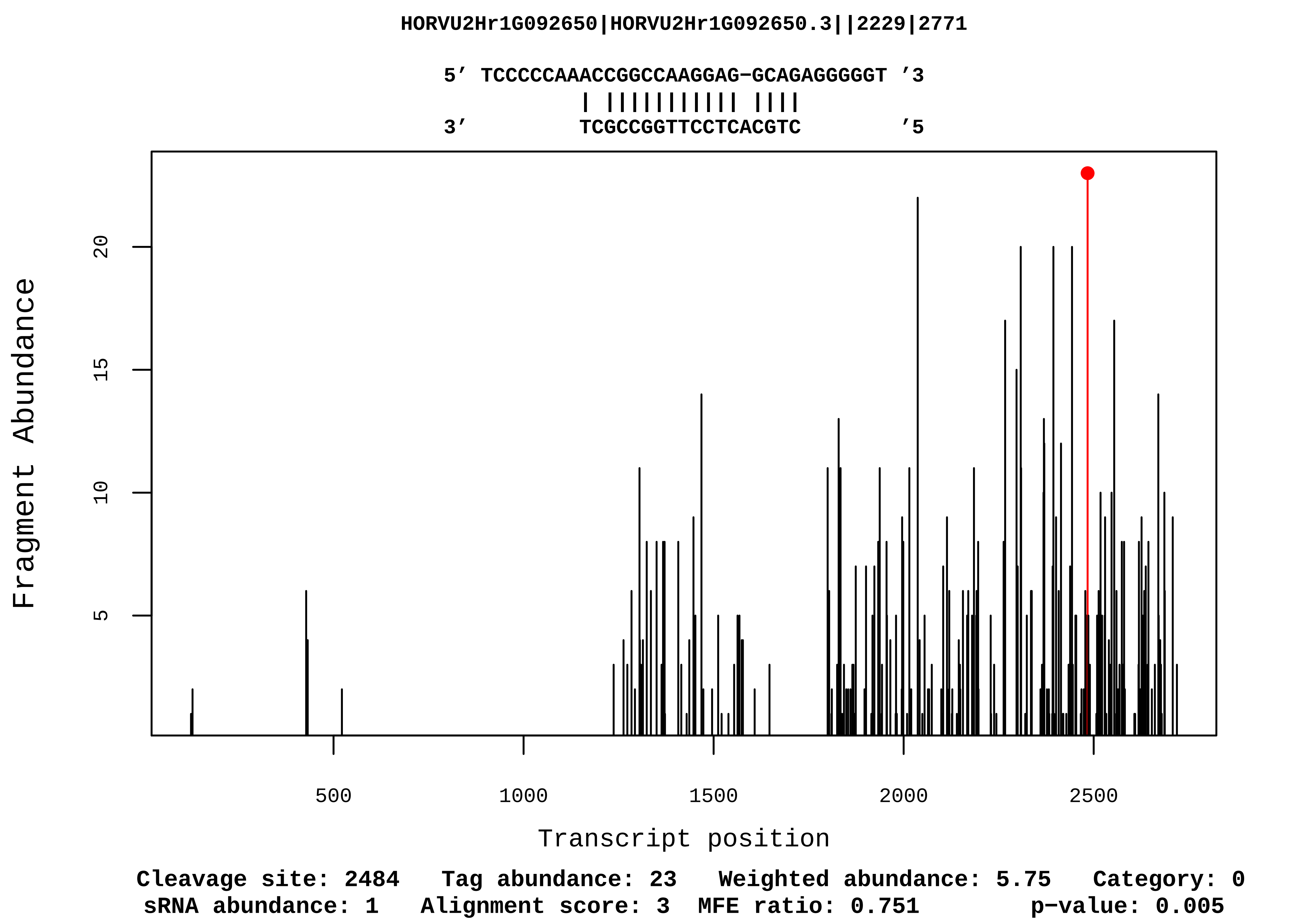

HORVU2Hr1G092650|HORVU2Hr1G092650.3||2229|2771
5′ TCCCCCAAACCGGCCAAGGAG-GCAGAGGGGGT ′3
3′ TCGCCGGTTCCTCACGTC ′5
Fragment Abundance
5
10
15
20
500
1000
1500
2000
2500
Transcript position
Cleavage site: 2484 Tag abundance: 23 Weighted abundance: 5.75 Category: 0
sRNA abundance: 1 Alignment score: 3 MFE ratio: 0.751 p-value: 0.005

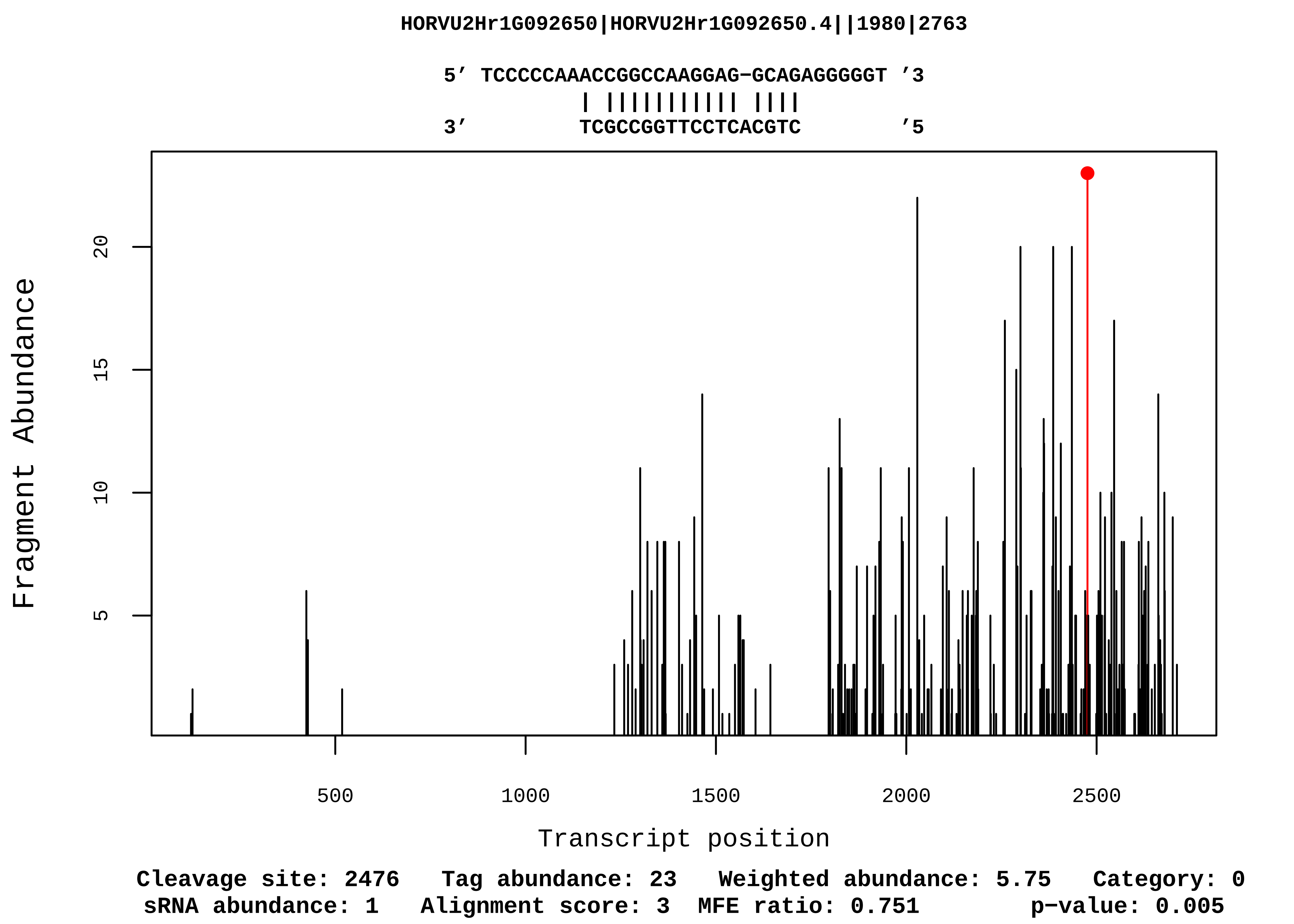

HORVU2Hr1G092650|HORVU2Hr1G092650.4||1980|2763
5′ TCCCCCAAACCGGCCAAGGAG-GCAGAGGGGGT ′3
3′ TCGCCGGTTCCTCACGTC ′5
Fragment Abundance
Transcript position
5
10
15
20
500
1000
1500
2000
2500
Cleavage site: 2476 Tag abundance: 23 Weighted abundance: 5.75 Category: 0
sRNA abundance: 1 Alignment score: 3 MFE ratio: 0.751 p-value: 0.005

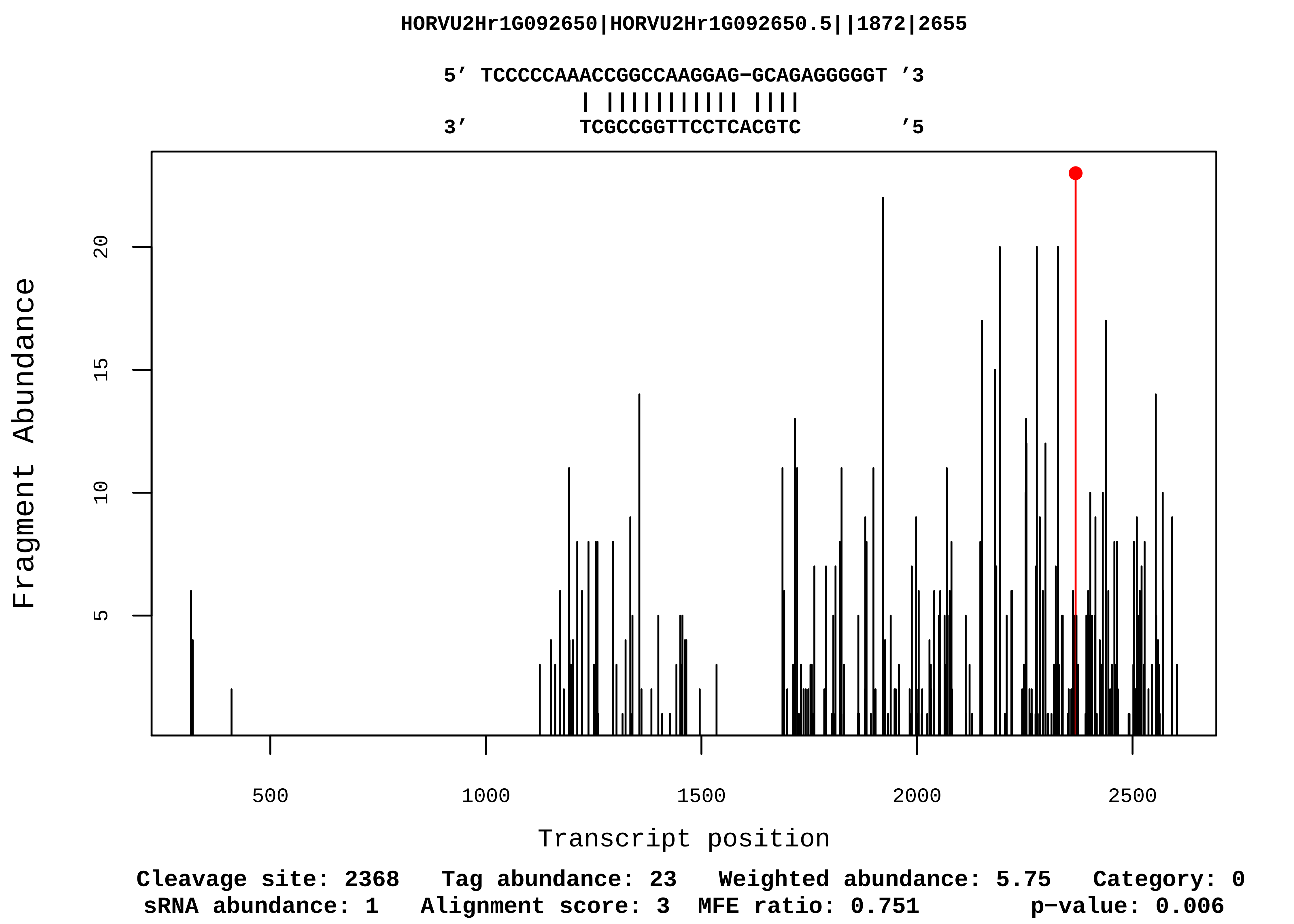

HORVU2Hr1G092650|HORVU2Hr1G092650.5||1872|2655
5′ TCCCCCAAACCGGCCAAGGAG−GCAGAGGGGGT ′3
| ||||||||||| ||||
3′ TCGCCGGTTCCTCACGTC ′5
Fragment Abundance
5
10
15
20
500
1000
1500
2000
2500
Transcript position
Cleavage site: 2368 Tag abundance: 23 Weighted abundance: 5.75 Category: 0
sRNA abundance: 1 Alignment score: 3 MFE ratio: 0.751 p−value: 0.006

HORVU2Hr1G092650|HORVU2Hr1G092650.8||306|755

```
5' TCCCCCAAACCGGCCAAGGAG-GCAGAGGGGGT '3
          | ||||||||||| ||||
3'        TCGCCGGTTCCTCACGTC         '5
```

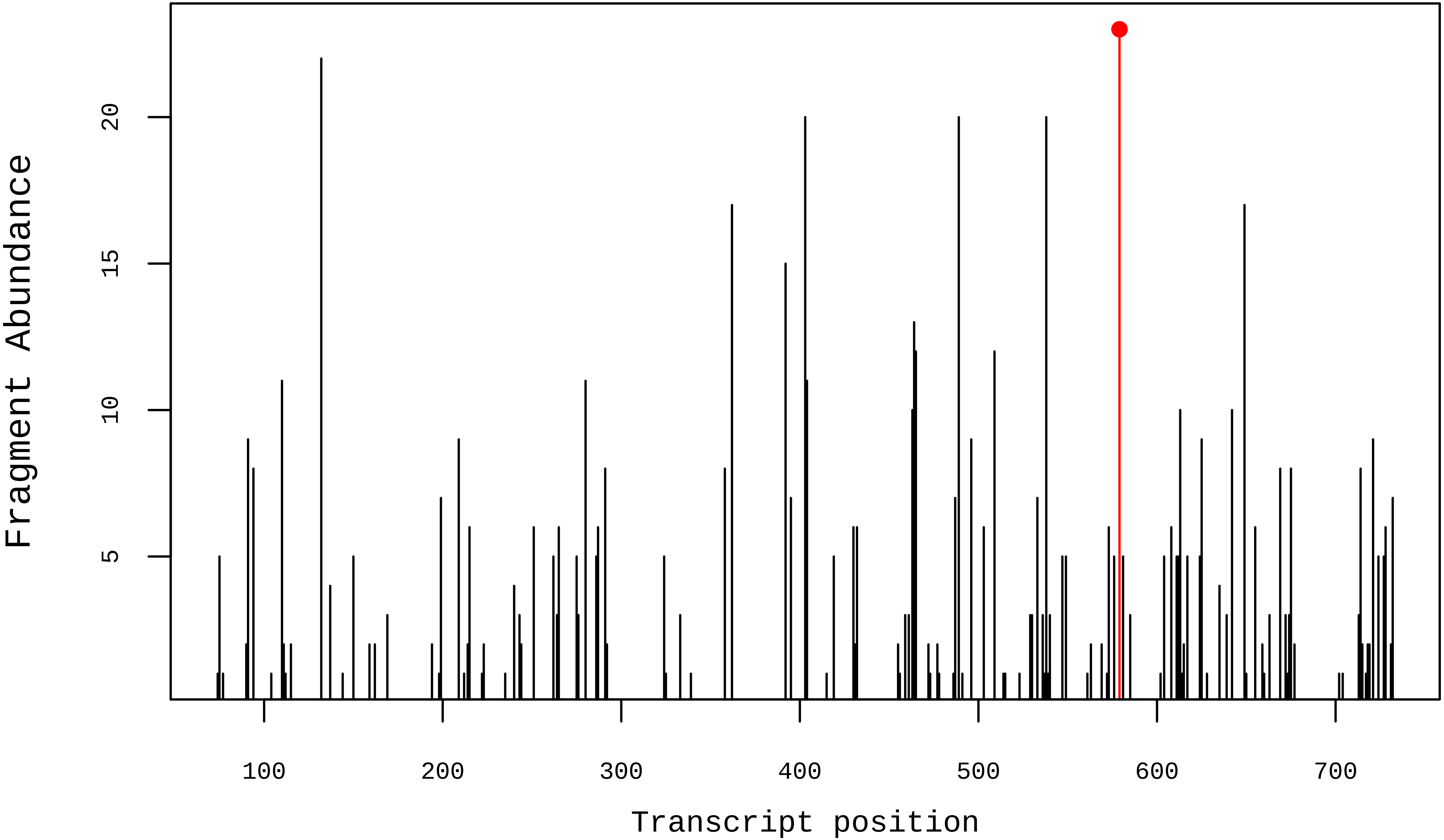

Cleavage site: 579 Tag abundance: 23 Weighted abundance: 5.75 Category: 0
sRNA abundance: 1 Alignment score: 3 MFE ratio: 0.751 p-value: 0.02

HORVU0Hr1G003660|HORVU0Hr1G003660.1||297|1955

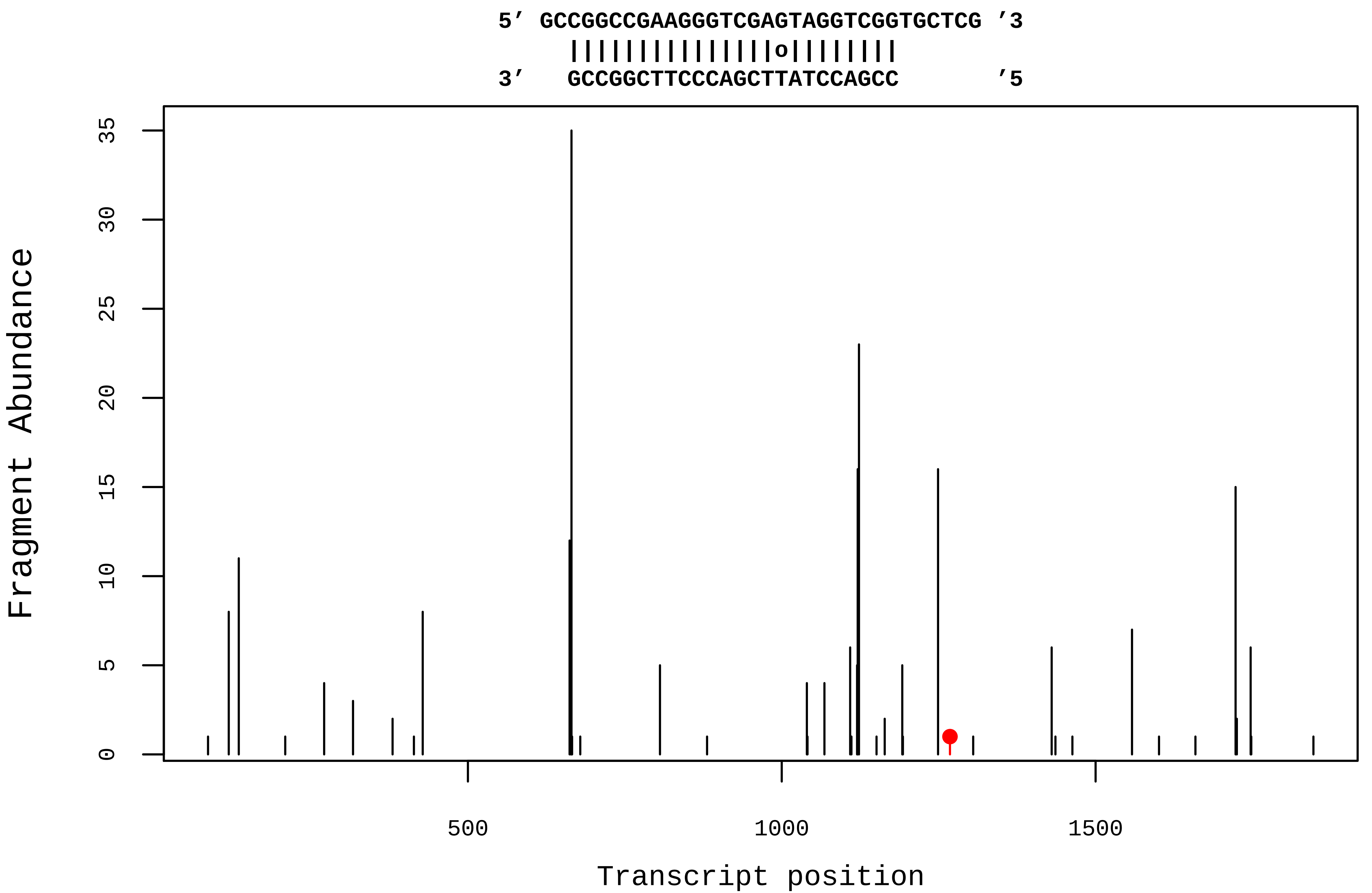

Cleavage site: 1268 Tag abundance: 1 Weighted abundance: 0.143 Category: 4
sRNA abundance: 1 Alignment score: 1 MFE ratio: 1 p-value: 0.046

HORVU0Hr1G023930|HORVU0Hr1G023930.3||396|2180

```
5' GTCGGCGGAAGGGTCGAGTAGGTCGGTGCTCG '3
      |||| ||||||||||o||||||||
3'    GCCGGCTTCCCAGCTTATCCAGCC        '5
```

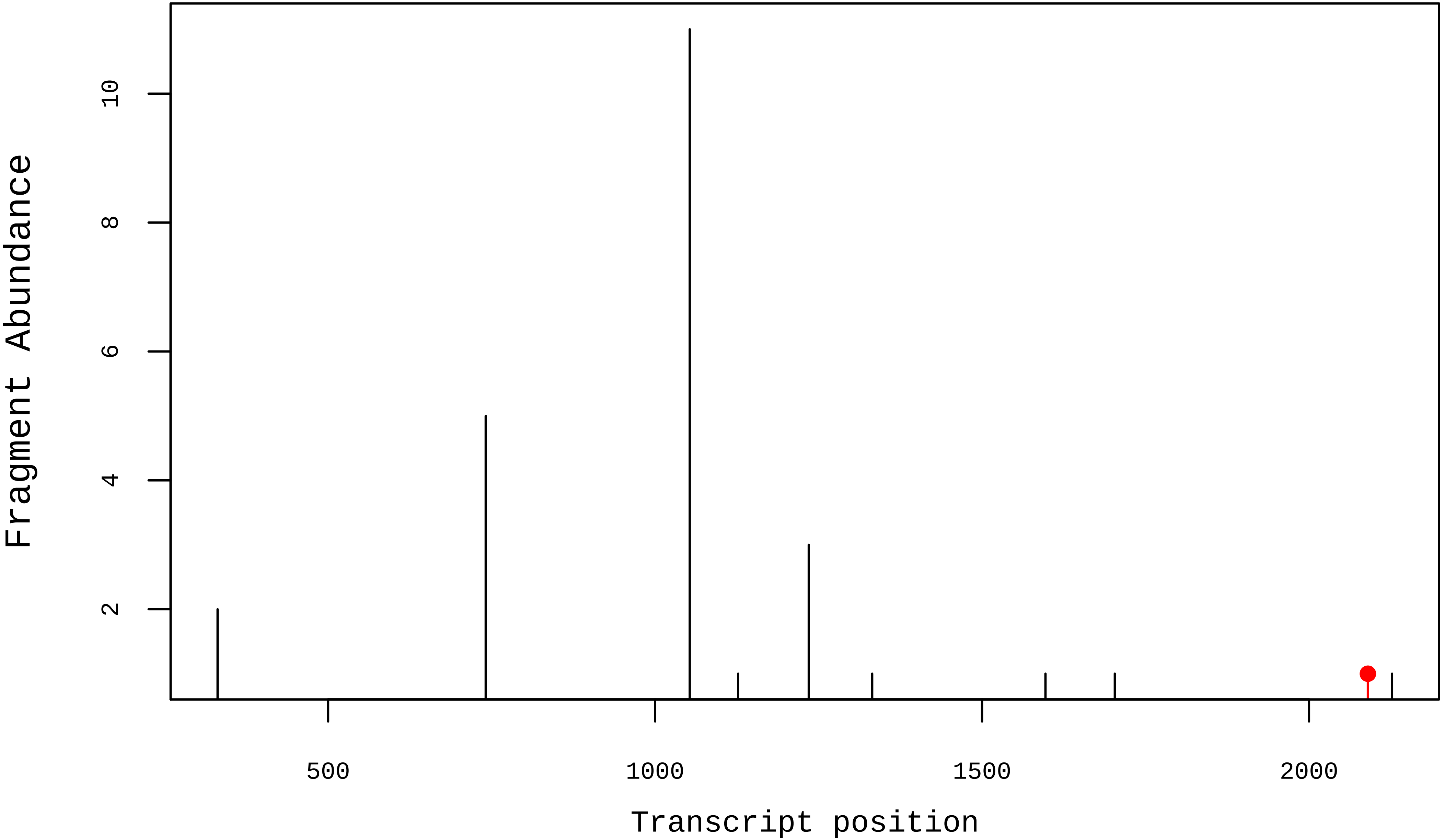

Cleavage site: 2090   Tag abundance: 1   Weighted abundance: 0.143   Category: 4
sRNA abundance: 1   Alignment score: 2   MFE ratio: 0.916   p-value: 0.019

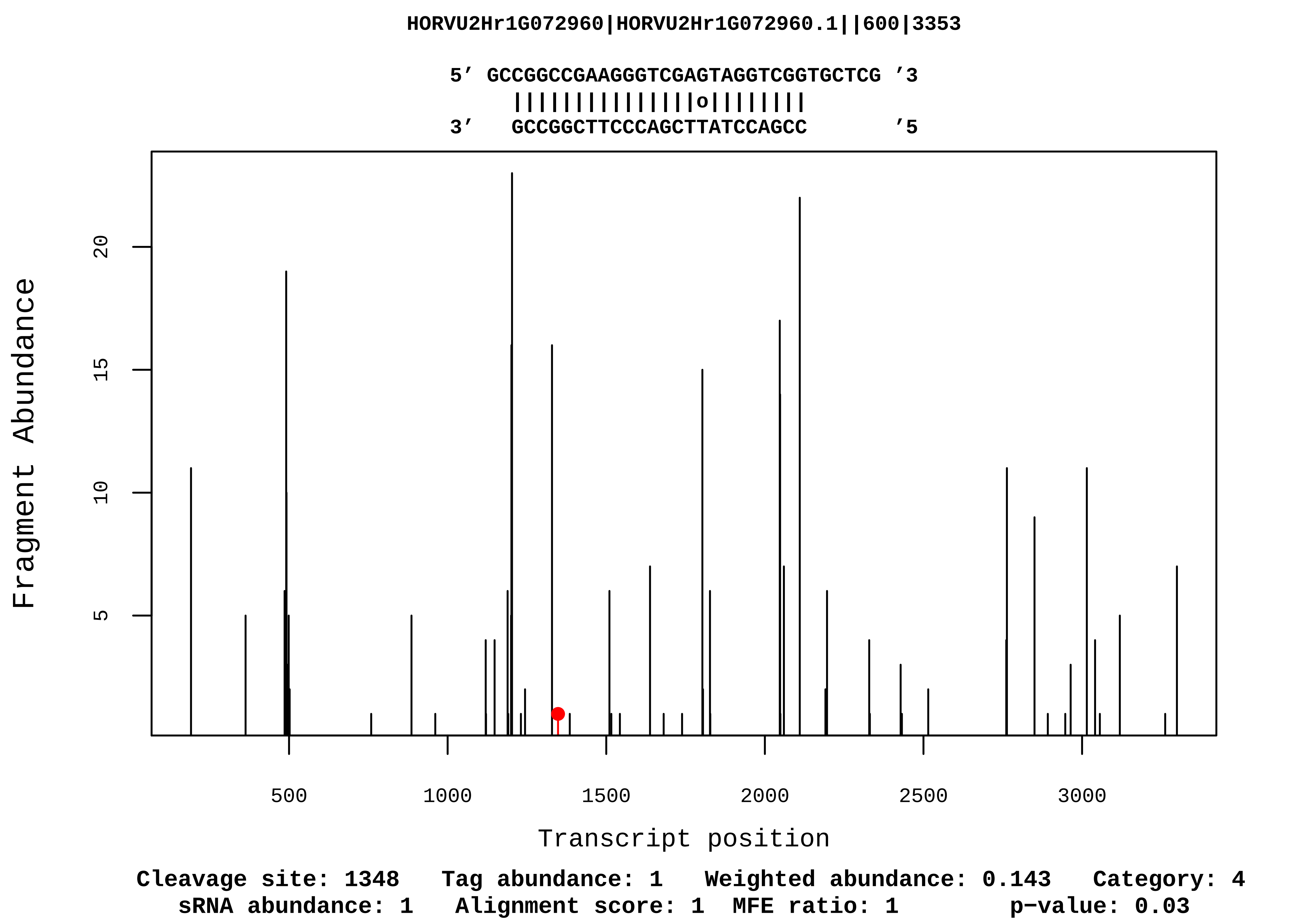
HORVU2Hr1G072960|HORVU2Hr1G072960.1||600|3353
5' GCCGGCCGAAGGGTCGAGTAGGTCGGTGCTCG '3
|||||||||||||||o||||||||
3' GCCGGCTTCCCAGCTTATCCAGCC '5
Fragment Abundance
5
10
15
20
500
1000
1500
2000
2500
3000
Transcript position
Cleavage site: 1348 Tag abundance: 1 Weighted abundance: 0.143 Category: 4
sRNA abundance: 1 Alignment score: 1 MFE ratio: 1 p-value: 0.03

HORVU5Hr1G015600|HORVU5Hr1G015600.1||156|510

```
5' GCCGGCCGAAGGGTCGAGTAGGTCGGTGCTCG '3
      |||||||||||||||o||||||||
3'    GCCGGCTTCCCAGCTTATCCAGCC        '5
```

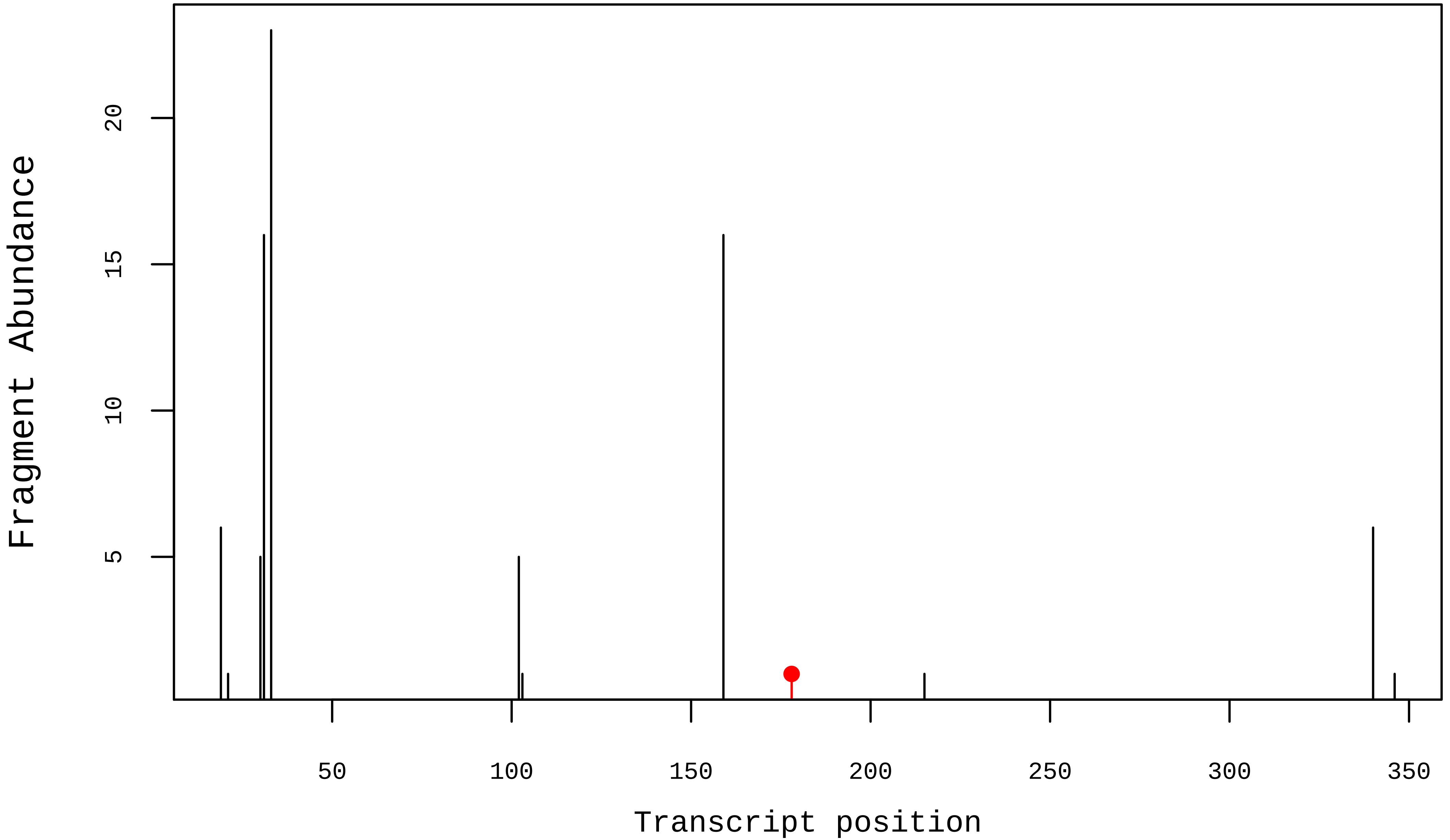

Cleavage site: 178   Tag abundance: 1   Weighted abundance: 0.143   Category: 4
sRNA abundance: 1   Alignment score: 1 MFE ratio: 1   p-value: 0.05

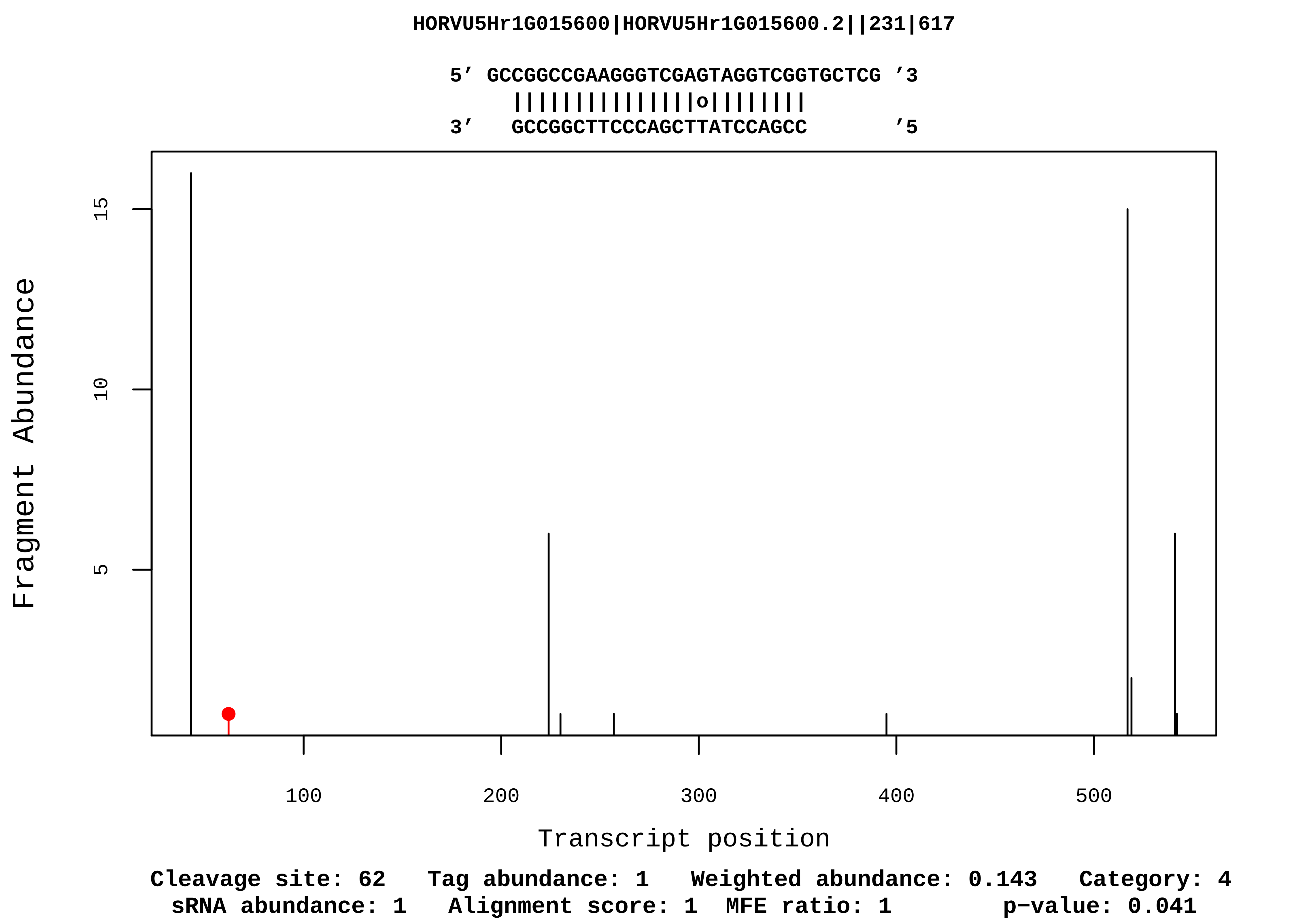

HORVU5Hr1G015600|HORVU5Hr1G015600.2||231|617
5' GCCGGCCGAAGGGTCGAGTAGGTCGGTGCTCG '3
||||||||||||||||o||||||||
3' GCCGGCTTCCCAGCTTATCCAGCC '5
Fragment Abundance
5
10
15
100
200
300
400
500
Transcript position
Cleavage site: 62 Tag abundance: 1 Weighted abundance: 0.143 Category: 4
sRNA abundance: 1 Alignment score: 1 MFE ratio: 1 p-value: 0.041

HORVU5Hr1G015600|HORVU5Hr1G015600.3||276|1709

```
5' GCCGGCCGAAGGGTCGAGTAGGTCGGTGCTCG '3
     |||||||||||||||o||||||||
3'   GCCGGCTTCCCAGCTTATCCAGCC       '5
```

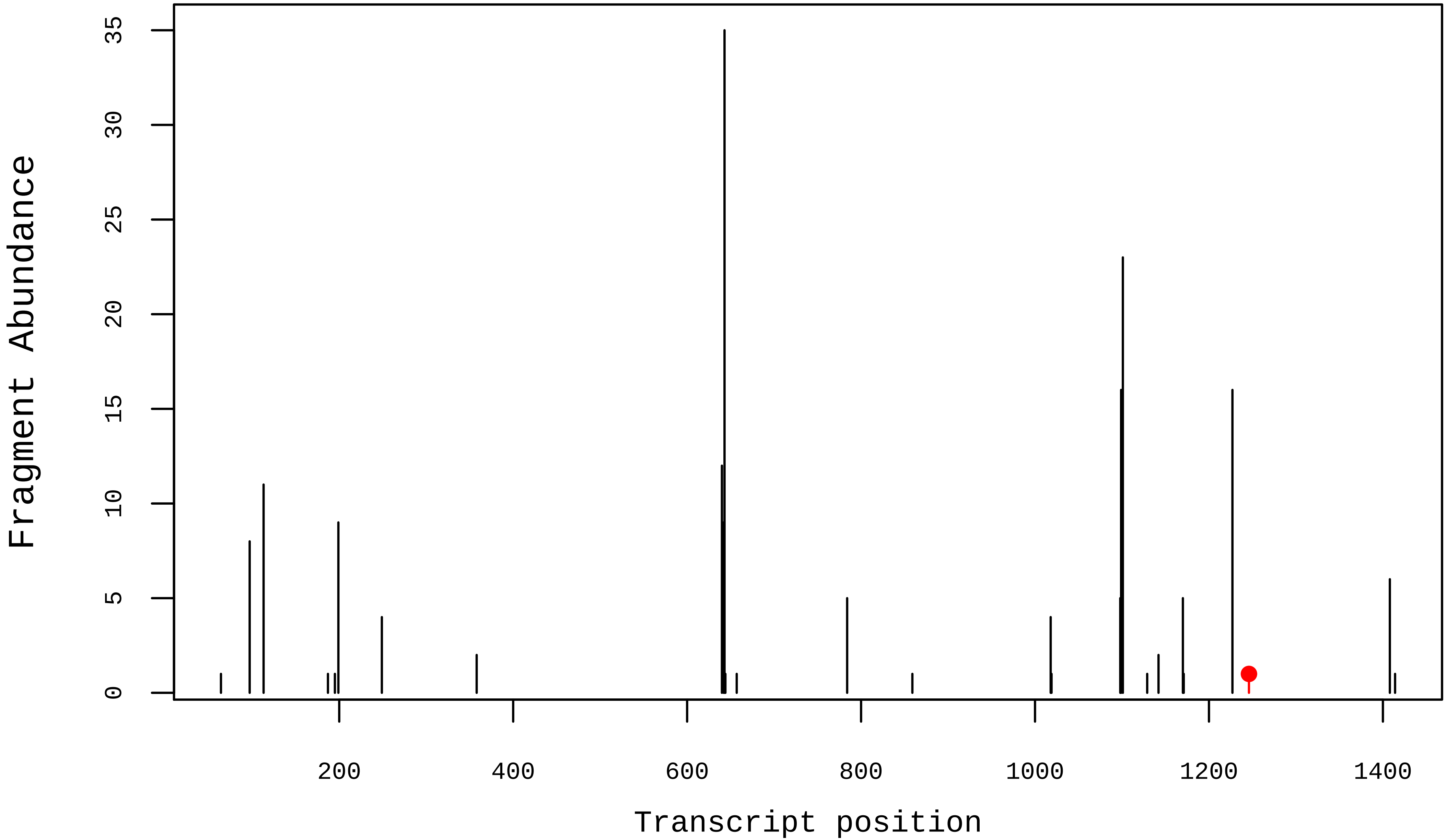

Cleavage site: 1246   Tag abundance: 1   Weighted abundance: 0.143   Category: 4
sRNA abundance: 1   Alignment score: 1   MFE ratio: 1   p-value: 0.032

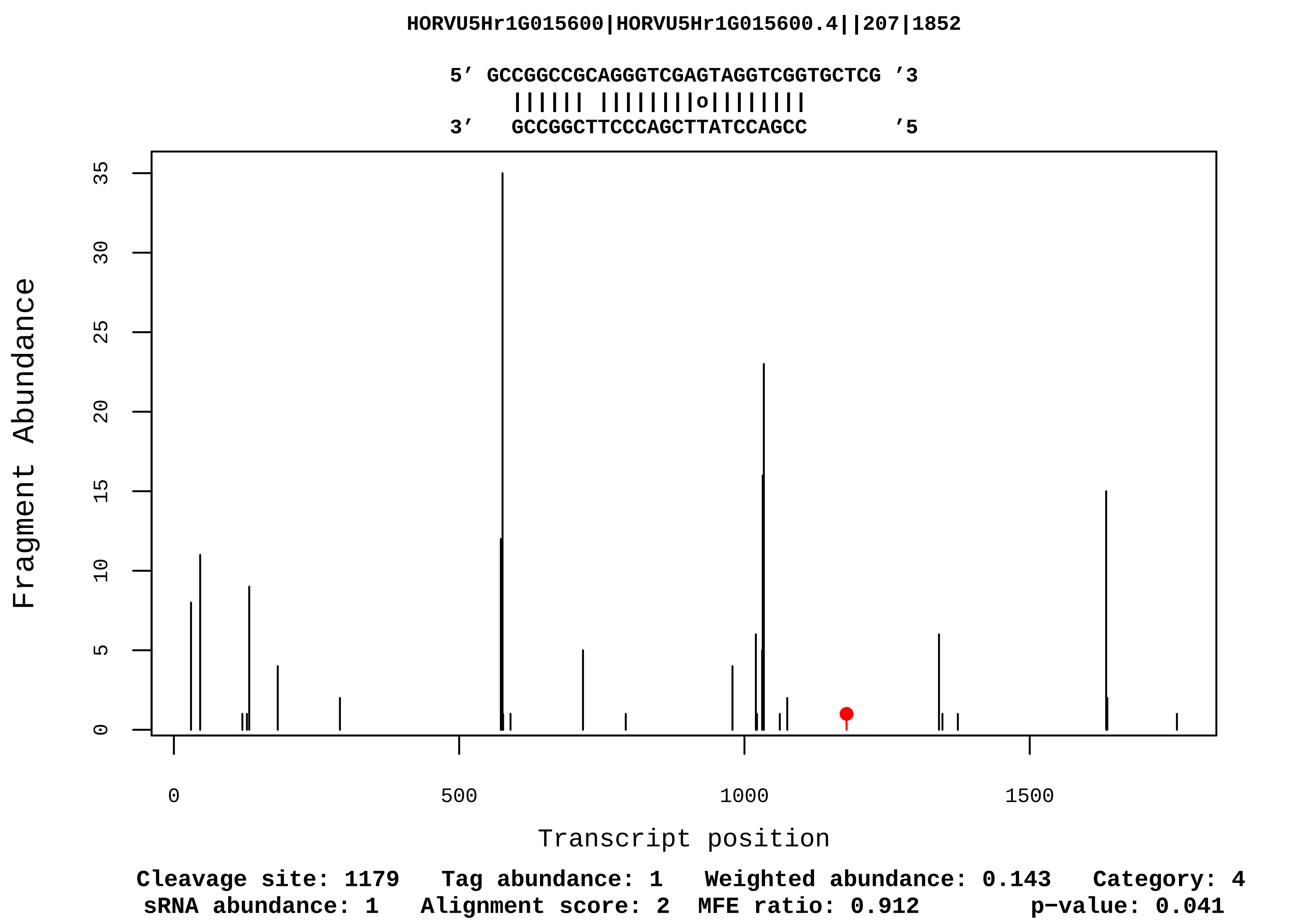
HORVU5Hr1G015600|HORVU5Hr1G015600.4||207|1852
5' GCCGGCCGCAGGGTCGAGTAGGTCGGTGCTCG '3
|||||| ||||||||o||||||||
3' GCCGGCTTCCCAGCTTATCCAGCC '5
Fragment Abundance
0
5
10
15
20
25
30
35
0
500
1000
1500
Transcript position
Cleavage site: 1179 Tag abundance: 1 Weighted abundance: 0.143 Category: 4
sRNA abundance: 1 Alignment score: 2 MFE ratio: 0.912 p-value: 0.041

HORVU3Hr1G078840|HORVU3Hr1G078840.1||1401|2887

```
5' CTGTTGCGT-GGGATCAGCTGGTGAATGCTCGG '3
             | |||||||| ||||o|||
3'           AGCCCTAGTCTACCATTTA       '5
```

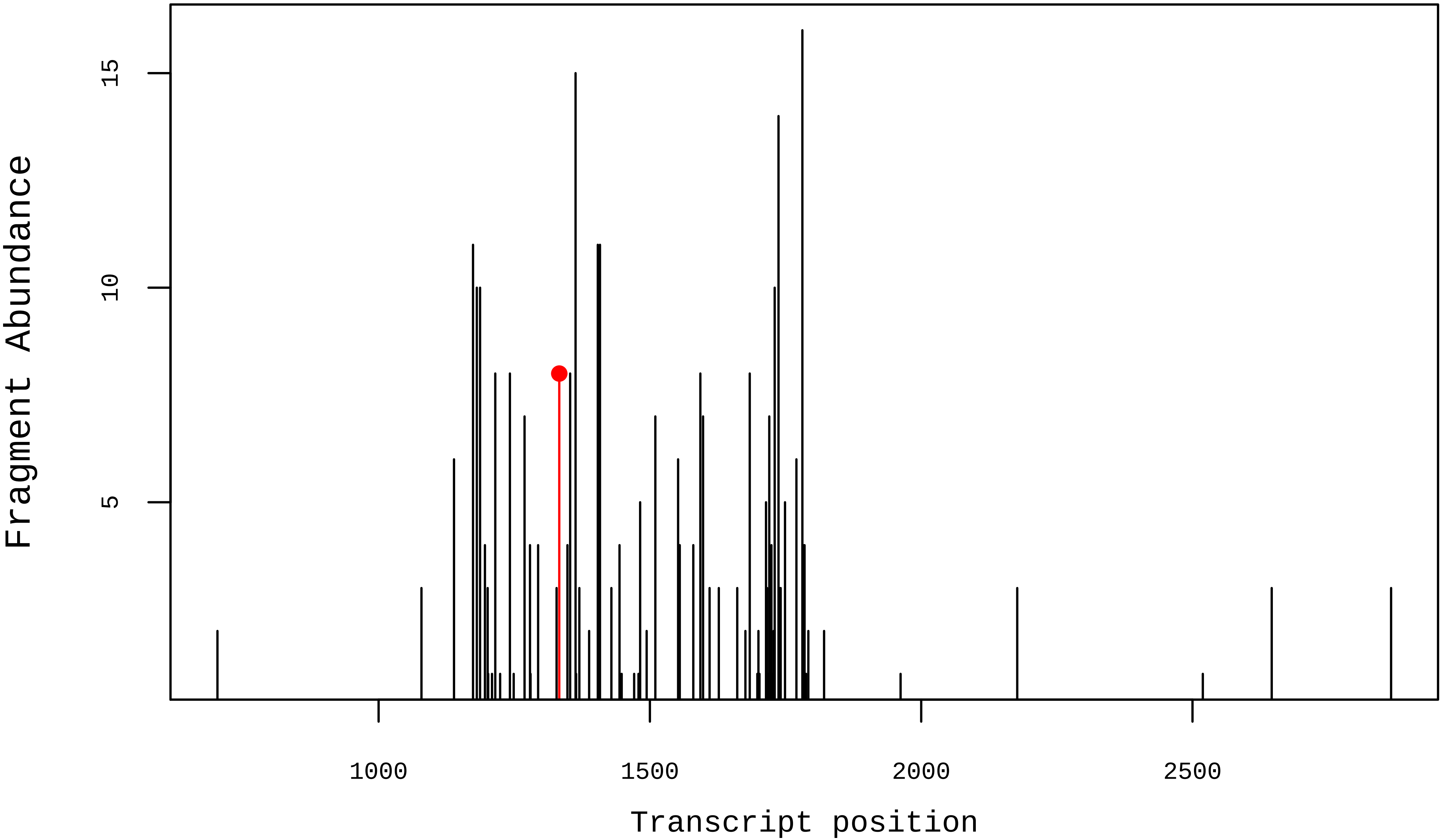

Cleavage site: 1333 Tag abundance: 8 Weighted abundance: 1.333 Category: 2
sRNA abundance: 1 Alignment score: 4 MFE ratio: 0.763 p-value: 0.043

HORVU3Hr1G078840|HORVU3Hr1G078840.2||717|2962

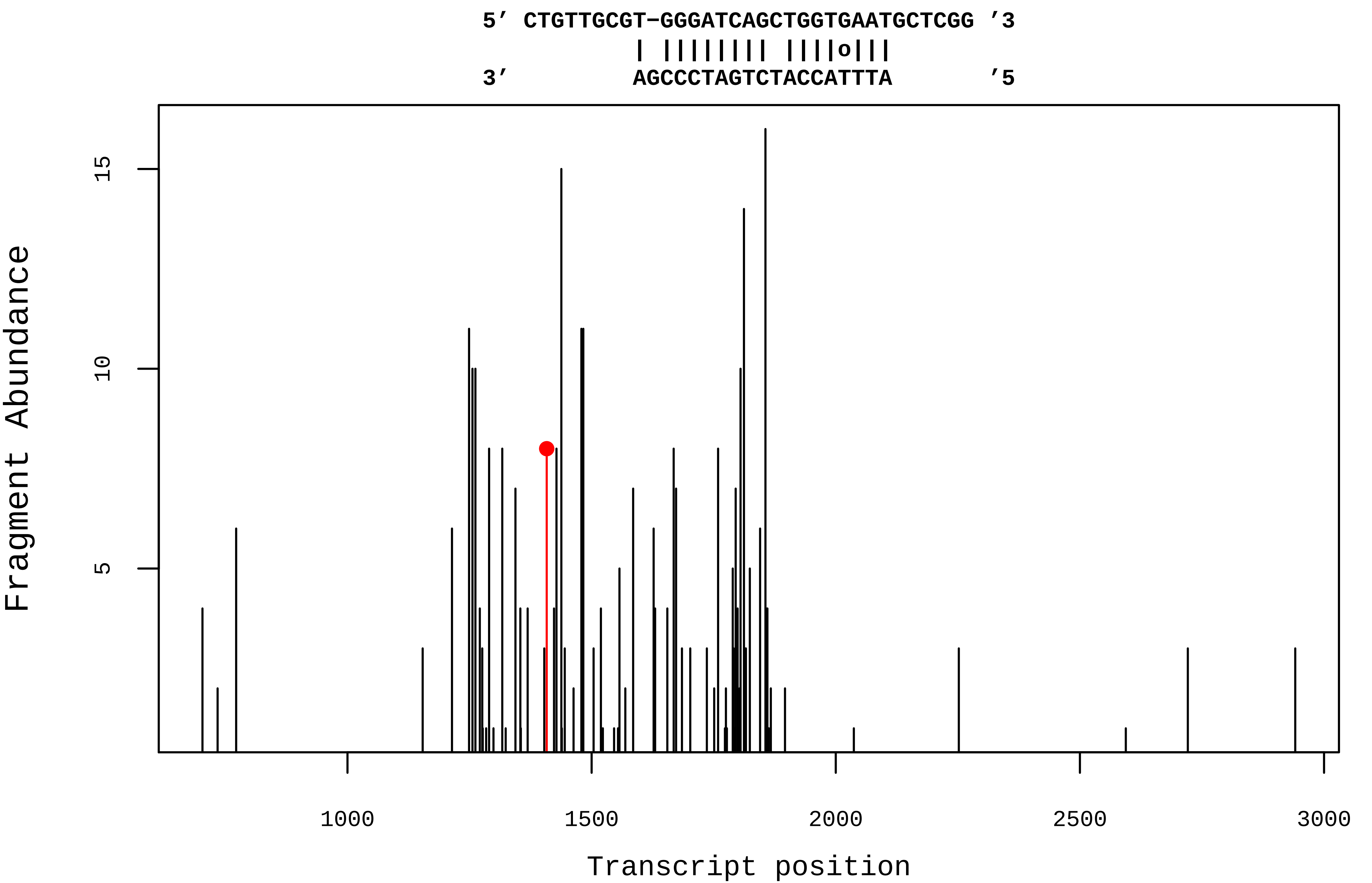

Cleavage site: 1408 Tag abundance: 8 Weighted abundance: 1.333 Category: 2
sRNA abundance: 1 Alignment score: 4 MFE ratio: 0.763 p-value: 0.044

HORVU3Hr1G078840|HORVU3Hr1G078840.4||1353|2233

```
5' CTGTTGCGT-GGGATCAGCTGGTGAATGCTCGG '3
             | |||||||| ||||o|||
3'           AGCCCTAGTCTACCATTTA       '5
```

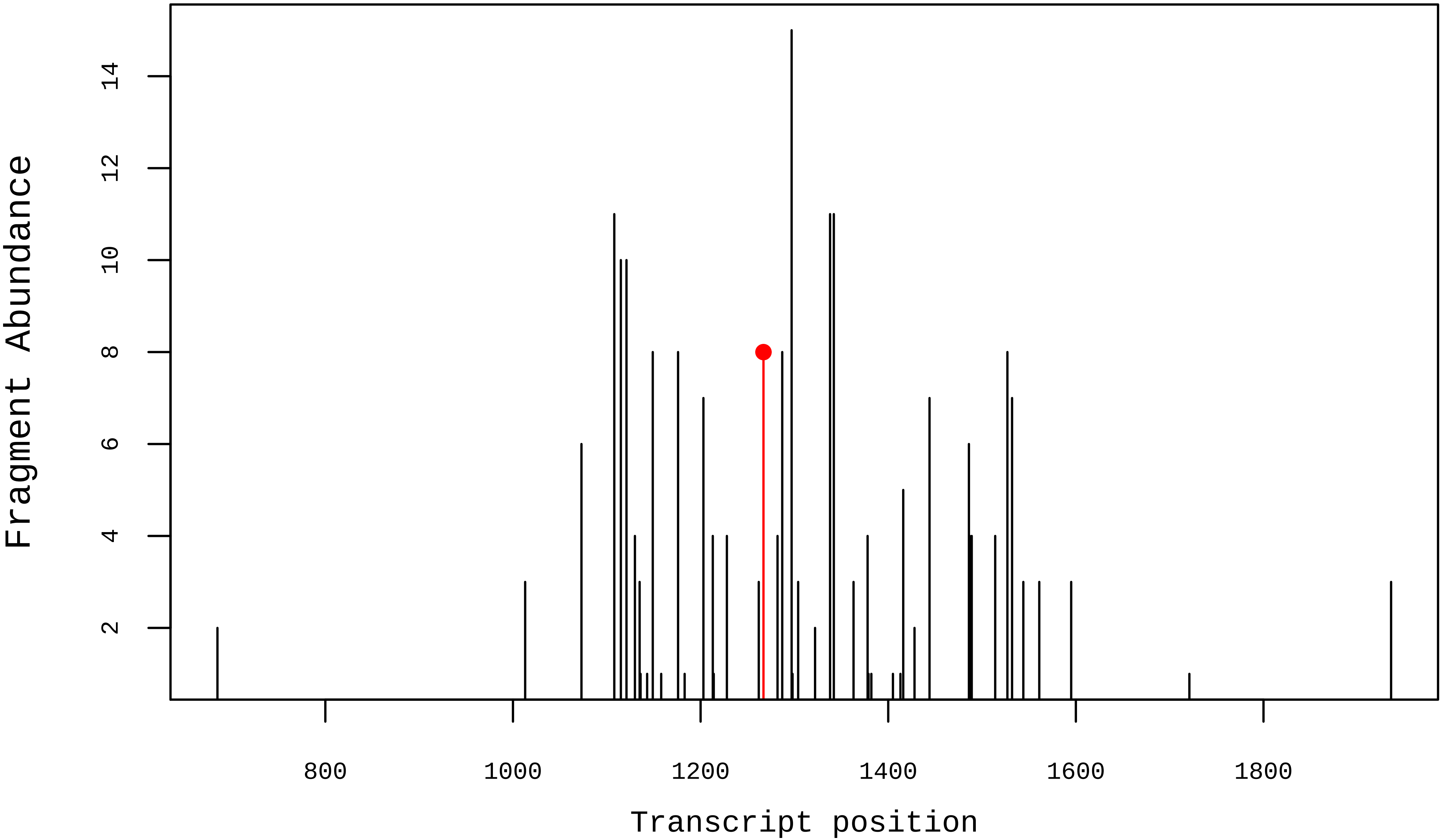

Cleavage site: 1267   Tag abundance: 8   Weighted abundance: 1.333   Category: 2
sRNA abundance: 1   Alignment score: 4   MFE ratio: 0.763   p-value: 0.04

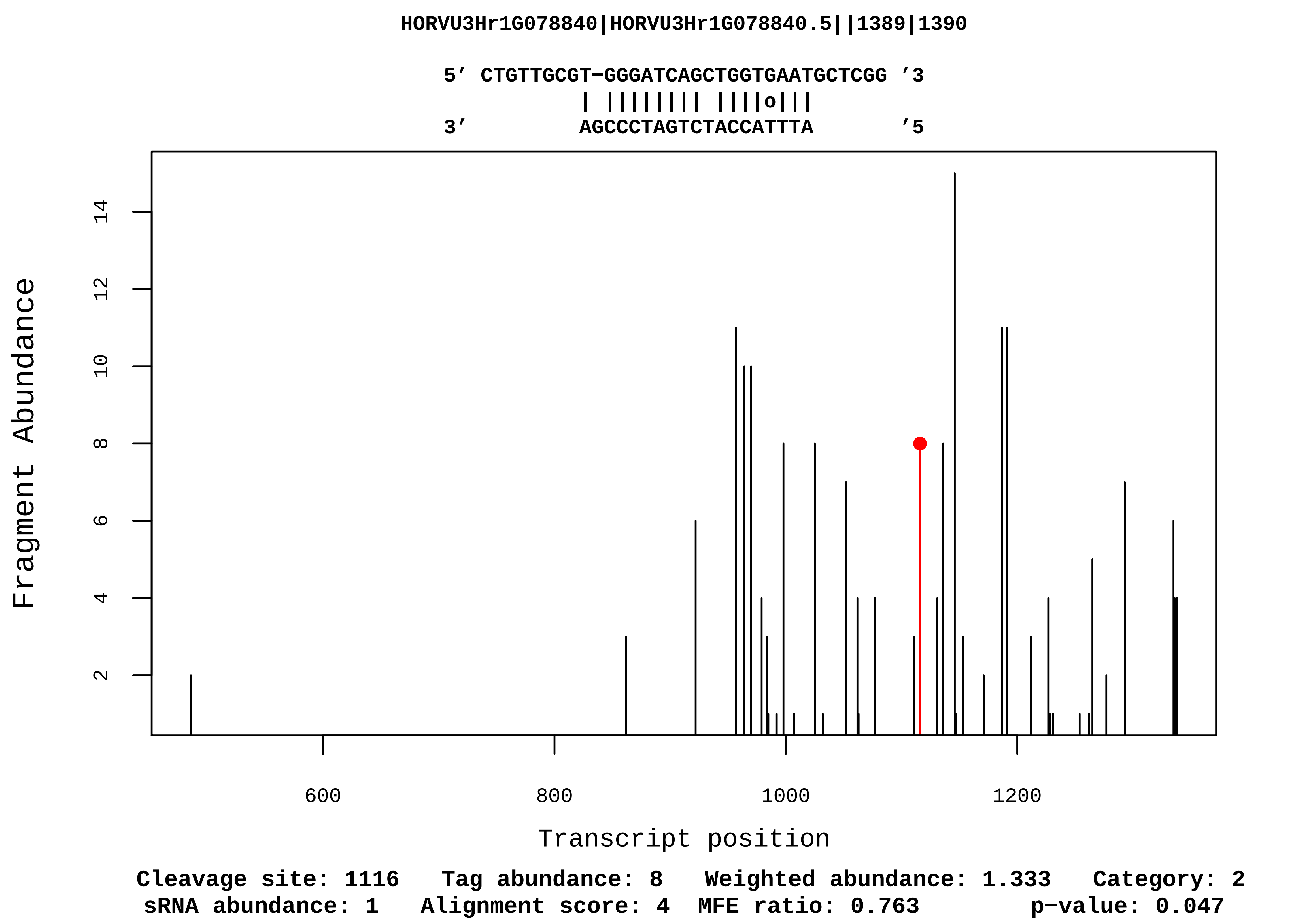

HORVU3Hr1G078840|HORVU3Hr1G078840.5||1389|1390
5' CTGTTGCGT-GGGATCAGCTGGTGAATGCTCGG '3
| |||||||| ||||o|||
3' AGCCCTAGTCTACCATTTA '5
Fragment Abundance
2
4
6
8
10
12
14
600
800
1000
1200
Transcript position
Cleavage site: 1116 Tag abundance: 8 Weighted abundance: 1.333 Category: 2
sRNA abundance: 1 Alignment score: 4 MFE ratio: 0.763 p-value: 0.047

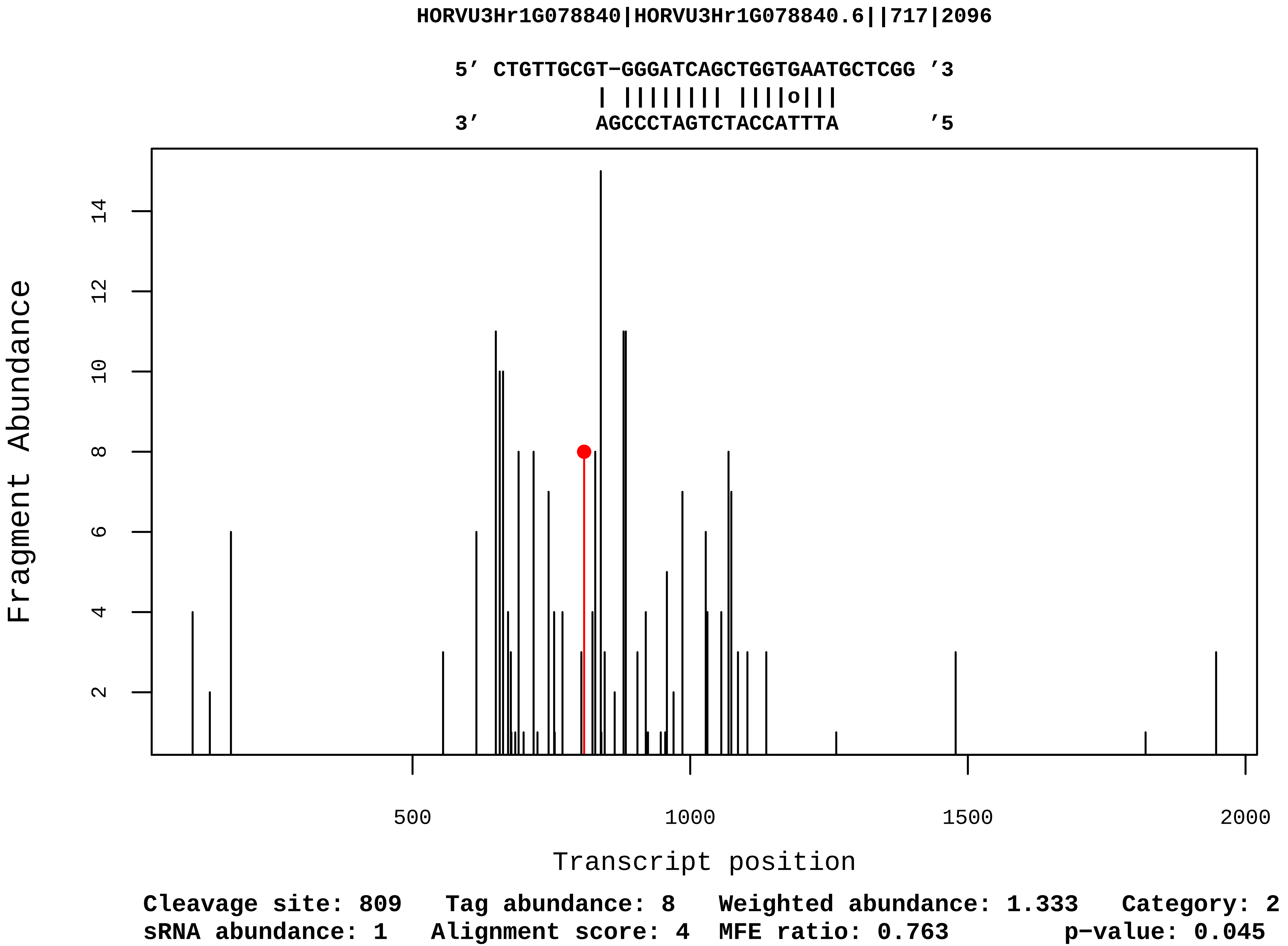

HORVU3Hr1G078840|HORVU3Hr1G078840.6||717|2096
5' CTGTTGCGT-GGGATCAGCTGGTGAATGCTCGG '3
| |||||||| ||||o|||
3' AGCCCTAGTCTACCATTTA '5
Fragment Abundance
2
4
6
8
10
12
14
500
1000
1500
2000
Transcript position
Cleavage site: 809 Tag abundance: 8 Weighted abundance: 1.333 Category: 2
sRNA abundance: 1 Alignment score: 4 MFE ratio: 0.763 p-value: 0.045

HORVU2Hr1G001690|HORVU2Hr1G001690.7||669|903

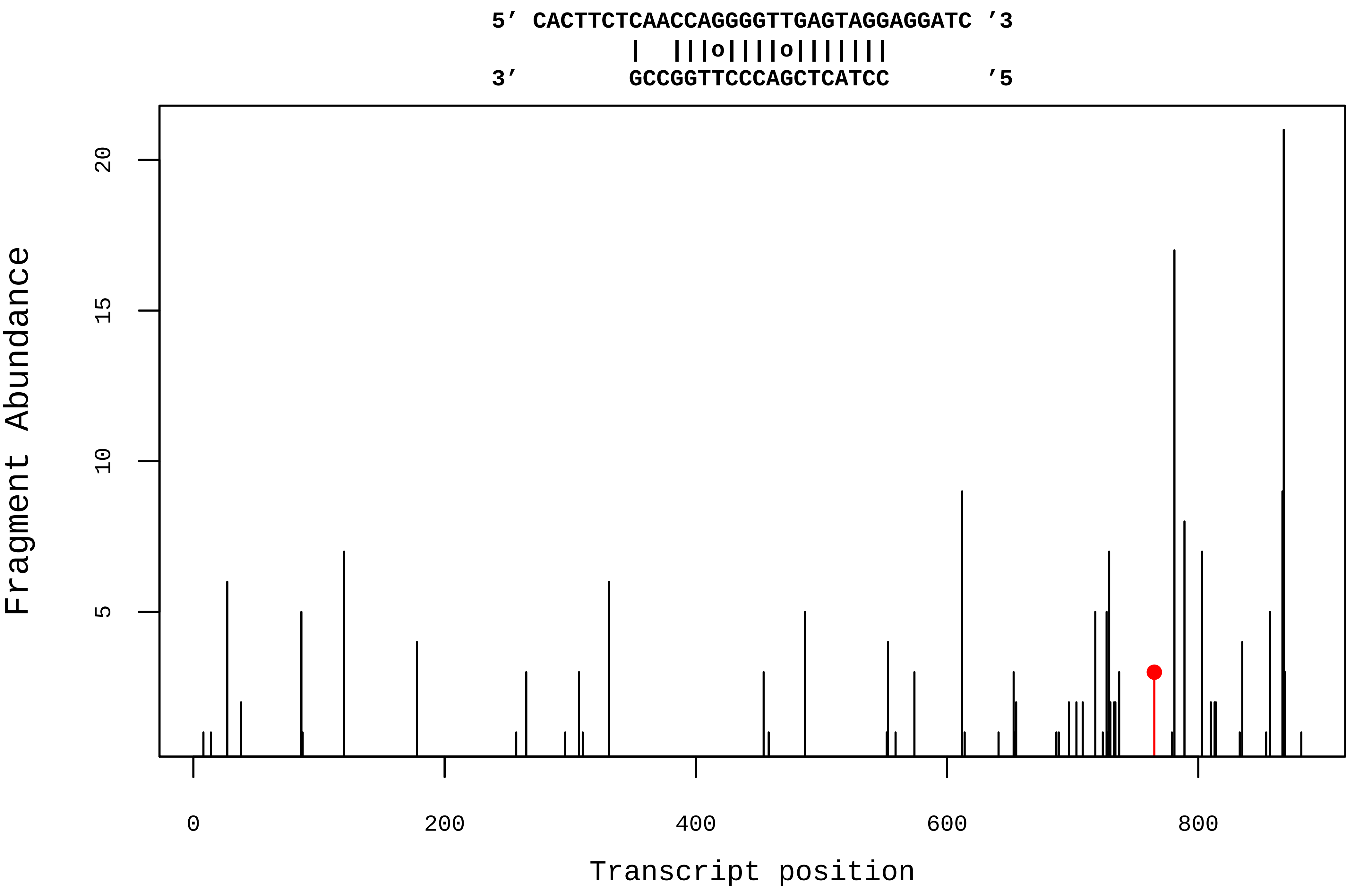

Cleavage site: 765 Tag abundance: 3 Weighted abundance: 0.375 Category: 3
sRNA abundance: 1 Alignment score: 4 MFE ratio: 0.743 p-value: 0.025

HORVU3Hr1G001430|HORVU3Hr1G001430.1||1884|2633

```
5' CAGAGTGCAACTGGA-CCTAAGACTGGTCTCAG '3
               |o||| |||||||||||
3'             CGGCCTAGGATTCTGACC       '5
```

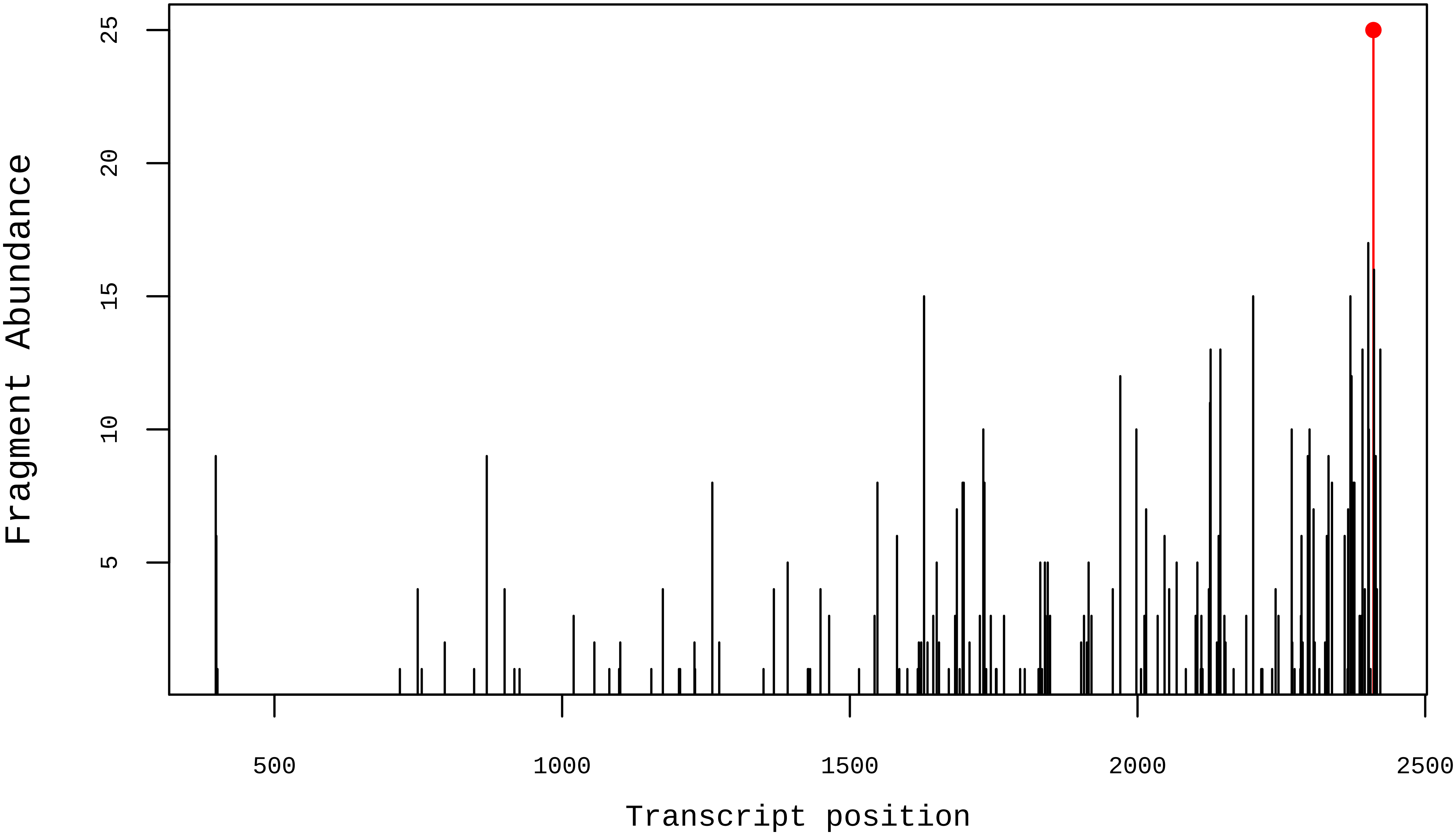

Cleavage site: 2410 Tag abundance: 25 Weighted abundance: 4.167 Category: 0
sRNA abundance: 1 Alignment score: 3.5 MFE ratio: 0.744 p-value: 0.002

HORVU3Hr1G082030|HORVU3Hr1G082030.1||606|2349

```
5' AAATCTAATCTGGTTTTTTCCATCATCATCCG '3
          |||||||||| ||||||||
3'        ACGACCAAAAA-GGTAGTAG       '5
```

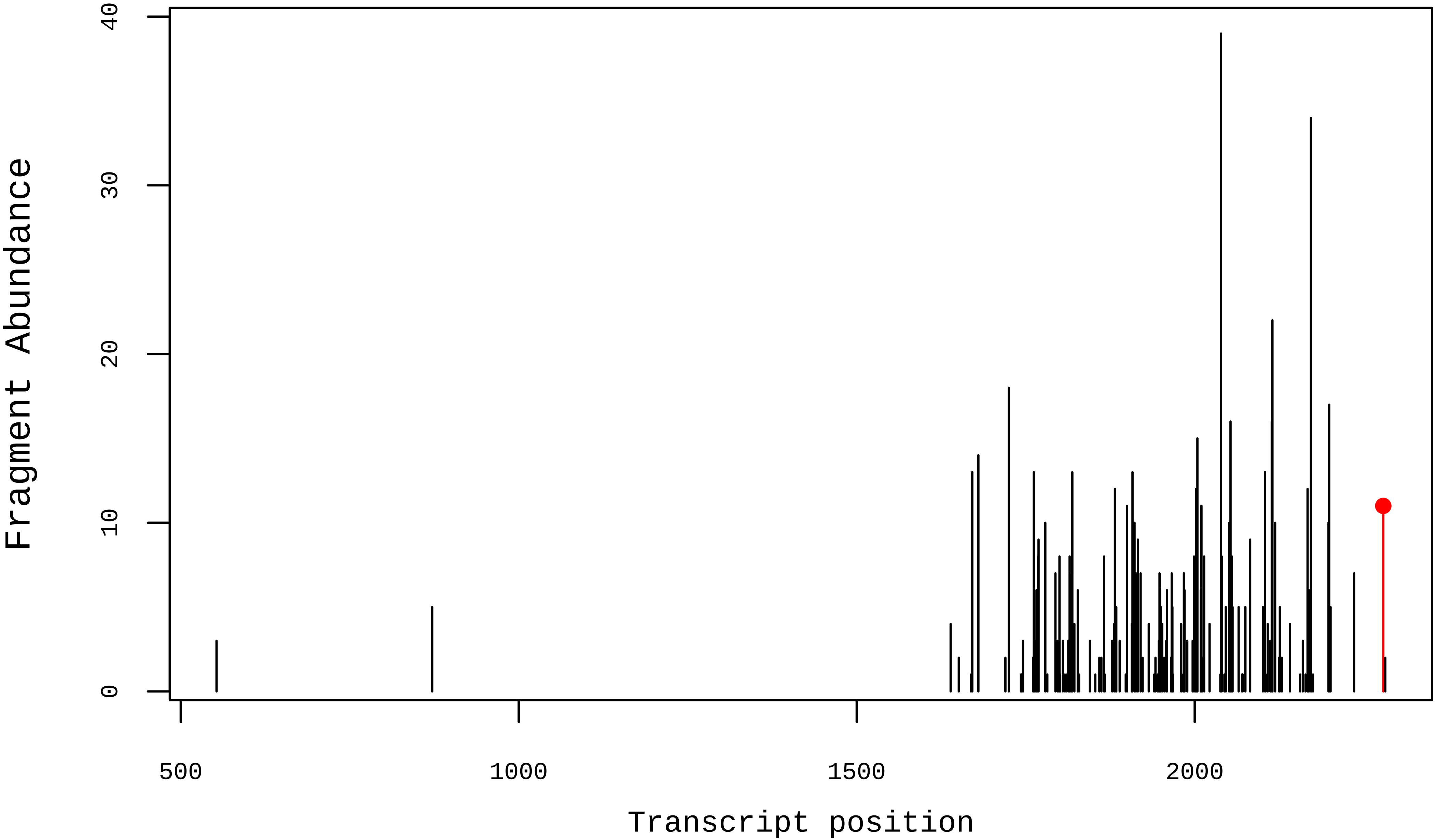

Cleavage site: 2279   Tag abundance: 11   Weighted abundance: 5.5   Category: 2
sRNA abundance: 1   Alignment score: 4   MFE ratio: 0.74   p-value: 0.03

HORVU3Hr1G082030|HORVU3Hr1G082030.2||939|2085

```
5' AAATCTAATCTGGTTTTTTCCATCATCATCCG '3
          |||||||||| ||||||||
3'      ACGACCAAAAA-GGTAGTAG      '5
```

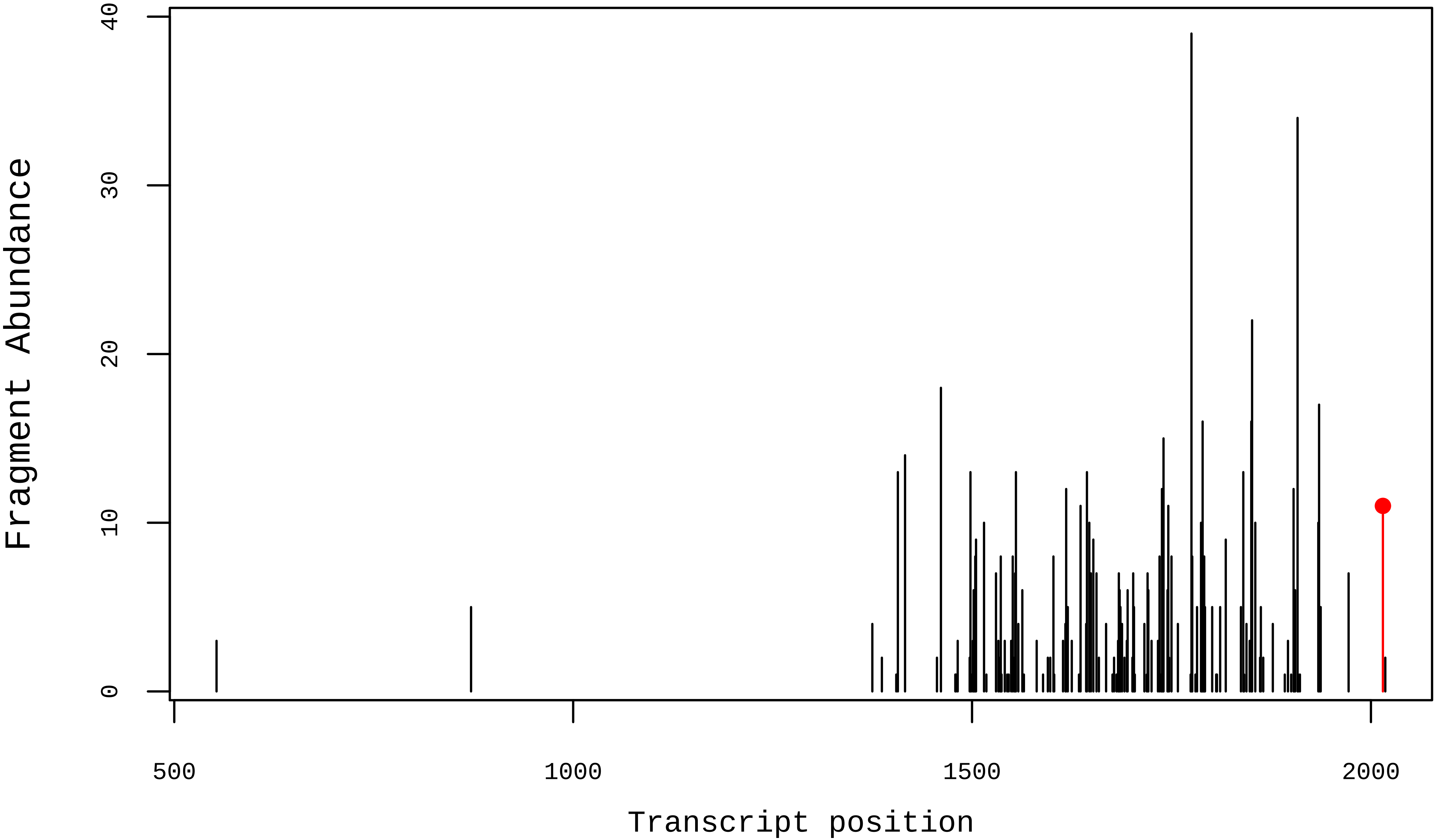

Cleavage site: 2015 Tag abundance: 11 Weighted abundance: 5.5 Category: 2
sRNA abundance: 1 Alignment score: 4 MFE ratio: 0.74 p-value: 0.034

HORVU3Hr1G020880|HORVU3Hr1G020880.1||1197|2552

```
5' TAAAAGTTGATGCACCAGCCGGGTATCGAACC '3
          | ||||||||||||| ||
3'        CCACGTGGTCGGCCCTTA         '5
```

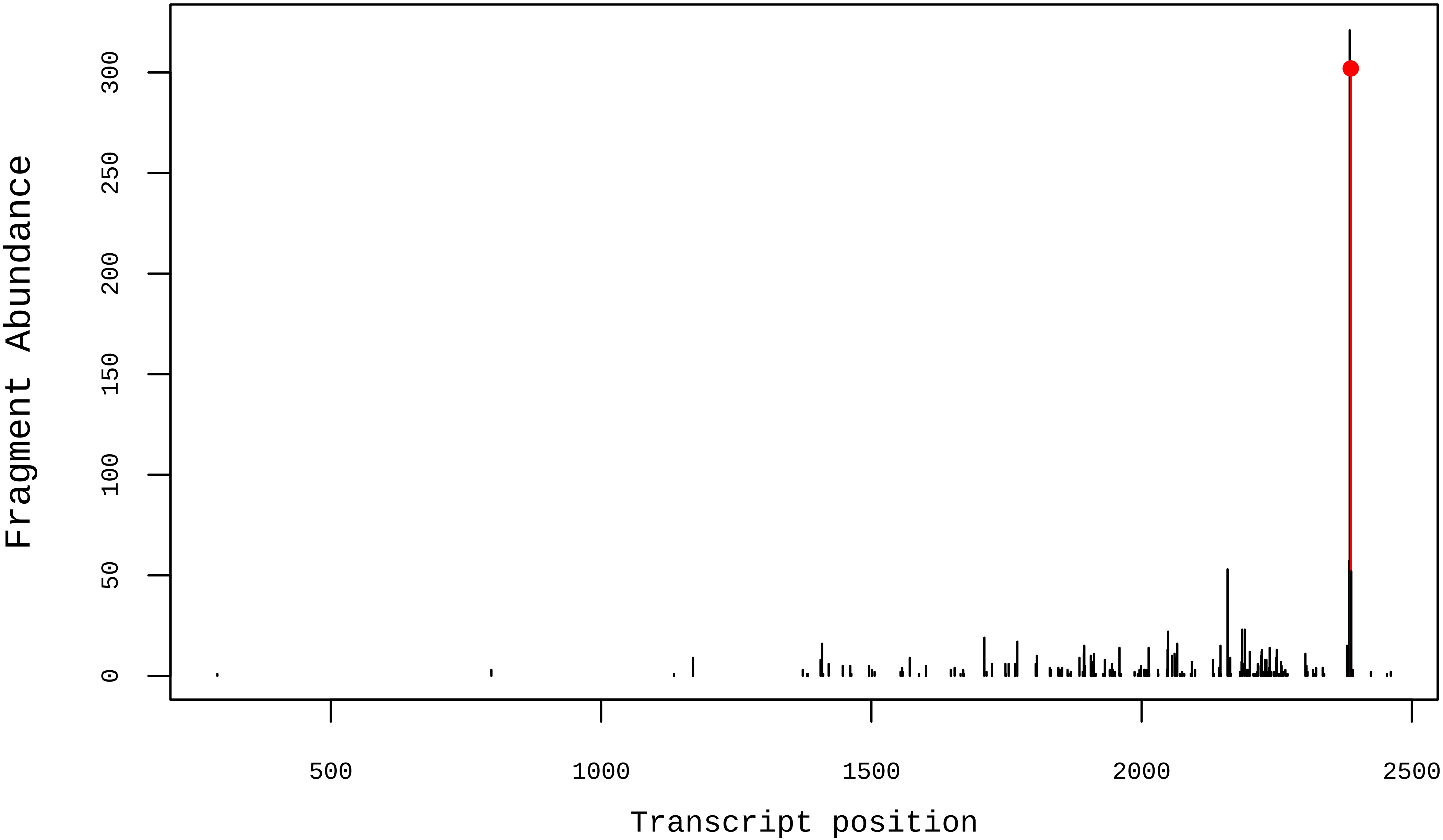

Cleavage site: 2387 Tag abundance: 302 Weighted abundance: 60.4 Category: 2
sRNA abundance: 1 Alignment score: 3 MFE ratio: 0.775 p-value: 0.035

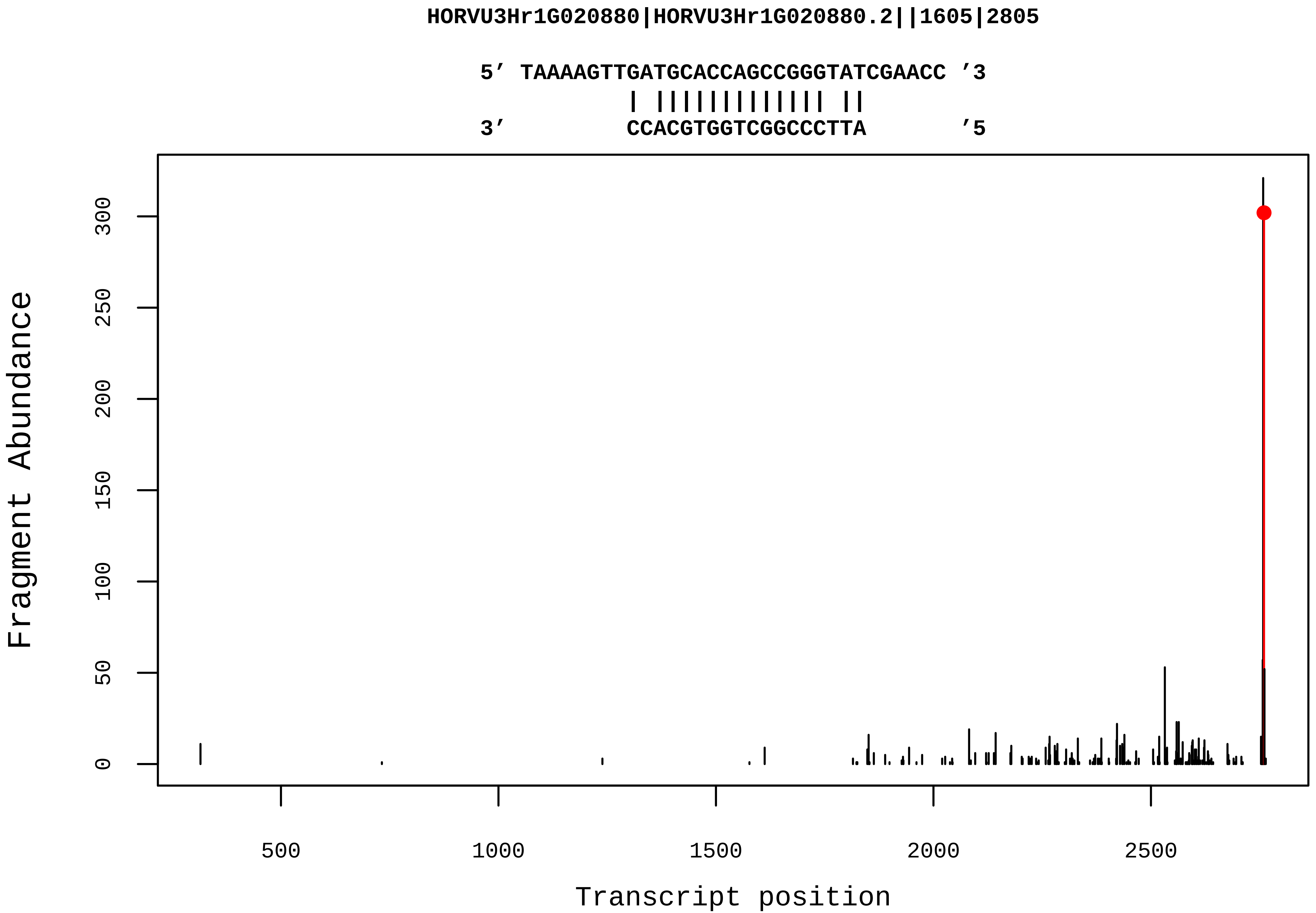

Cleavage site: 2760 Tag abundance: 302 Weighted abundance: 60.4 Category: 2
sRNA abundance: 1 Alignment score: 3 MFE ratio: 0.775 p-value: 0.027

HORVU3Hr1G020880|HORVU3Hr1G020880.3||1518|2718

```
5' TAAAAGTTGATGCACCAGCCGGGTATCGAACC '3
            | ||||||||||||| ||
3'          CCACGTGGTCGGCCCTTA       '5
```

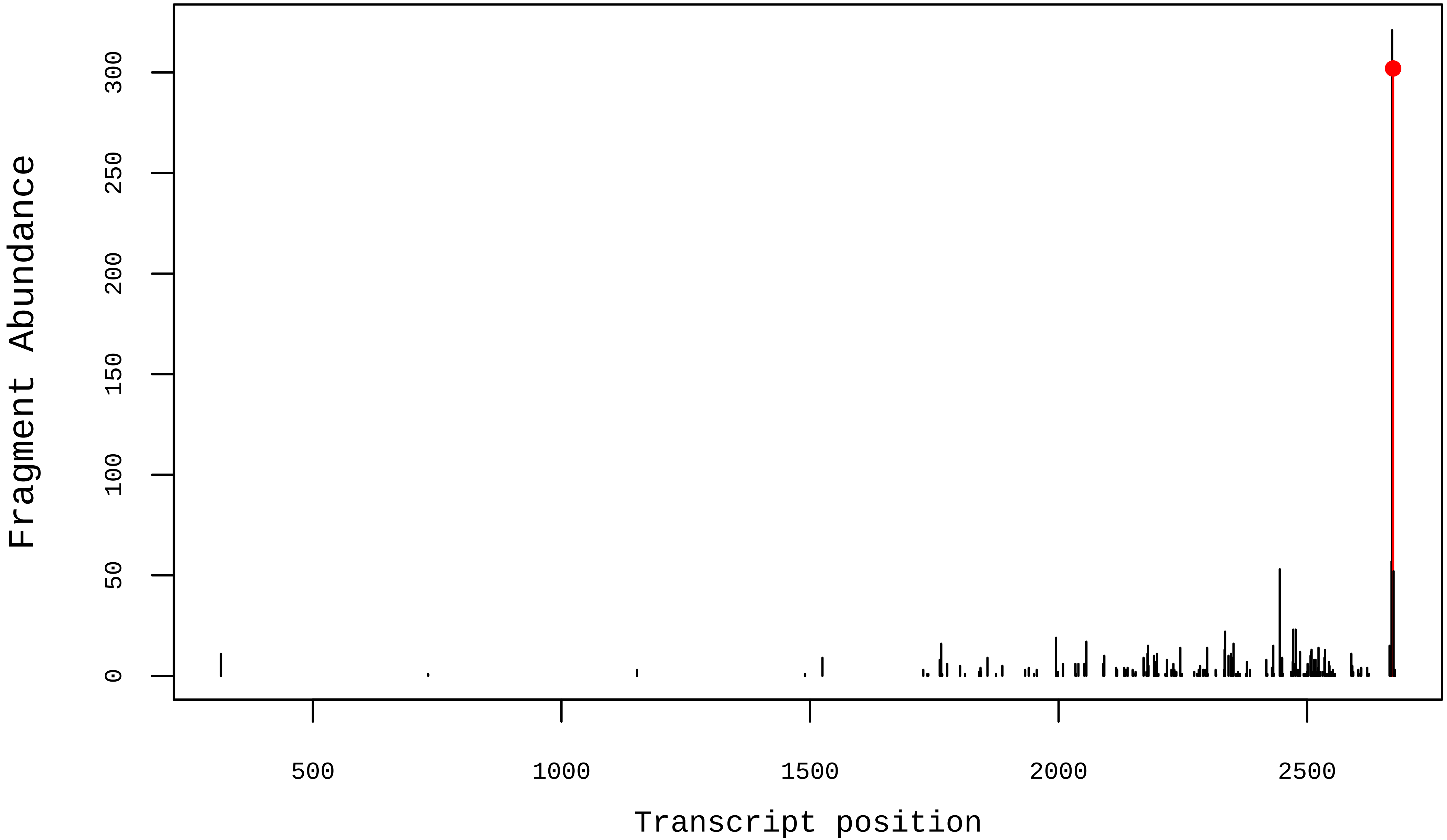

Cleavage site: 2673 Tag abundance: 302 Weighted abundance: 60.4 Category: 2
sRNA abundance: 1 Alignment score: 3 MFE ratio: 0.775 p-value: 0.027

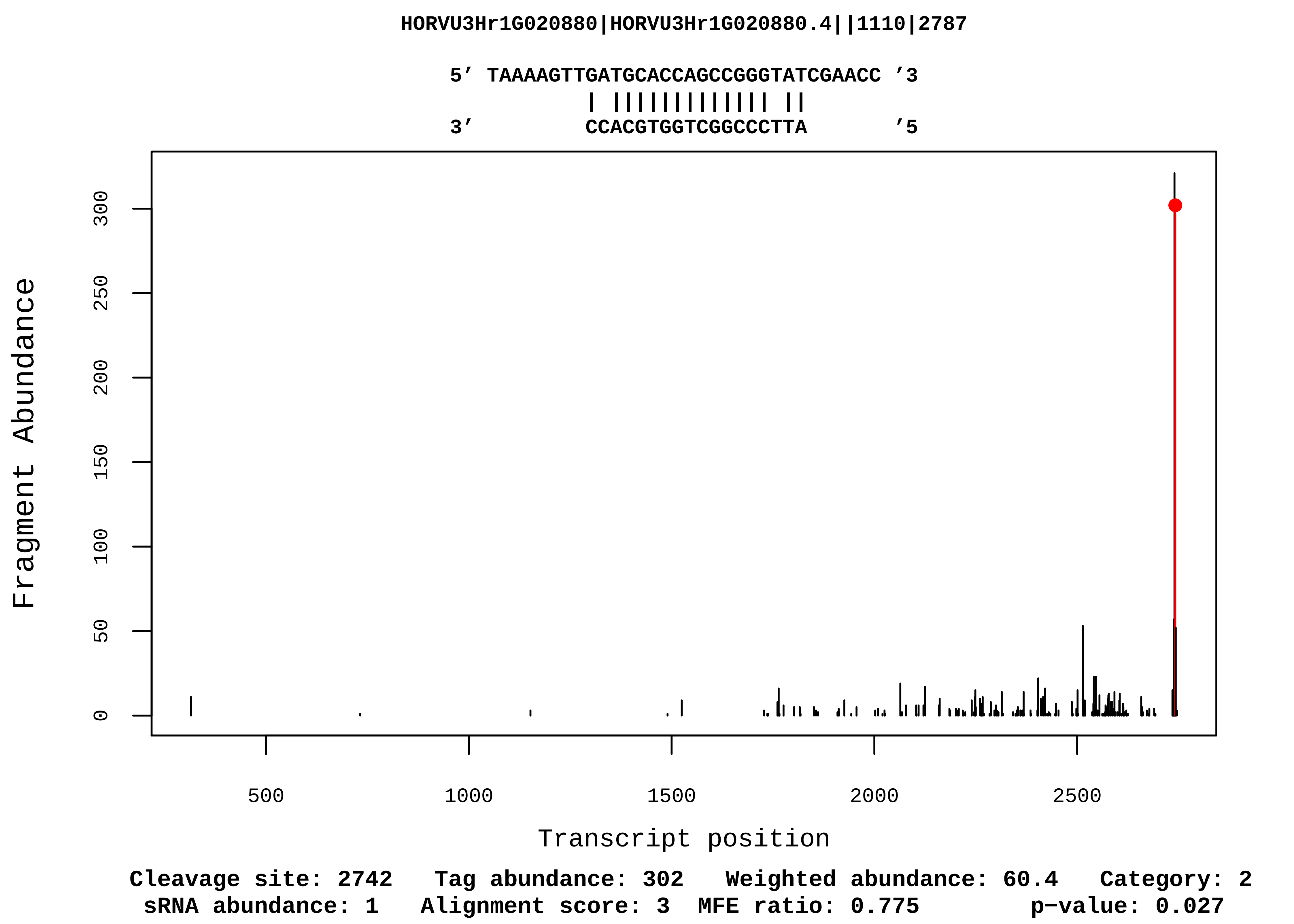
HORVU3Hr1G020880|HORVU3Hr1G020880.4||1110|2787
5' TAAAAGTTGATGCACCAGCCGGGTATCGAACC '3
| ||||||||||||| ||
3' CCACGTGGTCGGCCCTTA '5
Fragment Abundance
0
50
100
150
200
250
300
500
1000
1500
2000
2500
Transcript position
Cleavage site: 2742 Tag abundance: 302 Weighted abundance: 60.4 Category: 2
sRNA abundance: 1 Alignment score: 3 MFE ratio: 0.775 p-value: 0.027

HORVU3Hr1G020880|HORVU3Hr1G020880.5||1773|2572

```
5' TAAAAGTTGATGCACCAGCCGGGTATCGAACC '3
           | ||||||||||||| ||
3'         CCACGTGGTCGGCCCTTA        '5
```

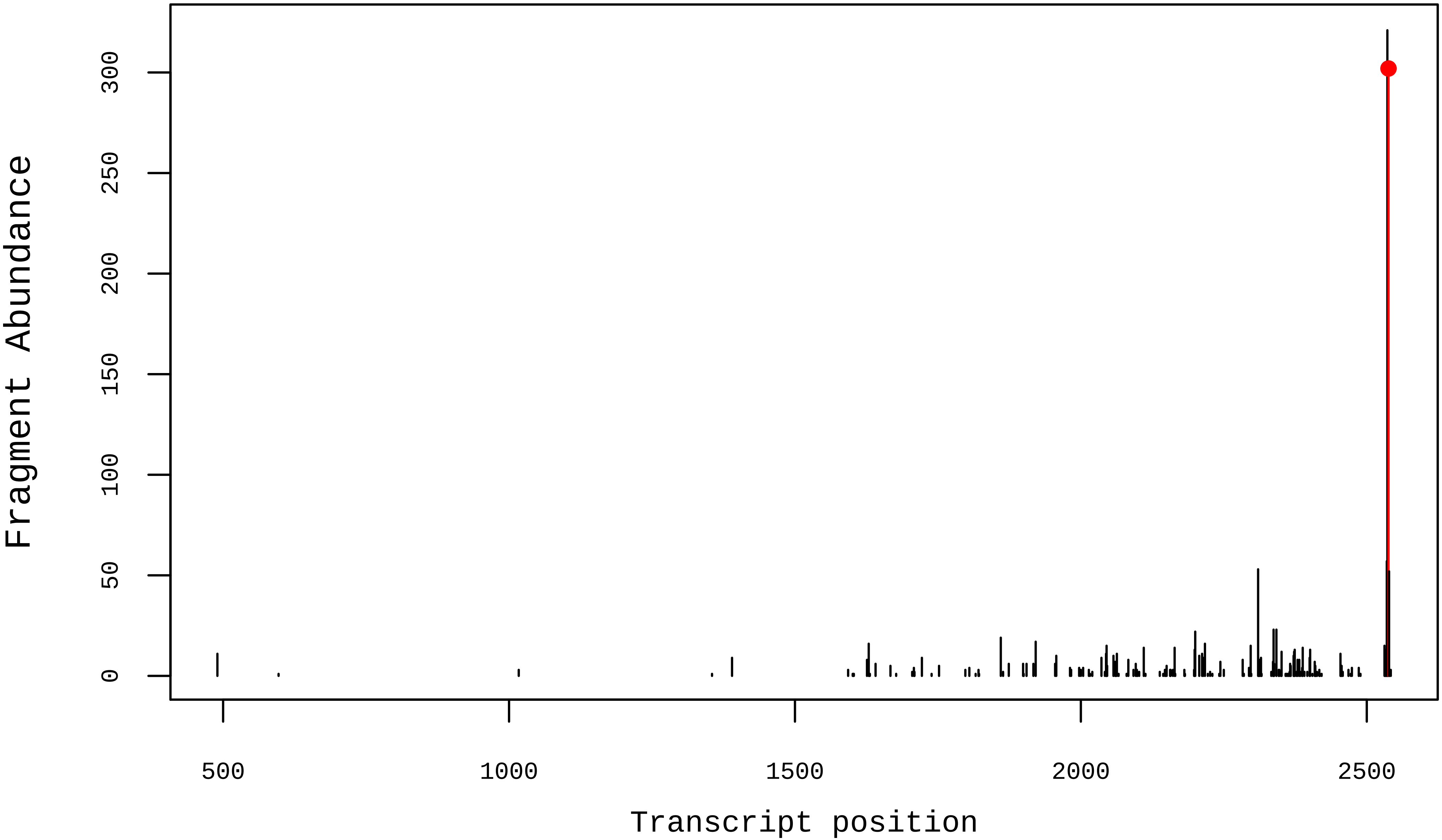

Cleavage site: 2538   Tag abundance: 302   Weighted abundance: 60.4   Category: 2
sRNA abundance: 1   Alignment score: 3   MFE ratio: 0.775   p-value: 0.029

HORVU7Hr1G007540|HORVU7Hr1G007540.1||873|3162

```
5' CATCAGTCAACGG-TGTCGTCCTCCGGGATGTA '3
          ||  || |||||||||||||
3'       CGTAACCAACAGCAGGAGGCC       '5
```

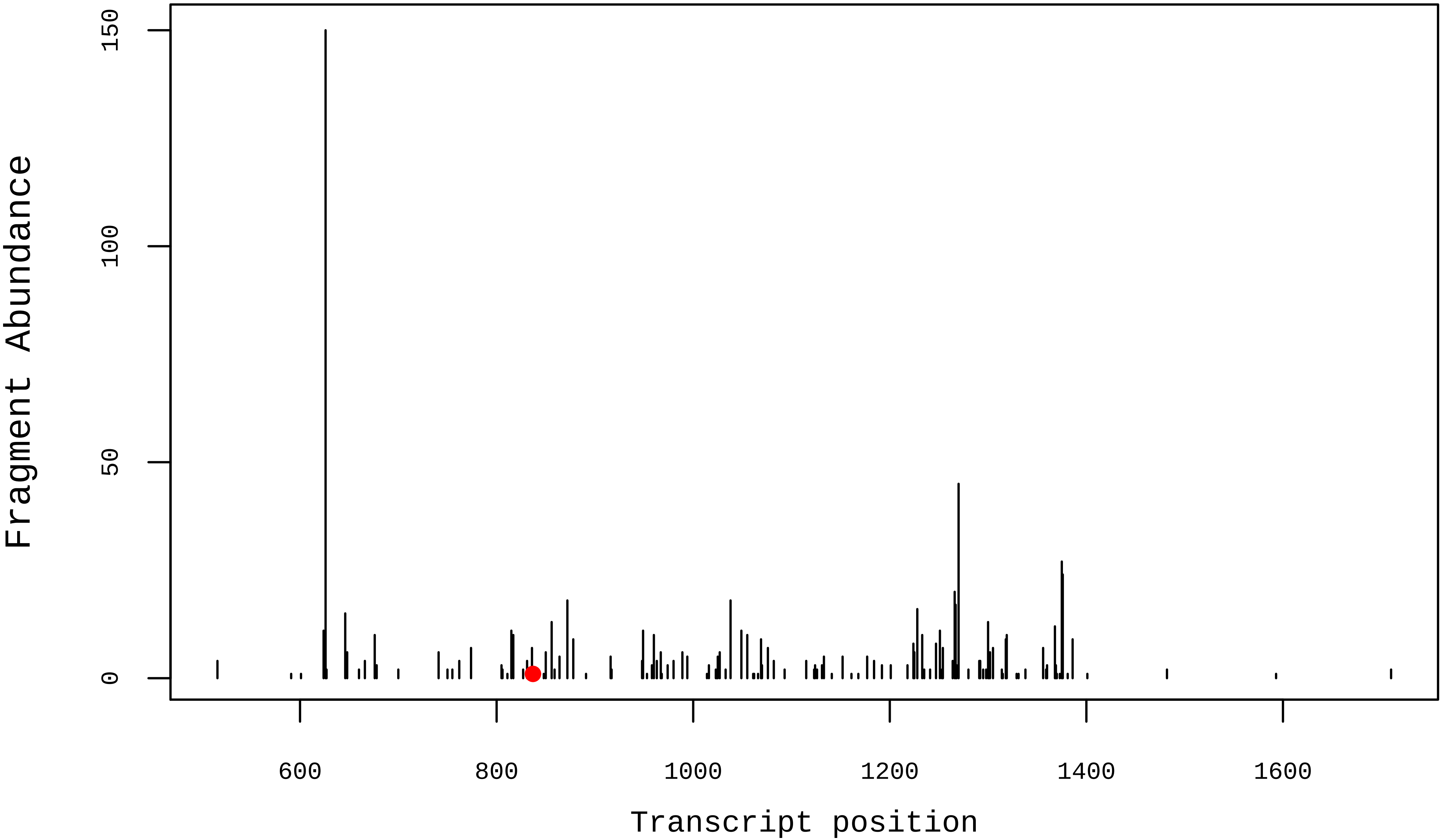

Cleavage site: 837   Tag abundance: 1   Weighted abundance: 0.5   Category: 4
sRNA abundance: 1   Alignment score: 4   MFE ratio: 0.708   p-value: 0.015

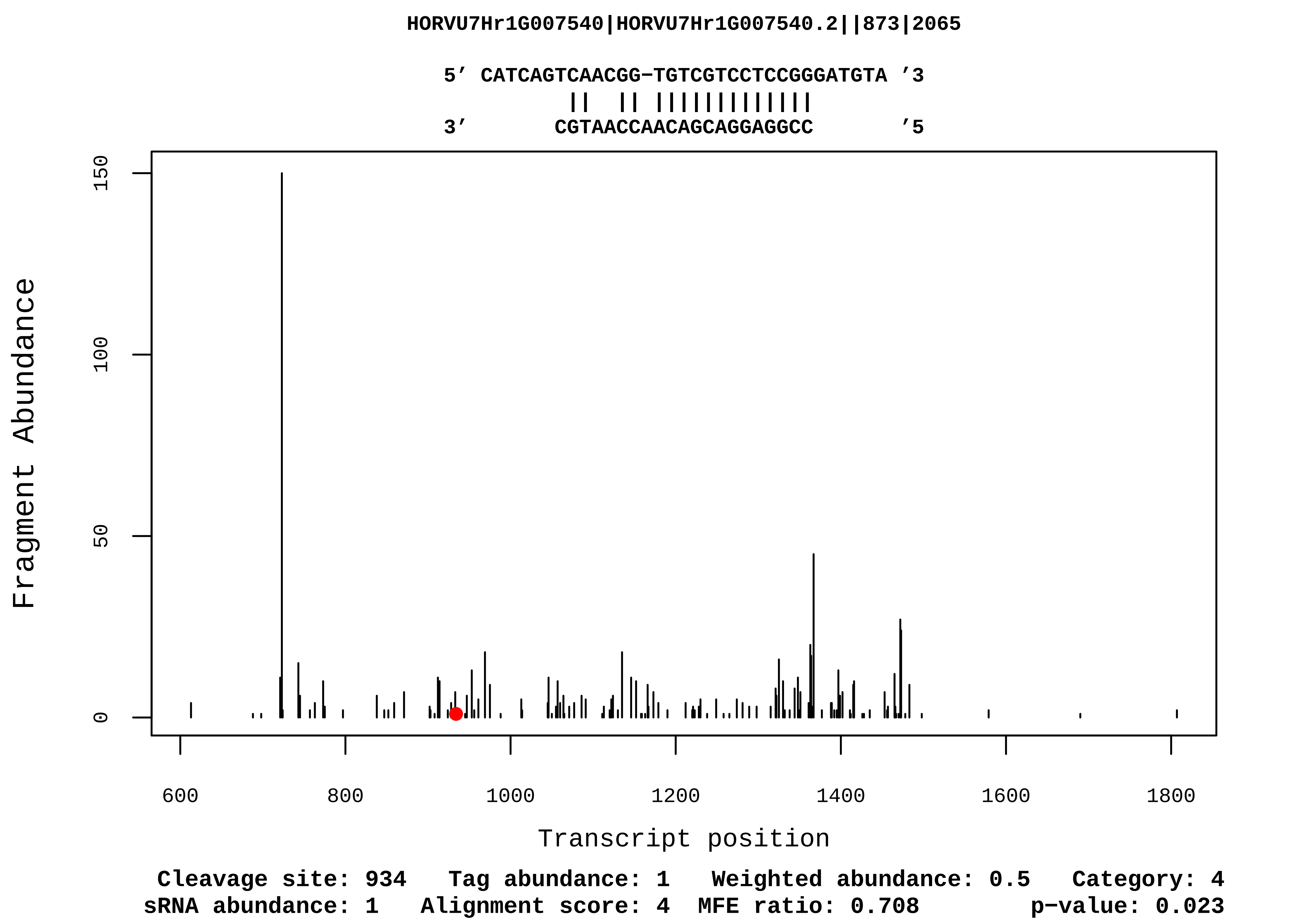
HORVU7Hr1G007540|HORVU7Hr1G007540.2||873|2065
5' CATCAGTCAACGG-TGTCGTCCTCCGGGATGTA '3
|| || |||||||||||||
3' CGTAACCAACAGCAGGAGGCC '5
Fragment Abundance
0
50
100
150
600
800
1000
1200
1400
1600
1800
Transcript position
Cleavage site: 934 Tag abundance: 1 Weighted abundance: 0.5 Category: 4
sRNA abundance: 1 Alignment score: 4 MFE ratio: 0.708 p-value: 0.023

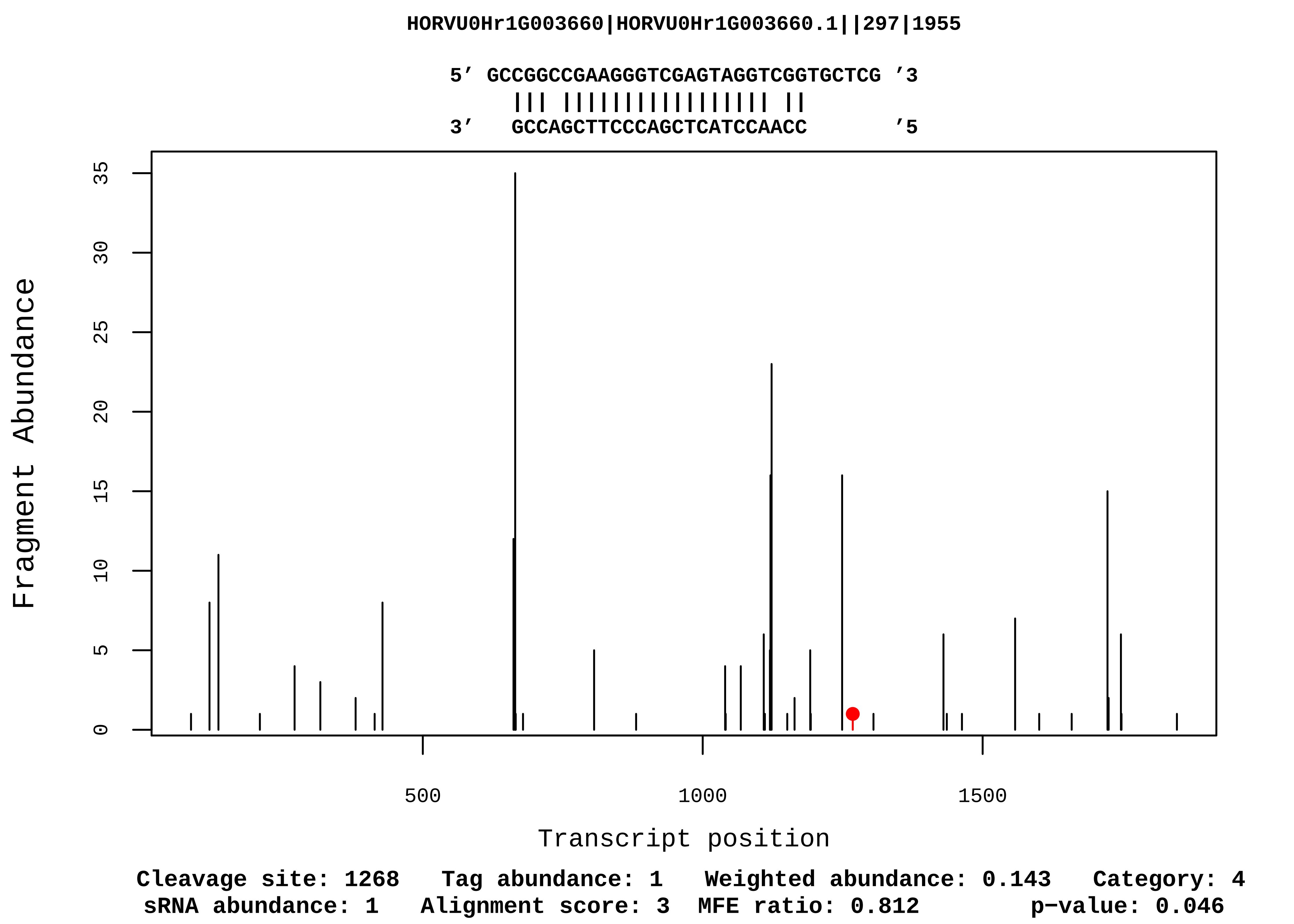
HORVU0Hr1G003660|HORVU0Hr1G003660.1||297|1955
5' GCCGGCCGAAGGGTCGAGTAGGTCGGTGCTCG '3
||| ||||||||||||||||| ||
3' GCCAGCTTCCCAGCTCATCCAACC '5
Fragment Abundance
0
5
10
15
20
25
30
35
500
1000
1500
Transcript position
Cleavage site: 1268 Tag abundance: 1 Weighted abundance: 0.143 Category: 4
sRNA abundance: 1 Alignment score: 3 MFE ratio: 0.812 p-value: 0.046

HORVU0Hr1G023930|HORVU0Hr1G023930.3||396|2180

```
5' GTCGGCGGAAGGGTCGAGTAGGTCGGTGCTCG '3
     |||  |||||||||||||||| ||
3'   GCCAGCTTCCCAGCTCATCCAACC        '5
```

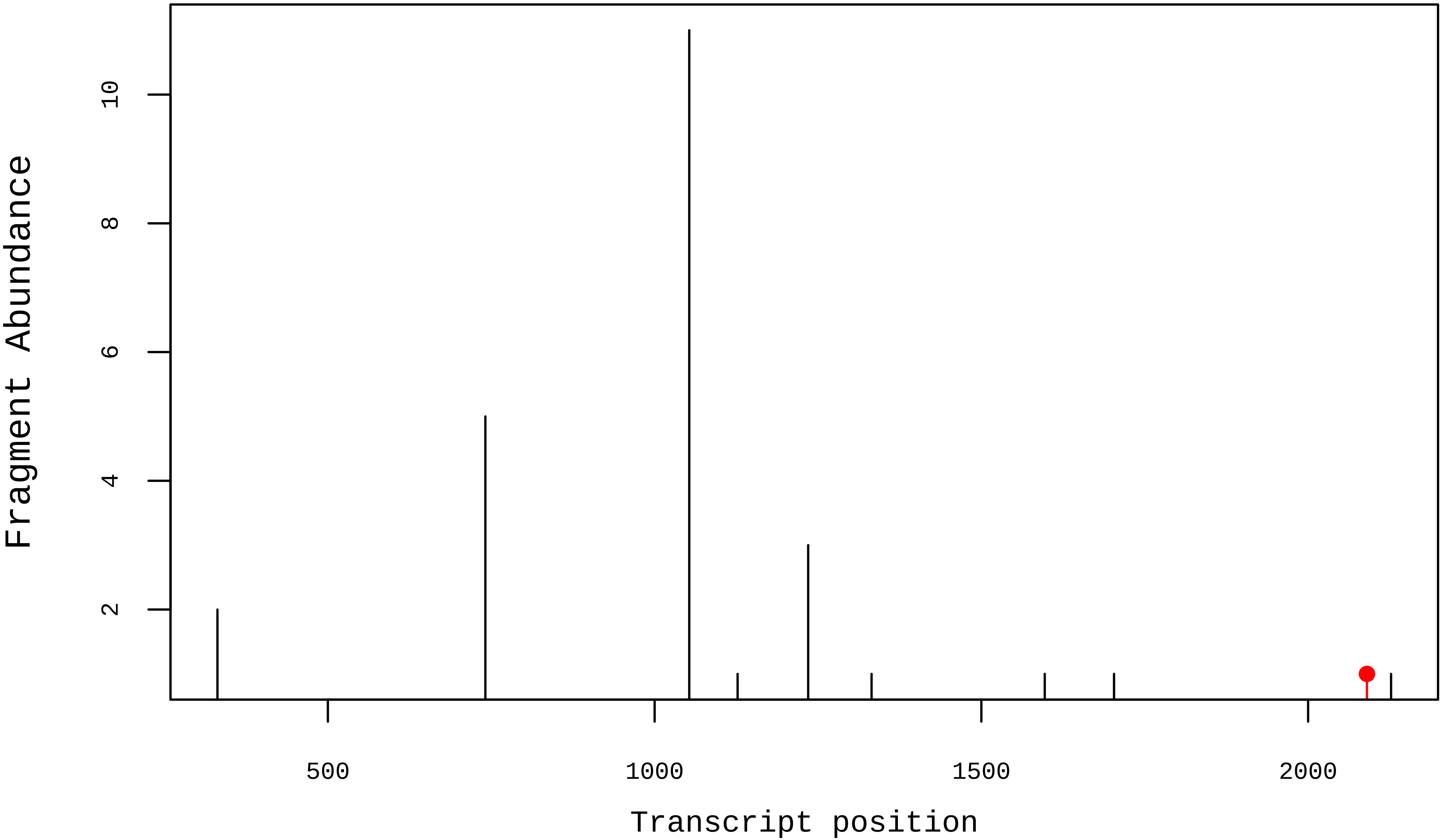

Cleavage site: 2090 Tag abundance: 1 Weighted abundance: 0.143 Category: 4
sRNA abundance: 1 Alignment score: 4 MFE ratio: 0.782 p-value: 0.019

HORVU2Hr1G072960|HORVU2Hr1G072960.1||600|3353

```
5' GCCGGCCGAAGGGTCGAGTAGGTCGGTGCTCG '3
      ||| ||||||||||||||||| ||
3'    GCCAGCTTCCCAGCTCATCCAACC       '5
```

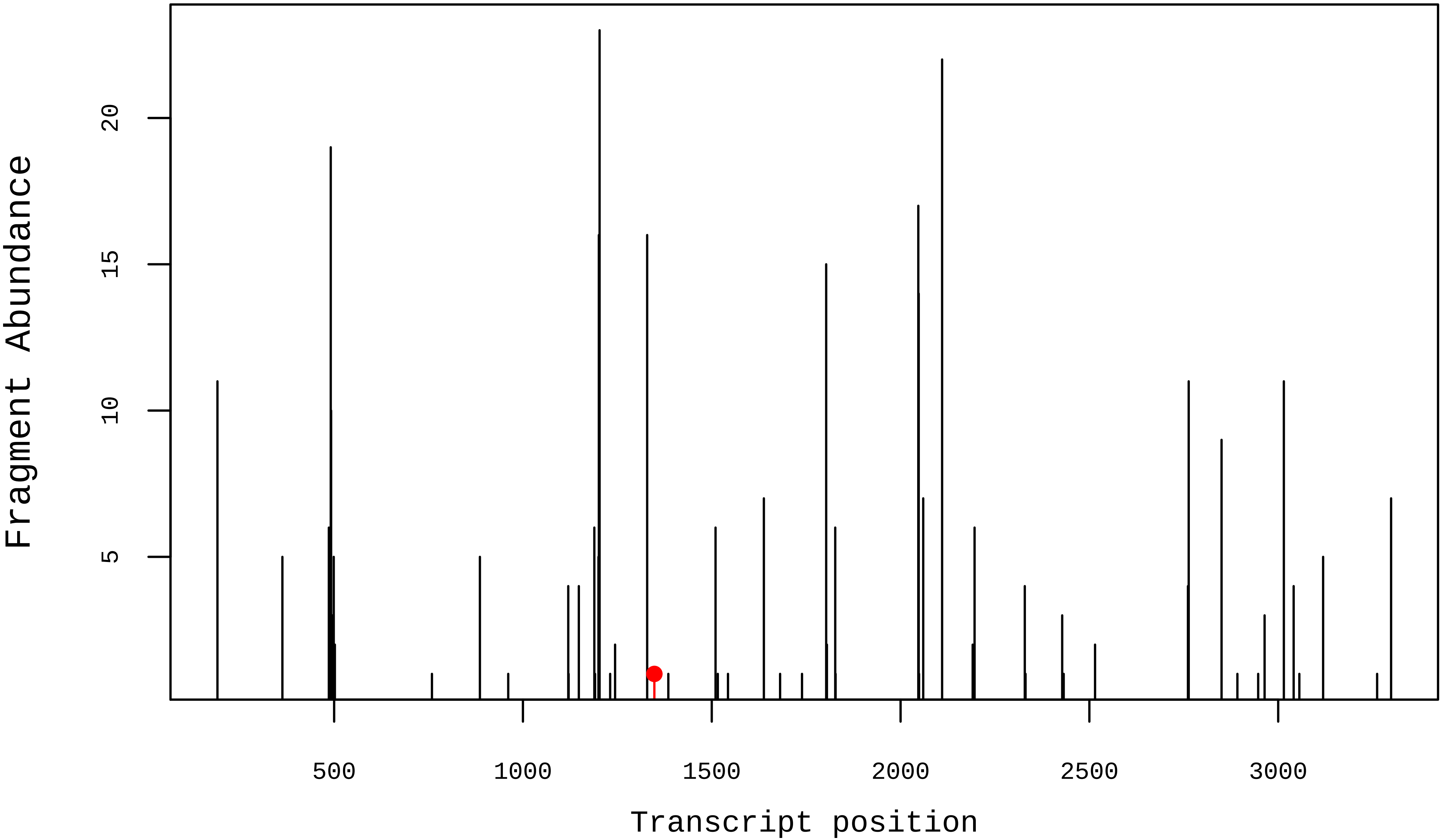

Cleavage site: 1348   Tag abundance: 1   Weighted abundance: 0.143   Category: 4
sRNA abundance: 1   Alignment score: 3   MFE ratio: 0.812   p-value: 0.03

HORVU5Hr1G015600|HORVU5Hr1G015600.1||156|510

```
5' GCCGGCCGAAGGGTCGAGTAGGTCGGTGCTCG '3
      ||| ||||||||||||||||| ||
3'    GCCAGCTTCCCAGCTCATCCAACC       '5
```

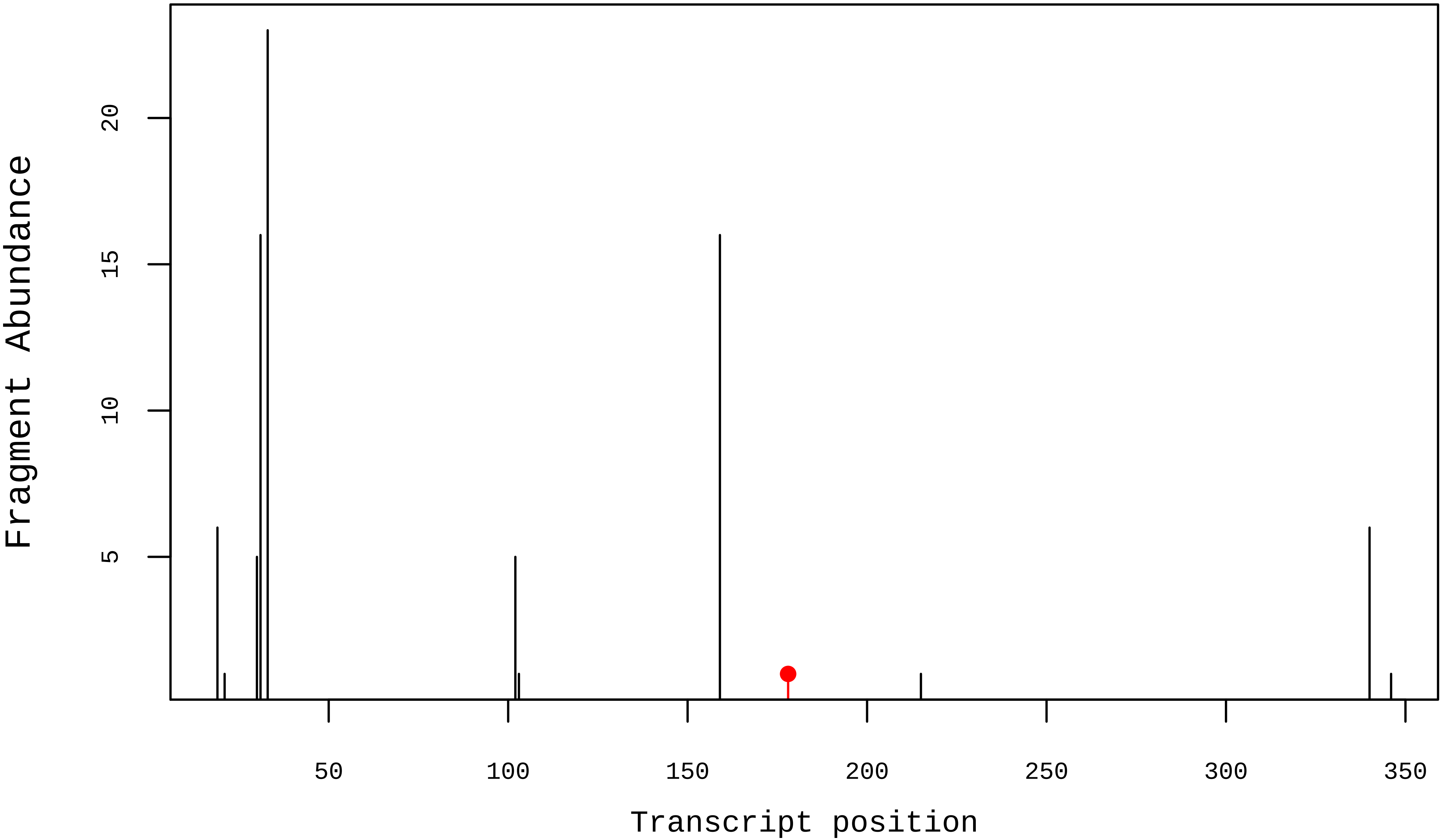

Cleavage site: 178 Tag abundance: 1 Weighted abundance: 0.143 Category: 4
sRNA abundance: 1 Alignment score: 3 MFE ratio: 0.812 p-value: 0.05

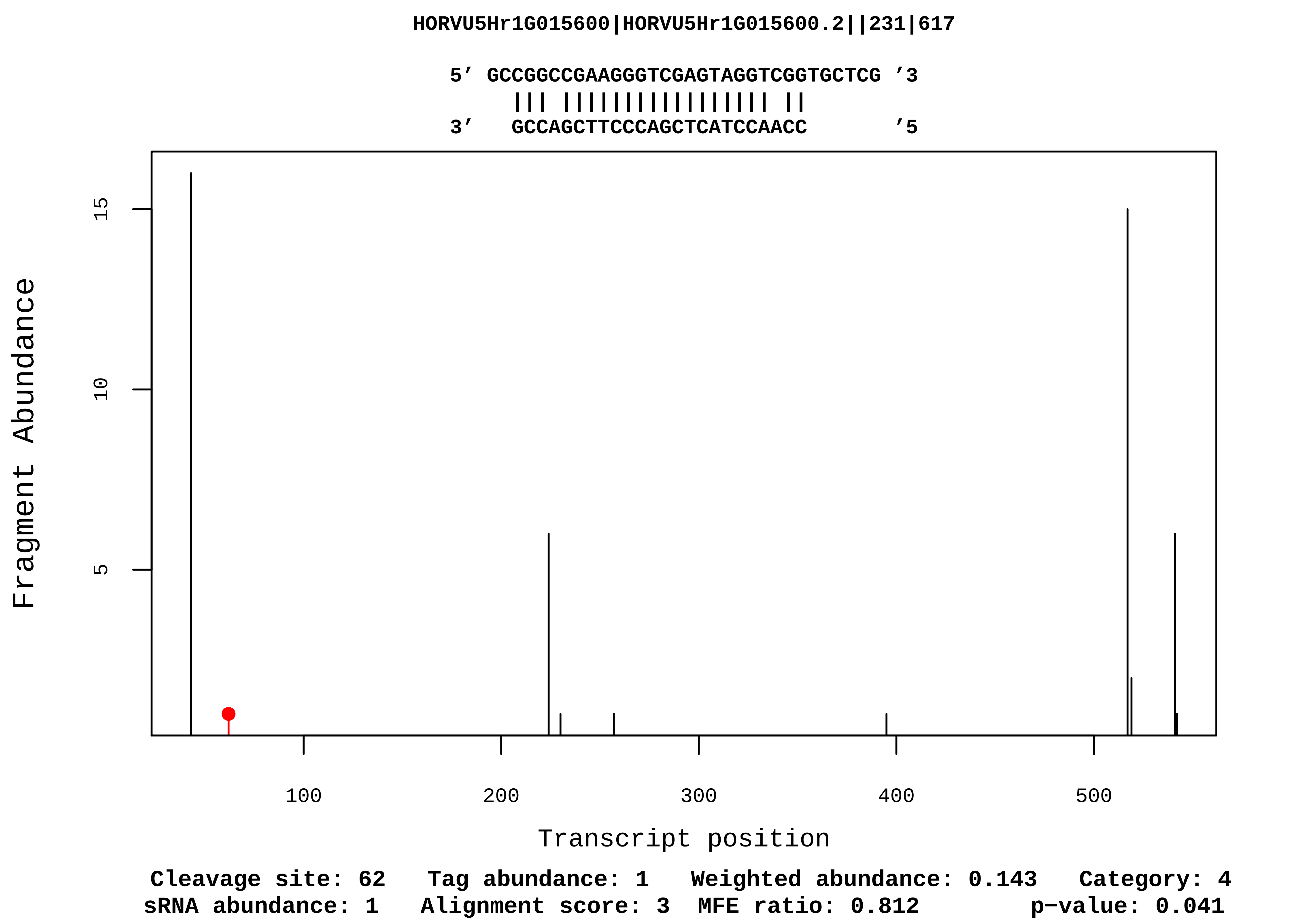
HORVU5Hr1G015600|HORVU5Hr1G015600.2||231|617
5' GCCGGCCGAAGGGTCGAGTAGGTCGGTGCTCG '3
||| ||||||||||||||||| ||
3' GCCAGCTTCCCAGCTCATCCAACC '5
Fragment Abundance
15
10
5
100
200
300
400
500
Transcript position
Cleavage site: 62 Tag abundance: 1 Weighted abundance: 0.143 Category: 4
sRNA abundance: 1 Alignment score: 3 MFE ratio: 0.812 p-value: 0.041

HORVU5Hr1G015600|HORVU5Hr1G015600.3||276|1709

```
5' GCCGGCCGAAGGGTCGAGTAGGTCGGTGCTCG '3
       ||| ||||||||||||||||| ||
3'   GCCAGCTTCCCAGCTCATCCAACC       '5
```

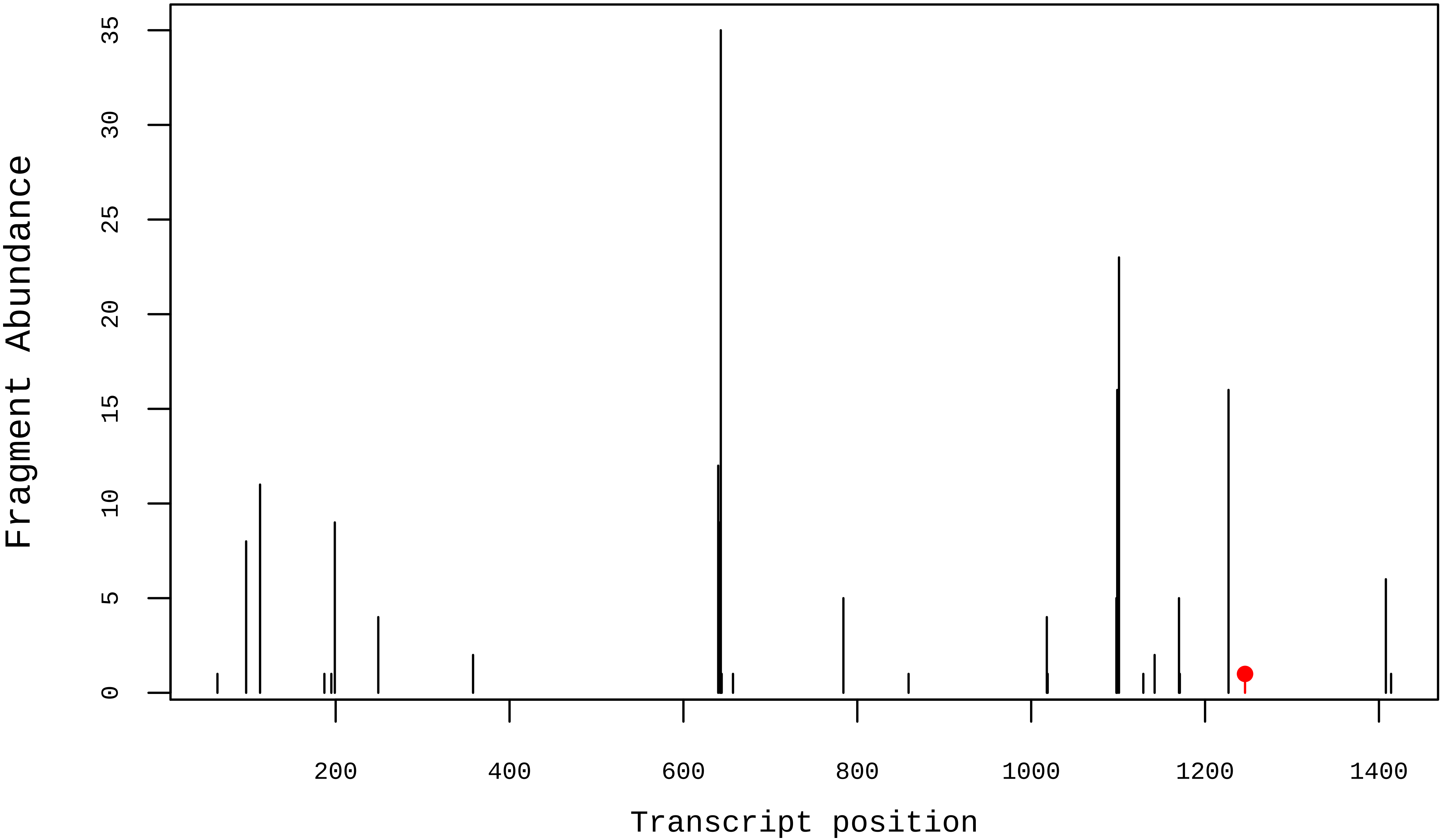

Cleavage site: 1246 Tag abundance: 1 Weighted abundance: 0.143 Category: 4
sRNA abundance: 1 Alignment score: 3 MFE ratio: 0.812 p-value: 0.032

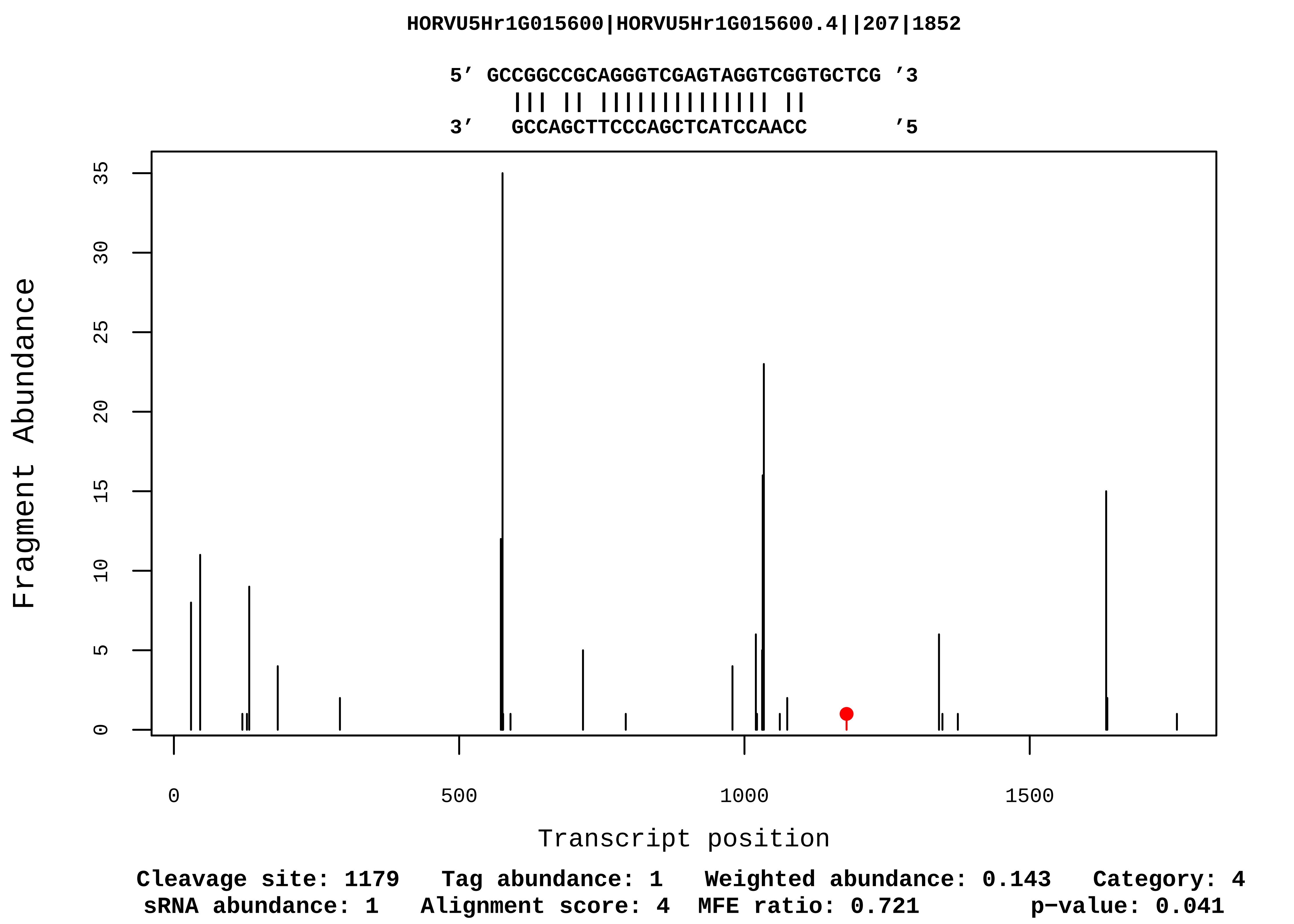
HORVU5Hr1G015600|HORVU5Hr1G015600.4||207|1852
5' GCCGGCCGCAGGGTCGAGTAGGTCGGTGCTCG '3
3' GCCAGCTTCCCAGCTCATCCAACC '5
Fragment Abundance
0
5
10
15
20
25
30
35
0
500
1000
1500
Transcript position
Cleavage site: 1179 Tag abundance: 1 Weighted abundance: 0.143 Category: 4
sRNA abundance: 1 Alignment score: 4 MFE ratio: 0.721 p-value: 0.041

HORVU0Hr1G003660|HORVU0Hr1G003660.1||297|1955

```
5' GCCGGCCGAAGGGTCGAGTAGGTCGGTGCTCG '3
      ||||||||||||||||||||||| |
3'   GCCGGCTTCCCAGCTCATCCAGC-A      '5
```

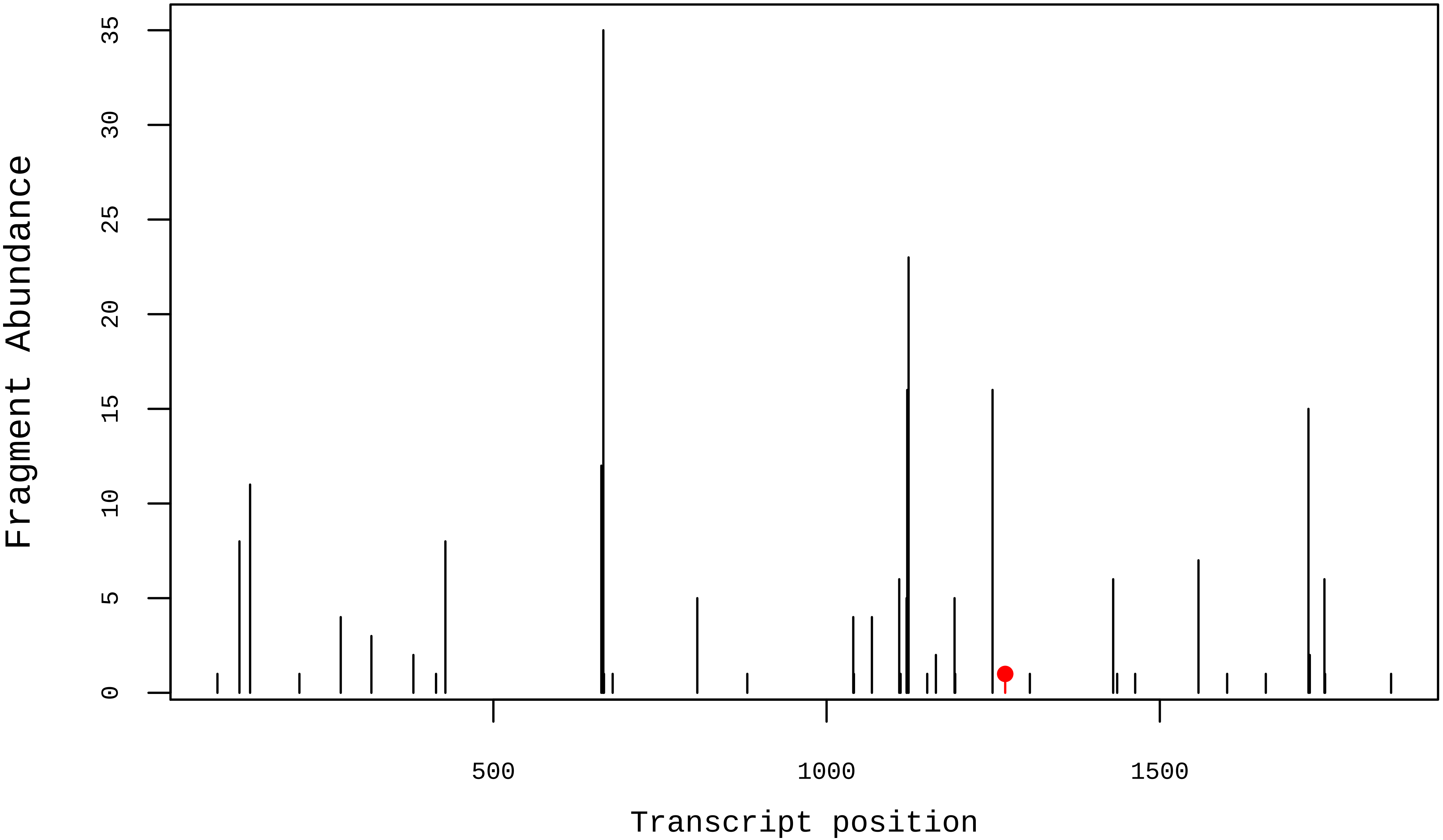

Cleavage site: 1268   Tag abundance: 1   Weighted abundance: 0.143   Category: 4
sRNA abundance: 1   Alignment score: 1   MFE ratio: 0.994   p-value: 0.046

HORVU0Hr1G023930|HORVU0Hr1G023930.3||396|2180

```
5' GTCGGCGGAAGGGTCGAGTAGGTCGGTGCTCG '3
      |||| |||||||||||||||||| |
3'    GCCGGCTTCCCAGCTCATCCAGC-A       '5
```

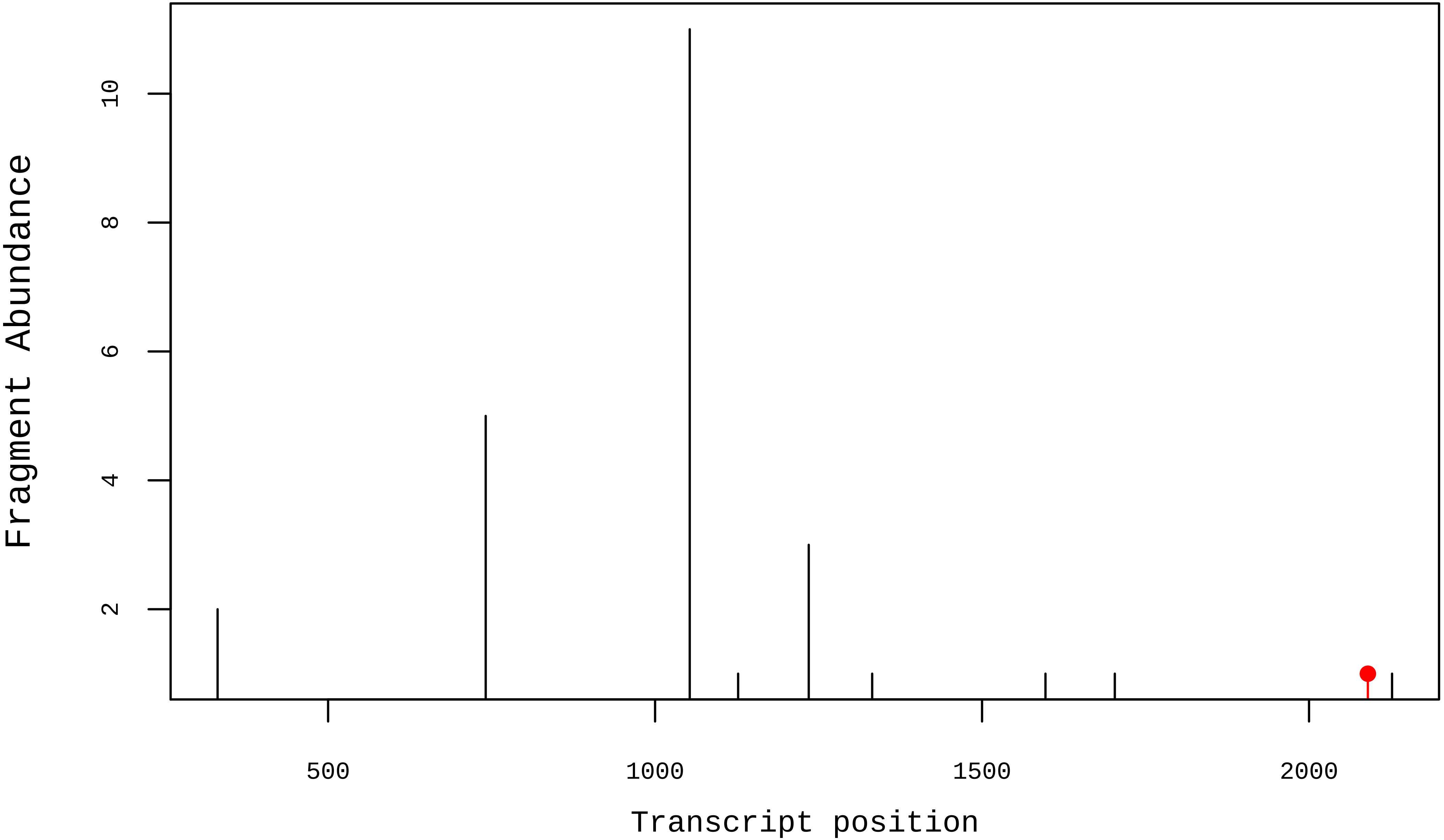

Cleavage site: 2090   Tag abundance: 1   Weighted abundance: 0.143   Category: 4
sRNA abundance: 1   Alignment score: 2   MFE ratio: 0.911   p-value: 0.019

HORVU2Hr1G072960|HORVU2Hr1G072960.1||600|3353

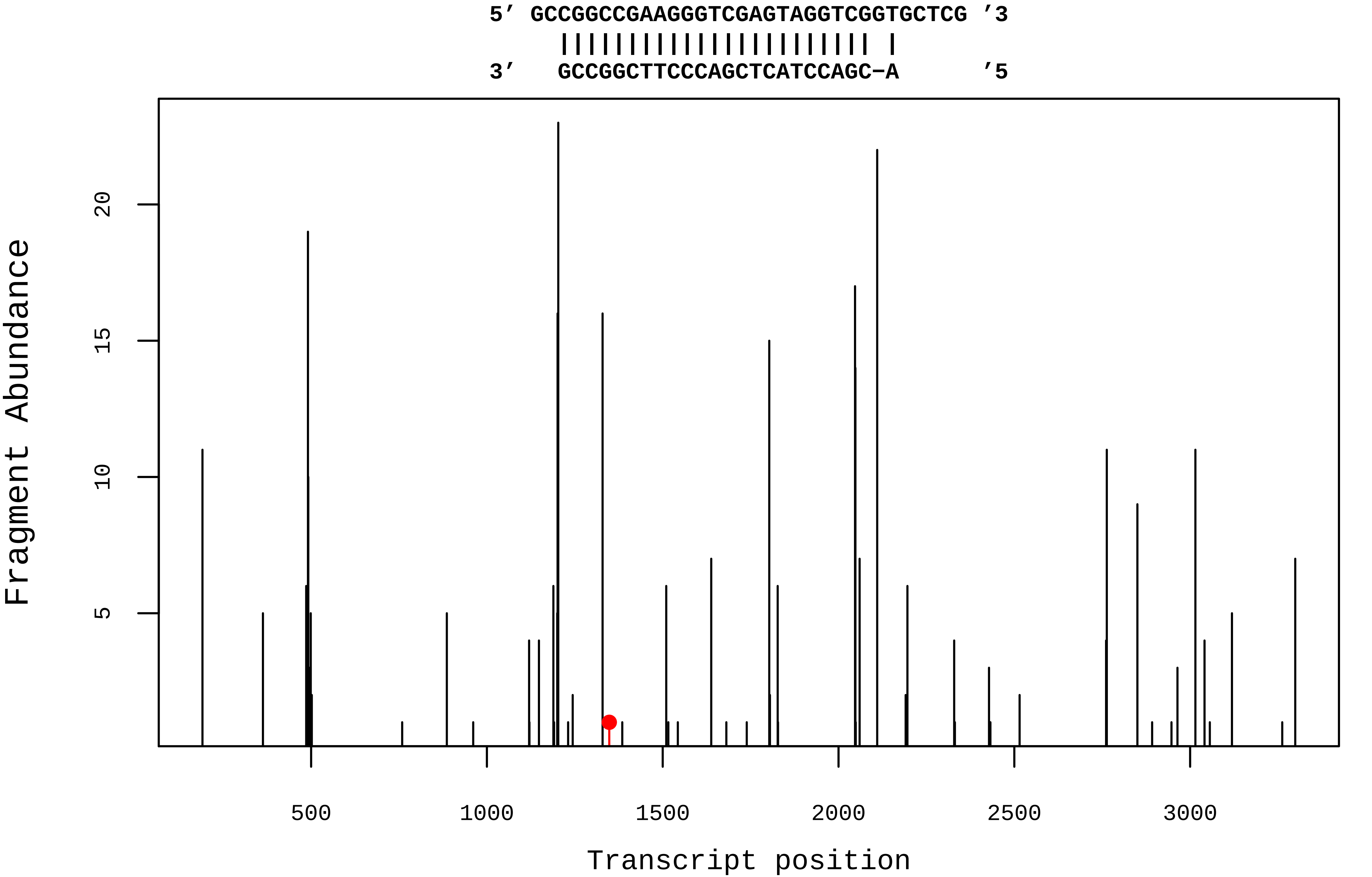

Cleavage site: 1348 Tag abundance: 1 Weighted abundance: 0.143 Category: 4
sRNA abundance: 1 Alignment score: 1 MFE ratio: 0.994 p-value: 0.03

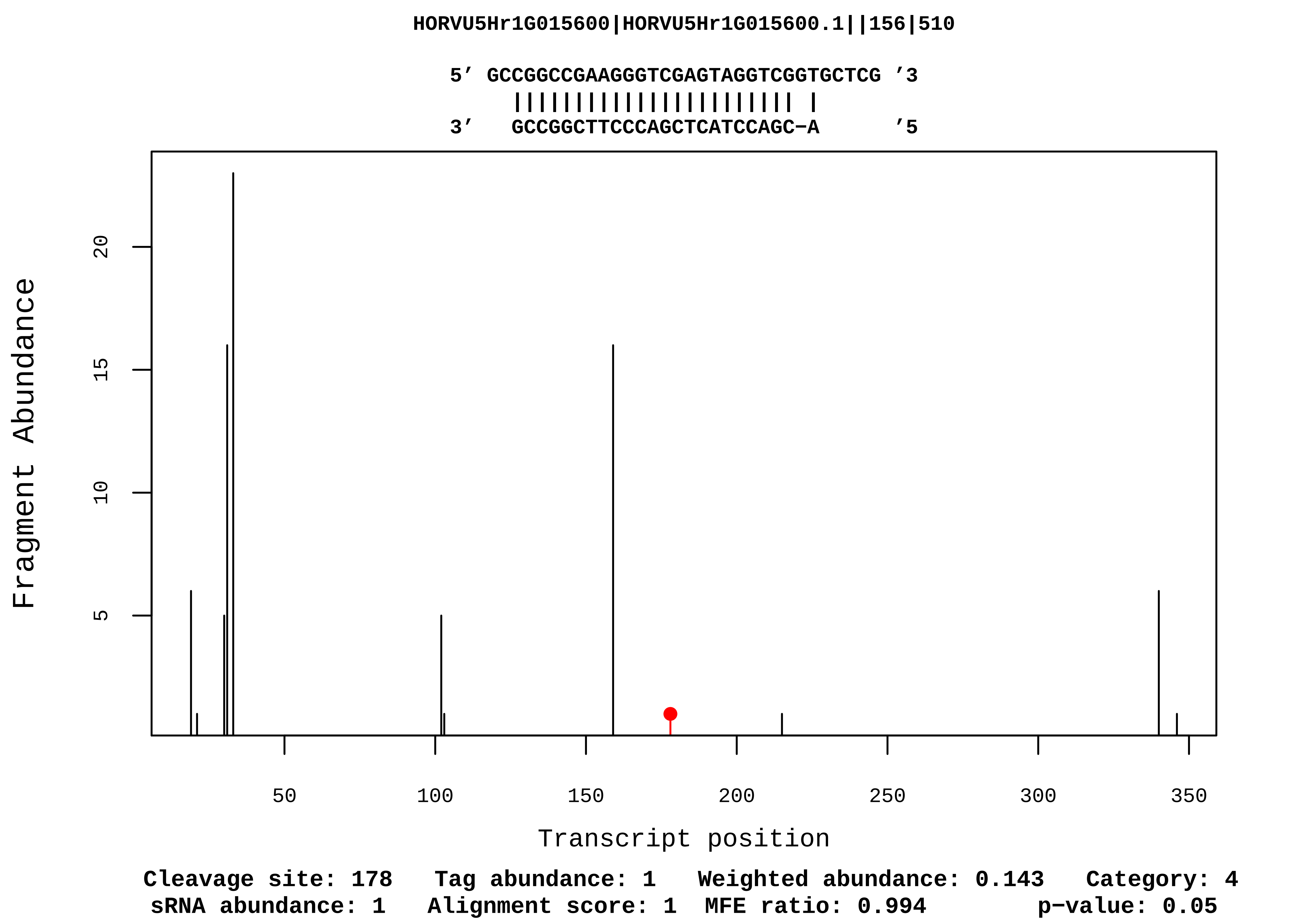
HORVU5Hr1G015600|HORVU5Hr1G015600.1||156|510
5' GCCGGCCGAAGGGTCGAGTAGGTCGGTGCTCG '3
3' GCCGGCTTCCCAGCTCATCCAGC-A '5
Fragment Abundance
5
10
15
20
50
100
150
200
250
300
350
Transcript position
Cleavage site: 178 Tag abundance: 1 Weighted abundance: 0.143 Category: 4
sRNA abundance: 1 Alignment score: 1 MFE ratio: 0.994 p-value: 0.05

HORVU5Hr1G015600|HORVU5Hr1G015600.2||231|617

```
5' GCCGGCCGAAGGGTCGAGTAGGTCGGTGCTCG '3
      |||||||||||||||||||||||| |
3'    GCCGGCTTCCCAGCTCATCCAGC-A      '5
```

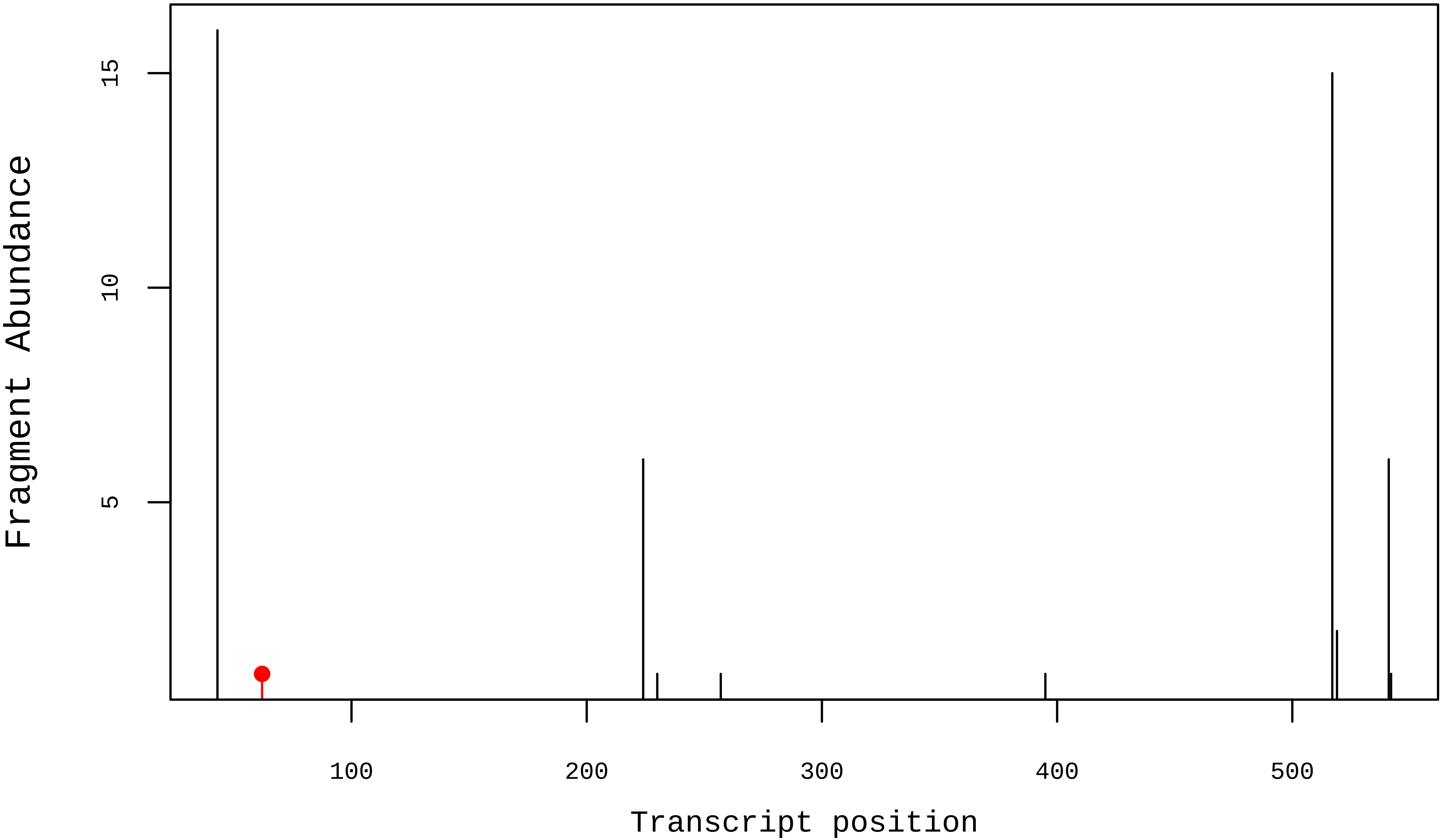

Cleavage site: 62 Tag abundance: 1 Weighted abundance: 0.143 Category: 4
sRNA abundance: 1 Alignment score: 1 MFE ratio: 0.994 p-value: 0.041

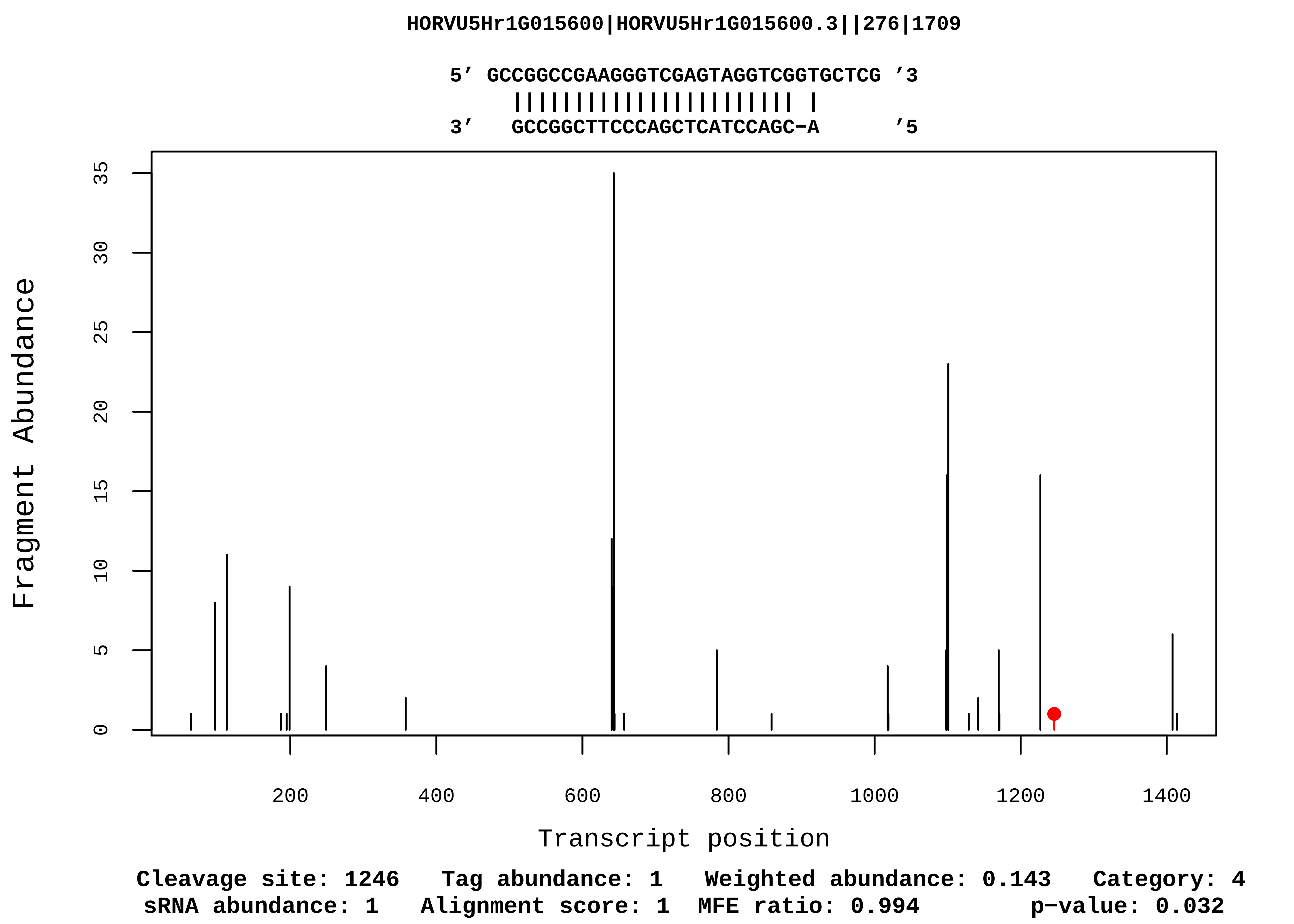
HORVU5Hr1G015600|HORVU5Hr1G015600.3||276|1709
5' GCCGGCCGAAGGGTCGAGTAGGTCGGTGCTCG '3
|||||||||||||||||||||||| |
3' GCCGGCTTCCCAGCTCATCCAGC-A '5
Fragment Abundance
0
5
10
15
20
25
30
35
200
400
600
800
1000
1200
1400
Transcript position
Cleavage site: 1246 Tag abundance: 1 Weighted abundance: 0.143 Category: 4
sRNA abundance: 1 Alignment score: 1 MFE ratio: 0.994 p-value: 0.032

HORVU5Hr1G015600|HORVU5Hr1G015600.4||207|1852

```
5' GCCGGCCGCAGGGTCGAGTAGGTCGGTGCTCG '3
       |||||| |||||||||||||||| |
3'    GCCGGCTTCCCAGCTCATCCAGC-A      '5
```

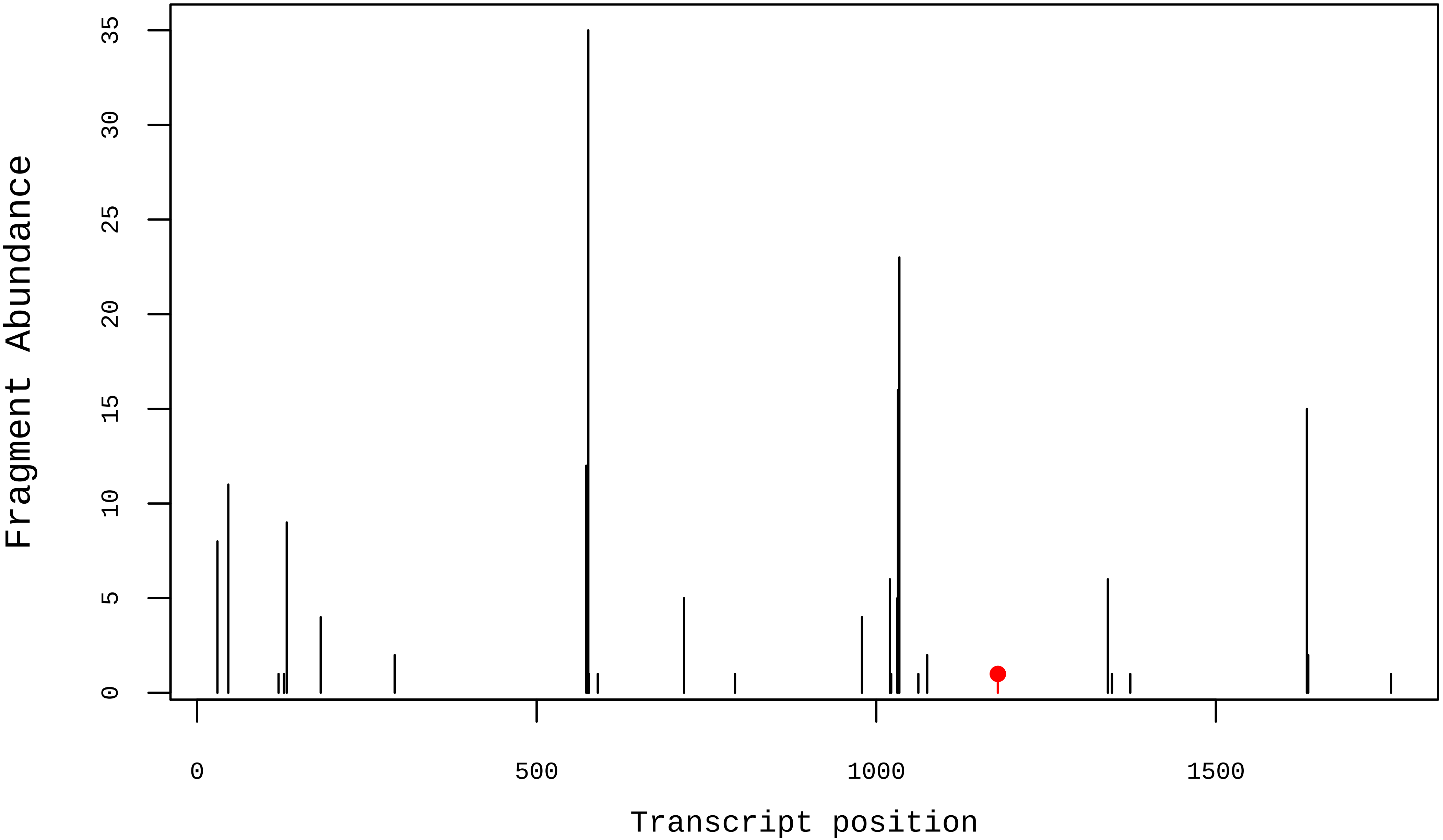

Cleavage site: 1179   Tag abundance: 1   Weighted abundance: 0.143   Category: 4
sRNA abundance: 1   Alignment score: 2   MFE ratio: 0.907   p-value: 0.041

HORVU5Hr1G007500|HORVU5Hr1G007500.1||411|812

```
5' TACGCCAACGG-CGAGCAATCTGCCATCACCCA '3
              |  |||||||||o||||
3'            CGAGCTCGTTAGGCGGTT       '5
```

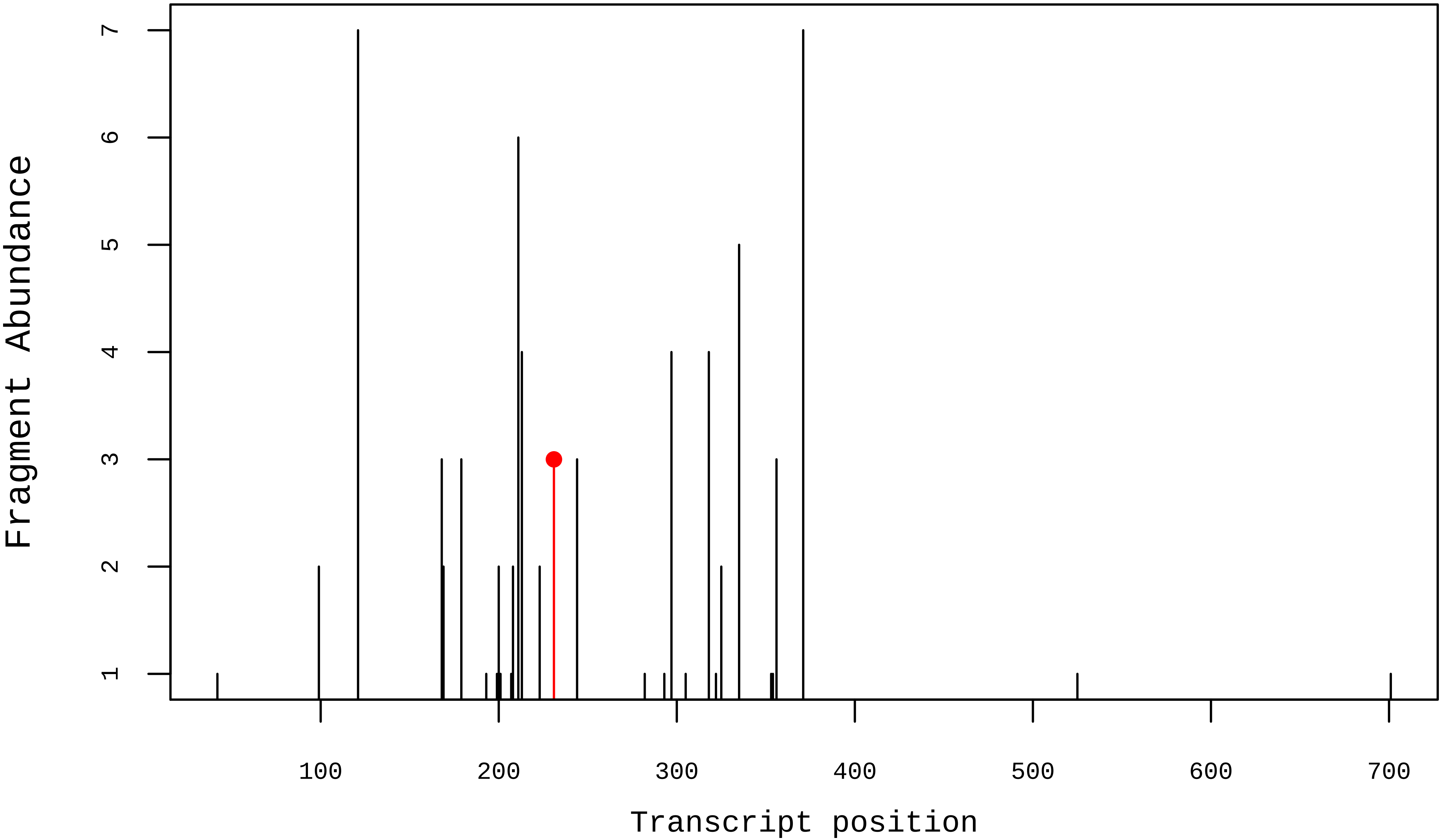

Cleavage site: 231 Tag abundance: 3 Weighted abundance: 1.5 Category: 3
sRNA abundance: 1 Alignment score: 4 MFE ratio: 0.742 p-value: 0.028

HORVU0Hr1G023930|HORVU0Hr1G023930.1||297|1937

```
5' CGTCAGCCTTTTATCTAATAAATGCGCCCCTC '3
     |||||||||||||||||||||||||
3'  CAGTCGGAAAATAGATTATTTACGC       '5
```

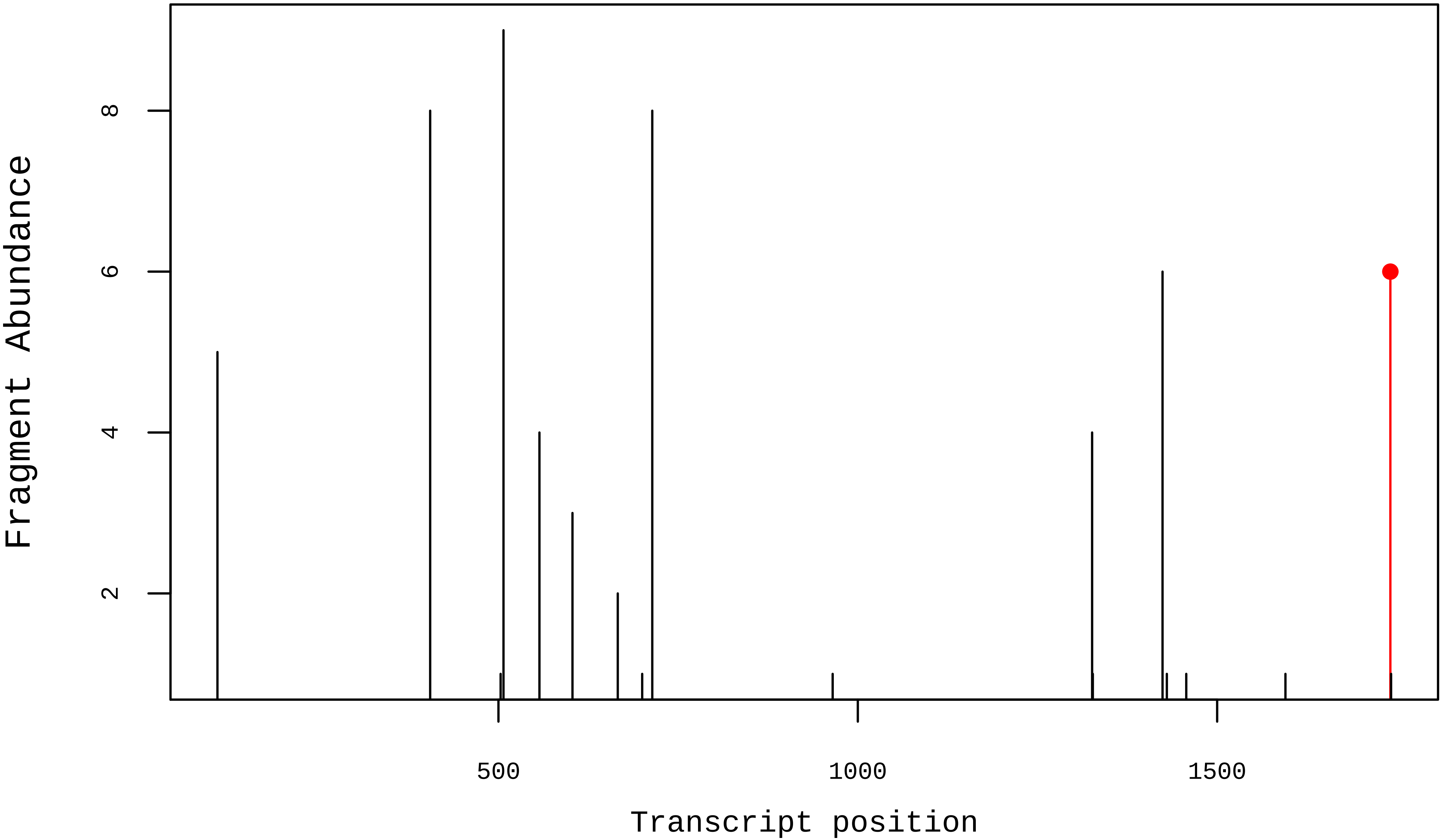

Cleavage site: 1741   Tag abundance: 6   Weighted abundance: 0.75   Category: 2
sRNA abundance: 1   Alignment score: 0   MFE ratio: 1   p-value: 0.017

HORVU5Hr1G015600|HORVU5Hr1G015600.16||228|588

```
5' CGTCAGCCTTTTATCTAATAAATGCGCCCCTC '3
     ||||||||||||||||||||||||
3'   CAGTCGGAAAATAGATTATTTACGC       '5
```

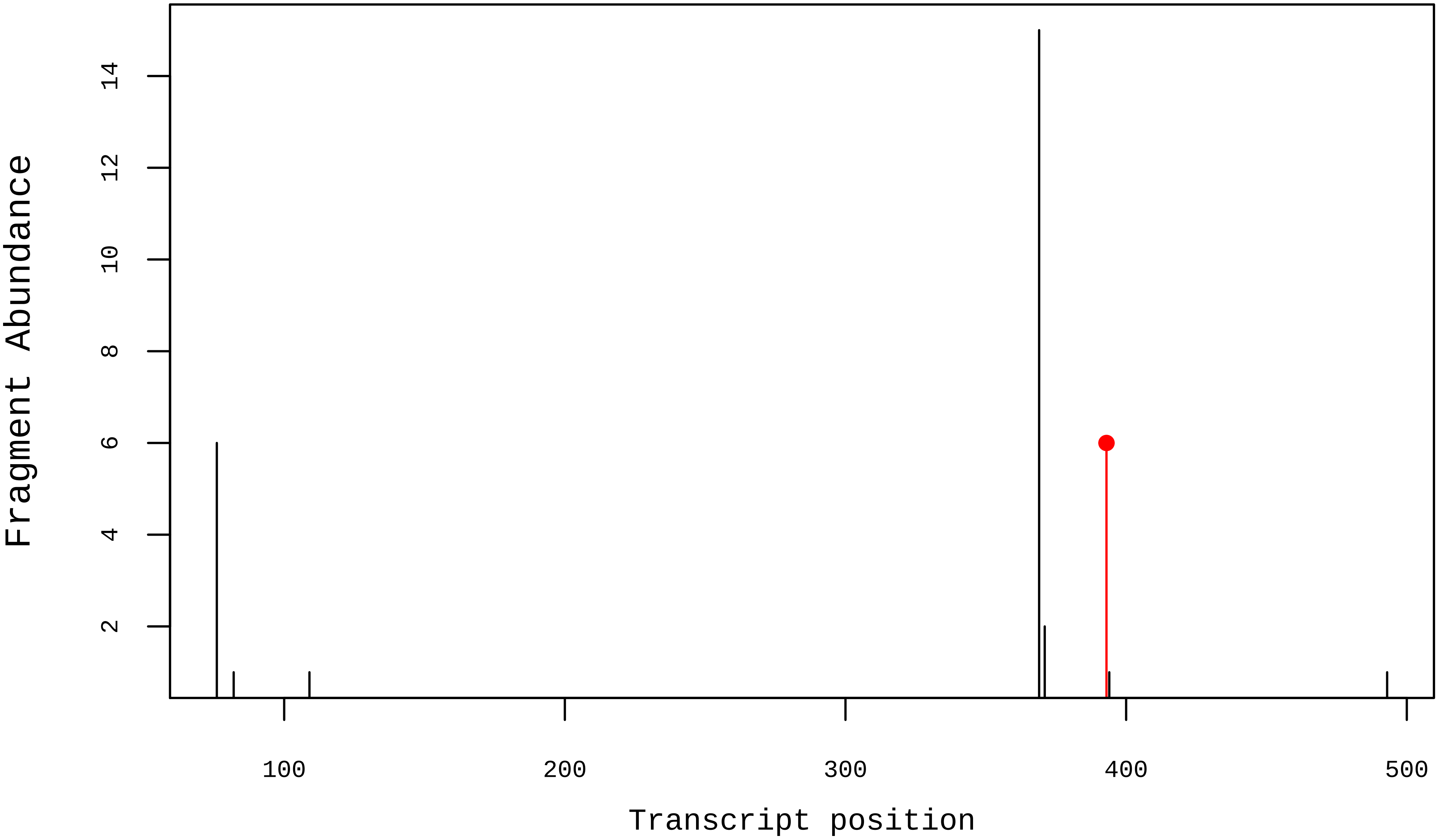

Cleavage site: 393   Tag abundance: 6   Weighted abundance: 0.75   Category: 3
sRNA abundance: 1   Alignment score: 0   MFE ratio: 1   p-value: 0.041

HORVU5Hr1G015600|HORVU5Hr1G015600.17||168|589

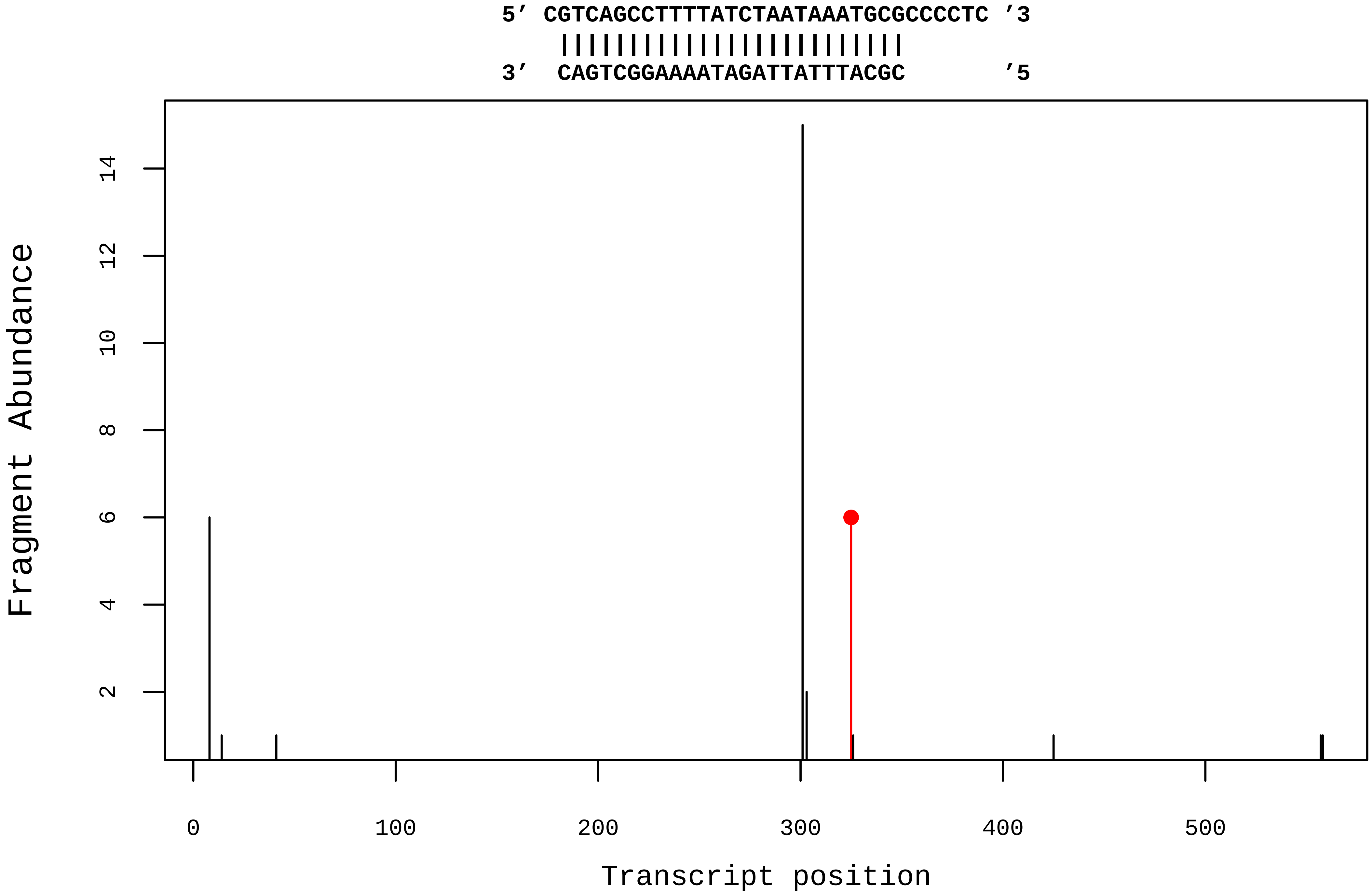

Cleavage site: 325 Tag abundance: 6 Weighted abundance: 0.75 Category: 3
sRNA abundance: 1 Alignment score: 0 MFE ratio: 1 p−value: 0.028

HORVU5Hr1G015600|HORVU5Hr1G015600.2||231|617

```
5’ CGTCAGCCTTTTATCTAATAAATGCGCCCCTC ’3
    ||||||||||||||||||||||||
3’  CAGTCGGAAAATAGATTATTTACGC       ’5
```

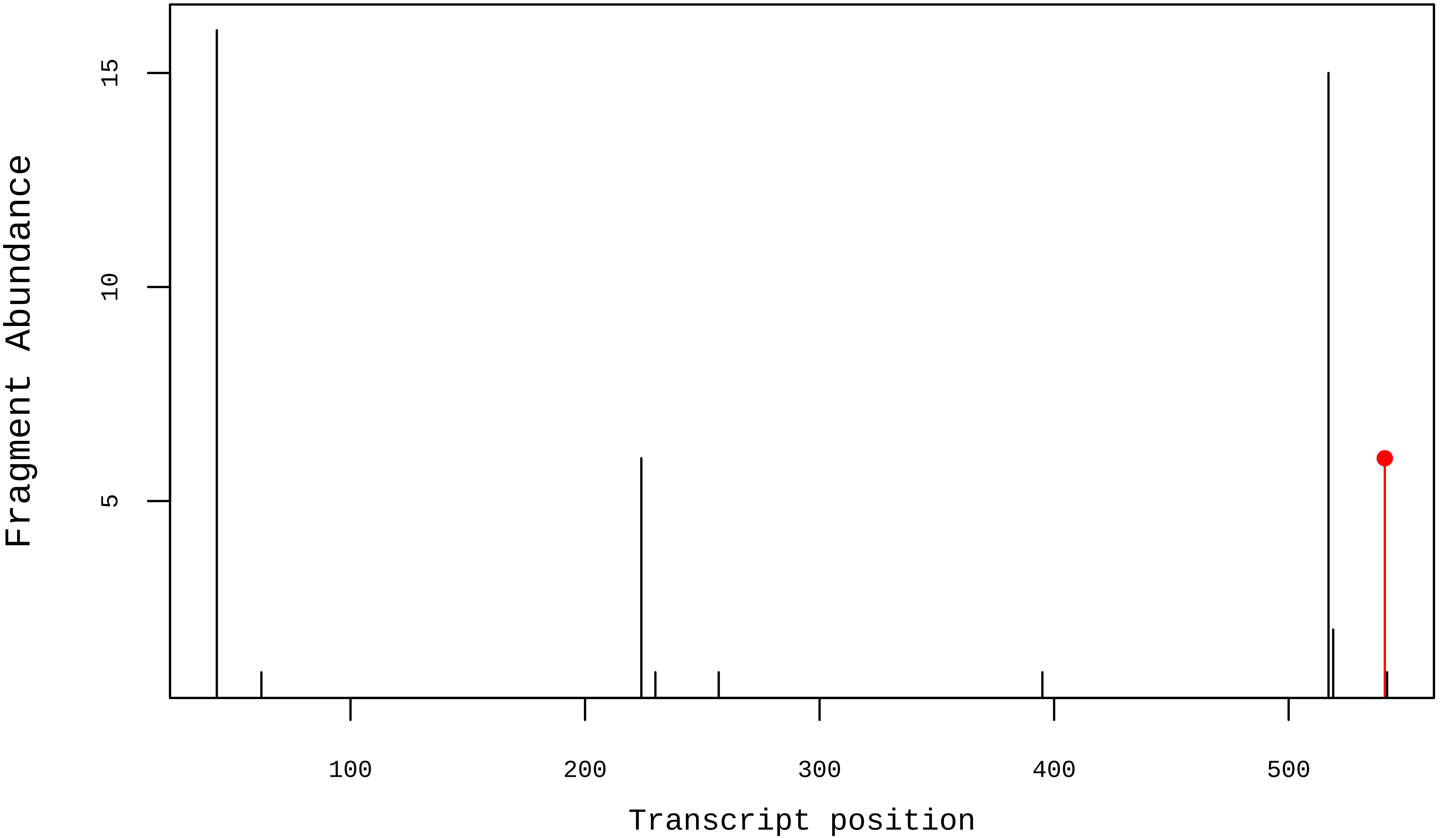

Cleavage site: 541   Tag abundance: 6   Weighted abundance: 0.75   Category: 3
sRNA abundance: 1   Alignment score: 0   MFE ratio: 1   p-value: 0.039

HORVU5Hr1G015600|HORVU5Hr1G015600.5||162|416

```
5' CGTCAGCCTTTTATCTAATAAATGCGCCCCTC '3
    ||||||||||||||||||||||||
3'  CAGTCGGAAAATAGATTATTTACGC       '5
```

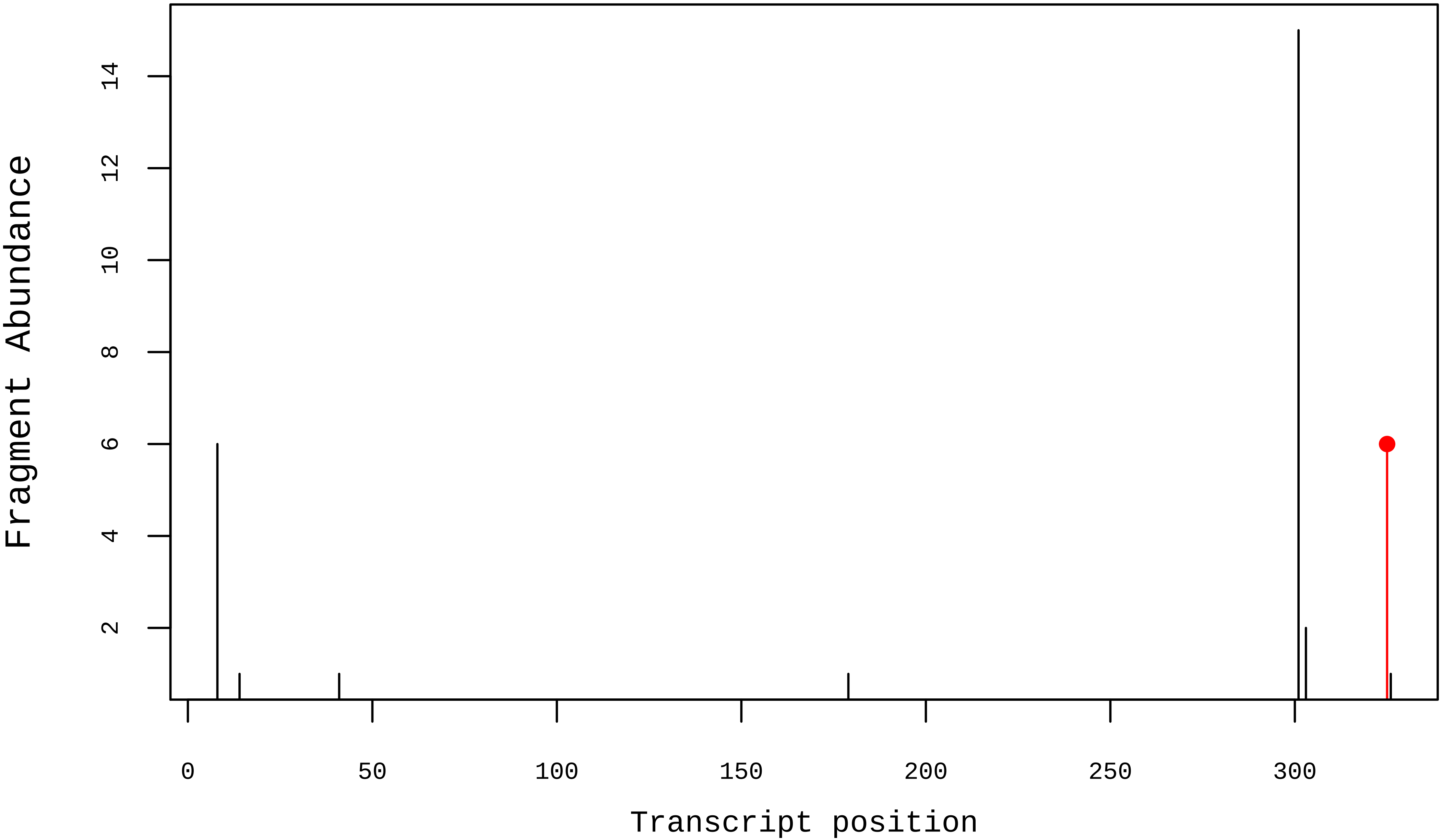

Cleavage site: 325 Tag abundance: 6 Weighted abundance: 0.75 Category: 3
sRNA abundance: 1 Alignment score: 0 MFE ratio: 1 p-value: 0.04

HORVU5Hr1G015600|HORVU5Hr1G015600.6||168|658

```
5' CGTCAGCCTTTTATCTAATAAATGCGCCCCTC '3
    ||||||||||||||||||||||||
3'  CAGTCGGAAAATAGATTATTTACGC       '5
```

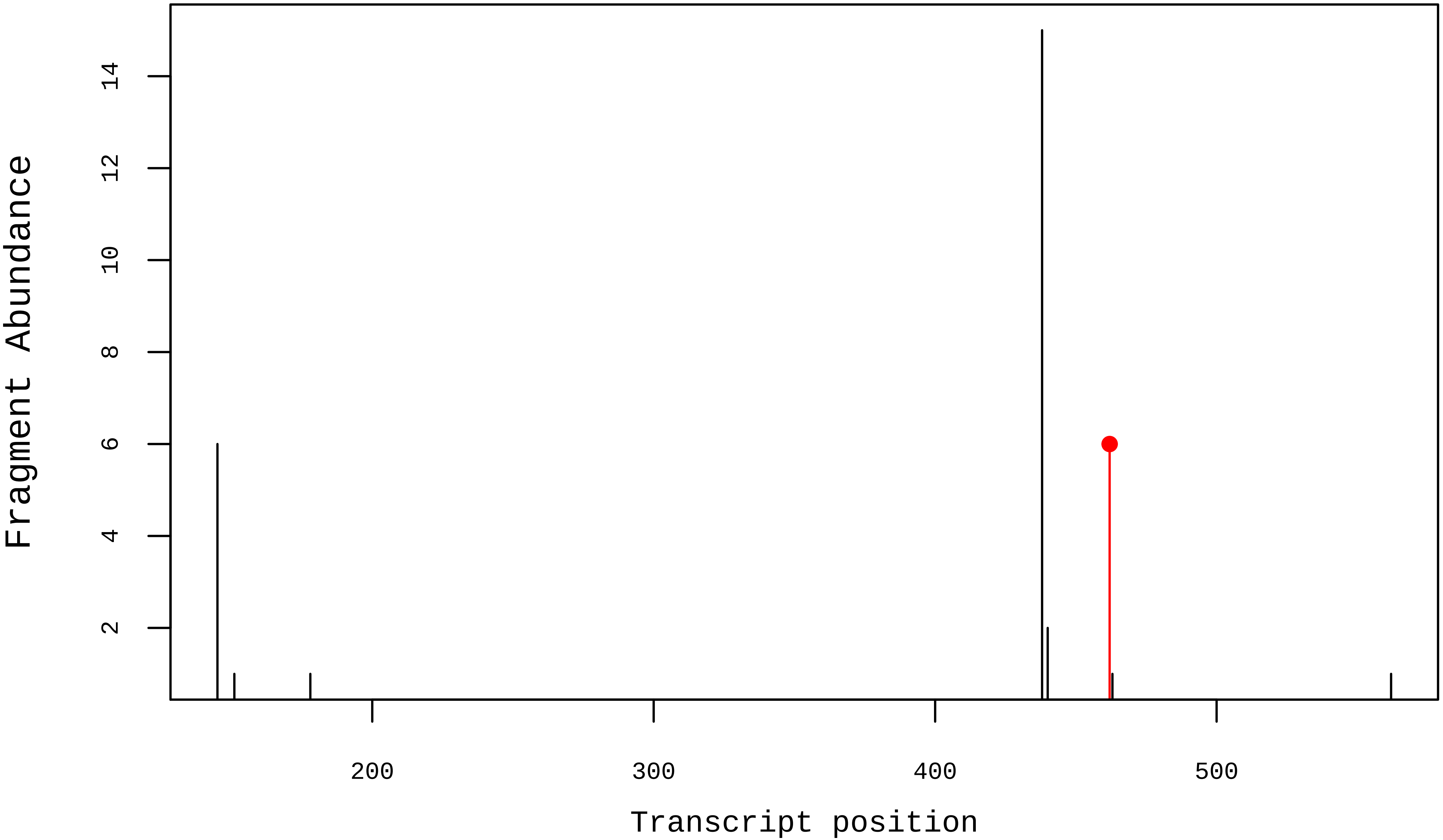

Cleavage site: 462   Tag abundance: 6   Weighted abundance: 0.75   Category: 3
sRNA abundance: 1   Alignment score: 0   MFE ratio: 1   p-value: 0.037

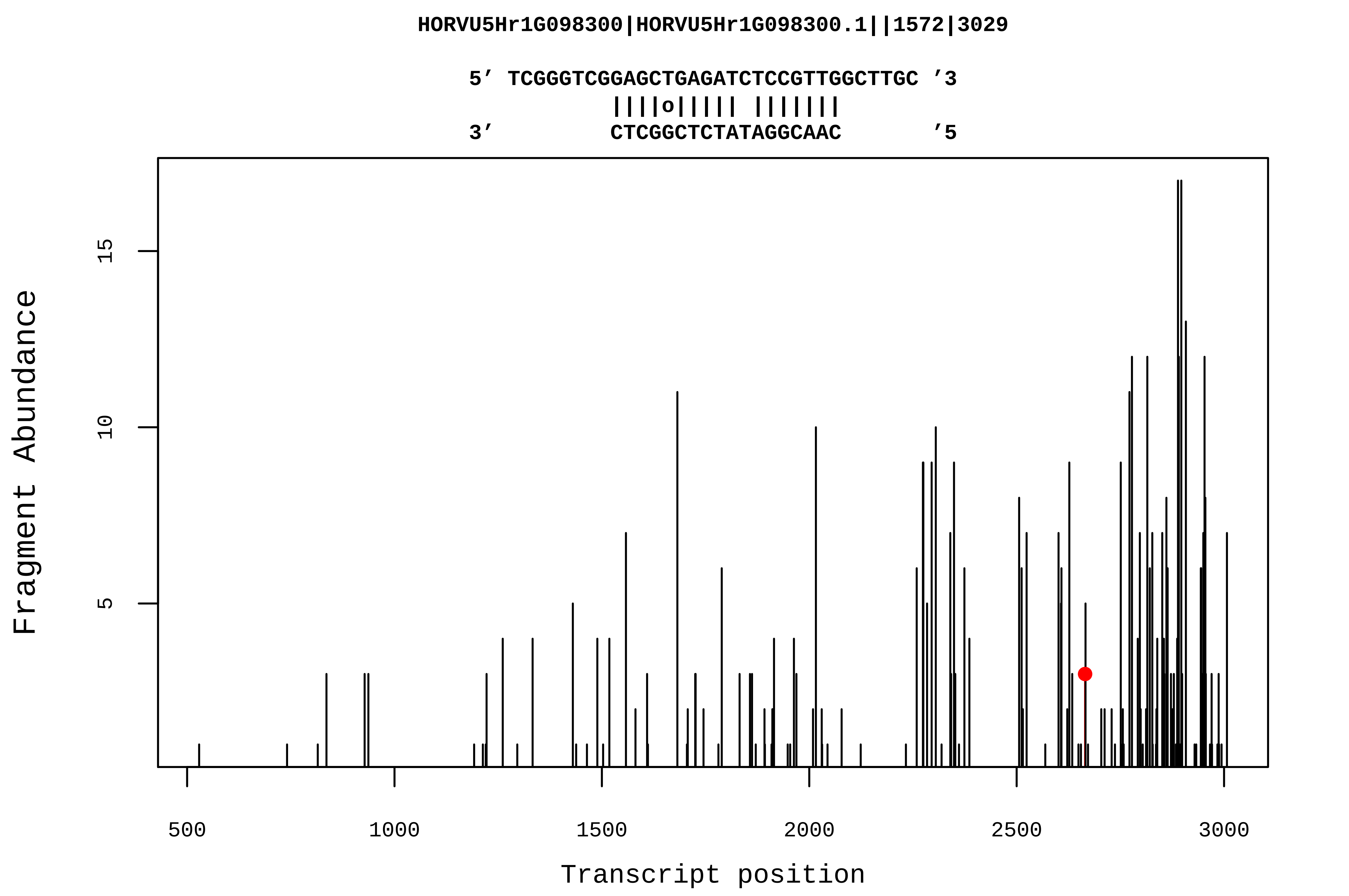

Cleavage site: 2665 Tag abundance: 3 Weighted abundance: 3 Category: 3
sRNA abundance: 1 Alignment score: 2.5 MFE ratio: 0.804 p-value: 0.02

HORVU4Hr1G053660|HORVU4Hr1G053660.1||921|1200

```
5' CATTTGTGGTAACATATTTGCACGTGAGTCGA '3
          ||||  ||||o||||||o||
3'        ACCAA-GTATGAACGTGTAC       '5
```

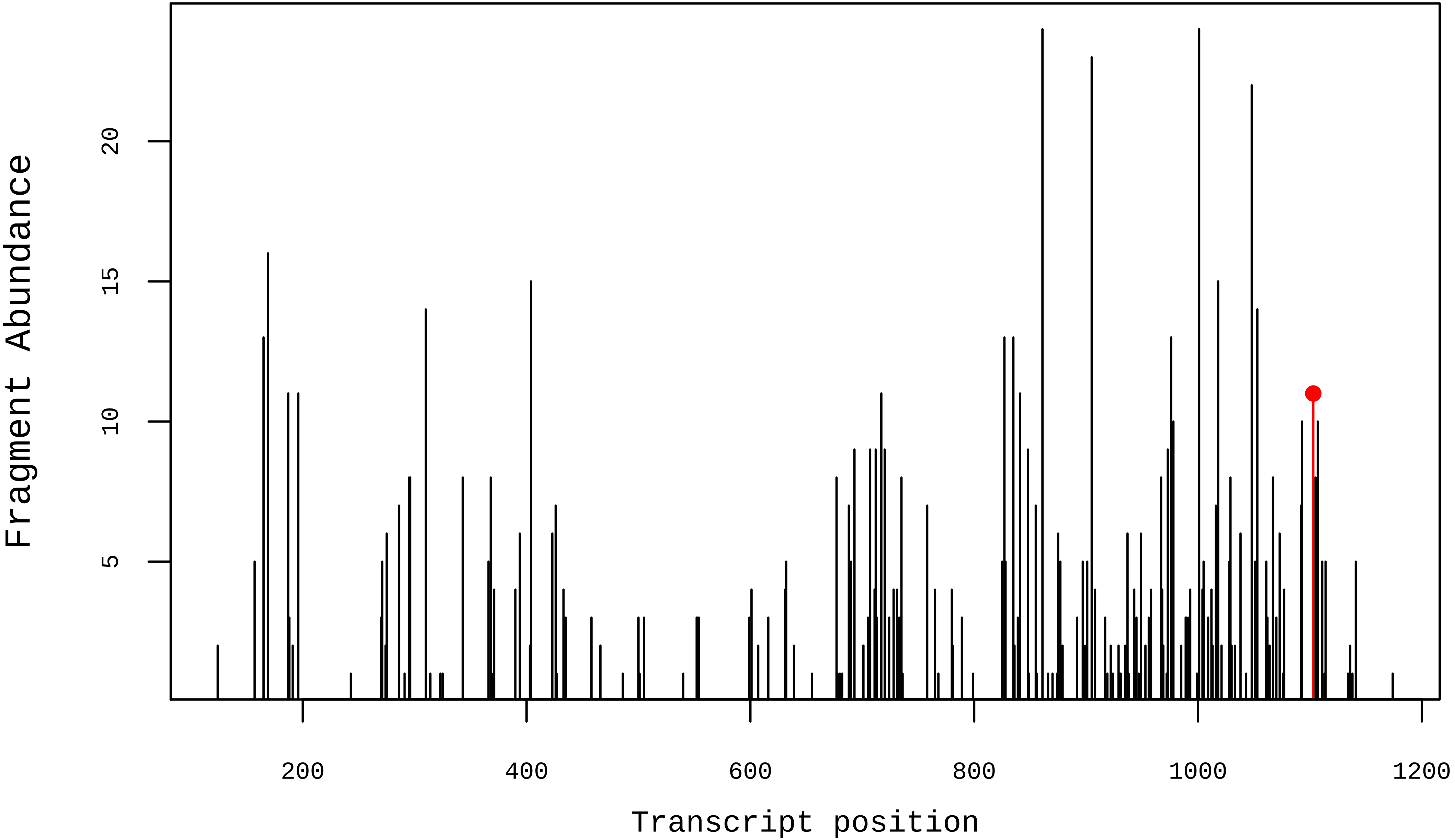

Cleavage site: 1103   Tag abundance: 11   Weighted abundance: 11   Category: 2
sRNA abundance: 1   Alignment score: 4   MFE ratio: 0.74   p-value: 0.042

HORVU1Hr1G012710|HORVU1Hr1G012710.2||324|836

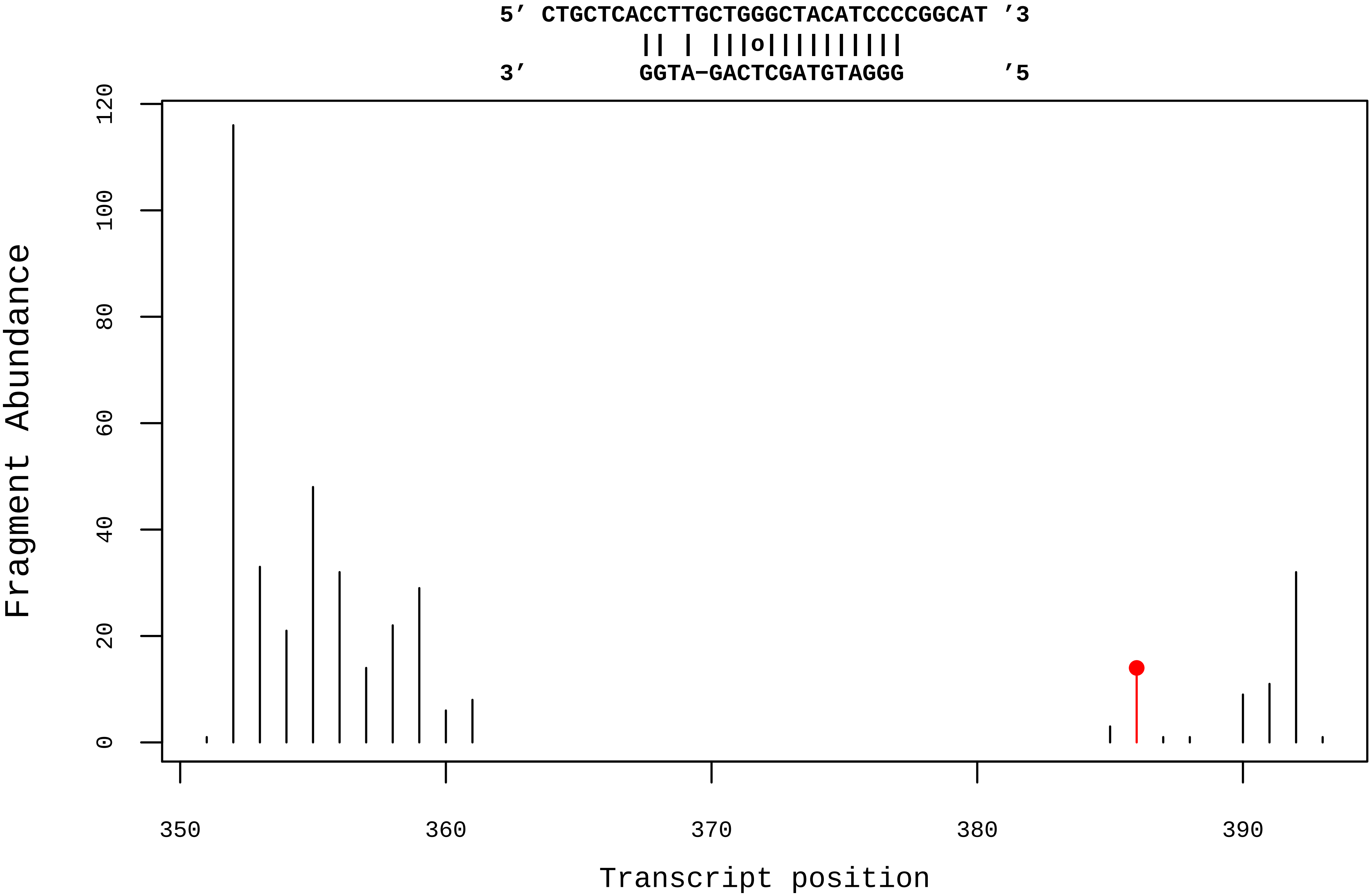

Cleavage site: 386 Tag abundance: 14 Weighted abundance: 4.667 Category: 3
sRNA abundance: 1 Alignment score: 3 MFE ratio: 0.772 p-value: 0.033

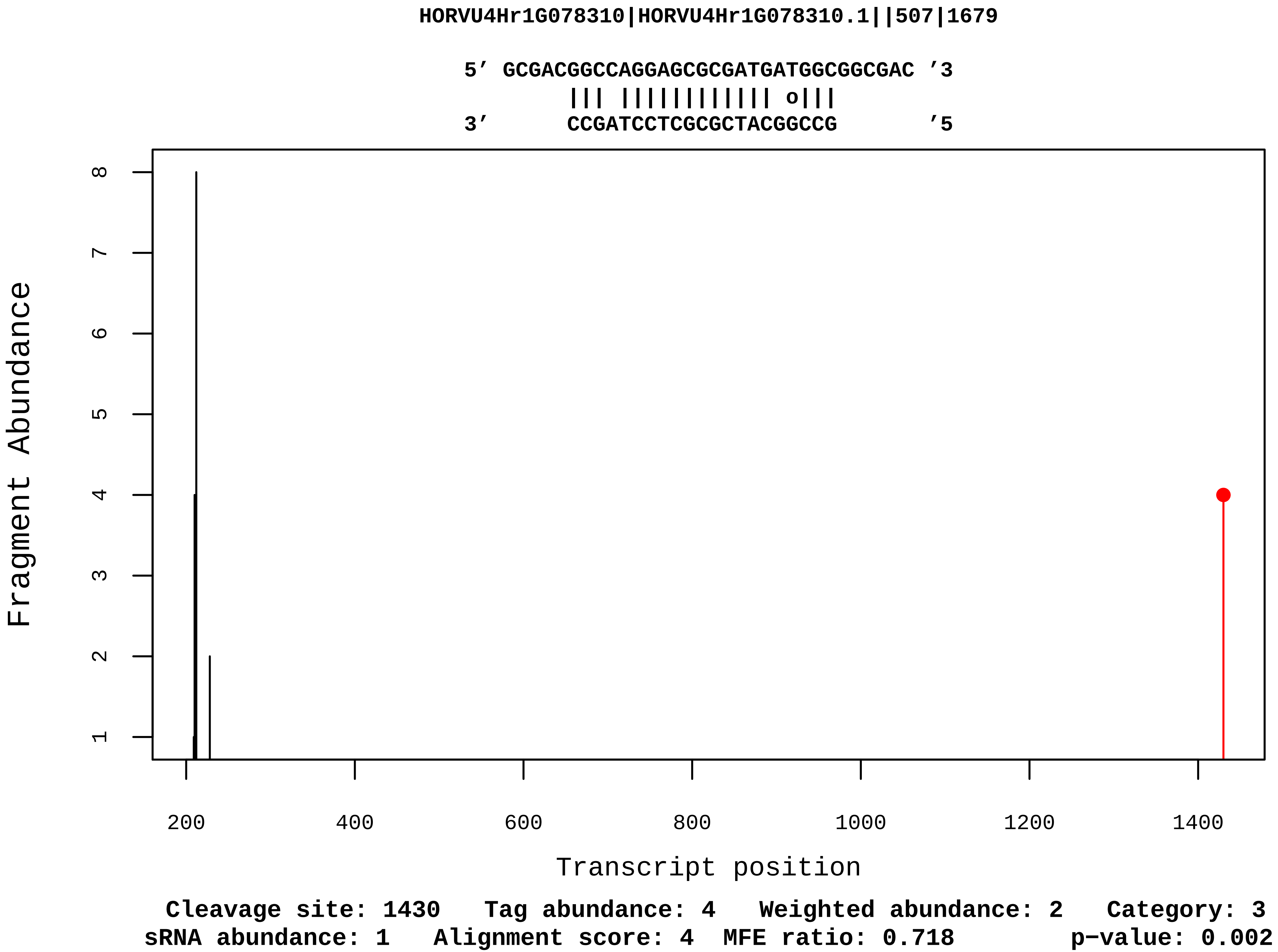

HORVU4Hr1G078310|HORVU4Hr1G078310.1||507|1679
5' GCGACGGCCAGGAGCGCGATGATGGCGGCGAC '3
||| ||||||||||||| o|||
3' CCGATCCTCGCGCTACGGCCG '5
Fragment Abundance
1
2
3
4
5
6
7
8
200
400
600
800
1000
1200
1400
Transcript position
Cleavage site: 1430 Tag abundance: 4 Weighted abundance: 2 Category: 3
sRNA abundance: 1 Alignment score: 4 MFE ratio: 0.718 p-value: 0.002

HORVU0Hr1G003660|HORVU0Hr1G003660.1||297|1955

```
5' GCCGGCCGAAGGGTCGAGTAGGTCGGTGCTCG '3
      |o|||||||||||||||||||||
3'    GTCGGCTTCCCAGCTCATCCAGCC       '5
```

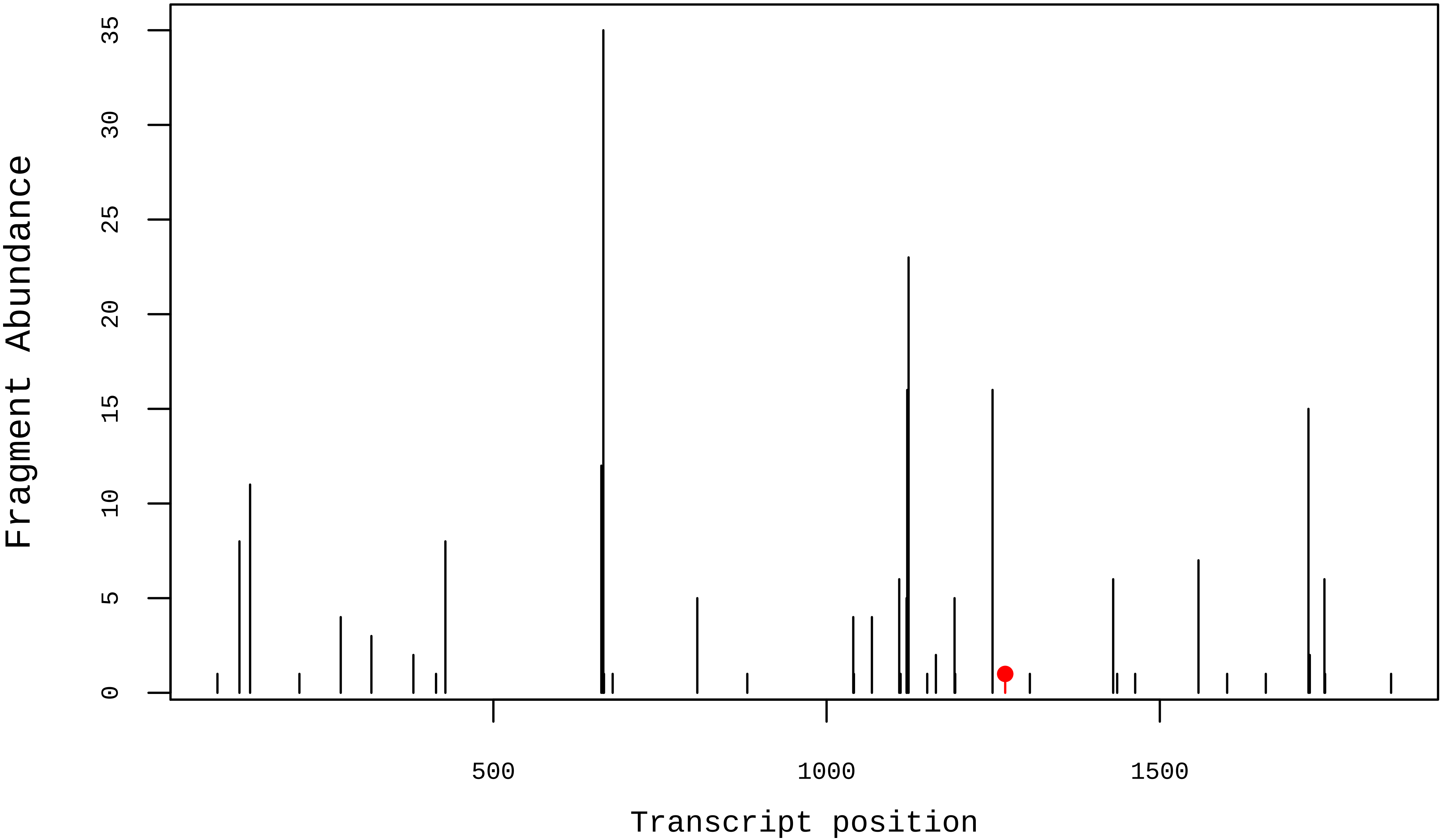

Cleavage site: 1268 Tag abundance: 1 Weighted abundance: 0.143 Category: 4
sRNA abundance: 1 Alignment score: 0.5 MFE ratio: 0.987 p-value: 0.046

HORVU0Hr1G023930|HORVU0Hr1G023930.3||396|2180

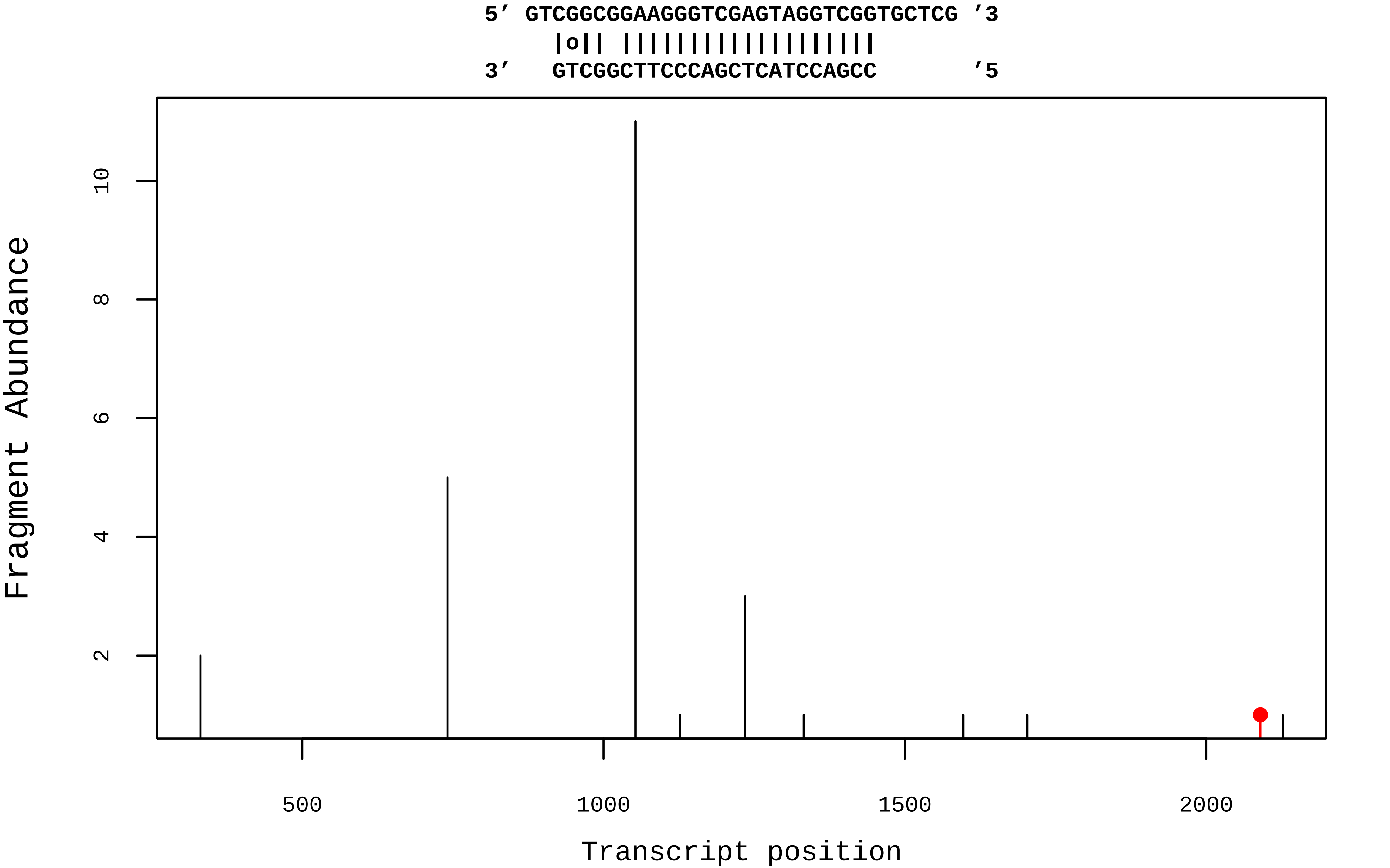

Cleavage site: 2090 Tag abundance: 1 Weighted abundance: 0.143 Category: 4
sRNA abundance: 1 Alignment score: 1.5 MFE ratio: 0.904 p-value: 0.019

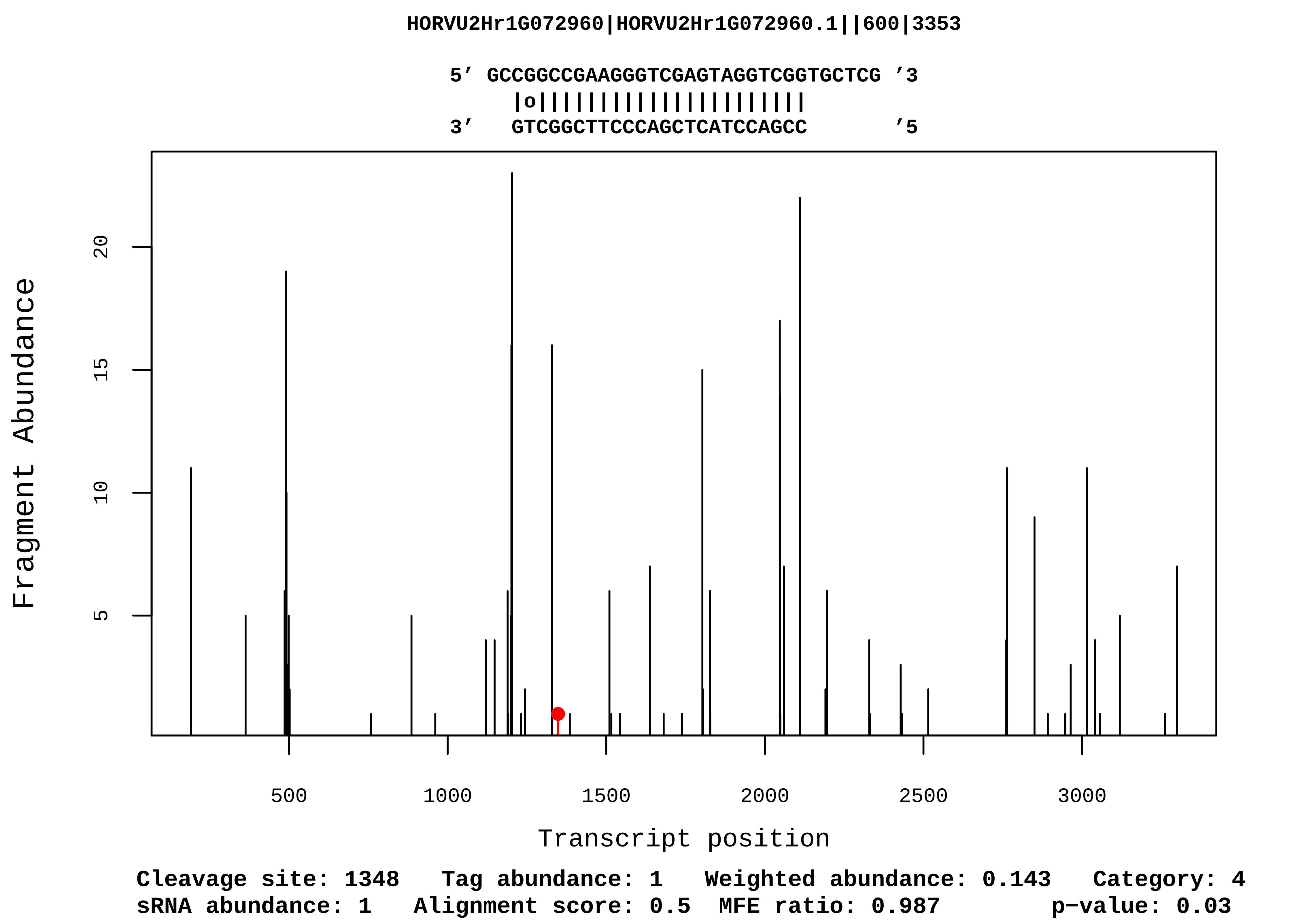
HORVU2Hr1G072960|HORVU2Hr1G072960.1||600|3353
5’ GCCGGCCGAAGGGTCGAGTAGGTCGGTGCTCG ’3
|o|||||||||||||||||||||
3’ GTCGGCTTCCCAGCTCATCCAGCC ’5
Fragment Abundance
5
10
15
20
500
1000
1500
2000
2500
3000
Transcript position
Cleavage site: 1348 Tag abundance: 1 Weighted abundance: 0.143 Category: 4
sRNA abundance: 1 Alignment score: 0.5 MFE ratio: 0.987 p-value: 0.03

HORVU5Hr1G015600|HORVU5Hr1G015600.1||156|510

```
5' GCCGGCCGAAGGGTCGAGTAGGTCGGTGCTCG '3
     |o|||||||||||||||||||||
3'   GTCGGCTTCCCAGCTCATCCAGCC       '5
```

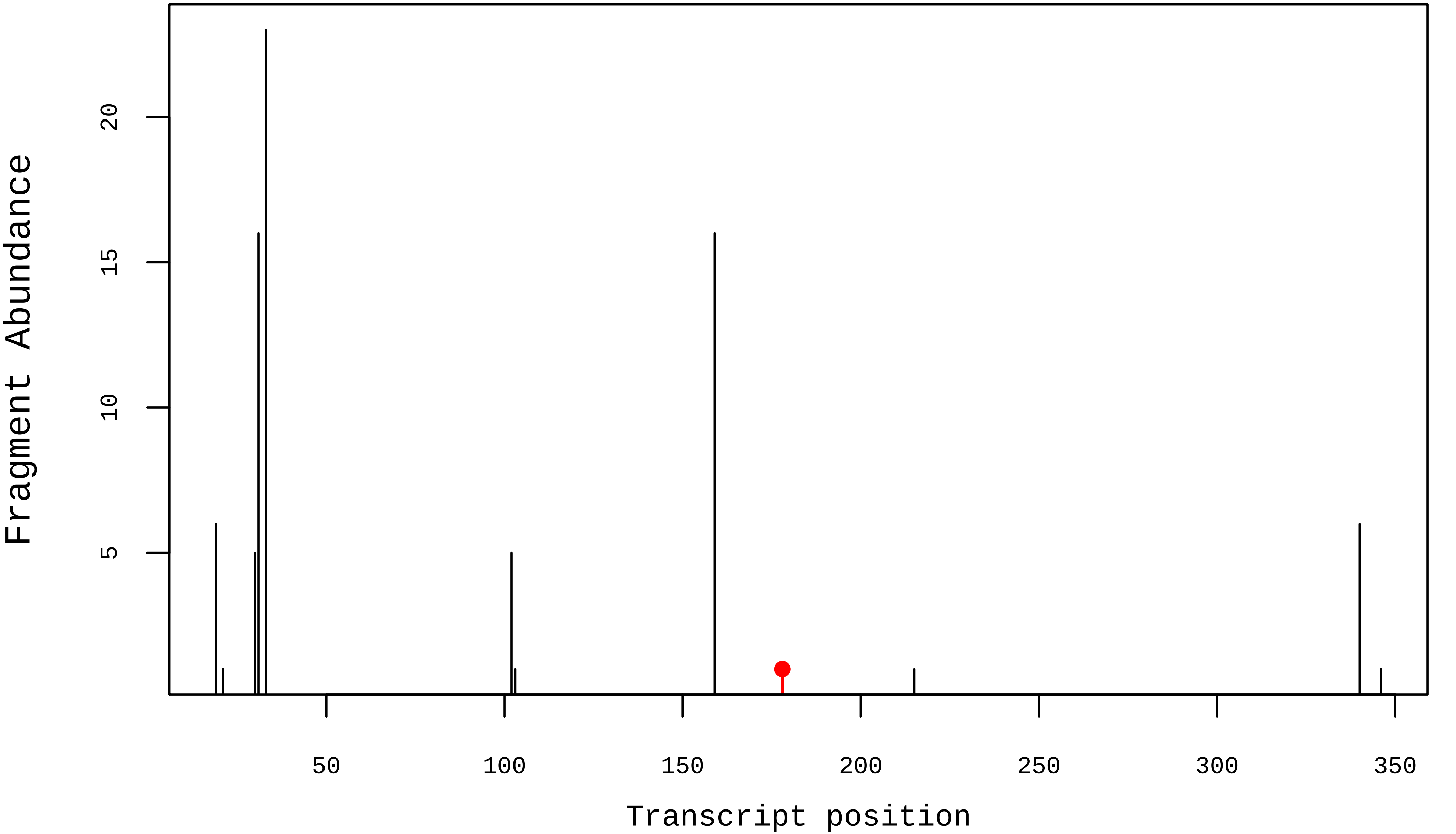

Cleavage site: 178 Tag abundance: 1 Weighted abundance: 0.143 Category: 4
sRNA abundance: 1 Alignment score: 0.5 MFE ratio: 0.987 p-value: 0.05

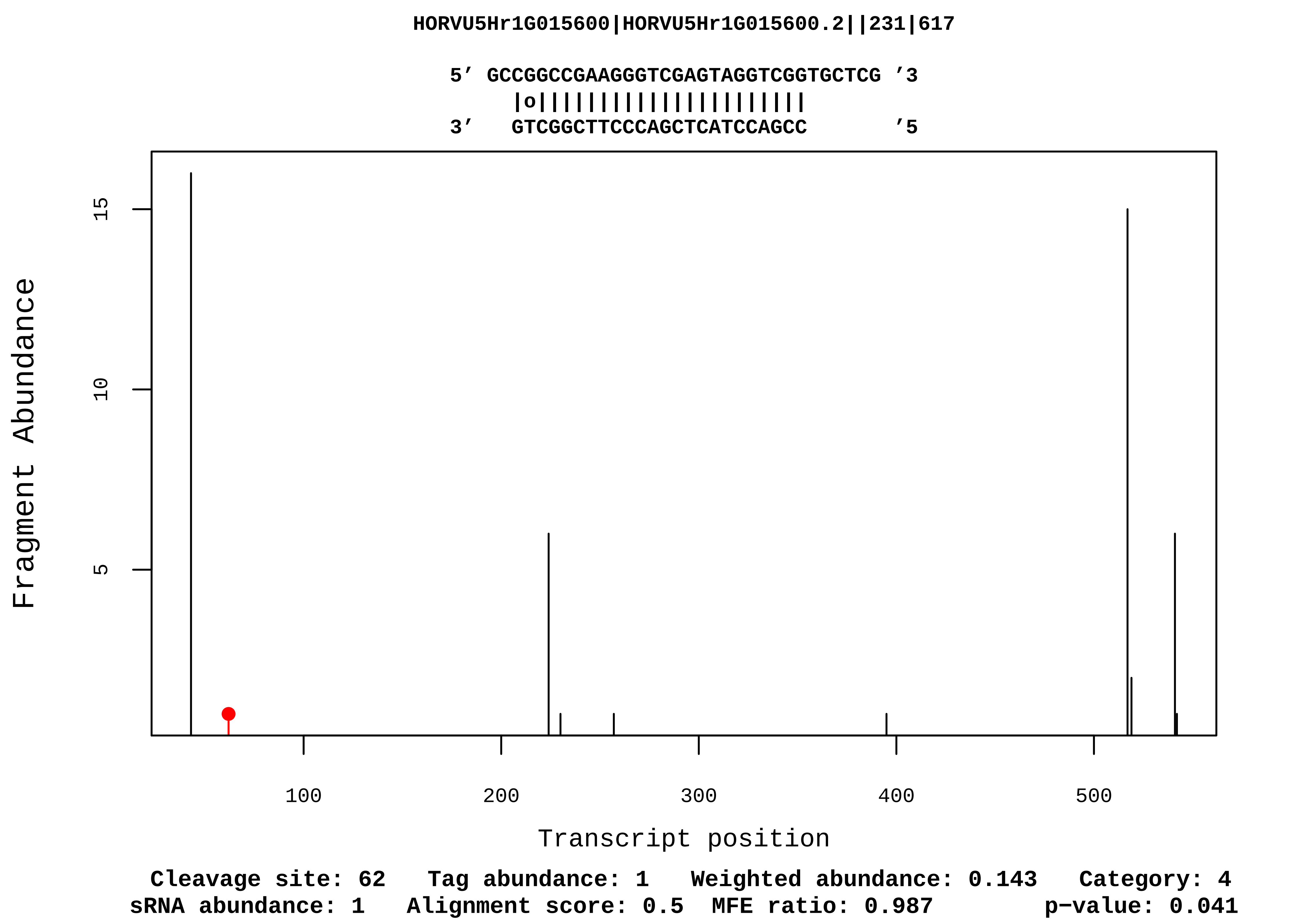

HORVU5Hr1G015600|HORVU5Hr1G015600.2||231|617
5′ GCCGGCCGAAGGGTCGAGTAGGTCGGTGCTCG ′3
|o|||||||||||||||||||||
3′ GTCGGCTTCCCAGCTCATCCAGCC ′5
Fragment Abundance
5
10
15
100
200
300
400
500
Transcript position
Cleavage site: 62 Tag abundance: 1 Weighted abundance: 0.143 Category: 4
sRNA abundance: 1 Alignment score: 0.5 MFE ratio: 0.987 p−value: 0.041

HORVU5Hr1G015600|HORVU5Hr1G015600.3||276|1709

```
5' GCCGGCCGAAGGGTCGAGTAGGTCGGTGCTCG '3
      |o|||||||||||||||||||||
3'    GTCGGCTTCCCAGCTCATCCAGCC       '5
```

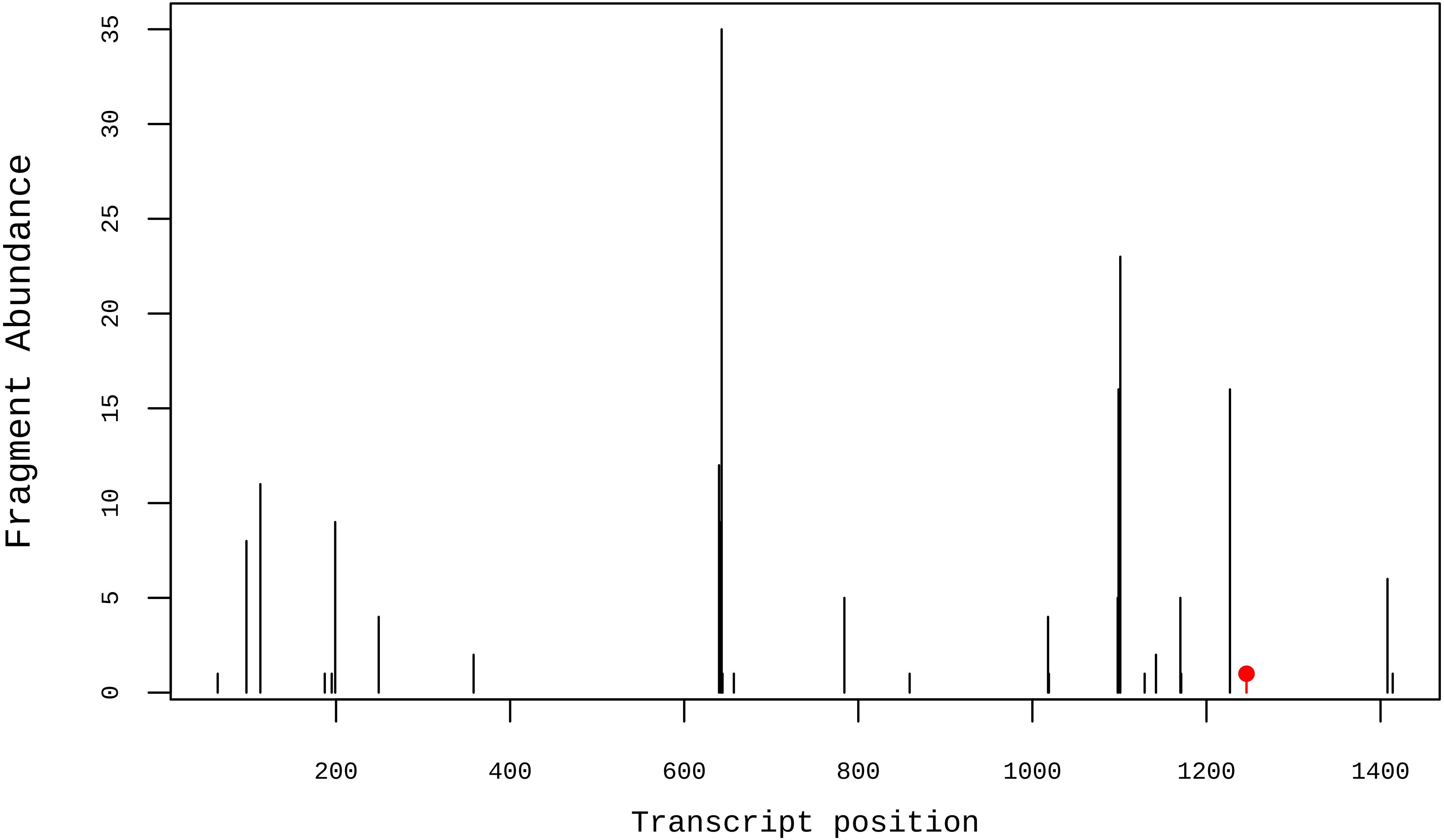

Cleavage site: 1246 Tag abundance: 1 Weighted abundance: 0.143 Category: 4
sRNA abundance: 1 Alignment score: 0.5 MFE ratio: 0.987 p-value: 0.032

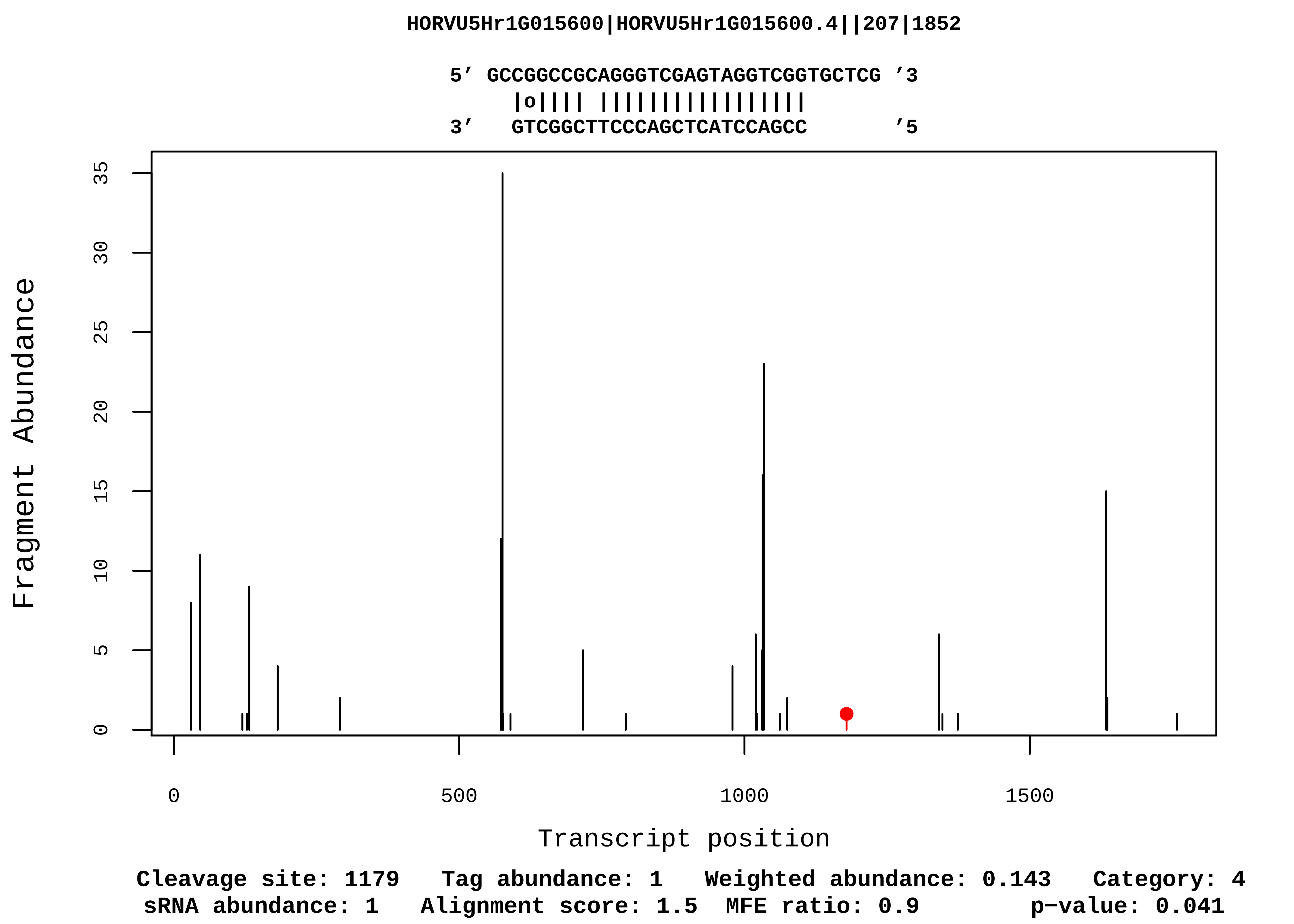
HORVU5Hr1G015600|HORVU5Hr1G015600.4||207|1852
5' GCCGGCCGCAGGGTCGAGTAGGTCGGTGCTCG '3
|o|||| ||||||||||||||||
3' GTCGGCTTCCCAGCTCATCCAGCC '5
Fragment Abundance
0
5
10
15
20
25
30
35
0
500
1000
1500
Transcript position
Cleavage site: 1179 Tag abundance: 1 Weighted abundance: 0.143 Category: 4
sRNA abundance: 1 Alignment score: 1.5 MFE ratio: 0.9 p-value: 0.041

HORVU7Hr1G048140|HORVU7Hr1G048140.2||3399|3592

```
5' TCGGCTGGCCAGCAGCGCT-CGCCAGCTCGGAG '3
          |||| |||||||| |o||||
3'        CCGGGCGTCGCGATGTGGTC         '5
```

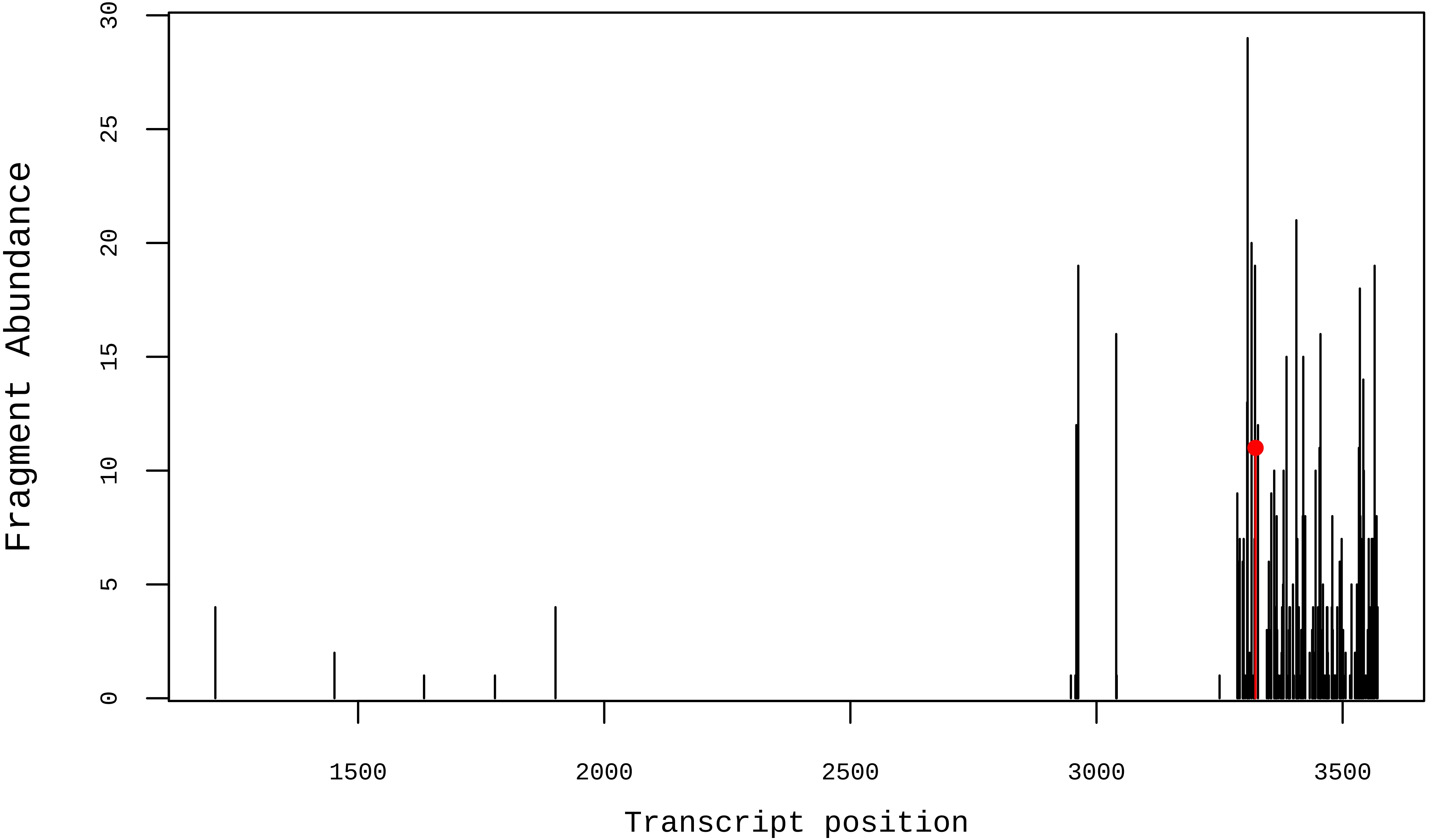

Cleavage site: 3323   Tag abundance: 11   Weighted abundance: 2.2   Category: 2
sRNA abundance: 1   Alignment score: 4 MFE ratio: 0.753   p-value: 0.04

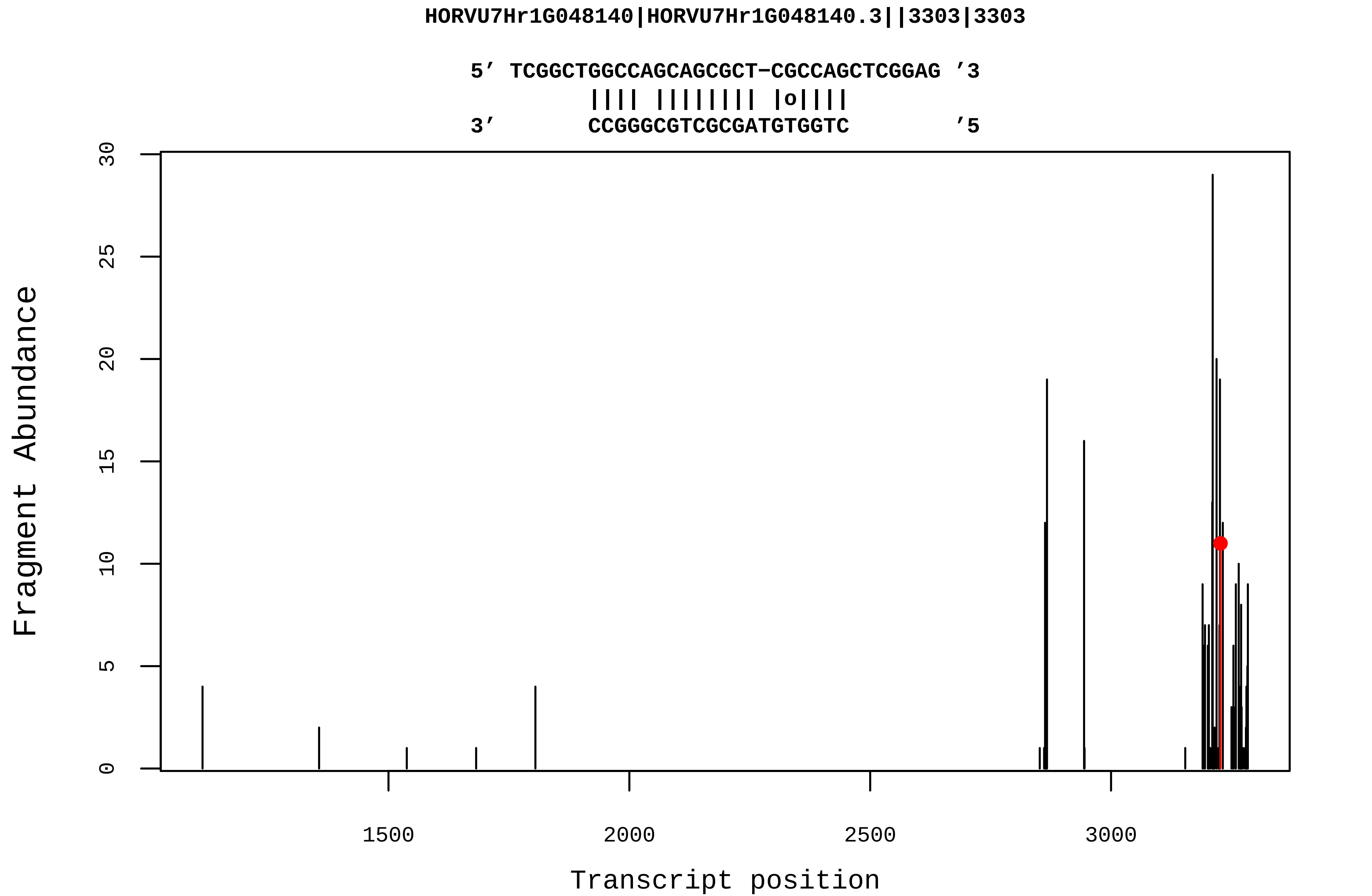

Cleavage site: 3227 Tag abundance: 11 Weighted abundance: 2.2 Category: 2
sRNA abundance: 1 Alignment score: 4 MFE ratio: 0.753 p-value: 0.018

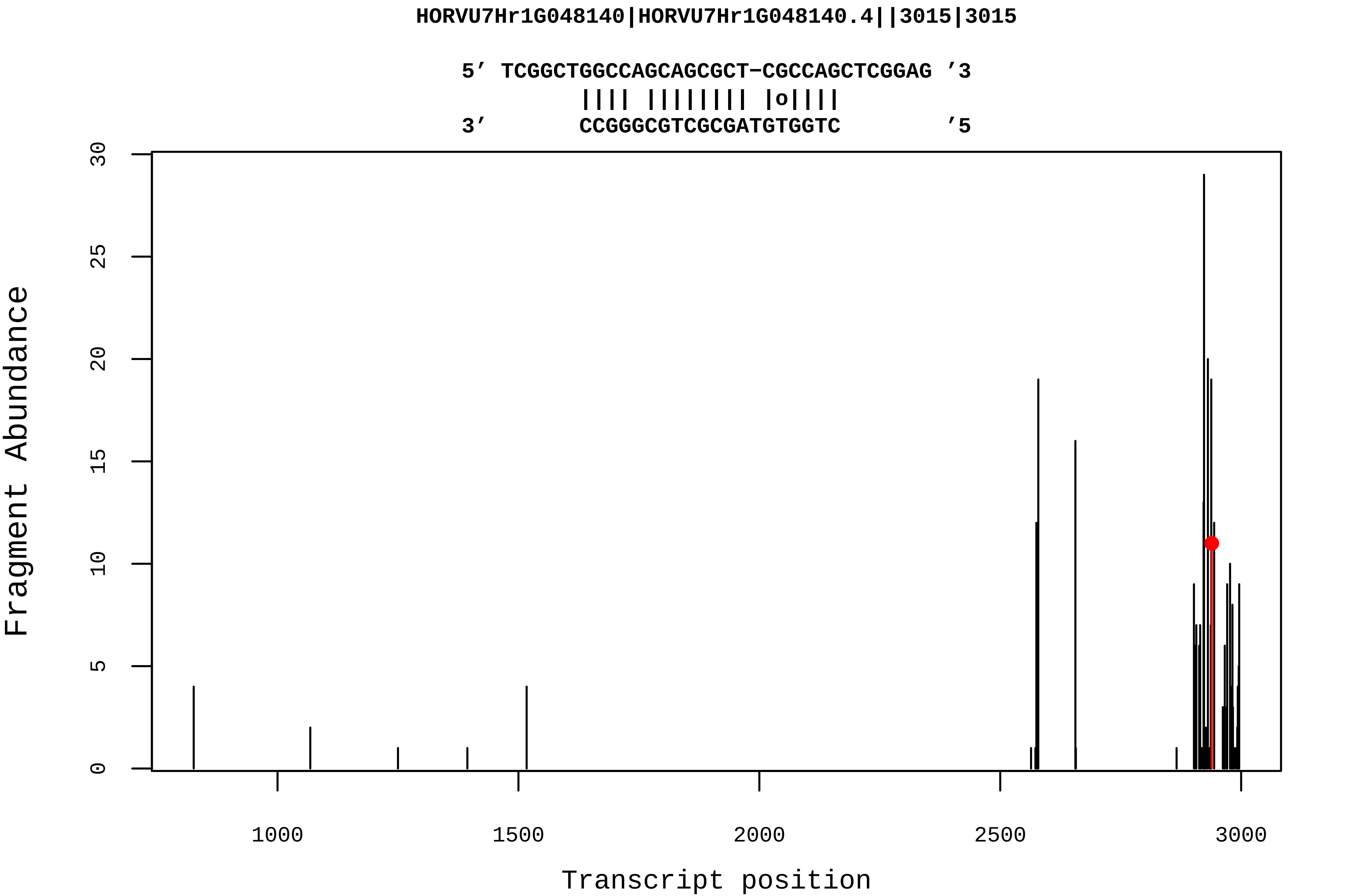

Cleavage site: 2939 Tag abundance: 11 Weighted abundance: 2.2 Category: 2
sRNA abundance: 1 Alignment score: 4 MFE ratio: 0.753 p-value: 0.02

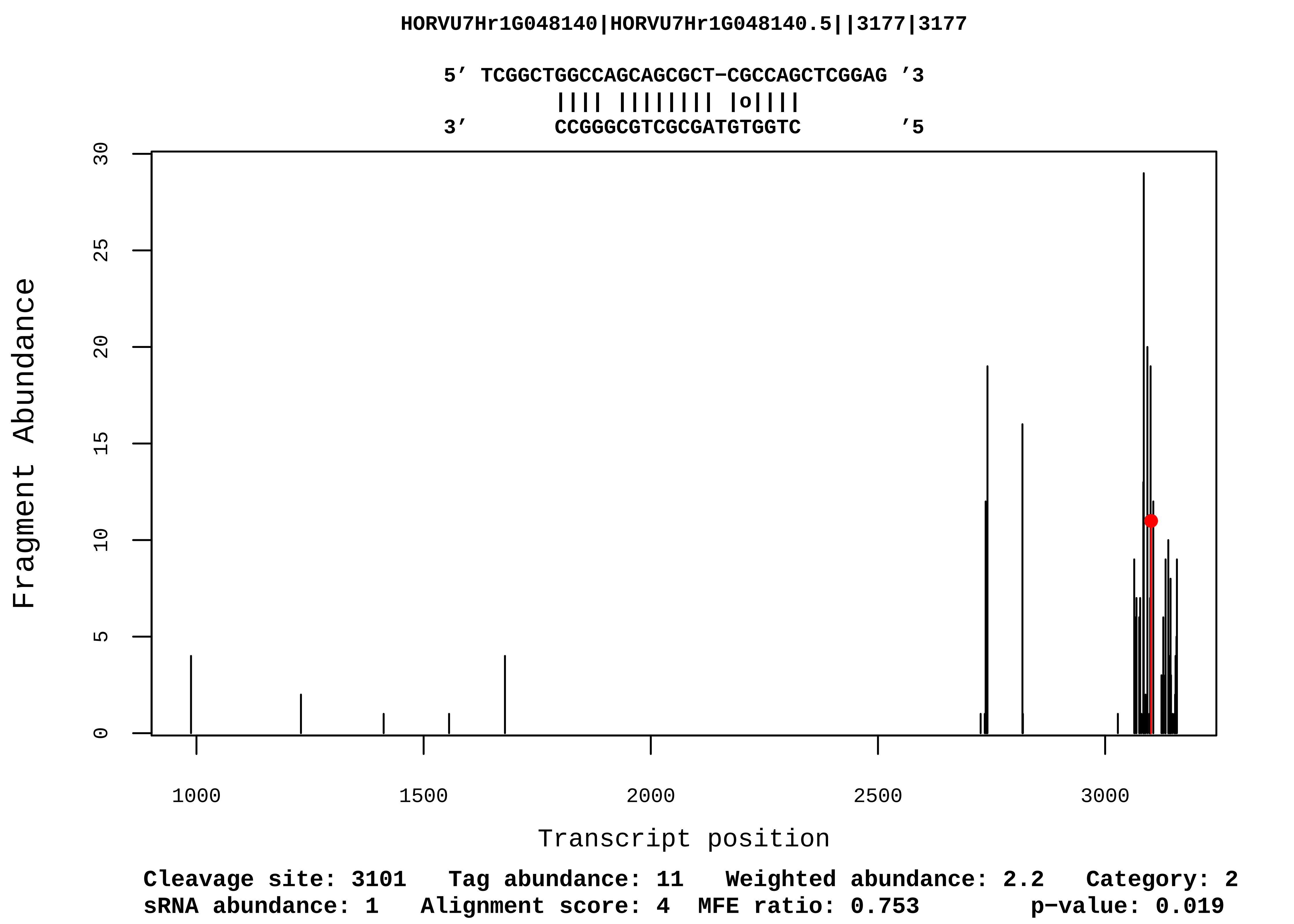
HORVU7Hr1G048140|HORVU7Hr1G048140.5||3177|3177
5' TCGGCTGGCCAGCAGCGCT-CGCCAGCTCGGAG '3
|||| |||||||| |o||||
3' CCGGGCGTCGCGATGTGGTC '5
Fragment Abundance
0
5
10
15
20
25
30
1000
1500
2000
2500
3000
Transcript position
Cleavage site: 3101 Tag abundance: 11 Weighted abundance: 2.2 Category: 2
sRNA abundance: 1 Alignment score: 4 MFE ratio: 0.753 p-value: 0.019

HORVU0Hr1G023930|HORVU0Hr1G023930.3||396|2180

```
5' GTCGGCGGAAGGGTCGAGTAGGTCGGTGCTCG '3
          |||||||||||||||| ||
3'        CTTCCCAGCTCATCCAACC        '5
```

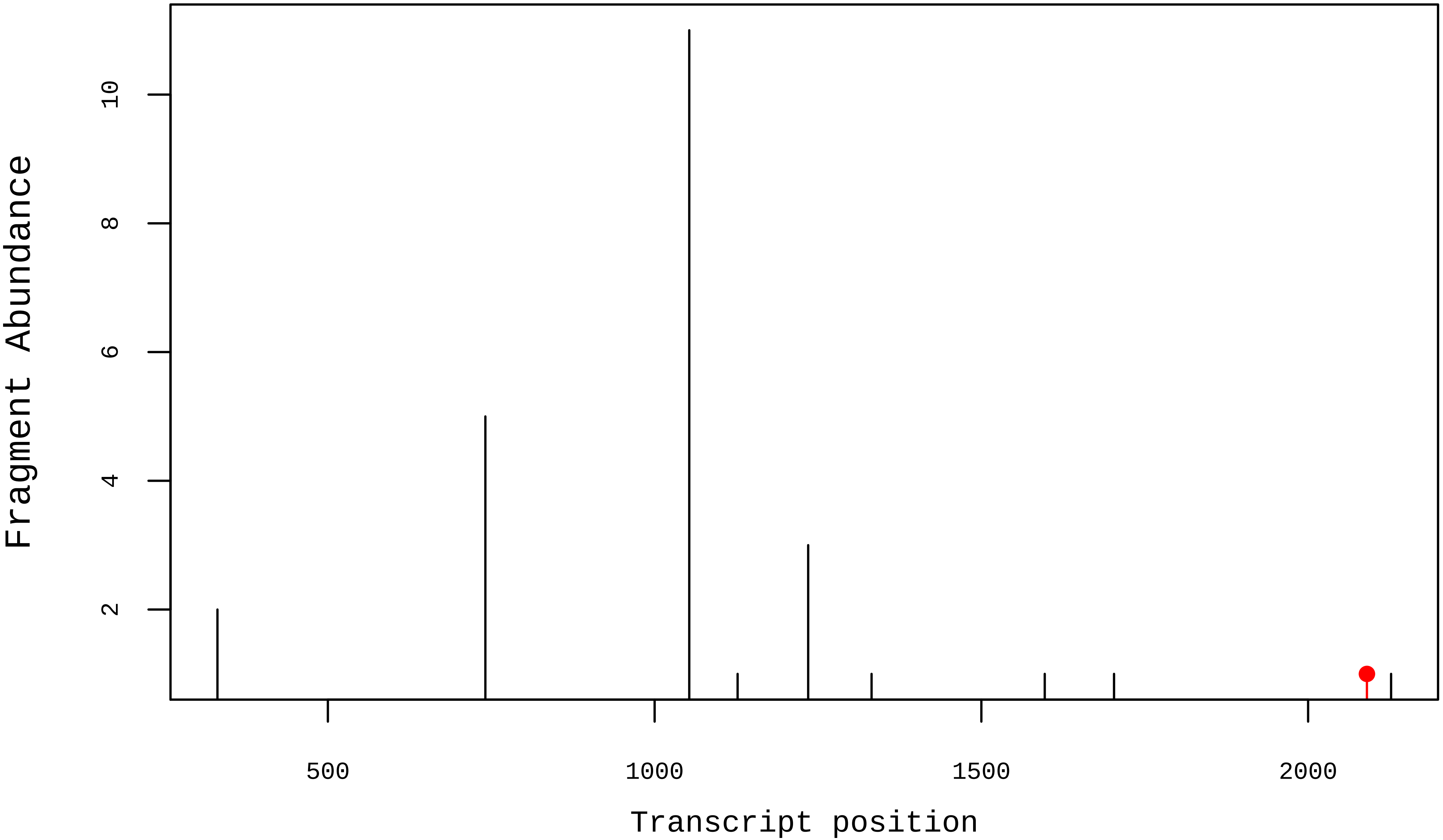

Cleavage site: 2090   Tag abundance: 1   Weighted abundance: 0.143   Category: 4
sRNA abundance: 1   Alignment score: 2   MFE ratio: 0.886   p-value: 0.017

HORVU2Hr1G072960|HORVU2Hr1G072960.1||600|3353

```
5' GCCGGCCGAAGGGTCGAGTAGGTCGGTGCTCG '3
             |||||||||||||||| ||
3'           CTTCCCAGCTCATCCAACC       '5
```

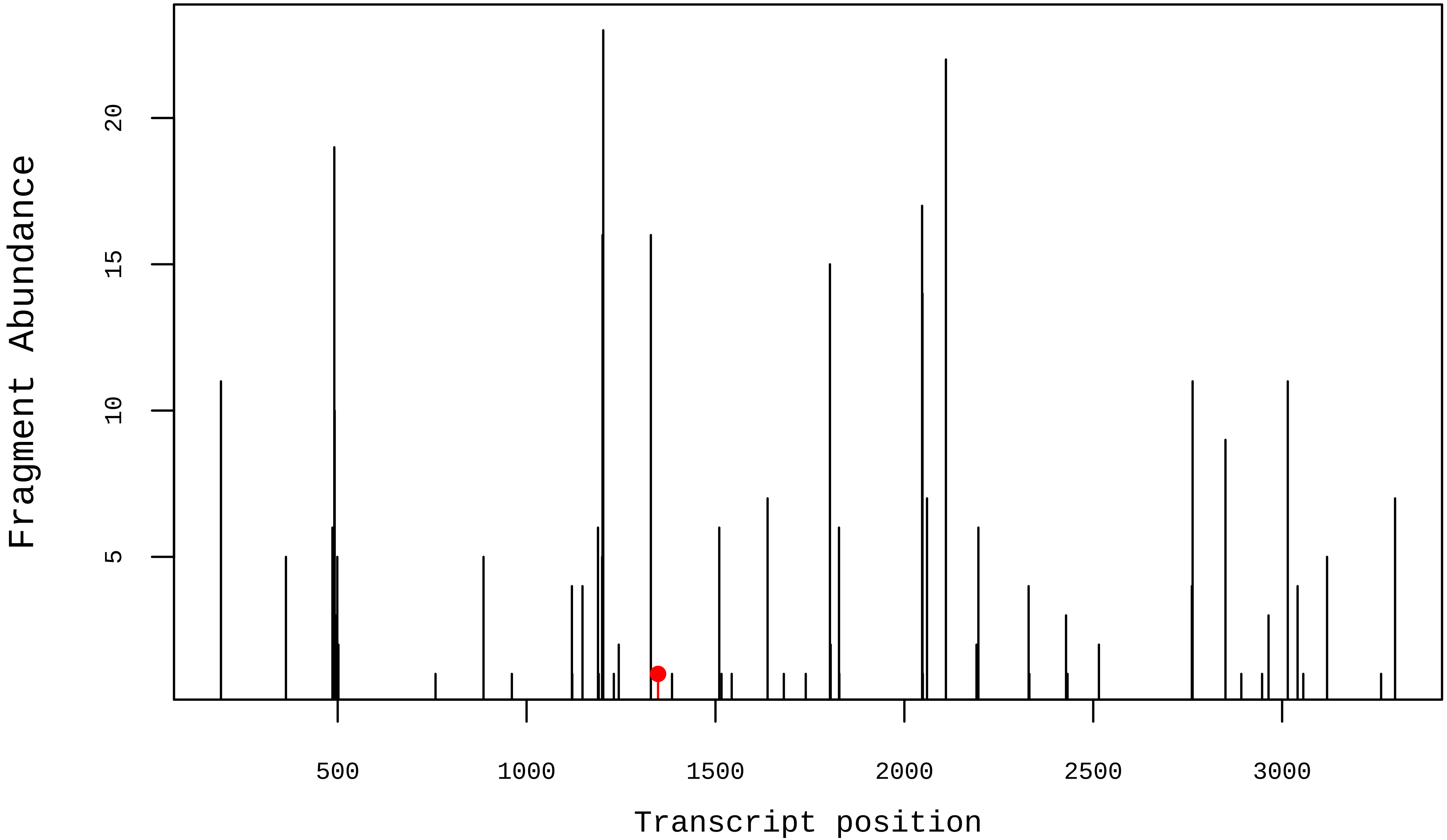

Cleavage site: 1348   Tag abundance: 1   Weighted abundance: 0.143   Category: 4
sRNA abundance: 1   Alignment score: 2   MFE ratio: 0.886   p-value: 0.035

HORVU5Hr1G015600|HORVU5Hr1G015600.2||231|617

```
5' GCCGGCCGAAGGGTCGAGTAGGTCGGTGCTCG '3
          |||||||||||||||| ||
3'        CTTCCCAGCTCATCCAACC        '5
```

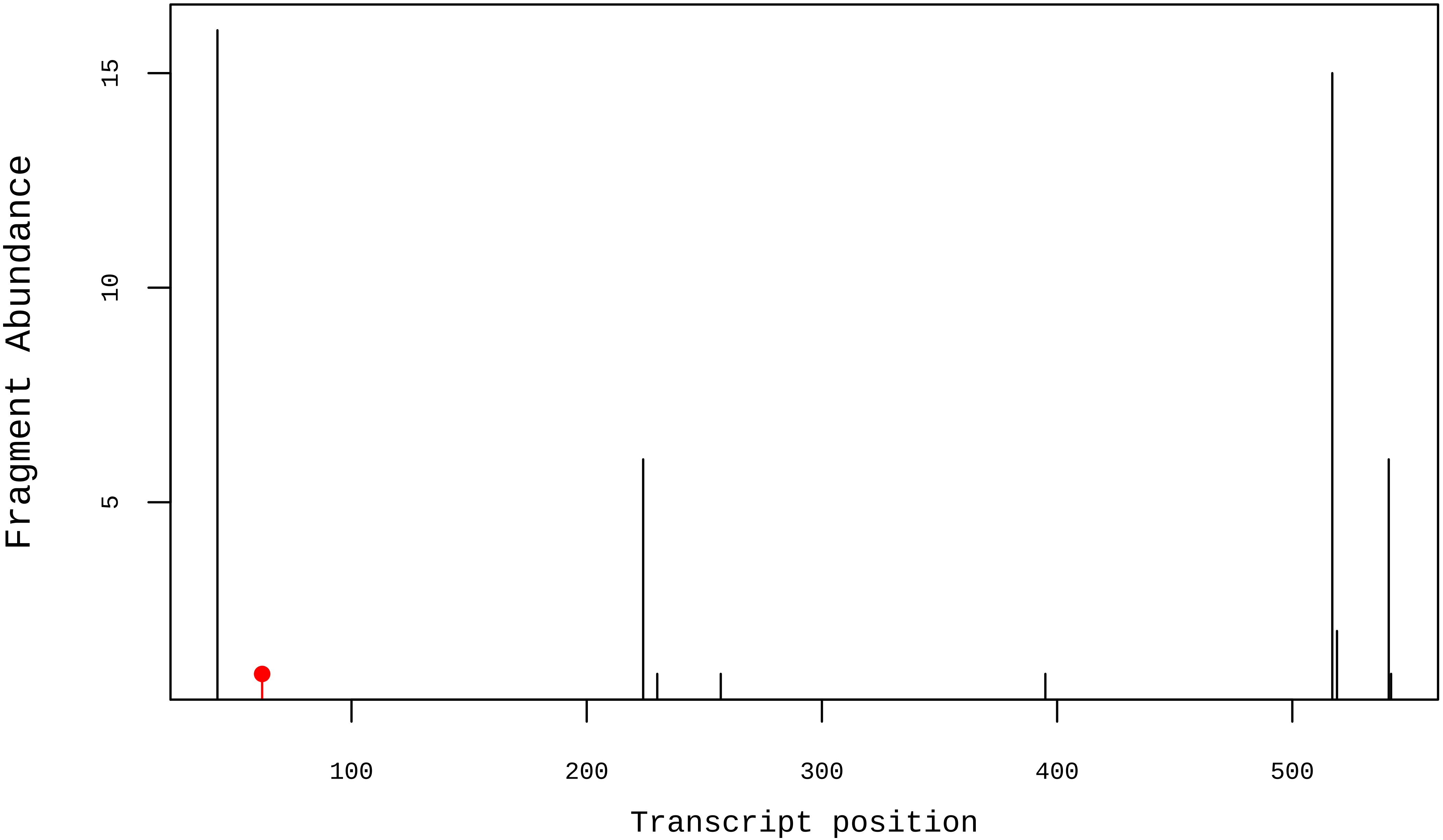

Cleavage site: 62   Tag abundance: 1   Weighted abundance: 0.143   Category: 4
sRNA abundance: 1   Alignment score: 2   MFE ratio: 0.886   p-value: 0.049

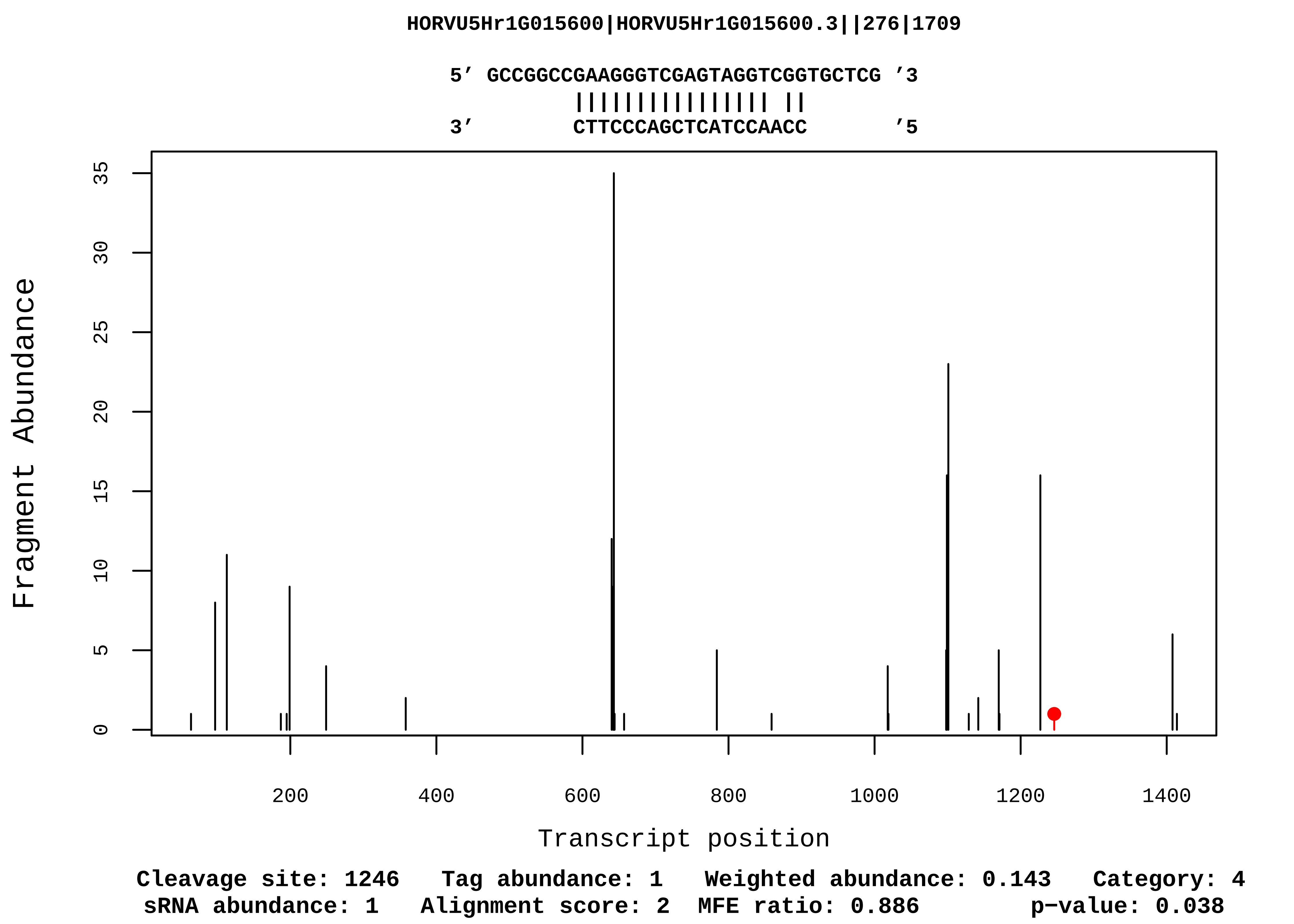
HORVU5Hr1G015600|HORVU5Hr1G015600.3||276|1709
5' GCCGGCCGAAGGGTCGAGTAGGTCGGTGCTCG '3
|||||||||||||||| ||
3' CTTCCCAGCTCATCCAACC '5
Fragment Abundance
0
5
10
15
20
25
30
35
200
400
600
800
1000
1200
1400
Transcript position
Cleavage site: 1246 Tag abundance: 1 Weighted abundance: 0.143 Category: 4
sRNA abundance: 1 Alignment score: 2 MFE ratio: 0.886 p-value: 0.038

HORVU5Hr1G015600|HORVU5Hr1G015600.4||207|1852

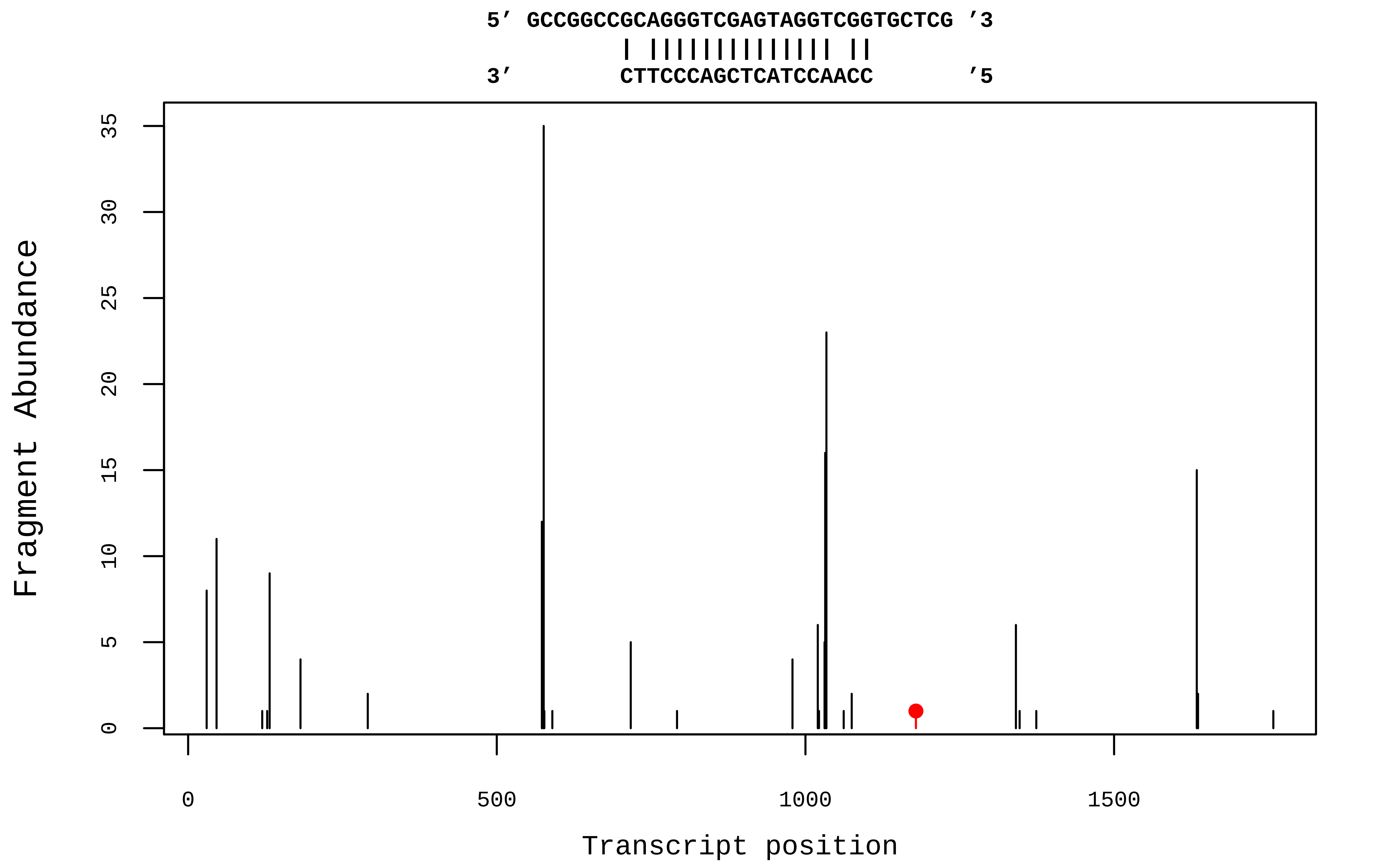

Cleavage site: 1179 Tag abundance: 1 Weighted abundance: 0.143 Category: 4
sRNA abundance: 1 Alignment score: 3 MFE ratio: 0.799 p-value: 0.041

HORVU7Hr1G116750|HORVU7Hr1G116750.1||870|1239

```
5' CATTCGATCGGGTCGAGTAGGTCG-GCAGCAAT '3
               ||||||||||||||||
3'            TCCCAGCTCATCCAGCTA        '5
```

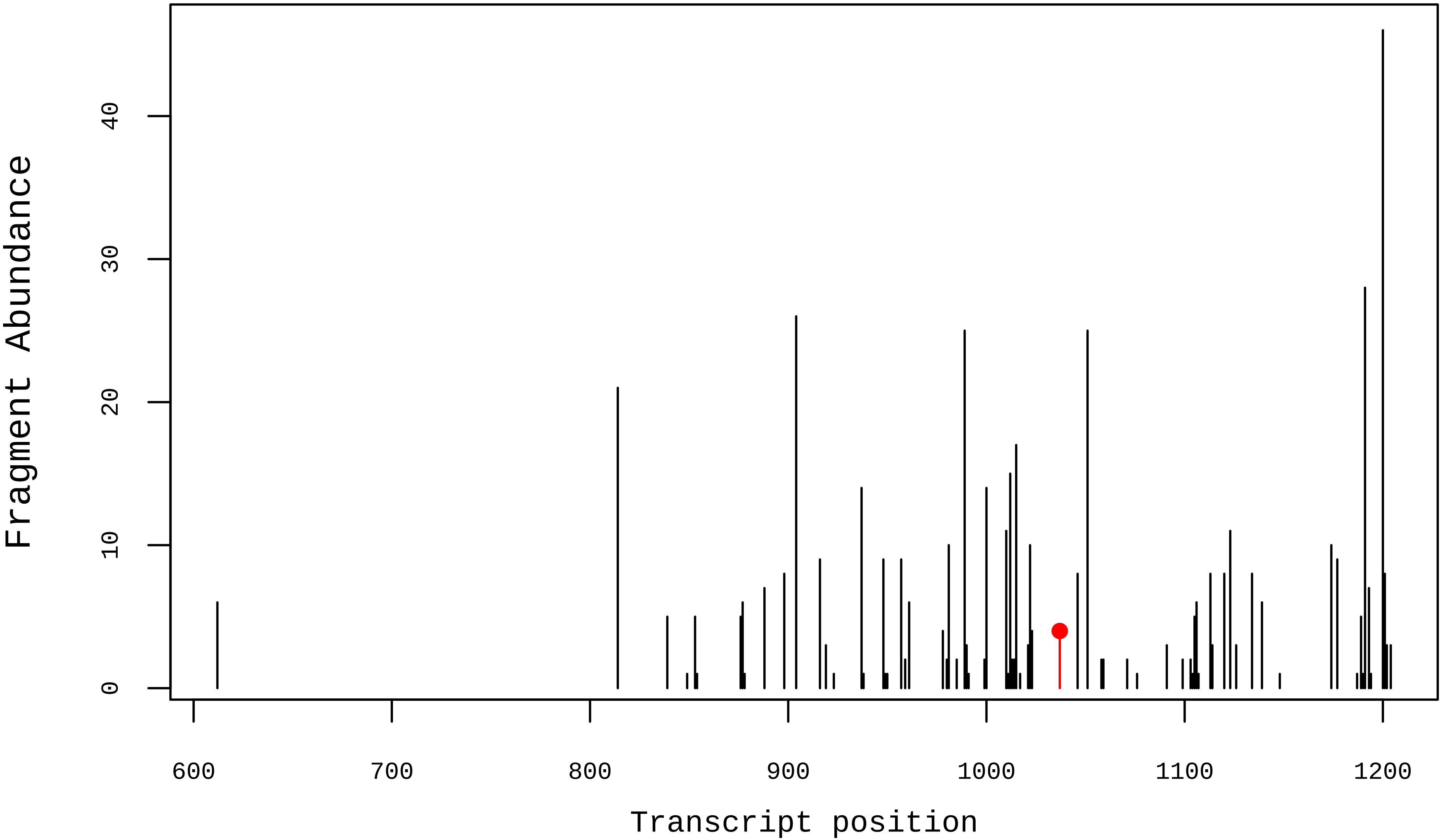

Cleavage site: 1037   Tag abundance: 4   Weighted abundance: 4   Category: 3
sRNA abundance: 1   Alignment score: 3   MFE ratio: 0.922   p-value: 0.033

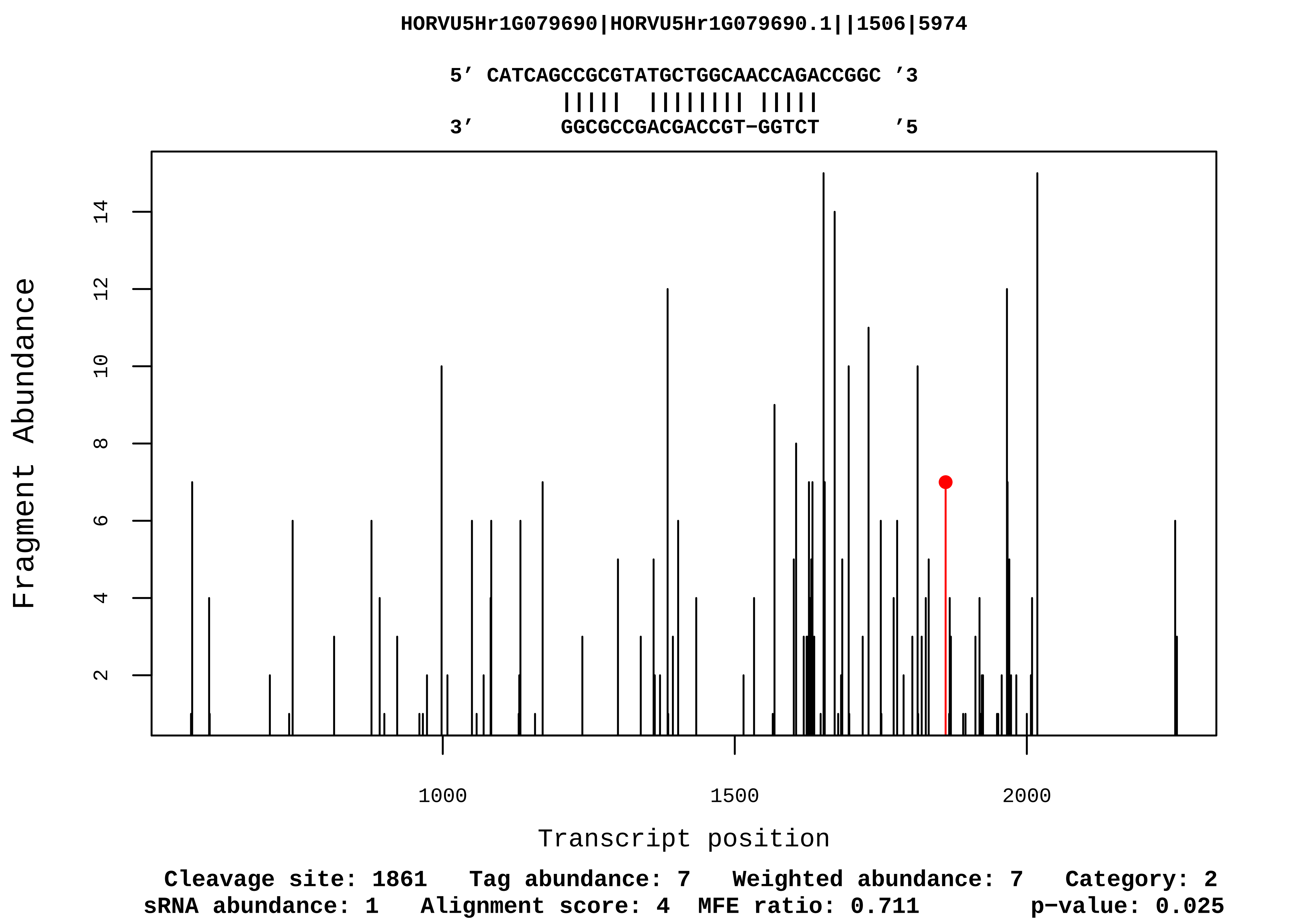

HORVU5Hr1G079690|HORVU5Hr1G079690.1||1506|5974
5' CATCAGCCGCGTATGCTGGCAACCAGACCGGC '3
3' GGCGCCGACGACCGT-GGTCT '5
Fragment Abundance
Transcript position
Cleavage site: 1861 Tag abundance: 7 Weighted abundance: 7 Category: 2
sRNA abundance: 1 Alignment score: 4 MFE ratio: 0.711 p-value: 0.025

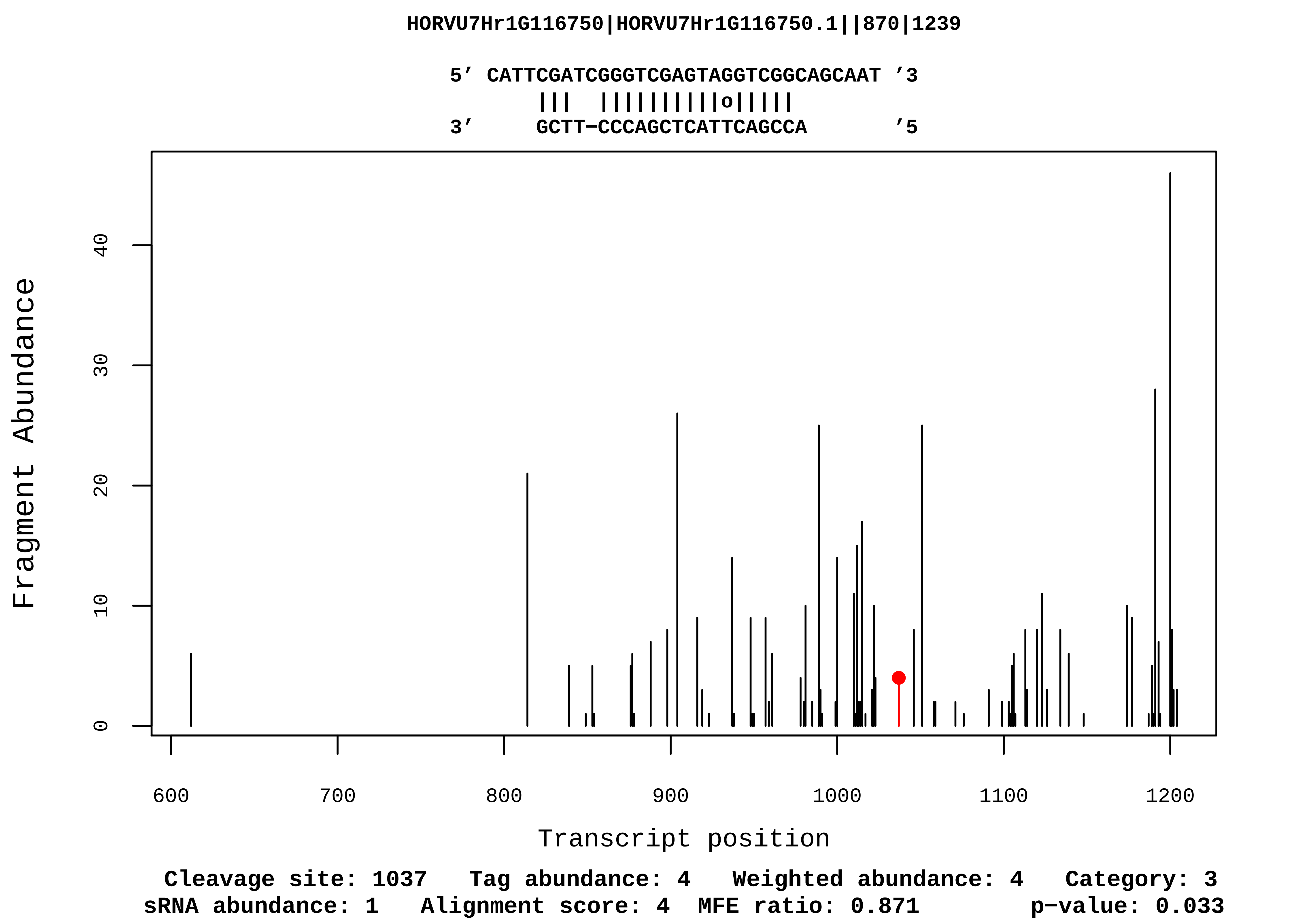

HORVU7Hr1G116750|HORVU7Hr1G116750.1||870|1239
5' CATTCGATCGGGTCGAGTAGGTCGGCAGCAAT '3
||| ||||||||||o|||||
3' GCTT-CCCAGCTCATTCAGCCA '5
Fragment Abundance
0
10
20
30
40
600
700
800
900
1000
1100
1200
Transcript position
Cleavage site: 1037 Tag abundance: 4 Weighted abundance: 4 Category: 3
sRNA abundance: 1 Alignment score: 4 MFE ratio: 0.871 p-value: 0.033

HORVU0Hr1G023910|HORVU0Hr1G023910.11||318|2086

```
5' AAACAGTCGGATTCCCCTTGTCCGTACCAGTT '3
       ||||||||||||||||||||||||
3'     GTCAGCCTAAGGGGAACAGGCAT       '5
```

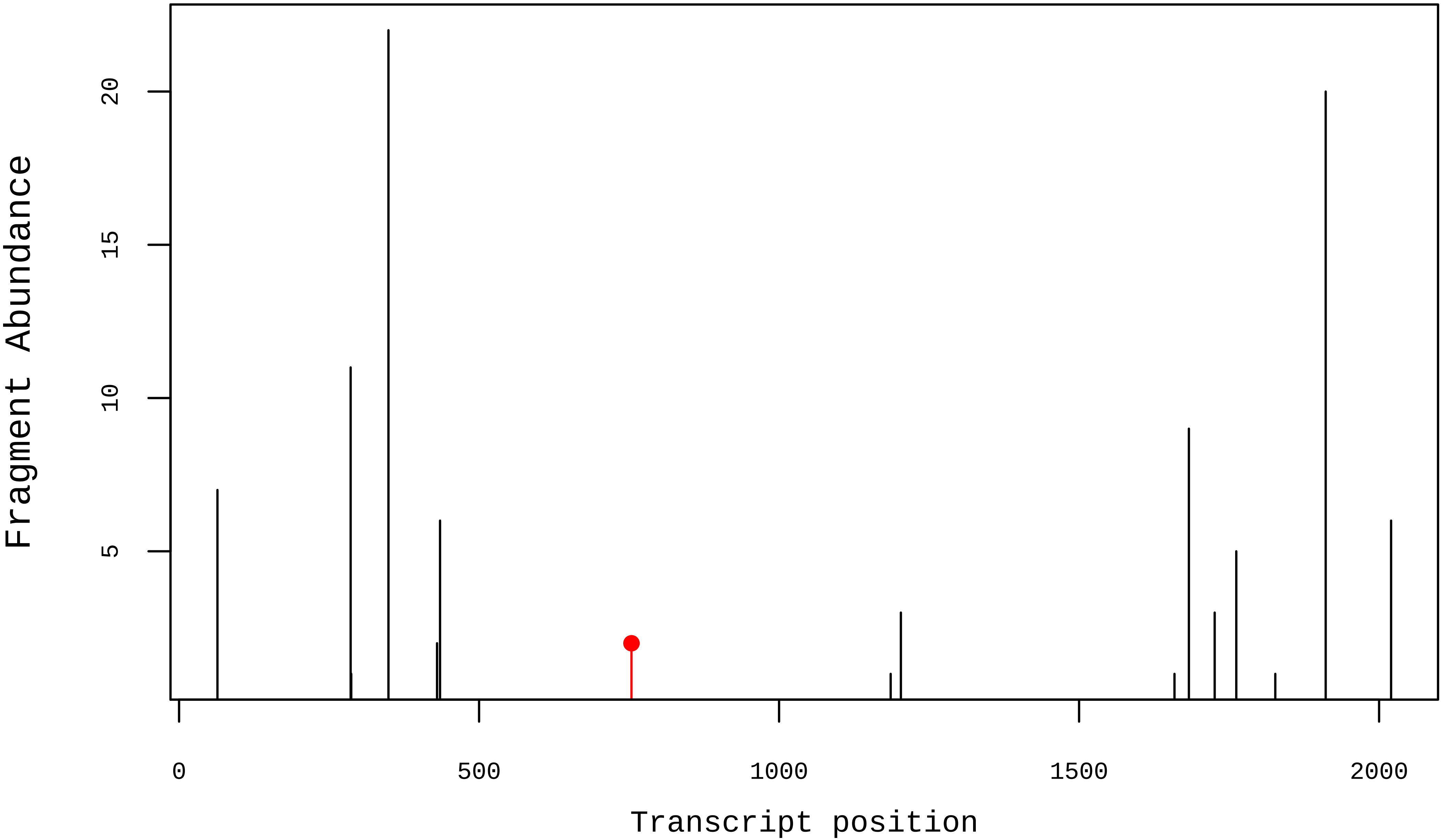

Cleavage site: 754   Tag abundance: 2   Weighted abundance: 0.1   Category: 3
sRNA abundance: 1   Alignment score: 0   MFE ratio: 1   p-value: 0.045

HORVU0Hr1G023910|HORVU0Hr1G023910.8||318|1667

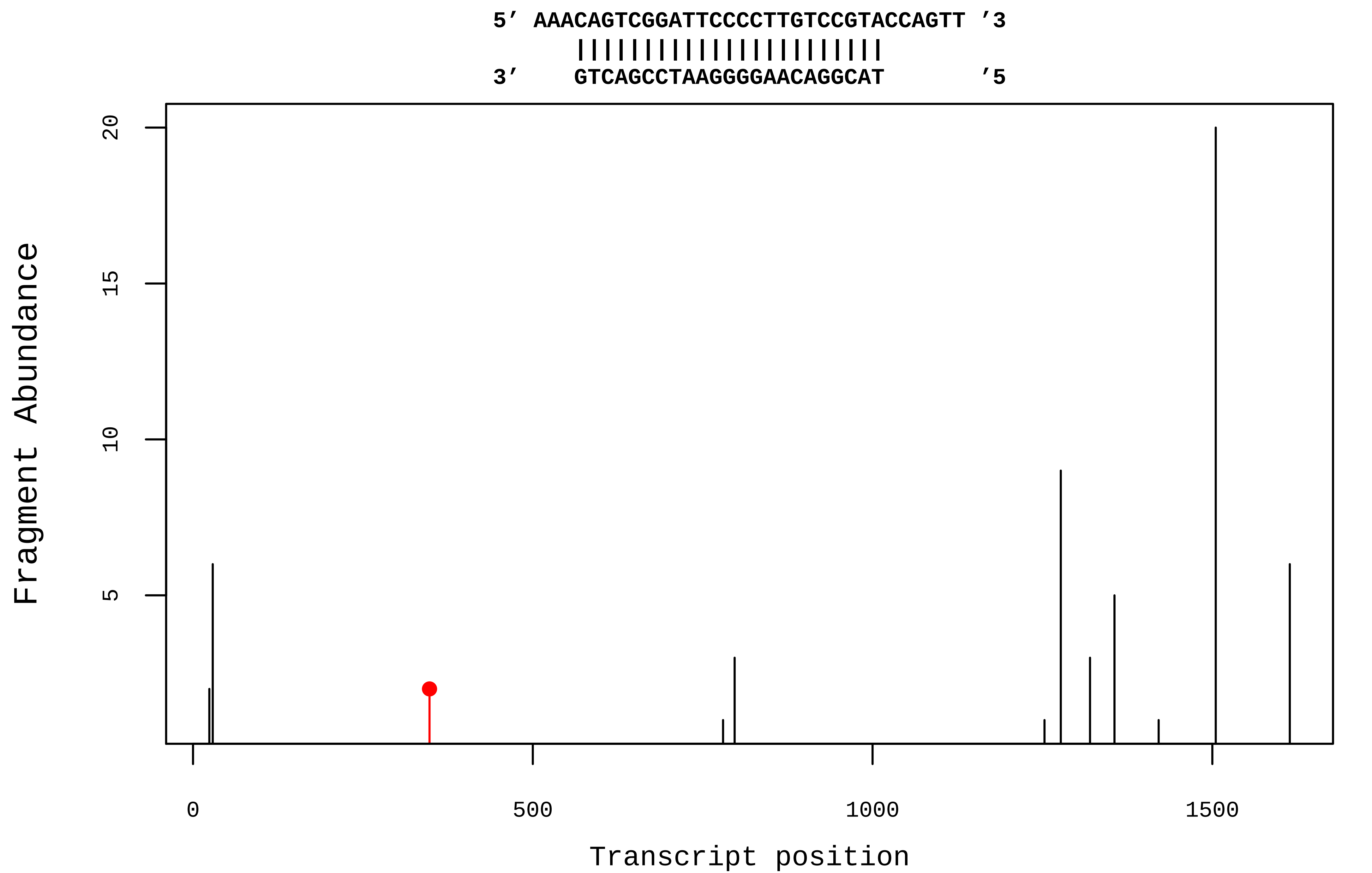

Cleavage site: 348 Tag abundance: 2 Weighted abundance: 0.1 Category: 3
sRNA abundance: 1 Alignment score: 0 MFE ratio: 1 p-value: 0.05

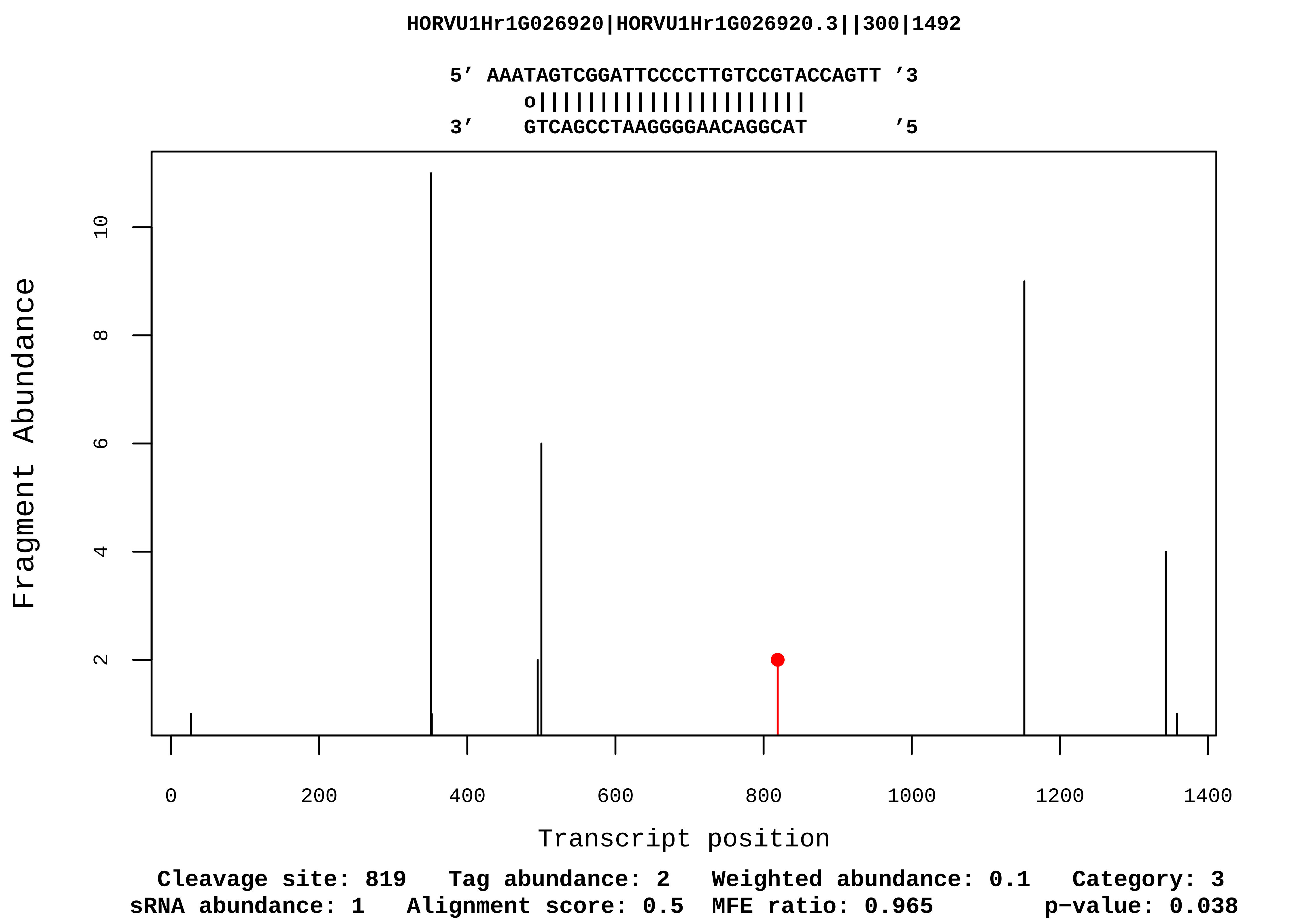

HORVU1Hr1G026920|HORVU1Hr1G026920.3||300|1492
5′ AAATAGTCGGATTCCCCTTGTCCGTACCAGTT ′3
o|||||||||||||||||||||
3′ GTCAGCCTAAGGGGAACAGGCAT ′5
Fragment Abundance
2
4
6
8
10
0
200
400
600
800
1000
1200
1400
Transcript position
Cleavage site: 819 Tag abundance: 2 Weighted abundance: 0.1 Category: 3
sRNA abundance: 1 Alignment score: 0.5 MFE ratio: 0.965 p−value: 0.038

HORVU1Hr1G026920|HORVU1Hr1G026920.4||300|2051

```
5' AAATAGTCGGATTCCCCTTGTCCGTACCAGTT '3
        o|||||||||||||||||||||
3'      GTCAGCCTAAGGGGAACAGGCAT       '5
```

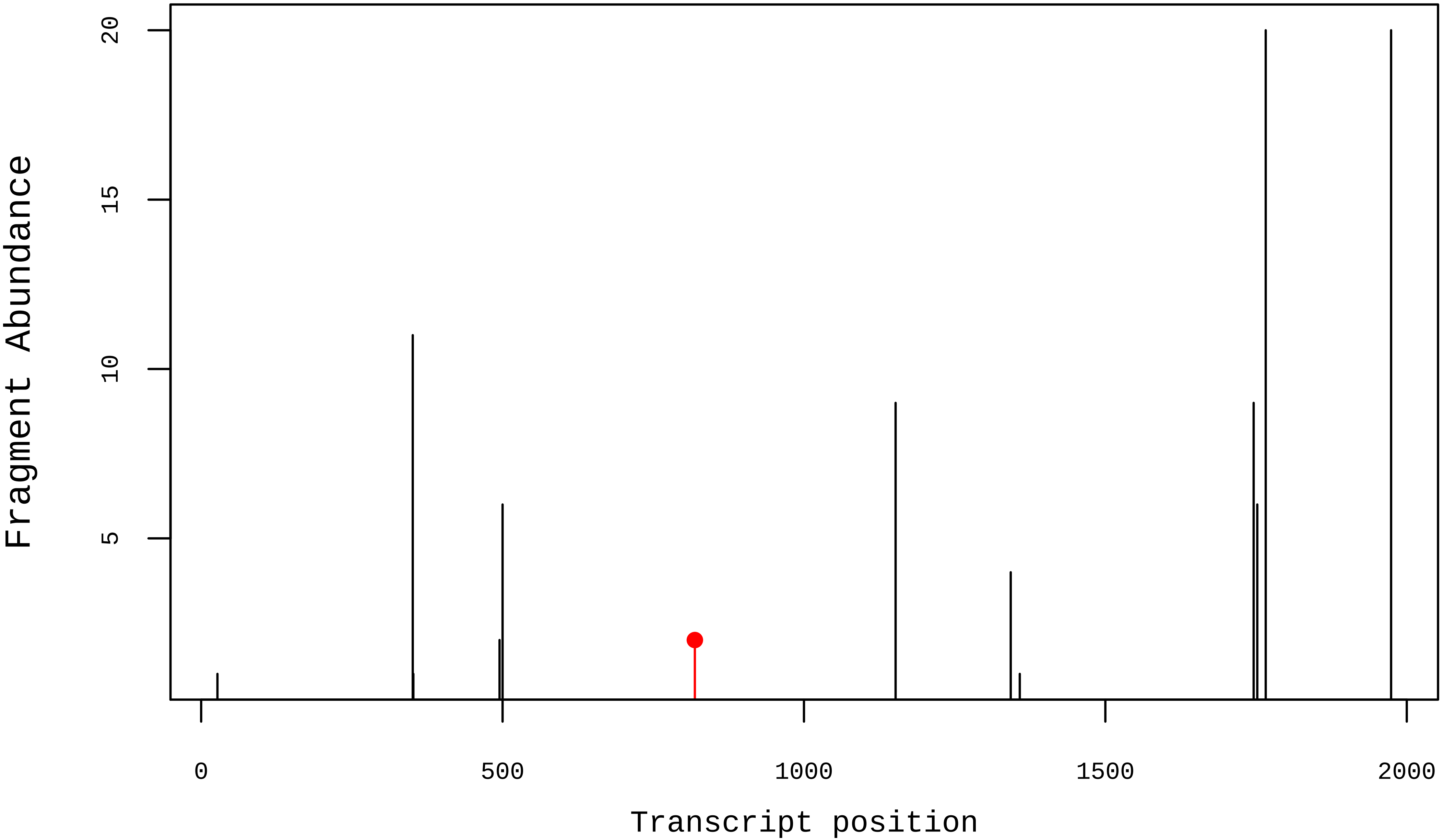

Cleavage site: 819 Tag abundance: 2 Weighted abundance: 0.1 Category: 3
sRNA abundance: 1 Alignment score: 0.5 MFE ratio: 0.965 p-value: 0.046

HORVU1Hr1G026920|HORVU1Hr1G026920.5||300|2039

```
5' AAATAGTCGGATTCCCCTTGTCCGTACCAGTT '3
        o||||||||||||||||||||||
3'      GTCAGCCTAAGGGGAACAGGCAT        '5
```

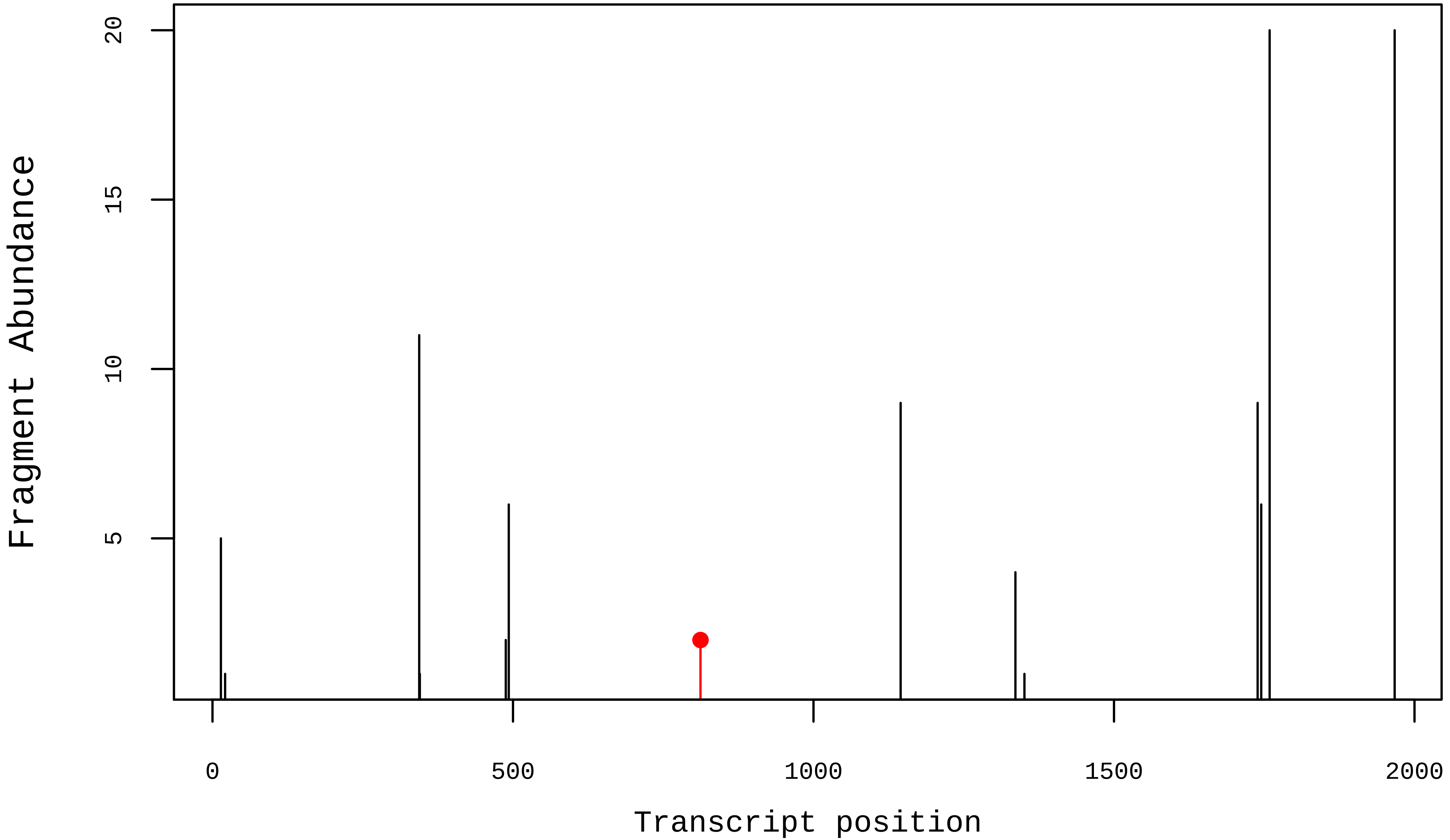

Cleavage site: 812 Tag abundance: 2 Weighted abundance: 0.1 Category: 3
sRNA abundance: 1 Alignment score: 0.5 MFE ratio: 0.965 p-value: 0.046

HORVU4Hr1G025470|HORVU4Hr1G025470.1||408|1650

```
5' AAACAGTCGGATTCCCCTTGTCCGTACCAGTT '3
        ||||||||||||||||||||||
3'      GTCAGCCTAAGGGGAACAGGCAT        '5
```

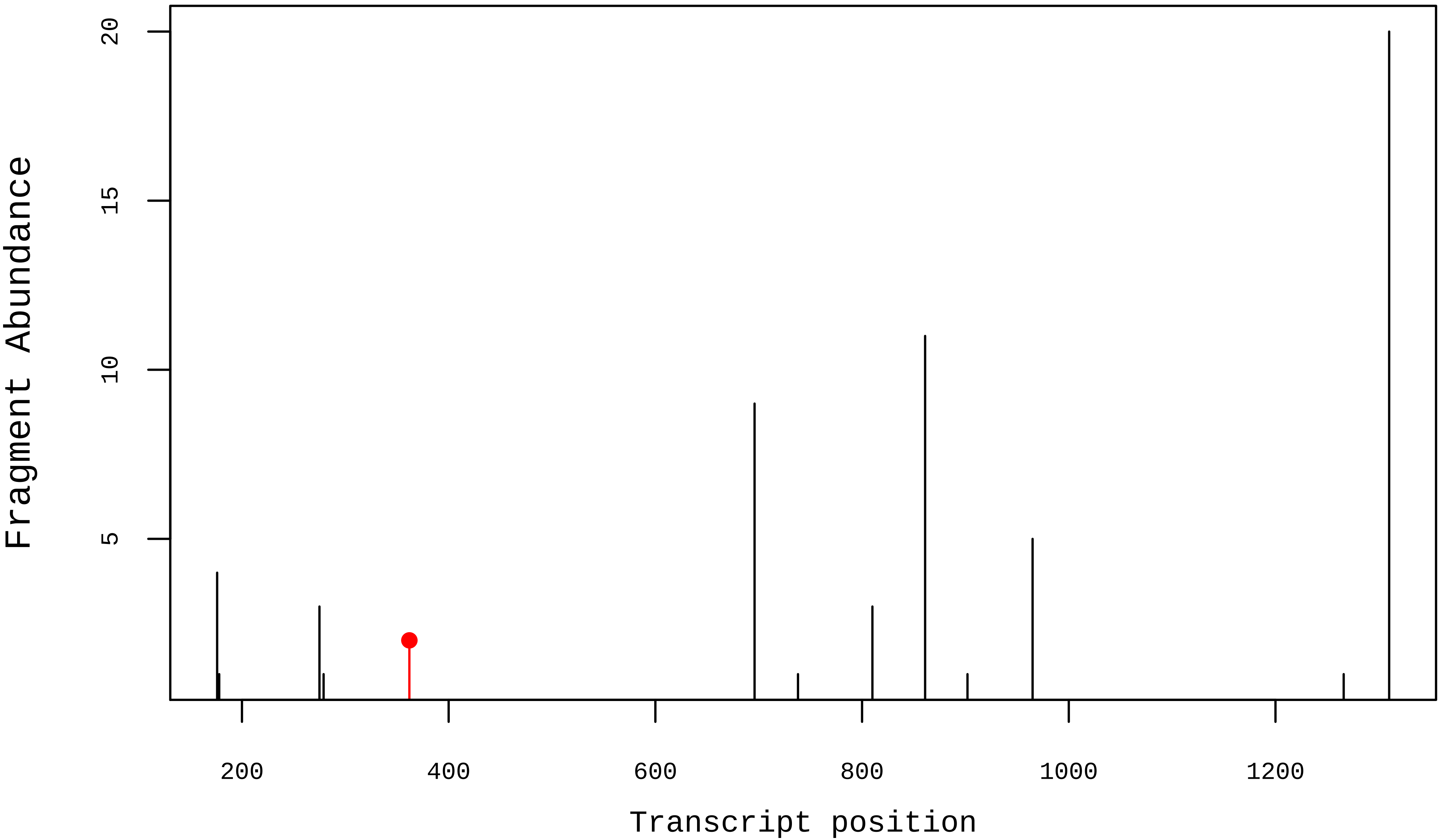

Cleavage site: 362   Tag abundance: 2   Weighted abundance: 0.1   Category: 3
sRNA abundance: 1   Alignment score: 0   MFE ratio: 1   p-value: 0.036

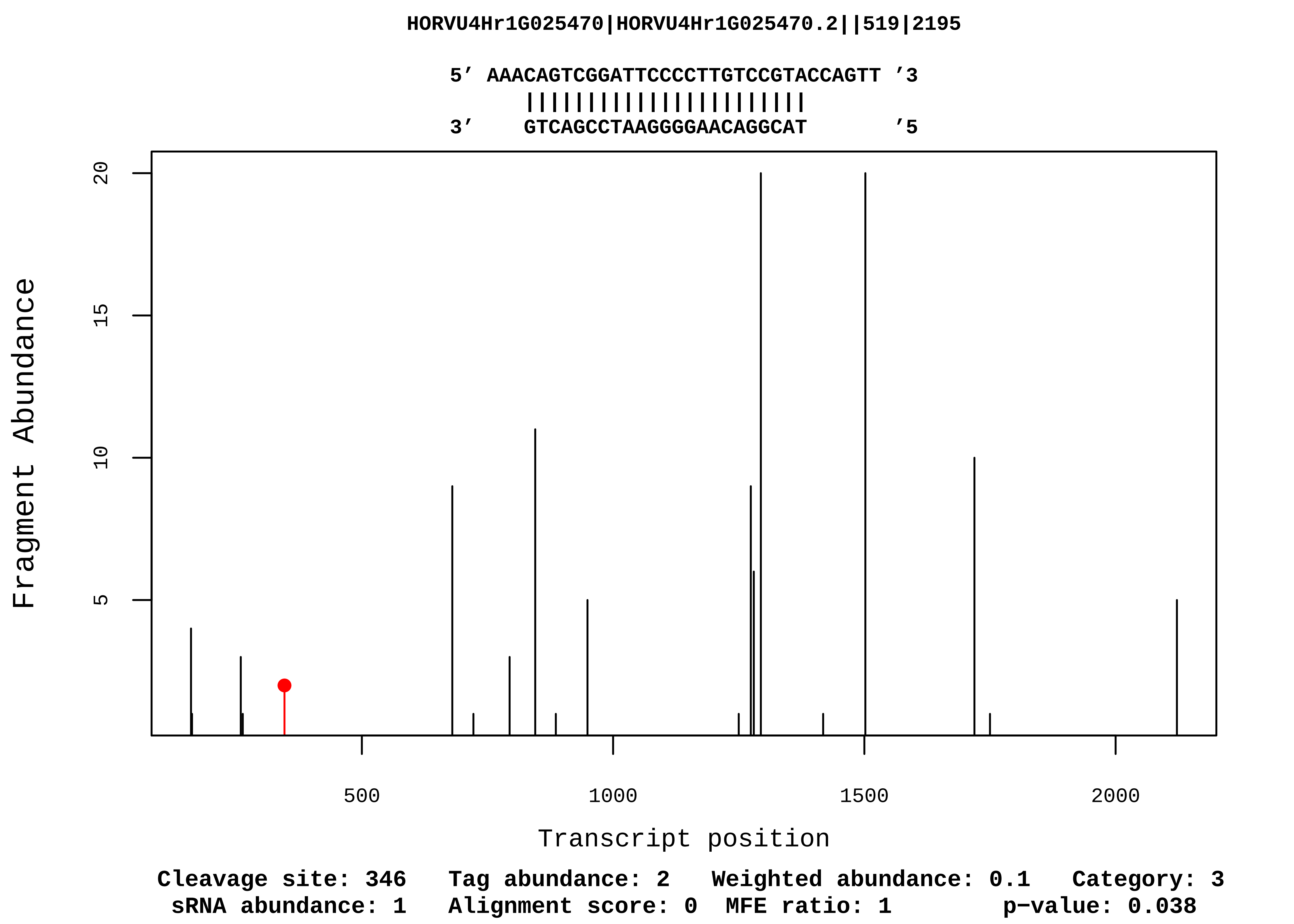

HORVU4Hr1G025470|HORVU4Hr1G025470.2||519|2195
5′ AAACAGTCGGATTCCCCTTGTCCGTACCAGTT ′3
||||||||||||||||||||||
3′ GTCAGCCTAAGGGGAACAGGCAT ′5
Fragment Abundance
5
10
15
20
500
1000
1500
2000
Transcript position
Cleavage site: 346 Tag abundance: 2 Weighted abundance: 0.1 Category: 3
sRNA abundance: 1 Alignment score: 0 MFE ratio: 1 p−value: 0.038

HORVU4Hr1G025590|HORVU4Hr1G025590.1||378|3386

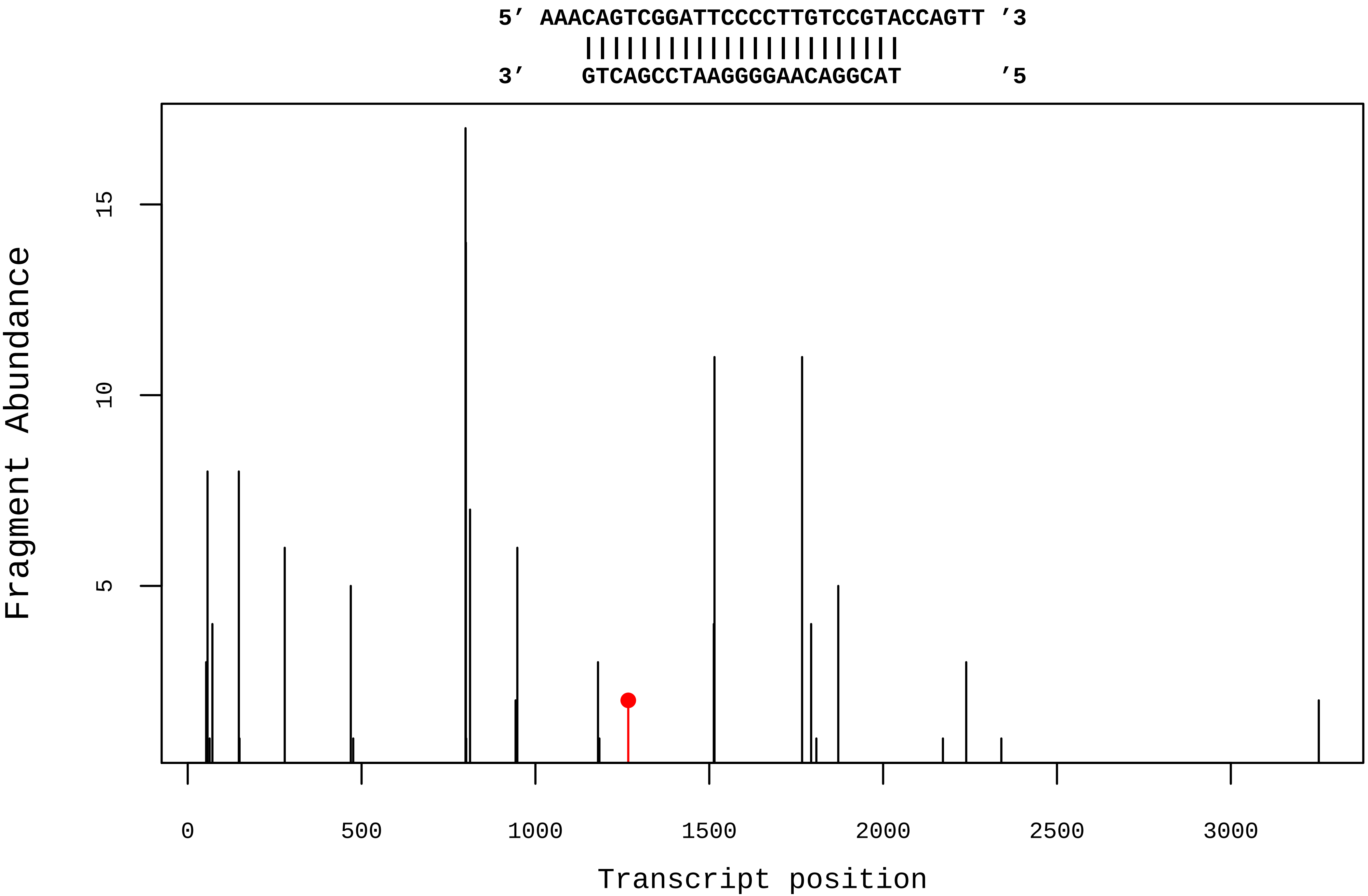

Cleavage site: 1267 Tag abundance: 2 Weighted abundance: 0.1 Category: 3
sRNA abundance: 1 Alignment score: 0 MFE ratio: 1 p-value: 0.045

HORVU5Hr1G015390|HORVU5Hr1G015390.1||318|1287

```
5' AAATAGTCGGATTCCCCTTGTCCGTACCAGTT '3
        o|||||||||||||||||||||
3'      GTCAGCCTAAGGGGAACAGGCAT       '5
```

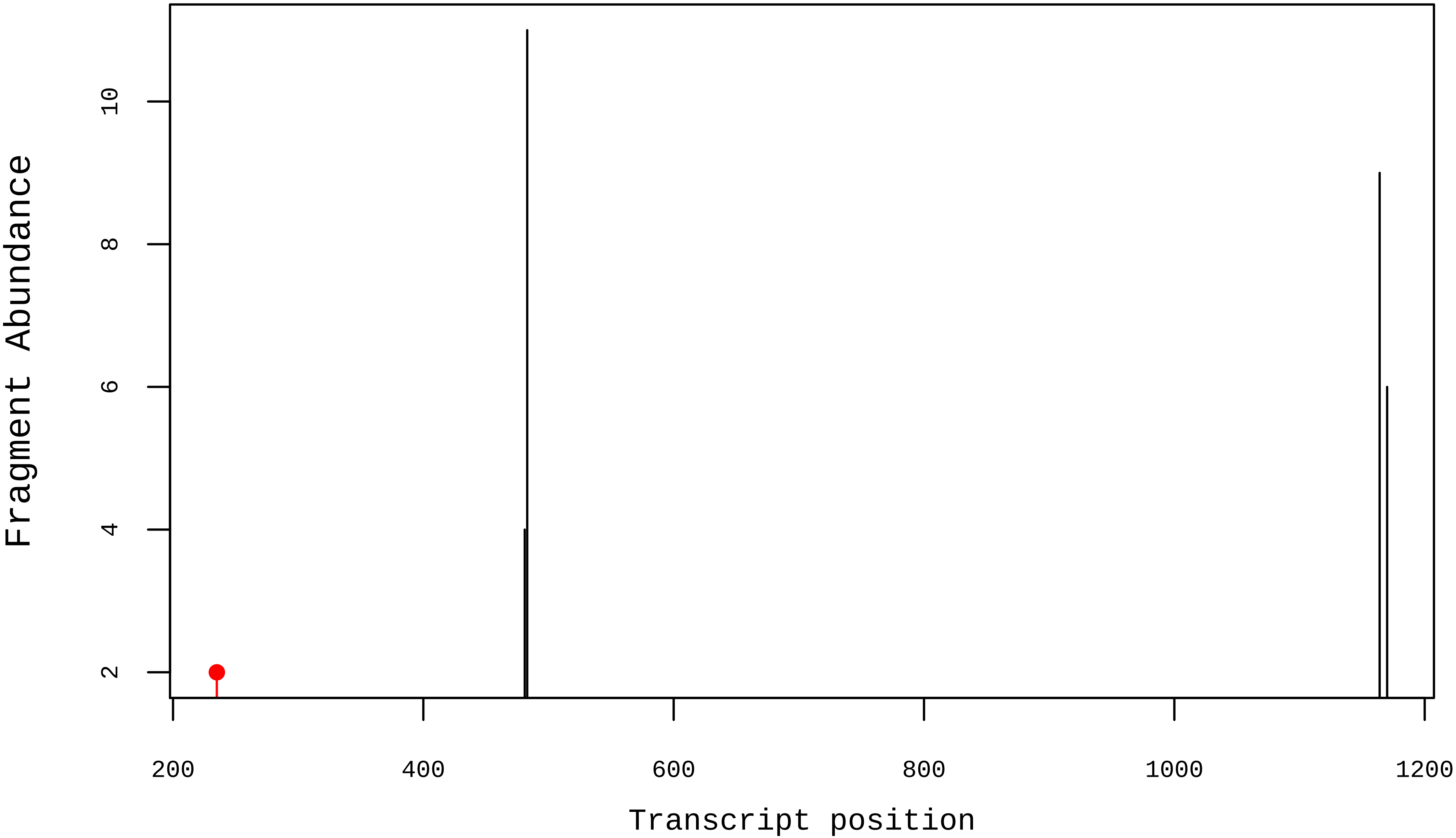

Cleavage site: 235   Tag abundance: 2   Weighted abundance: 0.1   Category: 3
sRNA abundance: 1   Alignment score: 0.5   MFE ratio: 0.965   p-value: 0.044

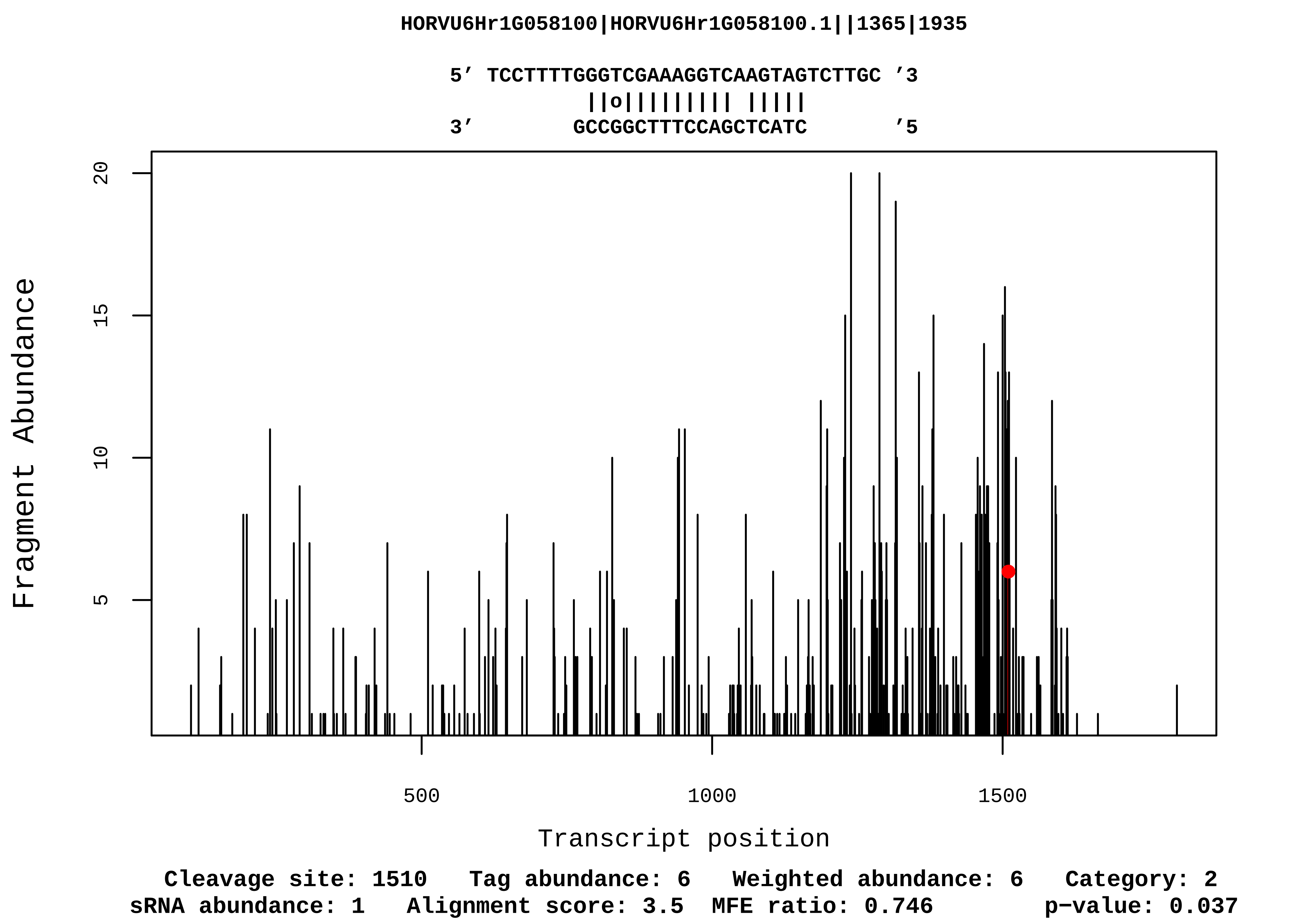

HORVU6Hr1G058100|HORVU6Hr1G058100.1||1365|1935
5' TCCTTTTGGGTCGAAAGGTCAAGTAGTCTTGC '3
||o||||||||| |||||
3' GCCGGCTTTCCAGCTCATC '5
Fragment Abundance
20
15
10
5
500
1000
1500
Transcript position
Cleavage site: 1510 Tag abundance: 6 Weighted abundance: 6 Category: 2
sRNA abundance: 1 Alignment score: 3.5 MFE ratio: 0.746 p-value: 0.037

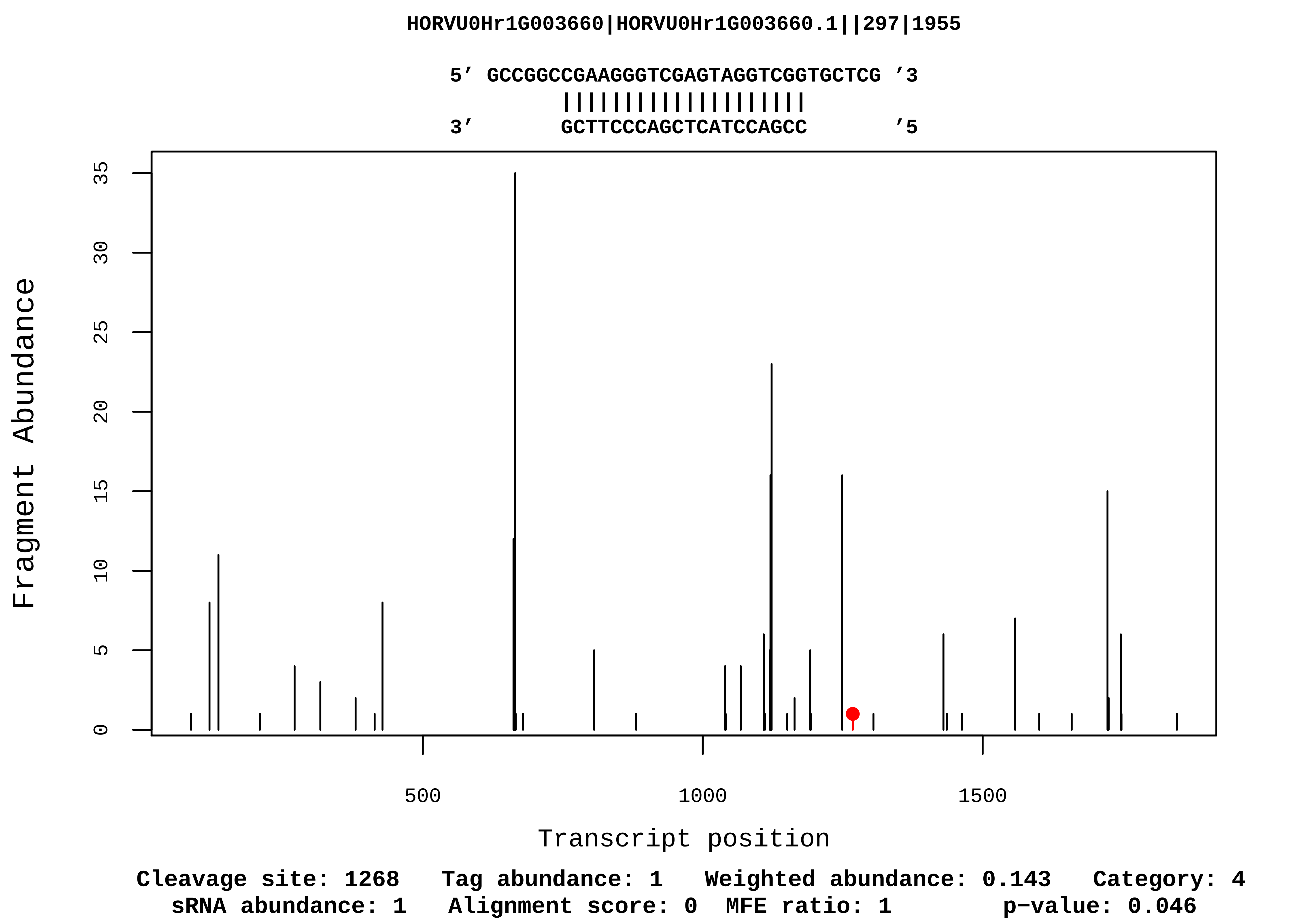

HORVU0Hr1G003660|HORVU0Hr1G003660.1||297|1955
5' GCCGGCCGAAGGGTCGAGTAGGTCGGTGCTCG '3
||||||||||||||||||||
3' GCTTCCCAGCTCATCCAGCC '5
Fragment Abundance
0
5
10
15
20
25
30
35
500
1000
1500
Transcript position
Cleavage site: 1268 Tag abundance: 1 Weighted abundance: 0.143 Category: 4
sRNA abundance: 1 Alignment score: 0 MFE ratio: 1 p-value: 0.046

HORVU0Hr1G023930|HORVU0Hr1G023930.3||396|2180

```
5' GTCGGCGGAAGGGTCGAGTAGGTCGGTGCTCG '3
               | ||||||||||||||||||
3'             G-CTTCCCAGCTCATCCAGCC       '5
```

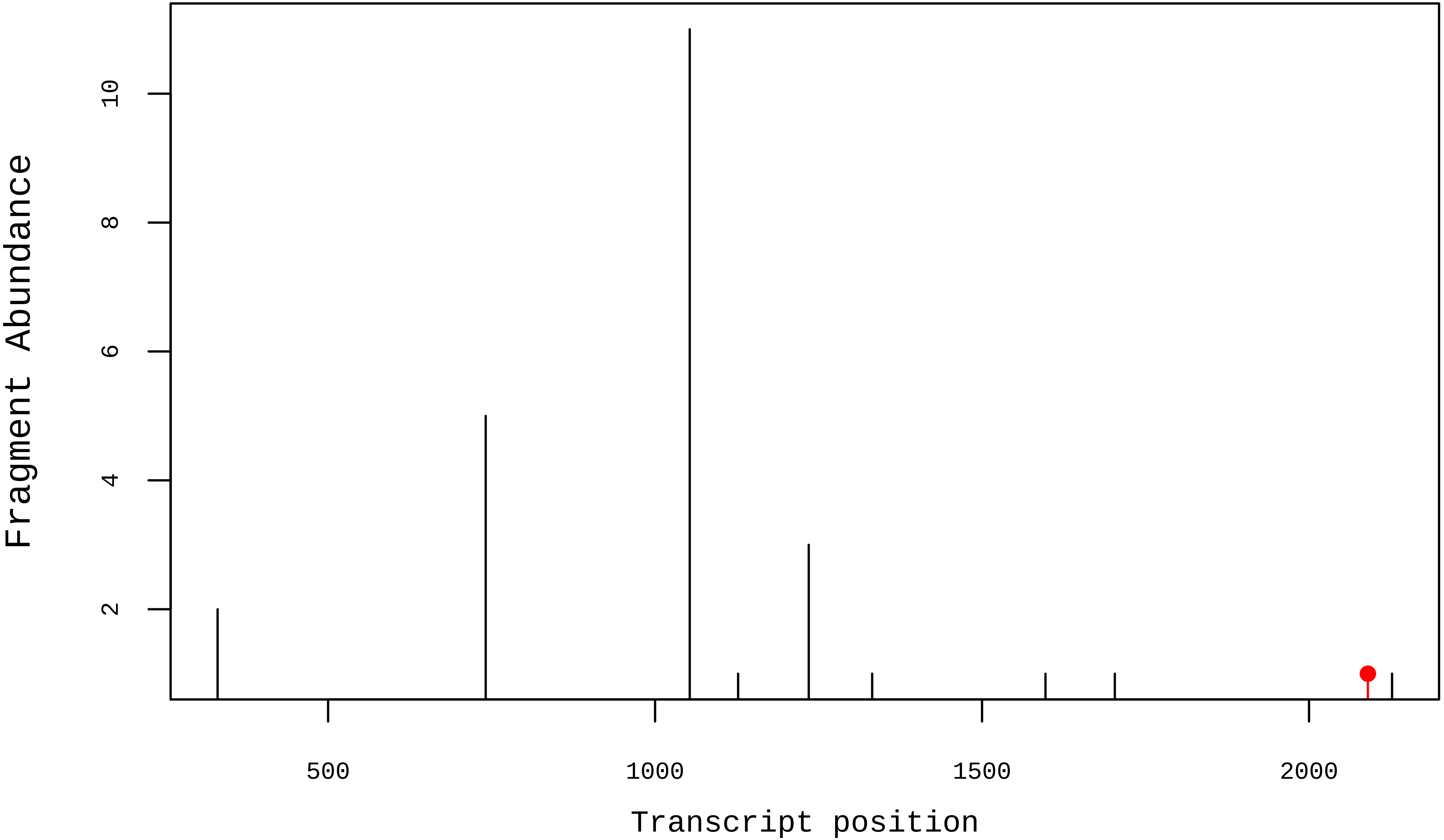

Cleavage site: 2090 Tag abundance: 1 Weighted abundance: 0.143 Category: 4
sRNA abundance: 1 Alignment score: 1 MFE ratio: 0.966 p-value: 0.019

HORVU2Hr1G072960|HORVU2Hr1G072960.1||600|3353

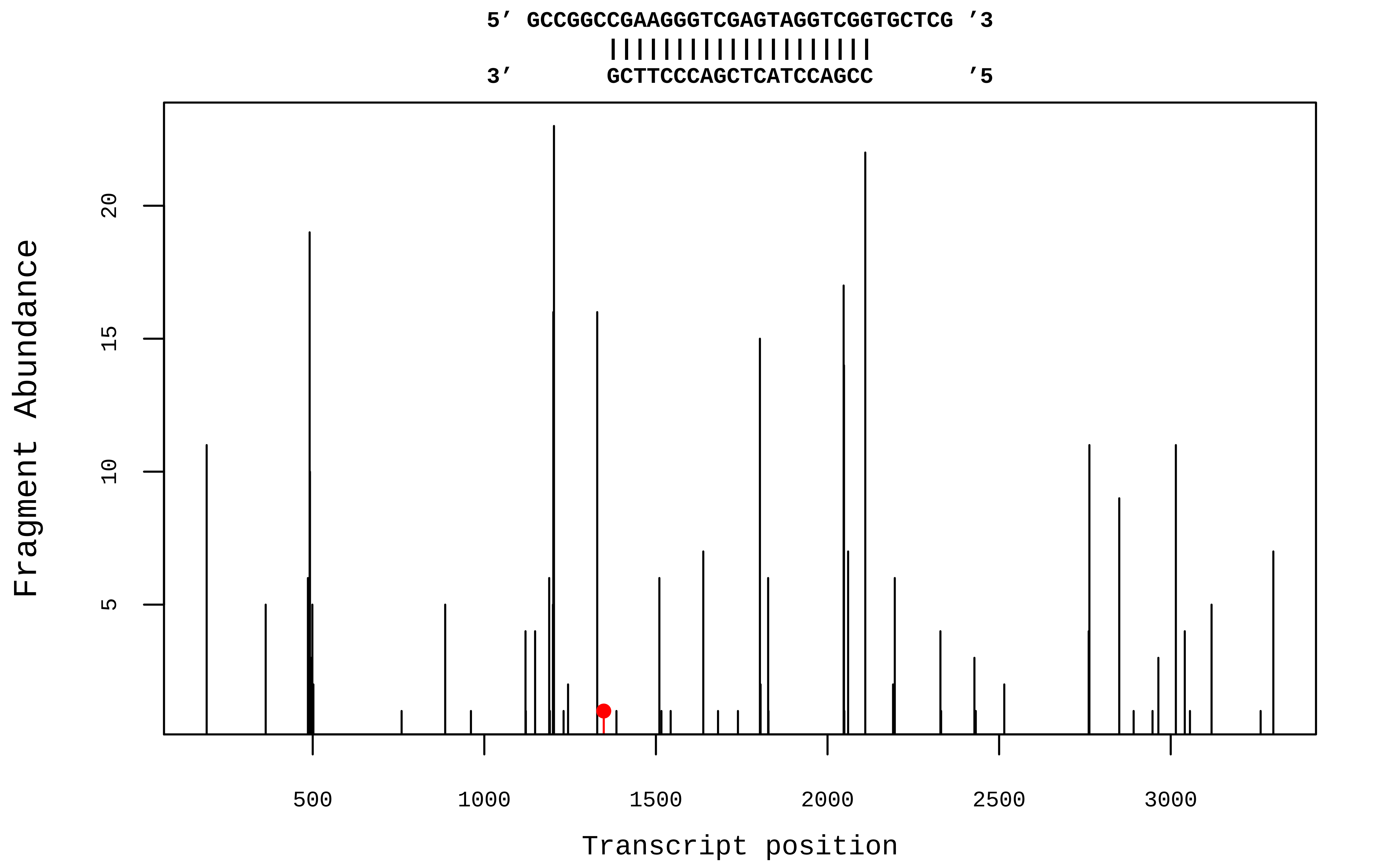

Cleavage site: 1348 Tag abundance: 1 Weighted abundance: 0.143 Category: 4
sRNA abundance: 1 Alignment score: 0 MFE ratio: 1 p-value: 0.03

HORVU5Hr1G015600|HORVU5Hr1G015600.1||156|510

```
5' GCCGGCCGAAGGGTCGAGTAGGTCGGTGCTCG '3
         |||||||||||||||||||
3'       GCTTCCCAGCTCATCCAGCC          '5
```

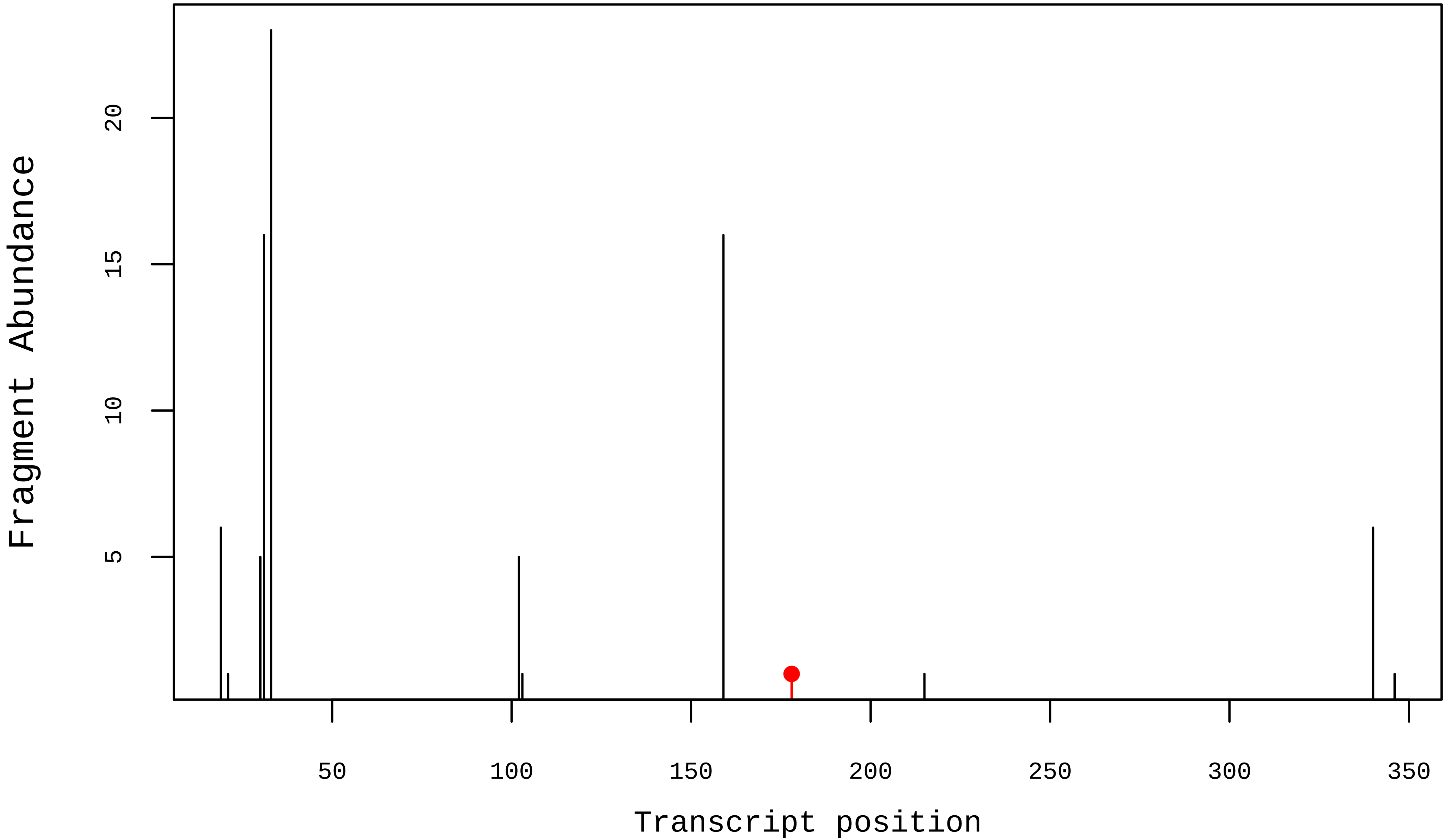

Cleavage site: 178   Tag abundance: 1   Weighted abundance: 0.143   Category: 4
sRNA abundance: 1   Alignment score: 0   MFE ratio: 1   p-value: 0.05

HORVU5Hr1G015600|HORVU5Hr1G015600.2||231|617

```
5' GCCGGCCGAAGGGTCGAGTAGGTCGGTGCTCG '3
         ||||||||||||||||||||
3'       GCTTCCCAGCTCATCCAGCC       '5
```

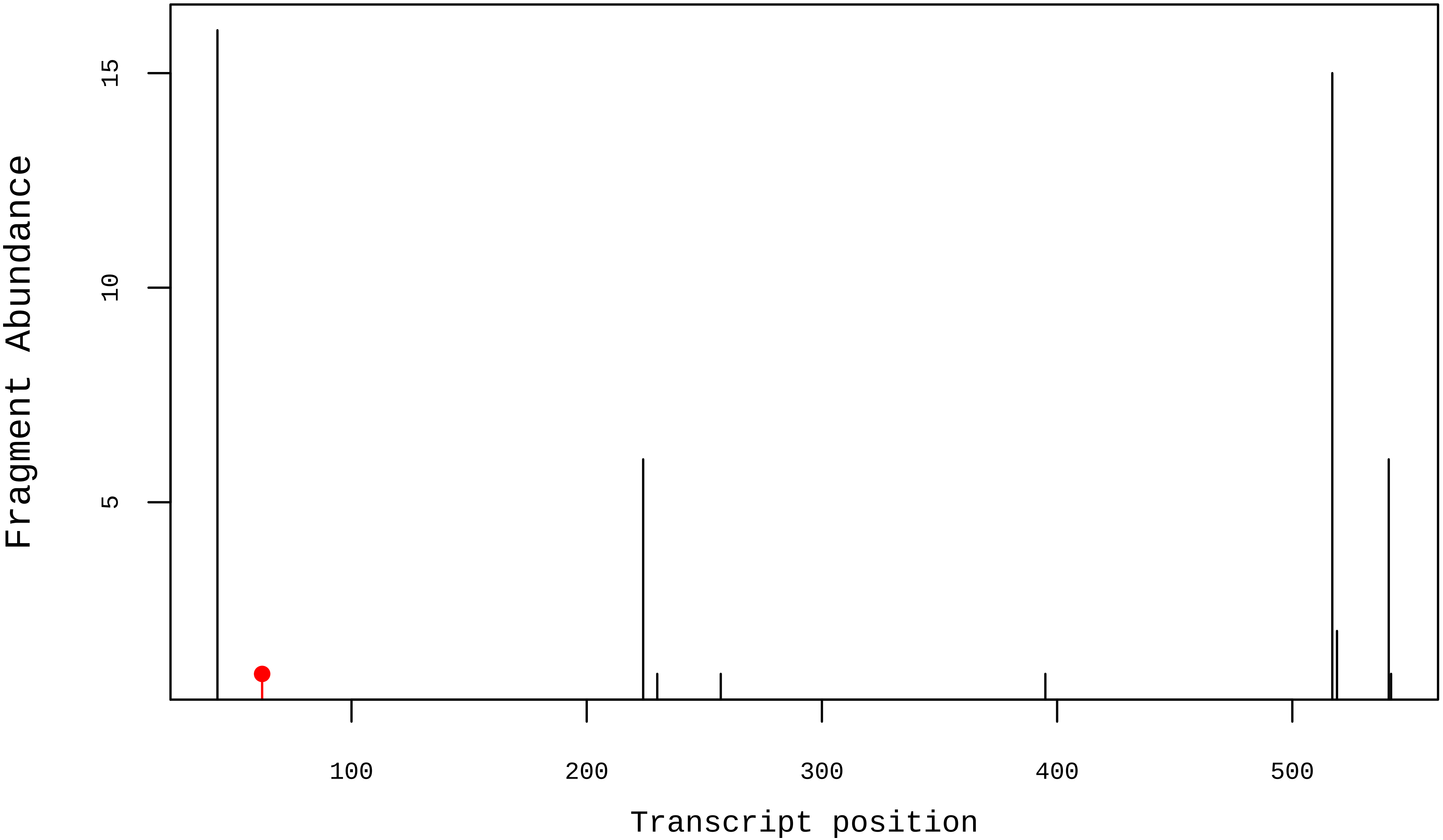

Cleavage site: 62 Tag abundance: 1 Weighted abundance: 0.143 Category: 4
sRNA abundance: 1 Alignment score: 0 MFE ratio: 1 p-value: 0.041

HORVU5Hr1G015600|HORVU5Hr1G015600.3||276|1709

```
5' GCCGGCCGAAGGGTCGAGTAGGTCGGTGCTCG '3
         |||||||||||||||||||
3'       GCTTCCCAGCTCATCCAGCC       '5
```

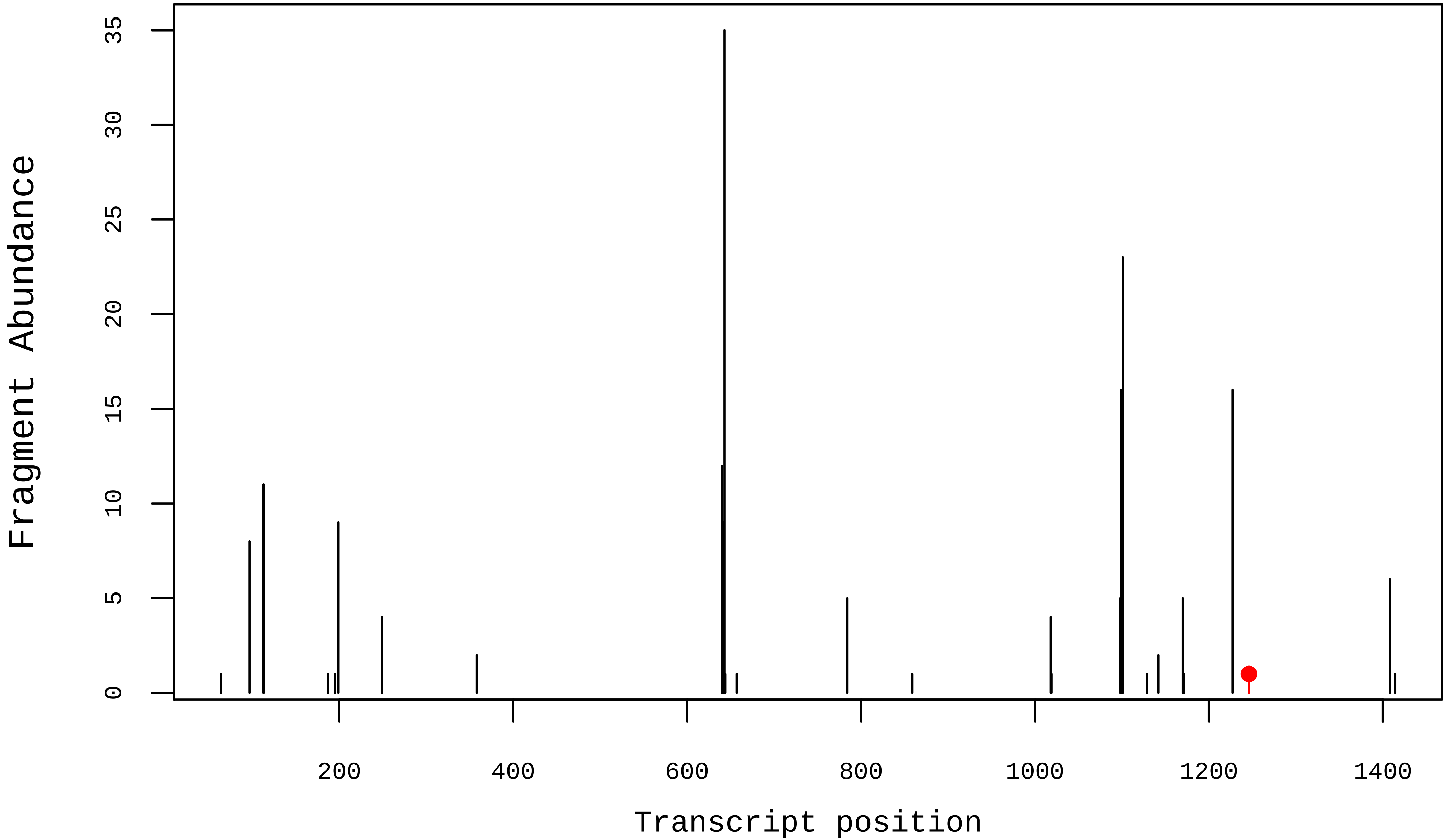

Cleavage site: 1246 Tag abundance: 1 Weighted abundance: 0.143 Category: 4
sRNA abundance: 1 Alignment score: 0 MFE ratio: 1 p-value: 0.032

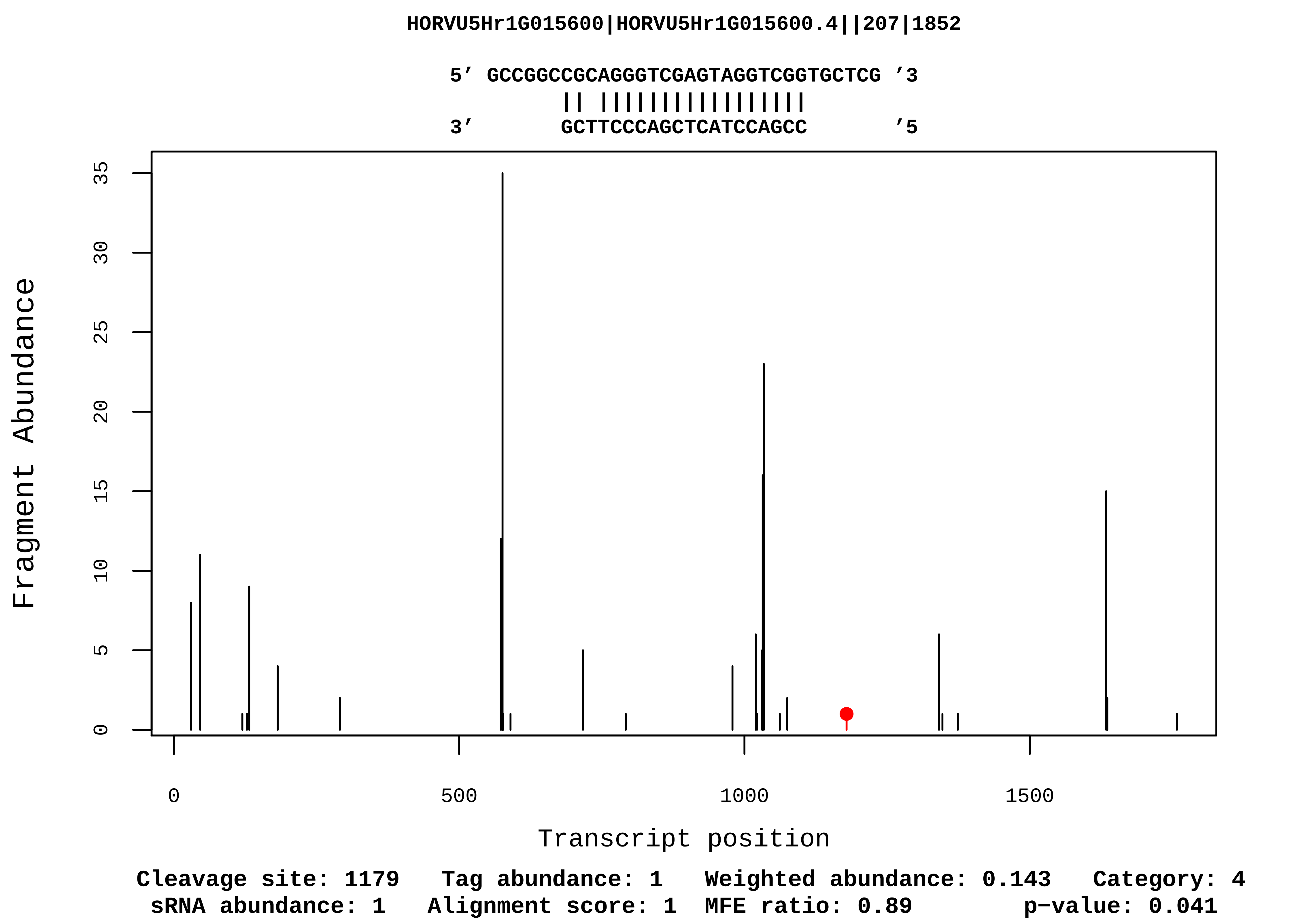
HORVU5Hr1G015600|HORVU5Hr1G015600.4||207|1852
5′ GCCGGCCGCAGGGTCGAGTAGGTCGGTGCTCG ′3
|| |||||||||||||||||
3′ GCTTCCCAGCTCATCCAGCC ′5
Fragment Abundance
0
5
10
15
20
25
30
35
0
500
1000
1500
Transcript position
Cleavage site: 1179 Tag abundance: 1 Weighted abundance: 0.143 Category: 4
sRNA abundance: 1 Alignment score: 1 MFE ratio: 0.89 p-value: 0.041

HORVU0Hr1G003660|HORVU0Hr1G003660.1||297|1955

```
5' GCCGGCCGAAGGGTCGAGTAGGTCGGTGCTCG '3
      ||o|||||||||||||||||||||
3'    GCTGGCTTCCCAGCTCATCCAGCC      '5
```

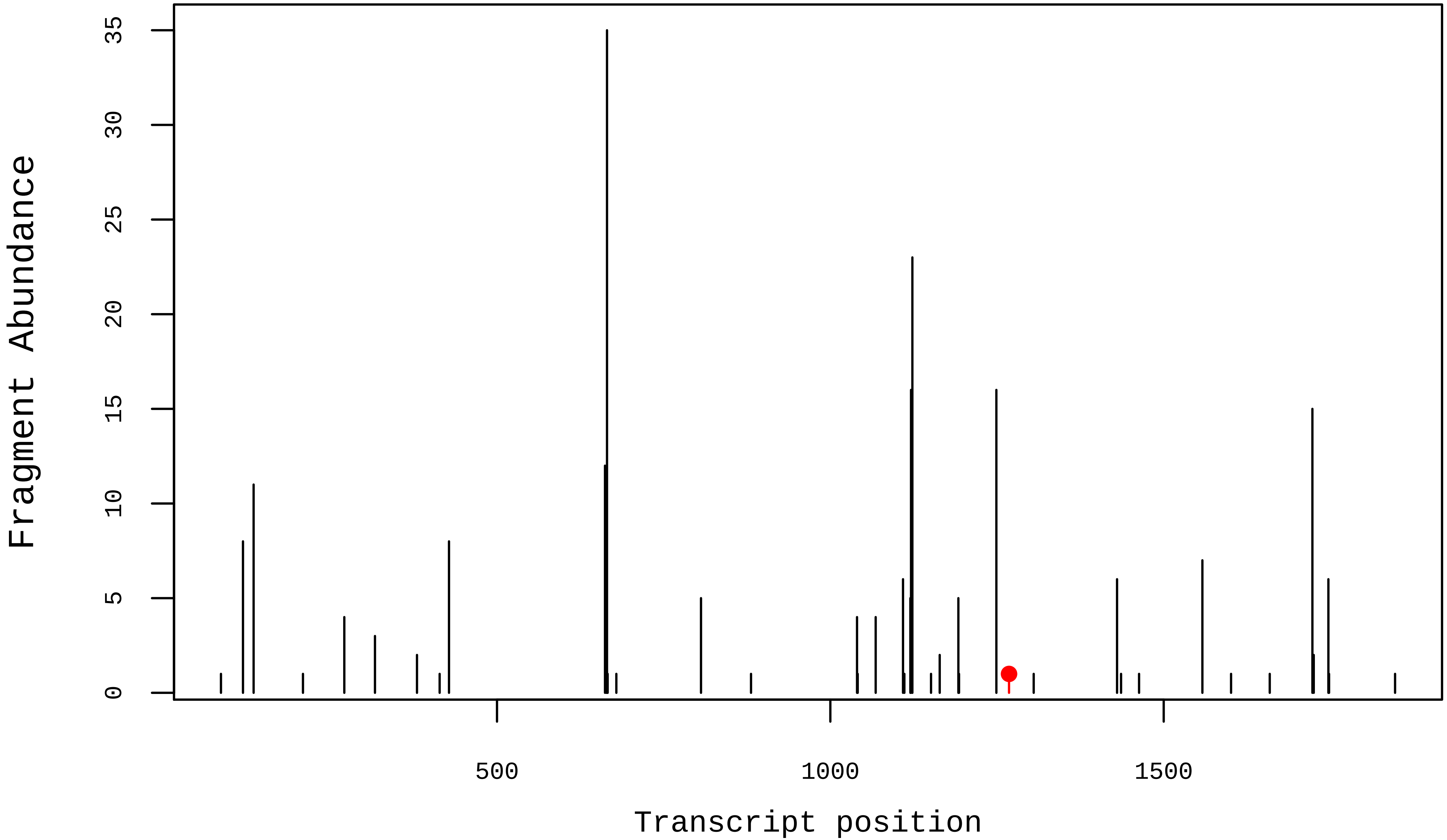

Cleavage site: 1268 Tag abundance: 1 Weighted abundance: 0.143 Category: 4
sRNA abundance: 1 Alignment score: 0.5 MFE ratio: 0.988 p-value: 0.046

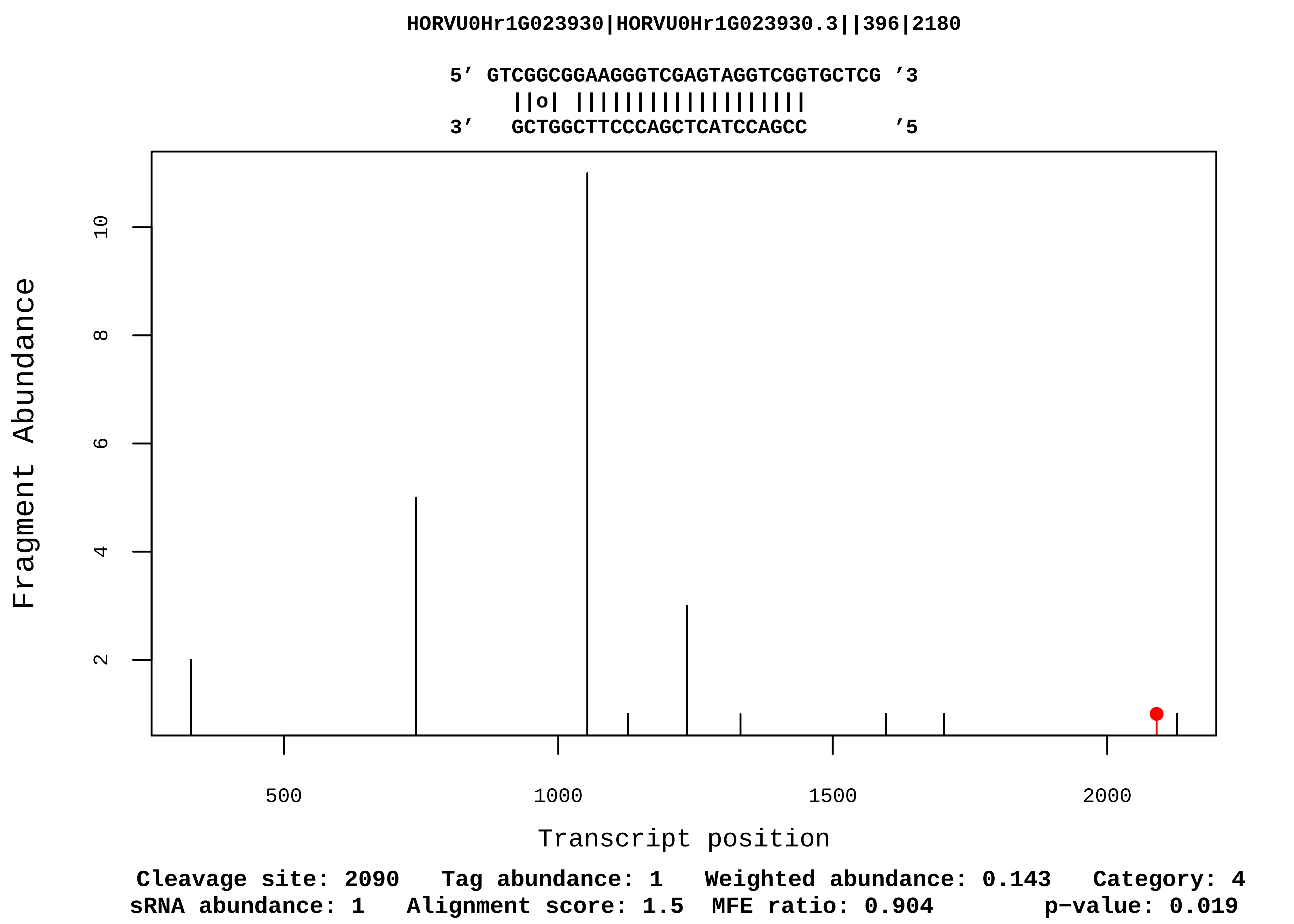
HORVU0Hr1G023930|HORVU0Hr1G023930.3||396|2180
5' GTCGGCGGAAGGGTCGAGTAGGTCGGTGCTCG '3
||o| ||||||||||||||||||
3' GCTGGCTTCCCAGCTCATCCAGCC '5
Fragment Abundance
2
4
6
8
10
500
1000
1500
2000
Transcript position
Cleavage site: 2090 Tag abundance: 1 Weighted abundance: 0.143 Category: 4
sRNA abundance: 1 Alignment score: 1.5 MFE ratio: 0.904 p-value: 0.019

HORVU2Hr1G072960|HORVU2Hr1G072960.1||600|3353

```
5' GCCGGCCGAAGGGTCGAGTAGGTCGGTGCTCG '3
      ||o|||||||||||||||||||||
3'    GCTGGCTTCCCAGCTCATCCAGCC       '5
```

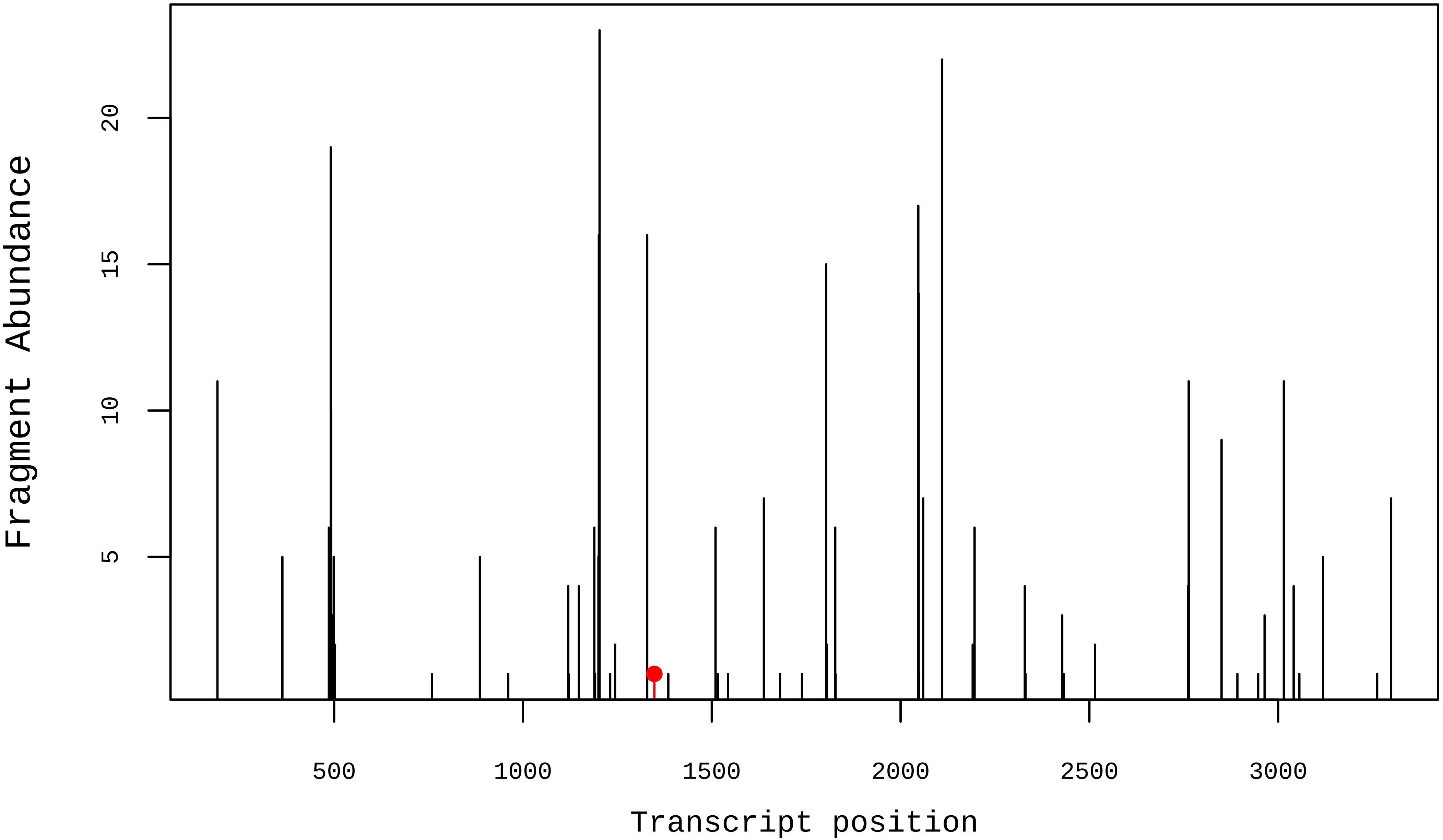

Cleavage site: 1348   Tag abundance: 1   Weighted abundance: 0.143   Category: 4
sRNA abundance: 1   Alignment score: 0.5   MFE ratio: 0.988   p-value: 0.03

HORVU5Hr1G015600|HORVU5Hr1G015600.1||156|510

```
5' GCCGGCCGAAGGGTCGAGTAGGTCGGTGCTCG '3
      ||o|||||||||||||||||||||
3'    GCTGGCTTCCCAGCTCATCCAGCC       '5
```

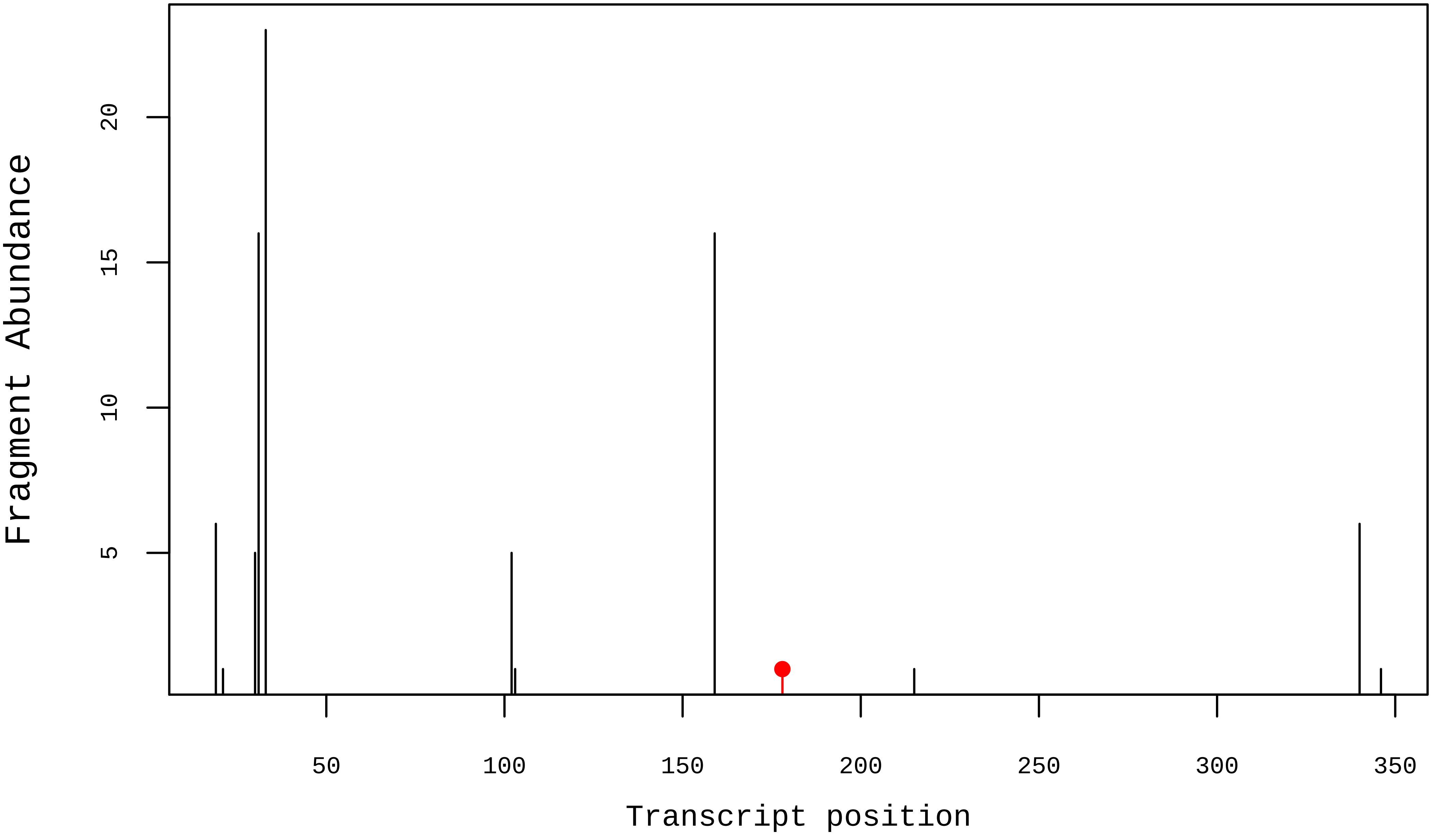

Cleavage site: 178   Tag abundance: 1   Weighted abundance: 0.143   Category: 4
sRNA abundance: 1   Alignment score: 0.5   MFE ratio: 0.988   p-value: 0.05

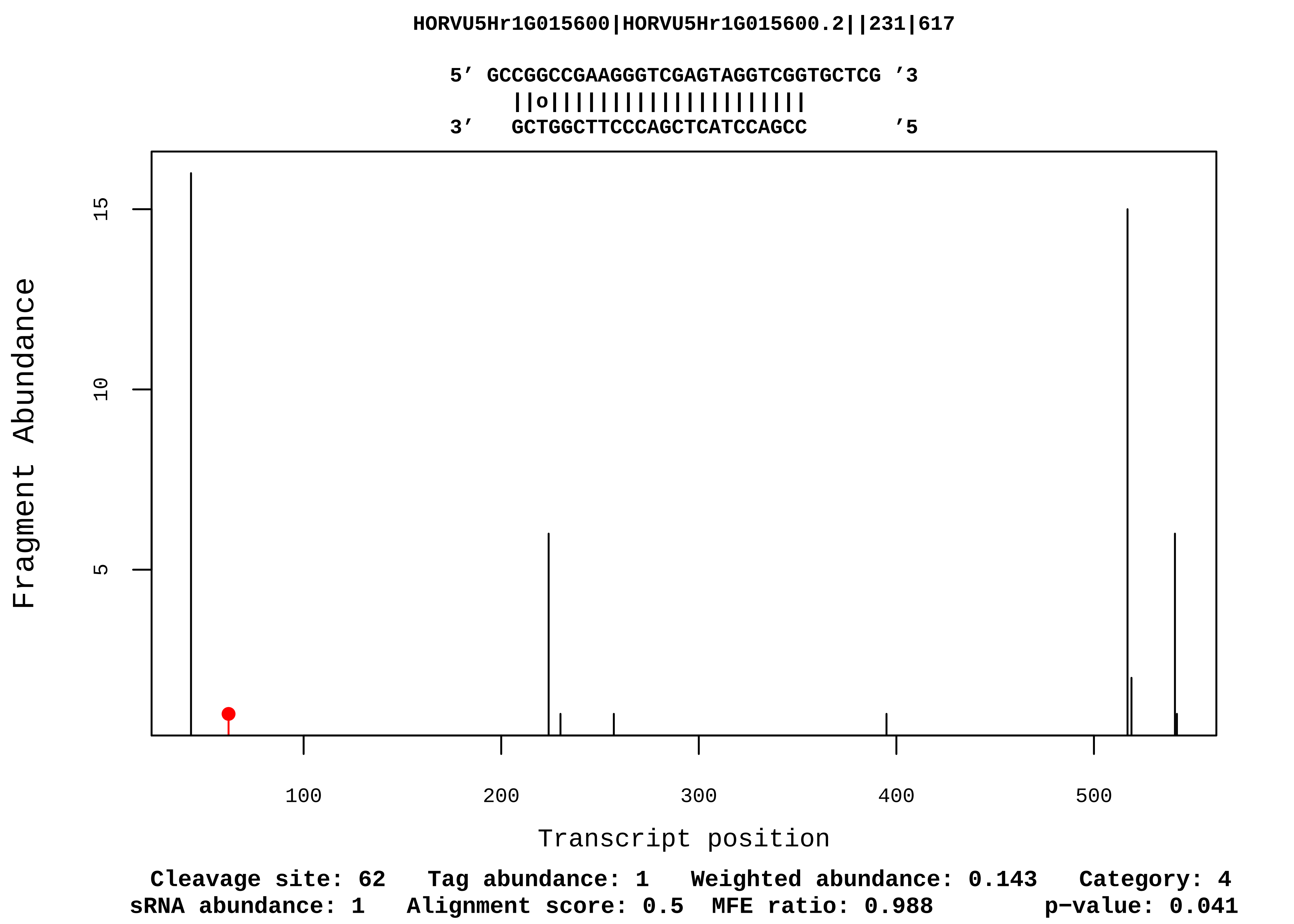
HORVU5Hr1G015600|HORVU5Hr1G015600.2||231|617
5' GCCGGCCGAAGGGTCGAGTAGGTCGGTGCTCG '3
||o|||||||||||||||||||||
3' GCTGGCTTCCCAGCTCATCCAGCC '5
Fragment Abundance
5
10
15
100
200
300
400
500
Transcript position
Cleavage site: 62 Tag abundance: 1 Weighted abundance: 0.143 Category: 4
sRNA abundance: 1 Alignment score: 0.5 MFE ratio: 0.988 p-value: 0.041

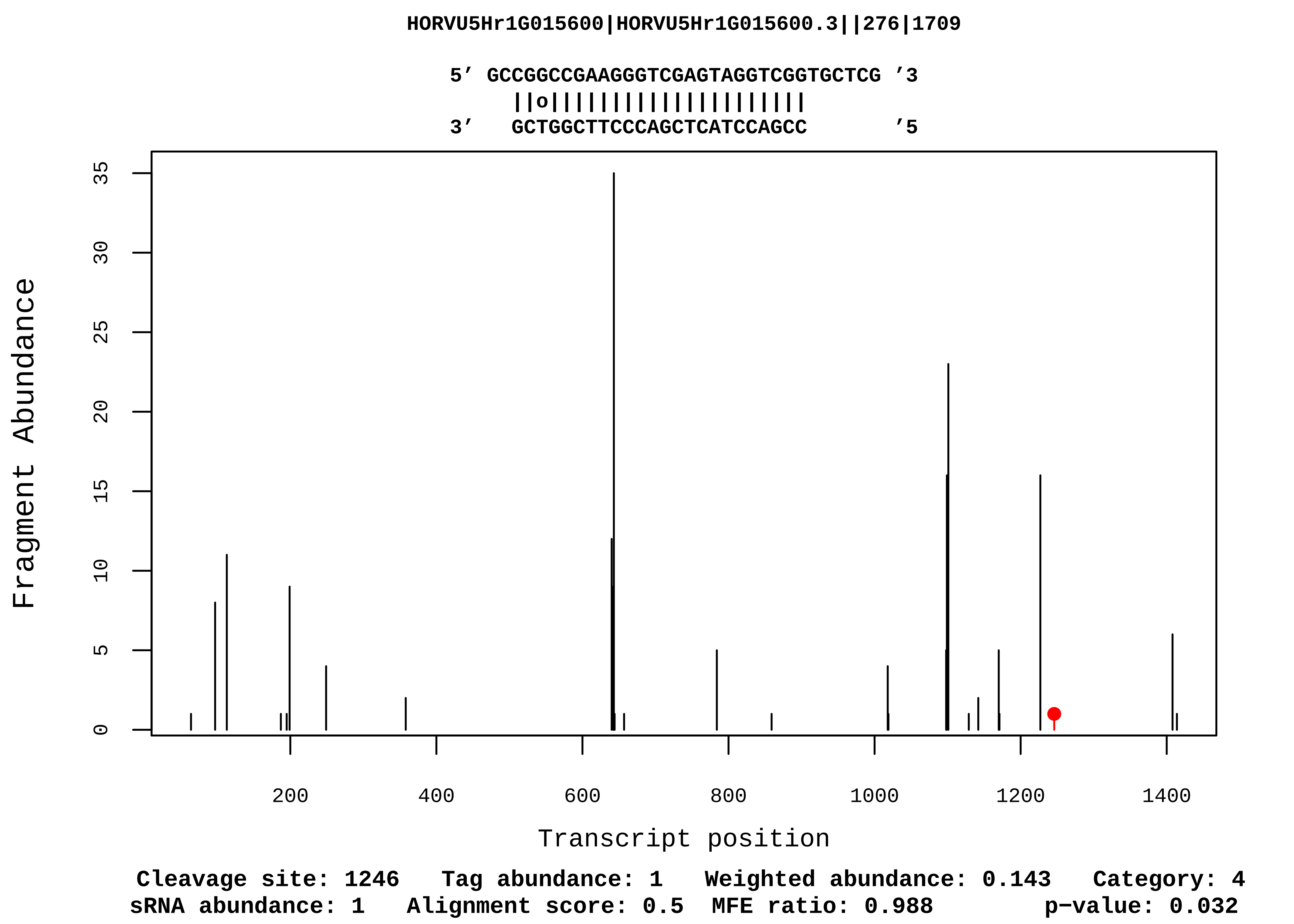
HORVU5Hr1G015600|HORVU5Hr1G015600.3||276|1709
5′ GCCGGCCGAAGGGTCGAGTAGGTCGGTGCTCG ′3
||o||||||||||||||||||||
3′ GCTGGCTTCCCAGCTCATCCAGCC ′5
Fragment Abundance
0
5
10
15
20
25
30
35
200
400
600
800
1000
1200
1400
Transcript position
Cleavage site: 1246 Tag abundance: 1 Weighted abundance: 0.143 Category: 4
sRNA abundance: 1 Alignment score: 0.5 MFE ratio: 0.988 p-value: 0.032

HORVU5Hr1G015600|HORVU5Hr1G015600.4||207|1852

```
5' GCCGGCCGCAGGGTCGAGTAGGTCGGTGCTCG '3
      ||o||| ||||||||||||||||
3'    GCTGGCTTCCCAGCTCATCCAGCC        '5
```

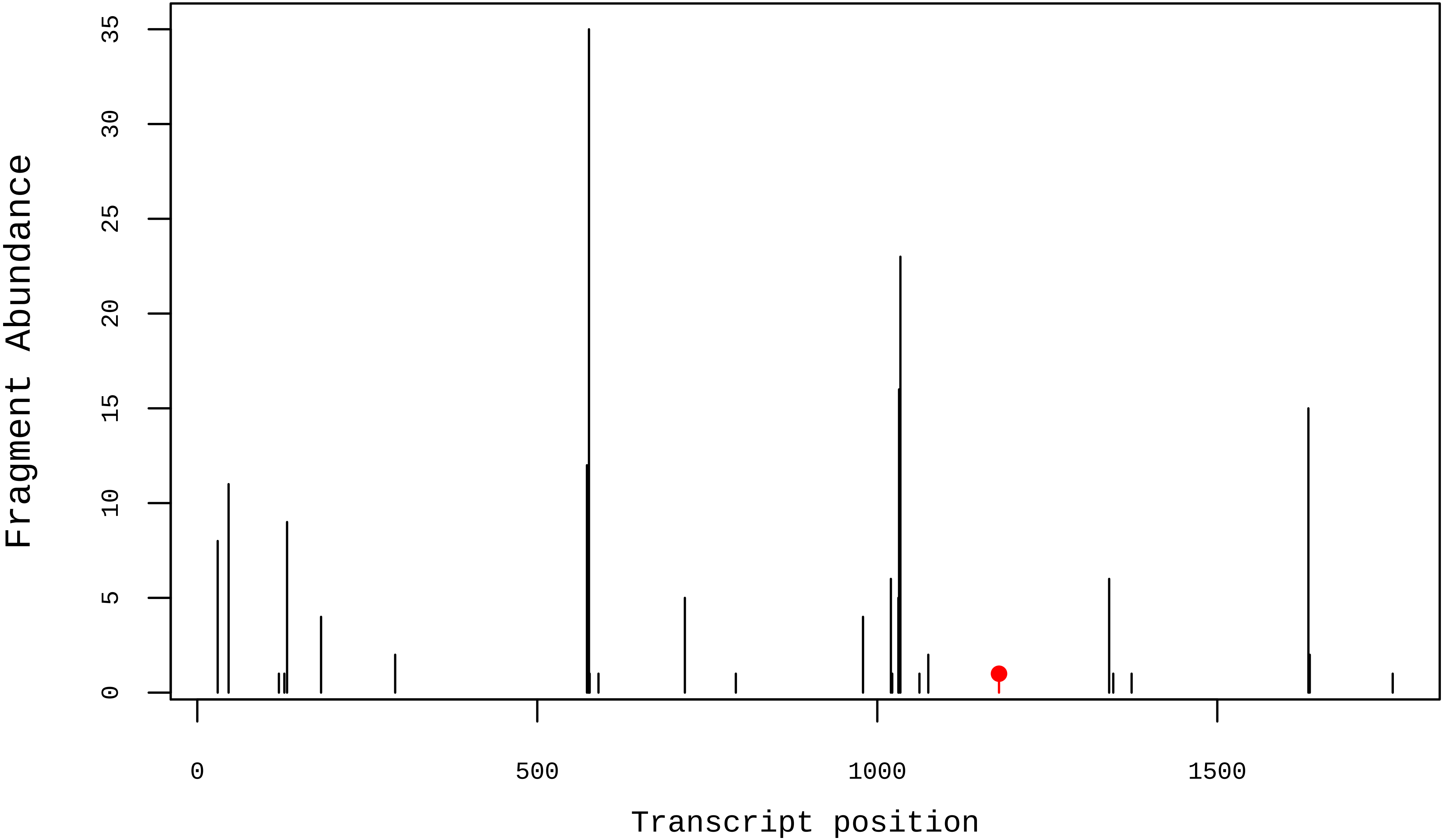

Cleavage site: 1179   Tag abundance: 1   Weighted abundance: 0.143   Category: 4
sRNA abundance: 1   Alignment score: 1.5   MFE ratio: 0.901   p-value: 0.041

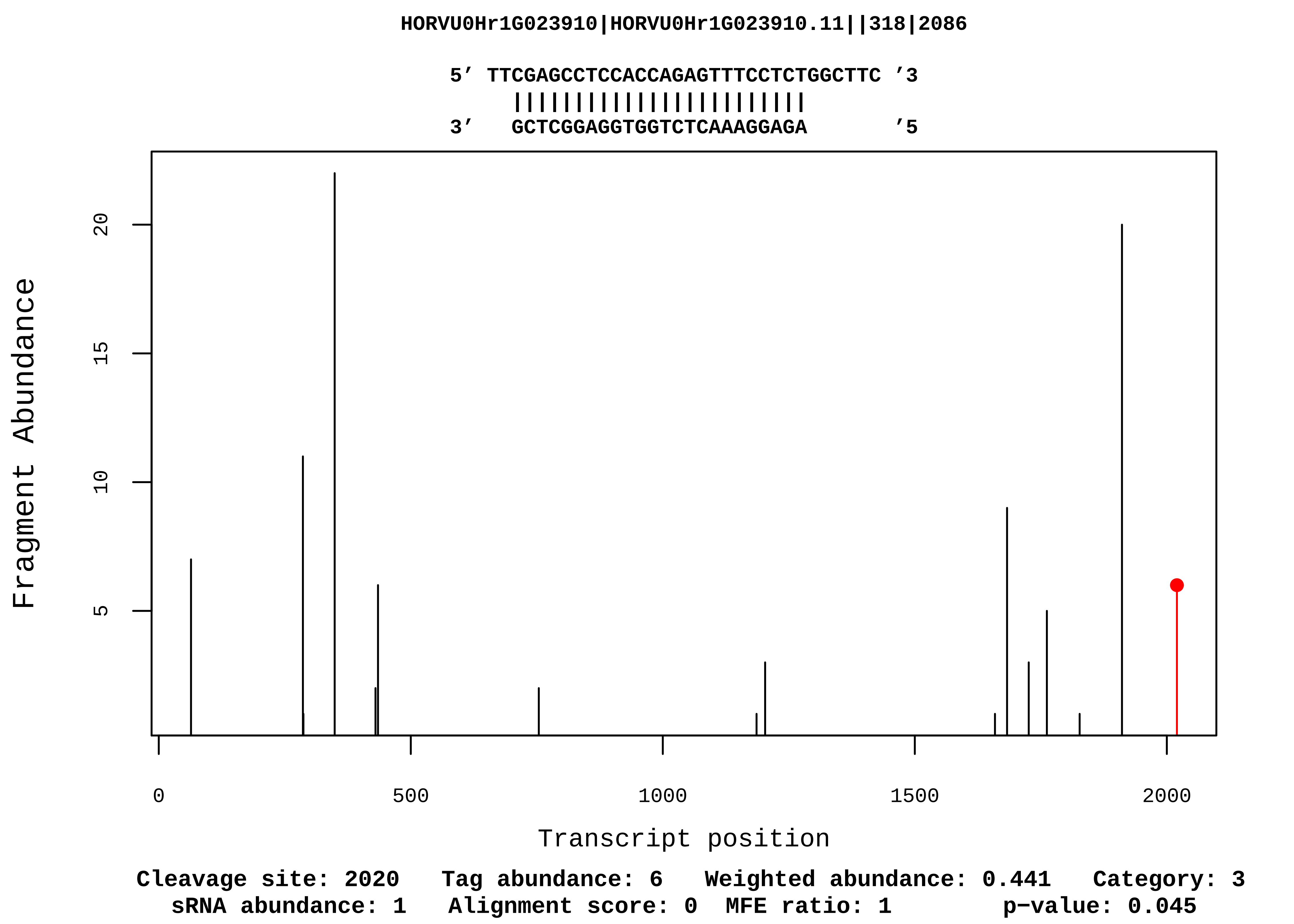
HORVU0Hr1G023910|HORVU0Hr1G023910.11||318|2086
5′ TTCGAGCCTCCACCAGAGTTTCCTCTGGCTTC ′3
||||||||||||||||||||||||
3′ GCTCGGAGGTGGTCTCAAAGGAGA ′5
Fragment Abundance
5
10
15
20
0
500
1000
1500
2000
Transcript position
Cleavage site: 2020 Tag abundance: 6 Weighted abundance: 0.441 Category: 3
sRNA abundance: 1 Alignment score: 0 MFE ratio: 1 p-value: 0.045

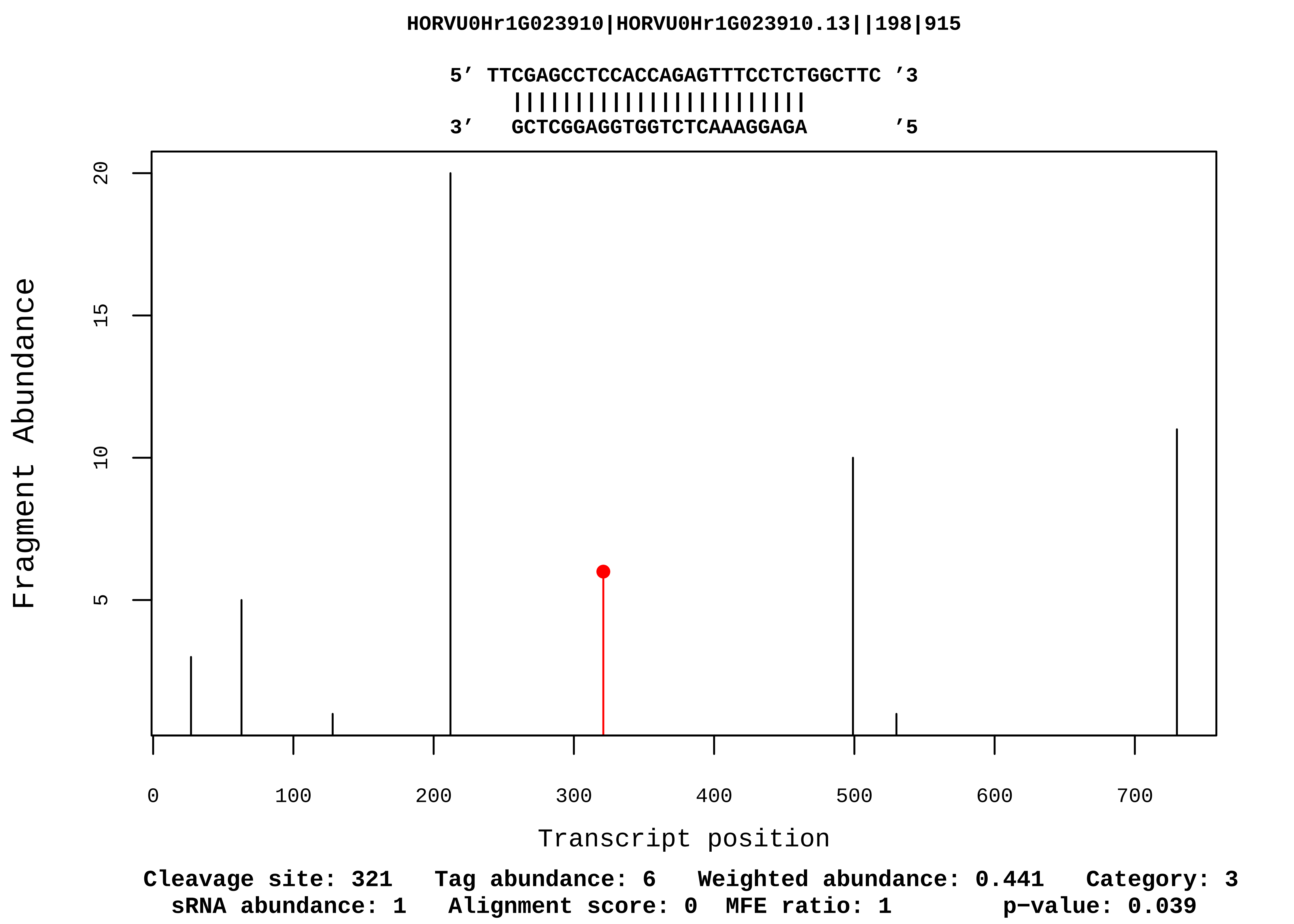
HORVU0Hr1G023910|HORVU0Hr1G023910.13||198|915
5' TTCGAGCCTCCACCAGAGTTTCCTCTGGCTTC '3
3' GCTCGGAGGTGGTCTCAAAGGAGA '5
Fragment Abundance
20
15
10
5
0
100
200
300
400
500
600
700
Transcript position
Cleavage site: 321 Tag abundance: 6 Weighted abundance: 0.441 Category: 3
sRNA abundance: 1 Alignment score: 0 MFE ratio: 1 p-value: 0.039

HORVU0Hr1G023910|HORVU0Hr1G023910.8||318|1667

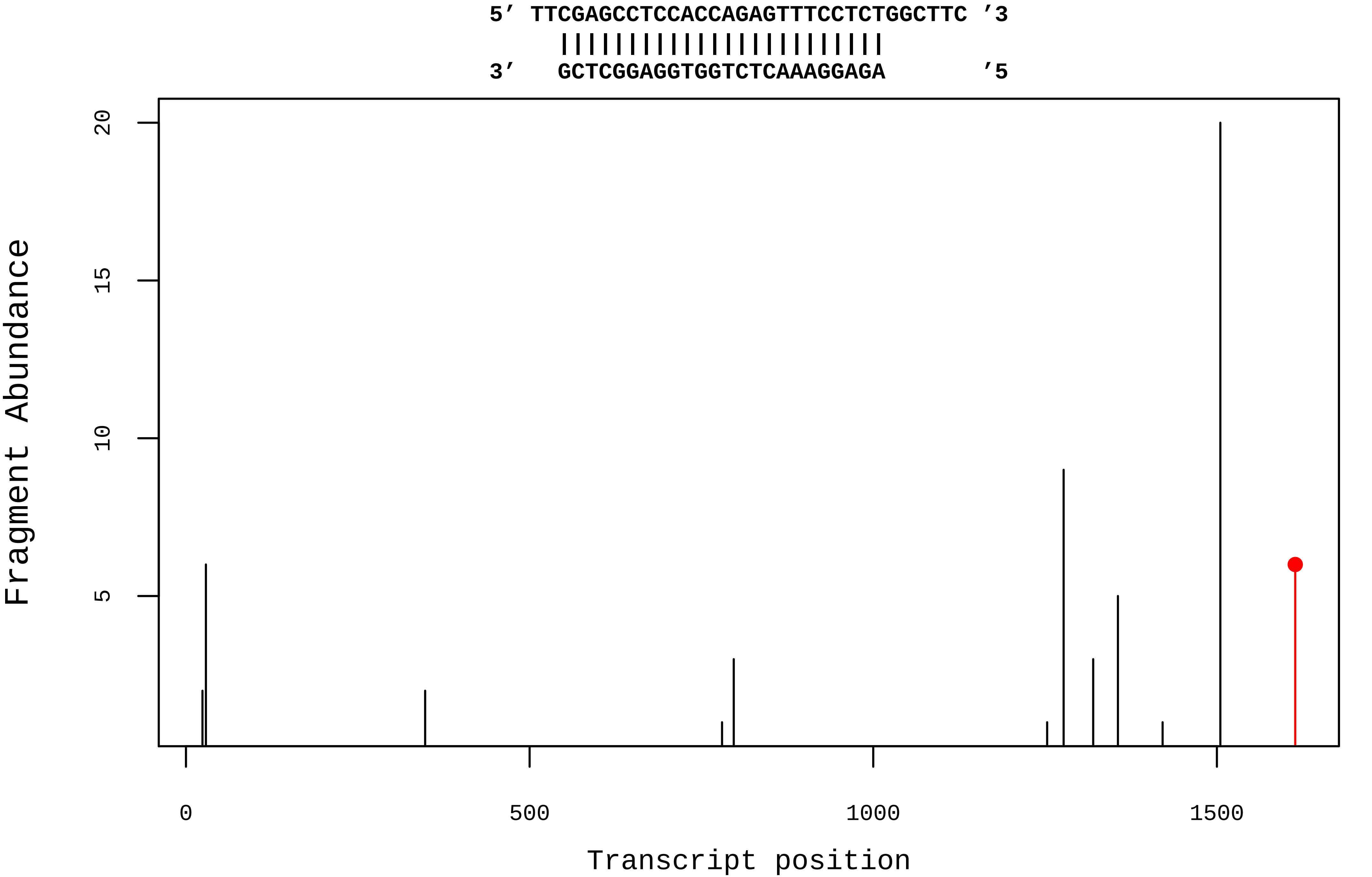

Cleavage site: 1614   Tag abundance: 6   Weighted abundance: 0.441   Category: 3
sRNA abundance: 1   Alignment score: 0   MFE ratio: 1   p-value: 0.05

HORVU1Hr1G027340|HORVU1Hr1G027340.1||246|418

```
5' TTCGAGCCTCCACCAGAGTTTCCTCTGGCTTC '3
      ||||||||||||||||||||||||
3'    GCTCGGAGGTGGTCTCAAAGGAGA       '5
```

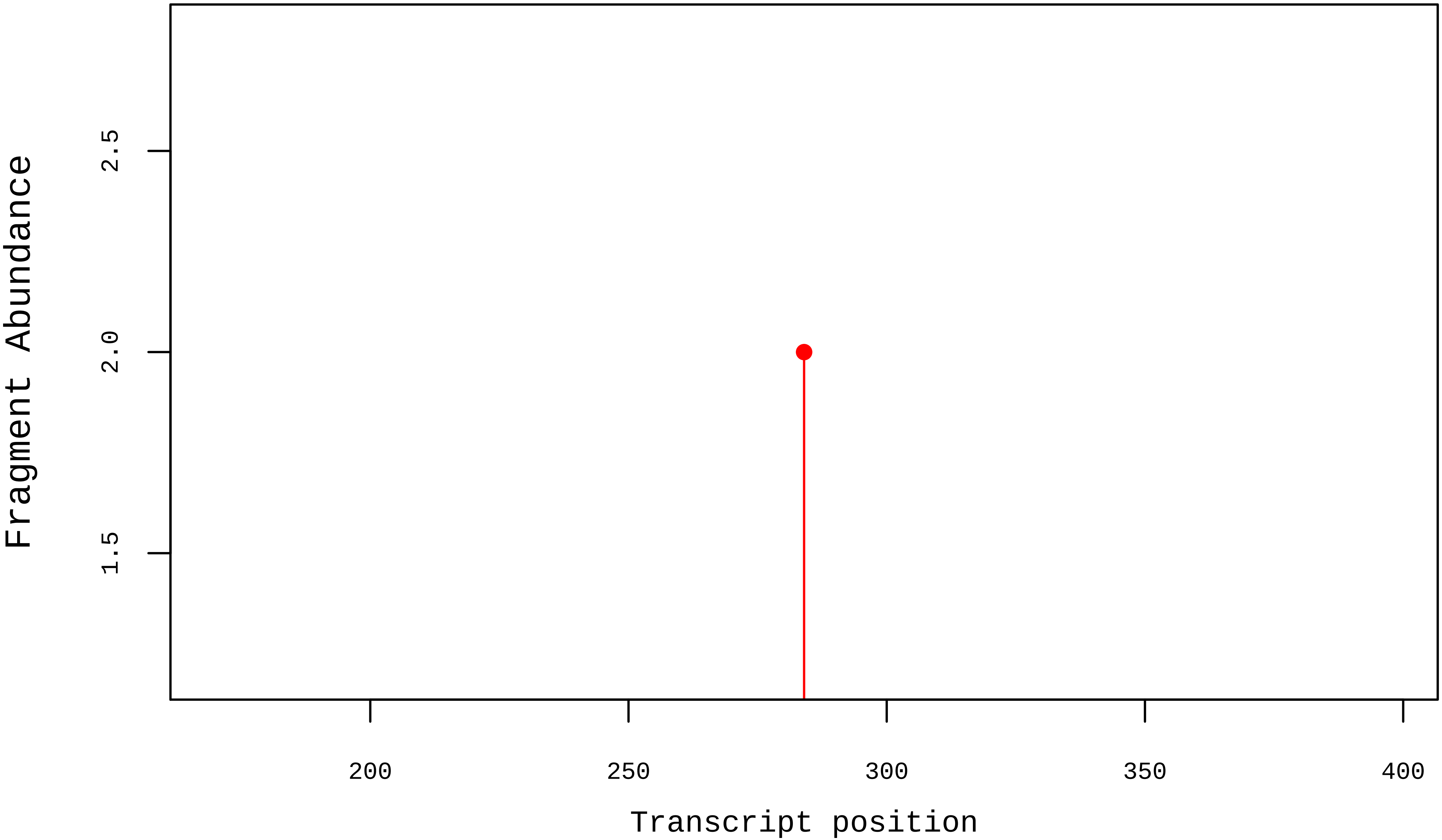

Cleavage site: 284   Tag abundance: 2   Weighted abundance: 0.133   Category: 0
sRNA abundance: 1   Alignment score: 0 MFE ratio: 1   p-value: 0.03

HORVU1Hr1G027340|HORVU1Hr1G027340.2||627|933

```
5' TTCGAGCCTCCACCAGAGTTTCCTCTGGCTTC '3
      |||||||||||||||||||||||
3'    GCTCGGAGGTGGTCTCAAAGGAGA       '5
```

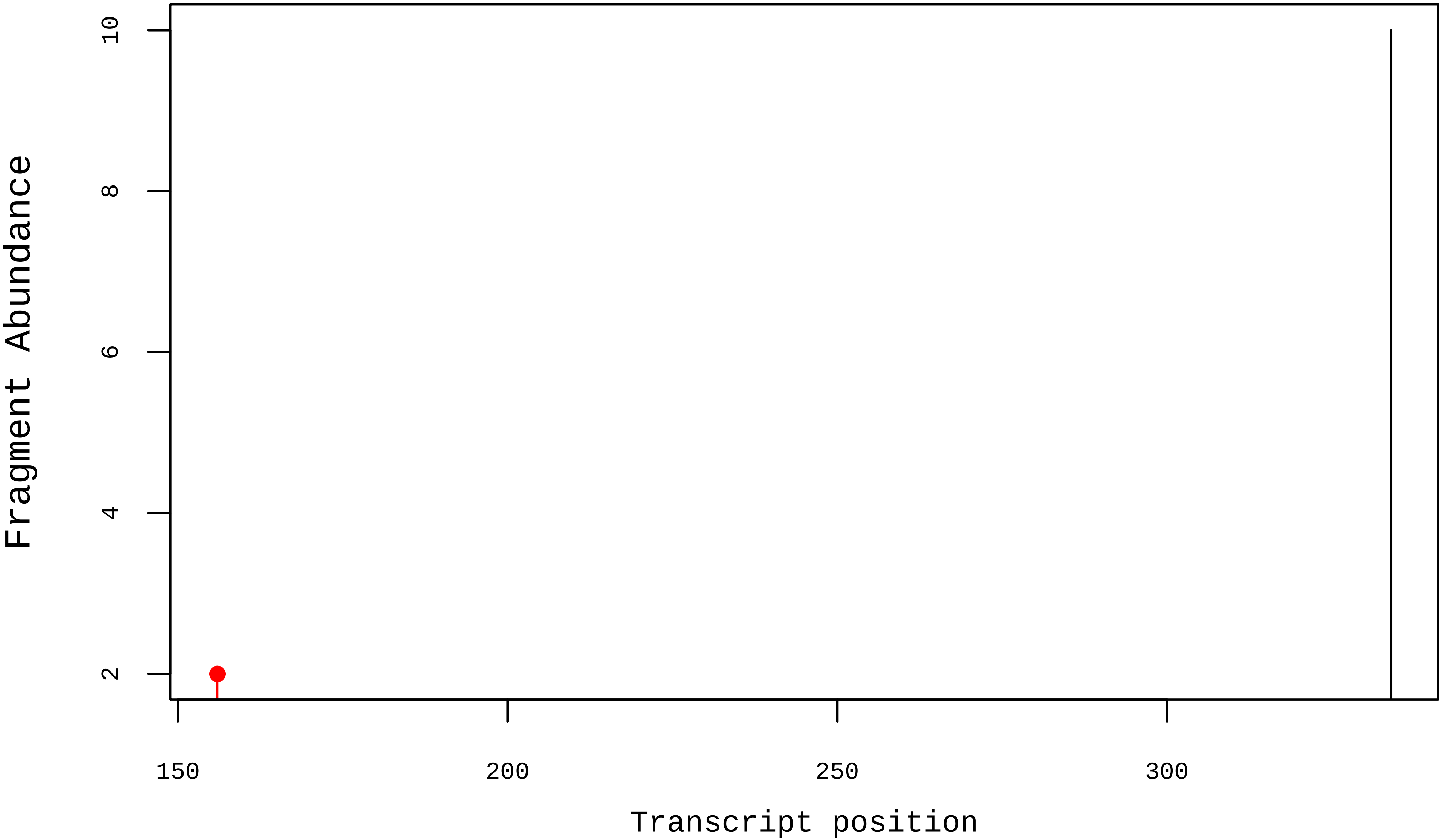

Cleavage site: 156 Tag abundance: 2 Weighted abundance: 0.133 Category: 3
sRNA abundance: 1 Alignment score: 0 MFE ratio: 1 p-value: 0.013

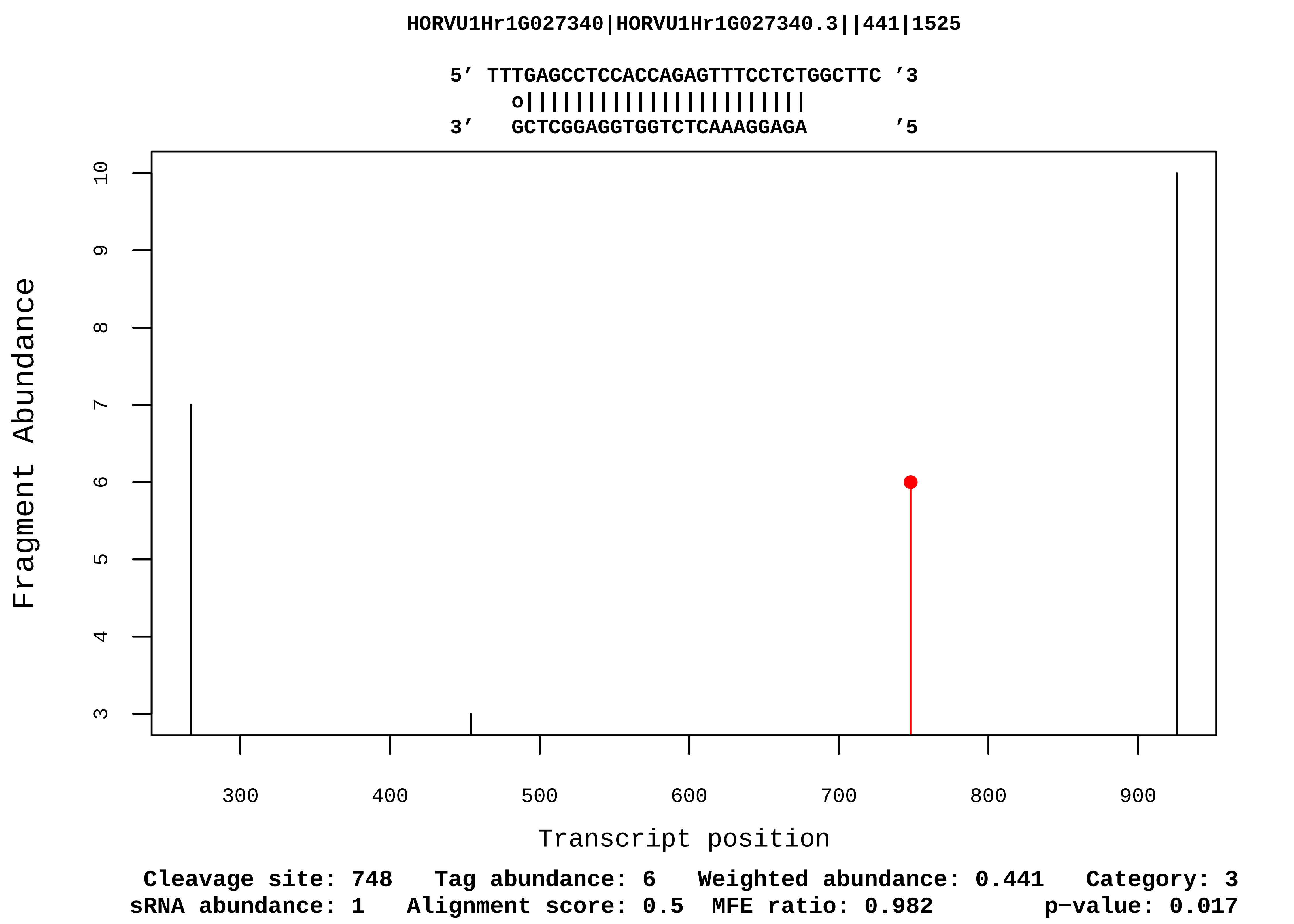
HORVU1Hr1G027340|HORVU1Hr1G027340.3||441|1525
5′ TTTGAGCCTCCACCAGAGTTTCCTCTGGCTTC ′3
o|||||||||||||||||||||||
3′ GCTCGGAGGTGGTCTCAAAGGAGA ′5
Fragment Abundance
3
4
5
6
7
8
9
10
300
400
500
600
700
800
900
Transcript position
Cleavage site: 748 Tag abundance: 6 Weighted abundance: 0.441 Category: 3
sRNA abundance: 1 Alignment score: 0.5 MFE ratio: 0.982 p-value: 0.017

HORVU7Hr1G053570|HORVU7Hr1G053570.1||378|1160

```
5' TTCGAGCCTCCACCAGAGTTTCCTCTGGCTTC '3
      ||||||||||||||||||||||||
3'    GCTCGGAGGTGGTCTCAAAGGAGA       '5
```

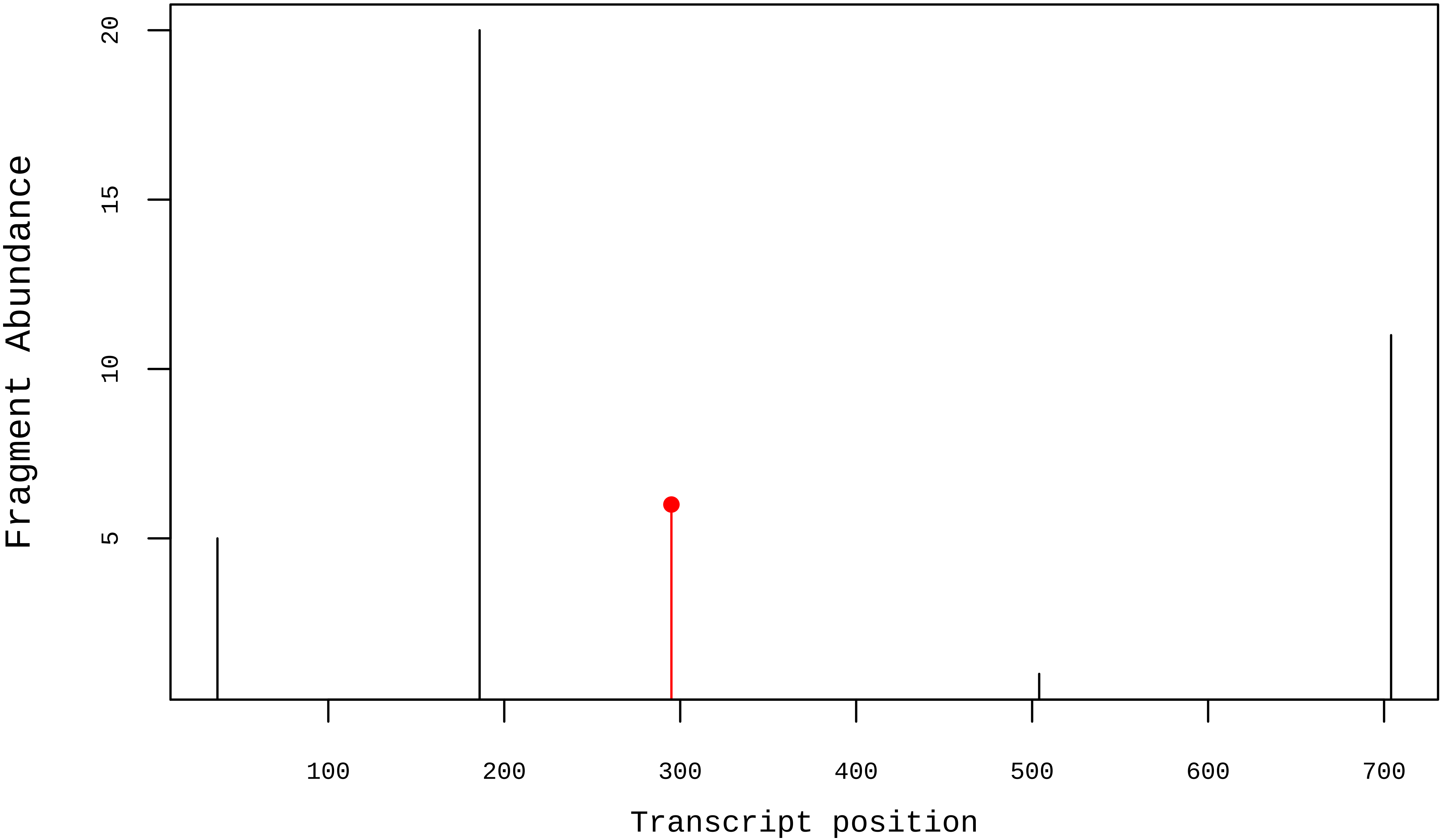

Cleavage site: 295   Tag abundance: 6   Weighted abundance: 0.441   Category: 3
sRNA abundance: 1   Alignment score: 0   MFE ratio: 1   p-value: 0.021

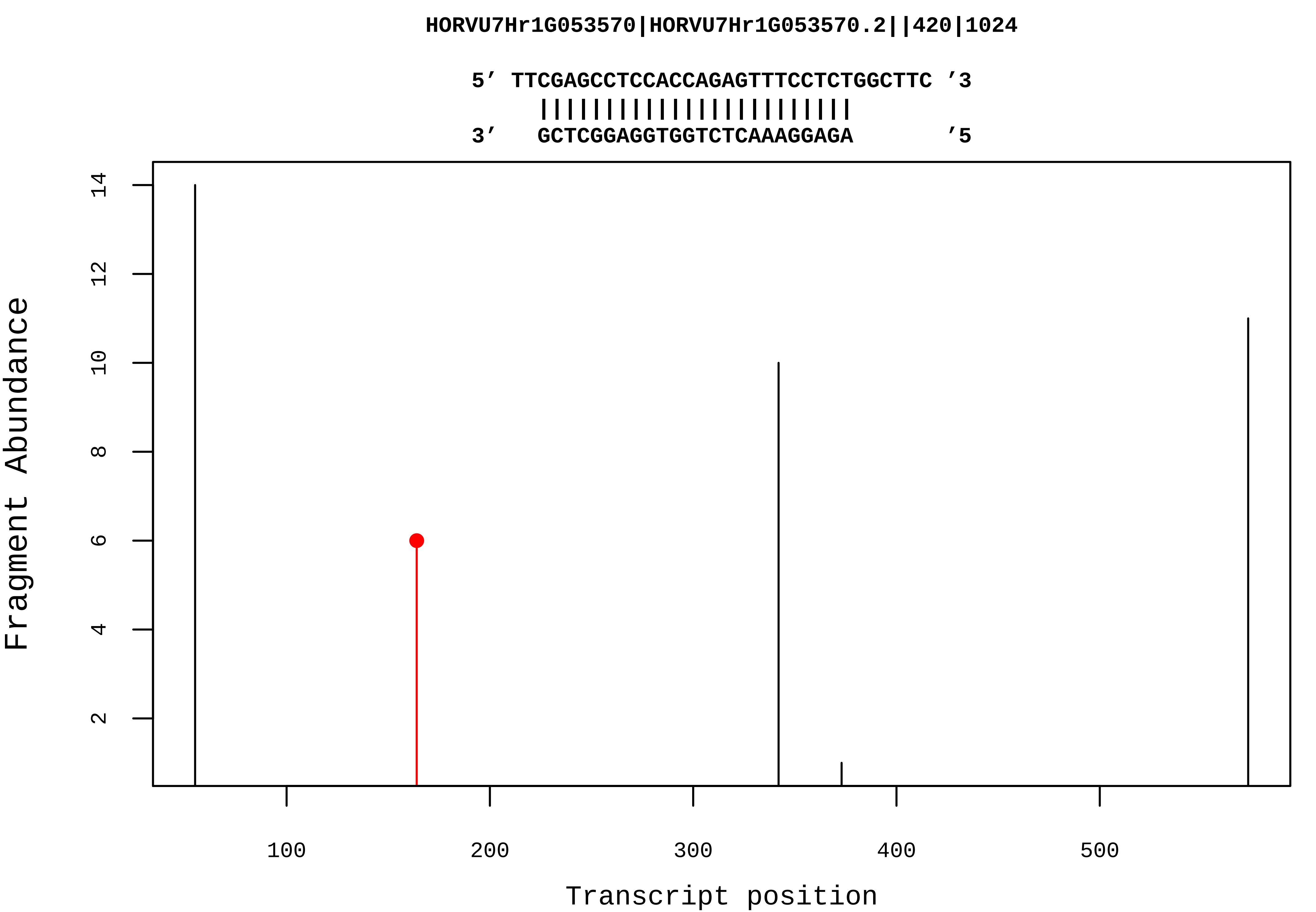

Cleavage site: 164 Tag abundance: 6 Weighted abundance: 0.441 Category: 3
sRNA abundance: 1 Alignment score: 0 MFE ratio: 1 p-value: 0.024

HORVU7Hr1G053570|HORVU7Hr1G053570.3||420|1292

```
5' TTCGAGCCTCCACCAGAGTTTCCTCTGGCTTC '3
      ||||||||||||||||||||||||
3'    GCTCGGAGGTGGTCTCAAAGGAGA       '5
```

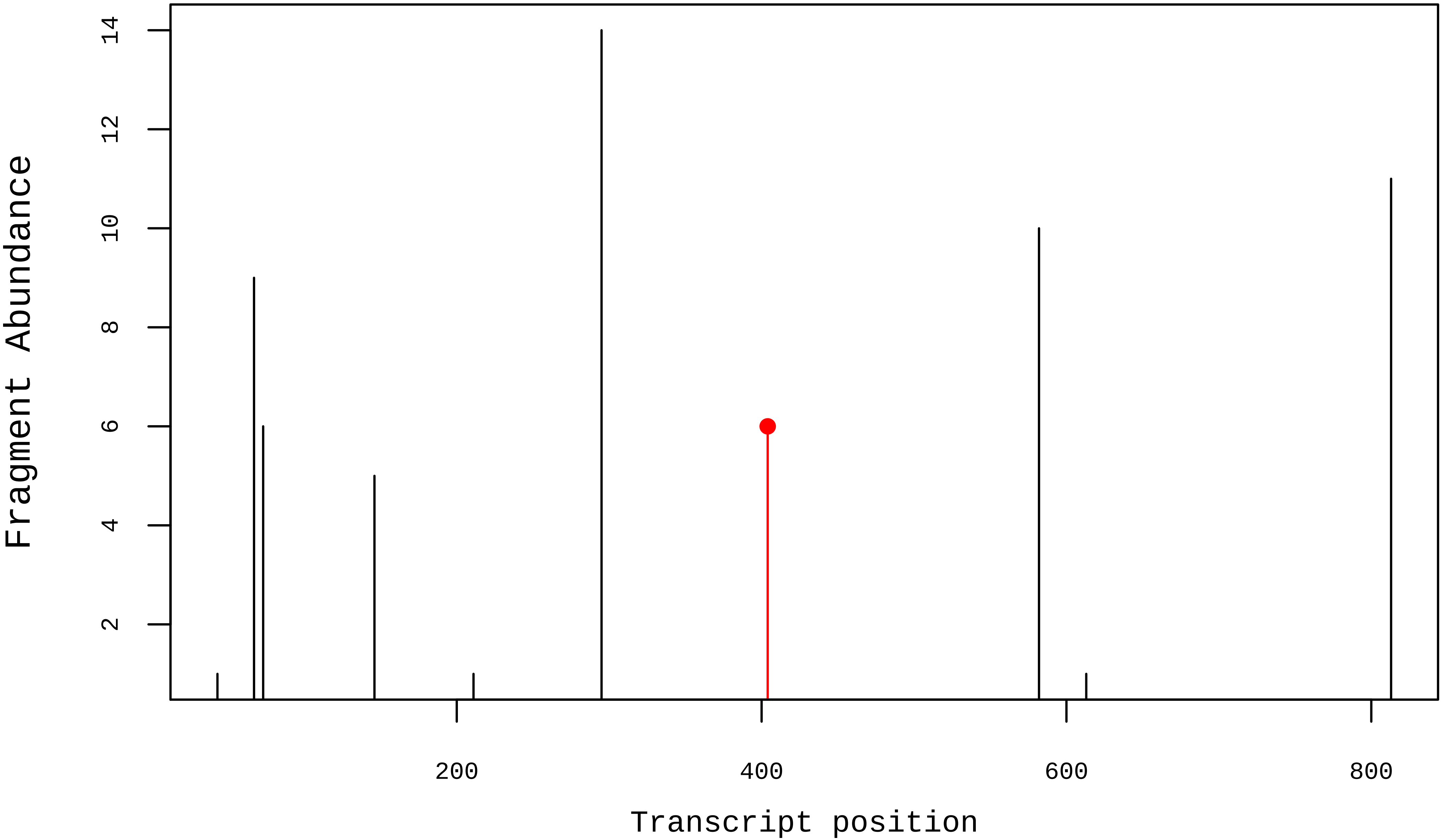

Cleavage site: 404   Tag abundance: 6   Weighted abundance: 0.441   Category: 3
sRNA abundance: 1   Alignment score: 0   MFE ratio: 1   p-value: 0.028

HORVU0Hr1G023930|HORVU0Hr1G023930.3||396|2180

```
5' GTCGGCGGAAGGGTCGAGTAGGTCGGTGCTCG '3
          ||| |||||||||||||||||
3'        GCCGTCCCAGCTCATCCAGCC       '5
```

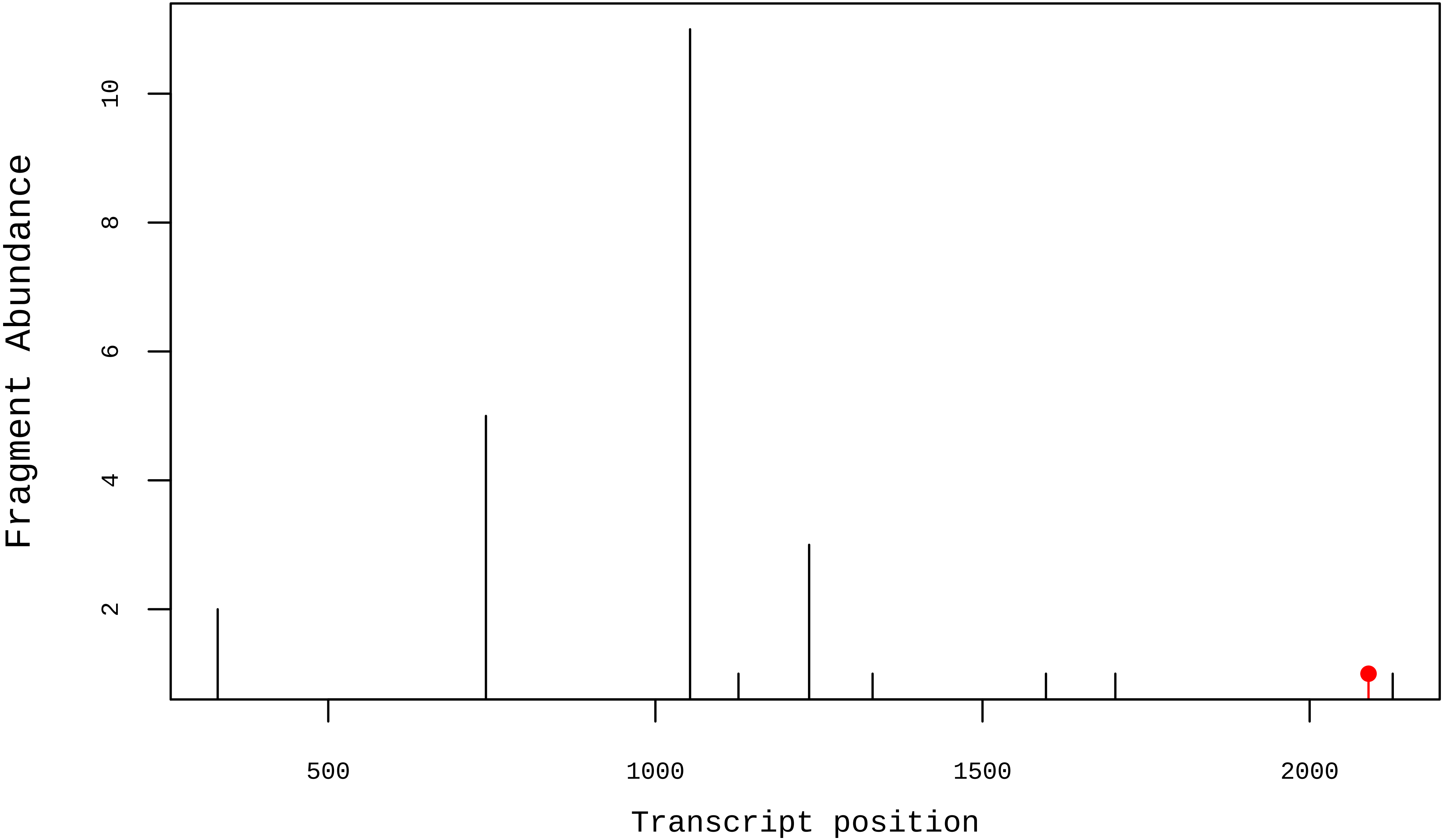

Cleavage site: 2090   Tag abundance: 1   Weighted abundance: 0.143   Category: 4
sRNA abundance: 1   Alignment score: 1   MFE ratio: 0.856   p-value: 0.006

HORVU2Hr1G072960|HORVU2Hr1G072960.1||600|3353

```
5' GCCGGCCGA-AGGGTCGAGTAGGTCGGTGCTCG '3
           ||  ||||||||||||||||
3'         GCCGTCCCAGCTCATCCAGCC       '5
```

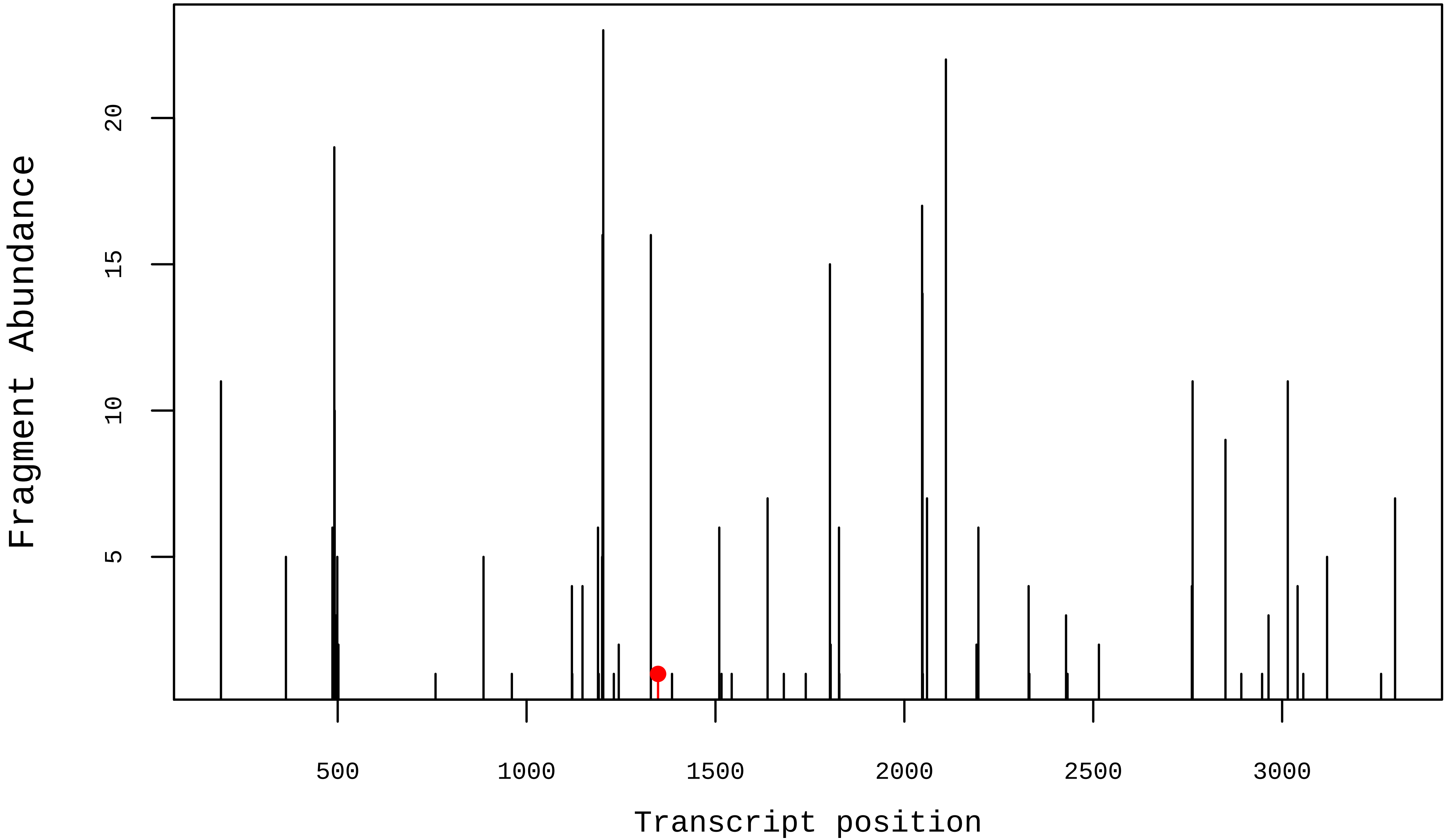

Cleavage site: 1348 Tag abundance: 1 Weighted abundance: 0.143 Category: 4
sRNA abundance: 1 Alignment score: 2 MFE ratio: 0.772 p-value: 0.041

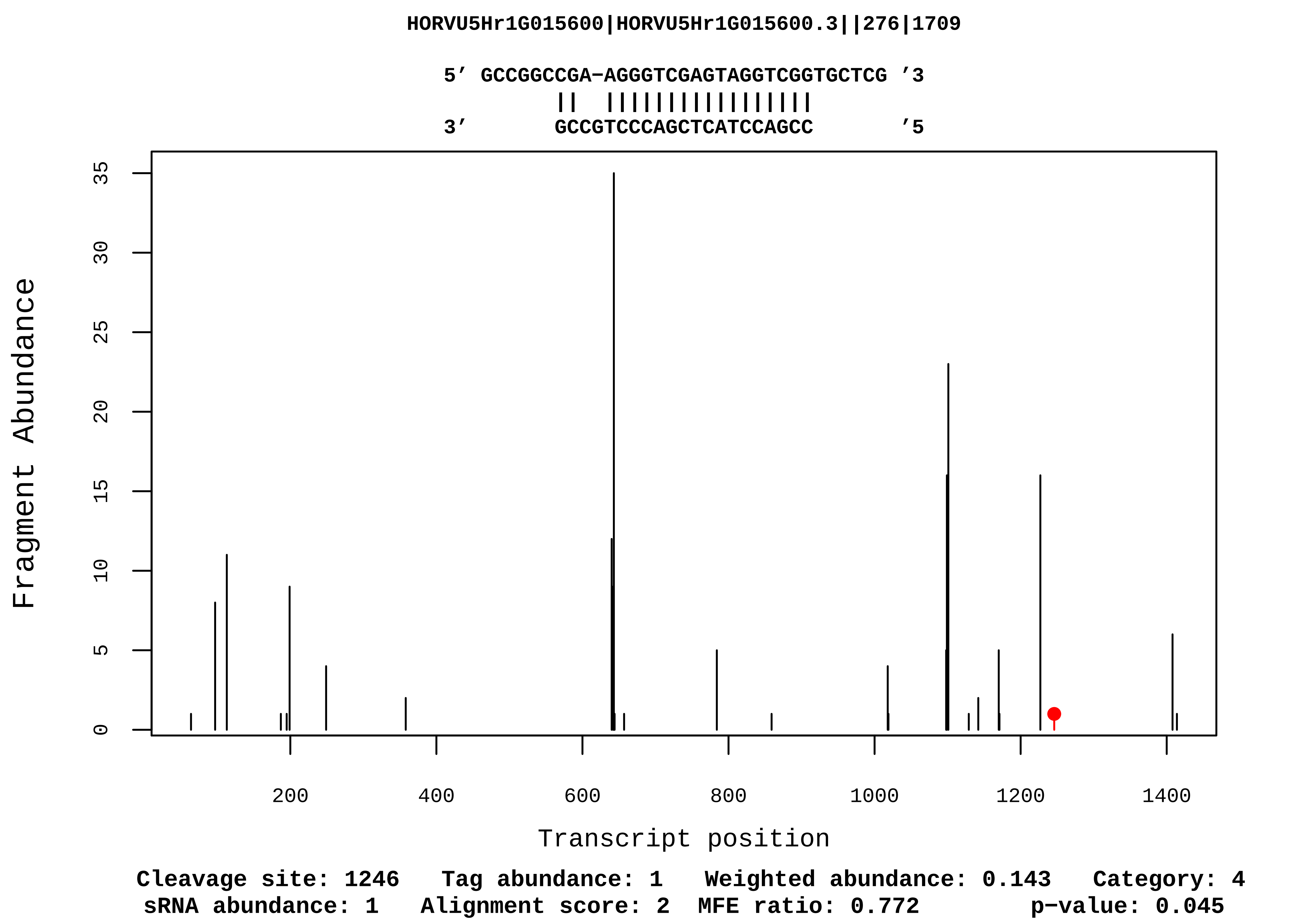
HORVU5Hr1G015600|HORVU5Hr1G015600.3||276|1709
5′ GCCGGCCGA−AGGGTCGAGTAGGTCGGTGCTCG ′3
3′ GCCGTCCCAGCTCATCCAGCC ′5
Fragment Abundance
0
5
10
15
20
25
30
35
200
400
600
800
1000
1200
1400
Transcript position
Cleavage site: 1246 Tag abundance: 1 Weighted abundance: 0.143 Category: 4
sRNA abundance: 1 Alignment score: 2 MFE ratio: 0.772 p−value: 0.045

HORVU5Hr1G015600|HORVU5Hr1G015600.4||207|1852

```
5' GCCGGCCGCAGGGTCGAGTAGGTCGGTGCTCG '3
          | ||||||||||||||||||
3'        GCCGTCCCAGCTCATCCAGCC       '5
```

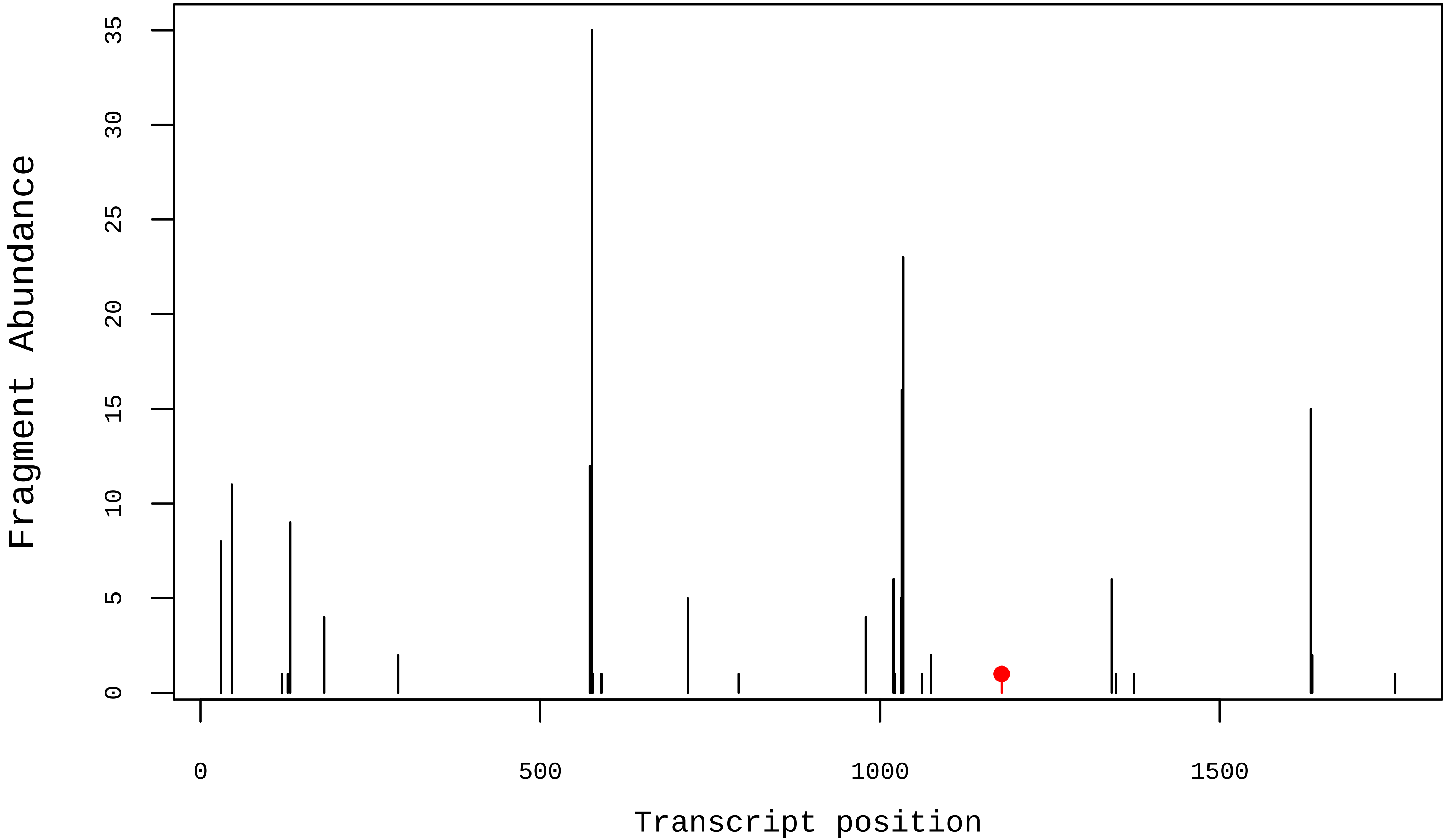

Cleavage site: 1179   Tag abundance: 1   Weighted abundance: 0.143   Category: 4
sRNA abundance: 1   Alignment score: 1   MFE ratio: 0.901   p-value: 0.012

HORVU0Hr1G023910|HORVU0Hr1G023910.11||318|2086

```
5' CACAATGTCTTCTGCCCGGATCGGCCCGATAA '3
     ||||||||||||o|||||||||||||
3'  TGTTACAGAAGGCGGGCCTAGCCGG       '5
```

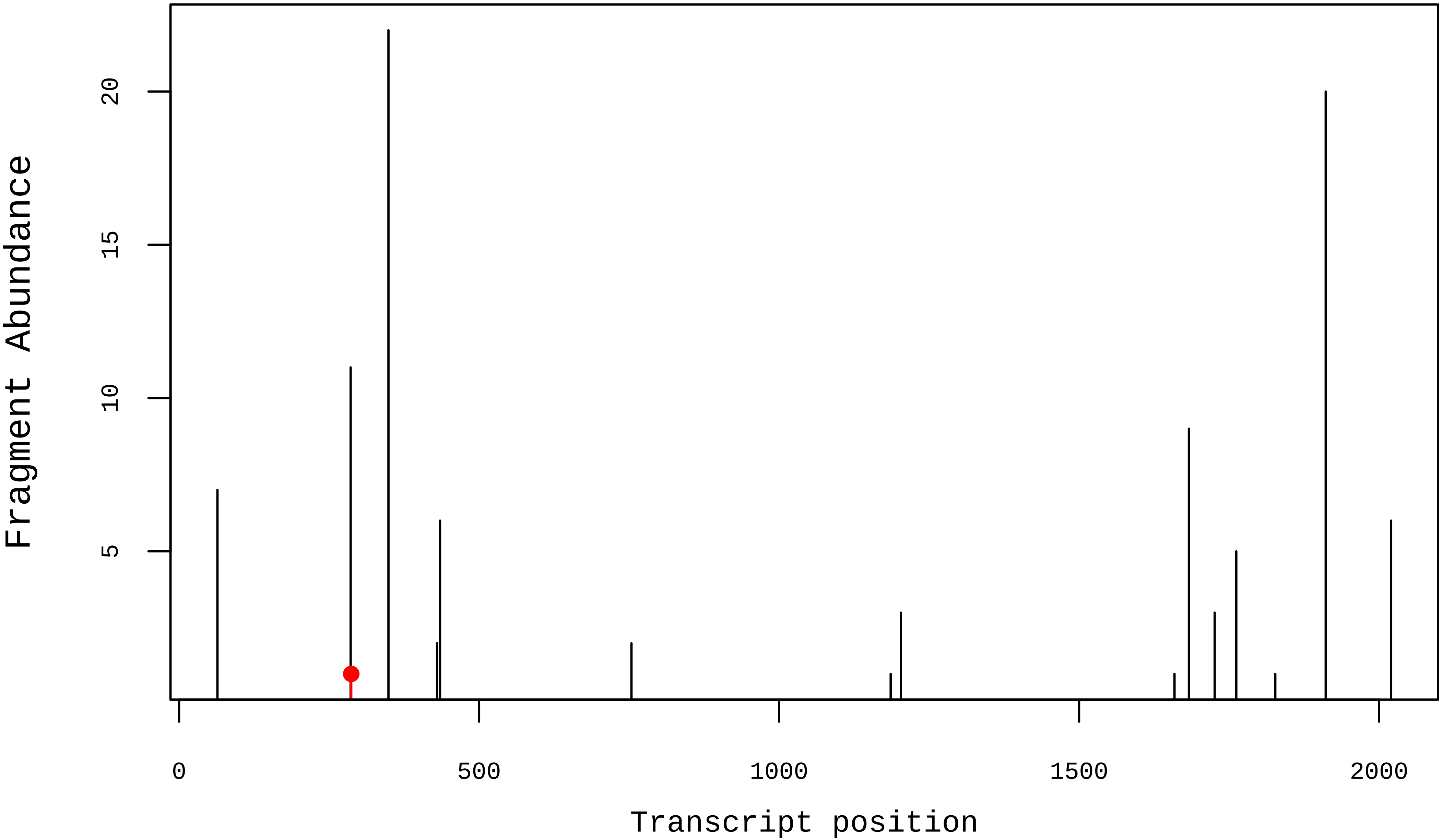

Cleavage site: 287 Tag abundance: 1 Weighted abundance: 0.043 Category: 4
sRNA abundance: 1 Alignment score: 0.5 MFE ratio: 0.959 p-value: 0.034

HORVU0Hr1G035470|HORVU0Hr1G035470.1||384|764

```
5' GACAATGTCTTCCGCCCGGATCGGCCCGATAA '3
    |||||||||||||||||||||||||
3'  TGTTACAGAAGGCGGGCCTAGCCGG       '5
```

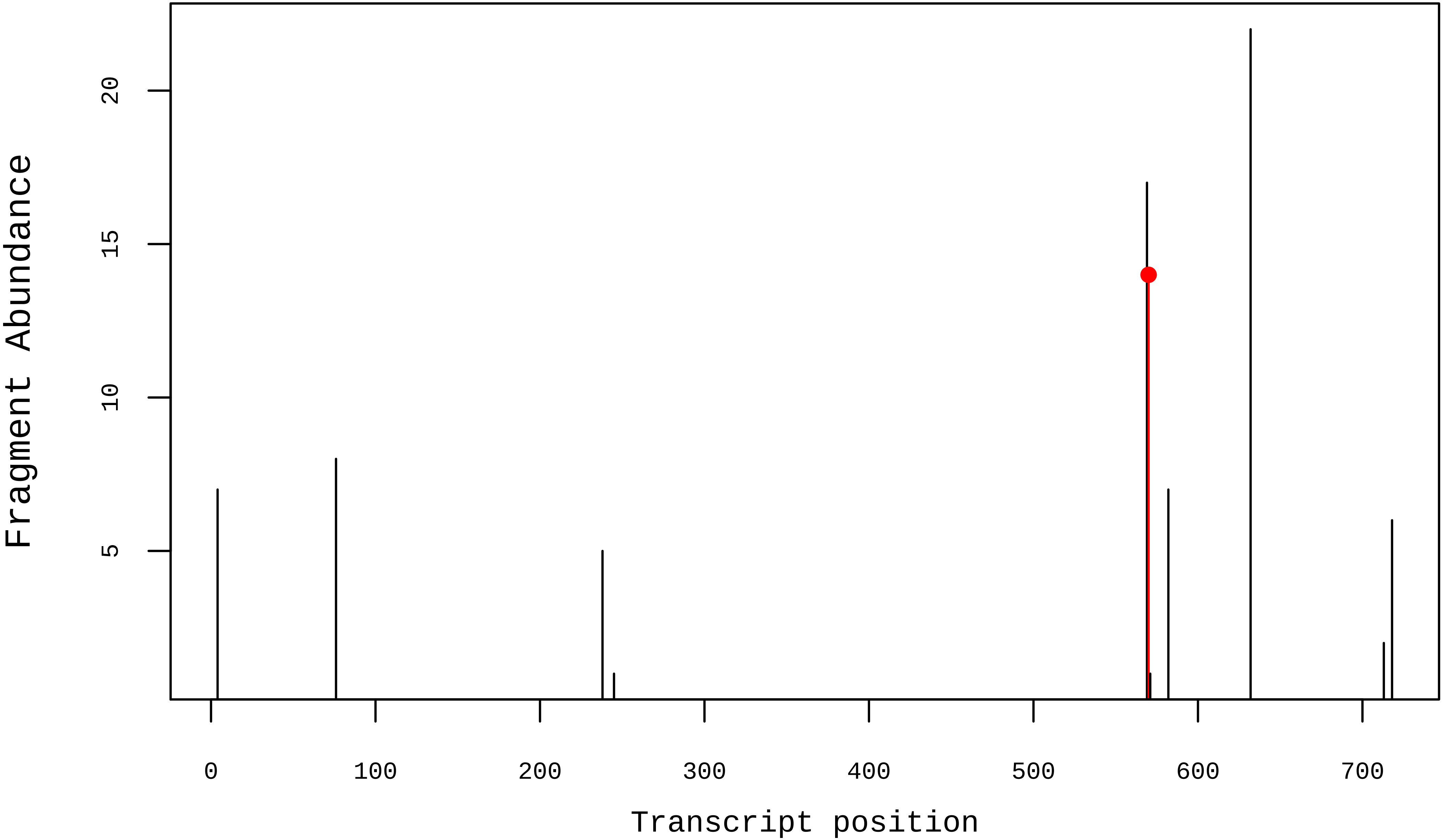

Cleavage site: 570   Tag abundance: 14   Weighted abundance: 0.808   Category: 2
sRNA abundance: 1   Alignment score: 0   MFE ratio: 1   p-value: 0.037

HORVU1Hr1G026920|HORVU1Hr1G026920.1||195|1105

```
5' GAAAATGTCTTCCGCCCGGATCGGCCCGATAA '3
     | ||||||||||||||||||||||||
3'   TGTTACAGAAGGCGGGCCTAGCCGG       '5
```

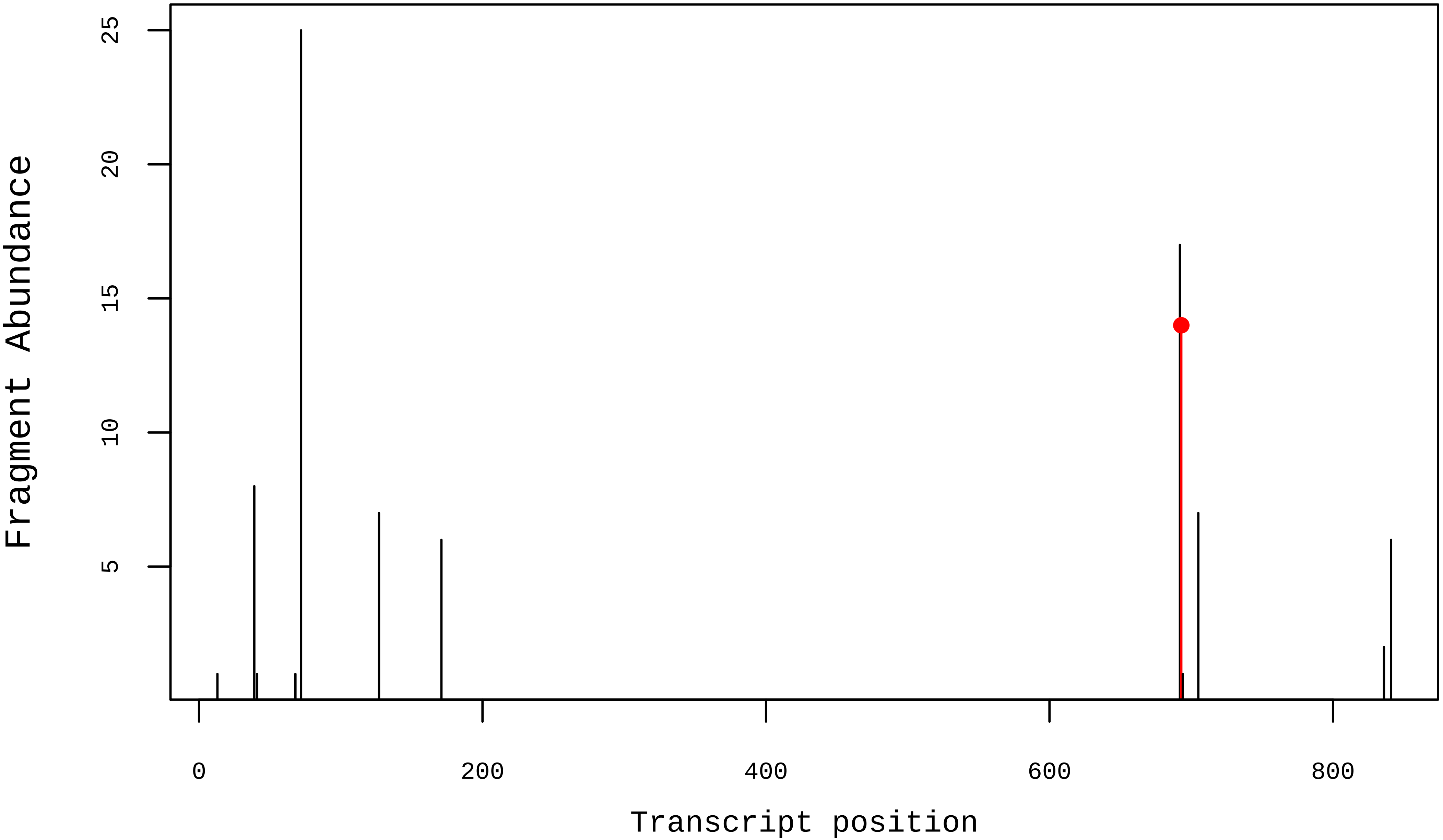

Cleavage site: 693 Tag abundance: 14 Weighted abundance: 0.808 Category: 2
sRNA abundance: 1 Alignment score: 1 MFE ratio: 0.94 p-value: 0.042

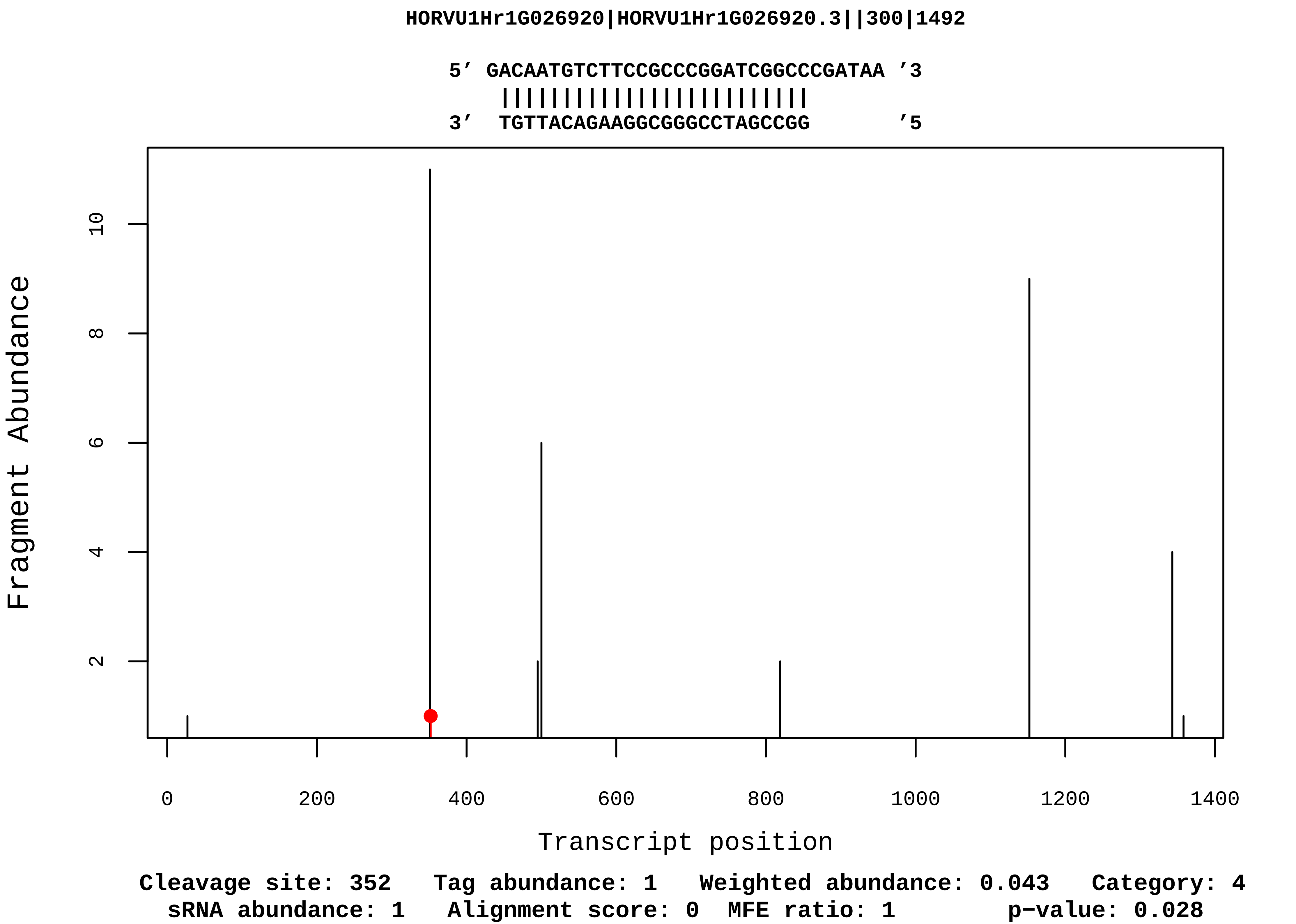

HORVU1Hr1G026920|HORVU1Hr1G026920.3||300|1492
5' GACAATGTCTTCCGCCCGGATCGGCCCGATAA '3
||||||||||||||||||||||||
3' TGTTACAGAAGGCGGGCCTAGCCGG '5
Fragment Abundance
2
4
6
8
10
0
200
400
600
800
1000
1200
1400
Transcript position
Cleavage site: 352 Tag abundance: 1 Weighted abundance: 0.043 Category: 4
sRNA abundance: 1 Alignment score: 0 MFE ratio: 1 p-value: 0.028

HORVU1Hr1G026920|HORVU1Hr1G026920.4||300|2051

```
5' GACAATGTCTTCCGCCCGGATCGGCCCGATAA '3
     ||||||||||||||||||||||||
3'  TGTTACAGAAGGCGGGCCTAGCCGG       '5
```

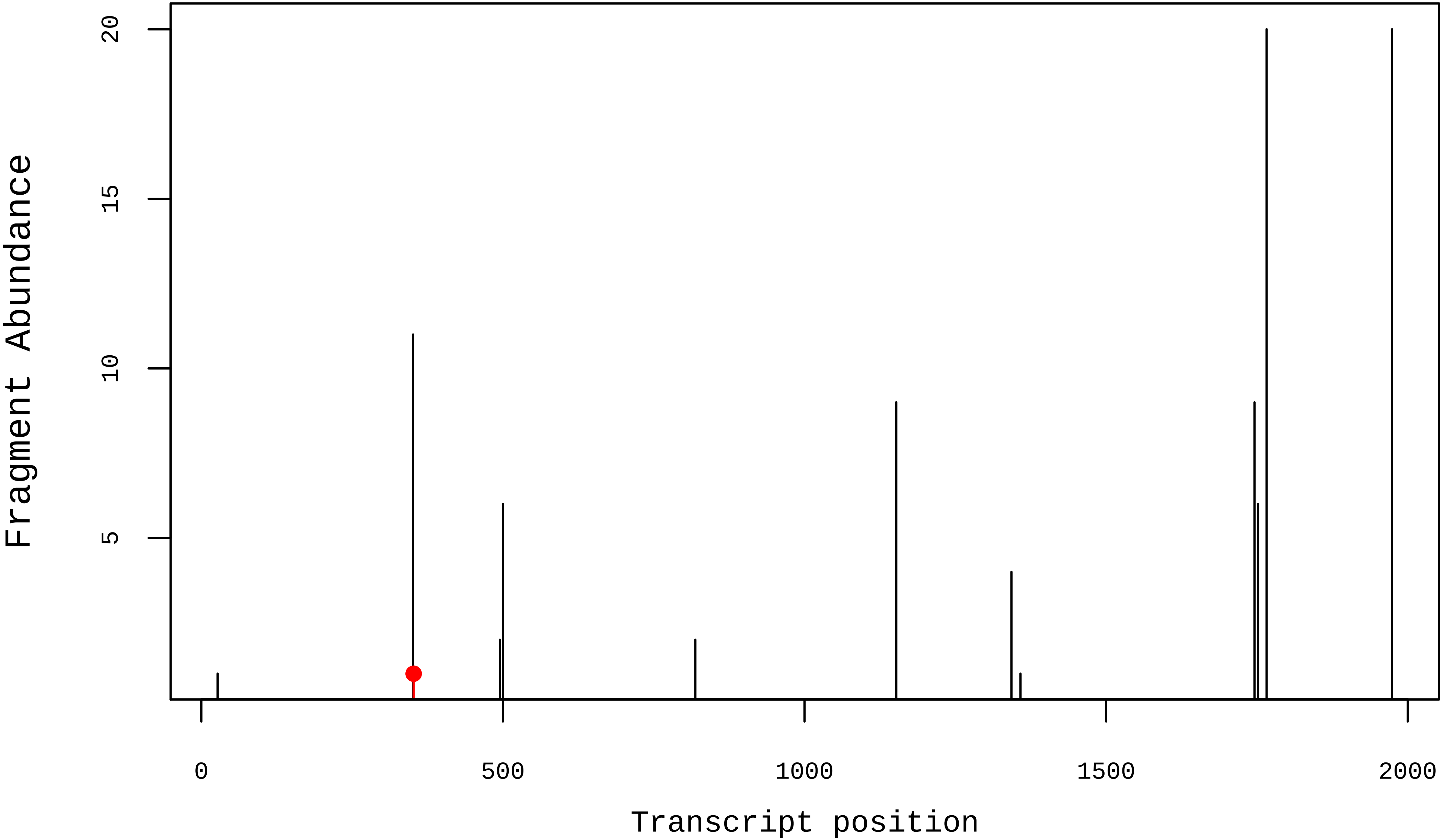

Cleavage site: 352   Tag abundance: 1   Weighted abundance: 0.043   Category: 4
sRNA abundance: 1   Alignment score: 0   MFE ratio: 1   p-value: 0.02

HORVU1Hr1G026920|HORVU1Hr1G026920.5||300|2039

```
5' GACAATGTCTTCCGCCCGGATCGGCCCGATAA '3
     ||||||||||||||||||||||||
3'  TGTTACAGAAGGCGGGCCTAGCCGG       '5
```

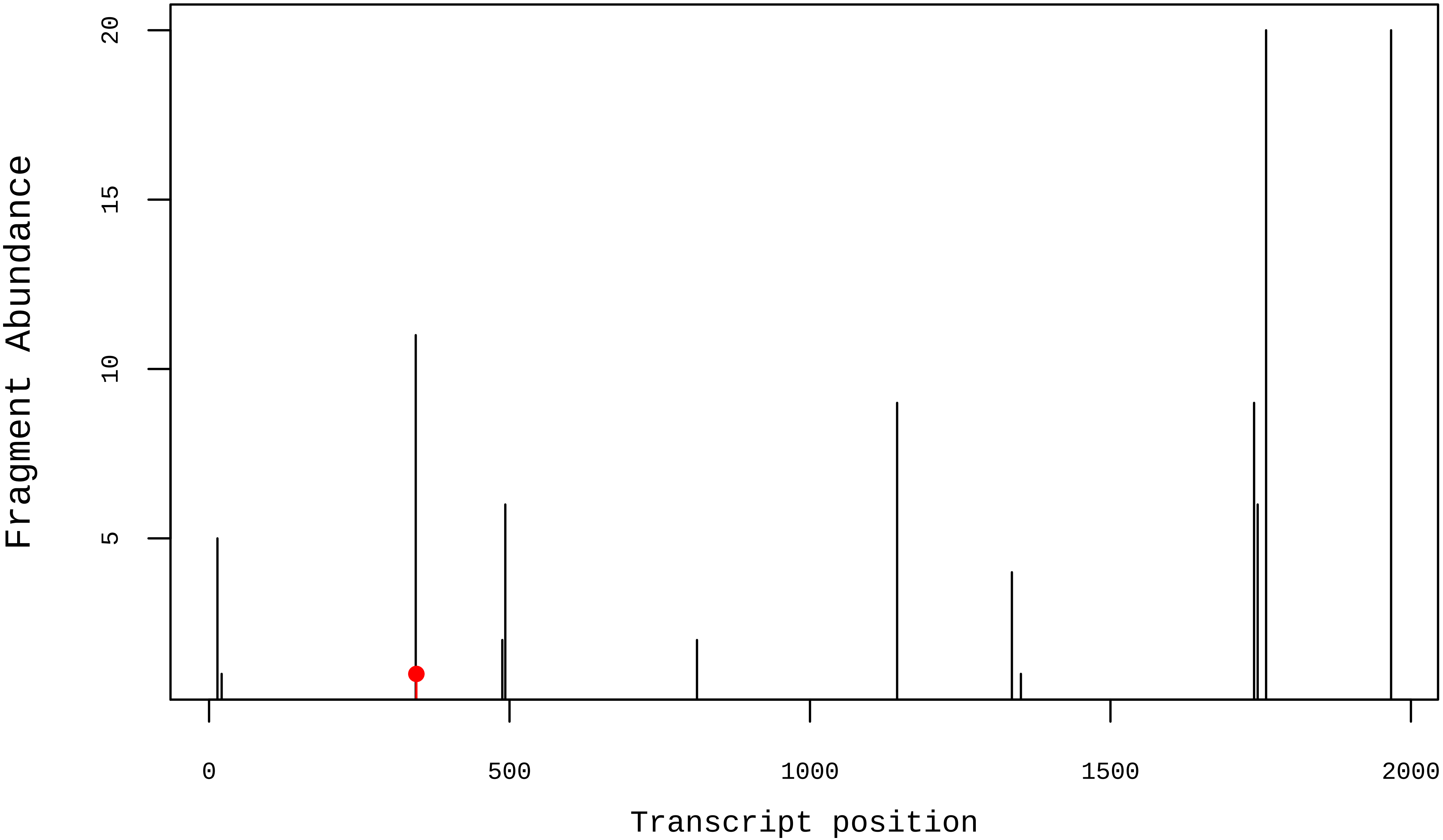

Cleavage site: 345 Tag abundance: 1 Weighted abundance: 0.043 Category: 4
sRNA abundance: 1 Alignment score: 0 MFE ratio: 1 p-value: 0.021

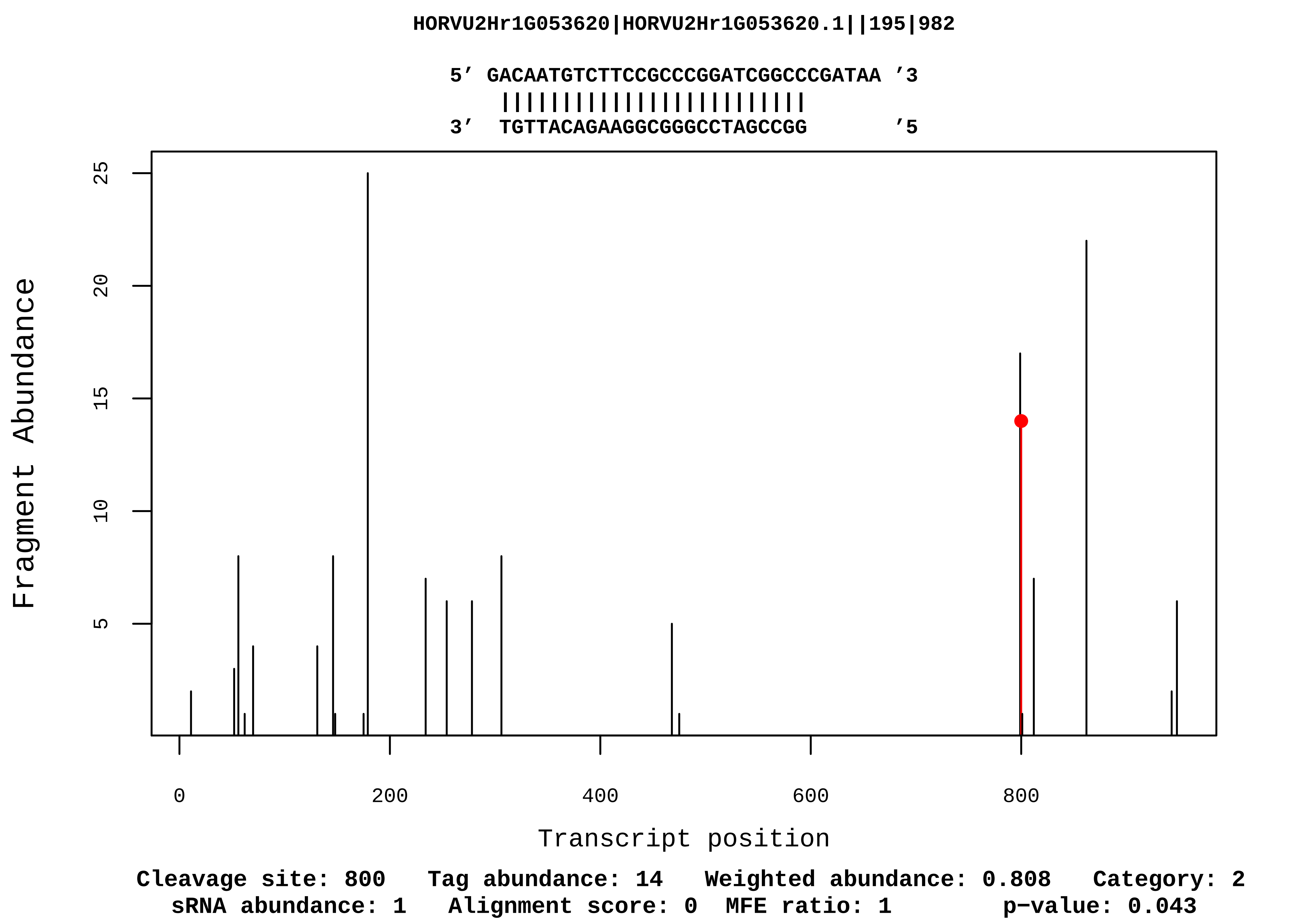
HORVU2Hr1G053620|HORVU2Hr1G053620.1||195|982
5′ GACAATGTCTTCCGCCCGGATCGGCCCGATAA ′3
||||||||||||||||||||||||
3′ TGTTACAGAAGGCGGGCCTAGCCGG ′5
Fragment Abundance
Transcript position
0
200
400
600
800
5
10
15
20
25
Cleavage site: 800 Tag abundance: 14 Weighted abundance: 0.808 Category: 2
sRNA abundance: 1 Alignment score: 0 MFE ratio: 1 p-value: 0.043

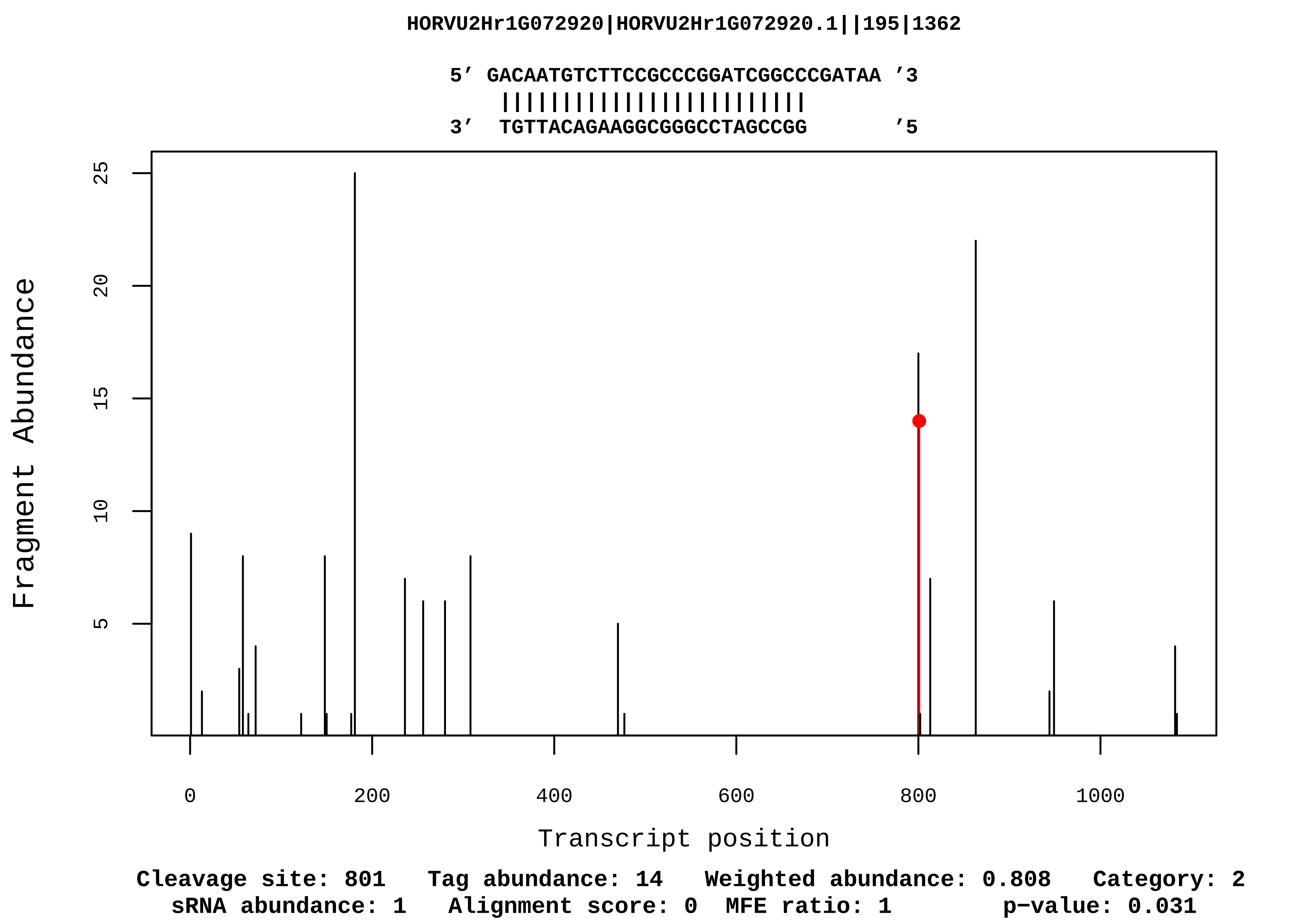

HORVU2Hr1G072920|HORVU2Hr1G072920.1||195|1362
5' GACAATGTCTTCCGCCCGGATCGGCCCGATAA '3
||||||||||||||||||||||||
3' TGTTACAGAAGGCGGGCCTAGCCGG '5
Fragment Abundance
25
20
15
10
5
0
200
400
600
800
1000
Transcript position
Cleavage site: 801 Tag abundance: 14 Weighted abundance: 0.808 Category: 2
sRNA abundance: 1 Alignment score: 0 MFE ratio: 1 p-value: 0.031

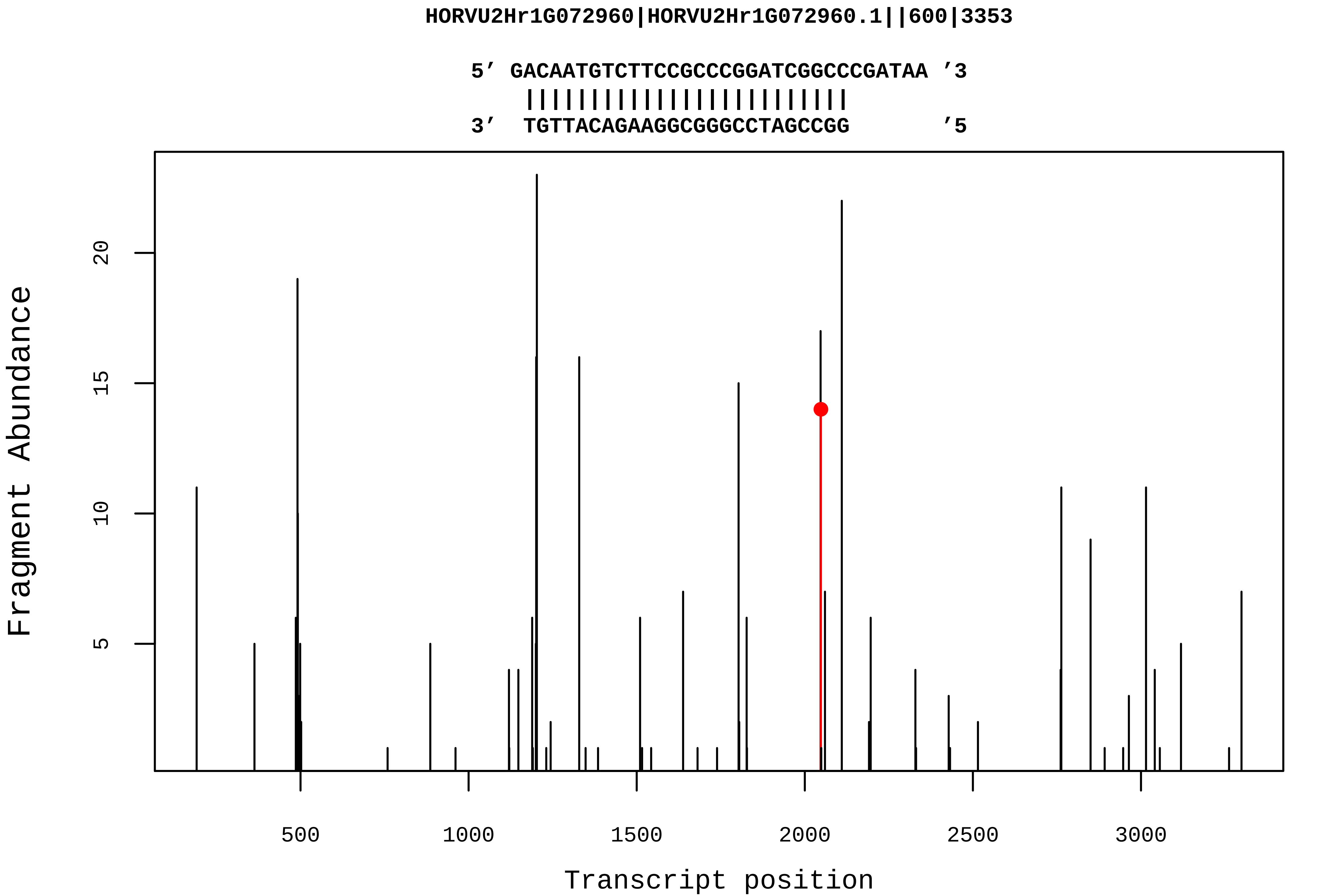

Cleavage site: 2048 Tag abundance: 14 Weighted abundance: 0.808 Category: 2
sRNA abundance: 1 Alignment score: 0 MFE ratio: 1 p-value: 0.049

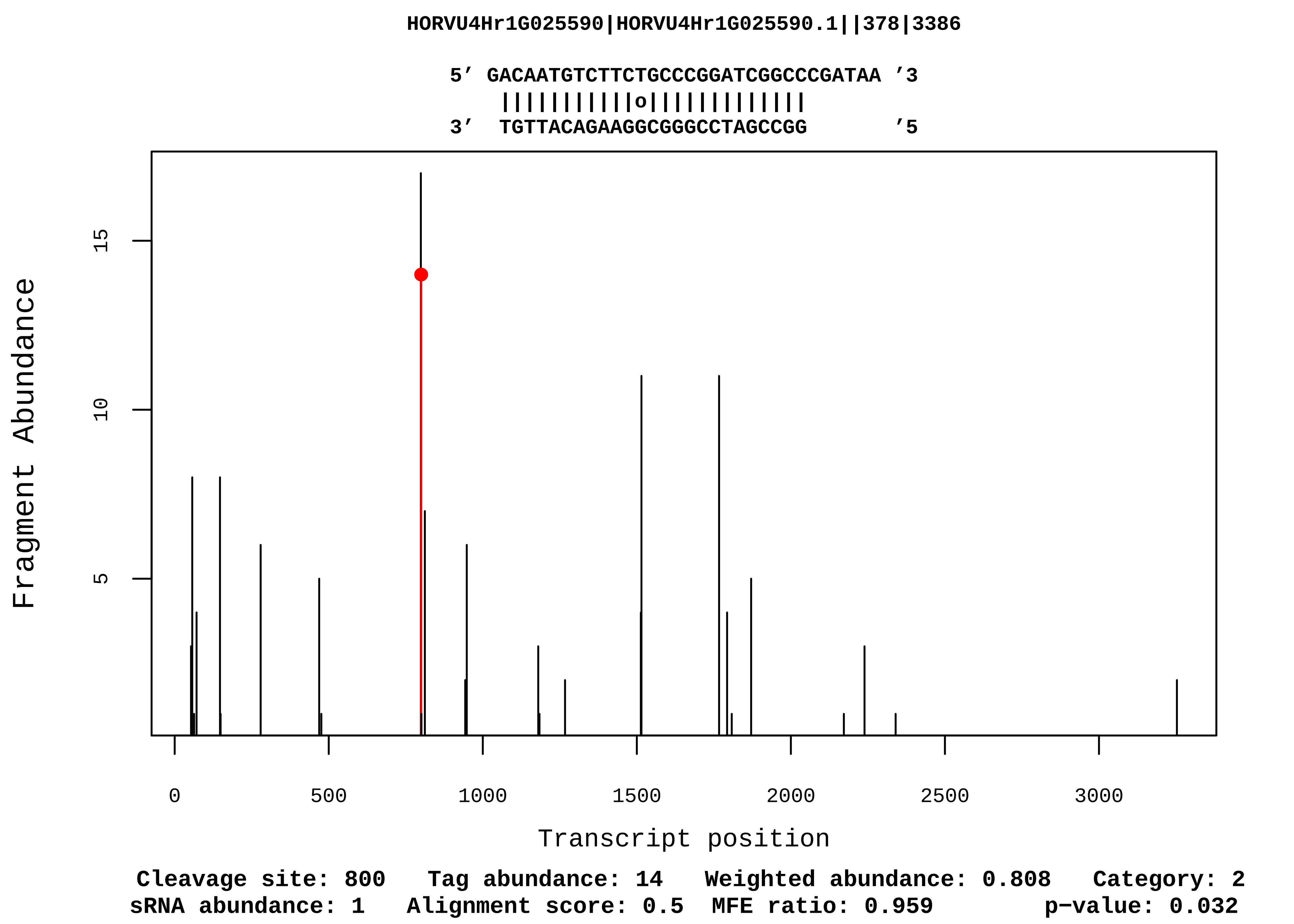

HORVU4Hr1G025590|HORVU4Hr1G025590.1||378|3386
5’ GACAATGTCTTCTGCCCGGATCGGCCCGATAA ’3
||||||||||||o|||||||||||||
3’ TGTTACAGAAGGCGGGCCTAGCCGG ’5
Fragment Abundance
5
10
15
0
500
1000
1500
2000
2500
3000
Transcript position
Cleavage site: 800 Tag abundance: 14 Weighted abundance: 0.808 Category: 2
sRNA abundance: 1 Alignment score: 0.5 MFE ratio: 0.959 p−value: 0.032

HORVU5Hr1G016060|HORVU5Hr1G016060.3||195|1001

```
5' GACAATGTCTTCCGCCCGGATCGGCCCGATAA '3
      |||||||||||||||||||||||||
3'    TGTTACAGAAGGCGGGCCTAGCCGG       '5
```

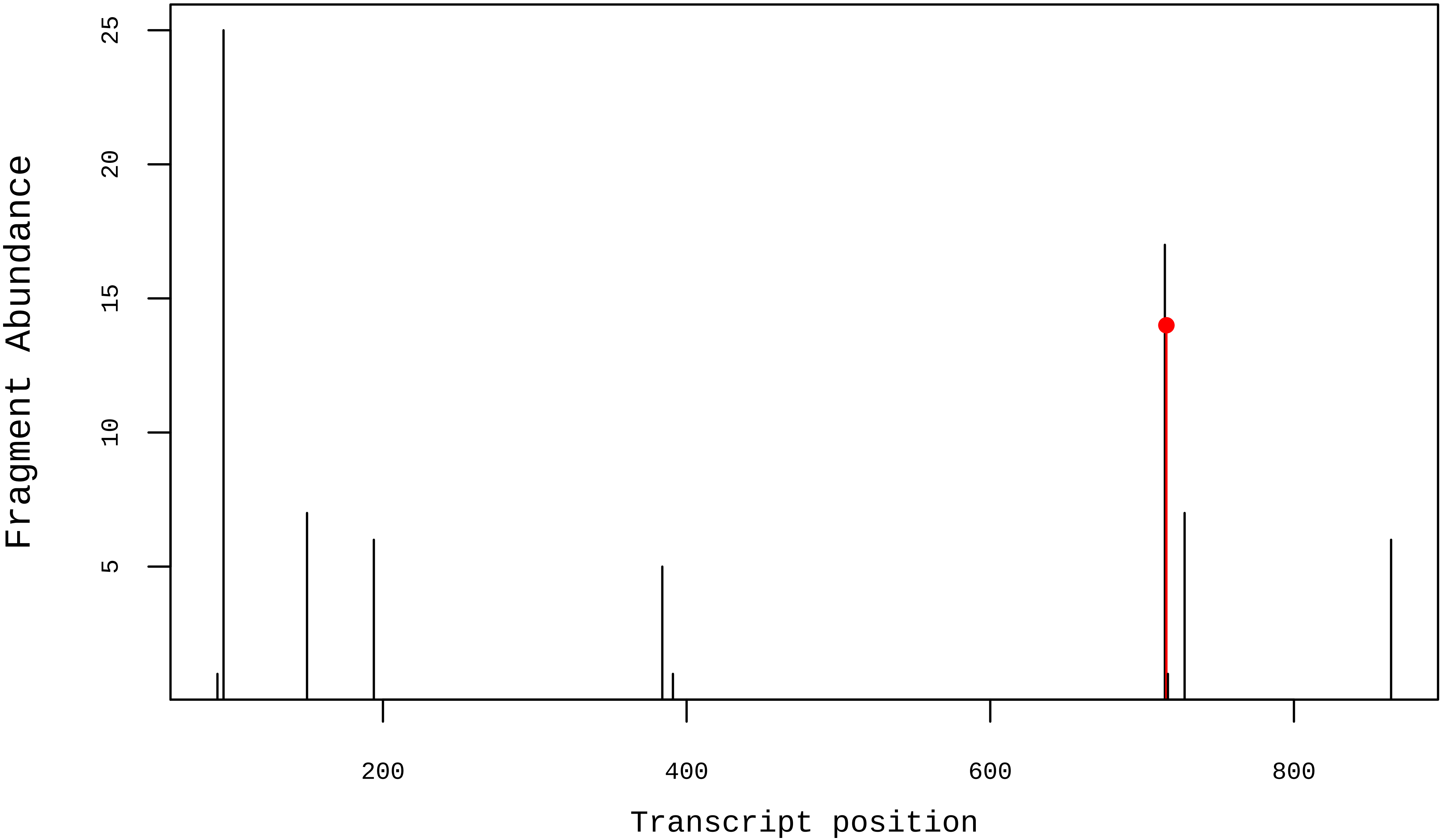

Cleavage site: 716   Tag abundance: 14   Weighted abundance: 0.808   Category: 2
sRNA abundance: 1   Alignment score: 0   MFE ratio: 1   p-value: 0.028

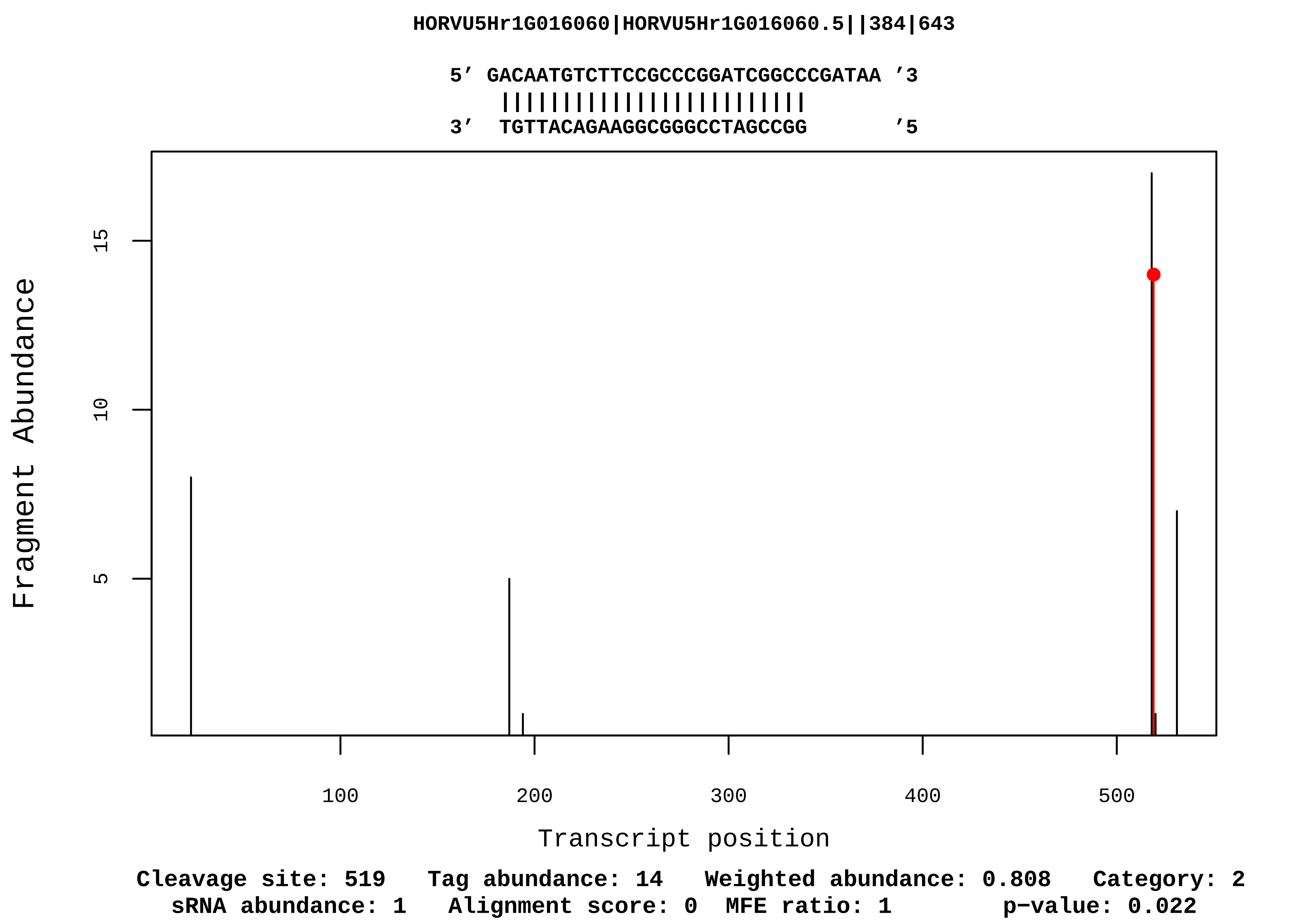
HORVU5Hr1G016060|HORVU5Hr1G016060.5||384|643
5′ GACAATGTCTTCCGCCCGGATCGGCCCGATAA ′3
3′ TGTTACAGAAGGCGGGCCTAGCCGG ′5
Fragment Abundance
5
10
15
100
200
300
400
500
Transcript position
Cleavage site: 519 Tag abundance: 14 Weighted abundance: 0.808 Category: 2
sRNA abundance: 1 Alignment score: 0 MFE ratio: 1 p-value: 0.022

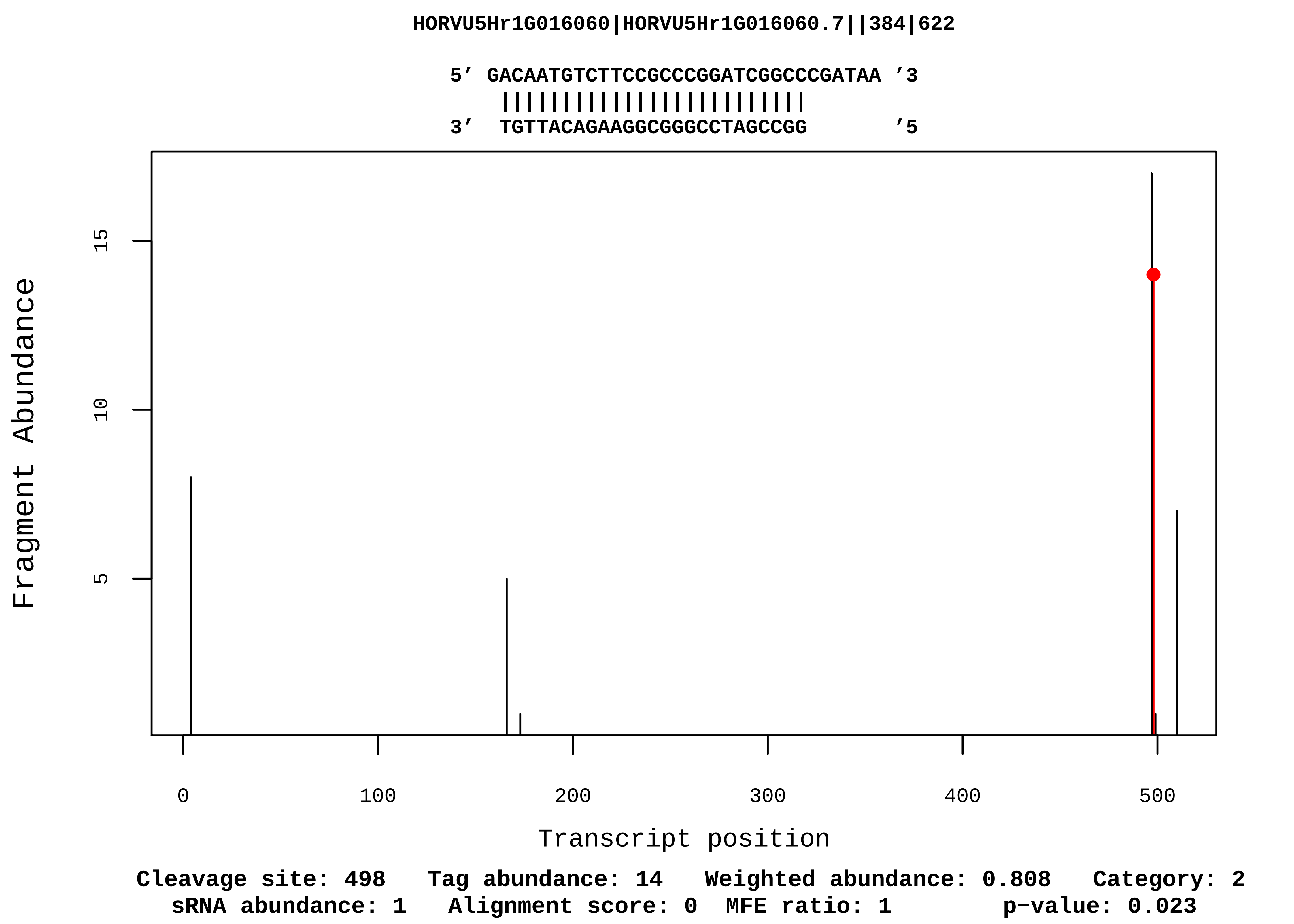
HORVU5Hr1G016060|HORVU5Hr1G016060.7||384|622
5' GACAATGTCTTCCGCCCGGATCGGCCCGATAA '3
||||||||||||||||||||||||
3' TGTTACAGAAGGCGGGCCTAGCCGG '5
Fragment Abundance
5
10
15
0
100
200
300
400
500
Transcript position
Cleavage site: 498 Tag abundance: 14 Weighted abundance: 0.808 Category: 2
sRNA abundance: 1 Alignment score: 0 MFE ratio: 1 p-value: 0.023

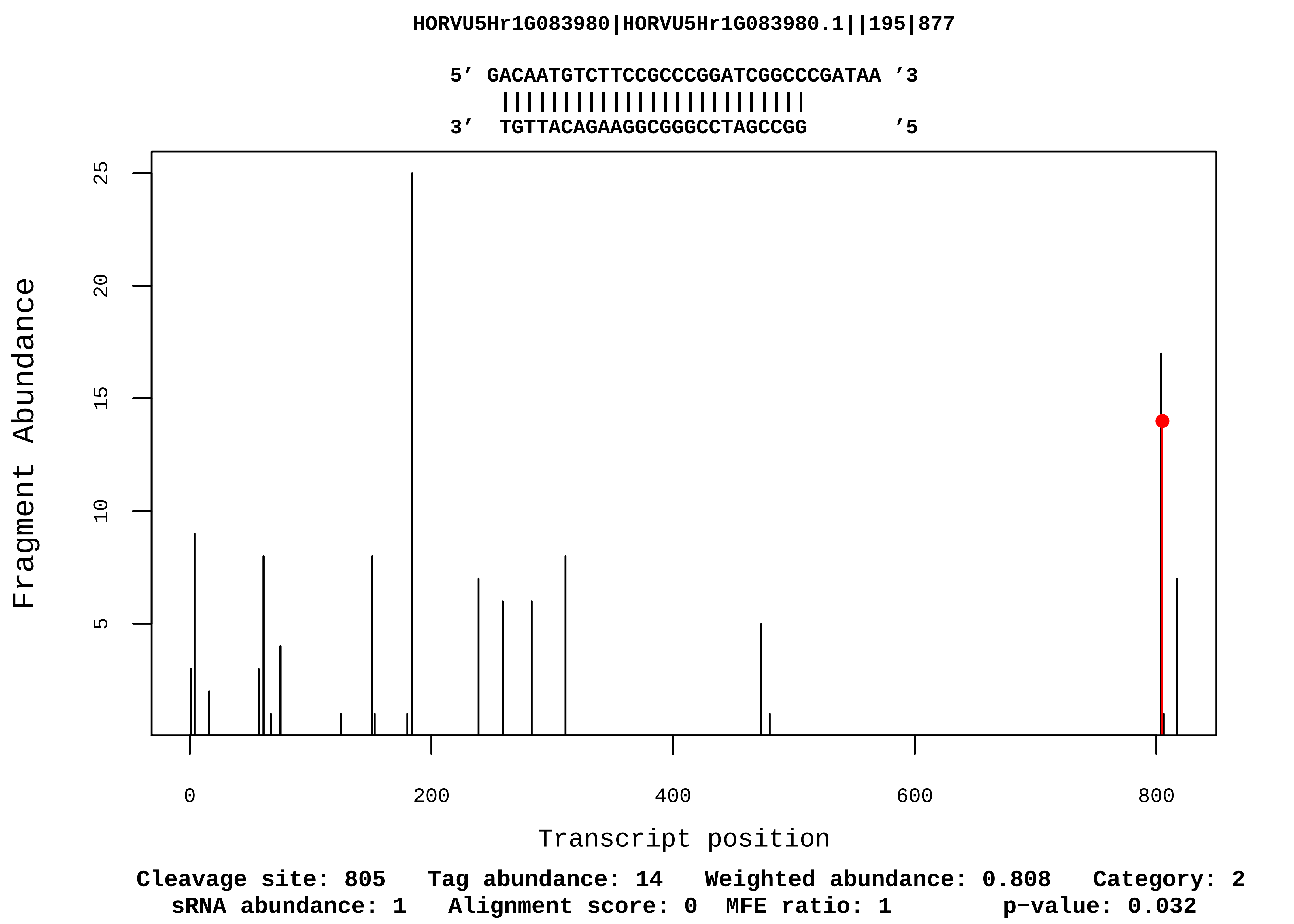
HORVU5Hr1G083980|HORVU5Hr1G083980.1||195|877
5′ GACAATGTCTTCCGCCCGGATCGGCCCGATAA ′3
||||||||||||||||||||||||
3′ TGTTACAGAAGGCGGGCCTAGCCGG ′5
Fragment Abundance
25
20
15
10
5
0
200
400
600
800
Transcript position
Cleavage site: 805 Tag abundance: 14 Weighted abundance: 0.808 Category: 2
sRNA abundance: 1 Alignment score: 0 MFE ratio: 1 p−value: 0.032

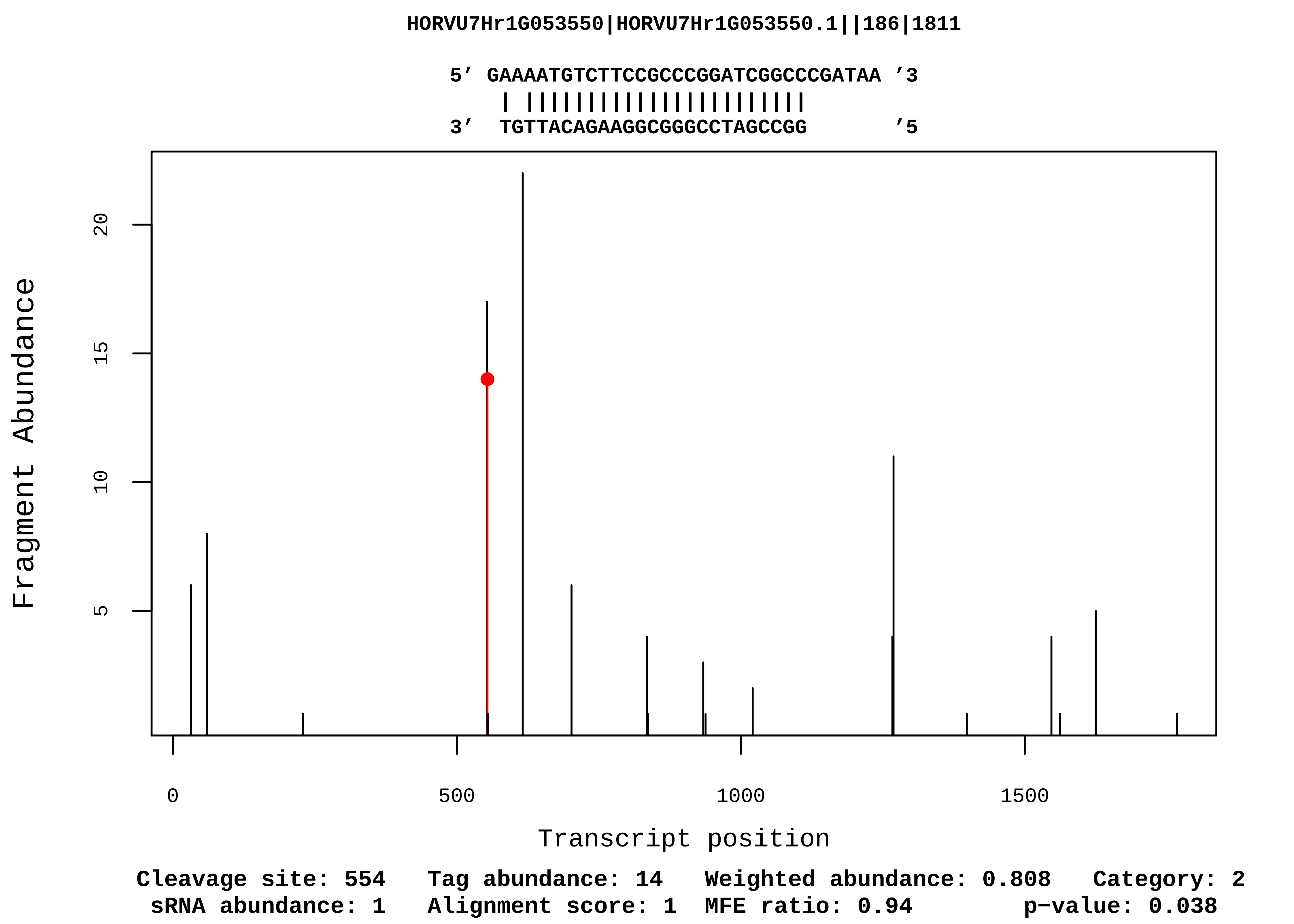
HORVU7Hr1G053550|HORVU7Hr1G053550.1||186|1811
5' GAAAATGTCTTCCGCCCGGATCGGCCCGATAA '3
| ||||||||||||||||||||||||
3' TGTTACAGAAGGCGGGCCTAGCCGG '5
Fragment Abundance
5
10
15
20
0
500
1000
1500
Transcript position
Cleavage site: 554 Tag abundance: 14 Weighted abundance: 0.808 Category: 2
sRNA abundance: 1 Alignment score: 1 MFE ratio: 0.94 p-value: 0.038

HORVU7Hr1G053550|HORVU7Hr1G053550.2||186|1810

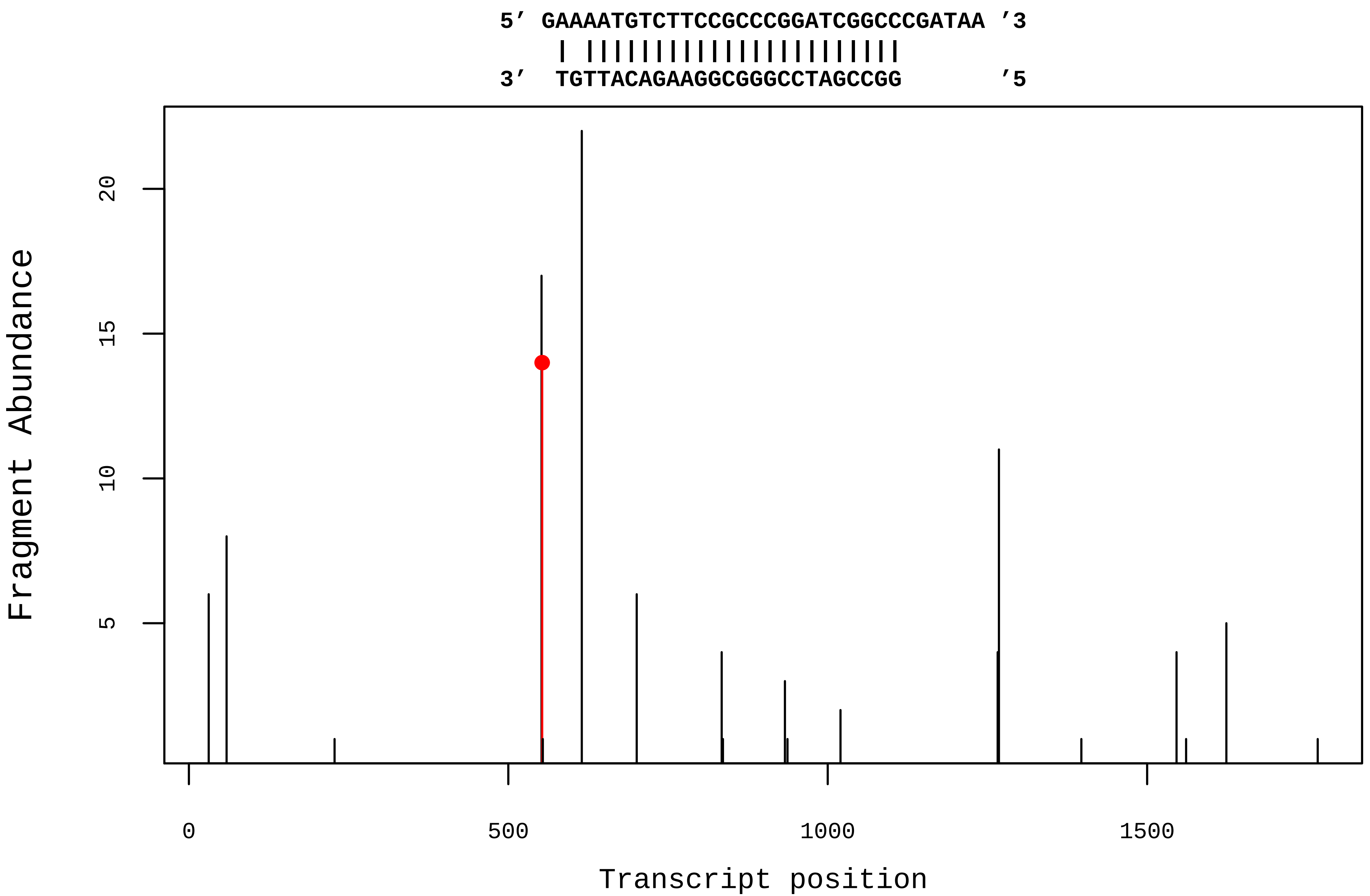

Cleavage site: 553 Tag abundance: 14 Weighted abundance: 0.808 Category: 2
sRNA abundance: 1 Alignment score: 1 MFE ratio: 0.94 p-value: 0.038

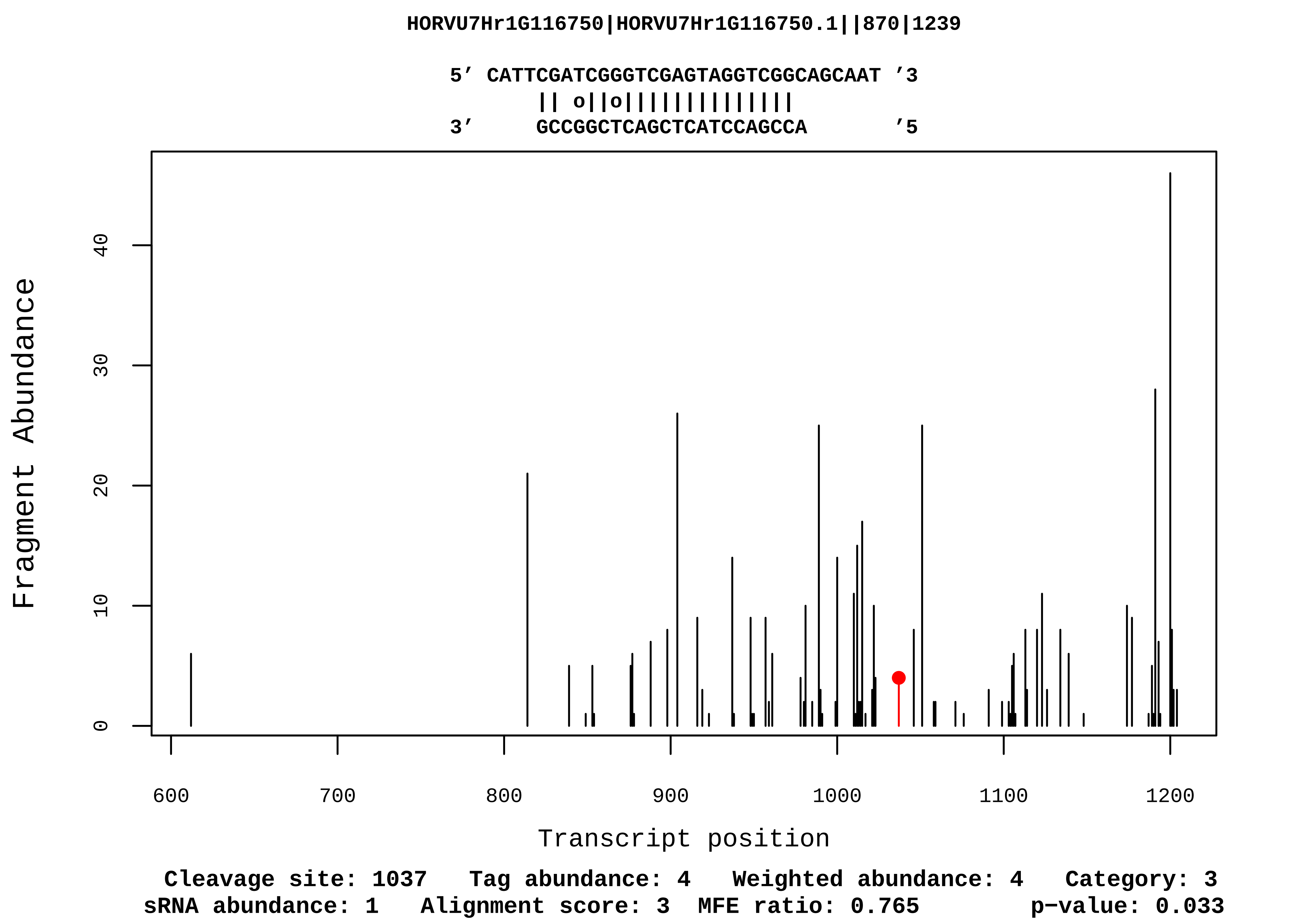
HORVU7Hr1G116750|HORVU7Hr1G116750.1||870|1239
5' CATTCGATCGGGTCGAGTAGGTCGGCAGCAAT '3
|| o||o||||||||||||||
3' GCCGGCTCAGCTCATCCAGCCA '5
Fragment Abundance
0
10
20
30
40
600
700
800
900
1000
1100
1200
Transcript position
Cleavage site: 1037 Tag abundance: 4 Weighted abundance: 4 Category: 3
sRNA abundance: 1 Alignment score: 3 MFE ratio: 0.765 p-value: 0.033

HORVU5Hr1G030460|HORVU5Hr1G030460.1||126|2860

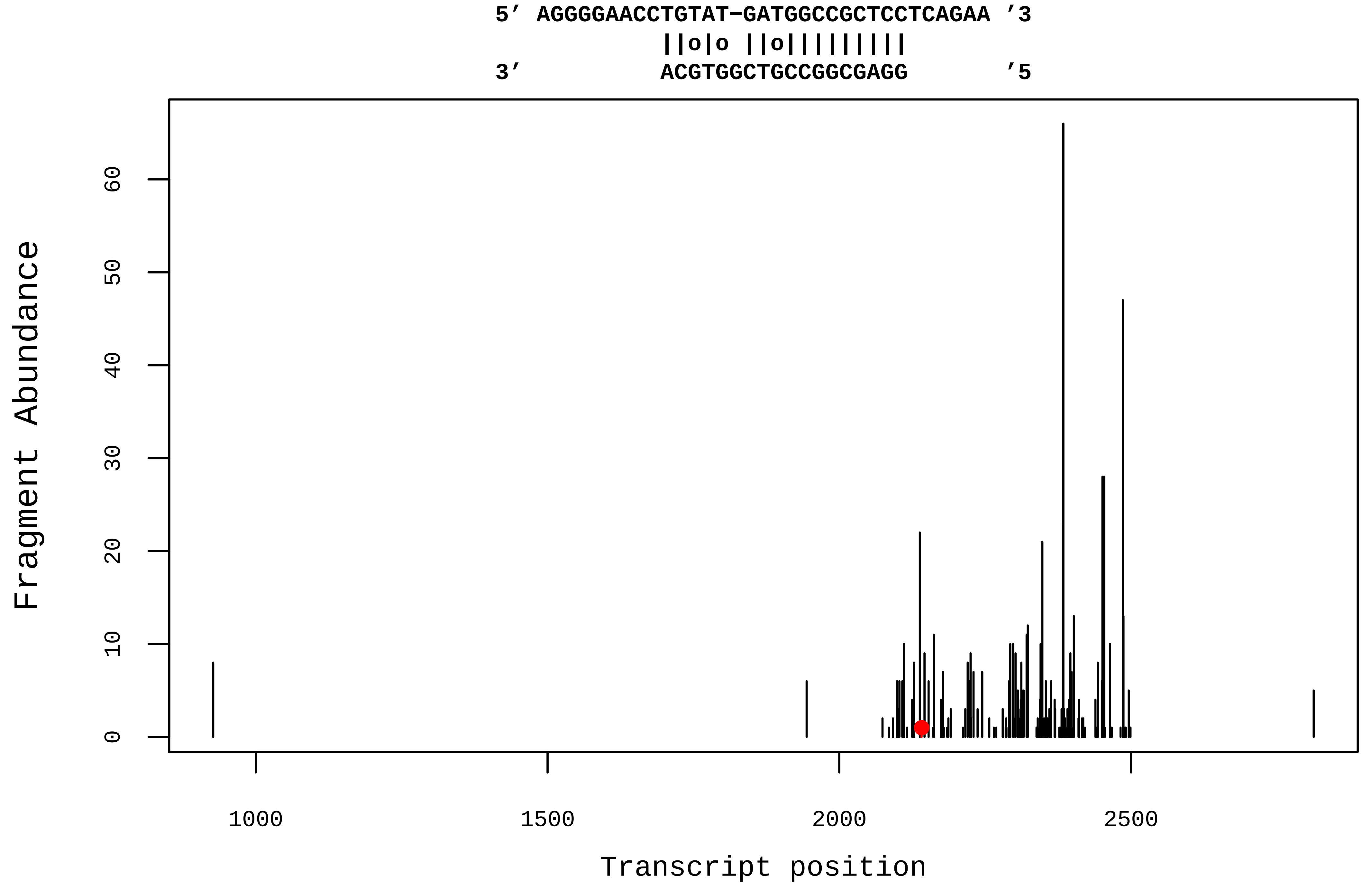

Cleavage site: 2141 Tag abundance: 1 Weighted abundance: 1 Category: 4
sRNA abundance: 1 Alignment score: 3 MFE ratio: 0.723 p−value: 0.011

HORVU1Hr1G091340|HORVU1Hr1G091340.1||906|2147

```
5' GGTGATCATCGGTGGGTTGAGTAGGTAGGTTA '3
             o||o ||||o||||||||
3'           GGCTTCCCAGCTCATCCA         '5
```

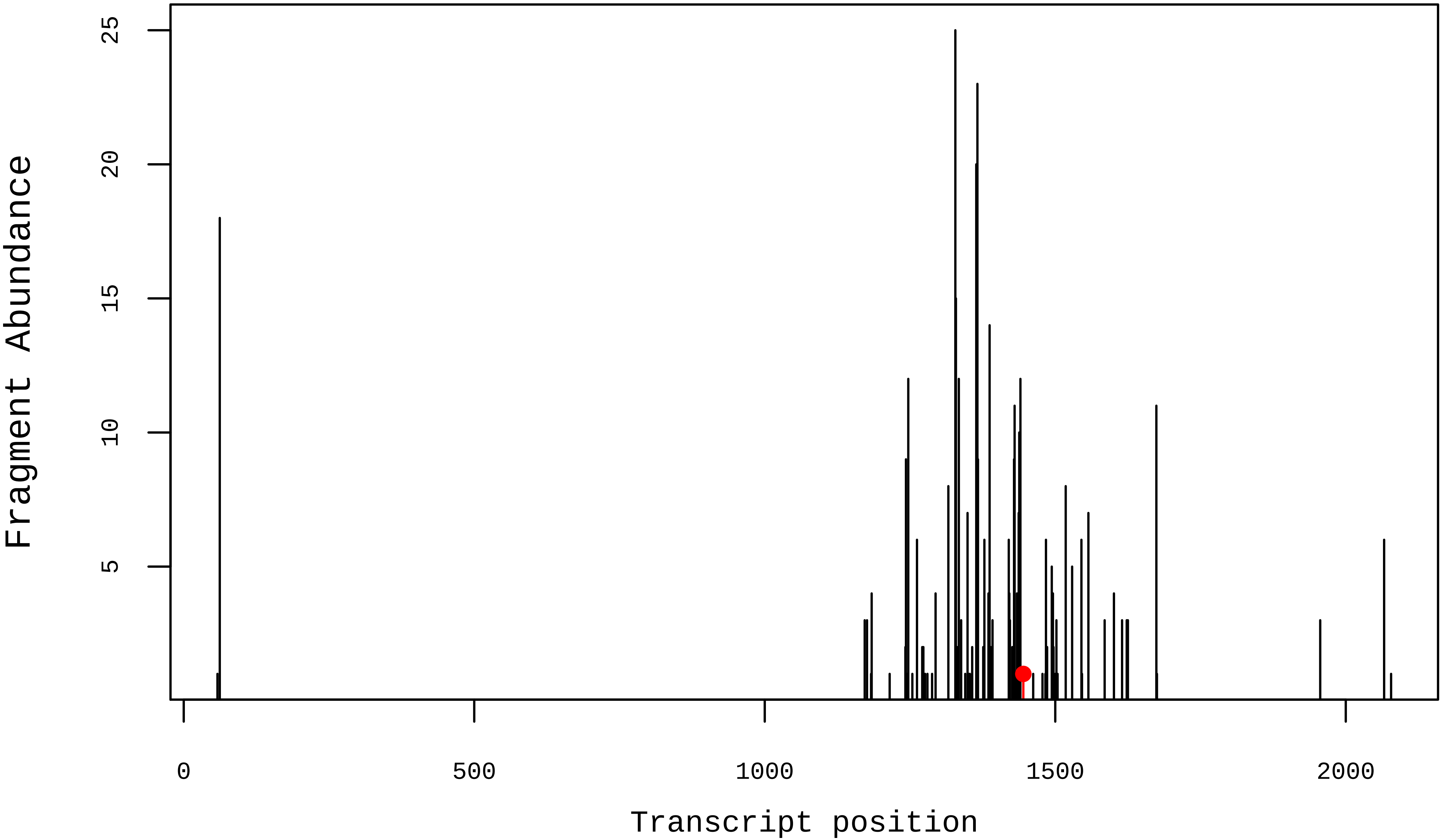

Cleavage site: 1445 Tag abundance: 1 Weighted abundance: 1 Category: 4
sRNA abundance: 1 Alignment score: 3 MFE ratio: 0.751 p-value: 0.011

HORVU0Hr1G023930|HORVU0Hr1G023930.3||396|2180

```
5' GTCGGCGGAAGGGTCGAGTAGGTCGGTGCTCG '3
           ||||||||||||||||||o
3'         CTTCCCAGCTCATCCAGCT         '5
```

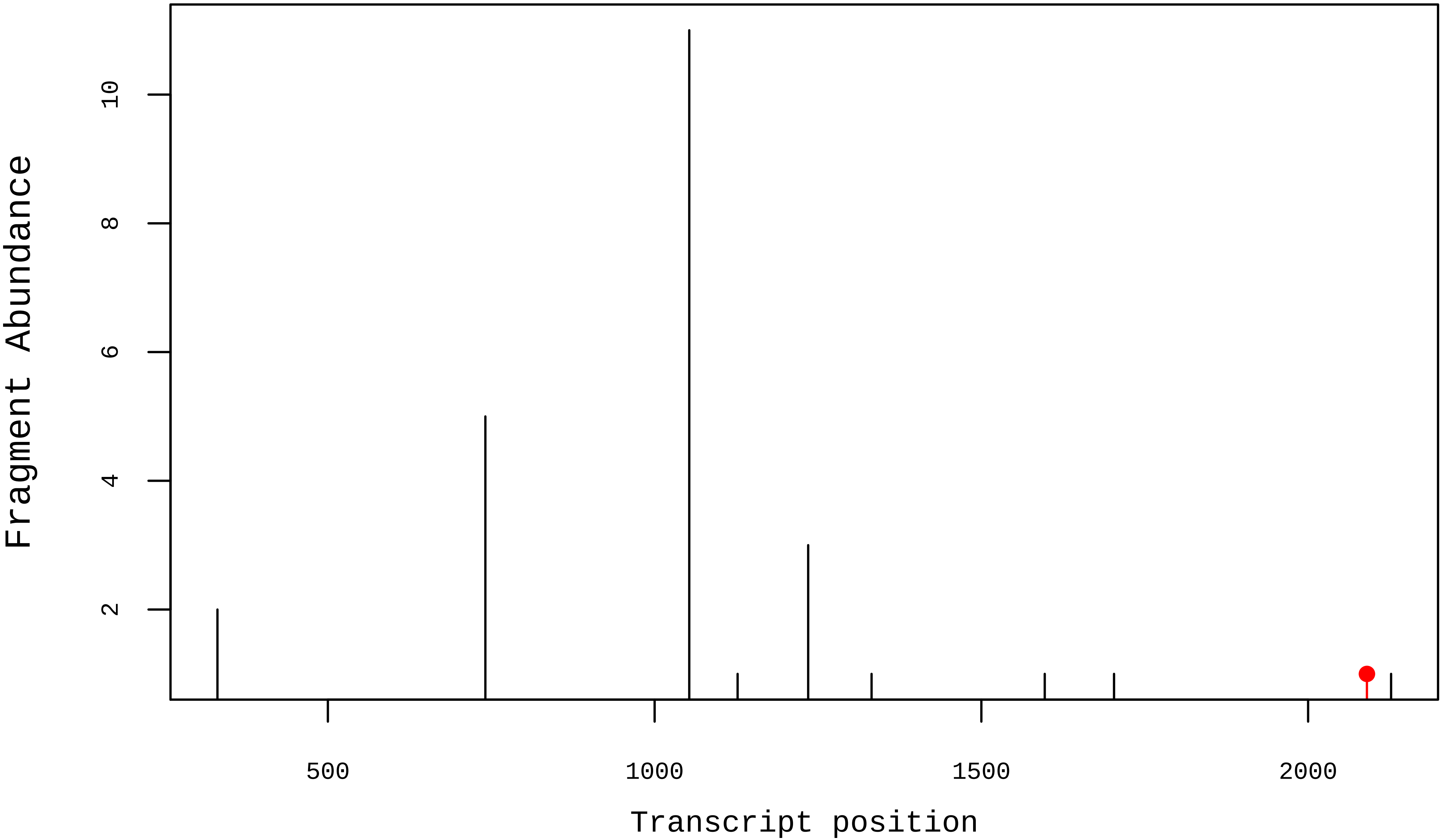

Cleavage site: 2090   Tag abundance: 1   Weighted abundance: 0.143   Category: 4
sRNA abundance: 1   Alignment score: 0.5   MFE ratio: 0.989   p-value: 0.017

HORVU2Hr1G072960|HORVU2Hr1G072960.1||600|3353

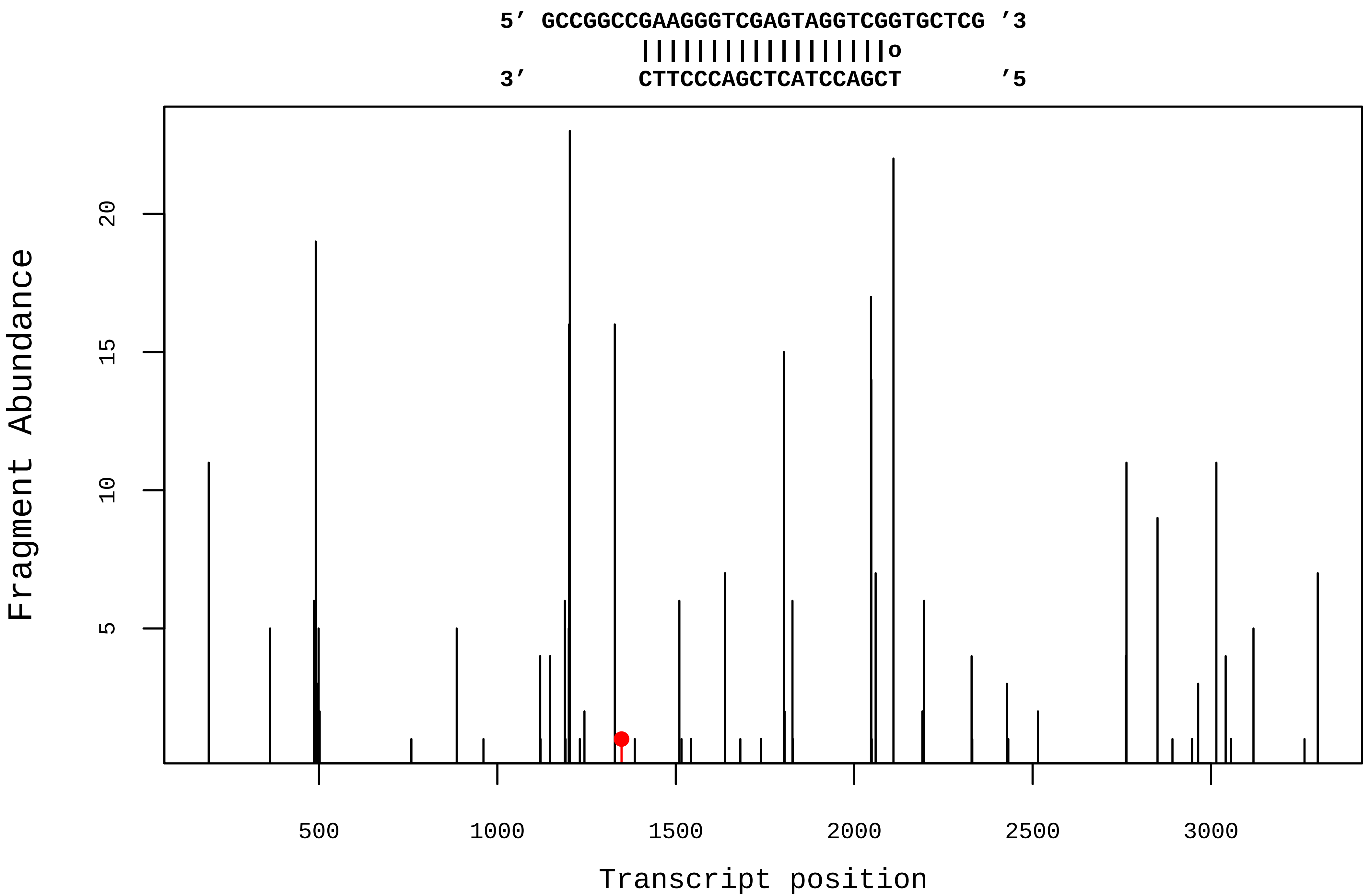

Cleavage site: 1348 Tag abundance: 1 Weighted abundance: 0.143 Category: 4
sRNA abundance: 1 Alignment score: 0.5 MFE ratio: 0.989 p-value: 0.035

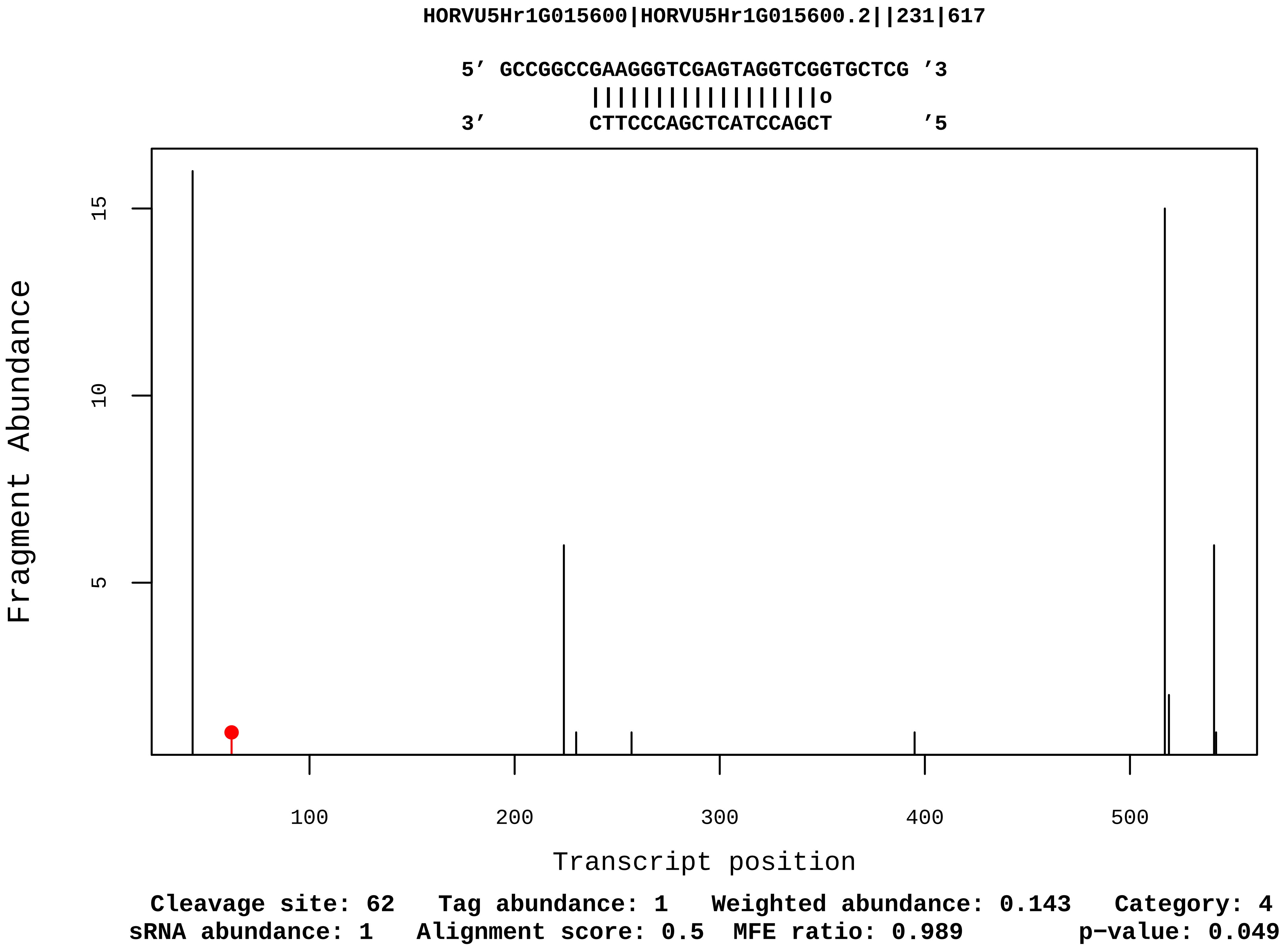
HORVU5Hr1G015600|HORVU5Hr1G015600.2||231|617
5' GCCGGCCGAAGGGTCGAGTAGGTCGGTGCTCG '3
||||||||||||||||||o
3' CTTCCCAGCTCATCCAGCT '5
Fragment Abundance
5
10
15
100
200
300
400
500
Transcript position
Cleavage site: 62 Tag abundance: 1 Weighted abundance: 0.143 Category: 4
sRNA abundance: 1 Alignment score: 0.5 MFE ratio: 0.989 p-value: 0.049

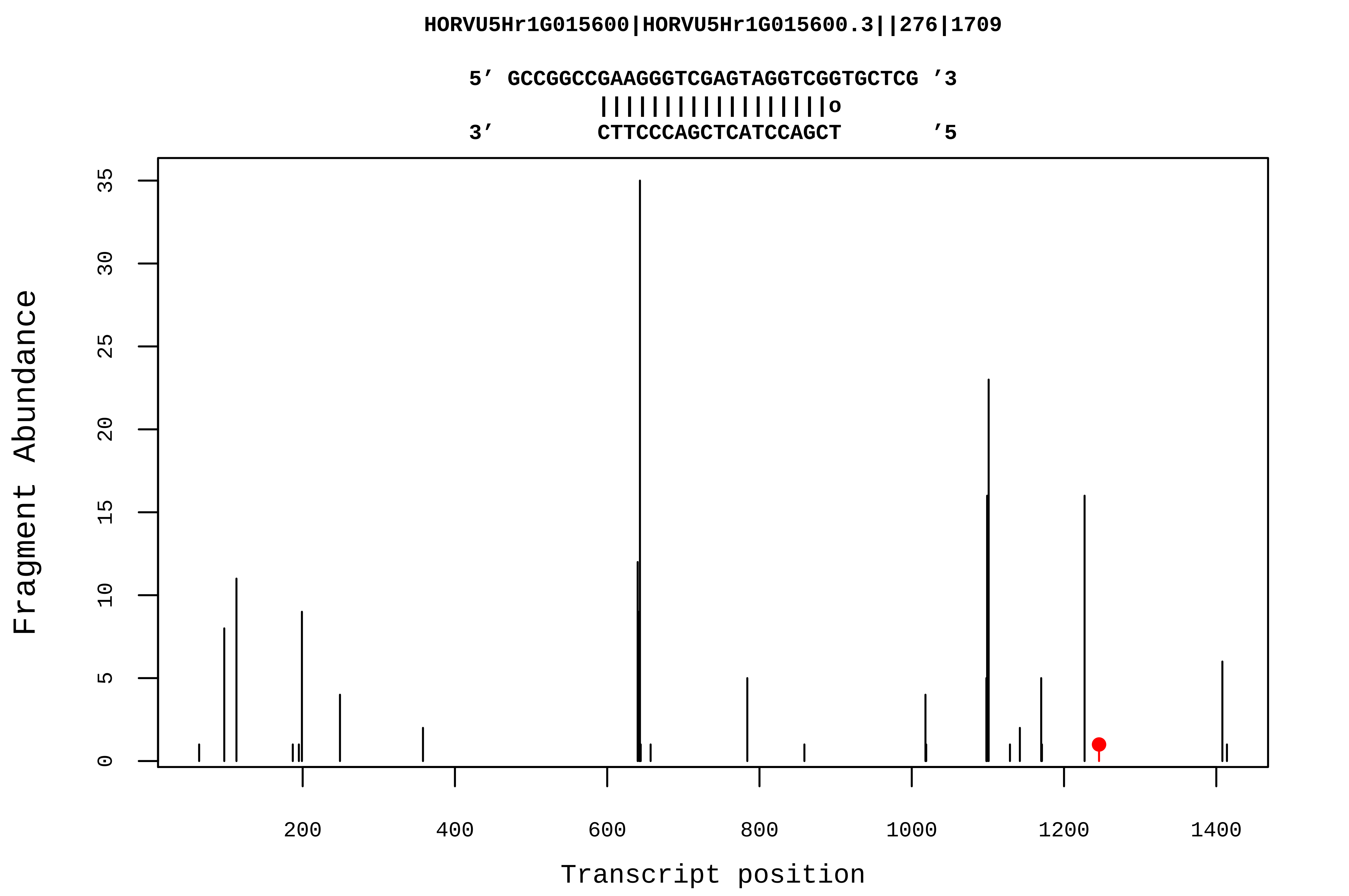

Cleavage site: 1246   Tag abundance: 1   Weighted abundance: 0.143   Category: 4
sRNA abundance: 1   Alignment score: 0.5   MFE ratio: 0.989   p-value: 0.038

HORVU5Hr1G015600|HORVU5Hr1G015600.4||207|1852

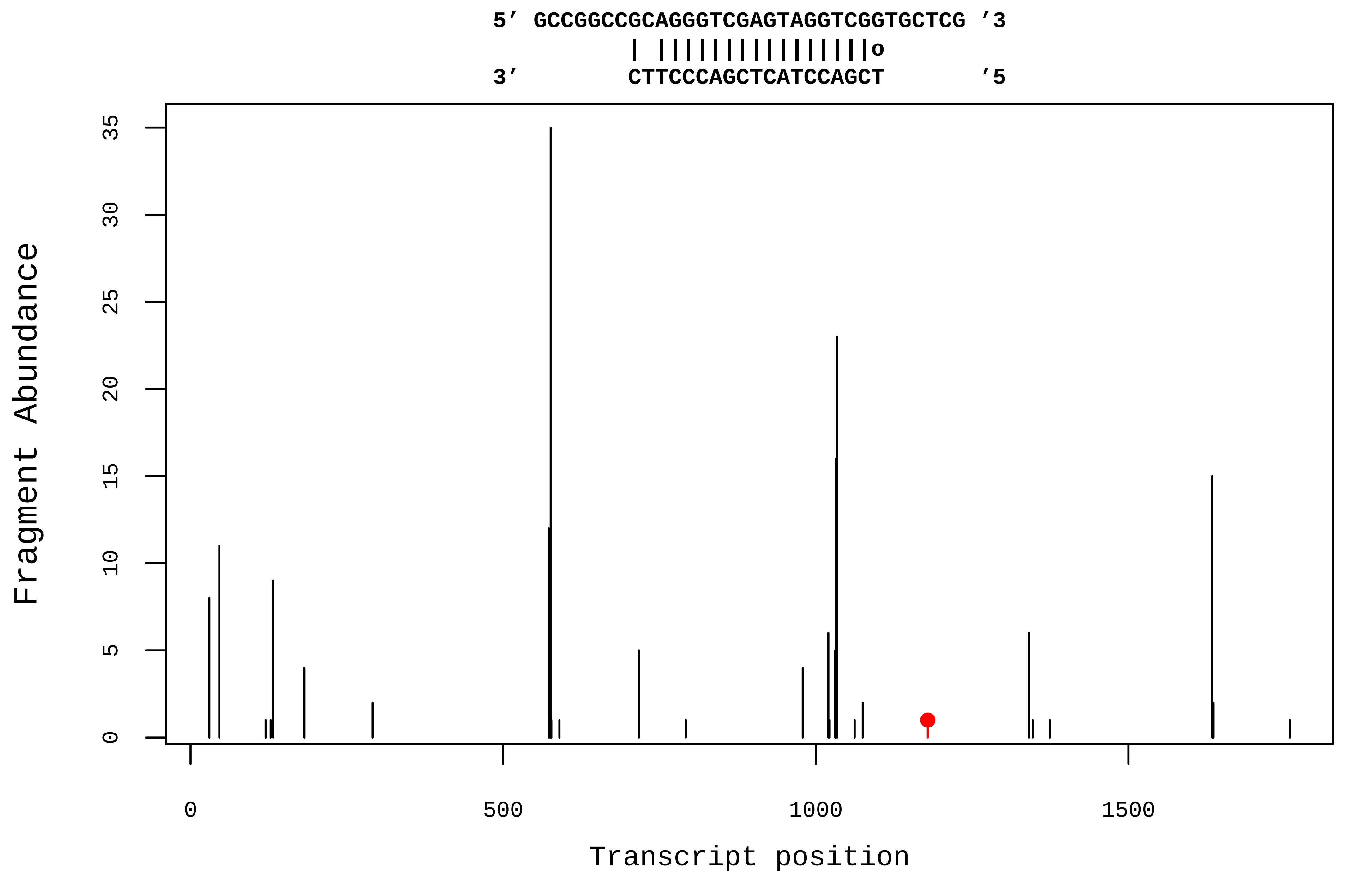

Cleavage site: 1179 Tag abundance: 1 Weighted abundance: 0.143 Category: 4
sRNA abundance: 1 Alignment score: 1.5 MFE ratio: 0.903 p-value: 0.041

HORVU4Hr1G085590|HORVU4Hr1G085590.1||2283|3703

```
5' GGTGCCGCCGGCCAAGGTGGTCGCGTAGGGCG '3
           ||||||||| ||||| |||
3'         GCCGGTTCC-CCAGCTCAT       '5
```

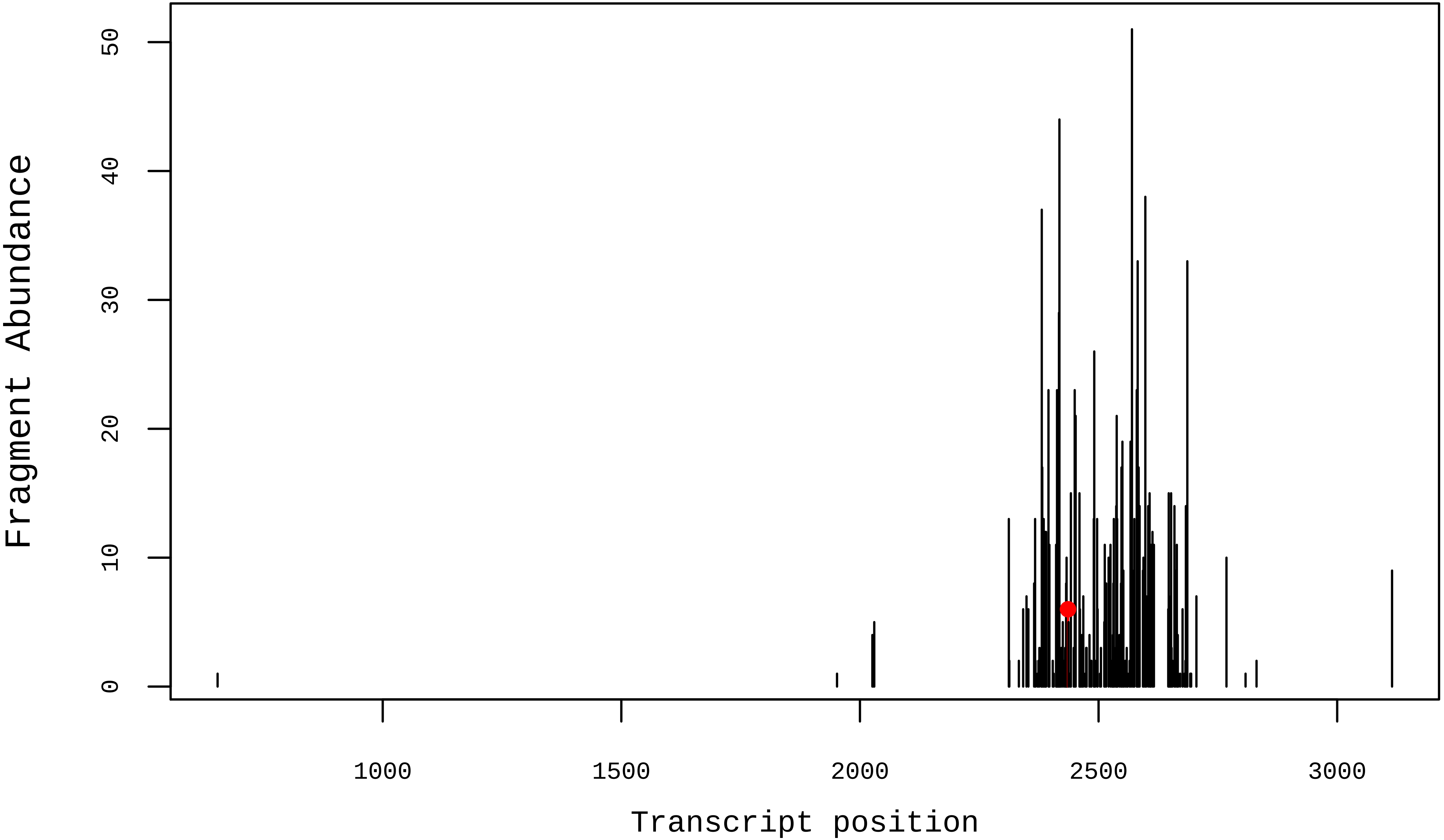

Cleavage site: 2436   Tag abundance: 6   Weighted abundance: 6   Category: 3
sRNA abundance: 1   Alignment score: 4   MFE ratio: 0.778   p-value: 0.021

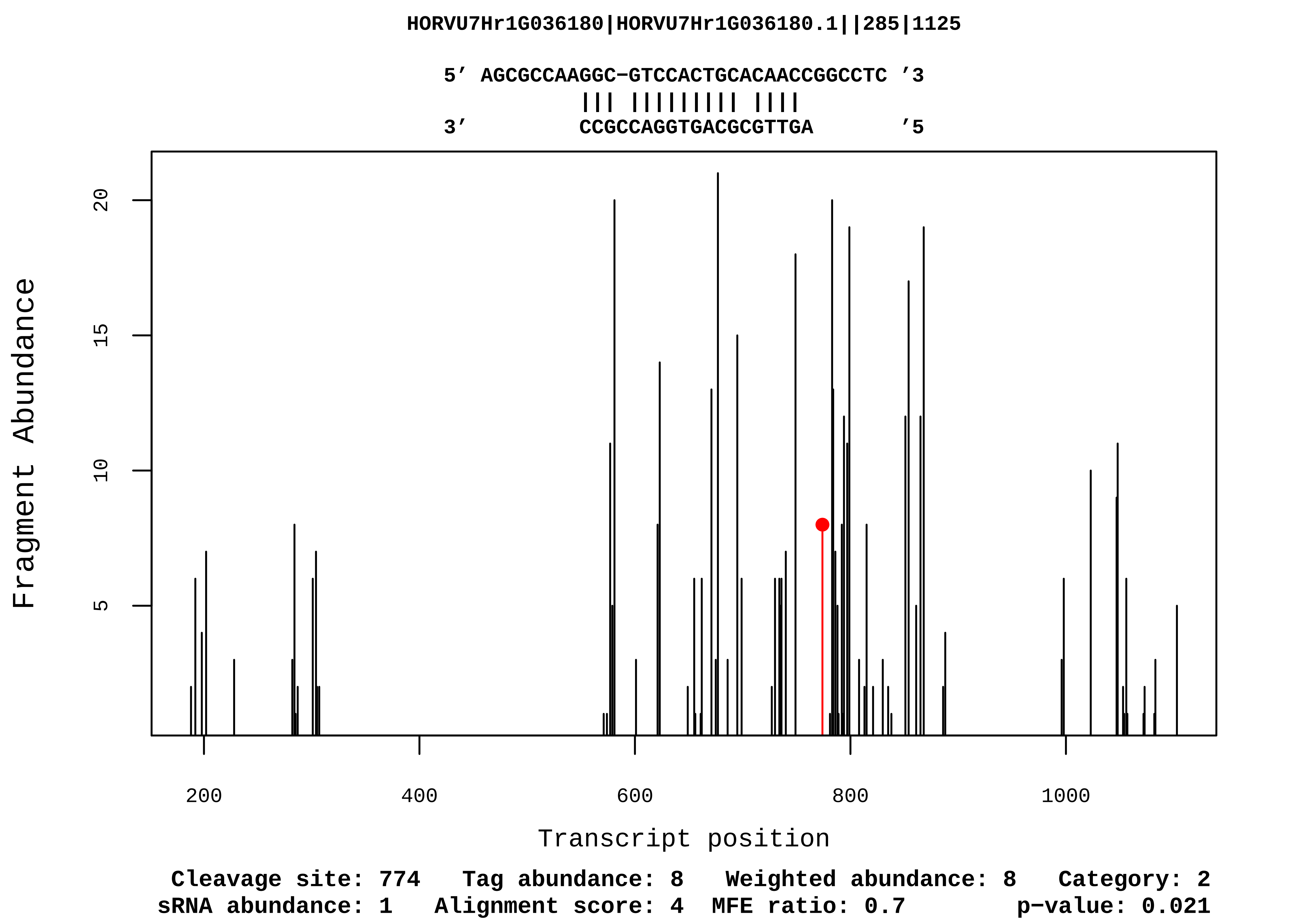

HORVU7Hr1G036180|HORVU7Hr1G036180.1||285|1125
5' AGCGCCAAGGC-GTCCACTGCACAACCGGCCTC '3
3' CCGCCAGGTGACGCGTTGA '5
Fragment Abundance
5
10
15
20
200
400
600
800
1000
Transcript position
Cleavage site: 774 Tag abundance: 8 Weighted abundance: 8 Category: 2
sRNA abundance: 1 Alignment score: 4 MFE ratio: 0.7 p-value: 0.021

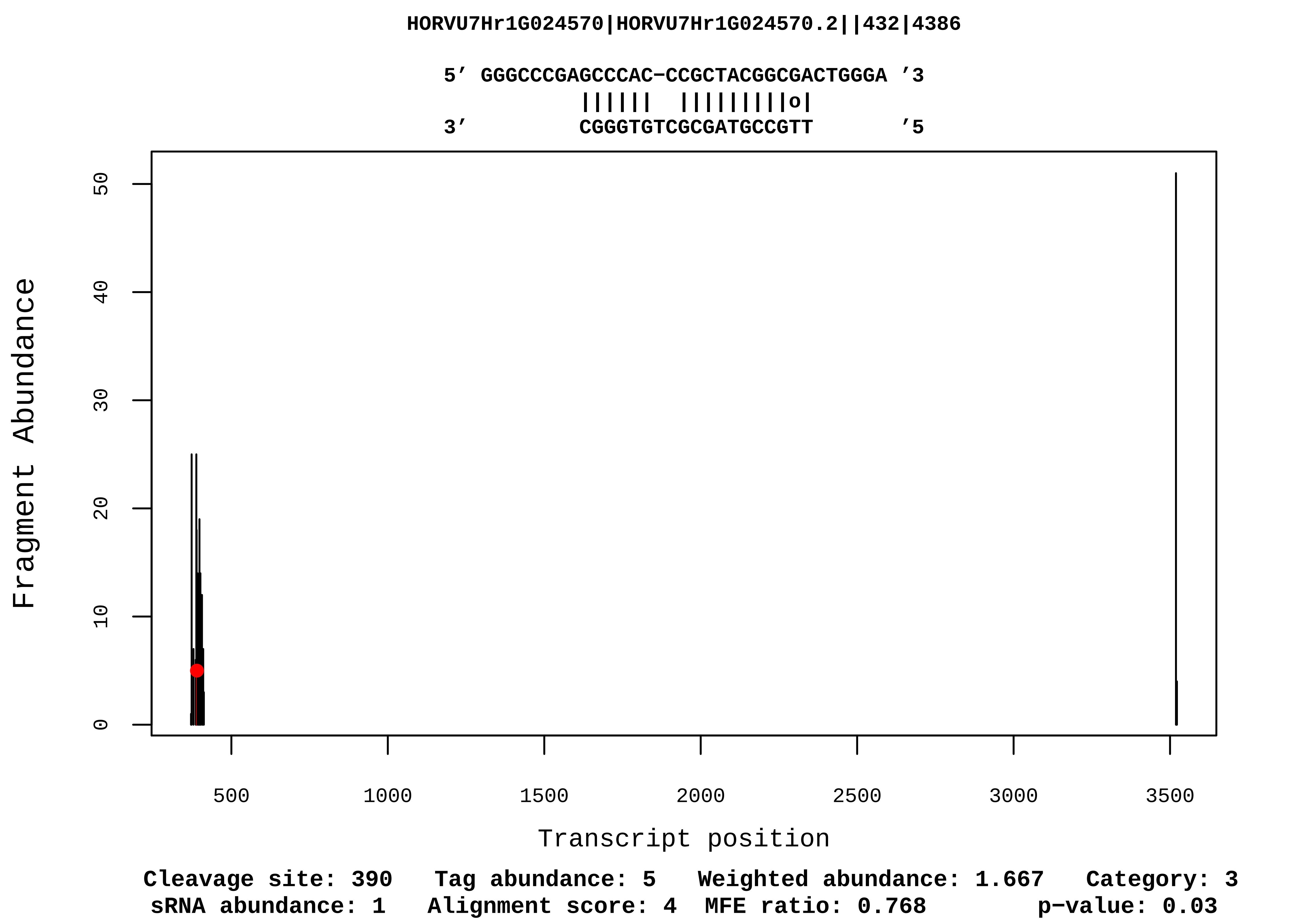
HORVU7Hr1G024570|HORVU7Hr1G024570.2||432|4386
5′ GGGCCCGAGCCCAC−CCGCTACGGCGACTGGGA ′3
|||||| |||||||||o|
3′ CGGGTGTCGCGATGCCGTT ′5
Fragment Abundance
0
10
20
30
40
50
500
1000
1500
2000
2500
3000
3500
Transcript position
Cleavage site: 390 Tag abundance: 5 Weighted abundance: 1.667 Category: 3
sRNA abundance: 1 Alignment score: 4 MFE ratio: 0.768 p−value: 0.03

HORVU1Hr1G019580|HORVU1Hr1G019580.2||1719|2297

```
5' GGTGGAGAAGCGGATGGGGAGGGA-GACTGGCC '3
            ||||||||o||||||
3'          CCGCCTACCTCTCCCTAA         '5
```

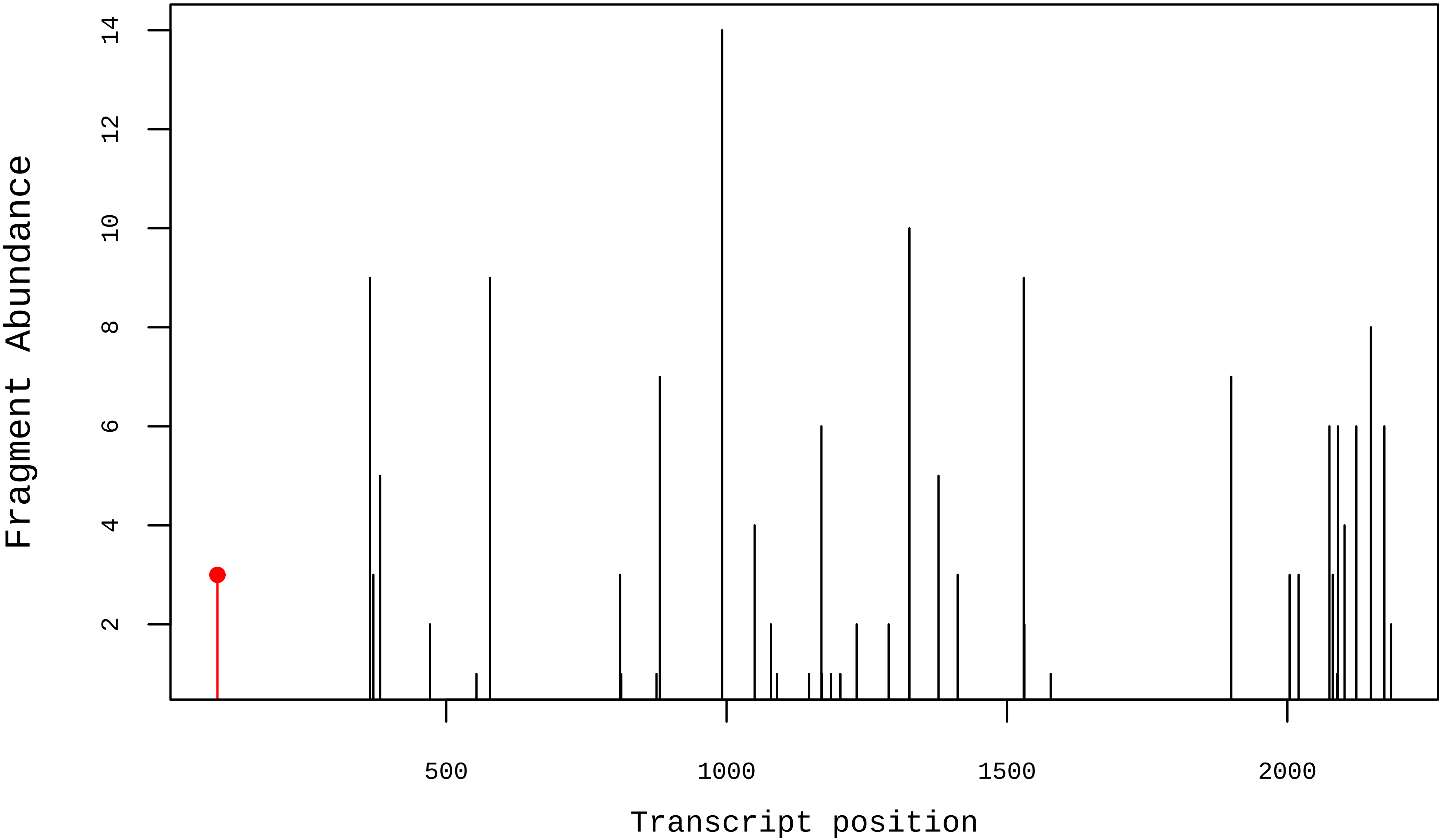

Cleavage site: 92   Tag abundance: 3   Weighted abundance: 0.6   Category: 3
sRNA abundance: 1   Alignment score: 4   MFE ratio: 0.875   p-value: 0.044

HORVU1Hr1G019580|HORVU1Hr1G019580.3||1014|2275

```
5' GGTGGAGAAGCGGATGGGGAGGGA-GACTGGCC '3
            ||||||||o||||||
3'          CCGCCTACCTCTCCCTAA         '5
```

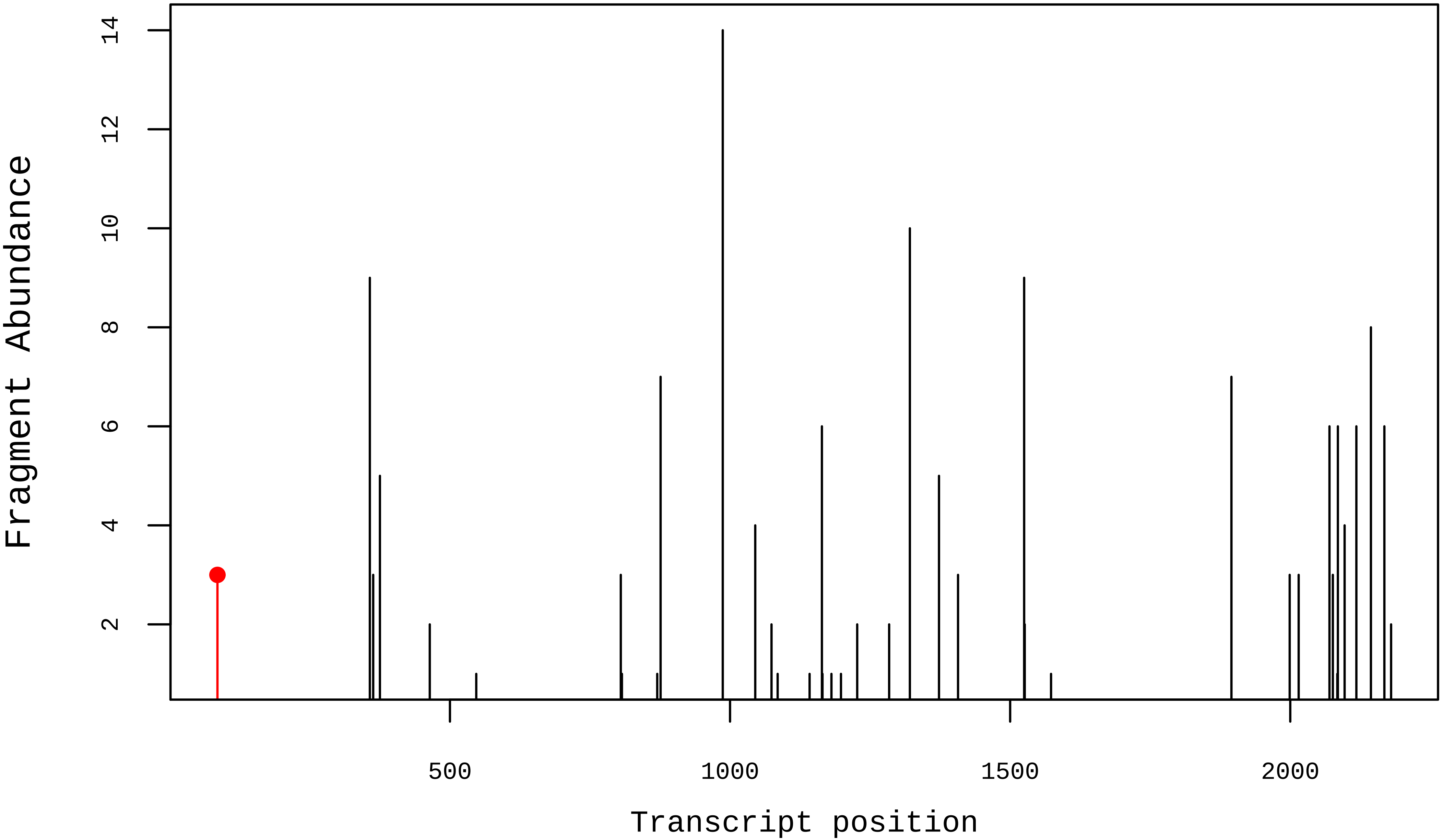

Cleavage site: 85   Tag abundance: 3   Weighted abundance: 0.6   Category: 3
sRNA abundance: 1   Alignment score: 4   MFE ratio: 0.875   p-value: 0.044

HORVU1Hr1G019580|HORVU1Hr1G019580.5||1698|2222

```
5' GGTGGAGAAGCGGATGGGGAGGGA-GACTGGCC '3
           ||||||||o||||||
3'         CCGCCTACCTCTCCCTAA        '5
```

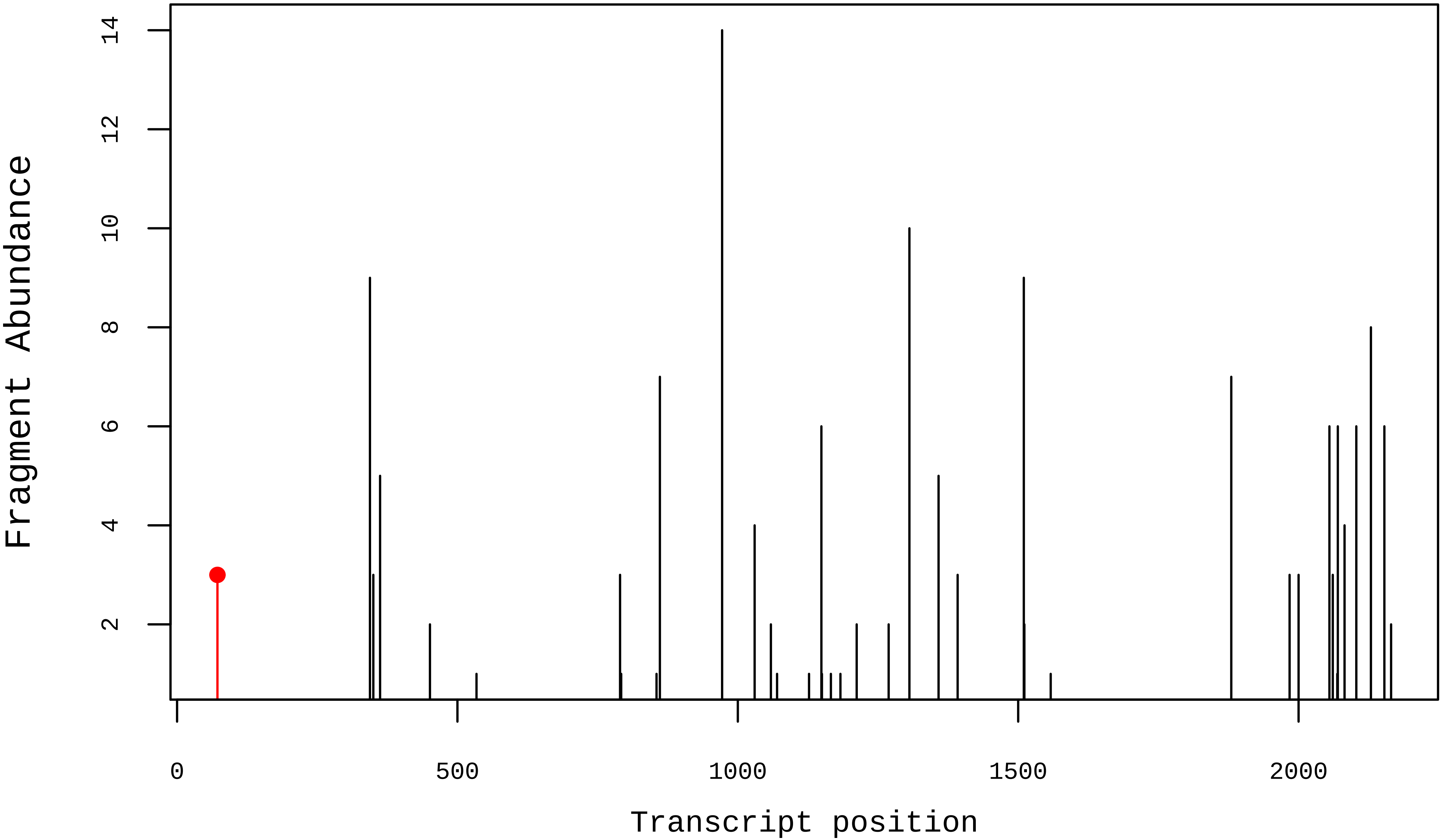

Cleavage site: 72   Tag abundance: 3   Weighted abundance: 0.6   Category: 3
sRNA abundance: 1   Alignment score: 4   MFE ratio: 0.875   p-value: 0.045

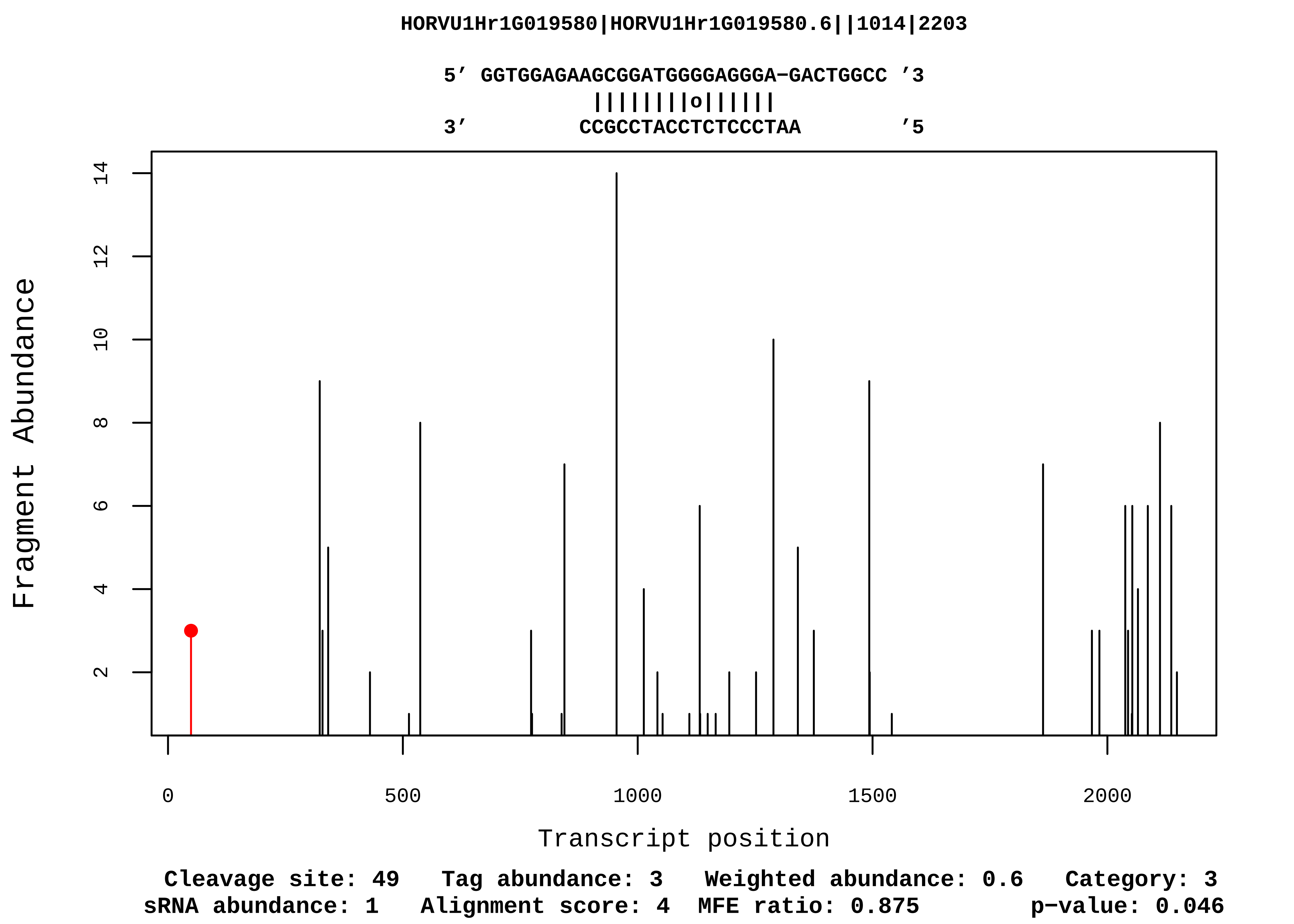
HORVU1Hr1G019580|HORVU1Hr1G019580.6||1014|2203
5' GGTGGAGAAGCGGATGGGGAGGGA-GACTGGCC '3
||||||||o||||||
3' CCGCCTACCTCTCCCTAA '5
Fragment Abundance
2
4
6
8
10
12
14
0
500
1000
1500
2000
Transcript position
Cleavage site: 49 Tag abundance: 3 Weighted abundance: 0.6 Category: 3
sRNA abundance: 1 Alignment score: 4 MFE ratio: 0.875 p-value: 0.046

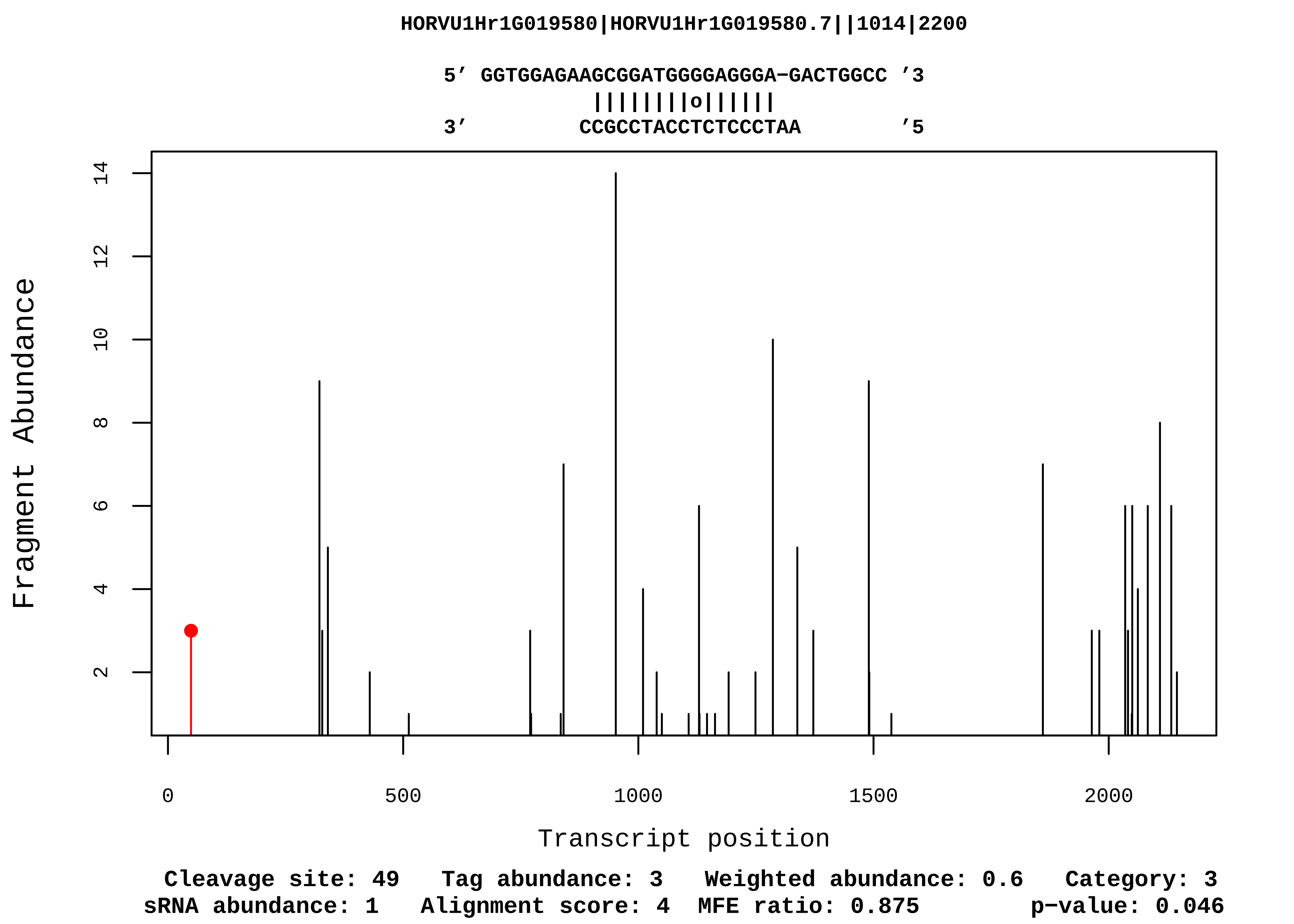
HORVU1Hr1G019580|HORVU1Hr1G019580.7||1014|2200
5′ GGTGGAGAAGCGGATGGGGAGGGA−GACTGGCC ′3
||||||||o||||||
3′ CCGCCTACCTCTCCCTAA ′5
Fragment Abundance
2
4
6
8
10
12
14
0
500
1000
1500
2000
Transcript position
Cleavage site: 49 Tag abundance: 3 Weighted abundance: 0.6 Category: 3
sRNA abundance: 1 Alignment score: 4 MFE ratio: 0.875 p-value: 0.046

HORVU4Hr1G021100|HORVU4Hr1G021100.2||159|181

```
5' CGACGGCGGCGGGATGGAGAGGGA-AATGAAAT '3
             |  |||||||||||||
3'           CCGCCTACCTCTCCCTAA        '5
```

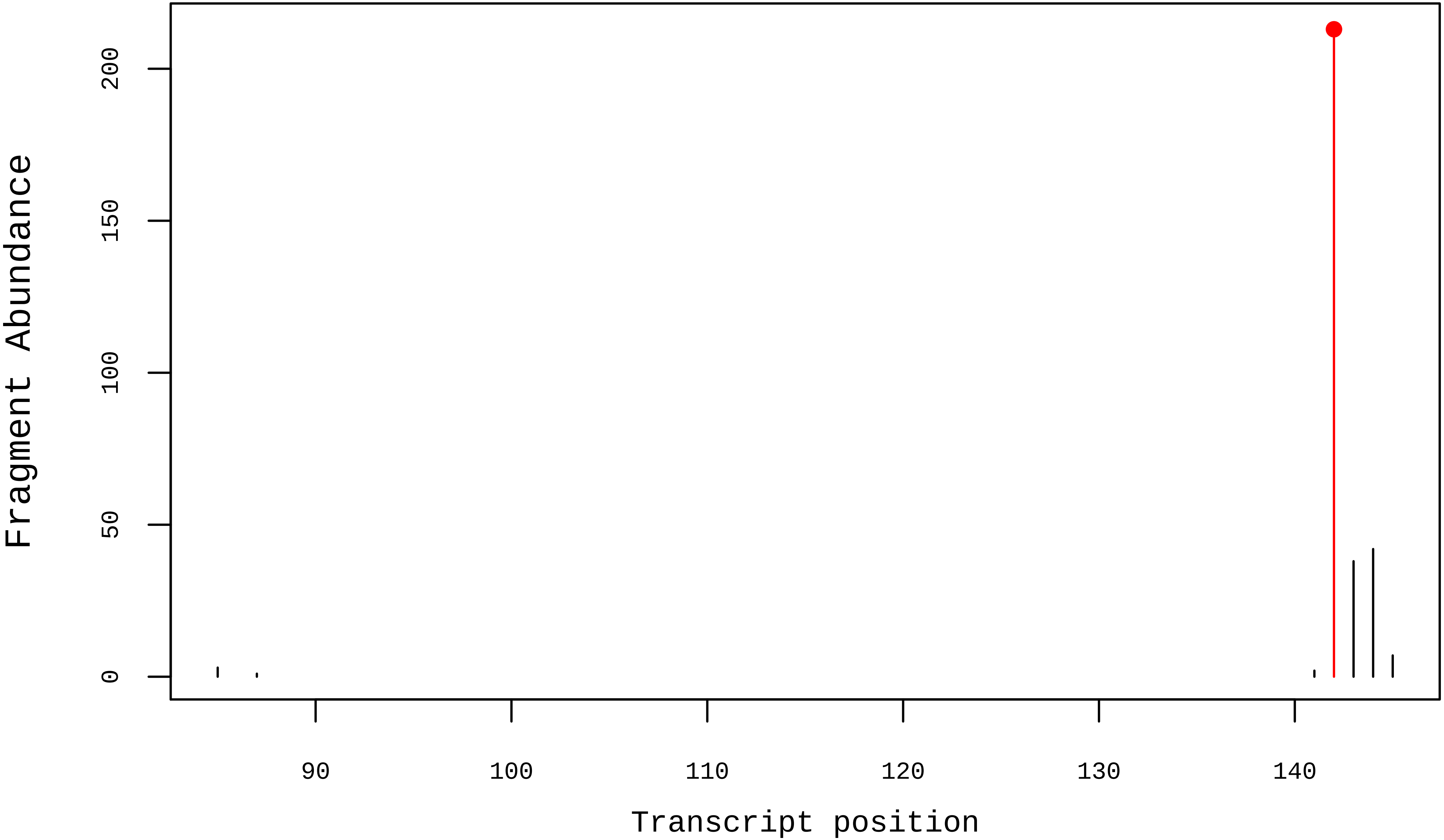

Cleavage site: 142   Tag abundance: 213   Weighted abundance: 106.5   Category: 0
sRNA abundance: 1   Alignment score: 4   MFE ratio: 0.79   p-value: 0.036

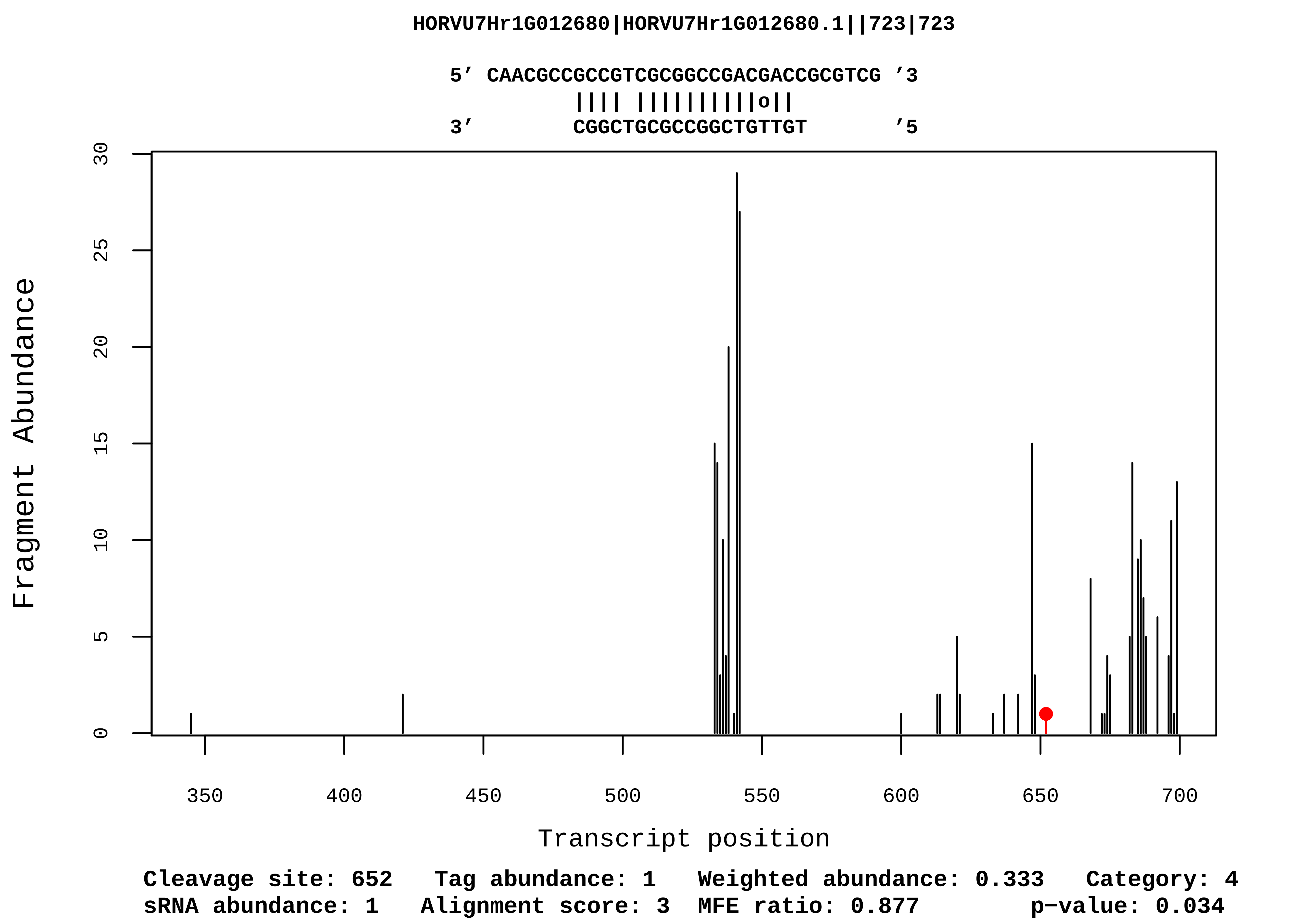
HORVU7Hr1G012680|HORVU7Hr1G012680.1||723|723
5' CAACGCCGCCGTCGCGGCCGACGACCGCGTCG '3
|||| ||||||||||o||
3' CGGCTGCGCCGGCTGTTGT '5
Fragment Abundance
0
5
10
15
20
25
30
350
400
450
500
550
600
650
700
Transcript position
Cleavage site: 652 Tag abundance: 1 Weighted abundance: 0.333 Category: 4
sRNA abundance: 1 Alignment score: 3 MFE ratio: 0.877 p-value: 0.034

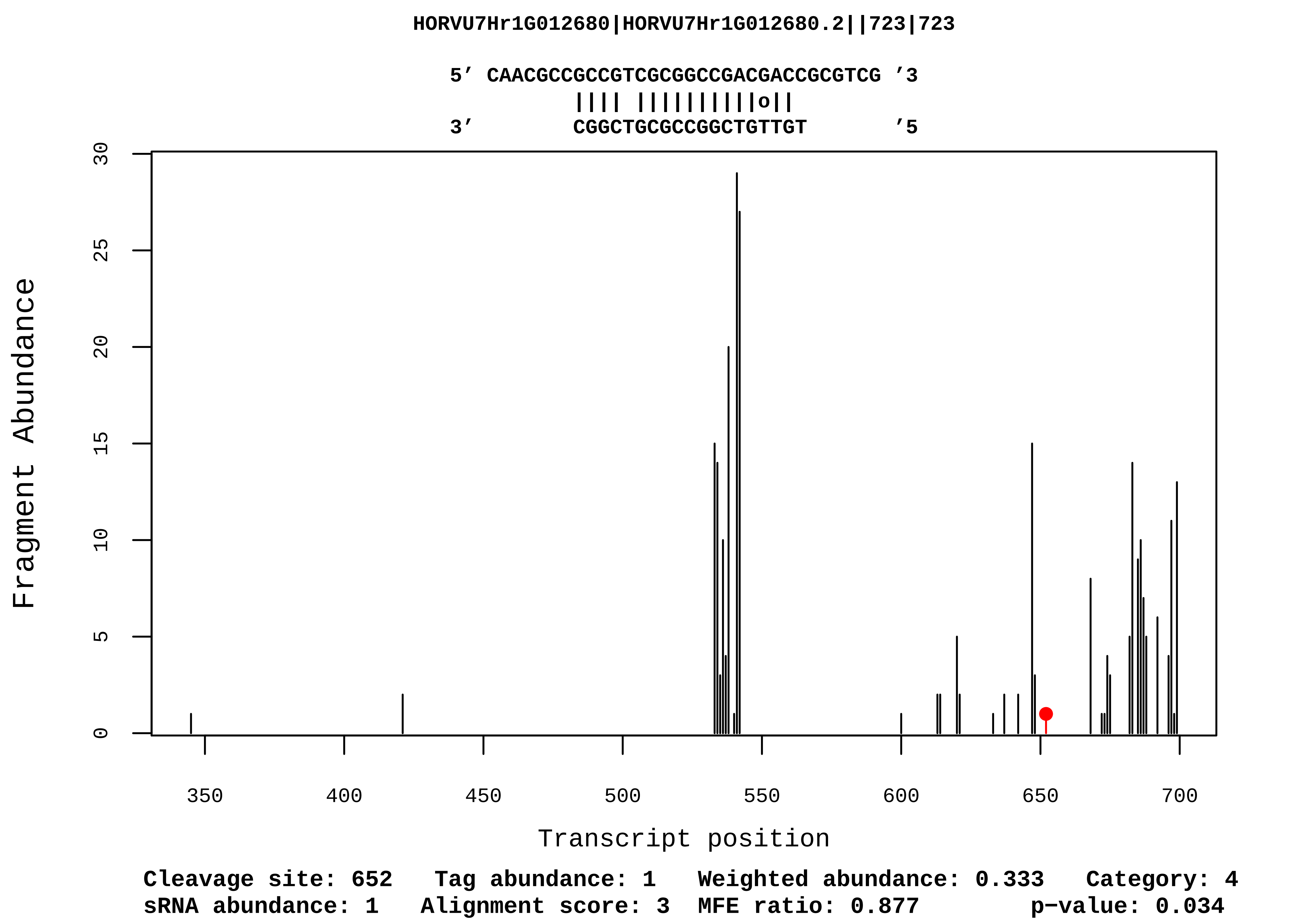
HORVU7Hr1G012680|HORVU7Hr1G012680.2||723|723
5′ CAACGCCGCCGTCGCGGCCGACGACCGCGTCG ′3
|||| ||||||||||o||
3′ CGGCTGCGCCGGCTGTTGT ′5
Fragment Abundance
0
5
10
15
20
25
30
350
400
450
500
550
600
650
700
Transcript position
Cleavage site: 652 Tag abundance: 1 Weighted abundance: 0.333 Category: 4
sRNA abundance: 1 Alignment score: 3 MFE ratio: 0.877 p-value: 0.034

HORVU7Hr1G012680|HORVU7Hr1G012680.3||717|717

```
5' CAACGCCGCCGTCGCGGCCGACGACCGCGTCG '3
          |||| ||||||||||o||
3'        CGGCTGCGCCGGCTGTTGT       '5
```

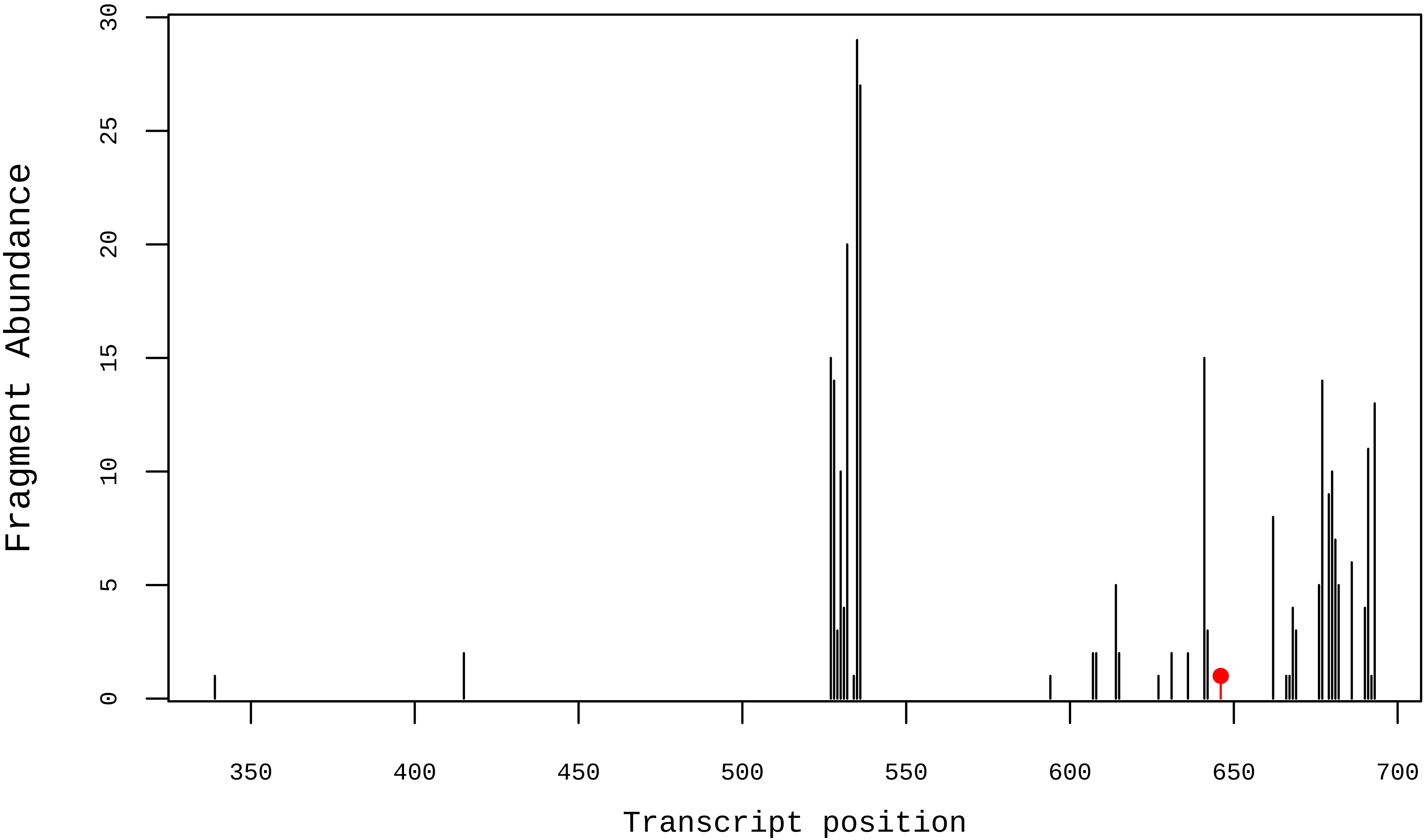

Cleavage site: 646   Tag abundance: 1   Weighted abundance: 0.333   Category: 4
sRNA abundance: 1   Alignment score: 3   MFE ratio: 0.877   p-value: 0.034

HORVU6Hr1G058100|HORVU6Hr1G058100.1||1365|1935

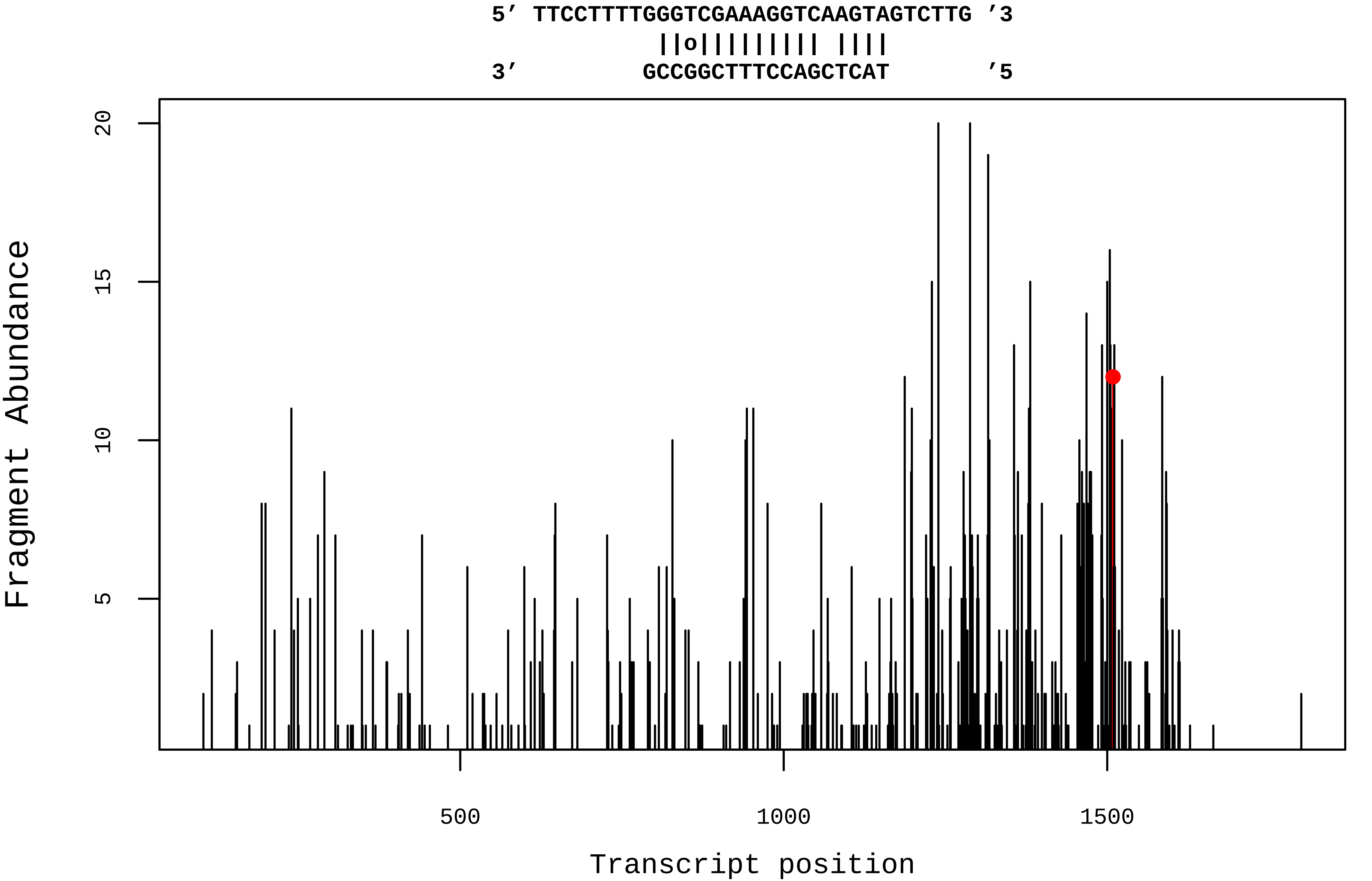

Cleavage site: 1509 Tag abundance: 12 Weighted abundance: 12 Category: 2
sRNA abundance: 1 Alignment score: 3.5 MFE ratio: 0.727 p-value: 0.037

HORVU7Hr1G122690|HORVU7Hr1G122690.20||702|1400

```
5' TGGTGGATCCGGCTATGGCAGTGGCGGTGGCT '3
         o ||o|||||||||| |||||
3'       GCGCTGATACCGTC-CCGCC       '5
```

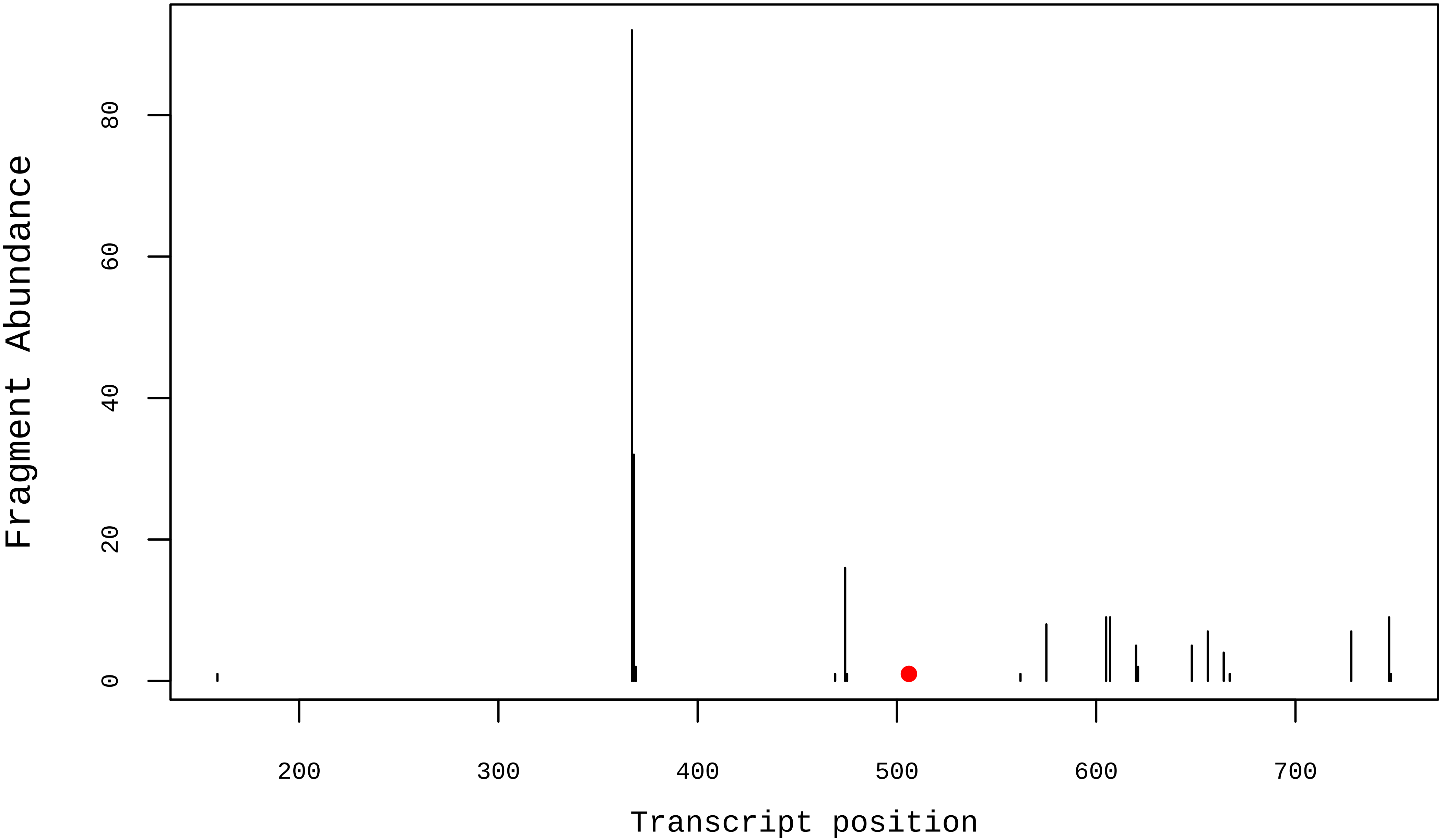

Cleavage site: 506 Tag abundance: 1 Weighted abundance: 0.25 Category: 4
sRNA abundance: 1 Alignment score: 4 MFE ratio: 0.771 p-value: 0.02

HORVU7Hr1G122690|HORVU7Hr1G122690.21||606|1606

```
5' TGGTGGATCCGGCTATGGCAGTGGCGGTGGCT '3
           o ||o||||||||| |||||
3'         GCGCTGATACCGTC-CCGCC        '5
```

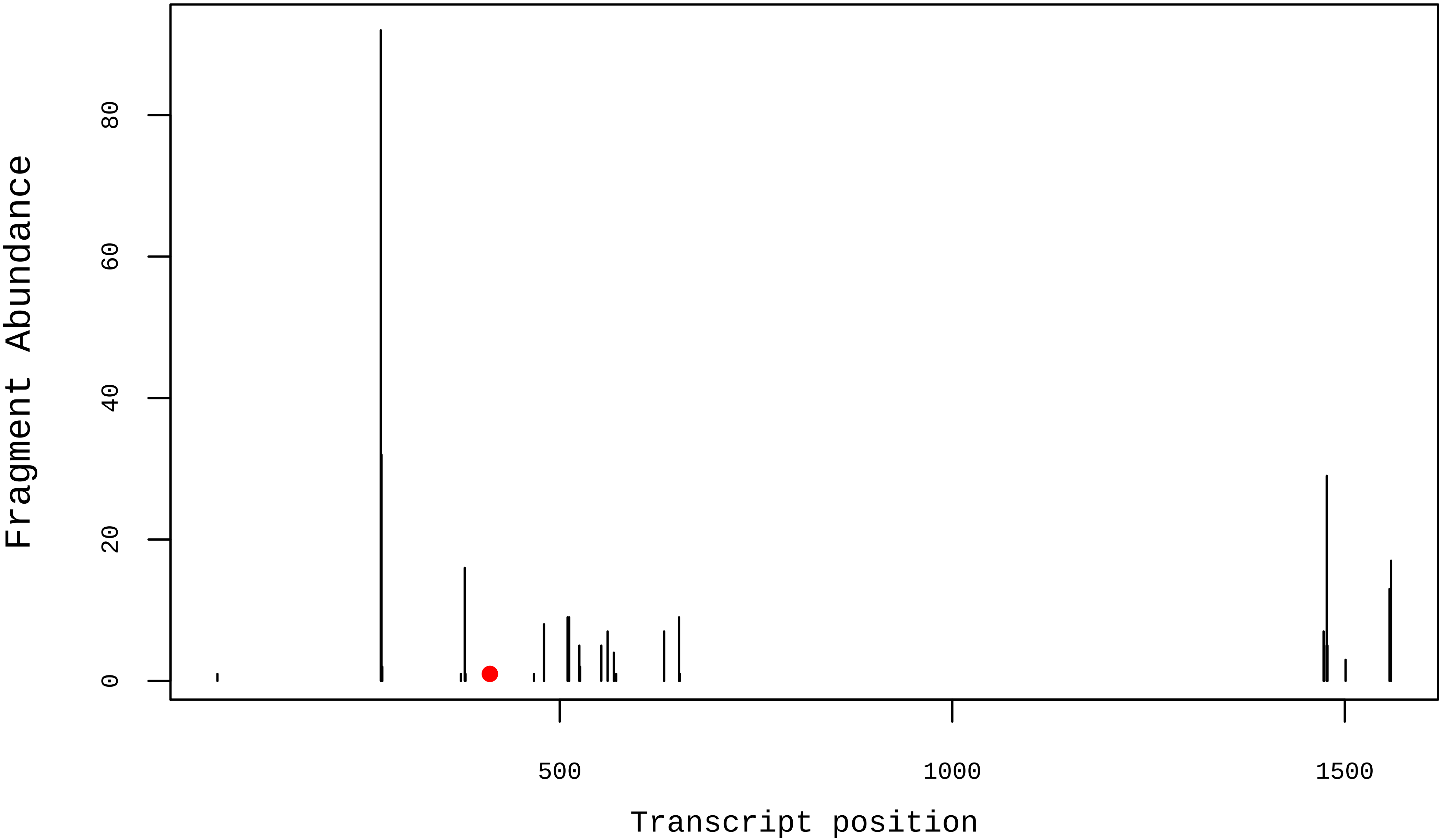

Cleavage site: 411 Tag abundance: 1 Weighted abundance: 0.25 Category: 4
sRNA abundance: 1 Alignment score: 4 MFE ratio: 0.771 p-value: 0.018

HORVU7Hr1G122690|HORVU7Hr1G122690.22||606|1859

```
5' TGGTGGATCCGGCTATGGCAGTGGCGGTGGCT '3
          o ||o||||||||| |||||
3'        GCGCTGATACCGTC-CCGCC       '5
```

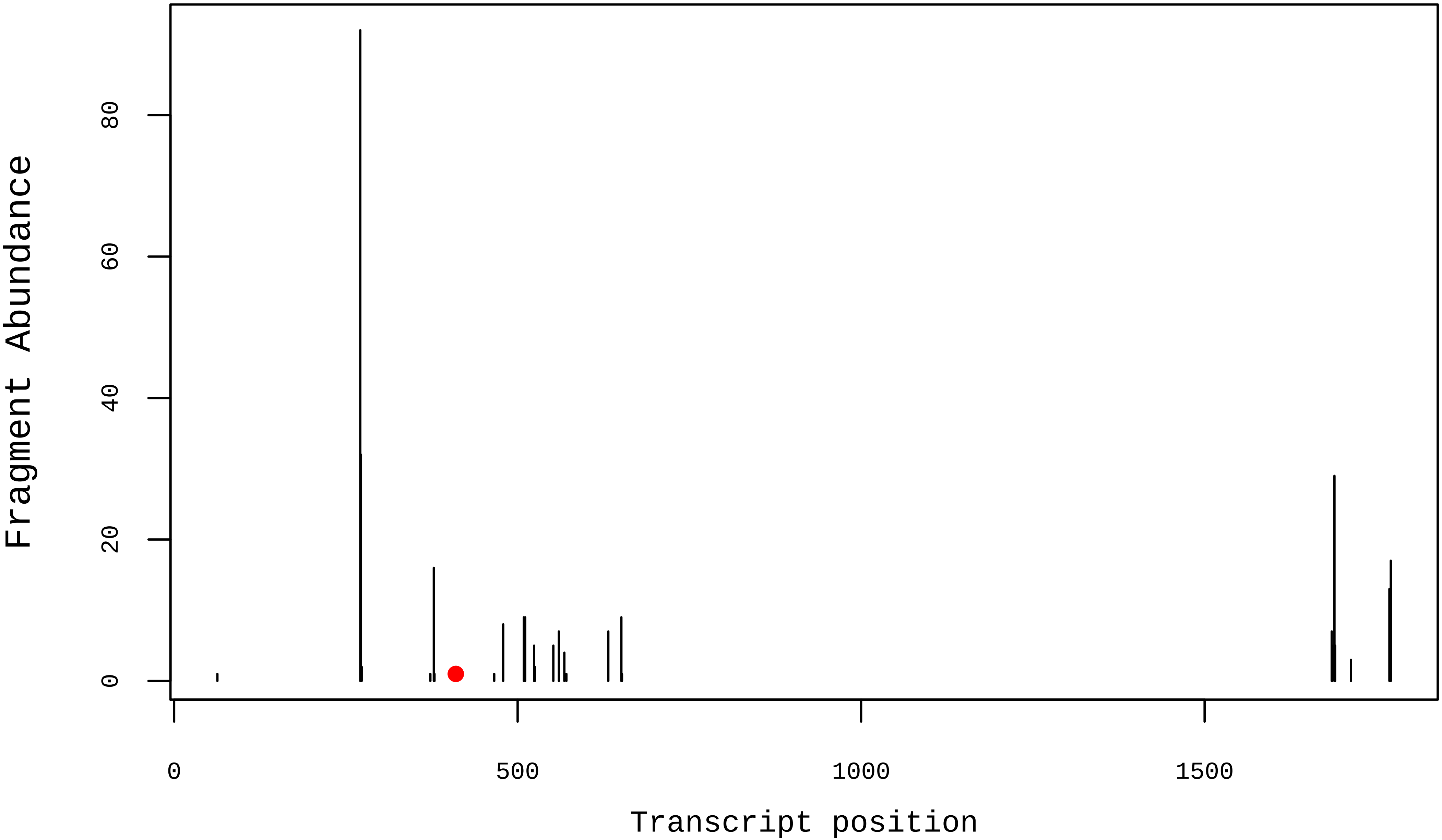

Cleavage site: 410   Tag abundance: 1   Weighted abundance: 0.25   Category: 4
sRNA abundance: 1   Alignment score: 4   MFE ratio: 0.771   p-value: 0.015

HORVU7Hr1G122690|HORVU7Hr1G122690.23||606|1379

```
5' TGGTGGATCCGGCTATGGCAGTGGCGGTGGCT '3
           o ||o|||||||||| |||||
3'         GCGCTGATACCGTC-CCGCC      '5
```

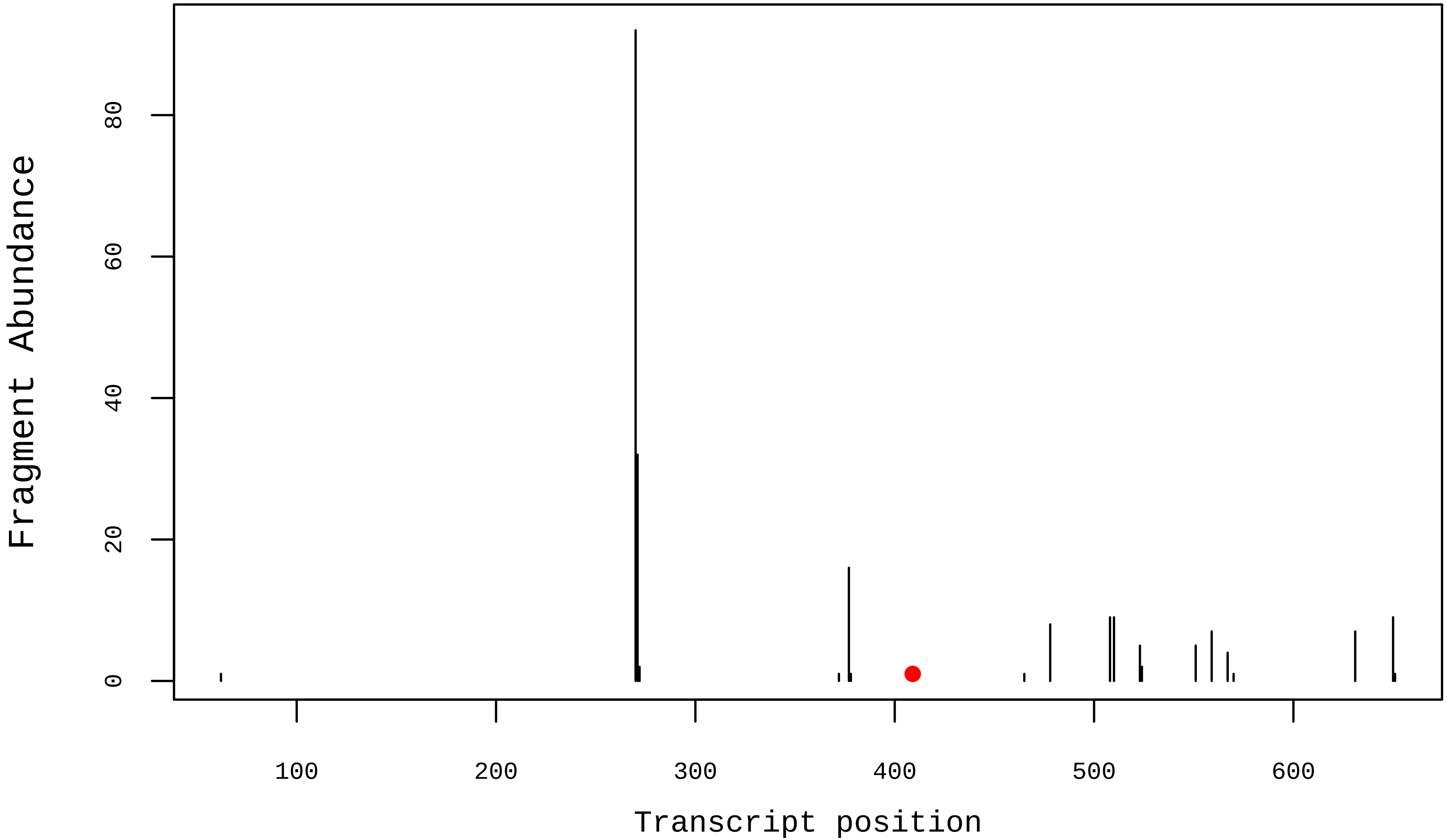

Cleavage site: 409 Tag abundance: 1 Weighted abundance: 0.25 Category: 4
sRNA abundance: 1 Alignment score: 4 MFE ratio: 0.771 p-value: 0.02

HORVU1Hr1G014610|HORVU1Hr1G014610.3||612|1281

```
5' GCAACTCGGCAGTTTCAGCGCAGTGCGCTCGG '3
          ||| ||| |||||||||||
3'        TCCGGCAACGTCGCGTCACG       '5
```

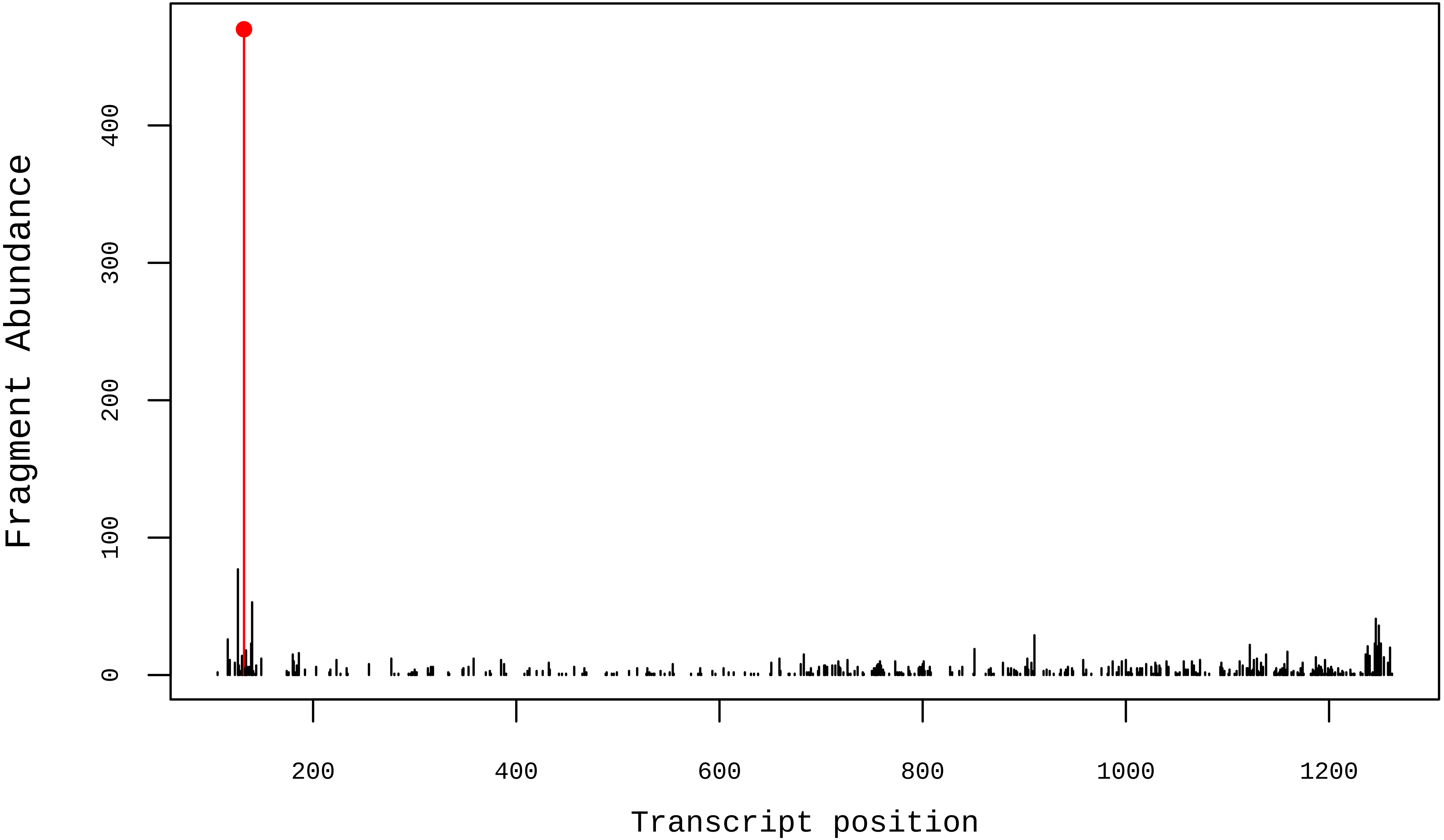

Cleavage site: 132 Tag abundance: 470 Weighted abundance: 117.5 Category: 0
sRNA abundance: 1 Alignment score: 4 MFE ratio: 0.701 p-value: 0.001

HORVU4Hr1G083870|HORVU4Hr1G083870.1||1746|3479

```
5' ATCTCGATGACCTGATCAGCGGCGGAGCCAGC '3
         |||| | |||||o|||||||
3'       TACTTG-CTAGTTGCCGCCT       '5
```

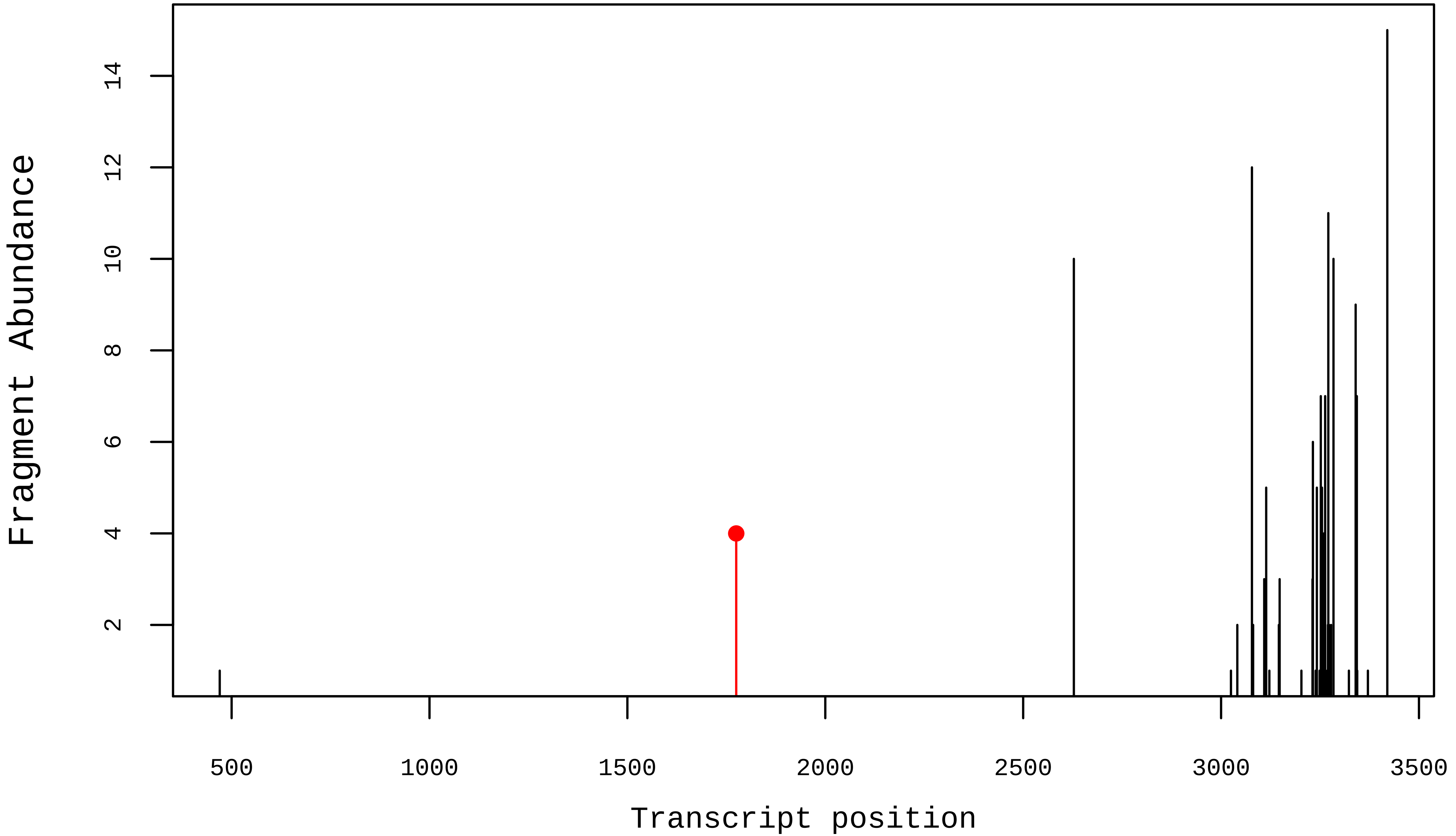

Cleavage site: 1775   Tag abundance: 4   Weighted abundance: 0.8   Category: 3
sRNA abundance: 1   Alignment score: 3   MFE ratio: 0.79   p-value: 0.023

HORVU4Hr1G083870|HORVU4Hr1G083870.3||1746|3378

```
5' ATCTCGATGACCTGATCAGCGGCGGAGCCAGC '3
         |||| | |||||o|||||||
3'       TACTTG-CTAGTTGCCGCCT       '5
```

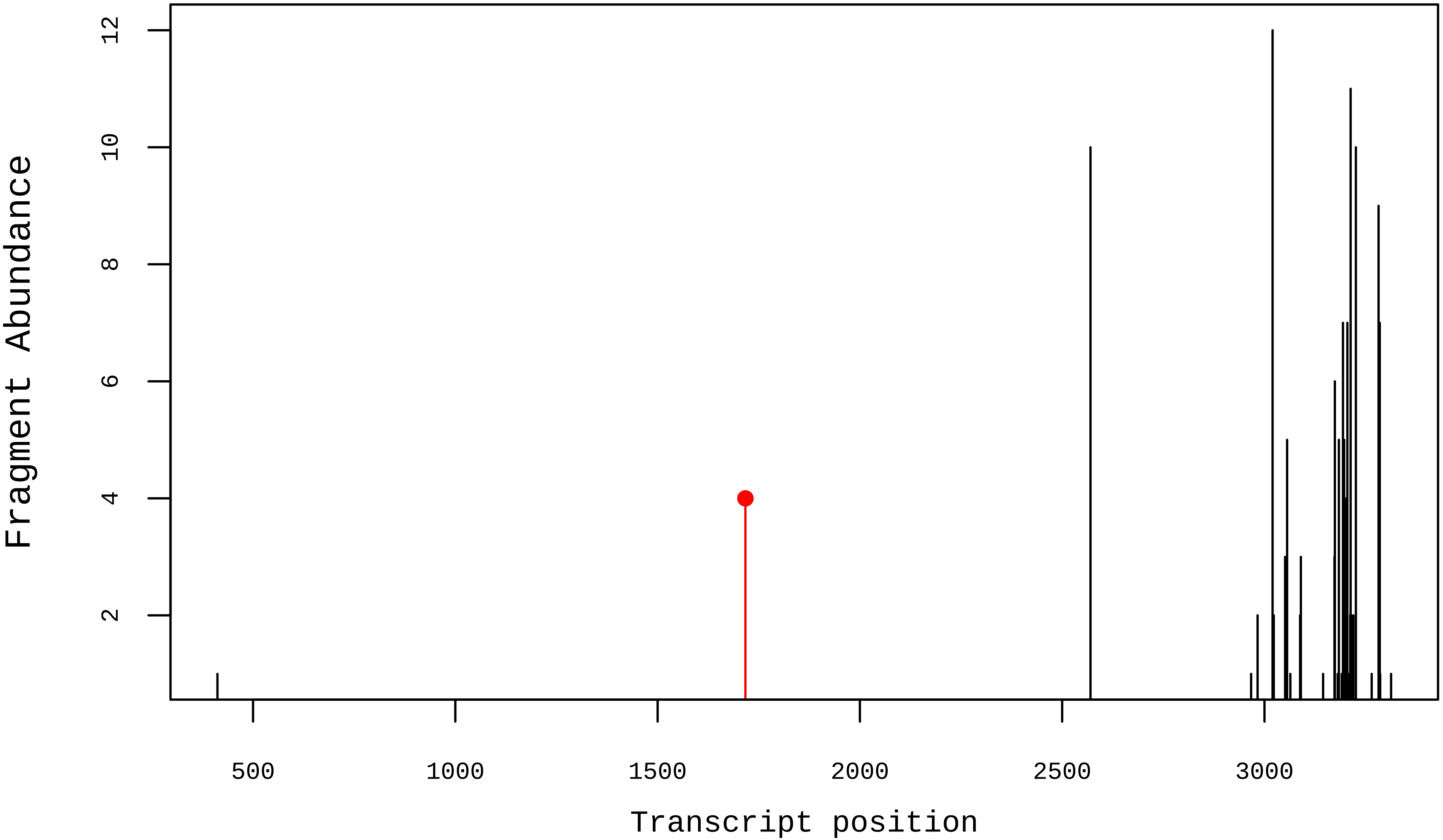

Cleavage site: 1717   Tag abundance: 4   Weighted abundance: 0.8   Category: 3
sRNA abundance: 1   Alignment score: 3   MFE ratio: 0.79   p-value: 0.024

HORVU4Hr1G083870|HORVU4Hr1G083870.4||1746|3334

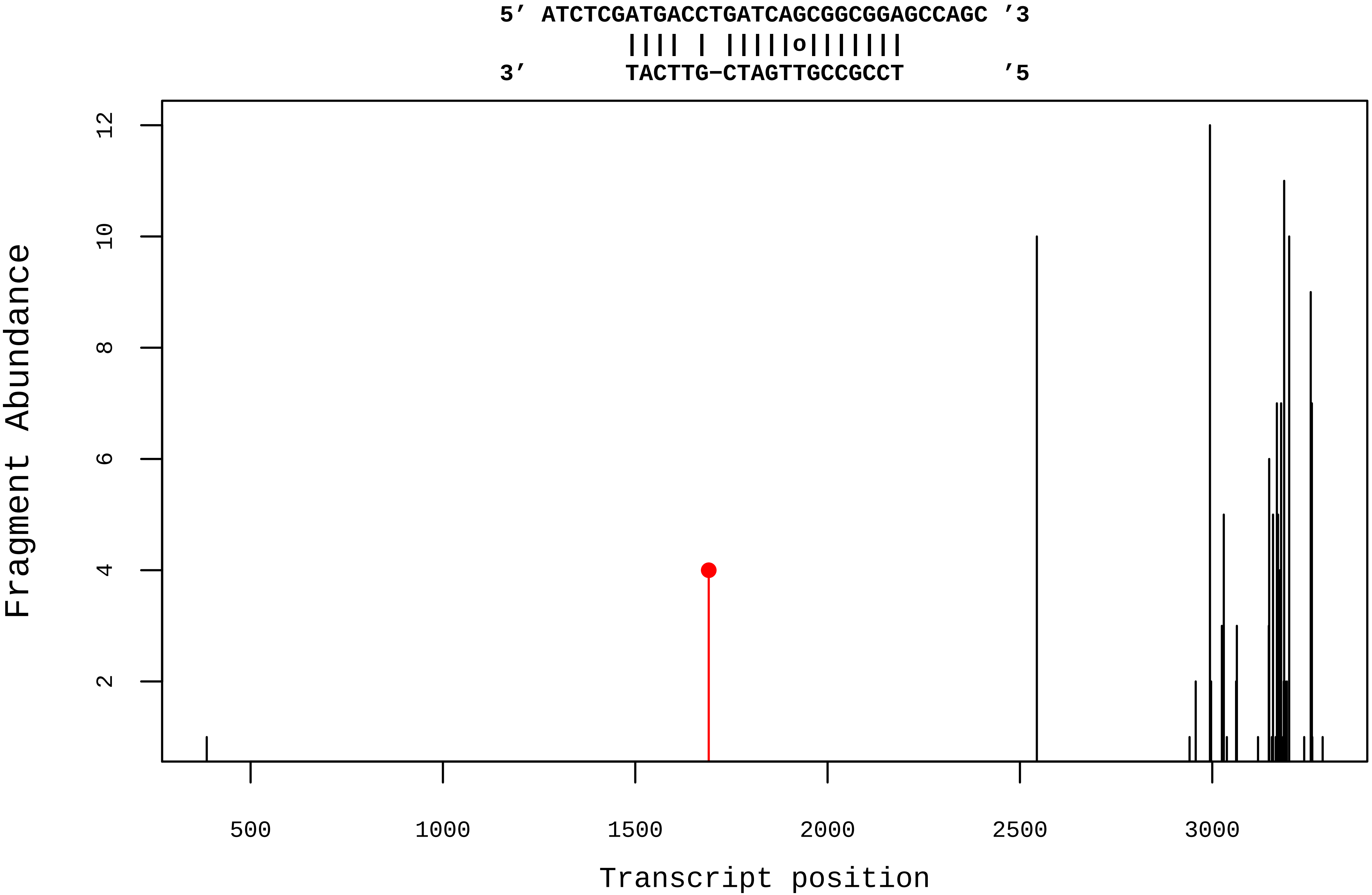

Cleavage site: 1691 Tag abundance: 4 Weighted abundance: 0.8 Category: 3
sRNA abundance: 1 Alignment score: 3 MFE ratio: 0.79 p-value: 0.024

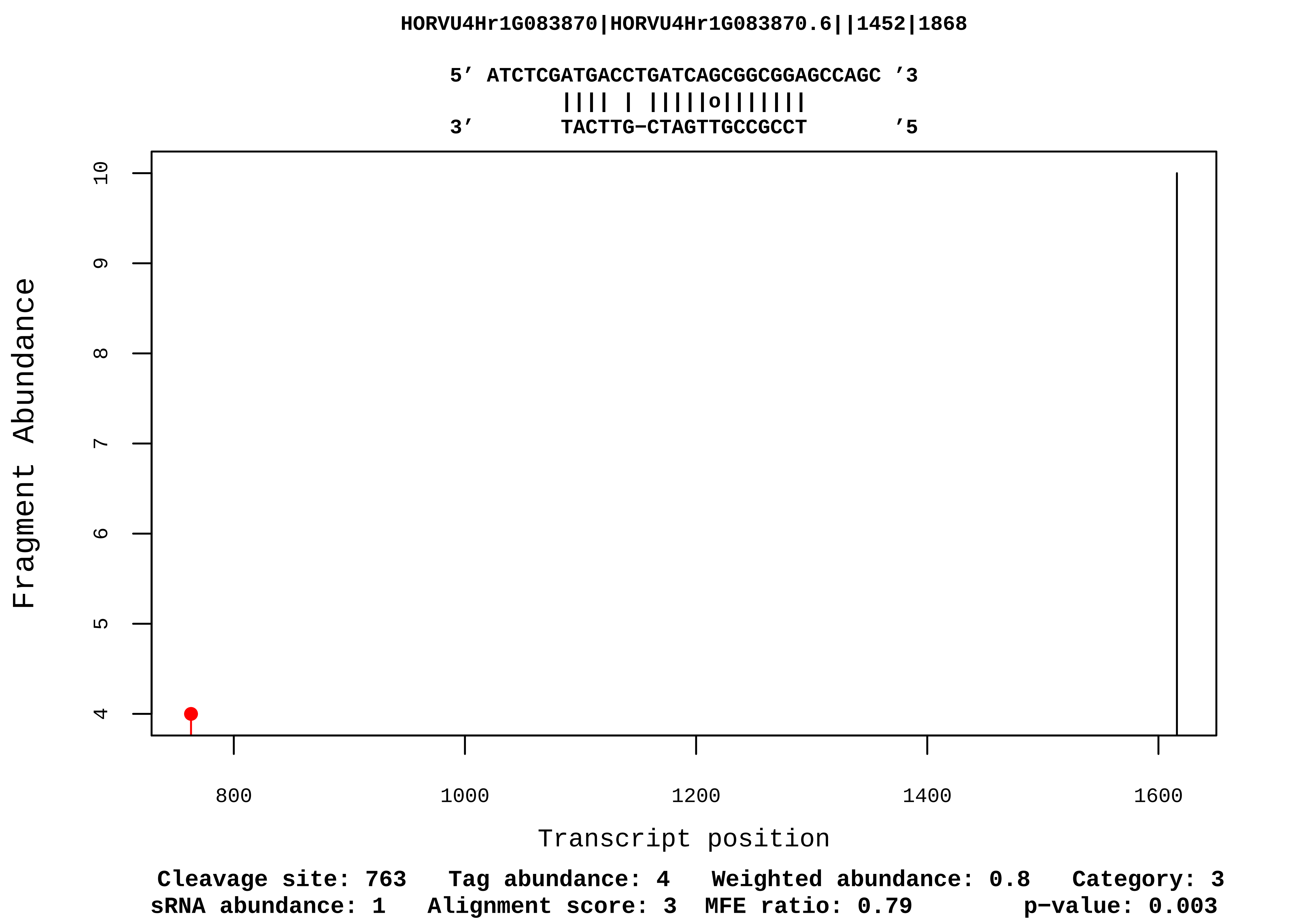

HORVU4Hr1G083870|HORVU4Hr1G083870.6||1452|1868
5' ATCTCGATGACCTGATCAGCGGCGGAGCCAGC '3
|||| | |||||o|||||||
3' TACTTG-CTAGTTGCCGCCT '5
Fragment Abundance
4
5
6
7
8
9
10
800
1000
1200
1400
1600
Transcript position
Cleavage site: 763 Tag abundance: 4 Weighted abundance: 0.8 Category: 3
sRNA abundance: 1 Alignment score: 3 MFE ratio: 0.79 p-value: 0.003

HORVU4Hr1G083870|HORVU4Hr1G083870.7||1440|1867

```
5' ATCTCGATGACCTGATCAGCGGCGGAGCCAGC '3
         |||| | |||||o|||||||
3'       TACTTG-CTAGTTGCCGCCT       '5
```

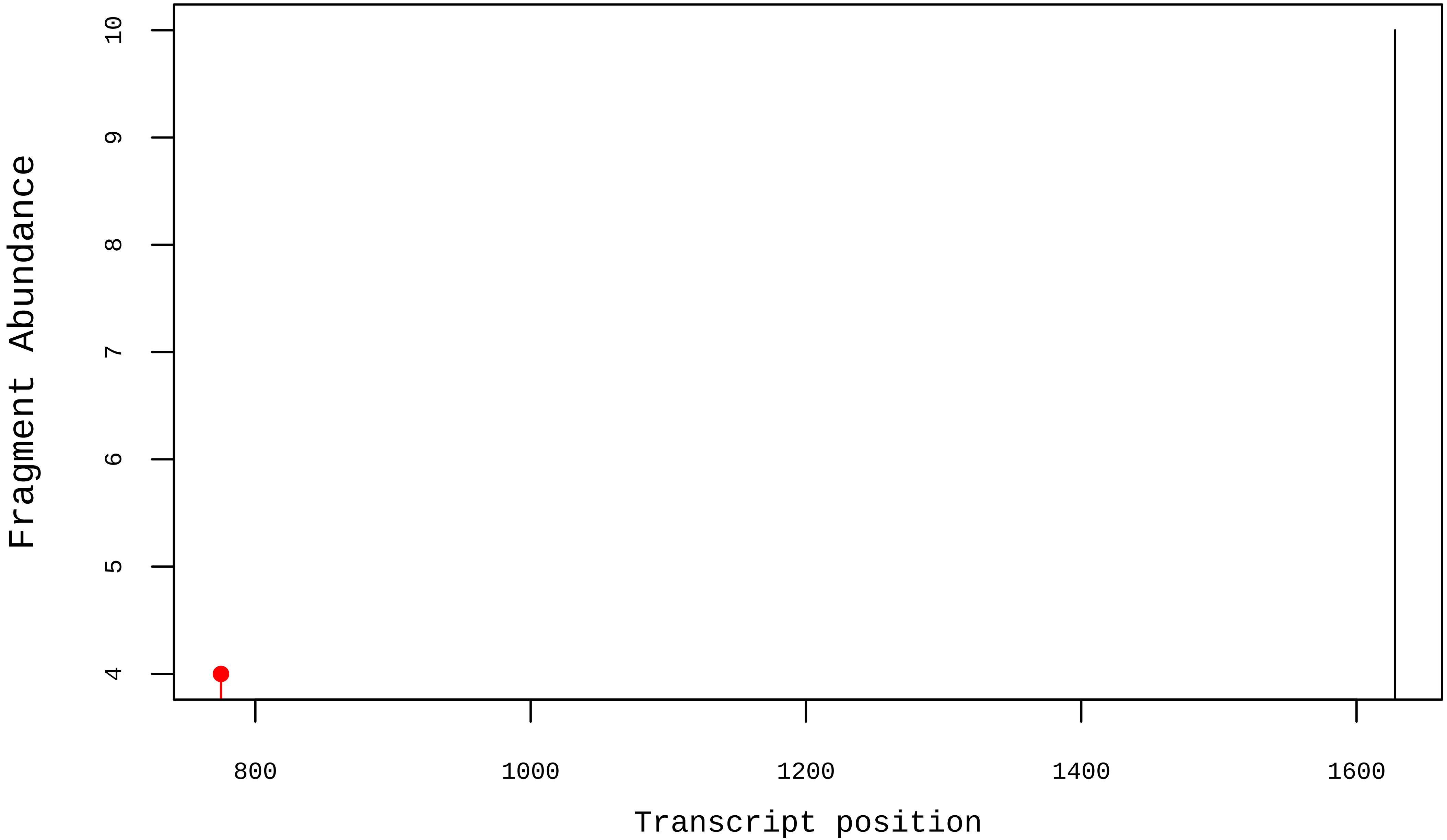

Cleavage site: 775 Tag abundance: 4 Weighted abundance: 0.8 Category: 3
sRNA abundance: 1 Alignment score: 3 MFE ratio: 0.79 p-value: 0.003

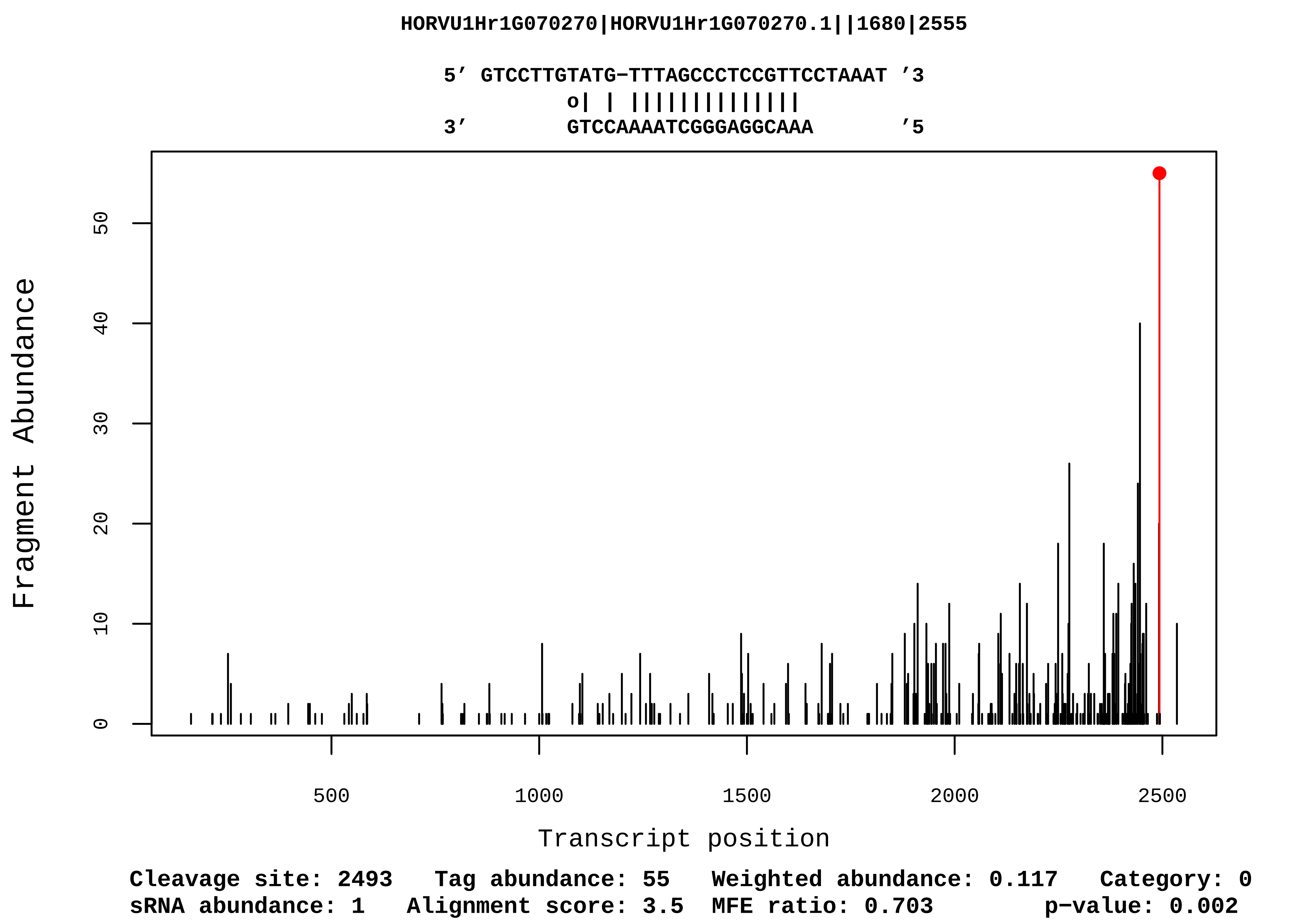

HORVU1Hr1G070270|HORVU1Hr1G070270.1||1680|2555
5' GTCCTTGTATG-TTTAGCCCTCCGTTCCTAAAT '3
o| | ||||||||||||||
3' GTCCAAAATCGGGAGGCAAA '5
Fragment Abundance
0
10
20
30
40
50
500
1000
1500
2000
2500
Transcript position
Cleavage site: 2493 Tag abundance: 55 Weighted abundance: 0.117 Category: 0
sRNA abundance: 1 Alignment score: 3.5 MFE ratio: 0.703 p-value: 0.002

HORVU1Hr1G070270|HORVU1Hr1G070270.2||1680|2333

```
5' GTCCTTGTATG-TTTAGCCCTCCGTTCCTAAAT '3
          o| | ||||||||||||||
3'        GTCCAAAATCGGGAGGCAAA       '5
```

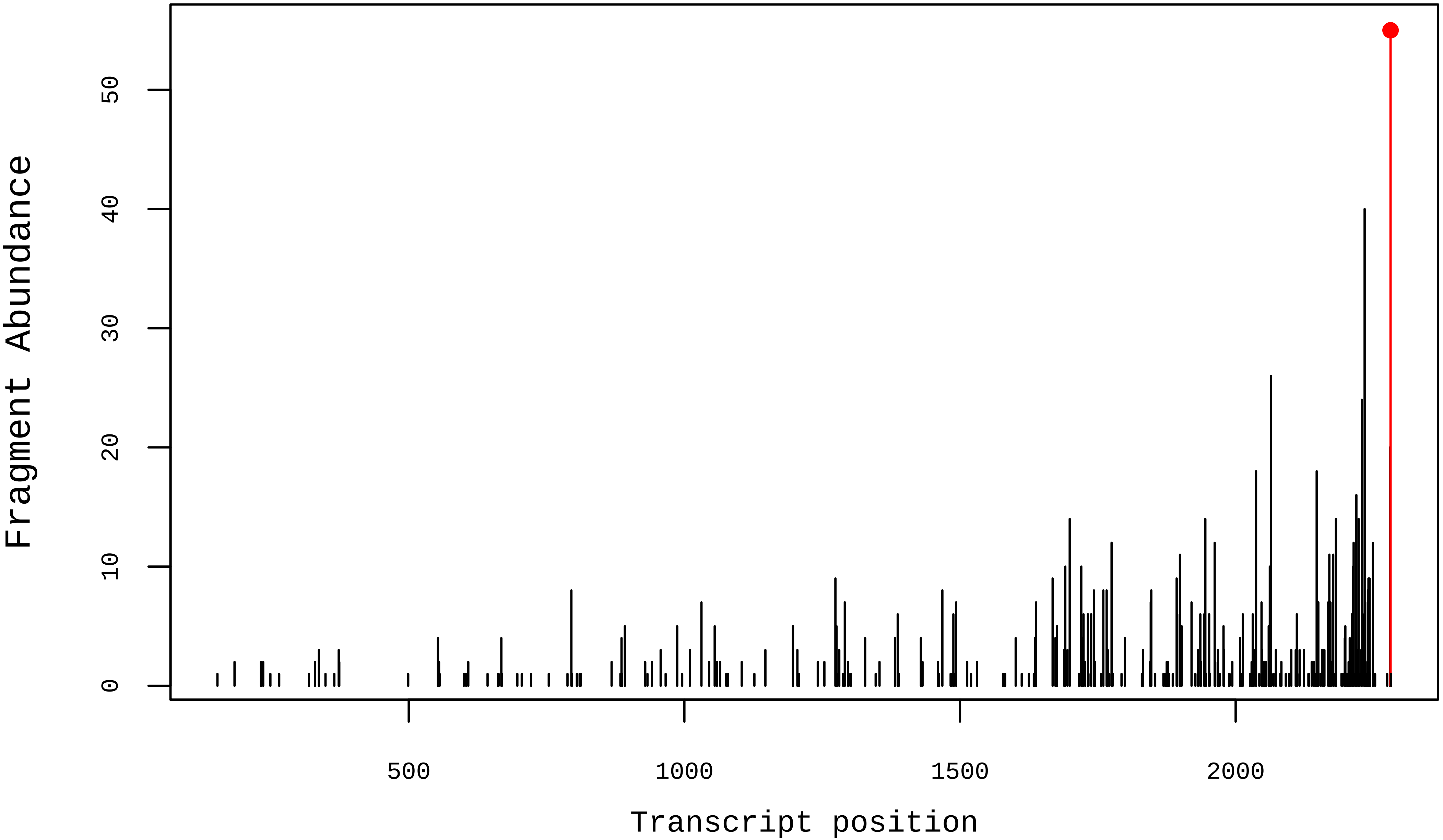

Cleavage site: 2281   Tag abundance: 55   Weighted abundance: 0.117   Category: 0
sRNA abundance: 1   Alignment score: 3.5   MFE ratio: 0.703   p-value: 0.002

HORVU1Hr1G070270|HORVU1Hr1G070270.3||1680|3936

```
5' GTCCTTGTATG-TTTAGCCCTCCGTTCCTAAAT '3
          o| | ||||||||||||||
3'        GTCCAAAATCGGGAGGCAAA        '5
```

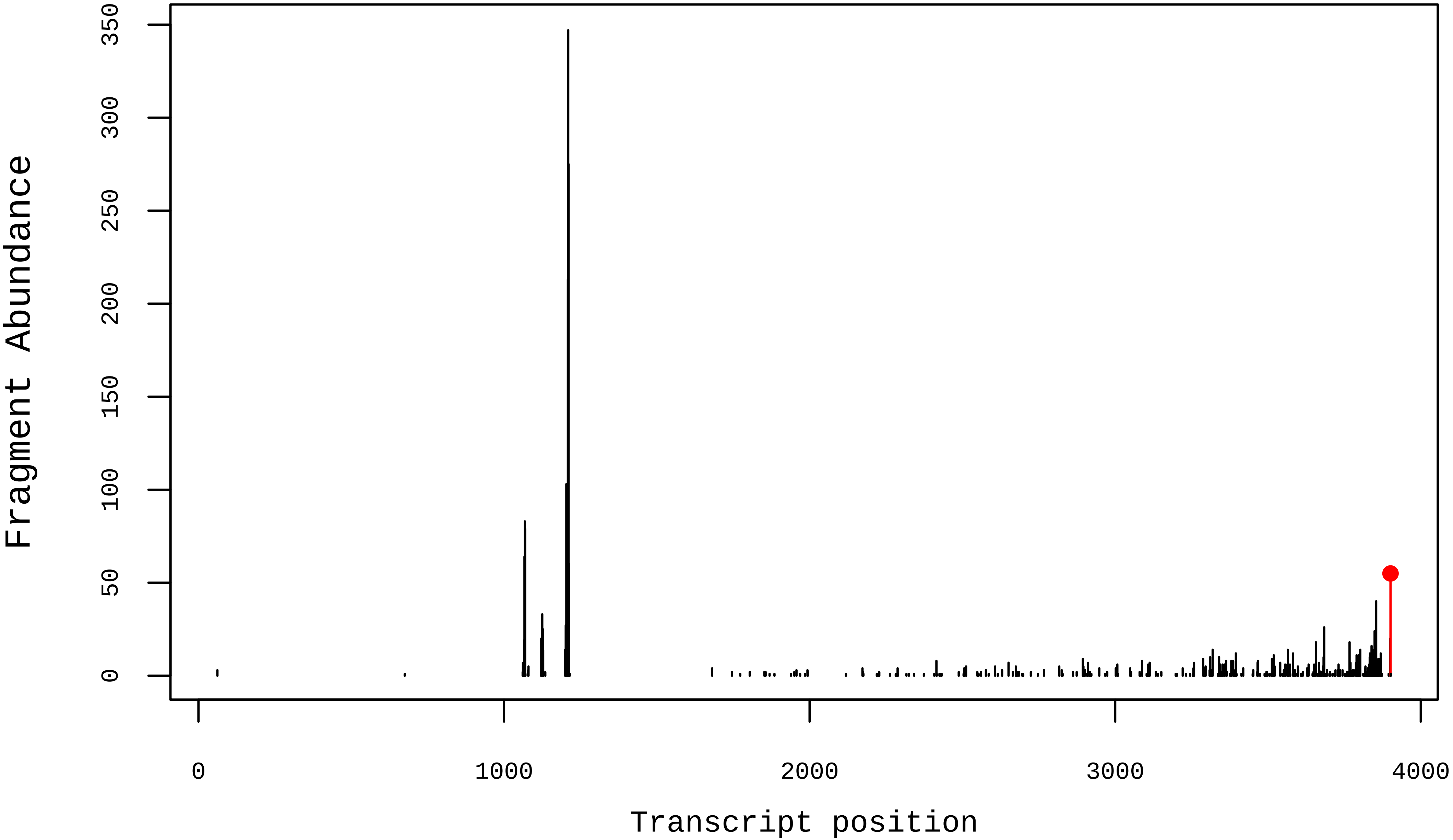

Cleavage site: 3901 Tag abundance: 55 Weighted abundance: 0.117 Category: 2
sRNA abundance: 1 Alignment score: 3.5 MFE ratio: 0.703 p-value: 0.033

HORVU3Hr1G065420|HORVU3Hr1G065420.1||1440|1976

```
5' TCGGTGCTGGAGTCGATCATGGCGGGTGTGCC '3
           || | ||||||||||||o
3'         CCACTGCTAGTACCGCCT         '5
```

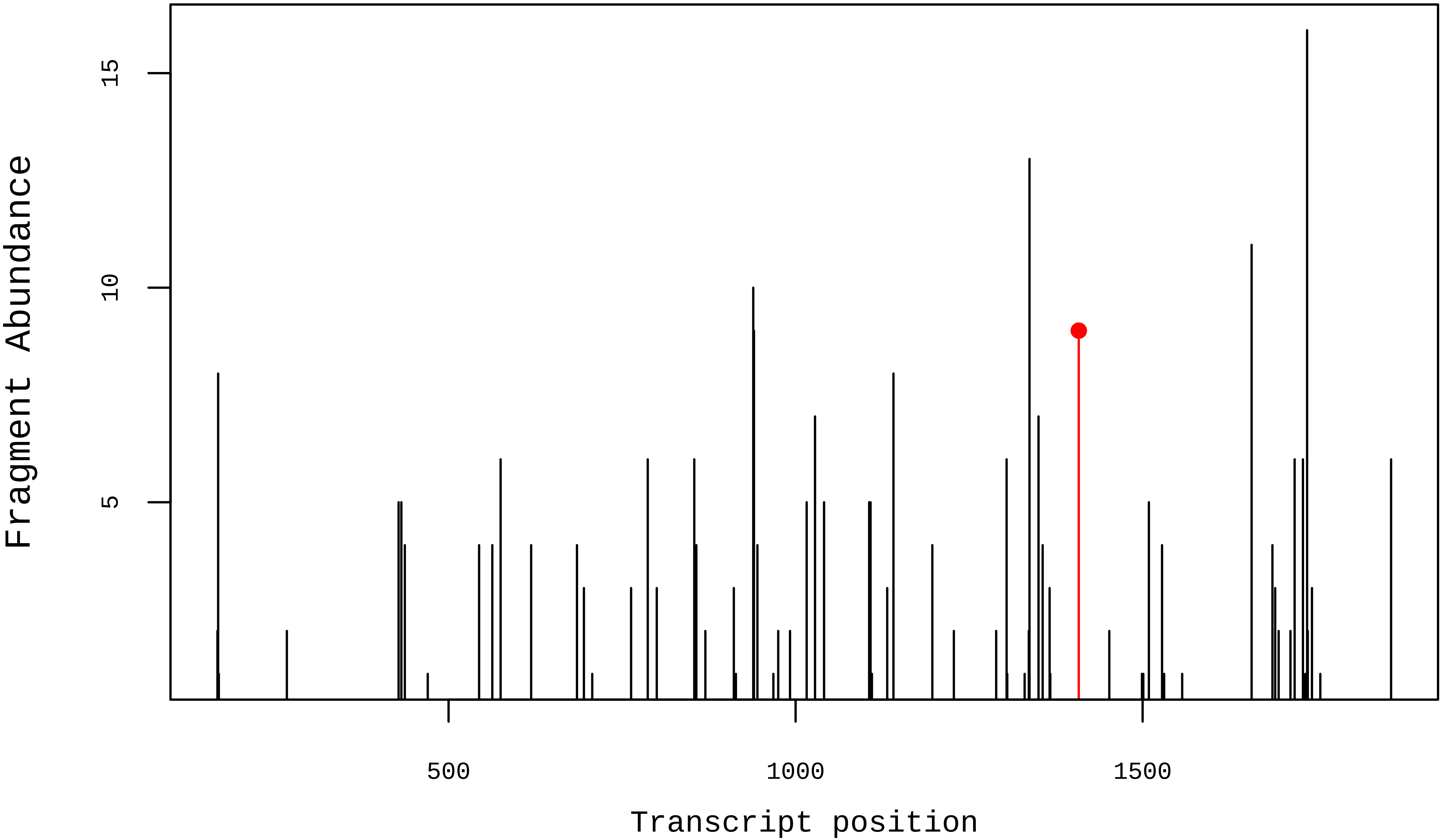

Cleavage site: 1408   Tag abundance: 9   Weighted abundance: 9   Category: 2
sRNA abundance: 1   Alignment score: 2.5   MFE ratio: 0.733   p-value: 0.012

HORVU0Hr1G003660|HORVU0Hr1G003660.1||297|1955

```
5′ GCCGGCCGAAGGGTCGAGTAGGTCGGTGCTCG ′3
      ||| ||||||||||||||||| ||
3′    GCCAGCTTCCCAGCTCATCCACCC       ′5
```

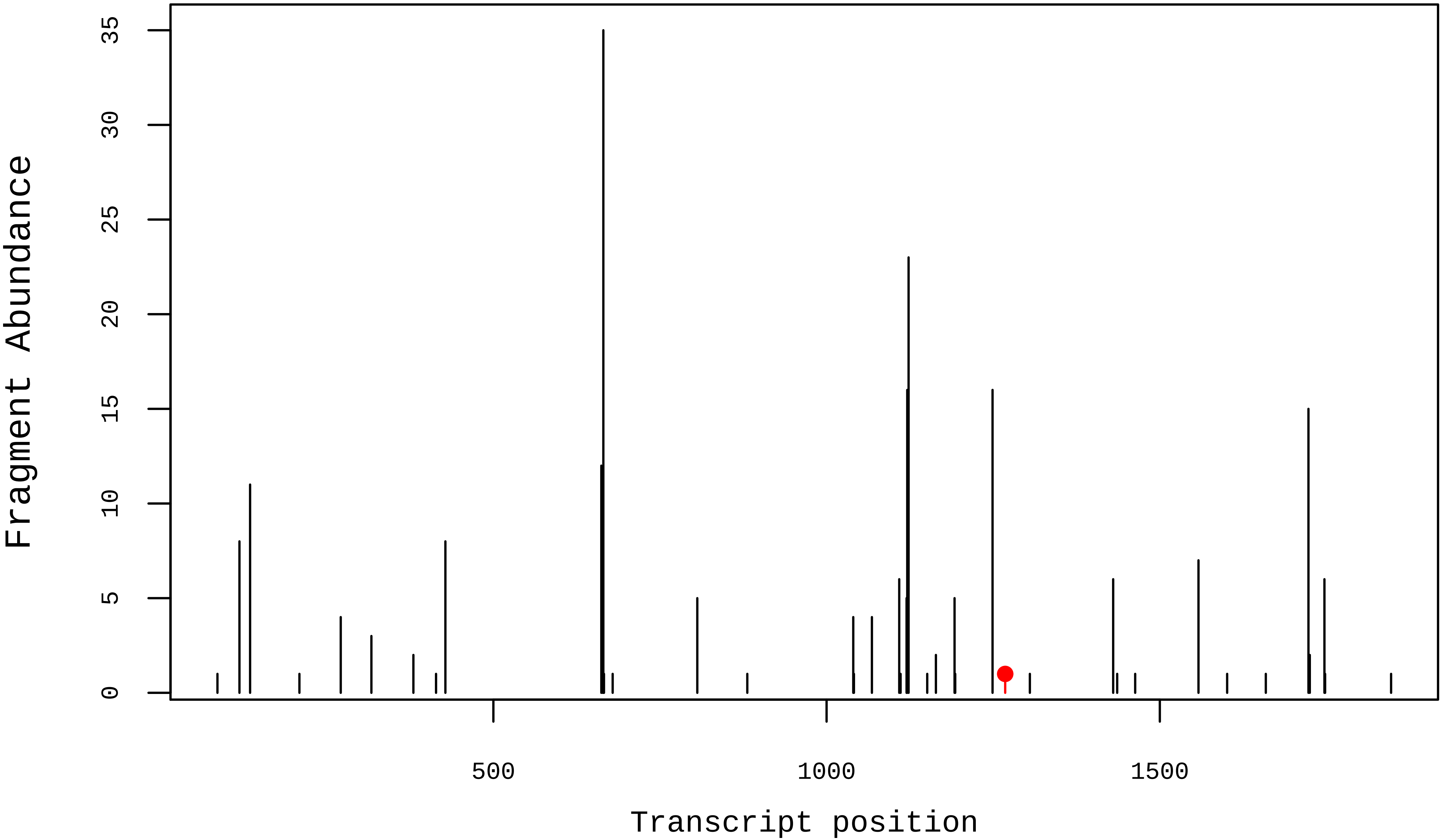

Cleavage site: 1268   Tag abundance: 1   Weighted abundance: 0.143   Category: 4
sRNA abundance: 1   Alignment score: 3   MFE ratio: 0.775   p-value: 0.046

HORVU0Hr1G023930|HORVU0Hr1G023930.3||396|2180

```
5' GTCGGCGGAAGGGTCGAGTAGGTCGGTGCTCG '3
       |||  ||||||||||||||||| ||
3'    GCCAGCTTCCCAGCTCATCCACCC       '5
```

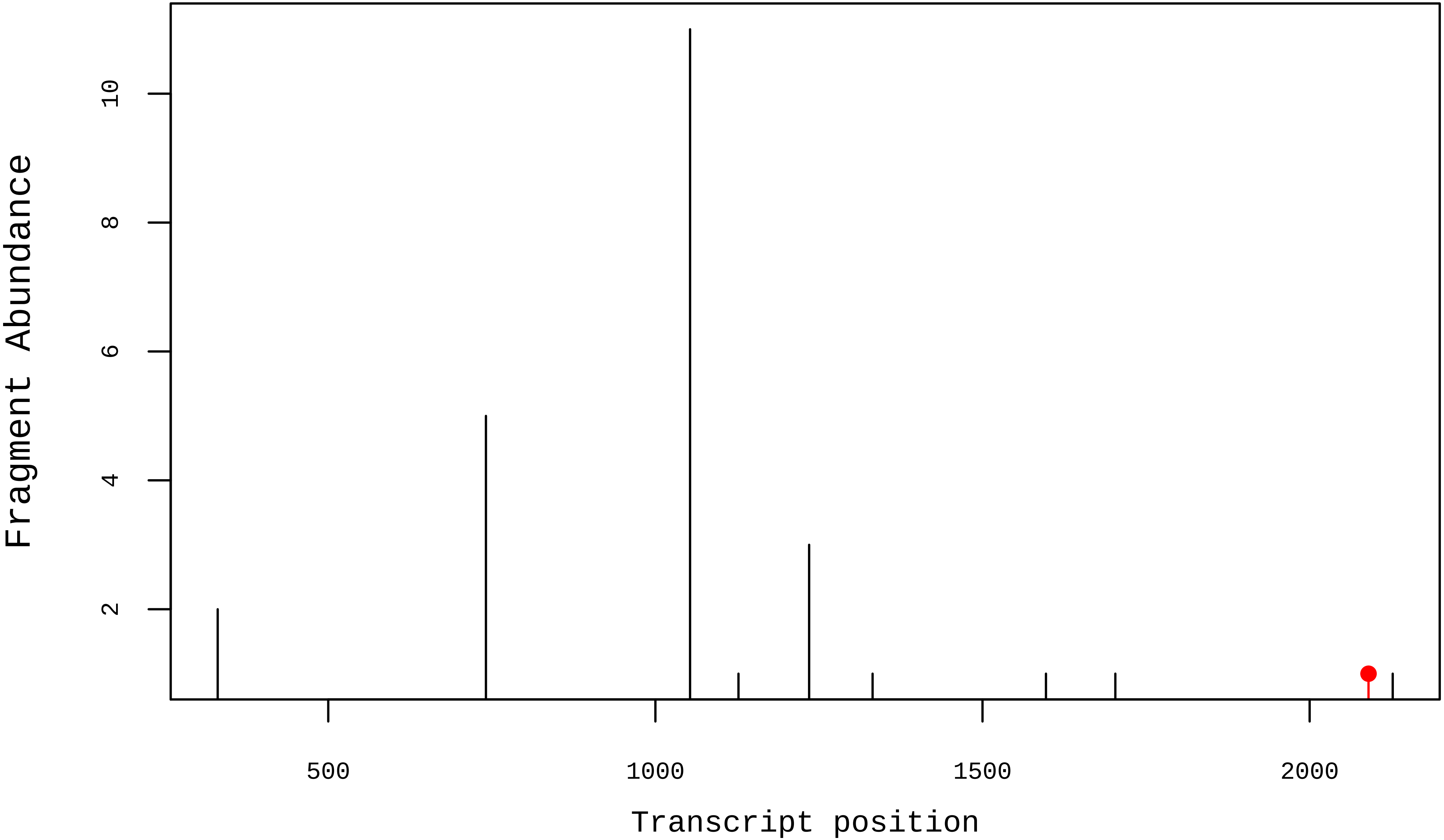

Cleavage site: 2090   Tag abundance: 1   Weighted abundance: 0.143   Category: 4
sRNA abundance: 1   Alignment score: 4   MFE ratio: 0.746   p-value: 0.017

HORVU2Hr1G072960|HORVU2Hr1G072960.1||600|3353

```
5' GCCGGCCGAAGGGTCGAGTAGGTCGGTGCTCG '3
       ||| ||||||||||||||||| ||
3'     GCCAGCTTCCCAGCTCATCCACCC        '5
```

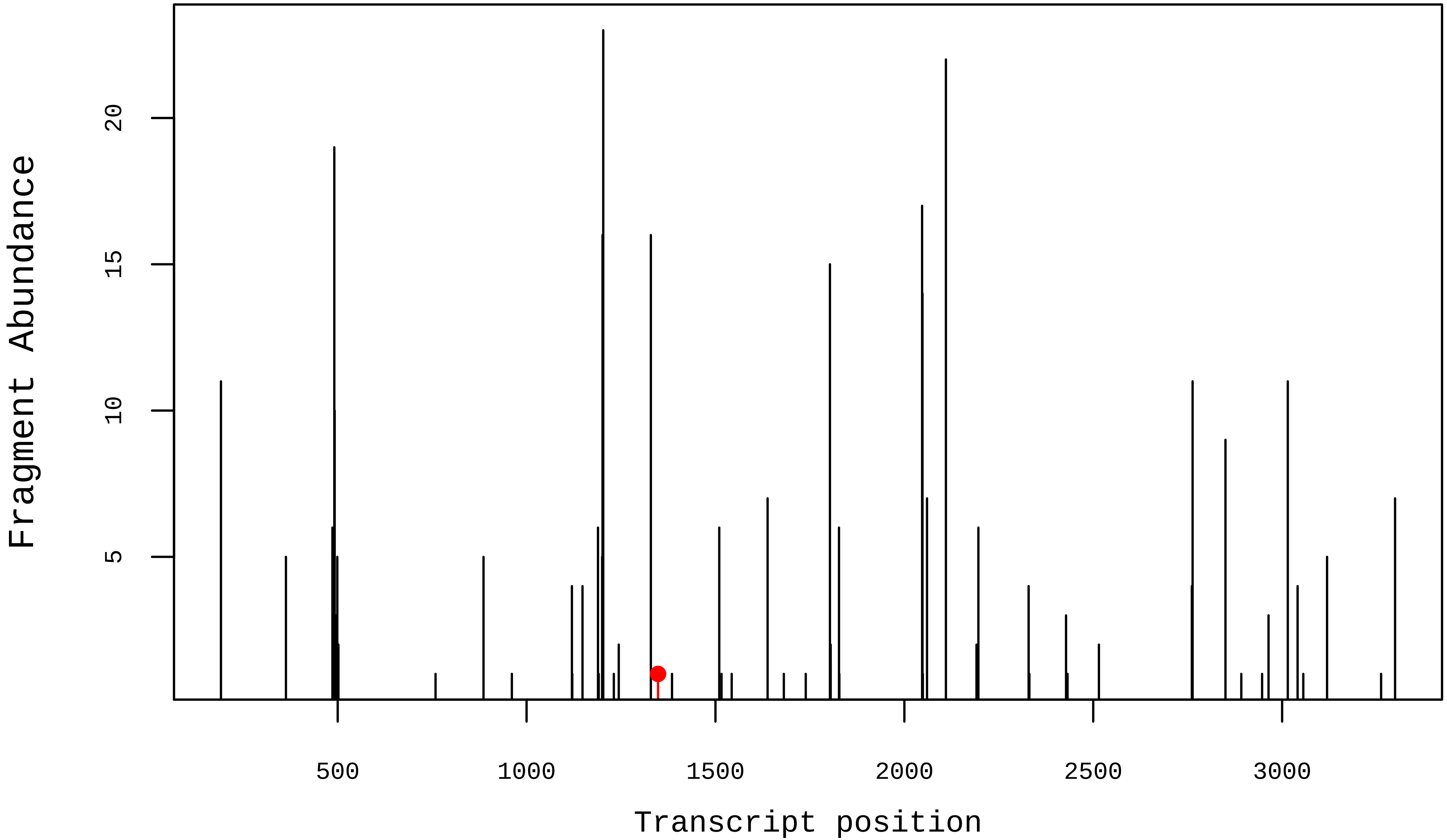

Cleavage site: 1348   Tag abundance: 1   Weighted abundance: 0.143   Category: 4
sRNA abundance: 1   Alignment score: 3   MFE ratio: 0.775   p-value: 0.03

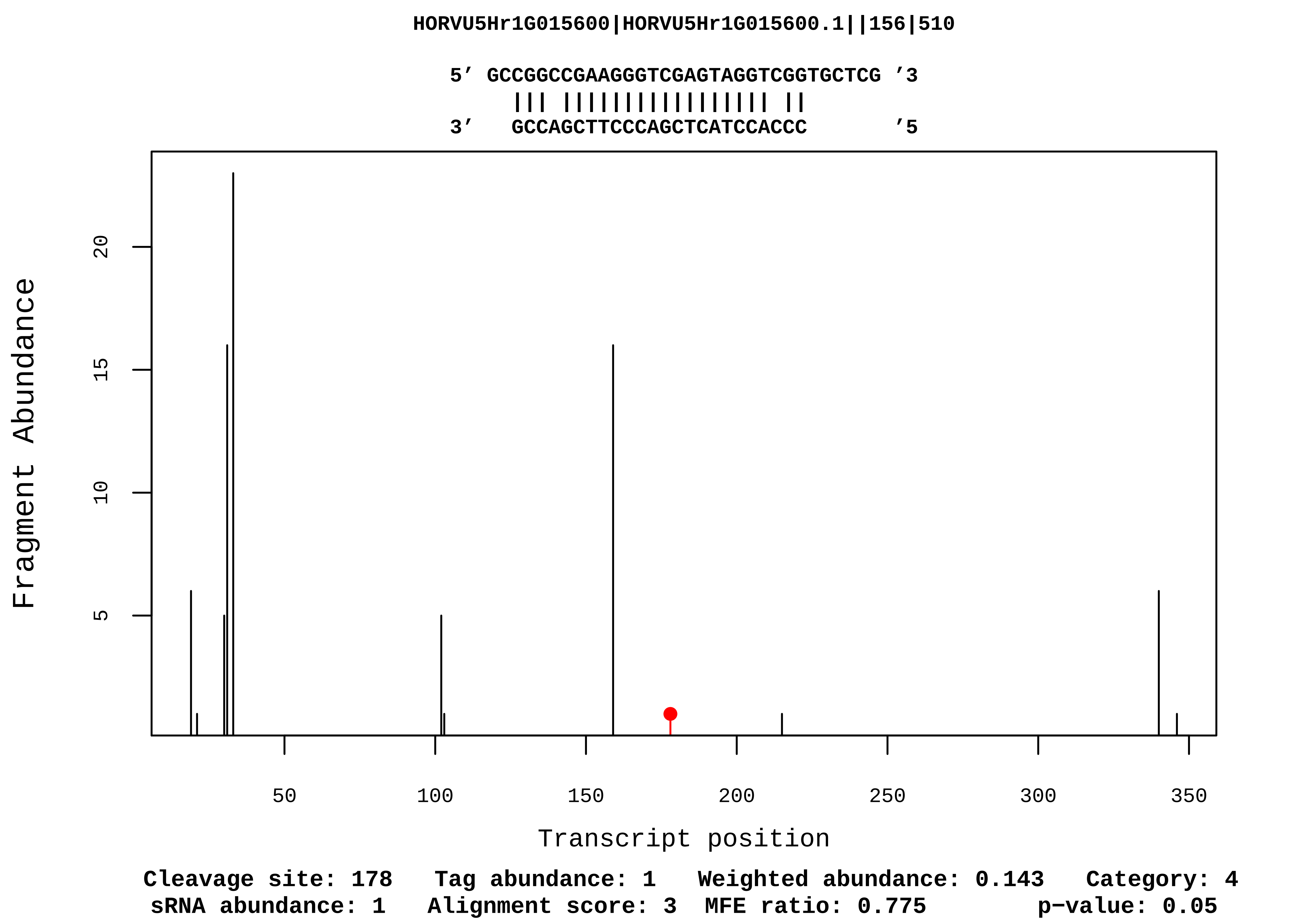
HORVU5Hr1G015600|HORVU5Hr1G015600.1||156|510
5' GCCGGCCGAAGGGTCGAGTAGGTCGGTGCTCG '3
3' GCCAGCTTCCCAGCTCATCCACCC '5
Fragment Abundance
5
10
15
20
50
100
150
200
250
300
350
Transcript position
Cleavage site: 178 Tag abundance: 1 Weighted abundance: 0.143 Category: 4
sRNA abundance: 1 Alignment score: 3 MFE ratio: 0.775 p-value: 0.05

HORVU5Hr1G015600|HORVU5Hr1G015600.2||231|617

```
5' GCCGGCCGAAGGGTCGAGTAGGTCGGTGCTCG '3
      ||| |||||||||||||||||| ||
3'    GCCAGCTTCCCAGCTCATCCACCC       '5
```

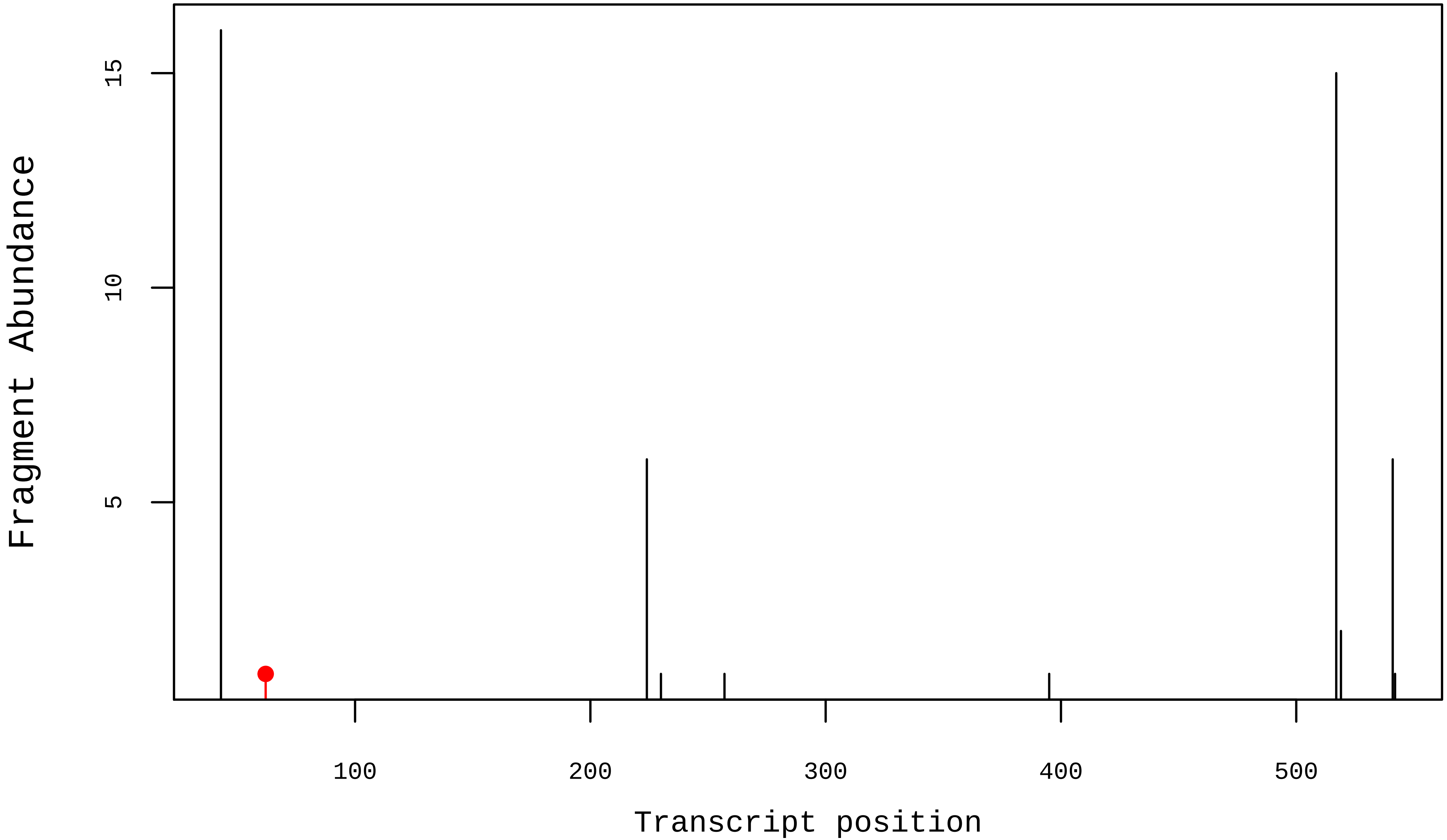

Cleavage site: 62 Tag abundance: 1 Weighted abundance: 0.143 Category: 4
sRNA abundance: 1 Alignment score: 3 MFE ratio: 0.775 p-value: 0.041

HORVU5Hr1G015600|HORVU5Hr1G015600.3||276|1709

```
5' GCCGGCCGAAGGGTCGAGTAGGTCGGTGCTCG '3
      ||| ||||||||||||||||| ||
3'    GCCAGCTTCCCAGCTCATCCACCC       '5
```

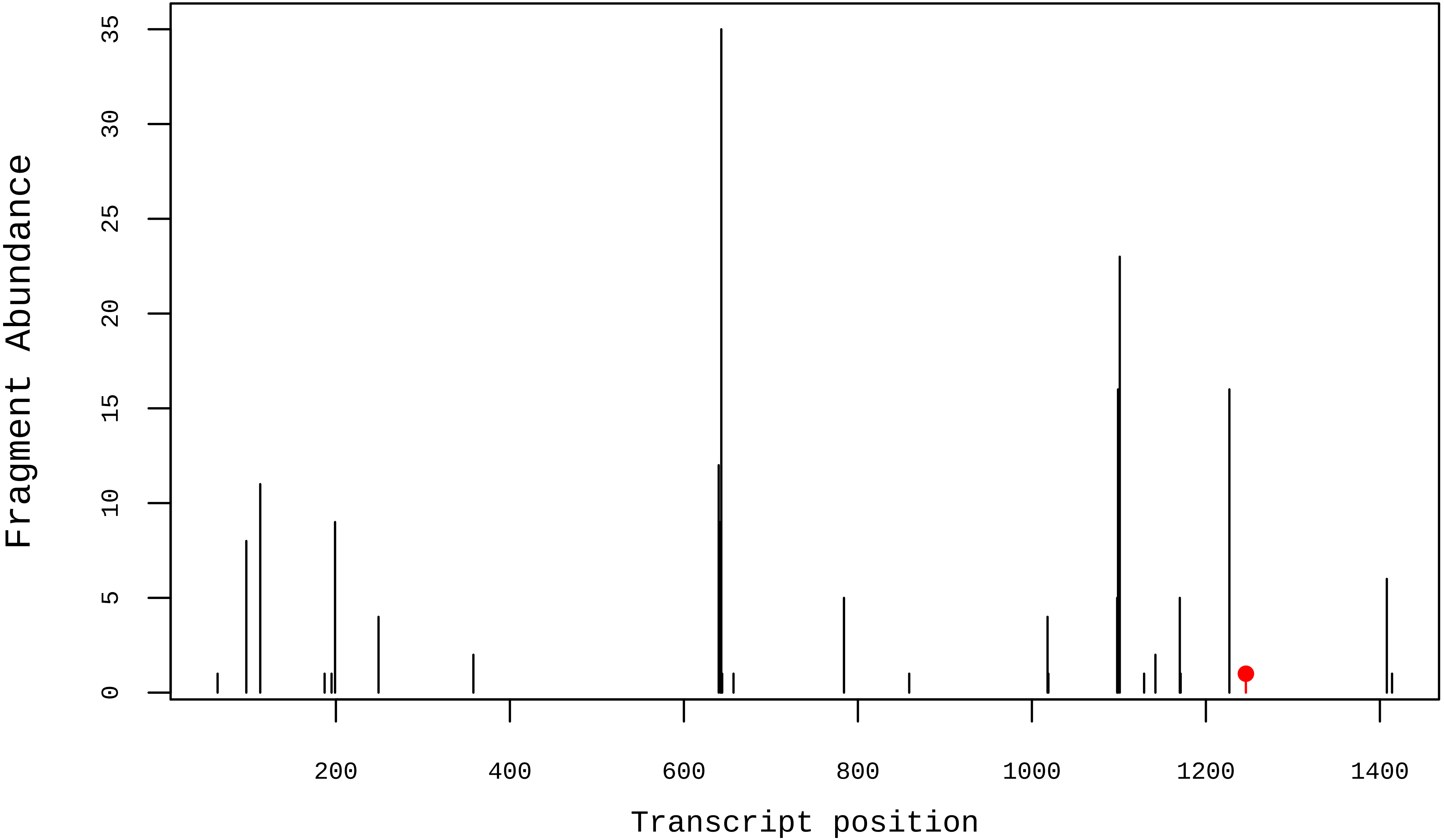

Cleavage site: 1246   Tag abundance: 1   Weighted abundance: 0.143   Category: 4
sRNA abundance: 1   Alignment score: 3   MFE ratio: 0.775   p-value: 0.032

HORVU7Hr1G116750|HORVU7Hr1G116750.1||870|1239

```
5' CATTCGATCGGGTCGAGTAGGTCGGCAGCAAT '3
         ||  ||||||||||||||||
3'       CTT-CCCAGCTCATCCAGCCA       '5
```

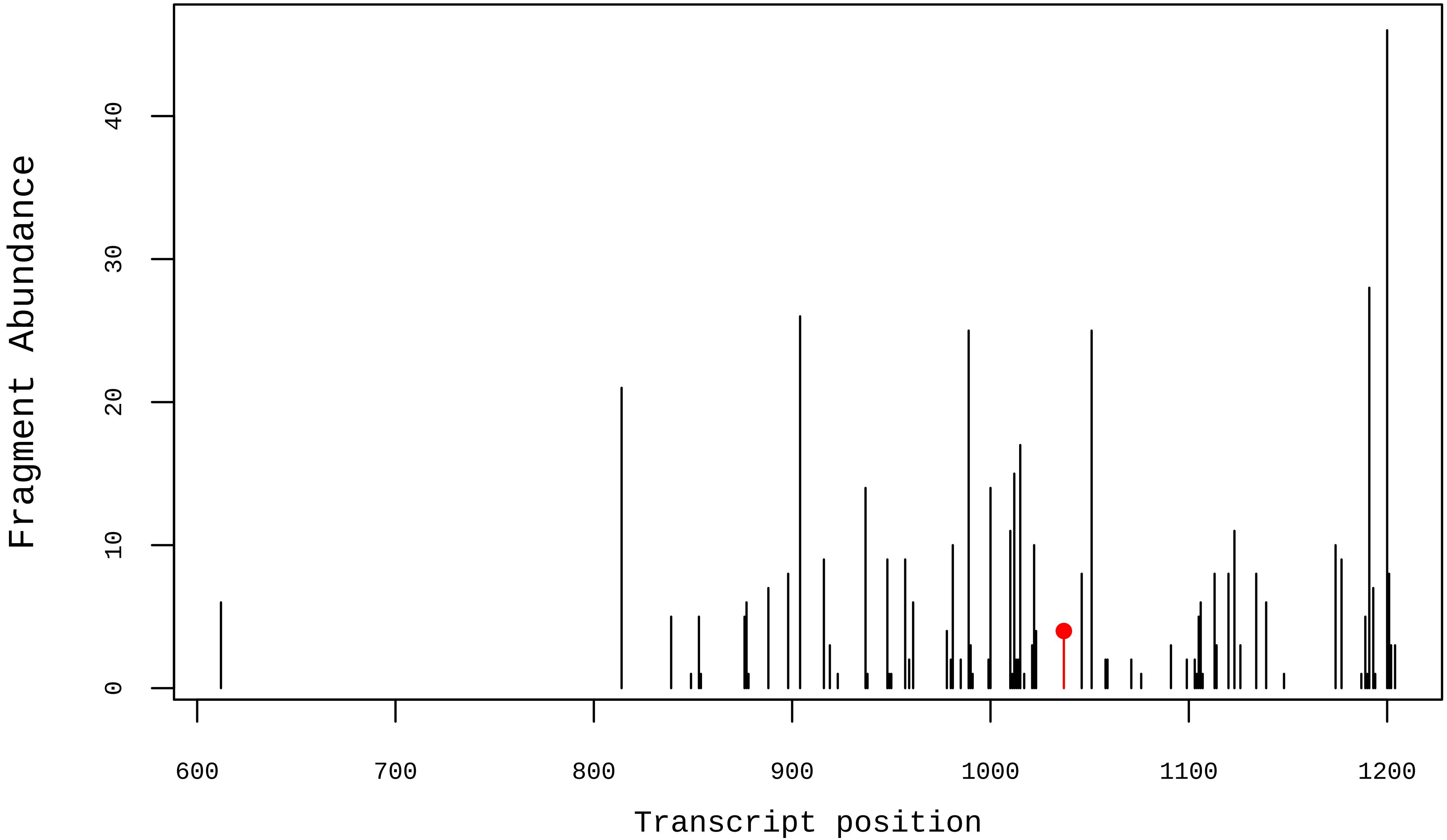

Cleavage site: 1037 Tag abundance: 4 Weighted abundance: 4 Category: 3
sRNA abundance: 1 Alignment score: 3 MFE ratio: 0.878 p-value: 0.033

HORVU6Hr1G058100|HORVU6Hr1G058100.1||1365|1935

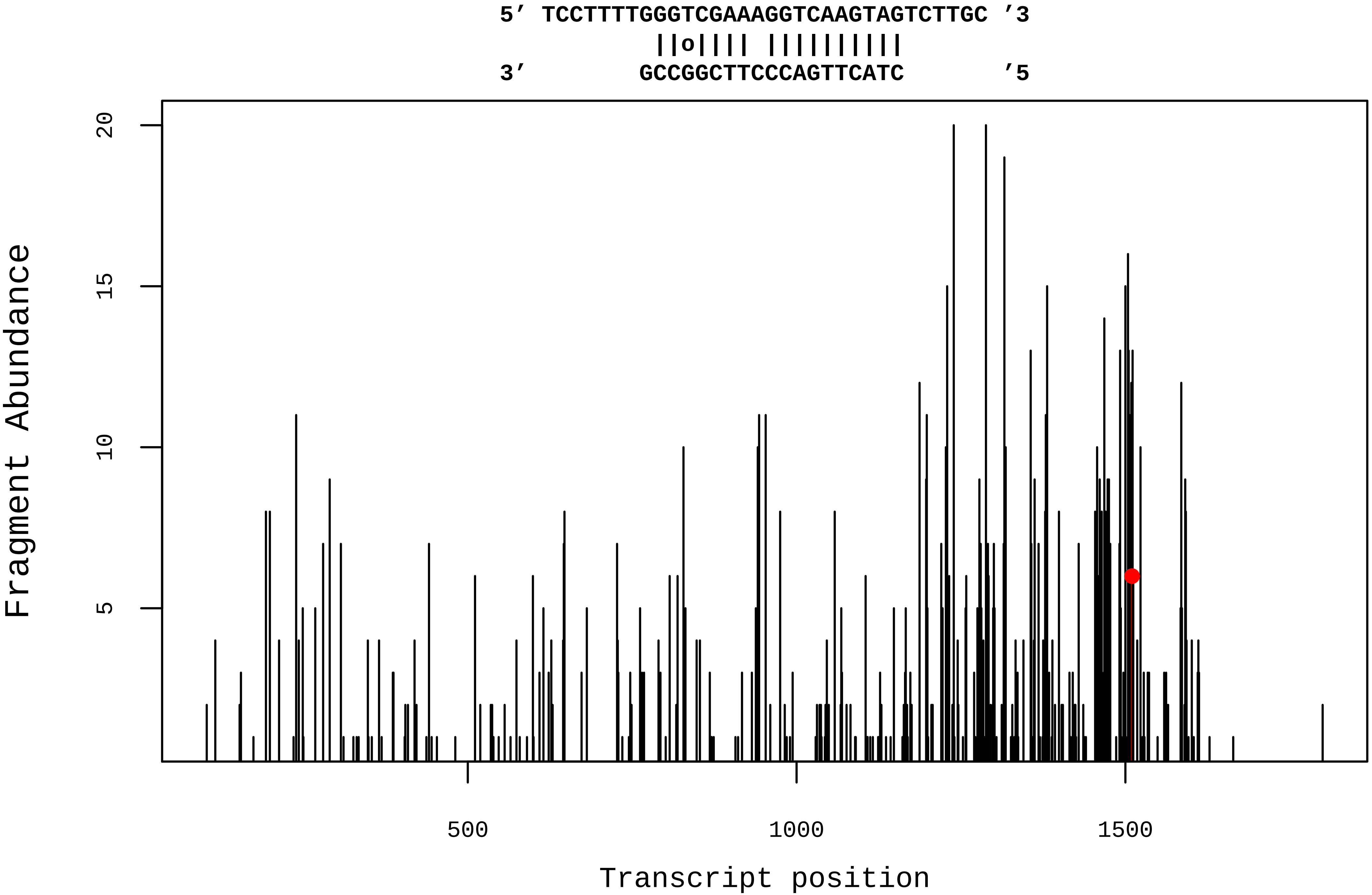

Cleavage site: 1510 Tag abundance: 6 Weighted abundance: 6 Category: 2
sRNA abundance: 1 Alignment score: 3.5 MFE ratio: 0.734 p−value: 0.037

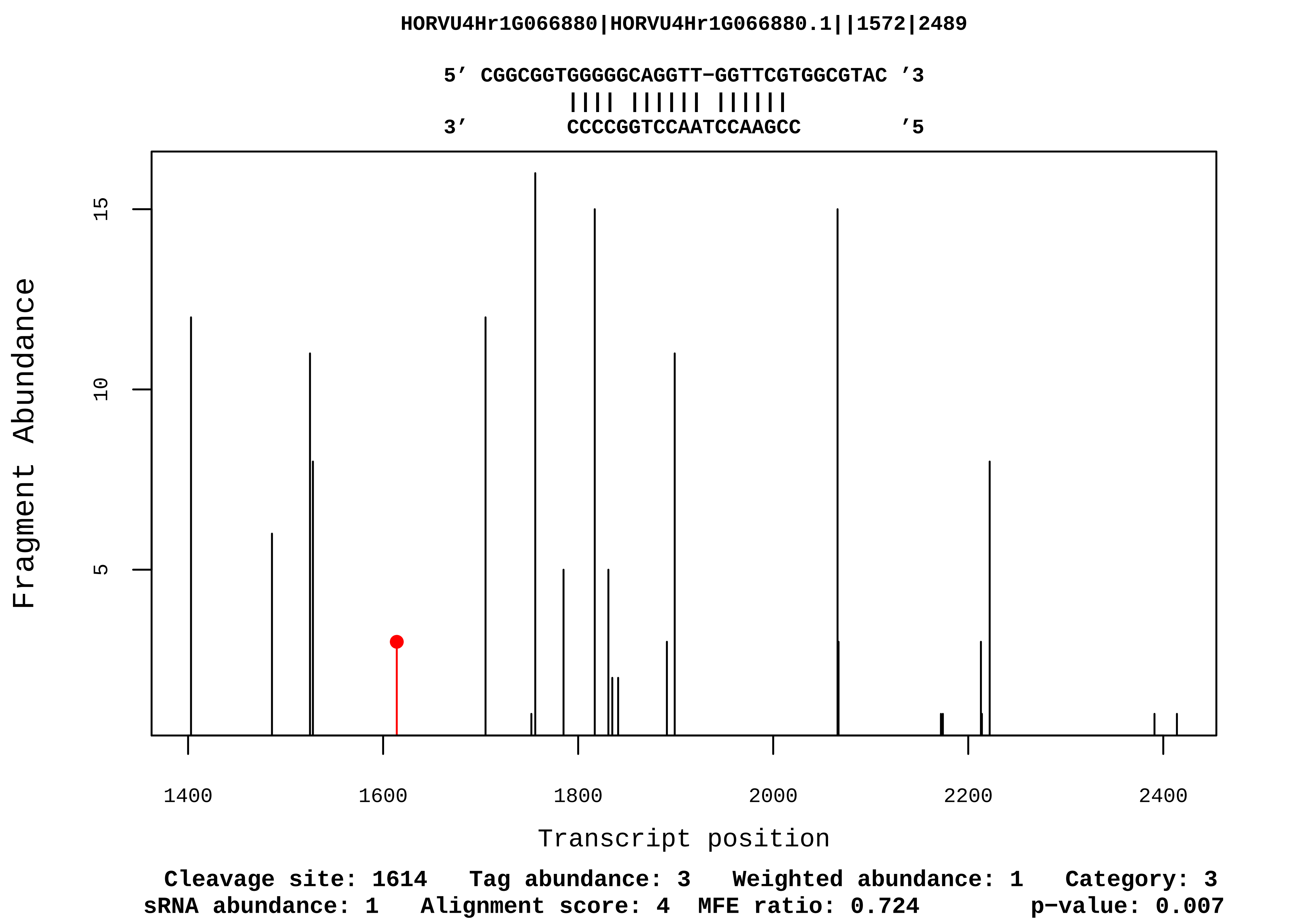

HORVU4Hr1G066880|HORVU4Hr1G066880.1||1572|2489
5' CGGCGGTGGGGGCAGGTT-GGTTCGTGGCGTAC '3
|||| |||||| ||||||
3' CCCCGGTCCAATCCAAGCC '5
Fragment Abundance
5
10
15
1400
1600
1800
2000
2200
2400
Transcript position
Cleavage site: 1614 Tag abundance: 3 Weighted abundance: 1 Category: 3
sRNA abundance: 1 Alignment score: 4 MFE ratio: 0.724 p-value: 0.007

HORVU4Hr1G066880|HORVU4Hr1G066880.2||654|1390

```
5' CGGCGGTGGGGGCAGGTT-GGTTCGTGGCGTAC '3
              |||| |||||| ||||||
3'            CCCCGGTCCAATCCAAGCC        '5
```

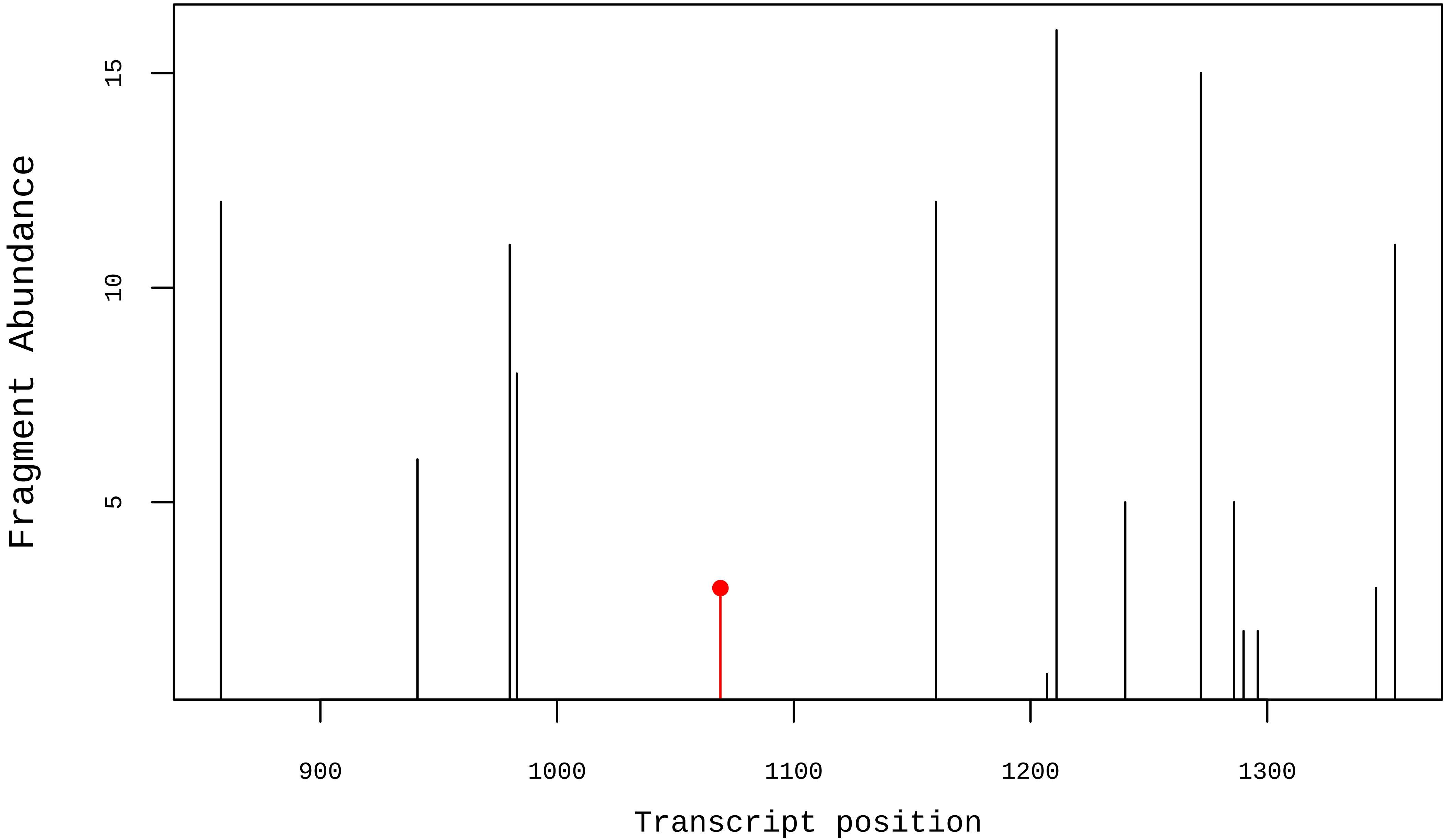

Cleavage site: 1069   Tag abundance: 3   Weighted abundance: 1   Category: 3
sRNA abundance: 1   Alignment score: 4   MFE ratio: 0.724   p-value: 0.01

HORVU0Hr1G023930|HORVU0Hr1G023930.1||297|1937

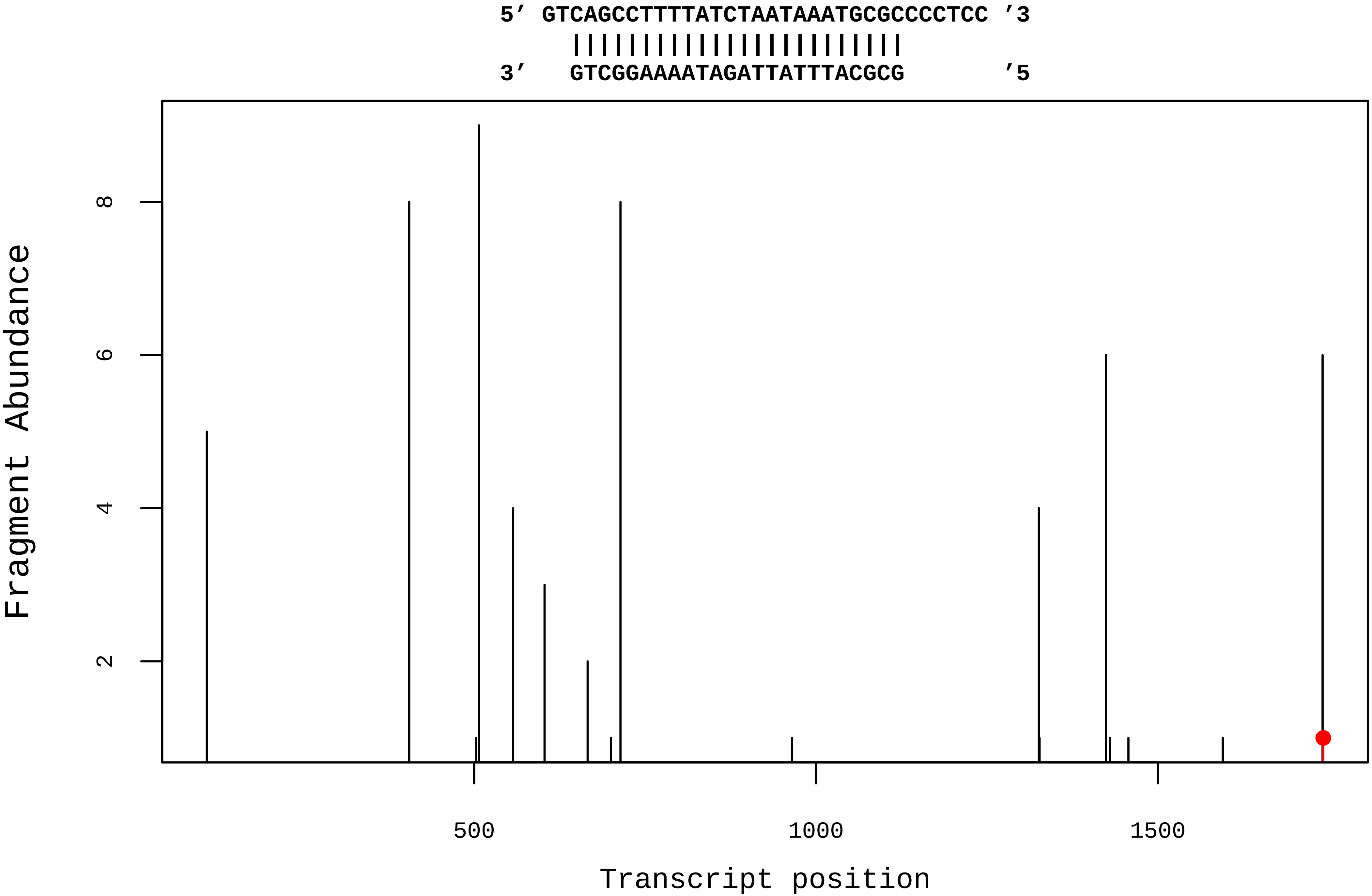

Cleavage site: 1742 Tag abundance: 1 Weighted abundance: 0.125 Category: 4
sRNA abundance: 1 Alignment score: 0 MFE ratio: 1 p-value: 0.033

HORVU2Hr1G072960|HORVU2Hr1G072960.1||600|3353

```
5' GTCAGCCTTTTATCTAATAAATGCGCCCCTCC '3
      ||||||||||||||||||||||||
3'    GTCGGAAAATAGATTATTTACGCG       '5
```

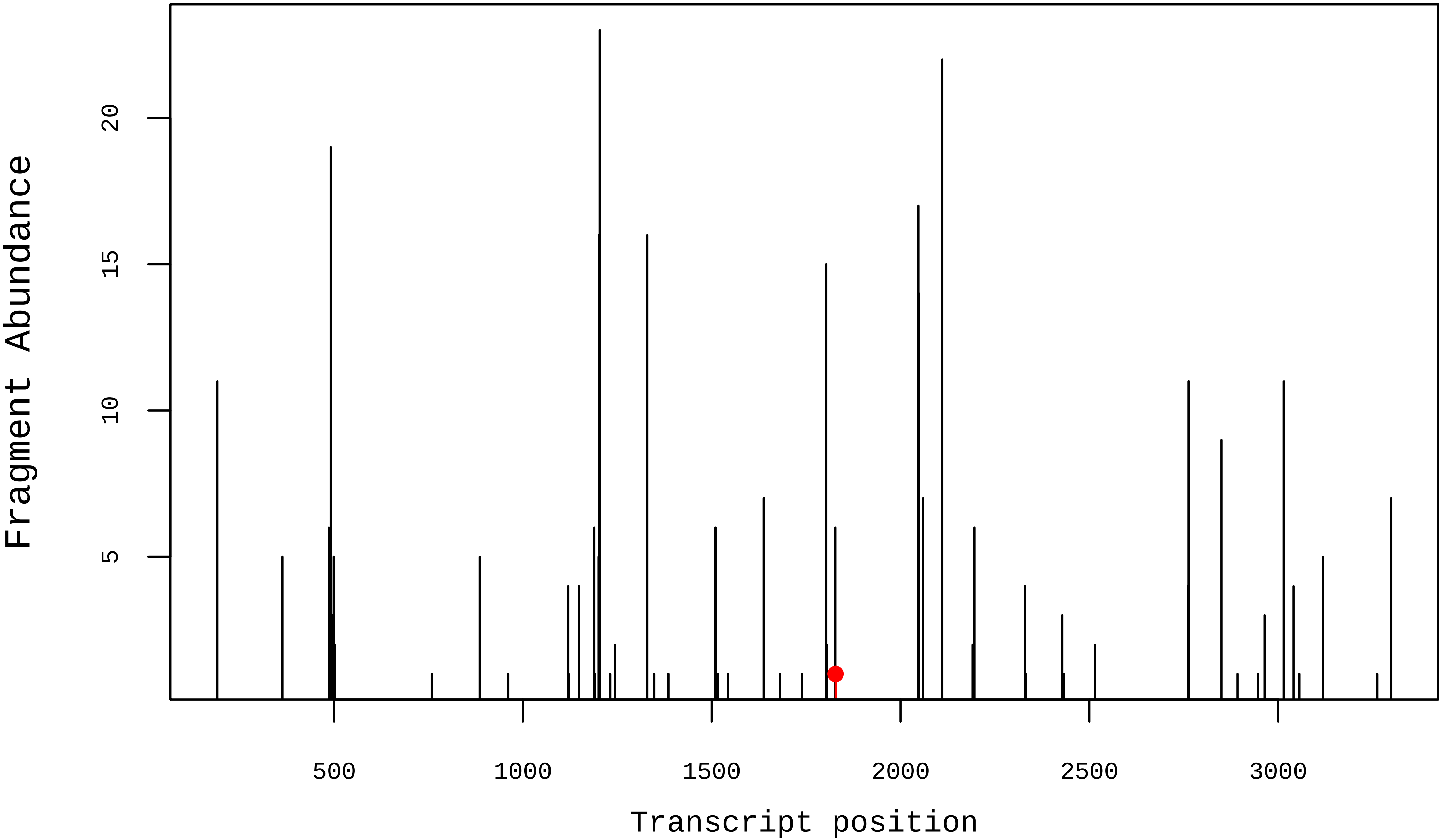

Cleavage site: 1828   Tag abundance: 1   Weighted abundance: 0.125   Category: 4
sRNA abundance: 1   Alignment score: 0   MFE ratio: 1   p-value: 0.047

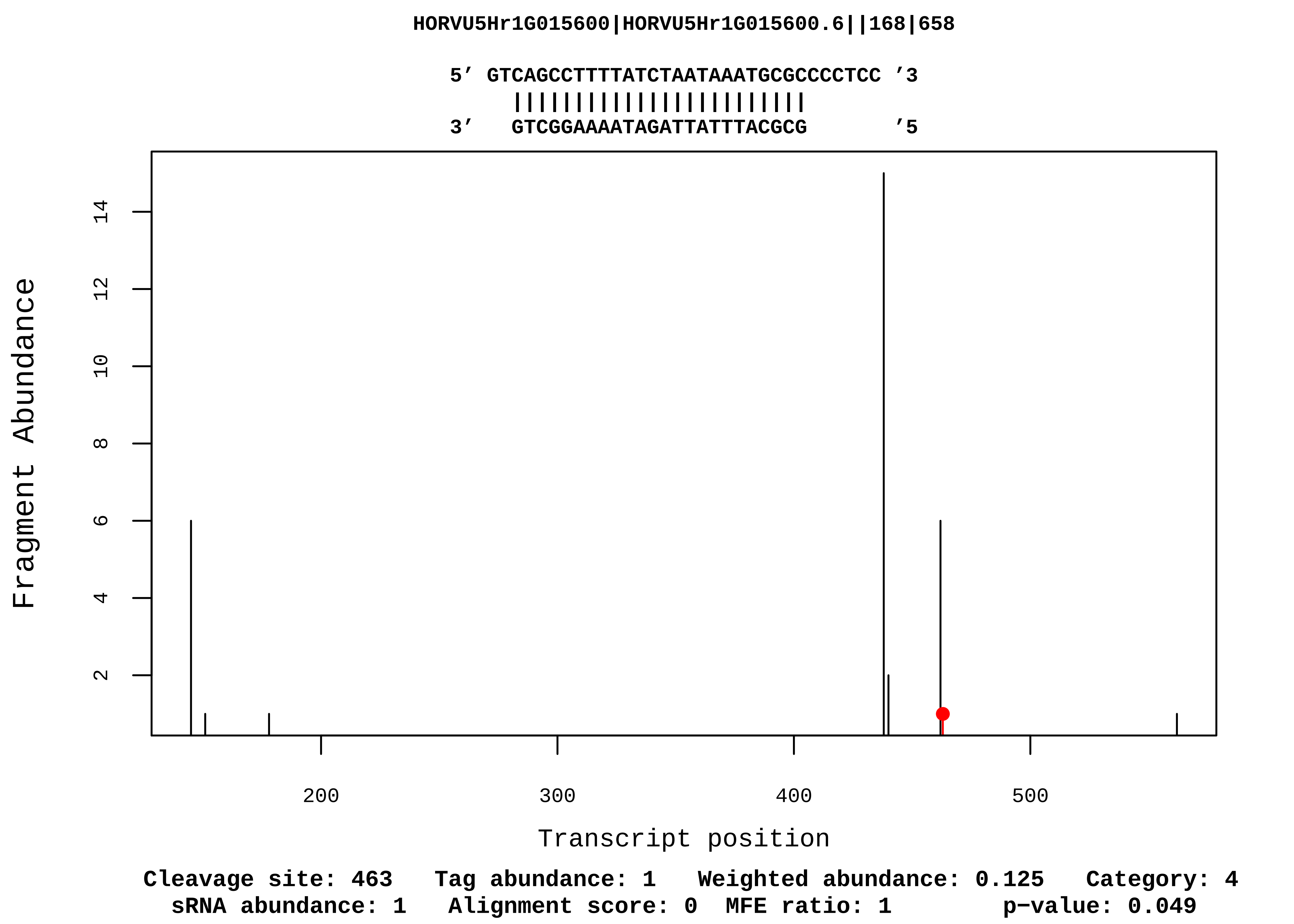

HORVU5Hr1G015600|HORVU5Hr1G015600.6||168|658
5′ GTCAGCCTTTTATCTAATAAATGCGCCCCTCC ′3
3′ GTCGGAAAATAGATTATTTACGCG ′5
Fragment Abundance
2
4
6
8
10
12
14
200
300
400
500
Transcript position
Cleavage site: 463 Tag abundance: 1 Weighted abundance: 0.125 Category: 4
sRNA abundance: 1 Alignment score: 0 MFE ratio: 1 p-value: 0.049

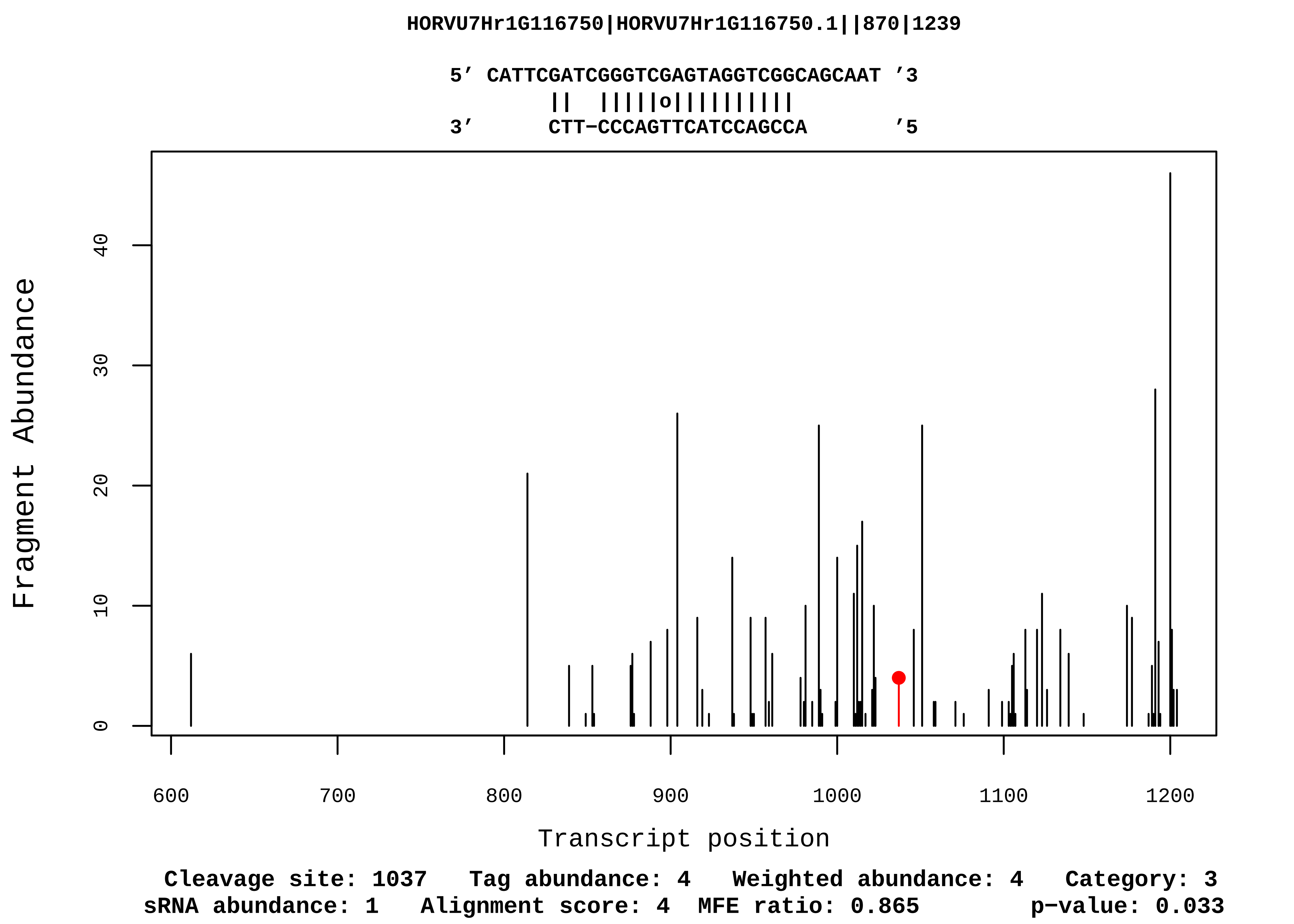
HORVU7Hr1G116750|HORVU7Hr1G116750.1||870|1239
5' CATTCGATCGGGTCGAGTAGGTCGGCAGCAAT '3
|| |||||o||||||||||
3'   CTT-CCCAGTTCATCCAGCCA   '5
Fragment Abundance
0
10
20
30
40
600
700
800
900
1000
1100
1200
Transcript position
Cleavage site: 1037   Tag abundance: 4   Weighted abundance: 4   Category: 3
sRNA abundance: 1   Alignment score: 4 MFE ratio: 0.865   p-value: 0.033

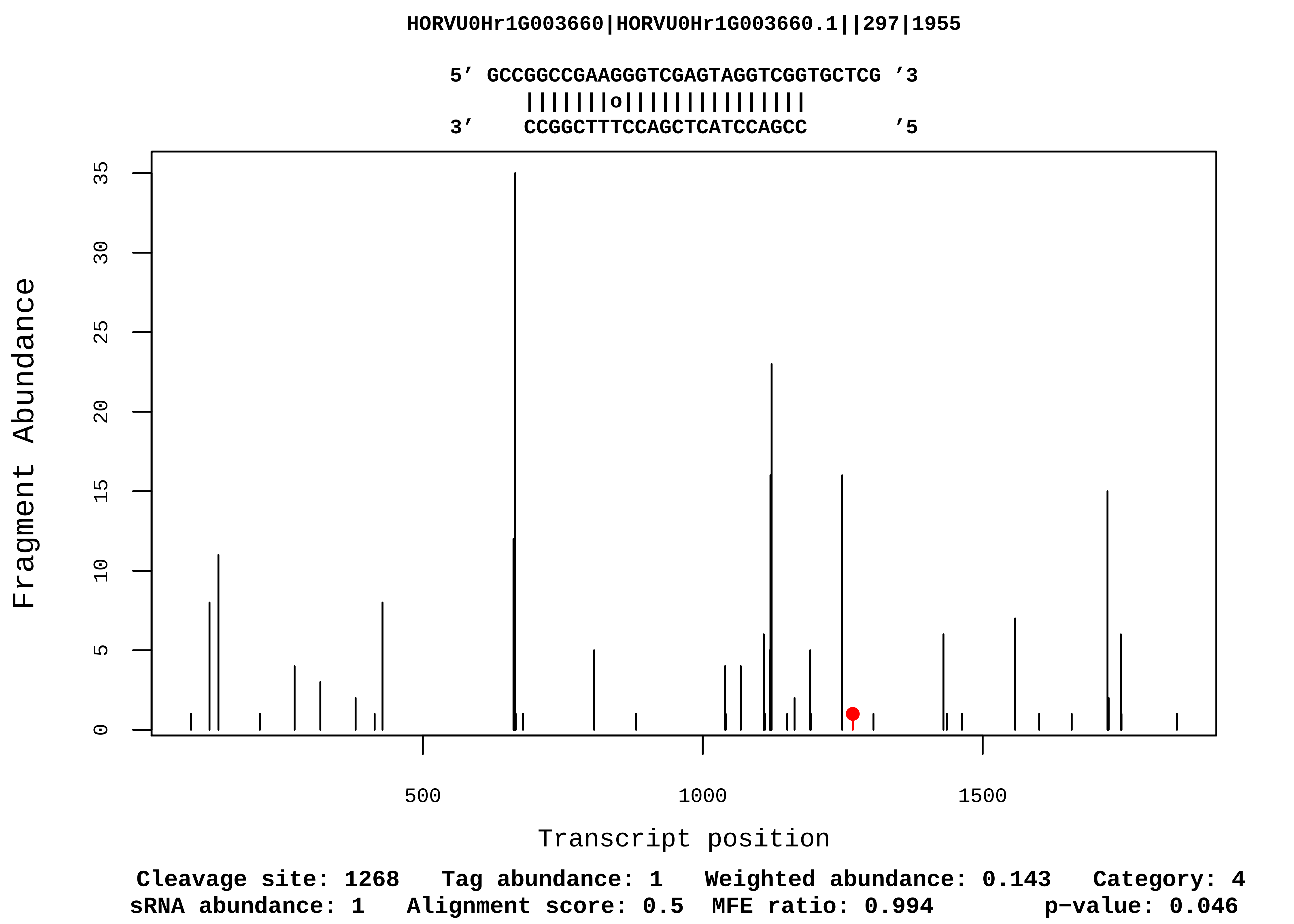

HORVU0Hr1G003660|HORVU0Hr1G003660.1||297|1955
5' GCCGGCCGAAGGGTCGAGTAGGTCGGTGCTCG '3
|||||||o||||||||||||||
3' CCGGCTTTCCAGCTCATCCAGCC '5
Fragment Abundance
0
5
10
15
20
25
30
35
500
1000
1500
Transcript position
Cleavage site: 1268 Tag abundance: 1 Weighted abundance: 0.143 Category: 4
sRNA abundance: 1 Alignment score: 0.5 MFE ratio: 0.994 p-value: 0.046

HORVU0Hr1G023930|HORVU0Hr1G023930.3||396|2180

```
5' GTCGGCGGAAGGGTCGAGTAGGTCGGTGCTCG '3
        ||| |||o|||||||||||||||
3'      CCGGCTTTCCAGCTCATCCAGCC        '5
```

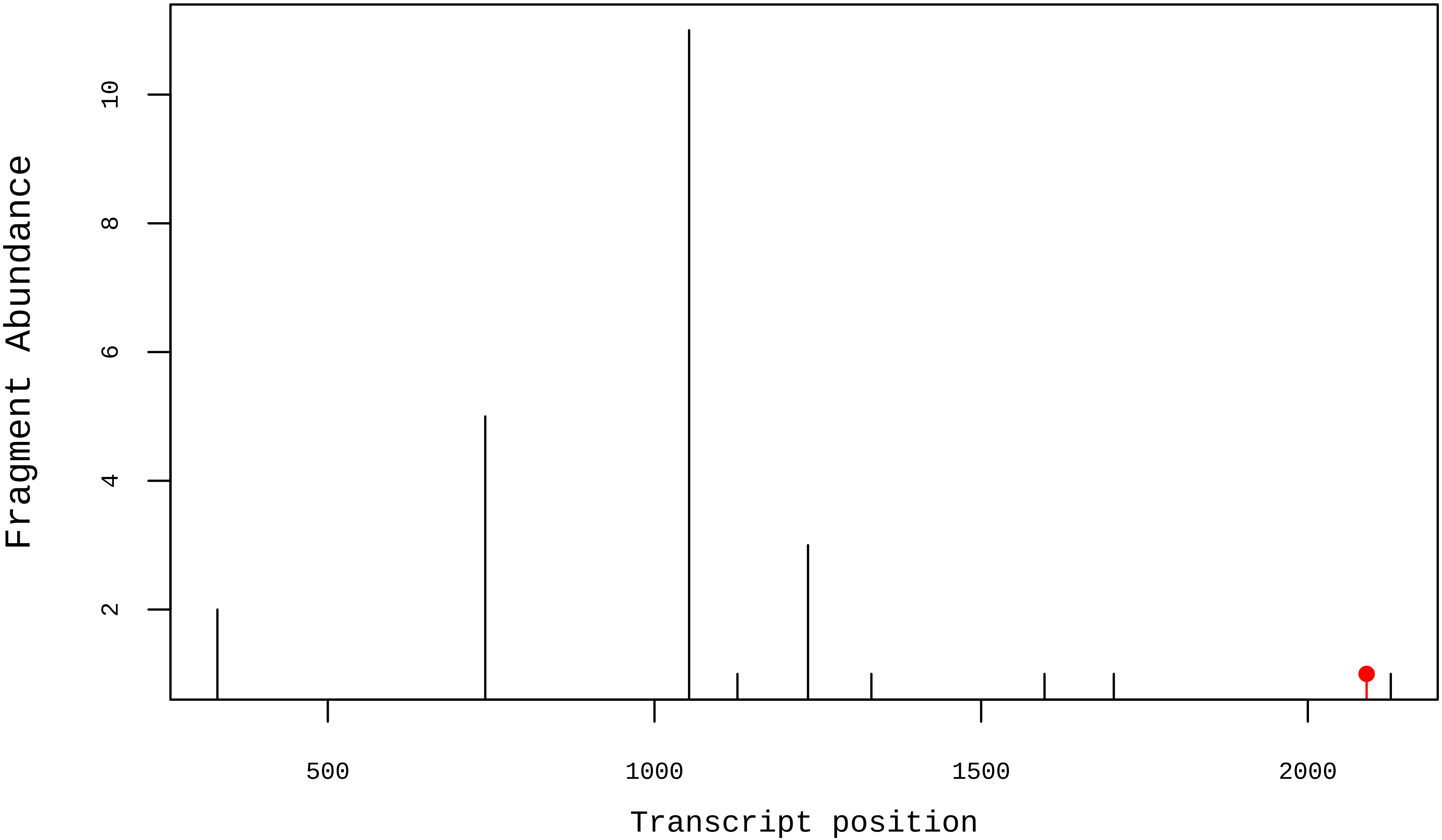

Cleavage site: 2090   Tag abundance: 1   Weighted abundance: 0.143   Category: 4
sRNA abundance: 1   Alignment score: 1.5   MFE ratio: 0.905   p-value: 0.019

HORVU2Hr1G072960|HORVU2Hr1G072960.1||600|3353

```
5' GCCGGCCGAAGGGTCGAGTAGGTCGGTGCTCG '3
        |||||||o|||||||||||||||
3'      CCGGCTTTCCAGCTCATCCAGCC        '5
```

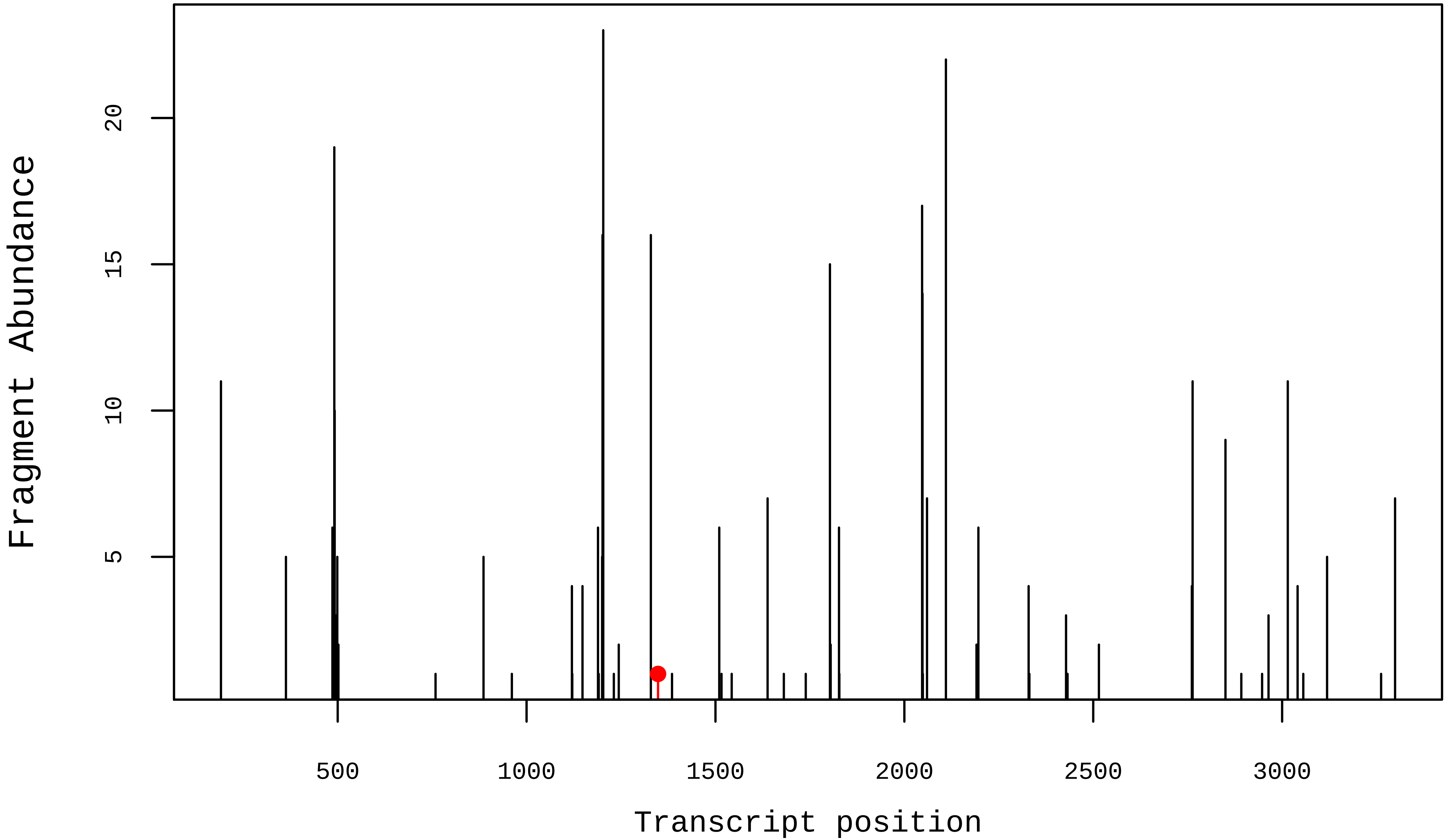

Cleavage site: 1348 Tag abundance: 1 Weighted abundance: 0.143 Category: 4
sRNA abundance: 1 Alignment score: 0.5 MFE ratio: 0.994 p-value: 0.03

HORVU5Hr1G015600|HORVU5Hr1G015600.1||156|510

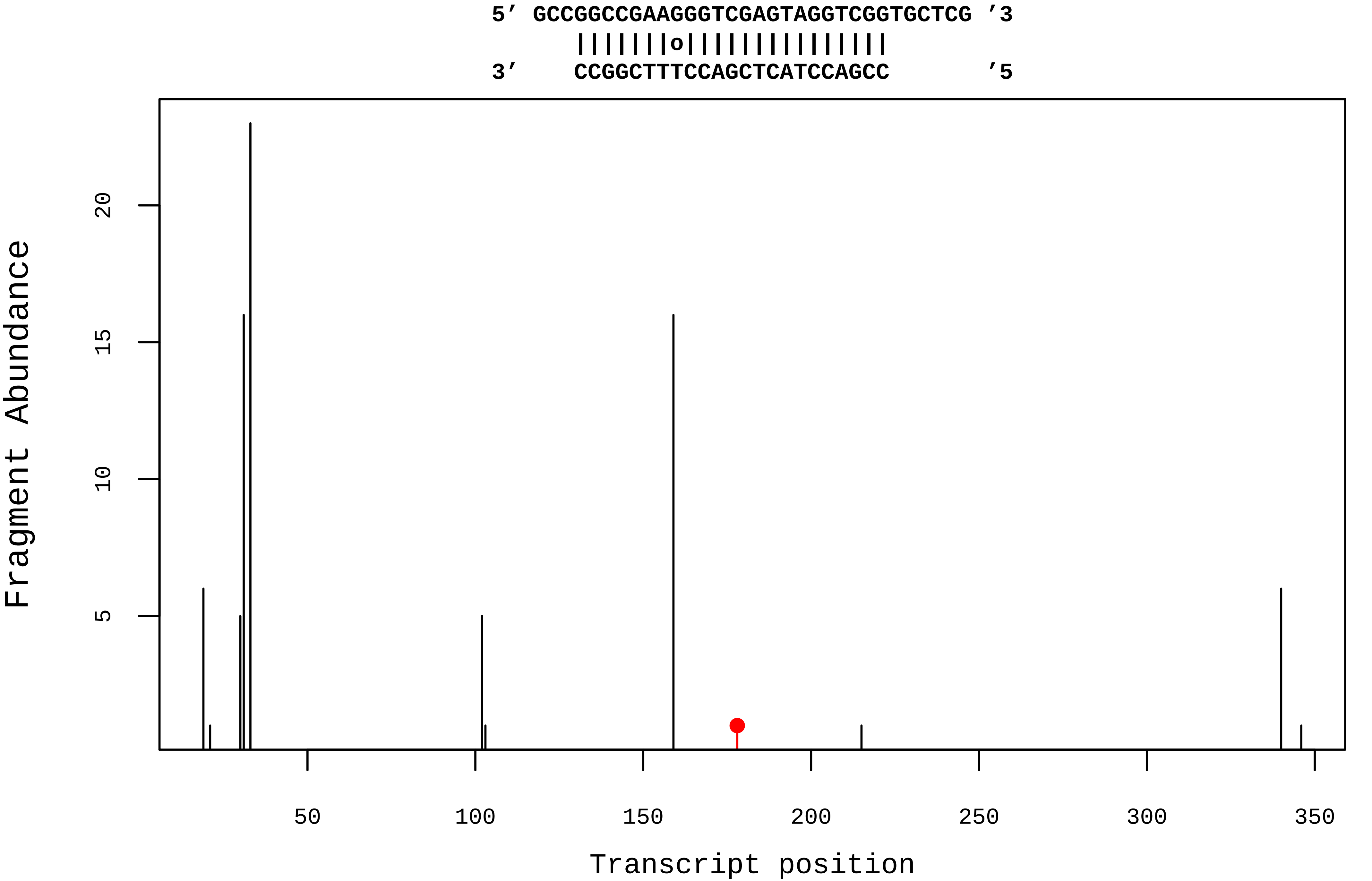

Cleavage site: 178 Tag abundance: 1 Weighted abundance: 0.143 Category: 4
sRNA abundance: 1 Alignment score: 0.5 MFE ratio: 0.994 p-value: 0.05

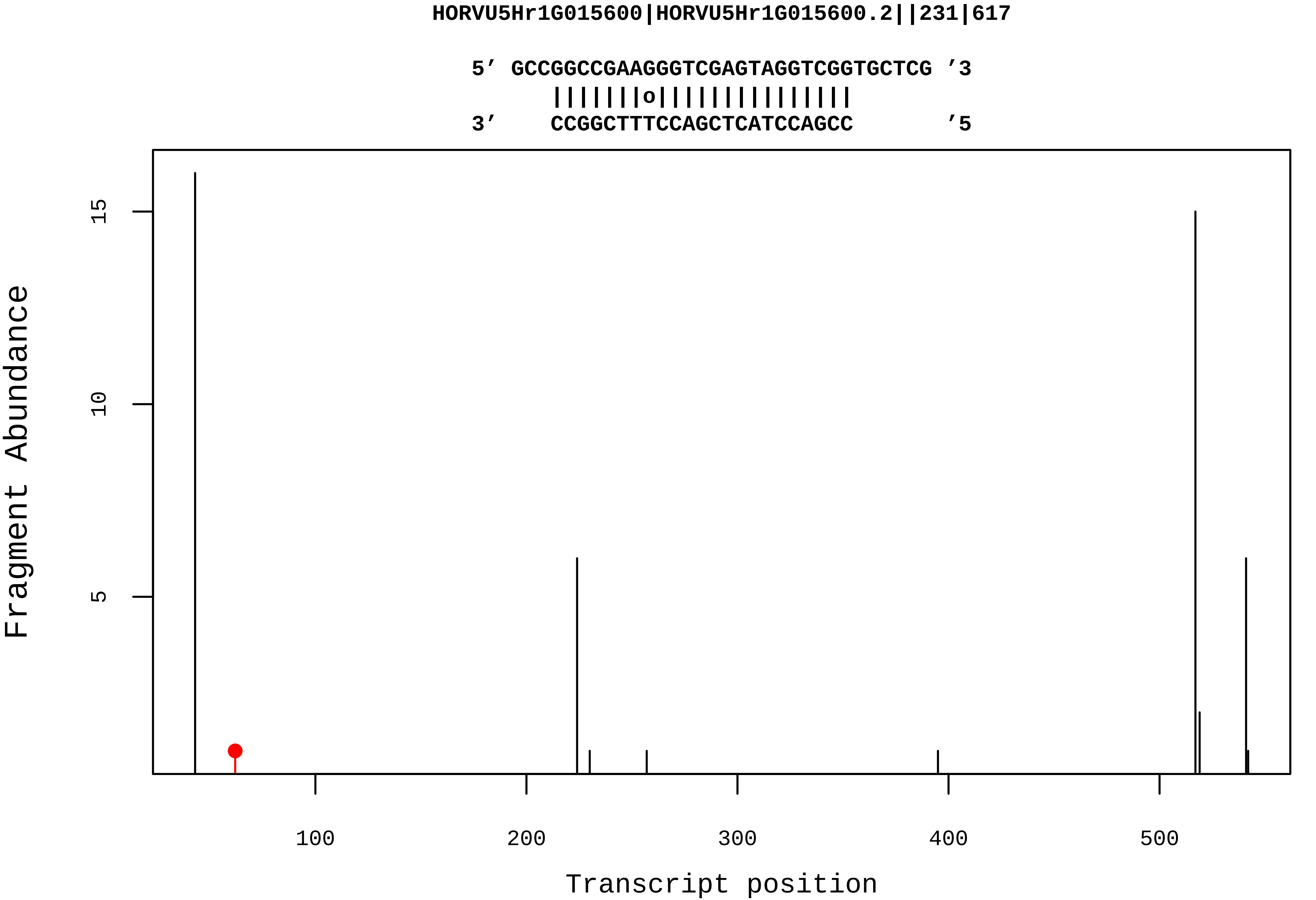

Cleavage site: 62 Tag abundance: 1 Weighted abundance: 0.143 Category: 4
sRNA abundance: 1 Alignment score: 0.5 MFE ratio: 0.994 p-value: 0.041

HORVU5Hr1G015600|HORVU5Hr1G015600.3||276|1709

```
5' GCCGGCCGAAGGGTCGAGTAGGTCGGTGCTCG '3
        |||||||o||||||||||||||
3'      CCGGCTTTCCAGCTCATCCAGCC       '5
```

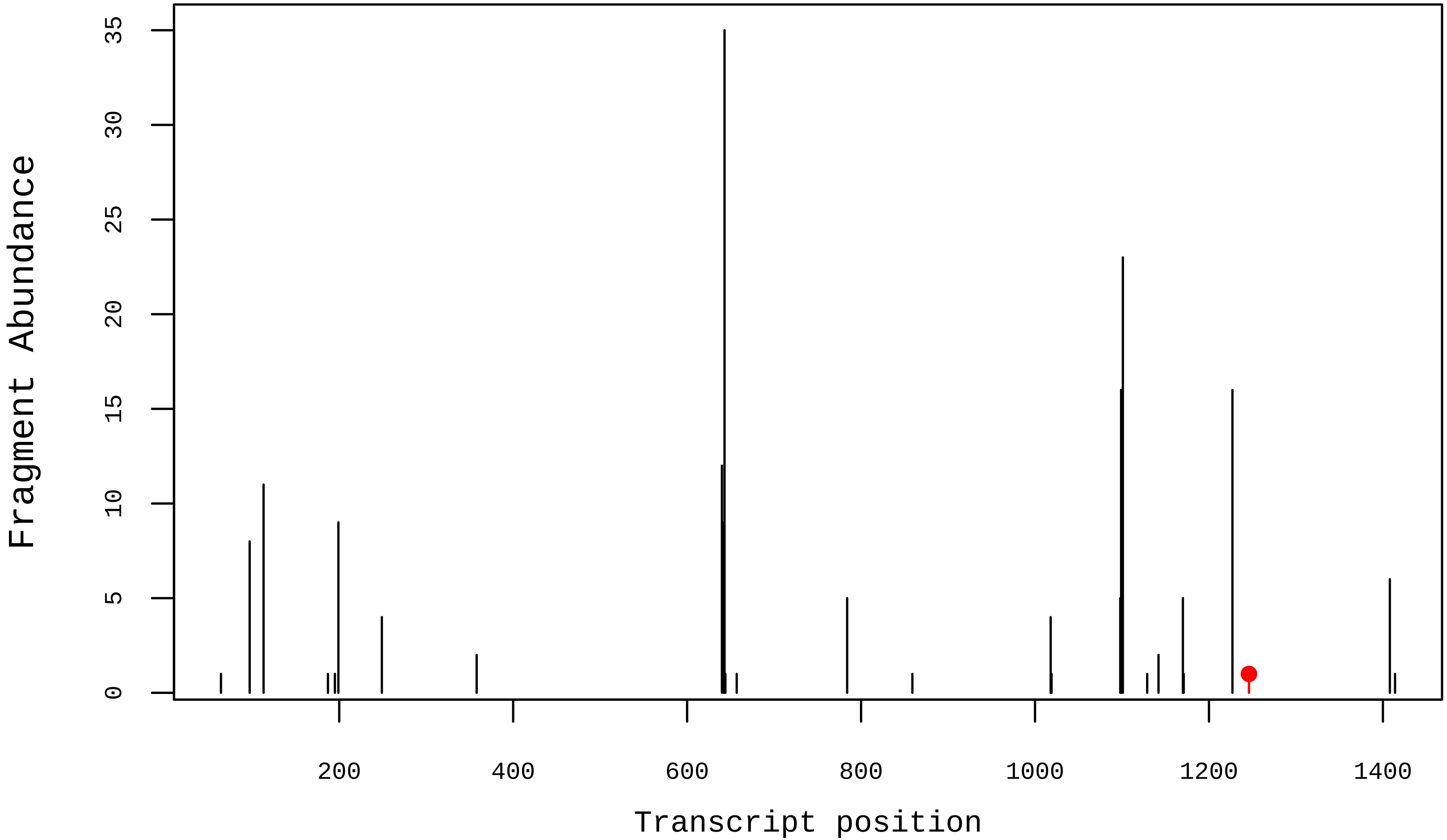

Cleavage site: 1246   Tag abundance: 1   Weighted abundance: 0.143   Category: 4
sRNA abundance: 1   Alignment score: 0.5   MFE ratio: 0.994   p-value: 0.032

HORVU5Hr1G015600|HORVU5Hr1G015600.4||207|1852

```
5' GCCGGCCGCAGGGTCGAGTAGGTCGGTGCTCG '3
       ||||| |o||||||||||||||
3'     CCGGCTTTCCAGCTCATCCAGCC       '5
```

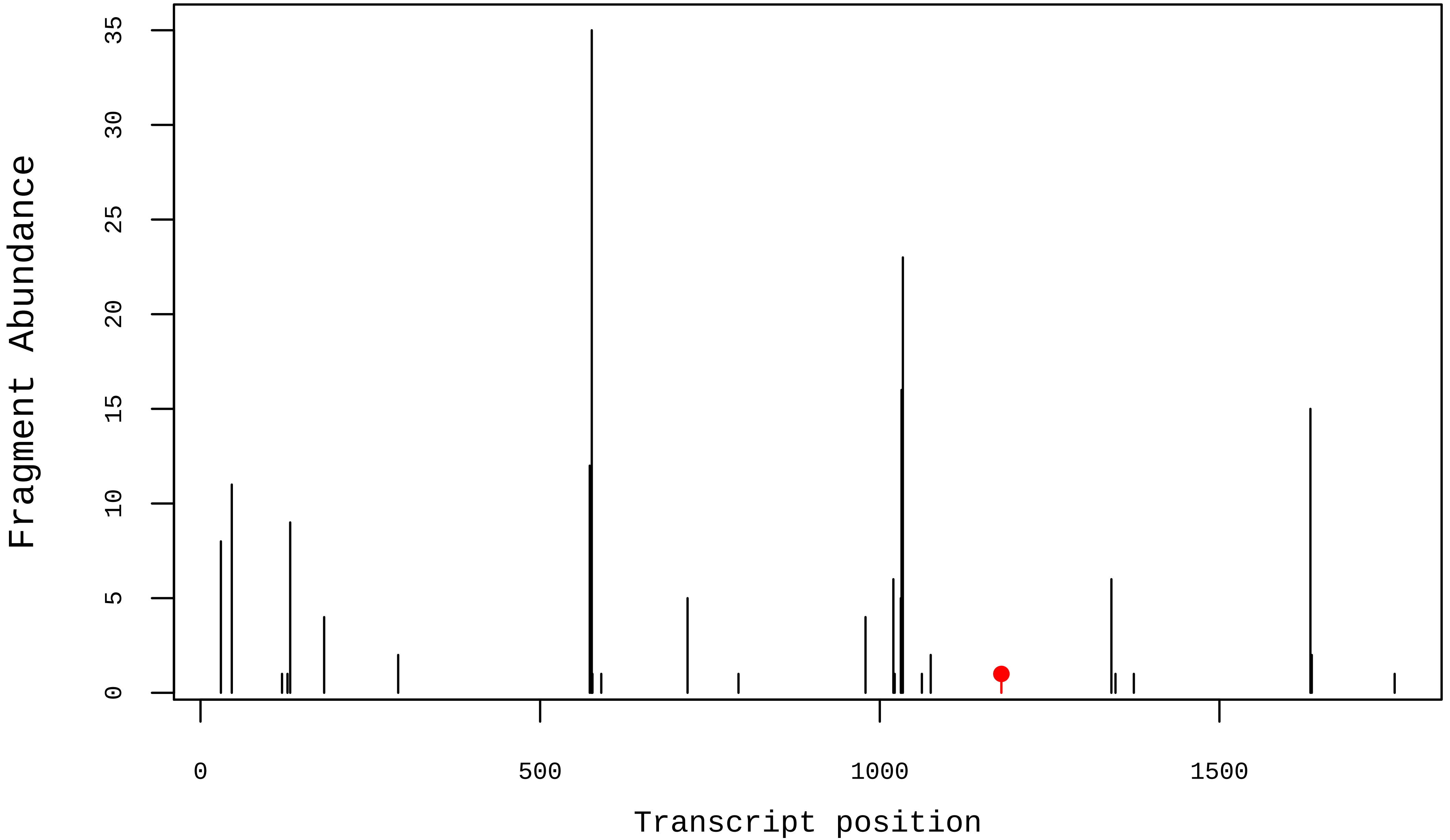

Cleavage site: 1179   Tag abundance: 1   Weighted abundance: 0.143   Category: 4
sRNA abundance: 1   Alignment score: 1.5   MFE ratio: 0.901   p-value: 0.041

HORVU0Hr1G023930|HORVU0Hr1G023930.3||396|2180

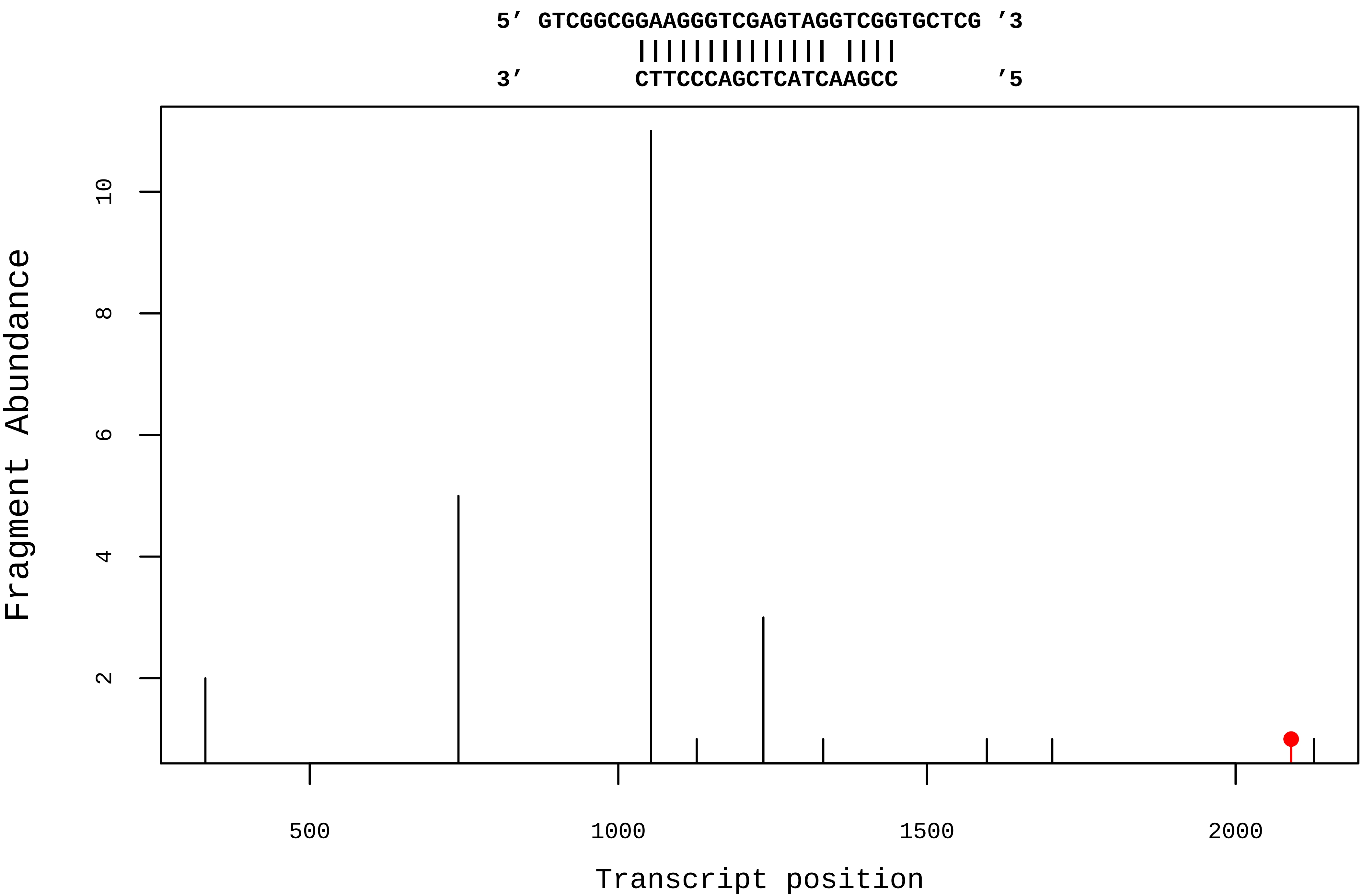

Cleavage site: 2090 Tag abundance: 1 Weighted abundance: 0.143 Category: 4
sRNA abundance: 1 Alignment score: 2 MFE ratio: 0.881 p-value: 0.017

HORVU2Hr1G072960|HORVU2Hr1G072960.1||600|3353

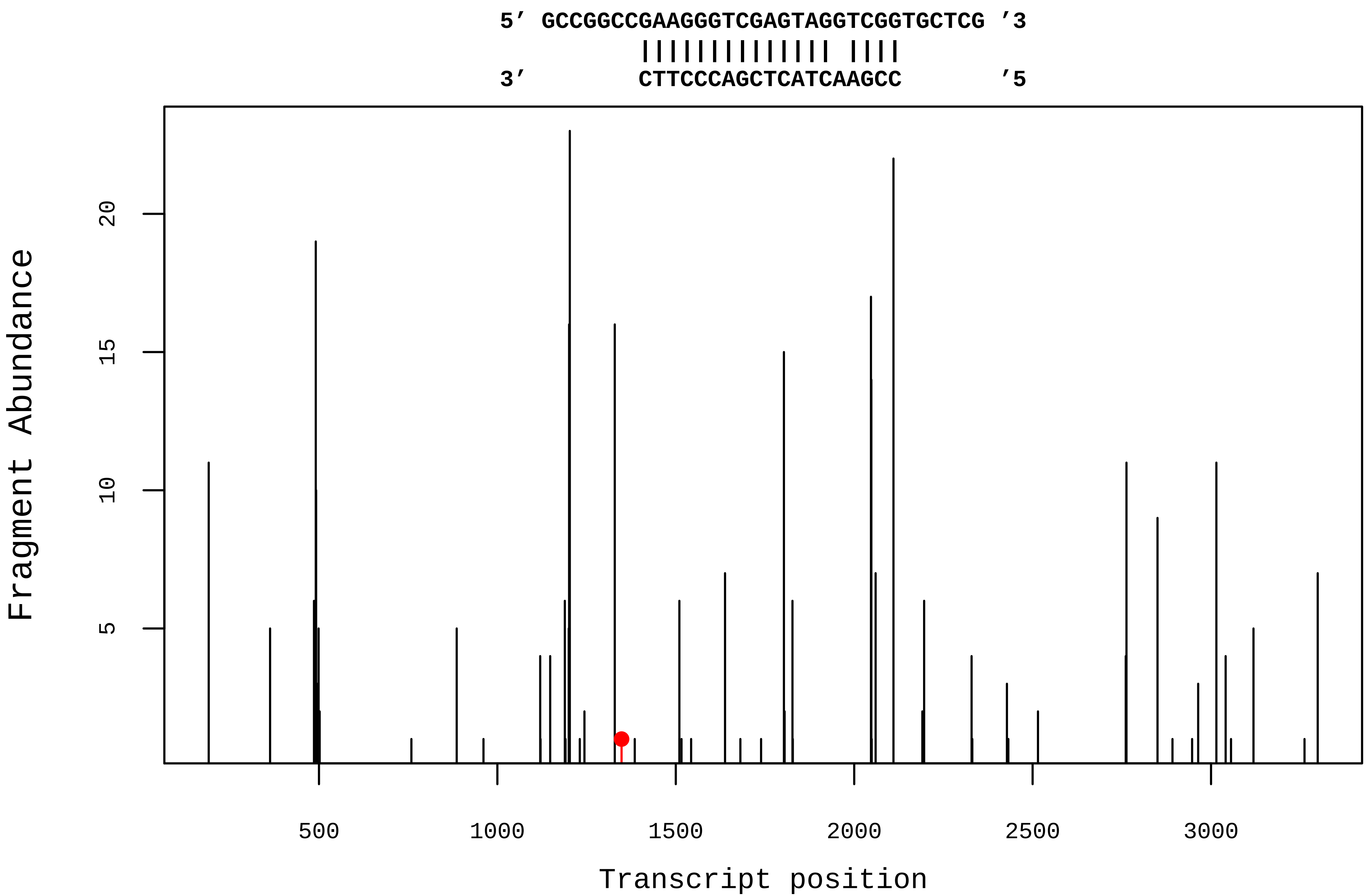

Cleavage site: 1348 Tag abundance: 1 Weighted abundance: 0.143 Category: 4
sRNA abundance: 1 Alignment score: 2 MFE ratio: 0.881 p-value: 0.035

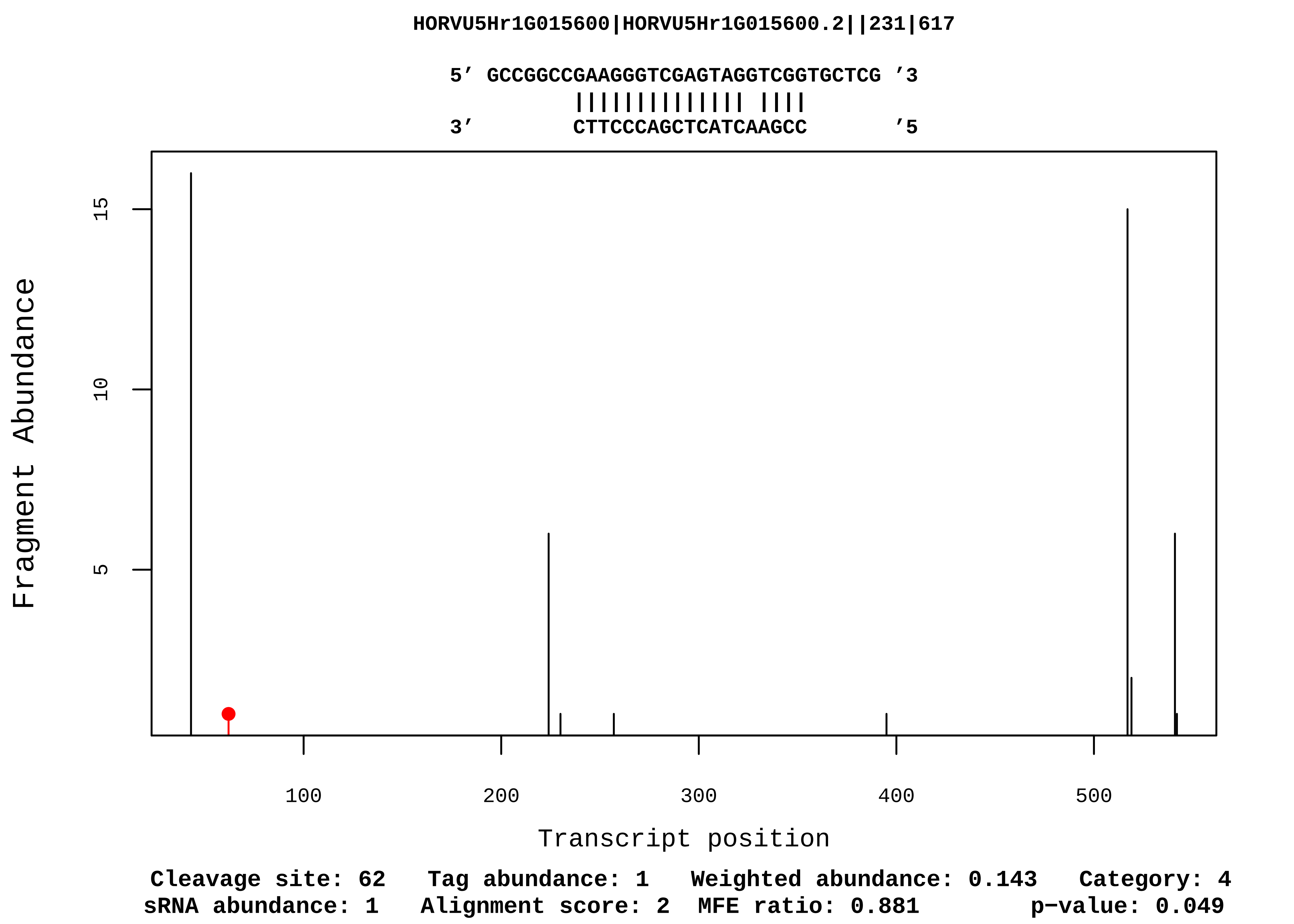
HORVU5Hr1G015600|HORVU5Hr1G015600.2||231|617
5' GCCGGCCGAAGGGTCGAGTAGGTCGGTGCTCG '3
|||||||||||||| ||||
3' CTTCCCAGCTCATCAAGCC '5
Fragment Abundance
5
10
15
100
200
300
400
500
Transcript position
Cleavage site: 62 Tag abundance: 1 Weighted abundance: 0.143 Category: 4
sRNA abundance: 1 Alignment score: 2 MFE ratio: 0.881 p-value: 0.049

HORVU5Hr1G015600|HORVU5Hr1G015600.3||276|1709

```
5' GCCGGCCGAAGGGTCGAGTAGGTCGGTGCTCG '3
          |||||||||||||| ||||
3'        CTTCCCAGCTCATCAAGCC        '5
```

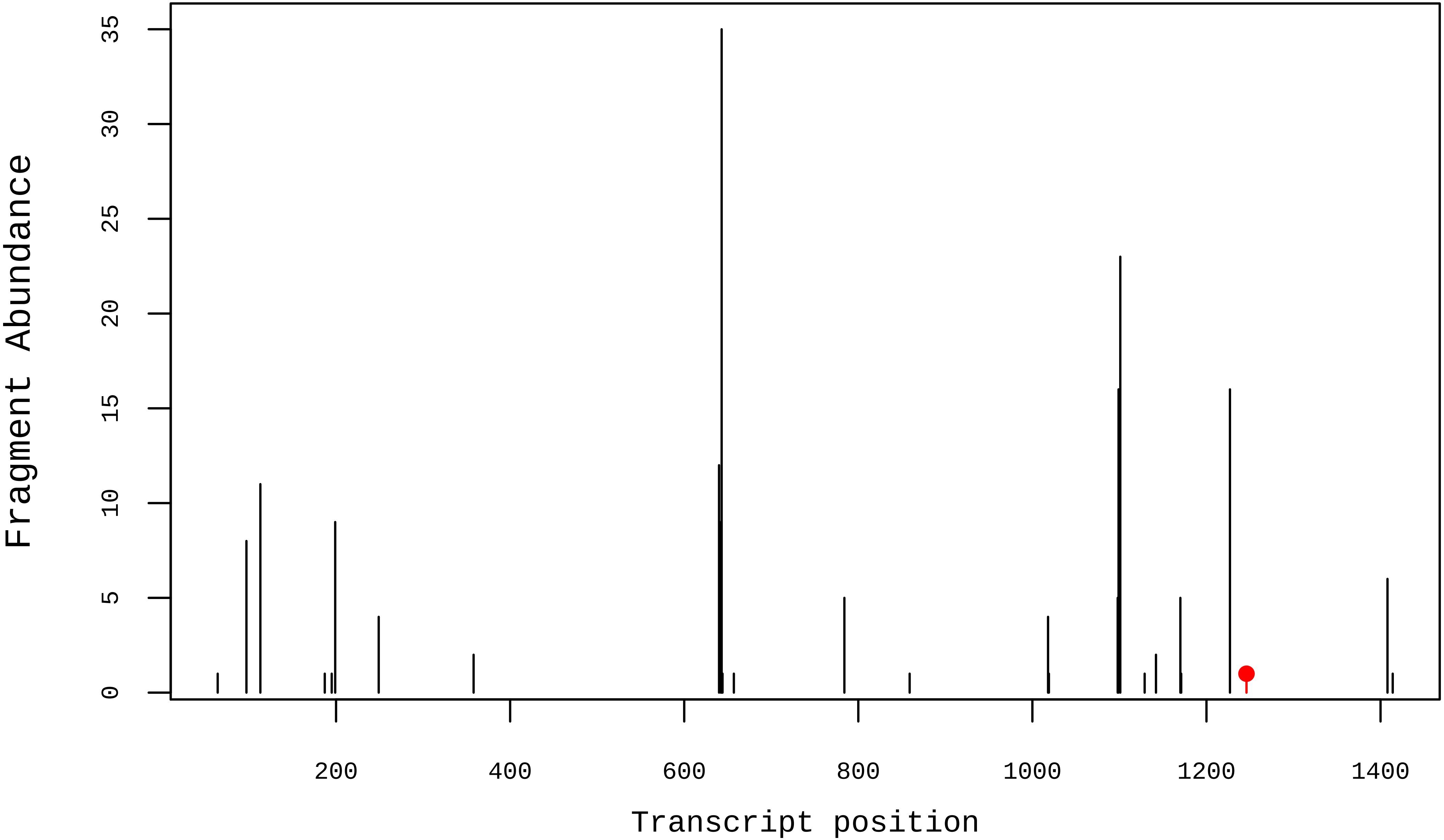

Cleavage site: 1246   Tag abundance: 1   Weighted abundance: 0.143   Category: 4
sRNA abundance: 1   Alignment score: 2   MFE ratio: 0.881   p-value: 0.038

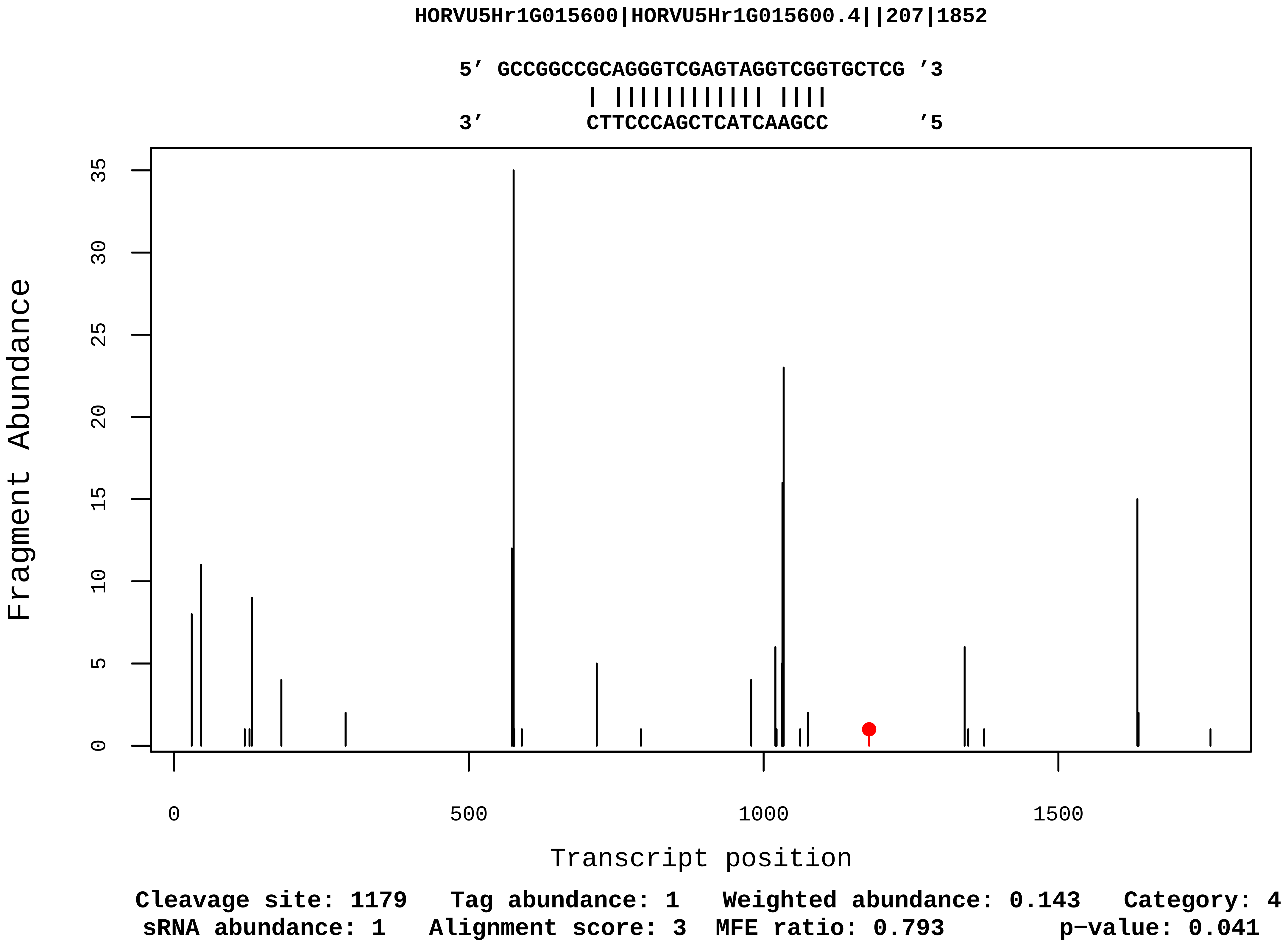
HORVU5Hr1G015600|HORVU5Hr1G015600.4||207|1852
5' GCCGGCCGCAGGGTCGAGTAGGTCGGTGCTCG '3
3' CTTCCCAGCTCATCAAGCC '5
Fragment Abundance
0
5
10
15
20
25
30
35
0
500
1000
1500
Transcript position
Cleavage site: 1179 Tag abundance: 1 Weighted abundance: 0.143 Category: 4
sRNA abundance: 1 Alignment score: 3 MFE ratio: 0.793 p-value: 0.041

HORVU0Hr1G003660|HORVU0Hr1G003660.1||297|1955

```
5' GCCGGCCGAAGGGTCGAGTAGGTCGGTGCTCG '3
      |||||||||||||||||||o||||
3'    GCCGGCTTCCCAGCTCATCTAGCC       '5
```

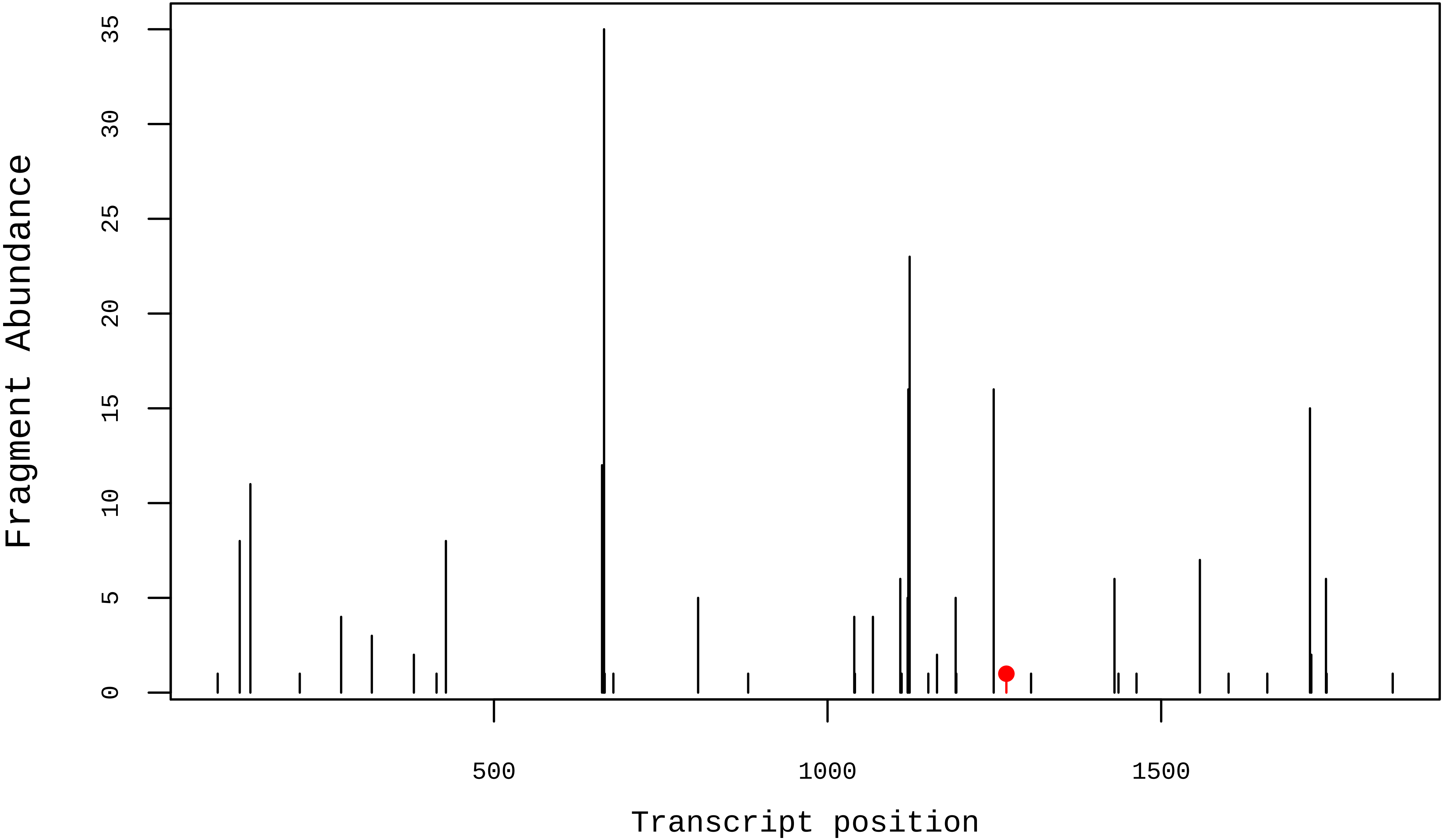

Cleavage site: 1268   Tag abundance: 1   Weighted abundance: 0.143   Category: 4
sRNA abundance: 1   Alignment score: 1   MFE ratio: 0.988   p-value: 0.046

HORVU0Hr1G023930|HORVU0Hr1G023930.3||396|2180

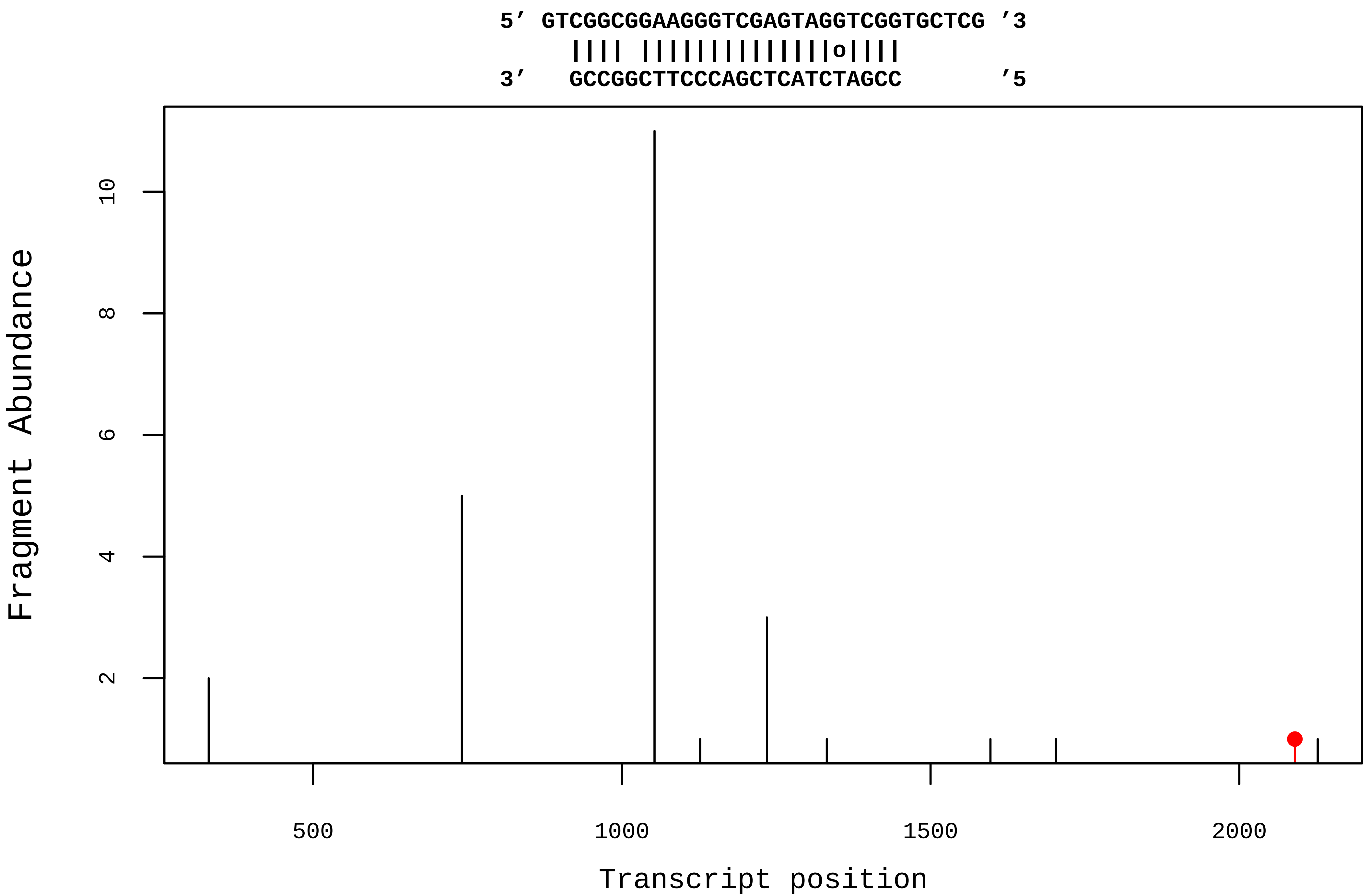

Cleavage site: 2090 Tag abundance: 1 Weighted abundance: 0.143 Category: 4
sRNA abundance: 1 Alignment score: 2 MFE ratio: 0.905 p-value: 0.019

HORVU2Hr1G072960|HORVU2Hr1G072960.1||600|3353

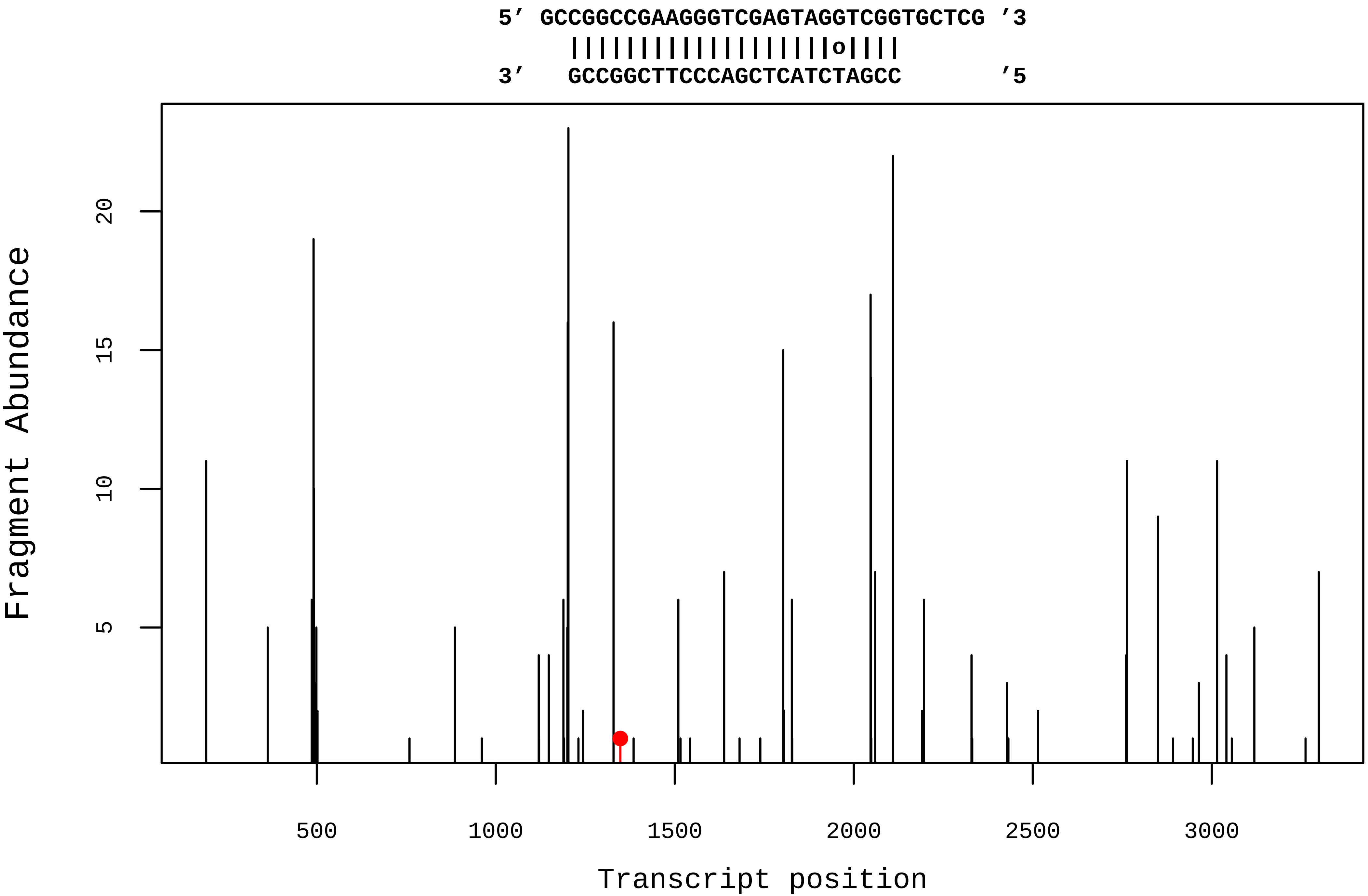

Cleavage site: 1348 Tag abundance: 1 Weighted abundance: 0.143 Category: 4
sRNA abundance: 1 Alignment score: 1 MFE ratio: 0.988 p-value: 0.03

HORVU5Hr1G015600|HORVU5Hr1G015600.1||156|510

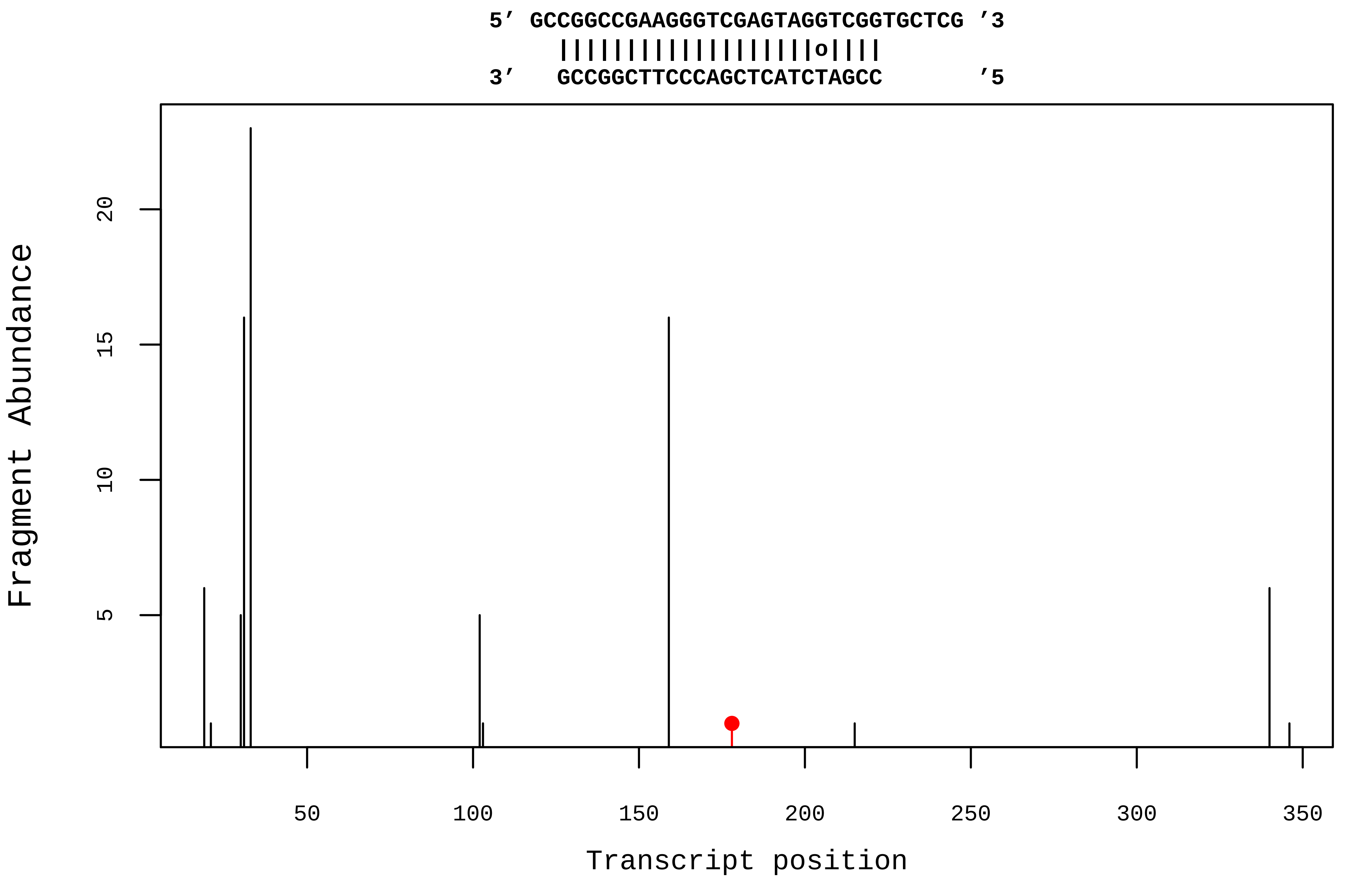

Cleavage site: 178 Tag abundance: 1 Weighted abundance: 0.143 Category: 4
sRNA abundance: 1 Alignment score: 1 MFE ratio: 0.988 p-value: 0.05

HORVU5Hr1G015600|HORVU5Hr1G015600.2||231|617

```
5' GCCGGCCGAAGGGTCGAGTAGGTCGGTGCTCG '3
      |||||||||||||||||||o||||
3'   GCCGGCTTCCCAGCTCATCTAGCC       '5
```

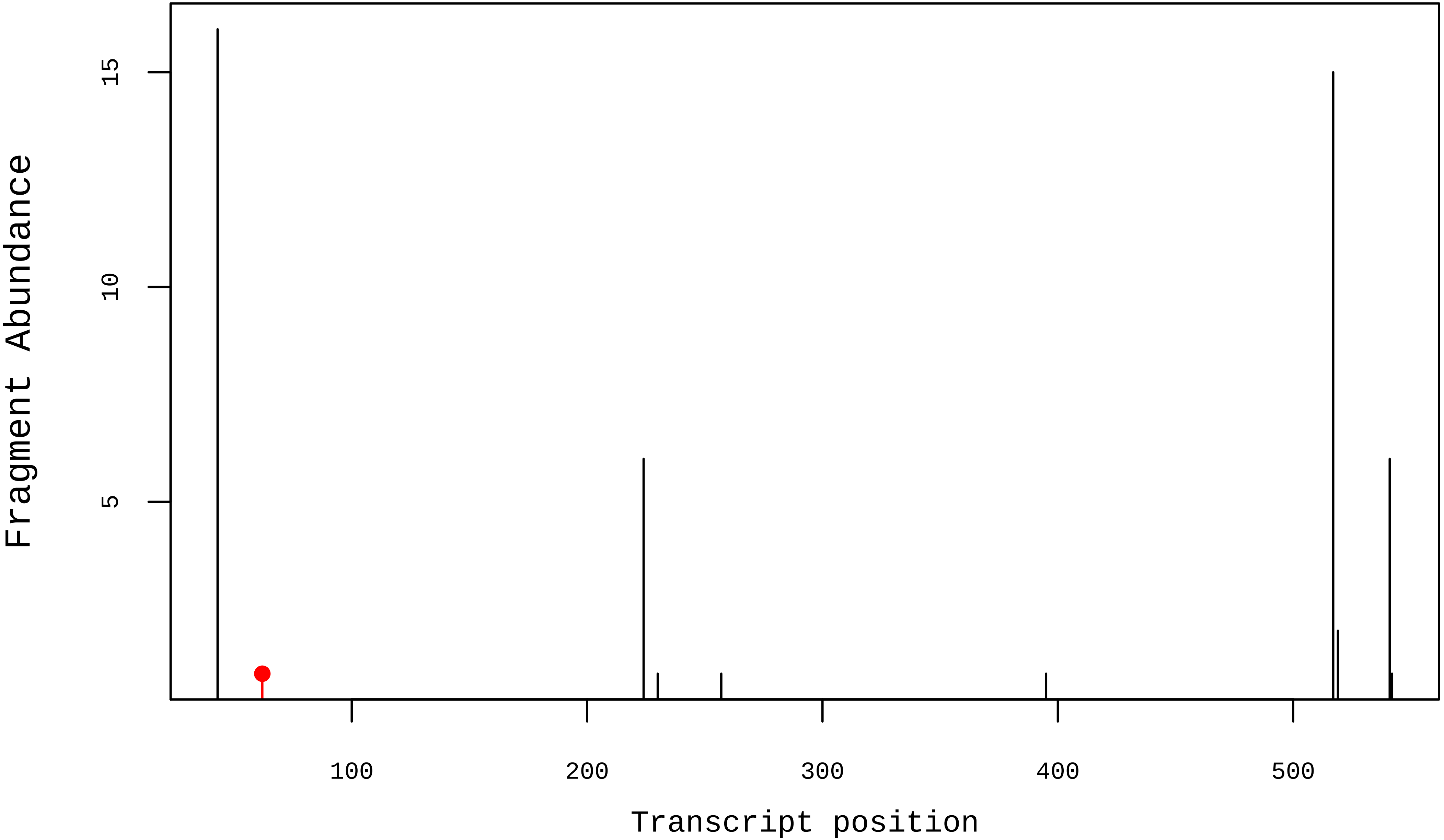

Cleavage site: 62   Tag abundance: 1   Weighted abundance: 0.143   Category: 4
sRNA abundance: 1   Alignment score: 1   MFE ratio: 0.988   p-value: 0.041

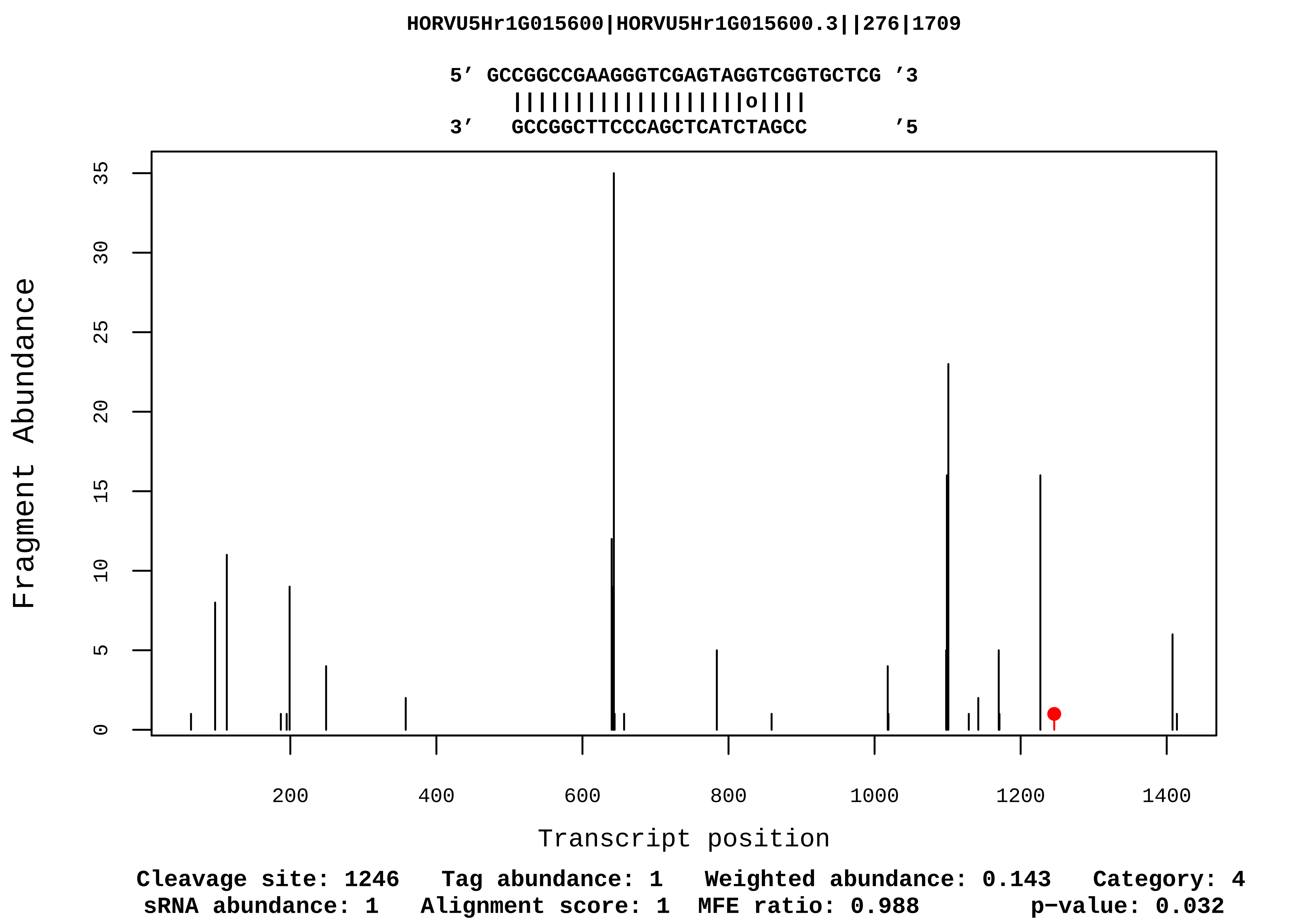

HORVU5Hr1G015600|HORVU5Hr1G015600.3||276|1709
5' GCCGGCCGAAGGGTCGAGTAGGTCGGTGCTCG '3
|||||||||||||||||||o||||
3' GCCGGCTTCCCAGCTCATCTAGCC '5
Fragment Abundance
0
5
10
15
20
25
30
35
200
400
600
800
1000
1200
1400
Transcript position
Cleavage site: 1246 Tag abundance: 1 Weighted abundance: 0.143 Category: 4
sRNA abundance: 1 Alignment score: 1 MFE ratio: 0.988 p-value: 0.032

HORVU5Hr1G015600|HORVU5Hr1G015600.4||207|1852

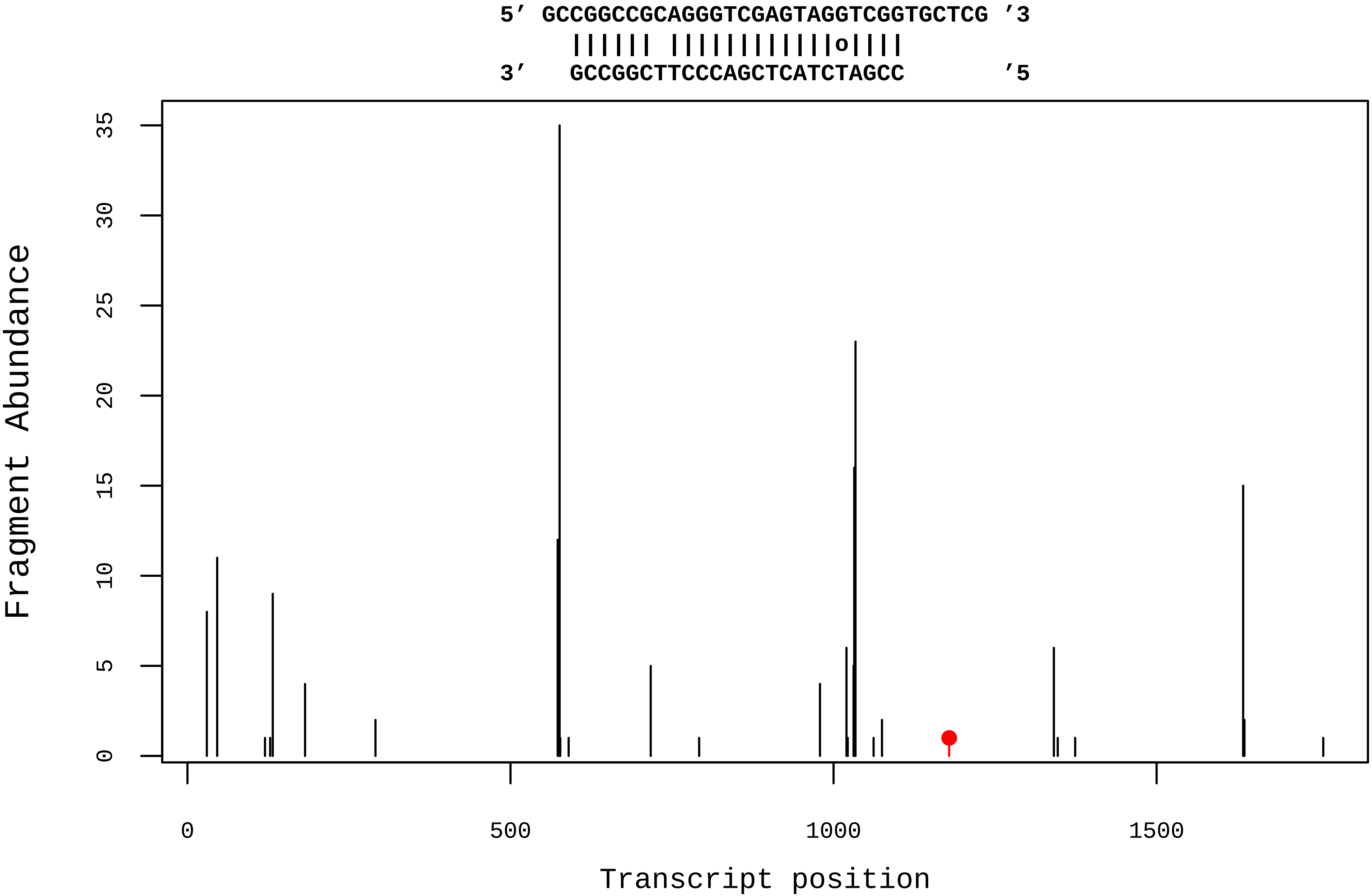

Cleavage site: 1179 Tag abundance: 1 Weighted abundance: 0.143 Category: 4
sRNA abundance: 1 Alignment score: 2 MFE ratio: 0.901 p-value: 0.041

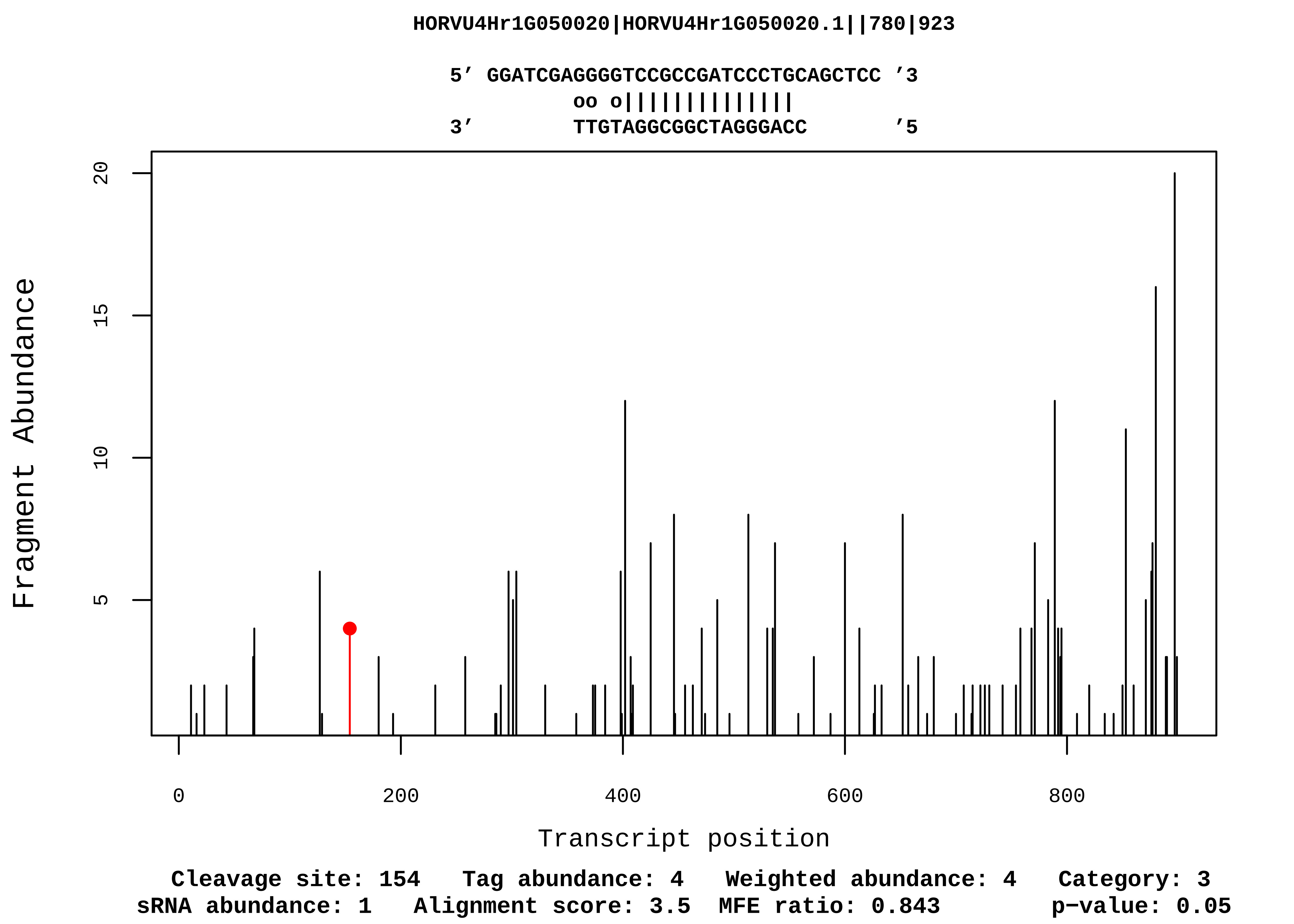
HORVU4Hr1G050020|HORVU4Hr1G050020.1||780|923
5' GGATCGAGGGGTCCGCCGATCCCTGCAGCTCC '3
oo o|||||||||||||
3' TTGTAGGCGGCTAGGGACC '5
Fragment Abundance
20
15
10
5
0
200
400
600
800
Transcript position
Cleavage site: 154 Tag abundance: 4 Weighted abundance: 4 Category: 3
sRNA abundance: 1 Alignment score: 3.5 MFE ratio: 0.843 p-value: 0.05

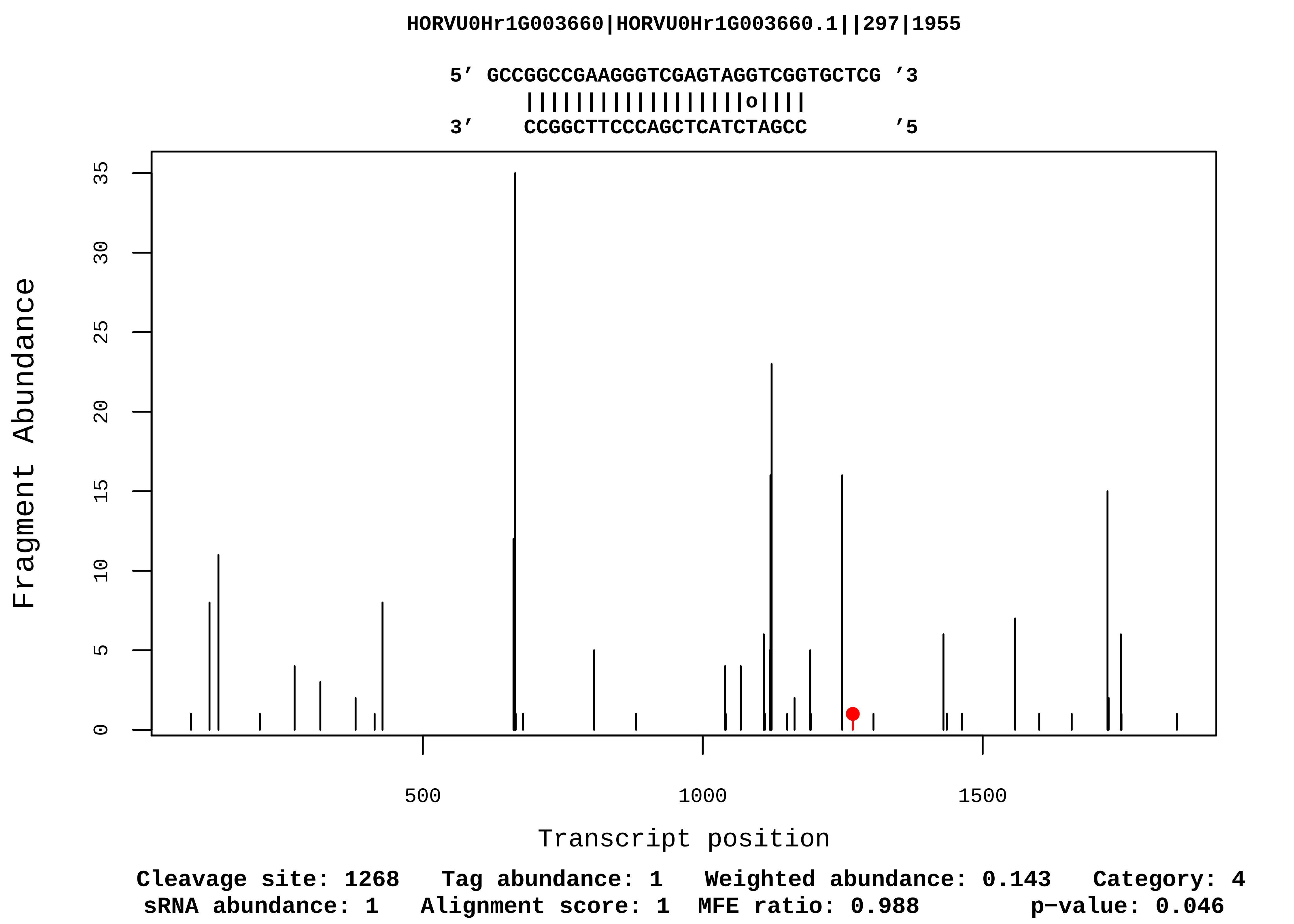
HORVU0Hr1G003660|HORVU0Hr1G003660.1||297|1955
5' GCCGGCCGAAGGGTCGAGTAGGTCGGTGCTCG '3
||||||||||||||||||o||||
3' CCGGCTTCCCAGCTCATCTAGCC '5
Fragment Abundance
0
5
10
15
20
25
30
35
500
1000
1500
Transcript position
Cleavage site: 1268 Tag abundance: 1 Weighted abundance: 0.143 Category: 4
sRNA abundance: 1 Alignment score: 1 MFE ratio: 0.988 p-value: 0.046

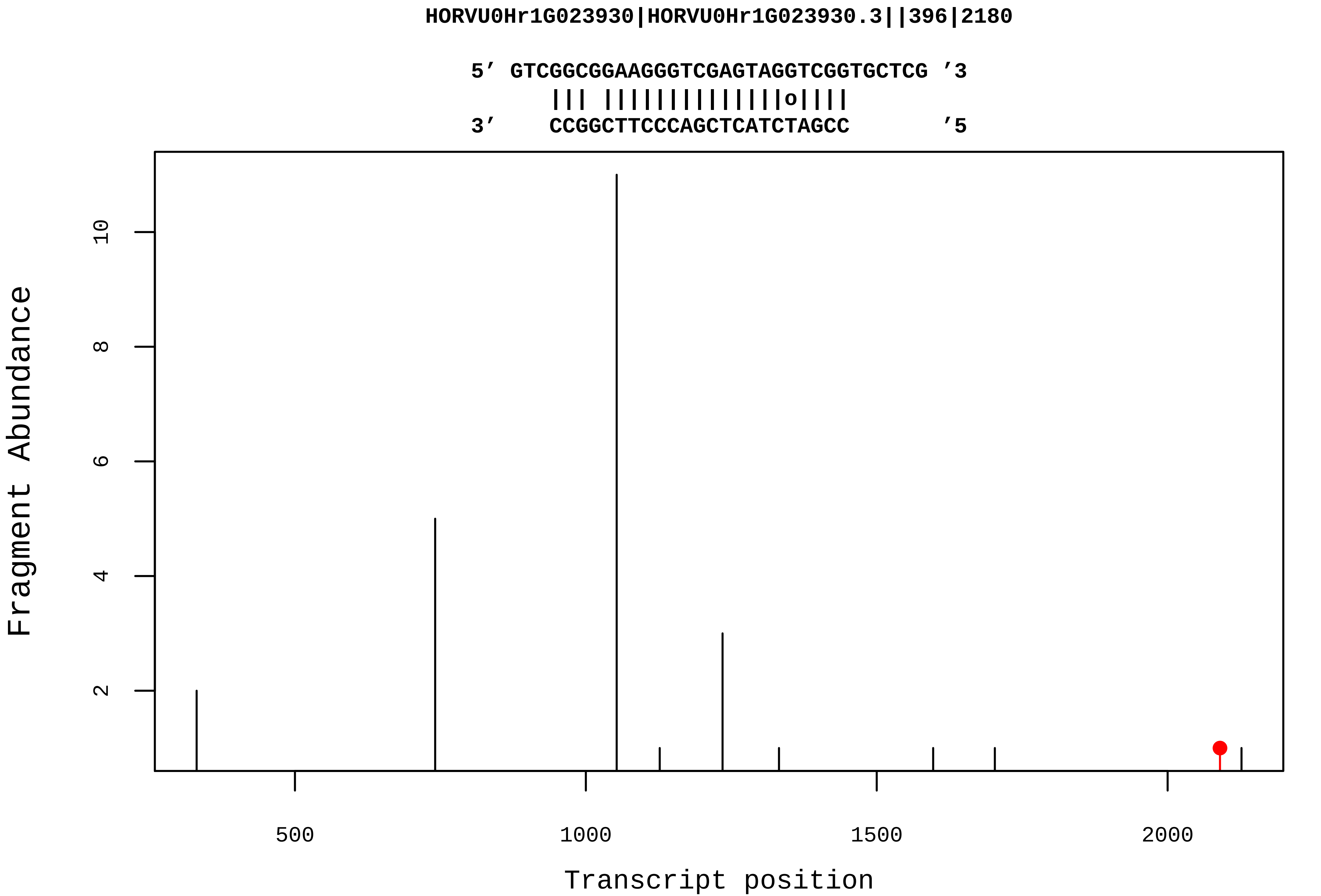

Cleavage site: 2090   Tag abundance: 1   Weighted abundance: 0.143   Category: 4
sRNA abundance: 1   Alignment score: 2   MFE ratio: 0.9   p-value: 0.019

HORVU2Hr1G072960|HORVU2Hr1G072960.1||600|3353

```
5' GCCGGCCGAAGGGTCGAGTAGGTCGGTGCTCG '3
        ||||||||||||||||||o||||
3'      CCGGCTTCCCAGCTCATCTAGCC       '5
```

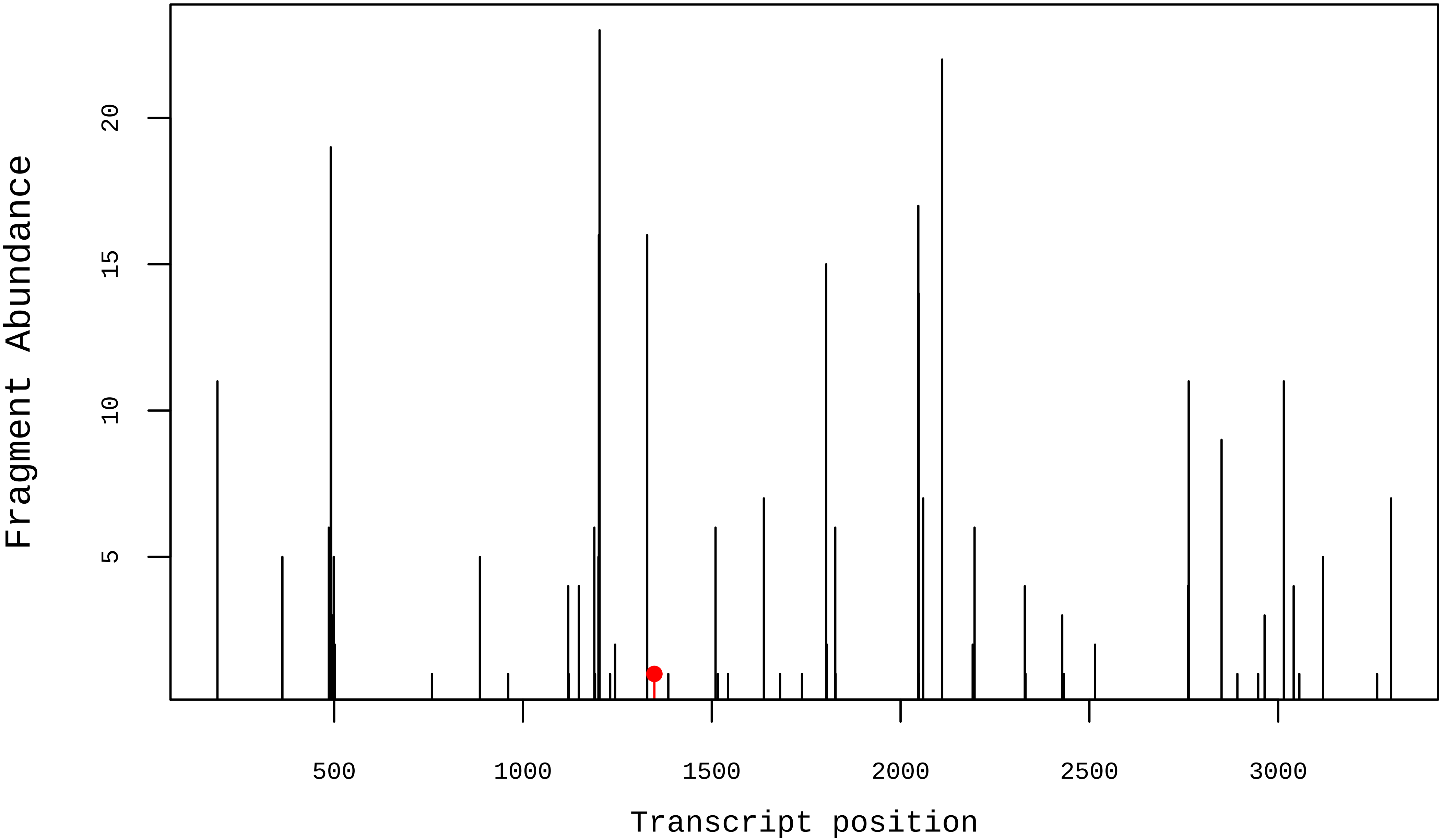

Cleavage site: 1348 Tag abundance: 1 Weighted abundance: 0.143 Category: 4
sRNA abundance: 1 Alignment score: 1 MFE ratio: 0.988 p-value: 0.03

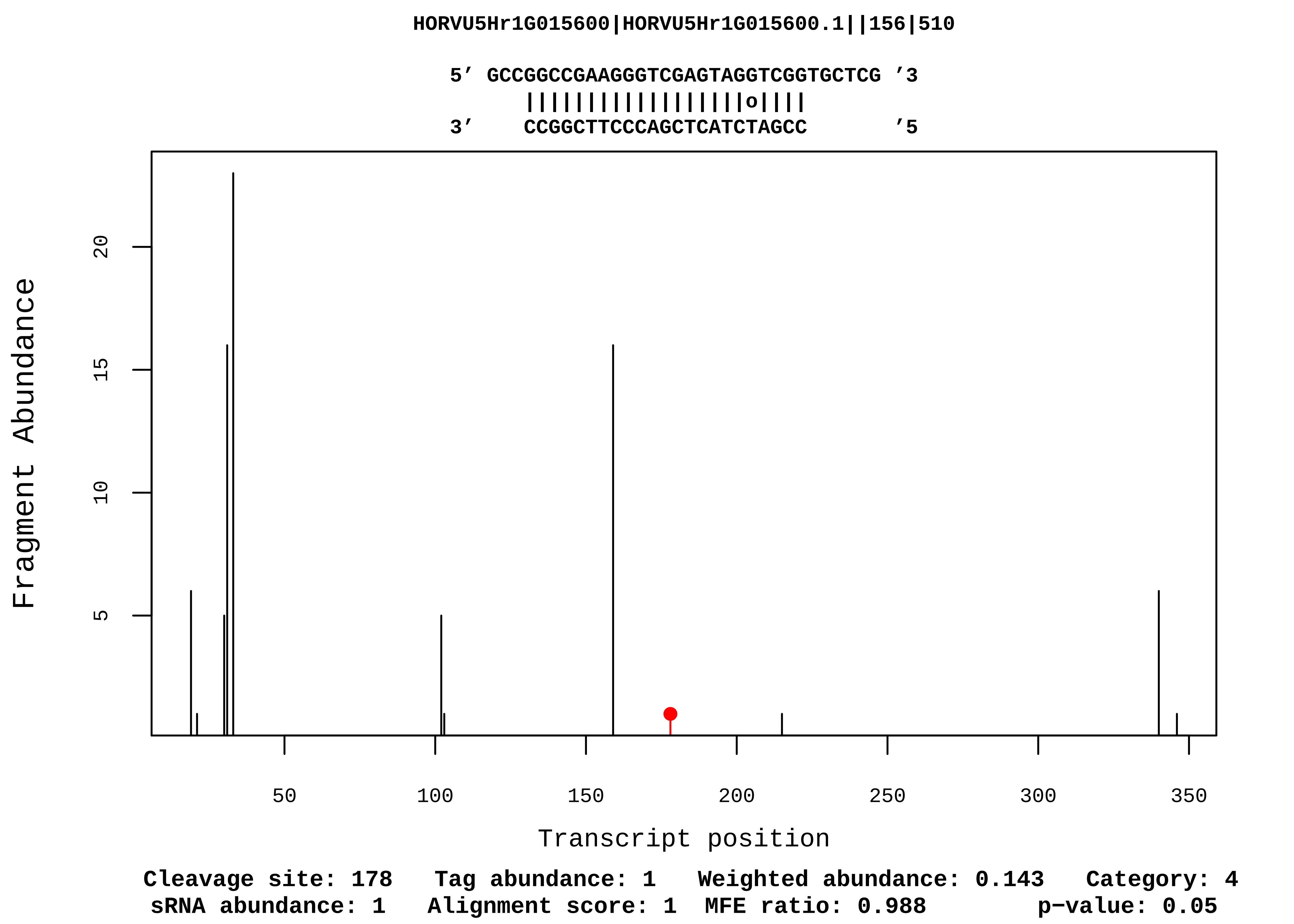
HORVU5Hr1G015600|HORVU5Hr1G015600.1||156|510
5' GCCGGCCGAAGGGTCGAGTAGGTCGGTGCTCG '3
||||||||||||||||||o||||
3' CCGGCTTCCCAGCTCATCTAGCC '5
Fragment Abundance
5
10
15
20
50
100
150
200
250
300
350
Transcript position
Cleavage site: 178 Tag abundance: 1 Weighted abundance: 0.143 Category: 4
sRNA abundance: 1 Alignment score: 1 MFE ratio: 0.988 p-value: 0.05

HORVU5Hr1G015600|HORVU5Hr1G015600.2||231|617

```
5' GCCGGCCGAAGGGTCGAGTAGGTCGGTGCTCG '3
       ||||||||||||||||||o||||
3'     CCGGCTTCCCAGCTCATCTAGCC        '5
```

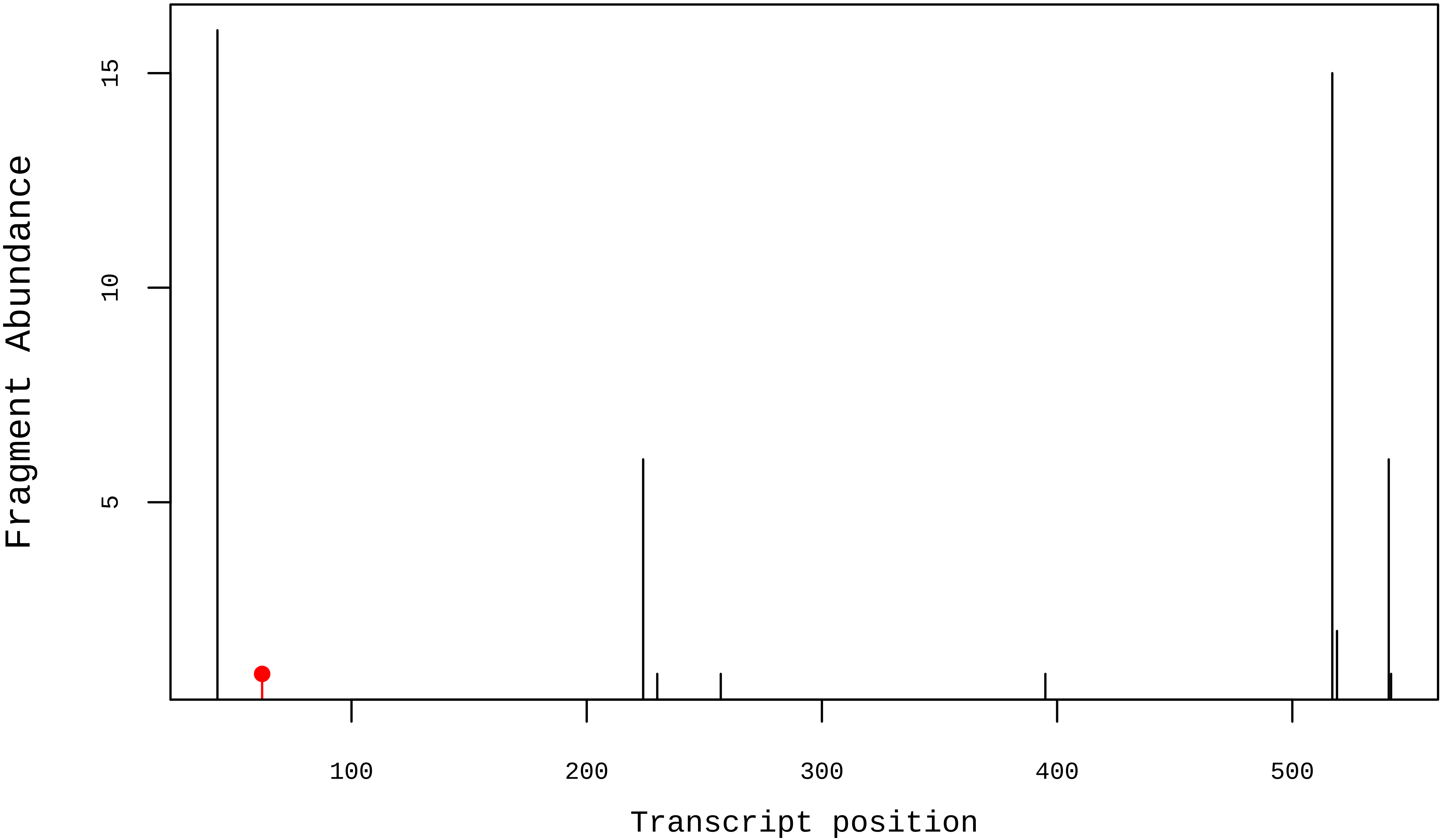

Cleavage site: 62   Tag abundance: 1   Weighted abundance: 0.143   Category: 4
sRNA abundance: 1   Alignment score: 1   MFE ratio: 0.988   p-value: 0.041

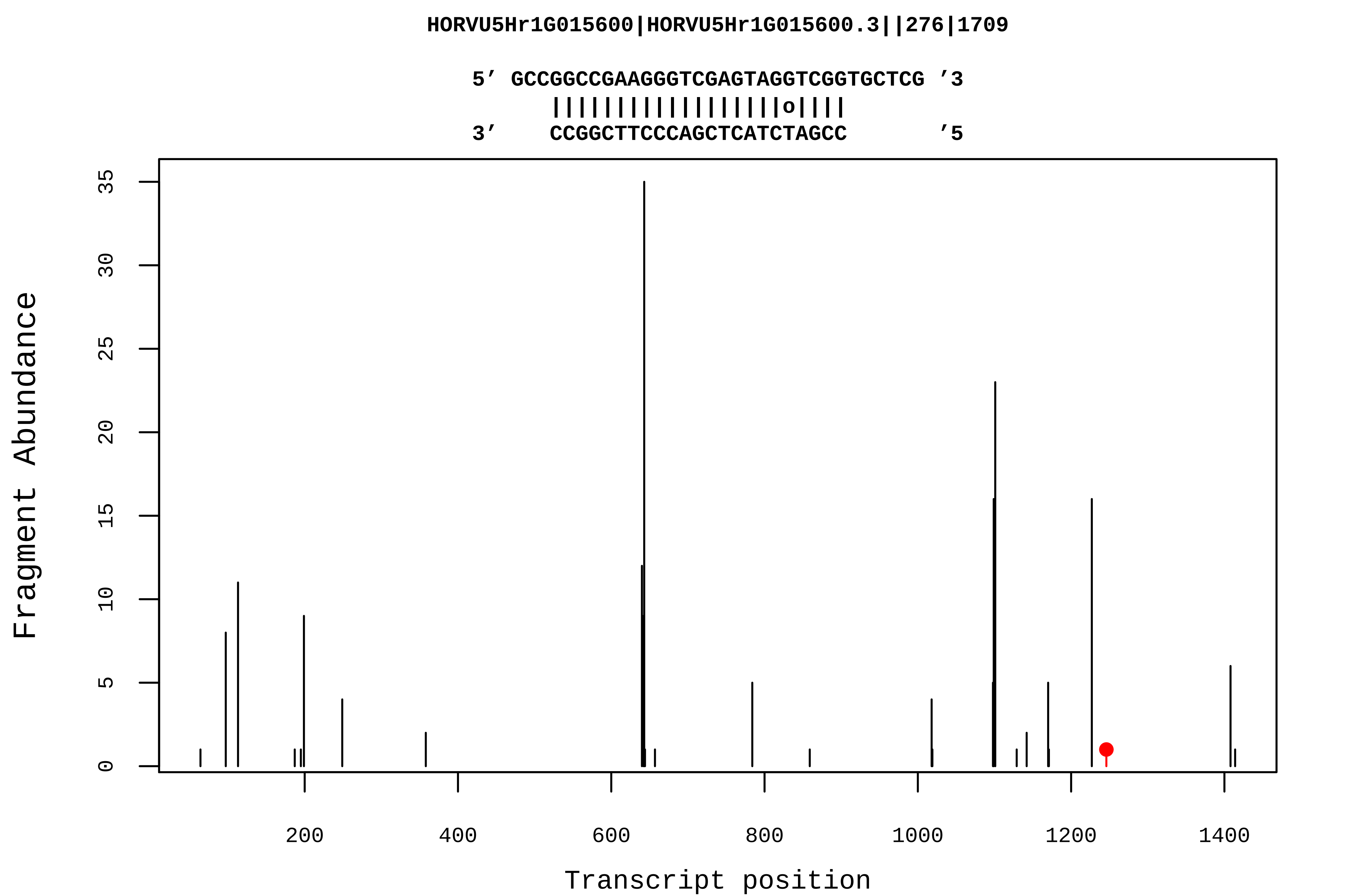

Cleavage site: 1246 Tag abundance: 1 Weighted abundance: 0.143 Category: 4
sRNA abundance: 1 Alignment score: 1 MFE ratio: 0.988 p-value: 0.032

HORVU5Hr1G015600|HORVU5Hr1G015600.4||207|1852

```
5' GCCGGCCGCAGGGTCGAGTAGGTCGGTGCTCG '3
        ||||| ||||||||||||o||||
3'      CCGGCTTCCCAGCTCATCTAGCC        '5
```

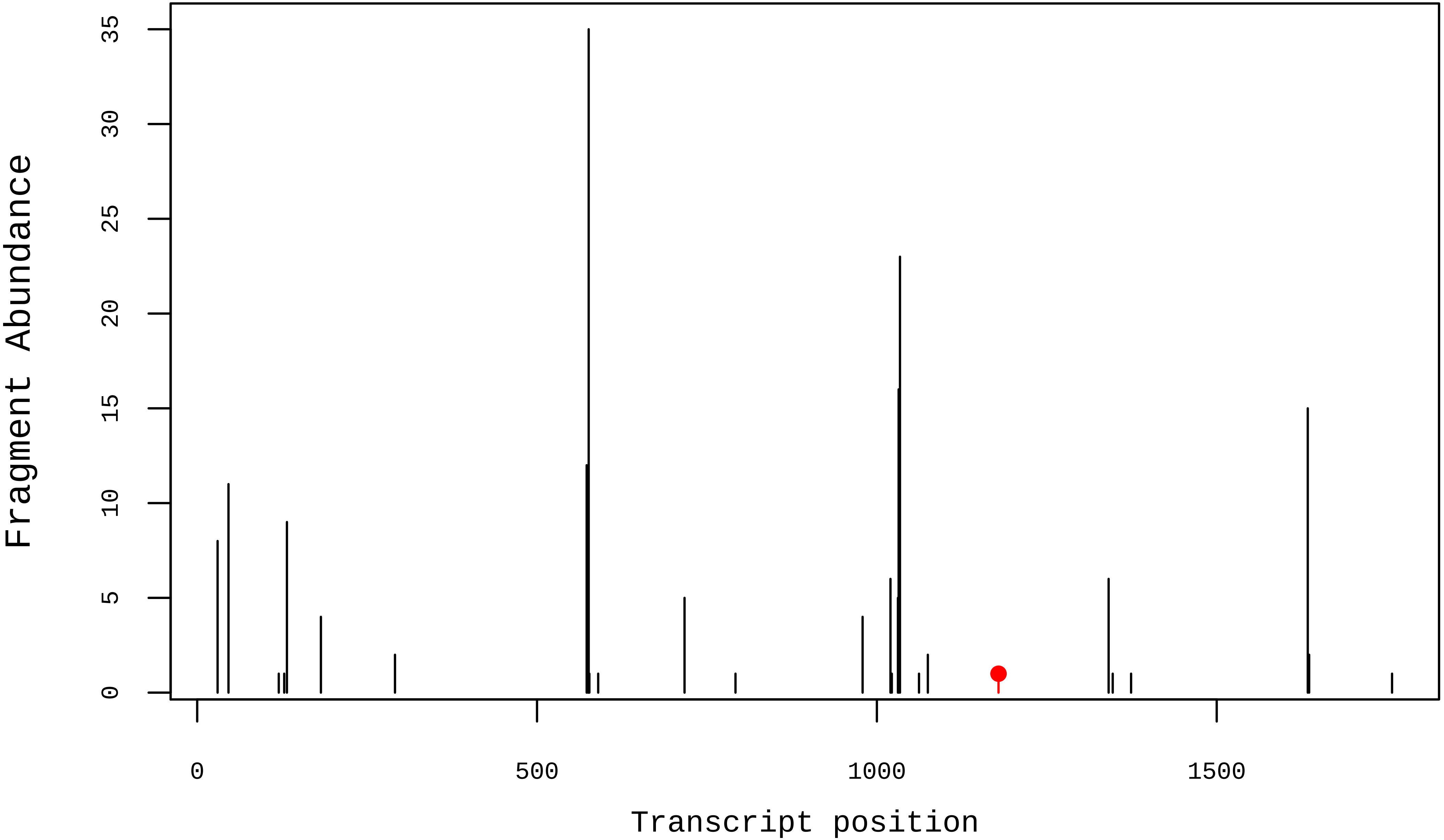

Cleavage site: 1179 Tag abundance: 1 Weighted abundance: 0.143 Category: 4
sRNA abundance: 1 Alignment score: 2 MFE ratio: 0.896 p-value: 0.041

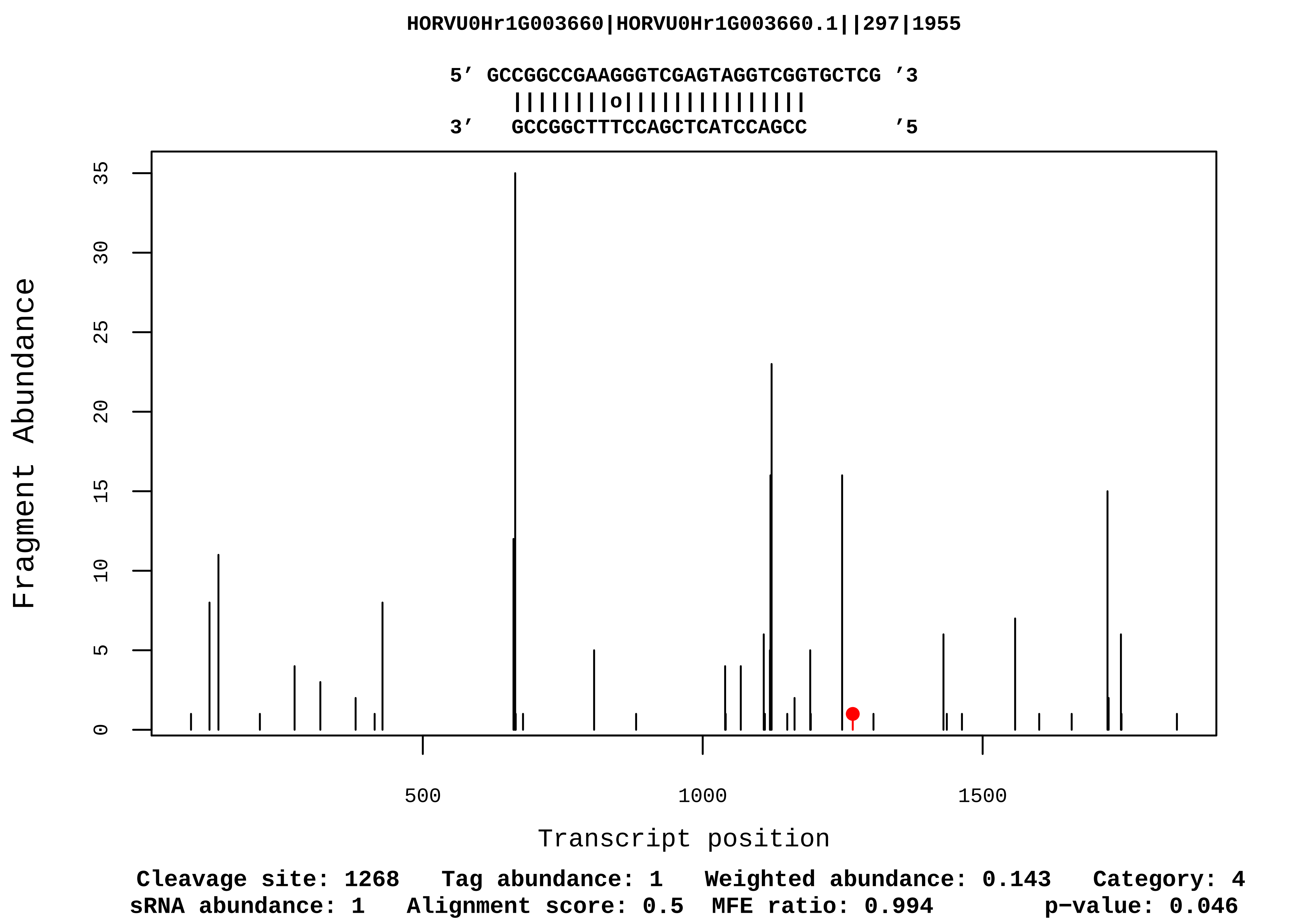
HORVU0Hr1G003660|HORVU0Hr1G003660.1||297|1955
5' GCCGGCCGAAGGGTCGAGTAGGTCGGTGCTCG '3
||||||||o|||||||||||||||
3' GCCGGCTTTCCAGCTCATCCAGCC '5
Fragment Abundance
0
5
10
15
20
25
30
35
500
1000
1500
Transcript position
Cleavage site: 1268 Tag abundance: 1 Weighted abundance: 0.143 Category: 4
sRNA abundance: 1 Alignment score: 0.5 MFE ratio: 0.994 p-value: 0.046

HORVU0Hr1G023930|HORVU0Hr1G023930.3||396|2180

```
5' GTCGGCGGAAGGGTCGAGTAGGTCGGTGCTCG '3
      |||| |||o||||||||||||||
3'    GCCGGCTTTCCAGCTCATCCAGCC       '5
```

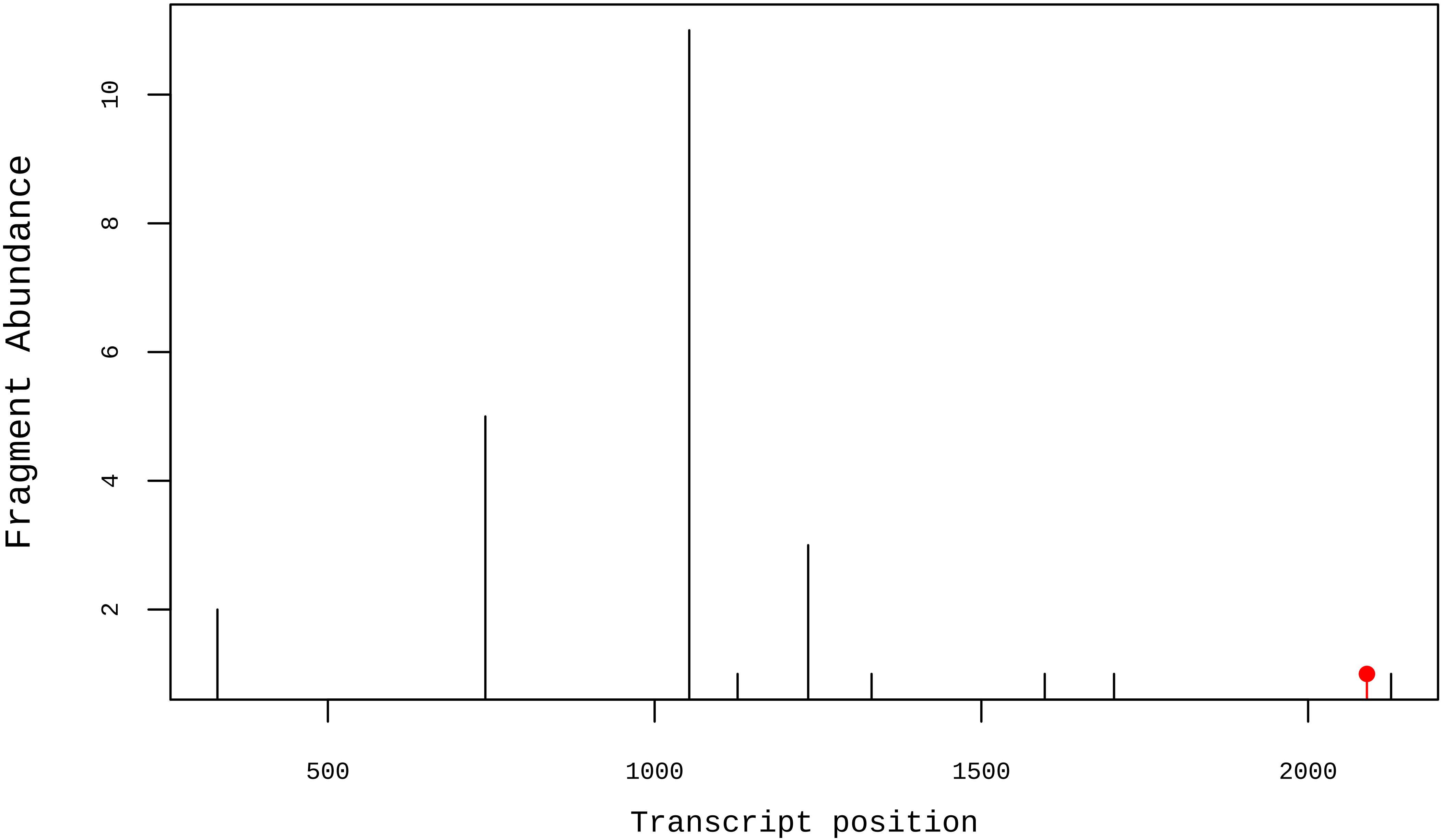

Cleavage site: 2090   Tag abundance: 1   Weighted abundance: 0.143   Category: 4
sRNA abundance: 1   Alignment score: 1.5   MFE ratio: 0.91   p-value: 0.019

HORVU2Hr1G072960|HORVU2Hr1G072960.1||600|3353

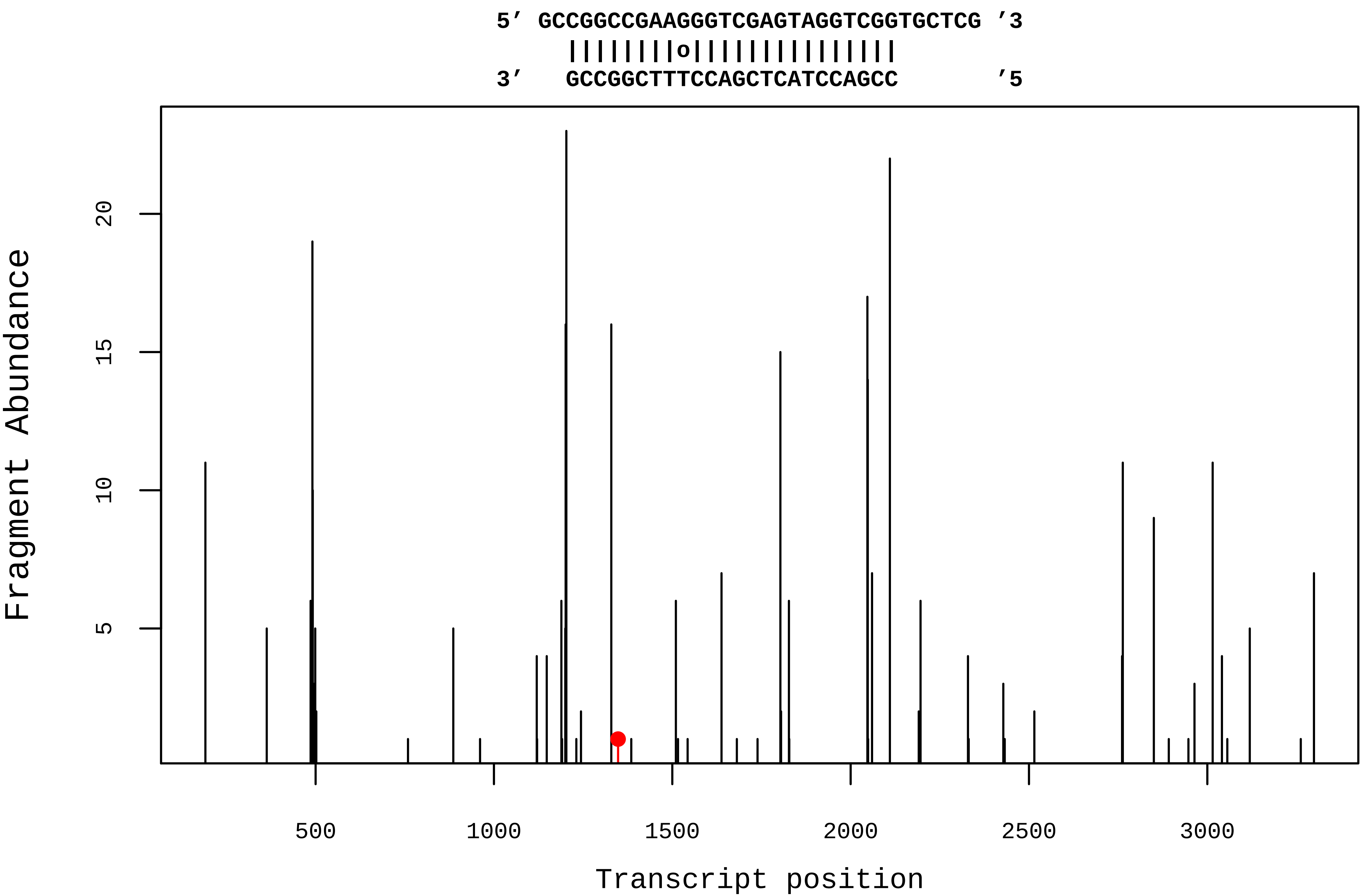

Cleavage site: 1348 Tag abundance: 1 Weighted abundance: 0.143 Category: 4
sRNA abundance: 1 Alignment score: 0.5 MFE ratio: 0.994 p-value: 0.03

HORVU5Hr1G015600|HORVU5Hr1G015600.1||156|510

```
5' GCCGGCCGAAGGGTCGAGTAGGTCGGTGCTCG '3
      ||||||||o|||||||||||||||
3'    GCCGGCTTTCCAGCTCATCCAGCC       '5
```

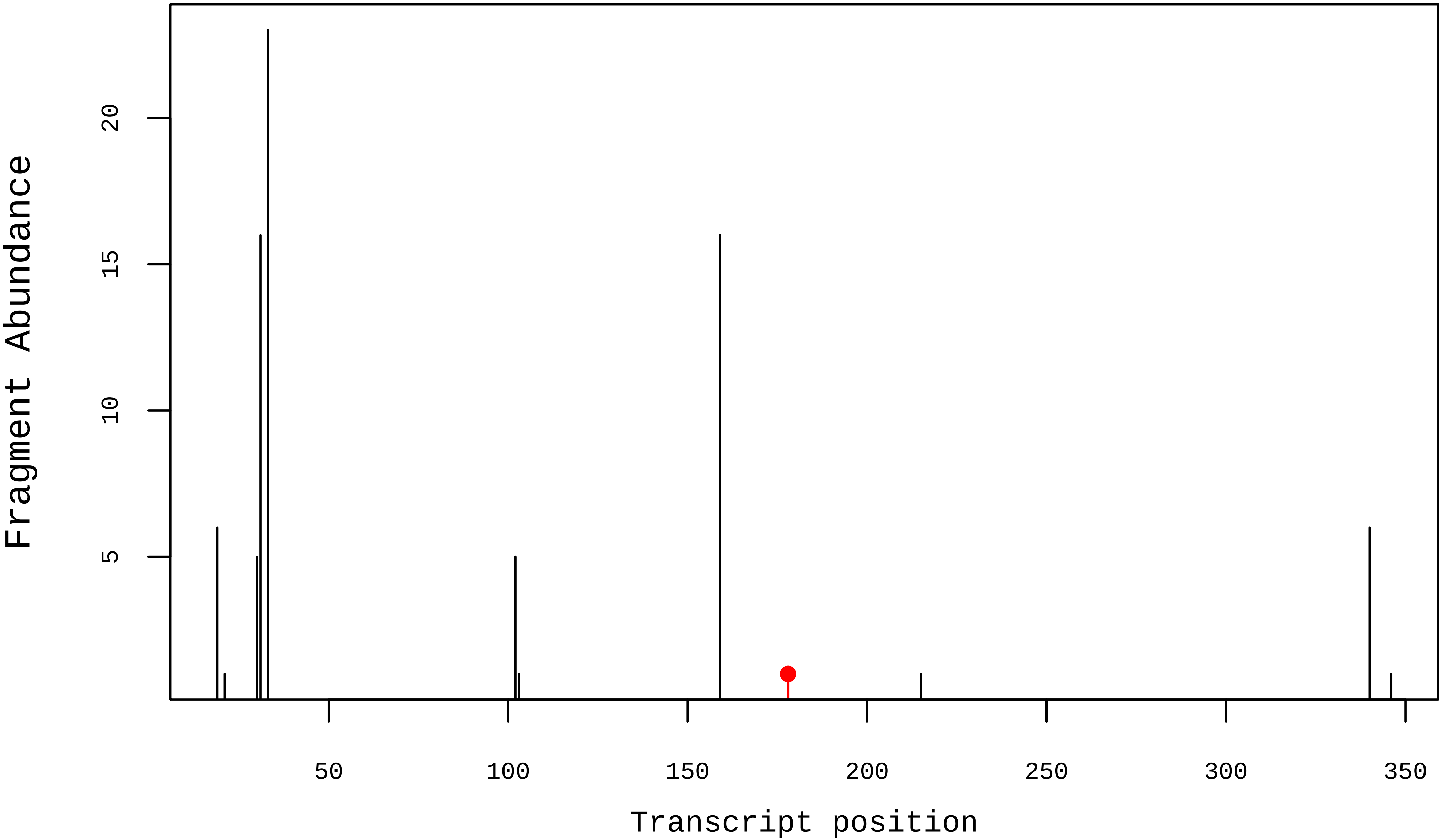

Cleavage site: 178   Tag abundance: 1   Weighted abundance: 0.143   Category: 4
sRNA abundance: 1   Alignment score: 0.5   MFE ratio: 0.994   p-value: 0.05

HORVU5Hr1G015600|HORVU5Hr1G015600.2||231|617

```
5' GCCGGCCGAAGGGTCGAGTAGGTCGGTGCTCG '3
       ||||||||o|||||||||||||||
3'     GCCGGCTTTCCAGCTCATCCAGCC        '5
```

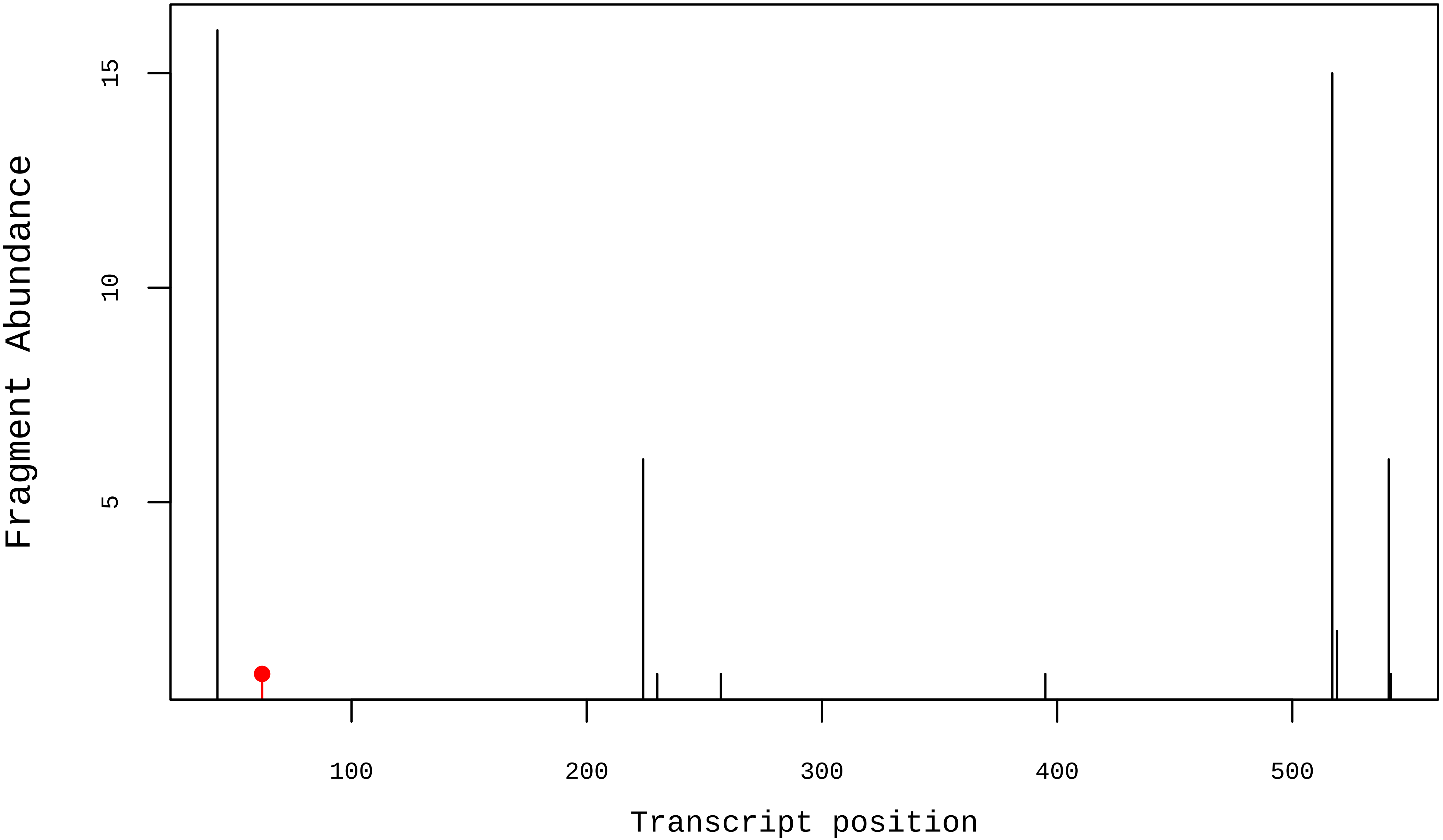

Cleavage site: 62   Tag abundance: 1   Weighted abundance: 0.143   Category: 4
sRNA abundance: 1   Alignment score: 0.5   MFE ratio: 0.994   p-value: 0.041

HORVU5Hr1G015600|HORVU5Hr1G015600.3||276|1709

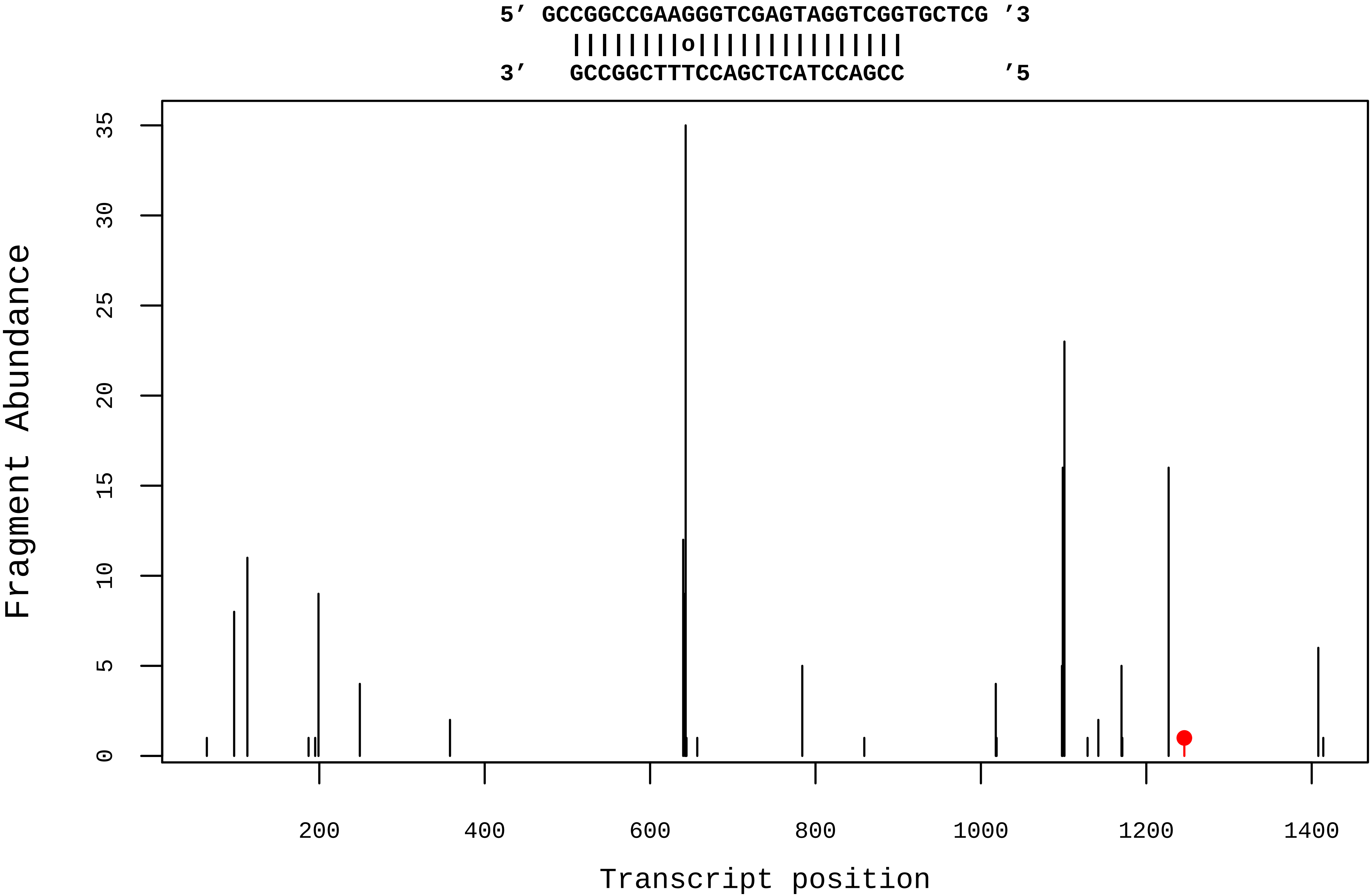

Cleavage site: 1246 Tag abundance: 1 Weighted abundance: 0.143 Category: 4
sRNA abundance: 1 Alignment score: 0.5 MFE ratio: 0.994 p-value: 0.032

HORVU5Hr1G015600|HORVU5Hr1G015600.4||207|1852

```
5' GCCGGCCGCAGGGTCGAGTAGGTCGGTGCTCG '3
       |||||| |o|||||||||||||||
3'     GCCGGCTTTCCAGCTCATCCAGCC       '5
```

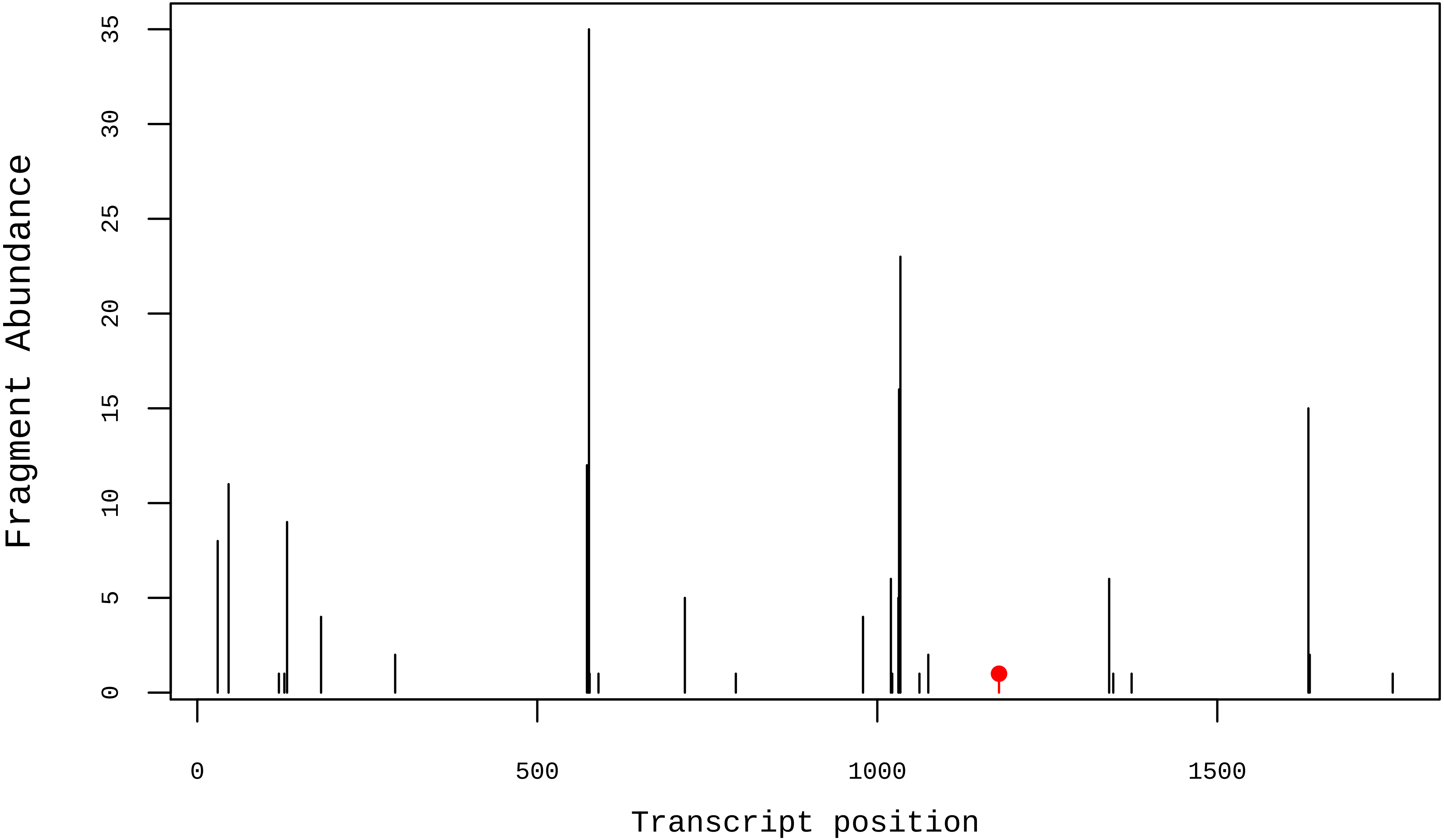

Cleavage site: 1179   Tag abundance: 1   Weighted abundance: 0.143   Category: 4
sRNA abundance: 1   Alignment score: 1.5   MFE ratio: 0.906   p-value: 0.041

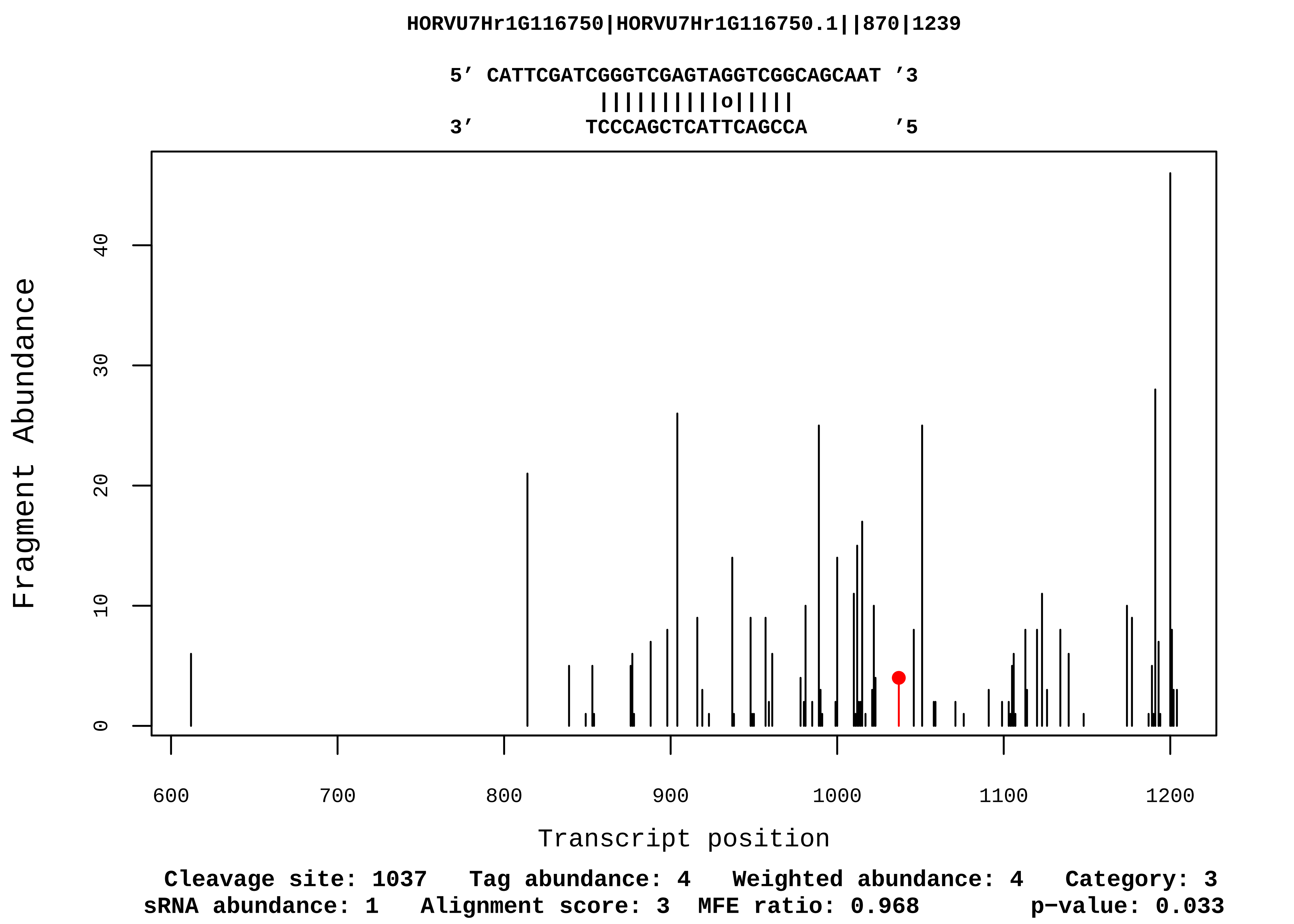
HORVU7Hr1G116750|HORVU7Hr1G116750.1||870|1239
5' CATTCGATCGGGTCGAGTAGGTCGGCAGCAAT '3
||||||||||o|||||
3' TCCCAGCTCATTCAGCCA '5
Fragment Abundance
0
10
20
30
40
600
700
800
900
1000
1100
1200
Transcript position
Cleavage site: 1037 Tag abundance: 4 Weighted abundance: 4 Category: 3
sRNA abundance: 1 Alignment score: 3 MFE ratio: 0.968 p-value: 0.033

HORVU7Hr1G116750|HORVU7Hr1G116750.1||870|1239

```
5' CATTCGATCGGGTCGAGTAGGTCGGCAGCAAT '3
       o|||  ||||||||||||||||
3'     GGCTT-CCCAGCTCATCCAGCCA       '5
```

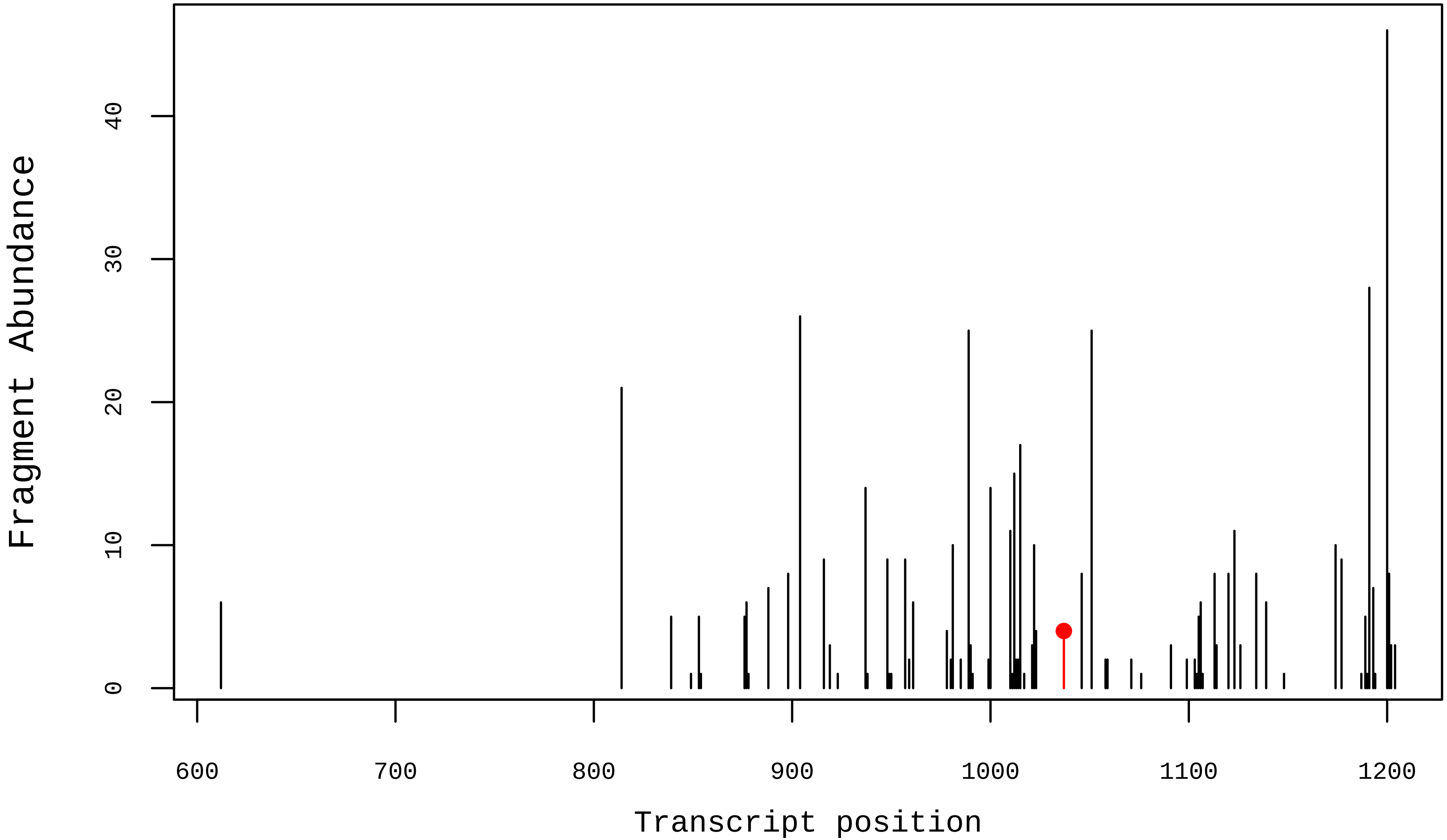

Cleavage site: 1037 Tag abundance: 4 Weighted abundance: 4 Category: 3
sRNA abundance: 1 Alignment score: 3.5 MFE ratio: 0.854 p-value: 0.033

HORVU0Hr1G003660|HORVU0Hr1G003660.1||297|1955

```
5' GCCGGCCGAAGGGTCGAGTAGGTCGGTGCTCG '3
       |||||||||||||||||||| |||
3'     GCCGGCTTCCCAGCTCATCCA-CCA      '5
```

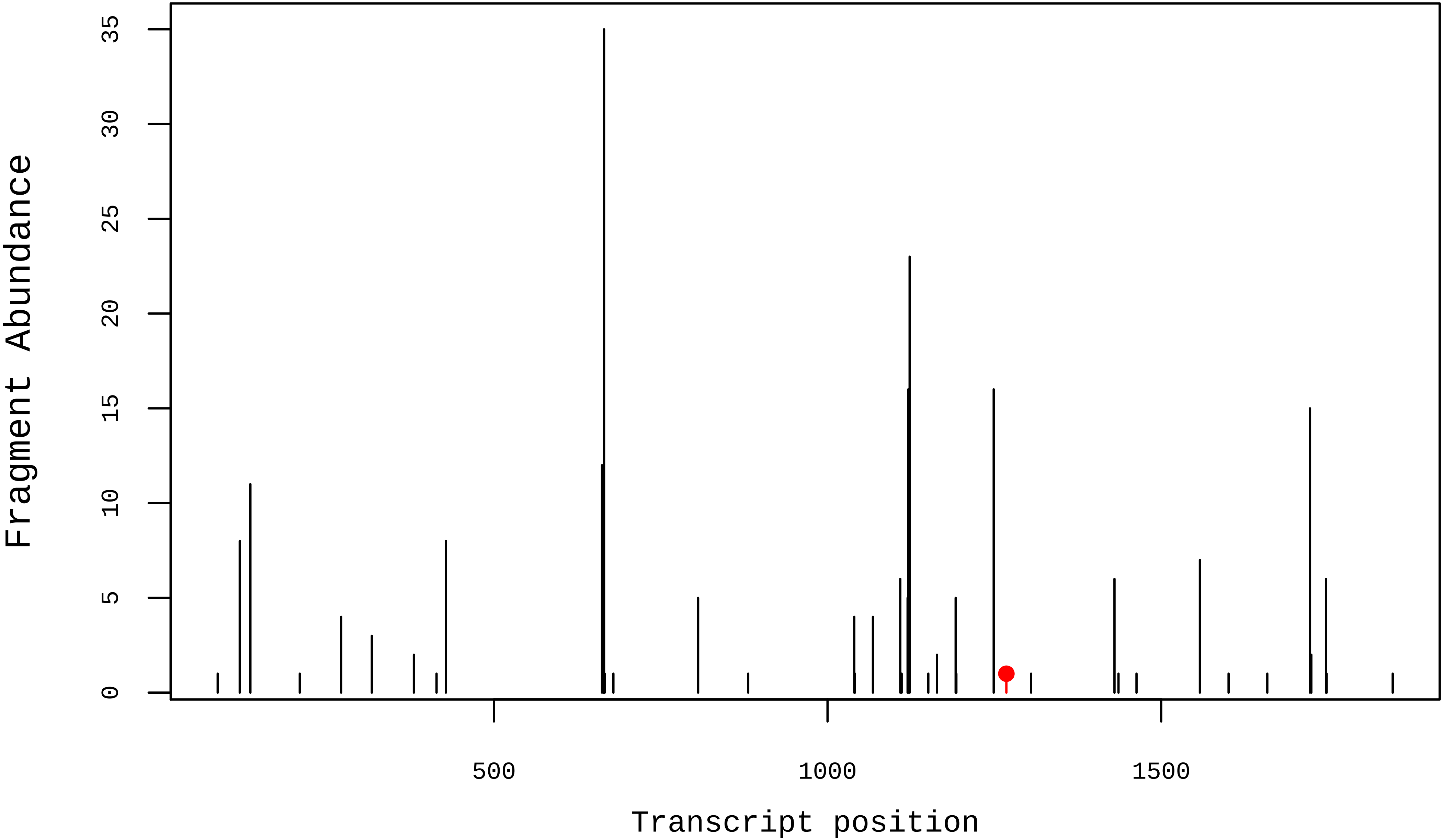

Cleavage site: 1268   Tag abundance: 1   Weighted abundance: 0.143   Category: 4
sRNA abundance: 1   Alignment score: 2   MFE ratio: 0.937   p-value: 0.046

HORVU0Hr1G023930|HORVU0Hr1G023930.3||396|2180

```
5' GTCGGCGGAAGGGTCGAGTAGGTCGGTGCTCG '3
       |||| ||||||||||||||||| |||
3'     GCCGGCTTCCCAGCTCATCCA-CCA       '5
```

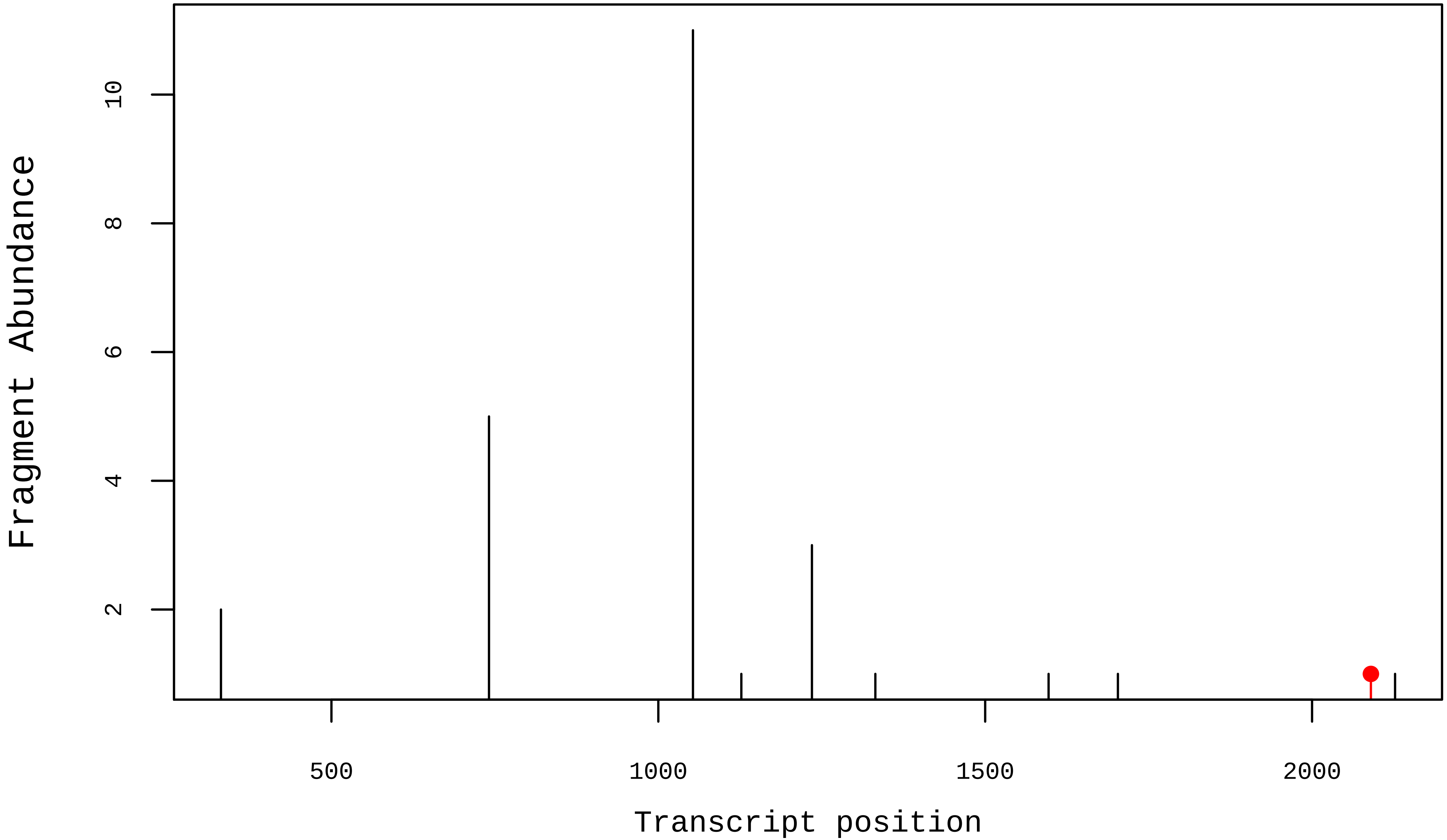

Cleavage site: 2090   Tag abundance: 1   Weighted abundance: 0.143   Category: 4
sRNA abundance: 1   Alignment score: 3   MFE ratio: 0.855   p-value: 0.019

HORVU2Hr1G072960|HORVU2Hr1G072960.1||600|3353

```
5' GCCGGCCGAAGGGTCGAGTAGGTCGGTGCTCG '3
      ||||||||||||||||||||| |||
3'    GCCGGCTTCCCAGCTCATCCA-CCA      '5
```

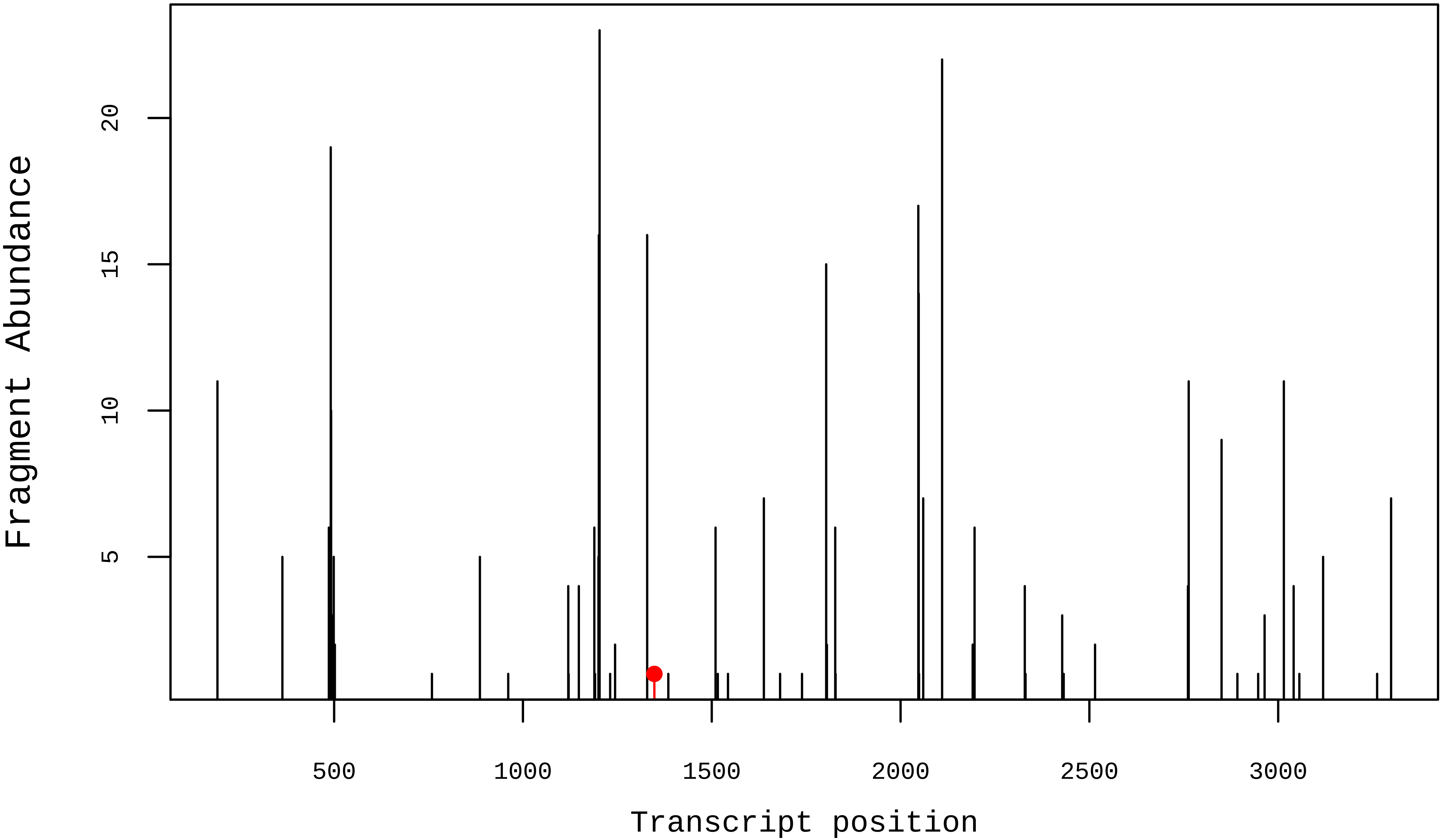

Cleavage site: 1348   Tag abundance: 1   Weighted abundance: 0.143   Category: 4
sRNA abundance: 1   Alignment score: 2   MFE ratio: 0.937   p-value: 0.03

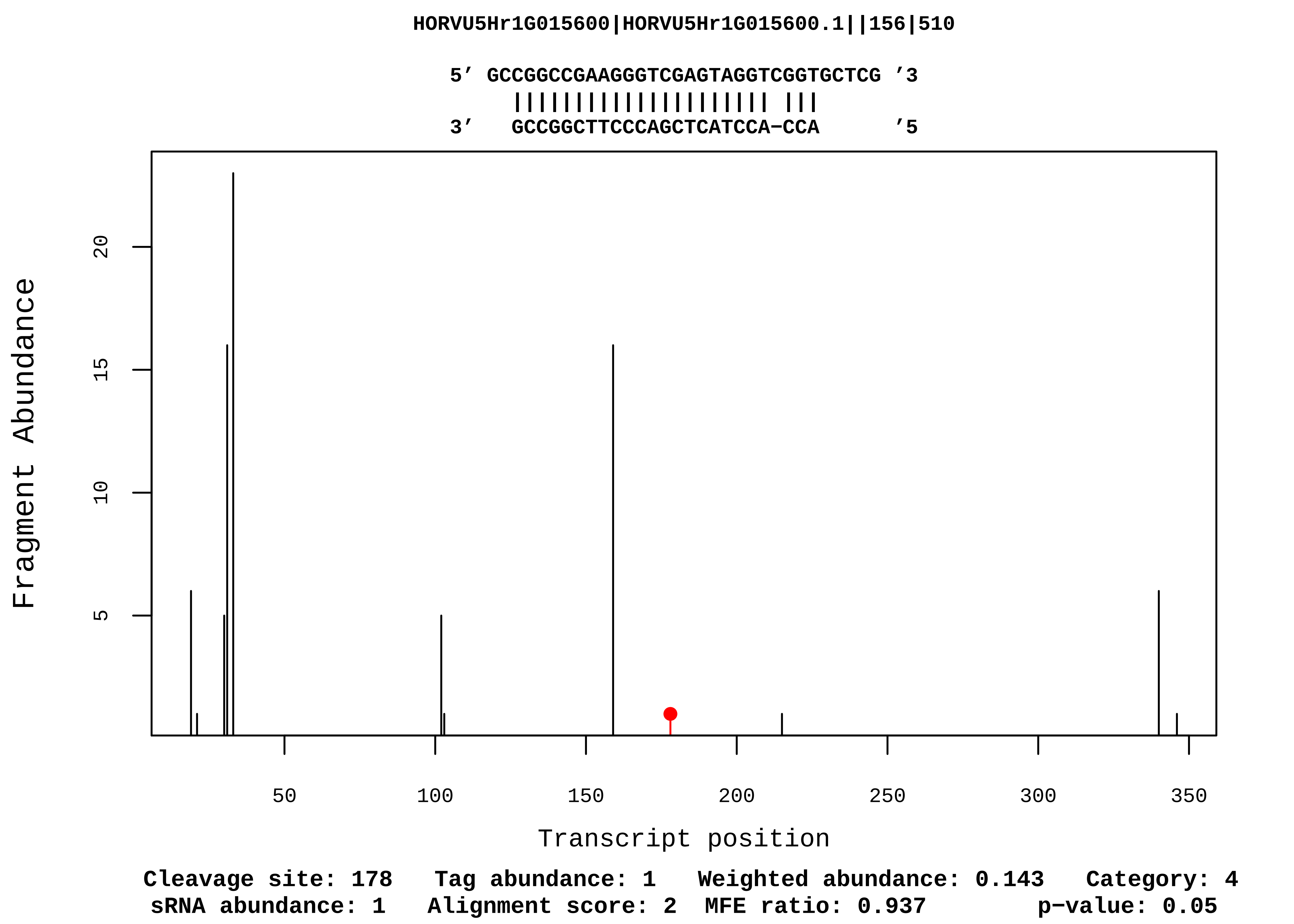
HORVU5Hr1G015600|HORVU5Hr1G015600.1||156|510
5′ GCCGGCCGAAGGGTCGAGTAGGTCGGTGCTCG ′3
3′ GCCGGCTTCCCAGCTCATCCA-CCA ′5
Fragment Abundance
5
10
15
20
50
100
150
200
250
300
350
Transcript position
Cleavage site: 178 Tag abundance: 1 Weighted abundance: 0.143 Category: 4
sRNA abundance: 1 Alignment score: 2 MFE ratio: 0.937 p-value: 0.05

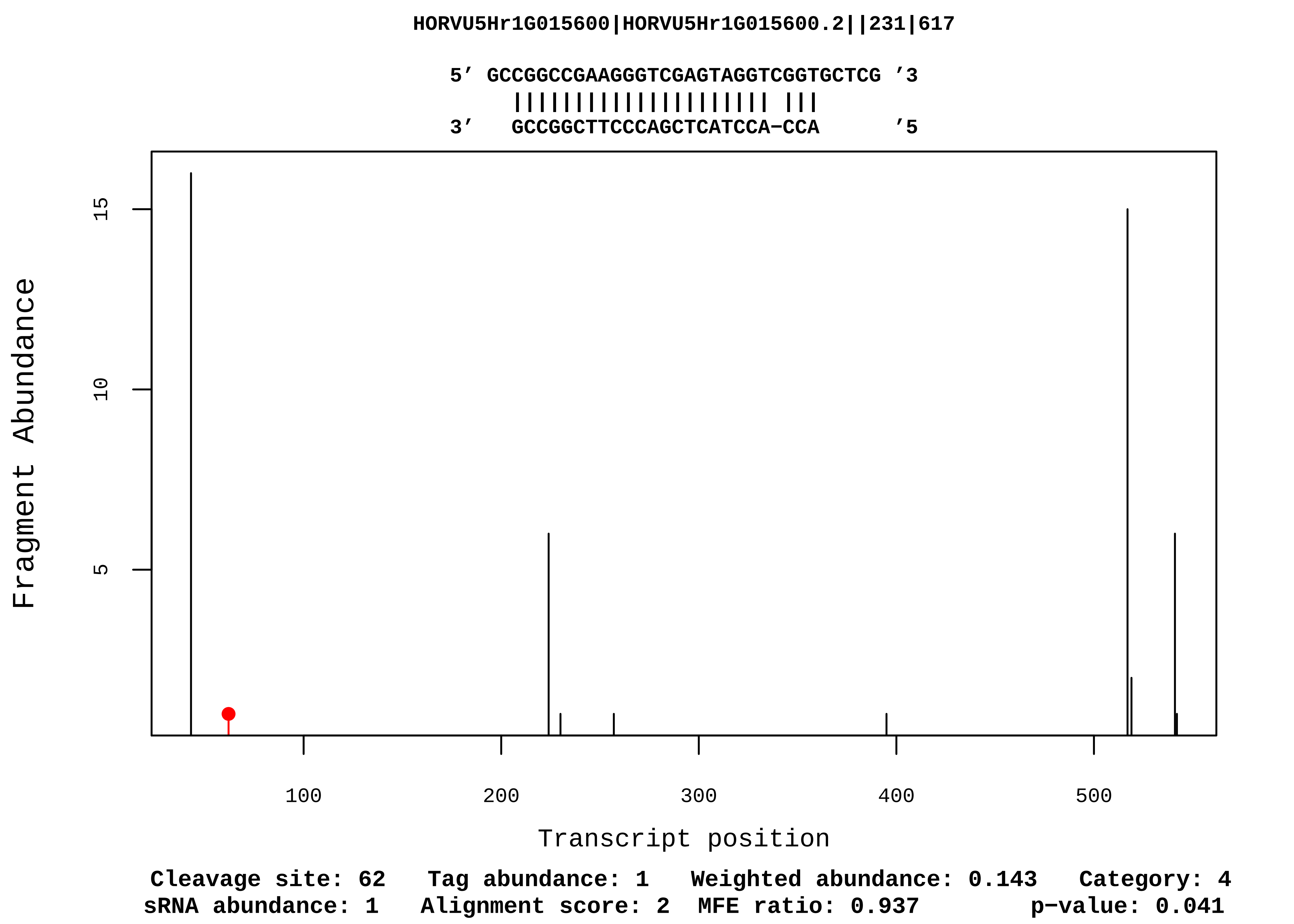
HORVU5Hr1G015600|HORVU5Hr1G015600.2||231|617
5' GCCGGCCGAAGGGTCGAGTAGGTCGGTGCTCG '3
|||||||||||||||||||| |||
3' GCCGGCTTCCCAGCTCATCCA-CCA '5
Fragment Abundance
5
10
15
100
200
300
400
500
Transcript position
Cleavage site: 62 Tag abundance: 1 Weighted abundance: 0.143 Category: 4
sRNA abundance: 1 Alignment score: 2 MFE ratio: 0.937 p-value: 0.041

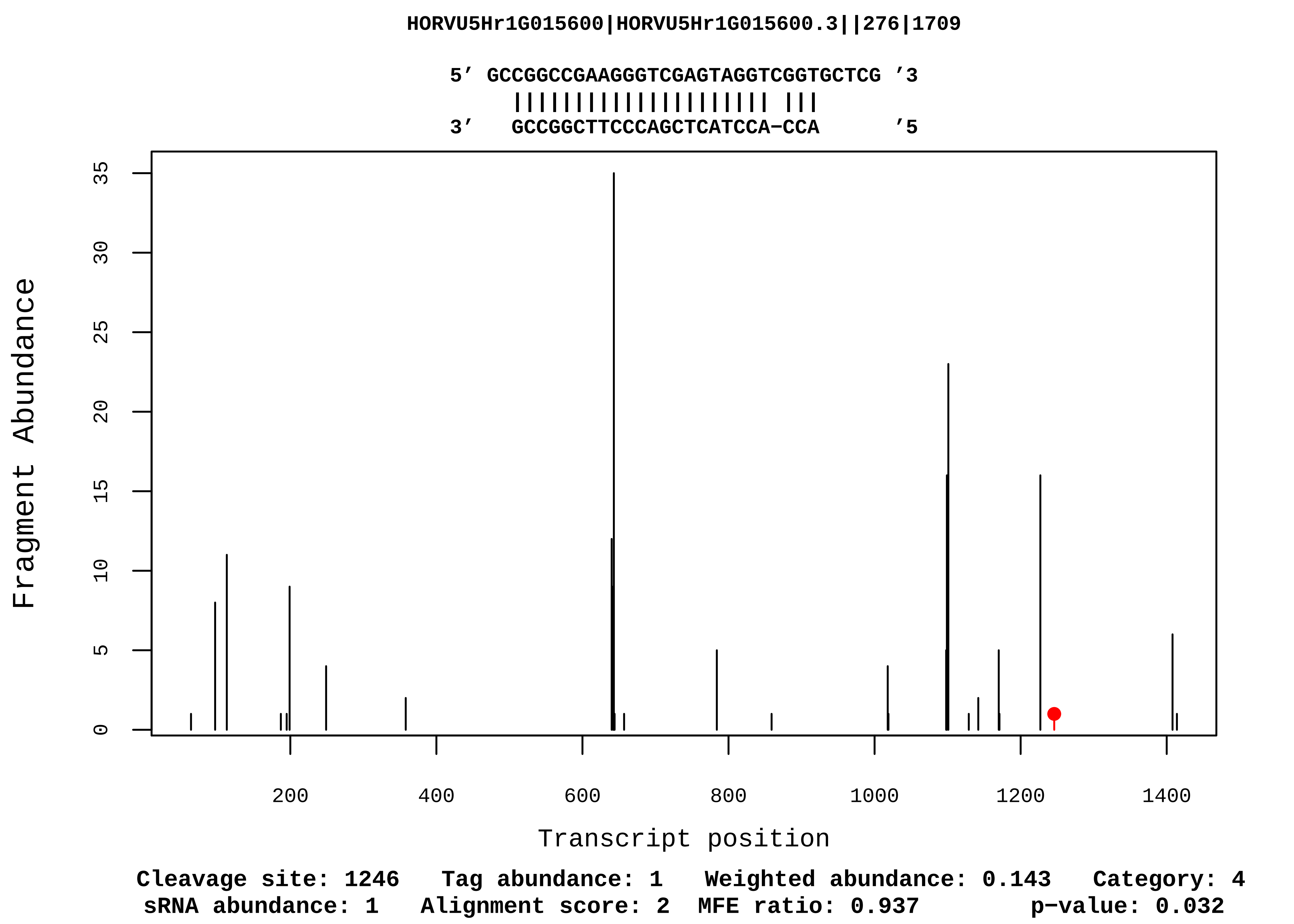

HORVU5Hr1G015600|HORVU5Hr1G015600.3||276|1709
5′ GCCGGCCGAAGGGTCGAGTAGGTCGGTGCTCG ′3
|||||||||||||||||||| |||
3′ GCCGGCTTCCCAGCTCATCCA-CCA ′5
Fragment Abundance
0
5
10
15
20
25
30
35
200
400
600
800
1000
1200
1400
Transcript position
Cleavage site: 1246 Tag abundance: 1 Weighted abundance: 0.143 Category: 4
sRNA abundance: 1 Alignment score: 2 MFE ratio: 0.937 p-value: 0.032

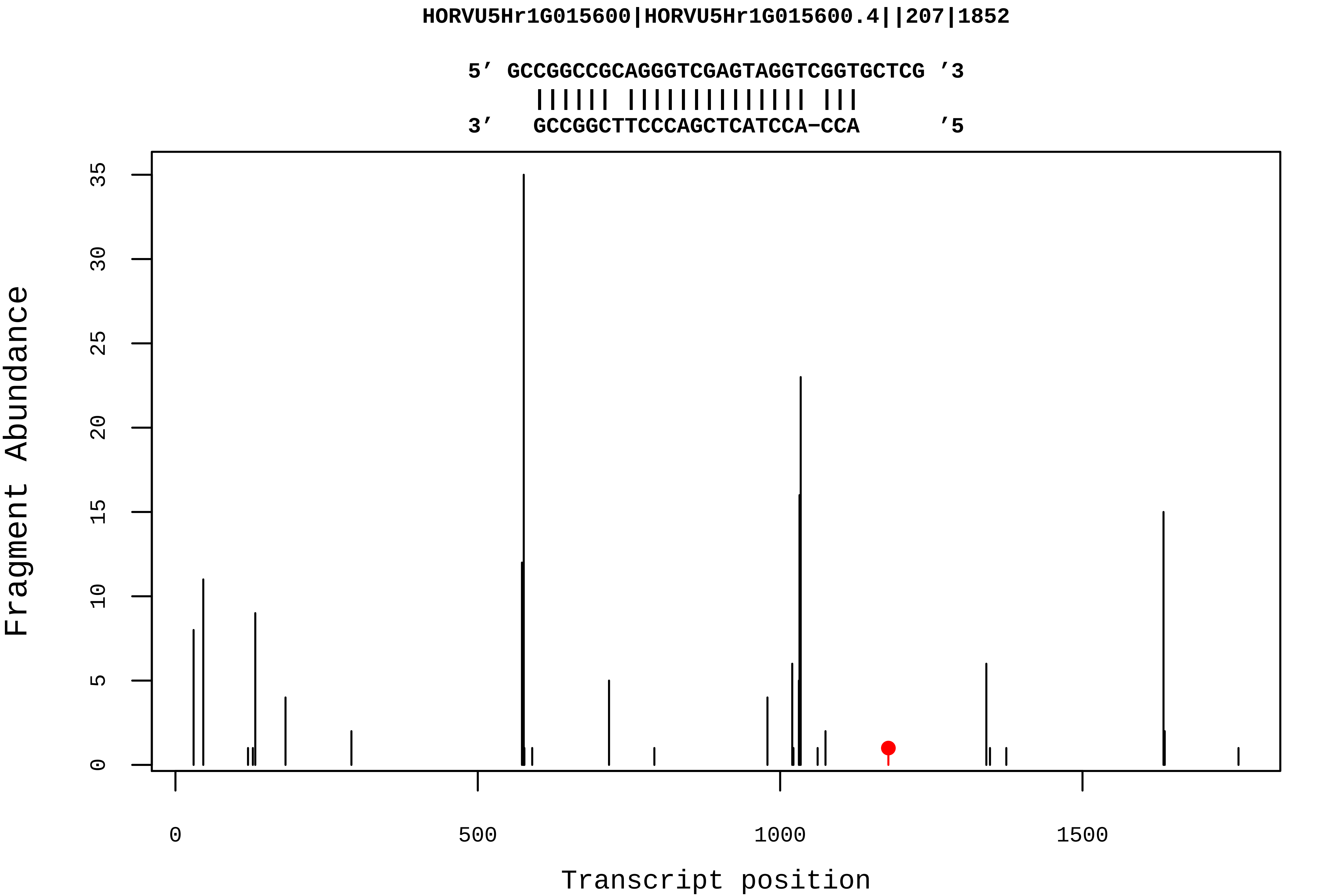

Cleavage site: 1179 Tag abundance: 1 Weighted abundance: 0.143 Category: 4
sRNA abundance: 1 Alignment score: 3 MFE ratio: 0.851 p-value: 0.041

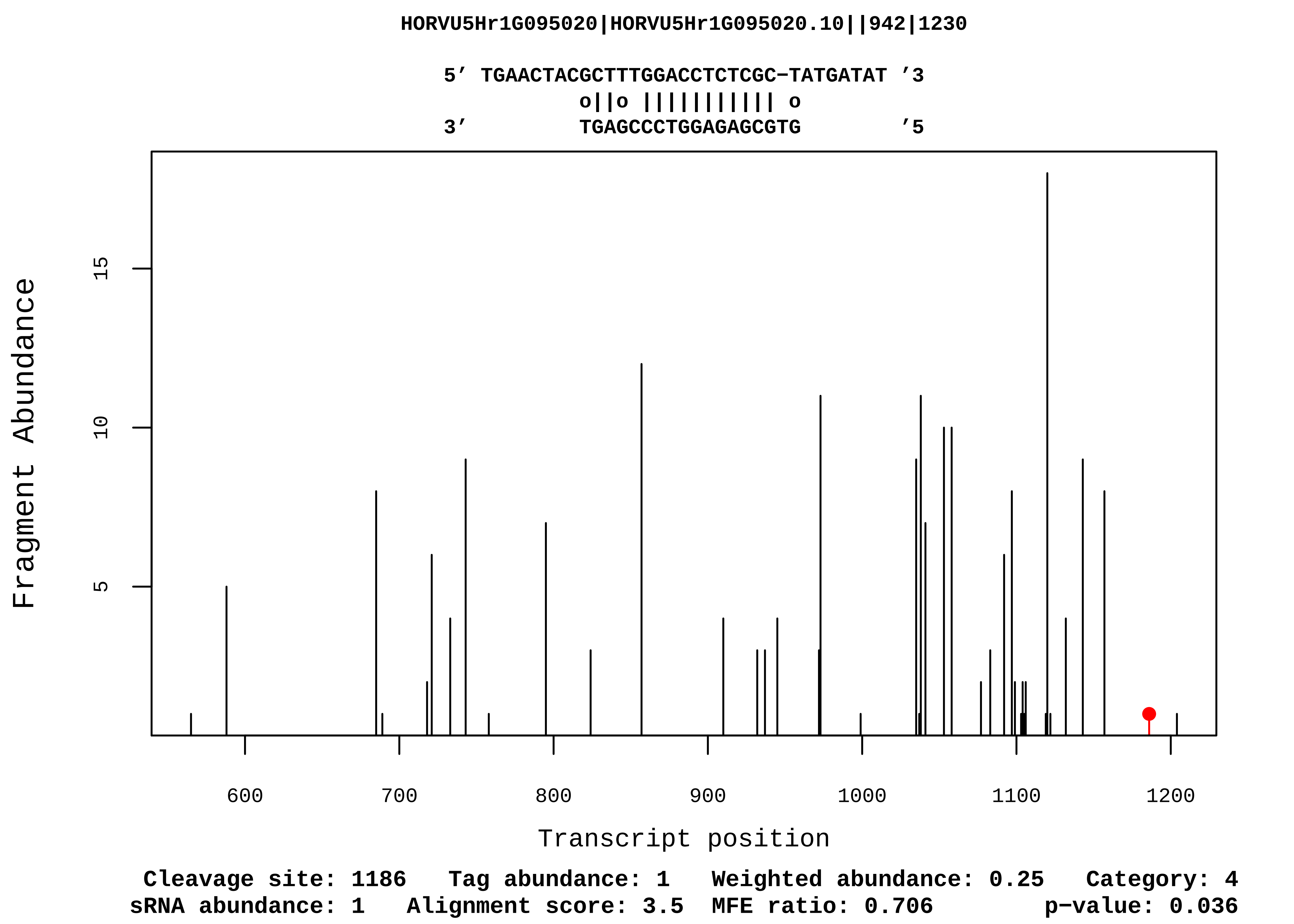

HORVU5Hr1G095020|HORVU5Hr1G095020.10||942|1230
5' TGAACTACGCTTTGGACCTCTCGC-TATGATAT '3
o||o ||||||||||| o
3' TGAGCCCTGGAGAGCGTG '5
Fragment Abundance
5
10
15
600
700
800
900
1000
1100
1200
Transcript position
Cleavage site: 1186 Tag abundance: 1 Weighted abundance: 0.25 Category: 4
sRNA abundance: 1 Alignment score: 3.5 MFE ratio: 0.706 p-value: 0.036

HORVU5Hr1G095020|HORVU5Hr1G095020.1||1755|2078

```
5' TGAACTACGCTTTGGACCTCTCGC-TATGATAT '3
           o||o ||||||||||| o
3'         TGAGCCCTGGAGAGCGTG        '5
```

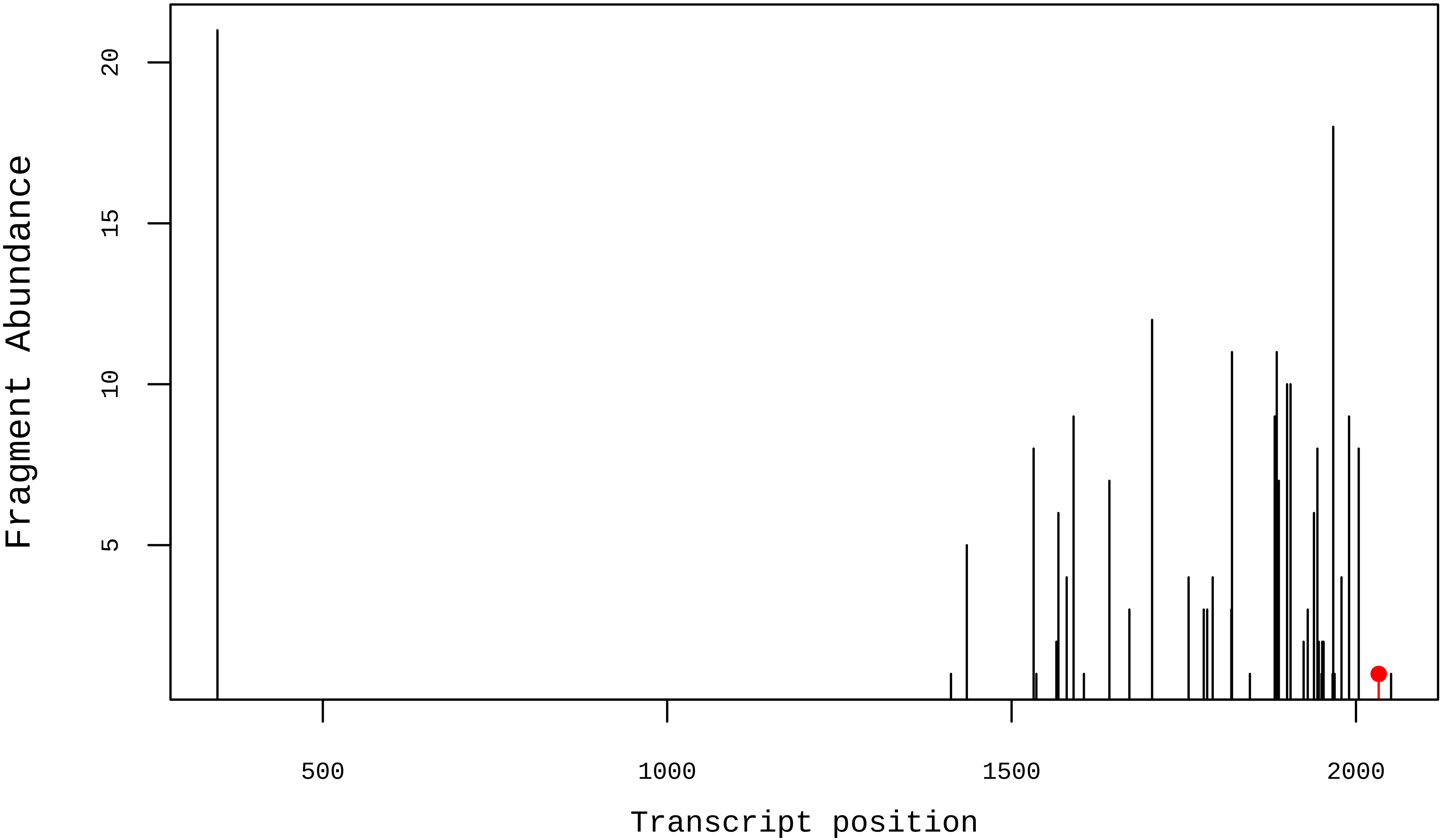

Cleavage site: 2033 Tag abundance: 1 Weighted abundance: 0.25 Category: 4
sRNA abundance: 1 Alignment score: 3.5 MFE ratio: 0.706 p-value: 0.021

HORVU5Hr1G095020|HORVU5Hr1G095020.8||789|1744

```
5' TGAACTACGCTTTGGACCTCTCGC-TATGATAT '3
           o||o ||||||||||| o
3'         TGAGCCCTGGAGAGCGTG         '5
```

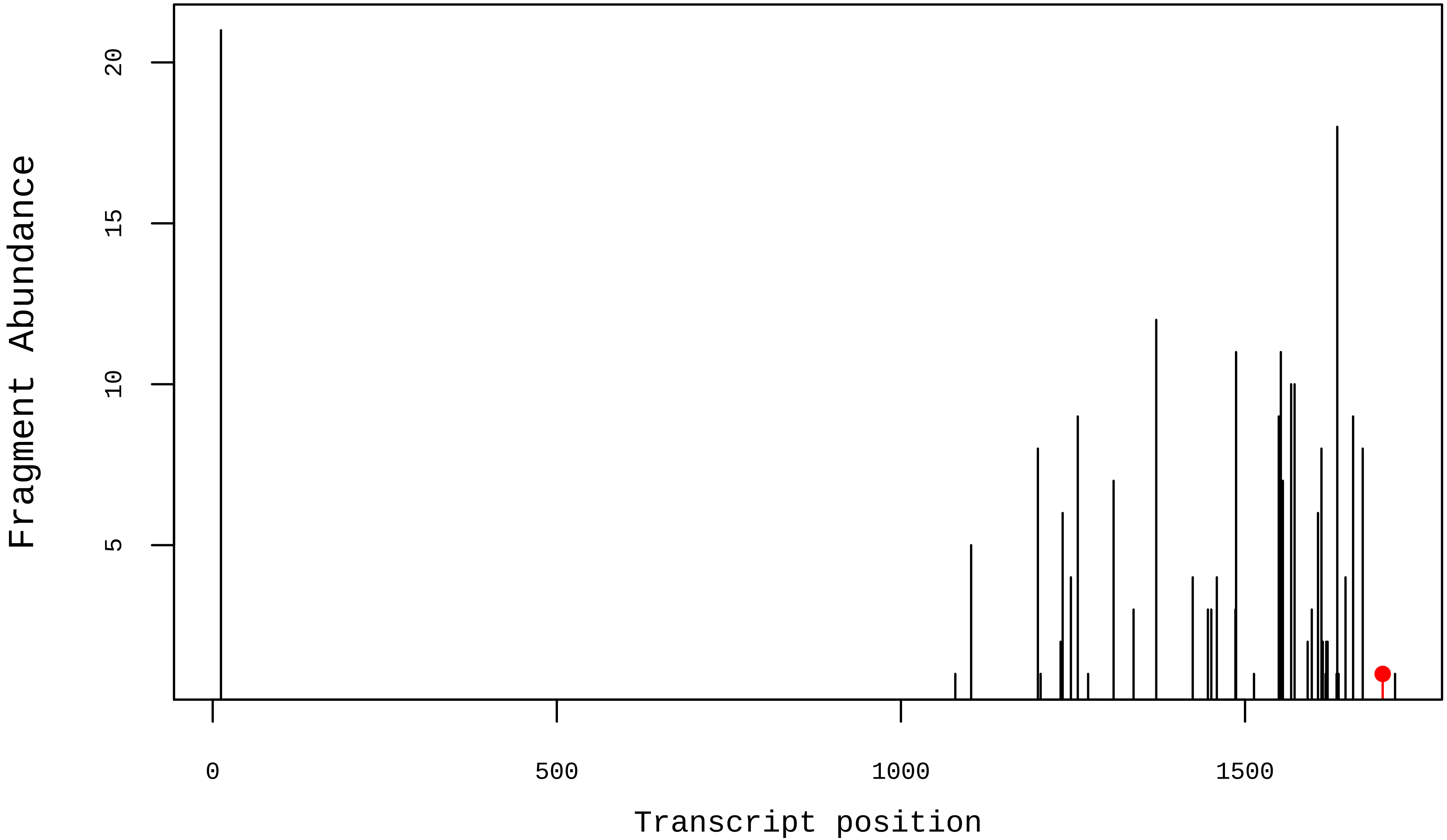

Cleavage site: 1700   Tag abundance: 1   Weighted abundance: 0.25   Category: 4
sRNA abundance: 1   Alignment score: 3.5   MFE ratio: 0.706   p-value: 0.025

HORVU5Hr1G095020|HORVU5Hr1G095020.9||789|1746

```
5’ TGAACTACGCTTTGGACCTCTCGC−TATGATAT ’3
              o||o ||||||||||| o
3’            TGAGCCCTGGAGAGCGTG         ’5
```

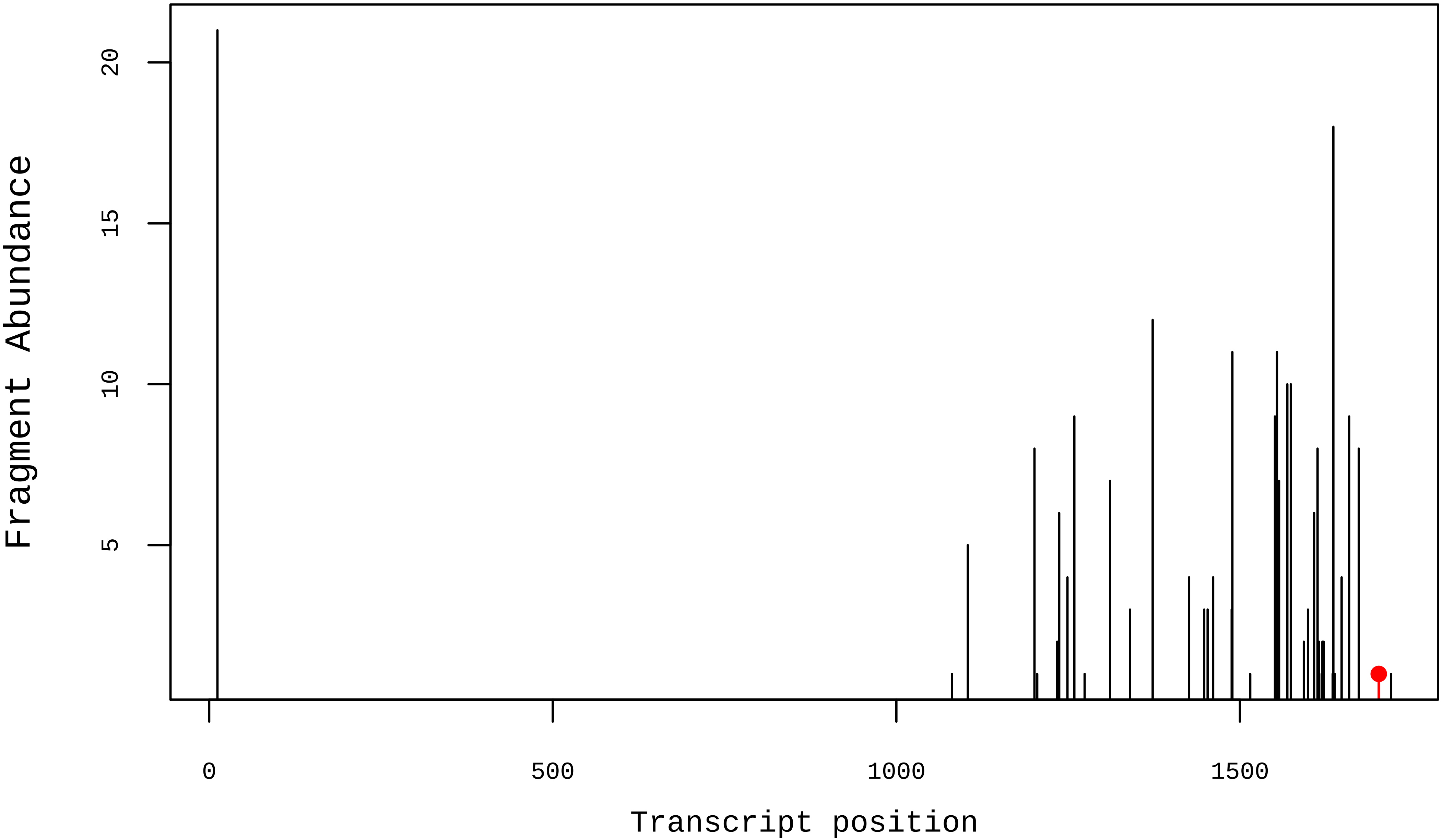

Cleavage site: 1702   Tag abundance: 1   Weighted abundance: 0.25   Category: 4
sRNA abundance: 1   Alignment score: 3.5   MFE ratio: 0.706   p-value: 0.025

HORVU3Hr1G094300|HORVU3Hr1G094300.1||675|2028

```
5' CTCATGGTCACCGACGGCTGCTTC-CTGGTGGA '3
               ||||||||||o|||o| |
3'         CGTGGCTGCCGGCGAGGCG         '5
```

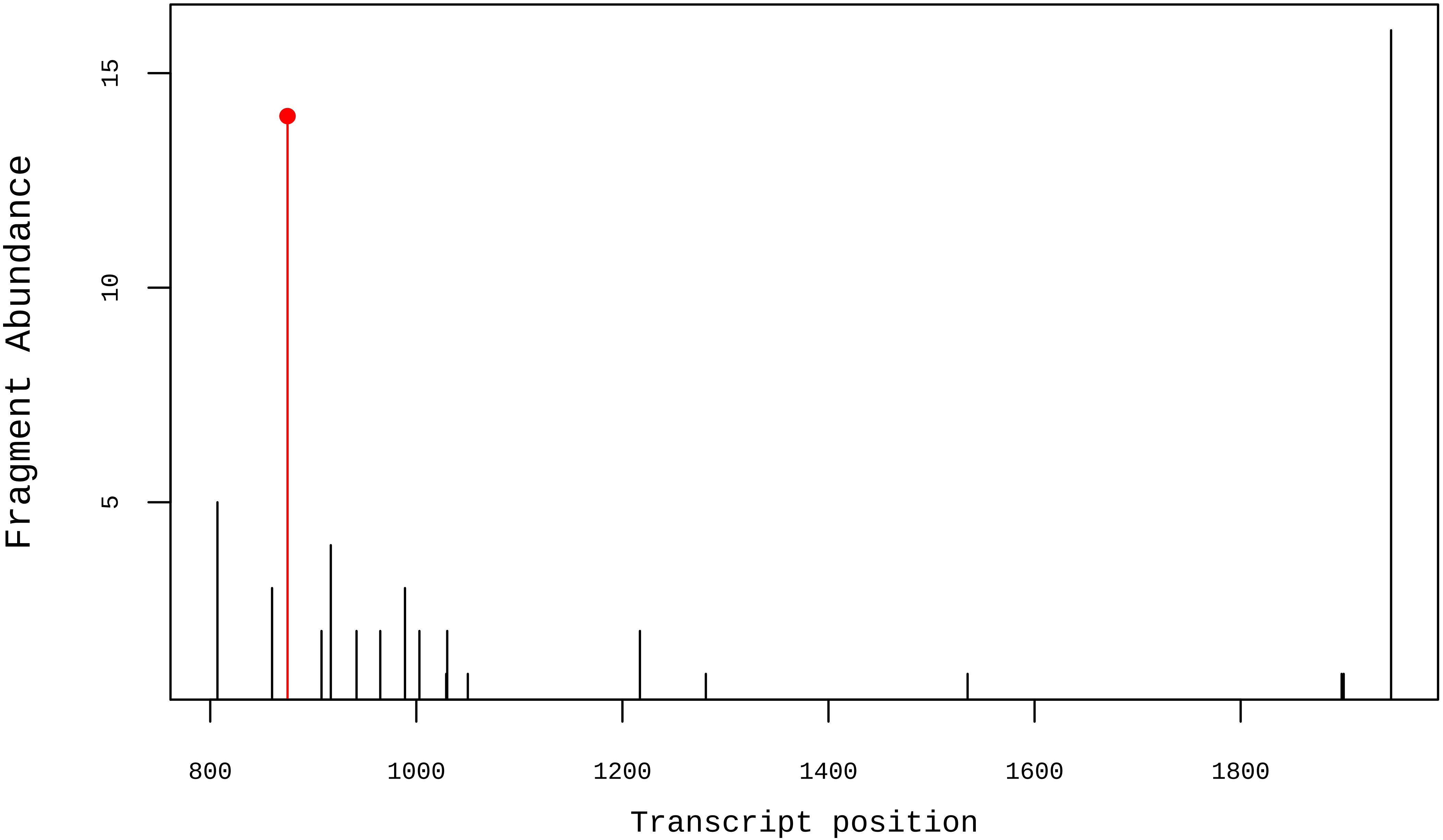

Cleavage site: 875   Tag abundance: 14   Weighted abundance: 2.8   Category: 2
sRNA abundance: 1   Alignment score: 4   MFE ratio: 0.722   p-value: 0.029

HORVU3Hr1G094300|HORVU3Hr1G094300.5||690|759

```
5' CTCATGGTCACCGACGGCTGCTTC-CTGGTGGA '3
            ||||||||||o|||o| |
3'          CGTGGCTGCCGGCGAGGCG        '5
```

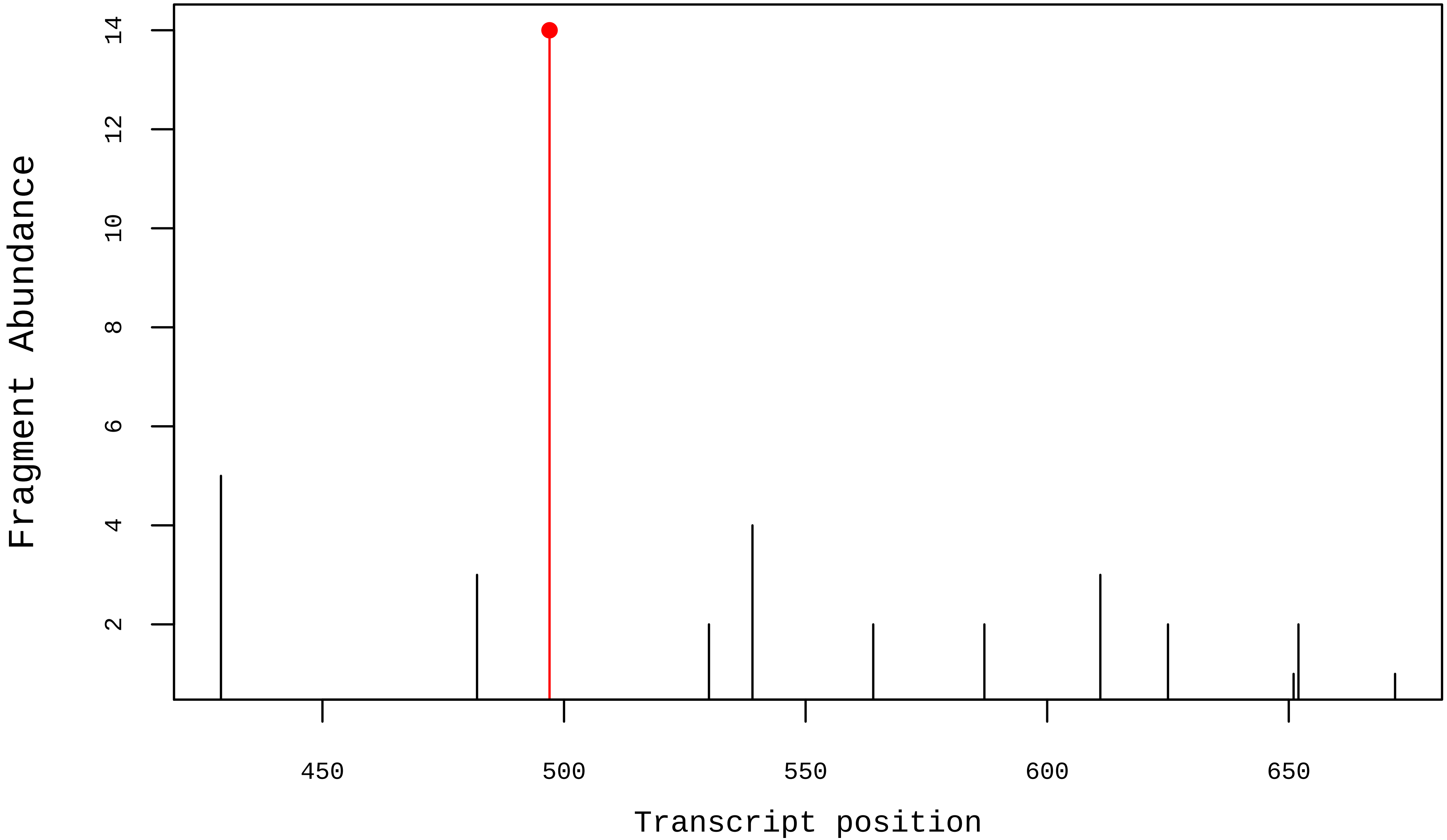

Cleavage site: 497   Tag abundance: 14   Weighted abundance: 2.8   Category: 0
sRNA abundance: 1   Alignment score: 4   MFE ratio: 0.722   p-value: 0.04

HORVU0Hr1G003660|HORVU0Hr1G003660.1||297|1955

```
5' GCCGGCCGAAGGGTCGAGTAGGTCGGTGCTCG '3
      |||||||||||||| |||||||||
3'    GCCGGCTTCCCAGCCCATCCAGCC       '5
```

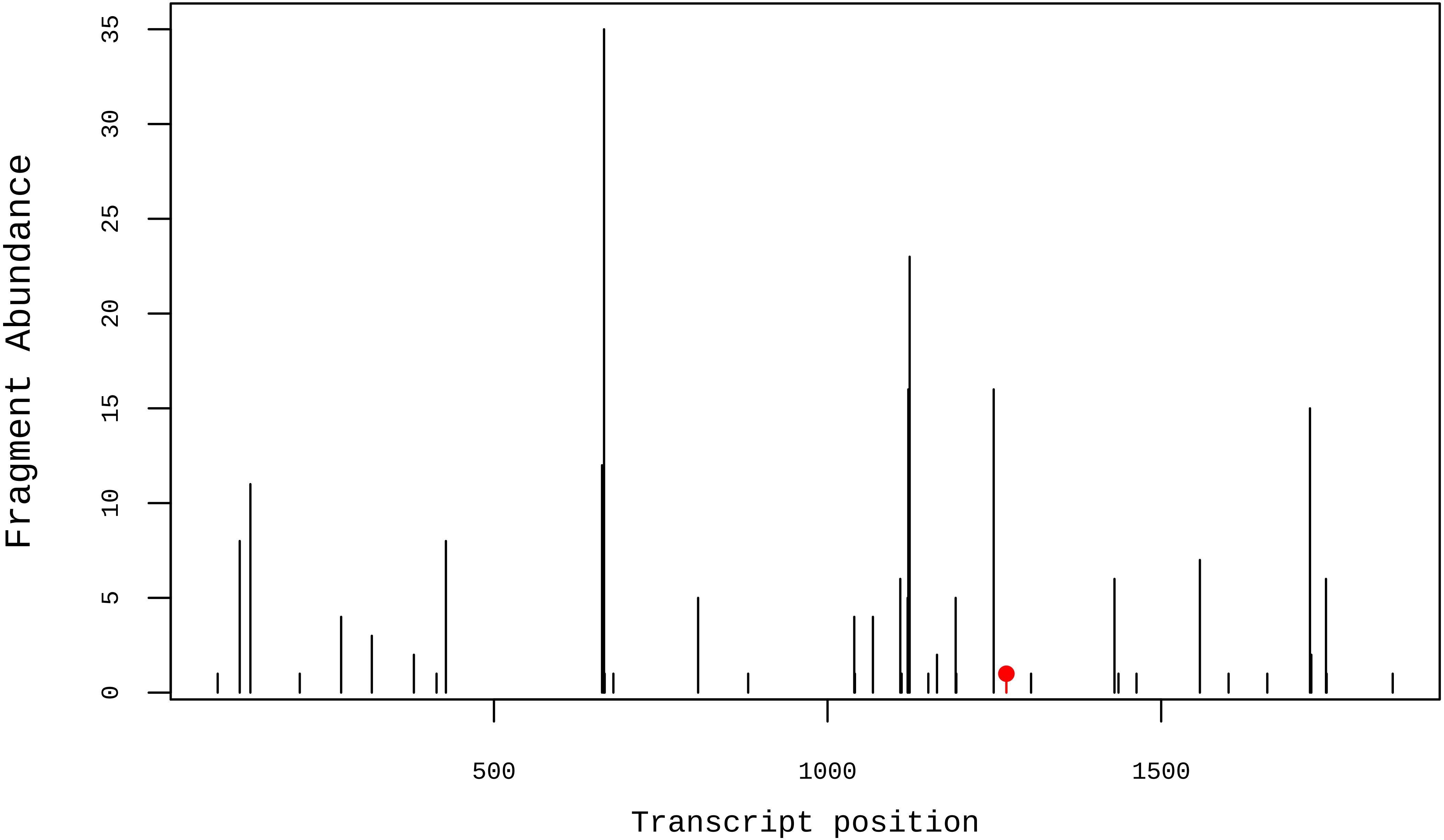

Cleavage site: 1268   Tag abundance: 1   Weighted abundance: 0.143   Category: 4
sRNA abundance: 1   Alignment score: 2 MFE ratio: 0.876   p-value: 0.046

HORVU0Hr1G023930|HORVU0Hr1G023930.3||396|2180

```
5' GTCGGCGGAAGGGTCGAGTAGGTCGGTGCTCG '3
      |||| ||||||||| |||||||||
3'    GCCGGCTTCCCAGCCCATCCAGCC       '5
```

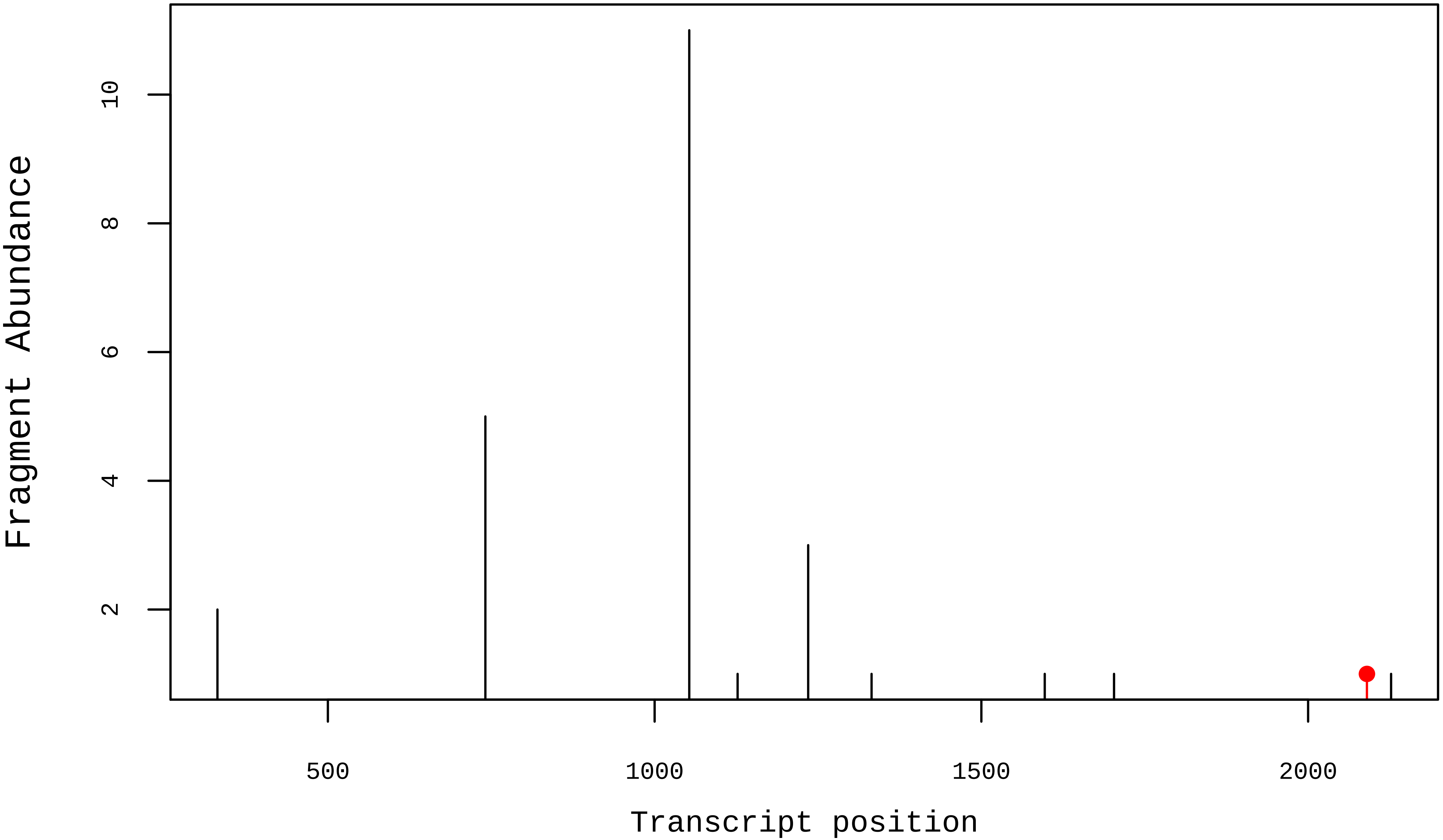

Cleavage site: 2090   Tag abundance: 1   Weighted abundance: 0.143   Category: 4
sRNA abundance: 1   Alignment score: 3   MFE ratio: 0.798   p-value: 0.019

HORVU2Hr1G072960|HORVU2Hr1G072960.1||600|3353

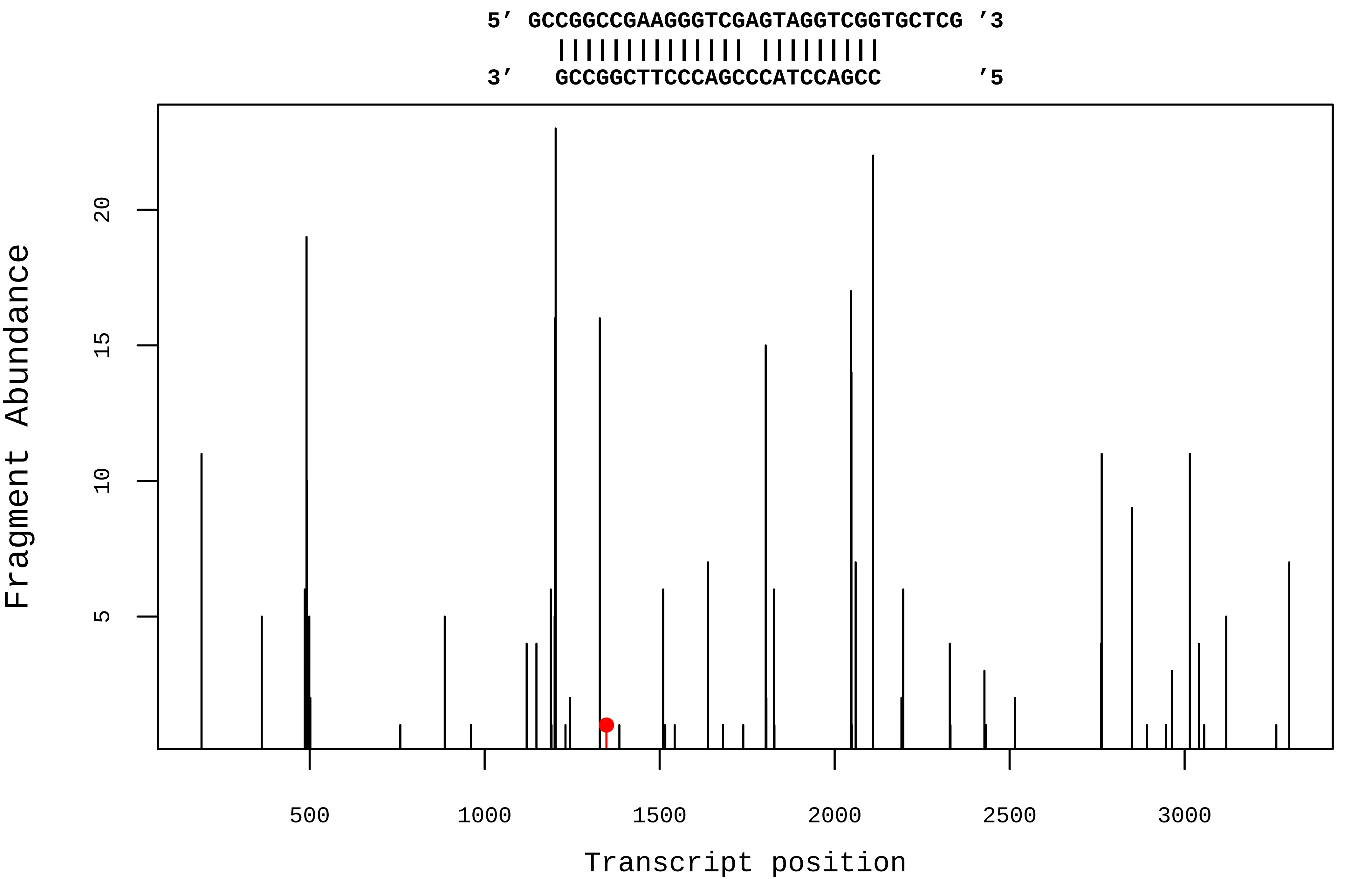

Cleavage site: 1348 Tag abundance: 1 Weighted abundance: 0.143 Category: 4
sRNA abundance: 1 Alignment score: 2 MFE ratio: 0.876 p-value: 0.03

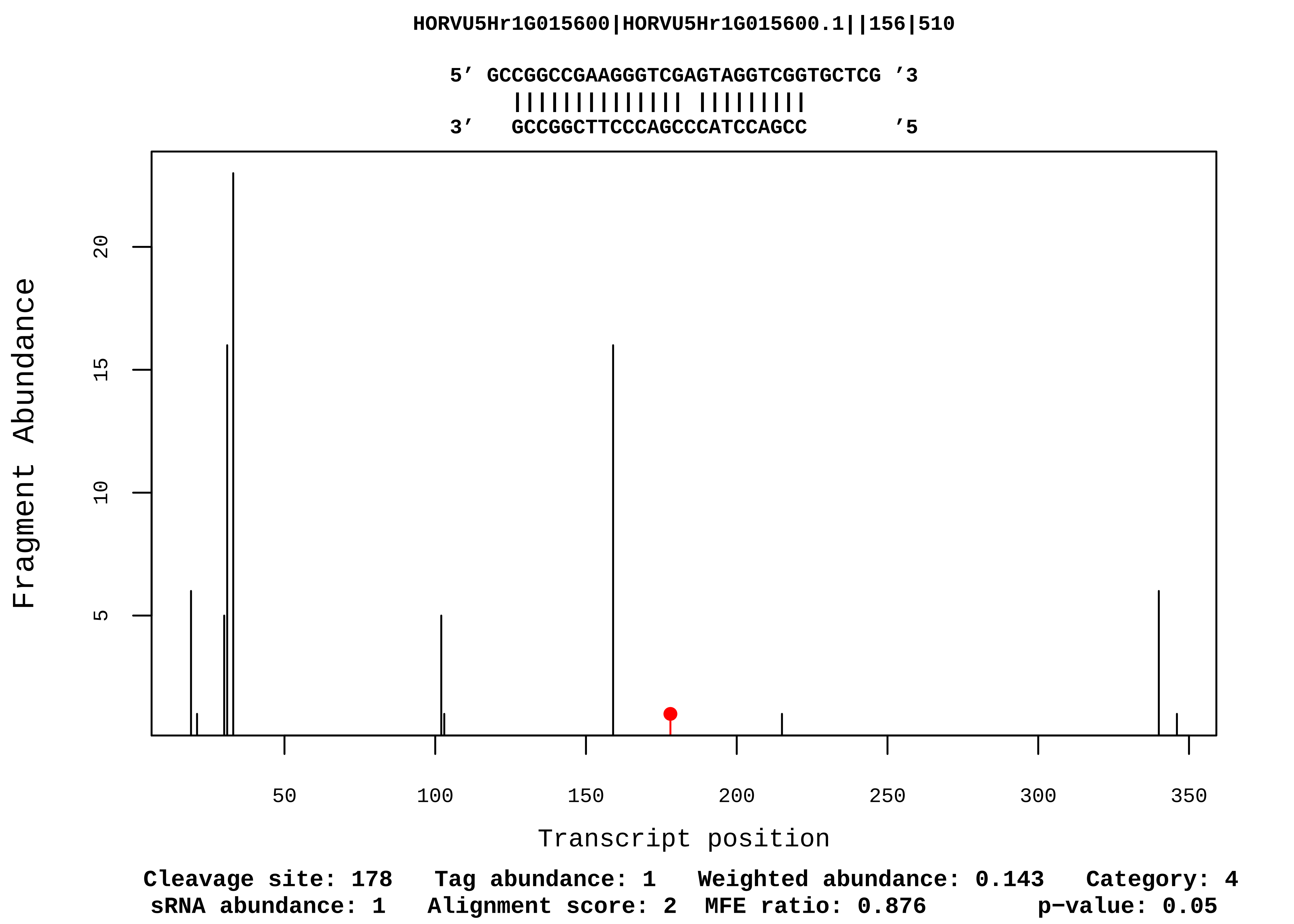
HORVU5Hr1G015600|HORVU5Hr1G015600.1||156|510
5' GCCGGCCGAAGGGTCGAGTAGGTCGGTGCTCG '3
3' GCCGGCTTCCCAGCCCATCCAGCC '5
Fragment Abundance
Transcript position
Cleavage site: 178 Tag abundance: 1 Weighted abundance: 0.143 Category: 4
sRNA abundance: 1 Alignment score: 2 MFE ratio: 0.876 p-value: 0.05

HORVU5Hr1G015600|HORVU5Hr1G015600.2||231|617

```
5' GCCGGCCGAAGGGTCGAGTAGGTCGGTGCTCG '3
      ||||||||||||||| ||||||||||
3'    GCCGGCTTCCCAGCCCATCCAGCC        '5
```

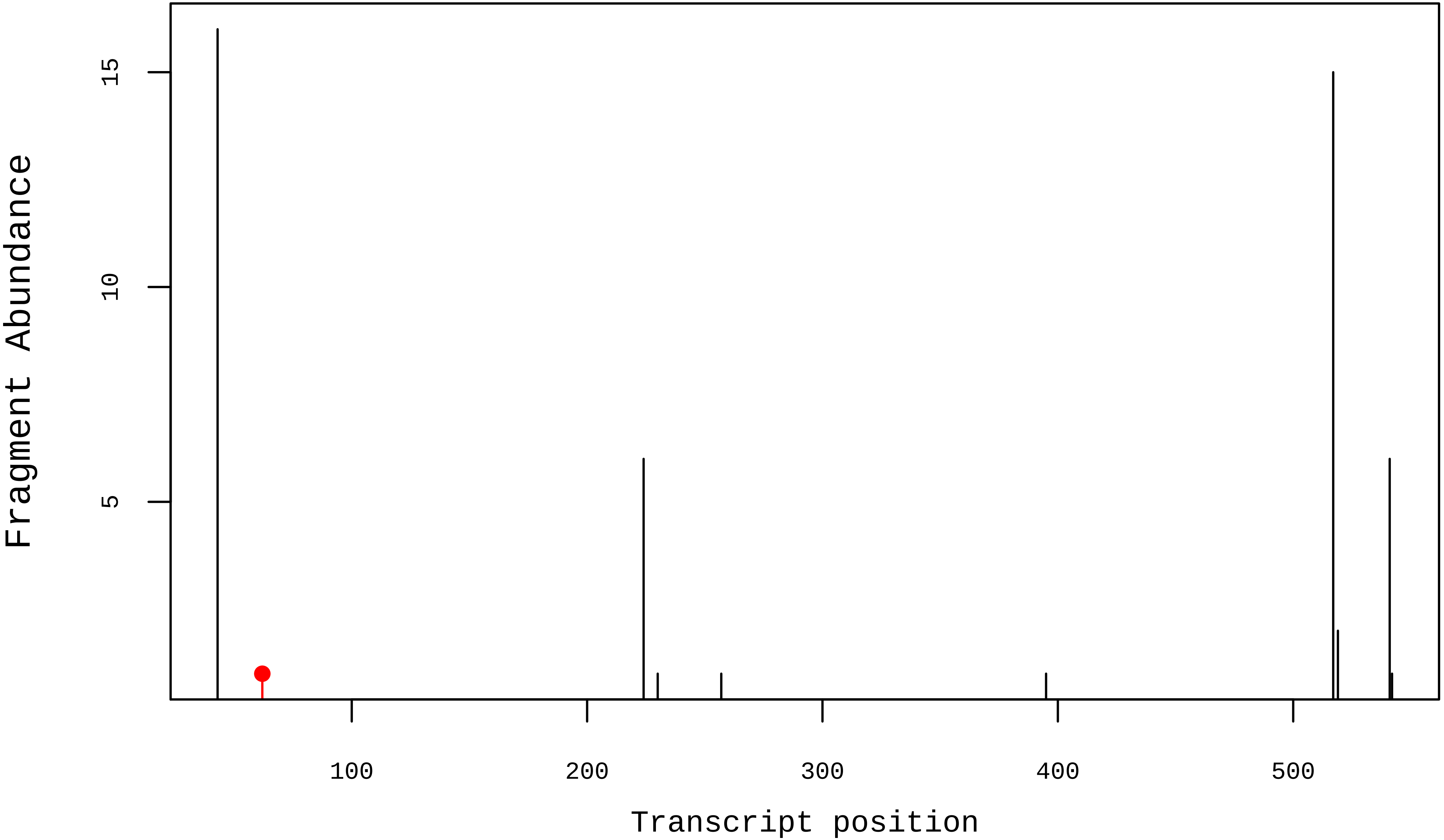

Cleavage site: 62 Tag abundance: 1 Weighted abundance: 0.143 Category: 4
sRNA abundance: 1 Alignment score: 2 MFE ratio: 0.876 p-value: 0.041

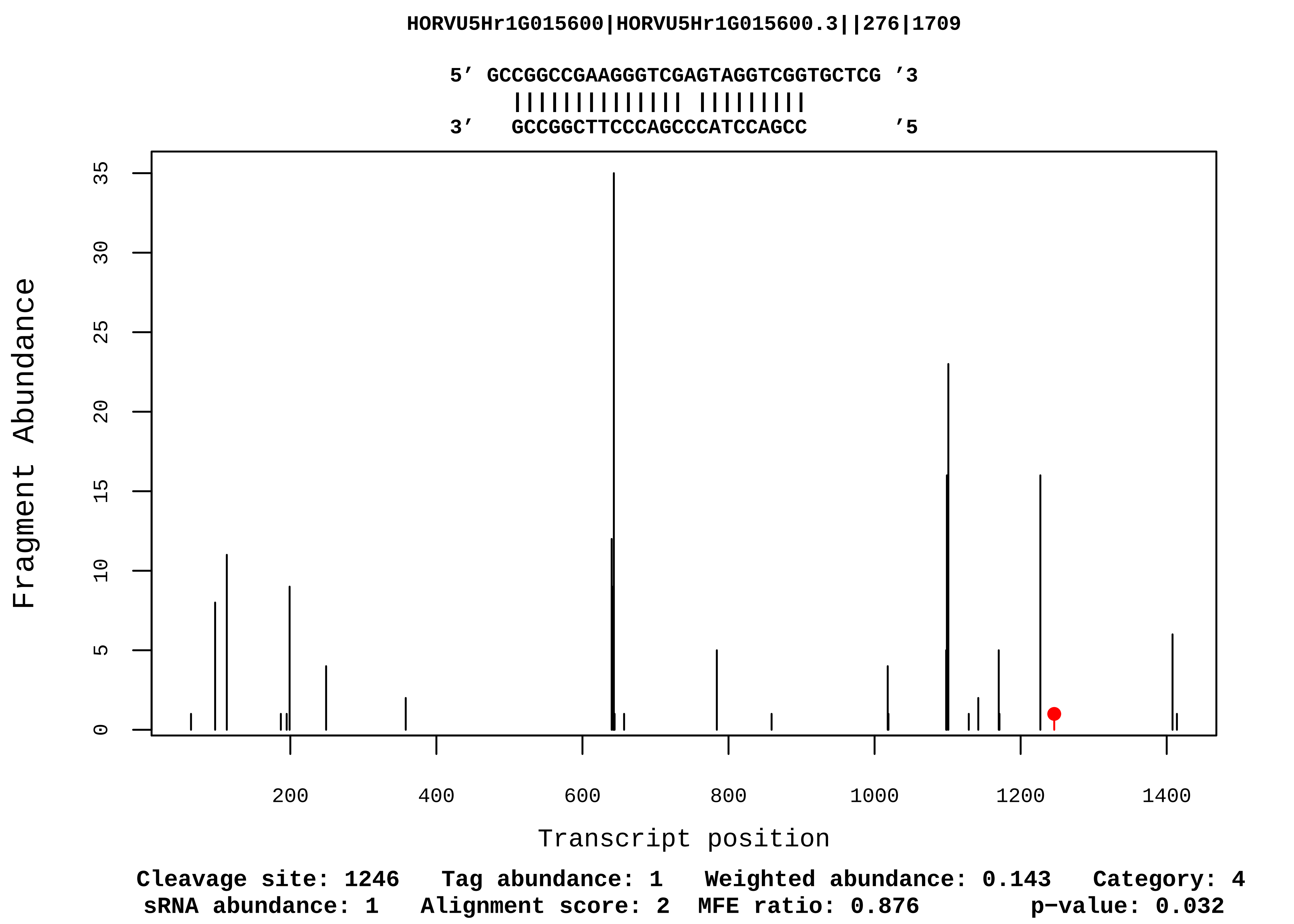
HORVU5Hr1G015600|HORVU5Hr1G015600.3||276|1709
5′ GCCGGCCGAAGGGTCGAGTAGGTCGGTGCTCG ′3
3′ GCCGGCTTCCCAGCCCATCCAGCC ′5
Fragment Abundance
0
5
10
15
20
25
30
35
200
400
600
800
1000
1200
1400
Transcript position
Cleavage site: 1246 Tag abundance: 1 Weighted abundance: 0.143 Category: 4
sRNA abundance: 1 Alignment score: 2 MFE ratio: 0.876 p-value: 0.032

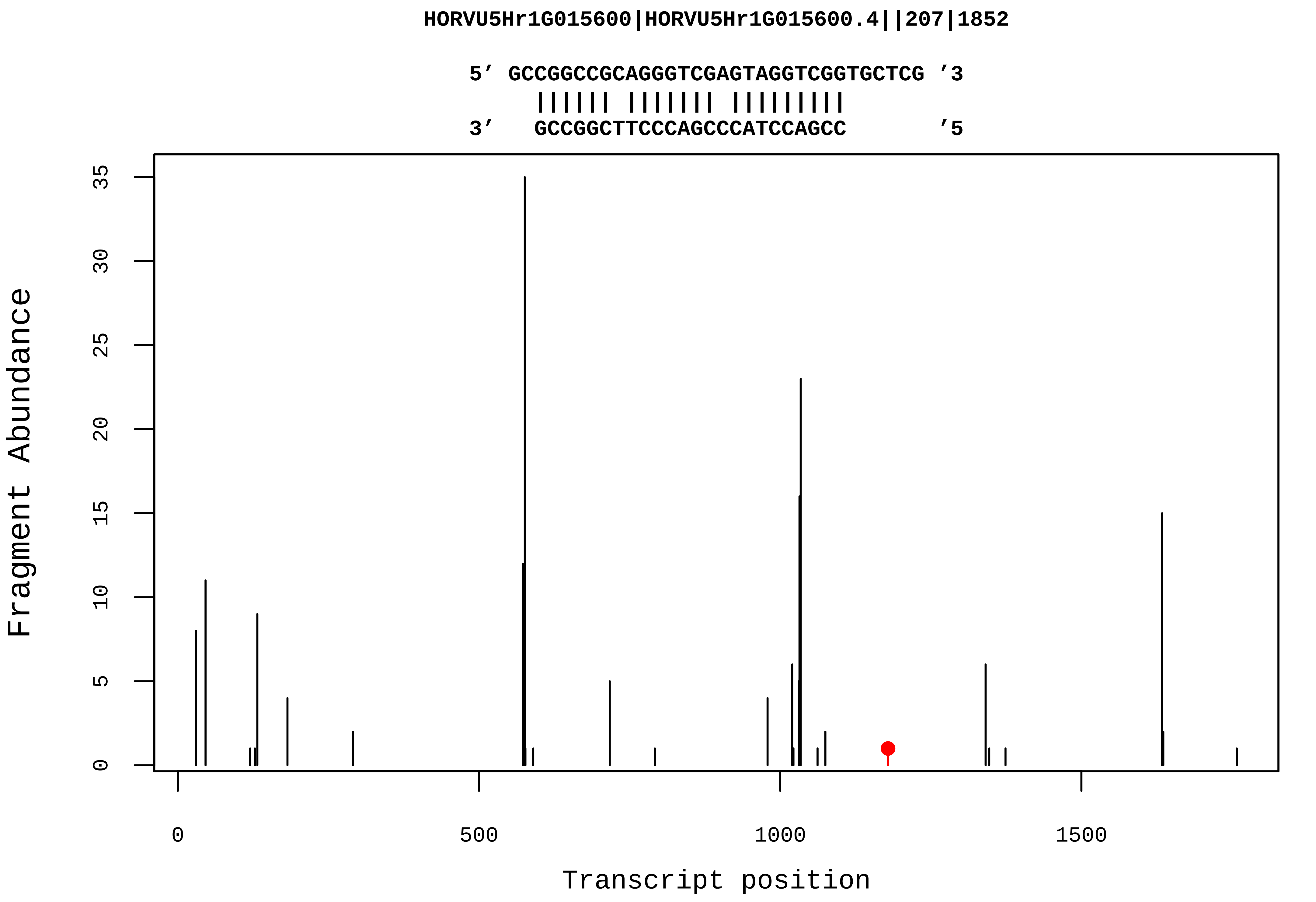

Cleavage site: 1179 Tag abundance: 1 Weighted abundance: 0.143 Category: 4
sRNA abundance: 1 Alignment score: 3 MFE ratio: 0.795 p-value: 0.041

HORVU2Hr1G108280|HORVU2Hr1G108280.1||360|855

```
5' AGGTGGCGCTGTCGGGCCAGGTCCGCTACCAG '3
         |o| |o|||o|||||o||||
3'       GTGTCGGCCTGGTCCGGGCG       '5
```

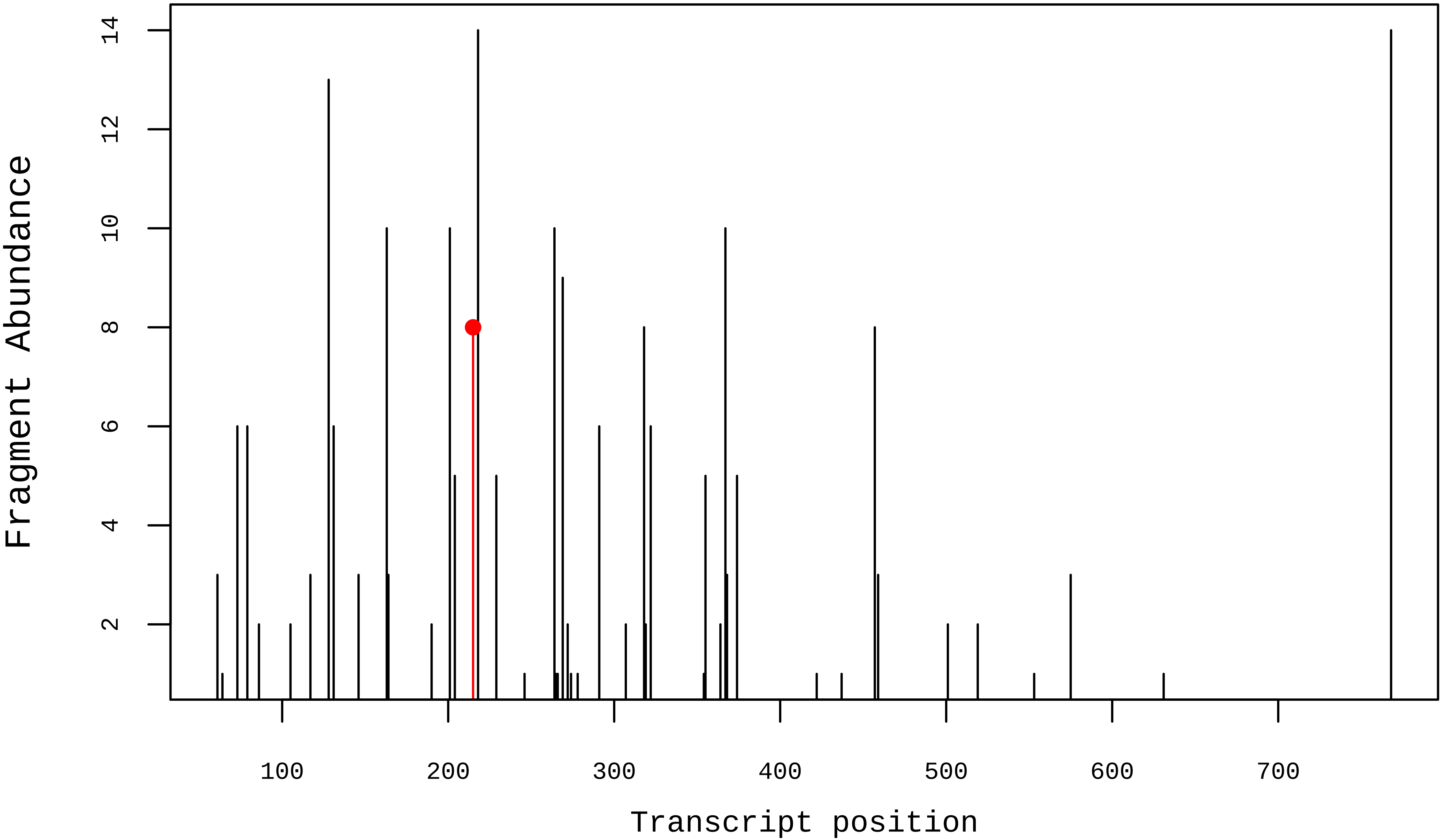

Cleavage site: 215 Tag abundance: 8 Weighted abundance: 4 Category: 2
sRNA abundance: 1 Alignment score: 4 MFE ratio: 0.771 p-value: 0.033

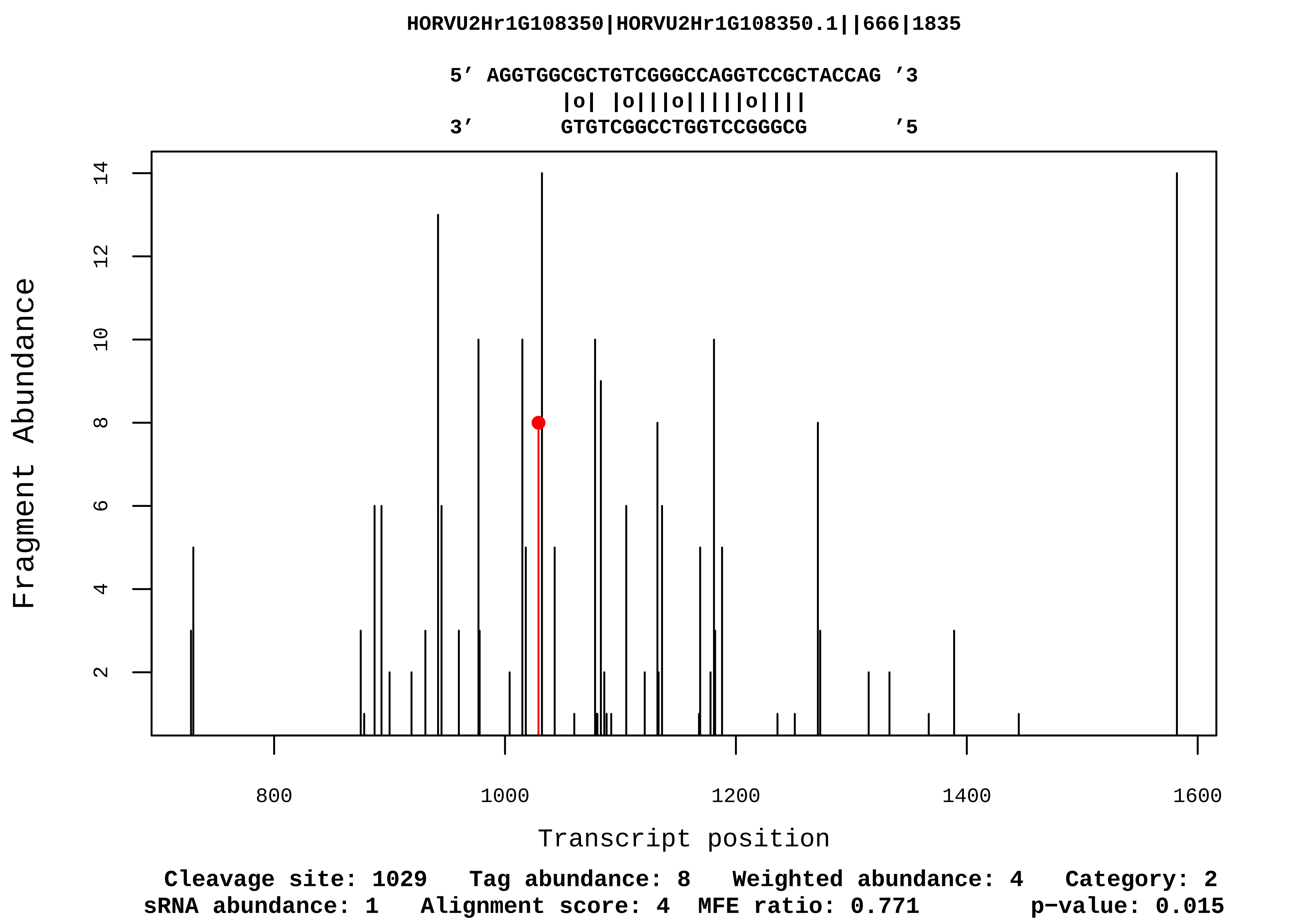
HORVU2Hr1G108350|HORVU2Hr1G108350.1||666|1835
5' AGGTGGCGCTGTCGGGCCAGGTCCGCTACCAG '3
|o| |o|||o|||||o||||
3' GTGTCGGCCTGGTCCGGGCG '5
Fragment Abundance
2
4
6
8
10
12
14
800
1000
1200
1400
1600
Transcript position
Cleavage site: 1029 Tag abundance: 8 Weighted abundance: 4 Category: 2
sRNA abundance: 1 Alignment score: 4 MFE ratio: 0.771 p-value: 0.015

HORVU6Hr1G056230|HORVU6Hr1G056230.1||939|5459

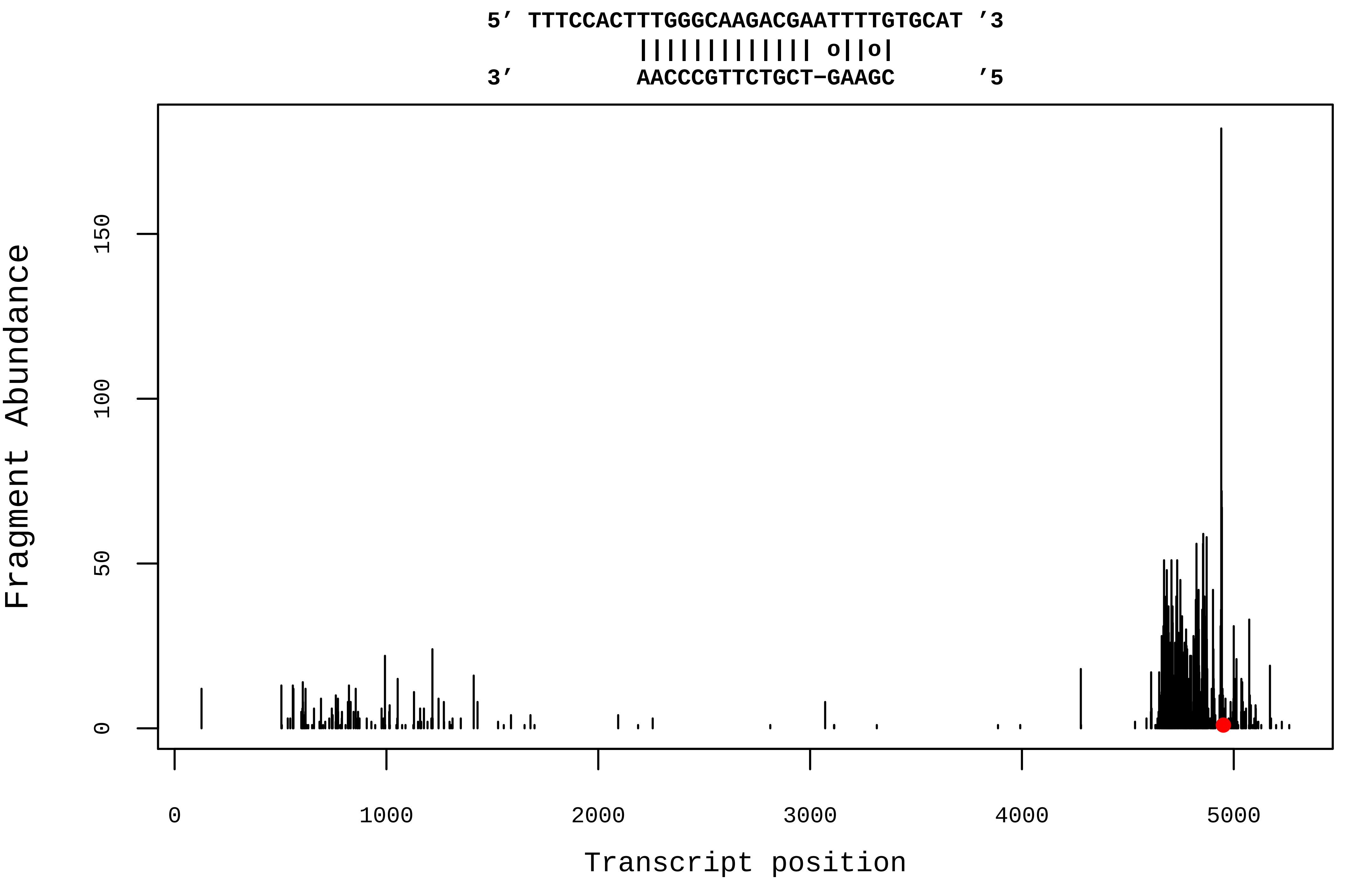

Cleavage site: 4951 Tag abundance: 1 Weighted abundance: 0.333 Category: 4
sRNA abundance: 1 Alignment score: 4 MFE ratio: 0.767 p-value: 0.038

HORVU7Hr1G021300|HORVU7Hr1G021300.1||357|358

```
5' CGGCCCTGCCCCAACGGCTCCGTCGAGGTGGT '3
        ||||  ||||o|||||||||
3'      CACGGT-TTGCTGAGGCAGCT       '5
```

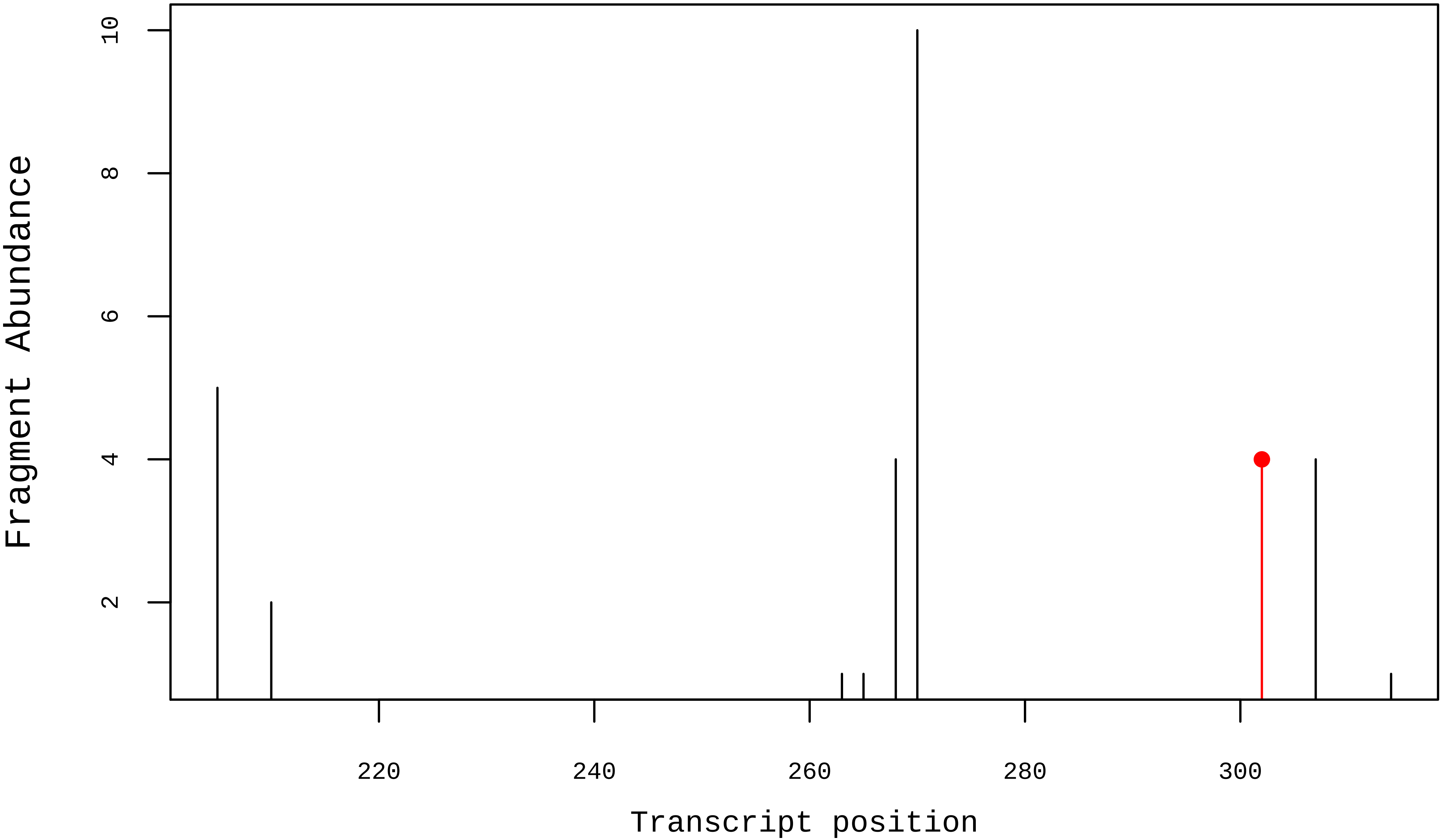

Cleavage site: 302 Tag abundance: 4 Weighted abundance: 0.667 Category: 3
sRNA abundance: 1 Alignment score: 4 MFE ratio: 0.803 p-value: 0.023

HORVU7Hr1G021300|HORVU7Hr1G021300.2||330|512

```
5' CGGCCCTGCCCCAACGGCTCCGTCGAGGTGGT '3
           ||||  ||||o|||||||||
3'       CACGGT-TTGCTGAGGCAGCT        '5
```

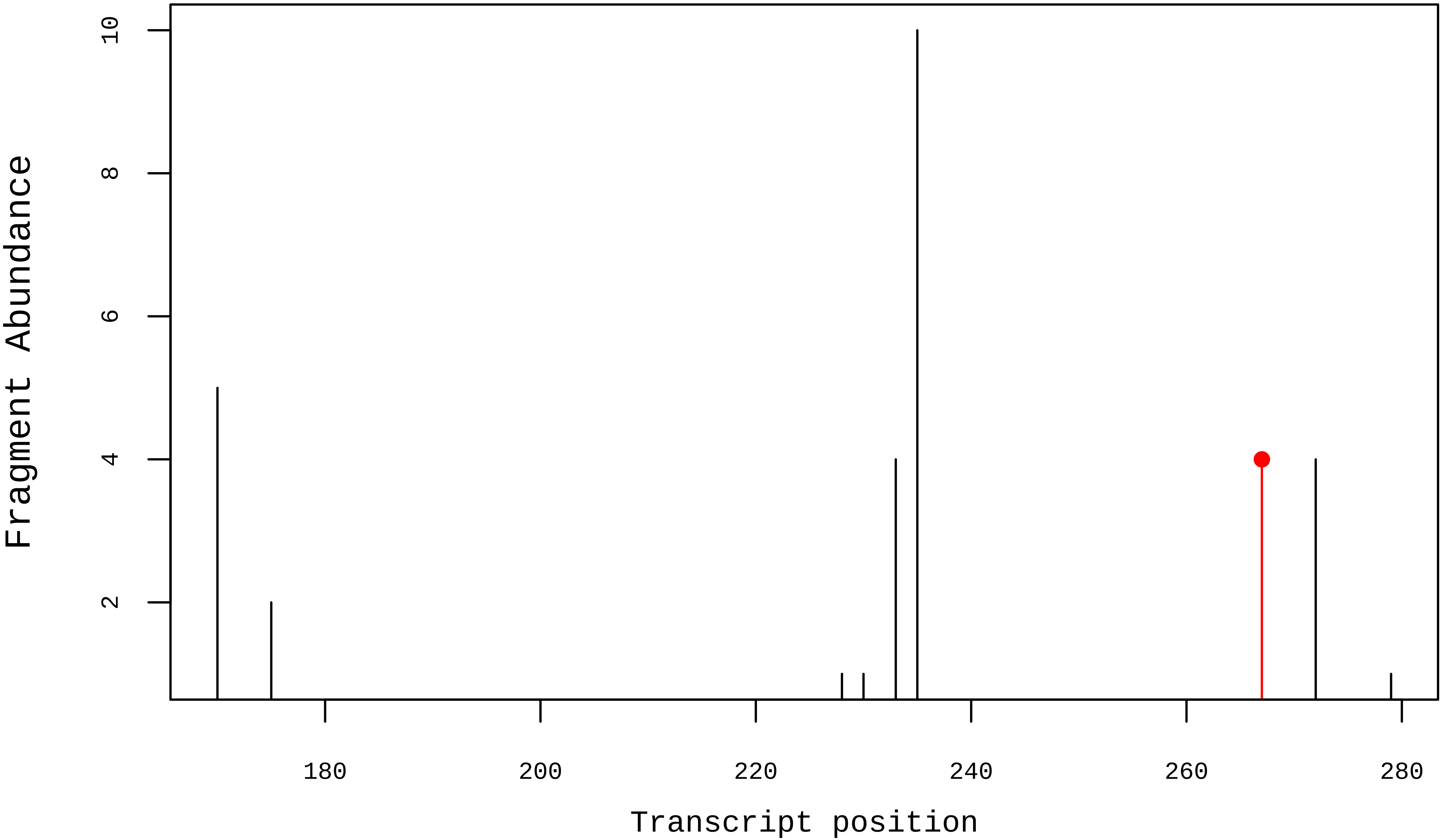

Cleavage site: 267   Tag abundance: 4   Weighted abundance: 0.667   Category: 3
sRNA abundance: 1   Alignment score: 4   MFE ratio: 0.803   p-value: 0.016

HORVU5Hr1G104230|HORVU5Hr1G104230.1||1473|7008

```
5' GCCGCCGGCGGACGCGCG-CCGGGCCGCTCGGC '3
          |||| ||o|| |||||||
3'        GCCTTCGTGCTGGCCCGG         '5
```

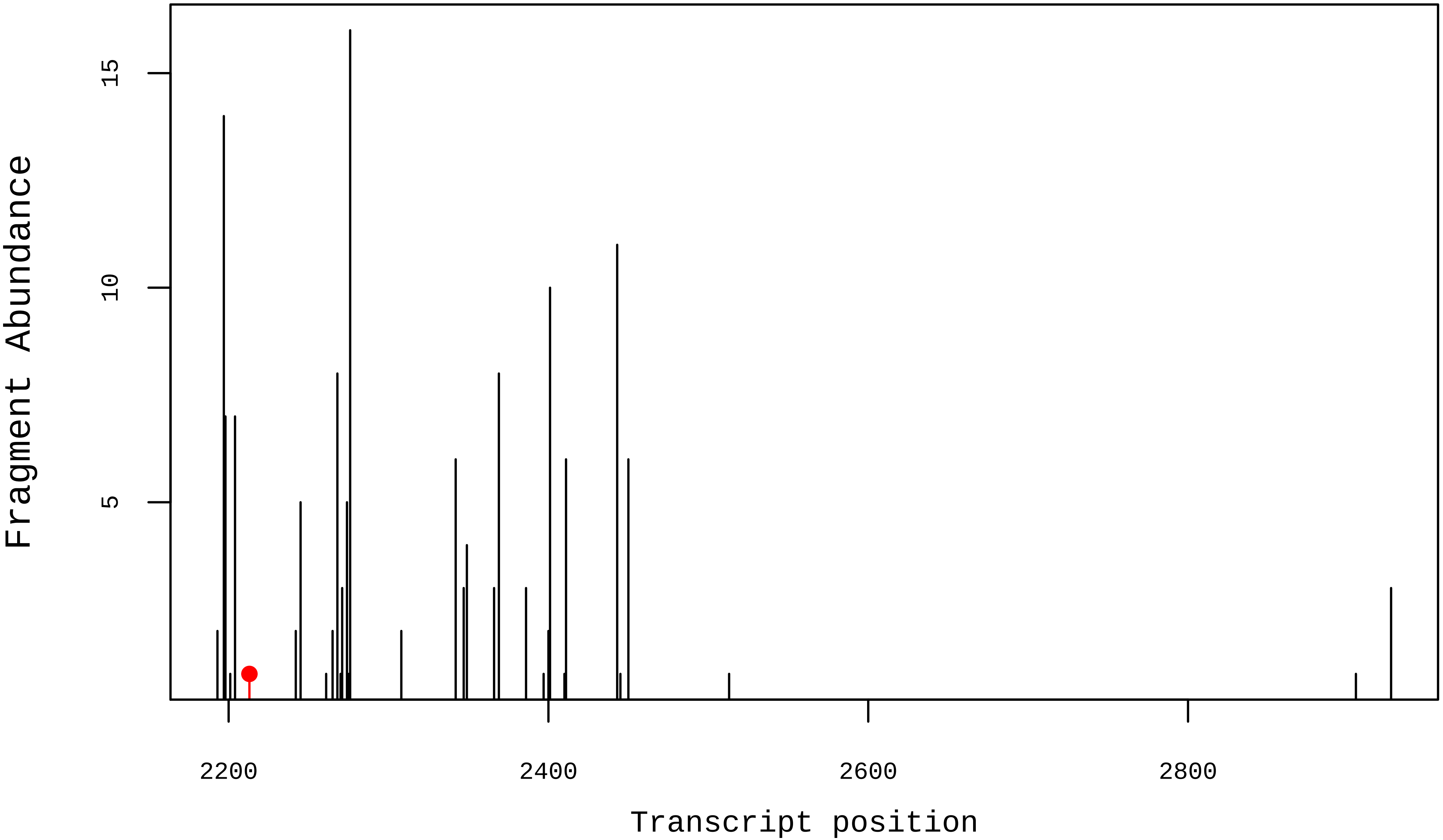

Cleavage site: 2213 Tag abundance: 1 Weighted abundance: 0.2 Category: 4
sRNA abundance: 1 Alignment score: 4 MFE ratio: 0.762 p-value: 0.034

HORVU5Hr1G104230|HORVU5Hr1G104230.2||912|3355

```
5' GCCGCCGGCGGACGCGCG-CCGGGCCGCTCGGC '3
           |||| ||o|| |||||||
3'         GCCTTCGTGCTGGCCCGG         '5
```

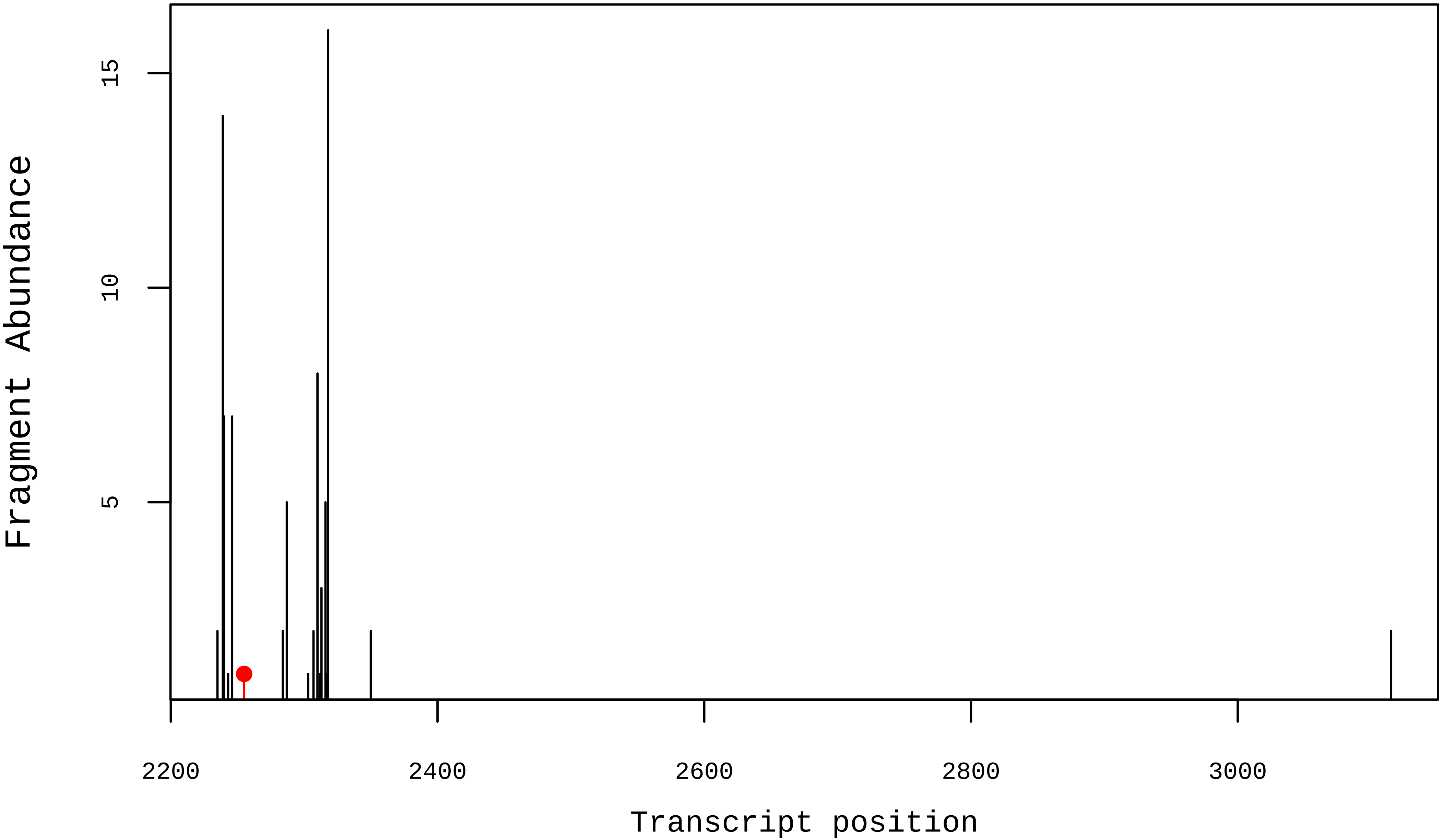

Cleavage site: 2255 Tag abundance: 1 Weighted abundance: 0.2 Category: 4
sRNA abundance: 1 Alignment score: 4 MFE ratio: 0.762 p-value: 0.035

HORVU5Hr1G104230|HORVU5Hr1G104230.4||1473|4875

```
5' GCCGCCGGCGGACGCGCG-CCGGGCCGCTCGGC '3
           |||| ||o|| |||||||
3'         GCCTTCGTGCTGGCCCGG          '5
```

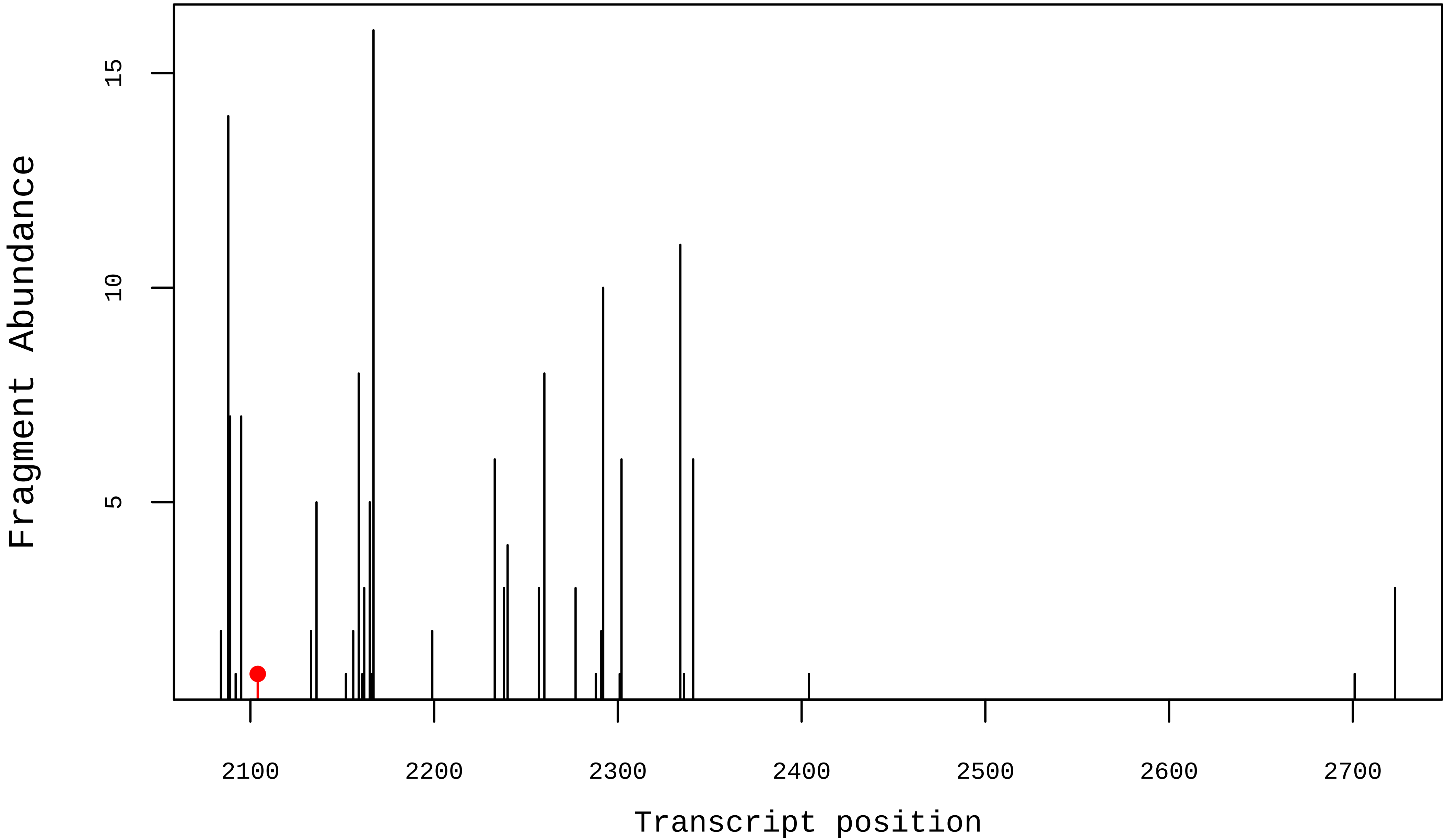

Cleavage site: 2104   Tag abundance: 1   Weighted abundance: 0.2   Category: 4
sRNA abundance: 1   Alignment score: 4   MFE ratio: 0.762   p-value: 0.048

HORVU6Hr1G017340|HORVU6Hr1G017340.1||1716|2872

```
5' GCAGCCTGCGGGAGCACAGACCGGGCGGAGGT '3
               |||o||||| ||||||||
3'             GCCTTCGTG-CTGGCCCGG       '5
```

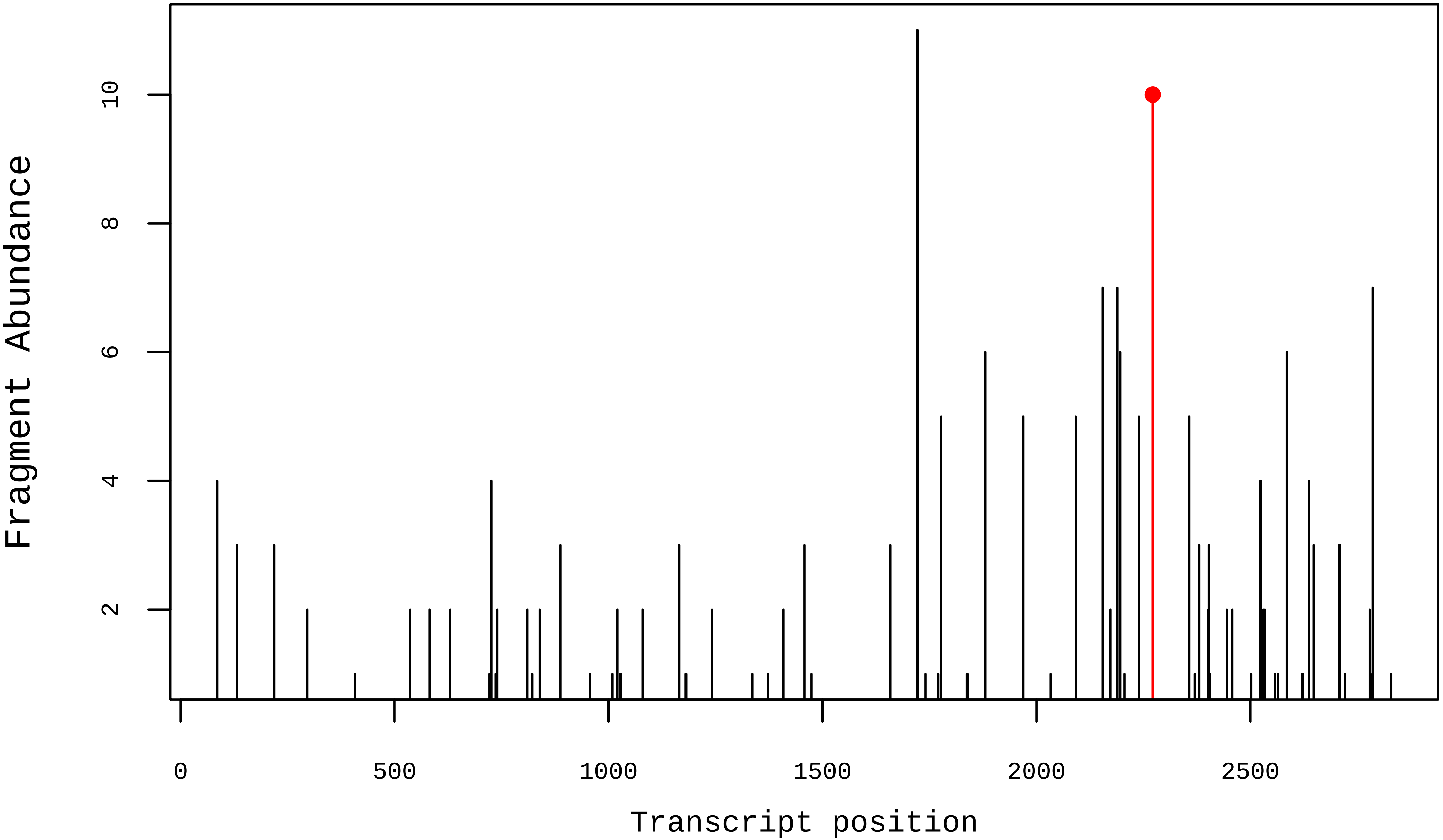

Cleavage site: 2272 Tag abundance: 10 Weighted abundance: 1.429 Category: 2
sRNA abundance: 1 Alignment score: 3.5 MFE ratio: 0.865 p-value: 0.039

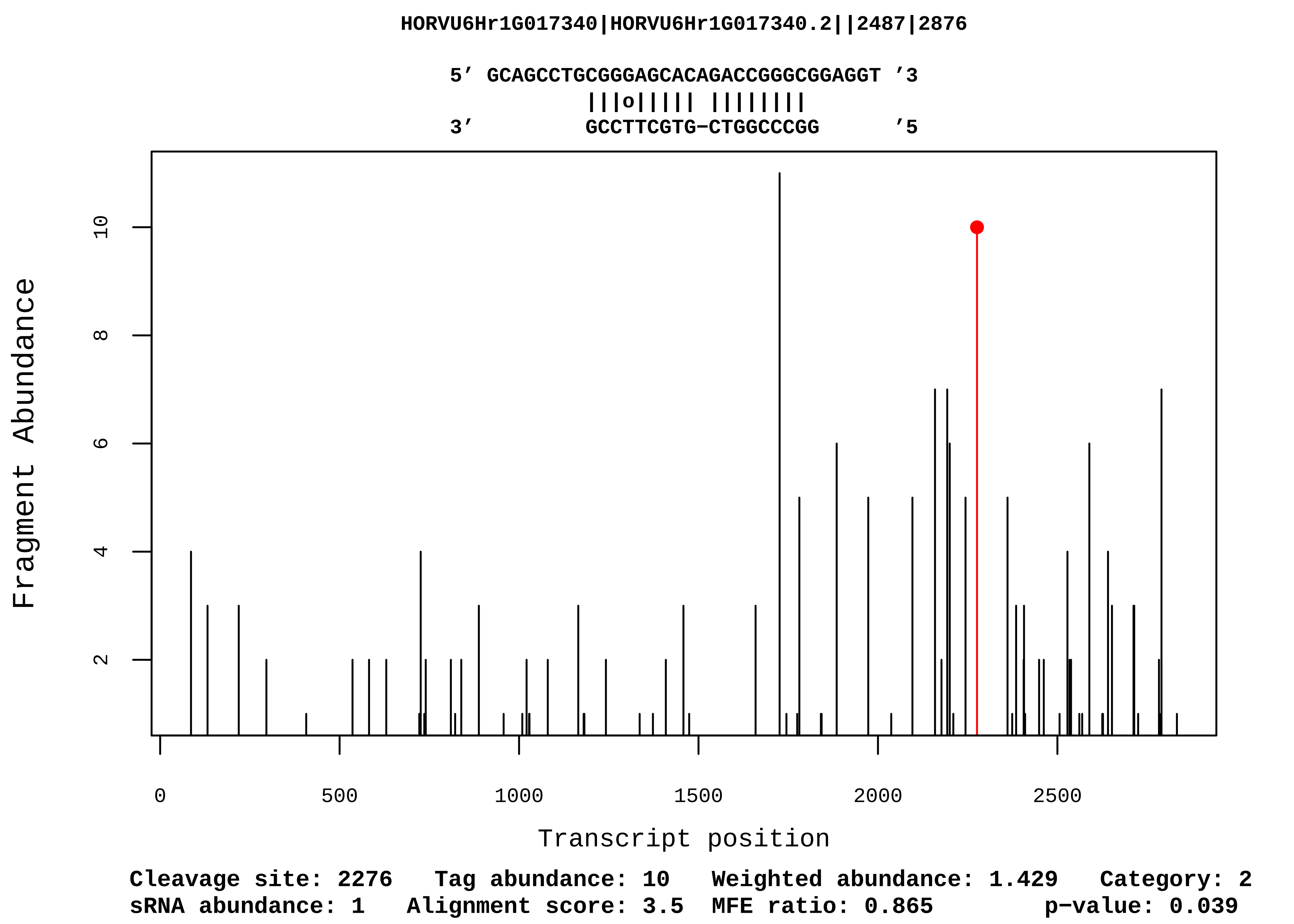
HORVU6Hr1G017340|HORVU6Hr1G017340.2||2487|2876
5' GCAGCCTGCGGGAGCACAGACCGGGCGGAGGT '3
|||o||||| ||||||||
3' GCCTTCGTG-CTGGCCCGG '5
Fragment Abundance
2
4
6
8
10
0
500
1000
1500
2000
2500
Transcript position
Cleavage site: 2276 Tag abundance: 10 Weighted abundance: 1.429 Category: 2
sRNA abundance: 1 Alignment score: 3.5 MFE ratio: 0.865 p-value: 0.039

HORVU6Hr1G017340|HORVU6Hr1G017340.3||1722|2867

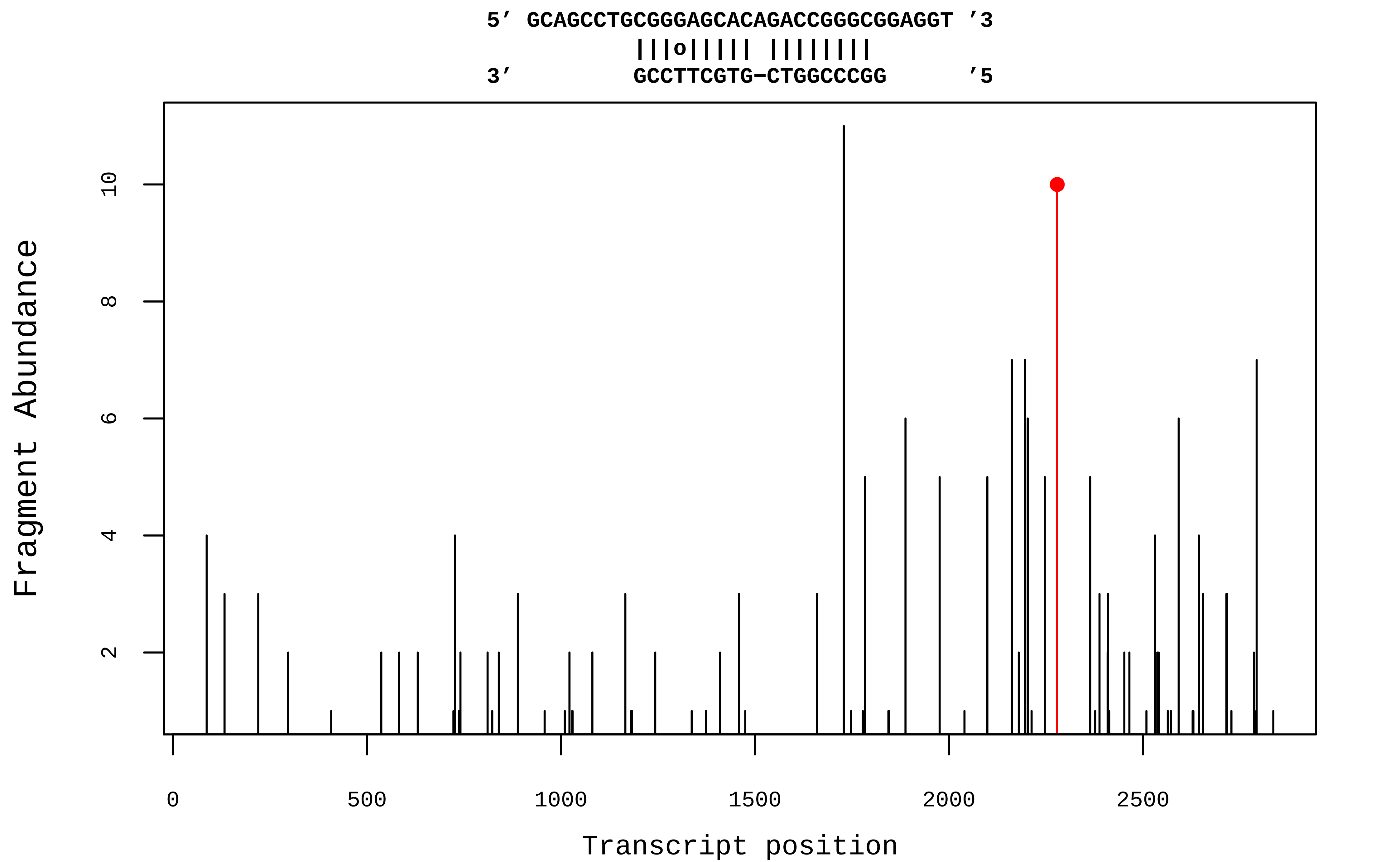

Cleavage site: 2279 Tag abundance: 10 Weighted abundance: 1.429 Category: 2
sRNA abundance: 1 Alignment score: 3.5 MFE ratio: 0.865 p-value: 0.039

HORVU6Hr1G017340|HORVU6Hr1G017340.4||1710|2795

```
5' GCAGCCTGCGGGAGCACAGACCGGGCGGAGGT '3
              |||o||||| |||||||||
3'            GCCTTCGTG-CTGGCCCGG       '5
```

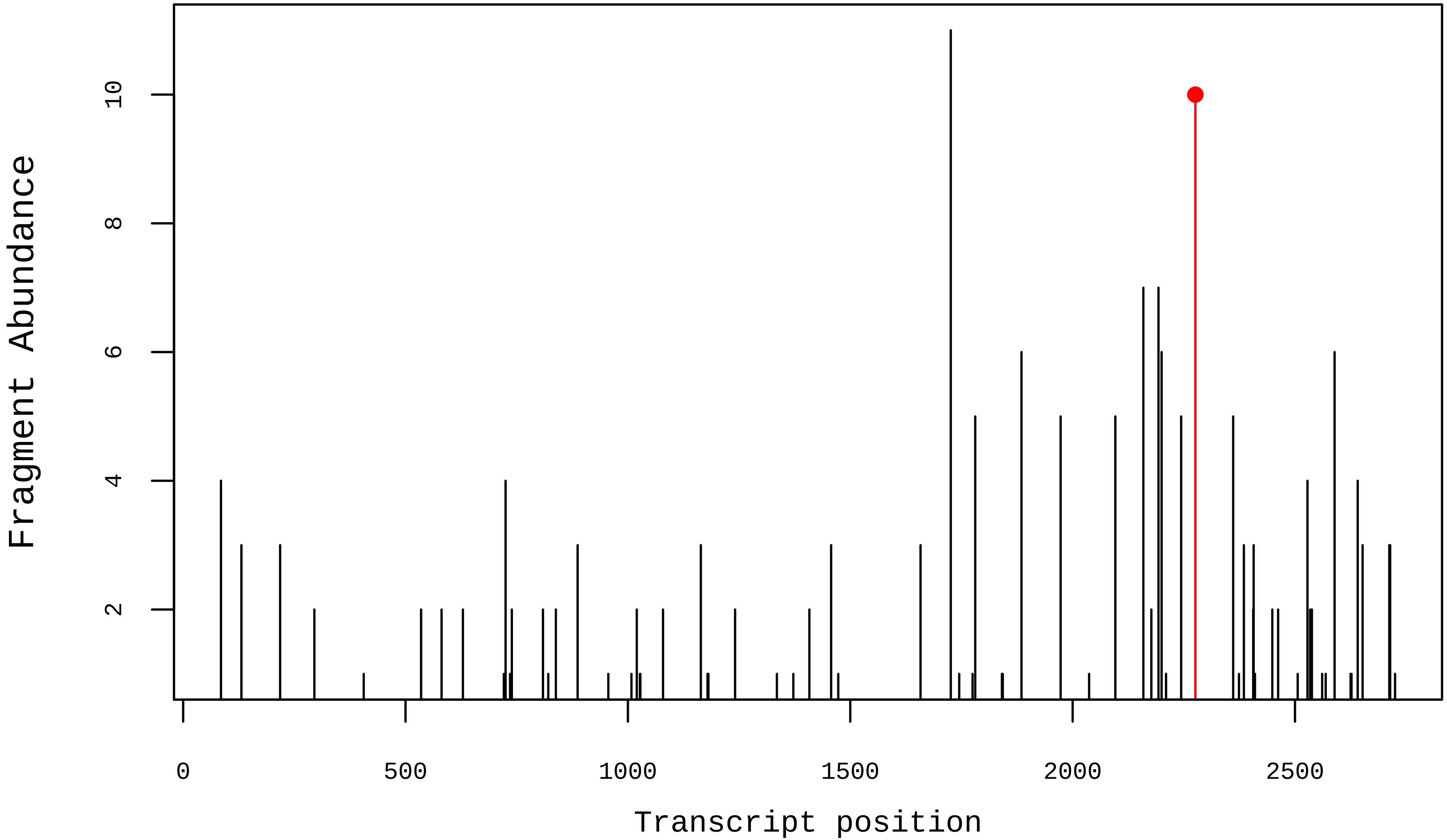

Cleavage site: 2276   Tag abundance: 10   Weighted abundance: 1.429   Category: 2
sRNA abundance: 1   Alignment score: 3.5   MFE ratio: 0.865   p-value: 0.037

HORVU6Hr1G017340|HORVU6Hr1G017340.5||2484|2790

5' GCAGCCTGCGGGAGCACAGACCGGGCGGAGGT '3
|||o||||| ||||||||
3' GCCTTCGTG-CTGGCCCGG '5

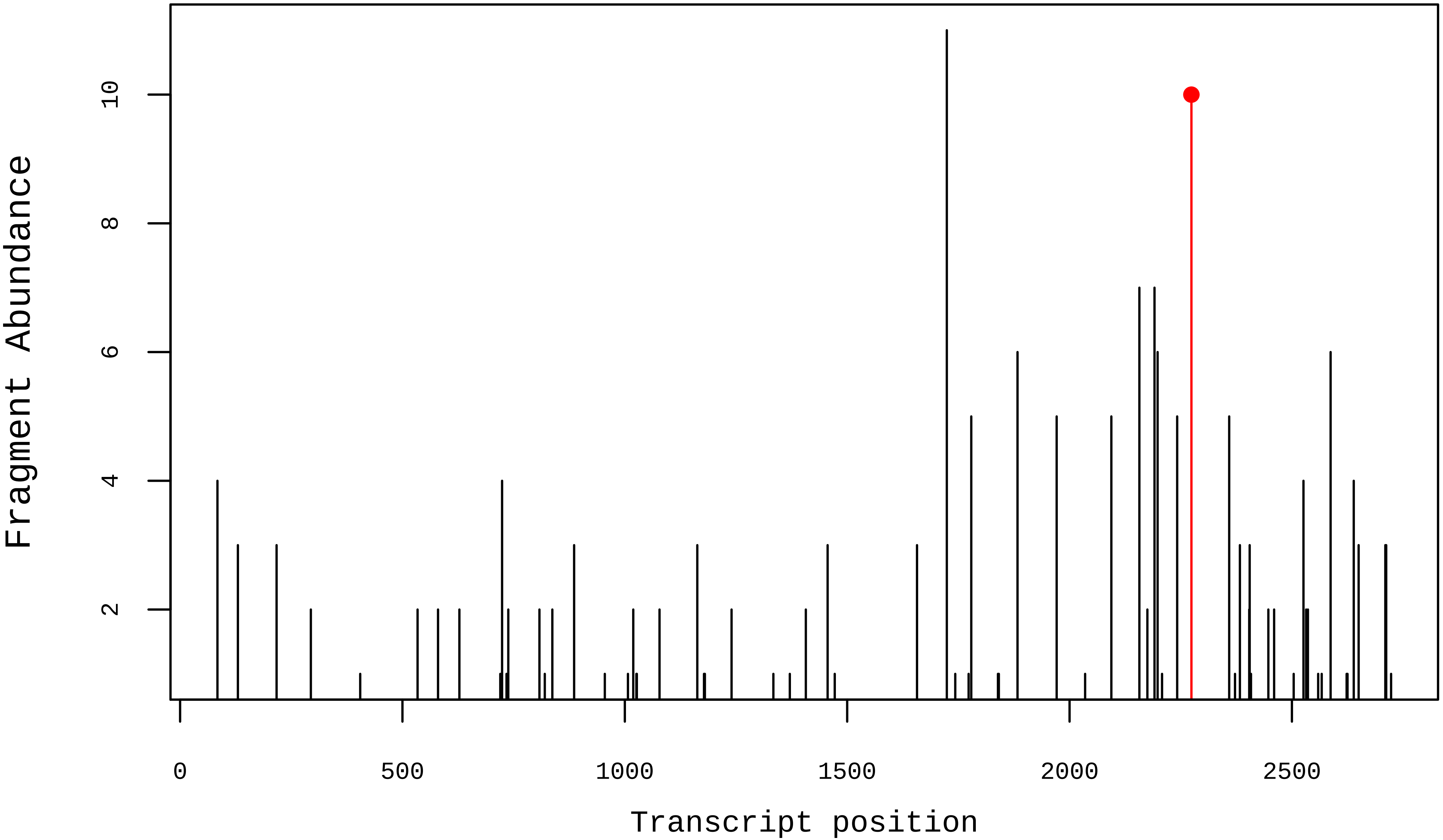

Cleavage site: 2274 Tag abundance: 10 Weighted abundance: 1.429 Category: 2
sRNA abundance: 1 Alignment score: 3.5 MFE ratio: 0.865 p-value: 0.037

HORVU6Hr1G017340|HORVU6Hr1G017340.6||2193|2722

```
5' GCAGCCTGCGGGAGCACAGACCGGGCGGAGGT '3
           |||o||||| |||||||||
3'         GCCTTCGTG-CTGGCCCGG      '5
```

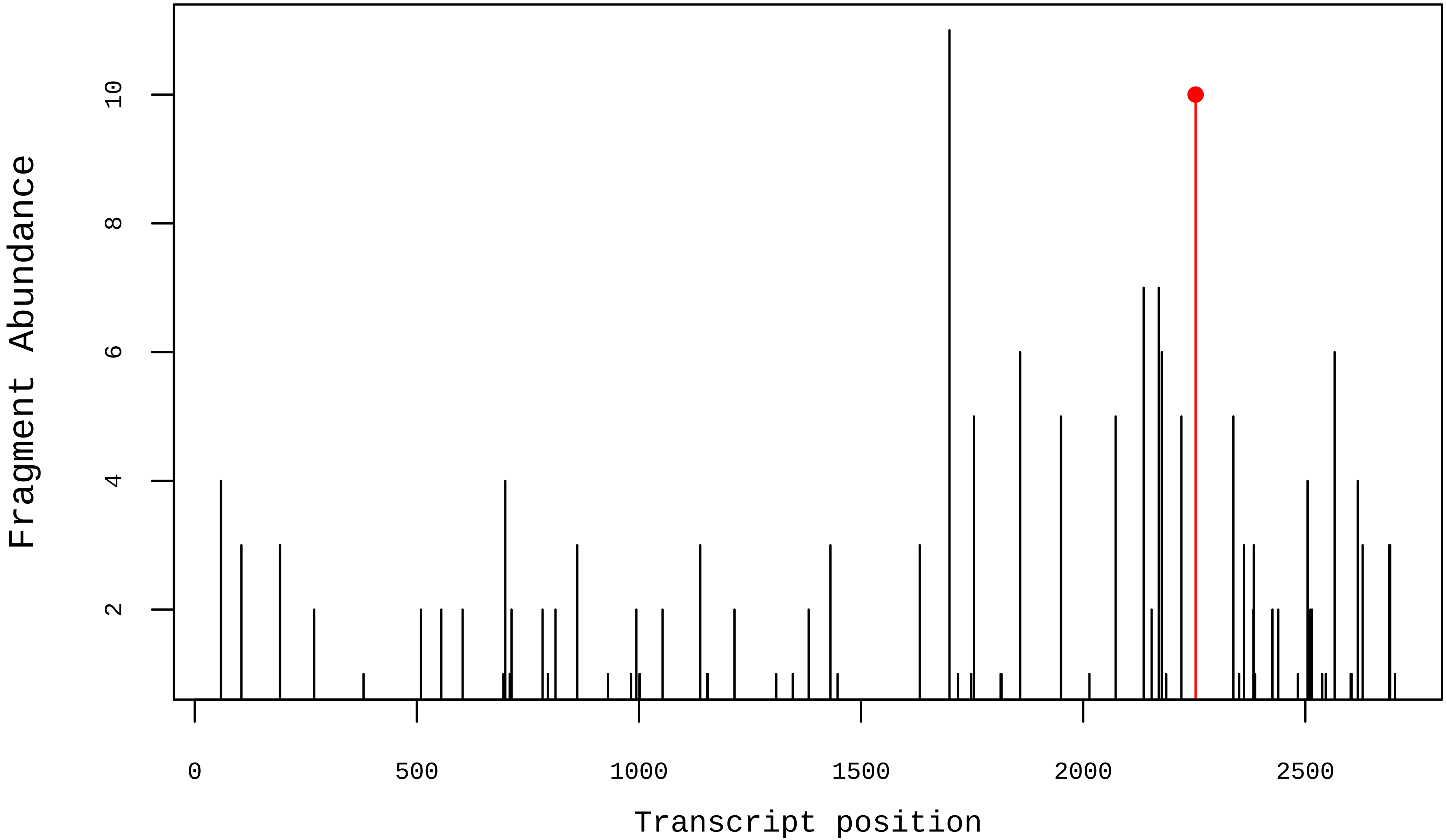

Cleavage site: 2253 Tag abundance: 10 Weighted abundance: 1.429 Category: 2
sRNA abundance: 1 Alignment score: 3.5 MFE ratio: 0.865 p-value: 0.038

HORVU6Hr1G017340|HORVU6Hr1G017340.7||4317|4317

```
5' GCAGCCTGCGGGAGCACAGACCGGGCGGAGGT '3
            |||o||||| |||||||||
3'          GCCTTCGTG-CTGGCCCGG        '5
```

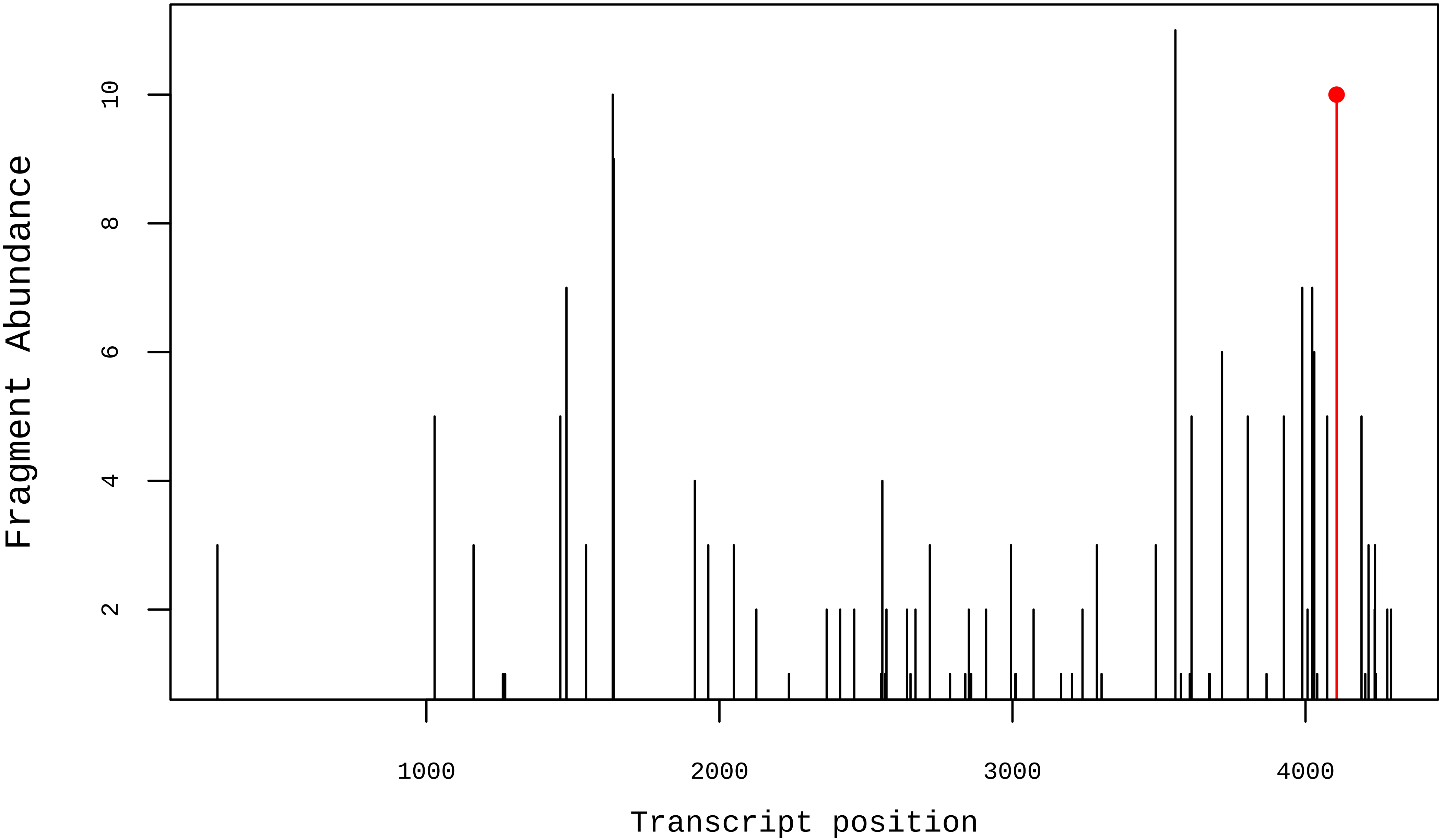

Cleavage site: 4106   Tag abundance: 10   Weighted abundance: 1.429   Category: 2
sRNA abundance: 1   Alignment score: 3.5   MFE ratio: 0.865   p-value: 0.024

HORVU2Hr1G021810|HORVU2Hr1G021810.1||285|528

```
5' ACGCTGGCGGAGGAGGTGCTGGGGAAGATGCA '3
            || ||||||||| ||||||o
3'          CCACCTCCACG-CCCCTTT      '5
```

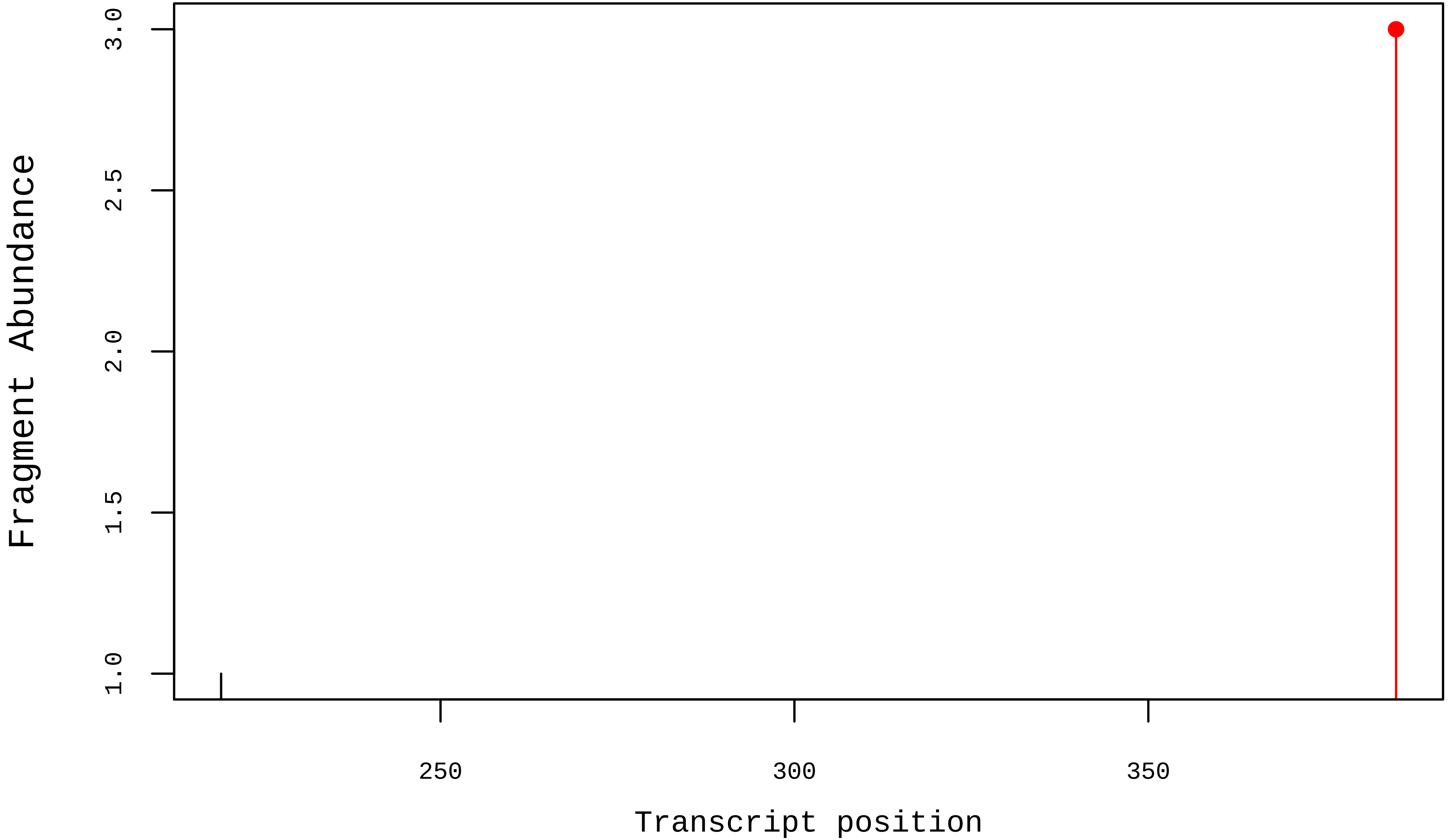

Cleavage site: 385   Tag abundance: 3   Weighted abundance: 0.386   Category: 0
sRNA abundance: 1   Alignment score: 3.5   MFE ratio: 0.789   p-value: 0.002

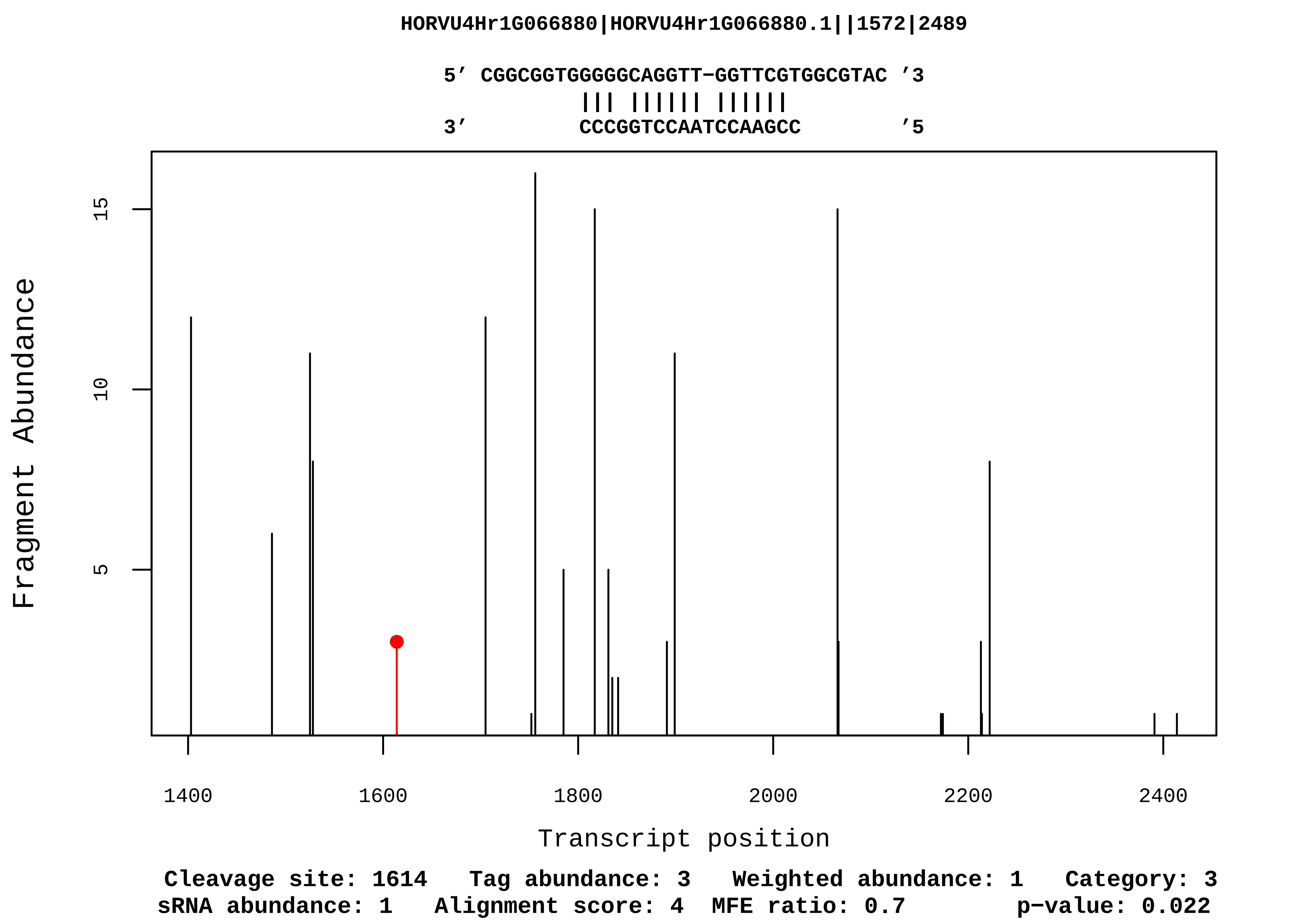
HORVU4Hr1G066880|HORVU4Hr1G066880.1||1572|2489
5' CGGCGGTGGGGGCAGGTT-GGTTCGTGGCGTAC '3
||| |||||| ||||||
3' CCCGGTCCAATCCAAGCC '5
Fragment Abundance
5
10
15
1400
1600
1800
2000
2200
2400
Transcript position
Cleavage site: 1614 Tag abundance: 3 Weighted abundance: 1 Category: 3
sRNA abundance: 1 Alignment score: 4 MFE ratio: 0.7 p-value: 0.022

HORVU4Hr1G066880|HORVU4Hr1G066880.2||654|1390

```
5' CGGCGGTGGGGGCAGGTT-GGTTCGTGGCGTAC '3
           ||| |||||| ||||||
3'         CCCGGTCCAATCCAAGCC         '5
```

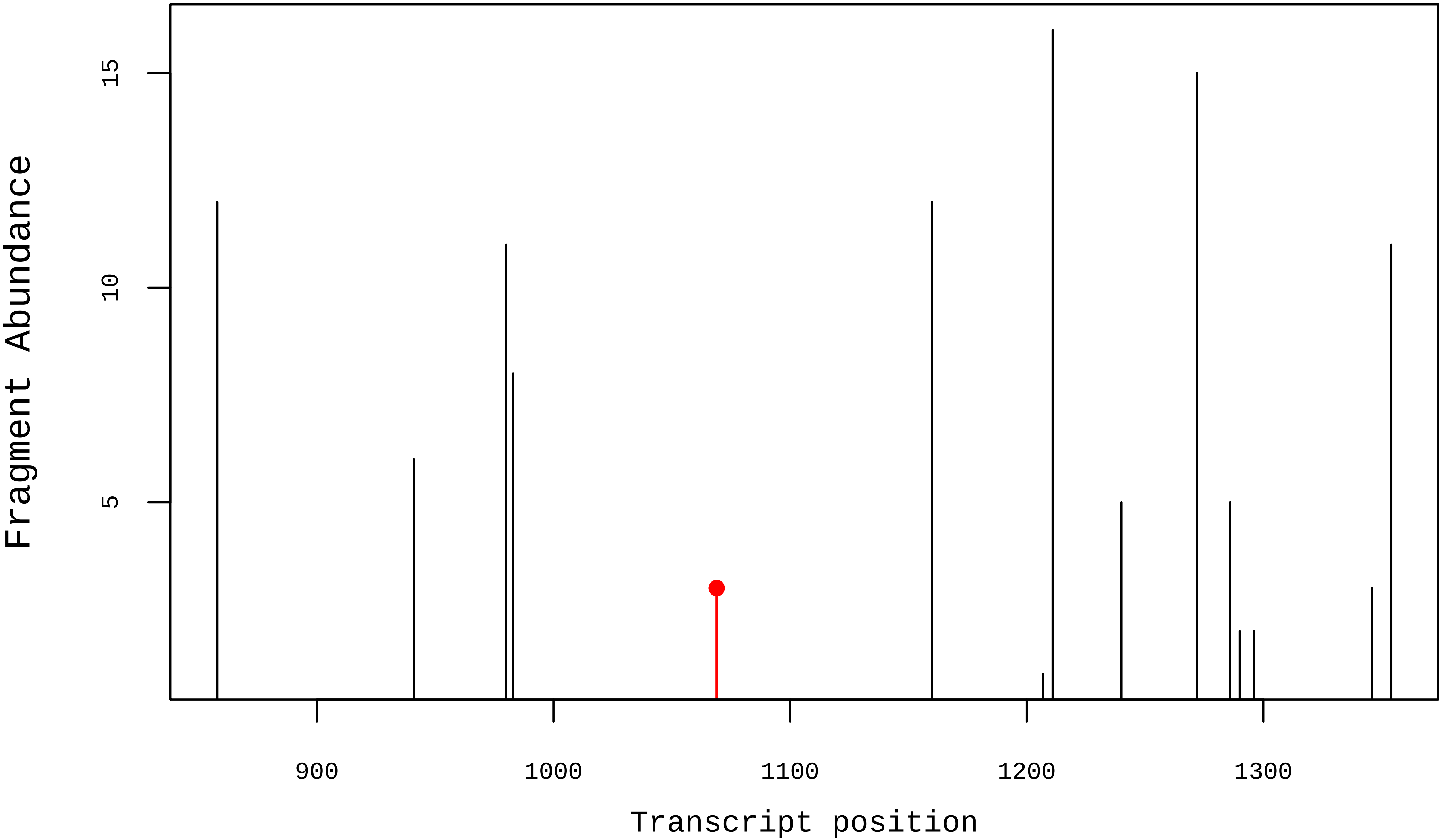

Cleavage site: 1069 Tag abundance: 3 Weighted abundance: 1 Category: 3
sRNA abundance: 1 Alignment score: 4 MFE ratio: 0.7 p-value: 0.03

HORVU0Hr1G023930|HORVU0Hr1G023930.1||297|1937

```
5' CGTCAGCCTTTTATCTAATAAATGCGCCCCTC '3
         ||||||||||||||||||||||
3'       TCGGAAAATAGATTATTTACGC       '5
```

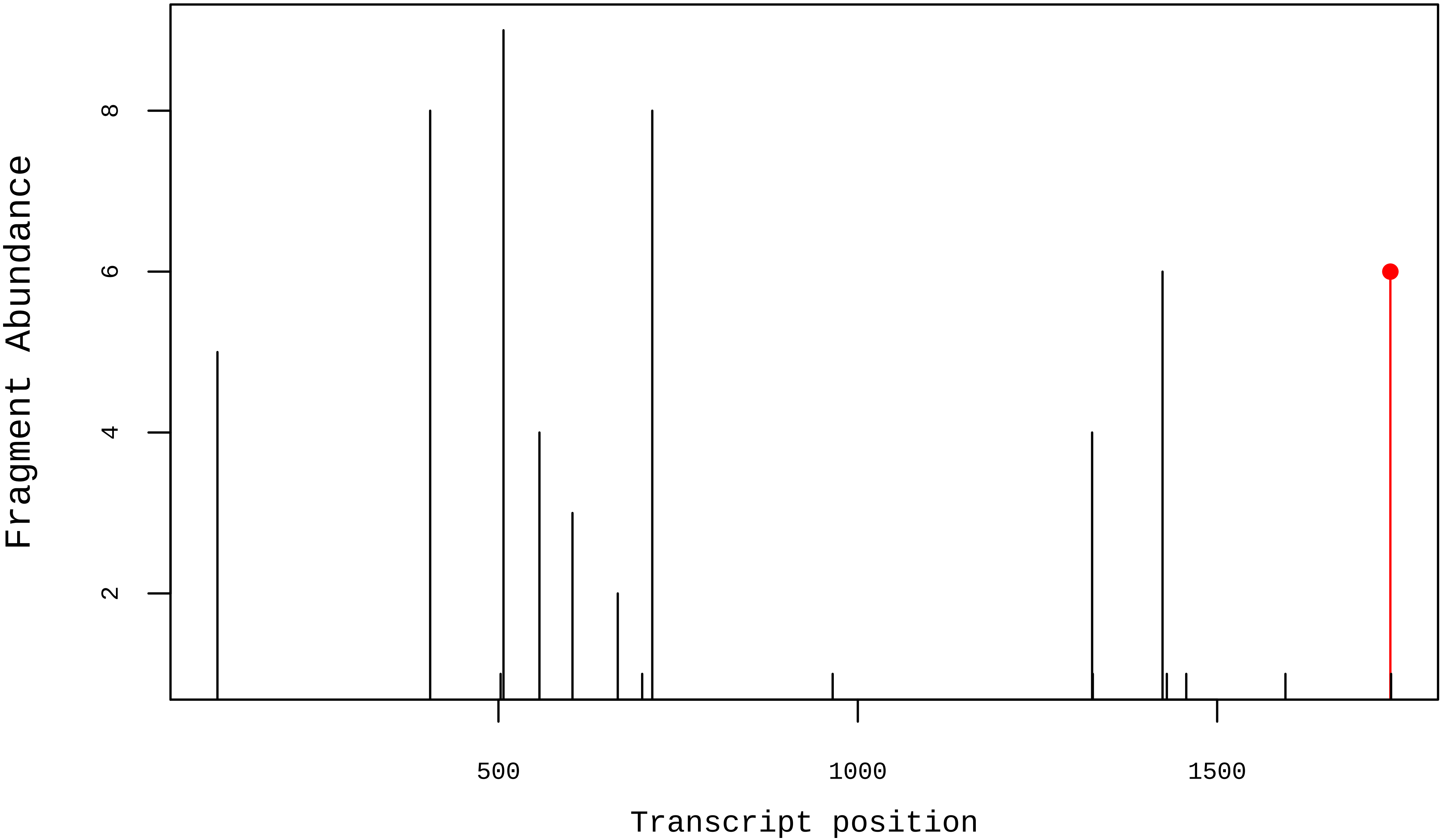

Cleavage site: 1741   Tag abundance: 6   Weighted abundance: 0.75   Category: 2
sRNA abundance: 1   Alignment score: 0   MFE ratio: 1   p-value: 0.017

HORVU5Hr1G015600|HORVU5Hr1G015600.16||228|588

```
5' CGTCAGCCTTTTATCTAATAAATGCGCCCCTC '3
         ||||||||||||||||||||||
3'       TCGGAAAATAGATTATTTACGC       '5
```

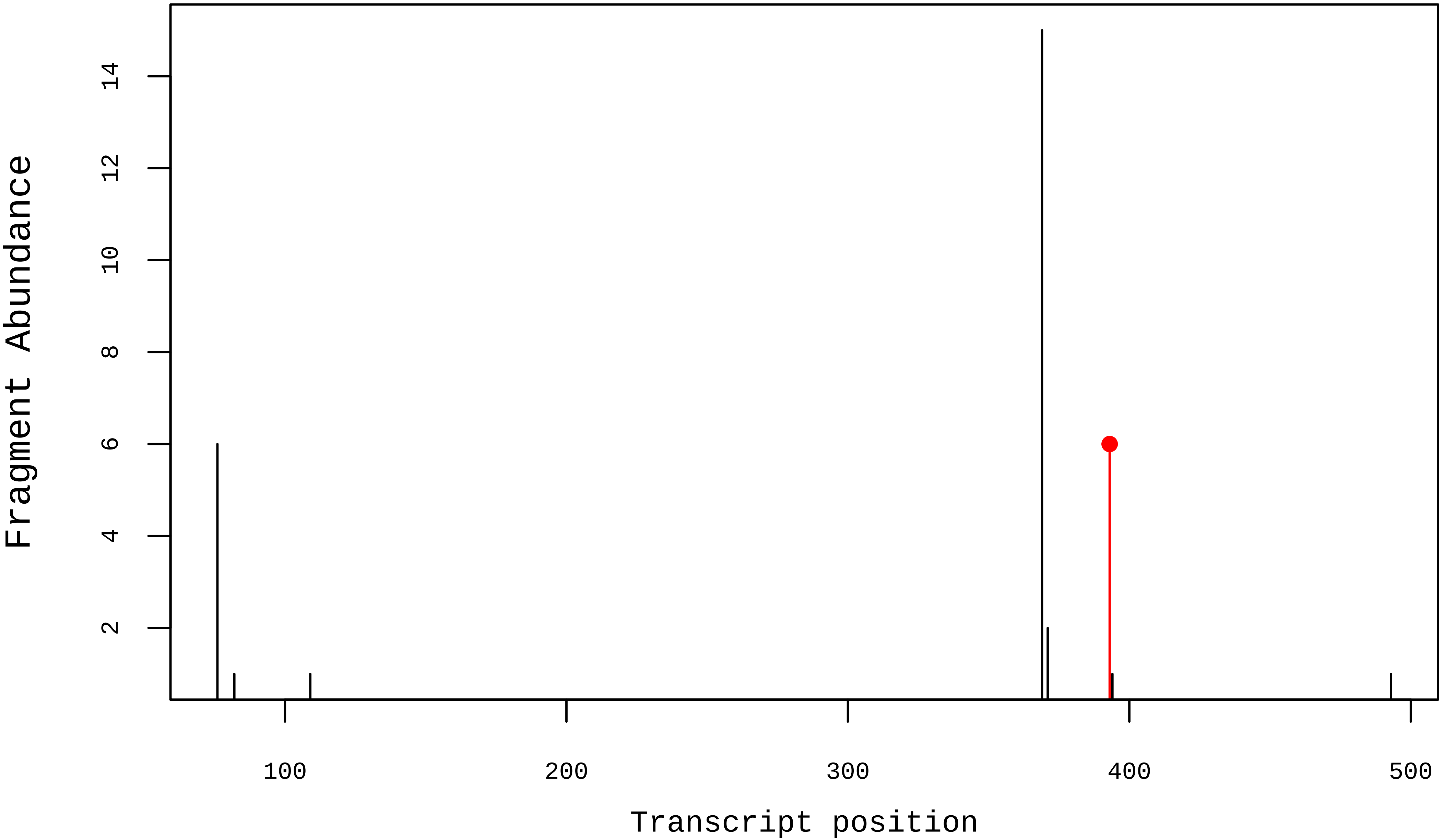

Cleavage site: 393   Tag abundance: 6   Weighted abundance: 0.75   Category: 3
sRNA abundance: 1   Alignment score: 0   MFE ratio: 1   p-value: 0.041

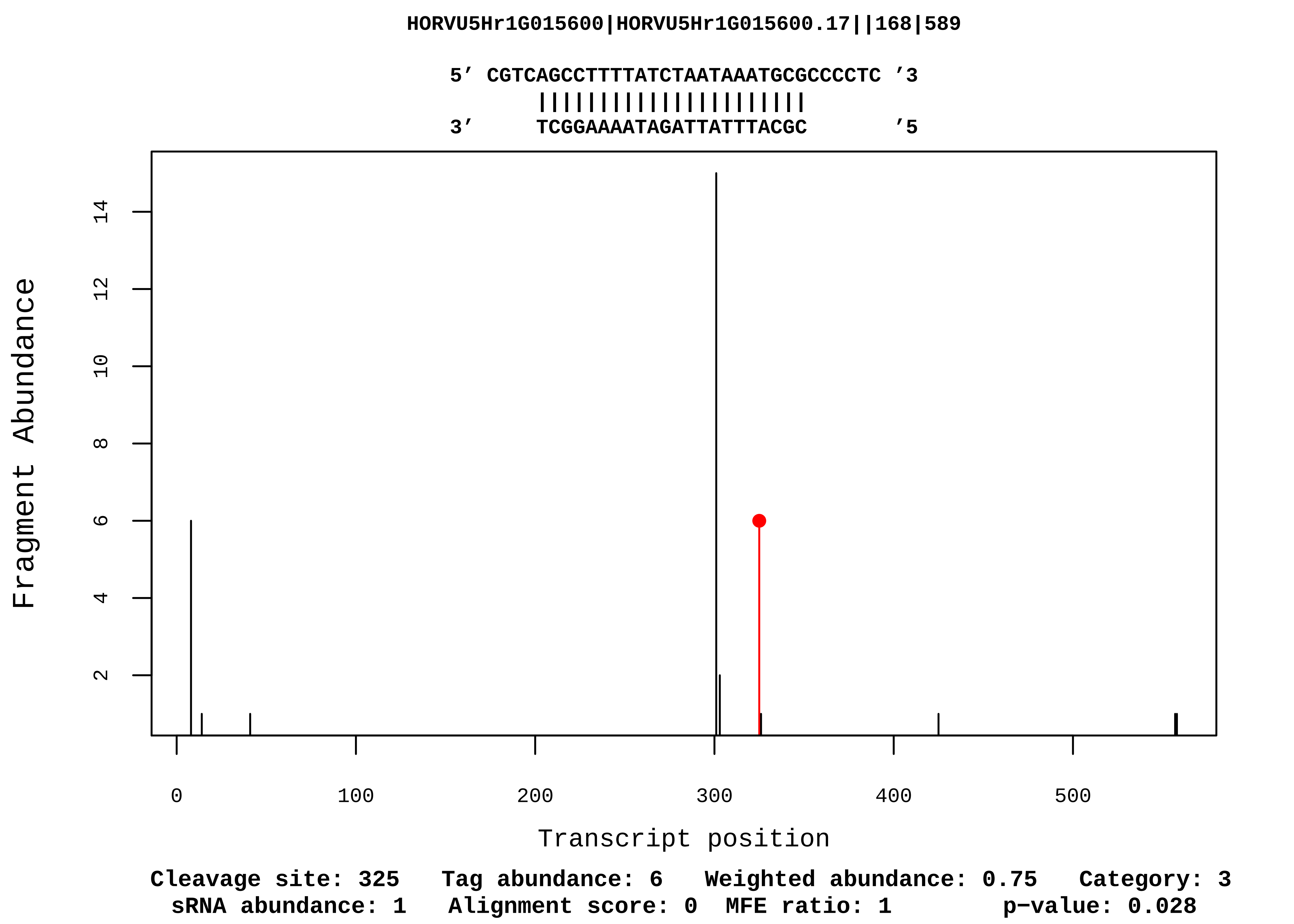
HORVU5Hr1G015600|HORVU5Hr1G015600.17||168|589
5' CGTCAGCCTTTTATCTAATAAATGCGCCCCTC '3
||||||||||||||||||||||
3' TCGGAAAATAGATTATTTACGC '5
Fragment Abundance
2
4
6
8
10
12
14
0
100
200
300
400
500
Transcript position
Cleavage site: 325 Tag abundance: 6 Weighted abundance: 0.75 Category: 3
sRNA abundance: 1 Alignment score: 0 MFE ratio: 1 p-value: 0.028

HORVU5Hr1G015600|HORVU5Hr1G015600.2||231|617

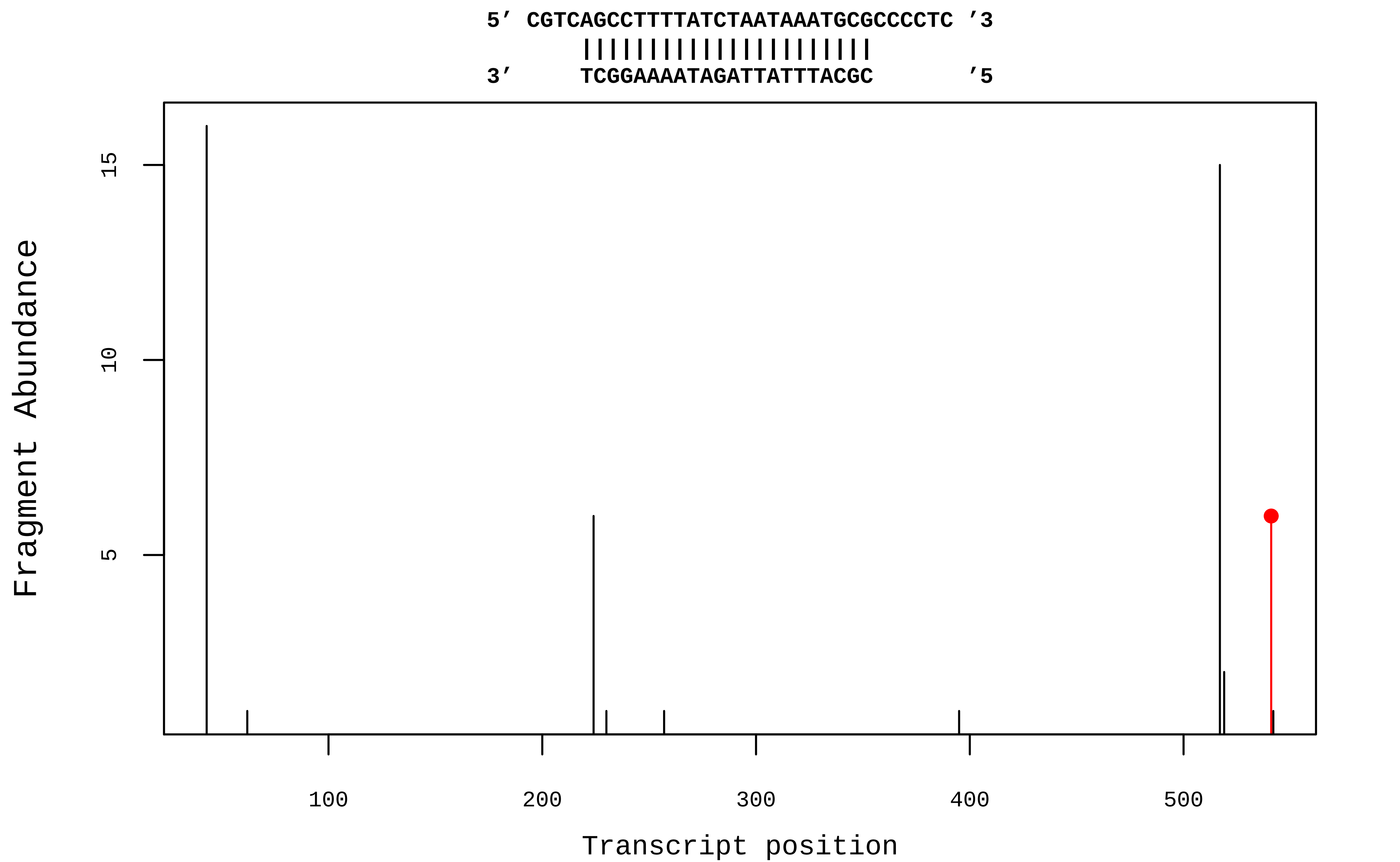

Cleavage site: 541   Tag abundance: 6   Weighted abundance: 0.75   Category: 3
sRNA abundance: 1   Alignment score: 0   MFE ratio: 1   p-value: 0.039

HORVU5Hr1G015600|HORVU5Hr1G015600.5||162|416

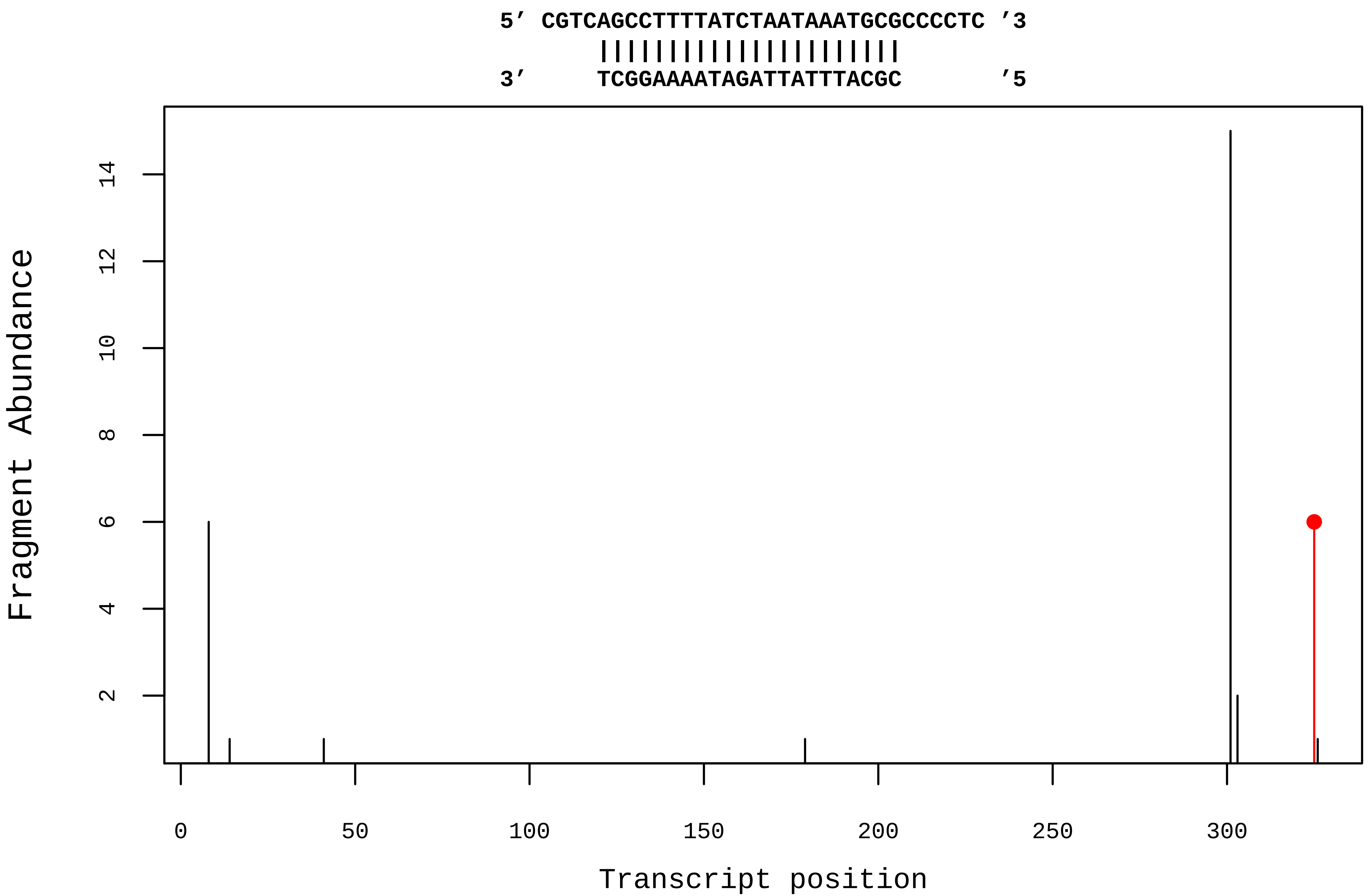

Cleavage site: 325 Tag abundance: 6 Weighted abundance: 0.75 Category: 3
sRNA abundance: 1 Alignment score: 0 MFE ratio: 1 p-value: 0.04

HORVU5Hr1G015600|HORVU5Hr1G015600.6||168|658

```
5' CGTCAGCCTTTTATCTAATAAATGCGCCCCTC '3
         ||||||||||||||||||||||
3'       TCGGAAAATAGATTATTTACGC       '5
```

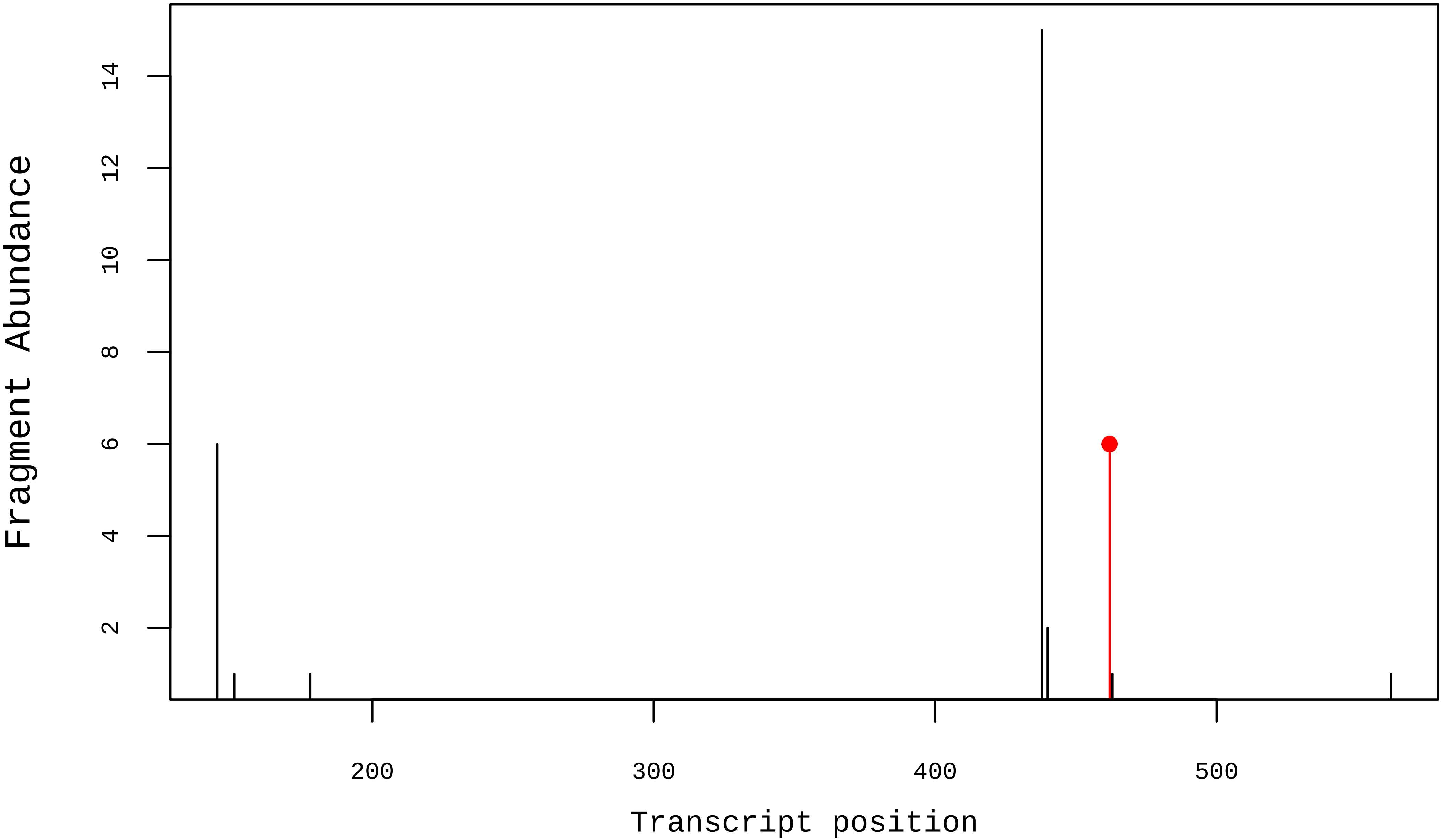

Cleavage site: 462 Tag abundance: 6 Weighted abundance: 0.75 Category: 3
sRNA abundance: 1 Alignment score: 0 MFE ratio: 1 p-value: 0.037

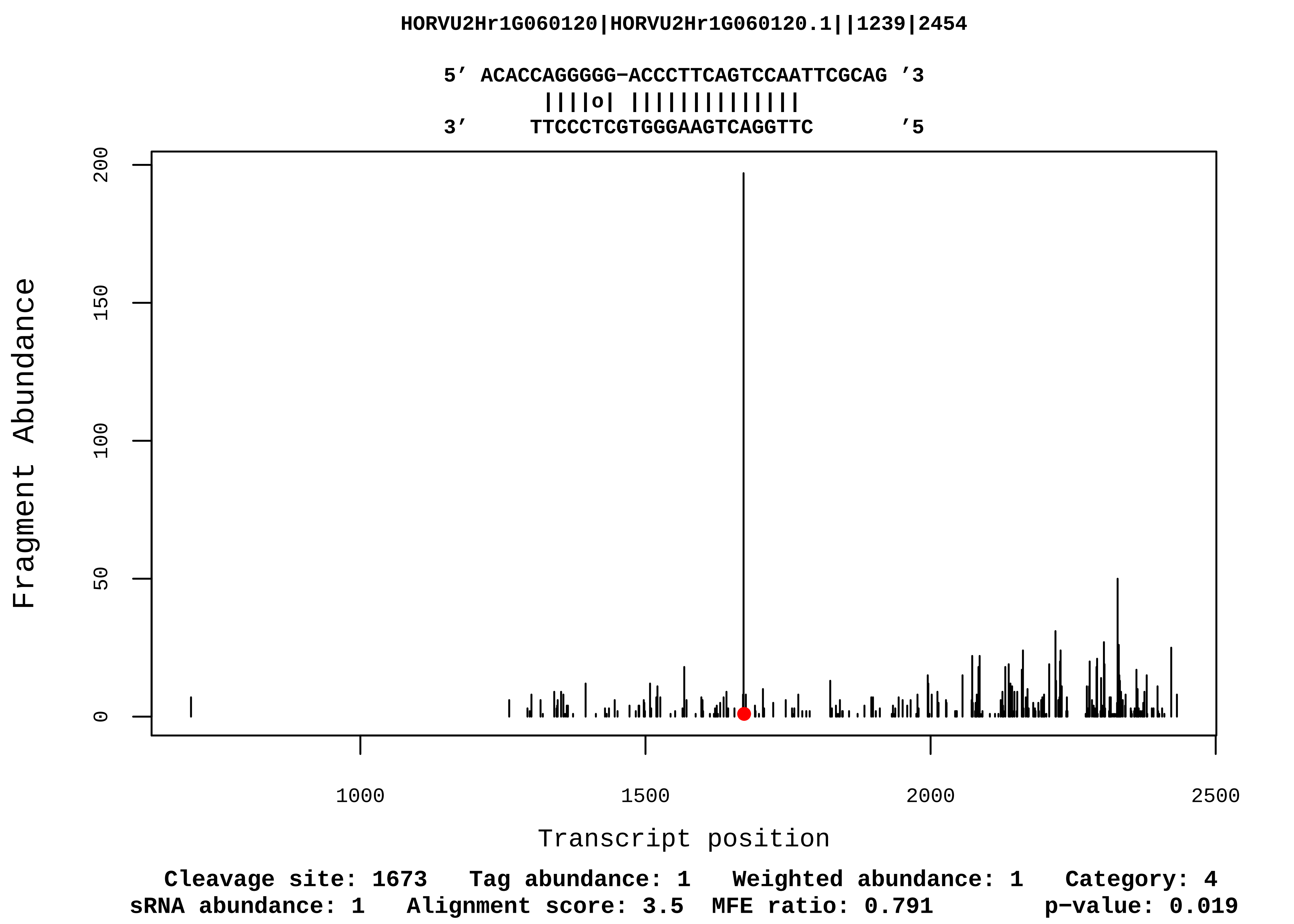

HORVU2Hr1G060120|HORVU2Hr1G060120.1||1239|2454
5' ACACCAGGGGG-ACCCTTCAGTCCAATTCGCAG '3
||||o| ||||||||||||||
3' TTCCCTCGTGGGAAGTCAGGTTC '5
Fragment Abundance
0
50
100
150
200
1000
1500
2000
2500
Transcript position
Cleavage site: 1673 Tag abundance: 1 Weighted abundance: 1 Category: 4
sRNA abundance: 1 Alignment score: 3.5 MFE ratio: 0.791 p-value: 0.019

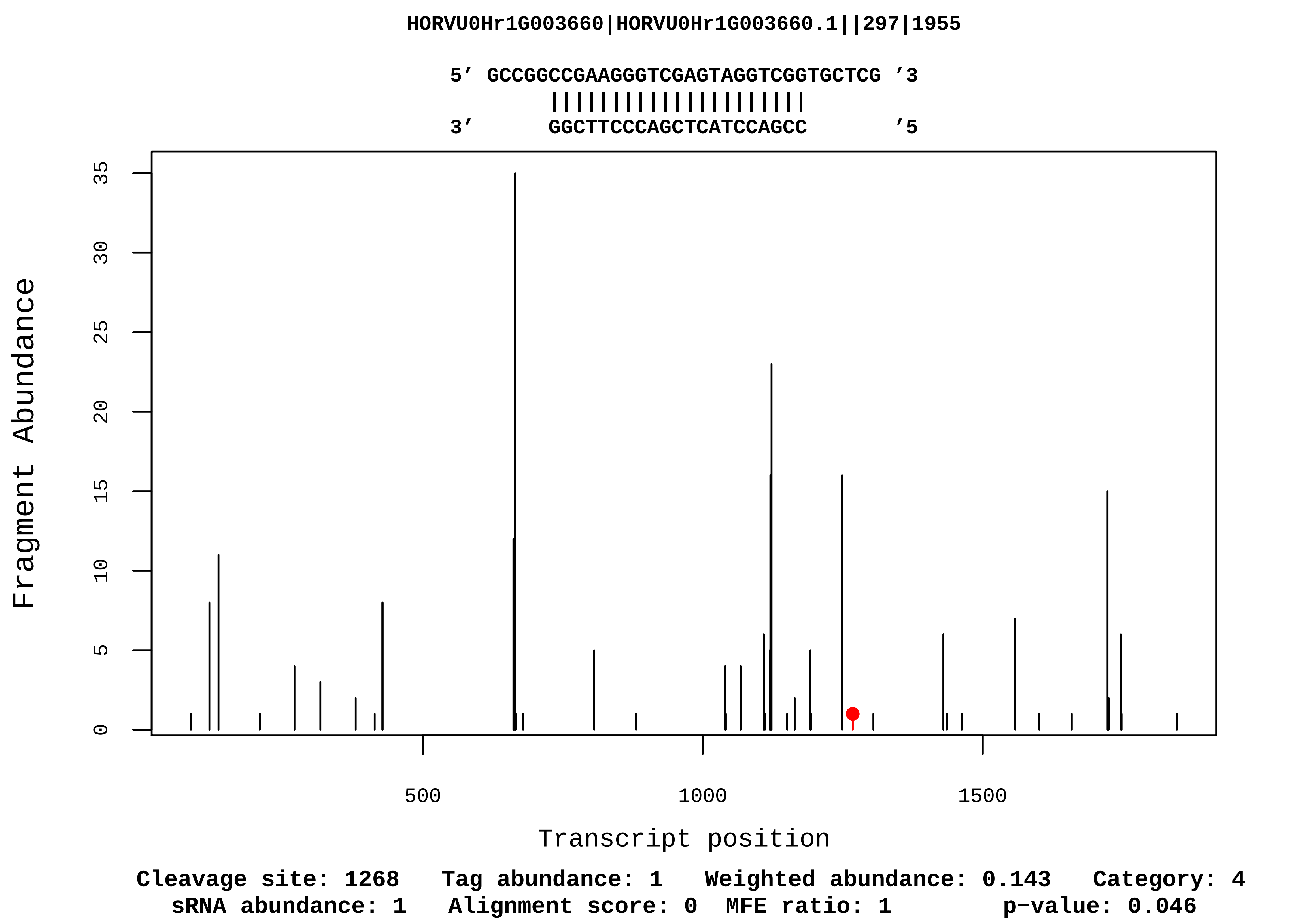
HORVU0Hr1G003660|HORVU0Hr1G003660.1||297|1955
5′ GCCGGCCGAAGGGTCGAGTAGGTCGGTGCTCG ′3
||||||||||||||||||||||
3′ GGCTTCCCAGCTCATCCAGCC ′5
Fragment Abundance
0
5
10
15
20
25
30
35
500
1000
1500
Transcript position
Cleavage site: 1268 Tag abundance: 1 Weighted abundance: 0.143 Category: 4
sRNA abundance: 1 Alignment score: 0 MFE ratio: 1 p−value: 0.046

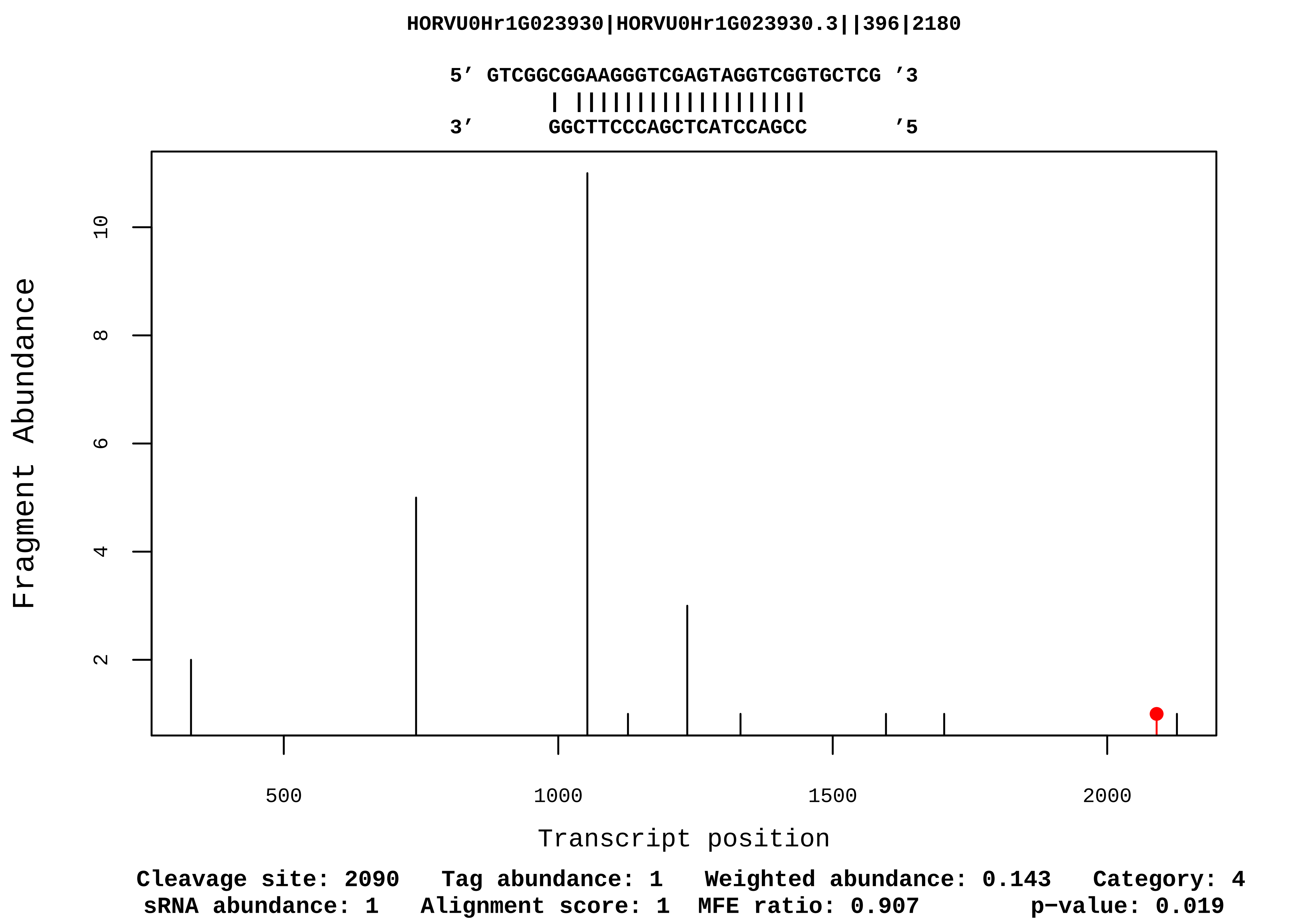
HORVU0Hr1G023930|HORVU0Hr1G023930.3||396|2180
5' GTCGGCGGAAGGGTCGAGTAGGTCGGTGCTCG '3
3' GGCTTCCCAGCTCATCCAGCC '5
Fragment Abundance
2
4
6
8
10
500
1000
1500
2000
Transcript position
Cleavage site: 2090 Tag abundance: 1 Weighted abundance: 0.143 Category: 4
sRNA abundance: 1 Alignment score: 1 MFE ratio: 0.907 p-value: 0.019

HORVU2Hr1G072960|HORVU2Hr1G072960.1||600|3353

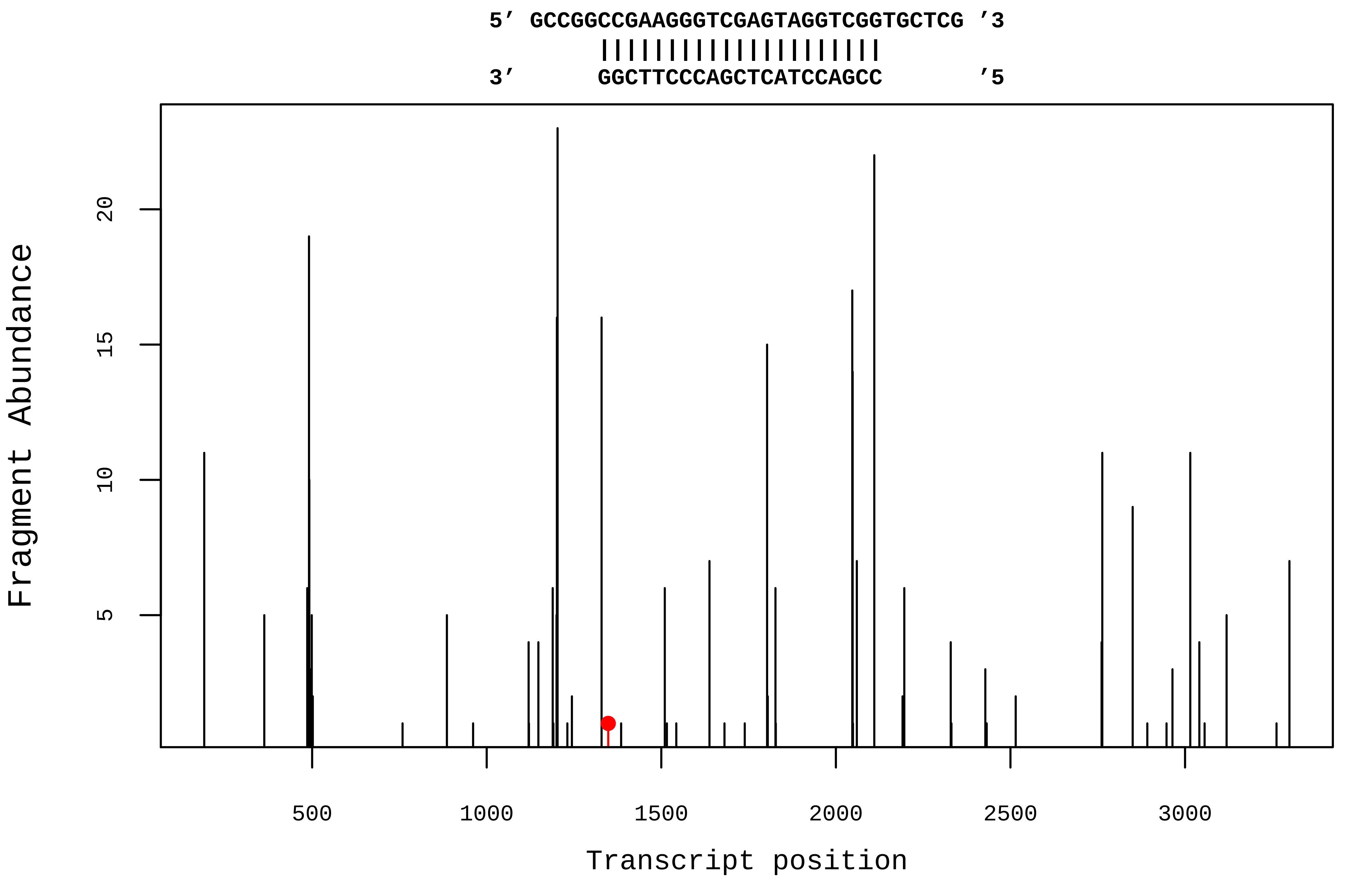

Cleavage site: 1348   Tag abundance: 1   Weighted abundance: 0.143   Category: 4
sRNA abundance: 1   Alignment score: 0   MFE ratio: 1   p-value: 0.03

HORVU5Hr1G015600|HORVU5Hr1G015600.1||156|510

```
5' GCCGGCCGAAGGGTCGAGTAGGTCGGTGCTCG '3
          ||||||||||||||||||||
3'         GGCTTCCCAGCTCATCCAGCC        '5
```

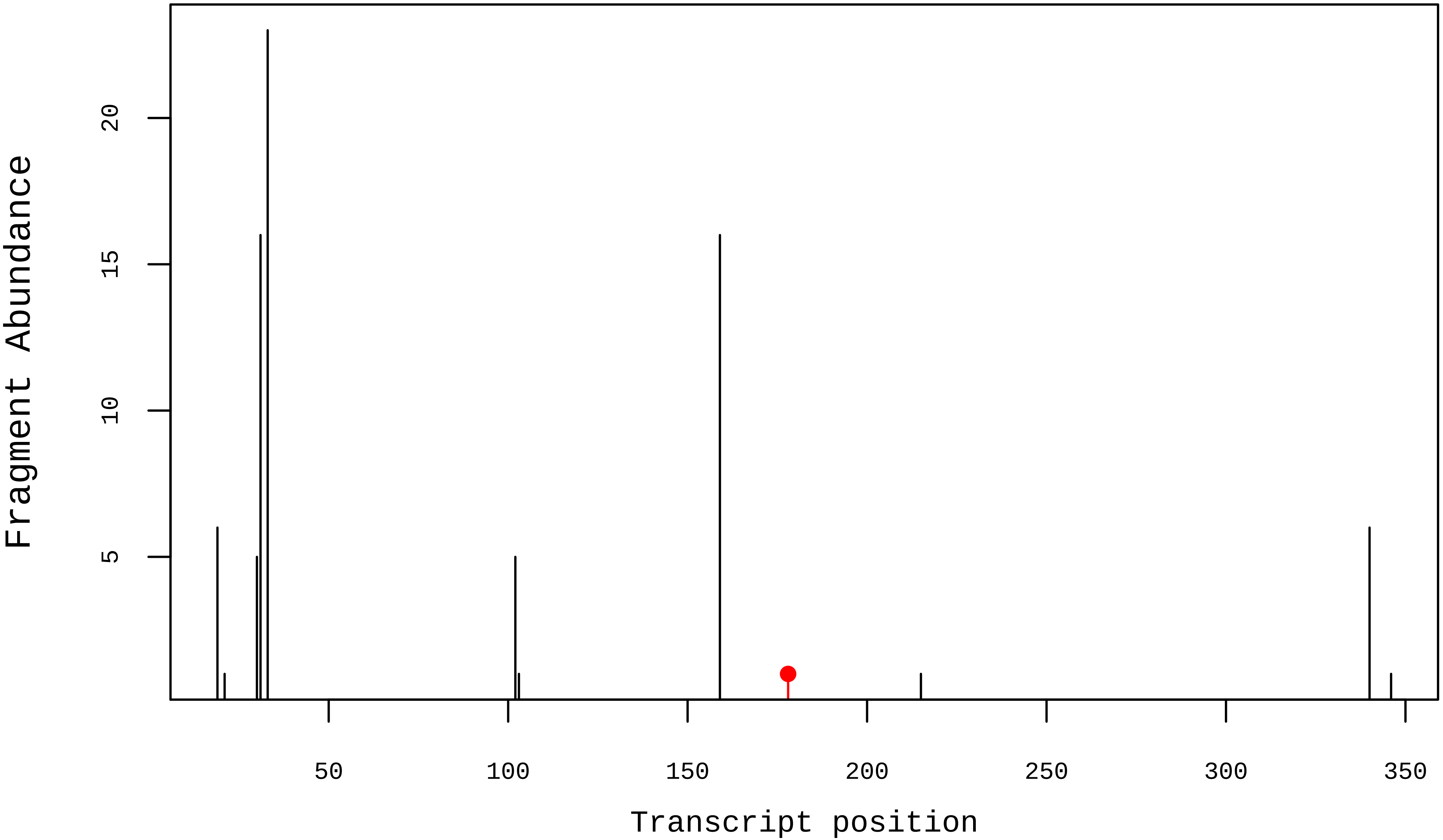

Cleavage site: 178   Tag abundance: 1   Weighted abundance: 0.143   Category: 4
sRNA abundance: 1   Alignment score: 0   MFE ratio: 1   p-value: 0.05

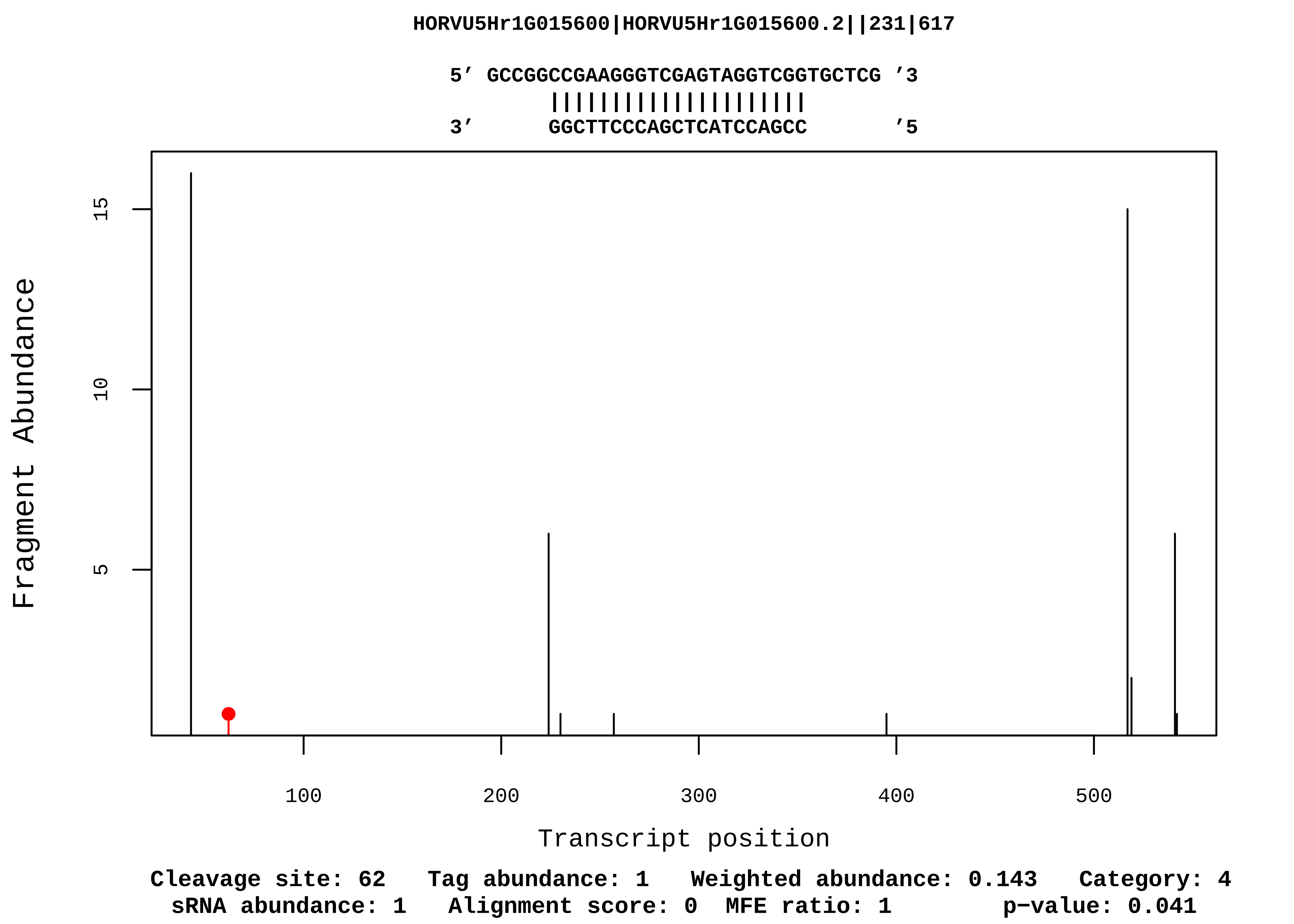
HORVU5Hr1G015600|HORVU5Hr1G015600.2||231|617
5' GCCGGCCGAAGGGTCGAGTAGGTCGGTGCTCG '3
||||||||||||||||||||||
3' GGCTTCCCAGCTCATCCAGCC '5
Fragment Abundance
5
10
15
100
200
300
400
500
Transcript position
Cleavage site: 62 Tag abundance: 1 Weighted abundance: 0.143 Category: 4
sRNA abundance: 1 Alignment score: 0 MFE ratio: 1 p-value: 0.041

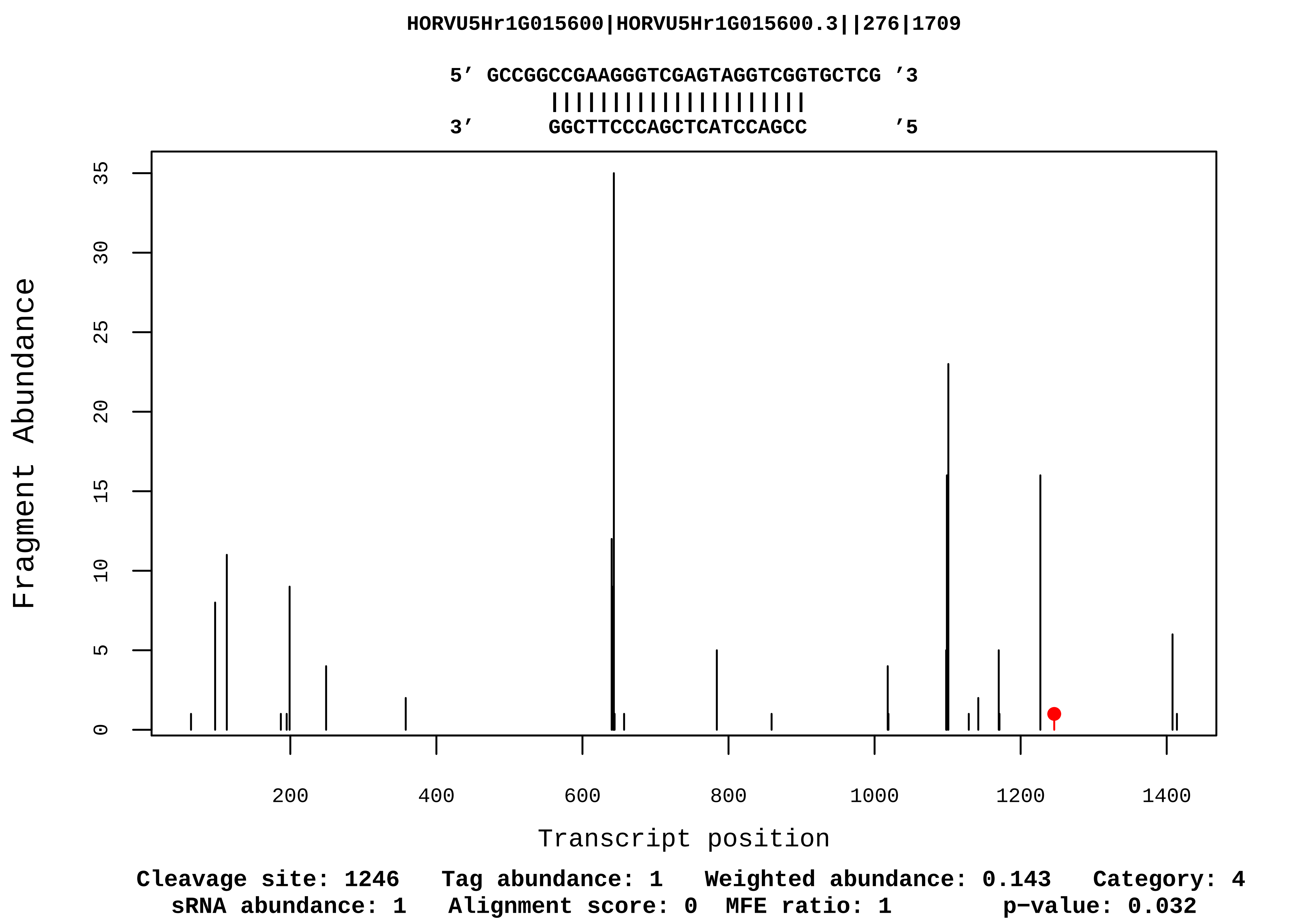
HORVU5Hr1G015600|HORVU5Hr1G015600.3||276|1709
5' GCCGGCCGAAGGGTCGAGTAGGTCGGTGCTCG '3
||||||||||||||||||||
3' GGCTTCCCAGCTCATCCAGCC '5
Fragment Abundance
0
5
10
15
20
25
30
35
200
400
600
800
1000
1200
1400
Transcript position
Cleavage site: 1246 Tag abundance: 1 Weighted abundance: 0.143 Category: 4
sRNA abundance: 1 Alignment score: 0 MFE ratio: 1 p-value: 0.032

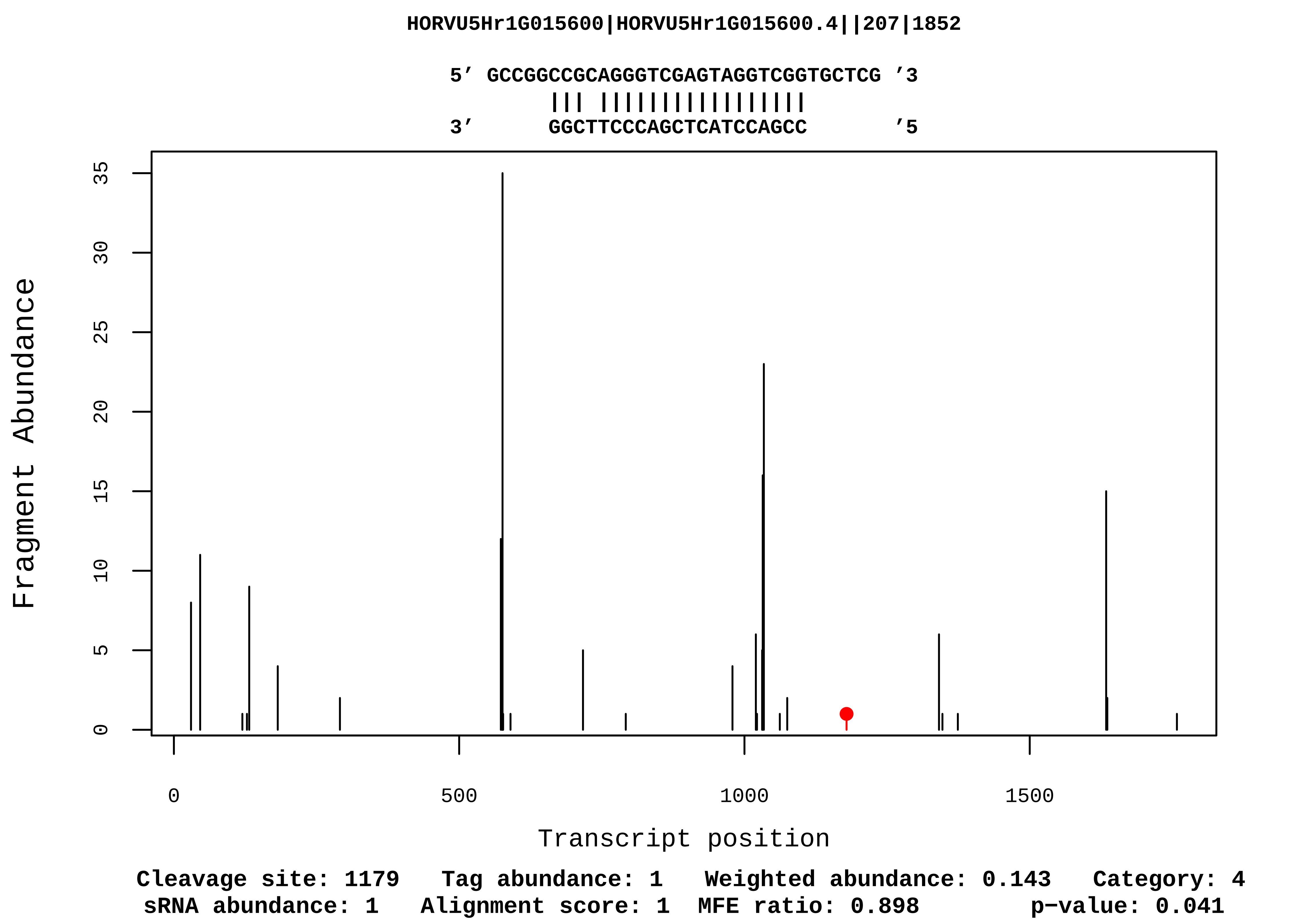
HORVU5Hr1G015600|HORVU5Hr1G015600.4||207|1852
5' GCCGGCCGCAGGGTCGAGTAGGTCGGTGCTCG '3
||| ||||||||||||||||
3' GGCTTCCCAGCTCATCCAGCC '5
Fragment Abundance
0
5
10
15
20
25
30
35
0
500
1000
1500
Transcript position
Cleavage site: 1179 Tag abundance: 1 Weighted abundance: 0.143 Category: 4
sRNA abundance: 1 Alignment score: 1 MFE ratio: 0.898 p-value: 0.041

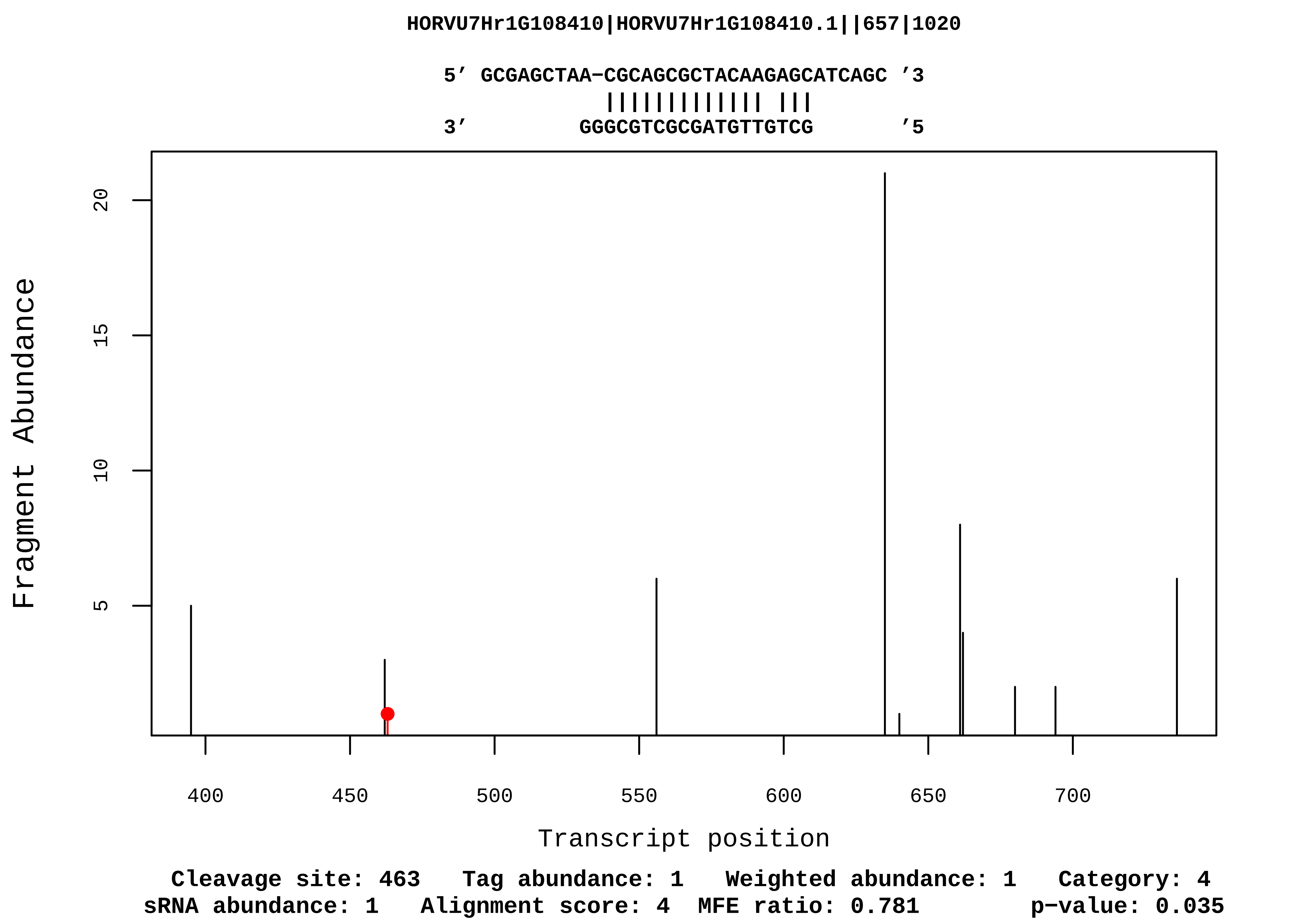
HORVU7Hr1G108410|HORVU7Hr1G108410.1||657|1020
5' GCGAGCTAA-CGCAGCGCTACAAGAGCATCAGC '3
||||||||||||| |||
3' GGGCGTCGCGATGTTGTCG '5
Fragment Abundance
5
10
15
20
400
450
500
550
600
650
700
Transcript position
Cleavage site: 463 Tag abundance: 1 Weighted abundance: 1 Category: 4
sRNA abundance: 1 Alignment score: 4 MFE ratio: 0.781 p-value: 0.035

HORVU1Hr1G070270|HORVU1Hr1G070270.3||1680|3936

```
5' GGTCCTTGTATG-TTTAGCCCTCCGTTCCTAAA '3
            o| | ||||||||||||||
3'          GTCCAAAATCGGGAGGCAA       '5
```

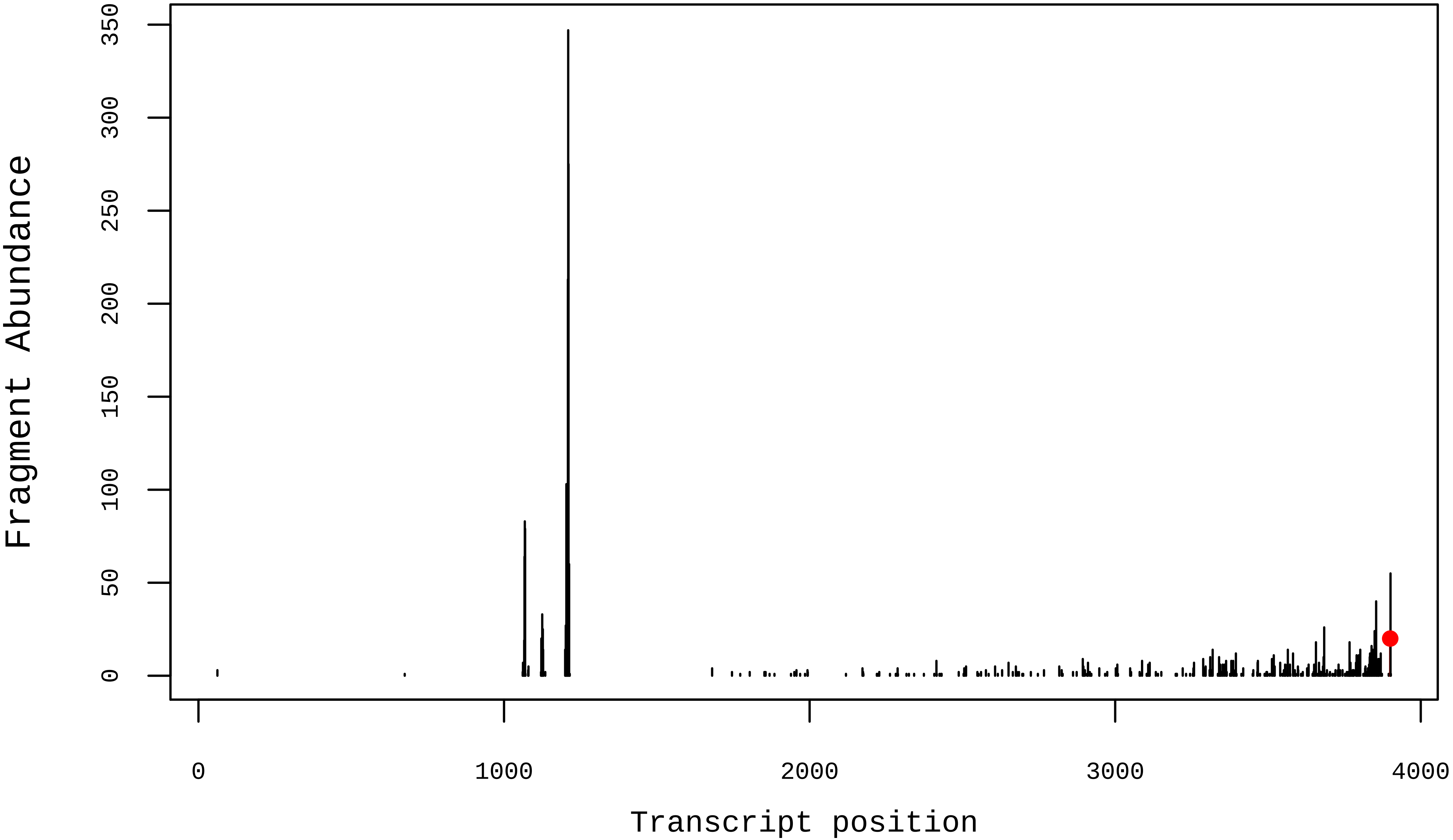

Cleavage site: 3900   Tag abundance: 20   Weighted abundance: 3.333   Category: 2
sRNA abundance: 1   Alignment score: 2.5   MFE ratio: 0.701   p-value: 0.033

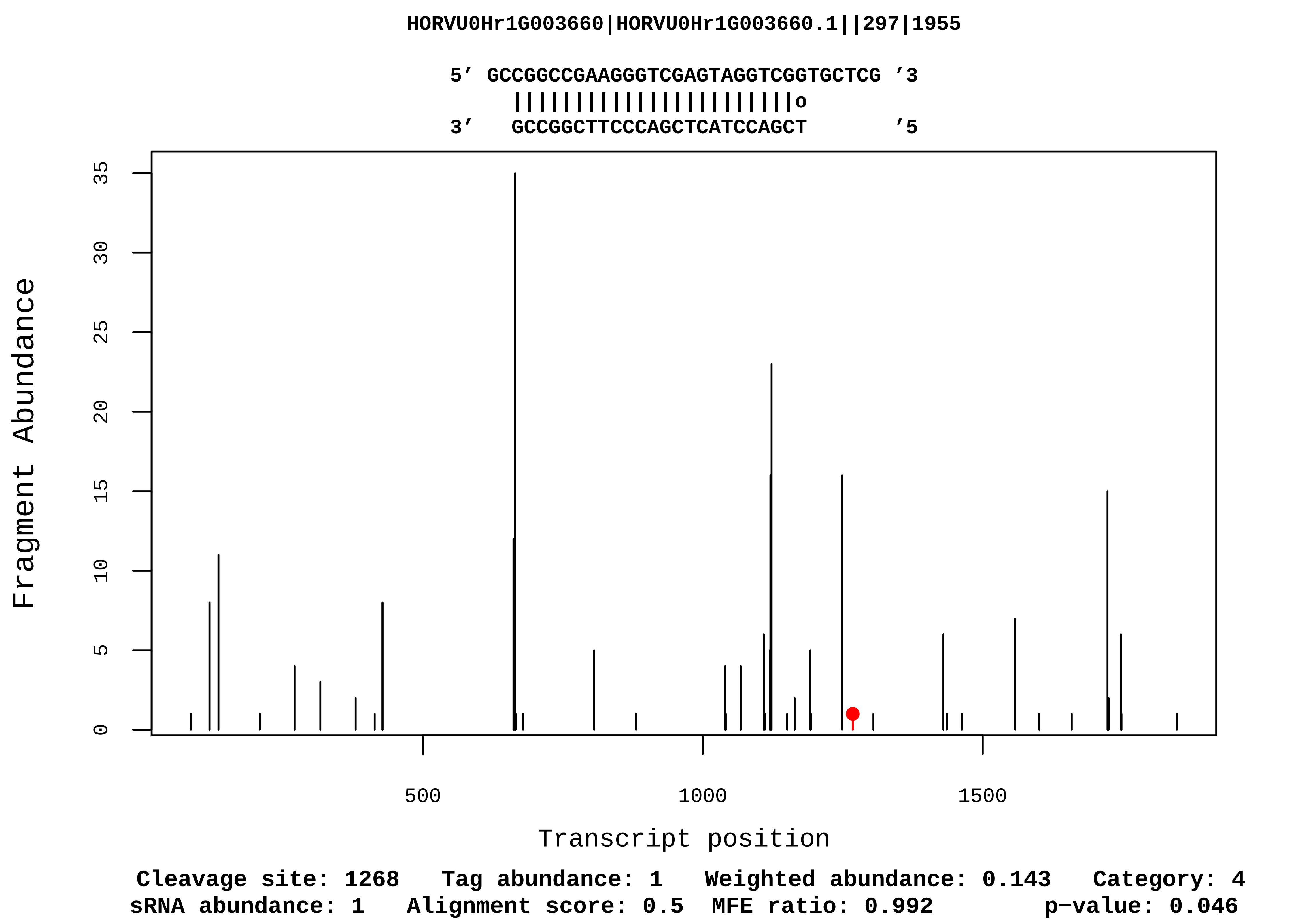

HORVU0Hr1G003660|HORVU0Hr1G003660.1||297|1955
5′ GCCGGCCGAAGGGTCGAGTAGGTCGGTGCTCG ′3
||||||||||||||||||||||o
3′ GCCGGCTTCCCAGCTCATCCAGCT ′5
Fragment Abundance
0
5
10
15
20
25
30
35
500
1000
1500
Transcript position
Cleavage site: 1268 Tag abundance: 1 Weighted abundance: 0.143 Category: 4
sRNA abundance: 1 Alignment score: 0.5 MFE ratio: 0.992 p-value: 0.046

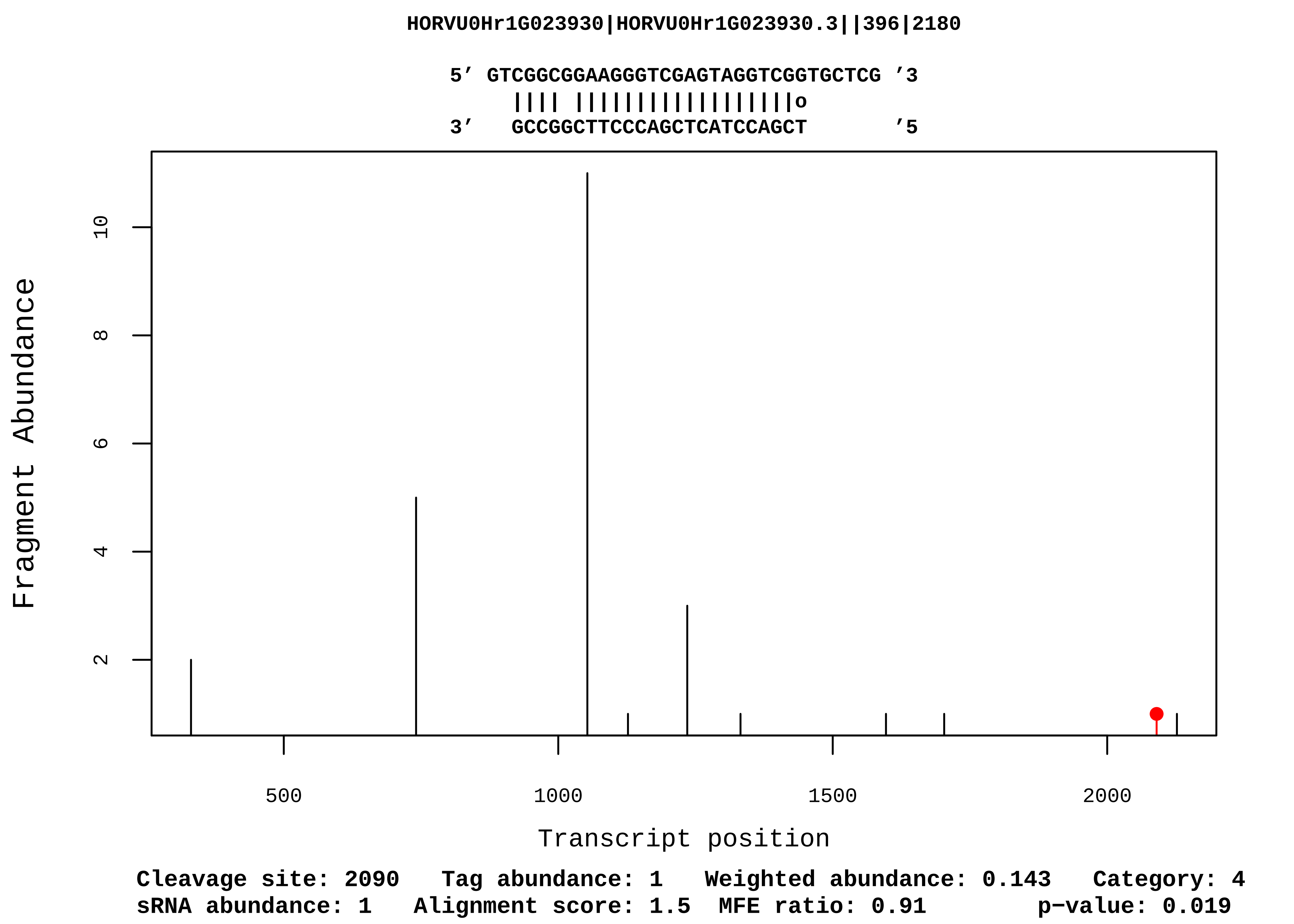
HORVU0Hr1G023930|HORVU0Hr1G023930.3||396|2180
5' GTCGGCGGAAGGGTCGAGTAGGTCGGTGCTCG '3
|||| ||||||||||||||||||o
3' GCCGGCTTCCCAGCTCATCCAGCT '5
Fragment Abundance
2
4
6
8
10
500
1000
1500
2000
Transcript position
Cleavage site: 2090 Tag abundance: 1 Weighted abundance: 0.143 Category: 4
sRNA abundance: 1 Alignment score: 1.5 MFE ratio: 0.91 p-value: 0.019

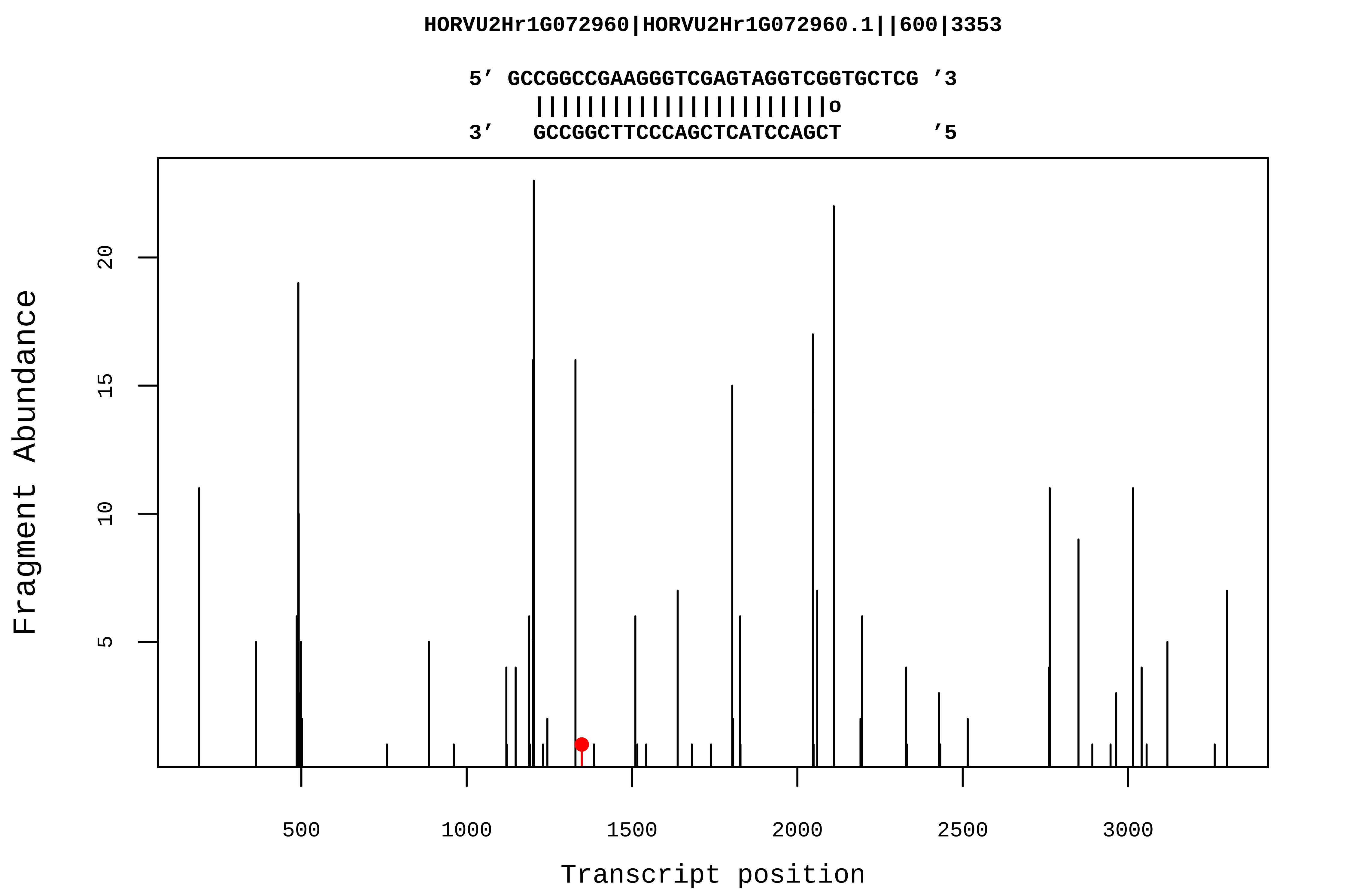

Cleavage site: 1348 Tag abundance: 1 Weighted abundance: 0.143 Category: 4
sRNA abundance: 1 Alignment score: 0.5 MFE ratio: 0.992 p-value: 0.03

HORVU5Hr1G015600|HORVU5Hr1G015600.1||156|510

```
5' GCCGGCCGAAGGGTCGAGTAGGTCGGTGCTCG '3
      ||||||||||||||||||||||o
3'    GCCGGCTTCCCAGCTCATCCAGCT       '5
```

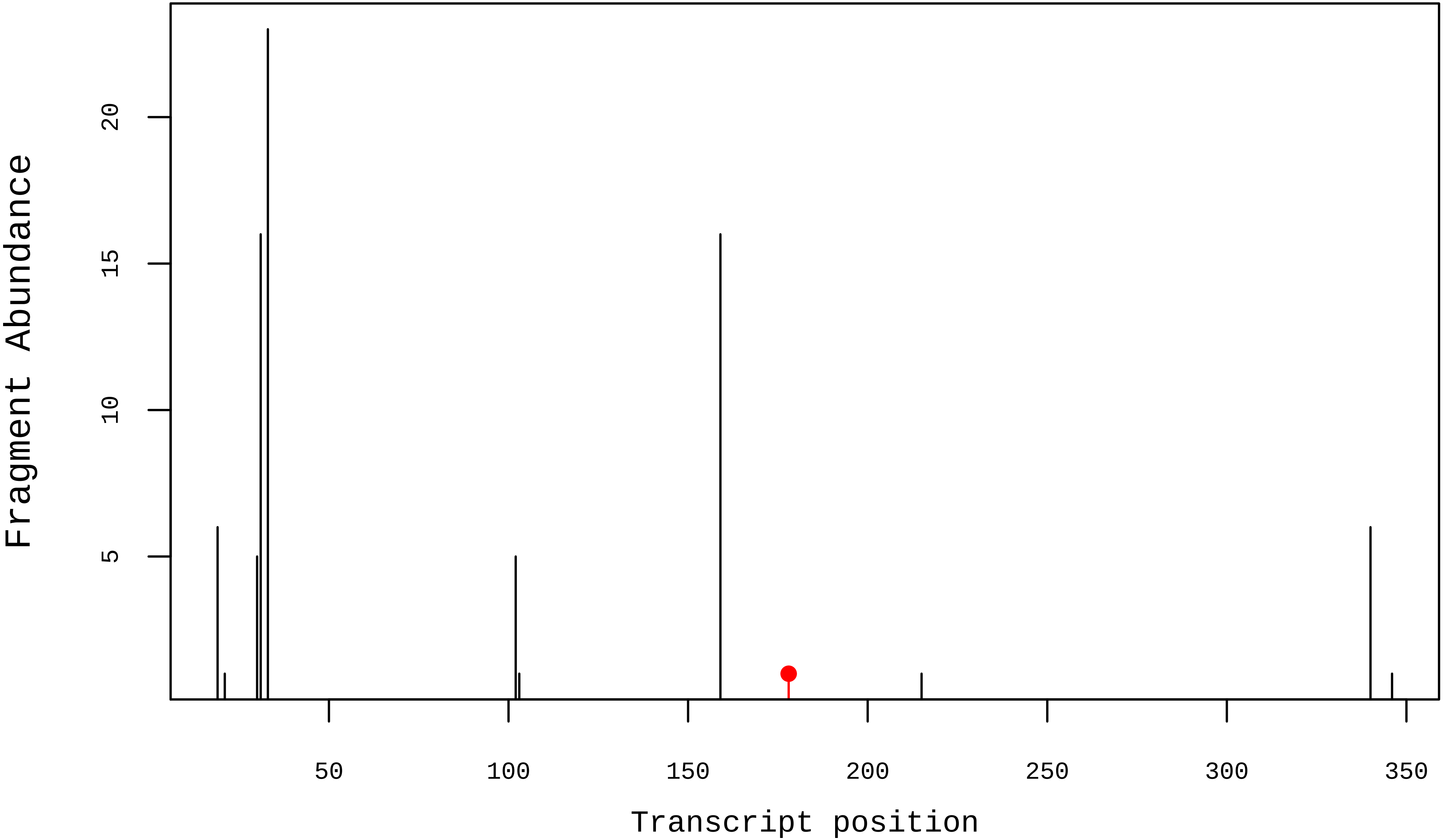

Cleavage site: 178 Tag abundance: 1 Weighted abundance: 0.143 Category: 4
sRNA abundance: 1 Alignment score: 0.5 MFE ratio: 0.992 p-value: 0.05

HORVU5Hr1G015600|HORVU5Hr1G015600.2||231|617

```
5' GCCGGCCGAAGGGTCGAGTAGGTCGGTGCTCG '3
     ||||||||||||||||||||||o
3'   GCCGGCTTCCCAGCTCATCCAGCT       '5
```

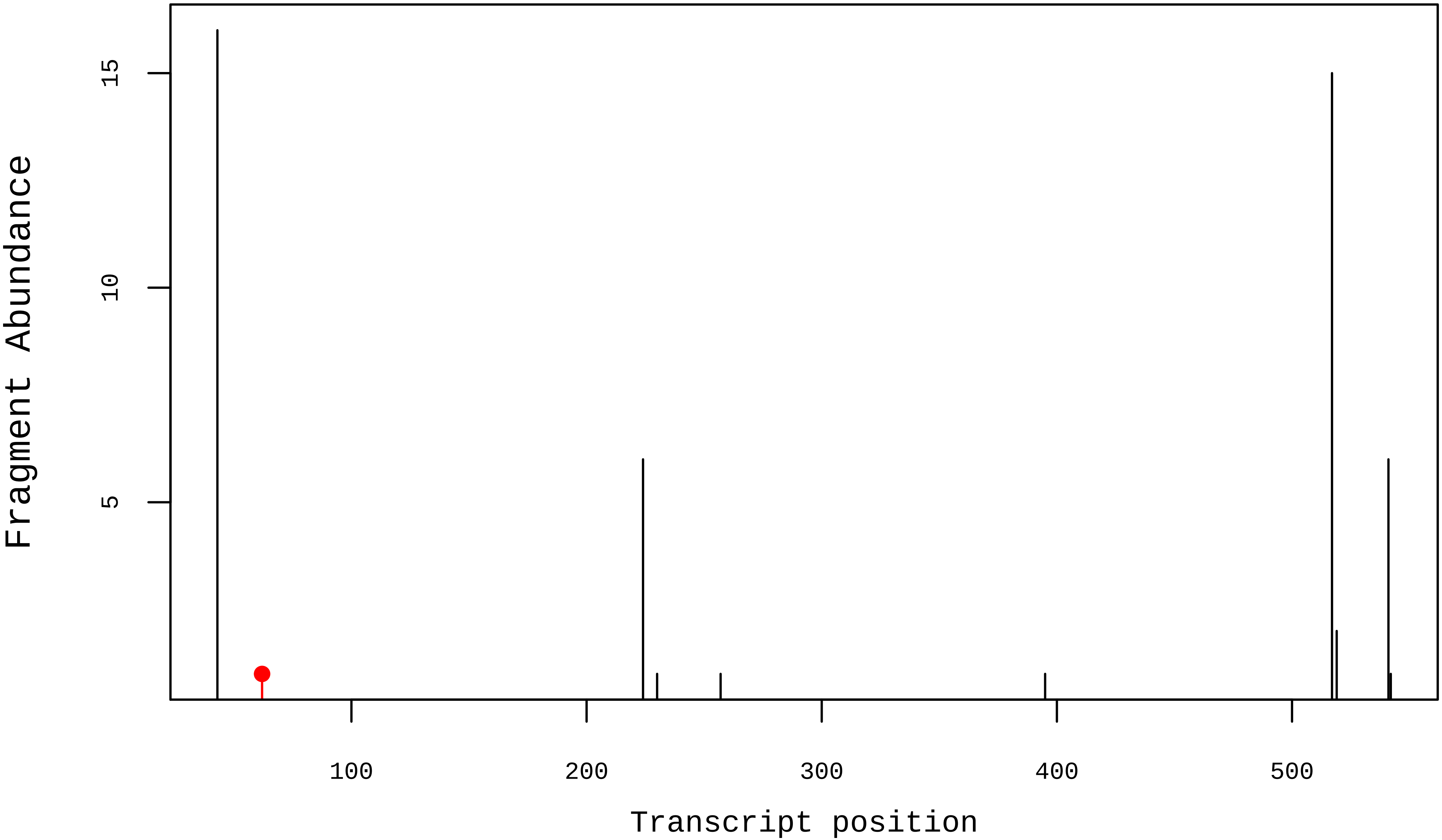

Cleavage site: 62   Tag abundance: 1   Weighted abundance: 0.143   Category: 4
sRNA abundance: 1   Alignment score: 0.5   MFE ratio: 0.992   p-value: 0.041

HORVU5Hr1G015600|HORVU5Hr1G015600.3||276|1709

```
5' GCCGGCCGAAGGGTCGAGTAGGTCGGTGCTCG '3
       ||||||||||||||||||||||o
3'     GCCGGCTTCCCAGCTCATCCAGCT       '5
```

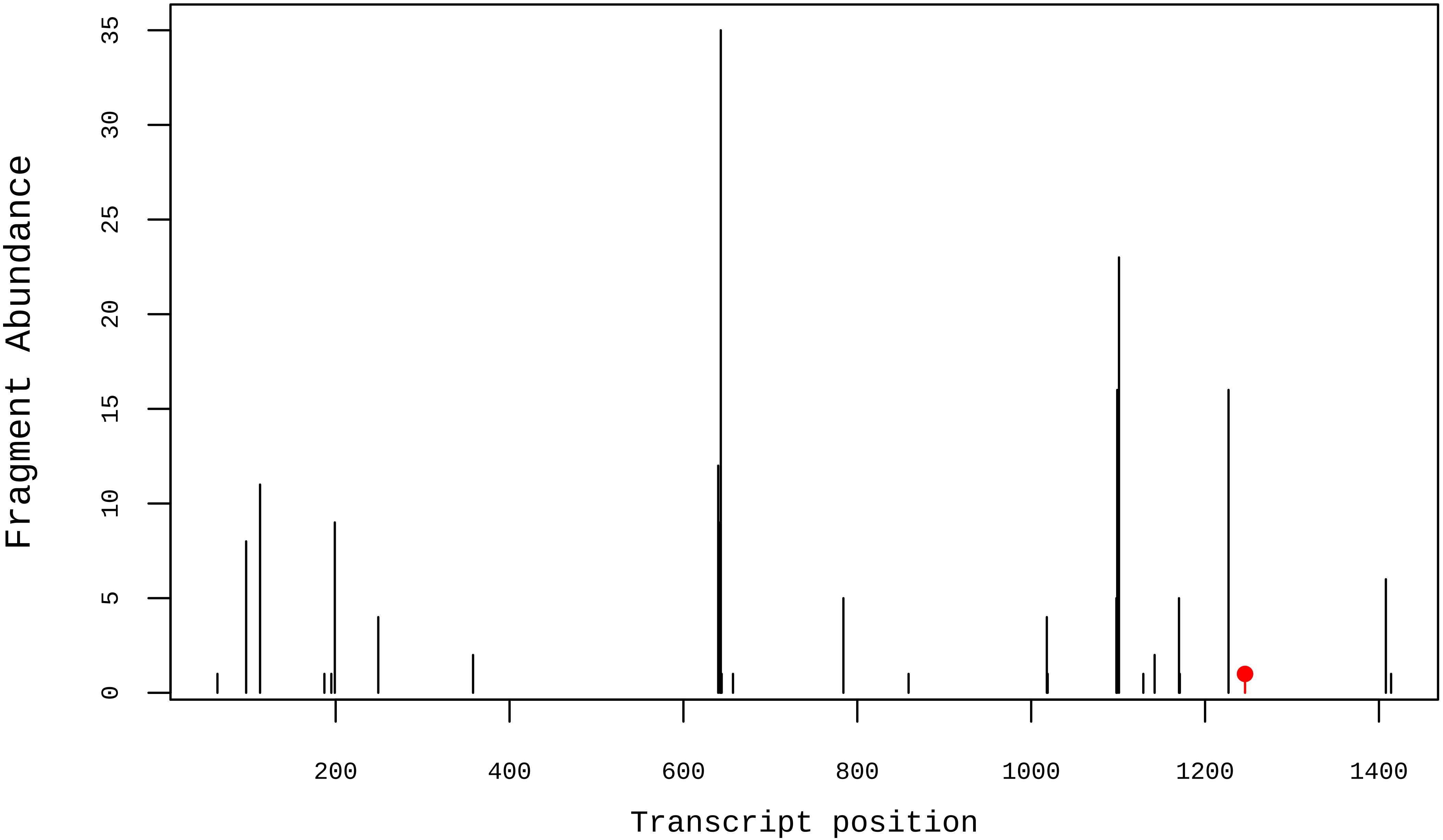

Cleavage site: 1246 Tag abundance: 1 Weighted abundance: 0.143 Category: 4
sRNA abundance: 1 Alignment score: 0.5 MFE ratio: 0.992 p-value: 0.032

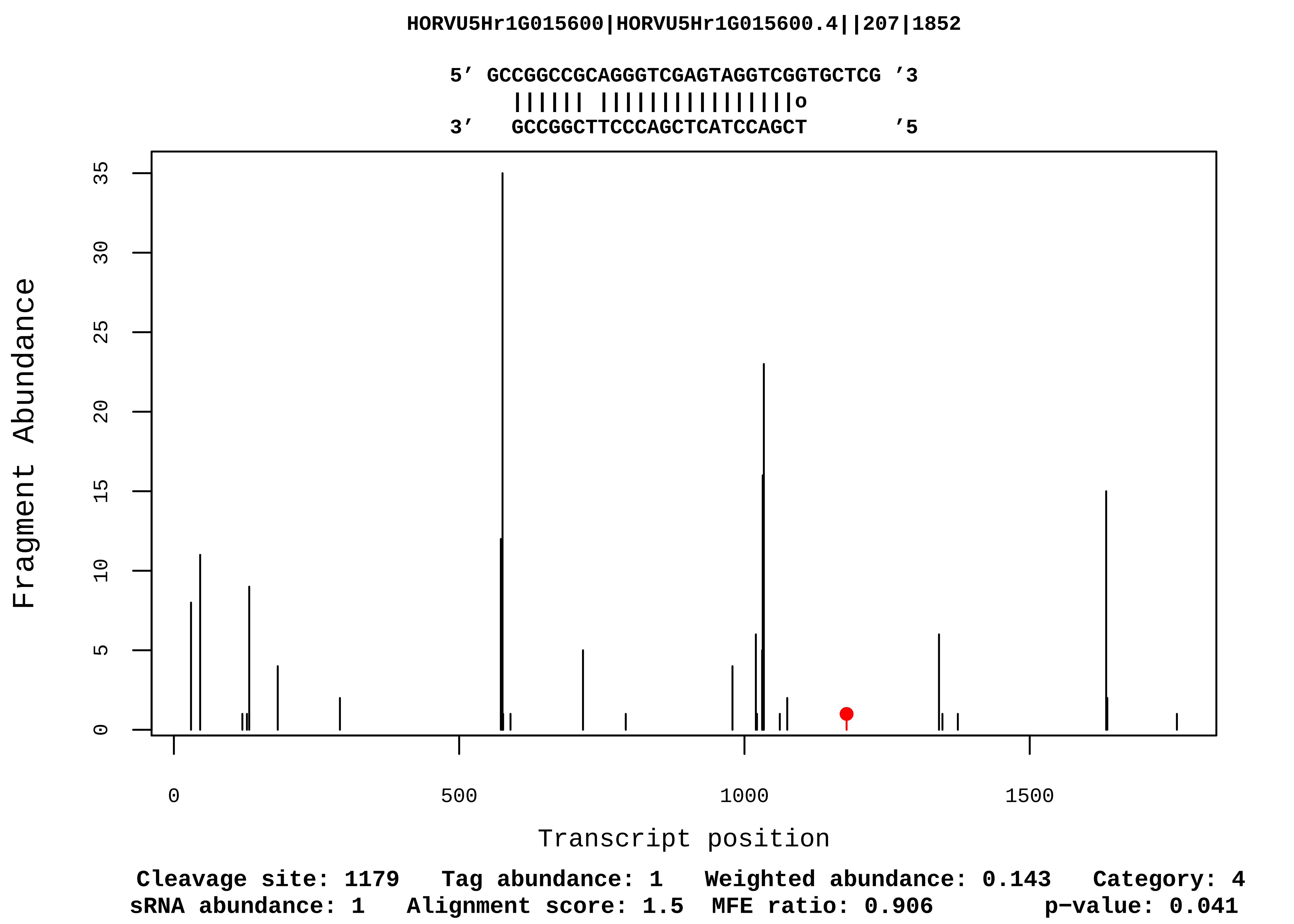
HORVU5Hr1G015600|HORVU5Hr1G015600.4||207|1852
5′ GCCGGCCGCAGGGTCGAGTAGGTCGGTGCTCG ′3
|||||| |||||||||||||||o
3′ GCCGGCTTCCCAGCTCATCCAGCT ′5
Fragment Abundance
0
5
10
15
20
25
30
35
0
500
1000
1500
Transcript position
Cleavage site: 1179 Tag abundance: 1 Weighted abundance: 0.143 Category: 4
sRNA abundance: 1 Alignment score: 1.5 MFE ratio: 0.906 p-value: 0.041

HORVU0Hr1G003660|HORVU0Hr1G003660.1||297|1955

```
5' AGGCGGACCGGCCGACCCGGCCCAAGGTCCAA '3
     ||||||||||||||||||||||||||
3'  CCGCCTGGCCGGCTGGGCCGGGTTC       '5
```

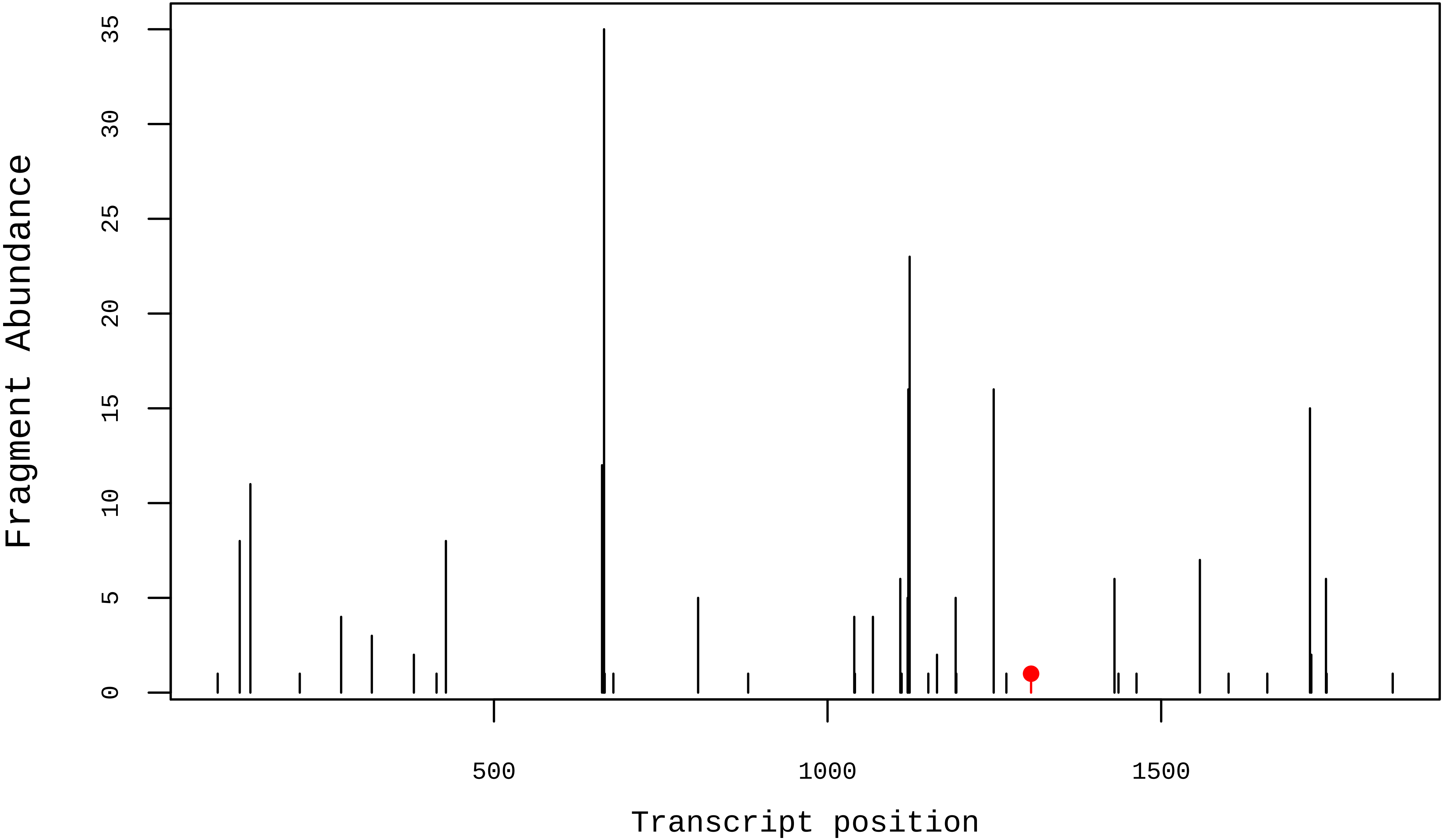

Cleavage site: 1305   Tag abundance: 1   Weighted abundance: 0.25   Category: 4
sRNA abundance: 1   Alignment score: 0   MFE ratio: 1   p-value: 0.009

HORVU0Hr1G023930|HORVU0Hr1G023930.3||396|2180

```
5' AGGCGGACCGGCTGACCCGGCCCAAGGTCCAA '3
     |||||||||||o||||||||||||
3'   CCGCCTGGCCGGCTGGGCCGGGTTC       '5
```

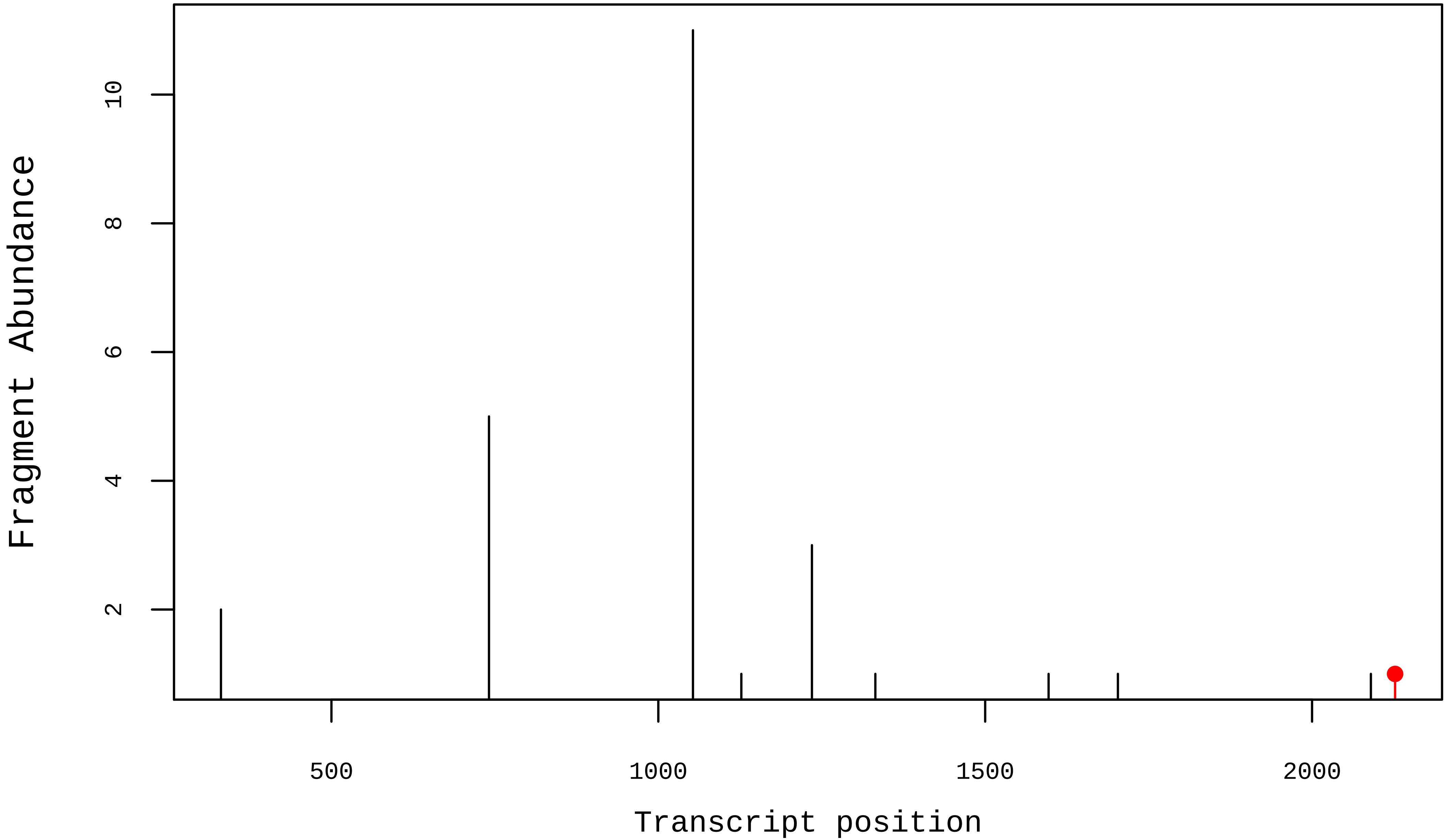

Cleavage site: 2127 Tag abundance: 1 Weighted abundance: 0.25 Category: 4
sRNA abundance: 1 Alignment score: 0.5 MFE ratio: 0.965 p-value: 0.006

HORVU2Hr1G072960|HORVU2Hr1G072960.1||600|3353

```
5' AGGCCGACCGGCCGACCCGGCCCAAGGTCCAA '3
     ||| ||||||||||||||||||||||
3'   CCGCCTGGCCGGCTGGGCCGGGTTC       '5
```

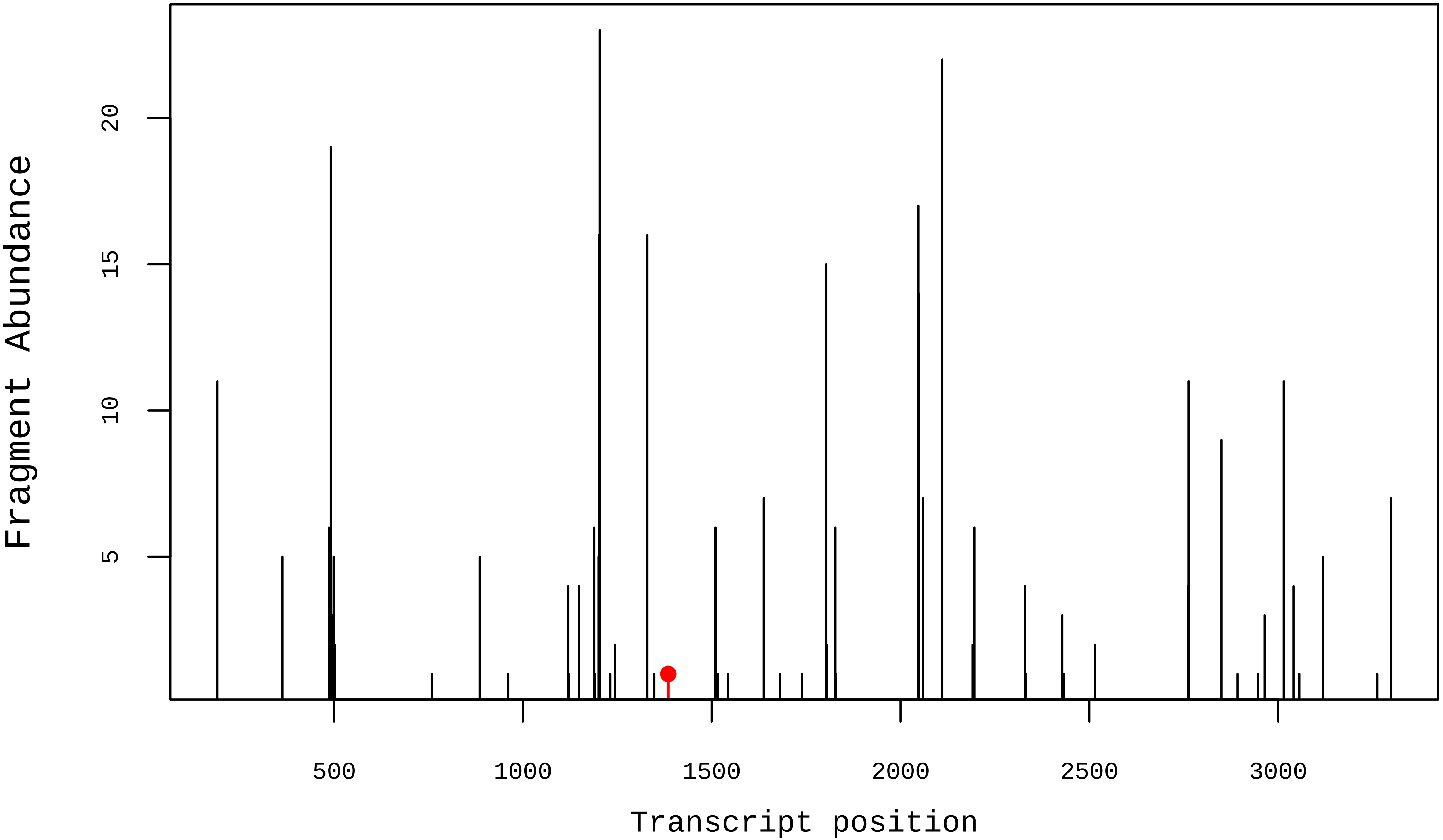

Cleavage site: 1385   Tag abundance: 1   Weighted abundance: 0.25   Category: 4
sRNA abundance: 1   Alignment score: 1   MFE ratio: 0.902   p-value: 0.024

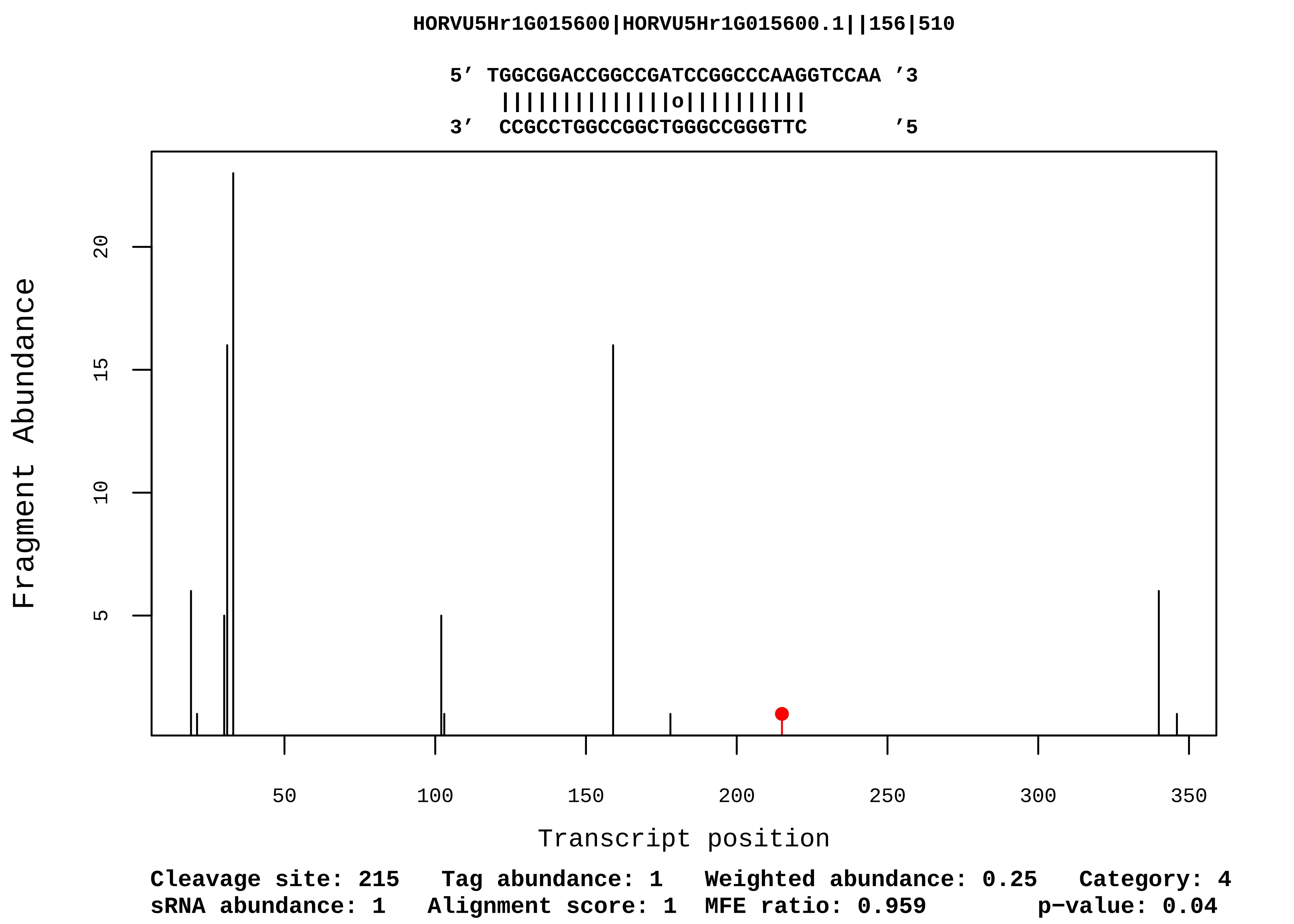
HORVU5Hr1G015600|HORVU5Hr1G015600.1||156|510
5′ TGGCGGACCGGCCGATCCGGCCCAAGGTCCAA ′3
||||||||||||||o||||||||||
3′ CCGCCTGGCCGGCTGGGCCGGGTTC ′5
Fragment Abundance
5
10
15
20
50
100
150
200
250
300
350
Transcript position
Cleavage site: 215 Tag abundance: 1 Weighted abundance: 0.25 Category: 4
sRNA abundance: 1 Alignment score: 1 MFE ratio: 0.959 p-value: 0.04

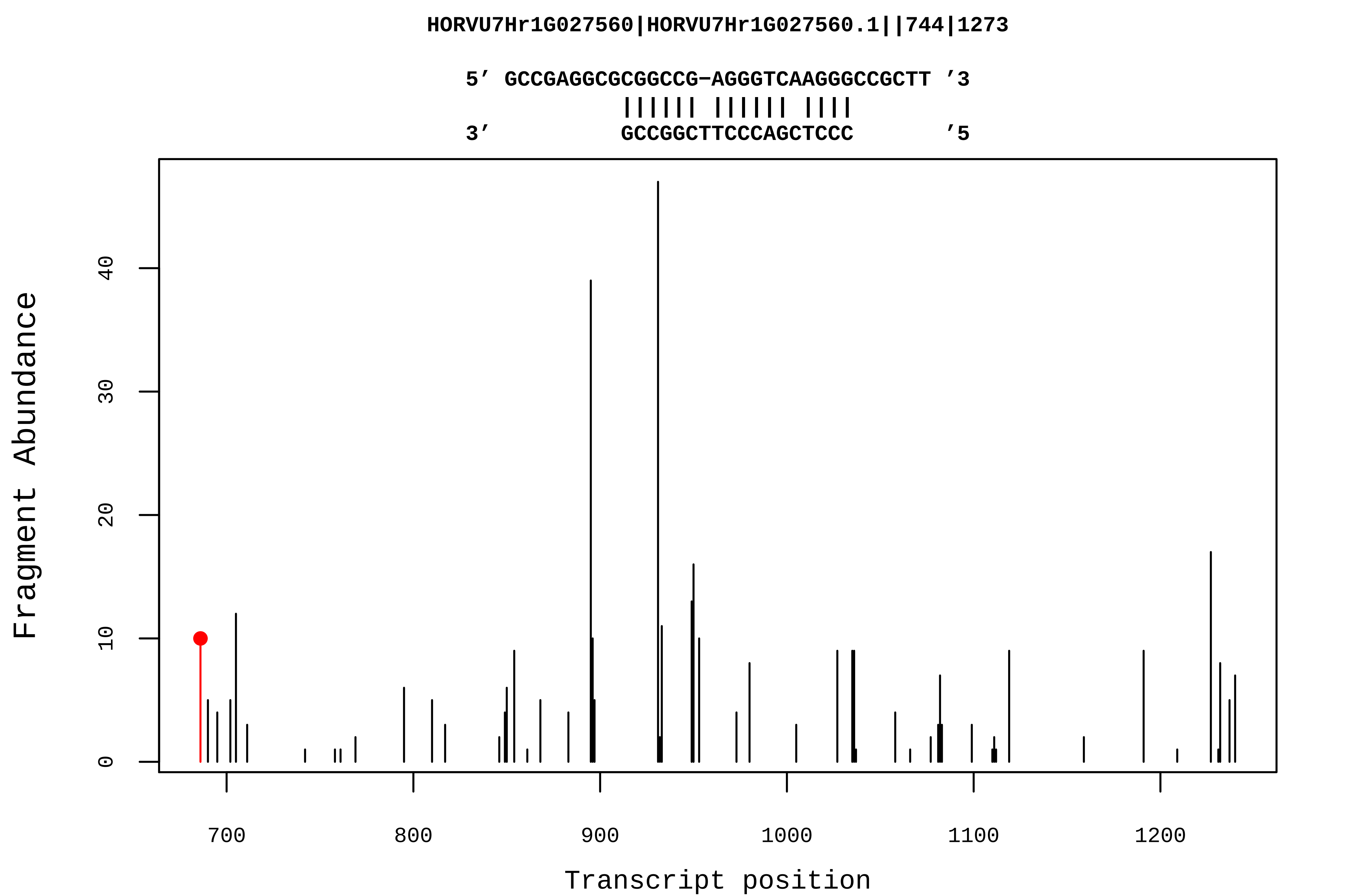

Cleavage site: 686 Tag abundance: 10 Weighted abundance: 10 Category: 2
sRNA abundance: 1 Alignment score: 4 MFE ratio: 0.743 p-value: 0.024

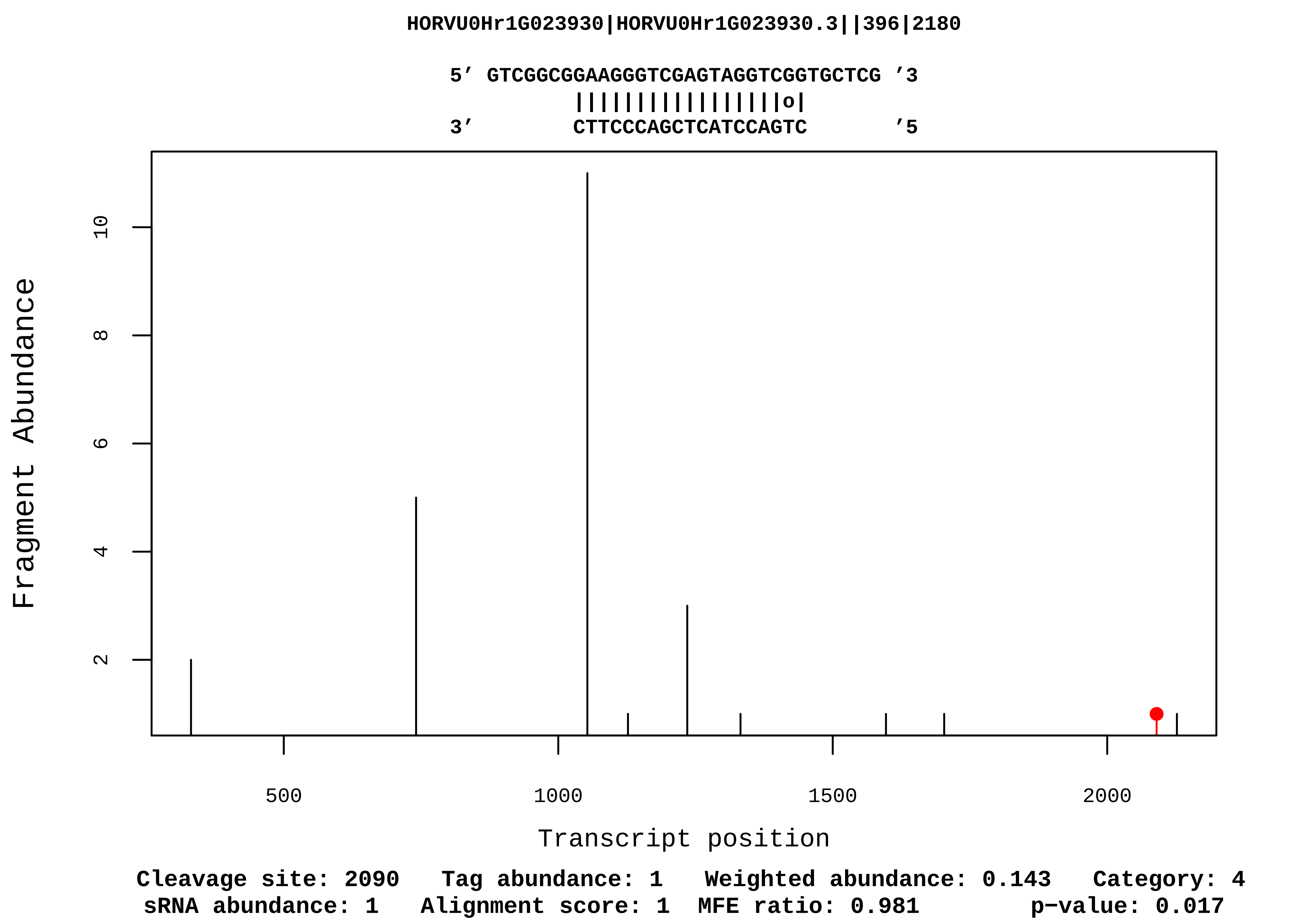
HORVU0Hr1G023930|HORVU0Hr1G023930.3||396|2180
5′ GTCGGCGGAAGGGTCGAGTAGGTCGGTGCTCG ′3
|||||||||||||||||o|
3′ CTTCCCAGCTCATCCAGTC ′5
Fragment Abundance
2
4
6
8
10
500
1000
1500
2000
Transcript position
Cleavage site: 2090 Tag abundance: 1 Weighted abundance: 0.143 Category: 4
sRNA abundance: 1 Alignment score: 1 MFE ratio: 0.981 p-value: 0.017

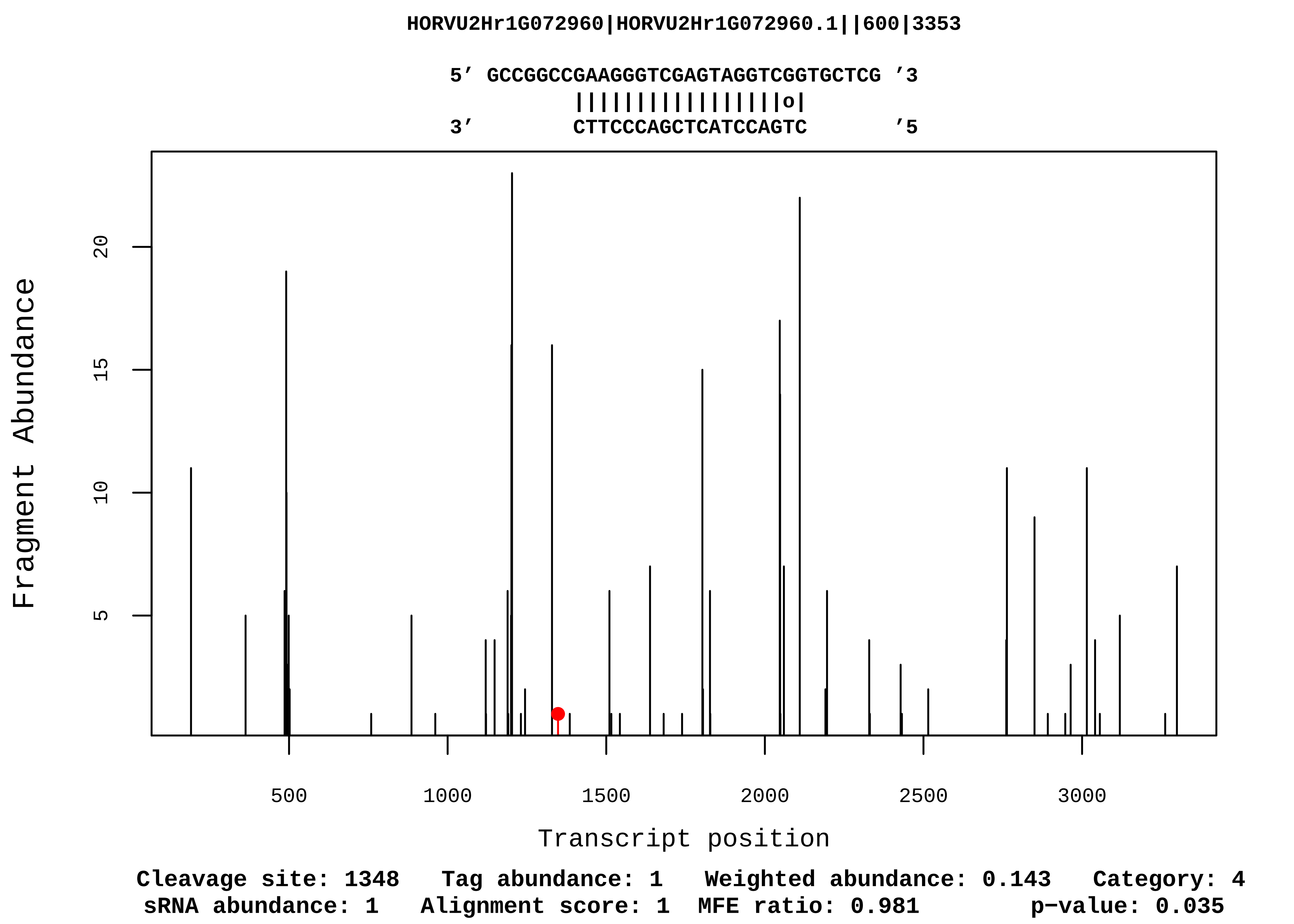
HORVU2Hr1G072960|HORVU2Hr1G072960.1||600|3353
5' GCCGGCCGAAGGGTCGAGTAGGTCGGTGCTCG '3
||||||||||||||||o|
3' CTTCCCAGCTCATCCAGTC '5
Fragment Abundance
5
10
15
20
500
1000
1500
2000
2500
3000
Transcript position
Cleavage site: 1348 Tag abundance: 1 Weighted abundance: 0.143 Category: 4
sRNA abundance: 1 Alignment score: 1 MFE ratio: 0.981 p-value: 0.035

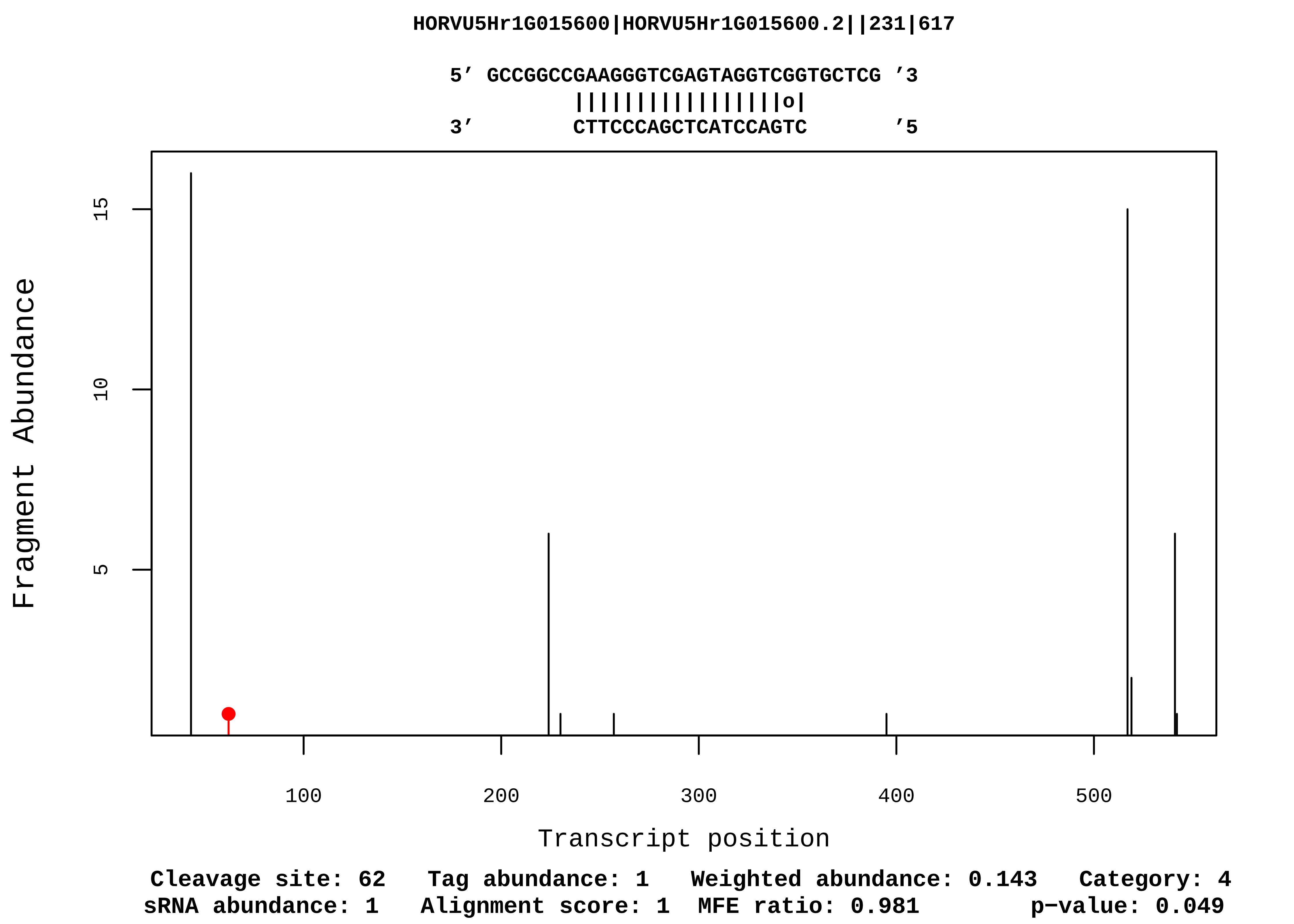
HORVU5Hr1G015600|HORVU5Hr1G015600.2||231|617
5' GCCGGCCGAAGGGTCGAGTAGGTCGGTGCTCG '3
||||||||||||||||o|
3' CTTCCCAGCTCATCCAGTC '5
Fragment Abundance
5
10
15
100
200
300
400
500
Transcript position
Cleavage site: 62 Tag abundance: 1 Weighted abundance: 0.143 Category: 4
sRNA abundance: 1 Alignment score: 1 MFE ratio: 0.981 p-value: 0.049

HORVU5Hr1G015600|HORVU5Hr1G015600.3||276|1709

```
5’ GCCGGCCGAAGGGTCGAGTAGGTCGGTGCTCG ’3
           |||||||||||||||||o|
3’         CTTCCCAGCTCATCCAGTC        ’5
```

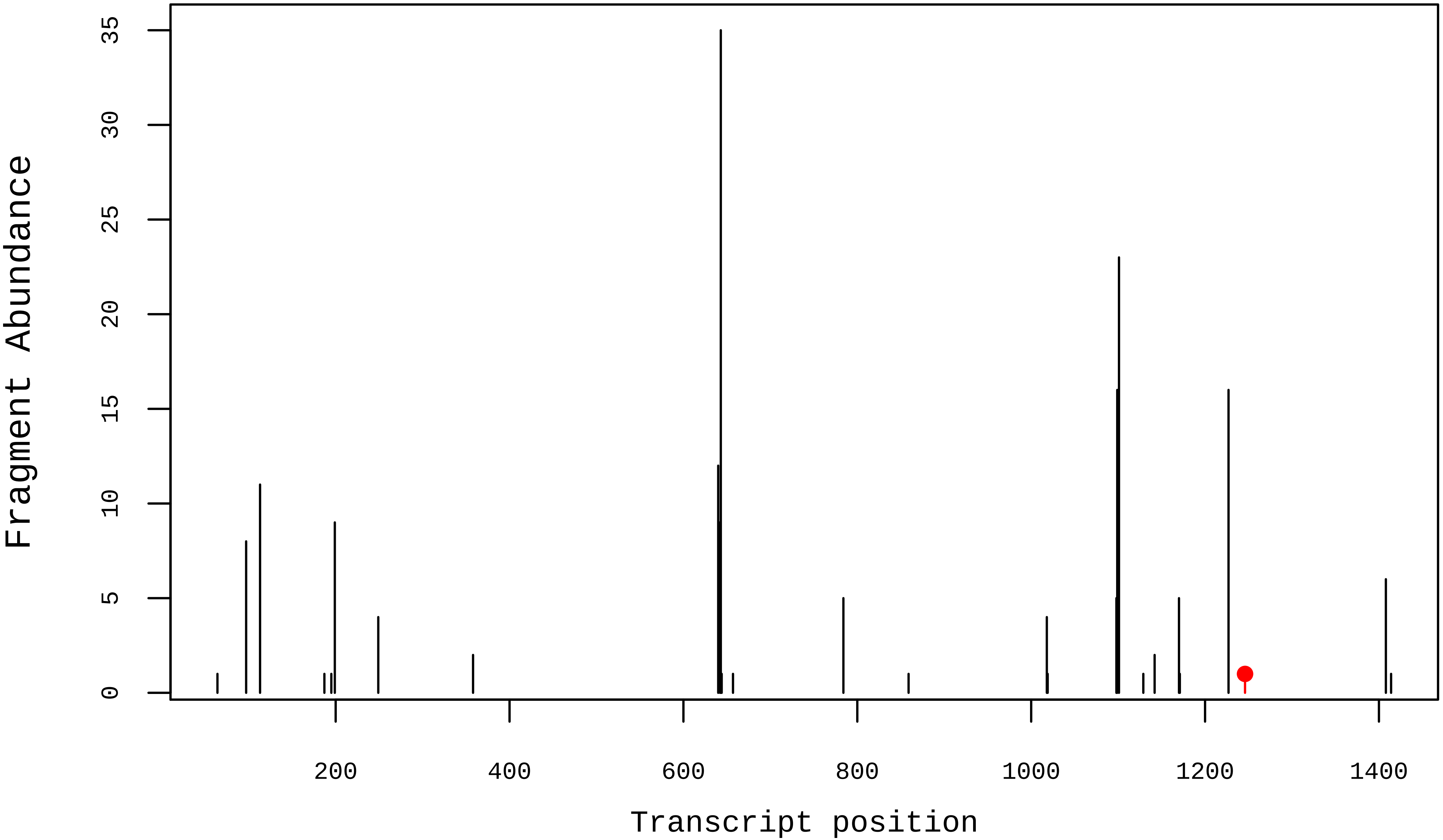

Cleavage site: 1246 Tag abundance: 1 Weighted abundance: 0.143 Category: 4
sRNA abundance: 1 Alignment score: 1 MFE ratio: 0.981 p-value: 0.038

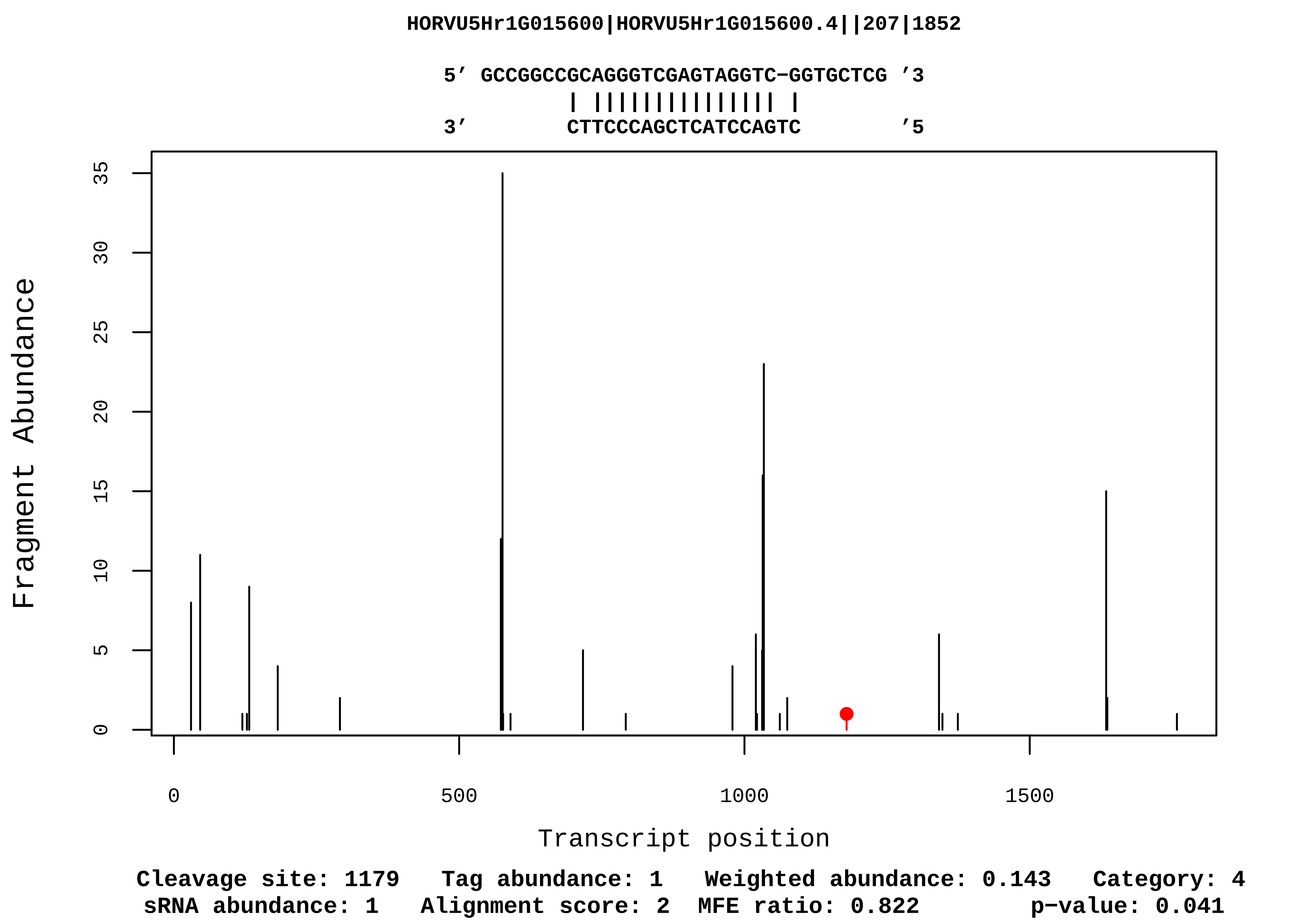

HORVU5Hr1G015600|HORVU5Hr1G015600.4||207|1852
5' GCCGGCCGCAGGGTCGAGTAGGTC-GGTGCTCG '3
3' CTTCCCAGCTCATCCAGTC '5
Fragment Abundance
0
5
10
15
20
25
30
35
0
500
1000
1500
Transcript position
Cleavage site: 1179 Tag abundance: 1 Weighted abundance: 0.143 Category: 4
sRNA abundance: 1 Alignment score: 2 MFE ratio: 0.822 p-value: 0.041

HORVU4Hr1G083870|HORVU4Hr1G083870.1||1746|3479

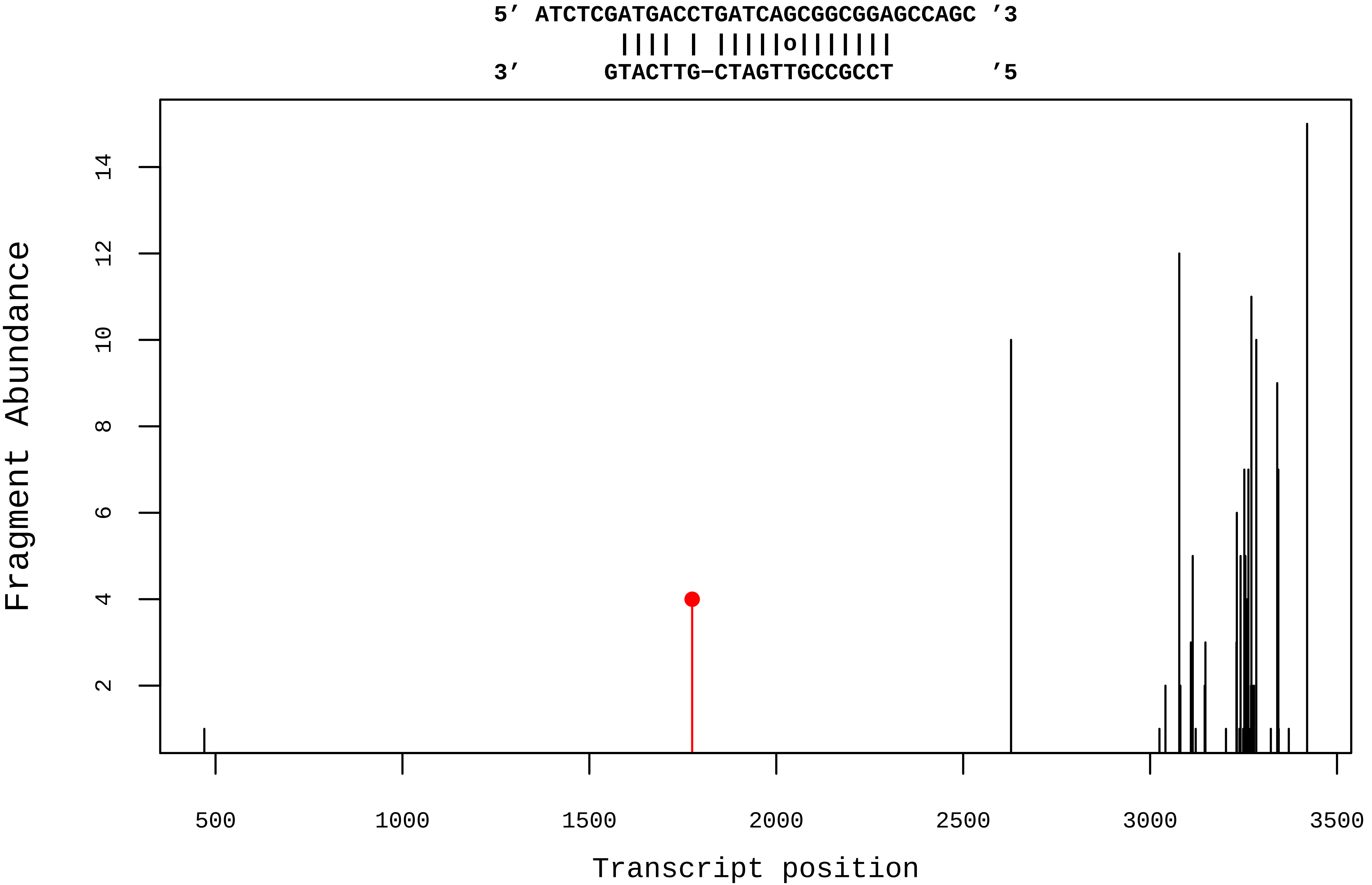

Cleavage site: 1775 Tag abundance: 4 Weighted abundance: 0.8 Category: 3
sRNA abundance: 1 Alignment score: 4 MFE ratio: 0.767 p-value: 0.023

HORVU4Hr1G083870|HORVU4Hr1G083870.3||1746|3378

```
5' ATCTCGATGACCTGATCAGCGGCGGAGCCAGC '3
          |||| | |||||o|||||||
3'      GTACTTG-CTAGTTGCCGCCT       '5
```

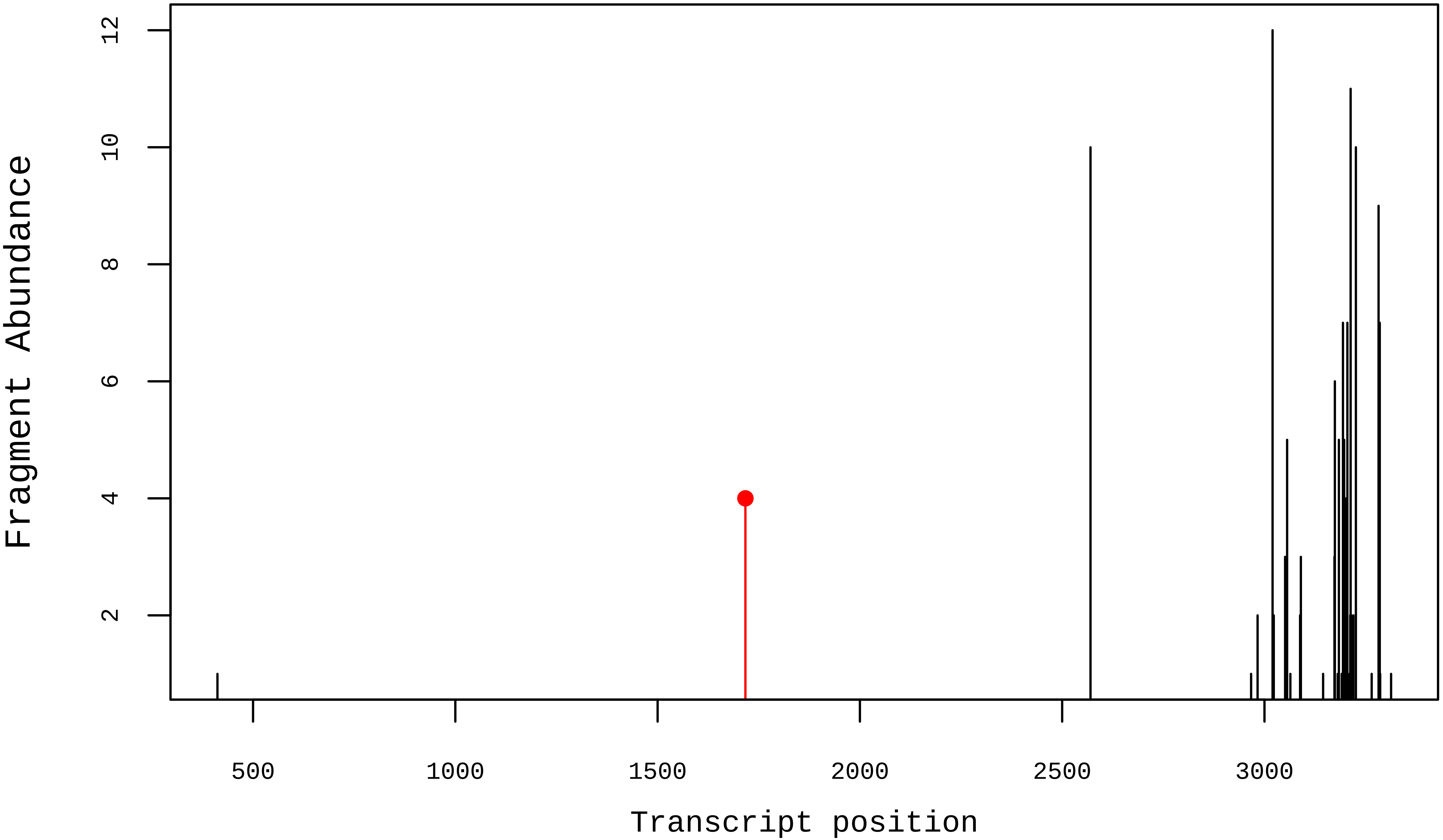

Cleavage site: 1717 Tag abundance: 4 Weighted abundance: 0.8 Category: 3
sRNA abundance: 1 Alignment score: 4 MFE ratio: 0.767 p-value: 0.024

HORVU4Hr1G083870|HORVU4Hr1G083870.4||1746|3334

```
5' ATCTCGATGACCTGATCAGCGGCGGAGCCAGC '3
           |||| | |||||o|||||||
3'      GTACTTG-CTAGTTGCCGCCT       '5
```

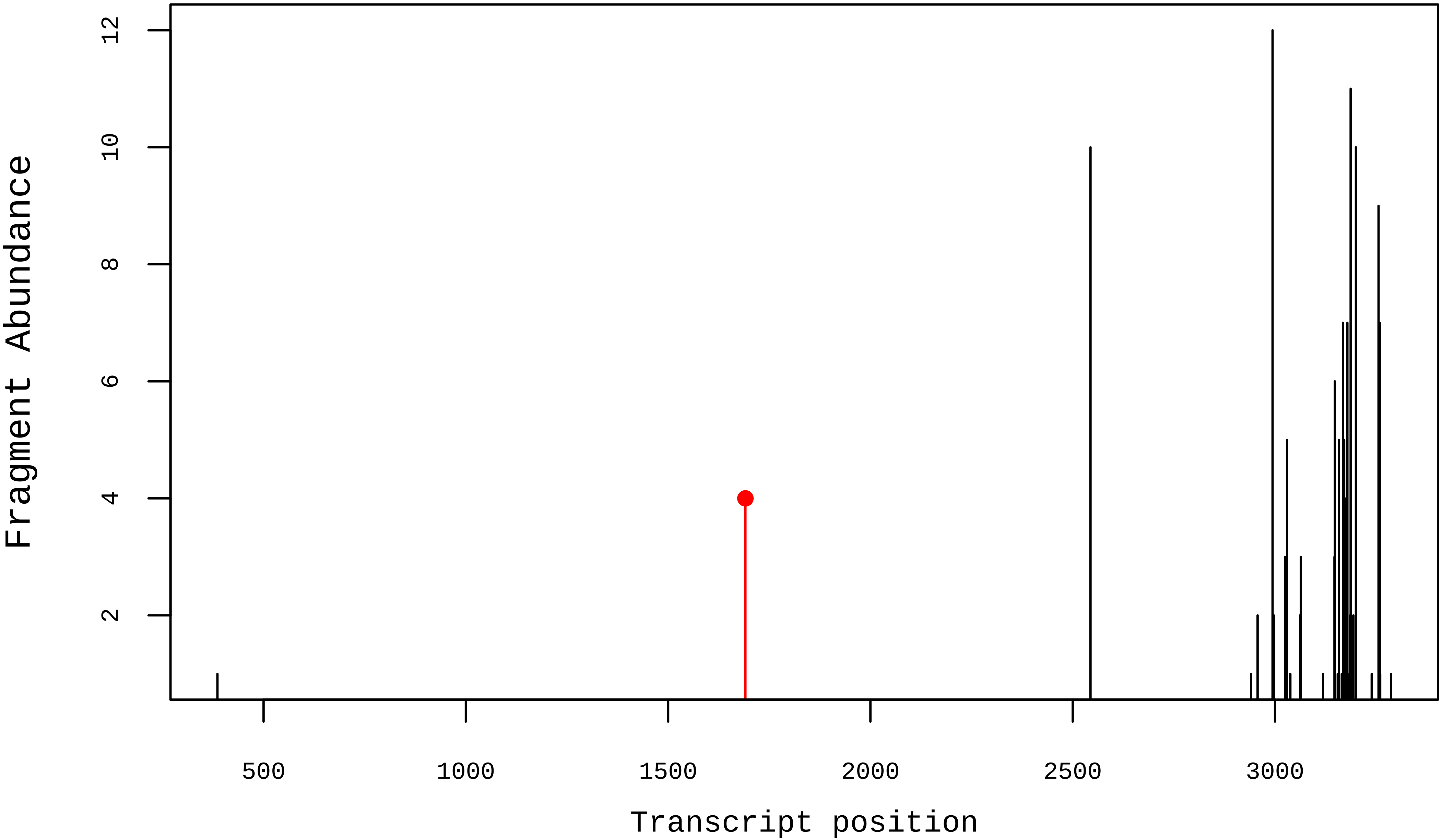

Cleavage site: 1691   Tag abundance: 4   Weighted abundance: 0.8   Category: 3
sRNA abundance: 1   Alignment score: 4   MFE ratio: 0.767   p-value: 0.024

HORVU4Hr1G083870|HORVU4Hr1G083870.6||1452|1868

```
5' ATCTCGATGACCTGATCAGCGGCGGAGCCAGC '3
         |||| | |||||o|||||||
3'       GTACTTG-CTAGTTGCCGCCT       '5
```

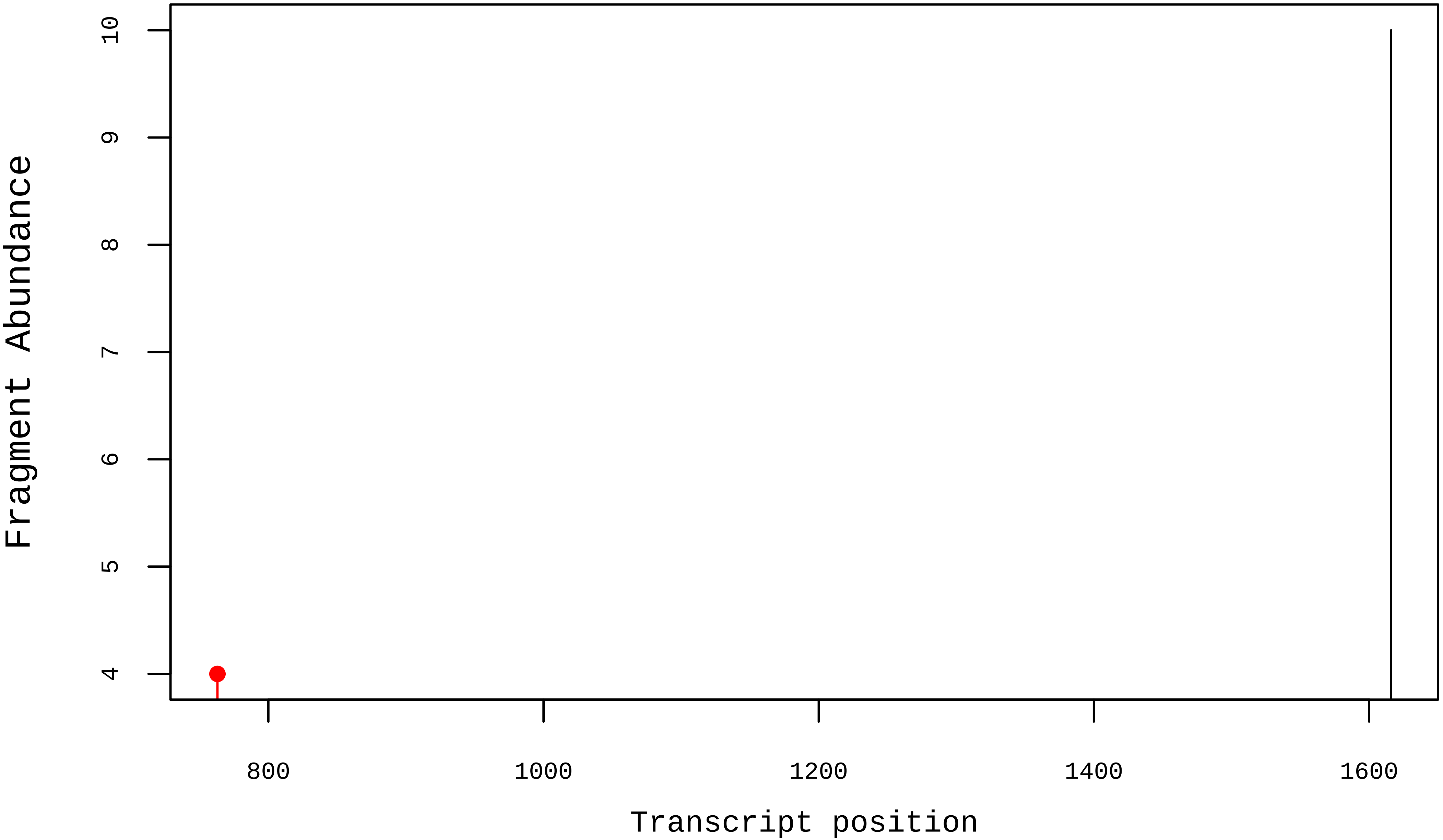

Cleavage site: 763   Tag abundance: 4   Weighted abundance: 0.8   Category: 3
sRNA abundance: 1   Alignment score: 4   MFE ratio: 0.767   p-value: 0.003

HORVU4Hr1G083870|HORVU4Hr1G083870.7||1440|1867

```
5' ATCTCGATGACCTGATCAGCGGCGGAGCCAGC '3
          |||| | |||||o|||||||
3'      GTACTTG-CTAGTTGCCGCCT       '5
```

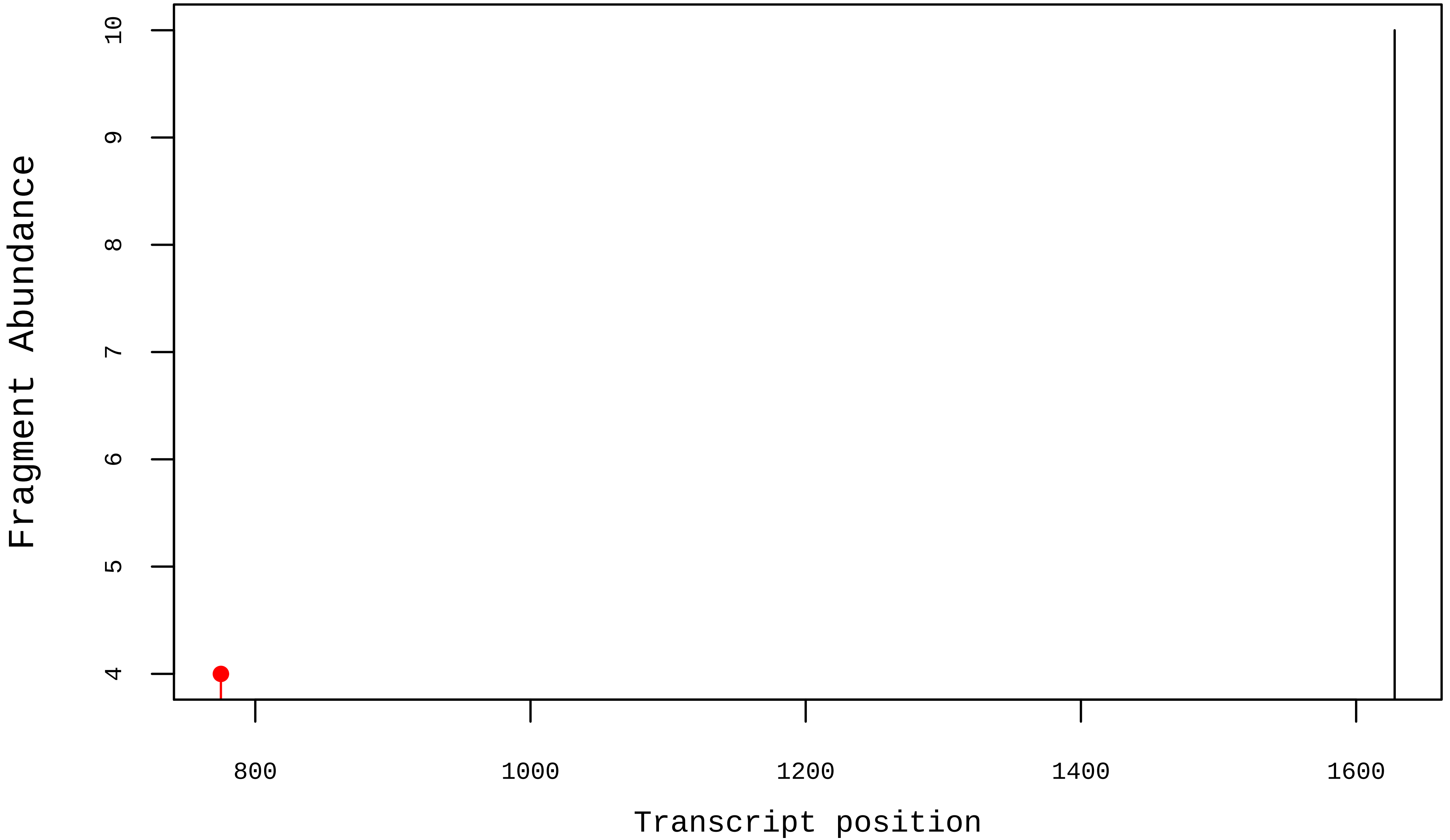

Cleavage site: 775   Tag abundance: 4   Weighted abundance: 0.8   Category: 3
sRNA abundance: 1   Alignment score: 4   MFE ratio: 0.767   p-value: 0.003

HORVU0Hr1G023930|HORVU0Hr1G023930.3||396|2180

```
5' GTCGGCGGAAGGGTCGAGTAGGTCGGTGCTCG '3
         |||  ||||||||||||||||
3'       GCCGGCCCAGCTCATCCAGCC       '5
```

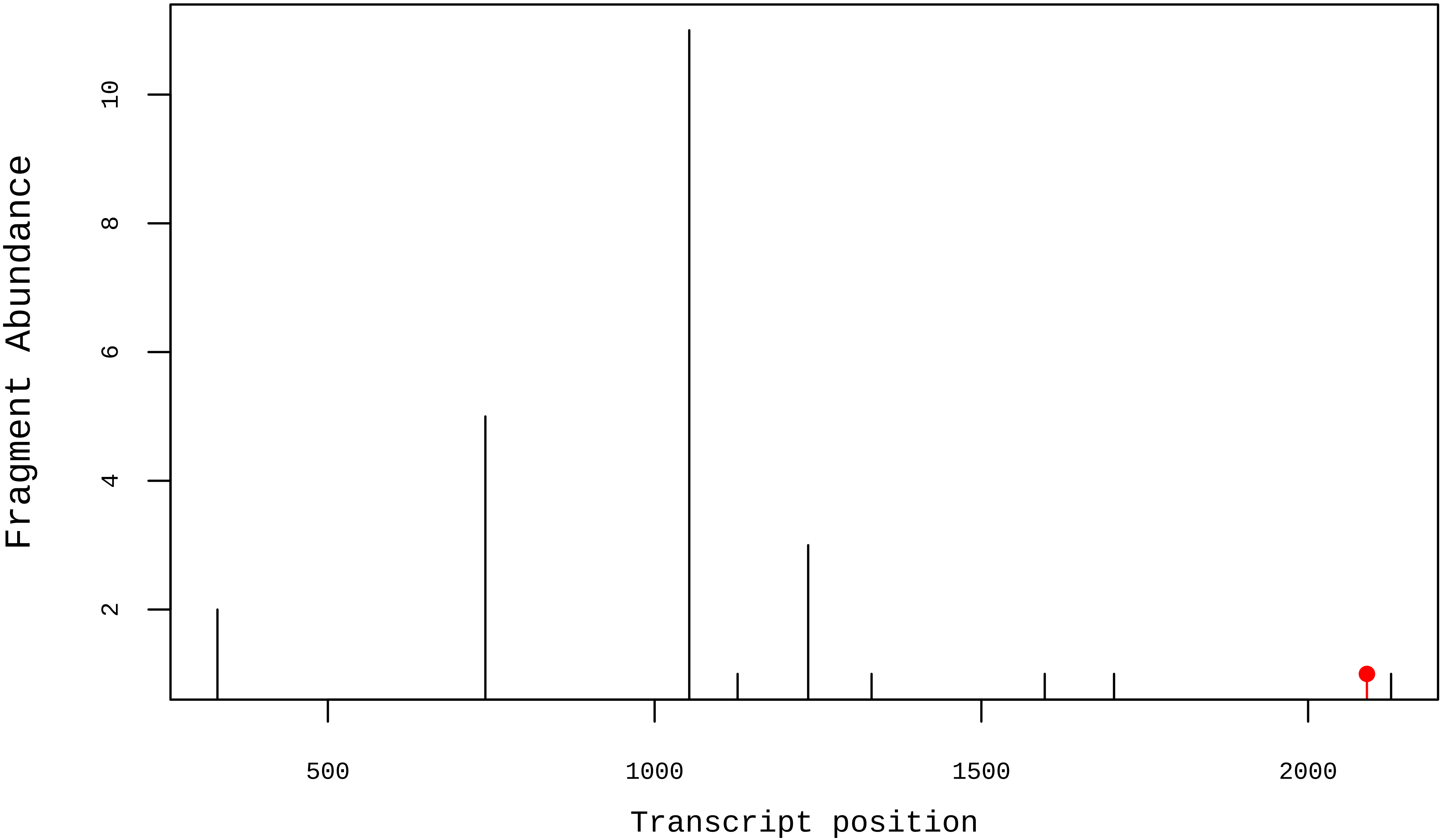

Cleavage site: 2090 Tag abundance: 1 Weighted abundance: 0.143 Category: 4
sRNA abundance: 1 Alignment score: 2 MFE ratio: 0.821 p-value: 0.006

HORVU2Hr1G072960|HORVU2Hr1G072960.1||600|3353

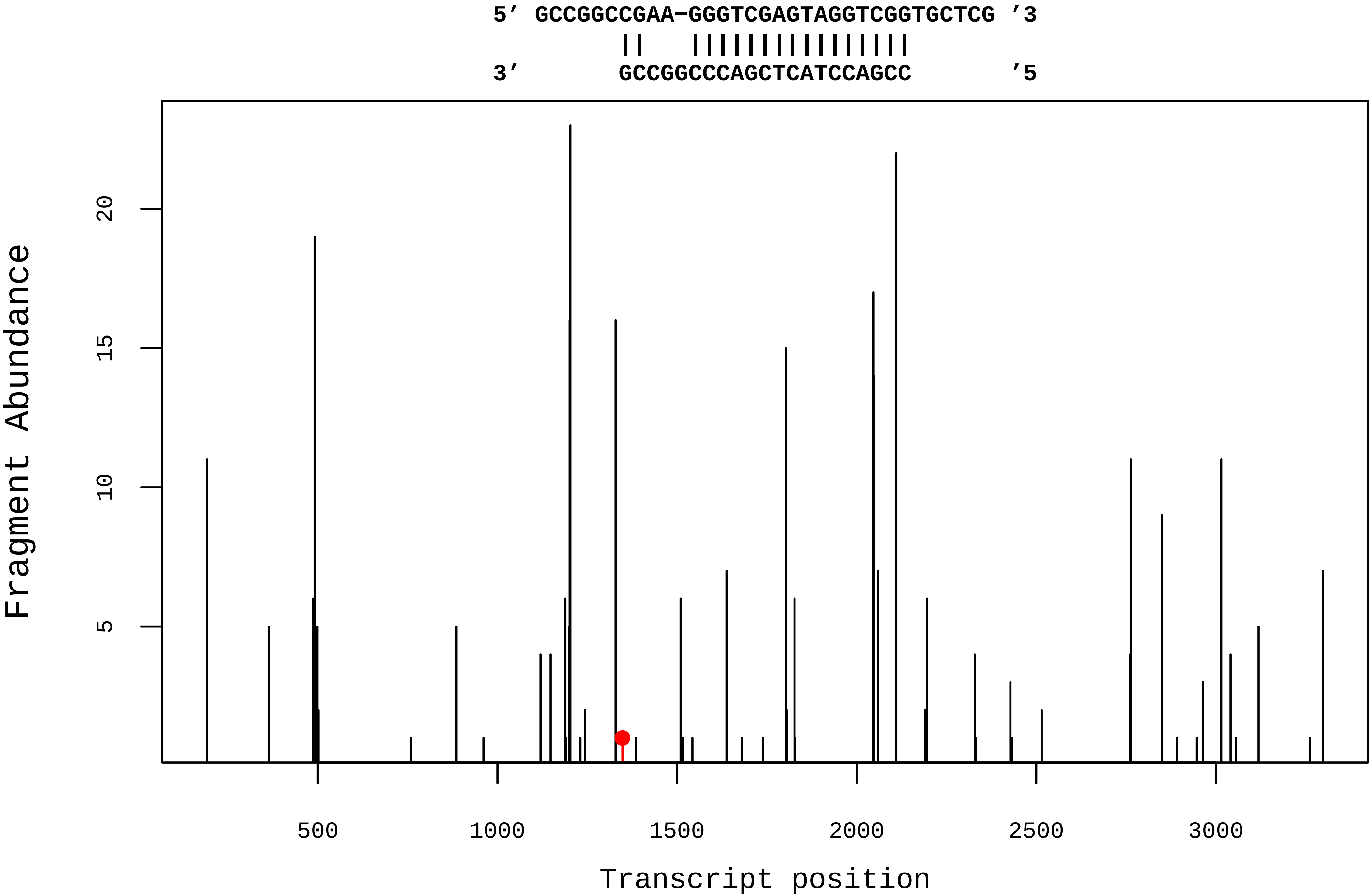

Cleavage site: 1348 Tag abundance: 1 Weighted abundance: 0.143 Category: 4
sRNA abundance: 1 Alignment score: 3 MFE ratio: 0.721 p-value: 0.041

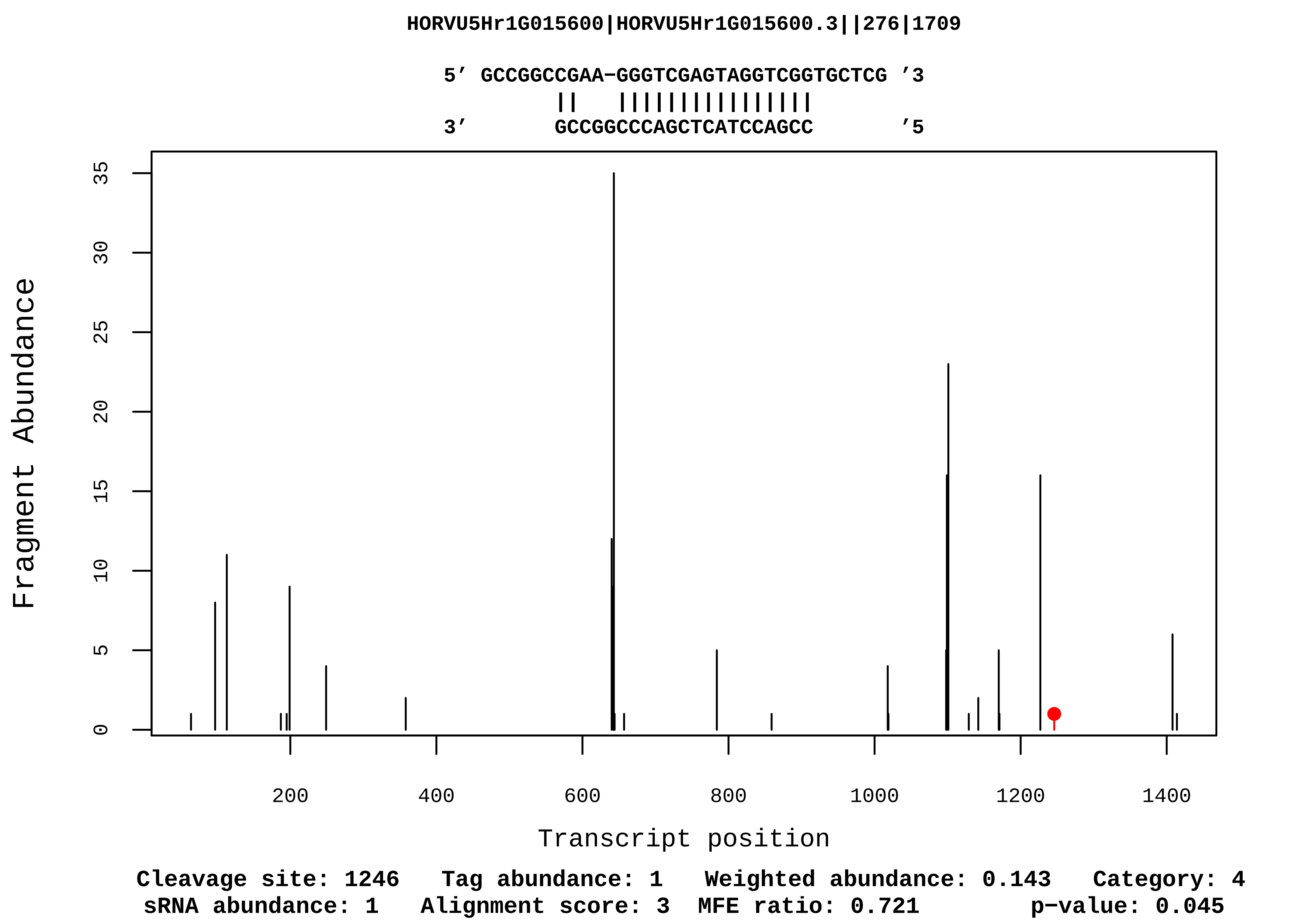

HORVU5Hr1G015600|HORVU5Hr1G015600.3||276|1709
5' GCCGGCCGAA-GGGTCGAGTAGGTCGGTGCTCG '3
|| ||||||||||||||||
3' GCCGGCCCAGCTCATCCAGCC '5
Fragment Abundance
0
5
10
15
20
25
30
35
200
400
600
800
1000
1200
1400
Transcript position
Cleavage site: 1246 Tag abundance: 1 Weighted abundance: 0.143 Category: 4
sRNA abundance: 1 Alignment score: 3 MFE ratio: 0.721 p-value: 0.045

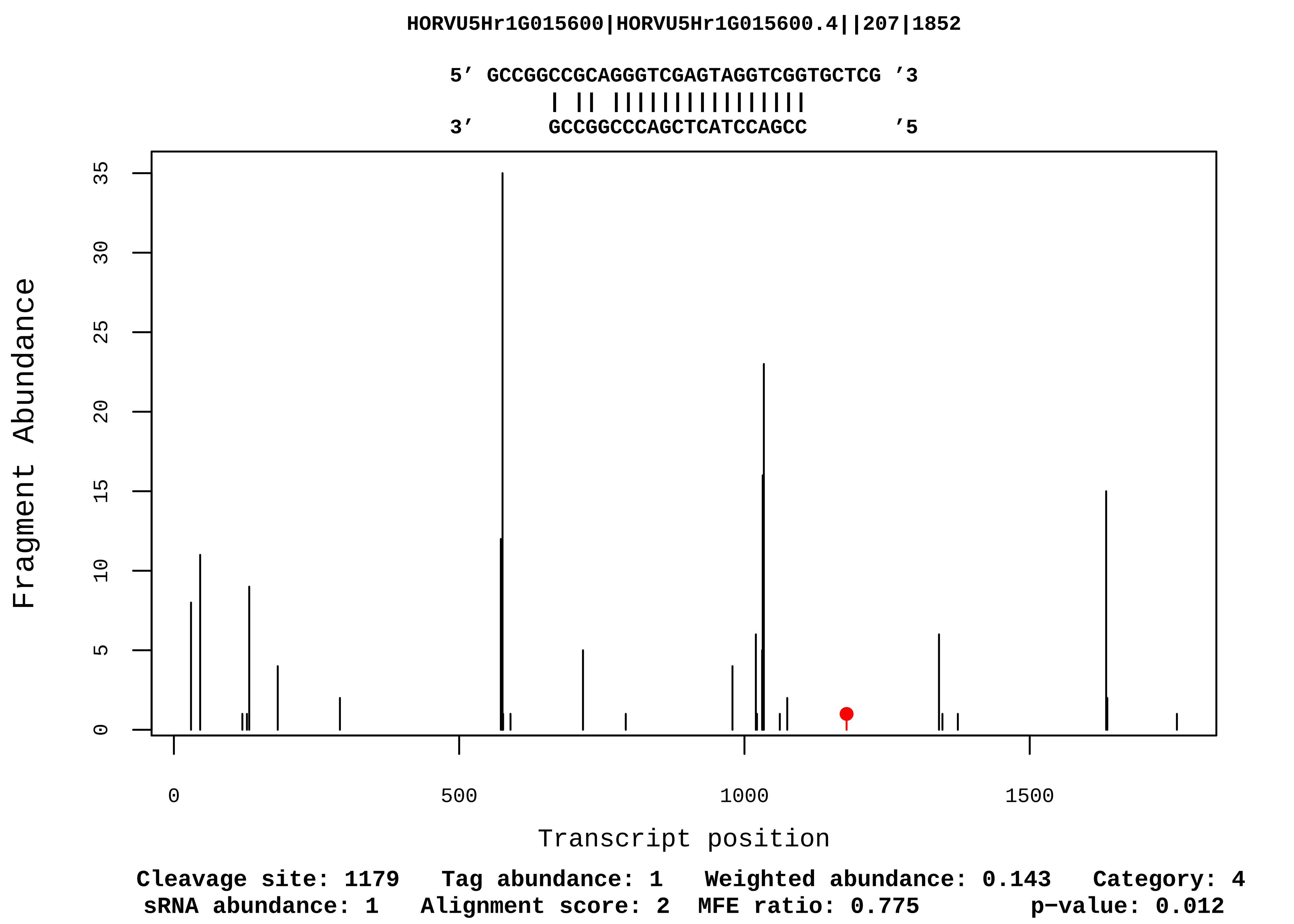

HORVU5Hr1G015600|HORVU5Hr1G015600.4||207|1852
5' GCCGGCCGCAGGGTCGAGTAGGTCGGTGCTCG '3
| || ||||||||||||||||
3' GCCGGCCCAGCTCATCCAGCC '5
Fragment Abundance
0
5
10
15
20
25
30
35
0
500
1000
1500
Transcript position
Cleavage site: 1179 Tag abundance: 1 Weighted abundance: 0.143 Category: 4
sRNA abundance: 1 Alignment score: 2 MFE ratio: 0.775 p-value: 0.012

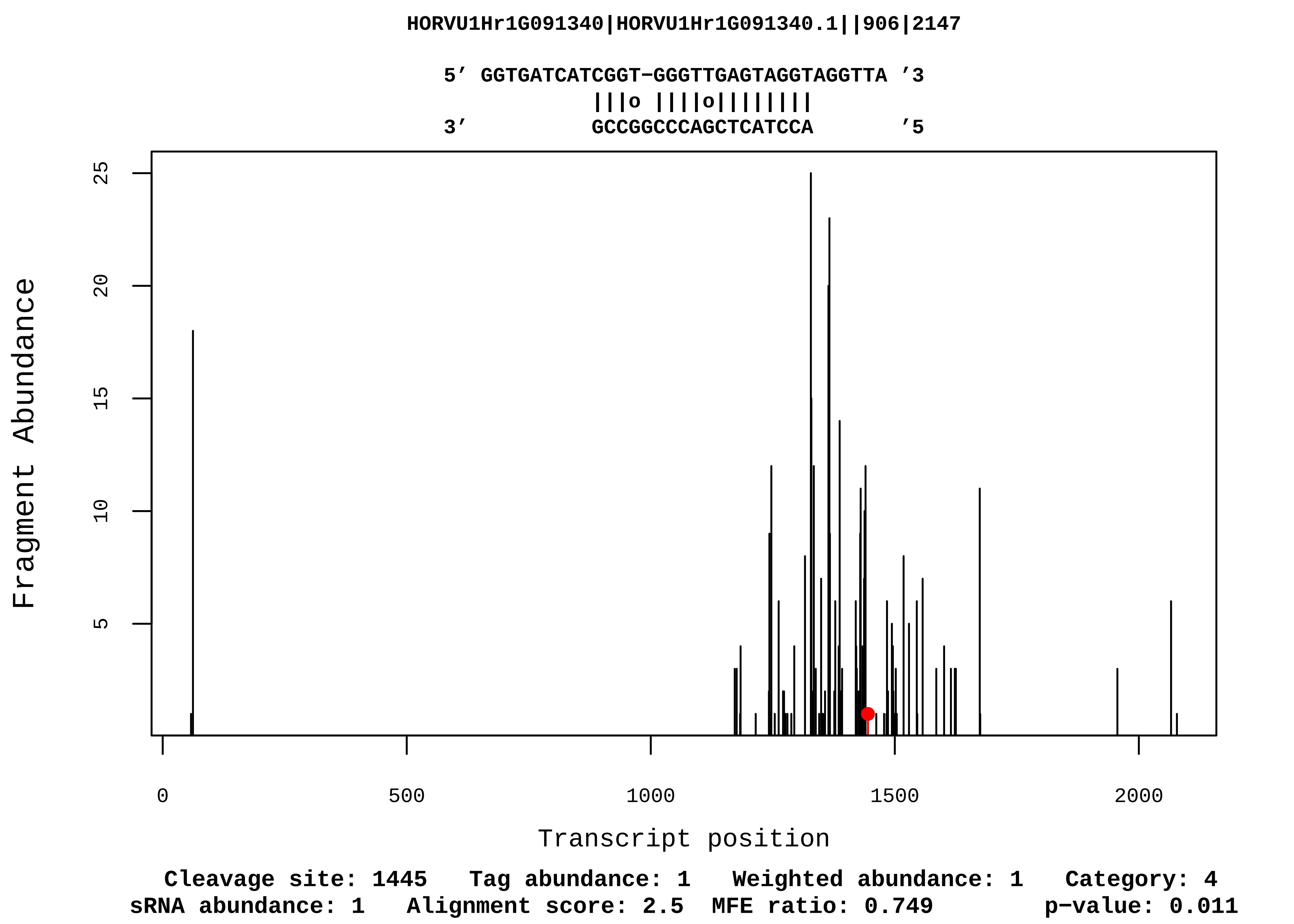
HORVU1Hr1G091340|HORVU1Hr1G091340.1||906|2147
5′ GGTGATCATCGGT−GGGTTGAGTAGGTAGGTTA ′3
|||o ||||o||||||||
3′ GCCGGCCCAGCTCATCCA ′5
Fragment Abundance
Transcript position
0
500
1000
1500
2000
5
10
15
20
25
Cleavage site: 1445 Tag abundance: 1 Weighted abundance: 1 Category: 4
sRNA abundance: 1 Alignment score: 2.5 MFE ratio: 0.749 p−value: 0.011

HORVU7Hr1G116750|HORVU7Hr1G116750.1||870|1239

```
5' CATTCGATCGGGTCGAGTAGGTCGGCAGCAAT '3
         || o|||||||||||||||||
3'       GCCGGCCCAGCTCATCCAGCCA       '5
```

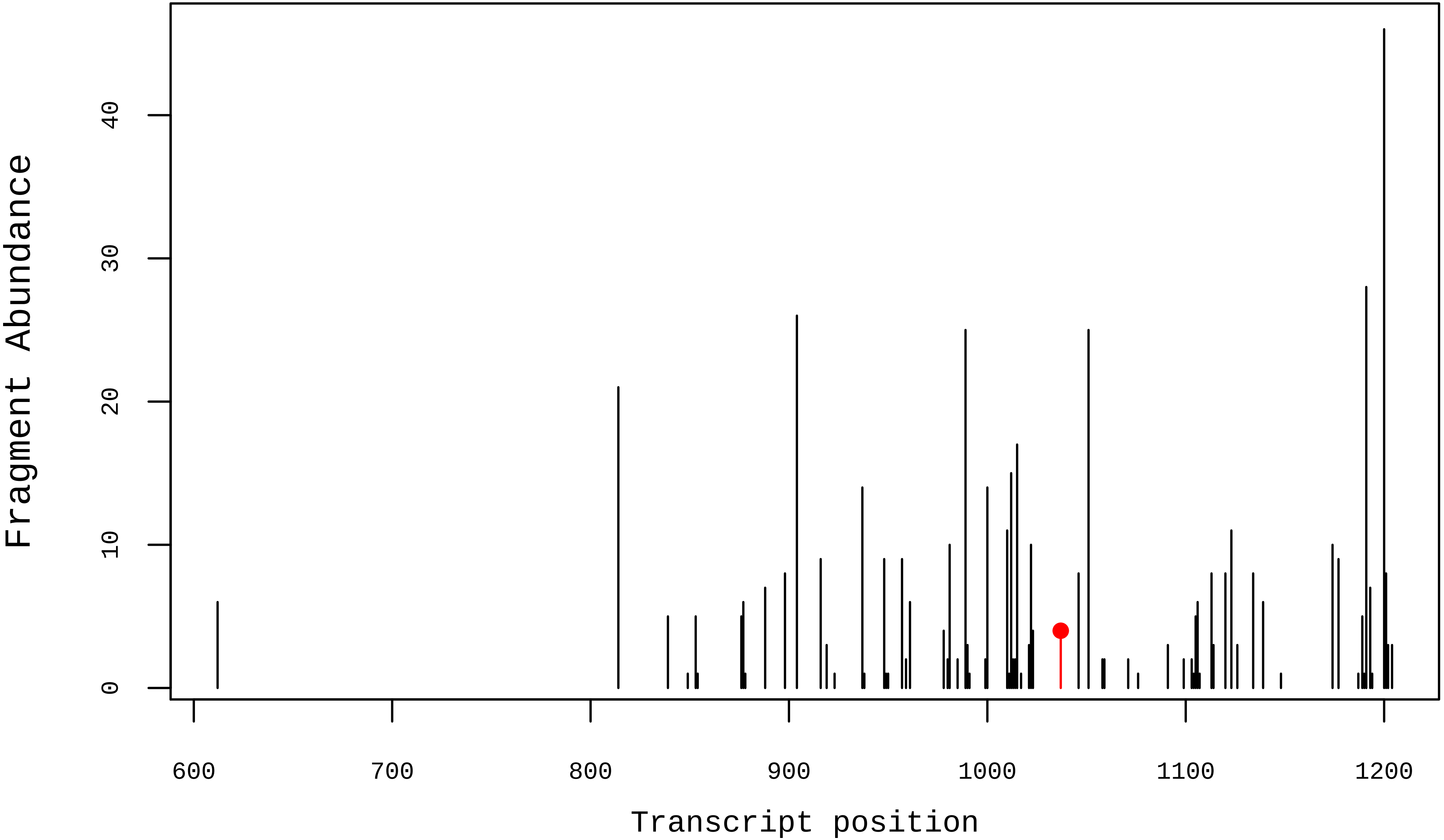

Cleavage site: 1037 Tag abundance: 4 Weighted abundance: 4 Category: 3
sRNA abundance: 1 Alignment score: 2.5 MFE ratio: 0.793 p-value: 0.033

HORVU0Hr1G003660|HORVU0Hr1G003660.1||297|1955

```
5' GCCGGCCGAAGGGTCGAGTAGGTCGGTGCTCG '3
      ||||||||||||||||||||o|||
3'    GCCGGCTTCCCAGCTCATCCGGCC       '5
```

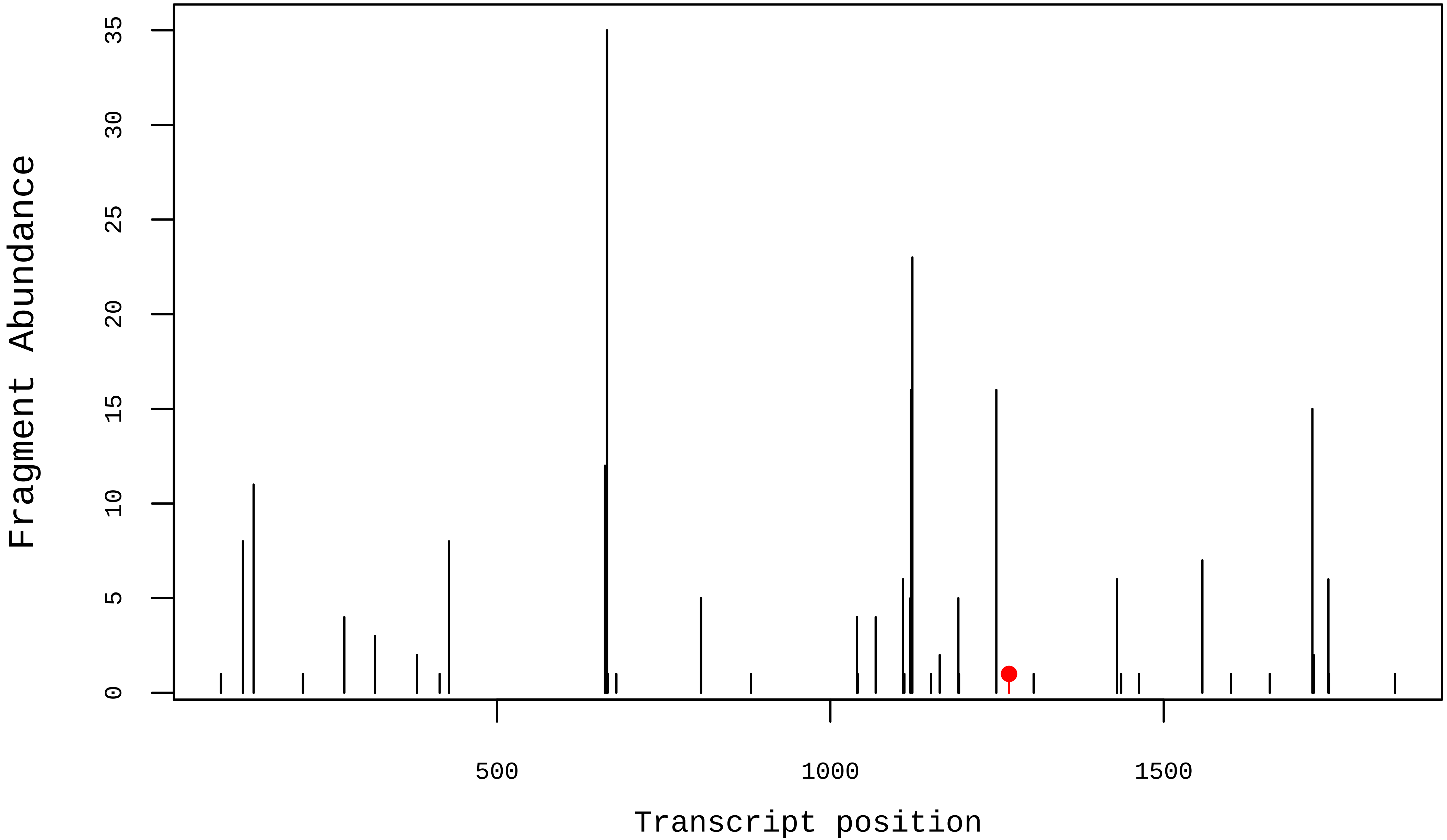

Cleavage site: 1268   Tag abundance: 1   Weighted abundance: 0.143   Category: 4
sRNA abundance: 1   Alignment score: 1   MFE ratio: 0.951   p-value: 0.046

HORVU0Hr1G023930|HORVU0Hr1G023930.3||396|2180

```
5' GTCGGCGGAAGGGTCGAGTAGGTCGGTGCTCG '3
      |||| |||||||||||||||o|||
3'    GCCGGCTTCCCAGCTCATCCGGCC       '5
```

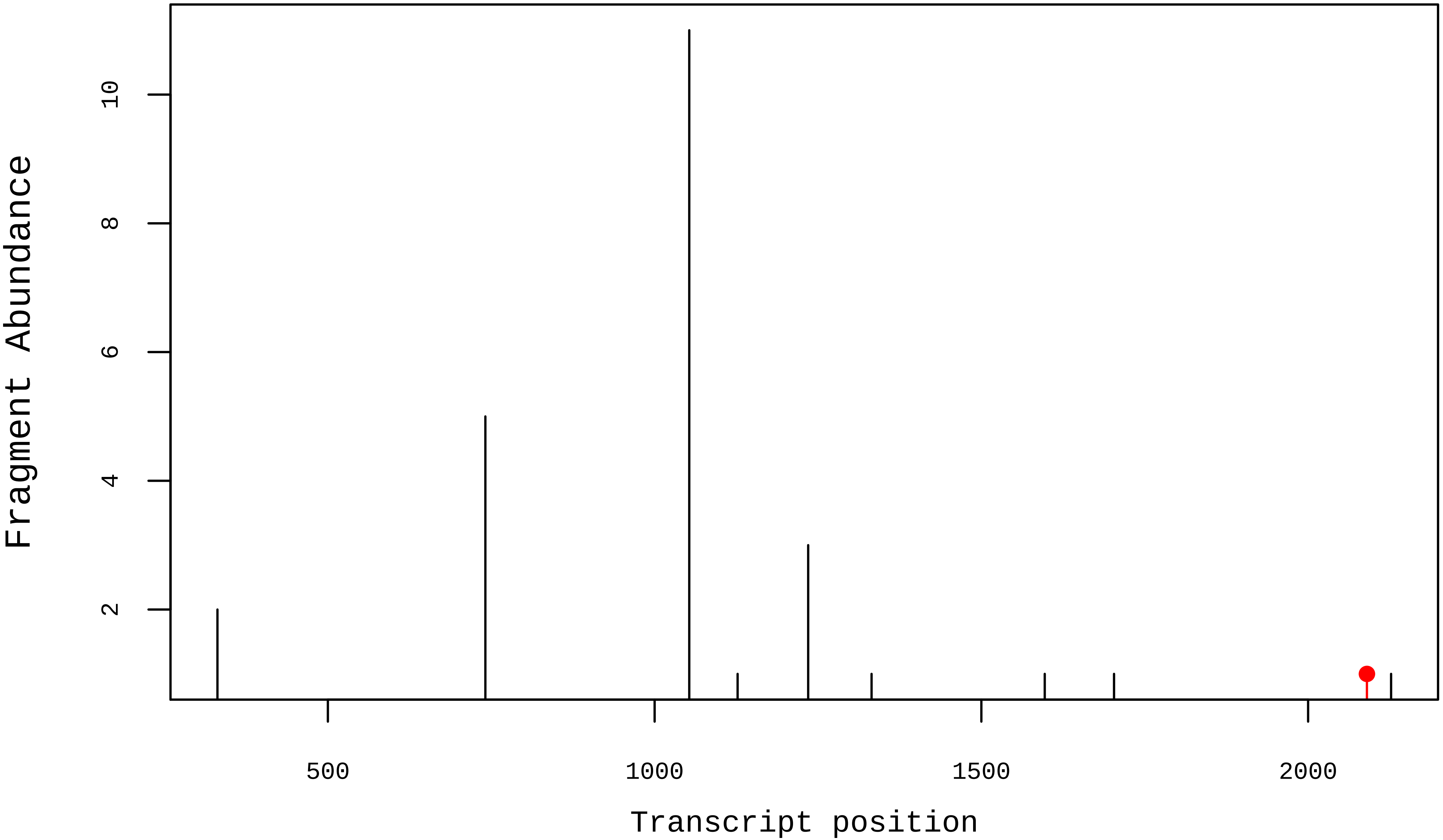

Cleavage site: 2090   Tag abundance: 1   Weighted abundance: 0.143   Category: 4
sRNA abundance: 1   Alignment score: 2   MFE ratio: 0.874   p-value: 0.019

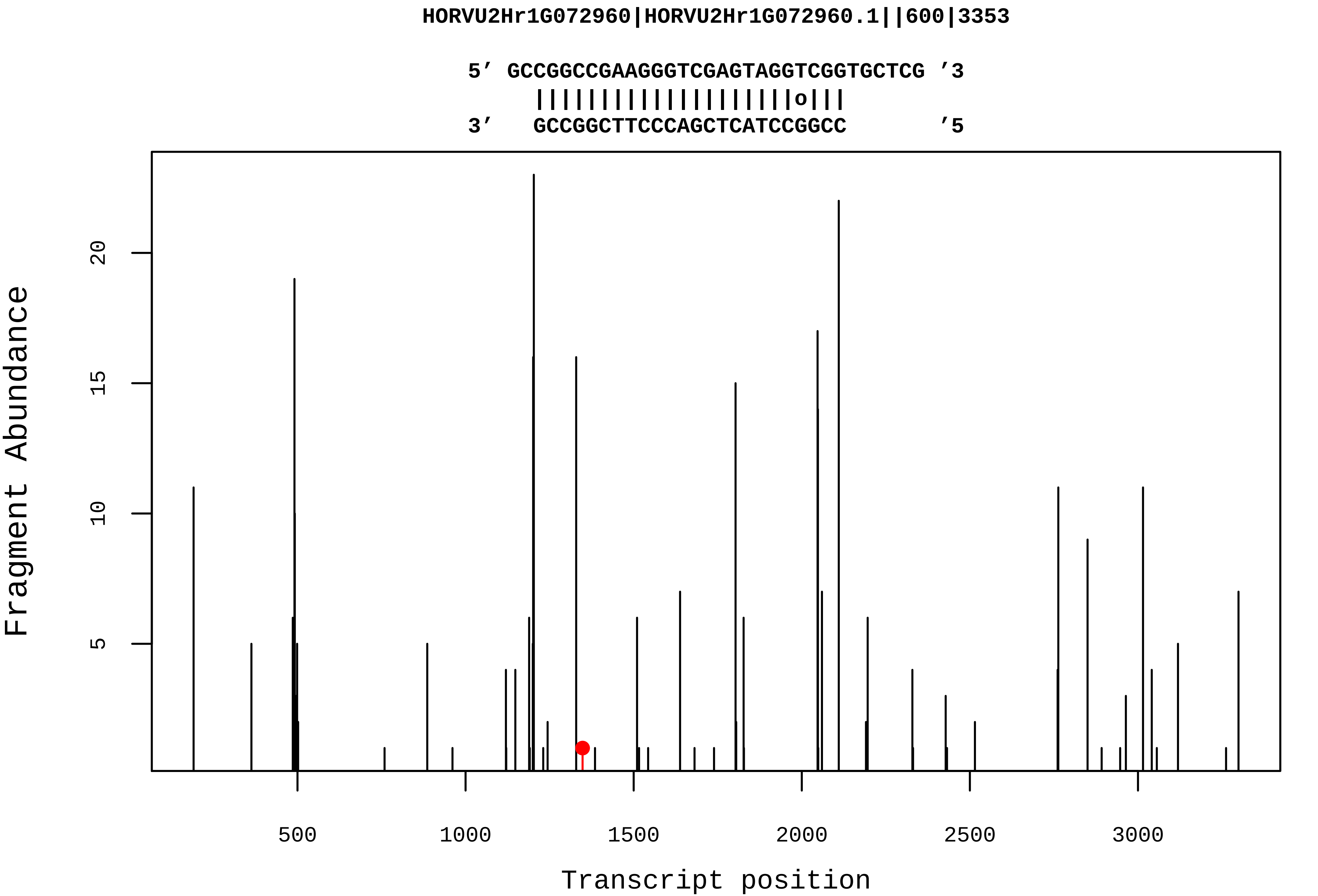

Cleavage site: 1348 Tag abundance: 1 Weighted abundance: 0.143 Category: 4
sRNA abundance: 1 Alignment score: 1 MFE ratio: 0.951 p-value: 0.03

HORVU5Hr1G015600|HORVU5Hr1G015600.1||156|510

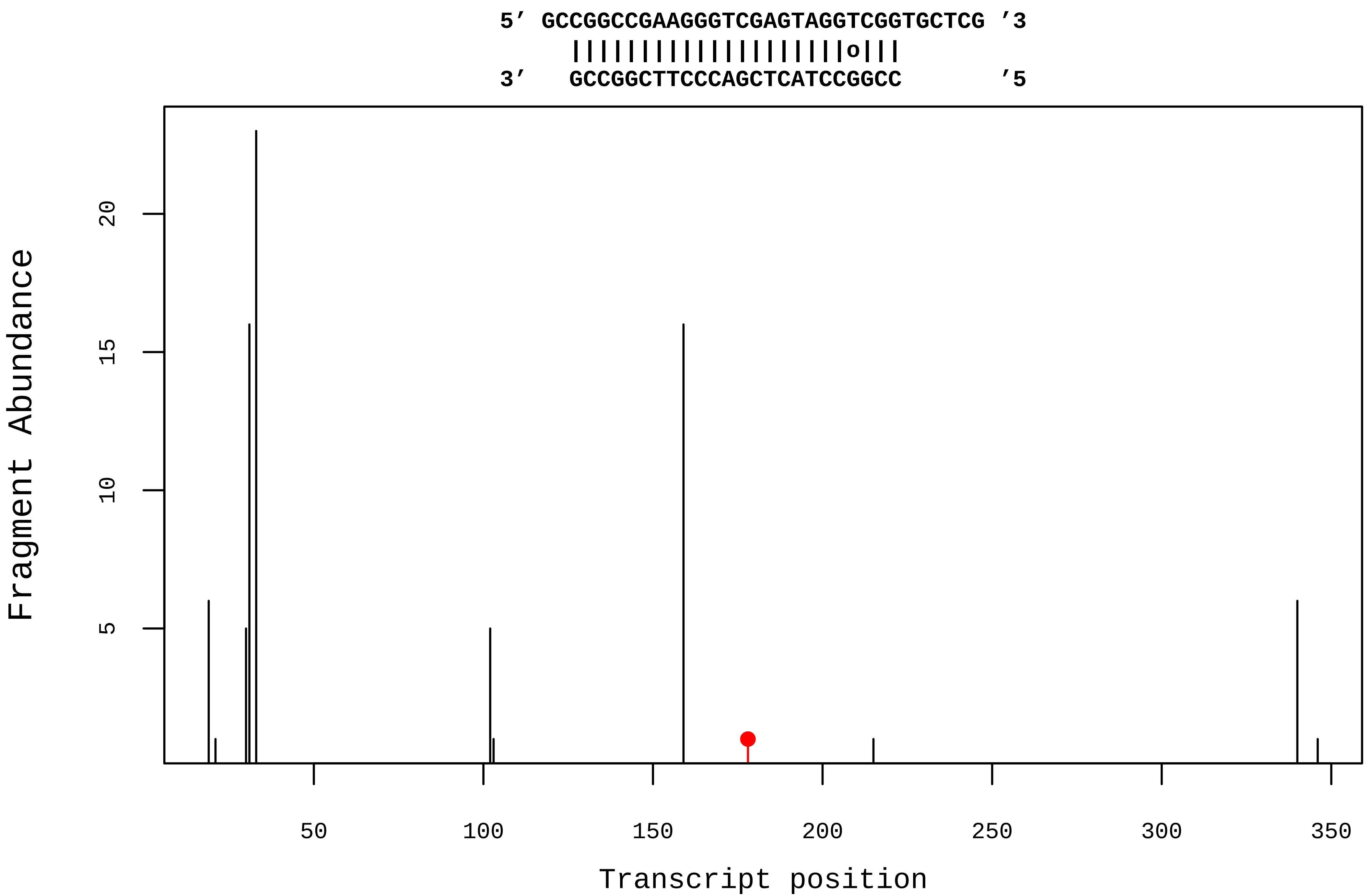

Cleavage site: 178 Tag abundance: 1 Weighted abundance: 0.143 Category: 4
sRNA abundance: 1 Alignment score: 1 MFE ratio: 0.951 p-value: 0.05

HORVU5Hr1G015600|HORVU5Hr1G015600.2||231|617

```
5' GCCGGCCGAAGGGTCGAGTAGGTCGGTGCTCG '3
       ||||||||||||||||||||o|||
3'    GCCGGCTTCCCAGCTCATCCGGCC        '5
```

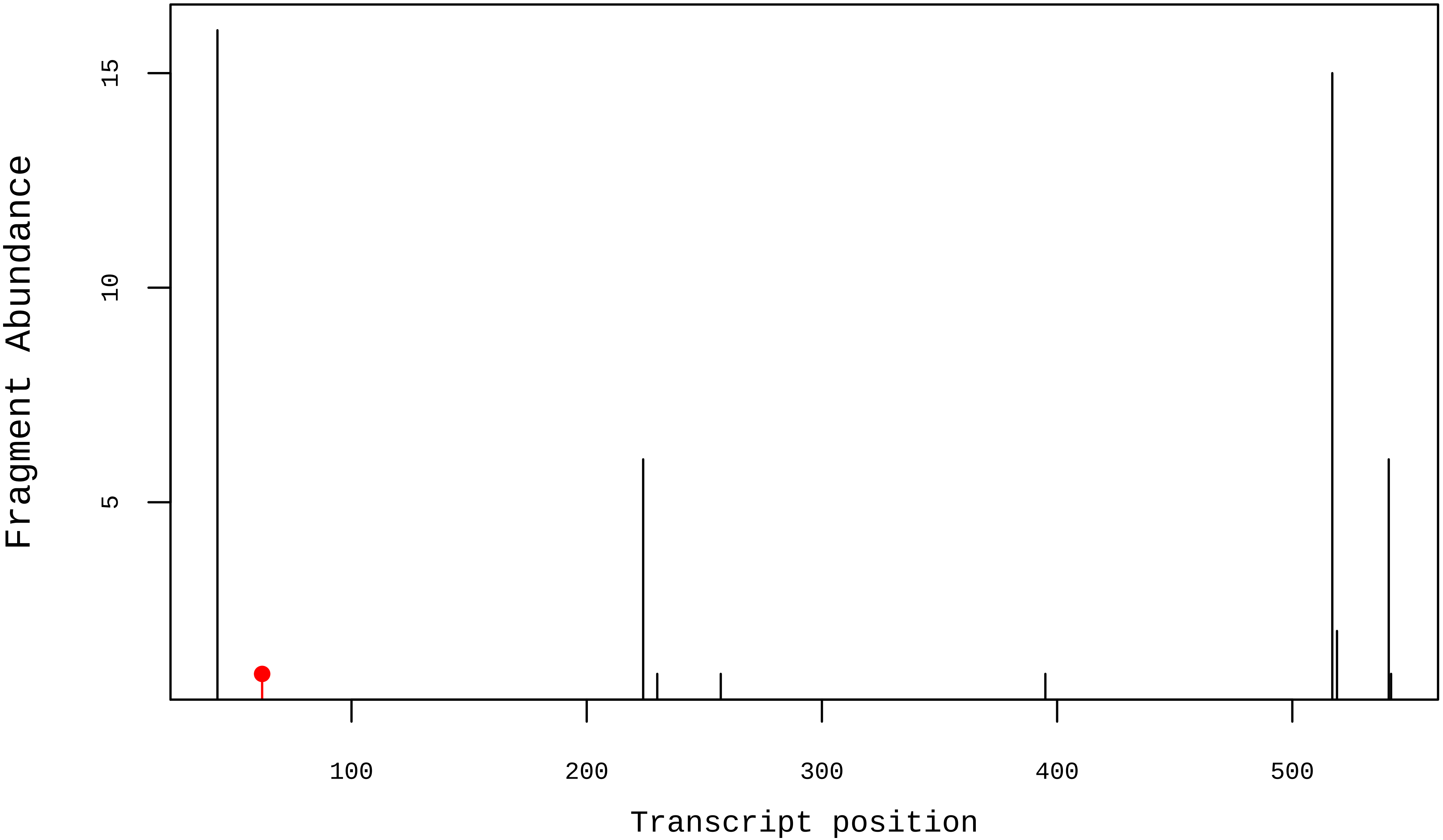

Cleavage site: 62 Tag abundance: 1 Weighted abundance: 0.143 Category: 4

sRNA abundance: 1 Alignment score: 1 MFE ratio: 0.951 p-value: 0.041

HORVU5Hr1G015600|HORVU5Hr1G015600.3||276|1709

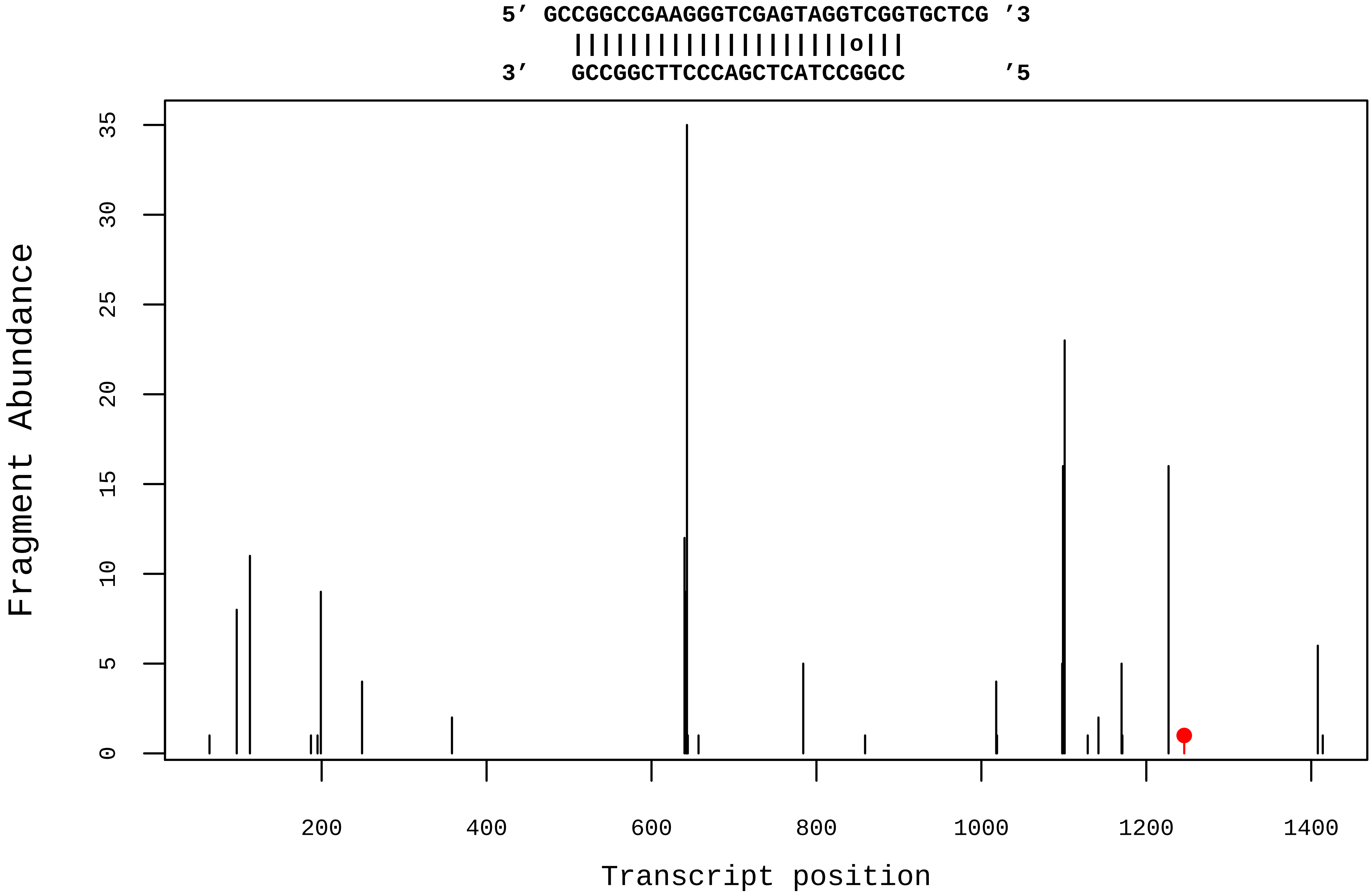

Cleavage site: 1246 Tag abundance: 1 Weighted abundance: 0.143 Category: 4
sRNA abundance: 1 Alignment score: 1 MFE ratio: 0.951 p-value: 0.032

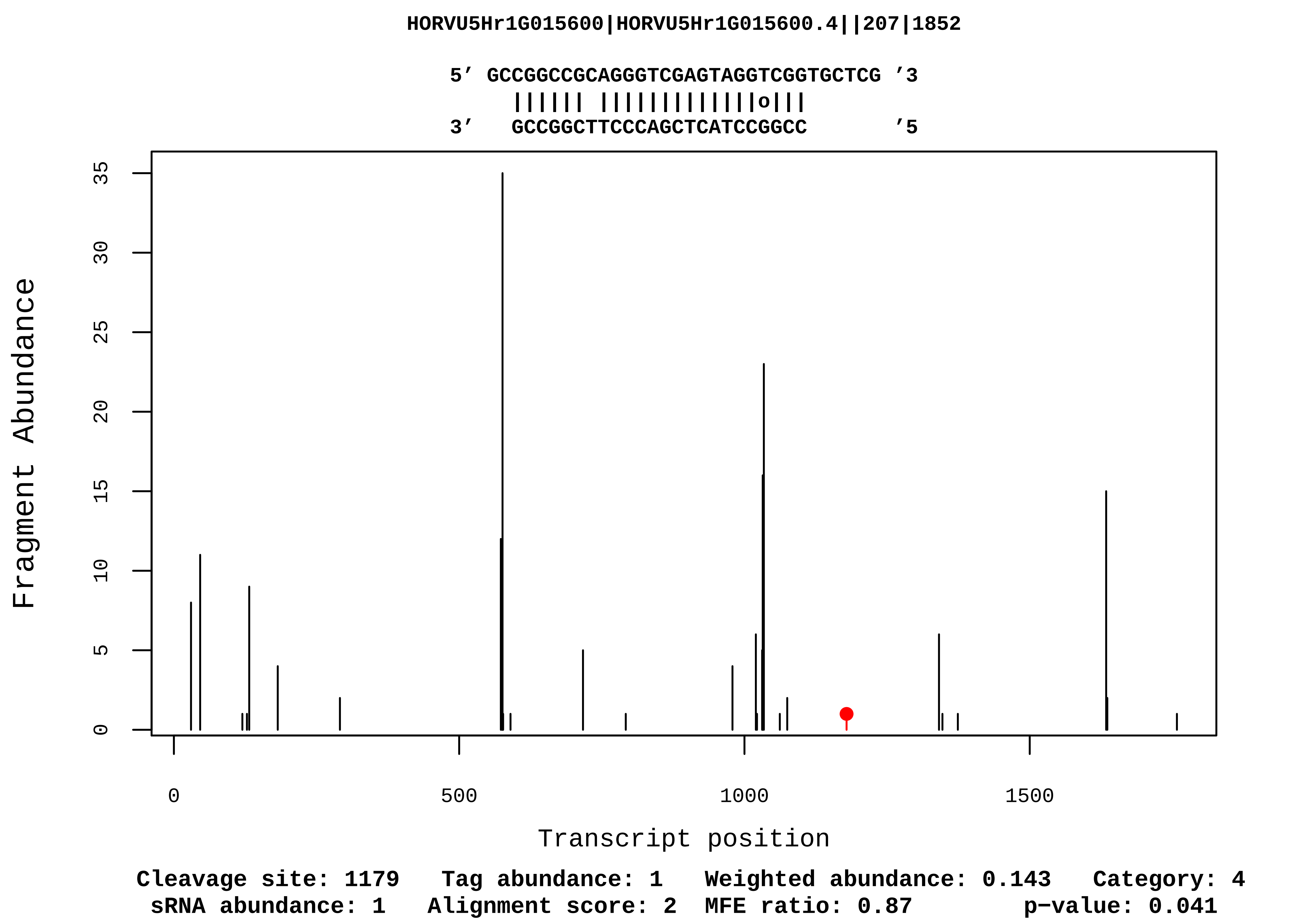
HORVU5Hr1G015600|HORVU5Hr1G015600.4||207|1852
5' GCCGGCCGCAGGGTCGAGTAGGTCGGTGCTCG '3
|||||| |||||||||||||o|||
3' GCCGGCTTCCCAGCTCATCCGGCC '5
Fragment Abundance
0
5
10
15
20
25
30
35
0
500
1000
1500
Transcript position
Cleavage site: 1179 Tag abundance: 1 Weighted abundance: 0.143 Category: 4
sRNA abundance: 1 Alignment score: 2 MFE ratio: 0.87 p-value: 0.041

HORVU0Hr1G023930|HORVU0Hr1G023930.1||297|1937

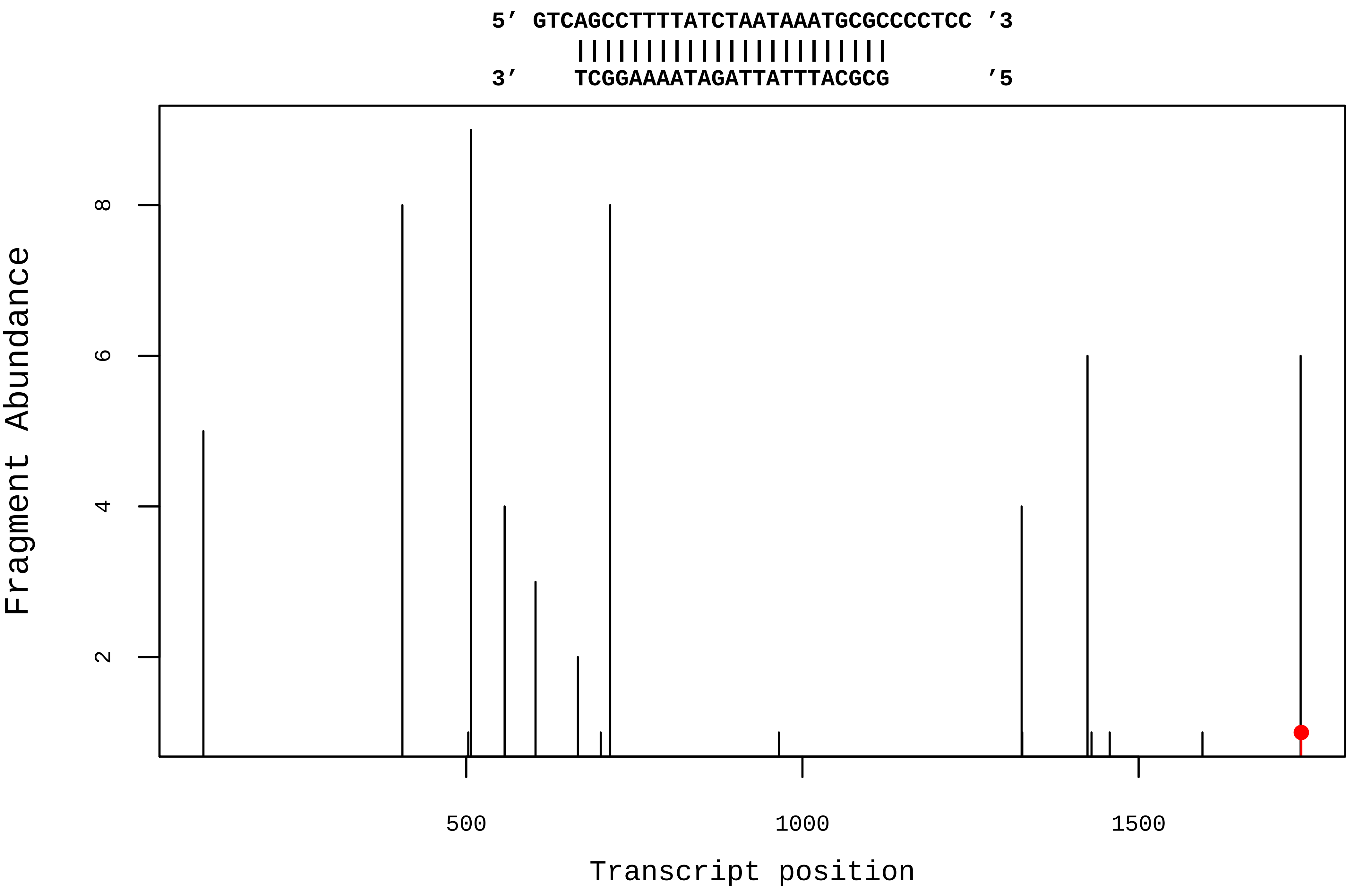

Cleavage site: 1742 Tag abundance: 1 Weighted abundance: 0.125 Category: 4
sRNA abundance: 1 Alignment score: 0 MFE ratio: 1 p−value: 0.033

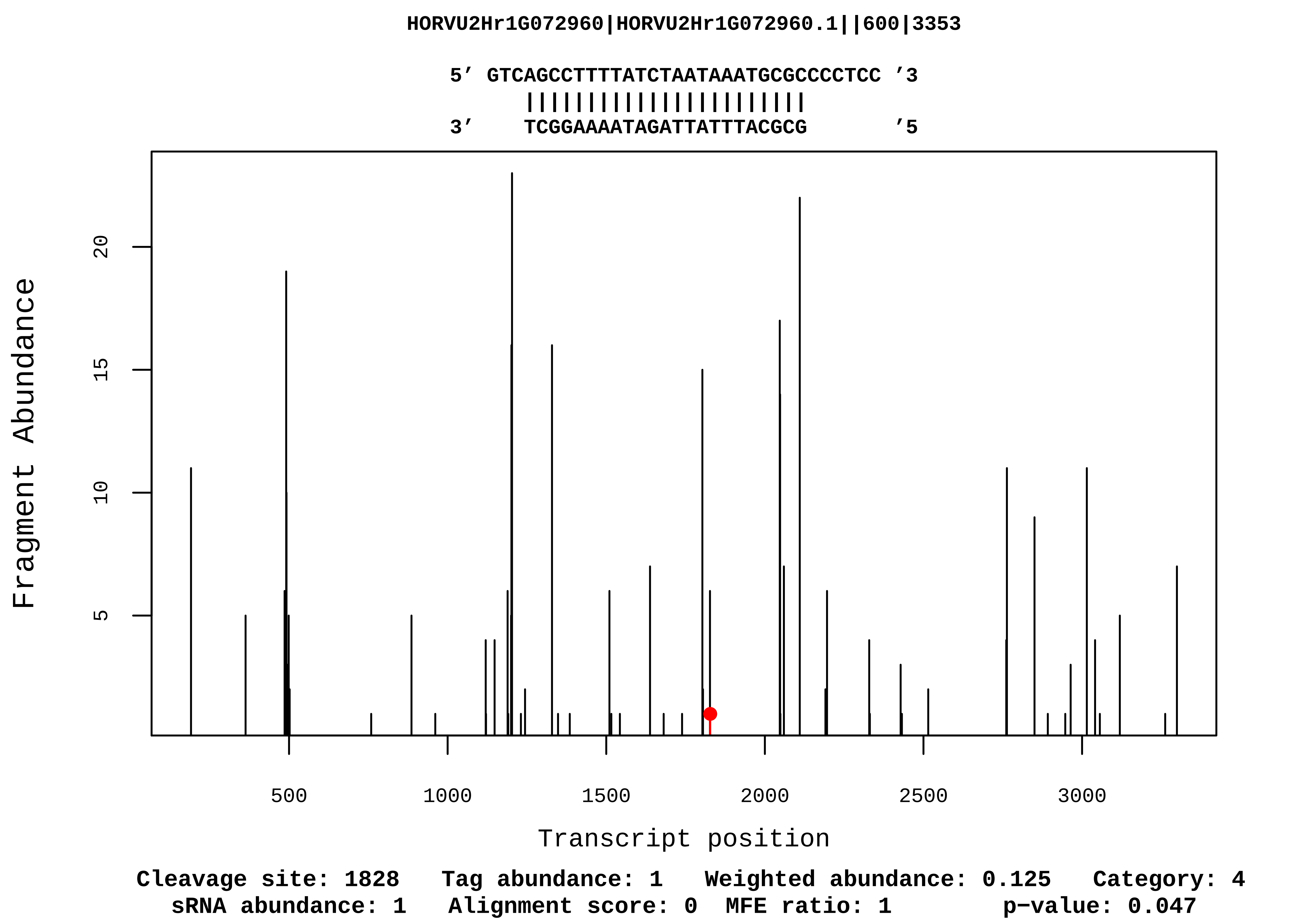
HORVU2Hr1G072960|HORVU2Hr1G072960.1||600|3353
5' GTCAGCCTTTTATCTAATAAATGCGCCCCTCC '3
||||||||||||||||||||||
3' TCGGAAAATAGATTATTTACGCG '5
Fragment Abundance
5
10
15
20
500
1000
1500
2000
2500
3000
Transcript position
Cleavage site: 1828 Tag abundance: 1 Weighted abundance: 0.125 Category: 4
sRNA abundance: 1 Alignment score: 0 MFE ratio: 1 p-value: 0.047

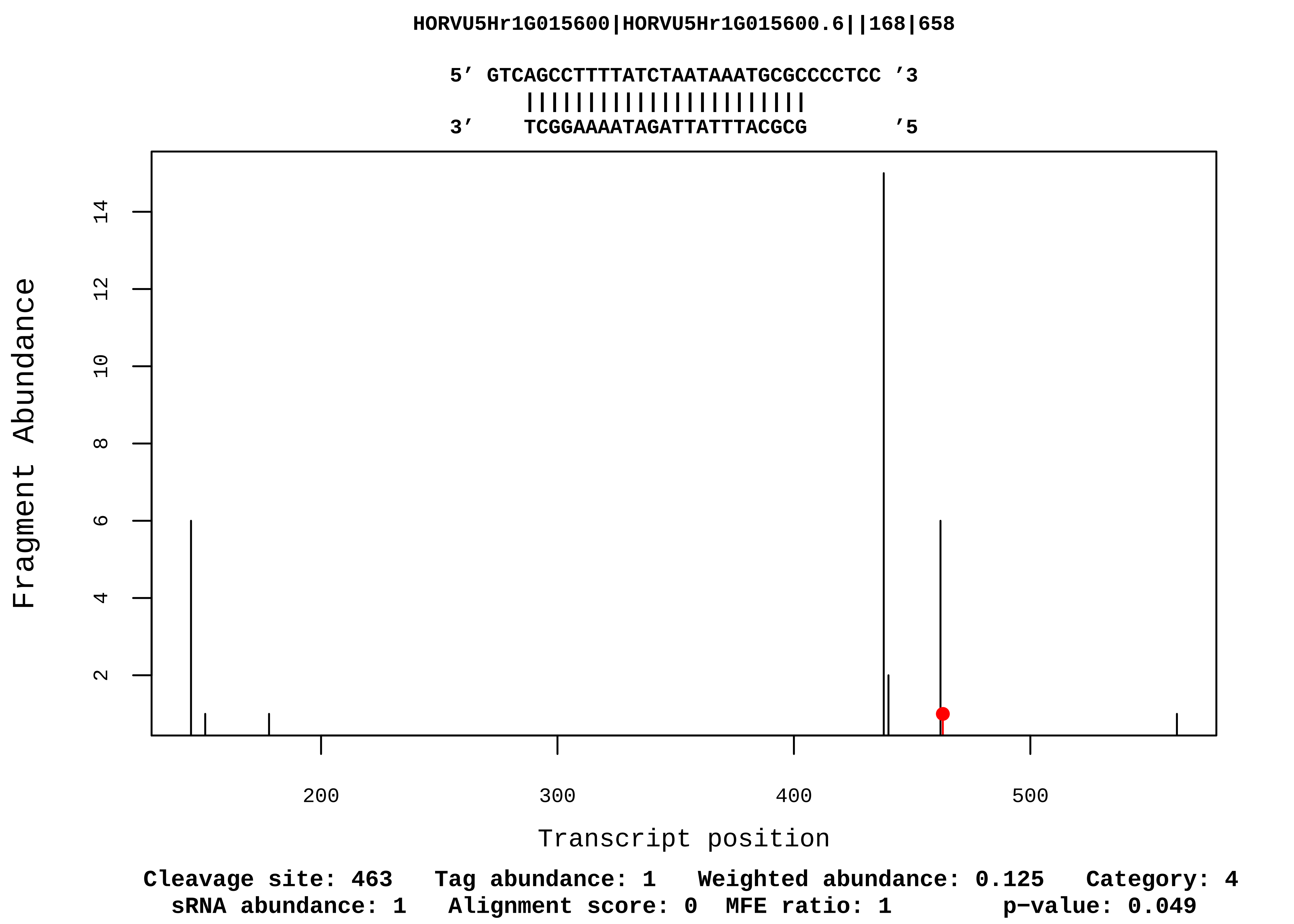
HORVU5Hr1G015600|HORVU5Hr1G015600.6||168|658
5′ GTCAGCCTTTTATCTAATAAATGCGCCCCTCC ′3
||||||||||||||||||||||
3′ TCGGAAAATAGATTATTTACGCG ′5
Fragment Abundance
2
4
6
8
10
12
14
200
300
400
500
Transcript position
Cleavage site: 463 Tag abundance: 1 Weighted abundance: 0.125 Category: 4
sRNA abundance: 1 Alignment score: 0 MFE ratio: 1 p-value: 0.049
