# Supplementary material for: Pi-starvation induced transcriptional changes in barley revealed by a comprehensive RNA-Seq and degradome analyses
Source: BMC Genomics. 2021 Mar 9;22:165. doi: 10.1186/s12864-021-07481-w (PMC7941915; doi:10.1186/s12864-021-07481-w)
Supplement: Supplementary file 28 — Additional file 28. miRbase annotation report from CLC Workbench (QIAGEN) analysis of shoot small RNAs. [file 12864_2021_7481_MOESM28_ESM.pdf]

Additional file 28. Annotation report from CLC Workbench (QIAGEN) analysis of shoot small RNAs.

Legend:

Shoot, sufficient Pi, biological replicate #1 - 170110_SNK268_A_L004_JDG-1-10_R1 trimmed Small RNA sample

Shoot, sufficient Pi, biological replicate #2 - 170110_SNK268_A_L004_JDG-1-11_R1 trimmed Small RNA sample

Shoot, sufficient Pi, biological replicate #3 - 170110_SNK268_A_L004_JDG-1-12_R1 trimmed Small RNA sample

Shoot, low-Pi, biological replicate #1 - 170110_SNK268_A_L004_JDG-1-4_R1 trimmed Small RNA sample

Shoot, low-Pi, biological replicate #2 - 170110_SNK268_A_L004_JDG-1-5_R1 trimmed Small RNA sample

Shoot, low-Pi, biological replicate #3 - 170110_SNK268_A_L004_JDG-1-6_R1 trimmed Small RNA sample

QIAGEN

Table of contents

1. Summary 3
2. Resources 4
3. Samples 5
    3.1 Sample: 170110_SNK268_A_L004_JDG-1-10_R1 trimmed Small RNA sample 6
    3.2 Sample: 170110_SNK268_A_L004_JDG-1-11_R1 trimmed Small RNA sample 9
    3.3 Sample: 170110_SNK268_A_L004_JDG-1-12_R1 trimmed Small RNA sample 13
    3.4 Sample: 170110_SNK268_A_L004_JDG-1-4_R1 trimmed Small RNA sample 16
    3.5 Sample: 170110_SNK268_A_L004_JDG-1-5_R1 trimmed Small RNA sample 20
    3.6 Sample: 170110_SNK268_A_L004_JDG-1-6_R1 trimmed Small RNA sample 23
4. Annotations (miRBase) 27

1. Summary

Name	Small RNAs	Annotated	Percentage	Ambiguously annotated
170110_SNK268_A_L004_JDG-1-10_R1 trimmed Small RNA sample	10 234 339	4 430	0,0%	685
170110_SNK268_A_L004_JDG-1-11_R1 trimmed Small RNA sample	10 234 339	4 430	0,0%	685
170110_SNK268_A_L004_JDG-1-12_R1 trimmed Small RNA sample	10 234 339	4 430	0,0%	685
170110_SNK268_A_L004_JDG-1-4_R1 trimmed Small RNA sample	10 234 339	4 430	0,0%	685
170110_SNK268_A_L004_JDG-1-5_R1 trimmed Small RNA sample	10 234 339	4 430	0,0%	685
170110_SNK268_A_L004_JDG-1-6_R1 trimmed Small RNA sample	10 234 339	4 430	0,0%	685

Name	Percentage	Reads	Annotated	Percentage
170110_SNK268_A_L004_JDG-1-10_R1 trimmed Small RNA sample	0,0%	13 414 482	856 943	6,4%
170110_SNK268_A_L004_JDG-1-11_R1 trimmed Small RNA sample	0,0%	12 249 247	774 026	6,3%
170110_SNK268_A_L004_JDG-1-12_R1 trimmed Small RNA sample	0,0%	10 640 396	588 430	5,5%
170110_SNK268_A_L004_JDG-1-4_R1 trimmed Small RNA sample	0,0%	14 644 436	733 431	5,0%

Name	Percentage	Reads	Annotated	Percentage
170110_SNK268_A_ L004_JDG-1-5_R1 trimmed Small RNA sample	0,0%	12 168 277	655 638	5,4%
170110_SNK268_A_ L004_JDG-1-6_R1 trimmed Small RNA sample	0,0%	10 565 625	616 282	5,8%

Name	Ambiguously annotated	Percentage
170110_SNK268_A_ L004_JDG-1-10_R1 trimmed Small RNA sample	99 671	0,7%
170110_SNK268_A_ L004_JDG-1-11_R1 trimmed Small RNA sample	76 809	0,6%
170110_SNK268_A_ L004_JDG-1-12_R1 trimmed Small RNA sample	74 626	0,7%
170110_SNK268_A_ L004_JDG-1-4_R1 trimmed Small RNA sample	87 176	0,6%
170110_SNK268_A_ L004_JDG-1-5_R1 trimmed Small RNA sample	77 514	0,6%
170110_SNK268_A_ L004_JDG-1-6_R1 trimmed Small RNA sample	74 749	0,7%

2. Resources

Resource	Sequences in resource	Sequences found	Percentage found
miRBase (Aegilops tauschii)	88	71	80,7%
miRBase (Arabidopsis lyrata)	205	63	30,7%
miRBase (Arabidopsis thaliana)	326	10	3,1%
miRBase (Brachypodium distachyon)	317	80	25,2%
miRBase (Brassica napus)	90	9	10,0%
miRBase (Brassica oleracea)	10	0	0,0%

Resource	Sequences in resource	Sequences found	Percentage found
miRBase (Brassica rapa)	96	3	3,1%
miRBase (Caenorhabditis elegans)	253	3	1,2%
miRBase (Chlamydomonas reinhardtii)	50	1	2,0%
miRBase (Cucumis melo)	120	30	25,0%
miRBase (Glycine max)	684	73	10,7%
miRBase (Glycine soja)	13	0	0,0%
miRBase (Gossypium arboreum)	1	0	0,0%
miRBase (Gossypium herbaceum)	1	0	0,0%
miRBase (Gossypium hirsutum)	78	6	7,7%
miRBase (Gossypium raimondii)	296	1	0,3%
miRBase (Hordeum vulgare)	69	55	79,7%
miRBase (Medicago truncatula)	672	22	3,3%
miRBase (Nicotiana tabacum)	162	10	6,2%
miRBase (Oryza sativa)	604	100	16,6%
miRBase (Phaseolus vulgaris)	8	1	12,5%
miRBase (Physcomitrella patens)	247	18	7,3%
miRBase (Pinus densata)	29	10	34,5%
miRBase (Picea abies)	594	42	7,1%
miRBase (Prunus persica)	180	8	4,4%
miRBase (Solanum lycopersicum)	112	7	6,2%
miRBase (Solanum tuberosum)	224	13	5,8%
miRBase (Sorghum bicolor)	205	25	12,2%
miRBase (Triticum aestivum)	122	43	35,2%
miRBase (Triticum turgidum)	1	0	0,0%
miRBase (Vitis vinifera)	163	19	11,7%
miRBase (Zea mays)	174	36	20,7%

3. Samples

3.1 Sample: 170110_SNK268_A_L004_JDG-1-10_R1 trimmed Small RNA sample

Reads

Annotation	Count	Percentage
Annotated	856 943	6,4%
- with miRBase (Aegilops tauschii)	406 891	47,5%
- with miRBase (Arabidopsis lyrata)	1 418	0,2%
- with miRBase (Arabidopsis thaliana)	3 562	0,4%
- with miRBase (Brachypodium distachyon)	12 930	1,5%
- with miRBase (Brassica napus)	522	0,1%
- with miRBase (Brassica oleracea)	0	0,0%
- with miRBase (Brassica rapa)	32	0,0%
- with miRBase (Caenorhabditis elegans)	0	0,0%
- with miRBase (Chlamydomonas reinhardtii)	0	0,0%
- with miRBase (Cucumis melo)	1 964	0,2%
- with miRBase (Glycine max)	266 112	31,1%
- with miRBase (Glycine soja)	0	0,0%
- with miRBase (Gossypium arboreum)	0	0,0%
- with miRBase (Gossypium herbaceum)	0	0,0%
- with miRBase (Gossypium hirsutum)	7	0,0%
- with miRBase (Gossypium raimondii)	448	0,1%
- with miRBase (Hordeum vulgare)	83 390	9,7%
- with miRBase (Medicago truncatula)	34	0,0%
- with miRBase (Nicotiana tabacum)	76	0,0%
- with miRBase (Oryza sativa)	62 886	7,3%
- with miRBase (Phaseolus vulgaris)	180	0,0%
- with miRBase (Physcomitrella patens)	3 179	0,4%
- with miRBase (Pinus densata)	10	0,0%
- with miRBase (Picea abies)	303	0,0%
- with miRBase (Prunus persica)	474	0,1%
- with miRBase (Solanum lycopersicum)	8	0,0%
- with miRBase (Solanum tuberosum)	114	0,0%
- with miRBase (Sorghum bicolor)	516	0,1%
- with miRBase (Triticum aestivum)	11 070	1,3%
- with miRBase (Triticum turgidum)	0	0,0%

Annotation	Count	Percentage
- with miRBase (Vitis vinifera)	45	0,0%
- with miRBase (Zea mays)	772	0,1%
Unannotated	12 557 539	93,6%
Total	13 414 482	100,0%

Small RNAs

Annotation	Count	Percentage
Annotated	4 430	0,0%
- with miRBase (Aegilops tauschii)	1 246	28,1%
- with miRBase (Arabidopsis lyrata)	129	2,9%
- with miRBase (Arabidopsis thaliana)	49	1,1%
- with miRBase (Brachypodium distachyon)	511	11,5%
- with miRBase (Brassica napus)	22	0,5%
- with miRBase (Brassica oleracea)	0	0,0%
- with miRBase (Brassica rapa)	5	0,1%
- with miRBase (Caenorhabditis elegans)	10	0,2%
- with miRBase (Chlamydomonas reinhardtii)	1	0,0%
- with miRBase (Cucumis melo)	50	1,1%
- with miRBase (Glycine max)	288	6,5%
- with miRBase (Glycine soja)	0	0,0%
- with miRBase (Gossypium arboreum)	0	0,0%
- with miRBase (Gossypium herbaceum)	0	0,0%
- with miRBase (Gossypium hirsutum)	8	0,2%
- with miRBase (Gossypium raimondii)	6	0,1%
- with miRBase (Hordeum vulgare)	795	17,9%
- with miRBase (Medicago truncatula)	28	0,6%
- with miRBase (Nicotiana tabacum)	22	0,5%
- with miRBase (Oryza sativa)	408	9,2%
- with miRBase (Phaseolus vulgaris)	8	0,2%
- with miRBase (Physcomitrella patens)	31	0,7%
- with miRBase (Pinus densata)	16	0,4%
- with miRBase (Picea abies)	44	1,0%
- with miRBase (Prunus persica)	16	0,4%
- with miRBase (Solanum lycopersicum)	12	0,3%
- with miRBase (Solanum tuberosum)	21	0,5%
- with miRBase (Sorghum bicolor)	60	1,4%

Annotation	Count	Percentage
- with miRBase (Triticum aestivum)	547	12,3%
- with miRBase (Triticum turgidum)	0	0,0%
- with miRBase (Vitis vinifera)	25	0,6%
- with miRBase (Zea mays)	72	1,6%
Unannotated	10 229 909	100,0%
Total	10 234 339	100,0%

Read count proportions

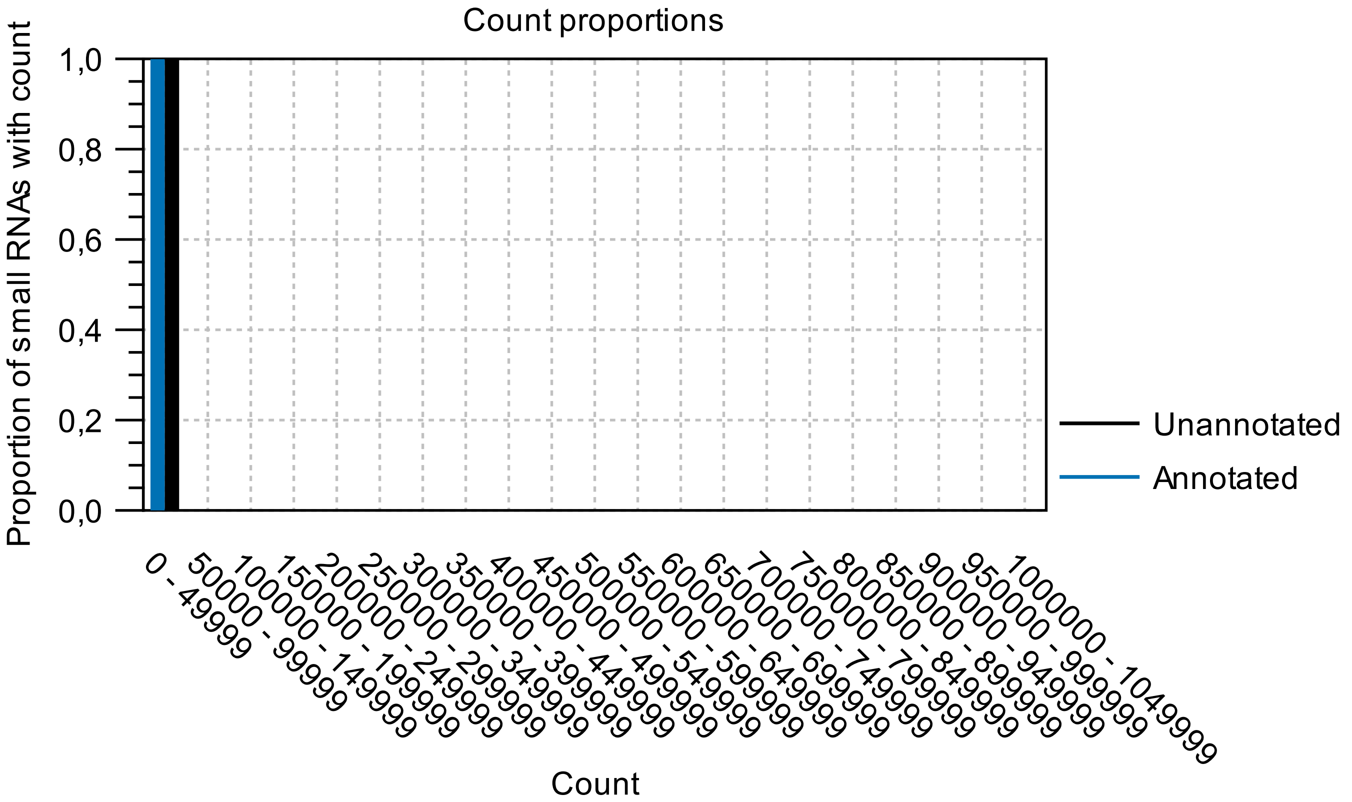

The histogram shows, for each interval of read counts, the proportion of annotated (respectively, unannotated) small RNAs with a read count in that interval. Annotated small RNAs may be expected to be associated with higher counts.

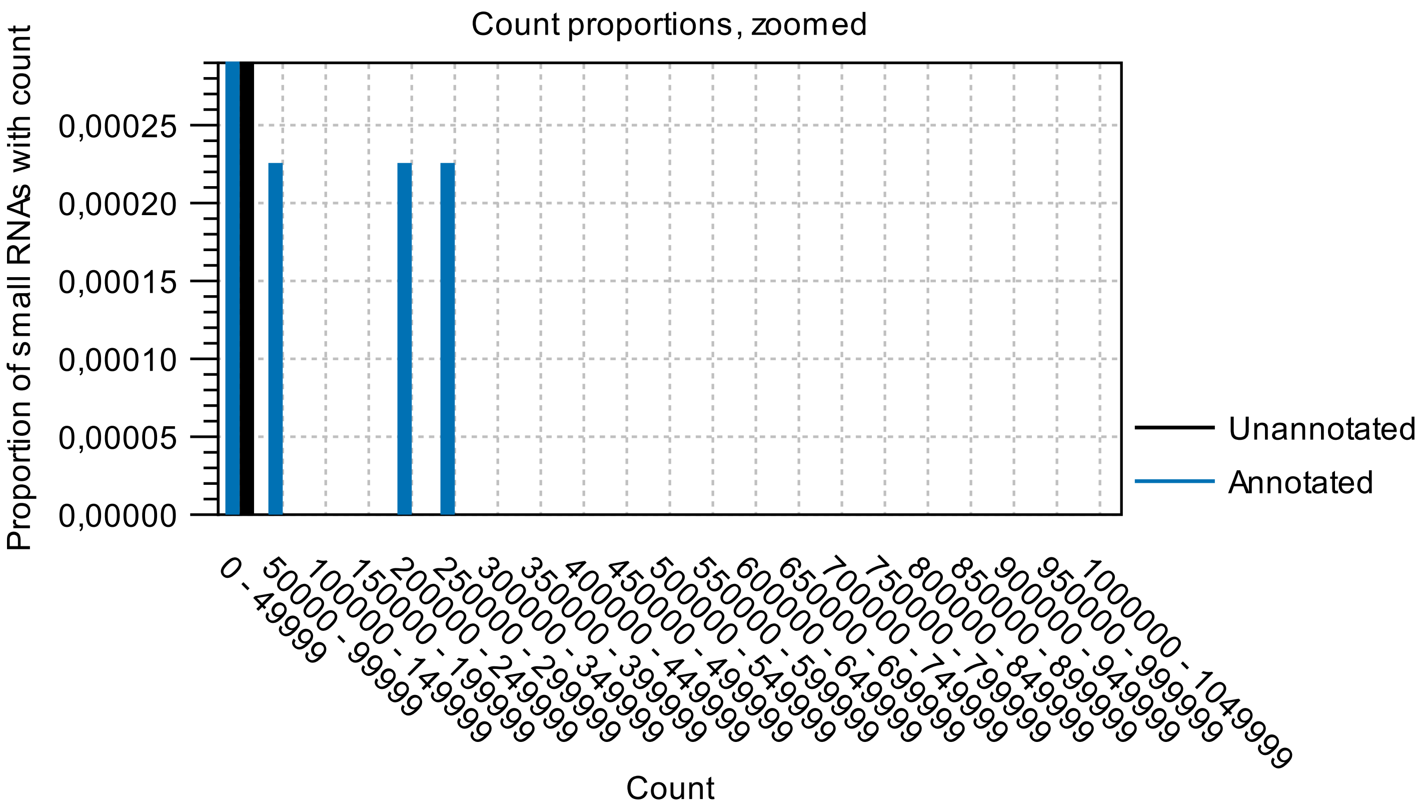

Zoomed version of the read count proportions histogram. Note that some bars extend beyond the plotting area.

3.2 Sample: 170110_SNK268_A_L004_JDG-1-11_R1 trimmed Small RNA sample

Reads

Annotation	Count	Percentage
Annotated	774 026	6,3%
- with miRBase (Aegilops tauschii)	366 945	47,4%
- with miRBase (Arabidopsis lyrata)	1 162	0,2%
- with miRBase (Arabidopsis thaliana)	3 273	0,4%
- with miRBase (Brachypodium distachyon)	9 867	1,3%
- with miRBase (Brassica napus)	432	0,1%
- with miRBase (Brassica oleracea)	0	0,0%
- with miRBase (Brassica rapa)	20	0,0%
- with miRBase (Caenorhabditis elegans)	0	0,0%
- with miRBase (Chlamydomonas reinhardtii)	0	0,0%

Annotation	Count	Percentage
- with miRBase (Cucumis melo)	1 550	0,2%
- with miRBase (Glycine max)	256 736	33,2%
- with miRBase (Glycine soja)	0	0,0%
- with miRBase (Gossypium arboreum)	0	0,0%
- with miRBase (Gossypium herbaceum)	0	0,0%
- with miRBase (Gossypium hirsutum)	9	0,0%
- with miRBase (Gossypium raimondii)	382	0,0%
- with miRBase (Hordeum vulgare)	67 970	8,8%
- with miRBase (Medicago truncatula)	36	0,0%
- with miRBase (Nicotiana tabacum)	49	0,0%
- with miRBase (Oryza sativa)	50 945	6,6%
- with miRBase (Phaseolus vulgaris)	187	0,0%
- with miRBase (Physcomitrella patens)	3 527	0,5%
- with miRBase (Pinus densata)	6	0,0%
- with miRBase (Picea abies)	197	0,0%
- with miRBase (Prunus persica)	698	0,1%
- with miRBase (Solanum lycopersicum)	12	0,0%
- with miRBase (Solanum tuberosum)	113	0,0%
- with miRBase (Sorghum bicolor)	523	0,1%
- with miRBase (Triticum aestivum)	8 744	1,1%
- with miRBase (Triticum turgidum)	0	0,0%
- with miRBase (Vitis vinifera)	37	0,0%
- with miRBase (Zea mays)	606	0,1%
Unannotated	11 475 221	93,7%
Total	12 249 247	100,0%

Small RNAs

Annotation	Count	Percentage
Annotated	4 430	0,0%
- with miRBase (Aegilops tauschii)	1 246	28,1%
- with miRBase (Arabidopsis lyrata)	129	2,9%
- with miRBase (Arabidopsis thaliana)	49	1,1%
- with miRBase (Brachypodium distachyon)	511	11,5%
- with miRBase (Brassica napus)	22	0,5%
- with miRBase (Brassica oleracea)	0	0,0%
- with miRBase (Brassica rapa)	5	0,1%
- with miRBase (Caenorhabditis elegans)	10	0,2%

Annotation	Count	Percentage
- with miRBase (Chlamydomonas reinhardtii)	1	0,0%
- with miRBase (Cucumis melo)	50	1,1%
- with miRBase (Glycine max)	288	6,5%
- with miRBase (Glycine soja)	0	0,0%
- with miRBase (Gossypium arboreum)	0	0,0%
- with miRBase (Gossypium herbaceum)	0	0,0%
- with miRBase (Gossypium hirsutum)	8	0,2%
- with miRBase (Gossypium raimondii)	6	0,1%
- with miRBase (Hordeum vulgare)	795	17,9%
- with miRBase (Medicago truncatula)	28	0,6%
- with miRBase (Nicotiana tabacum)	22	0,5%
- with miRBase (Oryza sativa)	408	9,2%
- with miRBase (Phaseolus vulgaris)	8	0,2%
- with miRBase (Physcomitrella patens)	31	0,7%
- with miRBase (Pinus densata)	16	0,4%
- with miRBase (Picea abies)	44	1,0%
- with miRBase (Prunus persica)	16	0,4%
- with miRBase (Solanum lycopersicum)	12	0,3%
- with miRBase (Solanum tuberosum)	21	0,5%
- with miRBase (Sorghum bicolor)	60	1,4%
- with miRBase (Triticum aestivum)	547	12,3%
- with miRBase (Triticum turgidum)	0	0,0%
- with miRBase (Vitis vinifera)	25	0,6%
- with miRBase (Zea mays)	72	1,6%
Unannotated	10 229 909	100,0%
Total	10 234 339	100,0%

Read count proportions

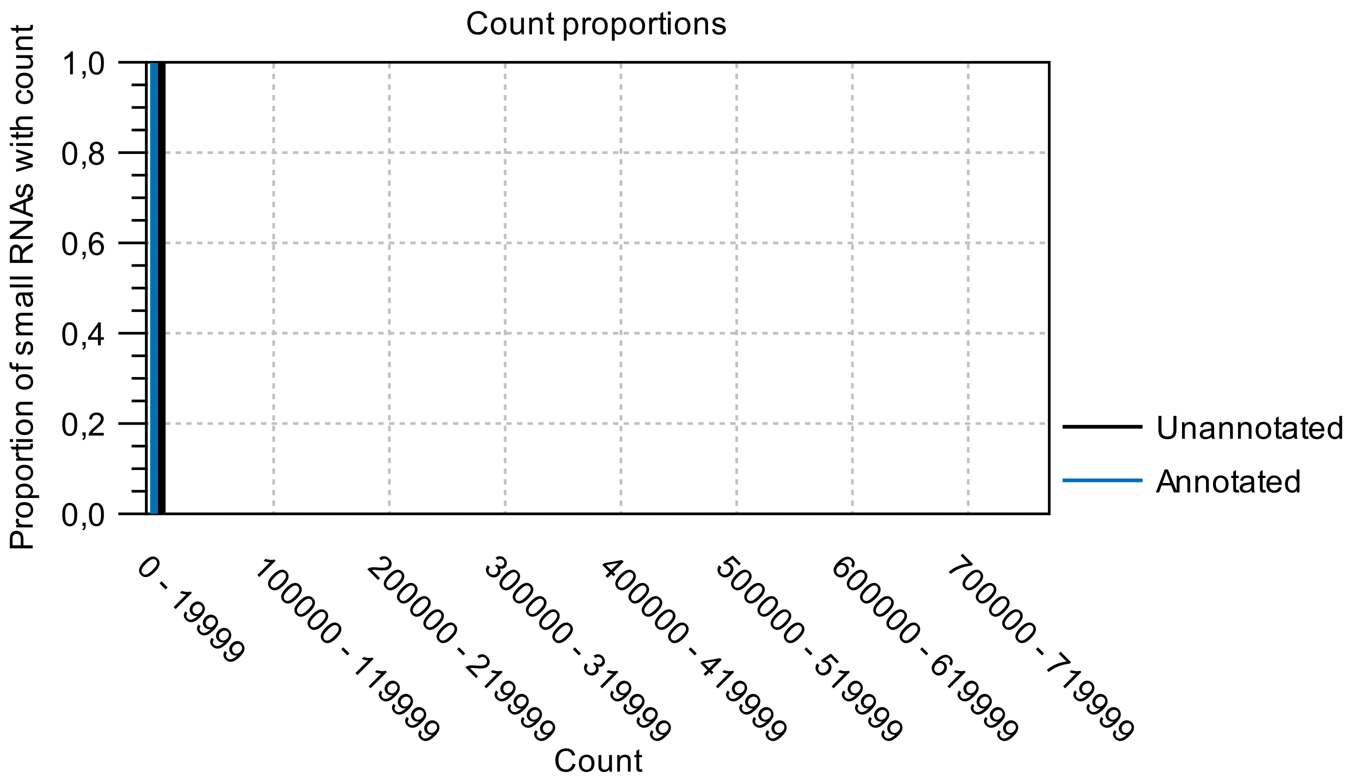

The histogram shows, for each interval of read counts, the proportion of annotated (respectively, unannotated) small RNAs with a read count in that interval. Annotated small RNAs may be expected to be associated with higher counts.

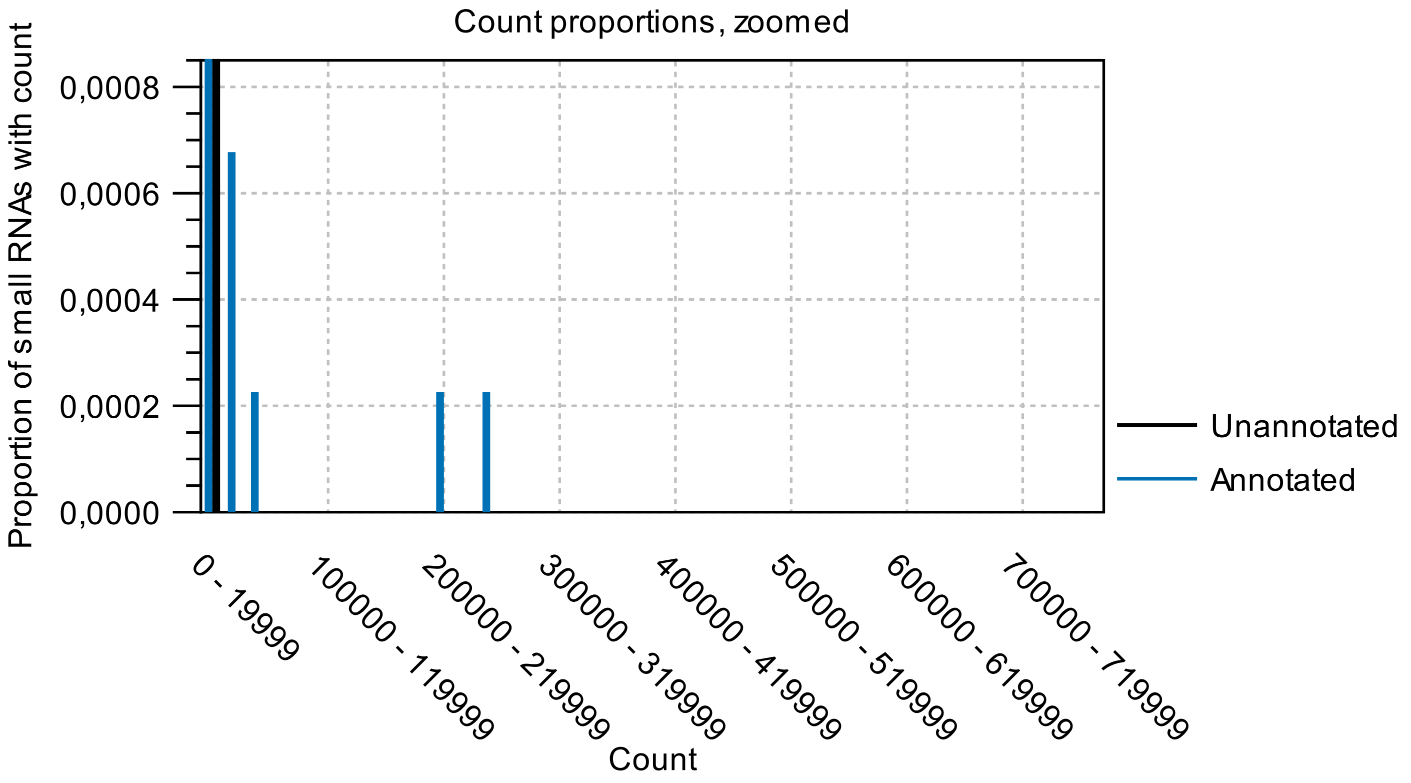

Zoomed version of the read count proportions histogram. Note that some bars extend beyond the plotting area.

3.3 Sample: 170110_SNK268_A_L004_JDG-1-12_R1 trimmed Small RNA sample

Reads

Annotation	Count	Percentage
Annotated	588 430	5,5%
- with miRBase (Aegilops tauschii)	254 920	43,3%
- with miRBase (Arabidopsis lyrata)	1 125	0,2%
- with miRBase (Arabidopsis thaliana)	3 059	0,5%
- with miRBase (Brachypodium distachyon)	7 357	1,3%
- with miRBase (Brassica napus)	377	0,1%
- with miRBase (Brassica oleracea)	0	0,0%
- with miRBase (Brassica rapa)	22	0,0%
- with miRBase (Caenorhabditis elegans)	0	0,0%
- with miRBase (Chlamydomonas reinhardtii)	0	0,0%
- with miRBase (Cucumis melo)	1 595	0,3%
- with miRBase (Glycine max)	210 355	35,7%
- with miRBase (Glycine soja)	0	0,0%
- with miRBase (Gossypium arboreum)	0	0,0%
- with miRBase (Gossypium herbaceum)	0	0,0%
- with miRBase (Gossypium hirsutum)	7	0,0%
- with miRBase (Gossypium raimondii)	373	0,1%
- with miRBase (Hordeum vulgare)	60 431	10,3%
- with miRBase (Medicago truncatula)	18	0,0%
- with miRBase (Nicotiana tabacum)	57	0,0%
- with miRBase (Oryza sativa)	37 196	6,3%
- with miRBase (Phaseolus vulgaris)	89	0,0%
- with miRBase (Physcomitrella patens)	2 770	0,5%
- with miRBase (Pinus densata)	25	0,0%
- with miRBase (Picea abies)	247	0,0%
- with miRBase (Prunus persica)	665	0,1%
- with miRBase (Solanum lycopersicum)	13	0,0%
- with miRBase (Solanum tuberosum)	66	0,0%

Annotation	Count	Percentage
- with miRBase (Sorghum bicolor)	345	0,1%
- with miRBase (Triticum aestivum)	6 838	1,2%
- with miRBase (Triticum turgidum)	0	0,0%
- with miRBase (Vitis vinifera)	36	0,0%
- with miRBase (Zea mays)	444	0,1%
Unannotated	10 051 966	94,5%
Total	10 640 396	100,0%

Small RNAs

Annotation	Count	Percentage
Annotated	4 430	0,0%
- with miRBase (Aegilops tauschii)	1 246	28,1%
- with miRBase (Arabidopsis lyrata)	129	2,9%
- with miRBase (Arabidopsis thaliana)	49	1,1%
- with miRBase (Brachypodium distachyon)	511	11,5%
- with miRBase (Brassica napus)	22	0,5%
- with miRBase (Brassica oleracea)	0	0,0%
- with miRBase (Brassica rapa)	5	0,1%
- with miRBase (Caenorhabditis elegans)	10	0,2%
- with miRBase (Chlamydomonas reinhardtii)	1	0,0%
- with miRBase (Cucumis melo)	50	1,1%
- with miRBase (Glycine max)	288	6,5%
- with miRBase (Glycine soja)	0	0,0%
- with miRBase (Gossypium arboreum)	0	0,0%
- with miRBase (Gossypium herbaceum)	0	0,0%
- with miRBase (Gossypium hirsutum)	8	0,2%
- with miRBase (Gossypium raimondii)	6	0,1%
- with miRBase (Hordeum vulgare)	795	17,9%
- with miRBase (Medicago truncatula)	28	0,6%
- with miRBase (Nicotiana tabacum)	22	0,5%
- with miRBase (Oryza sativa)	408	9,2%
- with miRBase (Phaseolus vulgaris)	8	0,2%
- with miRBase (Physcomitrella patens)	31	0,7%
- with miRBase (Pinus densata)	16	0,4%
- with miRBase (Picea abies)	44	1,0%
- with miRBase (Prunus persica)	16	0,4%

Annotation	Count	Percentage
- with miRBase (Solanum lycopersicum)	12	0,3%
- with miRBase (Solanum tuberosum)	21	0,5%
- with miRBase (Sorghum bicolor)	60	1,4%
- with miRBase (Triticum aestivum)	547	12,3%
- with miRBase (Triticum turgidum)	0	0,0%
- with miRBase (Vitis vinifera)	25	0,6%
- with miRBase (Zea mays)	72	1,6%
Unannotated	10 229 909	100,0%
Total	10 234 339	100,0%

Read count proportions

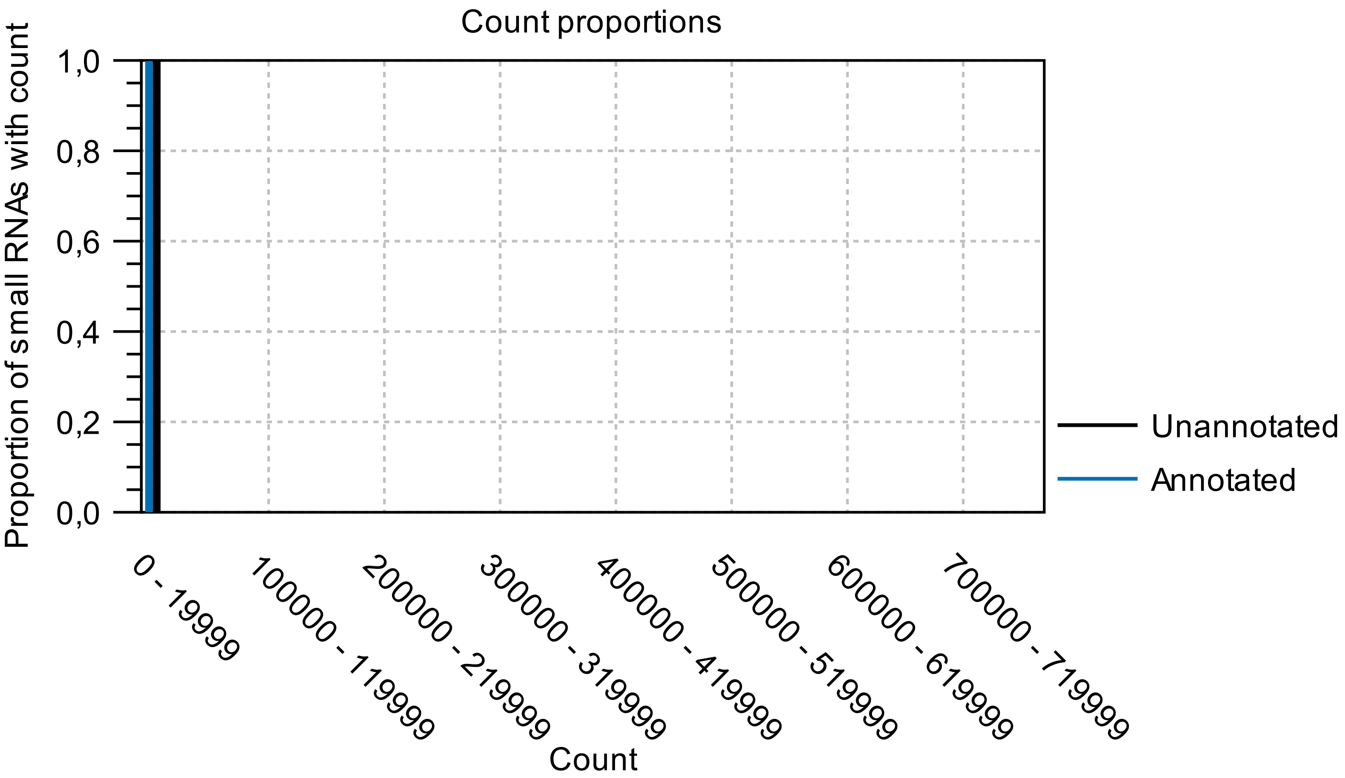

The histogram shows, for each interval of read counts, the proportion of annotated (respectively, unannotated) small RNAs with a read count in that interval. Annotated small RNAs may be expected to be associated with higher counts.

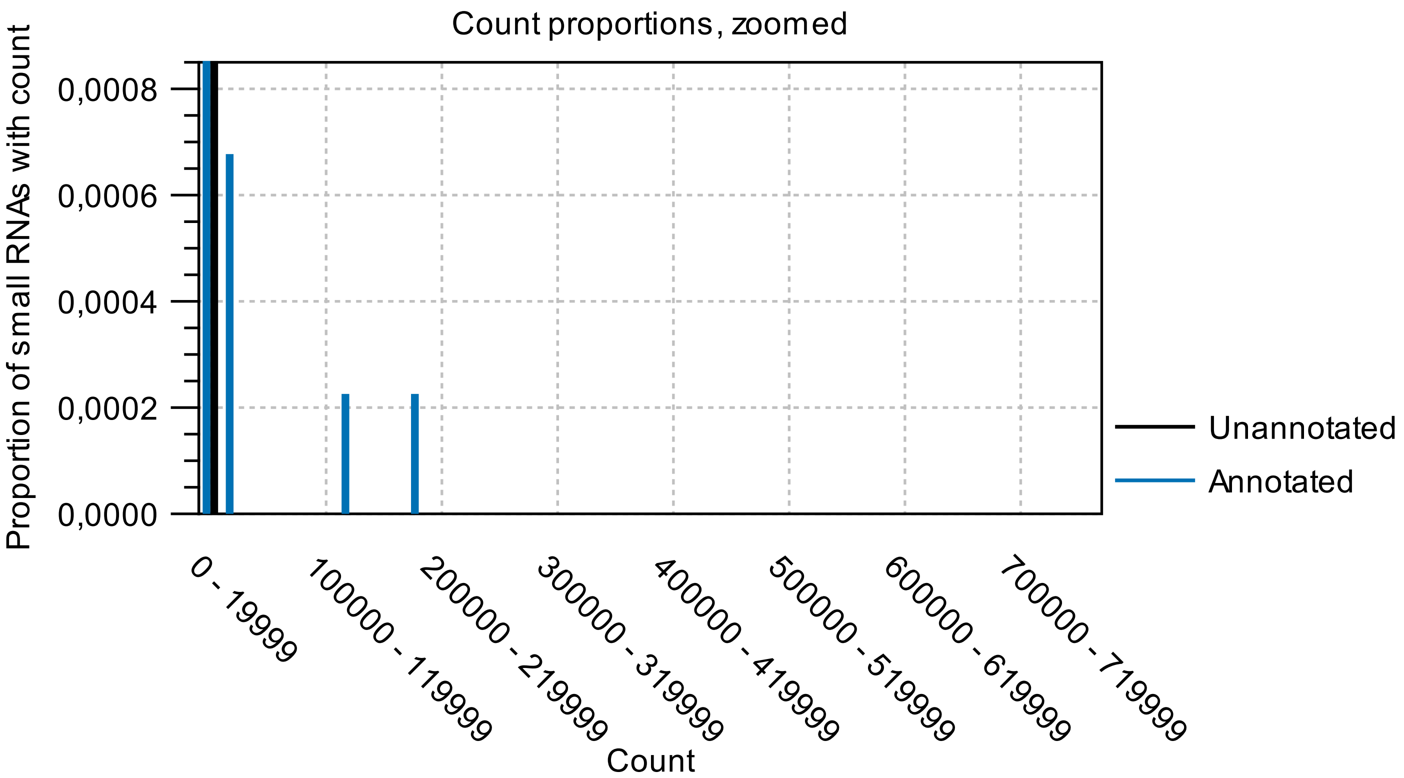

Zoomed version of the read count proportions histogram. Note that some bars extend beyond the plotting area.

3.4 Sample: 170110_SNK268_A_L004_JDG-1-4_R1 trimmed Small RNA sample

Reads

Annotation	Count	Percentage
Annotated	733 431	5,0%
- with miRBase (Aegilops tauschii)	345 420	47,1%
- with miRBase (Arabidopsis lyrata)	1 176	0,2%
- with miRBase (Arabidopsis thaliana)	3 509	0,5%
- with miRBase (Brachypodium distachyon)	17 945	2,4%
- with miRBase (Brassica napus)	411	0,1%
- with miRBase (Brassica oleracea)	0	0,0%
- with miRBase (Brassica rapa)	14	0,0%
- with miRBase (Caenorhabditis elegans)	2	0,0%
- with miRBase (Chlamydomonas reinhardtii)	0	0,0%
- with miRBase (Cucumis melo)	1 656	0,2%

Annotation	Count	Percentage
- with miRBase (Glycine max)	207 452	28,3%
- with miRBase (Glycine soja)	0	0,0%
- with miRBase (Gossypium arboreum)	0	0,0%
- with miRBase (Gossypium herbaceum)	0	0,0%
- with miRBase (Gossypium hirsutum)	9	0,0%
- with miRBase (Gossypium raimondii)	468	0,1%
- with miRBase (Hordeum vulgare)	68 858	9,4%
- with miRBase (Medicago truncatula)	41	0,0%
- with miRBase (Nicotiana tabacum)	104	0,0%
- with miRBase (Oryza sativa)	61 131	8,3%
- with miRBase (Phaseolus vulgaris)	1 885	0,3%
- with miRBase (Physcomitrella patens)	5 213	0,7%
- with miRBase (Pinus densata)	7	0,0%
- with miRBase (Picea abies)	318	0,0%
- with miRBase (Prunus persica)	827	0,1%
- with miRBase (Solanum lycopersicum)	14	0,0%
- with miRBase (Solanum tuberosum)	162	0,0%
- with miRBase (Sorghum bicolor)	617	0,1%
- with miRBase (Triticum aestivum)	15 518	2,1%
- with miRBase (Triticum turgidum)	0	0,0%
- with miRBase (Vitis vinifera)	53	0,0%
- with miRBase (Zea mays)	621	0,1%
Unannotated	13 911 005	95,0%
Total	14 644 436	100,0%

Small RNAs

Annotation	Count	Percentage
Annotated	4 430	0,0%
- with miRBase (Aegilops tauschii)	1 246	28,1%
- with miRBase (Arabidopsis lyrata)	129	2,9%
- with miRBase (Arabidopsis thaliana)	49	1,1%
- with miRBase (Brachypodium distachyon)	511	11,5%
- with miRBase (Brassica napus)	22	0,5%
- with miRBase (Brassica oleracea)	0	0,0%
- with miRBase (Brassica rapa)	5	0,1%
- with miRBase (Caenorhabditis elegans)	10	0,2%

Annotation	Count	Percentage
- with miRBase (Chlamydomonas reinhardtii)	1	0,0%
- with miRBase (Cucumis melo)	50	1,1%
- with miRBase (Glycine max)	288	6,5%
- with miRBase (Glycine soja)	0	0,0%
- with miRBase (Gossypium arboreum)	0	0,0%
- with miRBase (Gossypium herbaceum)	0	0,0%
- with miRBase (Gossypium hirsutum)	8	0,2%
- with miRBase (Gossypium raimondii)	6	0,1%
- with miRBase (Hordeum vulgare)	795	17,9%
- with miRBase (Medicago truncatula)	28	0,6%
- with miRBase (Nicotiana tabacum)	22	0,5%
- with miRBase (Oryza sativa)	408	9,2%
- with miRBase (Phaseolus vulgaris)	8	0,2%
- with miRBase (Physcomitrella patens)	31	0,7%
- with miRBase (Pinus densata)	16	0,4%
- with miRBase (Picea abies)	44	1,0%
- with miRBase (Prunus persica)	16	0,4%
- with miRBase (Solanum lycopersicum)	12	0,3%
- with miRBase (Solanum tuberosum)	21	0,5%
- with miRBase (Sorghum bicolor)	60	1,4%
- with miRBase (Triticum aestivum)	547	12,3%
- with miRBase (Triticum turgidum)	0	0,0%
- with miRBase (Vitis vinifera)	25	0,6%
- with miRBase (Zea mays)	72	1,6%
Unannotated	10 229 909	100,0%
Total	10 234 339	100,0%

Read count proportions

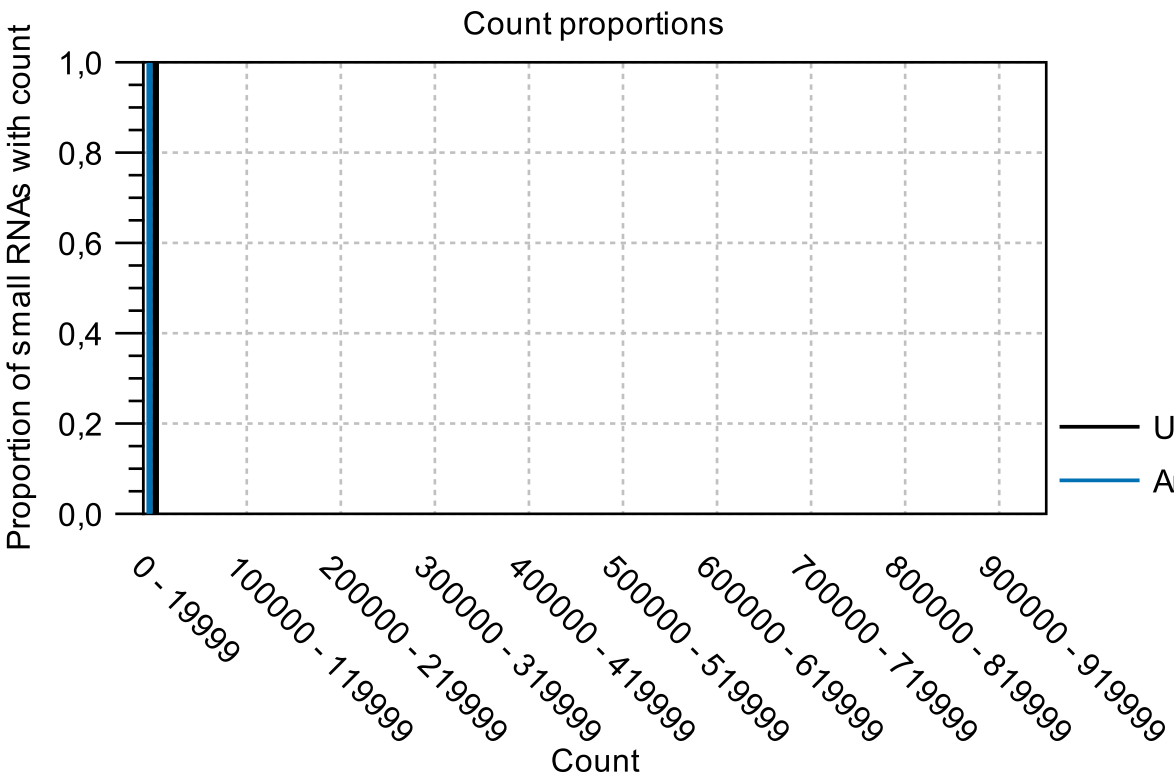

The histogram shows, for each interval of read counts, the proportion of annotated (respectively, unannotated) small RNAs with a read count in that interval. Annotated small RNAs may be expected to be associated with higher counts.

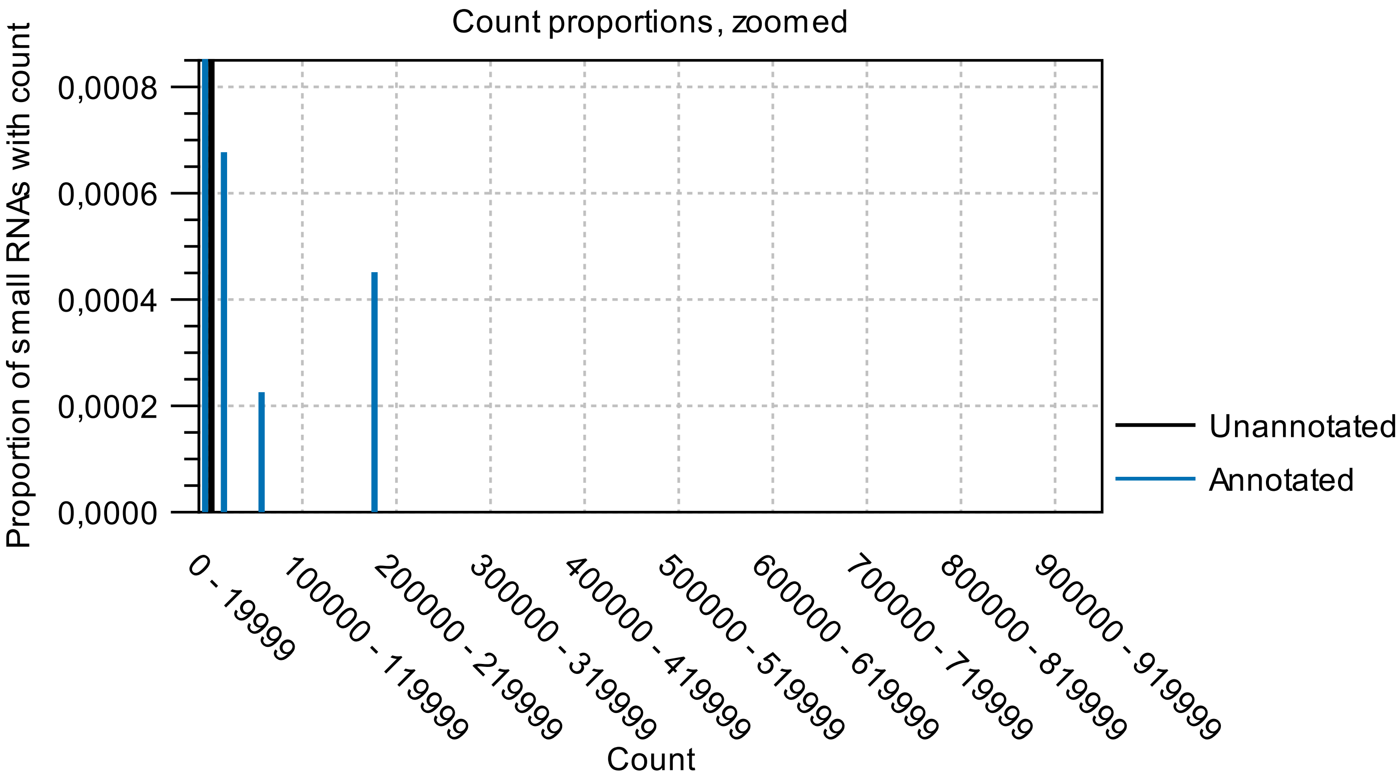

Zoomed version of the read count proportions histogram. Note that some bars extend beyond the plotting area.

3.5 Sample: 170110_SNK268_A_L004_JDG-1-5_R1 trimmed Small RNA sample

Reads

Annotation	Count	Percentage
Annotated	655 638	5,4%
- with miRBase (Aegilops tauschii)	308 775	47,1%
- with miRBase (Arabidopsis lyrata)	1 161	0,2%
- with miRBase (Arabidopsis thaliana)	2 846	0,4%
- with miRBase (Brachypodium distachyon)	13 561	2,1%
- with miRBase (Brassica napus)	339	0,1%
- with miRBase (Brassica oleracea)	0	0,0%
- with miRBase (Brassica rapa)	9	0,0%
- with miRBase (Caenorhabditis elegans)	1	0,0%
- with miRBase (Chlamydomonas reinhardtii)	1	0,0%
- with miRBase (Cucumis melo)	1 401	0,2%
- with miRBase (Glycine max)	195 103	29,8%
- with miRBase (Glycine soja)	0	0,0%
- with miRBase (Gossypium arboreum)	0	0,0%
- with miRBase (Gossypium herbaceum)	0	0,0%
- with miRBase (Gossypium hirsutum)	13	0,0%
- with miRBase (Gossypium raimondii)	366	0,1%
- with miRBase (Hordeum vulgare)	50 858	7,8%
- with miRBase (Medicago truncatula)	39	0,0%
- with miRBase (Nicotiana tabacum)	71	0,0%
- with miRBase (Oryza sativa)	59 481	9,1%
- with miRBase (Phaseolus vulgaris)	1 950	0,3%
- with miRBase (Physcomitrella patens)	4 053	0,6%
- with miRBase (Pinus densata)	10	0,0%
- with miRBase (Picea abies)	222	0,0%
- with miRBase (Prunus persica)	573	0,1%
- with miRBase (Solanum lycopersicum)	17	0,0%
- with miRBase (Solanum tuberosum)	359	0,1%

Annotation	Count	Percentage
- with miRBase (Sorghum bicolor)	454	0,1%
- with miRBase (Triticum aestivum)	13 471	2,1%
- with miRBase (Triticum turgidum)	0	0,0%
- with miRBase (Vitis vinifera)	49	0,0%
- with miRBase (Zea mays)	455	0,1%
Unannotated	11 512 639	94,6%
Total	12 168 277	100,0%

Small RNAs

Annotation	Count	Percentage
Annotated	4 430	0,0%
- with miRBase (Aegilops tauschii)	1 246	28,1%
- with miRBase (Arabidopsis lyrata)	129	2,9%
- with miRBase (Arabidopsis thaliana)	49	1,1%
- with miRBase (Brachypodium distachyon)	511	11,5%
- with miRBase (Brassica napus)	22	0,5%
- with miRBase (Brassica oleracea)	0	0,0%
- with miRBase (Brassica rapa)	5	0,1%
- with miRBase (Caenorhabditis elegans)	10	0,2%
- with miRBase (Chlamydomonas reinhardtii)	1	0,0%
- with miRBase (Cucumis melo)	50	1,1%
- with miRBase (Glycine max)	288	6,5%
- with miRBase (Glycine soja)	0	0,0%
- with miRBase (Gossypium arboreum)	0	0,0%
- with miRBase (Gossypium herbaceum)	0	0,0%
- with miRBase (Gossypium hirsutum)	8	0,2%
- with miRBase (Gossypium raimondii)	6	0,1%
- with miRBase (Hordeum vulgare)	795	17,9%
- with miRBase (Medicago truncatula)	28	0,6%
- with miRBase (Nicotiana tabacum)	22	0,5%
- with miRBase (Oryza sativa)	408	9,2%
- with miRBase (Phaseolus vulgaris)	8	0,2%
- with miRBase (Physcomitrella patens)	31	0,7%
- with miRBase (Pinus densata)	16	0,4%
- with miRBase (Picea abies)	44	1,0%
- with miRBase (Prunus persica)	16	0,4%

Annotation	Count	Percentage
- with miRBase (Solanum lycopersicum)	12	0,3%
- with miRBase (Solanum tuberosum)	21	0,5%
- with miRBase (Sorghum bicolor)	60	1,4%
- with miRBase (Triticum aestivum)	547	12,3%
- with miRBase (Triticum turgidum)	0	0,0%
- with miRBase (Vitis vinifera)	25	0,6%
- with miRBase (Zea mays)	72	1,6%
Unannotated	10 229 909	100,0%
Total	10 234 339	100,0%

Read count proportions

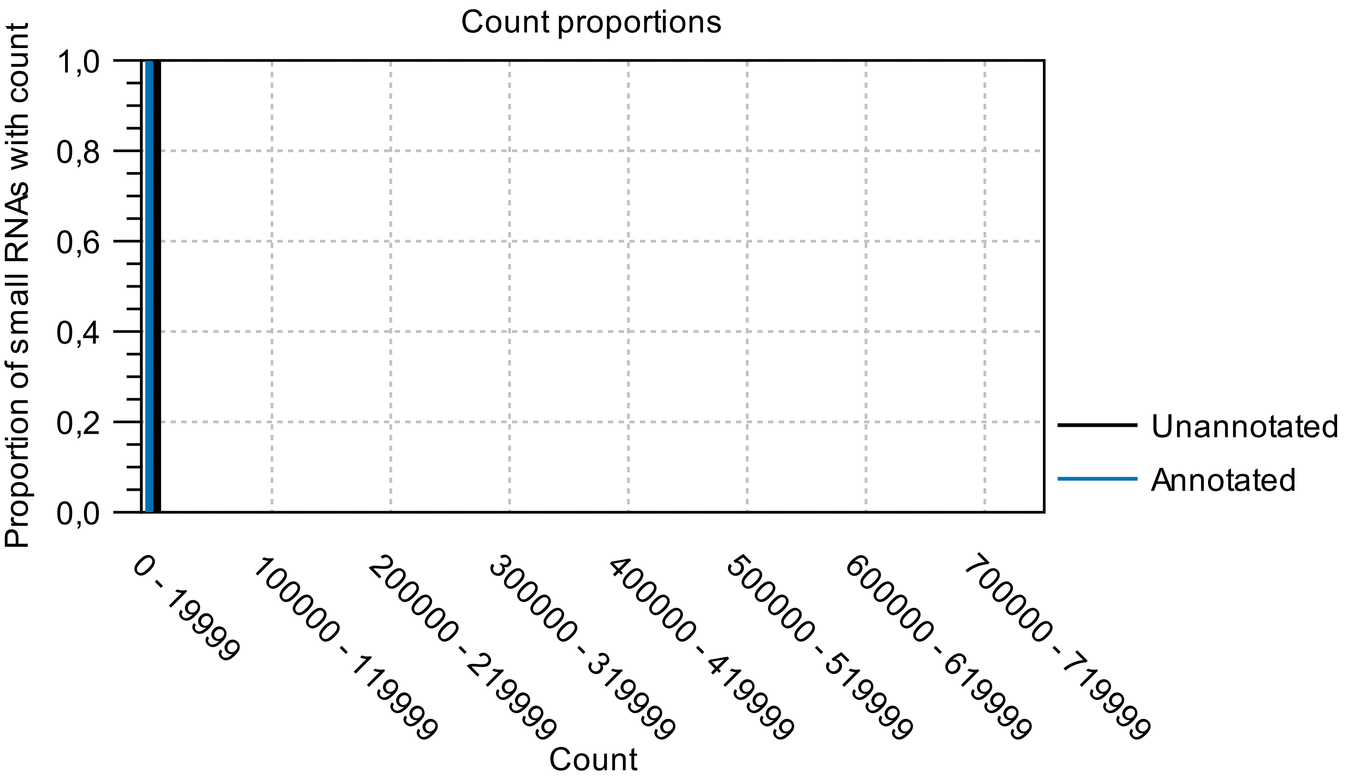

The histogram shows, for each interval of read counts, the proportion of annotated (respectively, unannotated) small RNAs with a read count in that interval. Annotated small RNAs may be expected to be associated with higher counts.

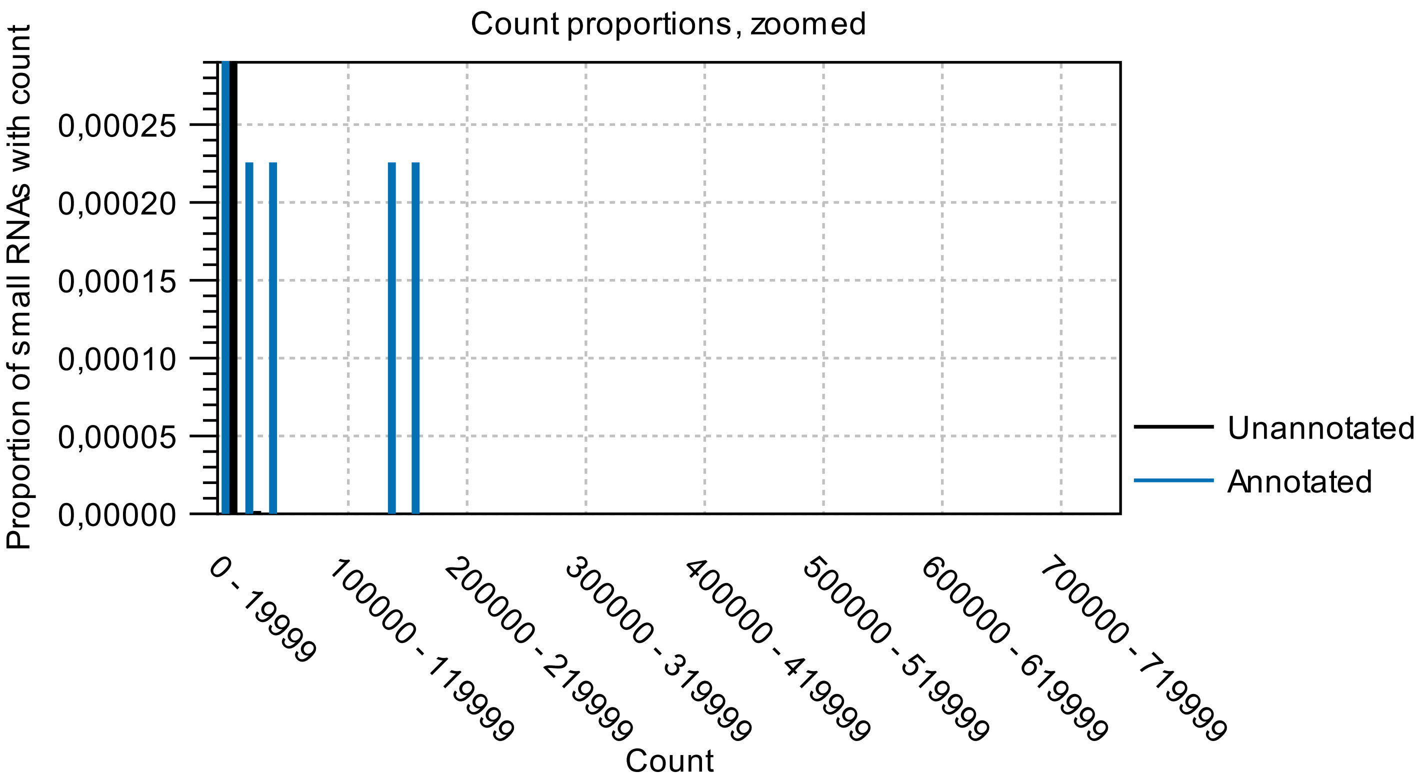

Zoomed version of the read count proportions histogram. Note that some bars extend beyond the plotting area.

3.6 Sample: 170110_SNK268_A_L004_JDG-1-6_R1 trimmed Small RNA sample

Reads

Annotation	Count	Percentage
Annotated	616 282	5,8%
- with miRBase (Aegilops tauschii)	324 672	52,7%
- with miRBase (Arabidopsis lyrata)	1 271	0,2%
- with miRBase (Arabidopsis thaliana)	2 177	0,4%
- with miRBase (Brachypodium distachyon)	12 903	2,1%
- with miRBase (Brassica napus)	243	0,0%
- with miRBase (Brassica oleracea)	0	0,0%
- with miRBase (Brassica rapa)	20	0,0%
- with miRBase (Caenorhabditis elegans)	49	0,0%
- with miRBase (Chlamydomonas reinhardtii)	2	0,0%
- with miRBase (Cucumis melo)	1 011	0,2%

Annotation	Count	Percentage
- with miRBase (Glycine max)	121 490	19,7%
- with miRBase (Glycine soja)	0	0,0%
- with miRBase (Gossypium arboreum)	0	0,0%
- with miRBase (Gossypium herbaceum)	0	0,0%
- with miRBase (Gossypium hirsutum)	10	0,0%
- with miRBase (Gossypium raimondii)	221	0,0%
- with miRBase (Hordeum vulgare)	93 596	15,2%
- with miRBase (Medicago truncatula)	43	0,0%
- with miRBase (Nicotiana tabacum)	85	0,0%
- with miRBase (Oryza sativa)	43 491	7,1%
- with miRBase (Phaseolus vulgaris)	1 896	0,3%
- with miRBase (Physcomitrella patens)	1 663	0,3%
- with miRBase (Pinus densata)	27	0,0%
- with miRBase (Picea abies)	156	0,0%
- with miRBase (Prunus persica)	617	0,1%
- with miRBase (Solanum lycopersicum)	15	0,0%
- with miRBase (Solanum tuberosum)	182	0,0%
- with miRBase (Sorghum bicolor)	351	0,1%
- with miRBase (Triticum aestivum)	9 084	1,5%
- with miRBase (Triticum turgidum)	0	0,0%
- with miRBase (Vitis vinifera)	52	0,0%
- with miRBase (Zea mays)	955	0,2%
Unannotated	9 949 343	94,2%
Total	10 565 625	100,0%

Small RNAs

Annotation	Count	Percentage
Annotated	4 430	0,0%
- with miRBase (Aegilops tauschii)	1 246	28,1%
- with miRBase (Arabidopsis lyrata)	129	2,9%
- with miRBase (Arabidopsis thaliana)	49	1,1%
- with miRBase (Brachypodium distachyon)	511	11,5%
- with miRBase (Brassica napus)	22	0,5%
- with miRBase (Brassica oleracea)	0	0,0%
- with miRBase (Brassica rapa)	5	0,1%
- with miRBase (Caenorhabditis elegans)	10	0,2%

Annotation	Count	Percentage
- with miRBase (Chlamydomonas reinhardtii)	1	0,0%
- with miRBase (Cucumis melo)	50	1,1%
- with miRBase (Glycine max)	288	6,5%
- with miRBase (Glycine soja)	0	0,0%
- with miRBase (Gossypium arboreum)	0	0,0%
- with miRBase (Gossypium herbaceum)	0	0,0%
- with miRBase (Gossypium hirsutum)	8	0,2%
- with miRBase (Gossypium raimondii)	6	0,1%
- with miRBase (Hordeum vulgare)	795	17,9%
- with miRBase (Medicago truncatula)	28	0,6%
- with miRBase (Nicotiana tabacum)	22	0,5%
- with miRBase (Oryza sativa)	408	9,2%
- with miRBase (Phaseolus vulgaris)	8	0,2%
- with miRBase (Physcomitrella patens)	31	0,7%
- with miRBase (Pinus densata)	16	0,4%
- with miRBase (Picea abies)	44	1,0%
- with miRBase (Prunus persica)	16	0,4%
- with miRBase (Solanum lycopersicum)	12	0,3%
- with miRBase (Solanum tuberosum)	21	0,5%
- with miRBase (Sorghum bicolor)	60	1,4%
- with miRBase (Triticum aestivum)	547	12,3%
- with miRBase (Triticum turgidum)	0	0,0%
- with miRBase (Vitis vinifera)	25	0,6%
- with miRBase (Zea mays)	72	1,6%
Unannotated	10 229 909	100,0%
Total	10 234 339	100,0%

Read count proportions

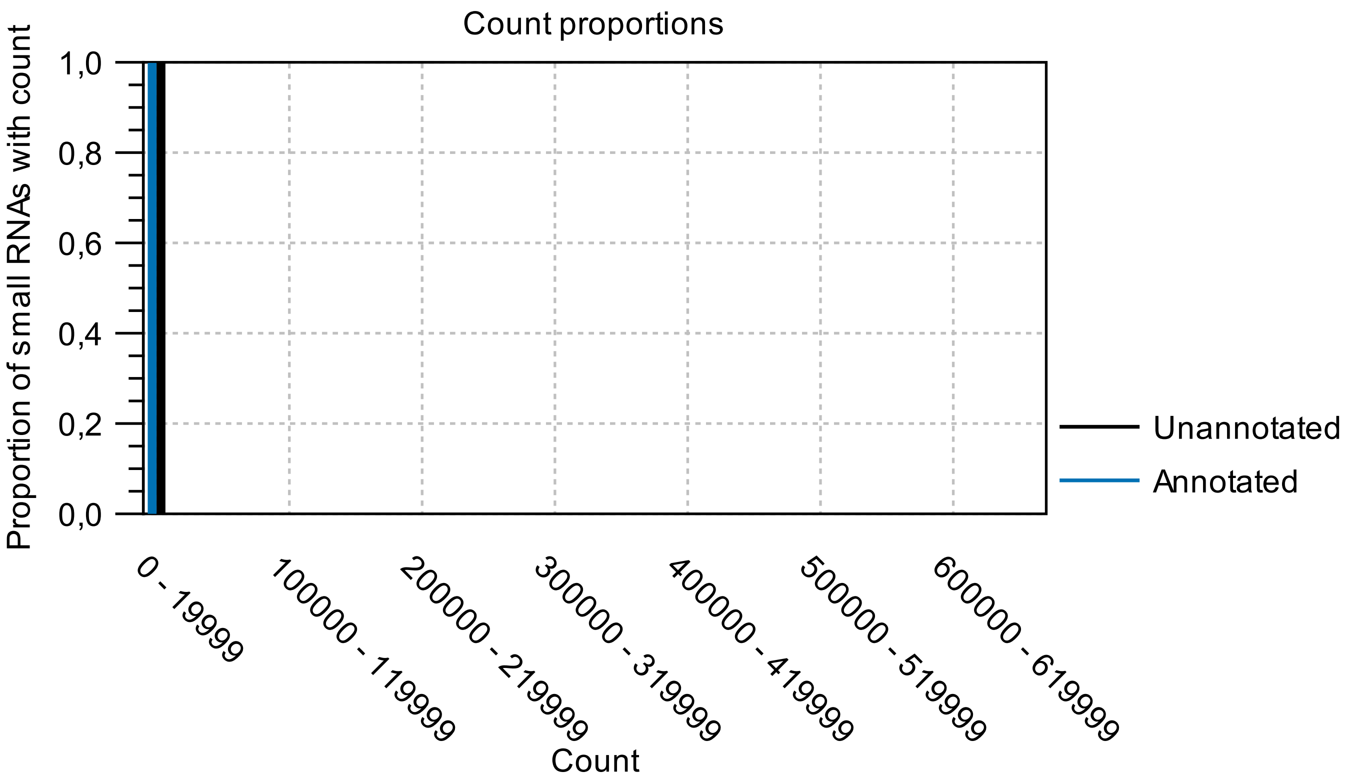

The histogram shows, for each interval of read counts, the proportion of annotated (respectively, unannotated) small RNAs with a read count in that interval. Annotated small RNAs may be expected to be associated with higher counts.

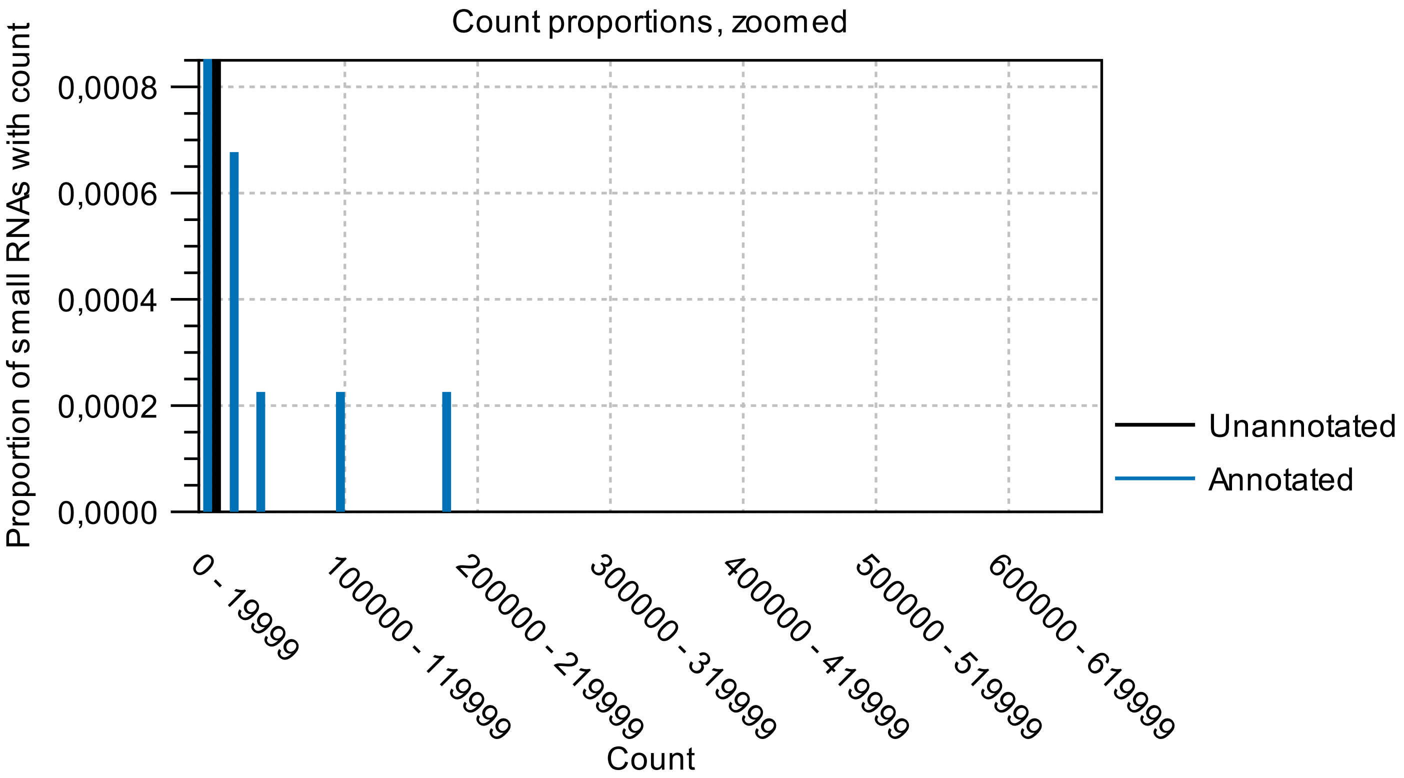

Zoomed version of the read count proportions histogram. Note that some bars extend beyond the plotting area.

4. Annotations (miRBase)

Organism	Total	Mature 5' total	Mature 5' exact matches	Mature 5' length variants
Aegilops tauschii	2 007 623	1 404 008	1 307 612	96 396
Arabidopsis lyrata	7 313	1 070	365	705
Arabidopsis thaliana	18 426	19	12	7
Brachypodium distachyon	74 563	28 136	16 024	12 112
Brassica napus	2 324	2 248	69	2 179
Brassica oleracea	0	0	0	0
Brassica rapa	117	115	109	6
Caenorhabditis elegans	52	45	24	21
Chlamydomonas reinhardtii	3	0	0	0
Cucumis melo	9 177	3 197	2 950	247
Glycine max	1 257 248	65 896	64 459	1 437
Glycine soja	0	0	0	0
Gossypium arboreum	0	0	0	0
Gossypium herbaceum	0	0	0	0
Gossypium hirsutum	55	53	9	44
Gossypium raimondii	2 258	0	0	0
Hordeum vulgare	425 103	372 321	196 270	176 051
Medicago truncatula	211	151	9	142
Nicotiana tabacum	442	407	368	39
Oryza sativa	315 130	17 203	489	16 714
Phaseolus vulgaris	6 187	6 187	5 988	199
Physcomitrella patens	20 405	9 949	4	9 945
Pinus densata	85	30	1	29
Picea abies	1 443	86	23	63
Prunus persica	3 854	30	6	24
Solanum lycopersicum	79	56	45	11
Solanum tuberosum	996	291	147	144
Sorghum bicolor	2 806	890	784	106
Triticum aestivum	64 725	2 737	2 417	320
Triticum turgidum	0	0	0	0

Organism	Total	Mature 5' total	Mature 5' exact matches	Mature 5' length variants
Vitis vinifera	272	141	33	108
Zea mays	3 853	1 111	998	113

Organism	Mature 5' mutant variants	Non-mature total	Mature 3'	Precursor
Aegilops tauschii	0	603 615	506 723	96 892
Arabidopsis lyrata	0	6 243	3 433	2 810
Arabidopsis thaliana	0	18 407	17 314	1 093
Brachypodium distachyon	0	46 427	28 267	18 160
Brassica napus	0	76	3	73
Brassica oleracea	0	0	0	0
Brassica rapa	0	2	0	2
Caenorhabditis elegans	0	7	0	7
Chlamydomonas reinhardtii	0	3	0	3
Cucumis melo	0	5 980	5 285	695
Glycine max	0	1 191 352	1 186 687	4 665
Glycine soja	0	0	0	0
Gossypium arboreum	0	0	0	0
Gossypium herbaceum	0	0	0	0
Gossypium hirsutum	0	2	1	1
Gossypium raimondii	0	2 258	0	2 258
Hordeum vulgare	0	52 782	25 319	27 463
Medicago truncatula	0	60	42	18
Nicotiana tabacum	0	35	24	11
Oryza sativa	0	297 927	157 090	140 837
Phaseolus vulgaris	0	0	0	0
Physcomitrella patens	0	10 456	259	10 197
Pinus densata	0	55	43	12
Picea abies	0	1 357	1 195	162
Prunus persica	0	3 824	0	3 824
Solanum lycopersicum	0	23	22	1
Solanum tuberosum	0	705	694	11
Sorghum bicolor	0	1 916	884	1 032
Triticum aestivum	0	61 988	54 884	7 104
Triticum turgidum	0	0	0	0
Vitis vinifera	0	131	70	61

Organism	Mature 5' mutant variants	Non-mature total	Mature 3'	Precursor
Zea mays	0	2 742	2 365	377
