# Supplementary material for: Pi-starvation induced transcriptional changes in barley revealed by a comprehensive RNA-Seq and degradome analyses
Source: BMC Genomics. 2021 Mar 9;22:165. doi: 10.1186/s12864-021-07481-w (PMC7941915; doi:10.1186/s12864-021-07481-w)
Supplement: Supplementary file 29 — Additional file 29. miRbase annotation report from CLC Workbench (QIAGEN) analysis of root small RNAs. [file 12864_2021_7481_MOESM29_ESM.pdf]

**Additional file 29.** Annotation report from CLC Workbench (QIAGEN) analysis of root small RNAs.

**Legend:**

Root, sufficient Pi, biological replicate #1 - 170110_SNK268_A_L004_JDG-1-7_R1 trimmed Small RNA sample

Root, sufficient Pi, biological replicate #2 - 170110_SNK268_A_L004_JDG-1-8_R1 trimmed Small RNA sample

Root, sufficient Pi, biological replicate #3 - 170110_SNK268_A_L004_JDG-1-9_R1 trimmed Small RNA sample

Root, low-Pi, biological replicate #1 - 170110_SNK268_A_L004_JDG-1-1_R1 trimmed Small RNA sample

Root, low-Pi, biological replicate #2 - 170110_SNK268_A_L004_JDG-1-2_R1 trimmed Small RNA sample

Root, low-Pi, biological replicate #3 - 170110_SNK268_A_L004_JDG-1-3_R1 trimmed Small RNA sample

QIAGEN

## Table of contents


1. Summary ........ 3
2. Resources ........ 4
3. Samples ........ 5
    3.1 Sample: 170110_SNK268_A_L004_JDG-1-7_R1 trimmed Small RNA sample ........ 6
    3.2 Sample: 170110_SNK268_A_L004_JDG-1-8_R1 trimmed Small RNA sample ........ 9
    3.3 Sample: 170110_SNK268_A_L004_JDG-1-9_R1 trimmed Small RNA sample ........ 13
    3.4 Sample: 170110_SNK268_A_L004_JDG-1-1_R1 trimmed Small RNA sample ........ 16
    3.5 Sample: 170110_SNK268_A_L004_JDG-1-2_R1 trimmed Small RNA sample ........ 20
    3.6 Sample: 170110_SNK268_A_L004_JDG-1-3_R1 trimmed Small RNA sample ........ 24
4. Annotations (miRBase) ........ 28

# 1. Summary

| Name | Small RNAs | Annotated | Percentage | Ambiguously annotated |
|---|---|---|---|---|
| 170110_SNK268_A_ L004_JDG-1-7_R1 trimmed Small RNA sample | 5 911 710 | 3 812 | 0,1% | 470 |
| 170110_SNK268_A_ L004_JDG-1-8_R1 trimmed Small RNA sample | 5 911 710 | 3 812 | 0,1% | 470 |
| 170110_SNK268_A_ L004_JDG-1-9_R1 trimmed Small RNA sample | 5 911 710 | 3 812 | 0,1% | 470 |
| 170110_SNK268_A_ L004_JDG-1-1_R1 trimmed Small RNA sample | 5 911 710 | 3 812 | 0,1% | 470 |
| 170110_SNK268_A_ L004_JDG-1-2_R1 trimmed Small RNA sample | 5 911 710 | 3 812 | 0,1% | 470 |
| 170110_SNK268_A_ L004_JDG-1-3_R1 trimmed Small RNA sample | 5 911 710 | 3 812 | 0,1% | 470 |

| Name | Percentage | Reads | Annotated | Percentage |
|---|---|---|---|---|
| 170110_SNK268_A_ L004_JDG-1-7_R1 trimmed Small RNA sample | 0,0% | 11 434 616 | 218 385 | 1,9% |
| 170110_SNK268_A_ L004_JDG-1-8_R1 trimmed Small RNA sample | 0,0% | 8 091 106 | 130 654 | 1,6% |
| 170110_SNK268_A_ L004_JDG-1-9_R1 trimmed Small RNA sample | 0,0% | 7 470 998 | 127 262 | 1,7% |
| 170110_SNK268_A_ L004_JDG-1-1_R1 trimmed Small RNA sample | 0,0% | 14 999 744 | 182 549 | 1,2% |

| Name | Percentage | Reads | Annotated | Percentage |
|---|---|---|---|---|
| 170110_SNK268_A_ L004_JDG-1-2_R1 trimmed Small RNA sample | 0,0% | 17 614 581 | 165 637 | 0,9% |
| 170110_SNK268_A_ L004_JDG-1-3_R1 trimmed Small RNA sample | 0,0% | 15 074 481 | 182 262 | 1,2% |

| Name | Ambiguously annotated | Percentage |
|---|---|---|
| 170110_SNK268_A_ L004_JDG-1-7_R1 trimmed Small RNA sample | 23 206 | 0,2% |
| 170110_SNK268_A_ L004_JDG-1-8_R1 trimmed Small RNA sample | 15 742 | 0,2% |
| 170110_SNK268_A_ L004_JDG-1-9_R1 trimmed Small RNA sample | 15 411 | 0,2% |
| 170110_SNK268_A_ L004_JDG-1-1_R1 trimmed Small RNA sample | 19 805 | 0,1% |
| 170110_SNK268_A_ L004_JDG-1-2_R1 trimmed Small RNA sample | 21 054 | 0,1% |
| 170110_SNK268_A_ L004_JDG-1-3_R1 trimmed Small RNA sample | 19 891 | 0,1% |

# 2. Resources

| Resource | Sequences in resource | Sequences found | Percentage found |
|---|---|---|---|
| miRBase (Aegilops tauschii) | 88 | 66 | 75,0% |
| miRBase (Arabidopsis lyrata) | 205 | 43 | 21,0% |
| miRBase (Arabidopsis thaliana) | 326 | 7 | 2,1% |
| miRBase (Brachypodium distachyon) | 317 | 72 | 22,7% |
| miRBase (Brassica napus) | 90 | 7 | 7,8% |
| miRBase (Brassica oleracea) | 10 | 0 | 0,0% |

| Resource | Sequences in resource | Sequences found | Percentage found |
|---|---|---|---|
| miRBase (Brassica rapa) | 96 | 4 | 4,2% |
| miRBase (Caenorhabditis elegans) | 253 | 1 | 0,4% |
| miRBase (Chlamydomonas reinhardtii) | 50 | 2 | 4,0% |
| miRBase (Cucumis melo) | 120 | 22 | 18,3% |
| miRBase (Glycine max) | 684 | 54 | 7,9% |
| miRBase (Glycine soja) | 13 | 0 | 0,0% |
| miRBase (Gossypium arboreum) | 1 | 0 | 0,0% |
| miRBase (Gossypium herbaceum) | 1 | 0 | 0,0% |
| miRBase (Gossypium hirsutum) | 78 | 5 | 6,4% |
| miRBase (Gossypium raimondii) | 296 | 3 | 1,0% |
| miRBase (Hordeum vulgare) | 69 | 57 | 82,6% |
| miRBase (Medicago truncatula) | 672 | 14 | 2,1% |
| miRBase (Nicotiana tabacum) | 162 | 7 | 4,3% |
| miRBase (Oryza sativa) | 604 | 64 | 10,6% |
| miRBase (Phaseolus vulgaris) | 8 | 1 | 12,5% |
| miRBase (Physcomitrella patens) | 247 | 16 | 6,5% |
| miRBase (Pinus densata) | 29 | 6 | 20,7% |
| miRBase (Picea abies) | 594 | 31 | 5,2% |
| miRBase (Prunus persica) | 180 | 3 | 1,7% |
| miRBase (Solanum lycopersicum) | 112 | 6 | 5,4% |
| miRBase (Solanum tuberosum) | 224 | 12 | 5,4% |
| miRBase (Sorghum bicolor) | 205 | 15 | 7,3% |
| miRBase (Triticum aestivum) | 122 | 39 | 32,0% |
| miRBase (Triticum turgidum) | 1 | 0 | 0,0% |
| miRBase (Vitis vinifera) | 163 | 12 | 7,4% |
| miRBase (Zea mays) | 174 | 22 | 12,6% |

# 3. Samples

## 3.1 Sample: 170110_SNK268_A_L004_JDG-1-7_R1 trimmed Small RNA sample

### *Reads*

| Annotation | Count | Percentage |
|---|---|---|
| Annotated | 218 385 | 1,9% |
| - with miRBase (Aegilops tauschii) | 80 347 | 36,8% |
| - with miRBase (Arabidopsis lyrata) | 3 389 | 1,6% |
| - with miRBase (Arabidopsis thaliana) | 3 397 | 1,6% |
| - with miRBase (Brachypodium distachyon) | 5 726 | 2,6% |
| - with miRBase (Brassica napus) | 51 | 0,0% |
| - with miRBase (Brassica oleracea) | 0 | 0,0% |
| - with miRBase (Brassica rapa) | 7 | 0,0% |
| - with miRBase (Caenorhabditis elegans) | 0 | 0,0% |
| - with miRBase (Chlamydomonas reinhardtii) | 0 | 0,0% |
| - with miRBase (Cucumis melo) | 784 | 0,4% |
| - with miRBase (Glycine max) | 83 931 | 38,4% |
| - with miRBase (Glycine soja) | 0 | 0,0% |
| - with miRBase (Gossypium arboreum) | 0 | 0,0% |
| - with miRBase (Gossypium herbaceum) | 0 | 0,0% |
| - with miRBase (Gossypium hirsutum) | 0 | 0,0% |
| - with miRBase (Gossypium raimondii) | 252 | 0,1% |
| - with miRBase (Hordeum vulgare) | 28 176 | 12,9% |
| - with miRBase (Medicago truncatula) | 10 | 0,0% |
| - with miRBase (Nicotiana tabacum) | 13 | 0,0% |
| - with miRBase (Oryza sativa) | 4 978 | 2,3% |
| - with miRBase (Phaseolus vulgaris) | 13 | 0,0% |
| - with miRBase (Physcomitrella patens) | 3 957 | 1,8% |
| - with miRBase (Pinus densata) | 0 | 0,0% |
| - with miRBase (Picea abies) | 133 | 0,1% |
| - with miRBase (Prunus persica) | 139 | 0,1% |
| - with miRBase (Solanum lycopersicum) | 1 | 0,0% |
| - with miRBase (Solanum tuberosum) | 57 | 0,0% |
| - with miRBase (Sorghum bicolor) | 73 | 0,0% |
| - with miRBase (Triticum aestivum) | 2 695 | 1,2% |
| - with miRBase (Triticum turgidum) | 0 | 0,0% |

| Annotation | Count | Percentage |
|---|---|---|
| - with miRBase (Vitis vinifera) | 16 | 0,0% |
| - with miRBase (Zea mays) | 240 | 0,1% |
| Unannotated | 11 216 231 | 98,1% |
| Total | 11 434 616 | 100,0% |

## *Small RNAs*

| Annotation | Count | Percentage |
|---|---|---|
| Annotated | 3 812 | 0,1% |
| - with miRBase (Aegilops tauschii) | 808 | 21,2% |
| - with miRBase (Arabidopsis lyrata) | 97 | 2,5% |
| - with miRBase (Arabidopsis thaliana) | 50 | 1,3% |
| - with miRBase (Brachypodium distachyon) | 395 | 10,4% |
| - with miRBase (Brassica napus) | 17 | 0,4% |
| - with miRBase (Brassica oleracea) | 0 | 0,0% |
| - with miRBase (Brassica rapa) | 5 | 0,1% |
| - with miRBase (Caenorhabditis elegans) | 1 | 0,0% |
| - with miRBase (Chlamydomonas reinhardtii) | 2 | 0,1% |
| - with miRBase (Cucumis melo) | 34 | 0,9% |
| - with miRBase (Glycine max) | 173 | 4,5% |
| - with miRBase (Glycine soja) | 0 | 0,0% |
| - with miRBase (Gossypium arboreum) | 0 | 0,0% |
| - with miRBase (Gossypium herbaceum) | 0 | 0,0% |
| - with miRBase (Gossypium hirsutum) | 4 | 0,1% |
| - with miRBase (Gossypium raimondii) | 10 | 0,3% |
| - with miRBase (Hordeum vulgare) | 1 318 | 34,6% |
| - with miRBase (Medicago truncatula) | 15 | 0,4% |
| - with miRBase (Nicotiana tabacum) | 13 | 0,3% |
| - with miRBase (Oryza sativa) | 302 | 7,9% |
| - with miRBase (Phaseolus vulgaris) | 4 | 0,1% |
| - with miRBase (Physcomitrella patens) | 32 | 0,8% |
| - with miRBase (Pinus densata) | 7 | 0,2% |
| - with miRBase (Picea abies) | 27 | 0,7% |
| - with miRBase (Prunus persica) | 6 | 0,2% |
| - with miRBase (Solanum lycopersicum) | 6 | 0,2% |
| - with miRBase (Solanum tuberosum) | 13 | 0,3% |
| - with miRBase (Sorghum bicolor) | 24 | 0,6% |

| Annotation | Count | Percentage |
|---|---|---|
| - with miRBase (Triticum aestivum) | 395 | 10,4% |
| - with miRBase (Triticum turgidum) | 0 | 0,0% |
| - with miRBase (Vitis vinifera) | 17 | 0,4% |
| - with miRBase (Zea mays) | 37 | 1,0% |
| Unannotated | 5 907 898 | 99,9% |
| Total | 5 911 710 | 100,0% |

### *Read count proportions*

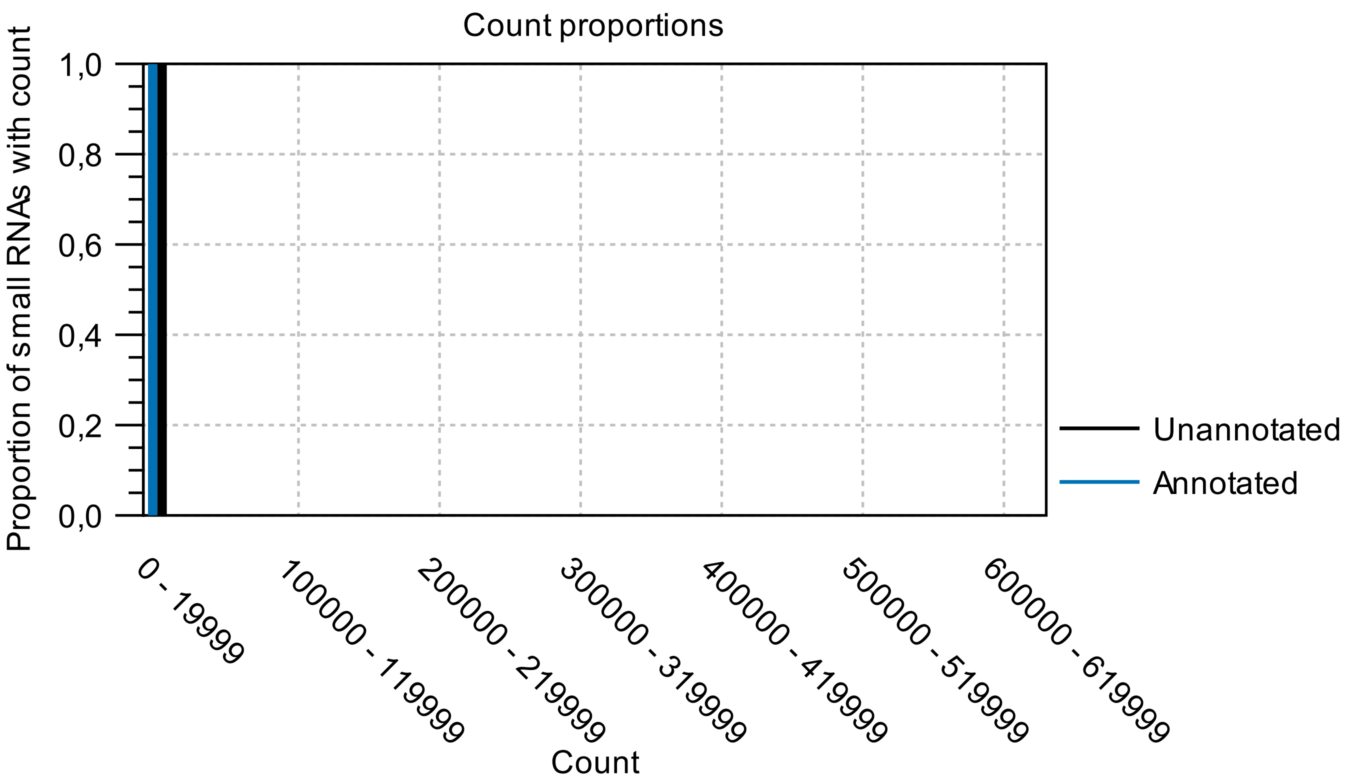


The histogram shows, for each interval of read counts, the proportion of annotated (respectively, unannotated) small RNAs with a read count in that interval. Annotated small RNAs may be expected to be associated with higher counts.

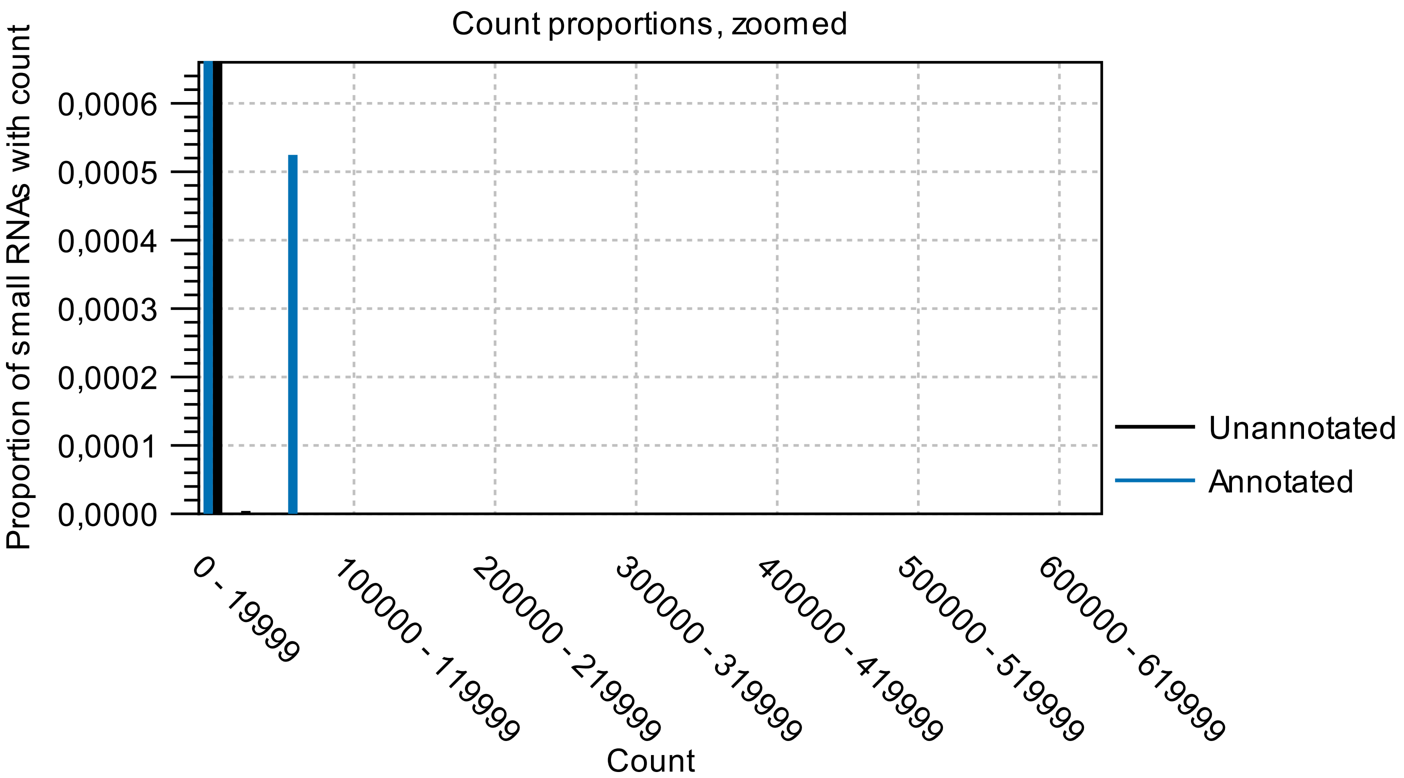


Zoomed version of the read count proportions histogram. Note that some bars extend beyond the plotting area.

## 3.2 Sample: 170110_SNK268_A_L004_JDG-1-8_R1 trimmed Small RNA sample

### *Reads*

| Annotation | Count | Percentage |
|---|---|---|
| Annotated | 130 654 | 1,6% |
| - with miRBase (Aegilops tauschii) | 47 422 | 36,3% |
| - with miRBase (Arabidopsis lyrata) | 1 734 | 1,3% |
| - with miRBase (Arabidopsis thaliana) | 2 640 | 2,0% |
| - with miRBase (Brachypodium distachyon) | 3 205 | 2,5% |
| - with miRBase (Brassica napus) | 30 | 0,0% |
| - with miRBase (Brassica oleracea) | 0 | 0,0% |
| - with miRBase (Brassica rapa) | 4 | 0,0% |
| - with miRBase (Caenorhabditis elegans) | 0 | 0,0% |
| - with miRBase (Chlamydomonas reinhardtii) | 1 | 0,0% |
| - with miRBase (Cucumis melo) | 645 | 0,5% |

| Annotation | Count | Percentage |
|---|---|---|
| - with miRBase (Glycine max) | 53 185 | 40,7% |
| - with miRBase (Glycine soja) | 0 | 0,0% |
| - with miRBase (Gossypium arboreum) | 0 | 0,0% |
| - with miRBase (Gossypium herbaceum) | 0 | 0,0% |
| - with miRBase (Gossypium hirsutum) | 1 | 0,0% |
| - with miRBase (Gossypium raimondii) | 286 | 0,2% |
| - with miRBase (Hordeum vulgare) | 11 643 | 8,9% |
| - with miRBase (Medicago truncatula) | 6 | 0,0% |
| - with miRBase (Nicotiana tabacum) | 19 | 0,0% |
| - with miRBase (Oryza sativa) | 5 119 | 3,9% |
| - with miRBase (Phaseolus vulgaris) | 12 | 0,0% |
| - with miRBase (Physcomitrella patens) | 2 132 | 1,6% |
| - with miRBase (Pinus densata) | 10 | 0,0% |
| - with miRBase (Picea abies) | 216 | 0,2% |
| - with miRBase (Prunus persica) | 96 | 0,1% |
| - with miRBase (Solanum lycopersicum) | 3 | 0,0% |
| - with miRBase (Solanum tuberosum) | 44 | 0,0% |
| - with miRBase (Sorghum bicolor) | 49 | 0,0% |
| - with miRBase (Triticum aestivum) | 1 902 | 1,5% |
| - with miRBase (Triticum turgidum) | 0 | 0,0% |
| - with miRBase (Vitis vinifera) | 15 | 0,0% |
| - with miRBase (Zea mays) | 235 | 0,2% |
| Unannotated | 7 960 452 | 98,4% |
| Total | 8 091 106 | 100,0% |

## *Small RNAs*

| Annotation | Count | Percentage |
|---|---|---|
| Annotated | 3 812 | 0,1% |
| - with miRBase (Aegilops tauschii) | 808 | 21,2% |
| - with miRBase (Arabidopsis lyrata) | 97 | 2,5% |
| - with miRBase (Arabidopsis thaliana) | 50 | 1,3% |
| - with miRBase (Brachypodium distachyon) | 395 | 10,4% |
| - with miRBase (Brassica napus) | 17 | 0,4% |
| - with miRBase (Brassica oleracea) | 0 | 0,0% |
| - with miRBase (Brassica rapa) | 5 | 0,1% |
| - with miRBase (Caenorhabditis elegans) | 1 | 0,0% |

| Annotation | Count | Percentage |
|---|---|---|
| - with miRBase (Chlamydomonas reinhardtii) | 2 | 0,1% |
| - with miRBase (Cucumis melo) | 34 | 0,9% |
| - with miRBase (Glycine max) | 173 | 4,5% |
| - with miRBase (Glycine soja) | 0 | 0,0% |
| - with miRBase (Gossypium arboreum) | 0 | 0,0% |
| - with miRBase (Gossypium herbaceum) | 0 | 0,0% |
| - with miRBase (Gossypium hirsutum) | 4 | 0,1% |
| - with miRBase (Gossypium raimondii) | 10 | 0,3% |
| - with miRBase (Hordeum vulgare) | 1 318 | 34,6% |
| - with miRBase (Medicago truncatula) | 15 | 0,4% |
| - with miRBase (Nicotiana tabacum) | 13 | 0,3% |
| - with miRBase (Oryza sativa) | 302 | 7,9% |
| - with miRBase (Phaseolus vulgaris) | 4 | 0,1% |
| - with miRBase (Physcomitrella patens) | 32 | 0,8% |
| - with miRBase (Pinus densata) | 7 | 0,2% |
| - with miRBase (Picea abies) | 27 | 0,7% |
| - with miRBase (Prunus persica) | 6 | 0,2% |
| - with miRBase (Solanum lycopersicum) | 6 | 0,2% |
| - with miRBase (Solanum tuberosum) | 13 | 0,3% |
| - with miRBase (Sorghum bicolor) | 24 | 0,6% |
| - with miRBase (Triticum aestivum) | 395 | 10,4% |
| - with miRBase (Triticum turgidum) | 0 | 0,0% |
| - with miRBase (Vitis vinifera) | 17 | 0,4% |
| - with miRBase (Zea mays) | 37 | 1,0% |
| Unannotated | 5 907 898 | 99,9% |
| Total | 5 911 710 | 100,0% |

## *Read count proportions*

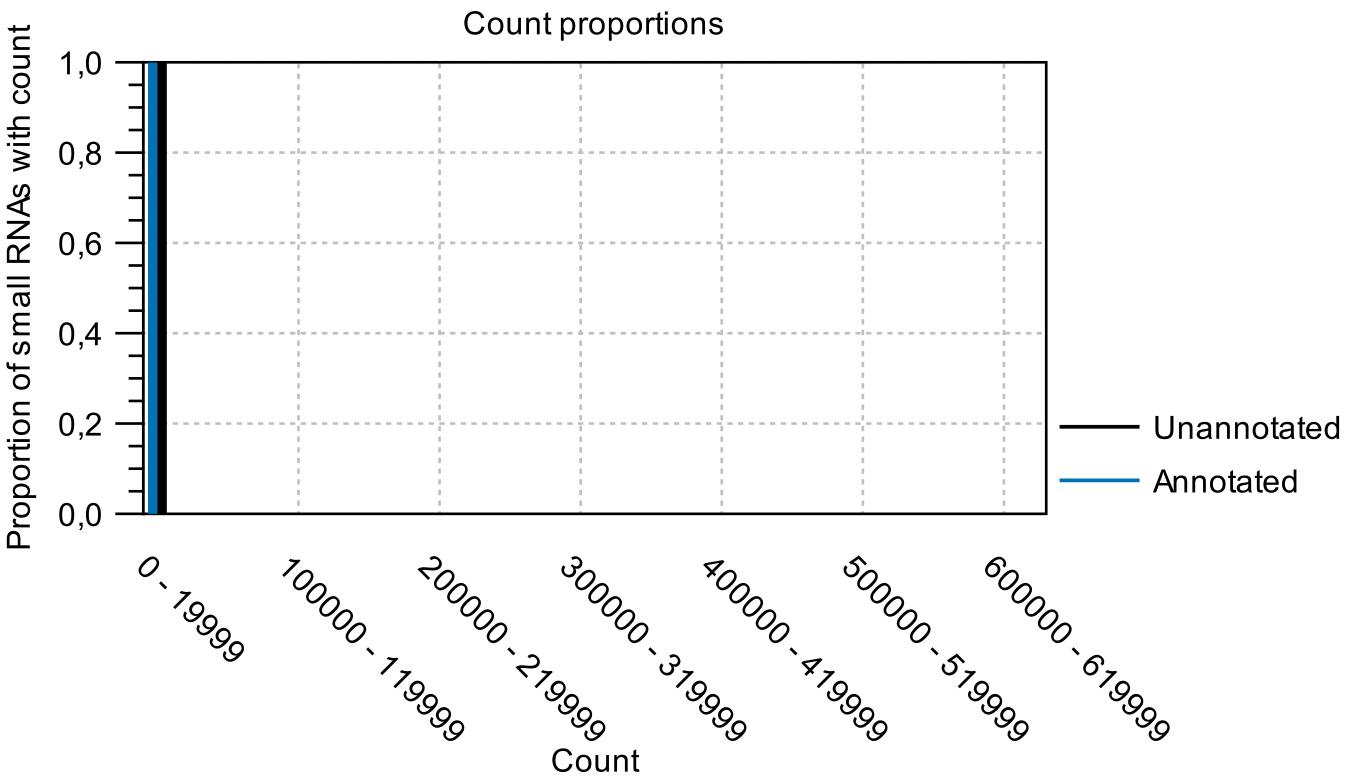


The histogram shows, for each interval of read counts, the proportion of annotated (respectively, unannotated) small RNAs with a read count in that interval. Annotated small RNAs may be expected to be associated with higher counts.

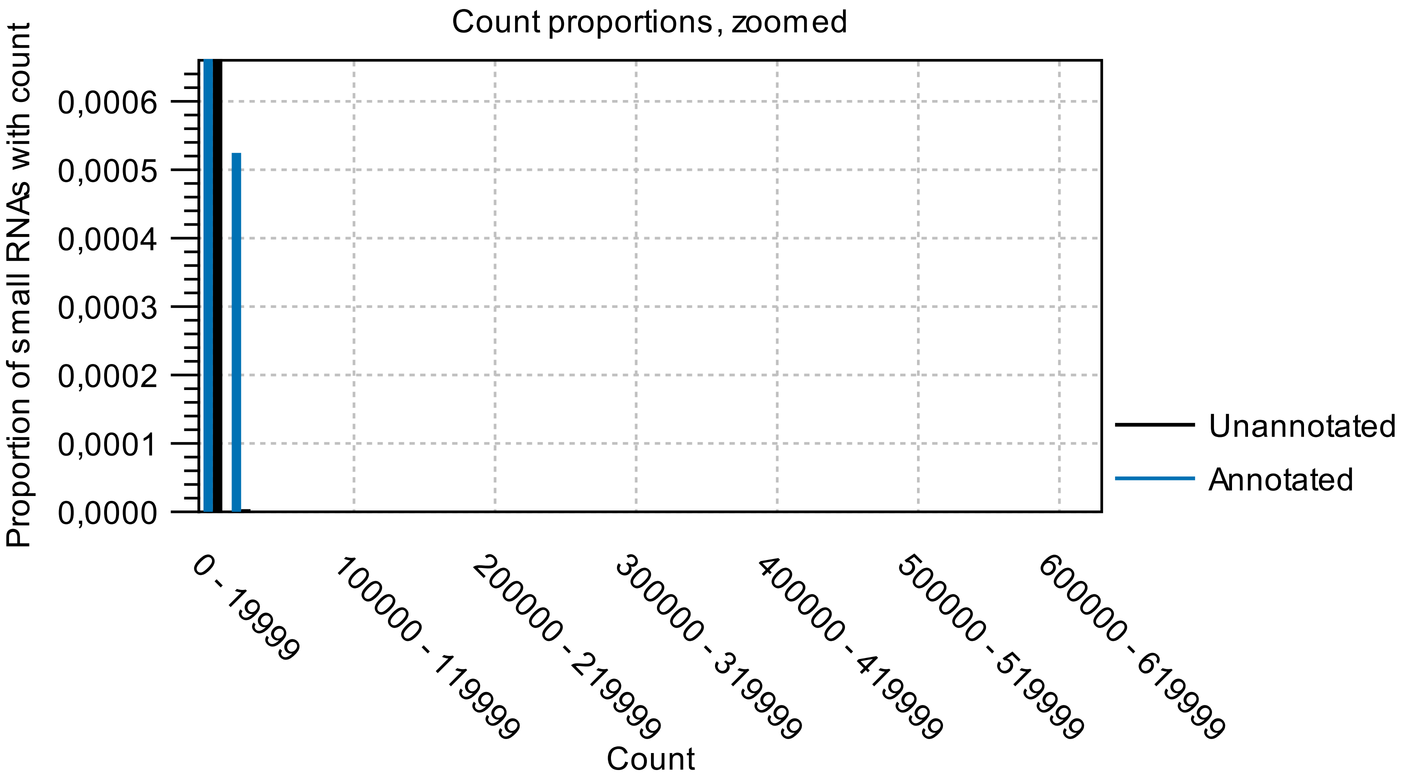

Zoomed version of the read count proportions histogram. Note that some bars extend beyond the plotting area.

## 3.3 Sample: 170110_SNK268_A_L004_JDG-1-9_R1 trimmed Small RNA sample

### *Reads*

| Annotation | Count | Percentage |
|---|---|---|
| Annotated | 127 262 | 1,7% |
| - with miRBase (Aegilops tauschii) | 50 961 | 40,0% |
| - with miRBase (Arabidopsis lyrata) | 1 955 | 1,5% |
| - with miRBase (Arabidopsis thaliana) | 3 525 | 2,8% |
| - with miRBase (Brachypodium distachyon) | 3 564 | 2,8% |
| - with miRBase (Brassica napus) | 41 | 0,0% |
| - with miRBase (Brassica oleracea) | 0 | 0,0% |
| - with miRBase (Brassica rapa) | 4 | 0,0% |
| - with miRBase (Caenorhabditis elegans) | 0 | 0,0% |
| - with miRBase (Chlamydomonas reinhardtii) | 1 | 0,0% |
| - with miRBase (Cucumis melo) | 461 | 0,4% |
| - with miRBase (Glycine max) | 42 981 | 33,8% |
| - with miRBase (Glycine soja) | 0 | 0,0% |
| - with miRBase (Gossypium arboreum) | 0 | 0,0% |
| - with miRBase (Gossypium herbaceum) | 0 | 0,0% |
| - with miRBase (Gossypium hirsutum) | 0 | 0,0% |
| - with miRBase (Gossypium raimondii) | 181 | 0,1% |
| - with miRBase (Hordeum vulgare) | 15 147 | 11,9% |
| - with miRBase (Medicago truncatula) | 8 | 0,0% |
| - with miRBase (Nicotiana tabacum) | 20 | 0,0% |
| - with miRBase (Oryza sativa) | 4 177 | 3,3% |
| - with miRBase (Phaseolus vulgaris) | 17 | 0,0% |
| - with miRBase (Physcomitrella patens) | 1 747 | 1,4% |
| - with miRBase (Pinus densata) | 0 | 0,0% |
| - with miRBase (Picea abies) | 106 | 0,1% |
| - with miRBase (Prunus persica) | 109 | 0,1% |
| - with miRBase (Solanum lycopersicum) | 3 | 0,0% |
| - with miRBase (Solanum tuberosum) | 39 | 0,0% |

| Annotation | Count | Percentage |
|---|---|---|
| - with miRBase (Sorghum bicolor) | 46 | 0,0% |
| - with miRBase (Triticum aestivum) | 1 924 | 1,5% |
| - with miRBase (Triticum turgidum) | 0 | 0,0% |
| - with miRBase (Vitis vinifera) | 14 | 0,0% |
| - with miRBase (Zea mays) | 231 | 0,2% |
| Unannotated | 7 343 736 | 98,3% |
| Total | 7 470 998 | 100,0% |

## *Small RNAs*

| Annotation | Count | Percentage |
|---|---|---|
| Annotated | 3 812 | 0,1% |
| - with miRBase (Aegilops tauschii) | 808 | 21,2% |
| - with miRBase (Arabidopsis lyrata) | 97 | 2,5% |
| - with miRBase (Arabidopsis thaliana) | 50 | 1,3% |
| - with miRBase (Brachypodium distachyon) | 395 | 10,4% |
| - with miRBase (Brassica napus) | 17 | 0,4% |
| - with miRBase (Brassica oleracea) | 0 | 0,0% |
| - with miRBase (Brassica rapa) | 5 | 0,1% |
| - with miRBase (Caenorhabditis elegans) | 1 | 0,0% |
| - with miRBase (Chlamydomonas reinhardtii) | 2 | 0,1% |
| - with miRBase (Cucumis melo) | 34 | 0,9% |
| - with miRBase (Glycine max) | 173 | 4,5% |
| - with miRBase (Glycine soja) | 0 | 0,0% |
| - with miRBase (Gossypium arboreum) | 0 | 0,0% |
| - with miRBase (Gossypium herbaceum) | 0 | 0,0% |
| - with miRBase (Gossypium hirsutum) | 4 | 0,1% |
| - with miRBase (Gossypium raimondii) | 10 | 0,3% |
| - with miRBase (Hordeum vulgare) | 1 318 | 34,6% |
| - with miRBase (Medicago truncatula) | 15 | 0,4% |
| - with miRBase (Nicotiana tabacum) | 13 | 0,3% |
| - with miRBase (Oryza sativa) | 302 | 7,9% |
| - with miRBase (Phaseolus vulgaris) | 4 | 0,1% |
| - with miRBase (Physcomitrella patens) | 32 | 0,8% |
| - with miRBase (Pinus densata) | 7 | 0,2% |
| - with miRBase (Picea abies) | 27 | 0,7% |
| - with miRBase (Prunus persica) | 6 | 0,2% |

| Annotation | Count | Percentage |
|---|---|---|
| - with miRBase (Solanum lycopersicum) | 6 | 0,2% |
| - with miRBase (Solanum tuberosum) | 13 | 0,3% |
| - with miRBase (Sorghum bicolor) | 24 | 0,6% |
| - with miRBase (Triticum aestivum) | 395 | 10,4% |
| - with miRBase (Triticum turgidum) | 0 | 0,0% |
| - with miRBase (Vitis vinifera) | 17 | 0,4% |
| - with miRBase (Zea mays) | 37 | 1,0% |
| Unannotated | 5 907 898 | 99,9% |
| Total | 5 911 710 | 100,0% |

## *Read count proportions*

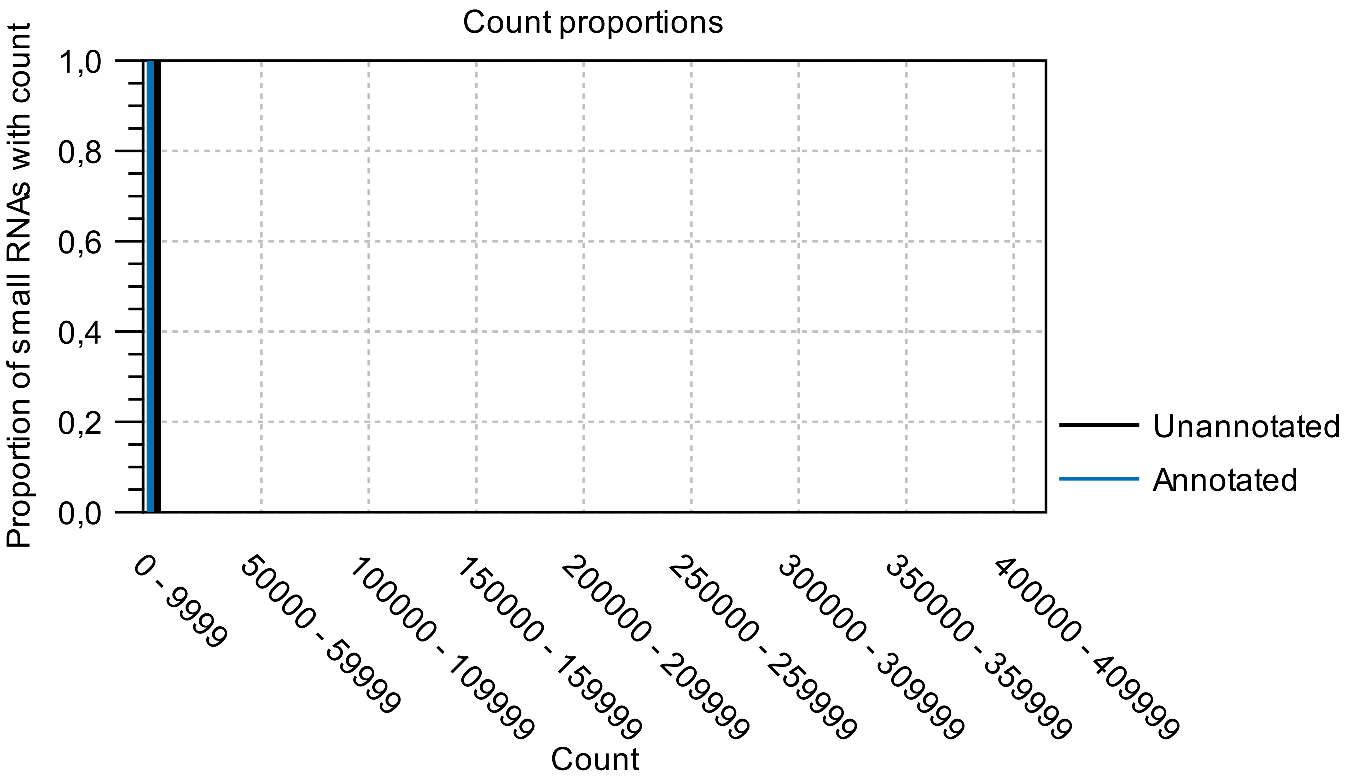


The histogram shows, for each interval of read counts, the proportion of annotated (respectively, unannotated) small RNAs with a read count in that interval. Annotated small RNAs may be expected to be associated with higher counts.

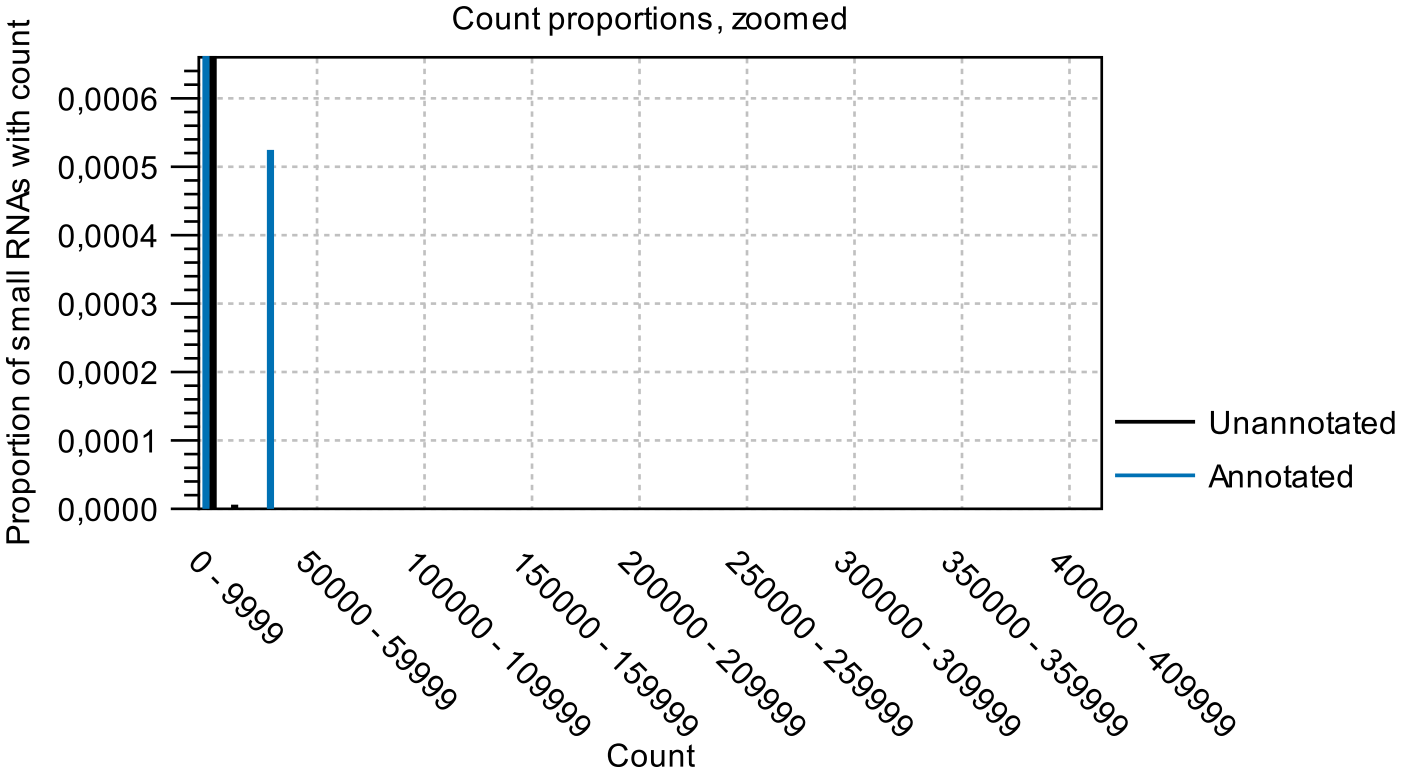


Zoomed version of the read count proportions histogram. Note that some bars extend beyond the plotting area.

## 3.4 Sample: 170110_SNK268_A_L004_JDG-1-1_R1 trimmed Small RNA sample

*Reads*

| Annotation | Count | Percentage |
|---|---|---|
| Annotated | 182 549 | 1,2% |
| - with miRBase (Aegilops tauschii) | 62 239 | 34,1% |
| - with miRBase (Arabidopsis lyrata) | 3 921 | 2,1% |
| - with miRBase (Arabidopsis thaliana) | 6 198 | 3,4% |
| - with miRBase (Brachypodium distachyon) | 5 247 | 2,9% |
| - with miRBase (Brassica napus) | 72 | 0,0% |
| - with miRBase (Brassica oleracea) | 0 | 0,0% |
| - with miRBase (Brassica rapa) | 3 | 0,0% |
| - with miRBase (Caenorhabditis elegans) | 1 | 0,0% |
| - with miRBase (Chlamydomonas reinhardtii) | 3 | 0,0% |
| - with miRBase (Cucumis melo) | 883 | 0,5% |

| Annotation | Count | Percentage |
|---|---|---|
| - with miRBase (Glycine max) | 72 167 | 39,5% |
| - with miRBase (Glycine soja) | 0 | 0,0% |
| - with miRBase (Gossypium arboreum) | 0 | 0,0% |
| - with miRBase (Gossypium herbaceum) | 0 | 0,0% |
| - with miRBase (Gossypium hirsutum) | 3 | 0,0% |
| - with miRBase (Gossypium raimondii) | 239 | 0,1% |
| - with miRBase (Hordeum vulgare) | 18 236 | 10,0% |
| - with miRBase (Medicago truncatula) | 10 | 0,0% |
| - with miRBase (Nicotiana tabacum) | 14 | 0,0% |
| - with miRBase (Oryza sativa) | 5 226 | 2,9% |
| - with miRBase (Phaseolus vulgaris) | 48 | 0,0% |
| - with miRBase (Physcomitrella patens) | 4 014 | 2,2% |
| - with miRBase (Pinus densata) | 2 | 0,0% |
| - with miRBase (Picea abies) | 169 | 0,1% |
| - with miRBase (Prunus persica) | 582 | 0,3% |
| - with miRBase (Solanum lycopersicum) | 3 | 0,0% |
| - with miRBase (Solanum tuberosum) | 61 | 0,0% |
| - with miRBase (Sorghum bicolor) | 40 | 0,0% |
| - with miRBase (Triticum aestivum) | 2 959 | 1,6% |
| - with miRBase (Triticum turgidum) | 0 | 0,0% |
| - with miRBase (Vitis vinifera) | 17 | 0,0% |
| - with miRBase (Zea mays) | 192 | 0,1% |
| Unannotated | 14 817 195 | 98,8% |
| Total | 14 999 744 | 100,0% |

## *Small RNAs*

| Annotation | Count | Percentage |
|---|---|---|
| Annotated | 3 812 | 0,1% |
| - with miRBase (Aegilops tauschii) | 808 | 21,2% |
| - with miRBase (Arabidopsis lyrata) | 97 | 2,5% |
| - with miRBase (Arabidopsis thaliana) | 50 | 1,3% |
| - with miRBase (Brachypodium distachyon) | 395 | 10,4% |
| - with miRBase (Brassica napus) | 17 | 0,4% |
| - with miRBase (Brassica oleracea) | 0 | 0,0% |
| - with miRBase (Brassica rapa) | 5 | 0,1% |
| - with miRBase (Caenorhabditis elegans) | 1 | 0,0% |

| Annotation | Count | Percentage |
|---|---|---|
| - with miRBase (Chlamydomonas reinhardtii) | 2 | 0,1% |
| - with miRBase (Cucumis melo) | 34 | 0,9% |
| - with miRBase (Glycine max) | 173 | 4,5% |
| - with miRBase (Glycine soja) | 0 | 0,0% |
| - with miRBase (Gossypium arboreum) | 0 | 0,0% |
| - with miRBase (Gossypium herbaceum) | 0 | 0,0% |
| - with miRBase (Gossypium hirsutum) | 4 | 0,1% |
| - with miRBase (Gossypium raimondii) | 10 | 0,3% |
| - with miRBase (Hordeum vulgare) | 1 318 | 34,6% |
| - with miRBase (Medicago truncatula) | 15 | 0,4% |
| - with miRBase (Nicotiana tabacum) | 13 | 0,3% |
| - with miRBase (Oryza sativa) | 302 | 7,9% |
| - with miRBase (Phaseolus vulgaris) | 4 | 0,1% |
| - with miRBase (Physcomitrella patens) | 32 | 0,8% |
| - with miRBase (Pinus densata) | 7 | 0,2% |
| - with miRBase (Picea abies) | 27 | 0,7% |
| - with miRBase (Prunus persica) | 6 | 0,2% |
| - with miRBase (Solanum lycopersicum) | 6 | 0,2% |
| - with miRBase (Solanum tuberosum) | 13 | 0,3% |
| - with miRBase (Sorghum bicolor) | 24 | 0,6% |
| - with miRBase (Triticum aestivum) | 395 | 10,4% |
| - with miRBase (Triticum turgidum) | 0 | 0,0% |
| - with miRBase (Vitis vinifera) | 17 | 0,4% |
| - with miRBase (Zea mays) | 37 | 1,0% |
| Unannotated | 5 907 898 | 99,9% |
| Total | 5 911 710 | 100,0% |

## *Read count proportions*

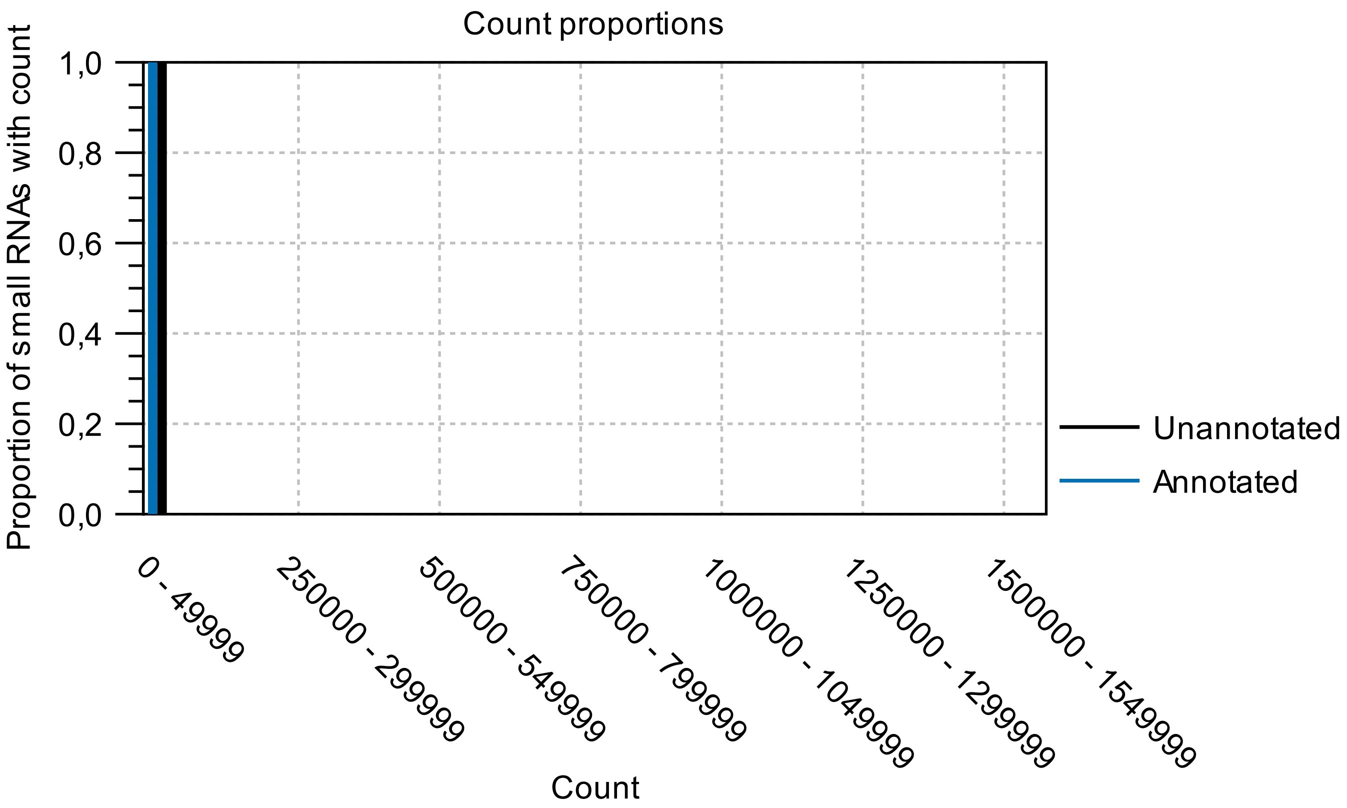


The histogram shows, for each interval of read counts, the proportion of annotated (respectively, unannotated) small RNAs with a read count in that interval. Annotated small RNAs may be expected to be associated with higher counts.

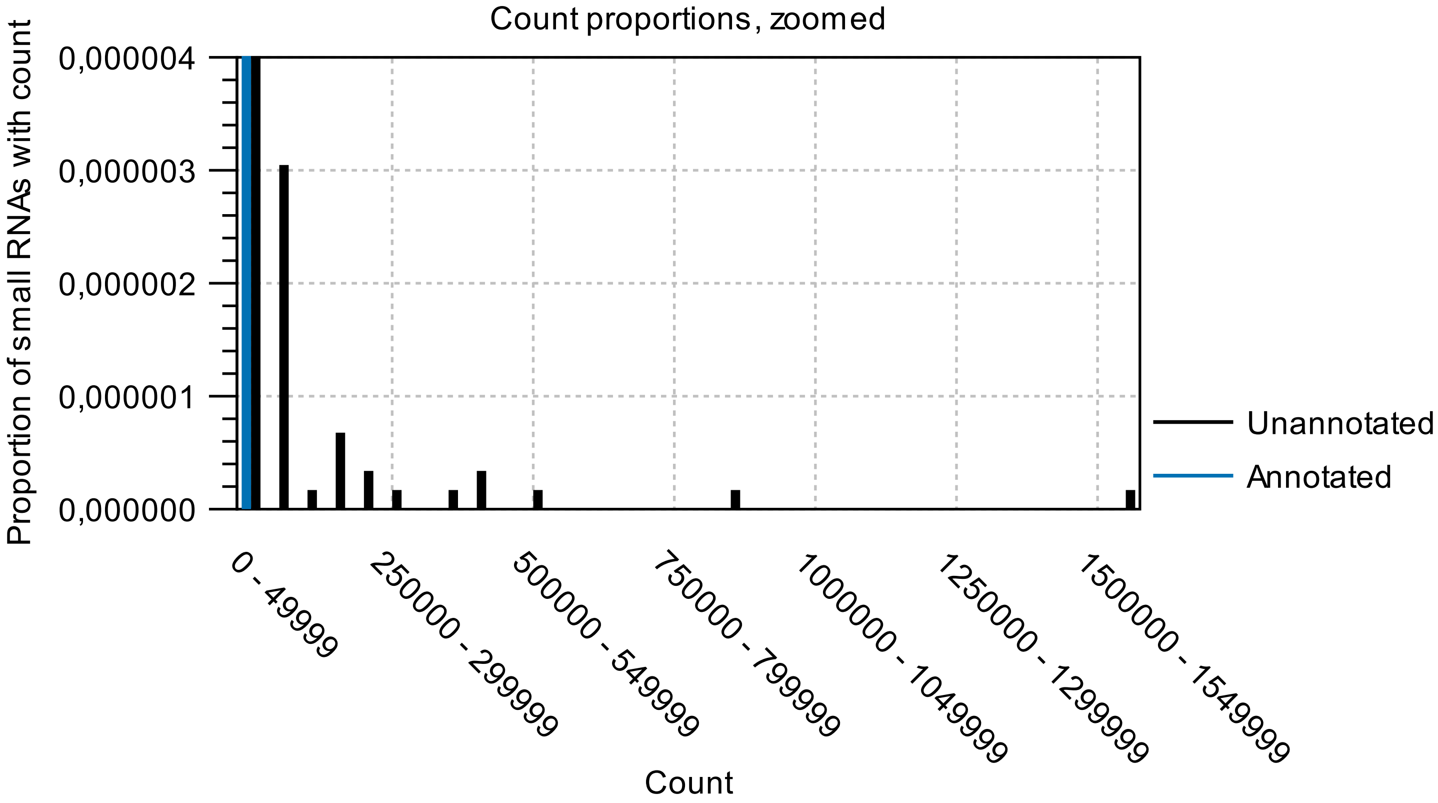


Zoomed version of the read count proportions histogram. Note that some bars extend beyond the plotting area.

## 3.5 Sample: 170110_SNK268_A_L004_JDG-1-2_R1 trimmed Small RNA sample

### *Reads*

| Annotation | Count | Percentage |
|---|---|---|
| Annotated | 165 637 | 0,9% |
| - with miRBase (Aegilops tauschii) | 57 423 | 34,7% |
| - with miRBase (Arabidopsis lyrata) | 3 356 | 2,0% |
| - with miRBase (Arabidopsis thaliana) | 5 261 | 3,2% |
| - with miRBase (Brachypodium distachyon) | 4 319 | 2,6% |
| - with miRBase (Brassica napus) | 69 | 0,0% |
| - with miRBase (Brassica oleracea) | 0 | 0,0% |
| - with miRBase (Brassica rapa) | 5 | 0,0% |
| - with miRBase (Caenorhabditis elegans) | 0 | 0,0% |
| - with miRBase (Chlamydomonas reinhardtii) | 0 | 0,0% |

| Annotation | Count | Percentage |
|---|---|---|
| - with miRBase (Cucumis melo) | 1 258 | 0,8% |
| - with miRBase (Glycine max) | 60 826 | 36,7% |
| - with miRBase (Glycine soja) | 0 | 0,0% |
| - with miRBase (Gossypium arboreum) | 0 | 0,0% |
| - with miRBase (Gossypium herbaceum) | 0 | 0,0% |
| - with miRBase (Gossypium hirsutum) | 1 | 0,0% |
| - with miRBase (Gossypium raimondii) | 269 | 0,2% |
| - with miRBase (Hordeum vulgare) | 18 539 | 11,2% |
| - with miRBase (Medicago truncatula) | 6 | 0,0% |
| - with miRBase (Nicotiana tabacum) | 17 | 0,0% |
| - with miRBase (Oryza sativa) | 6 767 | 4,1% |
| - with miRBase (Phaseolus vulgaris) | 54 | 0,0% |
| - with miRBase (Physcomitrella patens) | 3 179 | 1,9% |
| - with miRBase (Pinus densata) | 3 | 0,0% |
| - with miRBase (Picea abies) | 173 | 0,1% |
| - with miRBase (Prunus persica) | 389 | 0,2% |
| - with miRBase (Solanum lycopersicum) | 3 | 0,0% |
| - with miRBase (Solanum tuberosum) | 48 | 0,0% |
| - with miRBase (Sorghum bicolor) | 37 | 0,0% |
| - with miRBase (Triticum aestivum) | 3 449 | 2,1% |
| - with miRBase (Triticum turgidum) | 0 | 0,0% |
| - with miRBase (Vitis vinifera) | 13 | 0,0% |
| - with miRBase (Zea mays) | 173 | 0,1% |
| Unannotated | 17 448 944 | 99,1% |
| Total | 17 614 581 | 100,0% |

## *Small RNAs*

| Annotation | Count | Percentage |
|---|---|---|
| Annotated | 3 812 | 0,1% |
| - with miRBase (Aegilops tauschii) | 808 | 21,2% |
| - with miRBase (Arabidopsis lyrata) | 97 | 2,5% |
| - with miRBase (Arabidopsis thaliana) | 50 | 1,3% |
| - with miRBase (Brachypodium distachyon) | 395 | 10,4% |
| - with miRBase (Brassica napus) | 17 | 0,4% |
| - with miRBase (Brassica oleracea) | 0 | 0,0% |
| - with miRBase (Brassica rapa) | 5 | 0,1% |
| - with miRBase (Caenorhabditis elegans) | 1 | 0,0% |

| Annotation | Count | Percentage |
| --- | --- | --- |
| - with miRBase (Chlamydomonas reinhardtii) | 2 | 0,1% |
| - with miRBase (Cucumis melo) | 34 | 0,9% |
| - with miRBase (Glycine max) | 173 | 4,5% |
| - with miRBase (Glycine soja) | 0 | 0,0% |
| - with miRBase (Gossypium arboreum) | 0 | 0,0% |
| - with miRBase (Gossypium herbaceum) | 0 | 0,0% |
| - with miRBase (Gossypium hirsutum) | 4 | 0,1% |
| - with miRBase (Gossypium raimondii) | 10 | 0,3% |
| - with miRBase (Hordeum vulgare) | 1 318 | 34,6% |
| - with miRBase (Medicago truncatula) | 15 | 0,4% |
| - with miRBase (Nicotiana tabacum) | 13 | 0,3% |
| - with miRBase (Oryza sativa) | 302 | 7,9% |
| - with miRBase (Phaseolus vulgaris) | 4 | 0,1% |
| - with miRBase (Physcomitrella patens) | 32 | 0,8% |
| - with miRBase (Pinus densata) | 7 | 0,2% |
| - with miRBase (Picea abies) | 27 | 0,7% |
| - with miRBase (Prunus persica) | 6 | 0,2% |
| - with miRBase (Solanum lycopersicum) | 6 | 0,2% |
| - with miRBase (Solanum tuberosum) | 13 | 0,3% |
| - with miRBase (Sorghum bicolor) | 24 | 0,6% |
| - with miRBase (Triticum aestivum) | 395 | 10,4% |
| - with miRBase (Triticum turgidum) | 0 | 0,0% |
| - with miRBase (Vitis vinifera) | 17 | 0,4% |
| - with miRBase (Zea mays) | 37 | 1,0% |
| Unannotated | 5 907 898 | 99,9% |
| Total | 5 911 710 | 100,0% |

## *Read count proportions*

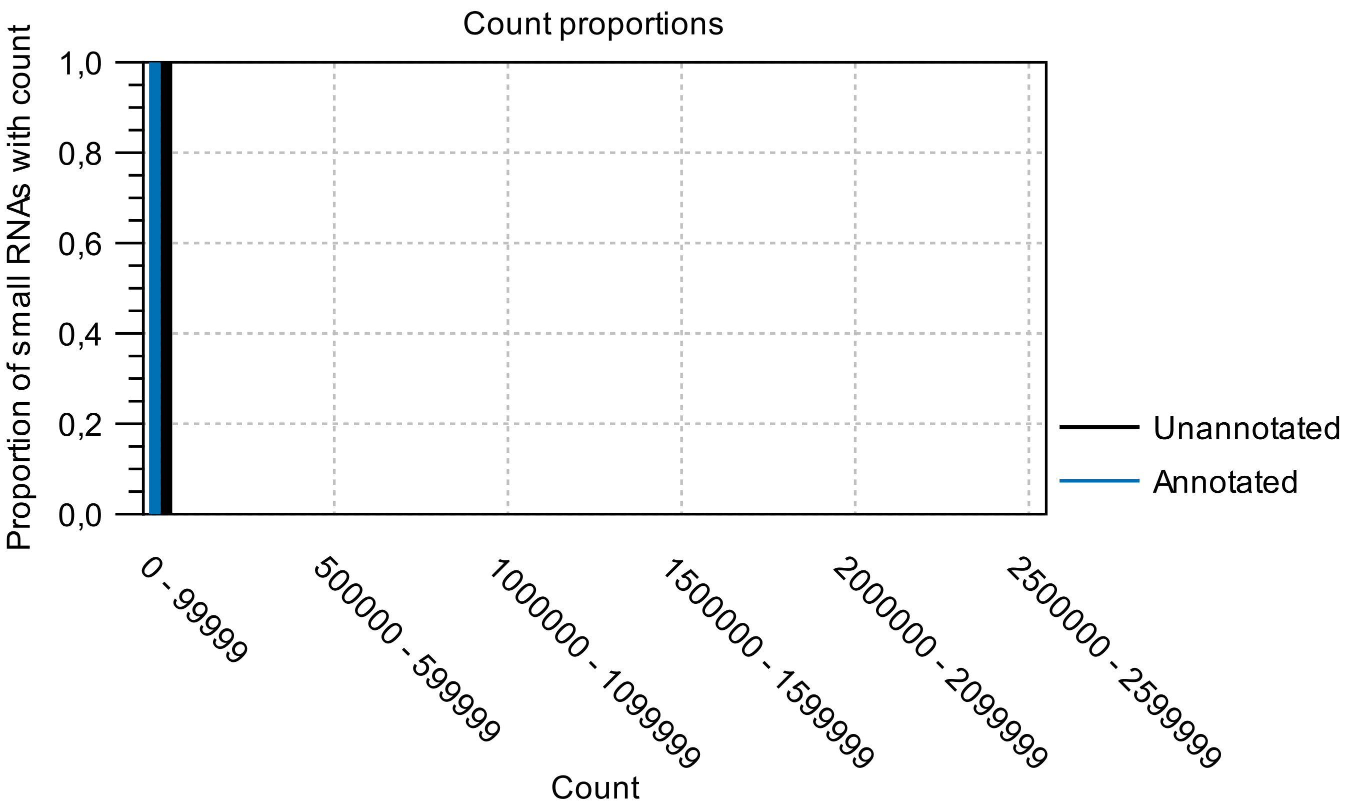


The histogram shows, for each interval of read counts, the proportion of annotated (respectively, unannotated) small RNAs with a read count in that interval. Annotated small RNAs may be expected to be associated with higher counts.

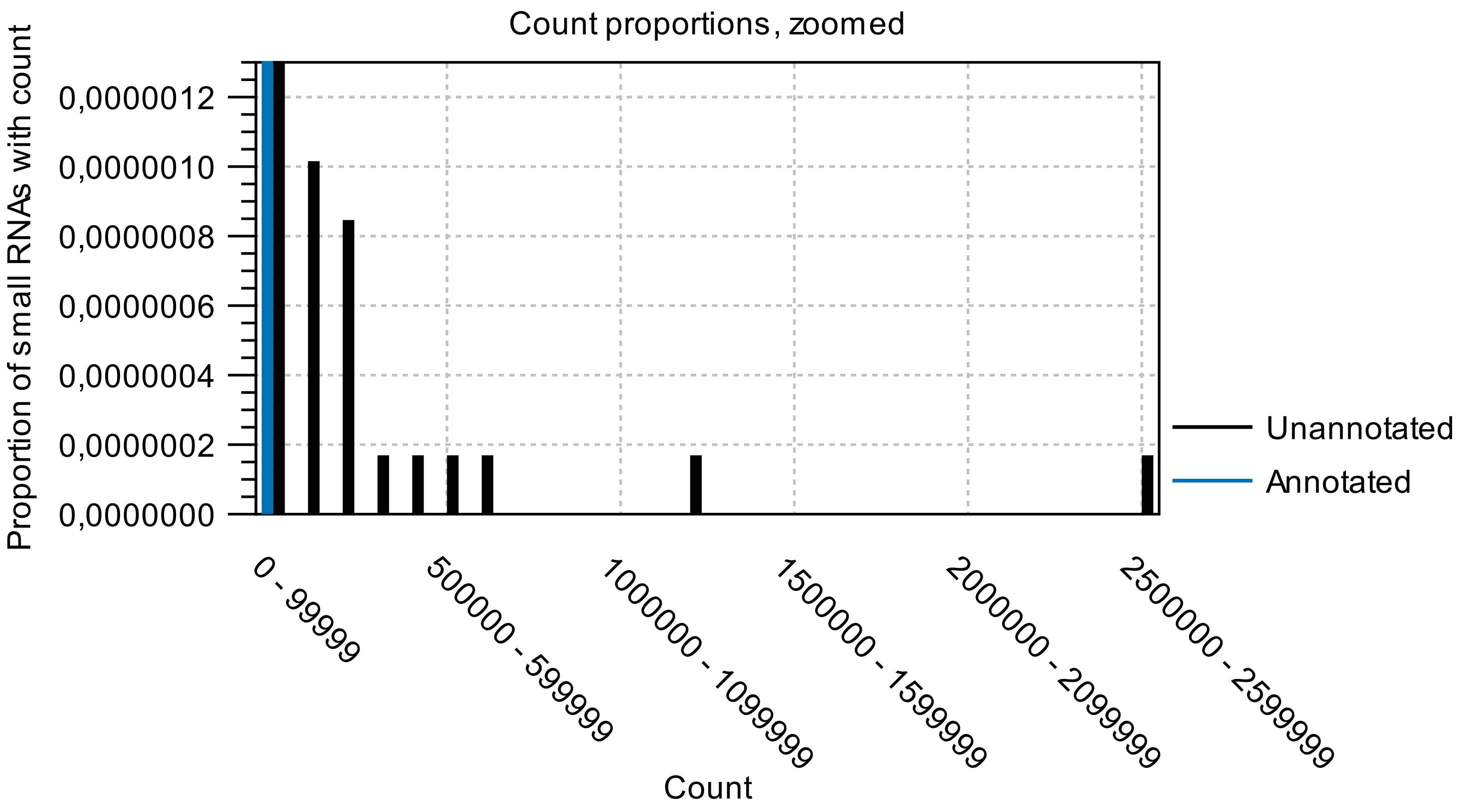


Zoomed version of the read count proportions histogram. Note that some bars extend beyond the plotting area.

## 3.6 Sample: 170110_SNK268_A_L004_JDG-1-3_R1 trimmed Small RNA sample

*Reads*

| Annotation | Count | Percentage |
|---|---|---|
| Annotated | 182 262 | 1,2% |
| - with miRBase (Aegilops tauschii) | 63 597 | 34,9% |
| - with miRBase (Arabidopsis lyrata) | 2 658 | 1,5% |
| - with miRBase (Arabidopsis thaliana) | 7 842 | 4,3% |
| - with miRBase (Brachypodium distachyon) | 4 947 | 2,7% |
| - with miRBase (Brassica napus) | 76 | 0,0% |
| - with miRBase (Brassica oleracea) | 0 | 0,0% |
| - with miRBase (Brassica rapa) | 4 | 0,0% |
| - with miRBase (Caenorhabditis elegans) | 0 | 0,0% |
| - with miRBase (Chlamydomonas reinhardtii) | 0 | 0,0% |

| Annotation | Count | Percentage |
|---|---|---|
| - with miRBase (Cucumis melo) | 958 | 0,5% |
| - with miRBase (Glycine max) | 73 950 | 40,6% |
| - with miRBase (Glycine soja) | 0 | 0,0% |
| - with miRBase (Gossypium arboreum) | 0 | 0,0% |
| - with miRBase (Gossypium herbaceum) | 0 | 0,0% |
| - with miRBase (Gossypium hirsutum) | 1 | 0,0% |
| - with miRBase (Gossypium raimondii) | 260 | 0,1% |
| - with miRBase (Hordeum vulgare) | 15 526 | 8,5% |
| - with miRBase (Medicago truncatula) | 7 | 0,0% |
| - with miRBase (Nicotiana tabacum) | 18 | 0,0% |
| - with miRBase (Oryza sativa) | 5 453 | 3,0% |
| - with miRBase (Phaseolus vulgaris) | 63 | 0,0% |
| - with miRBase (Physcomitrella patens) | 3 492 | 1,9% |
| - with miRBase (Pinus densata) | 3 | 0,0% |
| - with miRBase (Picea abies) | 128 | 0,1% |
| - with miRBase (Prunus persica) | 403 | 0,2% |
| - with miRBase (Solanum lycopersicum) | 5 | 0,0% |
| - with miRBase (Solanum tuberosum) | 47 | 0,0% |
| - with miRBase (Sorghum bicolor) | 31 | 0,0% |
| - with miRBase (Triticum aestivum) | 2 532 | 1,4% |
| - with miRBase (Triticum turgidum) | 0 | 0,0% |
| - with miRBase (Vitis vinifera) | 15 | 0,0% |
| - with miRBase (Zea mays) | 246 | 0,1% |
| Unannotated | 14 892 219 | 98,8% |
| Total | 15 074 481 | 100,0% |

## *Small RNAs*

| Annotation | Count | Percentage |
|---|---|---|
| Annotated | 3 812 | 0,1% |
| - with miRBase (Aegilops tauschii) | 808 | 21,2% |
| - with miRBase (Arabidopsis lyrata) | 97 | 2,5% |
| - with miRBase (Arabidopsis thaliana) | 50 | 1,3% |
| - with miRBase (Brachypodium distachyon) | 395 | 10,4% |
| - with miRBase (Brassica napus) | 17 | 0,4% |
| - with miRBase (Brassica oleracea) | 0 | 0,0% |
| - with miRBase (Brassica rapa) | 5 | 0,1% |
| - with miRBase (Caenorhabditis elegans) | 1 | 0,0% |

| Annotation | Count | Percentage |
|---|---|---|
| - with miRBase (Chlamydomonas reinhardtii) | 2 | 0,1% |
| - with miRBase (Cucumis melo) | 34 | 0,9% |
| - with miRBase (Glycine max) | 173 | 4,5% |
| - with miRBase (Glycine soja) | 0 | 0,0% |
| - with miRBase (Gossypium arboreum) | 0 | 0,0% |
| - with miRBase (Gossypium herbaceum) | 0 | 0,0% |
| - with miRBase (Gossypium hirsutum) | 4 | 0,1% |
| - with miRBase (Gossypium raimondii) | 10 | 0,3% |
| - with miRBase (Hordeum vulgare) | 1 318 | 34,6% |
| - with miRBase (Medicago truncatula) | 15 | 0,4% |
| - with miRBase (Nicotiana tabacum) | 13 | 0,3% |
| - with miRBase (Oryza sativa) | 302 | 7,9% |
| - with miRBase (Phaseolus vulgaris) | 4 | 0,1% |
| - with miRBase (Physcomitrella patens) | 32 | 0,8% |
| - with miRBase (Pinus densata) | 7 | 0,2% |
| - with miRBase (Picea abies) | 27 | 0,7% |
| - with miRBase (Prunus persica) | 6 | 0,2% |
| - with miRBase (Solanum lycopersicum) | 6 | 0,2% |
| - with miRBase (Solanum tuberosum) | 13 | 0,3% |
| - with miRBase (Sorghum bicolor) | 24 | 0,6% |
| - with miRBase (Triticum aestivum) | 395 | 10,4% |
| - with miRBase (Triticum turgidum) | 0 | 0,0% |
| - with miRBase (Vitis vinifera) | 17 | 0,4% |
| - with miRBase (Zea mays) | 37 | 1,0% |
| Unannotated | 5 907 898 | 99,9% |
| Total | 5 911 710 | 100,0% |

## *Read count proportions*

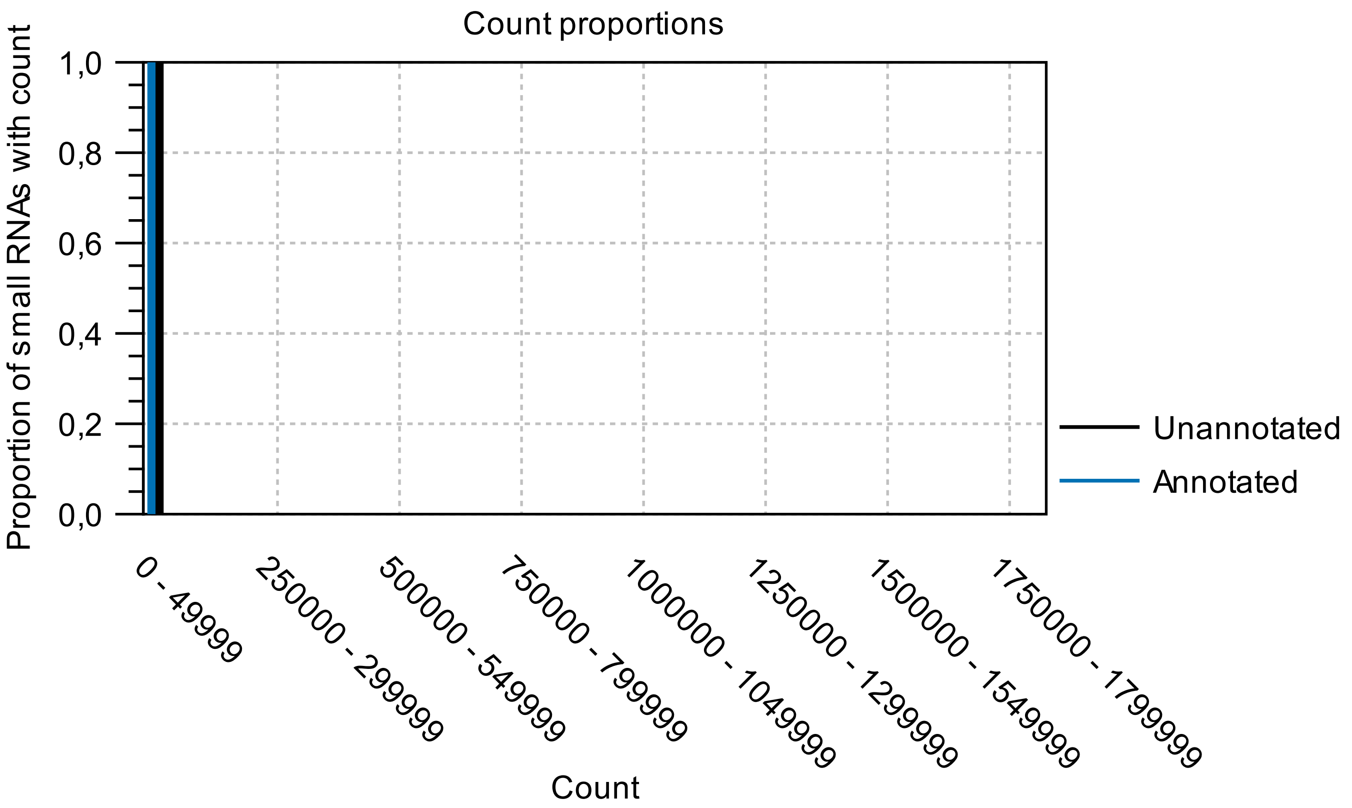


The histogram shows, for each interval of read counts, the proportion of annotated (respectively, unannotated) small RNAs with a read count in that interval. Annotated small RNAs may be expected to be associated with higher counts.

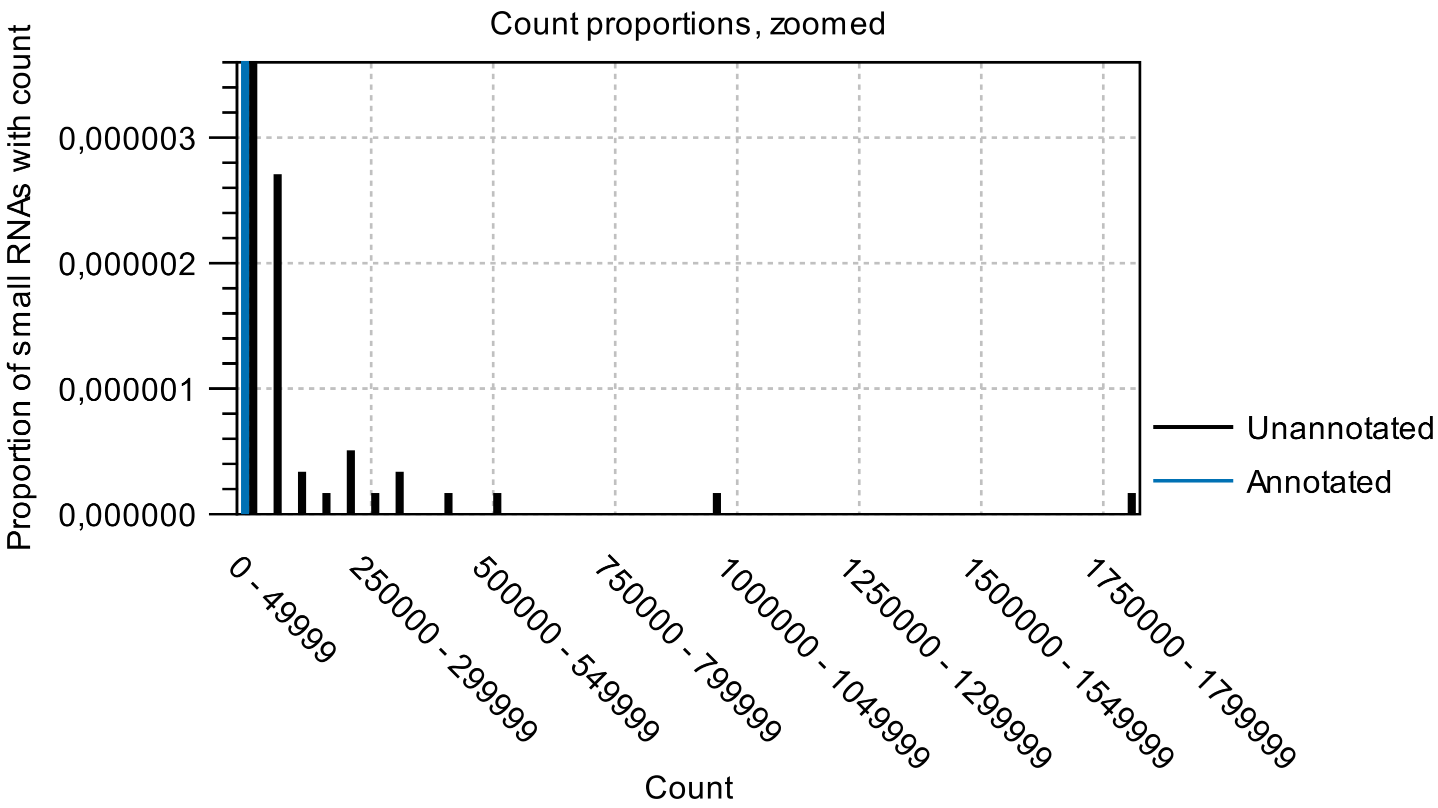


Zoomed version of the read count proportions histogram. Note that some bars extend beyond the plotting area.

# 4. Annotations (miRBase)

| Organism | Total | Mature 5' total | Mature 5' exact matches | Mature 5' length variants |
|---|---|---|---|---|
| Aegilops tauschii | 361 989 | 304 687 | 280 598 | 24 089 |
| Arabidopsis lyrata | 17 013 | 127 | 106 | 21 |
| Arabidopsis thaliana | 28 863 | 6 | 3 | 3 |
| Brachypodium distachyon | 27 008 | 5 283 | 2 689 | 2 594 |
| Brassica napus | 339 | 313 | 22 | 291 |
| Brassica oleracea | 0 | 0 | 0 | 0 |
| Brassica rapa | 27 | 25 | 22 | 3 |
| Caenorhabditis elegans | 1 | 1 | 1 | 0 |
| Chlamydomonas reinhardtii | 5 | 0 | 0 | 0 |
| Cucumis melo | 4 989 | 2 971 | 2 876 | 95 |
| Glycine max | 387 040 | 8 401 | 8 212 | 189 |

| Organism | Total | Mature 5' total | Mature 5' exact matches | Mature 5' length variants |
|---|---|---|---|---|
| Glycine soja | 0 | 0 | 0 | 0 |
| Gossypium arboreum | 0 | 0 | 0 | 0 |
| Gossypium herbaceum | 0 | 0 | 0 | 0 |
| Gossypium hirsutum | 6 | 5 | 3 | 2 |
| Gossypium raimondii | 1 487 | 3 | 0 | 3 |
| Hordeum vulgare | 107 267 | 97 120 | 45 179 | 51 941 |
| Medicago truncatula | 47 | 15 | 9 | 6 |
| Nicotiana tabacum | 101 | 30 | 23 | 7 |
| Oryza sativa | 31 720 | 1 641 | 51 | 1 590 |
| Phaseolus vulgaris | 207 | 207 | 201 | 6 |
| Physcomitrella patens | 18 521 | 12 046 | 9 | 12 037 |
| Pinus densata | 18 | 0 | 0 | 0 |
| Picea abies | 925 | 28 | 2 | 26 |
| Prunus persica | 1 718 | 1 | 1 | 0 |
| Solanum lycopersicum | 18 | 16 | 11 | 5 |
| Solanum tuberosum | 296 | 230 | 128 | 102 |
| Sorghum bicolor | 276 | 71 | 62 | 9 |
| Triticum aestivum | 15 461 | 660 | 578 | 82 |
| Triticum turgidum | 0 | 0 | 0 | 0 |
| Vitis vinifera | 90 | 29 | 12 | 17 |
| Zea mays | 1 317 | 170 | 148 | 22 |

| Organism | Mature 5' mutant variants | Non-mature total | Mature 3' | Precursor |
|---|---|---|---|---|
| Aegilops tauschii | 0 | 57 302 | 46 980 | 10 322 |
| Arabidopsis lyrata | 0 | 16 886 | 2 911 | 13 975 |
| Arabidopsis thaliana | 0 | 28 857 | 28 682 | 175 |
| Brachypodium distachyon | 0 | 21 725 | 18 028 | 3 697 |
| Brassica napus | 0 | 26 | 0 | 26 |
| Brassica oleracea | 0 | 0 | 0 | 0 |
| Brassica rapa | 0 | 2 | 1 | 1 |
| Caenorhabditis elegans | 0 | 0 | 0 | 0 |
| Chlamydomonas reinhardtii | 0 | 5 | 0 | 5 |
| Cucumis melo | 0 | 2 018 | 1 518 | 500 |
| Glycine max | 0 | 378 639 | 377 146 | 1 493 |
| Glycine soja | 0 | 0 | 0 | 0 |
| Gossypium arboreum | 0 | 0 | 0 | 0 |

| Organism | Mature 5' mutant variants | Non-mature total | Mature 3' | Precursor |
|---|---|---|---|---|
| Gossypium herbaceum | 0 | 0 | 0 | 0 |
| Gossypium hirsutum | 0 | 1 | 1 | 0 |
| Gossypium raimondii | 0 | 1 484 | 0 | 1 484 |
| Hordeum vulgare | 0 | 10 147 | 2 933 | 7 214 |
| Medicago truncatula | 0 | 32 | 23 | 9 |
| Nicotiana tabacum | 0 | 71 | 0 | 71 |
| Oryza sativa | 0 | 30 079 | 15 504 | 14 575 |
| Phaseolus vulgaris | 0 | 0 | 0 | 0 |
| Physcomitrella patens | 0 | 6 475 | 1 663 | 4 812 |
| Pinus densata | 0 | 18 | 10 | 8 |
| Picea abies | 0 | 897 | 865 | 32 |
| Prunus persica | 0 | 1 717 | 2 | 1 715 |
| Solanum lycopersicum | 0 | 2 | 1 | 1 |
| Solanum tuberosum | 0 | 66 | 62 | 4 |
| Sorghum bicolor | 0 | 205 | 172 | 33 |
| Triticum aestivum | 0 | 14 801 | 9 863 | 4 938 |
| Triticum turgidum | 0 | 0 | 0 | 0 |
| Vitis vinifera | 0 | 61 | 45 | 16 |
| Zea mays | 0 | 1 147 | 1 102 | 45 |
