# Supplementary material for: Pi-starvation induced transcriptional changes in barley revealed by a comprehensive RNA-Seq and degradome analyses
Source: BMC Genomics. 2021 Mar 9;22:165. doi: 10.1186/s12864-021-07481-w (PMC7941915; doi:10.1186/s12864-021-07481-w)
Supplement: Supplementary file 30 — Additional file 30. Spike-in quality control of RNA-Seq samples from barley shoots (low-Pi vs. control). Correlation between known and measured spike-in concentrations. [file 12864_2021_7481_MOESM30_ESM.pdf]

Additional file 30. Spike-in quality control of RNA-Seq samples from barley shoots (low-Pi vs. control). Correlation between known and measured spike-in concentrations.

Sample		Number of spike-ins detected	R^2
	S4	51/92	0.77
**-P**	S5	63/92	0.86
	S6	66/92	0.84
	S16	60/92	0.84
**+P**	S17	65/92	0.84
	S18	61/92	0.84
