# Supplementary material for: Pi-starvation induced transcriptional changes in barley revealed by a comprehensive RNA-Seq and degradome analyses
Source: BMC Genomics. 2021 Mar 9;22:165. doi: 10.1186/s12864-021-07481-w (PMC7941915; doi:10.1186/s12864-021-07481-w)
Supplement: Supplementary file 31 — Additional file 31. List of primers and probes used in this study. [file 12864_2021_7481_MOESM31_ESM.pdf]

Additional file 31. List of primers and probes used in this study.

	Name	Sequence (5' - '3)	Product size (bp)	Destiny
ddPCR analysis	**APO387-F**	CGTGACGCTGTGTTGCTTGT	61	Primers for ddPCR gene expression analysis of *HvARF1*
	**APO388-R**	CCGCATTCATCGCATTAGG		
	**APO796-F**	GGCGACTTCTCACCTCTAC	114	Primers for ddPCR gene expression analysis of *HvIPS1*
	**APO797-R**	CTGTGATCTTCACCGGTAGT		
	**APO704-F**	GTAGGCCTGACCTGCATCTG	163	Primers for ddPCR gene expression analysis of *HvSPX-MFS1*
	**APO705-R**	ACCAATGGCTGAGGAAACAG		
	**PS579-F**	ACAGGCTGAAGATGAGGCAC	127	Primers for ddPCR gene expression analysis of 3'5' -exonuclease
	**PS580-R**	TCAATCAAGGCAACTGCCATAC		
	**PS581-F**	CACGACACCAAGGGGAACAAG	123	Primers for ddPCR gene expression analysis of S1/P1 endonuclease
	**PS582-R**	GAAGTGCGCCAGGAACATCAG		
	**PS583-F**	GTCCTTCAACAGCCAGAACCC	130	Primers for ddPCR gene expression analysis of oxalate oxidase
	**PS584-R**	CTTGAGAAGTTCCACGACCCC		
	**PS585-F**	TCTCCTTCAACAGCCAAAACC	130	Primers for ddPCR gene expression analysis of oxalate oxidase 2
	**PS586-R**	TTGAGAAGTTCCACGACCCC		
Northern	**APO697**	TGTTTGCTGATGGTCATCTAA	-	Probe for mature hvu-miR827
	**U6_Probe**	TCATCCTTGCGCAGGGGCCA	-	Probe for U6 snRNA
