# Supplementary material for: Pi-starvation induced transcriptional changes in barley revealed by a comprehensive RNA-Seq and degradome analyses
Source: BMC Genomics. 2021 Mar 9;22:165. doi: 10.1186/s12864-021-07481-w (PMC7941915; doi:10.1186/s12864-021-07481-w)
Supplement: Supplementary file 32 — Additional file 32. Original, full-length blot of mature hvu-miR827 analysis. Lane 1: Decade™ Marker System (Invitrogen, Thermo Fisher Scientific); Lane 2: empty space (no sample loaded); Lane 3–5: RNA samples from root (Pi sufficient); Lane 6–8: shoot (Pi sufficient); Lane 9–11: root (low-Pi); Lane: 12–14: shoot (low-Pi). [file 12864_2021_7481_MOESM32_ESM.pdf]

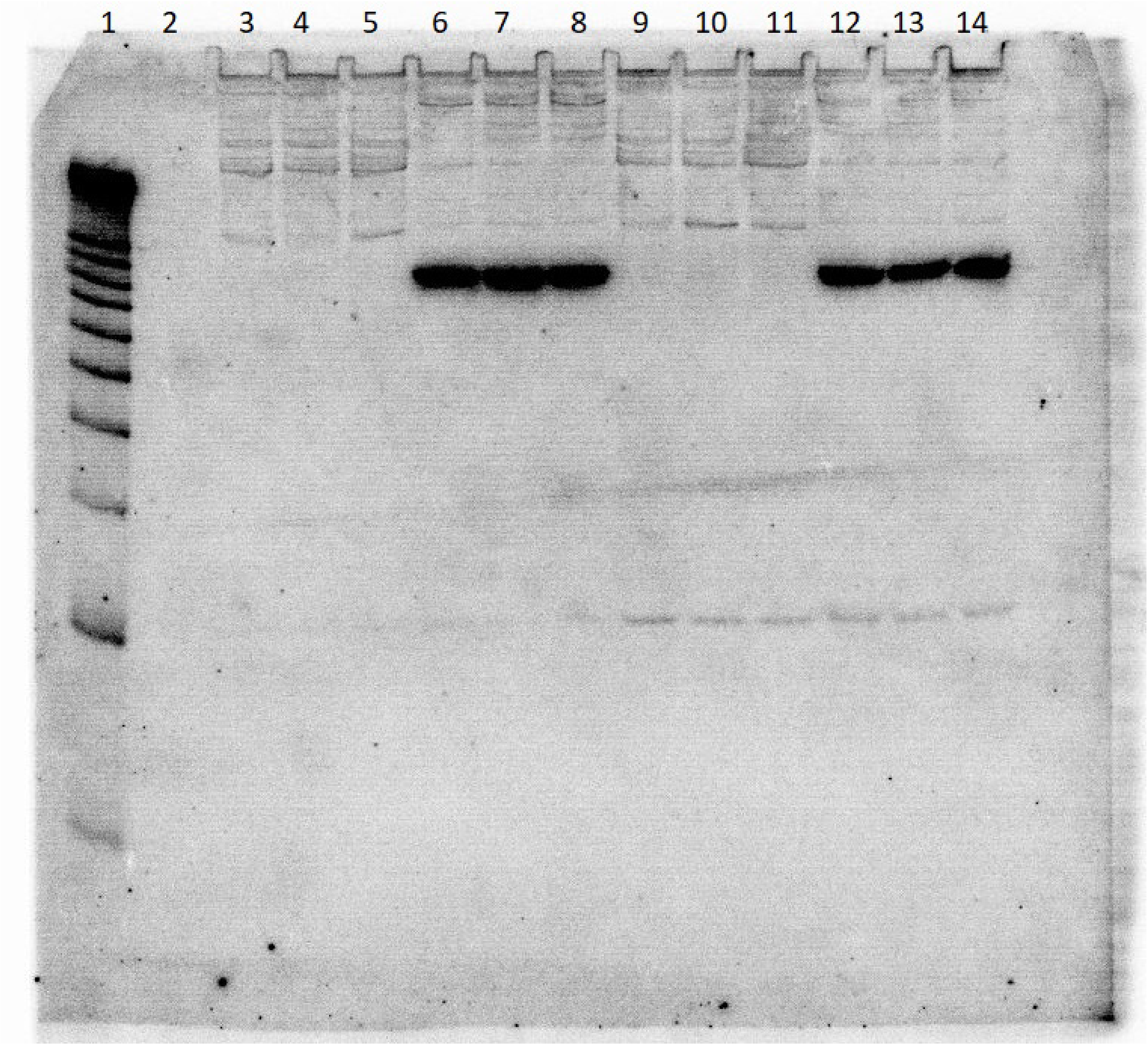

Additional file 32. Original, full-length blot of mature hvu-miR827 analysis. Lane 1: Decade™ Marker System (Invitrogen, Thermo Fisher Scientific); Lane 2: empty space (no sample loaded); Lane 3-5: RNA samples from root (Pi sufficient); Lane 6-8: shoot (Pi sufficient); Lane 9-11: root (low-Pi); Lane: 12-14: shoot (low-Pi).
